# Supplementary material for: Mesoderm-derived PDGFRA+ cells regulate the emergence of hematopoietic stem cells in the dorsal aorta
Source: Nat Cell Biol. 2022 Jul 28;24(8):1211–25. doi: 10.1038/s41556-022-00955-3 (PMC9359911; doi:10.1038/s41556-022-00955-3)
Supplement: Supplementary file 1 — Supplementary file containing the statistical analysis output. [file 41556_2022_955_MOESM1_ESM.pdf]

nature portfolio

https://doi.org/10.1038/s41556-022-00955-3

Supplementary information

Mesoderm-derived PDGFRA$^+$ cells regulate the emergence of hematopoietic stem cells in the dorsal aorta

In the format provided by the authors and unedited

The GLIMMIX Procedure

Model Information	
**Data Set**	WORK.FIG1D
**Response Variable**	Count
**Response Distribution**	Poisson
**Link Function**	Log
**Variance Function**	Default
**Variance Matrix Blocked By**	Dish_ID
**Estimation Technique**	Maximum Likelihood
**Likelihood Approximation**	Laplace
**Degrees of Freedom Method**	Containment

Class Level Information		
**Class**	**Levels**	**Values**
**Colony_Size**	3	Large Micro Small
**alpha**	4	high low minu plus
**gfp**	4	high low minus plus
**Dish_ID**	21	1 2 3 4 5 6 7 8 9 10 11 12 13 14 15 16 17 18 19 20 21

**Number of Observations Read**	63
**Number of Observations Used**	63

Dimensions	
**G-side Cov. Parameters**	1
**Columns in X**	64
**Columns in Z per Subject**	1
**Subjects (Blocks in V)**	21
**Max Obs per Subject**	3

The GLIMMIX Procedure

Optimization Information	
**Optimization Technique**	Dual Quasi-Newton
**Parameters in Optimization**	22
**Lower Boundaries**	1
**Upper Boundaries**	0
**Fixed Effects**	Not Profiled
**Starting From**	GLM estimates

Iteration History					
**Iteration**	**Restarts**	**Evaluations**	**Objective Function**	**Change**	**Max Gradient**
**0**	**0**	4	211.80461658	.	641.1376
**1**	**0**	3	210.74299409	1.06162250	0.113011
**2**	**0**	8	210.74298225	0.00001184	0.093253

Convergence criterion (GCONV=1E-8) satisfied.

Estimated G matrix is not positive definite.

Fit Statistics	
**-2 Log Likelihood**	210.74
**AIC (smaller is better)**	252.74
**AICC (smaller is better)**	275.28
**BIC (smaller is better)**	274.68
**CAIC (smaller is better)**	295.68
**HQIC (smaller is better)**	257.50

The GLIMMIX Procedure

Fit Statistics for Conditional Distribution	
-2 log L(Count | r. effects)	210.74
Pearson Chi-Square	12.60
Pearson Chi-Square / DF	0.20

Covariance Parameter Estimates			
Cov Parm	Subject	Estimate	Standard Error
Intercept	Dish_ID	2.17E-19	.

The GLIMMIX Procedure

Solutions for Fixed Effects								
Effect	Colony_Size	alpha	gfp	Estimate	Standard Error	DF	t Value	Pr > |t|
Intercept				3.5360	14.0686	14	0.25	0.8052
Colony_Size	Large			-0.1461	19.8965	28	-0.01	0.9942
Colony_Size	Micro			7.9260	17.2319	28	0.46	0.6491
Colony_Size	Small			0	.	.	.	.
alpha		high		-0.6457	14.0693	28	-0.05	0.9637
alpha		low		-1.3766	14.0700	28	-0.10	0.9228
alpha		minu		-9.2293	9.9483	28	-0.93	0.3615
alpha		plus		0	.	.	.	.
Colony_Size*alpha	Large	high		-0.4089	19.8978	28	-0.02	0.9838
Colony_Size*alpha	Large	low		-1.7257	19.9037	28	-0.09	0.9315
Colony_Size*alpha	Large	minu		0.1461	14.0695	28	0.01	0.9918
Colony_Size*alpha	Large	plus		0	.	.	.	.
Colony_Size*alpha	Micro	high		-8.6569	17.2335	28	-0.50	0.6194
Colony_Size*alpha	Micro	low		-7.4953	17.2338	28	-0.43	0.6670
Colony_Size*alpha	Micro	minu		-0.2405	14.0692	28	-0.02	0.9865
Colony_Size*alpha	Micro	plus		0	.	.	.	.
Colony_Size*alpha	Small	high		0	.	.	.	.
Colony_Size*alpha	Small	low		0	.	.	.	.
Colony_Size*alpha	Small	minu		0	.	.	.	.
Colony_Size*alpha	Small	plus		0	.	.	.	.
gfp			high	0.5920	0.2444	28	2.42	0.0222
gfp			low	0	.	.	.	.
gfp			minus	-0.00004	14.0683	28	-0.00	1.0000
gfp			plus	0	.	.	.	.

The GLIMMIX Procedure

Solutions for Fixed Effects								
**Effect**	**Colony_Size**	**alpha**	**gfp**	**Estimate**	**Standard Error**	**DF**	**t Value**	**Pr > |t|**
**Colony_Size*gfp**	Large		high	-6.5729	9.9633	28	-0.66	0.5148
**Colony_Size*gfp**	Large		low	0	.	.	.	.
**Colony_Size*gfp**	Large		minus	-0.00001	19.8960	28	-0.00	1.0000
**Colony_Size*gfp**	Large		plus	0	.	.	.	.
**Colony_Size*gfp**	Micro		high	0.4553	0.3058	28	1.49	0.1477
**Colony_Size*gfp**	Micro		low	0	.	.	.	.
**Colony_Size*gfp**	Micro		minus	-7.6856	17.2314	28	-0.45	0.6590
**Colony_Size*gfp**	Micro		plus	0	.	.	.	.
**Colony_Size*gfp**	Small		high	0	.	.	.	.
**Colony_Size*gfp**	Small		low	0	.	.	.	.
**Colony_Size*gfp**	Small		minus	0	.	.	.	.
**Colony_Size*gfp**	Small		plus	0	.	.	.	.
**alpha*gfp**		high	high	-1.1470	0.3325	28	-3.45	0.0018
**alpha*gfp**		high	low	0	.	.	.	.
**alpha*gfp**		low	high	0	.	.	.	.
**alpha*gfp**		low	low	0	.	.	.	.
**alpha*gfp**		minu	minus	0	.	.	.	.
**alpha*gfp**		minu	plus	0	.	.	.	.
**alpha*gfp**		plus	minus	0	.	.	.	.
**Colony_Siz*alpha*gfp**	Large	high	high	6.2588	9.9713	28	0.63	0.5353
**Colony_Siz*alpha*gfp**	Large	high	low	0	.	.	.	.
**Colony_Siz*alpha*gfp**	Large	low	high	0	.	.	.	.
**Colony_Siz*alpha*gfp**	Large	low	low	0	.	.	.	.
**Colony_Siz*alpha*gfp**	Large	minu	minus	0	.	.	.	.

The GLIMMIX Procedure

Solutions for Fixed Effects								
**Effect**	**Colony_Size**	**alpha**	**gfp**	**Estimate**	**Standard Error**	**DF**	**t Value**	**Pr > |t|**
**Colony_Siz*alpha*gfp**	Large	minu	plus	0	.	.	.	.
**Colony_Siz*alpha*gfp**	Large	plus	minus	0	.	.	.	.
**Colony_Siz*alpha*gfp**	Micro	high	high	0.8847	0.4476	28	1.98	0.0580
**Colony_Siz*alpha*gfp**	Micro	high	low	0	.	.	.	.
**Colony_Siz*alpha*gfp**	Micro	low	high	0	.	.	.	.
**Colony_Siz*alpha*gfp**	Micro	low	low	0	.	.	.	.
**Colony_Siz*alpha*gfp**	Micro	minu	minus	0	.	.	.	.
**Colony_Siz*alpha*gfp**	Micro	minu	plus	0	.	.	.	.
**Colony_Siz*alpha*gfp**	Micro	plus	minus	0	.	.	.	.
**Colony_Siz*alpha*gfp**	Small	high	high	0	.	.	.	.
**Colony_Siz*alpha*gfp**	Small	high	low	0	.	.	.	.
**Colony_Siz*alpha*gfp**	Small	low	high	0	.	.	.	.
**Colony_Siz*alpha*gfp**	Small	low	low	0	.	.	.	.
**Colony_Siz*alpha*gfp**	Small	minu	minus	0	.	.	.	.
**Colony_Siz*alpha*gfp**	Small	minu	plus	0	.	.	.	.
**Colony_Siz*alpha*gfp**	Small	plus	minus	0	.	.	.	.

Type III Tests of Fixed Effects				
**Effect**	**Num DF**	**Den DF**	**F Value**	**Pr > F**
**Colony_Size**	2	28	0.89	0.4205
**alpha**	2	28	1.65	0.2094
**Colony_Size*alpha**	4	28	2.82	0.0439
**gfp**	2	28	0.18	0.8327
**Colony_Size*gfp**	4	28	4.25	0.0082

The GLIMMIX Procedure

Type III Tests of Fixed Effects				
Effect	Num DF	Den DF	F Value	Pr > F
alpha*gfp	1	28	0.14	0.7133
Colony_Siz*alpha*gfp	2	28	2.12	0.1387

alpha*gfp Least Squares Means													
alpha	gfp	Estimate	Standard Error	DF	t Value	Pr > |t|	Alpha	Lower	Upper	Mean	Standard Error Mean	Lower Mean	Upper Mean
high	high	2.2486	0.1187	28	18.95	<.0001	0.05	2.0055	2.4917	9.4745	1.1243	7.4300	12.0816
high	low	2.4616	0.09958	28	24.72	<.0001	0.05	2.2577	2.6656	11.7241	1.1675	9.5607	14.3770
low	high	0.2319	3.3164	28	0.07	0.9448	0.05	-6.5615	7.0252	1.2609	4.1818	0.001414	1124.64
low	low	1.6790	0.1866	28	9.00	<.0001	0.05	1.2967	2.0613	5.3603	1.0004	3.6573	7.8565
minu	minus	-5.6934	5.7435	28	-0.99	0.3300	0.05	-17.4584	6.0716	0.003368	0.01935	2.618E-8	433.39
minu	plus	-3.1315	4.6900	28	-0.67	0.5098	0.05	-12.7385	6.4756	0.04365	0.2047	2.936E-6	649.09
plus	minus	3.5674	0.05635	28	63.30	<.0001	0.05	3.4520	3.6829	35.4253	1.9964	31.5631	39.7601

Differences of alpha*gfp Least Squares Means											
alpha	gfp	_alpha	_gfp	Estimate	Standard Error	DF	t Value	Pr > |t|	Alpha	Lower	Upper
high	high	high	low	-0.2130	0.1549	28	-1.38	0.1800	0.05	-0.5304	0.1043
high	high	low	high	2.0167	3.3185	28	0.61	0.5483	0.05	-4.7810	8.8145
high	high	low	low	0.5696	0.2212	28	2.58	0.0156	0.05	0.1165	1.0226
high	high	minu	minus	7.9420	5.7447	28	1.38	0.1778	0.05	-3.8255	19.7095
high	high	minu	plus	5.3801	4.6915	28	1.15	0.2612	0.05	-4.2301	14.9903
high	high	plus	minus	-1.3188	0.1314	28	-10.04	<.0001	0.05	-1.5879	-1.0497
high	low	low	high	2.2298	3.3179	28	0.67	0.5071	0.05	-4.5666	9.0262
high	low	low	low	0.7826	0.2115	28	3.70	0.0009	0.05	0.3493	1.2159

The GLIMMIX Procedure

Differences of alpha*gfp Least Squares Means											
alpha	gfp	_alpha	_gfp	Estimate	Standard Error	DF	t Value	Pr > |t|	Alpha	Lower	Upper
high	low	minu	minus	8.1550	5.7443	28	1.42	0.1667	0.05	-3.6117	19.9218
high	low	minu	plus	5.5931	4.6911	28	1.19	0.2432	0.05	-4.0162	15.2024
high	low	plus	minus	-1.1058	0.1144	28	-9.66	<.0001	0.05	-1.3402	-0.8714
low	high	low	low	-1.4472	3.3217	28	-0.44	0.6664	0.05	-8.2513	5.3569
low	high	minu	minus	5.9252	6.6322	28	0.89	0.3793	0.05	-7.6602	19.5107
low	high	minu	plus	3.3633	5.7441	28	0.59	0.5629	0.05	-8.4030	15.1296
low	high	plus	minus	-3.3356	3.3169	28	-1.01	0.3232	0.05	-10.1299	3.4588
low	low	minu	minus	7.3724	5.7465	28	1.28	0.2100	0.05	-4.3988	19.1436
low	low	minu	plus	4.8105	4.6937	28	1.02	0.3142	0.05	-4.8042	14.4252
low	low	plus	minus	-1.8884	0.1950	28	-9.69	<.0001	0.05	-2.2878	-1.4890
minu	minus	minu	plus	-2.5619	7.4152	28	-0.35	0.7323	0.05	-17.7512	12.6274
minu	minus	plus	minus	-9.2608	5.7437	28	-1.61	0.1181	0.05	-21.0263	2.5047
minu	plus	plus	minus	-6.6989	4.6904	28	-1.43	0.1643	0.05	-16.3067	2.9089

Colony_Siz*alpha*gfp Least Squares Means														
Colony_Size	alpha	gfp	Estimate	Standard Error	DF	t Value	Pr > |t|	Alpha	Lower	Upper	Mean	Standard Error Mean	Lower Mean	Upper Mean
Large	high	high	1.4662	0.2774	28	5.29	<.0001	0.05	0.8980	2.0344	4.3327	1.2017	2.4548	7.6473
Large	high	low	2.3353	0.1796	28	13.00	<.0001	0.05	1.9673	2.7032	10.3321	1.8558	7.1515	14.9271
Large	low	high	-5.6933	9.9477	28	-0.57	0.5717	0.05	-26.0703	14.6837	0.003368	0.03351	4.76E-12	2382526
Large	low	low	0.2875	0.5000	28	0.58	0.5699	0.05	-0.7367	1.3118	1.3331	0.6666	0.4787	3.7129
Large	minu	minus	-5.6934	9.9483	28	-0.57	0.5717	0.05	-26.0716	14.6848	0.003368	0.03351	4.76E-12	2385189
Large	minu	plus	-5.6934	9.9480	28	-0.57	0.5717	0.05	-26.0710	14.6843	0.003368	0.03351	4.76E-12	2383982
Large	plus	minus	3.3898	0.1060	28	31.98	<.0001	0.05	3.1727	3.6070	29.6614	3.1444	23.8718	36.8552

The GLIMMIX Procedure

Colony_Siz*alpha*gfp Least Squares Means

Colony_Size	alpha	gfp	Estimate	Standard Error	DF	t Value	Pr > |t|	Alpha	Lower	Upper	Mean	Standard Error Mean	Lower Mean	Upper Mean
Micro	high	high	2.9443	0.1325	28	22.23	<.0001	0.05	2.6730	3.2157	18.9981	2.5165	14.4834	24.9199
Micro	high	low	2.1594	0.1961	28	11.01	<.0001	0.05	1.7577	2.5611	8.6659	1.6996	5.7988	12.9505
Micro	low	high	3.6375	0.09366	28	38.84	<.0001	0.05	3.4456	3.8293	37.9956	3.5588	31.3624	46.0318
Micro	low	low	2.5902	0.1581	28	16.38	<.0001	0.05	2.2663	2.9141	13.3319	2.1080	9.6433	18.4314
Micro	minu	minus	-5.6934	9.9481	28	-0.57	0.5717	0.05	-26.0711	14.6844	0.003368	0.03351	4.76E-12	2384225
Micro	minu	plus	1.9923	0.2132	28	9.34	<.0001	0.05	1.5555	2.4290	7.3320	1.5633	4.7374	11.3476
Micro	plus	minus	3.7764	0.08738	28	43.22	<.0001	0.05	3.5975	3.9554	43.6602	3.8148	36.5053	52.2176
Small	high	high	2.3353	0.1796	28	13.00	<.0001	0.05	1.9674	2.7032	10.3324	1.8558	7.1518	14.9275
Small	high	low	2.8903	0.1361	28	21.24	<.0001	0.05	2.6115	3.1691	17.9986	2.4494	13.6198	23.7850
Small	low	high	2.7514	0.1459	28	18.86	<.0001	0.05	2.4526	3.0502	15.6650	2.2851	11.6188	21.1203
Small	low	low	2.1594	0.1961	28	11.01	<.0001	0.05	1.7576	2.5611	8.6659	1.6996	5.7988	12.9505
Small	minu	minus	-5.6933	9.9478	28	-0.57	0.5717	0.05	-26.0705	14.6839	0.003368	0.03351	4.76E-12	2382999
Small	minu	plus	-5.6933	9.9477	28	-0.57	0.5717	0.05	-26.0702	14.6836	0.003368	0.03351	4.76E-12	2382243
Small	plus	minus	3.5360	0.09854	28	35.88	<.0001	0.05	3.3341	3.7378	34.3291	3.3827	28.0545	42.0072

The GLIMMIX Procedure

Differences of Colony_Siz*alpha*gfp Least Squares Means													
Colony_Size	alpha	gfp	_Colony_Size	_alpha	_gfp	Estimate	Standard Error	DF	t Value	Pr > |t|	Alpha	Lower	Upper
Large	high	high	Large	high	low	-0.8691	0.3304	28	-2.63	0.0137	0.05	-1.5460	-0.1922
Large	high	high	Large	low	high	7.1595	9.9516	28	0.72	0.4778	0.05	-13.2254	27.5444
Large	high	high	Large	low	low	1.1787	0.5718	28	2.06	0.0487	0.05	0.007355	2.3500
Large	high	high	Large	minu	minus	7.1596	9.9522	28	0.72	0.4779	0.05	-13.2265	27.5458
Large	high	high	Large	minu	plus	7.1596	9.9519	28	0.72	0.4778	0.05	-13.2260	27.5451
Large	high	high	Large	plus	minus	-1.9237	0.2969	28	-6.48	<.0001	0.05	-2.5319	-1.3154
Large	high	high	Micro	high	high	-1.4781	0.3074	28	-4.81	<.0001	0.05	-2.1078	-0.8485
Large	high	high	Micro	high	low	-0.6932	0.3397	28	-2.04	0.0508	0.05	-1.3890	0.002642
Large	high	high	Micro	low	high	-2.1713	0.2928	28	-7.42	<.0001	0.05	-2.7710	-1.5716
Large	high	high	Micro	low	low	-1.1240	0.3193	28	-3.52	0.0015	0.05	-1.7780	-0.4700
Large	high	high	Micro	minu	minus	7.1596	9.9520	28	0.72	0.4778	0.05	-13.2261	27.5453
Large	high	high	Micro	minu	plus	-0.5261	0.3498	28	-1.50	0.1439	0.05	-1.2427	0.1906
Large	high	high	Micro	plus	minus	-2.3102	0.2908	28	-7.94	<.0001	0.05	-2.9059	-1.7146
Large	high	high	Small	high	high	-0.8691	0.3304	28	-2.63	0.0137	0.05	-1.5460	-0.1922
Large	high	high	Small	high	low	-1.4241	0.3090	28	-4.61	<.0001	0.05	-2.0570	-0.7912
Large	high	high	Small	low	high	-1.2852	0.3134	28	-4.10	0.0003	0.05	-1.9272	-0.6433
Large	high	high	Small	low	low	-0.6932	0.3397	28	-2.04	0.0508	0.05	-1.3890	0.002648
Large	high	high	Small	minu	minus	7.1595	9.9517	28	0.72	0.4778	0.05	-13.2255	27.5446
Large	high	high	Small	minu	plus	7.1595	9.9516	28	0.72	0.4778	0.05	-13.2254	27.5444
Large	high	high	Small	plus	minus	-2.0698	0.2944	28	-7.03	<.0001	0.05	-2.6728	-1.4669
Large	high	low	Large	low	high	8.0286	9.9493	28	0.81	0.4265	0.05	-12.3517	28.4089
Large	high	low	Large	low	low	2.0477	0.5313	28	3.85	0.0006	0.05	0.9594	3.1361
Large	high	low	Large	minu	minus	8.0287	9.9499	28	0.81	0.4265	0.05	-12.3529	28.4102
Large	high	low	Large	minu	plus	8.0286	9.9497	28	0.81	0.4265	0.05	-12.3523	28.4096

The GLIMMIX Procedure

Colony_Size	alpha	gfp	_Colony_Size	_alpha	_gfp	Estimate	Standard Error	DF	t Value	Pr > |t|	Alpha	Lower	Upper
Large	high	low	Large	plus	minus	-1.0546	0.2086	28	-5.06	<.0001	0.05	-1.4818	-0.6274
Large	high	low	Micro	high	high	-0.6091	0.2232	28	-2.73	0.0108	0.05	-1.0662	-0.1519
Large	high	low	Micro	high	low	0.1759	0.2659	28	0.66	0.5138	0.05	-0.3689	0.7206
Large	high	low	Micro	low	high	-1.3022	0.2026	28	-6.43	<.0001	0.05	-1.7172	-0.8873
Large	high	low	Micro	low	low	-0.2549	0.2393	28	-1.07	0.2959	0.05	-0.7451	0.2353
Large	high	low	Micro	minu	minus	8.0286	9.9497	28	0.81	0.4265	0.05	-12.3525	28.4097
Large	high	low	Micro	minu	plus	0.3430	0.2788	28	1.23	0.2288	0.05	-0.2281	0.9141
Large	high	low	Micro	plus	minus	-1.4412	0.1997	28	-7.22	<.0001	0.05	-1.8503	-1.0320
Large	high	low	Small	high	high	-0.00003	0.2540	28	-0.00	0.9999	0.05	-0.5204	0.5203
Large	high	low	Small	high	low	-0.5550	0.2253	28	-2.46	0.0202	0.05	-1.0166	-0.09343
Large	high	low	Small	low	high	-0.4162	0.2314	28	-1.80	0.0829	0.05	-0.8902	0.05780
Large	high	low	Small	low	low	0.1759	0.2659	28	0.66	0.5138	0.05	-0.3689	0.7206
Large	high	low	Small	minu	minus	8.0286	9.9494	28	0.81	0.4265	0.05	-12.3518	28.4090
Large	high	low	Small	minu	plus	8.0286	9.9493	28	0.81	0.4265	0.05	-12.3517	28.4088
Large	high	low	Small	plus	minus	-1.2007	0.2049	28	-5.86	<.0001	0.05	-1.6204	-0.7811
Large	low	high	Large	low	low	-5.9809	9.9603	28	-0.60	0.5530	0.05	-26.3836	14.4219
Large	low	high	Large	minu	minus	0.000099	14.0686	28	0.00	1.0000	0.05	-28.8182	28.8184
Large	low	high	Large	minu	plus	0.000045	14.0684	28	0.00	1.0000	0.05	-28.8178	28.8179
Large	low	high	Large	plus	minus	-9.0832	9.9483	28	-0.91	0.3690	0.05	-29.4613	11.2950
Large	low	high	Micro	high	high	-8.6377	9.9486	28	-0.87	0.3927	0.05	-29.0165	11.7411
Large	low	high	Micro	high	low	-7.8527	9.9497	28	-0.79	0.4366	0.05	-28.2337	12.5282
Large	low	high	Micro	low	high	-9.3308	9.9482	28	-0.94	0.3563	0.05	-29.7087	11.0471
Large	low	high	Micro	low	low	-8.2835	9.9490	28	-0.83	0.4121	0.05	-28.6631	12.0961
Large	low	high	Micro	minu	minus	0.000055	14.0685	28	0.00	1.0000	0.05	-28.8179	28.8180

Table title: Differences of Colony_Siz*alpha*gfp Least Squares Means

The GLIMMIX Procedure

Differences of Colony_Siz*alpha*gfp Least Squares Means

Colony_Size	alpha	gfp	_Colony_Size	_alpha	_gfp	Estimate	Standard Error	DF	t Value	Pr > |t|	Alpha	Lower	Upper
Large	low	high	Micro	minu	plus	-7.6856	9.9500	28	-0.77	0.4463	0.05	-28.0673	12.6961
Large	low	high	Micro	plus	minus	-9.4698	9.9481	28	-0.95	0.3493	0.05	-29.8475	10.9080
Large	low	high	Small	high	high	-8.0286	9.9493	28	-0.81	0.4265	0.05	-28.4089	12.3517
Large	low	high	Small	high	low	-8.5836	9.9487	28	-0.86	0.3956	0.05	-28.9625	11.7953
Large	low	high	Small	low	high	-8.4448	9.9488	28	-0.85	0.4032	0.05	-28.8239	11.9344
Large	low	high	Small	low	low	-7.8527	9.9497	28	-0.79	0.4366	0.05	-28.2337	12.5282
Large	low	high	Small	minu	minus	0.000016	14.0682	28	0.00	1.0000	0.05	-28.8175	28.8175
Large	low	high	Small	minu	plus	-0.00002	14.0682	28	-0.00	1.0000	0.05	-28.8174	28.8174
Large	low	high	Small	plus	minus	-9.2293	9.9482	28	-0.93	0.3615	0.05	-29.6073	11.1487
Large	low	low	Large	minu	minus	5.9810	9.9609	28	0.60	0.5530	0.05	-14.4230	26.3849
Large	low	low	Large	minu	plus	5.9809	9.9606	28	0.60	0.5530	0.05	-14.4224	26.3842
Large	low	low	Large	plus	minus	-3.1023	0.5111	28	-6.07	<.0001	0.05	-4.1494	-2.0553
Large	low	low	Micro	high	high	-2.6568	0.5173	28	-5.14	<.0001	0.05	-3.7164	-1.5972
Large	low	low	Micro	high	low	-1.8719	0.5371	28	-3.48	0.0016	0.05	-2.9721	-0.7716
Large	low	low	Micro	low	high	-3.3499	0.5087	28	-6.58	<.0001	0.05	-4.3920	-2.3078
Large	low	low	Micro	low	low	-2.3026	0.5244	28	-4.39	0.0001	0.05	-3.3769	-1.2284
Large	low	low	Micro	minu	minus	5.9809	9.9607	28	0.60	0.5530	0.05	-14.4226	26.3844
Large	low	low	Micro	minu	plus	-1.7047	0.5436	28	-3.14	0.0040	0.05	-2.8182	-0.5912
Large	low	low	Micro	plus	minus	-3.4889	0.5076	28	-6.87	<.0001	0.05	-4.5287	-2.4491
Large	low	low	Small	high	high	-2.0478	0.5313	28	-3.85	0.0006	0.05	-3.1361	-0.9594
Large	low	low	Small	high	low	-2.6028	0.5182	28	-5.02	<.0001	0.05	-3.6643	-1.5412
Large	low	low	Small	low	high	-2.4639	0.5209	28	-4.73	<.0001	0.05	-3.5309	-1.3969
Large	low	low	Small	low	low	-1.8719	0.5371	28	-3.48	0.0016	0.05	-2.9721	-0.7716
Large	low	low	Small	minu	minus	5.9809	9.9603	28	0.60	0.5530	0.05	-14.4219	26.3837

The GLIMMIX Procedure

Differences of Colony_Siz*alpha*gfp Least Squares Means

Colony_Size	alpha	gfp	_Colony_Size	_alpha	_gfp	Estimate	Standard Error	DF	t Value	Pr > |t|	Alpha	Lower	Upper
Large	low	low	Small	minu	plus	5.9808	9.9603	28	0.60	0.5530	0.05	-14.4219	26.3835
Large	low	low	Small	plus	minus	-3.2485	0.5097	28	-6.37	<.0001	0.05	-4.2924	-2.2045
Large	minu	minus	Large	minu	plus	-0.00005	14.0689	28	-0.00	1.0000	0.05	-28.8188	28.8187
Large	minu	minus	Large	plus	minus	-9.0833	9.9489	28	-0.91	0.3690	0.05	-29.4626	11.2961
Large	minu	minus	Micro	high	high	-8.6378	9.9492	28	-0.87	0.3927	0.05	-29.0178	11.7423
Large	minu	minus	Micro	high	low	-7.8528	9.9503	28	-0.79	0.4366	0.05	-28.2350	12.5294
Large	minu	minus	Micro	low	high	-9.3309	9.9488	28	-0.94	0.3563	0.05	-29.7100	11.0482
Large	minu	minus	Micro	low	low	-8.2836	9.9496	28	-0.83	0.4121	0.05	-28.6644	12.0972
Large	minu	minus	Micro	minu	minus	-0.00004	14.0689	28	-0.00	1.0000	0.05	-28.8189	28.8188
Large	minu	minus	Micro	minu	plus	-7.6857	9.9506	28	-0.77	0.4464	0.05	-28.0686	12.6972
Large	minu	minus	Micro	plus	minus	-9.4699	9.9487	28	-0.95	0.3493	0.05	-29.8489	10.9091
Large	minu	minus	Small	high	high	-8.0287	9.9499	28	-0.81	0.4265	0.05	-28.4102	12.3528
Large	minu	minus	Small	high	low	-8.5837	9.9493	28	-0.86	0.3956	0.05	-28.9638	11.7964
Large	minu	minus	Small	low	high	-8.4449	9.9494	28	-0.85	0.4032	0.05	-28.8253	11.9356
Large	minu	minus	Small	low	low	-7.8528	9.9503	28	-0.79	0.4366	0.05	-28.2350	12.5294
Large	minu	minus	Small	minu	minus	-0.00008	14.0688	28	-0.00	1.0000	0.05	-28.8186	28.8185
Large	minu	minus	Small	minu	plus	-0.00012	14.0686	28	-0.00	1.0000	0.05	-28.8184	28.8181
Large	minu	minus	Small	plus	minus	-9.2294	9.9488	28	-0.93	0.3615	0.05	-29.6086	11.1498
Large	minu	plus	Large	plus	minus	-9.0832	9.9486	28	-0.91	0.3690	0.05	-29.4620	11.2956
Large	minu	plus	Micro	high	high	-8.6377	9.9489	28	-0.87	0.3927	0.05	-29.0172	11.7418
Large	minu	plus	Micro	high	low	-7.8528	9.9500	28	-0.79	0.4366	0.05	-28.2344	12.5288
Large	minu	plus	Micro	low	high	-9.3308	9.9485	28	-0.94	0.3563	0.05	-29.7094	11.0477
Large	minu	plus	Micro	low	low	-8.2835	9.9493	28	-0.83	0.4121	0.05	-28.6638	12.0967
Large	minu	plus	Micro	minu	minus	0.000010	14.0687	28	0.00	1.0000	0.05	-28.8184	28.8184

The GLIMMIX Procedure

Differences of Colony_Siz*alpha*gfp Least Squares Means													
Colony_Size	alpha	gfp	_Colony_Size	_alpha	_gfp	Estimate	Standard Error	DF	t Value	Pr > |t|	Alpha	Lower	Upper
Large	minu	plus	Micro	minu	plus	-7.6856	9.9503	28	-0.77	0.4463	0.05	-28.0680	12.6967
Large	minu	plus	Micro	plus	minus	-9.4698	9.9484	28	-0.95	0.3493	0.05	-29.8482	10.9086
Large	minu	plus	Small	high	high	-8.0287	9.9497	28	-0.81	0.4265	0.05	-28.4096	12.3523
Large	minu	plus	Small	high	low	-8.5837	9.9490	28	-0.86	0.3956	0.05	-28.9632	11.7959
Large	minu	plus	Small	low	high	-8.4448	9.9491	28	-0.85	0.4032	0.05	-28.8246	11.9350
Large	minu	plus	Small	low	low	-7.8528	9.9500	28	-0.79	0.4366	0.05	-28.2344	12.5289
Large	minu	plus	Small	minu	minus	-0.00003	14.0686	28	-0.00	1.0000	0.05	-28.8182	28.8181
Large	minu	plus	Small	minu	plus	-0.00007	14.0684	28	-0.00	1.0000	0.05	-28.8179	28.8178
Large	minu	plus	Small	plus	minus	-9.2294	9.9485	28	-0.93	0.3615	0.05	-29.6080	11.1493
Large	plus	minus	Micro	high	high	0.4455	0.1697	28	2.63	0.0138	0.05	0.09798	0.7930
Large	plus	minus	Micro	high	low	1.2305	0.2229	28	5.52	<.0001	0.05	0.7738	1.6871
Large	plus	minus	Micro	low	high	-0.2476	0.1415	28	-1.75	0.0910	0.05	-0.5374	0.04214
Large	plus	minus	Micro	low	low	0.7997	0.1904	28	4.20	0.0002	0.05	0.4097	1.1896
Large	plus	minus	Micro	minu	minus	9.0832	9.9487	28	0.91	0.3690	0.05	-11.2957	29.4621
Large	plus	minus	Micro	minu	plus	1.3976	0.2381	28	5.87	<.0001	0.05	0.9098	1.8854
Large	plus	minus	Micro	plus	minus	-0.3866	0.1374	28	-2.81	0.0088	0.05	-0.6680	-0.1052
Large	plus	minus	Small	high	high	1.0546	0.2086	28	5.06	<.0001	0.05	0.6273	1.4818
Large	plus	minus	Small	high	low	0.4996	0.1725	28	2.90	0.0073	0.05	0.1462	0.8529
Large	plus	minus	Small	low	high	0.6384	0.1803	28	3.54	0.0014	0.05	0.2690	1.0078
Large	plus	minus	Small	low	low	1.2305	0.2229	28	5.52	<.0001	0.05	0.7738	1.6871
Large	plus	minus	Small	minu	minus	9.0832	9.9483	28	0.91	0.3690	0.05	-11.2951	29.4614
Large	plus	minus	Small	minu	plus	9.0831	9.9483	28	0.91	0.3690	0.05	-11.2950	29.4613
Large	plus	minus	Small	plus	minus	-0.1461	0.1447	28	-1.01	0.3213	0.05	-0.4426	0.1503
Micro	high	high	Micro	high	low	0.7849	0.2367	28	3.32	0.0025	0.05	0.3002	1.2697

The GLIMMIX Procedure

Differences of Colony_Siz*alpha*gfp Least Squares Means													
Colony_Size	alpha	gfp	_Colony_Size	_alpha	_gfp	Estimate	Standard Error	DF	t Value	Pr > |t|	Alpha	Lower	Upper
Micro	high	high	Micro	low	high	-0.6931	0.1622	28	-4.27	0.0002	0.05	-1.0254	-0.3608
Micro	high	high	Micro	low	low	0.3542	0.2063	28	1.72	0.0970	0.05	-0.06835	0.7767
Micro	high	high	Micro	minu	minus	8.6377	9.9490	28	0.87	0.3927	0.05	-11.7419	29.0173
Micro	high	high	Micro	minu	plus	0.9521	0.2510	28	3.79	0.0007	0.05	0.4379	1.4663
Micro	high	high	Micro	plus	minus	-0.8321	0.1587	28	-5.24	<.0001	0.05	-1.1571	-0.5071
Micro	high	high	Small	high	high	0.6091	0.2232	28	2.73	0.0109	0.05	0.1519	1.0662
Micro	high	high	Small	high	low	0.05405	0.1899	28	0.28	0.7781	0.05	-0.3350	0.4431
Micro	high	high	Small	low	high	0.1929	0.1970	28	0.98	0.3359	0.05	-0.2107	0.5965
Micro	high	high	Small	low	low	0.7849	0.2367	28	3.32	0.0025	0.05	0.3002	1.2697
Micro	high	high	Small	minu	minus	8.6377	9.9487	28	0.87	0.3927	0.05	-11.7412	29.0166
Micro	high	high	Small	minu	plus	8.6376	9.9486	28	0.87	0.3927	0.05	-11.7411	29.0164
Micro	high	high	Small	plus	minus	-0.5917	0.1651	28	-3.58	0.0013	0.05	-0.9298	-0.2535
Micro	high	low	Micro	low	high	-1.4781	0.2173	28	-6.80	<.0001	0.05	-1.9233	-1.0329
Micro	high	low	Micro	low	low	-0.4308	0.2519	28	-1.71	0.0983	0.05	-0.9468	0.08527
Micro	high	low	Micro	minu	minus	7.8528	9.9500	28	0.79	0.4366	0.05	-12.5290	28.2345
Micro	high	low	Micro	minu	plus	0.1671	0.2897	28	0.58	0.5686	0.05	-0.4263	0.7606
Micro	high	low	Micro	plus	minus	-1.6170	0.2147	28	-7.53	<.0001	0.05	-2.0568	-1.1772
Micro	high	low	Small	high	high	-0.1759	0.2659	28	-0.66	0.5138	0.05	-0.7206	0.3689
Micro	high	low	Small	high	low	-0.7309	0.2387	28	-3.06	0.0048	0.05	-1.2199	-0.2419
Micro	high	low	Small	low	high	-0.5920	0.2444	28	-2.42	0.0222	0.05	-1.0927	-0.09136
Micro	high	low	Small	low	low	2.402E-6	0.2774	28	0.00	1.0000	0.05	-0.5681	0.5681
Micro	high	low	Small	minu	minus	7.8527	9.9497	28	0.79	0.4366	0.05	-12.5283	28.2338
Micro	high	low	Small	minu	plus	7.8527	9.9496	28	0.79	0.4366	0.05	-12.5282	28.2336
Micro	high	low	Small	plus	minus	-1.3766	0.2195	28	-6.27	<.0001	0.05	-1.8262	-0.9270

The GLIMMIX Procedure

Differences of Colony_Siz*alpha*gfp Least Squares Means													
Colony_Size	alpha	gfp	_Colony_Size	_alpha	_gfp	Estimate	Standard Error	DF	t Value	Pr > |t|	Alpha	Lower	Upper
Micro	low	high	Micro	low	low	1.0473	0.1838	28	5.70	<.0001	0.05	0.6708	1.4238
Micro	low	high	Micro	minu	minus	9.3308	9.9485	28	0.94	0.3563	0.05	-11.0478	29.7095
Micro	low	high	Micro	minu	plus	1.6452	0.2329	28	7.06	<.0001	0.05	1.1682	2.1223
Micro	low	high	Micro	plus	minus	-0.1390	0.1281	28	-1.08	0.2872	0.05	-0.4014	0.1234
Micro	low	high	Small	high	high	1.3022	0.2026	28	6.43	<.0001	0.05	0.8872	1.7171
Micro	low	high	Small	high	low	0.7472	0.1652	28	4.52	0.0001	0.05	0.4088	1.0856
Micro	low	high	Small	low	high	0.8860	0.1734	28	5.11	<.0001	0.05	0.5309	1.2411
Micro	low	high	Small	low	low	1.4781	0.2173	28	6.80	<.0001	0.05	1.0329	1.9233
Micro	low	high	Small	minu	minus	9.3308	9.9482	28	0.94	0.3563	0.05	-11.0473	29.7089
Micro	low	high	Small	minu	plus	9.3308	9.9481	28	0.94	0.3563	0.05	-11.0470	29.7086
Micro	low	high	Small	plus	minus	0.1015	0.1360	28	0.75	0.4616	0.05	-0.1770	0.3800
Micro	low	low	Micro	minu	minus	8.2835	9.9494	28	0.83	0.4121	0.05	-12.0968	28.6639
Micro	low	low	Micro	minu	plus	0.5979	0.2654	28	2.25	0.0323	0.05	0.05416	1.1417
Micro	low	low	Micro	plus	minus	-1.1863	0.1807	28	-6.57	<.0001	0.05	-1.5563	-0.8162
Micro	low	low	Small	high	high	0.2549	0.2393	28	1.07	0.2959	0.05	-0.2353	0.7451
Micro	low	low	Small	high	low	-0.3001	0.2086	28	-1.44	0.1613	0.05	-0.7275	0.1272
Micro	low	low	Small	low	high	-0.1613	0.2151	28	-0.75	0.4597	0.05	-0.6019	0.2794
Micro	low	low	Small	low	low	0.4308	0.2519	28	1.71	0.0983	0.05	-0.08527	0.9468
Micro	low	low	Small	minu	minus	8.2835	9.9491	28	0.83	0.4121	0.05	-12.0962	28.6632
Micro	low	low	Small	minu	plus	8.2835	9.9489	28	0.83	0.4121	0.05	-12.0960	28.6629
Micro	low	low	Small	plus	minus	-0.9458	0.1863	28	-5.08	<.0001	0.05	-1.3275	-0.5642
Micro	minu	minus	Micro	minu	plus	-7.6856	9.9503	28	-0.77	0.4463	0.05	-28.0678	12.6966
Micro	minu	minus	Micro	plus	minus	-9.4698	9.9485	28	-0.95	0.3493	0.05	-29.8484	10.9087
Micro	minu	minus	Small	high	high	-8.0287	9.9497	28	-0.81	0.4265	0.05	-28.4097	12.3524

The GLIMMIX Procedure

Differences of Colony_Siz*alpha*gfp Least Squares Means													
Colony_Size	alpha	gfp	_Colony_Size	_alpha	_gfp	Estimate	Standard Error	DF	t Value	Pr > |t|	Alpha	Lower	Upper
Micro	minu	minus	Small	high	low	-8.5837	9.9490	28	-0.86	0.3956	0.05	-28.9633	11.7960
Micro	minu	minus	Small	low	high	-8.4448	9.9492	28	-0.85	0.4032	0.05	-28.8248	11.9351
Micro	minu	minus	Small	low	low	-7.8528	9.9500	28	-0.79	0.4366	0.05	-28.2345	12.5290
Micro	minu	minus	Small	minu	minus	-0.00004	14.0686	28	-0.00	1.0000	0.05	-28.8183	28.8182
Micro	minu	minus	Small	minu	plus	-0.00008	14.0685	28	-0.00	1.0000	0.05	-28.8180	28.8179
Micro	minu	minus	Small	plus	minus	-9.2294	9.9486	28	-0.93	0.3615	0.05	-29.6081	11.1494
Micro	minu	plus	Micro	plus	minus	-1.7842	0.2304	28	-7.74	<.0001	0.05	-2.2562	-1.3122
Micro	minu	plus	Small	high	high	-0.3430	0.2788	28	-1.23	0.2288	0.05	-0.9141	0.2280
Micro	minu	plus	Small	high	low	-0.8980	0.2529	28	-3.55	0.0014	0.05	-1.4162	-0.3799
Micro	minu	plus	Small	low	high	-0.7592	0.2583	28	-2.94	0.0065	0.05	-1.2884	-0.2300
Micro	minu	plus	Small	low	low	-0.1671	0.2897	28	-0.58	0.5686	0.05	-0.7606	0.4263
Micro	minu	plus	Small	minu	minus	7.6856	9.9503	28	0.77	0.4464	0.05	-12.6967	28.0679
Micro	minu	plus	Small	minu	plus	7.6856	9.9499	28	0.77	0.4463	0.05	-12.6958	28.0669
Micro	minu	plus	Small	plus	minus	-1.5437	0.2349	28	-6.57	<.0001	0.05	-2.0249	-1.0626
Micro	plus	minus	Small	high	high	1.4412	0.1997	28	7.22	<.0001	0.05	1.0320	1.8503
Micro	plus	minus	Small	high	low	0.8861	0.1617	28	5.48	<.0001	0.05	0.5549	1.2174
Micro	plus	minus	Small	low	high	1.0250	0.1700	28	6.03	<.0001	0.05	0.6767	1.3733
Micro	plus	minus	Small	low	low	1.6170	0.2147	28	7.53	<.0001	0.05	1.1772	2.0569
Micro	plus	minus	Small	minu	minus	9.4698	9.9482	28	0.95	0.3493	0.05	-10.9081	29.8477
Micro	plus	minus	Small	minu	plus	9.4697	9.9481	28	0.95	0.3493	0.05	-10.9080	29.8475
Micro	plus	minus	Small	plus	minus	0.2404	0.1317	28	1.83	0.0786	0.05	-0.02933	0.5102
Small	high	high	Small	high	low	-0.5550	0.2253	28	-2.46	0.0202	0.05	-1.0166	-0.09341
Small	high	high	Small	low	high	-0.4161	0.2314	28	-1.80	0.0829	0.05	-0.8901	0.05783
Small	high	high	Small	low	low	0.1759	0.2659	28	0.66	0.5138	0.05	-0.3689	0.7207

The GLIMMIX Procedure

Differences of Colony_Siz*alpha*gfp Least Squares Means													
Colony_Size	alpha	gfp	_Colony_Size	_alpha	_gfp	Estimate	Standard Error	DF	t Value	Pr > |t|	Alpha	Lower	Upper
Small	high	high	Small	minu	minus	8.0286	9.9494	28	0.81	0.4265	0.05	-12.3518	28.4091
Small	high	high	Small	minu	plus	8.0286	9.9493	28	0.81	0.4265	0.05	-12.3517	28.4088
Small	high	high	Small	plus	minus	-1.2007	0.2049	28	-5.86	<.0001	0.05	-1.6204	-0.7811
Small	high	low	Small	low	high	0.1389	0.1995	28	0.70	0.4921	0.05	-0.2698	0.5475
Small	high	low	Small	low	low	0.7309	0.2387	28	3.06	0.0048	0.05	0.2419	1.2199
Small	high	low	Small	minu	minus	8.5836	9.9487	28	0.86	0.3956	0.05	-11.7954	28.9627
Small	high	low	Small	minu	plus	8.5836	9.9486	28	0.86	0.3956	0.05	-11.7953	28.9624
Small	high	low	Small	plus	minus	-0.6457	0.1680	28	-3.84	0.0006	0.05	-0.9899	-0.3015
Small	low	high	Small	low	low	0.5920	0.2444	28	2.42	0.0222	0.05	0.09136	1.0927
Small	low	high	Small	minu	minus	8.4448	9.9489	28	0.85	0.4032	0.05	-11.9346	28.8241
Small	low	high	Small	minu	plus	8.4447	9.9488	28	0.85	0.4032	0.05	-11.9344	28.8238
Small	low	high	Small	plus	minus	-0.7846	0.1760	28	-4.46	0.0001	0.05	-1.1452	-0.4240
Small	low	low	Small	minu	minus	7.8527	9.9497	28	0.79	0.4366	0.05	-12.5284	28.2338
Small	low	low	Small	minu	plus	7.8527	9.9496	28	0.79	0.4366	0.05	-12.5282	28.2336
Small	low	low	Small	plus	minus	-1.3766	0.2195	28	-6.27	<.0001	0.05	-1.8262	-0.9270
Small	minu	minus	Small	minu	plus	-0.00004	14.0683	28	-0.00	1.0000	0.05	-28.8177	28.8177
Small	minu	minus	Small	plus	minus	-9.2293	9.9483	28	-0.93	0.3615	0.05	-29.6074	11.1488
Small	minu	plus	Small	plus	minus	-9.2293	9.9482	28	-0.93	0.3615	0.05	-29.6073	11.1487

The Mixed Procedure

Model Information	
**Data Set**	WORK.FIG1E
**Dependent Variable**	log_agg
**Covariance Structure**	Variance Components
**Subject Effect**	Replicate
**Estimation Method**	REML
**Residual Variance Method**	Profile
**Fixed Effects SE Method**	Model-Based
**Degrees of Freedom Method**	Containment

Class Level Information		
**Class**	**Levels**	**Values**
**Replicate**	6	1 2 3 4 5 6
**Group**	2	A+GFP+ A+GFP-

Dimensions	
**Covariance Parameters**	3
**Columns in X**	6
**Columns in Z per Subject**	2
**Subjects**	6
**Max Obs per Subject**	16

Number of Observations	
**Number of Observations Read**	96
**Number of Observations Used**	69
**Number of Observations Not Used**	27

The Mixed Procedure

Iteration History			
Iteration	Evaluations	-2 Res Log Like	Criterion
0	1	113.09107027	
1	4	113.09107027	0.00000000

Convergence criteria met.

Estimated G matrix is not positive definite.

Covariance Parameter Estimates		
Cov Parm	Subject	Estimate
Intercept	Replicate	0
Days	Replicate	0
Residual		0.2177

Fit Statistics	
-2 Res Log Likelihood	113.1
AIC (Smaller is Better)	115.1
AICC (Smaller is Better)	115.2
BIC (Smaller is Better)	114.9

Solution for Fixed Effects						
Effect	Group	Estimate	Standard Error	DF	t Value	Pr > |t|
Intercept		3.2666	0.1387	4	23.56	<.0001
Days		0.1177	0.001539	4	76.45	<.0001
Group	A+GFP+	0.7968	0.2300	57	3.46	0.0010
Group	A+GFP-	0	.	.	.	.

The Mixed Procedure

Solution for Fixed Effects						
**Effect**	**Group**	**Estimate**	**Standard Error**	**DF**	**t Value**	**Pr > |t|**
**Days*Group**	A+GFP+	-0.07795	0.004513	57	-17.27	<.0001
**Days*Group**	A+GFP-	0	.	.	.	.

Type 3 Tests of Fixed Effects				
**Effect**	**Num DF**	**Den DF**	**F Value**	**Pr > F**
**Days**	1	4	1216.93	<.0001
**Group**	1	57	12.00	0.0010
**Days*Group**	1	57	298.39	<.0001

Estimates					
**Label**	**Estimate**	**Standard Error**	**DF**	**t Value**	**Pr > |t|**
**a+gfp- slope**	0.1177	0.001539	57	76.45	<.0001
**a+gfp+ slope**	0.03973	0.004242	57	9.37	<.0001
**slope comparison**	-0.07795	0.004513	57	-17.27	<.0001

The GLIMMIX Procedure

Model Information	
**Data Set**	WORK.FIG1II
**Response Variable**	Count
**Response Distribution**	Poisson
**Link Function**	Log
**Variance Function**	Default
**Variance Matrix Blocked By**	Dish_ID
**Estimation Technique**	Maximum Likelihood
**Likelihood Approximation**	Laplace
**Degrees of Freedom Method**	Containment

Class Level Information		
**Class**	**Levels**	**Values**
**Colony_Size**	3	Large Micro Small
**Nestingfp**	2	minus plus
**Beta**	2	minus plus
**Dish_ID**	12	1 2 3 4 5 6 7 8 9 10 11 12

**Number of Observations Read**	36
**Number of Observations Used**	36

Dimensions	
**G-side Cov. Parameters**	1
**Columns in X**	36
**Columns in Z per Subject**	1
**Subjects (Blocks in V)**	12
**Max Obs per Subject**	3

The GLIMMIX Procedure

Optimization Information	
**Optimization Technique**	Dual Quasi-Newton
**Parameters in Optimization**	13
**Lower Boundaries**	1
**Upper Boundaries**	0
**Fixed Effects**	Not Profiled
**Starting From**	GLM estimates

Iteration History

Iteration	Restarts	Evaluations	Objective Function	Change	Max Gradient
**0**	**0**	4	139.59120981	.	397.8574
**1**	**0**	3	137.76887817	1.82233164	0.114836
**2**	**0**	4	137.76887468	0.00000350	0.055521

Convergence criterion (GCONV=1E-8) satisfied.

Estimated G matrix is not positive definite.

Fit Statistics	
**-2 Log Likelihood**	137.77
**AIC (smaller is better)**	161.77
**AICC (smaller is better)**	175.33
**BIC (smaller is better)**	167.59
**CAIC (smaller is better)**	179.59
**HQIC (smaller is better)**	159.61

The GLIMMIX Procedure

Fit Statistics for Conditional Distribution	
-2 log L(Count | r. effects)	137.77
Pearson Chi-Square	19.76
Pearson Chi-Square / DF	0.55

Covariance Parameter Estimates			
Cov Parm	Subject	Estimate	Standard Error
Intercept	Dish_ID	0	.

Solutions for Fixed Effects								
Effect	Colony_Size	Nestingfp	Beta	Estimate	Standard Error	DF	t Value	Pr > |t|
Intercept				4.0134	0.07762	8	51.71	<.0001
Colony_Size	Large			-2.1163	0.2367	16	-8.94	<.0001
Colony_Size	Micro			-0.1422	0.1139	16	-1.25	0.2298
Colony_Size	Small			0	.	.	.	.
Nestingfp		minus		-9.7065	9.9473	16	-0.98	0.3437
Nestingfp		plus		0	.	.	.	.
Colony_Siz*Nestingfp	Large	minus		2.1163	14.0693	16	0.15	0.8823
Colony_Siz*Nestingfp	Large	plus		0	.	.	.	.
Colony_Siz*Nestingfp	Micro	minus		9.8547	9.9480	16	0.99	0.3366
Colony_Siz*Nestingfp	Micro	plus		0	.	.	.	.
Colony_Siz*Nestingfp	Small	minus		0	.	.	.	.
Colony_Siz*Nestingfp	Small	plus		0	.	.	.	.
Beta			minus	-9.7065	9.9473	16	-0.98	0.3437
Beta			plus	0	.	.	.	.

The GLIMMIX Procedure

Solutions for Fixed Effects								
Effect	Colony_Size	Nestingfp	Beta	Estimate	Standard Error	DF	t Value	Pr > |t|
Colony_Size*Beta	Large		minus	2.1163	14.0693	16	0.15	0.8823
Colony_Size*Beta	Large		plus	0	.	.	.	.
Colony_Size*Beta	Micro		minus	0.1422	14.0678	16	0.01	0.9921
Colony_Size*Beta	Micro		plus	0	.	.	.	.
Colony_Size*Beta	Small		minus	0	.	.	.	.
Colony_Size*Beta	Small		plus	0	.	.	.	.
Nestingfp*Beta		minus	minus	19.5994	14.0674	16	1.39	0.1826
Nestingfp*Beta		minus	plus	0	.	.	.	.
Nestingfp*Beta		plus	minus	0	.	.	.	.
Nestingfp*Beta		plus	plus	0	.	.	.	.
Colony_*Nesting*Beta	Large	minus	minus	-2.4310	19.8960	16	-0.12	0.9043
Colony_*Nesting*Beta	Large	minus	plus	0	.	.	.	.
Colony_*Nesting*Beta	Large	plus	minus	0	.	.	.	.
Colony_*Nesting*Beta	Large	plus	plus	0	.	.	.	.
Colony_*Nesting*Beta	Micro	minus	minus	-9.5809	17.2295	16	-0.56	0.5859
Colony_*Nesting*Beta	Micro	minus	plus	0	.	.	.	.
Colony_*Nesting*Beta	Micro	plus	minus	0	.	.	.	.
Colony_*Nesting*Beta	Micro	plus	plus	0	.	.	.	.
Colony_*Nesting*Beta	Small	minus	minus	0	.	.	.	.
Colony_*Nesting*Beta	Small	minus	plus	0	.	.	.	.
Colony_*Nesting*Beta	Small	plus	minus	0	.	.	.	.
Colony_*Nesting*Beta	Small	plus	plus	0	.	.	.	.

The GLIMMIX Procedure

Type III Tests of Fixed Effects				
Effect	Num DF	Den DF	F Value	Pr > F
Colony_Size	2	16	0.32	0.7332
Nestingfp	1	16	0.32	0.5823
Colony_Siz*Nestingfp	2	16	0.22	0.8055
Beta	1	16	0.10	0.7592
Colony_Size*Beta	2	16	0.27	0.7692
Nestingfp*Beta	1	16	4.42	0.0516
Colony_*Nesting*Beta	2	16	0.18	0.8335

Nestingfp*Beta Least Squares Means													
Nestingfp	Beta	Estimate	Standard Error	DF	t Value	Pr > |t|	Alpha	Lower	Upper	Mean	Standard Error Mean	Lower Mean	Upper Mean
minus	minus	4.1860	0.04170	16	100.38	<.0001	0.05	4.0976	4.2744	65.7615	2.7424	60.1975	71.8399
minus	plus	-2.4557	4.6891	16	-0.52	0.6077	0.05	-12.3962	7.4849	0.08581	0.4024	4.134E-6	1780.86
plus	minus	-5.6932	5.7429	16	-0.99	0.3363	0.05	-17.8676	6.4812	0.003369	0.01935	1.739E-8	652.77
plus	plus	3.2605	0.08365	16	38.98	<.0001	0.05	3.0832	3.4379	26.0636	2.1801	21.8285	31.1203

Differences of Nestingfp*Beta Least Squares Means											
Nestingfp	Beta	_Nestingfp	_Beta	Estimate	Standard Error	DF	t Value	Pr > |t|	Alpha	Lower	Upper
minus	minus	minus	plus	6.6417	4.6892	16	1.42	0.1758	0.05	-3.2990	16.5824
minus	minus	plus	minus	9.8792	5.7430	16	1.72	0.1047	0.05	-2.2953	22.0538
minus	minus	plus	plus	0.9255	0.09347	16	9.90	<.0001	0.05	0.7274	1.1236
minus	plus	plus	minus	3.2375	7.4141	16	0.44	0.6682	0.05	-12.4796	18.9547
minus	plus	plus	plus	-5.7162	4.6899	16	-1.22	0.2406	0.05	-15.6583	4.2259
plus	minus	plus	plus	-8.9537	5.7435	16	-1.56	0.1386	0.05	-21.1294	3.2220

The GLIMMIX Procedure

Colony_*Nesting*Beta Least Squares Means

Colony_Size	Nestingfp	Beta	Estimate	Standard Error	DF	t Value	Pr > |t|	Alpha	Lower	Upper	Mean	Standard Error Mean	Lower Mean	Upper Mean
Large	minus	minus	3.8849	0.08276	16	46.94	<.0001	0.05	3.7095	4.0604	48.6643	4.0276	40.8332	57.9971
Large	minus	plus	-5.6932	9.9471	16	-0.57	0.5750	0.05	-26.7801	15.3937	0.003369	0.03351	2.34E-12	4846380
Large	plus	minus	-5.6932	9.9471	16	-0.57	0.5750	0.05	-26.7801	15.3937	0.003369	0.03351	2.34E-12	4846316
Large	plus	plus	1.8971	0.2236	16	8.48	<.0001	0.05	1.4231	2.3711	6.6665	1.4907	4.1498	10.7094
Micro	minus	minus	4.4735	0.06166	16	72.55	<.0001	0.05	4.3428	4.6042	87.6624	5.4056	76.9204	99.9044
Micro	minus	plus	4.0193	0.07738	16	51.94	<.0001	0.05	3.8553	4.1834	55.6644	4.3075	47.2426	65.5875
Micro	plus	minus	-5.6932	9.9472	16	-0.57	0.5750	0.05	-26.7802	15.3939	0.003369	0.03351	2.34E-12	4846934
Micro	plus	plus	3.8712	0.08333	16	46.45	<.0001	0.05	3.6945	4.0478	47.9986	3.9999	40.2259	57.2732
Small	minus	minus	4.1997	0.07071	16	59.39	<.0001	0.05	4.0498	4.3496	66.6641	4.7138	57.3841	77.4448
Small	minus	plus	-5.6932	9.9470	16	-0.57	0.5750	0.05	-26.7799	15.3936	0.003369	0.03351	2.34E-12	4845532
Small	plus	minus	-5.6932	9.9470	16	-0.57	0.5750	0.05	-26.7798	15.3935	0.003369	0.03351	2.34E-12	4845154
Small	plus	plus	4.0134	0.07762	16	51.71	<.0001	0.05	3.8488	4.1779	55.3321	4.2946	46.9375	65.2281

The GLIMMIX Procedure

Differences of Colony_*Nesting*Beta Least Squares Means													
Colony_Size	Nestingfp	Beta	_Colony_Size	_Nestingfp	_Beta	Estimate	Standard Error	DF	t Value	Pr > |t|	Alpha	Lower	Upper
Large	minus	minus	Large	minus	plus	9.5781	9.9474	16	0.96	0.3499	0.05	-11.5095	30.6658
Large	minus	minus	Large	plus	minus	9.5781	9.9474	16	0.96	0.3499	0.05	-11.5095	30.6658
Large	minus	minus	Large	plus	plus	1.9879	0.2384	16	8.34	<.0001	0.05	1.4824	2.4933
Large	minus	minus	Micro	minus	minus	-0.5885	0.1032	16	-5.70	<.0001	0.05	-0.8073	-0.3698
Large	minus	minus	Micro	minus	plus	-0.1344	0.1133	16	-1.19	0.2529	0.05	-0.3746	0.1058
Large	minus	minus	Micro	plus	minus	9.5781	9.9475	16	0.96	0.3499	0.05	-11.5096	30.6659
Large	minus	minus	Micro	plus	plus	0.01377	0.1174	16	0.12	0.9081	0.05	-0.2352	0.2628
Large	minus	minus	Small	minus	minus	-0.3147	0.1089	16	-2.89	0.0106	0.05	-0.5455	-0.08396
Large	minus	minus	Small	minus	plus	9.5781	9.9472	16	0.96	0.3499	0.05	-11.5090	30.6653
Large	minus	minus	Small	plus	minus	9.5781	9.9472	16	0.96	0.3499	0.05	-11.5090	30.6652
Large	minus	minus	Small	plus	plus	-0.1284	0.1135	16	-1.13	0.2744	0.05	-0.3689	0.1121
Large	minus	plus	Large	plus	minus	-1.38E-6	14.0673	16	-0.00	1.0000	0.05	-29.8214	29.8214
Large	minus	plus	Large	plus	plus	-7.5903	9.9496	16	-0.76	0.4566	0.05	-28.6825	13.5020
Large	minus	plus	Micro	minus	minus	-10.1667	9.9473	16	-1.02	0.3220	0.05	-31.2540	10.9207
Large	minus	plus	Micro	minus	plus	-9.7125	9.9474	16	-0.98	0.3434	0.05	-30.8001	11.3750
Large	minus	plus	Micro	plus	minus	-4.92E-7	14.0674	16	-0.00	1.0000	0.05	-29.8215	29.8215
Large	minus	plus	Micro	plus	plus	-9.5644	9.9475	16	-0.96	0.3506	0.05	-30.6520	11.5233
Large	minus	plus	Small	minus	minus	-9.8929	9.9474	16	-0.99	0.3348	0.05	-30.9803	11.1946
Large	minus	plus	Small	minus	plus	-8.01E-6	14.0674	16	-0.00	1.0000	0.05	-29.8215	29.8215
Large	minus	plus	Small	plus	minus	-9.38E-6	14.0672	16	-0.00	1.0000	0.05	-29.8212	29.8212
Large	minus	plus	Small	plus	plus	-9.7065	9.9474	16	-0.98	0.3437	0.05	-30.7941	11.3810
Large	plus	minus	Large	plus	plus	-7.5903	9.9496	16	-0.76	0.4566	0.05	-28.6825	13.5020
Large	plus	minus	Micro	minus	minus	-10.1667	9.9473	16	-1.02	0.3220	0.05	-31.2540	10.9206
Large	plus	minus	Micro	minus	plus	-9.7125	9.9474	16	-0.98	0.3434	0.05	-30.8001	11.3750

The GLIMMIX Procedure

Differences of Colony_*Nesting*Beta Least Squares Means													
Colony_Size	Nestingfp	Beta	_Colony_Size	_Nestingfp	_Beta	Estimate	Standard Error	DF	t Value	Pr > |t|	Alpha	Lower	Upper
Large	plus	minus	Micro	plus	minus	8.828E-7	14.0674	16	0.00	1.0000	0.05	-29.8215	29.8215
Large	plus	minus	Micro	plus	plus	-9.5644	9.9475	16	-0.96	0.3506	0.05	-30.6520	11.5233
Large	plus	minus	Small	minus	minus	-9.8929	9.9474	16	-0.99	0.3348	0.05	-30.9803	11.1946
Large	plus	minus	Small	minus	plus	-6.63E-6	14.0673	16	-0.00	1.0000	0.05	-29.8213	29.8213
Large	plus	minus	Small	plus	minus	-8.01E-6	14.0673	16	-0.00	1.0000	0.05	-29.8214	29.8214
Large	plus	minus	Small	plus	plus	-9.7065	9.9474	16	-0.98	0.3437	0.05	-30.7941	11.3810
Large	plus	plus	Micro	minus	minus	-2.5764	0.2320	16	-11.11	<.0001	0.05	-3.0681	-2.0847
Large	plus	plus	Micro	minus	plus	-2.1222	0.2366	16	-8.97	<.0001	0.05	-2.6239	-1.6206
Large	plus	plus	Micro	plus	minus	7.5903	9.9497	16	0.76	0.4566	0.05	-13.5021	28.6826
Large	plus	plus	Micro	plus	plus	-1.9741	0.2386	16	-8.27	<.0001	0.05	-2.4800	-1.4682
Large	plus	plus	Small	minus	minus	-2.3026	0.2345	16	-9.82	<.0001	0.05	-2.7997	-1.8054
Large	plus	plus	Small	minus	plus	7.5903	9.9495	16	0.76	0.4566	0.05	-13.5018	28.6823
Large	plus	plus	Small	plus	minus	7.5903	9.9495	16	0.76	0.4566	0.05	-13.5017	28.6823
Large	plus	plus	Small	plus	plus	-2.1163	0.2367	16	-8.94	<.0001	0.05	-2.6180	-1.6145
Micro	minus	minus	Micro	minus	plus	0.4542	0.09895	16	4.59	0.0003	0.05	0.2444	0.6639
Micro	minus	minus	Micro	plus	minus	10.1667	9.9472	16	1.02	0.3220	0.05	-10.9205	31.2538
Micro	minus	minus	Micro	plus	plus	0.6023	0.1037	16	5.81	<.0001	0.05	0.3826	0.8221
Micro	minus	minus	Small	minus	minus	0.2738	0.09382	16	2.92	0.0100	0.05	0.07493	0.4727
Micro	minus	minus	Small	minus	plus	10.1667	9.9471	16	1.02	0.3220	0.05	-10.9202	31.2535
Micro	minus	minus	Small	plus	minus	10.1667	9.9472	16	1.02	0.3220	0.05	-10.9204	31.2537
Micro	minus	minus	Small	plus	plus	0.4601	0.09913	16	4.64	0.0003	0.05	0.2500	0.6703
Micro	minus	plus	Micro	plus	minus	9.7125	9.9475	16	0.98	0.3434	0.05	-11.3752	30.8002
Micro	minus	plus	Micro	plus	plus	0.1482	0.1137	16	1.30	0.2110	0.05	-0.09291	0.3892
Micro	minus	plus	Small	minus	minus	-0.1803	0.1048	16	-1.72	0.1047	0.05	-0.4025	0.04189

The GLIMMIX Procedure

Differences of Colony_*Nesting*Beta Least Squares Means

Colony_Size	Nestingfp	Beta	_Colony_Size	_Nestingfp	_Beta	Estimate	Standard Error	DF	t Value	Pr > |t|	Alpha	Lower	Upper
Micro	minus	plus	Small	minus	plus	9.7125	9.9473	16	0.98	0.3434	0.05	-11.3749	30.7999
Micro	minus	plus	Small	plus	minus	9.7125	9.9473	16	0.98	0.3434	0.05	-11.3748	30.7998
Micro	minus	plus	Small	plus	plus	0.005987	0.1096	16	0.05	0.9571	0.05	-0.2264	0.2383
Micro	plus	minus	Micro	plus	plus	-9.5644	9.9475	16	-0.96	0.3506	0.05	-30.6521	11.5234
Micro	plus	minus	Small	minus	minus	-9.8929	9.9474	16	-0.99	0.3348	0.05	-30.9804	11.1947
Micro	plus	minus	Small	minus	plus	-7.51E-6	14.0673	16	-0.00	1.0000	0.05	-29.8213	29.8213
Micro	plus	minus	Small	plus	minus	-8.89E-6	14.0674	16	-0.00	1.0000	0.05	-29.8215	29.8215
Micro	plus	minus	Small	plus	plus	-9.7065	9.9475	16	-0.98	0.3437	0.05	-30.7942	11.3811
Micro	plus	plus	Small	minus	minus	-0.3285	0.1093	16	-3.01	0.0084	0.05	-0.5602	-0.09681
Micro	plus	plus	Small	minus	plus	9.5643	9.9474	16	0.96	0.3506	0.05	-11.5231	30.6518
Micro	plus	plus	Small	plus	minus	9.5643	9.9473	16	0.96	0.3506	0.05	-11.5231	30.6518
Micro	plus	plus	Small	plus	plus	-0.1422	0.1139	16	-1.25	0.2298	0.05	-0.3836	0.09923
Small	minus	minus	Small	minus	plus	9.8928	9.9471	16	0.99	0.3348	0.05	-11.1941	30.9798
Small	minus	minus	Small	plus	minus	9.8928	9.9471	16	0.99	0.3348	0.05	-11.1940	30.9797
Small	minus	minus	Small	plus	plus	0.1863	0.1050	16	1.77	0.0950	0.05	-0.03627	0.4089
Small	minus	plus	Small	plus	minus	-1.38E-6	14.0671	16	-0.00	1.0000	0.05	-29.8210	29.8210
Small	minus	plus	Small	plus	plus	-9.7065	9.9473	16	-0.98	0.3437	0.05	-30.7939	11.3809
Small	plus	minus	Small	plus	plus	-9.7065	9.9473	16	-0.98	0.3437	0.05	-30.7938	11.3808

The GLIMMIX Procedure

Model Information	
**Data Set**	WORK.FIG1III
**Response Variable**	Count
**Response Distribution**	Poisson
**Link Function**	Log
**Variance Function**	Default
**Variance Matrix**	Diagonal
**Estimation Technique**	Maximum Likelihood
**Degrees of Freedom Method**	Residual

Class Level Information		
**Class**	**Levels**	**Values**
**Nestingfp**	2	minus plus
**Beta**	2	minus plus
**Dish_ID**	12	1 2 3 4 5 6 7 8 9 10 11 12

**Number of Observations Read**	12
**Number of Observations Used**	12

Dimensions	
**Columns in X**	9
**Columns in Z**	0
**Subjects (Blocks in V)**	1
**Max Obs per Subject**	12

The GLIMMIX Procedure

Optimization Information	
**Optimization Technique**	Newton-Raphson
**Parameters in Optimization**	4
**Lower Boundaries**	0
**Upper Boundaries**	0
**Fixed Effects**	Not Profiled

Iteration History					
**Iteration**	**Restarts**	**Evaluations**	**Objective Function**	**Change**	**Max Gradient**
**0**	**0**	4	25.403133927	.	2.863996
**1**	**0**	3	25.032772266	0.37036166	0.223947
**2**	**0**	3	24.90443812	0.12833415	0.074692
**3**	**0**	3	24.857230976	0.04720714	0.027473
**4**	**0**	3	24.839864438	0.01736654	0.010107
**5**	**0**	3	24.833475646	0.00638879	0.003718
**6**	**0**	3	24.83112534	0.00235031	0.001368
**7**	**0**	3	24.830260711	0.00086463	0.000503
**8**	**0**	3	24.829942632	0.00031808	0.000185
**9**	**0**	3	24.829825617	0.00011701	0.000068
**10**	**0**	3	24.82978257	0.00004305	0.000025
**11**	**0**	3	24.829766734	0.00001584	9.216E-6

Convergence criterion (ABSGCONV=0.00001) satisfied.

The GLIMMIX Procedure

Fit Statistics	
-2 Log Likelihood	49.66
AIC (smaller is better)	57.66
AICC (smaller is better)	63.37
BIC (smaller is better)	59.60
CAIC (smaller is better)	63.60
HQIC (smaller is better)	56.94
Pearson Chi-Square	4.81
Pearson Chi-Square / DF	0.40

Parameter Estimates

Effect	Nestingfp	Beta	Estimate	Standard Error	DF	t Value	Pr > |t|
Intercept			4.4308	0.06299	8	70.34	<.0001
Nestingfp	minus		-0.7758	0.1122	8	-6.91	0.0001
Nestingfp	plus		0	.	.	.	.
Beta		minus	-3.0445	0.2955	8	-10.30	<.0001
Beta		plus	0	.	.	.	.
Nestingfp*Beta	minus	minus	-13.3036	329.40	8	-0.04	0.9688
Nestingfp*Beta	minus	plus	0	.	.	.	.
Nestingfp*Beta	plus	minus	0	.	.	.	.
Nestingfp*Beta	plus	plus	0	.	.	.	.

The GLIMMIX Procedure

Type III Tests of Fixed Effects				
Effect	Num DF	Den DF	F Value	Pr > F
Nestingfp	1	8	0.00	0.9651
Beta	1	8	0.00	0.9545
Nestingfp*Beta	1	8	0.00	0.9688

Nestingfp*Beta Least Squares Means													
Nestingfp	Beta	Estimate	Standard Error	DF	t Value	Pr > |t|	Alpha	Lower	Upper	Mean	Standard Error Mean	Lower Mean	Upper Mean
minus	minus	-12.6931	329.40	8	-0.04	0.9702	0.05	-772.29	746.90	3.072E-6	0.001012	0	.
minus	plus	3.6550	0.09285	8	39.37	<.0001	0.05	3.4409	3.8691	38.6667	3.5901	31.2141	47.8985
plus	minus	1.3863	0.2887	8	4.80	0.0014	0.05	0.7206	2.0520	4.0000	1.1547	2.0557	7.7833
plus	plus	4.4308	0.06299	8	70.34	<.0001	0.05	4.2856	4.5761	84.0000	5.2915	72.6426	97.1330

Differences of Nestingfp*Beta Least Squares Means											
Nestingfp	Beta	_Nestingfp	_Beta	Estimate	Standard Error	DF	t Value	Pr > |t|	Alpha	Lower	Upper
minus	minus	minus	plus	-16.3481	329.40	8	-0.05	0.9616	0.05	-775.94	743.25
minus	minus	plus	minus	-14.0794	329.40	8	-0.04	0.9670	0.05	-773.67	745.51
minus	minus	plus	plus	-17.1240	329.40	8	-0.05	0.9598	0.05	-776.72	742.47
minus	plus	plus	minus	2.2687	0.3032	8	7.48	<.0001	0.05	1.5694	2.9680
minus	plus	plus	plus	-0.7758	0.1122	8	-6.91	0.0001	0.05	-1.0346	-0.5171
plus	minus	plus	plus	-3.0445	0.2955	8	-10.30	<.0001	0.05	-3.7259	-2.3632

The GLIMMIX Procedure

Model Information	
**Data Set**	WORK.FIG3AIV
**Response Variable**	Count
**Response Distribution**	Poisson
**Link Function**	Log
**Variance Function**	Default
**Variance Matrix Blocked By**	Dish_ID
**Estimation Technique**	Maximum Likelihood
**Likelihood Approximation**	Laplace
**Degrees of Freedom Method**	Containment

Class Level Information		
**Class**	**Levels**	**Values**
**Colony_Size**	3	Large Micro Small
**alpha**	2	minus plus
**Mesp1eYFP**	2	minus plus
**Dish_ID**	12	1 2 3 4 5 6 7 8 9 10 11 12

**Number of Observations Read**	36
**Number of Observations Used**	36

Dimensions	
**G-side Cov. Parameters**	1
**Columns in X**	36
**Columns in Z per Subject**	1
**Subjects (Blocks in V)**	12
**Max Obs per Subject**	3

The GLIMMIX Procedure

Optimization Information	
**Optimization Technique**	Dual Quasi-Newton
**Parameters in Optimization**	13
**Lower Boundaries**	1
**Upper Boundaries**	0
**Fixed Effects**	Not Profiled
**Starting From**	GLM estimates

Iteration History					
**Iteration**	**Restarts**	**Evaluations**	**Objective Function**	**Change**	**Max Gradient**
**0**	**0**	4	128.75296945	.	449.0222
**1**	**0**	3	127.50541543	1.24755402	0.080248
**2**	**0**	6	127.50540566	0.00000977	0.074001

Convergence criterion (GCONV=1E-8) satisfied.

Estimated G matrix is not positive definite.

Fit Statistics	
**-2 Log Likelihood**	127.51
**AIC (smaller is better)**	151.51
**AICC (smaller is better)**	165.07
**BIC (smaller is better)**	157.32
**CAIC (smaller is better)**	169.32
**HQIC (smaller is better)**	149.35

The GLIMMIX Procedure

Fit Statistics for Conditional Distribution	
-2 log L(Count | r. effects)	127.51
Pearson Chi-Square	7.38
Pearson Chi-Square / DF	0.21

Covariance Parameter Estimates			
Cov Parm	Subject	Estimate	Standard Error
Intercept	Dish_ID	0	.

Solutions for Fixed Effects								
Effect	Colony_Size	alpha	Mesp1eYFP	Estimate	Standard Error	DF	t Value	Pr > |t|
Intercept				3.8501	0.08422	8	45.72	<.0001
Colony_Size	Large			-0.3141	0.1296	16	-2.42	0.0276
Colony_Size	Micro			0.09465	0.1164	16	0.81	0.4280
Colony_Size	Small			0	.	.	.	.
alpha		minus		-2.6462	0.3273	16	-8.09	<.0001
alpha		plus		0	.	.	.	.
Colony_Size*alpha	Large	minus		-6.5831	9.9535	16	-0.66	0.5178
Colony_Size*alpha	Large	plus		0	.	.	.	.
Colony_Size*alpha	Micro	minus		0.5472	0.4077	16	1.34	0.1983
Colony_Size*alpha	Micro	plus		0	.	.	.	.
Colony_Size*alpha	Small	minus		0	.	.	.	.
Colony_Size*alpha	Small	plus		0	.	.	.	.
Mesp1eYFP			minus	-0.00004	0.1191	16	-0.00	0.9998
Mesp1eYFP			plus	0	.	.	.	.

The GLIMMIX Procedure

Solutions for Fixed Effects								
Effect	Colony_Size	alpha	Mesp1eYFP	Estimate	Standard Error	DF	t Value	Pr > |t|
Colony_Siz*Mesp1eYFP	Large		minus	-0.2403	0.1904	16	-1.26	0.2250
Colony_Siz*Mesp1eYFP	Large		plus	0	.	.	.	.
Colony_Siz*Mesp1eYFP	Micro		minus	-0.2313	0.1696	16	-1.36	0.1916
Colony_Siz*Mesp1eYFP	Micro		plus	0	.	.	.	.
Colony_Siz*Mesp1eYFP	Small		minus	0	.	.	.	.
Colony_Siz*Mesp1eYFP	Small		plus	0	.	.	.	.
alpha*Mesp1eYFP		minus	minus	-6.8972	9.9535	16	-0.69	0.4983
alpha*Mesp1eYFP		minus	plus	0	.	.	.	.
alpha*Mesp1eYFP		plus	minus	0	.	.	.	.
alpha*Mesp1eYFP		plus	plus	0	.	.	.	.
Colony*alpha*Mesp1eY	Large	minus	minus	7.1374	17.2341	16	0.41	0.6843
Colony*alpha*Mesp1eY	Large	minus	plus	0	.	.	.	.
Colony*alpha*Mesp1eY	Large	plus	minus	0	.	.	.	.
Colony*alpha*Mesp1eY	Large	plus	plus	0	.	.	.	.
Colony*alpha*Mesp1eY	Micro	minus	minus	-0.4106	14.0750	16	-0.03	0.9771
Colony*alpha*Mesp1eY	Micro	minus	plus	0	.	.	.	.
Colony*alpha*Mesp1eY	Micro	plus	minus	0	.	.	.	.
Colony*alpha*Mesp1eY	Micro	plus	plus	0	.	.	.	.
Colony*alpha*Mesp1eY	Small	minus	minus	0	.	.	.	.
Colony*alpha*Mesp1eY	Small	minus	plus	0	.	.	.	.
Colony*alpha*Mesp1eY	Small	plus	minus	0	.	.	.	.
Colony*alpha*Mesp1eY	Small	plus	plus	0	.	.	.	.

The GLIMMIX Procedure

Type III Tests of Fixed Effects				
**Effect**	**Num DF**	**Den DF**	**F Value**	**Pr > F**
**Colony_Size**	2	16	0.13	0.8770
**alpha**	1	16	4.44	0.0513
**Colony_Size*alpha**	2	16	0.08	0.9206
**Mesp1eYFP**	1	16	0.56	0.4647
**Colony_Siz*Mesp1eYFP**	2	16	0.10	0.9022
**alpha*Mesp1eYFP**	1	16	0.49	0.4929
**Colony*alpha*Mesp1eY**	2	16	0.11	0.8971

alpha*Mesp1eYFP Least Squares Means													
**alpha**	**Mesp1eYFP**	**Estimate**	**Standard Error**	**DF**	**t Value**	**Pr > |t|**	**Alpha**	**Lower**	**Upper**	**Mean**	**Standard Error Mean**	**Lower Mean**	**Upper Mean**
minus	minus	-5.6934	5.7434	16	-0.99	0.3363	0.05	-17.8689	6.4822	0.003368	0.01935	1.736E-8	653.41
minus	plus	-0.8812	3.3184	16	-0.27	0.7940	0.05	-7.9160	6.1535	0.4143	1.3747	0.000365	470.37
plus	minus	3.6197	0.05535	16	65.40	<.0001	0.05	3.5024	3.7371	37.3281	2.0660	33.1954	41.9752
plus	plus	3.7770	0.05083	16	74.31	<.0001	0.05	3.6692	3.8847	43.6832	2.2204	39.2208	48.6533

Differences of alpha*Mesp1eYFP Least Squares Means											
**alpha**	**Mesp1eYFP**	**_alpha**	**_Mesp1eYFP**	**Estimate**	**Standard Error**	**DF**	**t Value**	**Pr > |t|**	**Alpha**	**Lower**	**Upper**
minus	minus	minus	plus	-4.8121	6.6332	16	-0.73	0.4786	0.05	-18.8738	9.2496
minus	minus	plus	minus	-9.3131	5.7437	16	-1.62	0.1245	0.05	-21.4892	2.8630
minus	minus	plus	plus	-9.4703	5.7437	16	-1.65	0.1187	0.05	-21.6464	2.7057
minus	plus	plus	minus	-4.5010	3.3189	16	-1.36	0.1939	0.05	-11.5367	2.5347
minus	plus	plus	plus	-4.6582	3.3188	16	-1.40	0.1796	0.05	-11.6938	2.3774
plus	minus	plus	plus	-0.1572	0.07515	16	-2.09	0.0527	0.05	-0.3165	0.002087

The SAS System

The GLIMMIX Procedure

Colony*alpha*Mesp1eY Least Squares Means														
Colony_Size	alpha	Mesp1eYFP	Estimate	Standard Error	DF	t Value	Pr > |t|	Alpha	Lower	Upper	Mean	Standard Error Mean	Lower Mean	Upper Mean
Large	minus	minus	-5.6934	9.9481	16	-0.57	0.5751	0.05	-26.7825	15.3957	0.003368	0.03351	2.34E-12	4855665
Large	minus	plus	-5.6933	9.9476	16	-0.57	0.5751	0.05	-26.7813	15.3947	0.003368	0.03351	2.34E-12	4851014
Large	plus	minus	3.2957	0.1111	16	29.66	<.0001	0.05	3.0602	3.5313	26.9966	2.9998	21.3308	34.1674
Large	plus	plus	3.5360	0.09854	16	35.89	<.0001	0.05	3.3272	3.7449	34.3308	3.3828	27.8589	42.3060
Micro	minus	minus	-5.6934	9.9481	16	-0.57	0.5751	0.05	-26.7824	15.3956	0.003368	0.03351	2.34E-12	4855427
Micro	minus	plus	1.8457	0.2294	16	8.04	<.0001	0.05	1.3593	2.3321	6.3325	1.4529	3.8936	10.2992
Micro	plus	minus	3.7135	0.09017	16	41.18	<.0001	0.05	3.5223	3.9046	40.9955	3.6966	33.8624	49.6311
Micro	plus	plus	3.9448	0.08032	16	49.11	<.0001	0.05	3.7745	4.1150	51.6635	4.1498	43.5745	61.2542
Small	minus	minus	-5.6933	9.9477	16	-0.57	0.5751	0.05	-26.7816	15.3949	0.003368	0.03351	2.34E-12	4852122
Small	minus	plus	1.2039	0.3162	16	3.81	0.0016	0.05	0.5335	1.8743	3.3330	1.0540	1.7048	6.5161
Small	plus	minus	3.8501	0.08422	16	45.72	<.0001	0.05	3.6715	4.0286	46.9959	3.9579	39.3118	56.1820
Small	plus	plus	3.8501	0.08422	16	45.72	<.0001	0.05	3.6716	4.0286	46.9977	3.9580	39.3135	56.1839

The GLIMMIX Procedure

Differences of Colony*alpha*Mesp1eY Least Squares Means													
Colony_Size	alpha	Mesp1eYFP	_Colony_Size	_alpha	_Mesp1eYFP	Estimate	Standard Error	DF	t Value	Pr > |t|	Alpha	Lower	Upper
Large	minus	minus	Large	minus	plus	-0.00010	14.0684	16	-0.00	1.0000	0.05	-29.8238	29.8236
Large	minus	minus	Large	plus	minus	-8.9891	9.9487	16	-0.90	0.3796	0.05	-30.0795	12.1013
Large	minus	minus	Large	plus	plus	-9.2294	9.9486	16	-0.93	0.3673	0.05	-30.3195	11.8606
Large	minus	minus	Micro	minus	minus	-0.00003	14.0687	16	-0.00	1.0000	0.05	-29.8244	29.8244
Large	minus	minus	Micro	minus	plus	-7.5391	9.9508	16	-0.76	0.4597	0.05	-28.6338	13.5556
Large	minus	minus	Micro	plus	minus	-9.4069	9.9485	16	-0.95	0.3584	0.05	-30.4968	11.6831
Large	minus	minus	Micro	plus	plus	-9.6382	9.9484	16	-0.97	0.3471	0.05	-30.7279	11.4516
Large	minus	minus	Small	minus	minus	-0.00008	14.0685	16	-0.00	1.0000	0.05	-29.8239	29.8238
Large	minus	minus	Small	minus	plus	-6.8973	9.9531	16	-0.69	0.4983	0.05	-27.9970	14.2024
Large	minus	minus	Small	plus	minus	-9.5435	9.9485	16	-0.96	0.3517	0.05	-30.6333	11.5464
Large	minus	minus	Small	plus	plus	-9.5435	9.9485	16	-0.96	0.3517	0.05	-30.6333	11.5463
Large	minus	plus	Large	plus	minus	-8.9890	9.9482	16	-0.90	0.3796	0.05	-30.0783	12.1003
Large	minus	plus	Large	plus	plus	-9.2293	9.9481	16	-0.93	0.3673	0.05	-30.3184	11.8597
Large	minus	plus	Micro	minus	minus	0.000075	14.0684	16	0.00	1.0000	0.05	-29.8236	29.8237
Large	minus	plus	Micro	minus	plus	-7.5390	9.9503	16	-0.76	0.4597	0.05	-28.6326	13.5546
Large	minus	plus	Micro	plus	minus	-9.4068	9.9480	16	-0.95	0.3584	0.05	-30.4956	11.6821
Large	minus	plus	Micro	plus	plus	-9.6381	9.9479	16	-0.97	0.3470	0.05	-30.7267	11.4506
Large	minus	plus	Small	minus	minus	0.000025	14.0681	16	0.00	1.0000	0.05	-29.8231	29.8231
Large	minus	plus	Small	minus	plus	-6.8972	9.9526	16	-0.69	0.4982	0.05	-27.9958	14.2015
Large	minus	plus	Small	plus	minus	-9.5434	9.9480	16	-0.96	0.3517	0.05	-30.6321	11.5454
Large	minus	plus	Small	plus	plus	-9.5434	9.9480	16	-0.96	0.3517	0.05	-30.6322	11.5454
Large	plus	minus	Large	plus	plus	-0.2403	0.1485	16	-1.62	0.1252	0.05	-0.5552	0.07451
Large	plus	minus	Micro	minus	minus	8.9891	9.9487	16	0.90	0.3796	0.05	-12.1012	30.0794
Large	plus	minus	Micro	minus	plus	1.4500	0.2549	16	5.69	<.0001	0.05	0.9096	1.9904

The GLIMMIX Procedure

Differences of Colony*alpha*Mesp1eY Least Squares Means													
Colony_Size	alpha	Mesp1eYFP	_Colony_Size	_alpha	_Mesp1eYFP	Estimate	Standard Error	DF	t Value	Pr > |t|	Alpha	Lower	Upper
Large	plus	minus	Micro	plus	minus	-0.4177	0.1431	16	-2.92	0.0100	0.05	-0.7211	-0.1144
Large	plus	minus	Micro	plus	plus	-0.6490	0.1371	16	-4.73	0.0002	0.05	-0.9397	-0.3584
Large	plus	minus	Small	minus	minus	8.9890	9.9484	16	0.90	0.3796	0.05	-12.1005	30.0786
Large	plus	minus	Small	minus	plus	2.0918	0.3352	16	6.24	<.0001	0.05	1.3813	2.8024
Large	plus	minus	Small	plus	minus	-0.5543	0.1394	16	-3.98	0.0011	0.05	-0.8499	-0.2588
Large	plus	minus	Small	plus	plus	-0.5544	0.1394	16	-3.98	0.0011	0.05	-0.8500	-0.2588
Large	plus	plus	Micro	minus	minus	9.2294	9.9486	16	0.93	0.3673	0.05	-11.8606	30.3194
Large	plus	plus	Micro	minus	plus	1.6903	0.2497	16	6.77	<.0001	0.05	1.1610	2.2197
Large	plus	plus	Micro	plus	minus	-0.1774	0.1336	16	-1.33	0.2027	0.05	-0.4606	0.1057
Large	plus	plus	Micro	plus	plus	-0.4087	0.1271	16	-3.21	0.0054	0.05	-0.6782	-0.1392
Large	plus	plus	Small	minus	minus	9.2294	9.9482	16	0.93	0.3673	0.05	-11.8599	30.3187
Large	plus	plus	Small	minus	plus	2.3322	0.3312	16	7.04	<.0001	0.05	1.6300	3.0344
Large	plus	plus	Small	plus	minus	-0.3140	0.1296	16	-2.42	0.0276	0.05	-0.5888	-0.03923
Large	plus	plus	Small	plus	plus	-0.3141	0.1296	16	-2.42	0.0276	0.05	-0.5888	-0.03927
Micro	minus	minus	Micro	minus	plus	-7.5391	9.9507	16	-0.76	0.4597	0.05	-28.6337	13.5555
Micro	minus	minus	Micro	plus	minus	-9.4068	9.9485	16	-0.95	0.3584	0.05	-30.4967	11.6830
Micro	minus	minus	Micro	plus	plus	-9.6381	9.9484	16	-0.97	0.3471	0.05	-30.7278	11.4515
Micro	minus	minus	Small	minus	minus	-0.00005	14.0685	16	-0.00	1.0000	0.05	-29.8240	29.8239
Micro	minus	minus	Small	minus	plus	-6.8972	9.9531	16	-0.69	0.4983	0.05	-27.9969	14.2024
Micro	minus	minus	Small	plus	minus	-9.5434	9.9484	16	-0.96	0.3517	0.05	-30.6332	11.5463
Micro	minus	minus	Small	plus	plus	-9.5435	9.9484	16	-0.96	0.3517	0.05	-30.6332	11.5463
Micro	minus	plus	Micro	plus	minus	-1.8678	0.2465	16	-7.58	<.0001	0.05	-2.3903	-1.3452
Micro	minus	plus	Micro	plus	plus	-2.0991	0.2431	16	-8.64	<.0001	0.05	-2.6144	-1.5837
Micro	minus	plus	Small	minus	minus	7.5390	9.9504	16	0.76	0.4597	0.05	-13.5548	28.6329

The GLIMMIX Procedure

Differences of Colony*alpha*Mesp1eY Least Squares Means													
Colony_Size	alpha	Mesp1eYFP	_Colony_Size	_alpha	_Mesp1eYFP	Estimate	Standard Error	DF	t Value	Pr > |t|	Alpha	Lower	Upper
Micro	minus	plus	Small	minus	plus	0.6418	0.3907	16	1.64	0.1199	0.05	-0.1864	1.4701
Micro	minus	plus	Small	plus	minus	-2.0044	0.2444	16	-8.20	<.0001	0.05	-2.5225	-1.4863
Micro	minus	plus	Small	plus	plus	-2.0044	0.2444	16	-8.20	<.0001	0.05	-2.5225	-1.4863
Micro	plus	minus	Micro	plus	plus	-0.2313	0.1208	16	-1.92	0.0735	0.05	-0.4873	0.02471
Micro	plus	minus	Small	minus	minus	9.4068	9.9481	16	0.95	0.3584	0.05	-11.6823	30.4959
Micro	plus	minus	Small	minus	plus	2.5096	0.3288	16	7.63	<.0001	0.05	1.8125	3.2067
Micro	plus	minus	Small	plus	minus	-0.1366	0.1234	16	-1.11	0.2846	0.05	-0.3982	0.1250
Micro	plus	minus	Small	plus	plus	-0.1366	0.1234	16	-1.11	0.2845	0.05	-0.3982	0.1249
Micro	plus	plus	Small	minus	minus	9.6381	9.9481	16	0.97	0.3470	0.05	-11.4509	30.7270
Micro	plus	plus	Small	minus	plus	2.7409	0.3263	16	8.40	<.0001	0.05	2.0492	3.4326
Micro	plus	plus	Small	plus	minus	0.09469	0.1164	16	0.81	0.4278	0.05	-0.1520	0.3414
Micro	plus	plus	Small	plus	plus	0.09465	0.1164	16	0.81	0.4280	0.05	-0.1521	0.3414
Small	minus	minus	Small	minus	plus	-6.8972	9.9528	16	-0.69	0.4983	0.05	-27.9961	14.2017
Small	minus	minus	Small	plus	minus	-9.5434	9.9481	16	-0.96	0.3517	0.05	-30.6324	11.5456
Small	minus	minus	Small	plus	plus	-9.5434	9.9481	16	-0.96	0.3517	0.05	-30.6324	11.5456
Small	minus	plus	Small	plus	minus	-2.6462	0.3273	16	-8.09	<.0001	0.05	-3.3400	-1.9524
Small	minus	plus	Small	plus	plus	-2.6462	0.3273	16	-8.09	<.0001	0.05	-3.3400	-1.9525
Small	plus	minus	Small	plus	plus	-0.00004	0.1191	16	-0.00	0.9998	0.05	-0.2525	0.2524

The GLIMMIX Procedure

Model Information	
Data Set	WORK.FIG3BIV
Response Variable	Count
Response Distribution	Poisson
Link Function	Log
Variance Function	Default
Variance Matrix Blocked By	Dish_ID
Estimation Technique	Maximum Likelihood
Likelihood Approximation	Laplace
Degrees of Freedom Method	Containment

Class Level Information		
Class	Levels	Values
Colony_Size	3	Large Micro Small
alpha	2	minus plus
Wnt1eYFP	2	minus plus
Dish_ID	12	1 2 3 4 5 6 7 8 9 10 11 12

Number of Observations Read	36
Number of Observations Used	36

Dimensions	
G-side Cov. Parameters	1
Columns in X	36
Columns in Z per Subject	1
Subjects (Blocks in V)	12
Max Obs per Subject	3

The GLIMMIX Procedure

Optimization Information	
**Optimization Technique**	Dual Quasi-Newton
**Parameters in Optimization**	13
**Lower Boundaries**	1
**Upper Boundaries**	0
**Fixed Effects**	Not Profiled
**Starting From**	GLM estimates

Iteration History					
**Iteration**	**Restarts**	**Evaluations**	**Objective Function**	**Change**	**Max Gradient**
**0**	**0**	4	138.23682368	.	311.4114
**1**	**0**	3	136.5292135	1.70761018	0.062449
**2**	**0**	6	136.52920519	0.00000831	0.108836

Convergence criterion (GCONV=1E-8) satisfied.

Estimated G matrix is not positive definite.

Fit Statistics	
**-2 Log Likelihood**	136.53
**AIC (smaller is better)**	160.53
**AICC (smaller is better)**	174.09
**BIC (smaller is better)**	166.35
**CAIC (smaller is better)**	178.35
**HQIC (smaller is better)**	158.37

The GLIMMIX Procedure

Fit Statistics for Conditional Distribution	
-2 log L(Count | r. effects)	136.53
Pearson Chi-Square	9.13
Pearson Chi-Square / DF	0.25

Covariance Parameter Estimates			
Cov Parm	Subject	Estimate	Standard Error
Intercept	Dish_ID	0	.

Solutions for Fixed Effects								
Effect	Colony_Size	alpha	Wnt1eYFP	Estimate	Standard Error	DF	t Value	Pr > |t|
Intercept				3.7766	0.08737	8	43.23	<.0001
Colony_Size	Large			-0.3319	0.1352	16	-2.46	0.0259
Colony_Size	Micro			-0.1302	0.1278	16	-1.02	0.3233
Colony_Size	Small			0	.	.	.	.
alpha		minus		-9.4698	9.9477	16	-0.95	0.3553
alpha		plus		0	.	.	.	.
Colony_Size*alpha	Large	minus		0.3318	14.0688	16	0.02	0.9815
Colony_Size*alpha	Large	plus		0	.	.	.	.
Colony_Size*alpha	Micro	minus		0.1302	14.0684	16	0.01	0.9927
Colony_Size*alpha	Micro	plus		0	.	.	.	.
Colony_Size*alpha	Small	minus		0	.	.	.	.
Colony_Size*alpha	Small	plus		0	.	.	.	.
Wnt1eYFP			minus	0.000051	0.1236	16	0.00	0.9997
Wnt1eYFP			plus	0	.	.	.	.

The GLIMMIX Procedure

Solutions for Fixed Effects								
Effect	Colony_Size	alpha	Wnt1eYFP	Estimate	Standard Error	DF	t Value	Pr > |t|
Colony_Size*Wnt1eYFP	Large		minus	0.03140	0.1903	16	0.17	0.8710
Colony_Size*Wnt1eYFP	Large		plus	0	.	.	.	.
Colony_Size*Wnt1eYFP	Micro		minus	0.3791	0.1730	16	2.19	0.0435
Colony_Size*Wnt1eYFP	Micro		plus	0	.	.	.	.
Colony_Size*Wnt1eYFP	Small		minus	0	.	.	.	.
Colony_Size*Wnt1eYFP	Small		plus	0	.	.	.	.
alpha*Wnt1eYFP		minus	minus	8.7842	9.9489	16	0.88	0.3903
alpha*Wnt1eYFP		minus	plus	0	.	.	.	.
alpha*Wnt1eYFP		plus	minus	0	.	.	.	.
alpha*Wnt1eYFP		plus	plus	0	.	.	.	.
Colony*alpha*Wnt1eYF	Large	minus	minus	-8.8156	17.2314	16	-0.51	0.6159
Colony*alpha*Wnt1eYF	Large	minus	plus	0	.	.	.	.
Colony*alpha*Wnt1eYF	Large	plus	minus	0	.	.	.	.
Colony*alpha*Wnt1eYF	Large	plus	plus	0	.	.	.	.
Colony*alpha*Wnt1eYF	Micro	minus	minus	-0.8310	14.0703	16	-0.06	0.9536
Colony*alpha*Wnt1eYF	Micro	minus	plus	0	.	.	.	.
Colony*alpha*Wnt1eYF	Micro	plus	minus	0	.	.	.	.
Colony*alpha*Wnt1eYF	Micro	plus	plus	0	.	.	.	.
Colony*alpha*Wnt1eYF	Small	minus	minus	0	.	.	.	.
Colony*alpha*Wnt1eYF	Small	minus	plus	0	.	.	.	.
Colony*alpha*Wnt1eYF	Small	plus	minus	0	.	.	.	.
Colony*alpha*Wnt1eYF	Small	plus	plus	0	.	.	.	.

The GLIMMIX Procedure

Type III Tests of Fixed Effects				
Effect	Num DF	Den DF	F Value	Pr > F
Colony_Size	2	16	0.17	0.8425
alpha	1	16	3.88	0.0665
Colony_Size*alpha	2	16	0.13	0.8826
Wnt1eYFP	1	16	0.78	0.3914
Colony_Size*Wnt1eYFP	2	16	0.15	0.8589
alpha*Wnt1eYFP	1	16	0.70	0.4135
Colony*alpha*Wnt1eYF	2	16	0.14	0.8667

alpha*Wnt1eYFP Least Squares Means													
alpha	Wnt1eYFP	Estimate	Standard Error	DF	t Value	Pr > |t|	Alpha	Lower	Upper	Mean	Standard Error Mean	Lower Mean	Upper Mean
minus	minus	0.01222	3.3166	16	0.00	0.9971	0.05	-7.0187	7.0431	1.0123	3.3574	0.000895	1144.93
minus	plus	-5.6933	5.7432	16	-0.99	0.3363	0.05	-17.8683	6.4817	0.003369	0.01935	1.737E-8	653.10
plus	minus	3.7594	0.05153	16	72.96	<.0001	0.05	3.6502	3.8686	42.9223	2.2116	38.4809	47.8764
plus	plus	3.6225	0.05474	16	66.18	<.0001	0.05	3.5065	3.7386	37.4316	2.0490	33.3305	42.0374

Differences of alpha*Wnt1eYFP Least Squares Means											
alpha	Wnt1eYFP	_alpha	_Wnt1eYFP	Estimate	Standard Error	DF	t Value	Pr > |t|	Alpha	Lower	Upper
minus	minus	minus	plus	5.7055	6.6321	16	0.86	0.4023	0.05	-8.3538	19.7648
minus	minus	plus	minus	-3.7472	3.3170	16	-1.13	0.2753	0.05	-10.7789	3.2846
minus	minus	plus	plus	-3.6103	3.3171	16	-1.09	0.2925	0.05	-10.6421	3.4215
minus	plus	plus	minus	-9.4527	5.7434	16	-1.65	0.1193	0.05	-21.6282	2.7228
minus	plus	plus	plus	-9.3158	5.7434	16	-1.62	0.1243	0.05	-21.4914	2.8598
plus	minus	plus	plus	0.1369	0.07518	16	1.82	0.0874	0.05	-0.02249	0.2962

The GLIMMIX Procedure

Colony*alpha*Wnt1eYF Least Squares Means														
Colony_Size	alpha	Wnt1eYFP	Estimate	Standard Error	DF	t Value	Pr > |t|	Alpha	Lower	Upper	Mean	Standard Error Mean	Lower Mean	Upper Mean
Large	minus	minus	-5.6934	9.9478	16	-0.57	0.5751	0.05	-26.7818	15.3951	0.003368	0.03351	2.34E-12	4853059
Large	minus	plus	-5.6933	9.9479	16	-0.57	0.5751	0.05	-26.7821	15.3954	0.003368	0.03351	2.34E-12	4854213
Large	plus	minus	3.4761	0.1015	16	34.24	<.0001	0.05	3.2608	3.6913	32.3331	3.2829	26.0716	40.0984
Large	plus	plus	3.4446	0.1031	16	33.40	<.0001	0.05	3.2260	3.6633	31.3320	3.2317	25.1783	38.9896
Micro	minus	minus	2.6390	0.1543	16	17.10	<.0001	0.05	2.3119	2.9662	13.9998	2.1602	10.0939	19.4171
Micro	minus	plus	-5.6933	9.9475	16	-0.57	0.5751	0.05	-26.7809	15.3944	0.003369	0.03351	2.34E-12	4849765
Micro	plus	minus	4.0255	0.07715	16	52.18	<.0001	0.05	3.8619	4.1890	56.0059	4.3207	47.5562	65.9570
Micro	plus	plus	3.6463	0.09325	16	39.10	<.0001	0.05	3.4487	3.8440	38.3339	3.5746	31.4580	46.7126
Small	minus	minus	3.0910	0.1231	16	25.11	<.0001	0.05	2.8300	3.3519	21.9985	2.7079	16.9459	28.5576
Small	minus	plus	-5.6933	9.9473	16	-0.57	0.5750	0.05	-26.7807	15.3942	0.003369	0.03351	2.34E-12	4848447
Small	plus	minus	3.7766	0.08737	16	43.23	<.0001	0.05	3.5914	3.9618	43.6685	3.8153	36.2854	52.5540
Small	plus	plus	3.7766	0.08737	16	43.23	<.0001	0.05	3.5914	3.9618	43.6663	3.8151	36.2834	52.5515

The GLIMMIX Procedure

Differences of Colony*alpha*Wnt1eYF Least Squares Means													
Colony_Size	alpha	Wnt1eYFP	_Colony_Size	_alpha	_Wnt1eYFP	Estimate	Standard Error	DF	t Value	Pr > |t|	Alpha	Lower	Upper
Large	minus	minus	Large	minus	plus	-3.46E-6	14.0684	16	-0.00	1.0000	0.05	-29.8236	29.8236
Large	minus	minus	Large	plus	minus	-9.1694	9.9484	16	-0.92	0.3704	0.05	-30.2590	11.9201
Large	minus	minus	Large	plus	plus	-9.1380	9.9484	16	-0.92	0.3720	0.05	-30.2276	11.9516
Large	minus	minus	Micro	minus	minus	-8.3324	9.9490	16	-0.84	0.4146	0.05	-29.4234	12.7586
Large	minus	minus	Micro	minus	plus	-0.00010	14.0681	16	-0.00	1.0000	0.05	-29.8232	29.8230
Large	minus	minus	Micro	plus	minus	-9.7188	9.9481	16	-0.98	0.3431	0.05	-30.8079	11.3703
Large	minus	minus	Micro	plus	plus	-9.3397	9.9483	16	-0.94	0.3618	0.05	-30.4291	11.7497
Large	minus	minus	Small	minus	minus	-8.7843	9.9486	16	-0.88	0.3903	0.05	-29.8744	12.3058
Large	minus	minus	Small	minus	plus	-0.00010	14.0681	16	-0.00	1.0000	0.05	-29.8232	29.8230
Large	minus	minus	Small	plus	minus	-9.4700	9.9482	16	-0.95	0.3553	0.05	-30.5593	11.6193
Large	minus	minus	Small	plus	plus	-9.4699	9.9482	16	-0.95	0.3553	0.05	-30.5592	11.6194
Large	minus	plus	Large	plus	minus	-9.1694	9.9485	16	-0.92	0.3704	0.05	-30.2592	11.9204
Large	minus	plus	Large	plus	plus	-9.1380	9.9485	16	-0.92	0.3720	0.05	-30.2278	11.9519
Large	minus	plus	Micro	minus	minus	-8.3324	9.9491	16	-0.84	0.4146	0.05	-29.4236	12.7589
Large	minus	plus	Micro	minus	plus	-0.00010	14.0682	16	-0.00	1.0000	0.05	-29.8233	29.8231
Large	minus	plus	Micro	plus	minus	-9.7188	9.9482	16	-0.98	0.3431	0.05	-30.8081	11.3705
Large	minus	plus	Micro	plus	plus	-9.3397	9.9484	16	-0.94	0.3618	0.05	-30.4293	11.7499
Large	minus	plus	Small	minus	minus	-8.7843	9.9487	16	-0.88	0.3903	0.05	-29.8746	12.3060
Large	minus	plus	Small	minus	plus	-0.00009	14.0682	16	-0.00	1.0000	0.05	-29.8233	29.8231
Large	minus	plus	Small	plus	minus	-9.4700	9.9483	16	-0.95	0.3553	0.05	-30.5595	11.6195
Large	minus	plus	Small	plus	plus	-9.4699	9.9483	16	-0.95	0.3553	0.05	-30.5594	11.6196
Large	plus	minus	Large	plus	plus	0.03145	0.1447	16	0.22	0.8307	0.05	-0.2754	0.3383
Large	plus	minus	Micro	minus	minus	0.8370	0.1847	16	4.53	0.0003	0.05	0.4455	1.2286
Large	plus	minus	Micro	minus	plus	9.1693	9.9480	16	0.92	0.3704	0.05	-11.9194	30.2581

The GLIMMIX Procedure

Differences of Colony*alpha*Wnt1eYF Least Squares Means													
Colony_Size	alpha	Wnt1eYFP	_Colony_Size	_alpha	_Wnt1eYFP	Estimate	Standard Error	DF	t Value	Pr > |t|	Alpha	Lower	Upper
Large	plus	minus	Micro	plus	minus	-0.5494	0.1275	16	-4.31	0.0005	0.05	-0.8197	-0.2790
Large	plus	minus	Micro	plus	plus	-0.1702	0.1379	16	-1.23	0.2347	0.05	-0.4625	0.1220
Large	plus	minus	Small	minus	minus	0.3851	0.1596	16	2.41	0.0282	0.05	0.04685	0.7234
Large	plus	minus	Small	minus	plus	9.1693	9.9479	16	0.92	0.3704	0.05	-11.9192	30.2579
Large	plus	minus	Small	plus	minus	-0.3005	0.1339	16	-2.24	0.0394	0.05	-0.5845	-0.01658
Large	plus	minus	Small	plus	plus	-0.3005	0.1340	16	-2.24	0.0394	0.05	-0.5844	-0.01652
Large	plus	plus	Micro	minus	minus	0.8056	0.1856	16	4.34	0.0005	0.05	0.4121	1.1991
Large	plus	plus	Micro	minus	plus	9.1379	9.9480	16	0.92	0.3720	0.05	-11.9509	30.2267
Large	plus	plus	Micro	plus	minus	-0.5808	0.1288	16	-4.51	0.0004	0.05	-0.8539	-0.3078
Large	plus	plus	Micro	plus	plus	-0.2017	0.1390	16	-1.45	0.1662	0.05	-0.4965	0.09307
Large	plus	plus	Small	minus	minus	0.3537	0.1606	16	2.20	0.0427	0.05	0.01322	0.6941
Large	plus	plus	Small	minus	plus	9.1379	9.9479	16	0.92	0.3720	0.05	-11.9507	30.2265
Large	plus	plus	Small	plus	minus	-0.3320	0.1352	16	-2.46	0.0259	0.05	-0.6185	-0.04543
Large	plus	plus	Small	plus	plus	-0.3319	0.1352	16	-2.46	0.0259	0.05	-0.6185	-0.04538
Micro	minus	minus	Micro	minus	plus	8.3323	9.9487	16	0.84	0.4146	0.05	-12.7579	29.4225
Micro	minus	minus	Micro	plus	minus	-1.3864	0.1725	16	-8.04	<.0001	0.05	-1.7521	-1.0207
Micro	minus	minus	Micro	plus	plus	-1.0073	0.1803	16	-5.59	<.0001	0.05	-1.3895	-0.6251
Micro	minus	minus	Small	minus	minus	-0.4519	0.1974	16	-2.29	0.0360	0.05	-0.8704	-0.03349
Micro	minus	minus	Small	minus	plus	8.3323	9.9486	16	0.84	0.4146	0.05	-12.7577	29.4223
Micro	minus	minus	Small	plus	minus	-1.1376	0.1773	16	-6.42	<.0001	0.05	-1.5135	-0.7617
Micro	minus	minus	Small	plus	plus	-1.1375	0.1773	16	-6.42	<.0001	0.05	-1.5134	-0.7616
Micro	minus	plus	Micro	plus	minus	-9.7187	9.9478	16	-0.98	0.3431	0.05	-30.8070	11.3696
Micro	minus	plus	Micro	plus	plus	-9.3396	9.9479	16	-0.94	0.3618	0.05	-30.4282	11.7490
Micro	minus	plus	Small	minus	minus	-8.7842	9.9482	16	-0.88	0.3903	0.05	-29.8735	12.3051

The GLIMMIX Procedure

Differences of Colony*alpha*Wnt1eYF Least Squares Means

Colony_Size	alpha	Wnt1eYFP	_Colony_Size	_alpha	_Wnt1eYFP	Estimate	Standard Error	DF	t Value	Pr > |t|	Alpha	Lower	Upper
Micro	minus	plus	Small	minus	plus	2.733E-6	14.0679	16	0.00	1.0000	0.05	-29.8225	29.8225
Micro	minus	plus	Small	plus	minus	-9.4699	9.9479	16	-0.95	0.3553	0.05	-30.5584	11.6186
Micro	minus	plus	Small	plus	plus	-9.4698	9.9479	16	-0.95	0.3553	0.05	-30.5583	11.6187
Micro	plus	minus	Micro	plus	plus	0.3791	0.1210	16	3.13	0.0064	0.05	0.1226	0.6357
Micro	plus	minus	Small	minus	minus	0.9345	0.1453	16	6.43	<.0001	0.05	0.6265	1.2424
Micro	plus	minus	Small	minus	plus	9.7187	9.9476	16	0.98	0.3431	0.05	-11.3693	30.8068
Micro	plus	minus	Small	plus	minus	0.2488	0.1166	16	2.13	0.0486	0.05	0.001746	0.4959
Micro	plus	minus	Small	plus	plus	0.2489	0.1166	16	2.14	0.0485	0.05	0.001793	0.4960
Micro	plus	plus	Small	minus	minus	0.5554	0.1544	16	3.60	0.0024	0.05	0.2280	0.8827
Micro	plus	plus	Small	minus	plus	9.3396	9.9478	16	0.94	0.3618	0.05	-11.7488	30.4279
Micro	plus	plus	Small	plus	minus	-0.1303	0.1278	16	-1.02	0.3231	0.05	-0.4012	0.1406
Micro	plus	plus	Small	plus	plus	-0.1302	0.1278	16	-1.02	0.3233	0.05	-0.4011	0.1407
Small	minus	minus	Small	minus	plus	8.7842	9.9481	16	0.88	0.3903	0.05	-12.3048	29.8733
Small	minus	minus	Small	plus	minus	-0.6857	0.1509	16	-4.54	0.0003	0.05	-1.0056	-0.3657
Small	minus	minus	Small	plus	plus	-0.6856	0.1509	16	-4.54	0.0003	0.05	-1.0056	-0.3656
Small	minus	plus	Small	plus	minus	-9.4699	9.9477	16	-0.95	0.3553	0.05	-30.5581	11.6184
Small	minus	plus	Small	plus	plus	-9.4698	9.9477	16	-0.95	0.3553	0.05	-30.5581	11.6184
Small	plus	minus	Small	plus	plus	0.000051	0.1236	16	0.00	0.9997	0.05	-0.2619	0.2620

The GLIMMIX Procedure

Model Information	
**Data Set**	WORK.FIG3CIV
**Response Variable**	Count
**Response Distribution**	Poisson
**Link Function**	Log
**Variance Function**	Default
**Variance Matrix Blocked By**	Dish_ID
**Estimation Technique**	Maximum Likelihood
**Likelihood Approximation**	Laplace
**Degrees of Freedom Method**	Containment

Class Level Information		
**Class**	**Levels**	**Values**
**Colony_Size**	3	Large Micro Small
**alpha**	2	minus plus
**Mesp1eYFP**	2	minus plus
**Dish_ID**	12	1 2 3 4 5 6 7 8 9 10 11 12

**Number of Observations Read**	36
**Number of Observations Used**	36

Dimensions	
**G-side Cov. Parameters**	1
**Columns in X**	36
**Columns in Z per Subject**	1
**Subjects (Blocks in V)**	12
**Max Obs per Subject**	3

The GLIMMIX Procedure

Optimization Information	
**Optimization Technique**	Dual Quasi-Newton
**Parameters in Optimization**	13
**Lower Boundaries**	1
**Upper Boundaries**	0
**Fixed Effects**	Not Profiled
**Starting From**	GLM estimates

Iteration History					
**Iteration**	**Restarts**	**Evaluations**	**Objective Function**	**Change**	**Max Gradient**
**0**	**0**	4	90.003358073	.	147.7156
**1**	**0**	5	89.855923588	0.14743448	145.6155
**2**	**0**	2	89.116193063	0.73973053	7.690353
**3**	**0**	3	89.115464605	0.00072846	1.224078
**4**	**0**	2	89.115264933	0.00019967	0.243868
**5**	**0**	4	89.115227325	0.00003761	0.381296
**6**	**0**	4	89.114975918	0.00025141	0.21591
**7**	**0**	4	89.113949142	0.00102678	1.426568
**8**	**0**	2	89.11337956	0.00056958	4.162647
**9**	**0**	2	89.112482198	0.00089736	0.270415
**10**	**0**	2	89.110933525	0.00154867	3.214711
**11**	**0**	4	89.106391643	0.00454188	6.5065
**12**	**0**	4	89.093477069	0.01291457	5.159968
**13**	**0**	3	89.088878039	0.00459903	1.746353
**14**	**0**	2	89.082688197	0.00618984	1.447585
**15**	**0**	3	89.082372861	0.00031534	0.472092

The GLIMMIX Procedure

Iteration History					
Iteration	Restarts	Evaluations	Objective Function	Change	Max Gradient
16	0	6	89.063692755	0.01868011	5.813502
17	0	3	89.051142721	0.01255003	0.348636
18	0	2	89.049249074	0.00189365	3.888735
19	0	4	89.041661709	0.00758736	0.820754
20	0	4	89.003101789	0.03855992	0.28302
21	0	2	88.994711575	0.00839021	2.950325
22	0	2	88.983468438	0.01124314	0.236852
23	0	3	88.982276578	0.00119186	0.508815
24	0	4	88.977511197	0.00476538	0.190626
25	0	3	88.974991369	0.00251983	0.067221
26	0	3	88.974299376	0.00069199	0.033198
27	0	3	88.973955282	0.00034409	0.08921
28	0	3	88.973835942	0.00011934	0.02959
29	0	3	88.973781067	0.00005488	0.038402
30	0	3	88.973763125	0.00001794	0.009105
31	0	3	88.973754418	0.00000871	0.013867
32	0	3	88.9737521	0.00000232	0.002524
33	0	2	88.973751276	0.00000082	0.008286

Convergence criterion (GCONV=1E-8) satisfied.

The GLIMMIX Procedure

Fit Statistics	
-2 Log Likelihood	88.97
AIC (smaller is better)	114.97
AICC (smaller is better)	131.52
BIC (smaller is better)	121.28
CAIC (smaller is better)	134.28
HQIC (smaller is better)	112.64

Fit Statistics for Conditional Distribution	
-2 log L(Count | r. effects)	86.71
Pearson Chi-Square	6.68
Pearson Chi-Square / DF	0.19

Covariance Parameter Estimates

Cov Parm	Subject	Estimate	Standard Error
Intercept	Dish_ID	0.001958	0.005579

Solutions for Fixed Effects

Effect	Colony_Size	alpha	Mesp1eYFP	Estimate	Standard Error	DF	t Value	Pr > |t|
Intercept				1.4656	0.2785	8	5.26	0.0008
Colony_Size	Large			-19.5270	0.1145	16	-170.47	<.0001
Colony_Size	Micro			2.7381	0.2861	16	9.57	<.0001
Colony_Size	Small			0	.	.	.	.
alpha		minus		-80.5183	0	16	-Infty	<.0001
alpha		plus		0	.	.	.	.

The GLIMMIX Procedure

Solutions for Fixed Effects								
**Effect**	**Colony_Size**	**alpha**	**Mesp1eYFP**	**Estimate**	**Standard Error**	**DF**	**t Value**	**Pr > |t|**
**Colony_Size*alpha**	Large	minus		-17.2181	0	16	-Infty	<.0001
**Colony_Size*alpha**	Large	plus		0	.	.	.	.
**Colony_Size*alpha**	Micro	minus		-27.1648	0	16	-Infty	<.0001
**Colony_Size*alpha**	Micro	plus		0	.	.	.	.
**Colony_Size*alpha**	Small	minus		0	.	.	.	.
**Colony_Size*alpha**	Small	plus		0	.	.	.	.
**Mesp1eYFP**			minus	2.5995	0.2897	16	8.97	<.0001
**Mesp1eYFP**			plus	0	.	.	.	.
**Colony_Siz*Mesp1eYFP**	Large		minus	19.2675	0	16	Infty	<.0001
**Colony_Siz*Mesp1eYFP**	Large		plus	0	.	.	.	.
**Colony_Siz*Mesp1eYFP**	Micro		minus	-2.8340	0.3064	16	-9.25	<.0001
**Colony_Siz*Mesp1eYFP**	Micro		plus	0	.	.	.	.
**Colony_Siz*Mesp1eYFP**	Small		minus	0	.	.	.	.
**Colony_Siz*Mesp1eYFP**	Small		plus	0	.	.	.	.
**alpha*Mesp1eYFP**		minus	minus	-39.1508	0	16	-Infty	<.0001
**alpha*Mesp1eYFP**		minus	plus	0	.	.	.	.
**alpha*Mesp1eYFP**		plus	minus	0	.	.	.	.
**alpha*Mesp1eYFP**		plus	plus	0	.	.	.	.
**Colony*alpha*Mesp1eY**	Large	minus	minus	-19.0555	0	16	-Infty	<.0001
**Colony*alpha*Mesp1eY**	Large	minus	plus	0	.	.	.	.
**Colony*alpha*Mesp1eY**	Large	plus	minus	0	.	.	.	.
**Colony*alpha*Mesp1eY**	Large	plus	plus	0	.	.	.	.
**Colony*alpha*Mesp1eY**	Micro	minus	minus	-9.3226	0	16	-Infty	<.0001
**Colony*alpha*Mesp1eY**	Micro	minus	plus	0	.	.	.	.

The GLIMMIX Procedure

Solutions for Fixed Effects								
**Effect**	**Colony_Size**	**alpha**	**Mesp1eYFP**	**Estimate**	**Standard Error**	**DF**	**t Value**	**Pr > |t|**
**Colony*alpha*Mesp1eY**	Micro	plus	minus	0	.	.	.	.
**Colony*alpha*Mesp1eY**	Micro	plus	plus	0	.	.	.	.
**Colony*alpha*Mesp1eY**	Small	minus	minus	0	.	.	.	.
**Colony*alpha*Mesp1eY**	Small	minus	plus	0	.	.	.	.
**Colony*alpha*Mesp1eY**	Small	plus	minus	0	.	.	.	.
**Colony*alpha*Mesp1eY**	Small	plus	plus	0	.	.	.	.

Type III Tests of Fixed Effects				
**Effect**	**Num DF**	**Den DF**	**F Value**	**Pr > F**
**Colony_Size**	2	16	22613.3	<.0001
**alpha**	1	16	Infty	<.0001
**Colony_Size*alpha**	2	16	Infty	<.0001
**Mesp1eYFP**	1	16	6704.34	<.0001
**Colony_Siz*Mesp1eYFP**	1	16	598.39	<.0001
**alpha*Mesp1eYFP**	1	16	Infty	<.0001
**Colony*alpha*Mesp1eY**	2	16	Infty	<.0001

alpha*Mesp1eYFP Least Squares Means													
**alpha**	**Mesp1eYFP**	**Estimate**	**Standard Error**	**DF**	**t Value**	**Pr > |t|**	**Alpha**	**Lower**	**Upper**	**Mean**	**Standard Error Mean**	**Lower Mean**	**Upper Mean**
minus	minus	-139.98	0.05309	16	-2636.7	<.0001	0.05	-140.09	-139.86	1.62E-61	8.59E-63	1.45E-61	1.81E-61
minus	plus	-99.4432	0.1920	16	-518.02	<.0001	0.05	-99.8502	-99.0363	6.49E-44	1.25E-44	4.32E-44	9.75E-44
plus	minus	3.9467	0.05309	16	74.34	<.0001	0.05	3.8341	4.0592	51.7629	2.7480	46.2533	57.9289
plus	plus	-4.1307	0.1920	16	-21.52	<.0001	0.05	-4.5376	-3.7237	0.01607	0.003085	0.01070	0.02414

The GLIMMIX Procedure

Differences of alpha*Mesp1eYFP Least Squares Means											
alpha	Mesp1eYFP	_alpha	_Mesp1eYFP	Estimate	Standard Error	DF	t Value	Pr > |t|	Alpha	Lower	Upper
minus	minus	minus	plus	-40.5328	0.1982	16	-204.52	<.0001	0.05	-40.9530	-40.1127
minus	minus	plus	minus	-143.92	0	16	-Infty	<.0001	.	.	.
minus	minus	plus	plus	-135.85	0.1982	16	-685.43	<.0001	0.05	-136.27	-135.43
minus	plus	plus	minus	-103.39	0.1982	16	-521.67	<.0001	0.05	-103.81	-102.97
minus	plus	plus	plus	-95.3126	0	16	-Infty	<.0001	.	.	.
plus	minus	plus	plus	8.0773	0.1982	16	40.76	<.0001	0.05	7.6572	8.4975

Colony*alpha*Mesp1eY Least Squares Means														
Colony_Size	alpha	Mesp1eYFP	Estimate	Standard Error	DF	t Value	Pr > |t|	Alpha	Lower	Upper	Mean	Standard Error Mean	Lower Mean	Upper Mean
Large	minus	minus	-152.14	0.08983	16	-1693.7	<.0001	0.05	-152.33	-151.95	8.47E-67	7.61E-68	7E-67	1.02E-66
Large	minus	plus	-115.80	0.3011	16	-384.53	<.0001	0.05	-116.44	-115.16	5.12E-51	1.54E-51	2.71E-51	9.7E-51
Large	plus	minus	3.8056	0.08983	16	42.37	<.0001	0.05	3.6152	3.9961	44.9537	4.0380	37.1592	54.3831
Large	plus	plus	-18.0614	0.3011	16	-59.98	<.0001	0.05	-18.6998	-17.4230	1.432E-8	4.313E-9	7.565E-9	2.712E-8
Micro	minus	minus	-152.19	0.08337	16	-1825.4	<.0001	0.05	-152.36	-152.01	8.05E-67	6.71E-68	6.75E-67	9.61E-67
Micro	minus	plus	-103.48	0.07506	16	-1378.5	<.0001	0.05	-103.64	-103.32	1.15E-45	8.61E-47	9.78E-46	1.34E-45
Micro	plus	minus	3.9693	0.08337	16	47.61	<.0001	0.05	3.7925	4.1460	52.9455	4.4142	44.3682	63.1810
Micro	plus	plus	4.2038	0.07506	16	56.00	<.0001	0.05	4.0446	4.3629	66.9369	5.0246	57.0895	78.4830
Small	minus	minus	-115.60	0.07985	16	-1447.8	<.0001	0.05	-115.77	-115.43	6.22E-51	4.97E-52	5.25E-51	7.37E-51
Small	minus	plus	-79.0527	0.2785	16	-283.84	<.0001	0.05	-79.6431	-78.4622	4.65E-35	1.3E-35	2.58E-35	8.4E-35
Small	plus	minus	4.0651	0.07985	16	50.91	<.0001	0.05	3.8959	4.2344	58.2723	4.6530	49.1980	69.0203
Small	plus	plus	1.4656	0.2785	16	5.26	<.0001	0.05	0.8752	2.0560	4.3302	1.2060	2.3994	7.8148

The GLIMMIX Procedure

Differences of Colony*alpha*Mesp1eY Least Squares Means													
Colony_Size	alpha	Mesp1eYFP	_Colony_Size	_alpha	_Mesp1eYFP	Estimate	Standard Error	DF	t Value	Pr > |t|	Alpha	Lower	Upper
Large	minus	minus	Large	minus	plus	-36.3393	0.2897	16	-125.44	<.0001	0.05	-36.9534	-35.7252
Large	minus	minus	Large	plus	minus	-155.94	0	16	-Infty	<.0001	.	.	.
Large	minus	minus	Large	plus	plus	-134.08	0.2897	16	-462.80	<.0001	0.05	-134.69	-133.46
Large	minus	minus	Micro	minus	minus	0.05014	0.1170	16	0.43	0.6741	0.05	-0.1980	0.2982
Large	minus	minus	Micro	minus	plus	-48.6577	0.1170	16	-415.88	<.0001	0.05	-48.9058	-48.4097
Large	minus	minus	Micro	plus	minus	-156.11	0.1170	16	-1333.9	<.0001	0.05	-156.35	-155.86
Large	minus	minus	Micro	plus	plus	-156.34	0.1170	16	-1336.2	<.0001	0.05	-156.59	-156.09
Large	minus	minus	Small	minus	minus	-36.5331	0.1145	16	-318.94	<.0001	0.05	-36.7760	-36.2903
Large	minus	minus	Small	minus	plus	-73.0844	0.2926	16	-249.77	<.0001	0.05	-73.7047	-72.4641
Large	minus	minus	Small	plus	minus	-156.20	0.1145	16	-1363.6	<.0001	0.05	-156.45	-155.96
Large	minus	minus	Small	plus	plus	-153.60	0.2926	16	-524.94	<.0001	0.05	-154.22	-152.98
Large	minus	plus	Large	plus	minus	-119.60	0.2897	16	-412.85	<.0001	0.05	-120.22	-118.99
Large	minus	plus	Large	plus	plus	-97.7364	0	16	-Infty	<.0001	.	.	.
Large	minus	plus	Micro	minus	minus	36.3894	0.3124	16	116.47	<.0001	0.05	35.7271	37.0518
Large	minus	plus	Micro	minus	plus	-12.3184	0.3082	16	-39.97	<.0001	0.05	-12.9718	-11.6650
Large	minus	plus	Micro	plus	minus	-119.77	0.3124	16	-383.32	<.0001	0.05	-120.43	-119.10
Large	minus	plus	Micro	plus	plus	-120.00	0.3082	16	-389.33	<.0001	0.05	-120.65	-119.35
Large	minus	plus	Small	minus	minus	-0.1938	0.3294	16	-0.59	0.5644	0.05	-0.8920	0.5044
Large	minus	plus	Small	minus	plus	-36.7451	0.1145	16	-320.79	<.0001	0.05	-36.9879	-36.5023
Large	minus	plus	Small	plus	minus	-119.86	0.3294	16	-363.93	<.0001	0.05	-120.56	-119.16
Large	minus	plus	Small	plus	plus	-117.26	0.1145	16	-1023.7	<.0001	0.05	-117.51	-117.02
Large	plus	minus	Large	plus	plus	21.8670	0.2897	16	75.48	<.0001	0.05	21.2528	22.4811
Large	plus	minus	Micro	minus	minus	155.99	0.1170	16	1332.92	<.0001	0.05	155.74	156.24
Large	plus	minus	Micro	minus	plus	107.28	0.1170	16	916.96	<.0001	0.05	107.04	107.53

The GLIMMIX Procedure

Differences of Colony*alpha*Mesp1eY Least Squares Means													
Colony_Size	alpha	Mesp1eYFP	_Colony_Size	_alpha	_Mesp1eYFP	Estimate	Standard Error	DF	t Value	Pr > |t|	Alpha	Lower	Upper
Large	plus	minus	Micro	plus	minus	-0.1636	0.1170	16	-1.40	0.1811	0.05	-0.4117	0.08446
Large	plus	minus	Micro	plus	plus	-0.3981	0.1170	16	-3.40	0.0036	0.05	-0.6461	-0.1501
Large	plus	minus	Small	minus	minus	119.41	0.1145	16	1042.45	<.0001	0.05	119.17	119.65
Large	plus	minus	Small	minus	plus	82.8583	0.2926	16	283.17	<.0001	0.05	82.2380	83.4786
Large	plus	minus	Small	plus	minus	-0.2595	0.1145	16	-2.27	0.0377	0.05	-0.5023	-0.01666
Large	plus	minus	Small	plus	plus	2.3400	0.2926	16	8.00	<.0001	0.05	1.7197	2.9603
Large	plus	plus	Micro	minus	minus	134.13	0.3124	16	429.27	<.0001	0.05	133.46	134.79
Large	plus	plus	Micro	minus	plus	85.4180	0.3082	16	277.13	<.0001	0.05	84.7646	86.0714
Large	plus	plus	Micro	plus	minus	-22.0306	0.3124	16	-70.51	<.0001	0.05	-22.6930	-21.3683
Large	plus	plus	Micro	plus	plus	-22.2651	0.3082	16	-72.24	<.0001	0.05	-22.9185	-21.6117
Large	plus	plus	Small	minus	minus	97.5426	0.3294	16	296.16	<.0001	0.05	96.8444	98.2408
Large	plus	plus	Small	minus	plus	60.9913	0.1145	16	532.46	<.0001	0.05	60.7485	61.2341
Large	plus	plus	Small	plus	minus	-22.1265	0.3294	16	-67.18	<.0001	0.05	-22.8247	-21.4283
Large	plus	plus	Small	plus	plus	-19.5270	0.1145	16	-170.47	<.0001	0.05	-19.7698	-19.2841
Micro	minus	minus	Micro	minus	plus	-48.7079	0.1121	16	-434.42	<.0001	0.05	-48.9456	-48.4702
Micro	minus	minus	Micro	plus	minus	-156.16	0	16	-Infty	<.0001	.	.	.
Micro	minus	minus	Micro	plus	plus	-156.39	0.1121	16	-1394.8	<.0001	0.05	-156.63	-156.15
Micro	minus	minus	Small	minus	minus	-36.5833	0.1096	16	-333.91	<.0001	0.05	-36.8155	-36.3510
Micro	minus	minus	Small	minus	plus	-73.1345	0.2907	16	-251.59	<.0001	0.05	-73.7508	-72.5183
Micro	minus	minus	Small	plus	minus	-156.25	0.1096	16	-1426.2	<.0001	0.05	-156.48	-156.02
Micro	minus	minus	Small	plus	plus	-153.65	0.2907	16	-528.57	<.0001	0.05	-154.27	-153.04
Micro	minus	plus	Micro	plus	minus	-107.45	0.1121	16	-958.32	<.0001	0.05	-107.69	-107.21
Micro	minus	plus	Micro	plus	plus	-107.68	0	16	-Infty	<.0001	.	.	.
Micro	minus	plus	Small	minus	minus	12.1246	0.1095	16	110.70	<.0001	0.05	11.8924	12.3568

The GLIMMIX Procedure

Differences of Colony*alpha*Mesp1eY Least Squares Means													
Colony_Size	alpha	Mesp1eYFP	_Colony_Size	_alpha	_Mesp1eYFP	Estimate	Standard Error	DF	t Value	Pr > |t|	Alpha	Lower	Upper
Micro	minus	plus	Small	minus	plus	-24.4267	0.2861	16	-85.36	<.0001	0.05	-25.0333	-23.8201
Micro	minus	plus	Small	plus	minus	-107.54	0.1095	16	-981.89	<.0001	0.05	-107.78	-107.31
Micro	minus	plus	Small	plus	plus	-104.94	0.2861	16	-366.75	<.0001	0.05	-105.55	-104.34
Micro	plus	minus	Micro	plus	plus	-0.2345	0.1121	16	-2.09	0.0528	0.05	-0.4722	0.003200
Micro	plus	minus	Small	minus	minus	119.57	0.1096	16	1091.39	<.0001	0.05	119.34	119.81
Micro	plus	minus	Small	minus	plus	83.0219	0.2907	16	285.60	<.0001	0.05	82.4057	83.6382
Micro	plus	minus	Small	plus	minus	-0.09586	0.1096	16	-0.87	0.3945	0.05	-0.3281	0.1364
Micro	plus	minus	Small	plus	plus	2.5036	0.2907	16	8.61	<.0001	0.05	1.8874	3.1199
Micro	plus	plus	Small	minus	minus	119.81	0.1095	16	1093.85	<.0001	0.05	119.58	120.04
Micro	plus	plus	Small	minus	plus	83.2564	0.2861	16	290.95	<.0001	0.05	82.6498	83.8630
Micro	plus	plus	Small	plus	minus	0.1386	0.1095	16	1.27	0.2238	0.05	-0.09357	0.3708
Micro	plus	plus	Small	plus	plus	2.7381	0.2861	16	9.57	<.0001	0.05	2.1315	3.3447
Small	minus	minus	Small	minus	plus	-36.5513	0.2897	16	-126.17	<.0001	0.05	-37.1654	-35.9371
Small	minus	minus	Small	plus	minus	-119.67	0	16	-Infty	<.0001	.	.	.
Small	minus	minus	Small	plus	plus	-117.07	0.2897	16	-404.10	<.0001	0.05	-117.68	-116.46
Small	minus	plus	Small	plus	minus	-83.1178	0.2897	16	-286.91	<.0001	0.05	-83.7319	-82.5036
Small	minus	plus	Small	plus	plus	-80.5183	0	16	-Infty	<.0001	.	.	.
Small	plus	minus	Small	plus	plus	2.5995	0.2897	16	8.97	<.0001	0.05	1.9854	3.2137

The GLIMMIX Procedure

Model Information	
**Data Set**	WORK.FIG3DIV
**Response Variable**	Count
**Response Distribution**	Poisson
**Link Function**	Log
**Variance Function**	Default
**Variance Matrix Blocked By**	Dish_ID
**Estimation Technique**	Maximum Likelihood
**Likelihood Approximation**	Laplace
**Degrees of Freedom Method**	Containment

Class Level Information		
**Class**	**Levels**	**Values**
**Colony_Size**	3	Large Micro Small
**alpha**	2	minus plus
**Wnt1eYFP**	2	minus plus
**Dish_ID**	12	1 2 3 4 5 6 7 8 9 10 11 12

**Number of Observations Read**	36
**Number of Observations Used**	36

Dimensions	
**G-side Cov. Parameters**	1
**Columns in X**	36
**Columns in Z per Subject**	1
**Subjects (Blocks in V)**	12
**Max Obs per Subject**	3

The GLIMMIX Procedure

Optimization Information	
Optimization Technique	Dual Quasi-Newton
Parameters in Optimization	13
Lower Boundaries	1
Upper Boundaries	0
Fixed Effects	Not Profiled
Starting From	GLM estimates

Iteration History					
Iteration	Restarts	Evaluations	Objective Function	Change	Max Gradient
0	0	4	109.36030569	.	138.7172
1	0	2	108.68505512	0.67525057	100.7516
2	0	3	108.63376919	0.05128592	17.05899
3	0	2	108.63140094	0.00236825	4.78363
4	0	2	108.6312118	0.00018915	0.632666
5	0	2	108.63121018	0.00000162	0.634575
6	0	2	108.63120746	0.00000271	0.6308
7	0	4	108.6311879	0.00001956	1.792574
8	0	4	108.63103869	0.00014921	0.121248
9	0	3	108.63103861	0.00000007	0.121249

Convergence criterion (GCONV=1E-8) satisfied.

The GLIMMIX Procedure

Fit Statistics	
-2 Log Likelihood	108.63
AIC (smaller is better)	134.63
AICC (smaller is better)	151.18
BIC (smaller is better)	140.93
CAIC (smaller is better)	153.93
HQIC (smaller is better)	132.30

Fit Statistics for Conditional Distribution	
-2 log L(Count | r. effects)	107.10
Pearson Chi-Square	10.23
Pearson Chi-Square / DF	0.28

Covariance Parameter Estimates

Cov Parm	Subject	Estimate	Standard Error
Intercept	Dish_ID	0.001191	0.005682

Solutions for Fixed Effects

Effect	Colony_Size	alpha	Wnt1eYFP	Estimate	Standard Error	DF	t Value	Pr > |t|
Intercept				4.1739	0.07438	8	56.12	<.0001
Colony_Size	Large			-0.2964	0.1097	16	-2.70	0.0157
Colony_Size	Micro			-0.2896	0.1094	16	-2.65	0.0176
Colony_Size	Small			0	.	.	.	.
alpha		minus		-9.8676	9.9472	16	-0.99	0.3360
alpha		plus		0	.	.	.	.

The GLIMMIX Procedure

Solutions for Fixed Effects								
Effect	Colony_Size	alpha	Wnt1eYFP	Estimate	Standard Error	DF	t Value	Pr > |t|
Colony_Size*alpha	Large	minus		0.2962	14.0683	16	0.02	0.9835
Colony_Size*alpha	Large	plus		0	.	.	.	.
Colony_Size*alpha	Micro	minus		0.2894	14.0684	16	0.02	0.9838
Colony_Size*alpha	Micro	plus		0	.	.	.	.
Colony_Size*alpha	Small	minus		0	.	.	.	.
Colony_Size*alpha	Small	plus		0	.	.	.	.
Wnt1eYFP			minus	-0.5283	0.1209	16	-4.37	0.0005
Wnt1eYFP			plus	0	.	.	.	.
Colony_Size*Wnt1eYFP	Large		minus	-1.0147	0.2302	16	-4.41	0.0004
Colony_Size*Wnt1eYFP	Large		plus	0	.	.	.	.
Colony_Size*Wnt1eYFP	Micro		minus	-0.00126	0.1797	16	-0.01	0.9945
Colony_Size*Wnt1eYFP	Micro		plus	0	.	.	.	.
Colony_Size*Wnt1eYFP	Small		minus	0	.	.	.	.
Colony_Size*Wnt1eYFP	Small		plus	0	.	.	.	.
alpha*Wnt1eYFP		minus	minus	0.5280	14.0687	16	0.04	0.9705
alpha*Wnt1eYFP		minus	plus	0	.	.	.	.
alpha*Wnt1eYFP		plus	minus	0	.	.	.	.
alpha*Wnt1eYFP		plus	plus	0	.	.	.	.
Colony*alpha*Wnt1eYF	Large	minus	minus	1.0147	19.8976	16	0.05	0.9600
Colony*alpha*Wnt1eYF	Large	minus	plus	0	.	.	.	.
Colony*alpha*Wnt1eYF	Large	plus	minus	0	.	.	.	.
Colony*alpha*Wnt1eYF	Large	plus	plus	0	.	.	.	.
Colony*alpha*Wnt1eYF	Micro	minus	minus	0.001178	19.8973	16	0.00	1.0000
Colony*alpha*Wnt1eYF	Micro	minus	plus	0	.	.	.	.

The GLIMMIX Procedure

Solutions for Fixed Effects								
Effect	Colony_Size	alpha	Wnt1eYFP	Estimate	Standard Error	DF	t Value	Pr > |t|
Colony*alpha*Wnt1eYF	Micro	plus	minus	0	.	.	.	.
Colony*alpha*Wnt1eYF	Micro	plus	plus	0	.	.	.	.
Colony*alpha*Wnt1eYF	Small	minus	minus	0	.	.	.	.
Colony*alpha*Wnt1eYF	Small	minus	plus	0	.	.	.	.
Colony*alpha*Wnt1eYF	Small	plus	minus	0	.	.	.	.
Colony*alpha*Wnt1eYF	Small	plus	plus	0	.	.	.	.

Type III Tests of Fixed Effects				
Effect	Num DF	Den DF	F Value	Pr > F
Colony_Size	2	16	0.00	0.9967
alpha	1	16	5.17	0.0370
Colony_Size*alpha	2	16	0.00	0.9967
Wnt1eYFP	1	16	0.01	0.9163
Colony_Size*Wnt1eYFP	2	16	0.00	0.9983
alpha*Wnt1eYFP	1	16	0.01	0.9164
Colony*alpha*Wnt1eYF	2	16	0.00	0.9983

alpha*Wnt1eYFP Least Squares Means													
alpha	Wnt1eYFP	Estimate	Standard Error	DF	t Value	Pr > |t|	Alpha	Lower	Upper	Mean	Standard Error Mean	Lower Mean	Upper Mean
minus	minus	-5.6942	5.7441	16	-0.99	0.3363	0.05	-17.8712	6.4829	0.003366	0.01933	1.732E-8	653.84
minus	plus	-5.6939	5.7432	16	-0.99	0.3362	0.05	-17.8690	6.4812	0.003367	0.01933	1.736E-8	652.77
plus	minus	3.1116	0.07906	16	39.36	<.0001	0.05	2.9440	3.2792	22.4573	1.7755	18.9920	26.5549
plus	plus	3.9785	0.05002	16	79.55	<.0001	0.05	3.8725	4.0846	53.4393	2.6728	48.0632	59.4167

The GLIMMIX Procedure

Differences of alpha*Wnt1eYFP Least Squares Means											
alpha	Wnt1eYFP	_alpha	_Wnt1eYFP	Estimate	Standard Error	DF	t Value	Pr > |t|	Alpha	Lower	Upper
minus	minus	minus	plus	-0.00029	8.1228	16	-0.00	1.0000	0.05	-17.2199	17.2194
minus	minus	plus	minus	-8.8058	5.7447	16	-1.53	0.1448	0.05	-20.9839	3.3724
minus	minus	plus	plus	-9.6727	5.7443	16	-1.68	0.1116	0.05	-21.8502	2.5048
minus	plus	plus	minus	-8.8055	5.7438	16	-1.53	0.1448	0.05	-20.9817	3.3708
minus	plus	plus	plus	-9.6724	5.7434	16	-1.68	0.1116	0.05	-21.8480	2.5031
plus	minus	plus	plus	-0.8669	0.09347	16	-9.28	<.0001	0.05	-1.0651	-0.6688

Colony*alpha*Wnt1eYF Least Squares Means														
Colony_Size	alpha	Wnt1eYFP	Estimate	Standard Error	DF	t Value	Pr > |t|	Alpha	Lower	Upper	Mean	Standard Error Mean	Lower Mean	Upper Mean
Large	minus	minus	-5.6942	9.9493	16	-0.57	0.5751	0.05	-26.7858	15.3974	0.003365	0.03348	2.33E-12	4864127
Large	minus	plus	-5.6939	9.9479	16	-0.57	0.5750	0.05	-26.7824	15.3946	0.003366	0.03349	2.34E-12	4850654
Large	plus	minus	2.3345	0.1808	16	12.92	<.0001	0.05	1.9513	2.7177	10.3241	1.8662	7.0378	15.1450
Large	plus	plus	3.8775	0.08545	16	45.38	<.0001	0.05	3.6963	4.0586	48.3017	4.1273	40.2989	57.8938
Micro	minus	minus	-5.6943	9.9497	16	-0.57	0.5751	0.05	-26.7866	15.3981	0.003365	0.03348	2.33E-12	4867409
Micro	minus	plus	-5.6939	9.9480	16	-0.57	0.5750	0.05	-26.7828	15.3949	0.003366	0.03349	2.34E-12	4852166
Micro	plus	minus	3.3548	0.1097	16	30.58	<.0001	0.05	3.1222	3.5874	28.6392	3.1423	22.6958	36.1389
Micro	plus	plus	3.8843	0.08517	16	45.60	<.0001	0.05	3.7037	4.0649	48.6332	4.1422	40.5992	58.2571
Small	minus	minus	-5.6940	9.9483	16	-0.57	0.5750	0.05	-26.7834	15.3954	0.003366	0.03349	2.33E-12	4854598
Small	minus	plus	-5.6938	9.9469	16	-0.57	0.5750	0.05	-26.7803	15.3928	0.003367	0.03349	2.34E-12	4841852
Small	plus	minus	3.6456	0.09541	16	38.21	<.0001	0.05	3.4433	3.8479	38.3055	3.6547	31.2911	46.8923
Small	plus	plus	4.1739	0.07438	16	56.12	<.0001	0.05	4.0162	4.3315	64.9660	4.8321	55.4891	76.0613

The GLIMMIX Procedure

Differences of Colony*alpha*Wnt1eYF Least Squares Means													
Colony_Size	alpha	Wnt1eYFP	_Colony_Size	_alpha	_Wnt1eYFP	Estimate	Standard Error	DF	t Value	Pr > |t|	Alpha	Lower	Upper
Large	minus	minus	Large	minus	plus	-0.00029	14.0694	16	-0.00	1.0000	0.05	-29.8261	29.8256
Large	minus	minus	Large	plus	minus	-8.0287	9.9510	16	-0.81	0.4316	0.05	-29.1238	13.0664
Large	minus	minus	Large	plus	plus	-9.5717	9.9497	16	-0.96	0.3504	0.05	-30.6641	11.5207
Large	minus	minus	Micro	minus	minus	0.000067	14.0707	16	0.00	1.0000	0.05	-29.8284	29.8286
Large	minus	minus	Micro	minus	plus	-0.00026	14.0695	16	-0.00	1.0000	0.05	-29.8264	29.8258
Large	minus	minus	Micro	plus	minus	-9.0490	9.9499	16	-0.91	0.3766	0.05	-30.1419	12.0439
Large	minus	minus	Micro	plus	plus	-9.5785	9.9497	16	-0.96	0.3500	0.05	-30.6709	11.5139
Large	minus	minus	Small	minus	minus	-0.00021	14.0697	16	-0.00	1.0000	0.05	-29.8266	29.8262
Large	minus	minus	Small	minus	plus	-0.00045	14.0689	16	-0.00	1.0000	0.05	-29.8251	29.8242
Large	minus	minus	Small	plus	minus	-9.3398	9.9498	16	-0.94	0.3618	0.05	-30.4324	11.7528
Large	minus	minus	Small	plus	plus	-9.8681	9.9496	16	-0.99	0.3361	0.05	-30.9603	11.2241
Large	minus	plus	Large	plus	minus	-8.0284	9.9495	16	-0.81	0.4315	0.05	-29.1204	13.0636
Large	minus	plus	Large	plus	plus	-9.5714	9.9482	16	-0.96	0.3503	0.05	-30.6607	11.5179
Large	minus	plus	Micro	minus	minus	0.000360	14.0697	16	0.00	1.0000	0.05	-29.8260	29.8267
Large	minus	plus	Micro	minus	plus	0.000033	14.0685	16	0.00	1.0000	0.05	-29.8238	29.8239
Large	minus	plus	Micro	plus	minus	-9.0487	9.9485	16	-0.91	0.3766	0.05	-30.1385	12.0411
Large	minus	plus	Micro	plus	plus	-9.5782	9.9482	16	-0.96	0.3500	0.05	-30.6675	11.5111
Large	minus	plus	Small	minus	minus	0.000086	14.0687	16	0.00	1.0000	0.05	-29.8242	29.8244
Large	minus	plus	Small	minus	plus	-0.00016	14.0678	16	-0.00	1.0000	0.05	-29.8226	29.8223
Large	minus	plus	Small	plus	minus	-9.3395	9.9483	16	-0.94	0.3618	0.05	-30.4290	11.7500
Large	minus	plus	Small	plus	plus	-9.8678	9.9481	16	-0.99	0.3360	0.05	-30.9569	11.2213
Large	plus	minus	Large	plus	plus	-1.5430	0.1999	16	-7.72	<.0001	0.05	-1.9667	-1.1192
Large	plus	minus	Micro	minus	minus	8.0288	9.9513	16	0.81	0.4316	0.05	-13.0671	29.1246
Large	plus	minus	Micro	minus	plus	8.0284	9.9497	16	0.81	0.4316	0.05	-13.0639	29.1208

The GLIMMIX Procedure

Differences of Colony*alpha*Wnt1eYF Least Squares Means

Colony_Size	alpha	Wnt1eYFP	_Colony_Size	_alpha	_Wnt1eYFP	Estimate	Standard Error	DF	t Value	Pr > |t|	Alpha	Lower	Upper
Large	plus	minus	Micro	plus	minus	-1.0203	0.2095	16	-4.87	0.0002	0.05	-1.4645	-0.5761
Large	plus	minus	Micro	plus	plus	-1.5498	0.1998	16	-7.76	<.0001	0.05	-1.9733	-1.1263
Large	plus	minus	Small	minus	minus	8.0285	9.9499	16	0.81	0.4316	0.05	-13.0644	29.1214
Large	plus	minus	Small	minus	plus	8.0282	9.9486	16	0.81	0.4315	0.05	-13.0618	29.1183
Large	plus	minus	Small	plus	minus	-1.3111	0.2024	16	-6.48	<.0001	0.05	-1.7402	-0.8821
Large	plus	minus	Small	plus	plus	-1.8394	0.1954	16	-9.41	<.0001	0.05	-2.2537	-1.4251
Large	plus	plus	Micro	minus	minus	9.5717	9.9500	16	0.96	0.3504	0.05	-11.5214	30.6649
Large	plus	plus	Micro	minus	plus	9.5714	9.9484	16	0.96	0.3503	0.05	-11.5182	30.6611
Large	plus	plus	Micro	plus	minus	0.5227	0.1390	16	3.76	0.0017	0.05	0.2280	0.8174
Large	plus	plus	Micro	plus	plus	-0.00684	0.1172	16	-0.06	0.9542	0.05	-0.2554	0.2417
Large	plus	plus	Small	minus	minus	9.5715	9.9487	16	0.96	0.3503	0.05	-11.5187	30.6617
Large	plus	plus	Small	minus	plus	9.5712	9.9473	16	0.96	0.3503	0.05	-11.5161	30.6586
Large	plus	plus	Small	plus	minus	0.2319	0.1280	16	1.81	0.0889	0.05	-0.03951	0.5033
Large	plus	plus	Small	plus	plus	-0.2964	0.1097	16	-2.70	0.0157	0.05	-0.5289	-0.06393
Micro	minus	minus	Micro	minus	plus	-0.00033	14.0698	16	-0.00	1.0000	0.05	-29.8270	29.8263
Micro	minus	minus	Micro	plus	minus	-9.0490	9.9503	16	-0.91	0.3766	0.05	-30.1427	12.0446
Micro	minus	minus	Micro	plus	plus	-9.5786	9.9500	16	-0.96	0.3500	0.05	-30.6717	11.5145
Micro	minus	minus	Small	minus	minus	-0.00027	14.0700	16	-0.00	1.0000	0.05	-29.8272	29.8267
Micro	minus	minus	Small	minus	plus	-0.00052	14.0691	16	-0.00	1.0000	0.05	-29.8257	29.8247
Micro	minus	minus	Small	plus	minus	-9.3399	9.9501	16	-0.94	0.3619	0.05	-30.4332	11.7534
Micro	minus	minus	Small	plus	plus	-9.8681	9.9499	16	-0.99	0.3361	0.05	-30.9611	11.2248
Micro	minus	plus	Micro	plus	minus	-9.0487	9.9486	16	-0.91	0.3766	0.05	-30.1389	12.0414
Micro	minus	plus	Micro	plus	plus	-9.5783	9.9484	16	-0.96	0.3500	0.05	-30.6679	11.5114
Micro	minus	plus	Small	minus	minus	0.000053	14.0688	16	0.00	1.0000	0.05	-29.8245	29.8246

The GLIMMIX Procedure

Differences of Colony*alpha*Wnt1eYF Least Squares Means													
Colony_Size	alpha	Wnt1eYFP	_Colony_Size	_alpha	_Wnt1eYFP	Estimate	Standard Error	DF	t Value	Pr > |t|	Alpha	Lower	Upper
Micro	minus	plus	Small	minus	plus	-0.00019	14.0679	16	-0.00	1.0000	0.05	-29.8229	29.8225
Micro	minus	plus	Small	plus	minus	-9.3395	9.9485	16	-0.94	0.3618	0.05	-30.4294	11.7503
Micro	minus	plus	Small	plus	plus	-9.8678	9.9483	16	-0.99	0.3360	0.05	-30.9573	11.2217
Micro	plus	minus	Micro	plus	plus	-0.5295	0.1388	16	-3.81	0.0015	0.05	-0.8239	-0.2352
Micro	plus	minus	Small	minus	minus	9.0488	9.9489	16	0.91	0.3766	0.05	-12.0419	30.1395
Micro	plus	minus	Small	minus	plus	9.0485	9.9475	16	0.91	0.3765	0.05	-12.0393	30.1364
Micro	plus	minus	Small	plus	minus	-0.2908	0.1426	16	-2.04	0.0582	0.05	-0.5931	0.01143
Micro	plus	minus	Small	plus	plus	-0.8191	0.1325	16	-6.18	<.0001	0.05	-1.1000	-0.5382
Micro	plus	plus	Small	minus	minus	9.5783	9.9487	16	0.96	0.3500	0.05	-11.5119	30.6685
Micro	plus	plus	Small	minus	plus	9.5781	9.9473	16	0.96	0.3499	0.05	-11.5093	30.6654
Micro	plus	plus	Small	plus	minus	0.2387	0.1278	16	1.87	0.0803	0.05	-0.03228	0.5097
Micro	plus	plus	Small	plus	plus	-0.2896	0.1094	16	-2.65	0.0176	0.05	-0.5216	-0.05755
Small	minus	minus	Small	minus	plus	-0.00024	14.0681	16	-0.00	1.0000	0.05	-29.8234	29.8229
Small	minus	minus	Small	plus	minus	-9.3396	9.9487	16	-0.94	0.3618	0.05	-30.4300	11.7508
Small	minus	minus	Small	plus	plus	-9.8679	9.9486	16	-0.99	0.3360	0.05	-30.9579	11.2222
Small	minus	plus	Small	plus	minus	-9.3393	9.9474	16	-0.94	0.3618	0.05	-30.4269	11.7482
Small	minus	plus	Small	plus	plus	-9.8676	9.9472	16	-0.99	0.3360	0.05	-30.9548	11.2195
Small	plus	minus	Small	plus	plus	-0.5283	0.1209	16	-4.37	0.0005	0.05	-0.7846	-0.2720

The GLIMMIX Procedure

Model Information	
**Data Set**	WORK.FIG4AIII
**Response Variable**	Count
**Response Distribution**	Poisson
**Link Function**	Log
**Variance Function**	Default
**Variance Matrix Blocked By**	Dish_ID
**Estimation Technique**	Maximum Likelihood
**Likelihood Approximation**	Laplace
**Degrees of Freedom Method**	Containment

Class Level Information		
**Class**	**Levels**	**Values**
**Colony_Type**	3	BFU-E CFU-GEMM CFU-GM
**Condition**	22	10.5EC+!0.5Me 11.5 EC Only 11.5EC+11.5Me 13.5EC+10.5Me 13.5EC+11.5Me Ao-EC Only AoEC+10.5MesP AoEC+11.5MesP E10.5-EC Only E10.5-MesP1+ E10.5-MesP1- E11.5MesP1+On E11.5MesP1-On E13.5 EC Only H-EC Only H-EC+10.5MesP H-EC+11.5MesP I-EC Only I-EC+10.5MesP I-EC+11.5MesP L-E only L-E+11.5Mesp1
**Dish_ID**	72	1 2 3 4 5 6 7 8 9 10 11 12 13 14 15 16 17 18 19 20 21 22 23 24 25 26 27 28 29 30 31 32 33 34 35 36 37 38 39 40 41 42 43 44 45 46 47 48 49 50 51 52 53 54 55 56 57 58 59 60 61 62 63 64 65 66 67 68 69 70 71 72

**Number of Observations Read**	216
**Number of Observations Used**	216

Dimensions	
**G-side Cov. Parameters**	1
**Columns in X**	92
**Columns in Z per Subject**	1
**Subjects (Blocks in V)**	72
**Max Obs per Subject**	3

The GLIMMIX Procedure

Optimization Information	
**Optimization Technique**	Dual Quasi-Newton
**Parameters in Optimization**	67
**Lower Boundaries**	1
**Upper Boundaries**	0
**Fixed Effects**	Not Profiled
**Starting From**	GLM estimates

The GLIMMIX Procedure

Iteration History

Iteration	Restarts	Evaluations	Objective Function	Change	Max Gradient
0	0	4	1511.2398655	.	1460.939
1	0	2	1167.1114165	344.12844900	198.067
2	0	3	1162.1748261	4.93659042	42.61286
3	0	3	1161.4390164	0.73580968	40.01952
4	0	6	1145.2461636	16.19285278	82.32246
5	0	4	1133.3090007	11.93716297	33.94357
6	0	3	1129.5182957	3.79070497	15.67617
7	0	3	1128.6246755	0.89362017	14.12455
8	0	4	1125.7267608	2.89791473	26.4537
9	0	4	1118.5733629	7.15339793	33.89422
10	0	3	1115.1501924	3.42317048	25.85825
11	0	3	1114.8082994	0.34189296	3.670649
12	0	3	1114.7832718	0.02502768	2.102771
13	0	4	1114.6826044	0.10066731	5.73105
14	0	2	1114.5315975	0.15100699	7.091101
15	0	2	1114.4554315	0.07616600	5.684974
16	0	2	1114.3491596	0.10627183	3.455984
17	0	2	1114.1669906	0.18216904	4.107378
18	0	2	1113.8674715	0.29951911	7.570414
19	0	2	1113.5042166	0.36325493	6.208393
20	0	2	1113.2529108	0.25130577	6.86211
21	0	2	1113.1204401	0.13247071	6.032478
22	0	2	1112.9205568	0.19988327	5.228772
23	0	2	1112.639025	0.28153183	4.549196

The GLIMMIX Procedure

Iteration History					
Iteration	Restarts	Evaluations	Objective Function	Change	Max Gradient
**24**	**0**	3	1112.4994218	0.13960318	2.117515
**25**	**0**	3	1112.4730308	0.02639103	1.386604
**26**	**0**	4	1112.3958121	0.07721865	2.442479
**27**	**0**	3	1112.3719977	0.02381441	1.462078
**28**	**0**	3	1112.3660773	0.00592042	0.588279
**29**	**0**	3	1112.3651784	0.00089890	0.160606
**30**	**0**	3	1112.3646082	0.00057014	0.199074
**31**	**0**	4	1112.3629416	0.00166663	0.497214
**32**	**0**	2	1112.3610683	0.00187327	0.256285
**33**	**0**	2	1112.3596443	0.00142408	0.367014
**34**	**0**	2	1112.3574574	0.00218682	0.288764
**35**	**0**	4	1112.3374123	0.02004513	1.2069
**36**	**0**	4	1112.1384866	0.19892572	3.027169
**37**	**0**	2	1111.8458693	0.29261726	3.310532
**38**	**0**	3	1111.7020192	0.14385011	0.655963
**39**	**0**	3	1111.6976213	0.00439789	0.250931
**40**	**0**	3	1111.6969318	0.00068955	0.163466
**41**	**0**	2	1111.6967198	0.00021199	0.372702
**42**	**0**	4	1111.695706	0.00101375	0.163745
**43**	**0**	6	1111.6487932	0.04691284	2.908342
**44**	**0**	3	1111.6253472	0.02344602	0.243902
**45**	**0**	3	1111.6184906	0.00685659	0.272569
**46**	**0**	2	1111.6074277	0.01106284	0.609713
**47**	**0**	3	1111.6039177	0.00351001	0.357656

The GLIMMIX Procedure

Iteration History					
Iteration	Restarts	Evaluations	Objective Function	Change	Max Gradient
48	0	3	1111.6031726	0.00074509	0.257193
49	0	2	1111.6023246	0.00084806	0.382893
50	0	4	1111.5934366	0.00888799	1.513468
51	0	4	1111.5661025	0.02733411	0.996352
52	0	4	1111.4488493	0.11725316	2.508095
53	0	3	1111.4271768	0.02167248	0.35848
54	0	3	1111.4257087	0.00146815	0.348201
55	0	2	1111.4235882	0.00212054	0.374644
56	0	4	1111.4002328	0.02335533	2.175885
57	0	4	1111.3108237	0.08940908	1.713678
58	0	2	1111.1711407	0.13968302	0.909629
59	0	3	1111.1527538	0.01838692	0.307477
60	0	3	1111.147214	0.00553985	0.393262
61	0	2	1111.1398508	0.00736319	0.488378
62	0	2	1111.1303497	0.00950108	0.136576
63	0	3	1111.1266055	0.00374422	0.268358
64	0	3	1111.1250248	0.00158070	0.05974
65	0	3	1111.1243945	0.00063030	0.120917
66	0	3	1111.1241445	0.00025000	0.028492
67	0	3	1111.1240452	0.00009926	0.039684
68	0	3	1111.1240176	0.00002766	0.010088
69	0	3	1111.1240114	0.00000619	0.010335

Convergence criterion (GCONV=1E-8) satisfied.

The GLIMMIX Procedure

Fit Statistics	
**-2 Log Likelihood**	1111.12
**AIC (smaller is better)**	1245.12
**AICC (smaller is better)**	1306.69
**BIC (smaller is better)**	1397.66
**CAIC (smaller is better)**	1464.66
**HQIC (smaller is better)**	1305.85

Fit Statistics for Conditional Distribution	
**-2 log L(Count | r. effects)**	797.15
**Pearson Chi-Square**	135.27
**Pearson Chi-Square / DF**	0.63

Covariance Parameter Estimates

Cov Parm	Subject	Estimate	Standard Error
**Intercept**	Dish_ID	4.2758	1.1534

The GLIMMIX Procedure

Solutions for Fixed Effects							
Effect	Colony_Type	Condition	Estimate	Standard Error	DF	t Value	Pr > |t|
Intercept			6.4692	1.1941	50	5.42	<.0001
Colony_Type	BFU-E		-1.4231	0	100	-Infty	<.0001
Colony_Type	CFU-GEMM		-1.9454	0.06423	100	-30.29	<.0001
Colony_Type	CFU-GM		0	.	.	.	.
Condition		10.5EC+!0.5Me	-3.6799	1.5102	100	-2.44	0.0166
Condition		11.5 EC Only	-3.2338	1.6924	100	-1.91	0.0589
Condition		11.5EC+11.5Me	-3.6299	1.5100	100	-2.40	0.0181
Condition		13.5EC+10.5Me	-0.4019	1.6887	100	-0.24	0.8124
Condition		13.5EC+11.5Me	-0.09847	1.6887	100	-0.06	0.9536
Condition		Ao-EC Only	-46.7804	0	100	-Infty	<.0001
Condition		AoEC+10.5MesP	-0.04450	1.6887	100	-0.03	0.9790
Condition		AoEC+11.5MesP	-0.07725	1.6887	100	-0.05	0.9636
Condition		E10.5-EC Only	-46.7804	0	100	-Infty	<.0001
Condition		E10.5-MesP1+	-46.7804	0	100	-Infty	<.0001
Condition		E10.5-MesP1-	-46.7804	0	100	-Infty	<.0001
Condition		E11.5MesP1+On	-46.7804	0	100	-Infty	<.0001
Condition		E11.5MesP1-On	-46.7804	0	100	-Infty	<.0001
Condition		E13.5 EC Only	-46.7804	0	100	-Infty	<.0001
Condition		H-EC Only	-46.7804	0	100	-Infty	<.0001
Condition		H-EC+10.5MesP	-0.06284	1.6887	100	-0.04	0.9704
Condition		H-EC+11.5MesP	-0.1592	1.6887	100	-0.09	0.9251
Condition		I-EC Only	-46.7804	0	100	-Infty	<.0001
Condition		I-EC+10.5MesP	-2.5777	1.6905	100	-1.52	0.1305
Condition		I-EC+11.5MesP	-3.2688	1.6926	100	-1.93	0.0563

The GLIMMIX Procedure

Solutions for Fixed Effects							
**Effect**	**Colony_Type**	**Condition**	**Estimate**	**Standard Error**	**DF**	**t Value**	**Pr > |t|**
**Condition**		L-E only	-46.7804	0	100	-Infty	<.0001
**Condition**		L-E+11.5Mesp1	0	.	.	.	.
**Colony_Typ*Condition**	BFU-E	10.5EC+!0.5Me	0.1386	0.06912	100	2.00	0.0477
**Colony_Typ*Condition**	BFU-E	11.5 EC Only	-15.7542	608.05	100	-0.03	0.9794
**Colony_Typ*Condition**	BFU-E	11.5EC+11.5Me	0.1382	0.06803	100	2.03	0.0448
**Colony_Typ*Condition**	BFU-E	13.5EC+10.5Me	-0.3320	0.08845	100	-3.75	0.0003
**Colony_Typ*Condition**	BFU-E	13.5EC+11.5Me	0.01554	0.07447	100	0.21	0.8351
**Colony_Typ*Condition**	BFU-E	Ao-EC Only	-9.9427	0	100	-Infty	<.0001
**Colony_Typ*Condition**	BFU-E	AoEC+10.5MesP	0.08657	0.07239	100	1.20	0.2345
**Colony_Typ*Condition**	BFU-E	AoEC+11.5MesP	0.03734	0.07377	100	0.51	0.6138
**Colony_Typ*Condition**	BFU-E	E10.5-EC Only	-9.9427	0	100	-Infty	<.0001
**Colony_Typ*Condition**	BFU-E	E10.5-MesP1+	-9.9427	0	100	-Infty	<.0001
**Colony_Typ*Condition**	BFU-E	E10.5-MesP1-	-9.9427	0	100	-Infty	<.0001
**Colony_Typ*Condition**	BFU-E	E11.5MesP1+On	-9.9427	0	100	-Infty	<.0001
**Colony_Typ*Condition**	BFU-E	E11.5MesP1-On	-9.9427	0	100	-Infty	<.0001
**Colony_Typ*Condition**	BFU-E	E13.5 EC Only	-9.9427	0	100	-Infty	<.0001
**Colony_Typ*Condition**	BFU-E	H-EC Only	-9.9427	0	100	-Infty	<.0001
**Colony_Typ*Condition**	BFU-E	H-EC+10.5MesP	0.01182	0.07384	100	0.16	0.8731
**Colony_Typ*Condition**	BFU-E	H-EC+11.5MesP	-0.03021	0.07651	100	-0.39	0.6938
**Colony_Typ*Condition**	BFU-E	I-EC Only	-9.9427	0	100	-Infty	<.0001
**Colony_Typ*Condition**	BFU-E	I-EC+10.5MesP	-16.4422	608.44	100	-0.03	0.9785
**Colony_Typ*Condition**	BFU-E	I-EC+11.5MesP	-15.7292	608.31	100	-0.03	0.9794
**Colony_Typ*Condition**	BFU-E	L-E only	-9.9427	0	100	-Infty	<.0001
**Colony_Typ*Condition**	BFU-E	L-E+11.5Mesp1	0	.	.	.	.

The GLIMMIX Procedure

Solutions for Fixed Effects							
Effect	Colony_Type	Condition	Estimate	Standard Error	DF	t Value	Pr > |t|
Colony_Typ*Condition	CFU-GEMM	10.5EC+!0.5Me	0.4427	0.08156	100	5.43	<.0001
Colony_Typ*Condition	CFU-GEMM	11.5 EC Only	-15.2306	607.65	100	-0.03	0.9801
Colony_Typ*Condition	CFU-GEMM	11.5EC+11.5Me	0.3308	0.08185	100	4.04	0.0001
Colony_Typ*Condition	CFU-GEMM	13.5EC+10.5Me	0.6625	0.08738	100	7.58	<.0001
Colony_Typ*Condition	CFU-GEMM	13.5EC+11.5Me	-0.01887	0.09350	100	-0.20	0.8404
Colony_Typ*Condition	CFU-GEMM	Ao-EC Only	-9.4183	0	100	-Infty	<.0001
Colony_Typ*Condition	CFU-GEMM	AoEC+10.5MesP	-0.05403	0.09291	100	-0.58	0.5622
Colony_Typ*Condition	CFU-GEMM	AoEC+11.5MesP	0.1396	0.08985	100	1.55	0.1234
Colony_Typ*Condition	CFU-GEMM	E10.5-EC Only	-9.4183	0	100	-Infty	<.0001
Colony_Typ*Condition	CFU-GEMM	E10.5-MesP1+	-9.4183	0	100	-Infty	<.0001
Colony_Typ*Condition	CFU-GEMM	E10.5-MesP1-	-9.4183	0	100	-Infty	<.0001
Colony_Typ*Condition	CFU-GEMM	E11.5MesP1+On	-9.4183	0	100	-Infty	<.0001
Colony_Typ*Condition	CFU-GEMM	E11.5MesP1-On	-9.4183	0	100	-Infty	<.0001
Colony_Typ*Condition	CFU-GEMM	E13.5 EC Only	-9.4183	0	100	-Infty	<.0001
Colony_Typ*Condition	CFU-GEMM	H-EC Only	-9.4183	0	100	-Infty	<.0001
Colony_Typ*Condition	CFU-GEMM	H-EC+10.5MesP	-0.1044	0.09450	100	-1.10	0.2721
Colony_Typ*Condition	CFU-GEMM	H-EC+11.5MesP	-0.1166	0.09744	100	-1.20	0.2341
Colony_Typ*Condition	CFU-GEMM	I-EC Only	-9.4183	0	100	-Infty	<.0001
Colony_Typ*Condition	CFU-GEMM	I-EC+10.5MesP	1.1215	0.1590	100	7.05	<.0001
Colony_Typ*Condition	CFU-GEMM	I-EC+11.5MesP	1.0803	0.2203	100	4.90	<.0001
Colony_Typ*Condition	CFU-GEMM	L-E only	-9.4183	0	100	-Infty	<.0001
Colony_Typ*Condition	CFU-GEMM	L-E+11.5Mesp1	0	.	.	.	.
Colony_Typ*Condition	CFU-GM	10.5EC+!0.5Me	0	.	.	.	.
Colony_Typ*Condition	CFU-GM	11.5 EC Only	0	.	.	.	.

The SAS System

The GLIMMIX Procedure

Solutions for Fixed Effects							
Effect	Colony_Type	Condition	Estimate	Standard Error	DF	t Value	Pr > |t|
**Colony_Typ*Condition**	CFU-GM	11.5EC+11.5Me	0	.	.	.	.
**Colony_Typ*Condition**	CFU-GM	13.5EC+10.5Me	0	.	.	.	.
**Colony_Typ*Condition**	CFU-GM	13.5EC+11.5Me	0	.	.	.	.
**Colony_Typ*Condition**	CFU-GM	Ao-EC Only	0	.	.	.	.
**Colony_Typ*Condition**	CFU-GM	AoEC+10.5MesP	0	.	.	.	.
**Colony_Typ*Condition**	CFU-GM	AoEC+11.5MesP	0	.	.	.	.
**Colony_Typ*Condition**	CFU-GM	E10.5-EC Only	0	.	.	.	.
**Colony_Typ*Condition**	CFU-GM	E10.5-MesP1+	0	.	.	.	.
**Colony_Typ*Condition**	CFU-GM	E10.5-MesP1-	0	.	.	.	.
**Colony_Typ*Condition**	CFU-GM	E11.5MesP1+On	0	.	.	.	.
**Colony_Typ*Condition**	CFU-GM	E11.5MesP1-On	0	.	.	.	.
**Colony_Typ*Condition**	CFU-GM	E13.5 EC Only	0	.	.	.	.
**Colony_Typ*Condition**	CFU-GM	H-EC Only	0	.	.	.	.
**Colony_Typ*Condition**	CFU-GM	H-EC+10.5MesP	0	.	.	.	.
**Colony_Typ*Condition**	CFU-GM	H-EC+11.5MesP	0	.	.	.	.
**Colony_Typ*Condition**	CFU-GM	I-EC Only	0	.	.	.	.
**Colony_Typ*Condition**	CFU-GM	I-EC+10.5MesP	0	.	.	.	.
**Colony_Typ*Condition**	CFU-GM	I-EC+11.5MesP	0	.	.	.	.
**Colony_Typ*Condition**	CFU-GM	L-E only	0	.	.	.	.
**Colony_Typ*Condition**	CFU-GM	L-E+11.5Mesp1	0	.	.	.	.

The GLIMMIX Procedure

Type III Tests of Fixed Effects				
Effect	Num DF	Den DF	F Value	Pr > F
Colony_Type	2	100	0.04	0.9567
Condition	21	100	9.42E35	<.0001
Colony_Typ*Condition	22	100	11.13	<.0001

Condition Least Squares Means												
Condition	Estimate	Standard Error	DF	t Value	Pr > |t|	Alpha	Lower	Upper	Mean	Standard Error Mean	Lower Mean	Upper Mean
10.5EC+!0.5Me	1.8603	0.9251	100	2.01	0.0470	0.05	0.02493	3.6956	6.4255	5.9441	1.0252	40.2702
11.5 EC Only	-8.2157	286.54	100	-0.03	0.9772	0.05	-576.71	560.28	0.000270	0.07748	345E-253	2.12E243
11.5EC+11.5Me	1.8728	0.9246	100	2.03	0.0455	0.05	0.03833	3.7073	6.5065	6.0162	1.0391	40.7426
13.5EC+10.5Me	5.0546	1.1947	100	4.23	<.0001	0.05	2.6844	7.4249	156.75	187.27	14.6495	1677.21
13.5EC+11.5Me	5.2468	1.1946	100	4.39	<.0001	0.05	2.8769	7.6168	189.96	226.92	17.7585	2032.04
Ao-EC Only	-47.8876	1.1943	100	-40.10	<.0001	0.05	-50.2570	-45.5183	1.59E-21	1.9E-21	1.49E-22	1.7E-20
AoEC+10.5MesP	5.3128	1.1945	100	4.45	<.0001	0.05	2.9429	7.6827	202.91	242.38	18.9699	2170.38
AoEC+11.5MesP	5.3281	1.1945	100	4.46	<.0001	0.05	2.9583	7.6980	206.05	246.14	19.2643	2203.99
E10.5-EC Only	-47.8876	1.1943	100	-40.10	<.0001	0.05	-50.2570	-45.5183	1.59E-21	1.9E-21	1.49E-22	1.7E-20
E10.5-MesP1+	-47.8876	1.1943	100	-40.10	<.0001	0.05	-50.2570	-45.5183	1.59E-21	1.9E-21	1.49E-22	1.7E-20
E10.5-MesP1-	-47.8876	1.1943	100	-40.10	<.0001	0.05	-50.2570	-45.5183	1.59E-21	1.9E-21	1.49E-22	1.7E-20
E11.5MesP1+On	-47.8876	1.1943	100	-40.10	<.0001	0.05	-50.2570	-45.5183	1.59E-21	1.9E-21	1.49E-22	1.7E-20
E11.5MesP1-On	-47.8876	1.1943	100	-40.10	<.0001	0.05	-50.2570	-45.5183	1.59E-21	1.9E-21	1.49E-22	1.7E-20
E13.5 EC Only	-47.8876	1.1943	100	-40.10	<.0001	0.05	-50.2570	-45.5183	1.59E-21	1.9E-21	1.49E-22	1.7E-20
H-EC Only	-47.8876	1.1943	100	-40.10	<.0001	0.05	-50.2570	-45.5183	1.59E-21	1.9E-21	1.49E-22	1.7E-20
H-EC+10.5MesP	5.2527	1.1946	100	4.40	<.0001	0.05	2.8828	7.6227	191.09	228.26	17.8636	2044.03
H-EC+11.5MesP	5.1383	1.1946	100	4.30	<.0001	0.05	2.7682	7.5084	170.42	203.59	15.9299	1823.27

The GLIMMIX Procedure

Condition Least Squares Means

Condition	Estimate	Standard Error	DF	t Value	Pr > |t|	Alpha	Lower	Upper	Mean	Standard Error Mean	Lower Mean	Upper Mean
I-EC Only	-47.8876	1.1943	100	-40.10	<.0001	0.05	-50.2570	-45.5183	1.59E-21	1.9E-21	1.49E-22	1.7E-20
I-EC+10.5MesP	-2.3382	202.82	100	-0.01	0.9908	0.05	-404.72	400.05	0.09650	19.5716	17E-177	5.47E173
I-EC+11.5MesP	-2.8053	202.77	100	-0.01	0.9890	0.05	-405.10	399.49	0.06049	12.2653	116E-178	3.15E173
L-E only	-47.8876	1.1943	100	-40.10	<.0001	0.05	-50.2570	-45.5183	1.59E-21	1.9E-21	1.49E-22	1.7E-20
L-E+11.5Mesp1	5.3464	1.1943	100	4.48	<.0001	0.05	2.9770	7.7158	209.85	250.62	19.6296	2243.45

The GLIMMIX Procedure

Differences of Condition Least Squares Means

Condition	_Condition	Estimate	Standard Error	DF	t Value	Pr > |t|	Alpha	Lower	Upper
10.5EC+!0.5Me	11.5 EC Only	10.0759	286.55	100	0.04	0.9720	0.05	-558.42	578.57
10.5EC+!0.5Me	11.5EC+11.5Me	-0.01253	1.2852	100	-0.01	0.9922	0.05	-2.5623	2.5372
10.5EC+!0.5Me	13.5EC+10.5Me	-3.1944	1.5109	100	-2.11	0.0370	0.05	-6.1919	-0.1969
10.5EC+!0.5Me	13.5EC+11.5Me	-3.3866	1.5108	100	-2.24	0.0272	0.05	-6.3840	-0.3891
10.5EC+!0.5Me	Ao-EC Only	49.7479	1.5106	100	32.93	<.0001	0.05	46.7508	52.7450
10.5EC+!0.5Me	AoEC+10.5MesP	-3.4525	1.5108	100	-2.29	0.0244	0.05	-6.4499	-0.4551
10.5EC+!0.5Me	AoEC+11.5MesP	-3.4679	1.5108	100	-2.30	0.0238	0.05	-6.4653	-0.4704
10.5EC+!0.5Me	E10.5-EC Only	49.7479	1.5106	100	32.93	<.0001	0.05	46.7508	52.7450
10.5EC+!0.5Me	E10.5-MesP1+	49.7479	1.5106	100	32.93	<.0001	0.05	46.7508	52.7450
10.5EC+!0.5Me	E10.5-MesP1-	49.7479	1.5106	100	32.93	<.0001	0.05	46.7508	52.7450
10.5EC+!0.5Me	E11.5MesP1+On	49.7479	1.5106	100	32.93	<.0001	0.05	46.7508	52.7450
10.5EC+!0.5Me	E11.5MesP1-On	49.7479	1.5106	100	32.93	<.0001	0.05	46.7508	52.7450
10.5EC+!0.5Me	E13.5 EC Only	49.7479	1.5106	100	32.93	<.0001	0.05	46.7508	52.7450
10.5EC+!0.5Me	H-EC Only	49.7479	1.5106	100	32.93	<.0001	0.05	46.7508	52.7450
10.5EC+!0.5Me	H-EC+10.5MesP	-3.3924	1.5108	100	-2.25	0.0269	0.05	-6.3899	-0.3950
10.5EC+!0.5Me	H-EC+11.5MesP	-3.2780	1.5109	100	-2.17	0.0324	0.05	-6.2757	-0.2804
10.5EC+!0.5Me	I-EC Only	49.7479	1.5106	100	32.93	<.0001	0.05	46.7508	52.7450
10.5EC+!0.5Me	I-EC+10.5MesP	4.1985	202.82	100	0.02	0.9835	0.05	-398.19	406.59
10.5EC+!0.5Me	I-EC+11.5MesP	4.6656	202.78	100	0.02	0.9817	0.05	-397.64	406.97
10.5EC+!0.5Me	L-E only	49.7479	1.5106	100	32.93	<.0001	0.05	46.7508	52.7450
10.5EC+!0.5Me	L-E+11.5Mesp1	-3.4861	1.5106	100	-2.31	0.0231	0.05	-6.4832	-0.4891
11.5 EC Only	11.5EC+11.5Me	-10.0885	286.55	100	-0.04	0.9720	0.05	-578.59	558.41
11.5 EC Only	13.5EC+10.5Me	-13.2703	286.55	100	-0.05	0.9632	0.05	-581.77	555.23
11.5 EC Only	13.5EC+11.5Me	-13.4625	286.55	100	-0.05	0.9626	0.05	-581.96	555.04

The GLIMMIX Procedure

Differences of Condition Least Squares Means									
Condition	_Condition	Estimate	Standard Error	DF	t Value	Pr > |t|	Alpha	Lower	Upper
11.5 EC Only	Ao-EC Only	39.6720	286.55	100	0.14	0.8902	0.05	-528.83	608.17
11.5 EC Only	AoEC+10.5MesP	-13.5284	286.55	100	-0.05	0.9624	0.05	-582.03	554.97
11.5 EC Only	AoEC+11.5MesP	-13.5438	286.55	100	-0.05	0.9624	0.05	-582.04	554.96
11.5 EC Only	E10.5-EC Only	39.6720	286.55	100	0.14	0.8902	0.05	-528.83	608.17
11.5 EC Only	E10.5-MesP1+	39.6720	286.55	100	0.14	0.8902	0.05	-528.83	608.17
11.5 EC Only	E10.5-MesP1-	39.6720	286.55	100	0.14	0.8902	0.05	-528.83	608.17
11.5 EC Only	E11.5MesP1+On	39.6720	286.55	100	0.14	0.8902	0.05	-528.83	608.17
11.5 EC Only	E11.5MesP1-On	39.6720	286.55	100	0.14	0.8902	0.05	-528.83	608.17
11.5 EC Only	E13.5 EC Only	39.6720	286.55	100	0.14	0.8902	0.05	-528.83	608.17
11.5 EC Only	H-EC Only	39.6720	286.55	100	0.14	0.8902	0.05	-528.83	608.17
11.5 EC Only	H-EC+10.5MesP	-13.4684	286.55	100	-0.05	0.9626	0.05	-581.97	555.03
11.5 EC Only	H-EC+11.5MesP	-13.3540	286.55	100	-0.05	0.9629	0.05	-581.85	555.15
11.5 EC Only	I-EC Only	39.6720	286.55	100	0.14	0.8902	0.05	-528.83	608.17
11.5 EC Only	I-EC+10.5MesP	-5.8774	351.06	100	-0.02	0.9867	0.05	-702.37	690.61
11.5 EC Only	I-EC+11.5MesP	-5.4104	351.03	100	-0.02	0.9877	0.05	-701.85	691.03
11.5 EC Only	L-E only	39.6720	286.55	100	0.14	0.8902	0.05	-528.83	608.17
11.5 EC Only	L-E+11.5Mesp1	-13.5621	286.55	100	-0.05	0.9623	0.05	-582.06	554.94
11.5EC+11.5Me	13.5EC+10.5Me	-3.1818	1.5106	100	-2.11	0.0377	0.05	-6.1788	-0.1849
11.5EC+11.5Me	13.5EC+11.5Me	-3.3740	1.5106	100	-2.23	0.0277	0.05	-6.3710	-0.3771
11.5EC+11.5Me	Ao-EC Only	49.7604	1.5104	100	32.95	<.0001	0.05	46.7639	52.7570
11.5EC+11.5Me	AoEC+10.5MesP	-3.4400	1.5105	100	-2.28	0.0249	0.05	-6.4368	-0.4431
11.5EC+11.5Me	AoEC+11.5MesP	-3.4553	1.5106	100	-2.29	0.0243	0.05	-6.4523	-0.4584
11.5EC+11.5Me	E10.5-EC Only	49.7604	1.5104	100	32.95	<.0001	0.05	46.7639	52.7570
11.5EC+11.5Me	E10.5-MesP1+	49.7604	1.5104	100	32.95	<.0001	0.05	46.7639	52.7570

The GLIMMIX Procedure

Differences of Condition Least Squares Means									
Condition	_Condition	Estimate	Standard Error	DF	t Value	Pr > |t|	Alpha	Lower	Upper
11.5EC+11.5Me	E10.5-MesP1-	49.7604	1.5104	100	32.95	<.0001	0.05	46.7639	52.7570
11.5EC+11.5Me	E11.5MesP1+On	49.7604	1.5104	100	32.95	<.0001	0.05	46.7639	52.7570
11.5EC+11.5Me	E11.5MesP1-On	49.7604	1.5104	100	32.95	<.0001	0.05	46.7639	52.7570
11.5EC+11.5Me	E13.5 EC Only	49.7604	1.5104	100	32.95	<.0001	0.05	46.7639	52.7570
11.5EC+11.5Me	H-EC Only	49.7604	1.5104	100	32.95	<.0001	0.05	46.7639	52.7570
11.5EC+11.5Me	H-EC+10.5MesP	-3.3799	1.5106	100	-2.24	0.0275	0.05	-6.3769	-0.3830
11.5EC+11.5Me	H-EC+11.5MesP	-3.2655	1.5107	100	-2.16	0.0330	0.05	-6.2626	-0.2684
11.5EC+11.5Me	I-EC Only	49.7604	1.5104	100	32.95	<.0001	0.05	46.7639	52.7570
11.5EC+11.5Me	I-EC+10.5MesP	4.2110	202.82	100	0.02	0.9835	0.05	-398.18	406.60
11.5EC+11.5Me	I-EC+11.5MesP	4.6781	202.78	100	0.02	0.9816	0.05	-397.63	406.98
11.5EC+11.5Me	L-E only	49.7604	1.5104	100	32.95	<.0001	0.05	46.7639	52.7570
11.5EC+11.5Me	L-E+11.5Mesp1	-3.4736	1.5104	100	-2.30	0.0235	0.05	-6.4701	-0.4771
13.5EC+10.5Me	13.5EC+11.5Me	-0.1922	1.6895	100	-0.11	0.9097	0.05	-3.5440	3.1597
13.5EC+10.5Me	Ao-EC Only	52.9423	1.6892	100	31.34	<.0001	0.05	49.5909	56.2937
13.5EC+10.5Me	AoEC+10.5MesP	-0.2581	1.6894	100	-0.15	0.8789	0.05	-3.6099	3.0937
13.5EC+10.5Me	AoEC+11.5MesP	-0.2735	1.6894	100	-0.16	0.8717	0.05	-3.6253	3.0783
13.5EC+10.5Me	E10.5-EC Only	52.9423	1.6892	100	31.34	<.0001	0.05	49.5909	56.2937
13.5EC+10.5Me	E10.5-MesP1+	52.9423	1.6892	100	31.34	<.0001	0.05	49.5909	56.2937
13.5EC+10.5Me	E10.5-MesP1-	52.9423	1.6892	100	31.34	<.0001	0.05	49.5909	56.2937
13.5EC+10.5Me	E11.5MesP1+On	52.9423	1.6892	100	31.34	<.0001	0.05	49.5909	56.2937
13.5EC+10.5Me	E11.5MesP1-On	52.9423	1.6892	100	31.34	<.0001	0.05	49.5909	56.2937
13.5EC+10.5Me	E13.5 EC Only	52.9423	1.6892	100	31.34	<.0001	0.05	49.5909	56.2937
13.5EC+10.5Me	H-EC Only	52.9423	1.6892	100	31.34	<.0001	0.05	49.5909	56.2937
13.5EC+10.5Me	H-EC+10.5MesP	-0.1981	1.6895	100	-0.12	0.9069	0.05	-3.5499	3.1538

The GLIMMIX Procedure

Differences of Condition Least Squares Means									
Condition	_Condition	Estimate	Standard Error	DF	t Value	Pr > |t|	Alpha	Lower	Upper
13.5EC+10.5Me	H-EC+11.5MesP	-0.08365	1.6895	100	-0.05	0.9606	0.05	-3.4356	3.2683
13.5EC+10.5Me	I-EC Only	52.9423	1.6892	100	31.34	<.0001	0.05	49.5909	56.2937
13.5EC+10.5Me	I-EC+10.5MesP	7.3929	202.82	100	0.04	0.9710	0.05	-395.00	409.78
13.5EC+10.5Me	I-EC+11.5MesP	7.8600	202.78	100	0.04	0.9692	0.05	-394.45	410.17
13.5EC+10.5Me	L-E only	52.9423	1.6892	100	31.34	<.0001	0.05	49.5909	56.2937
13.5EC+10.5Me	L-E+11.5Mesp1	-0.2918	1.6892	100	-0.17	0.8632	0.05	-3.6432	3.0596
13.5EC+11.5Me	Ao-EC Only	53.1345	1.6891	100	31.46	<.0001	0.05	49.7832	56.4857
13.5EC+11.5Me	AoEC+10.5MesP	-0.06593	1.6893	100	-0.04	0.9689	0.05	-3.4175	3.2857
13.5EC+11.5Me	AoEC+11.5MesP	-0.08131	1.6893	100	-0.05	0.9617	0.05	-3.4329	3.2703
13.5EC+11.5Me	E10.5-EC Only	53.1345	1.6891	100	31.46	<.0001	0.05	49.7832	56.4857
13.5EC+11.5Me	E10.5-MesP1+	53.1345	1.6891	100	31.46	<.0001	0.05	49.7832	56.4857
13.5EC+11.5Me	E10.5-MesP1-	53.1345	1.6891	100	31.46	<.0001	0.05	49.7832	56.4857
13.5EC+11.5Me	E11.5MesP1+On	53.1345	1.6891	100	31.46	<.0001	0.05	49.7832	56.4857
13.5EC+11.5Me	E11.5MesP1-On	53.1345	1.6891	100	31.46	<.0001	0.05	49.7832	56.4857
13.5EC+11.5Me	E13.5 EC Only	53.1345	1.6891	100	31.46	<.0001	0.05	49.7832	56.4857
13.5EC+11.5Me	H-EC Only	53.1345	1.6891	100	31.46	<.0001	0.05	49.7832	56.4857
13.5EC+11.5Me	H-EC+10.5MesP	-0.00589	1.6894	100	-0.00	0.9972	0.05	-3.3575	3.3457
13.5EC+11.5Me	H-EC+11.5MesP	0.1085	1.6894	100	0.06	0.9489	0.05	-3.2432	3.4603
13.5EC+11.5Me	I-EC Only	53.1345	1.6891	100	31.46	<.0001	0.05	49.7832	56.4857
13.5EC+11.5Me	I-EC+10.5MesP	7.5851	202.82	100	0.04	0.9702	0.05	-394.81	409.98
13.5EC+11.5Me	I-EC+11.5MesP	8.0521	202.78	100	0.04	0.9684	0.05	-394.25	410.36
13.5EC+11.5Me	L-E only	53.1345	1.6891	100	31.46	<.0001	0.05	49.7832	56.4857
13.5EC+11.5Me	L-E+11.5Mesp1	-0.09958	1.6891	100	-0.06	0.9531	0.05	-3.4508	3.2516
Ao-EC Only	AoEC+10.5MesP	-53.2004	1.6891	100	-31.50	<.0001	0.05	-56.5516	-49.8492

The GLIMMIX Procedure

Differences of Condition Least Squares Means									
Condition	_Condition	Estimate	Standard Error	DF	t Value	Pr > |t|	Alpha	Lower	Upper
Ao-EC Only	AoEC+11.5MesP	-53.2158	1.6891	100	-31.51	<.0001	0.05	-56.5669	-49.8646
Ao-EC Only	E10.5-EC Only	1.07E-14	.	.	.	.	.	.	.
Ao-EC Only	E10.5-MesP1+	1.97E-13	.	.	.	.	.	.	.
Ao-EC Only	E10.5-MesP1-	1.34E-13	.	.	.	.	.	.	.
Ao-EC Only	E11.5MesP1+On	1.79E-13	.	.	.	.	.	.	.
Ao-EC Only	E11.5MesP1-On	2.53E-14	.	.	.	.	.	.	.
Ao-EC Only	E13.5 EC Only	1.06E-13	.	.	.	.	.	.	.
Ao-EC Only	H-EC Only	1.02E-13	.	.	.	.	.	.	.
Ao-EC Only	H-EC+10.5MesP	-53.1403	1.6891	100	-31.46	<.0001	0.05	-56.4916	-49.7891
Ao-EC Only	H-EC+11.5MesP	-53.0259	1.6892	100	-31.39	<.0001	0.05	-56.3772	-49.6746
Ao-EC Only	I-EC Only	1.26E-13	.	.	.	.	.	.	.
Ao-EC Only	I-EC+10.5MesP	-45.5494	202.82	100	-0.22	0.8228	0.05	-447.94	356.84
Ao-EC Only	I-EC+11.5MesP	-45.0823	202.78	100	-0.22	0.8245	0.05	-447.39	357.22
Ao-EC Only	L-E only	2.36E-13	.	.	.	.	.	.	.
Ao-EC Only	L-E+11.5Mesp1	-53.2340	0	100	-Infty	<.0001	.	.	.
AoEC+10.5MesP	AoEC+11.5MesP	-0.01539	1.6893	100	-0.01	0.9928	0.05	-3.3669	3.3362
AoEC+10.5MesP	E10.5-EC Only	53.2004	1.6891	100	31.50	<.0001	0.05	49.8492	56.5516
AoEC+10.5MesP	E10.5-MesP1+	53.2004	1.6891	100	31.50	<.0001	0.05	49.8492	56.5516
AoEC+10.5MesP	E10.5-MesP1-	53.2004	1.6891	100	31.50	<.0001	0.05	49.8492	56.5516
AoEC+10.5MesP	E11.5MesP1+On	53.2004	1.6891	100	31.50	<.0001	0.05	49.8492	56.5516
AoEC+10.5MesP	E11.5MesP1-On	53.2004	1.6891	100	31.50	<.0001	0.05	49.8492	56.5516
AoEC+10.5MesP	E13.5 EC Only	53.2004	1.6891	100	31.50	<.0001	0.05	49.8492	56.5516
AoEC+10.5MesP	H-EC Only	53.2004	1.6891	100	31.50	<.0001	0.05	49.8492	56.5516
AoEC+10.5MesP	H-EC+10.5MesP	0.06003	1.6893	100	0.04	0.9717	0.05	-3.2916	3.4116

The GLIMMIX Procedure

Differences of Condition Least Squares Means

Condition	_Condition	Estimate	Standard Error	DF	t Value	Pr > |t|	Alpha	Lower	Upper
AoEC+10.5MesP	H-EC+11.5MesP	0.1745	1.6894	100	0.10	0.9180	0.05	-3.1772	3.5261
AoEC+10.5MesP	I-EC Only	53.2004	1.6891	100	31.50	<.0001	0.05	49.8492	56.5516
AoEC+10.5MesP	I-EC+10.5MesP	7.6510	202.82	100	0.04	0.9700	0.05	-394.74	410.04
AoEC+10.5MesP	I-EC+11.5MesP	8.1181	202.78	100	0.04	0.9681	0.05	-394.19	410.42
AoEC+10.5MesP	L-E only	53.2004	1.6891	100	31.50	<.0001	0.05	49.8492	56.5516
AoEC+10.5MesP	L-E+11.5Mesp1	-0.03365	1.6891	100	-0.02	0.9841	0.05	-3.3848	3.3175
AoEC+11.5MesP	E10.5-EC Only	53.2158	1.6891	100	31.51	<.0001	0.05	49.8646	56.5669
AoEC+11.5MesP	E10.5-MesP1+	53.2158	1.6891	100	31.51	<.0001	0.05	49.8646	56.5669
AoEC+11.5MesP	E10.5-MesP1-	53.2158	1.6891	100	31.51	<.0001	0.05	49.8646	56.5669
AoEC+11.5MesP	E11.5MesP1+On	53.2158	1.6891	100	31.51	<.0001	0.05	49.8646	56.5669
AoEC+11.5MesP	E11.5MesP1-On	53.2158	1.6891	100	31.51	<.0001	0.05	49.8646	56.5669
AoEC+11.5MesP	E13.5 EC Only	53.2158	1.6891	100	31.51	<.0001	0.05	49.8646	56.5669
AoEC+11.5MesP	H-EC Only	53.2158	1.6891	100	31.51	<.0001	0.05	49.8646	56.5669
AoEC+11.5MesP	H-EC+10.5MesP	0.07542	1.6893	100	0.04	0.9645	0.05	-3.2762	3.4270
AoEC+11.5MesP	H-EC+11.5MesP	0.1898	1.6894	100	0.11	0.9107	0.05	-3.1618	3.5415
AoEC+11.5MesP	I-EC Only	53.2158	1.6891	100	31.51	<.0001	0.05	49.8646	56.5669
AoEC+11.5MesP	I-EC+10.5MesP	7.6664	202.82	100	0.04	0.9699	0.05	-394.73	410.06
AoEC+11.5MesP	I-EC+11.5MesP	8.1335	202.78	100	0.04	0.9681	0.05	-394.17	410.44
AoEC+11.5MesP	L-E only	53.2158	1.6891	100	31.51	<.0001	0.05	49.8646	56.5669
AoEC+11.5MesP	L-E+11.5Mesp1	-0.01826	1.6891	100	-0.01	0.9914	0.05	-3.3694	3.3329
E10.5-EC Only	E10.5-MesP1+	1.87E-13	.	.	.	.	.	.	.
E10.5-EC Only	E10.5-MesP1-	1.23E-13	.	.	.	.	.	.	.
E10.5-EC Only	E11.5MesP1+On	1.68E-13	.	.	.	.	.	.	.
E10.5-EC Only	E11.5MesP1-On	1.47E-14	.	.	.	.	.	.	.

The GLIMMIX Procedure

Differences of Condition Least Squares Means									
Condition	_Condition	Estimate	Standard Error	DF	t Value	Pr > |t|	Alpha	Lower	Upper
E10.5-EC Only	E13.5 EC Only	9.55E-14	.	.	.	.	.	.	.
E10.5-EC Only	H-EC Only	9.1E-14	.	.	.	.	.	.	.
E10.5-EC Only	H-EC+10.5MesP	-53.1403	1.6891	100	-31.46	<.0001	0.05	-56.4916	-49.7891
E10.5-EC Only	H-EC+11.5MesP	-53.0259	1.6892	100	-31.39	<.0001	0.05	-56.3772	-49.6746
E10.5-EC Only	I-EC Only	1.15E-13	.	.	.	.	.	.	.
E10.5-EC Only	I-EC+10.5MesP	-45.5494	202.82	100	-0.22	0.8228	0.05	-447.94	356.84
E10.5-EC Only	I-EC+11.5MesP	-45.0823	202.78	100	-0.22	0.8245	0.05	-447.39	357.22
E10.5-EC Only	L-E only	2.26E-13	.	.	.	.	.	.	.
E10.5-EC Only	L-E+11.5Mesp1	-53.2340	0	100	-Infty	<.0001	.	.	.
E10.5-MesP1+	E10.5-MesP1-	-635E-16	.	.	.	.	.	.	.
E10.5-MesP1+	E11.5MesP1+On	-187E-16	.	.	.	.	.	.	.
E10.5-MesP1+	E11.5MesP1-On	-172E-15	.	.	.	.	.	.	.
E10.5-MesP1+	E13.5 EC Only	-91E-15	.	.	.	.	.	.	.
E10.5-MesP1+	H-EC Only	-955E-16	.	.	.	.	.	.	.
E10.5-MesP1+	H-EC+10.5MesP	-53.1403	1.6891	100	-31.46	<.0001	0.05	-56.4916	-49.7891
E10.5-MesP1+	H-EC+11.5MesP	-53.0259	1.6892	100	-31.39	<.0001	0.05	-56.3772	-49.6746
E10.5-MesP1+	I-EC Only	-711E-16	.	.	.	.	.	.	.
E10.5-MesP1+	I-EC+10.5MesP	-45.5494	202.82	100	-0.22	0.8228	0.05	-447.94	356.84
E10.5-MesP1+	I-EC+11.5MesP	-45.0823	202.78	100	-0.22	0.8245	0.05	-447.39	357.22
E10.5-MesP1+	L-E only	3.91E-14	.	.	.	.	.	.	.
E10.5-MesP1+	L-E+11.5Mesp1	-53.2340	0	100	-Infty	<.0001	.	.	.
E10.5-MesP1-	E11.5MesP1+On	4.49E-14	.	.	.	.	.	.	.
E10.5-MesP1-	E11.5MesP1-On	-108E-15	.	.	.	.	.	.	.
E10.5-MesP1-	E13.5 EC Only	-275E-16	.	.	.	.	.	.	.

The GLIMMIX Procedure

Differences of Condition Least Squares Means									
Condition	_Condition	Estimate	Standard Error	DF	t Value	Pr > |t|	Alpha	Lower	Upper
E10.5-MesP1-	H-EC Only	-32E-15	.	.	.	.	.	.	.
E10.5-MesP1-	H-EC+10.5MesP	-53.1403	1.6891	100	-31.46	<.0001	0.05	-56.4916	-49.7891
E10.5-MesP1-	H-EC+11.5MesP	-53.0259	1.6892	100	-31.39	<.0001	0.05	-56.3772	-49.6746
E10.5-MesP1-	I-EC Only	-755E-17	.	.	.	.	.	.	.
E10.5-MesP1-	I-EC+10.5MesP	-45.5494	202.82	100	-0.22	0.8228	0.05	-447.94	356.84
E10.5-MesP1-	I-EC+11.5MesP	-45.0823	202.78	100	-0.22	0.8245	0.05	-447.39	357.22
E10.5-MesP1-	L-E only	1.03E-13	.	.	.	.	.	.	.
E10.5-MesP1-	L-E+11.5Mesp1	-53.2340	0	100	-Infty	<.0001	.	.	.
E11.5MesP1+On	E11.5MesP1-On	-153E-15	.	.	.	.	.	.	.
E11.5MesP1+On	E13.5 EC Only	-724E-16	.	.	.	.	.	.	.
E11.5MesP1+On	H-EC Only	-768E-16	.	.	.	.	.	.	.
E11.5MesP1+On	H-EC+10.5MesP	-53.1403	1.6891	100	-31.46	<.0001	0.05	-56.4916	-49.7891
E11.5MesP1+On	H-EC+11.5MesP	-53.0259	1.6892	100	-31.39	<.0001	0.05	-56.3772	-49.6746
E11.5MesP1+On	I-EC Only	-524E-16	.	.	.	.	.	.	.
E11.5MesP1+On	I-EC+10.5MesP	-45.5494	202.82	100	-0.22	0.8228	0.05	-447.94	356.84
E11.5MesP1+On	I-EC+11.5MesP	-45.0823	202.78	100	-0.22	0.8245	0.05	-447.39	357.22
E11.5MesP1+On	L-E only	5.77E-14	.	.	.	.	.	.	.
E11.5MesP1+On	L-E+11.5Mesp1	-53.2340	0	100	-Infty	<.0001	.	.	.
E11.5MesP1-On	E13.5 EC Only	8.08E-14	.	.	.	.	.	.	.
E11.5MesP1-On	H-EC Only	7.64E-14	.	.	.	.	.	.	.
E11.5MesP1-On	H-EC+10.5MesP	-53.1403	1.6891	100	-31.46	<.0001	0.05	-56.4916	-49.7891
E11.5MesP1-On	H-EC+11.5MesP	-53.0259	1.6892	100	-31.39	<.0001	0.05	-56.3772	-49.6746
E11.5MesP1-On	I-EC Only	1.01E-13	.	.	.	.	.	.	.
E11.5MesP1-On	I-EC+10.5MesP	-45.5494	202.82	100	-0.22	0.8228	0.05	-447.94	356.84

The GLIMMIX Procedure

Differences of Condition Least Squares Means									
Condition	_Condition	Estimate	Standard Error	DF	t Value	Pr > |t|	Alpha	Lower	Upper
E11.5MesP1-On	I-EC+11.5MesP	-45.0823	202.78	100	-0.22	0.8245	0.05	-447.39	357.22
E11.5MesP1-On	L-E only	2.11E-13	.	.	.	.	.	.	.
E11.5MesP1-On	L-E+11.5Mesp1	-53.2340	0	100	-Infty	<.0001	.	.	.
E13.5 EC Only	H-EC Only	-444E-17	.	.	.	.	.	.	.
E13.5 EC Only	H-EC+10.5MesP	-53.1403	1.6891	100	-31.46	<.0001	0.05	-56.4916	-49.7891
E13.5 EC Only	H-EC+11.5MesP	-53.0259	1.6892	100	-31.39	<.0001	0.05	-56.3772	-49.6746
E13.5 EC Only	I-EC Only	2E-14	.	.	.	.	.	.	.
E13.5 EC Only	I-EC+10.5MesP	-45.5494	202.82	100	-0.22	0.8228	0.05	-447.94	356.84
E13.5 EC Only	I-EC+11.5MesP	-45.0823	202.78	100	-0.22	0.8245	0.05	-447.39	357.22
E13.5 EC Only	L-E only	1.3E-13	.	.	.	.	.	.	.
E13.5 EC Only	L-E+11.5Mesp1	-53.2340	0	100	-Infty	<.0001	.	.	.
H-EC Only	H-EC+10.5MesP	-53.1403	1.6891	100	-31.46	<.0001	0.05	-56.4916	-49.7891
H-EC Only	H-EC+11.5MesP	-53.0259	1.6892	100	-31.39	<.0001	0.05	-56.3772	-49.6746
H-EC Only	I-EC Only	2.44E-14	.	.	.	.	.	.	.
H-EC Only	I-EC+10.5MesP	-45.5494	202.82	100	-0.22	0.8228	0.05	-447.94	356.84
H-EC Only	I-EC+11.5MesP	-45.0823	202.78	100	-0.22	0.8245	0.05	-447.39	357.22
H-EC Only	L-E only	1.35E-13	.	.	.	.	.	.	.
H-EC Only	L-E+11.5Mesp1	-53.2340	0	100	-Infty	<.0001	.	.	.
H-EC+10.5MesP	H-EC+11.5MesP	0.1144	1.6894	100	0.07	0.9461	0.05	-3.2373	3.4662
H-EC+10.5MesP	I-EC Only	53.1403	1.6891	100	31.46	<.0001	0.05	49.7891	56.4916
H-EC+10.5MesP	I-EC+10.5MesP	7.5909	202.82	100	0.04	0.9702	0.05	-394.80	409.98
H-EC+10.5MesP	I-EC+11.5MesP	8.0580	202.78	100	0.04	0.9684	0.05	-394.25	410.36
H-EC+10.5MesP	L-E only	53.1403	1.6891	100	31.46	<.0001	0.05	49.7891	56.4916
H-EC+10.5MesP	L-E+11.5Mesp1	-0.09368	1.6891	100	-0.06	0.9559	0.05	-3.4449	3.2575

The GLIMMIX Procedure

Differences of Condition Least Squares Means									
Condition	_Condition	Estimate	Standard Error	DF	t Value	Pr > |t|	Alpha	Lower	Upper
H-EC+11.5MesP	I-EC Only	53.0259	1.6892	100	31.39	<.0001	0.05	49.6746	56.3772
H-EC+11.5MesP	I-EC+10.5MesP	7.4765	202.82	100	0.04	0.9707	0.05	-394.91	409.87
H-EC+11.5MesP	I-EC+11.5MesP	7.9436	202.78	100	0.04	0.9688	0.05	-394.36	410.25
H-EC+11.5MesP	L-E only	53.0259	1.6892	100	31.39	<.0001	0.05	49.6746	56.3772
H-EC+11.5MesP	L-E+11.5Mesp1	-0.2081	1.6892	100	-0.12	0.9022	0.05	-3.5594	3.1432
I-EC Only	I-EC+10.5MesP	-45.5494	202.82	100	-0.22	0.8228	0.05	-447.94	356.84
I-EC Only	I-EC+11.5MesP	-45.0823	202.78	100	-0.22	0.8245	0.05	-447.39	357.22
I-EC Only	L-E only	1.1E-13	.	.	.	.	.	.	.
I-EC Only	L-E+11.5Mesp1	-53.2340	0	100	-Infty	<.0001	.	.	.
I-EC+10.5MesP	I-EC+11.5MesP	0.4671	286.80	100	0.00	0.9987	0.05	-568.53	569.46
I-EC+10.5MesP	L-E only	45.5494	202.82	100	0.22	0.8228	0.05	-356.84	447.94
I-EC+10.5MesP	L-E+11.5Mesp1	-7.6846	202.82	100	-0.04	0.9699	0.05	-410.08	394.71
I-EC+11.5MesP	L-E only	45.0823	202.78	100	0.22	0.8245	0.05	-357.22	447.39
I-EC+11.5MesP	L-E+11.5Mesp1	-8.1517	202.78	100	-0.04	0.9680	0.05	-410.46	394.15
L-E only	L-E+11.5Mesp1	-53.2340	0	100	-Infty	<.0001	.	.	.

The GLIMMIX Procedure

Colony_Typ*Condition Least Squares Means													
Colony_Type	Condition	Estimate	Standard Error	DF	t Value	Pr > |t|	Alpha	Lower	Upper	Mean	Standard Error Mean	Lower Mean	Upper Mean
BFU-E	10.5EC+!0.5Me	1.5048	0.9272	100	1.62	0.1078	0.05	-0.3348	3.3444	4.5033	4.1756	0.7155	28.3438
BFU-E	11.5 EC Only	-13.9419	608.05	100	-0.02	0.9818	0.05	-1220.29	1192.41	8.813E-7	0.000536	0	.
BFU-E	11.5EC+11.5Me	1.5544	0.9267	100	1.68	0.0966	0.05	-0.2842	3.3930	4.7323	4.3855	0.7526	29.7547
BFU-E	13.5EC+10.5Me	4.3122	1.1974	100	3.60	0.0005	0.05	1.9365	6.6879	74.6056	89.3357	6.9346	802.64
BFU-E	13.5EC+11.5Me	4.9632	1.1964	100	4.15	<.0001	0.05	2.5896	7.3369	143.05	171.15	13.3243	1535.87
BFU-E	Ao-EC Only	-51.6769	1.1941	100	-43.28	<.0001	0.05	-54.0459	-49.3079	3.61E-23	4.31E-23	3.37E-24	3.85E-22
BFU-E	AoEC+10.5MesP	5.0882	1.1963	100	4.25	<.0001	0.05	2.7149	7.4616	162.10	193.92	15.1027	1739.89
BFU-E	AoEC+11.5MesP	5.0062	1.1964	100	4.18	<.0001	0.05	2.6327	7.3798	149.34	178.67	13.9114	1603.23
BFU-E	E10.5-EC Only	-51.6769	1.1941	100	-43.28	<.0001	0.05	-54.0459	-49.3079	3.61E-23	4.31E-23	3.37E-24	3.85E-22
BFU-E	E10.5-MesP1+	-51.6769	1.1941	100	-43.28	<.0001	0.05	-54.0459	-49.3079	3.61E-23	4.31E-23	3.37E-24	3.85E-22
BFU-E	E10.5-MesP1-	-51.6769	1.1941	100	-43.28	<.0001	0.05	-54.0459	-49.3079	3.61E-23	4.31E-23	3.37E-24	3.85E-22
BFU-E	E11.5MesP1+On	-51.6769	1.1941	100	-43.28	<.0001	0.05	-54.0459	-49.3079	3.61E-23	4.31E-23	3.37E-24	3.85E-22
BFU-E	E11.5MesP1-On	-51.6769	1.1941	100	-43.28	<.0001	0.05	-54.0459	-49.3079	3.61E-23	4.31E-23	3.37E-24	3.85E-22
BFU-E	E13.5 EC Only	-51.6769	1.1941	100	-43.28	<.0001	0.05	-54.0459	-49.3079	3.61E-23	4.31E-23	3.37E-24	3.85E-22
BFU-E	H-EC Only	-51.6769	1.1941	100	-43.28	<.0001	0.05	-54.0459	-49.3079	3.61E-23	4.31E-23	3.37E-24	3.85E-22
BFU-E	H-EC+10.5MesP	4.9951	1.1964	100	4.18	<.0001	0.05	2.6216	7.3687	147.69	176.69	13.7577	1585.53
BFU-E	H-EC+11.5MesP	4.8568	1.1965	100	4.06	<.0001	0.05	2.4829	7.2307	128.61	153.89	11.9754	1381.18
BFU-E	I-EC Only	-51.6769	1.1941	100	-43.28	<.0001	0.05	-54.0459	-49.3079	3.61E-23	4.31E-23	3.37E-24	3.85E-22
BFU-E	I-EC+10.5MesP	-13.9738	608.44	100	-0.02	0.9817	0.05	-1221.11	1193.16	8.536E-7	0.000519	0	.
BFU-E	I-EC+11.5MesP	-13.9518	608.31	100	-0.02	0.9817	0.05	-1220.83	1192.93	8.726E-7	0.000531	0	.
BFU-E	L-E only	-51.6769	1.1941	100	-43.28	<.0001	0.05	-54.0459	-49.3079	3.61E-23	4.31E-23	3.37E-24	3.85E-22
BFU-E	L-E+11.5Mesp1	5.0462	1.1941	100	4.23	<.0001	0.05	2.6772	7.4151	155.42	185.58	14.5438	1660.93
CFU-GEMM	10.5EC+!0.5Me	1.2867	0.9260	100	1.39	0.1678	0.05	-0.5505	3.1238	3.6207	3.3528	0.5766	22.7333
CFU-GEMM	11.5 EC Only	-13.9406	607.65	100	-0.02	0.9817	0.05	-1219.50	1191.62	8.824E-7	0.000536	0	.

The GLIMMIX Procedure

Colony_Typ*Condition Least Squares Means

Colony_Type	Condition	Estimate	Standard Error	DF	t Value	Pr > |t|	Alpha	Lower	Upper	Mean	Standard Error Mean	Lower Mean	Upper Mean
CFU-GEMM	11.5EC+11.5Me	1.2247	0.9256	100	1.32	0.1888	0.05	-0.6117	3.0611	3.4032	3.1500	0.5424	21.3506
CFU-GEMM	13.5EC+10.5Me	4.7844	1.1956	100	4.00	0.0001	0.05	2.4123	7.1565	119.63	143.03	11.1596	1282.43
CFU-GEMM	13.5EC+11.5Me	4.4065	1.1960	100	3.68	0.0004	0.05	2.0336	6.7794	81.9817	98.0513	7.6418	879.50
CFU-GEMM	Ao-EC Only	-51.6748	1.1958	100	-43.21	<.0001	0.05	-54.0472	-49.3024	3.61E-23	4.32E-23	3.37E-24	3.87E-22
CFU-GEMM	AoEC+10.5MesP	4.4253	1.1960	100	3.70	0.0004	0.05	2.0526	6.7981	83.5386	99.9086	7.7879	896.10
CFU-GEMM	AoEC+11.5MesP	4.5862	1.1957	100	3.84	0.0002	0.05	2.2139	6.9585	98.1211	117.33	9.1514	1052.05
CFU-GEMM	E10.5-EC Only	-51.6748	1.1958	100	-43.21	<.0001	0.05	-54.0472	-49.3024	3.61E-23	4.32E-23	3.37E-24	3.87E-22
CFU-GEMM	E10.5-MesP1+	-51.6748	1.1958	100	-43.21	<.0001	0.05	-54.0472	-49.3024	3.61E-23	4.32E-23	3.37E-24	3.87E-22
CFU-GEMM	E10.5-MesP1-	-51.6748	1.1958	100	-43.21	<.0001	0.05	-54.0472	-49.3024	3.61E-23	4.32E-23	3.37E-24	3.87E-22
CFU-GEMM	E11.5MesP1+On	-51.6748	1.1958	100	-43.21	<.0001	0.05	-54.0472	-49.3024	3.61E-23	4.32E-23	3.37E-24	3.87E-22
CFU-GEMM	E11.5MesP1-On	-51.6748	1.1958	100	-43.21	<.0001	0.05	-54.0472	-49.3024	3.61E-23	4.32E-23	3.37E-24	3.87E-22
CFU-GEMM	E13.5 EC Only	-51.6748	1.1958	100	-43.21	<.0001	0.05	-54.0472	-49.3024	3.61E-23	4.32E-23	3.37E-24	3.87E-22
CFU-GEMM	H-EC Only	-51.6748	1.1958	100	-43.21	<.0001	0.05	-54.0472	-49.3024	3.61E-23	4.32E-23	3.37E-24	3.87E-22
CFU-GEMM	H-EC+10.5MesP	4.3566	1.1961	100	3.64	0.0004	0.05	1.9836	6.7296	77.9946	93.2882	7.2692	836.84
CFU-GEMM	H-EC+11.5MesP	4.2480	1.1963	100	3.55	0.0006	0.05	1.8745	6.6216	69.9680	83.7058	6.5177	751.11
CFU-GEMM	I-EC Only	-51.6748	1.1958	100	-43.21	<.0001	0.05	-54.0472	-49.3024	3.61E-23	4.32E-23	3.37E-24	3.87E-22
CFU-GEMM	I-EC+10.5MesP	3.0676	1.1997	100	2.56	0.0121	0.05	0.6873	5.4478	21.4898	25.7820	1.9884	232.25
CFU-GEMM	I-EC+11.5MesP	2.3353	1.2067	100	1.94	0.0558	0.05	-0.05870	4.7294	10.3331	12.4688	0.9430	113.23
CFU-GEMM	L-E only	-51.6748	1.1958	100	-43.21	<.0001	0.05	-54.0472	-49.3024	3.61E-23	4.32E-23	3.37E-24	3.87E-22
CFU-GEMM	L-E+11.5Mesp1	4.5238	1.1958	100	3.78	0.0003	0.05	2.1514	6.8962	92.1885	110.24	8.5971	988.56
CFU-GM	10.5EC+!0.5Me	2.7893	0.9246	100	3.02	0.0032	0.05	0.9549	4.6238	16.2703	15.0443	2.5983	101.88
CFU-GM	11.5 EC Only	3.2354	1.1994	100	2.70	0.0082	0.05	0.8559	5.6150	25.4171	30.4851	2.3534	274.51
CFU-GM	11.5EC+11.5Me	2.8393	0.9242	100	3.07	0.0027	0.05	1.0057	4.6729	17.1036	15.8074	2.7337	107.01
CFU-GM	13.5EC+10.5Me	6.0673	1.1942	100	5.08	<.0001	0.05	3.6981	8.4365	431.52	515.31	40.3713	4612.44

The GLIMMIX Procedure

Colony_Typ*Condition Least Squares Means													
Colony_Type	Condition	Estimate	Standard Error	DF	t Value	Pr > |t|	Alpha	Lower	Upper	Mean	Standard Error Mean	Lower Mean	Upper Mean
CFU-GM	13.5EC+11.5Me	6.3708	1.1941	100	5.34	<.0001	0.05	4.0017	8.7398	584.50	697.95	54.6931	6246.61
CFU-GM	Ao-EC Only	-40.3111	1.1941	100	-33.76	<.0001	0.05	-42.6801	-37.9422	3.11E-18	3.72E-18	2.91E-19	3.33E-17
CFU-GM	AoEC+10.5MesP	6.4247	1.1941	100	5.38	<.0001	0.05	4.0557	8.7937	616.92	736.64	57.7273	6592.82
CFU-GM	AoEC+11.5MesP	6.3920	1.1941	100	5.35	<.0001	0.05	4.0230	8.7610	597.04	712.91	55.8663	6380.48
CFU-GM	E10.5-EC Only	-40.3111	1.1941	100	-33.76	<.0001	0.05	-42.6801	-37.9422	3.11E-18	3.72E-18	2.91E-19	3.33E-17
CFU-GM	E10.5-MesP1+	-40.3111	1.1941	100	-33.76	<.0001	0.05	-42.6801	-37.9422	3.11E-18	3.72E-18	2.91E-19	3.33E-17
CFU-GM	E10.5-MesP1-	-40.3111	1.1941	100	-33.76	<.0001	0.05	-42.6801	-37.9422	3.11E-18	3.72E-18	2.91E-19	3.33E-17
CFU-GM	E11.5MesP1+On	-40.3111	1.1941	100	-33.76	<.0001	0.05	-42.6801	-37.9422	3.11E-18	3.72E-18	2.91E-19	3.33E-17
CFU-GM	E11.5MesP1-On	-40.3111	1.1941	100	-33.76	<.0001	0.05	-42.6801	-37.9422	3.11E-18	3.72E-18	2.91E-19	3.33E-17
CFU-GM	E13.5 EC Only	-40.3111	1.1941	100	-33.76	<.0001	0.05	-42.6801	-37.9422	3.11E-18	3.72E-18	2.91E-19	3.33E-17
CFU-GM	H-EC Only	-40.3111	1.1941	100	-33.76	<.0001	0.05	-42.6801	-37.9422	3.11E-18	3.72E-18	2.91E-19	3.33E-17
CFU-GM	H-EC+10.5MesP	6.4064	1.1941	100	5.37	<.0001	0.05	4.0374	8.7754	605.70	723.26	56.6776	6473.05
CFU-GM	H-EC+11.5MesP	6.3101	1.1941	100	5.28	<.0001	0.05	3.9410	8.6791	550.08	656.85	51.4704	5878.88
CFU-GM	I-EC Only	-40.3111	1.1941	100	-33.76	<.0001	0.05	-42.6801	-37.9422	3.11E-18	3.72E-18	2.91E-19	3.33E-17
CFU-GM	I-EC+10.5MesP	3.8915	1.1967	100	3.25	0.0016	0.05	1.5172	6.2658	48.9845	58.6208	4.5596	526.24
CFU-GM	I-EC+11.5MesP	3.2005	1.1996	100	2.67	0.0089	0.05	0.8205	5.5805	24.5441	29.4432	2.2716	265.19
CFU-GM	L-E only	-40.3111	1.1941	100	-33.76	<.0001	0.05	-42.6801	-37.9422	3.11E-18	3.72E-18	2.91E-19	3.33E-17
CFU-GM	L-E+11.5Mesp1	6.4692	1.1941	100	5.42	<.0001	0.05	4.1002	8.8382	644.99	770.15	60.3552	6892.68

The GLIMMIX Procedure

Differences of Colony_Typ*Condition Least Squares Means											
Colony_Type	Condition	_Colony_Type	_Condition	Estimate	Standard Error	DF	t Value	Pr > |t|	Alpha	Lower	Upper
BFU-E	10.5EC+!0.5Me	BFU-E	11.5 EC Only	15.4467	608.05	100	0.03	0.9798	0.05	-1190.91	1221.80
BFU-E	10.5EC+!0.5Me	BFU-E	11.5EC+11.5Me	-0.04959	1.2882	100	-0.04	0.9694	0.05	-2.6053	2.5061
BFU-E	10.5EC+!0.5Me	BFU-E	13.5EC+10.5Me	-2.8074	1.5143	100	-1.85	0.0667	0.05	-5.8118	0.1970
BFU-E	10.5EC+!0.5Me	BFU-E	13.5EC+11.5Me	-3.4584	1.5136	100	-2.28	0.0244	0.05	-6.4614	-0.4554
BFU-E	10.5EC+!0.5Me	BFU-E	Ao-EC Only	53.1817	1.5118	100	35.18	<.0001	0.05	50.1824	56.1811
BFU-E	10.5EC+!0.5Me	BFU-E	AoEC+10.5MesP	-3.5834	1.5135	100	-2.37	0.0198	0.05	-6.5861	-0.5807
BFU-E	10.5EC+!0.5Me	BFU-E	AoEC+11.5MesP	-3.5014	1.5136	100	-2.31	0.0228	0.05	-6.5044	-0.4985
BFU-E	10.5EC+!0.5Me	BFU-E	E10.5-EC Only	53.1817	1.5118	100	35.18	<.0001	0.05	50.1824	56.1811
BFU-E	10.5EC+!0.5Me	BFU-E	E10.5-MesP1+	53.1817	1.5118	100	35.18	<.0001	0.05	50.1824	56.1811
BFU-E	10.5EC+!0.5Me	BFU-E	E10.5-MesP1-	53.1817	1.5118	100	35.18	<.0001	0.05	50.1824	56.1811
BFU-E	10.5EC+!0.5Me	BFU-E	E11.5MesP1+On	53.1817	1.5118	100	35.18	<.0001	0.05	50.1824	56.1811
BFU-E	10.5EC+!0.5Me	BFU-E	E11.5MesP1-On	53.1817	1.5118	100	35.18	<.0001	0.05	50.1824	56.1811
BFU-E	10.5EC+!0.5Me	BFU-E	E13.5 EC Only	53.1817	1.5118	100	35.18	<.0001	0.05	50.1824	56.1811
BFU-E	10.5EC+!0.5Me	BFU-E	H-EC Only	53.1817	1.5118	100	35.18	<.0001	0.05	50.1824	56.1811
BFU-E	10.5EC+!0.5Me	BFU-E	H-EC+10.5MesP	-3.4903	1.5136	100	-2.31	0.0232	0.05	-6.4932	-0.4874
BFU-E	10.5EC+!0.5Me	BFU-E	H-EC+11.5MesP	-3.3520	1.5138	100	-2.21	0.0291	0.05	-6.3552	-0.3487
BFU-E	10.5EC+!0.5Me	BFU-E	I-EC Only	53.1817	1.5118	100	35.18	<.0001	0.05	50.1824	56.1811
BFU-E	10.5EC+!0.5Me	BFU-E	I-EC+10.5MesP	15.4786	608.44	100	0.03	0.9798	0.05	-1191.66	1222.61
BFU-E	10.5EC+!0.5Me	BFU-E	I-EC+11.5MesP	15.4566	608.32	100	0.03	0.9798	0.05	-1191.42	1222.34
BFU-E	10.5EC+!0.5Me	BFU-E	L-E only	53.1817	1.5118	100	35.18	<.0001	0.05	50.1824	56.1811
BFU-E	10.5EC+!0.5Me	BFU-E	L-E+11.5Mesp1	-3.5413	1.5118	100	-2.34	0.0211	0.05	-6.5407	-0.5420
BFU-E	10.5EC+!0.5Me	CFU-GEMM	10.5EC+!0.5Me	0.2182	0.08546	100	2.55	0.0122	0.05	0.04861	0.3877
BFU-E	10.5EC+!0.5Me	CFU-GEMM	11.5 EC Only	15.4454	607.65	100	0.03	0.9798	0.05	-1190.11	1221.00
BFU-E	10.5EC+!0.5Me	CFU-GEMM	11.5EC+11.5Me	0.2801	1.2874	100	0.22	0.8282	0.05	-2.2740	2.8343

The GLIMMIX Procedure

Differences of Colony_Typ*Condition Least Squares Means											
Colony_Type	Condition	_Colony_Type	_Condition	Estimate	Standard Error	DF	t Value	Pr > |t|	Alpha	Lower	Upper
BFU-E	10.5EC+!0.5Me	CFU-GEMM	13.5EC+10.5Me	-3.2796	1.5129	100	-2.17	0.0326	0.05	-6.2812	-0.2780
BFU-E	10.5EC+!0.5Me	CFU-GEMM	13.5EC+11.5Me	-2.9017	1.5133	100	-1.92	0.0580	0.05	-5.9040	0.1007
BFU-E	10.5EC+!0.5Me	CFU-GEMM	Ao-EC Only	53.1796	1.5132	100	35.14	<.0001	0.05	50.1776	56.1817
BFU-E	10.5EC+!0.5Me	CFU-GEMM	AoEC+10.5MesP	-2.9205	1.5133	100	-1.93	0.0564	0.05	-5.9227	0.08176
BFU-E	10.5EC+!0.5Me	CFU-GEMM	AoEC+11.5MesP	-3.0814	1.5131	100	-2.04	0.0443	0.05	-6.0833	-0.07942
BFU-E	10.5EC+!0.5Me	CFU-GEMM	E10.5-EC Only	53.1796	1.5132	100	35.14	<.0001	0.05	50.1776	56.1817
BFU-E	10.5EC+!0.5Me	CFU-GEMM	E10.5-MesP1+	53.1796	1.5132	100	35.14	<.0001	0.05	50.1776	56.1817
BFU-E	10.5EC+!0.5Me	CFU-GEMM	E10.5-MesP1-	53.1796	1.5132	100	35.14	<.0001	0.05	50.1776	56.1817
BFU-E	10.5EC+!0.5Me	CFU-GEMM	E11.5MesP1+On	53.1796	1.5132	100	35.14	<.0001	0.05	50.1776	56.1817
BFU-E	10.5EC+!0.5Me	CFU-GEMM	E11.5MesP1-On	53.1796	1.5132	100	35.14	<.0001	0.05	50.1776	56.1817
BFU-E	10.5EC+!0.5Me	CFU-GEMM	E13.5 EC Only	53.1796	1.5132	100	35.14	<.0001	0.05	50.1776	56.1817
BFU-E	10.5EC+!0.5Me	CFU-GEMM	H-EC Only	53.1796	1.5132	100	35.14	<.0001	0.05	50.1776	56.1817
BFU-E	10.5EC+!0.5Me	CFU-GEMM	H-EC+10.5MesP	-2.8518	1.5134	100	-1.88	0.0624	0.05	-5.8543	0.1507
BFU-E	10.5EC+!0.5Me	CFU-GEMM	H-EC+11.5MesP	-2.7432	1.5136	100	-1.81	0.0729	0.05	-5.7462	0.2597
BFU-E	10.5EC+!0.5Me	CFU-GEMM	I-EC Only	53.1796	1.5132	100	35.14	<.0001	0.05	50.1776	56.1817
BFU-E	10.5EC+!0.5Me	CFU-GEMM	I-EC+10.5MesP	-1.5628	1.5161	100	-1.03	0.3051	0.05	-4.5706	1.4451
BFU-E	10.5EC+!0.5Me	CFU-GEMM	I-EC+11.5MesP	-0.8305	1.5220	100	-0.55	0.5865	0.05	-3.8501	2.1891
BFU-E	10.5EC+!0.5Me	CFU-GEMM	L-E only	53.1796	1.5132	100	35.14	<.0001	0.05	50.1776	56.1817
BFU-E	10.5EC+!0.5Me	CFU-GEMM	L-E+11.5Mesp1	-3.0190	1.5132	100	-2.00	0.0487	0.05	-6.0211	-0.01694
BFU-E	10.5EC+!0.5Me	CFU-GM	10.5EC+!0.5Me	-1.2845	0.06912	100	-18.58	<.0001	0.05	-1.4217	-1.1474
BFU-E	10.5EC+!0.5Me	CFU-GM	11.5 EC Only	-1.7306	1.5161	100	-1.14	0.2564	0.05	-4.7386	1.2774
BFU-E	10.5EC+!0.5Me	CFU-GM	11.5EC+11.5Me	-1.3345	1.2864	100	-1.04	0.3021	0.05	-3.8866	1.2177
BFU-E	10.5EC+!0.5Me	CFU-GM	13.5EC+10.5Me	-4.5625	1.5118	100	-3.02	0.0032	0.05	-7.5618	-1.5632
BFU-E	10.5EC+!0.5Me	CFU-GM	13.5EC+11.5Me	-4.8659	1.5118	100	-3.22	0.0017	0.05	-7.8653	-1.8666

The GLIMMIX Procedure

Differences of Colony_Typ*Condition Least Squares Means											
Colony_Type	Condition	_Colony_Type	_Condition	Estimate	Standard Error	DF	t Value	Pr > |t|	Alpha	Lower	Upper
BFU-E	10.5EC+!0.5Me	CFU-GM	Ao-EC Only	41.8160	1.5118	100	27.66	<.0001	0.05	38.8166	44.8153
BFU-E	10.5EC+!0.5Me	CFU-GM	AoEC+10.5MesP	-4.9199	1.5118	100	-3.25	0.0016	0.05	-7.9192	-1.9206
BFU-E	10.5EC+!0.5Me	CFU-GM	AoEC+11.5MesP	-4.8872	1.5118	100	-3.23	0.0017	0.05	-7.8865	-1.8878
BFU-E	10.5EC+!0.5Me	CFU-GM	E10.5-EC Only	41.8160	1.5118	100	27.66	<.0001	0.05	38.8166	44.8153
BFU-E	10.5EC+!0.5Me	CFU-GM	E10.5-MesP1+	41.8160	1.5118	100	27.66	<.0001	0.05	38.8166	44.8153
BFU-E	10.5EC+!0.5Me	CFU-GM	E10.5-MesP1-	41.8160	1.5118	100	27.66	<.0001	0.05	38.8166	44.8153
BFU-E	10.5EC+!0.5Me	CFU-GM	E11.5MesP1+On	41.8160	1.5118	100	27.66	<.0001	0.05	38.8166	44.8153
BFU-E	10.5EC+!0.5Me	CFU-GM	E11.5MesP1-On	41.8160	1.5118	100	27.66	<.0001	0.05	38.8166	44.8153
BFU-E	10.5EC+!0.5Me	CFU-GM	E13.5 EC Only	41.8160	1.5118	100	27.66	<.0001	0.05	38.8166	44.8153
BFU-E	10.5EC+!0.5Me	CFU-GM	H-EC Only	41.8160	1.5118	100	27.66	<.0001	0.05	38.8166	44.8153
BFU-E	10.5EC+!0.5Me	CFU-GM	H-EC+10.5MesP	-4.9016	1.5118	100	-3.24	0.0016	0.05	-7.9009	-1.9022
BFU-E	10.5EC+!0.5Me	CFU-GM	H-EC+11.5MesP	-4.8052	1.5118	100	-3.18	0.0020	0.05	-7.8047	-1.8058
BFU-E	10.5EC+!0.5Me	CFU-GM	I-EC Only	41.8160	1.5118	100	27.66	<.0001	0.05	38.8166	44.8153
BFU-E	10.5EC+!0.5Me	CFU-GM	I-EC+10.5MesP	-2.3867	1.5137	100	-1.58	0.1180	0.05	-5.3898	0.6165
BFU-E	10.5EC+!0.5Me	CFU-GM	I-EC+11.5MesP	-1.6957	1.5164	100	-1.12	0.2662	0.05	-4.7041	1.3128
BFU-E	10.5EC+!0.5Me	CFU-GM	L-E only	41.8160	1.5118	100	27.66	<.0001	0.05	38.8166	44.8153
BFU-E	10.5EC+!0.5Me	CFU-GM	L-E+11.5Mesp1	-4.9644	1.5118	100	-3.28	0.0014	0.05	-7.9638	-1.9650
BFU-E	11.5 EC Only	BFU-E	11.5EC+11.5Me	-15.4963	608.05	100	-0.03	0.9797	0.05	-1221.85	1190.86
BFU-E	11.5 EC Only	BFU-E	13.5EC+10.5Me	-18.2541	608.05	100	-0.03	0.9761	0.05	-1224.61	1188.10
BFU-E	11.5 EC Only	BFU-E	13.5EC+11.5Me	-18.9051	608.05	100	-0.03	0.9753	0.05	-1225.26	1187.45
BFU-E	11.5 EC Only	BFU-E	Ao-EC Only	37.7350	608.05	100	0.06	0.9506	0.05	-1168.62	1244.09
BFU-E	11.5 EC Only	BFU-E	AoEC+10.5MesP	-19.0301	608.05	100	-0.03	0.9751	0.05	-1225.38	1187.32
BFU-E	11.5 EC Only	BFU-E	AoEC+11.5MesP	-18.9481	608.05	100	-0.03	0.9752	0.05	-1225.30	1187.41
BFU-E	11.5 EC Only	BFU-E	E10.5-EC Only	37.7350	608.05	100	0.06	0.9506	0.05	-1168.62	1244.09

The GLIMMIX Procedure

Differences of Colony_Typ*Condition Least Squares Means											
Colony_Type	Condition	_Colony_Type	_Condition	Estimate	Standard Error	DF	t Value	Pr > |t|	Alpha	Lower	Upper
BFU-E	11.5 EC Only	BFU-E	E10.5-MesP1+	37.7350	608.05	100	0.06	0.9506	0.05	-1168.62	1244.09
BFU-E	11.5 EC Only	BFU-E	E10.5-MesP1-	37.7350	608.05	100	0.06	0.9506	0.05	-1168.62	1244.09
BFU-E	11.5 EC Only	BFU-E	E11.5MesP1+On	37.7350	608.05	100	0.06	0.9506	0.05	-1168.62	1244.09
BFU-E	11.5 EC Only	BFU-E	E11.5MesP1-On	37.7350	608.05	100	0.06	0.9506	0.05	-1168.62	1244.09
BFU-E	11.5 EC Only	BFU-E	E13.5 EC Only	37.7350	608.05	100	0.06	0.9506	0.05	-1168.62	1244.09
BFU-E	11.5 EC Only	BFU-E	H-EC Only	37.7350	608.05	100	0.06	0.9506	0.05	-1168.62	1244.09
BFU-E	11.5 EC Only	BFU-E	H-EC+10.5MesP	-18.9370	608.05	100	-0.03	0.9752	0.05	-1225.29	1187.42
BFU-E	11.5 EC Only	BFU-E	H-EC+11.5MesP	-18.7987	608.05	100	-0.03	0.9754	0.05	-1225.15	1187.55
BFU-E	11.5 EC Only	BFU-E	I-EC Only	37.7350	608.05	100	0.06	0.9506	0.05	-1168.62	1244.09
BFU-E	11.5 EC Only	BFU-E	I-EC+10.5MesP	0.03188	860.19	100	0.00	1.0000	0.05	-1706.56	1706.62
BFU-E	11.5 EC Only	BFU-E	I-EC+11.5MesP	0.009895	860.10	100	0.00	1.0000	0.05	-1706.40	1706.42
BFU-E	11.5 EC Only	BFU-E	L-E only	37.7350	608.05	100	0.06	0.9506	0.05	-1168.62	1244.09
BFU-E	11.5 EC Only	BFU-E	L-E+11.5Mesp1	-18.9880	608.05	100	-0.03	0.9752	0.05	-1225.34	1187.37
BFU-E	11.5 EC Only	CFU-GEMM	10.5EC+!0.5Me	-15.2285	608.05	100	-0.03	0.9801	0.05	-1221.58	1191.12
BFU-E	11.5 EC Only	CFU-GEMM	11.5 EC Only	-0.00132	859.63	100	-0.00	1.0000	0.05	-1705.47	1705.47
BFU-E	11.5 EC Only	CFU-GEMM	11.5EC+11.5Me	-15.1666	608.05	100	-0.02	0.9802	0.05	-1221.52	1191.19
BFU-E	11.5 EC Only	CFU-GEMM	13.5EC+10.5Me	-18.7263	608.05	100	-0.03	0.9755	0.05	-1225.08	1187.63
BFU-E	11.5 EC Only	CFU-GEMM	13.5EC+11.5Me	-18.3484	608.05	100	-0.03	0.9760	0.05	-1224.70	1188.00
BFU-E	11.5 EC Only	CFU-GEMM	Ao-EC Only	37.7329	608.05	100	0.06	0.9506	0.05	-1168.62	1244.09
BFU-E	11.5 EC Only	CFU-GEMM	AoEC+10.5MesP	-18.3672	608.05	100	-0.03	0.9760	0.05	-1224.72	1187.99
BFU-E	11.5 EC Only	CFU-GEMM	AoEC+11.5MesP	-18.5281	608.05	100	-0.03	0.9758	0.05	-1224.88	1187.83
BFU-E	11.5 EC Only	CFU-GEMM	E10.5-EC Only	37.7329	608.05	100	0.06	0.9506	0.05	-1168.62	1244.09
BFU-E	11.5 EC Only	CFU-GEMM	E10.5-MesP1+	37.7329	608.05	100	0.06	0.9506	0.05	-1168.62	1244.09
BFU-E	11.5 EC Only	CFU-GEMM	E10.5-MesP1-	37.7329	608.05	100	0.06	0.9506	0.05	-1168.62	1244.09

The GLIMMIX Procedure

Differences of Colony_Typ*Condition Least Squares Means											
Colony_Type	Condition	_Colony_Type	_Condition	Estimate	Standard Error	DF	t Value	Pr > |t|	Alpha	Lower	Upper
BFU-E	11.5 EC Only	CFU-GEMM	E11.5MesP1+On	37.7329	608.05	100	0.06	0.9506	0.05	-1168.62	1244.09
BFU-E	11.5 EC Only	CFU-GEMM	E11.5MesP1-On	37.7329	608.05	100	0.06	0.9506	0.05	-1168.62	1244.09
BFU-E	11.5 EC Only	CFU-GEMM	E13.5 EC Only	37.7329	608.05	100	0.06	0.9506	0.05	-1168.62	1244.09
BFU-E	11.5 EC Only	CFU-GEMM	H-EC Only	37.7329	608.05	100	0.06	0.9506	0.05	-1168.62	1244.09
BFU-E	11.5 EC Only	CFU-GEMM	H-EC+10.5MesP	-18.2985	608.05	100	-0.03	0.9761	0.05	-1224.65	1188.05
BFU-E	11.5 EC Only	CFU-GEMM	H-EC+11.5MesP	-18.1899	608.05	100	-0.03	0.9762	0.05	-1224.54	1188.16
BFU-E	11.5 EC Only	CFU-GEMM	I-EC Only	37.7329	608.05	100	0.06	0.9506	0.05	-1168.62	1244.09
BFU-E	11.5 EC Only	CFU-GEMM	I-EC+10.5MesP	-17.0095	608.05	100	-0.03	0.9777	0.05	-1223.36	1189.34
BFU-E	11.5 EC Only	CFU-GEMM	I-EC+11.5MesP	-16.2772	608.05	100	-0.03	0.9787	0.05	-1222.63	1190.08
BFU-E	11.5 EC Only	CFU-GEMM	L-E only	37.7329	608.05	100	0.06	0.9506	0.05	-1168.62	1244.09
BFU-E	11.5 EC Only	CFU-GEMM	L-E+11.5Mesp1	-18.4657	608.05	100	-0.03	0.9758	0.05	-1224.82	1187.89
BFU-E	11.5 EC Only	CFU-GM	10.5EC+!0.5Me	-16.7312	608.05	100	-0.03	0.9781	0.05	-1223.08	1189.62
BFU-E	11.5 EC Only	CFU-GM	11.5 EC Only	-17.1773	608.05	100	-0.03	0.9775	0.05	-1223.53	1189.17
BFU-E	11.5 EC Only	CFU-GM	11.5EC+11.5Me	-16.7812	608.05	100	-0.03	0.9780	0.05	-1223.13	1189.57
BFU-E	11.5 EC Only	CFU-GM	13.5EC+10.5Me	-20.0092	608.05	100	-0.03	0.9738	0.05	-1226.36	1186.34
BFU-E	11.5 EC Only	CFU-GM	13.5EC+11.5Me	-20.3126	608.05	100	-0.03	0.9734	0.05	-1226.67	1186.04
BFU-E	11.5 EC Only	CFU-GM	Ao-EC Only	26.3693	608.05	100	0.04	0.9655	0.05	-1179.98	1232.72
BFU-E	11.5 EC Only	CFU-GM	AoEC+10.5MesP	-20.3666	608.05	100	-0.03	0.9733	0.05	-1226.72	1185.99
BFU-E	11.5 EC Only	CFU-GM	AoEC+11.5MesP	-20.3339	608.05	100	-0.03	0.9734	0.05	-1226.69	1186.02
BFU-E	11.5 EC Only	CFU-GM	E10.5-EC Only	26.3693	608.05	100	0.04	0.9655	0.05	-1179.98	1232.72
BFU-E	11.5 EC Only	CFU-GM	E10.5-MesP1+	26.3693	608.05	100	0.04	0.9655	0.05	-1179.98	1232.72
BFU-E	11.5 EC Only	CFU-GM	E10.5-MesP1-	26.3693	608.05	100	0.04	0.9655	0.05	-1179.98	1232.72
BFU-E	11.5 EC Only	CFU-GM	E11.5MesP1+On	26.3693	608.05	100	0.04	0.9655	0.05	-1179.98	1232.72
BFU-E	11.5 EC Only	CFU-GM	E11.5MesP1-On	26.3693	608.05	100	0.04	0.9655	0.05	-1179.98	1232.72

The GLIMMIX Procedure

Differences of Colony_Typ*Condition Least Squares Means											
Colony_Type	Condition	_Colony_Type	_Condition	Estimate	Standard Error	DF	t Value	Pr > |t|	Alpha	Lower	Upper
BFU-E	11.5 EC Only	CFU-GM	E13.5 EC Only	26.3693	608.05	100	0.04	0.9655	0.05	-1179.98	1232.72
BFU-E	11.5 EC Only	CFU-GM	H-EC Only	26.3693	608.05	100	0.04	0.9655	0.05	-1179.98	1232.72
BFU-E	11.5 EC Only	CFU-GM	H-EC+10.5MesP	-20.3483	608.05	100	-0.03	0.9734	0.05	-1226.70	1186.00
BFU-E	11.5 EC Only	CFU-GM	H-EC+11.5MesP	-20.2519	608.05	100	-0.03	0.9735	0.05	-1226.61	1186.10
BFU-E	11.5 EC Only	CFU-GM	I-EC Only	26.3693	608.05	100	0.04	0.9655	0.05	-1179.98	1232.72
BFU-E	11.5 EC Only	CFU-GM	I-EC+10.5MesP	-17.8334	608.05	100	-0.03	0.9767	0.05	-1224.19	1188.52
BFU-E	11.5 EC Only	CFU-GM	I-EC+11.5MesP	-17.1424	608.05	100	-0.03	0.9776	0.05	-1223.50	1189.21
BFU-E	11.5 EC Only	CFU-GM	L-E only	26.3693	608.05	100	0.04	0.9655	0.05	-1179.98	1232.72
BFU-E	11.5 EC Only	CFU-GM	L-E+11.5Mesp1	-20.4111	608.05	100	-0.03	0.9733	0.05	-1226.76	1185.94
BFU-E	11.5EC+11.5Me	BFU-E	13.5EC+10.5Me	-2.7578	1.5140	100	-1.82	0.0715	0.05	-5.7616	0.2460
BFU-E	11.5EC+11.5Me	BFU-E	13.5EC+11.5Me	-3.4088	1.5133	100	-2.25	0.0265	0.05	-6.4112	-0.4065
BFU-E	11.5EC+11.5Me	BFU-E	Ao-EC Only	53.2313	1.5115	100	35.22	<.0001	0.05	50.2326	56.2301
BFU-E	11.5EC+11.5Me	BFU-E	AoEC+10.5MesP	-3.5338	1.5132	100	-2.34	0.0215	0.05	-6.5359	-0.5317
BFU-E	11.5EC+11.5Me	BFU-E	AoEC+11.5MesP	-3.4518	1.5133	100	-2.28	0.0247	0.05	-6.4542	-0.4495
BFU-E	11.5EC+11.5Me	BFU-E	E10.5-EC Only	53.2313	1.5115	100	35.22	<.0001	0.05	50.2326	56.2301
BFU-E	11.5EC+11.5Me	BFU-E	E10.5-MesP1+	53.2313	1.5115	100	35.22	<.0001	0.05	50.2326	56.2301
BFU-E	11.5EC+11.5Me	BFU-E	E10.5-MesP1-	53.2313	1.5115	100	35.22	<.0001	0.05	50.2326	56.2301
BFU-E	11.5EC+11.5Me	BFU-E	E11.5MesP1+On	53.2313	1.5115	100	35.22	<.0001	0.05	50.2326	56.2301
BFU-E	11.5EC+11.5Me	BFU-E	E11.5MesP1-On	53.2313	1.5115	100	35.22	<.0001	0.05	50.2326	56.2301
BFU-E	11.5EC+11.5Me	BFU-E	E13.5 EC Only	53.2313	1.5115	100	35.22	<.0001	0.05	50.2326	56.2301
BFU-E	11.5EC+11.5Me	BFU-E	H-EC Only	53.2313	1.5115	100	35.22	<.0001	0.05	50.2326	56.2301
BFU-E	11.5EC+11.5Me	BFU-E	H-EC+10.5MesP	-3.4407	1.5133	100	-2.27	0.0251	0.05	-6.4430	-0.4384
BFU-E	11.5EC+11.5Me	BFU-E	H-EC+11.5MesP	-3.3024	1.5135	100	-2.18	0.0315	0.05	-6.3050	-0.2997
BFU-E	11.5EC+11.5Me	BFU-E	I-EC Only	53.2313	1.5115	100	35.22	<.0001	0.05	50.2326	56.2301

The GLIMMIX Procedure

Differences of Colony_Typ*Condition Least Squares Means											
Colony_Type	Condition	_Colony_Type	_Condition	Estimate	Standard Error	DF	t Value	Pr > |t|	Alpha	Lower	Upper
BFU-E	11.5EC+11.5Me	BFU-E	I-EC+10.5MesP	15.5282	608.44	100	0.03	0.9797	0.05	-1191.61	1222.66
BFU-E	11.5EC+11.5Me	BFU-E	I-EC+11.5MesP	15.5062	608.32	100	0.03	0.9797	0.05	-1191.37	1222.39
BFU-E	11.5EC+11.5Me	BFU-E	L-E only	53.2313	1.5115	100	35.22	<.0001	0.05	50.2326	56.2301
BFU-E	11.5EC+11.5Me	BFU-E	L-E+11.5Mesp1	-3.4917	1.5115	100	-2.31	0.0229	0.05	-6.4905	-0.4930
BFU-E	11.5EC+11.5Me	CFU-GEMM	10.5EC+!0.5Me	0.2678	1.2873	100	0.21	0.8357	0.05	-2.2862	2.8218
BFU-E	11.5EC+11.5Me	CFU-GEMM	11.5 EC Only	15.4950	607.65	100	0.03	0.9797	0.05	-1190.06	1221.05
BFU-E	11.5EC+11.5Me	CFU-GEMM	11.5EC+11.5Me	0.3297	0.08486	100	3.89	0.0002	0.05	0.1614	0.4981
BFU-E	11.5EC+11.5Me	CFU-GEMM	13.5EC+10.5Me	-3.2300	1.5126	100	-2.14	0.0352	0.05	-6.2309	-0.2290
BFU-E	11.5EC+11.5Me	CFU-GEMM	13.5EC+11.5Me	-2.8521	1.5130	100	-1.89	0.0623	0.05	-5.8538	0.1497
BFU-E	11.5EC+11.5Me	CFU-GEMM	Ao-EC Only	53.2292	1.5129	100	35.18	<.0001	0.05	50.2278	56.2307
BFU-E	11.5EC+11.5Me	CFU-GEMM	AoEC+10.5MesP	-2.8709	1.5129	100	-1.90	0.0606	0.05	-5.8725	0.1307
BFU-E	11.5EC+11.5Me	CFU-GEMM	AoEC+11.5MesP	-3.0318	1.5128	100	-2.00	0.0478	0.05	-6.0331	-0.03045
BFU-E	11.5EC+11.5Me	CFU-GEMM	E10.5-EC Only	53.2292	1.5129	100	35.18	<.0001	0.05	50.2278	56.2307
BFU-E	11.5EC+11.5Me	CFU-GEMM	E10.5-MesP1+	53.2292	1.5129	100	35.18	<.0001	0.05	50.2278	56.2307
BFU-E	11.5EC+11.5Me	CFU-GEMM	E10.5-MesP1-	53.2292	1.5129	100	35.18	<.0001	0.05	50.2278	56.2307
BFU-E	11.5EC+11.5Me	CFU-GEMM	E11.5MesP1+On	53.2292	1.5129	100	35.18	<.0001	0.05	50.2278	56.2307
BFU-E	11.5EC+11.5Me	CFU-GEMM	E11.5MesP1-On	53.2292	1.5129	100	35.18	<.0001	0.05	50.2278	56.2307
BFU-E	11.5EC+11.5Me	CFU-GEMM	E13.5 EC Only	53.2292	1.5129	100	35.18	<.0001	0.05	50.2278	56.2307
BFU-E	11.5EC+11.5Me	CFU-GEMM	H-EC Only	53.2292	1.5129	100	35.18	<.0001	0.05	50.2278	56.2307
BFU-E	11.5EC+11.5Me	CFU-GEMM	H-EC+10.5MesP	-2.8022	1.5131	100	-1.85	0.0670	0.05	-5.8041	0.1996
BFU-E	11.5EC+11.5Me	CFU-GEMM	H-EC+11.5MesP	-2.6936	1.5133	100	-1.78	0.0781	0.05	-5.6960	0.3087
BFU-E	11.5EC+11.5Me	CFU-GEMM	I-EC Only	53.2292	1.5129	100	35.18	<.0001	0.05	50.2278	56.2307
BFU-E	11.5EC+11.5Me	CFU-GEMM	I-EC+10.5MesP	-1.5132	1.5158	100	-1.00	0.3206	0.05	-4.5204	1.4941
BFU-E	11.5EC+11.5Me	CFU-GEMM	I-EC+11.5MesP	-0.7809	1.5217	100	-0.51	0.6089	0.05	-3.7999	2.2381

The GLIMMIX Procedure

Differences of Colony_Typ*Condition Least Squares Means

Colony_Type	Condition	_Colony_Type	_Condition	Estimate	Standard Error	DF	t Value	Pr > |t|	Alpha	Lower	Upper
BFU-E	11.5EC+11.5Me	CFU-GEMM	L-E only	53.2292	1.5129	100	35.18	<.0001	0.05	50.2278	56.2307
BFU-E	11.5EC+11.5Me	CFU-GEMM	L-E+11.5Mesp1	-2.9694	1.5129	100	-1.96	0.0524	0.05	-5.9709	0.03203
BFU-E	11.5EC+11.5Me	CFU-GM	10.5EC+!0.5Me	-1.2349	1.2863	100	-0.96	0.3394	0.05	-3.7870	1.3171
BFU-E	11.5EC+11.5Me	CFU-GM	11.5 EC Only	-1.6810	1.5158	100	-1.11	0.2701	0.05	-4.6884	1.3263
BFU-E	11.5EC+11.5Me	CFU-GM	11.5EC+11.5Me	-1.2849	0.06803	100	-18.89	<.0001	0.05	-1.4198	-1.1499
BFU-E	11.5EC+11.5Me	CFU-GM	13.5EC+10.5Me	-4.5129	1.5114	100	-2.99	0.0036	0.05	-7.5116	-1.5142
BFU-E	11.5EC+11.5Me	CFU-GM	13.5EC+11.5Me	-4.8164	1.5115	100	-3.19	0.0019	0.05	-7.8151	-1.8176
BFU-E	11.5EC+11.5Me	CFU-GM	Ao-EC Only	41.8655	1.5115	100	27.70	<.0001	0.05	38.8668	44.8643
BFU-E	11.5EC+11.5Me	CFU-GM	AoEC+10.5MesP	-4.8703	1.5114	100	-3.22	0.0017	0.05	-7.8690	-1.8716
BFU-E	11.5EC+11.5Me	CFU-GM	AoEC+11.5MesP	-4.8376	1.5115	100	-3.20	0.0018	0.05	-7.8363	-1.8388
BFU-E	11.5EC+11.5Me	CFU-GM	E10.5-EC Only	41.8655	1.5115	100	27.70	<.0001	0.05	38.8668	44.8643
BFU-E	11.5EC+11.5Me	CFU-GM	E10.5-MesP1+	41.8655	1.5115	100	27.70	<.0001	0.05	38.8668	44.8643
BFU-E	11.5EC+11.5Me	CFU-GM	E10.5-MesP1-	41.8655	1.5115	100	27.70	<.0001	0.05	38.8668	44.8643
BFU-E	11.5EC+11.5Me	CFU-GM	E11.5MesP1+On	41.8655	1.5115	100	27.70	<.0001	0.05	38.8668	44.8643
BFU-E	11.5EC+11.5Me	CFU-GM	E11.5MesP1-On	41.8655	1.5115	100	27.70	<.0001	0.05	38.8668	44.8643
BFU-E	11.5EC+11.5Me	CFU-GM	E13.5 EC Only	41.8655	1.5115	100	27.70	<.0001	0.05	38.8668	44.8643
BFU-E	11.5EC+11.5Me	CFU-GM	H-EC Only	41.8655	1.5115	100	27.70	<.0001	0.05	38.8668	44.8643
BFU-E	11.5EC+11.5Me	CFU-GM	H-EC+10.5MesP	-4.8520	1.5115	100	-3.21	0.0018	0.05	-7.8507	-1.8533
BFU-E	11.5EC+11.5Me	CFU-GM	H-EC+11.5MesP	-4.7557	1.5115	100	-3.15	0.0022	0.05	-7.7545	-1.7568
BFU-E	11.5EC+11.5Me	CFU-GM	I-EC Only	41.8655	1.5115	100	27.70	<.0001	0.05	38.8668	44.8643
BFU-E	11.5EC+11.5Me	CFU-GM	I-EC+10.5MesP	-2.3371	1.5134	100	-1.54	0.1257	0.05	-5.3396	0.6654
BFU-E	11.5EC+11.5Me	CFU-GM	I-EC+11.5MesP	-1.6461	1.5161	100	-1.09	0.2802	0.05	-4.6539	1.3618
BFU-E	11.5EC+11.5Me	CFU-GM	L-E only	41.8655	1.5115	100	27.70	<.0001	0.05	38.8668	44.8643
BFU-E	11.5EC+11.5Me	CFU-GM	L-E+11.5Mesp1	-4.9148	1.5115	100	-3.25	0.0016	0.05	-7.9136	-1.9161

The GLIMMIX Procedure

Differences of Colony_Typ*Condition Least Squares Means											
Colony_Type	Condition	_Colony_Type	_Condition	Estimate	Standard Error	DF	t Value	Pr > |t|	Alpha	Lower	Upper
BFU-E	13.5EC+10.5Me	BFU-E	13.5EC+11.5Me	-0.6510	1.6927	100	-0.38	0.7014	0.05	-4.0093	2.7073
BFU-E	13.5EC+10.5Me	BFU-E	Ao-EC Only	55.9891	1.6910	100	33.11	<.0001	0.05	52.6341	59.3441
BFU-E	13.5EC+10.5Me	BFU-E	AoEC+10.5MesP	-0.7760	1.6926	100	-0.46	0.6476	0.05	-4.1341	2.5821
BFU-E	13.5EC+10.5Me	BFU-E	AoEC+11.5MesP	-0.6940	1.6927	100	-0.41	0.6827	0.05	-4.0522	2.6642
BFU-E	13.5EC+10.5Me	BFU-E	E10.5-EC Only	55.9891	1.6910	100	33.11	<.0001	0.05	52.6341	59.3441
BFU-E	13.5EC+10.5Me	BFU-E	E10.5-MesP1+	55.9891	1.6910	100	33.11	<.0001	0.05	52.6341	59.3441
BFU-E	13.5EC+10.5Me	BFU-E	E10.5-MesP1-	55.9891	1.6910	100	33.11	<.0001	0.05	52.6341	59.3441
BFU-E	13.5EC+10.5Me	BFU-E	E11.5MesP1+On	55.9891	1.6910	100	33.11	<.0001	0.05	52.6341	59.3441
BFU-E	13.5EC+10.5Me	BFU-E	E11.5MesP1-On	55.9891	1.6910	100	33.11	<.0001	0.05	52.6341	59.3441
BFU-E	13.5EC+10.5Me	BFU-E	E13.5 EC Only	55.9891	1.6910	100	33.11	<.0001	0.05	52.6341	59.3441
BFU-E	13.5EC+10.5Me	BFU-E	H-EC Only	55.9891	1.6910	100	33.11	<.0001	0.05	52.6341	59.3441
BFU-E	13.5EC+10.5Me	BFU-E	H-EC+10.5MesP	-0.6829	1.6927	100	-0.40	0.6875	0.05	-4.0411	2.6753
BFU-E	13.5EC+10.5Me	BFU-E	H-EC+11.5MesP	-0.5446	1.6928	100	-0.32	0.7484	0.05	-3.9030	2.8139
BFU-E	13.5EC+10.5Me	BFU-E	I-EC Only	55.9891	1.6910	100	33.11	<.0001	0.05	52.6341	59.3441
BFU-E	13.5EC+10.5Me	BFU-E	I-EC+10.5MesP	18.2860	608.44	100	0.03	0.9761	0.05	-1188.85	1225.42
BFU-E	13.5EC+10.5Me	BFU-E	I-EC+11.5MesP	18.2640	608.32	100	0.03	0.9761	0.05	-1188.62	1225.15
BFU-E	13.5EC+10.5Me	BFU-E	L-E only	55.9891	1.6910	100	33.11	<.0001	0.05	52.6341	59.3441
BFU-E	13.5EC+10.5Me	BFU-E	L-E+11.5Mesp1	-0.7339	1.6910	100	-0.43	0.6652	0.05	-4.0889	2.6211
BFU-E	13.5EC+10.5Me	CFU-GEMM	10.5EC+!0.5Me	3.0256	1.5136	100	2.00	0.0483	0.05	0.02263	6.0285
BFU-E	13.5EC+10.5Me	CFU-GEMM	11.5 EC Only	18.2528	607.65	100	0.03	0.9761	0.05	-1187.31	1223.81
BFU-E	13.5EC+10.5Me	CFU-GEMM	11.5EC+11.5Me	3.0875	1.5133	100	2.04	0.0440	0.05	0.08507	6.0899
BFU-E	13.5EC+10.5Me	CFU-GEMM	13.5EC+10.5Me	-0.4722	0.1065	100	-4.44	<.0001	0.05	-0.6834	-0.2610
BFU-E	13.5EC+10.5Me	CFU-GEMM	13.5EC+11.5Me	-0.09428	1.6924	100	-0.06	0.9557	0.05	-3.4520	3.2634
BFU-E	13.5EC+10.5Me	CFU-GEMM	Ao-EC Only	55.9870	1.6923	100	33.08	<.0001	0.05	52.6296	59.3445

The SAS System

The GLIMMIX Procedure

Differences of Colony_Typ*Condition Least Squares Means											
Colony_Type	Condition	_Colony_Type	_Condition	Estimate	Standard Error	DF	t Value	Pr > |t|	Alpha	Lower	Upper
BFU-E	13.5EC+10.5Me	CFU-GEMM	AoEC+10.5MesP	-0.1131	1.6924	100	-0.07	0.9469	0.05	-3.4707	3.2446
BFU-E	13.5EC+10.5Me	CFU-GEMM	AoEC+11.5MesP	-0.2740	1.6922	100	-0.16	0.8717	0.05	-3.6313	3.0833
BFU-E	13.5EC+10.5Me	CFU-GEMM	E10.5-EC Only	55.9870	1.6923	100	33.08	<.0001	0.05	52.6296	59.3445
BFU-E	13.5EC+10.5Me	CFU-GEMM	E10.5-MesP1+	55.9870	1.6923	100	33.08	<.0001	0.05	52.6296	59.3445
BFU-E	13.5EC+10.5Me	CFU-GEMM	E10.5-MesP1-	55.9870	1.6923	100	33.08	<.0001	0.05	52.6296	59.3445
BFU-E	13.5EC+10.5Me	CFU-GEMM	E11.5MesP1+On	55.9870	1.6923	100	33.08	<.0001	0.05	52.6296	59.3445
BFU-E	13.5EC+10.5Me	CFU-GEMM	E11.5MesP1-On	55.9870	1.6923	100	33.08	<.0001	0.05	52.6296	59.3445
BFU-E	13.5EC+10.5Me	CFU-GEMM	E13.5 EC Only	55.9870	1.6923	100	33.08	<.0001	0.05	52.6296	59.3445
BFU-E	13.5EC+10.5Me	CFU-GEMM	H-EC Only	55.9870	1.6923	100	33.08	<.0001	0.05	52.6296	59.3445
BFU-E	13.5EC+10.5Me	CFU-GEMM	H-EC+10.5MesP	-0.04442	1.6925	100	-0.03	0.9791	0.05	-3.4023	3.3134
BFU-E	13.5EC+10.5Me	CFU-GEMM	H-EC+11.5MesP	0.06418	1.6927	100	0.04	0.9698	0.05	-3.2940	3.4224
BFU-E	13.5EC+10.5Me	CFU-GEMM	I-EC Only	55.9870	1.6923	100	33.08	<.0001	0.05	52.6296	59.3445
BFU-E	13.5EC+10.5Me	CFU-GEMM	I-EC+10.5MesP	1.2446	1.6951	100	0.73	0.4645	0.05	-2.1183	4.6076
BFU-E	13.5EC+10.5Me	CFU-GEMM	I-EC+11.5MesP	1.9769	1.7000	100	1.16	0.2477	0.05	-1.3959	5.3496
BFU-E	13.5EC+10.5Me	CFU-GEMM	L-E only	55.9870	1.6923	100	33.08	<.0001	0.05	52.6296	59.3445
BFU-E	13.5EC+10.5Me	CFU-GEMM	L-E+11.5Mesp1	-0.2116	1.6923	100	-0.13	0.9007	0.05	-3.5690	3.1458
BFU-E	13.5EC+10.5Me	CFU-GM	10.5EC+!0.5Me	1.5229	1.5128	100	1.01	0.3165	0.05	-1.4784	4.5241
BFU-E	13.5EC+10.5Me	CFU-GM	11.5 EC Only	1.0768	1.6948	100	0.64	0.5267	0.05	-2.2857	4.4393
BFU-E	13.5EC+10.5Me	CFU-GM	11.5EC+11.5Me	1.4729	1.5125	100	0.97	0.3325	0.05	-1.5278	4.4737
BFU-E	13.5EC+10.5Me	CFU-GM	13.5EC+10.5Me	-1.7551	0.08845	100	-19.84	<.0001	0.05	-1.9306	-1.5796
BFU-E	13.5EC+10.5Me	CFU-GM	13.5EC+11.5Me	-2.0586	1.6911	100	-1.22	0.2264	0.05	-5.4136	1.2965
BFU-E	13.5EC+10.5Me	CFU-GM	Ao-EC Only	44.6234	1.6910	100	26.39	<.0001	0.05	41.2684	47.9783
BFU-E	13.5EC+10.5Me	CFU-GM	AoEC+10.5MesP	-2.1125	1.6911	100	-1.25	0.2145	0.05	-5.4675	1.2425
BFU-E	13.5EC+10.5Me	CFU-GM	AoEC+11.5MesP	-2.0798	1.6911	100	-1.23	0.2216	0.05	-5.4348	1.2753

The GLIMMIX Procedure

Differences of Colony_Typ*Condition Least Squares Means											
Colony_Type	Condition	_Colony_Type	_Condition	Estimate	Standard Error	DF	t Value	Pr > |t|	Alpha	Lower	Upper
BFU-E	13.5EC+10.5Me	CFU-GM	E10.5-EC Only	44.6234	1.6910	100	26.39	<.0001	0.05	41.2684	47.9783
BFU-E	13.5EC+10.5Me	CFU-GM	E10.5-MesP1+	44.6234	1.6910	100	26.39	<.0001	0.05	41.2684	47.9783
BFU-E	13.5EC+10.5Me	CFU-GM	E10.5-MesP1-	44.6234	1.6910	100	26.39	<.0001	0.05	41.2684	47.9783
BFU-E	13.5EC+10.5Me	CFU-GM	E11.5MesP1+On	44.6234	1.6910	100	26.39	<.0001	0.05	41.2684	47.9783
BFU-E	13.5EC+10.5Me	CFU-GM	E11.5MesP1-On	44.6234	1.6910	100	26.39	<.0001	0.05	41.2684	47.9783
BFU-E	13.5EC+10.5Me	CFU-GM	E13.5 EC Only	44.6234	1.6910	100	26.39	<.0001	0.05	41.2684	47.9783
BFU-E	13.5EC+10.5Me	CFU-GM	H-EC Only	44.6234	1.6910	100	26.39	<.0001	0.05	41.2684	47.9783
BFU-E	13.5EC+10.5Me	CFU-GM	H-EC+10.5MesP	-2.0942	1.6911	100	-1.24	0.2185	0.05	-5.4492	1.2608
BFU-E	13.5EC+10.5Me	CFU-GM	H-EC+11.5MesP	-1.9978	1.6911	100	-1.18	0.2402	0.05	-5.3529	1.3572
BFU-E	13.5EC+10.5Me	CFU-GM	I-EC Only	44.6234	1.6910	100	26.39	<.0001	0.05	41.2684	47.9783
BFU-E	13.5EC+10.5Me	CFU-GM	I-EC+10.5MesP	0.4207	1.6929	100	0.25	0.8042	0.05	-2.9380	3.7794
BFU-E	13.5EC+10.5Me	CFU-GM	I-EC+11.5MesP	1.1117	1.6950	100	0.66	0.5134	0.05	-2.2510	4.4745
BFU-E	13.5EC+10.5Me	CFU-GM	L-E only	44.6234	1.6910	100	26.39	<.0001	0.05	41.2684	47.9783
BFU-E	13.5EC+10.5Me	CFU-GM	L-E+11.5Mesp1	-2.1570	1.6910	100	-1.28	0.2051	0.05	-5.5120	1.1980
BFU-E	13.5EC+11.5Me	BFU-E	Ao-EC Only	56.6401	1.6903	100	33.51	<.0001	0.05	53.2866	59.9937
BFU-E	13.5EC+11.5Me	BFU-E	AoEC+10.5MesP	-0.1250	1.6919	100	-0.07	0.9413	0.05	-3.4816	3.2316
BFU-E	13.5EC+11.5Me	BFU-E	AoEC+11.5MesP	-0.04302	1.6919	100	-0.03	0.9798	0.05	-3.3998	3.3137
BFU-E	13.5EC+11.5Me	BFU-E	E10.5-EC Only	56.6401	1.6903	100	33.51	<.0001	0.05	53.2866	59.9937
BFU-E	13.5EC+11.5Me	BFU-E	E10.5-MesP1+	56.6401	1.6903	100	33.51	<.0001	0.05	53.2866	59.9937
BFU-E	13.5EC+11.5Me	BFU-E	E10.5-MesP1-	56.6401	1.6903	100	33.51	<.0001	0.05	53.2866	59.9937
BFU-E	13.5EC+11.5Me	BFU-E	E11.5MesP1+On	56.6401	1.6903	100	33.51	<.0001	0.05	53.2866	59.9937
BFU-E	13.5EC+11.5Me	BFU-E	E11.5MesP1-On	56.6401	1.6903	100	33.51	<.0001	0.05	53.2866	59.9937
BFU-E	13.5EC+11.5Me	BFU-E	E13.5 EC Only	56.6401	1.6903	100	33.51	<.0001	0.05	53.2866	59.9937
BFU-E	13.5EC+11.5Me	BFU-E	H-EC Only	56.6401	1.6903	100	33.51	<.0001	0.05	53.2866	59.9937

The GLIMMIX Procedure

Differences of Colony_Typ*Condition Least Squares Means											
Colony_Type	Condition	_Colony_Type	_Condition	Estimate	Standard Error	DF	t Value	Pr > |t|	Alpha	Lower	Upper
BFU-E	13.5EC+11.5Me	BFU-E	H-EC+10.5MesP	-0.03191	1.6919	100	-0.02	0.9850	0.05	-3.3887	3.3248
BFU-E	13.5EC+11.5Me	BFU-E	H-EC+11.5MesP	0.1064	1.6921	100	0.06	0.9500	0.05	-3.2506	3.4635
BFU-E	13.5EC+11.5Me	BFU-E	I-EC Only	56.6401	1.6903	100	33.51	<.0001	0.05	53.2866	59.9937
BFU-E	13.5EC+11.5Me	BFU-E	I-EC+10.5MesP	18.9370	608.44	100	0.03	0.9752	0.05	-1188.20	1226.07
BFU-E	13.5EC+11.5Me	BFU-E	I-EC+11.5MesP	18.9150	608.32	100	0.03	0.9753	0.05	-1187.97	1225.80
BFU-E	13.5EC+11.5Me	BFU-E	L-E only	56.6401	1.6903	100	33.51	<.0001	0.05	53.2866	59.9937
BFU-E	13.5EC+11.5Me	BFU-E	L-E+11.5Mesp1	-0.08293	1.6903	100	-0.05	0.9610	0.05	-3.4365	3.2706
BFU-E	13.5EC+11.5Me	CFU-GEMM	10.5EC+!0.5Me	3.6766	1.5129	100	2.43	0.0169	0.05	0.6751	6.6781
BFU-E	13.5EC+11.5Me	CFU-GEMM	11.5 EC Only	18.9038	607.65	100	0.03	0.9752	0.05	-1186.66	1224.46
BFU-E	13.5EC+11.5Me	CFU-GEMM	11.5EC+11.5Me	3.7385	1.5126	100	2.47	0.0151	0.05	0.7375	6.7395
BFU-E	13.5EC+11.5Me	CFU-GEMM	13.5EC+10.5Me	0.1788	1.6914	100	0.11	0.9160	0.05	-3.1769	3.5346
BFU-E	13.5EC+11.5Me	CFU-GEMM	13.5EC+11.5Me	0.5567	0.1008	100	5.52	<.0001	0.05	0.3567	0.7567
BFU-E	13.5EC+11.5Me	CFU-GEMM	Ao-EC Only	56.6380	1.6915	100	33.48	<.0001	0.05	53.2821	59.9940
BFU-E	13.5EC+11.5Me	CFU-GEMM	AoEC+10.5MesP	0.5379	1.6917	100	0.32	0.7512	0.05	-2.8183	3.8941
BFU-E	13.5EC+11.5Me	CFU-GEMM	AoEC+11.5MesP	0.3770	1.6915	100	0.22	0.8241	0.05	-2.9789	3.7329
BFU-E	13.5EC+11.5Me	CFU-GEMM	E10.5-EC Only	56.6380	1.6915	100	33.48	<.0001	0.05	53.2821	59.9940
BFU-E	13.5EC+11.5Me	CFU-GEMM	E10.5-MesP1+	56.6380	1.6915	100	33.48	<.0001	0.05	53.2821	59.9940
BFU-E	13.5EC+11.5Me	CFU-GEMM	E10.5-MesP1-	56.6380	1.6915	100	33.48	<.0001	0.05	53.2821	59.9940
BFU-E	13.5EC+11.5Me	CFU-GEMM	E11.5MesP1+On	56.6380	1.6915	100	33.48	<.0001	0.05	53.2821	59.9940
BFU-E	13.5EC+11.5Me	CFU-GEMM	E11.5MesP1-On	56.6380	1.6915	100	33.48	<.0001	0.05	53.2821	59.9940
BFU-E	13.5EC+11.5Me	CFU-GEMM	E13.5 EC Only	56.6380	1.6915	100	33.48	<.0001	0.05	53.2821	59.9940
BFU-E	13.5EC+11.5Me	CFU-GEMM	H-EC Only	56.6380	1.6915	100	33.48	<.0001	0.05	53.2821	59.9940
BFU-E	13.5EC+11.5Me	CFU-GEMM	H-EC+10.5MesP	0.6066	1.6917	100	0.36	0.7207	0.05	-2.7498	3.9630
BFU-E	13.5EC+11.5Me	CFU-GEMM	H-EC+11.5MesP	0.7152	1.6919	100	0.42	0.6734	0.05	-2.6416	4.0719

The GLIMMIX Procedure

Differences of Colony_Typ*Condition Least Squares Means											
Colony_Type	Condition	_Colony_Type	_Condition	Estimate	Standard Error	DF	t Value	Pr > |t|	Alpha	Lower	Upper
BFU-E	13.5EC+11.5Me	CFU-GEMM	I-EC Only	56.6380	1.6915	100	33.48	<.0001	0.05	53.2821	59.9940
BFU-E	13.5EC+11.5Me	CFU-GEMM	I-EC+10.5MesP	1.8956	1.6943	100	1.12	0.2659	0.05	-1.4659	5.2571
BFU-E	13.5EC+11.5Me	CFU-GEMM	I-EC+11.5MesP	2.6279	1.6993	100	1.55	0.1251	0.05	-0.7434	5.9992
BFU-E	13.5EC+11.5Me	CFU-GEMM	L-E only	56.6380	1.6915	100	33.48	<.0001	0.05	53.2821	59.9940
BFU-E	13.5EC+11.5Me	CFU-GEMM	L-E+11.5Mesp1	0.4394	1.6915	100	0.26	0.7956	0.05	-2.9166	3.7953
BFU-E	13.5EC+11.5Me	CFU-GM	10.5EC+!0.5Me	2.1739	1.5120	100	1.44	0.1536	0.05	-0.8260	5.1737
BFU-E	13.5EC+11.5Me	CFU-GM	11.5 EC Only	1.7278	1.6941	100	1.02	0.3102	0.05	-1.6332	5.0888
BFU-E	13.5EC+11.5Me	CFU-GM	11.5EC+11.5Me	2.1239	1.5118	100	1.40	0.1631	0.05	-0.8754	5.1233
BFU-E	13.5EC+11.5Me	CFU-GM	13.5EC+10.5Me	-1.1041	1.6904	100	-0.65	0.5152	0.05	-4.4578	2.2496
BFU-E	13.5EC+11.5Me	CFU-GM	13.5EC+11.5Me	-1.4075	0.07447	100	-18.90	<.0001	0.05	-1.5553	-1.2598
BFU-E	13.5EC+11.5Me	CFU-GM	Ao-EC Only	45.2744	1.6903	100	26.78	<.0001	0.05	41.9208	48.6279
BFU-E	13.5EC+11.5Me	CFU-GM	AoEC+10.5MesP	-1.4615	1.6903	100	-0.86	0.3893	0.05	-4.8151	1.8920
BFU-E	13.5EC+11.5Me	CFU-GM	AoEC+11.5MesP	-1.4288	1.6903	100	-0.85	0.4000	0.05	-4.7823	1.9248
BFU-E	13.5EC+11.5Me	CFU-GM	E10.5-EC Only	45.2744	1.6903	100	26.78	<.0001	0.05	41.9208	48.6279
BFU-E	13.5EC+11.5Me	CFU-GM	E10.5-MesP1+	45.2744	1.6903	100	26.78	<.0001	0.05	41.9208	48.6279
BFU-E	13.5EC+11.5Me	CFU-GM	E10.5-MesP1-	45.2744	1.6903	100	26.78	<.0001	0.05	41.9208	48.6279
BFU-E	13.5EC+11.5Me	CFU-GM	E11.5MesP1+On	45.2744	1.6903	100	26.78	<.0001	0.05	41.9208	48.6279
BFU-E	13.5EC+11.5Me	CFU-GM	E11.5MesP1-On	45.2744	1.6903	100	26.78	<.0001	0.05	41.9208	48.6279
BFU-E	13.5EC+11.5Me	CFU-GM	E13.5 EC Only	45.2744	1.6903	100	26.78	<.0001	0.05	41.9208	48.6279
BFU-E	13.5EC+11.5Me	CFU-GM	H-EC Only	45.2744	1.6903	100	26.78	<.0001	0.05	41.9208	48.6279
BFU-E	13.5EC+11.5Me	CFU-GM	H-EC+10.5MesP	-1.4432	1.6903	100	-0.85	0.3953	0.05	-4.7967	1.9104
BFU-E	13.5EC+11.5Me	CFU-GM	H-EC+11.5MesP	-1.3468	1.6903	100	-0.80	0.4275	0.05	-4.7004	2.0067
BFU-E	13.5EC+11.5Me	CFU-GM	I-EC Only	45.2744	1.6903	100	26.78	<.0001	0.05	41.9208	48.6279
BFU-E	13.5EC+11.5Me	CFU-GM	I-EC+10.5MesP	1.0717	1.6922	100	0.63	0.5280	0.05	-2.2855	4.4290

The GLIMMIX Procedure

Differences of Colony_Typ*Condition Least Squares Means											
Colony_Type	Condition	_Colony_Type	_Condition	Estimate	Standard Error	DF	t Value	Pr > |t|	Alpha	Lower	Upper
BFU-E	13.5EC+11.5Me	CFU-GM	I-EC+11.5MesP	1.7628	1.6942	100	1.04	0.3006	0.05	-1.5986	5.1241
BFU-E	13.5EC+11.5Me	CFU-GM	L-E only	45.2744	1.6903	100	26.78	<.0001	0.05	41.9208	48.6279
BFU-E	13.5EC+11.5Me	CFU-GM	L-E+11.5Mesp1	-1.5060	1.6903	100	-0.89	0.3751	0.05	-4.8595	1.8475
BFU-E	Ao-EC Only	BFU-E	AoEC+10.5MesP	-56.7651	1.6902	100	-33.58	<.0001	0.05	-60.1185	-53.4118
BFU-E	Ao-EC Only	BFU-E	AoEC+11.5MesP	-56.6832	1.6903	100	-33.53	<.0001	0.05	-60.0366	-53.3297
BFU-E	Ao-EC Only	BFU-E	E10.5-EC Only	1.6E-14	.	.	.	.	.	.	.
BFU-E	Ao-EC Only	BFU-E	E10.5-MesP1+	5.65E-13	.	.	.	.	.	.	.
BFU-E	Ao-EC Only	BFU-E	E10.5-MesP1-	4.17E-13	.	.	.	.	.	.	.
BFU-E	Ao-EC Only	BFU-E	E11.5MesP1+On	4.97E-13	.	.	.	.	.	.	.
BFU-E	Ao-EC Only	BFU-E	E11.5MesP1-On	2.47E-13	.	.	.	.	.	.	.
BFU-E	Ao-EC Only	BFU-E	E13.5 EC Only	1.07E-13	.	.	.	.	.	.	.
BFU-E	Ao-EC Only	BFU-E	H-EC Only	1.6E-14	.	.	.	.	.	.	.
BFU-E	Ao-EC Only	BFU-E	H-EC+10.5MesP	-56.6721	1.6903	100	-33.53	<.0001	0.05	-60.0255	-53.3186
BFU-E	Ao-EC Only	BFU-E	H-EC+11.5MesP	-56.5337	1.6904	100	-33.44	<.0001	0.05	-59.8874	-53.1800
BFU-E	Ao-EC Only	BFU-E	I-EC Only	3.13E-13	.	.	.	.	.	.	.
BFU-E	Ao-EC Only	BFU-E	I-EC+10.5MesP	-37.7032	608.44	100	-0.06	0.9507	0.05	-1244.84	1169.43
BFU-E	Ao-EC Only	BFU-E	I-EC+11.5MesP	-37.7251	608.32	100	-0.06	0.9507	0.05	-1244.61	1169.16
BFU-E	Ao-EC Only	BFU-E	L-E only	5.35E-13	.	.	.	.	.	.	.
BFU-E	Ao-EC Only	BFU-E	L-E+11.5Mesp1	-56.7231	0	100	-Infty	<.0001	.	.	.
BFU-E	Ao-EC Only	CFU-GEMM	10.5EC+!0.5Me	-52.9636	1.5111	100	-35.05	<.0001	0.05	-55.9615	-49.9657
BFU-E	Ao-EC Only	CFU-GEMM	11.5 EC Only	-37.7364	607.65	100	-0.06	0.9506	0.05	-1243.30	1167.82
BFU-E	Ao-EC Only	CFU-GEMM	11.5EC+11.5Me	-52.9016	1.5108	100	-35.02	<.0001	0.05	-55.8990	-49.9042
BFU-E	Ao-EC Only	CFU-GEMM	13.5EC+10.5Me	-56.4613	1.6898	100	-33.41	<.0001	0.05	-59.8138	-53.1089
BFU-E	Ao-EC Only	CFU-GEMM	13.5EC+11.5Me	-56.0834	1.6900	100	-33.18	<.0001	0.05	-59.4364	-52.7304

The GLIMMIX Procedure

Differences of Colony_Typ*Condition Least Squares Means											
Colony_Type	Condition	_Colony_Type	_Condition	Estimate	Standard Error	DF	t Value	Pr > |t|	Alpha	Lower	Upper
BFU-E	Ao-EC Only	CFU-GEMM	Ao-EC Only	-0.00210	0.06423	100	-0.03	0.9740	0.05	-0.1295	0.1253
BFU-E	Ao-EC Only	CFU-GEMM	AoEC+10.5MesP	-56.1022	1.6900	100	-33.20	<.0001	0.05	-59.4551	-52.7493
BFU-E	Ao-EC Only	CFU-GEMM	AoEC+11.5MesP	-56.2631	1.6898	100	-33.29	<.0001	0.05	-59.6157	-52.9105
BFU-E	Ao-EC Only	CFU-GEMM	E10.5-EC Only	-0.00210	0.06423	100	-0.03	0.9740	0.05	-0.1295	0.1253
BFU-E	Ao-EC Only	CFU-GEMM	E10.5-MesP1+	-0.00210	0.06423	100	-0.03	0.9740	0.05	-0.1295	0.1253
BFU-E	Ao-EC Only	CFU-GEMM	E10.5-MesP1-	-0.00210	0.06423	100	-0.03	0.9740	0.05	-0.1295	0.1253
BFU-E	Ao-EC Only	CFU-GEMM	E11.5MesP1+On	-0.00210	0.06423	100	-0.03	0.9740	0.05	-0.1295	0.1253
BFU-E	Ao-EC Only	CFU-GEMM	E11.5MesP1-On	-0.00210	0.06423	100	-0.03	0.9740	0.05	-0.1295	0.1253
BFU-E	Ao-EC Only	CFU-GEMM	E13.5 EC Only	-0.00210	0.06423	100	-0.03	0.9740	0.05	-0.1295	0.1253
BFU-E	Ao-EC Only	CFU-GEMM	H-EC Only	-0.00210	0.06423	100	-0.03	0.9740	0.05	-0.1295	0.1253
BFU-E	Ao-EC Only	CFU-GEMM	H-EC+10.5MesP	-56.0336	1.6901	100	-33.15	<.0001	0.05	-59.3867	-52.6805
BFU-E	Ao-EC Only	CFU-GEMM	H-EC+11.5MesP	-55.9250	1.6903	100	-33.09	<.0001	0.05	-59.2784	-52.5715
BFU-E	Ao-EC Only	CFU-GEMM	I-EC Only	-0.00210	0.06423	100	-0.03	0.9740	0.05	-0.1295	0.1253
BFU-E	Ao-EC Only	CFU-GEMM	I-EC+10.5MesP	-54.7445	1.6927	100	-32.34	<.0001	0.05	-58.1027	-51.3863
BFU-E	Ao-EC Only	CFU-GEMM	I-EC+11.5MesP	-54.0123	1.6976	100	-31.82	<.0001	0.05	-57.3803	-50.6443
BFU-E	Ao-EC Only	CFU-GEMM	L-E only	-0.00210	0.06423	100	-0.03	0.9740	0.05	-0.1295	0.1253
BFU-E	Ao-EC Only	CFU-GEMM	L-E+11.5Mesp1	-56.2008	0.06423	100	-874.93	<.0001	0.05	-56.3282	-56.0733
BFU-E	Ao-EC Only	CFU-GM	10.5EC+!0.5Me	-54.4663	1.5102	100	-36.07	<.0001	0.05	-57.4625	-51.4700
BFU-E	Ao-EC Only	CFU-GM	11.5 EC Only	-54.9123	1.6924	100	-32.45	<.0001	0.05	-58.2701	-51.5546
BFU-E	Ao-EC Only	CFU-GM	11.5EC+11.5Me	-54.5162	1.5100	100	-36.10	<.0001	0.05	-57.5119	-51.5205
BFU-E	Ao-EC Only	CFU-GM	13.5EC+10.5Me	-57.7442	1.6887	100	-34.19	<.0001	0.05	-61.0946	-54.3938
BFU-E	Ao-EC Only	CFU-GM	13.5EC+11.5Me	-58.0477	1.6887	100	-34.37	<.0001	0.05	-61.3980	-54.6974
BFU-E	Ao-EC Only	CFU-GM	Ao-EC Only	-11.3658	0	100	-Infty	<.0001	.	.	.
BFU-E	Ao-EC Only	CFU-GM	AoEC+10.5MesP	-58.1017	1.6887	100	-34.41	<.0001	0.05	-61.4519	-54.7514

The GLIMMIX Procedure

Differences of Colony_Typ*Condition Least Squares Means											
Colony_Type	Condition	_Colony_Type	_Condition	Estimate	Standard Error	DF	t Value	Pr > |t|	Alpha	Lower	Upper
BFU-E	Ao-EC Only	CFU-GM	AoEC+11.5MesP	-58.0689	1.6887	100	-34.39	<.0001	0.05	-61.4192	-54.7186
BFU-E	Ao-EC Only	CFU-GM	E10.5-EC Only	-11.3658	0	100	-Infty	<.0001	.	.	.
BFU-E	Ao-EC Only	CFU-GM	E10.5-MesP1+	-11.3658	0	100	-Infty	<.0001	.	.	.
BFU-E	Ao-EC Only	CFU-GM	E10.5-MesP1-	-11.3658	0	100	-Infty	<.0001	.	.	.
BFU-E	Ao-EC Only	CFU-GM	E11.5MesP1+On	-11.3658	0	100	-Infty	<.0001	.	.	.
BFU-E	Ao-EC Only	CFU-GM	E11.5MesP1-On	-11.3658	0	100	-Infty	<.0001	.	.	.
BFU-E	Ao-EC Only	CFU-GM	E13.5 EC Only	-11.3658	0	100	-Infty	<.0001	.	.	.
BFU-E	Ao-EC Only	CFU-GM	H-EC Only	-11.3658	0	100	-Infty	<.0001	.	.	.
BFU-E	Ao-EC Only	CFU-GM	H-EC+10.5MesP	-58.0833	1.6887	100	-34.40	<.0001	0.05	-61.4336	-54.7330
BFU-E	Ao-EC Only	CFU-GM	H-EC+11.5MesP	-57.9870	1.6887	100	-34.34	<.0001	0.05	-61.3373	-54.6367
BFU-E	Ao-EC Only	CFU-GM	I-EC Only	-11.3658	0	100	-Infty	<.0001	.	.	.
BFU-E	Ao-EC Only	CFU-GM	I-EC+10.5MesP	-55.5684	1.6905	100	-32.87	<.0001	0.05	-58.9224	-52.2144
BFU-E	Ao-EC Only	CFU-GM	I-EC+11.5MesP	-54.8774	1.6926	100	-32.42	<.0001	0.05	-58.2354	-51.5194
BFU-E	Ao-EC Only	CFU-GM	L-E only	-11.3658	0	100	-Infty	<.0001	.	.	.
BFU-E	Ao-EC Only	CFU-GM	L-E+11.5Mesp1	-58.1462	0	100	-Infty	<.0001	.	.	.
BFU-E	AoEC+10.5MesP	BFU-E	AoEC+11.5MesP	0.08198	1.6918	100	0.05	0.9614	0.05	-3.2746	3.4385
BFU-E	AoEC+10.5MesP	BFU-E	E10.5-EC Only	56.7651	1.6902	100	33.58	<.0001	0.05	53.4118	60.1185
BFU-E	AoEC+10.5MesP	BFU-E	E10.5-MesP1+	56.7651	1.6902	100	33.58	<.0001	0.05	53.4118	60.1185
BFU-E	AoEC+10.5MesP	BFU-E	E10.5-MesP1-	56.7651	1.6902	100	33.58	<.0001	0.05	53.4118	60.1185
BFU-E	AoEC+10.5MesP	BFU-E	E11.5MesP1+On	56.7651	1.6902	100	33.58	<.0001	0.05	53.4118	60.1185
BFU-E	AoEC+10.5MesP	BFU-E	E11.5MesP1-On	56.7651	1.6902	100	33.58	<.0001	0.05	53.4118	60.1185
BFU-E	AoEC+10.5MesP	BFU-E	E13.5 EC Only	56.7651	1.6902	100	33.58	<.0001	0.05	53.4118	60.1185
BFU-E	AoEC+10.5MesP	BFU-E	H-EC Only	56.7651	1.6902	100	33.58	<.0001	0.05	53.4118	60.1185
BFU-E	AoEC+10.5MesP	BFU-E	H-EC+10.5MesP	0.09309	1.6918	100	0.06	0.9562	0.05	-3.2635	3.4496

The GLIMMIX Procedure

Differences of Colony_Typ*Condition Least Squares Means											
Colony_Type	Condition	_Colony_Type	_Condition	Estimate	Standard Error	DF	t Value	Pr > |t|	Alpha	Lower	Upper
BFU-E	AoEC+10.5MesP	BFU-E	H-EC+11.5MesP	0.2315	1.6920	100	0.14	0.8915	0.05	-3.1254	3.5883
BFU-E	AoEC+10.5MesP	BFU-E	I-EC Only	56.7651	1.6902	100	33.58	<.0001	0.05	53.4118	60.1185
BFU-E	AoEC+10.5MesP	BFU-E	I-EC+10.5MesP	19.0620	608.44	100	0.03	0.9751	0.05	-1188.08	1226.20
BFU-E	AoEC+10.5MesP	BFU-E	I-EC+11.5MesP	19.0400	608.32	100	0.03	0.9751	0.05	-1187.84	1225.92
BFU-E	AoEC+10.5MesP	BFU-E	L-E only	56.7651	1.6902	100	33.58	<.0001	0.05	53.4118	60.1185
BFU-E	AoEC+10.5MesP	BFU-E	L-E+11.5Mesp1	0.04207	1.6902	100	0.02	0.9802	0.05	-3.3113	3.3954
BFU-E	AoEC+10.5MesP	CFU-GEMM	10.5EC+!0.5Me	3.8016	1.5127	100	2.51	0.0136	0.05	0.8003	6.8028
BFU-E	AoEC+10.5MesP	CFU-GEMM	11.5 EC Only	19.0288	607.65	100	0.03	0.9751	0.05	-1186.53	1224.59
BFU-E	AoEC+10.5MesP	CFU-GEMM	11.5EC+11.5Me	3.8635	1.5125	100	2.55	0.0121	0.05	0.8628	6.8643
BFU-E	AoEC+10.5MesP	CFU-GEMM	13.5EC+10.5Me	0.3038	1.6913	100	0.18	0.8578	0.05	-3.0517	3.6594
BFU-E	AoEC+10.5MesP	CFU-GEMM	13.5EC+11.5Me	0.6817	1.6916	100	0.40	0.6878	0.05	-2.6744	4.0378
BFU-E	AoEC+10.5MesP	CFU-GEMM	Ao-EC Only	56.7631	1.6914	100	33.56	<.0001	0.05	53.4073	60.1188
BFU-E	AoEC+10.5MesP	CFU-GEMM	AoEC+10.5MesP	0.6629	0.09872	100	6.72	<.0001	0.05	0.4671	0.8588
BFU-E	AoEC+10.5MesP	CFU-GEMM	AoEC+11.5MesP	0.5020	1.6914	100	0.30	0.7672	0.05	-2.8537	3.8577
BFU-E	AoEC+10.5MesP	CFU-GEMM	E10.5-EC Only	56.7631	1.6914	100	33.56	<.0001	0.05	53.4073	60.1188
BFU-E	AoEC+10.5MesP	CFU-GEMM	E10.5-MesP1+	56.7631	1.6914	100	33.56	<.0001	0.05	53.4073	60.1188
BFU-E	AoEC+10.5MesP	CFU-GEMM	E10.5-MesP1-	56.7631	1.6914	100	33.56	<.0001	0.05	53.4073	60.1188
BFU-E	AoEC+10.5MesP	CFU-GEMM	E11.5MesP1+On	56.7631	1.6914	100	33.56	<.0001	0.05	53.4073	60.1188
BFU-E	AoEC+10.5MesP	CFU-GEMM	E11.5MesP1-On	56.7631	1.6914	100	33.56	<.0001	0.05	53.4073	60.1188
BFU-E	AoEC+10.5MesP	CFU-GEMM	E13.5 EC Only	56.7631	1.6914	100	33.56	<.0001	0.05	53.4073	60.1188
BFU-E	AoEC+10.5MesP	CFU-GEMM	H-EC Only	56.7631	1.6914	100	33.56	<.0001	0.05	53.4073	60.1188
BFU-E	AoEC+10.5MesP	CFU-GEMM	H-EC+10.5MesP	0.7316	1.6916	100	0.43	0.6663	0.05	-2.6246	4.0878
BFU-E	AoEC+10.5MesP	CFU-GEMM	H-EC+11.5MesP	0.8402	1.6918	100	0.50	0.6206	0.05	-2.5164	4.1967
BFU-E	AoEC+10.5MesP	CFU-GEMM	I-EC Only	56.7631	1.6914	100	33.56	<.0001	0.05	53.4073	60.1188

The GLIMMIX Procedure

Differences of Colony_Typ*Condition Least Squares Means

Colony_Type	Condition	_Colony_Type	_Condition	Estimate	Standard Error	DF	t Value	Pr > |t|	Alpha	Lower	Upper
BFU-E	AoEC+10.5MesP	CFU-GEMM	I-EC+10.5MesP	2.0206	1.6942	100	1.19	0.2358	0.05	-1.3407	5.3819
BFU-E	AoEC+10.5MesP	CFU-GEMM	I-EC+11.5MesP	2.7529	1.6992	100	1.62	0.1084	0.05	-0.6182	6.1240
BFU-E	AoEC+10.5MesP	CFU-GEMM	L-E only	56.7631	1.6914	100	33.56	<.0001	0.05	53.4073	60.1188
BFU-E	AoEC+10.5MesP	CFU-GEMM	L-E+11.5Mesp1	0.5644	1.6914	100	0.33	0.7393	0.05	-2.7914	3.9201
BFU-E	AoEC+10.5MesP	CFU-GM	10.5EC+!0.5Me	2.2989	1.5119	100	1.52	0.1315	0.05	-0.7007	5.2985
BFU-E	AoEC+10.5MesP	CFU-GM	11.5 EC Only	1.8528	1.6940	100	1.09	0.2767	0.05	-1.5080	5.2136
BFU-E	AoEC+10.5MesP	CFU-GM	11.5EC+11.5Me	2.2489	1.5117	100	1.49	0.1400	0.05	-0.7501	5.2480
BFU-E	AoEC+10.5MesP	CFU-GM	13.5EC+10.5Me	-0.9791	1.6903	100	-0.58	0.5637	0.05	-4.3326	2.3744
BFU-E	AoEC+10.5MesP	CFU-GM	13.5EC+11.5Me	-1.2825	1.6902	100	-0.76	0.4498	0.05	-4.6359	2.0708
BFU-E	AoEC+10.5MesP	CFU-GM	Ao-EC Only	45.3994	1.6902	100	26.86	<.0001	0.05	42.0460	48.7527
BFU-E	AoEC+10.5MesP	CFU-GM	AoEC+10.5MesP	-1.3365	0.07239	100	-18.46	<.0001	0.05	-1.4801	-1.1929
BFU-E	AoEC+10.5MesP	CFU-GM	AoEC+11.5MesP	-1.3038	1.6902	100	-0.77	0.4423	0.05	-4.6571	2.0496
BFU-E	AoEC+10.5MesP	CFU-GM	E10.5-EC Only	45.3994	1.6902	100	26.86	<.0001	0.05	42.0460	48.7527
BFU-E	AoEC+10.5MesP	CFU-GM	E10.5-MesP1+	45.3994	1.6902	100	26.86	<.0001	0.05	42.0460	48.7527
BFU-E	AoEC+10.5MesP	CFU-GM	E10.5-MesP1-	45.3994	1.6902	100	26.86	<.0001	0.05	42.0460	48.7527
BFU-E	AoEC+10.5MesP	CFU-GM	E11.5MesP1+On	45.3994	1.6902	100	26.86	<.0001	0.05	42.0460	48.7527
BFU-E	AoEC+10.5MesP	CFU-GM	E11.5MesP1-On	45.3994	1.6902	100	26.86	<.0001	0.05	42.0460	48.7527
BFU-E	AoEC+10.5MesP	CFU-GM	E13.5 EC Only	45.3994	1.6902	100	26.86	<.0001	0.05	42.0460	48.7527
BFU-E	AoEC+10.5MesP	CFU-GM	H-EC Only	45.3994	1.6902	100	26.86	<.0001	0.05	42.0460	48.7527
BFU-E	AoEC+10.5MesP	CFU-GM	H-EC+10.5MesP	-1.3182	1.6902	100	-0.78	0.4373	0.05	-4.6715	2.0352
BFU-E	AoEC+10.5MesP	CFU-GM	H-EC+11.5MesP	-1.2218	1.6902	100	-0.72	0.4714	0.05	-4.5752	2.1316
BFU-E	AoEC+10.5MesP	CFU-GM	I-EC Only	45.3994	1.6902	100	26.86	<.0001	0.05	42.0460	48.7527
BFU-E	AoEC+10.5MesP	CFU-GM	I-EC+10.5MesP	1.1967	1.6921	100	0.71	0.4811	0.05	-2.1603	4.5538
BFU-E	AoEC+10.5MesP	CFU-GM	I-EC+11.5MesP	1.8878	1.6941	100	1.11	0.2678	0.05	-1.4734	5.2489

The GLIMMIX Procedure

Differences of Colony_Typ*Condition Least Squares Means											
Colony_Type	Condition	_Colony_Type	_Condition	Estimate	Standard Error	DF	t Value	Pr > |t|	Alpha	Lower	Upper
BFU-E	AoEC+10.5MesP	CFU-GM	L-E only	45.3994	1.6902	100	26.86	<.0001	0.05	42.0460	48.7527
BFU-E	AoEC+10.5MesP	CFU-GM	L-E+11.5Mesp1	-1.3810	1.6902	100	-0.82	0.4158	0.05	-4.7343	1.9723
BFU-E	AoEC+11.5MesP	BFU-E	E10.5-EC Only	56.6832	1.6903	100	33.53	<.0001	0.05	53.3297	60.0366
BFU-E	AoEC+11.5MesP	BFU-E	E10.5-MesP1+	56.6832	1.6903	100	33.53	<.0001	0.05	53.3297	60.0366
BFU-E	AoEC+11.5MesP	BFU-E	E10.5-MesP1-	56.6832	1.6903	100	33.53	<.0001	0.05	53.3297	60.0366
BFU-E	AoEC+11.5MesP	BFU-E	E11.5MesP1+On	56.6832	1.6903	100	33.53	<.0001	0.05	53.3297	60.0366
BFU-E	AoEC+11.5MesP	BFU-E	E11.5MesP1-On	56.6832	1.6903	100	33.53	<.0001	0.05	53.3297	60.0366
BFU-E	AoEC+11.5MesP	BFU-E	E13.5 EC Only	56.6832	1.6903	100	33.53	<.0001	0.05	53.3297	60.0366
BFU-E	AoEC+11.5MesP	BFU-E	H-EC Only	56.6832	1.6903	100	33.53	<.0001	0.05	53.3297	60.0366
BFU-E	AoEC+11.5MesP	BFU-E	H-EC+10.5MesP	0.01111	1.6919	100	0.01	0.9948	0.05	-3.3456	3.3678
BFU-E	AoEC+11.5MesP	BFU-E	H-EC+11.5MesP	0.1495	1.6920	100	0.09	0.9298	0.05	-3.2075	3.5064
BFU-E	AoEC+11.5MesP	BFU-E	I-EC Only	56.6832	1.6903	100	33.53	<.0001	0.05	53.3297	60.0366
BFU-E	AoEC+11.5MesP	BFU-E	I-EC+10.5MesP	18.9800	608.44	100	0.03	0.9752	0.05	-1188.16	1226.12
BFU-E	AoEC+11.5MesP	BFU-E	I-EC+11.5MesP	18.9580	608.32	100	0.03	0.9752	0.05	-1187.92	1225.84
BFU-E	AoEC+11.5MesP	BFU-E	L-E only	56.6832	1.6903	100	33.53	<.0001	0.05	53.3297	60.0366
BFU-E	AoEC+11.5MesP	BFU-E	L-E+11.5Mesp1	-0.03991	1.6903	100	-0.02	0.9812	0.05	-3.3934	3.3136
BFU-E	AoEC+11.5MesP	CFU-GEMM	10.5EC+!0.5Me	3.7196	1.5129	100	2.46	0.0157	0.05	0.7181	6.7211
BFU-E	AoEC+11.5MesP	CFU-GEMM	11.5 EC Only	18.9468	607.65	100	0.03	0.9752	0.05	-1186.61	1224.51
BFU-E	AoEC+11.5MesP	CFU-GEMM	11.5EC+11.5Me	3.7815	1.5126	100	2.50	0.0140	0.05	0.7806	6.7825
BFU-E	AoEC+11.5MesP	CFU-GEMM	13.5EC+10.5Me	0.2218	1.6914	100	0.13	0.8959	0.05	-3.1338	3.5775
BFU-E	AoEC+11.5MesP	CFU-GEMM	13.5EC+11.5Me	0.5997	1.6917	100	0.35	0.7237	0.05	-2.7565	3.9560
BFU-E	AoEC+11.5MesP	CFU-GEMM	Ao-EC Only	56.6811	1.6915	100	33.51	<.0001	0.05	53.3252	60.0370
BFU-E	AoEC+11.5MesP	CFU-GEMM	AoEC+10.5MesP	0.5809	1.6916	100	0.34	0.7320	0.05	-2.7752	3.9371
BFU-E	AoEC+11.5MesP	CFU-GEMM	AoEC+11.5MesP	0.4200	0.09689	100	4.34	<.0001	0.05	0.2278	0.6123

The GLIMMIX Procedure

Differences of Colony_Typ*Condition Least Squares Means											
Colony_Type	Condition	_Colony_Type	_Condition	Estimate	Standard Error	DF	t Value	Pr > |t|	Alpha	Lower	Upper
BFU-E	AoEC+11.5MesP	CFU-GEMM	E10.5-EC Only	56.6811	1.6915	100	33.51	<.0001	0.05	53.3252	60.0370
BFU-E	AoEC+11.5MesP	CFU-GEMM	E10.5-MesP1+	56.6811	1.6915	100	33.51	<.0001	0.05	53.3252	60.0370
BFU-E	AoEC+11.5MesP	CFU-GEMM	E10.5-MesP1-	56.6811	1.6915	100	33.51	<.0001	0.05	53.3252	60.0370
BFU-E	AoEC+11.5MesP	CFU-GEMM	E11.5MesP1+On	56.6811	1.6915	100	33.51	<.0001	0.05	53.3252	60.0370
BFU-E	AoEC+11.5MesP	CFU-GEMM	E11.5MesP1-On	56.6811	1.6915	100	33.51	<.0001	0.05	53.3252	60.0370
BFU-E	AoEC+11.5MesP	CFU-GEMM	E13.5 EC Only	56.6811	1.6915	100	33.51	<.0001	0.05	53.3252	60.0370
BFU-E	AoEC+11.5MesP	CFU-GEMM	H-EC Only	56.6811	1.6915	100	33.51	<.0001	0.05	53.3252	60.0370
BFU-E	AoEC+11.5MesP	CFU-GEMM	H-EC+10.5MesP	0.6496	1.6917	100	0.38	0.7018	0.05	-2.7067	4.0059
BFU-E	AoEC+11.5MesP	CFU-GEMM	H-EC+11.5MesP	0.7582	1.6919	100	0.45	0.6550	0.05	-2.5985	4.1149
BFU-E	AoEC+11.5MesP	CFU-GEMM	I-EC Only	56.6811	1.6915	100	33.51	<.0001	0.05	53.3252	60.0370
BFU-E	AoEC+11.5MesP	CFU-GEMM	I-EC+10.5MesP	1.9387	1.6943	100	1.14	0.2553	0.05	-1.4228	5.3001
BFU-E	AoEC+11.5MesP	CFU-GEMM	I-EC+11.5MesP	2.6709	1.6992	100	1.57	0.1191	0.05	-0.7003	6.0421
BFU-E	AoEC+11.5MesP	CFU-GEMM	L-E only	56.6811	1.6915	100	33.51	<.0001	0.05	53.3252	60.0370
BFU-E	AoEC+11.5MesP	CFU-GEMM	L-E+11.5Mesp1	0.4824	1.6915	100	0.29	0.7761	0.05	-2.8735	3.8383
BFU-E	AoEC+11.5MesP	CFU-GM	10.5EC+!0.5Me	2.2169	1.5120	100	1.47	0.1457	0.05	-0.7829	5.2167
BFU-E	AoEC+11.5MesP	CFU-GM	11.5 EC Only	1.7708	1.6941	100	1.05	0.2984	0.05	-1.5901	5.1318
BFU-E	AoEC+11.5MesP	CFU-GM	11.5EC+11.5Me	2.1670	1.5118	100	1.43	0.1549	0.05	-0.8323	5.1662
BFU-E	AoEC+11.5MesP	CFU-GM	13.5EC+10.5Me	-1.0611	1.6904	100	-0.63	0.5316	0.05	-4.4147	2.2925
BFU-E	AoEC+11.5MesP	CFU-GM	13.5EC+11.5Me	-1.3645	1.6903	100	-0.81	0.4214	0.05	-4.7180	1.9890
BFU-E	AoEC+11.5MesP	CFU-GM	Ao-EC Only	45.3174	1.6903	100	26.81	<.0001	0.05	41.9639	48.6708
BFU-E	AoEC+11.5MesP	CFU-GM	AoEC+10.5MesP	-1.4185	1.6903	100	-0.84	0.4034	0.05	-4.7720	1.9350
BFU-E	AoEC+11.5MesP	CFU-GM	AoEC+11.5MesP	-1.3857	0.07377	100	-18.79	<.0001	0.05	-1.5321	-1.2394
BFU-E	AoEC+11.5MesP	CFU-GM	E10.5-EC Only	45.3174	1.6903	100	26.81	<.0001	0.05	41.9639	48.6708
BFU-E	AoEC+11.5MesP	CFU-GM	E10.5-MesP1+	45.3174	1.6903	100	26.81	<.0001	0.05	41.9639	48.6708

The GLIMMIX Procedure

Differences of Colony_Typ*Condition Least Squares Means											
Colony_Type	Condition	_Colony_Type	_Condition	Estimate	Standard Error	DF	t Value	Pr > |t|	Alpha	Lower	Upper
BFU-E	AoEC+11.5MesP	CFU-GM	E10.5-MesP1-	45.3174	1.6903	100	26.81	<.0001	0.05	41.9639	48.6708
BFU-E	AoEC+11.5MesP	CFU-GM	E11.5MesP1+On	45.3174	1.6903	100	26.81	<.0001	0.05	41.9639	48.6708
BFU-E	AoEC+11.5MesP	CFU-GM	E11.5MesP1-On	45.3174	1.6903	100	26.81	<.0001	0.05	41.9639	48.6708
BFU-E	AoEC+11.5MesP	CFU-GM	E13.5 EC Only	45.3174	1.6903	100	26.81	<.0001	0.05	41.9639	48.6708
BFU-E	AoEC+11.5MesP	CFU-GM	H-EC Only	45.3174	1.6903	100	26.81	<.0001	0.05	41.9639	48.6708
BFU-E	AoEC+11.5MesP	CFU-GM	H-EC+10.5MesP	-1.4002	1.6903	100	-0.83	0.4094	0.05	-4.7536	1.9533
BFU-E	AoEC+11.5MesP	CFU-GM	H-EC+11.5MesP	-1.3038	1.6903	100	-0.77	0.4423	0.05	-4.6573	2.0497
BFU-E	AoEC+11.5MesP	CFU-GM	I-EC Only	45.3174	1.6903	100	26.81	<.0001	0.05	41.9639	48.6708
BFU-E	AoEC+11.5MesP	CFU-GM	I-EC+10.5MesP	1.1147	1.6922	100	0.66	0.5116	0.05	-2.2425	4.4719
BFU-E	AoEC+11.5MesP	CFU-GM	I-EC+11.5MesP	1.8058	1.6942	100	1.07	0.2891	0.05	-1.5555	5.1670
BFU-E	AoEC+11.5MesP	CFU-GM	L-E only	45.3174	1.6903	100	26.81	<.0001	0.05	41.9639	48.6708
BFU-E	AoEC+11.5MesP	CFU-GM	L-E+11.5Mesp1	-1.4630	1.6903	100	-0.87	0.3888	0.05	-4.8165	1.8905
BFU-E	E10.5-EC Only	BFU-E	E10.5-MesP1+	5.49E-13	.	.	.	.	.	.	.
BFU-E	E10.5-EC Only	BFU-E	E10.5-MesP1-	4.01E-13	.	.	.	.	.	.	.
BFU-E	E10.5-EC Only	BFU-E	E11.5MesP1+On	4.81E-13	.	.	.	.	.	.	.
BFU-E	E10.5-EC Only	BFU-E	E11.5MesP1-On	2.31E-13	.	.	.	.	.	.	.
BFU-E	E10.5-EC Only	BFU-E	E13.5 EC Only	9.06E-14	.	.	.	.	.	.	.
BFU-E	E10.5-EC Only	BFU-E	H-EC Only	0	.	.	.	.	.	.	.
BFU-E	E10.5-EC Only	BFU-E	H-EC+10.5MesP	-56.6721	1.6903	100	-33.53	<.0001	0.05	-60.0255	-53.3186
BFU-E	E10.5-EC Only	BFU-E	H-EC+11.5MesP	-56.5337	1.6904	100	-33.44	<.0001	0.05	-59.8874	-53.1800
BFU-E	E10.5-EC Only	BFU-E	I-EC Only	2.97E-13	.	.	.	.	.	.	.
BFU-E	E10.5-EC Only	BFU-E	I-EC+10.5MesP	-37.7032	608.44	100	-0.06	0.9507	0.05	-1244.84	1169.43
BFU-E	E10.5-EC Only	BFU-E	I-EC+11.5MesP	-37.7251	608.32	100	-0.06	0.9507	0.05	-1244.61	1169.16
BFU-E	E10.5-EC Only	BFU-E	L-E only	5.19E-13	.	.	.	.	.	.	.

The GLIMMIX Procedure

Differences of Colony_Typ*Condition Least Squares Means											
Colony_Type	Condition	_Colony_Type	_Condition	Estimate	Standard Error	DF	t Value	Pr > |t|	Alpha	Lower	Upper
BFU-E	E10.5-EC Only	BFU-E	L-E+11.5Mesp1	-56.7231	0	100	-Infty	<.0001	.	.	.
BFU-E	E10.5-EC Only	CFU-GEMM	10.5EC+!0.5Me	-52.9636	1.5111	100	-35.05	<.0001	0.05	-55.9615	-49.9657
BFU-E	E10.5-EC Only	CFU-GEMM	11.5 EC Only	-37.7364	607.65	100	-0.06	0.9506	0.05	-1243.30	1167.82
BFU-E	E10.5-EC Only	CFU-GEMM	11.5EC+11.5Me	-52.9016	1.5108	100	-35.02	<.0001	0.05	-55.8990	-49.9042
BFU-E	E10.5-EC Only	CFU-GEMM	13.5EC+10.5Me	-56.4613	1.6898	100	-33.41	<.0001	0.05	-59.8138	-53.1089
BFU-E	E10.5-EC Only	CFU-GEMM	13.5EC+11.5Me	-56.0834	1.6900	100	-33.18	<.0001	0.05	-59.4364	-52.7304
BFU-E	E10.5-EC Only	CFU-GEMM	Ao-EC Only	-0.00210	0.06423	100	-0.03	0.9740	0.05	-0.1295	0.1253
BFU-E	E10.5-EC Only	CFU-GEMM	AoEC+10.5MesP	-56.1022	1.6900	100	-33.20	<.0001	0.05	-59.4551	-52.7493
BFU-E	E10.5-EC Only	CFU-GEMM	AoEC+11.5MesP	-56.2631	1.6898	100	-33.29	<.0001	0.05	-59.6157	-52.9105
BFU-E	E10.5-EC Only	CFU-GEMM	E10.5-EC Only	-0.00210	0.06423	100	-0.03	0.9740	0.05	-0.1295	0.1253
BFU-E	E10.5-EC Only	CFU-GEMM	E10.5-MesP1+	-0.00210	0.06423	100	-0.03	0.9740	0.05	-0.1295	0.1253
BFU-E	E10.5-EC Only	CFU-GEMM	E10.5-MesP1-	-0.00210	0.06423	100	-0.03	0.9740	0.05	-0.1295	0.1253
BFU-E	E10.5-EC Only	CFU-GEMM	E11.5MesP1+On	-0.00210	0.06423	100	-0.03	0.9740	0.05	-0.1295	0.1253
BFU-E	E10.5-EC Only	CFU-GEMM	E11.5MesP1-On	-0.00210	0.06423	100	-0.03	0.9740	0.05	-0.1295	0.1253
BFU-E	E10.5-EC Only	CFU-GEMM	E13.5 EC Only	-0.00210	0.06423	100	-0.03	0.9740	0.05	-0.1295	0.1253
BFU-E	E10.5-EC Only	CFU-GEMM	H-EC Only	-0.00210	0.06423	100	-0.03	0.9740	0.05	-0.1295	0.1253
BFU-E	E10.5-EC Only	CFU-GEMM	H-EC+10.5MesP	-56.0336	1.6901	100	-33.15	<.0001	0.05	-59.3867	-52.6805
BFU-E	E10.5-EC Only	CFU-GEMM	H-EC+11.5MesP	-55.9250	1.6903	100	-33.09	<.0001	0.05	-59.2784	-52.5715
BFU-E	E10.5-EC Only	CFU-GEMM	I-EC Only	-0.00210	0.06423	100	-0.03	0.9740	0.05	-0.1295	0.1253
BFU-E	E10.5-EC Only	CFU-GEMM	I-EC+10.5MesP	-54.7445	1.6927	100	-32.34	<.0001	0.05	-58.1027	-51.3863
BFU-E	E10.5-EC Only	CFU-GEMM	I-EC+11.5MesP	-54.0123	1.6976	100	-31.82	<.0001	0.05	-57.3803	-50.6443
BFU-E	E10.5-EC Only	CFU-GEMM	L-E only	-0.00210	0.06423	100	-0.03	0.9740	0.05	-0.1295	0.1253
BFU-E	E10.5-EC Only	CFU-GEMM	L-E+11.5Mesp1	-56.2008	0.06423	100	-874.93	<.0001	0.05	-56.3282	-56.0733
BFU-E	E10.5-EC Only	CFU-GM	10.5EC+!0.5Me	-54.4663	1.5102	100	-36.07	<.0001	0.05	-57.4625	-51.4700

The GLIMMIX Procedure

Differences of Colony_Typ*Condition Least Squares Means											
Colony_Type	Condition	_Colony_Type	_Condition	Estimate	Standard Error	DF	t Value	Pr > |t|	Alpha	Lower	Upper
BFU-E	E10.5-EC Only	CFU-GM	11.5 EC Only	-54.9123	1.6924	100	-32.45	<.0001	0.05	-58.2701	-51.5546
BFU-E	E10.5-EC Only	CFU-GM	11.5EC+11.5Me	-54.5162	1.5100	100	-36.10	<.0001	0.05	-57.5119	-51.5205
BFU-E	E10.5-EC Only	CFU-GM	13.5EC+10.5Me	-57.7442	1.6887	100	-34.19	<.0001	0.05	-61.0946	-54.3938
BFU-E	E10.5-EC Only	CFU-GM	13.5EC+11.5Me	-58.0477	1.6887	100	-34.37	<.0001	0.05	-61.3980	-54.6974
BFU-E	E10.5-EC Only	CFU-GM	Ao-EC Only	-11.3658	0	100	-Infty	<.0001	.	.	.
BFU-E	E10.5-EC Only	CFU-GM	AoEC+10.5MesP	-58.1017	1.6887	100	-34.41	<.0001	0.05	-61.4519	-54.7514
BFU-E	E10.5-EC Only	CFU-GM	AoEC+11.5MesP	-58.0689	1.6887	100	-34.39	<.0001	0.05	-61.4192	-54.7186
BFU-E	E10.5-EC Only	CFU-GM	E10.5-EC Only	-11.3658	0	100	-Infty	<.0001	.	.	.
BFU-E	E10.5-EC Only	CFU-GM	E10.5-MesP1+	-11.3658	0	100	-Infty	<.0001	.	.	.
BFU-E	E10.5-EC Only	CFU-GM	E10.5-MesP1-	-11.3658	0	100	-Infty	<.0001	.	.	.
BFU-E	E10.5-EC Only	CFU-GM	E11.5MesP1+On	-11.3658	0	100	-Infty	<.0001	.	.	.
BFU-E	E10.5-EC Only	CFU-GM	E11.5MesP1-On	-11.3658	0	100	-Infty	<.0001	.	.	.
BFU-E	E10.5-EC Only	CFU-GM	E13.5 EC Only	-11.3658	0	100	-Infty	<.0001	.	.	.
BFU-E	E10.5-EC Only	CFU-GM	H-EC Only	-11.3658	0	100	-Infty	<.0001	.	.	.
BFU-E	E10.5-EC Only	CFU-GM	H-EC+10.5MesP	-58.0833	1.6887	100	-34.40	<.0001	0.05	-61.4336	-54.7330
BFU-E	E10.5-EC Only	CFU-GM	H-EC+11.5MesP	-57.9870	1.6887	100	-34.34	<.0001	0.05	-61.3373	-54.6367
BFU-E	E10.5-EC Only	CFU-GM	I-EC Only	-11.3658	0	100	-Infty	<.0001	.	.	.
BFU-E	E10.5-EC Only	CFU-GM	I-EC+10.5MesP	-55.5684	1.6905	100	-32.87	<.0001	0.05	-58.9224	-52.2144
BFU-E	E10.5-EC Only	CFU-GM	I-EC+11.5MesP	-54.8774	1.6926	100	-32.42	<.0001	0.05	-58.2354	-51.5194
BFU-E	E10.5-EC Only	CFU-GM	L-E only	-11.3658	0	100	-Infty	<.0001	.	.	.
BFU-E	E10.5-EC Only	CFU-GM	L-E+11.5Mesp1	-58.1462	0	100	-Infty	<.0001	.	.	.
BFU-E	E10.5-MesP1+	BFU-E	E10.5-MesP1-	-147E-15	.	.	.	.	.	.	.
BFU-E	E10.5-MesP1+	BFU-E	E11.5MesP1+On	-675E-16	.	.	.	.	.	.	.
BFU-E	E10.5-MesP1+	BFU-E	E11.5MesP1-On	-318E-15	.	.	.	.	.	.	.

The GLIMMIX Procedure

Differences of Colony_Typ*Condition Least Squares Means											
Colony_Type	Condition	_Colony_Type	_Condition	Estimate	Standard Error	DF	t Value	Pr > |t|	Alpha	Lower	Upper
BFU-E	E10.5-MesP1+	BFU-E	E13.5 EC Only	-458E-15	.	.	.	.	.	.	.
BFU-E	E10.5-MesP1+	BFU-E	H-EC Only	-549E-15	.	.	.	.	.	.	.
BFU-E	E10.5-MesP1+	BFU-E	H-EC+10.5MesP	-56.6721	1.6903	100	-33.53	<.0001	0.05	-60.0255	-53.3186
BFU-E	E10.5-MesP1+	BFU-E	H-EC+11.5MesP	-56.5337	1.6904	100	-33.44	<.0001	0.05	-59.8874	-53.1800
BFU-E	E10.5-MesP1+	BFU-E	I-EC Only	-252E-15	.	.	.	.	.	.	.
BFU-E	E10.5-MesP1+	BFU-E	I-EC+10.5MesP	-37.7032	608.44	100	-0.06	0.9507	0.05	-1244.84	1169.43
BFU-E	E10.5-MesP1+	BFU-E	I-EC+11.5MesP	-37.7251	608.32	100	-0.06	0.9507	0.05	-1244.61	1169.16
BFU-E	E10.5-MesP1+	BFU-E	L-E only	-302E-16	.	.	.	.	.	.	.
BFU-E	E10.5-MesP1+	BFU-E	L-E+11.5Mesp1	-56.7231	0	100	-Infty	<.0001	.	.	.
BFU-E	E10.5-MesP1+	CFU-GEMM	10.5EC+!0.5Me	-52.9636	1.5111	100	-35.05	<.0001	0.05	-55.9615	-49.9657
BFU-E	E10.5-MesP1+	CFU-GEMM	11.5 EC Only	-37.7364	607.65	100	-0.06	0.9506	0.05	-1243.30	1167.82
BFU-E	E10.5-MesP1+	CFU-GEMM	11.5EC+11.5Me	-52.9016	1.5108	100	-35.02	<.0001	0.05	-55.8990	-49.9042
BFU-E	E10.5-MesP1+	CFU-GEMM	13.5EC+10.5Me	-56.4613	1.6898	100	-33.41	<.0001	0.05	-59.8138	-53.1089
BFU-E	E10.5-MesP1+	CFU-GEMM	13.5EC+11.5Me	-56.0834	1.6900	100	-33.18	<.0001	0.05	-59.4364	-52.7304
BFU-E	E10.5-MesP1+	CFU-GEMM	Ao-EC Only	-0.00210	0.06423	100	-0.03	0.9740	0.05	-0.1295	0.1253
BFU-E	E10.5-MesP1+	CFU-GEMM	AoEC+10.5MesP	-56.1022	1.6900	100	-33.20	<.0001	0.05	-59.4551	-52.7493
BFU-E	E10.5-MesP1+	CFU-GEMM	AoEC+11.5MesP	-56.2631	1.6898	100	-33.29	<.0001	0.05	-59.6157	-52.9105
BFU-E	E10.5-MesP1+	CFU-GEMM	E10.5-EC Only	-0.00210	0.06423	100	-0.03	0.9740	0.05	-0.1295	0.1253
BFU-E	E10.5-MesP1+	CFU-GEMM	E10.5-MesP1+	-0.00210	0.06423	100	-0.03	0.9740	0.05	-0.1295	0.1253
BFU-E	E10.5-MesP1+	CFU-GEMM	E10.5-MesP1-	-0.00210	0.06423	100	-0.03	0.9740	0.05	-0.1295	0.1253
BFU-E	E10.5-MesP1+	CFU-GEMM	E11.5MesP1+On	-0.00210	0.06423	100	-0.03	0.9740	0.05	-0.1295	0.1253
BFU-E	E10.5-MesP1+	CFU-GEMM	E11.5MesP1-On	-0.00210	0.06423	100	-0.03	0.9740	0.05	-0.1295	0.1253
BFU-E	E10.5-MesP1+	CFU-GEMM	E13.5 EC Only	-0.00210	0.06423	100	-0.03	0.9740	0.05	-0.1295	0.1253
BFU-E	E10.5-MesP1+	CFU-GEMM	H-EC Only	-0.00210	0.06423	100	-0.03	0.9740	0.05	-0.1295	0.1253

The GLIMMIX Procedure

Differences of Colony_Typ*Condition Least Squares Means											
Colony_Type	Condition	_Colony_Type	_Condition	Estimate	Standard Error	DF	t Value	Pr > |t|	Alpha	Lower	Upper
BFU-E	E10.5-MesP1+	CFU-GEMM	H-EC+10.5MesP	-56.0336	1.6901	100	-33.15	<.0001	0.05	-59.3867	-52.6805
BFU-E	E10.5-MesP1+	CFU-GEMM	H-EC+11.5MesP	-55.9250	1.6903	100	-33.09	<.0001	0.05	-59.2784	-52.5715
BFU-E	E10.5-MesP1+	CFU-GEMM	I-EC Only	-0.00210	0.06423	100	-0.03	0.9740	0.05	-0.1295	0.1253
BFU-E	E10.5-MesP1+	CFU-GEMM	I-EC+10.5MesP	-54.7445	1.6927	100	-32.34	<.0001	0.05	-58.1027	-51.3863
BFU-E	E10.5-MesP1+	CFU-GEMM	I-EC+11.5MesP	-54.0123	1.6976	100	-31.82	<.0001	0.05	-57.3803	-50.6443
BFU-E	E10.5-MesP1+	CFU-GEMM	L-E only	-0.00210	0.06423	100	-0.03	0.9740	0.05	-0.1295	0.1253
BFU-E	E10.5-MesP1+	CFU-GEMM	L-E+11.5Mesp1	-56.2008	0.06423	100	-874.93	<.0001	0.05	-56.3282	-56.0733
BFU-E	E10.5-MesP1+	CFU-GM	10.5EC+!0.5Me	-54.4663	1.5102	100	-36.07	<.0001	0.05	-57.4625	-51.4700
BFU-E	E10.5-MesP1+	CFU-GM	11.5 EC Only	-54.9123	1.6924	100	-32.45	<.0001	0.05	-58.2701	-51.5546
BFU-E	E10.5-MesP1+	CFU-GM	11.5EC+11.5Me	-54.5162	1.5100	100	-36.10	<.0001	0.05	-57.5119	-51.5205
BFU-E	E10.5-MesP1+	CFU-GM	13.5EC+10.5Me	-57.7442	1.6887	100	-34.19	<.0001	0.05	-61.0946	-54.3938
BFU-E	E10.5-MesP1+	CFU-GM	13.5EC+11.5Me	-58.0477	1.6887	100	-34.37	<.0001	0.05	-61.3980	-54.6974
BFU-E	E10.5-MesP1+	CFU-GM	Ao-EC Only	-11.3658	0	100	-Infty	<.0001	.	.	.
BFU-E	E10.5-MesP1+	CFU-GM	AoEC+10.5MesP	-58.1017	1.6887	100	-34.41	<.0001	0.05	-61.4519	-54.7514
BFU-E	E10.5-MesP1+	CFU-GM	AoEC+11.5MesP	-58.0689	1.6887	100	-34.39	<.0001	0.05	-61.4192	-54.7186
BFU-E	E10.5-MesP1+	CFU-GM	E10.5-EC Only	-11.3658	0	100	-Infty	<.0001	.	.	.
BFU-E	E10.5-MesP1+	CFU-GM	E10.5-MesP1+	-11.3658	0	100	-Infty	<.0001	.	.	.
BFU-E	E10.5-MesP1+	CFU-GM	E10.5-MesP1-	-11.3658	0	100	-Infty	<.0001	.	.	.
BFU-E	E10.5-MesP1+	CFU-GM	E11.5MesP1+On	-11.3658	0	100	-Infty	<.0001	.	.	.
BFU-E	E10.5-MesP1+	CFU-GM	E11.5MesP1-On	-11.3658	0	100	-Infty	<.0001	.	.	.
BFU-E	E10.5-MesP1+	CFU-GM	E13.5 EC Only	-11.3658	0	100	-Infty	<.0001	.	.	.
BFU-E	E10.5-MesP1+	CFU-GM	H-EC Only	-11.3658	0	100	-Infty	<.0001	.	.	.
BFU-E	E10.5-MesP1+	CFU-GM	H-EC+10.5MesP	-58.0833	1.6887	100	-34.40	<.0001	0.05	-61.4336	-54.7330
BFU-E	E10.5-MesP1+	CFU-GM	H-EC+11.5MesP	-57.9870	1.6887	100	-34.34	<.0001	0.05	-61.3373	-54.6367

The GLIMMIX Procedure

Differences of Colony_Typ*Condition Least Squares Means											
Colony_Type	Condition	_Colony_Type	_Condition	Estimate	Standard Error	DF	t Value	Pr > |t|	Alpha	Lower	Upper
BFU-E	E10.5-MesP1+	CFU-GM	I-EC Only	-11.3658	0	100	-Infty	<.0001	.	.	.
BFU-E	E10.5-MesP1+	CFU-GM	I-EC+10.5MesP	-55.5684	1.6905	100	-32.87	<.0001	0.05	-58.9224	-52.2144
BFU-E	E10.5-MesP1+	CFU-GM	I-EC+11.5MesP	-54.8774	1.6926	100	-32.42	<.0001	0.05	-58.2354	-51.5194
BFU-E	E10.5-MesP1+	CFU-GM	L-E only	-11.3658	0	100	-Infty	<.0001	.	.	.
BFU-E	E10.5-MesP1+	CFU-GM	L-E+11.5Mesp1	-58.1462	0	100	-Infty	<.0001	.	.	.
BFU-E	E10.5-MesP1-	BFU-E	E11.5MesP1+On	7.99E-14	.	.	.	.	.	.	.
BFU-E	E10.5-MesP1-	BFU-E	E11.5MesP1-On	-171E-15	.	.	.	.	.	.	.
BFU-E	E10.5-MesP1-	BFU-E	E13.5 EC Only	-311E-15	.	.	.	.	.	.	.
BFU-E	E10.5-MesP1-	BFU-E	H-EC Only	-401E-15	.	.	.	.	.	.	.
BFU-E	E10.5-MesP1-	BFU-E	H-EC+10.5MesP	-56.6721	1.6903	100	-33.53	<.0001	0.05	-60.0255	-53.3186
BFU-E	E10.5-MesP1-	BFU-E	H-EC+11.5MesP	-56.5337	1.6904	100	-33.44	<.0001	0.05	-59.8874	-53.1800
BFU-E	E10.5-MesP1-	BFU-E	I-EC Only	-105E-15	.	.	.	.	.	.	.
BFU-E	E10.5-MesP1-	BFU-E	I-EC+10.5MesP	-37.7032	608.44	100	-0.06	0.9507	0.05	-1244.84	1169.43
BFU-E	E10.5-MesP1-	BFU-E	I-EC+11.5MesP	-37.7251	608.32	100	-0.06	0.9507	0.05	-1244.61	1169.16
BFU-E	E10.5-MesP1-	BFU-E	L-E only	1.17E-13	.	.	.	.	.	.	.
BFU-E	E10.5-MesP1-	BFU-E	L-E+11.5Mesp1	-56.7231	0	100	-Infty	<.0001	.	.	.
BFU-E	E10.5-MesP1-	CFU-GEMM	10.5EC+!0.5Me	-52.9636	1.5111	100	-35.05	<.0001	0.05	-55.9615	-49.9657
BFU-E	E10.5-MesP1-	CFU-GEMM	11.5 EC Only	-37.7364	607.65	100	-0.06	0.9506	0.05	-1243.30	1167.82
BFU-E	E10.5-MesP1-	CFU-GEMM	11.5EC+11.5Me	-52.9016	1.5108	100	-35.02	<.0001	0.05	-55.8990	-49.9042
BFU-E	E10.5-MesP1-	CFU-GEMM	13.5EC+10.5Me	-56.4613	1.6898	100	-33.41	<.0001	0.05	-59.8138	-53.1089
BFU-E	E10.5-MesP1-	CFU-GEMM	13.5EC+11.5Me	-56.0834	1.6900	100	-33.18	<.0001	0.05	-59.4364	-52.7304
BFU-E	E10.5-MesP1-	CFU-GEMM	Ao-EC Only	-0.00210	0.06423	100	-0.03	0.9740	0.05	-0.1295	0.1253
BFU-E	E10.5-MesP1-	CFU-GEMM	AoEC+10.5MesP	-56.1022	1.6900	100	-33.20	<.0001	0.05	-59.4551	-52.7493
BFU-E	E10.5-MesP1-	CFU-GEMM	AoEC+11.5MesP	-56.2631	1.6898	100	-33.29	<.0001	0.05	-59.6157	-52.9105

The GLIMMIX Procedure

Differences of Colony_Typ*Condition Least Squares Means											
Colony_Type	Condition	_Colony_Type	_Condition	Estimate	Standard Error	DF	t Value	Pr > |t|	Alpha	Lower	Upper
BFU-E	E10.5-MesP1-	CFU-GEMM	E10.5-EC Only	-0.00210	0.06423	100	-0.03	0.9740	0.05	-0.1295	0.1253
BFU-E	E10.5-MesP1-	CFU-GEMM	E10.5-MesP1+	-0.00210	0.06423	100	-0.03	0.9740	0.05	-0.1295	0.1253
BFU-E	E10.5-MesP1-	CFU-GEMM	E10.5-MesP1-	-0.00210	0.06423	100	-0.03	0.9740	0.05	-0.1295	0.1253
BFU-E	E10.5-MesP1-	CFU-GEMM	E11.5MesP1+On	-0.00210	0.06423	100	-0.03	0.9740	0.05	-0.1295	0.1253
BFU-E	E10.5-MesP1-	CFU-GEMM	E11.5MesP1-On	-0.00210	0.06423	100	-0.03	0.9740	0.05	-0.1295	0.1253
BFU-E	E10.5-MesP1-	CFU-GEMM	E13.5 EC Only	-0.00210	0.06423	100	-0.03	0.9740	0.05	-0.1295	0.1253
BFU-E	E10.5-MesP1-	CFU-GEMM	H-EC Only	-0.00210	0.06423	100	-0.03	0.9740	0.05	-0.1295	0.1253
BFU-E	E10.5-MesP1-	CFU-GEMM	H-EC+10.5MesP	-56.0336	1.6901	100	-33.15	<.0001	0.05	-59.3867	-52.6805
BFU-E	E10.5-MesP1-	CFU-GEMM	H-EC+11.5MesP	-55.9250	1.6903	100	-33.09	<.0001	0.05	-59.2784	-52.5715
BFU-E	E10.5-MesP1-	CFU-GEMM	I-EC Only	-0.00210	0.06423	100	-0.03	0.9740	0.05	-0.1295	0.1253
BFU-E	E10.5-MesP1-	CFU-GEMM	I-EC+10.5MesP	-54.7445	1.6927	100	-32.34	<.0001	0.05	-58.1027	-51.3863
BFU-E	E10.5-MesP1-	CFU-GEMM	I-EC+11.5MesP	-54.0123	1.6976	100	-31.82	<.0001	0.05	-57.3803	-50.6443
BFU-E	E10.5-MesP1-	CFU-GEMM	L-E only	-0.00210	0.06423	100	-0.03	0.9740	0.05	-0.1295	0.1253
BFU-E	E10.5-MesP1-	CFU-GEMM	L-E+11.5Mesp1	-56.2008	0.06423	100	-874.93	<.0001	0.05	-56.3282	-56.0733
BFU-E	E10.5-MesP1-	CFU-GM	10.5EC+!0.5Me	-54.4663	1.5102	100	-36.07	<.0001	0.05	-57.4625	-51.4700
BFU-E	E10.5-MesP1-	CFU-GM	11.5 EC Only	-54.9123	1.6924	100	-32.45	<.0001	0.05	-58.2701	-51.5546
BFU-E	E10.5-MesP1-	CFU-GM	11.5EC+11.5Me	-54.5162	1.5100	100	-36.10	<.0001	0.05	-57.5119	-51.5205
BFU-E	E10.5-MesP1-	CFU-GM	13.5EC+10.5Me	-57.7442	1.6887	100	-34.19	<.0001	0.05	-61.0946	-54.3938
BFU-E	E10.5-MesP1-	CFU-GM	13.5EC+11.5Me	-58.0477	1.6887	100	-34.37	<.0001	0.05	-61.3980	-54.6974
BFU-E	E10.5-MesP1-	CFU-GM	Ao-EC Only	-11.3658	0	100	-Infty	<.0001	.	.	.
BFU-E	E10.5-MesP1-	CFU-GM	AoEC+10.5MesP	-58.1017	1.6887	100	-34.41	<.0001	0.05	-61.4519	-54.7514
BFU-E	E10.5-MesP1-	CFU-GM	AoEC+11.5MesP	-58.0689	1.6887	100	-34.39	<.0001	0.05	-61.4192	-54.7186
BFU-E	E10.5-MesP1-	CFU-GM	E10.5-EC Only	-11.3658	0	100	-Infty	<.0001	.	.	.
BFU-E	E10.5-MesP1-	CFU-GM	E10.5-MesP1+	-11.3658	0	100	-Infty	<.0001	.	.	.

The GLIMMIX Procedure

Differences of Colony_Typ*Condition Least Squares Means

Colony_Type	Condition	_Colony_Type	_Condition	Estimate	Standard Error	DF	t Value	Pr > |t|	Alpha	Lower	Upper
BFU-E	E10.5-MesP1-	CFU-GM	E10.5-MesP1-	-11.3658	0	100	-Infty	<.0001	.	.	.
BFU-E	E10.5-MesP1-	CFU-GM	E11.5MesP1+On	-11.3658	0	100	-Infty	<.0001	.	.	.
BFU-E	E10.5-MesP1-	CFU-GM	E11.5MesP1-On	-11.3658	0	100	-Infty	<.0001	.	.	.
BFU-E	E10.5-MesP1-	CFU-GM	E13.5 EC Only	-11.3658	0	100	-Infty	<.0001	.	.	.
BFU-E	E10.5-MesP1-	CFU-GM	H-EC Only	-11.3658	0	100	-Infty	<.0001	.	.	.
BFU-E	E10.5-MesP1-	CFU-GM	H-EC+10.5MesP	-58.0833	1.6887	100	-34.40	<.0001	0.05	-61.4336	-54.7330
BFU-E	E10.5-MesP1-	CFU-GM	H-EC+11.5MesP	-57.9870	1.6887	100	-34.34	<.0001	0.05	-61.3373	-54.6367
BFU-E	E10.5-MesP1-	CFU-GM	I-EC Only	-11.3658	0	100	-Infty	<.0001	.	.	.
BFU-E	E10.5-MesP1-	CFU-GM	I-EC+10.5MesP	-55.5684	1.6905	100	-32.87	<.0001	0.05	-58.9224	-52.2144
BFU-E	E10.5-MesP1-	CFU-GM	I-EC+11.5MesP	-54.8774	1.6926	100	-32.42	<.0001	0.05	-58.2354	-51.5194
BFU-E	E10.5-MesP1-	CFU-GM	L-E only	-11.3658	0	100	-Infty	<.0001	.	.	.
BFU-E	E10.5-MesP1-	CFU-GM	L-E+11.5Mesp1	-58.1462	0	100	-Infty	<.0001	.	.	.
BFU-E	E11.5MesP1+On	BFU-E	E11.5MesP1-On	-25E-14	.	.	.	.	.	.	.
BFU-E	E11.5MesP1+On	BFU-E	E13.5 EC Only	-391E-15	.	.	.	.	.	.	.
BFU-E	E11.5MesP1+On	BFU-E	H-EC Only	-481E-15	.	.	.	.	.	.	.
BFU-E	E11.5MesP1+On	BFU-E	H-EC+10.5MesP	-56.6721	1.6903	100	-33.53	<.0001	0.05	-60.0255	-53.3186
BFU-E	E11.5MesP1+On	BFU-E	H-EC+11.5MesP	-56.5337	1.6904	100	-33.44	<.0001	0.05	-59.8874	-53.1800
BFU-E	E11.5MesP1+On	BFU-E	I-EC Only	-185E-15	.	.	.	.	.	.	.
BFU-E	E11.5MesP1+On	BFU-E	I-EC+10.5MesP	-37.7032	608.44	100	-0.06	0.9507	0.05	-1244.84	1169.43
BFU-E	E11.5MesP1+On	BFU-E	I-EC+11.5MesP	-37.7251	608.32	100	-0.06	0.9507	0.05	-1244.61	1169.16
BFU-E	E11.5MesP1+On	BFU-E	L-E only	3.73E-14	.	.	.	.	.	.	.
BFU-E	E11.5MesP1+On	BFU-E	L-E+11.5Mesp1	-56.7231	0	100	-Infty	<.0001	.	.	.
BFU-E	E11.5MesP1+On	CFU-GEMM	10.5EC+!0.5Me	-52.9636	1.5111	100	-35.05	<.0001	0.05	-55.9615	-49.9657
BFU-E	E11.5MesP1+On	CFU-GEMM	11.5 EC Only	-37.7364	607.65	100	-0.06	0.9506	0.05	-1243.30	1167.82

The GLIMMIX Procedure

Differences of Colony_Typ*Condition Least Squares Means											
Colony_Type	Condition	_Colony_Type	_Condition	Estimate	Standard Error	DF	t Value	Pr > |t|	Alpha	Lower	Upper
BFU-E	E11.5MesP1+On	CFU-GEMM	11.5EC+11.5Me	-52.9016	1.5108	100	-35.02	<.0001	0.05	-55.8990	-49.9042
BFU-E	E11.5MesP1+On	CFU-GEMM	13.5EC+10.5Me	-56.4613	1.6898	100	-33.41	<.0001	0.05	-59.8138	-53.1089
BFU-E	E11.5MesP1+On	CFU-GEMM	13.5EC+11.5Me	-56.0834	1.6900	100	-33.18	<.0001	0.05	-59.4364	-52.7304
BFU-E	E11.5MesP1+On	CFU-GEMM	Ao-EC Only	-0.00210	0.06423	100	-0.03	0.9740	0.05	-0.1295	0.1253
BFU-E	E11.5MesP1+On	CFU-GEMM	AoEC+10.5MesP	-56.1022	1.6900	100	-33.20	<.0001	0.05	-59.4551	-52.7493
BFU-E	E11.5MesP1+On	CFU-GEMM	AoEC+11.5MesP	-56.2631	1.6898	100	-33.29	<.0001	0.05	-59.6157	-52.9105
BFU-E	E11.5MesP1+On	CFU-GEMM	E10.5-EC Only	-0.00210	0.06423	100	-0.03	0.9740	0.05	-0.1295	0.1253
BFU-E	E11.5MesP1+On	CFU-GEMM	E10.5-MesP1+	-0.00210	0.06423	100	-0.03	0.9740	0.05	-0.1295	0.1253
BFU-E	E11.5MesP1+On	CFU-GEMM	E10.5-MesP1-	-0.00210	0.06423	100	-0.03	0.9740	0.05	-0.1295	0.1253
BFU-E	E11.5MesP1+On	CFU-GEMM	E11.5MesP1+On	-0.00210	0.06423	100	-0.03	0.9740	0.05	-0.1295	0.1253
BFU-E	E11.5MesP1+On	CFU-GEMM	E11.5MesP1-On	-0.00210	0.06423	100	-0.03	0.9740	0.05	-0.1295	0.1253
BFU-E	E11.5MesP1+On	CFU-GEMM	E13.5 EC Only	-0.00210	0.06423	100	-0.03	0.9740	0.05	-0.1295	0.1253
BFU-E	E11.5MesP1+On	CFU-GEMM	H-EC Only	-0.00210	0.06423	100	-0.03	0.9740	0.05	-0.1295	0.1253
BFU-E	E11.5MesP1+On	CFU-GEMM	H-EC+10.5MesP	-56.0336	1.6901	100	-33.15	<.0001	0.05	-59.3867	-52.6805
BFU-E	E11.5MesP1+On	CFU-GEMM	H-EC+11.5MesP	-55.9250	1.6903	100	-33.09	<.0001	0.05	-59.2784	-52.5715
BFU-E	E11.5MesP1+On	CFU-GEMM	I-EC Only	-0.00210	0.06423	100	-0.03	0.9740	0.05	-0.1295	0.1253
BFU-E	E11.5MesP1+On	CFU-GEMM	I-EC+10.5MesP	-54.7445	1.6927	100	-32.34	<.0001	0.05	-58.1027	-51.3863
BFU-E	E11.5MesP1+On	CFU-GEMM	I-EC+11.5MesP	-54.0123	1.6976	100	-31.82	<.0001	0.05	-57.3803	-50.6443
BFU-E	E11.5MesP1+On	CFU-GEMM	L-E only	-0.00210	0.06423	100	-0.03	0.9740	0.05	-0.1295	0.1253
BFU-E	E11.5MesP1+On	CFU-GEMM	L-E+11.5Mesp1	-56.2008	0.06423	100	-874.93	<.0001	0.05	-56.3282	-56.0733
BFU-E	E11.5MesP1+On	CFU-GM	10.5EC+!0.5Me	-54.4663	1.5102	100	-36.07	<.0001	0.05	-57.4625	-51.4700
BFU-E	E11.5MesP1+On	CFU-GM	11.5 EC Only	-54.9123	1.6924	100	-32.45	<.0001	0.05	-58.2701	-51.5546
BFU-E	E11.5MesP1+On	CFU-GM	11.5EC+11.5Me	-54.5162	1.5100	100	-36.10	<.0001	0.05	-57.5119	-51.5205
BFU-E	E11.5MesP1+On	CFU-GM	13.5EC+10.5Me	-57.7442	1.6887	100	-34.19	<.0001	0.05	-61.0946	-54.3938

The GLIMMIX Procedure

Differences of Colony_Typ*Condition Least Squares Means											
Colony_Type	Condition	_Colony_Type	_Condition	Estimate	Standard Error	DF	t Value	Pr > |t|	Alpha	Lower	Upper
BFU-E	E11.5MesP1+On	CFU-GM	13.5EC+11.5Me	-58.0477	1.6887	100	-34.37	<.0001	0.05	-61.3980	-54.6974
BFU-E	E11.5MesP1+On	CFU-GM	Ao-EC Only	-11.3658	0	100	-Infty	<.0001	.	.	.
BFU-E	E11.5MesP1+On	CFU-GM	AoEC+10.5MesP	-58.1017	1.6887	100	-34.41	<.0001	0.05	-61.4519	-54.7514
BFU-E	E11.5MesP1+On	CFU-GM	AoEC+11.5MesP	-58.0689	1.6887	100	-34.39	<.0001	0.05	-61.4192	-54.7186
BFU-E	E11.5MesP1+On	CFU-GM	E10.5-EC Only	-11.3658	0	100	-Infty	<.0001	.	.	.
BFU-E	E11.5MesP1+On	CFU-GM	E10.5-MesP1+	-11.3658	0	100	-Infty	<.0001	.	.	.
BFU-E	E11.5MesP1+On	CFU-GM	E10.5-MesP1-	-11.3658	0	100	-Infty	<.0001	.	.	.
BFU-E	E11.5MesP1+On	CFU-GM	E11.5MesP1+On	-11.3658	0	100	-Infty	<.0001	.	.	.
BFU-E	E11.5MesP1+On	CFU-GM	E11.5MesP1-On	-11.3658	0	100	-Infty	<.0001	.	.	.
BFU-E	E11.5MesP1+On	CFU-GM	E13.5 EC Only	-11.3658	0	100	-Infty	<.0001	.	.	.
BFU-E	E11.5MesP1+On	CFU-GM	H-EC Only	-11.3658	0	100	-Infty	<.0001	.	.	.
BFU-E	E11.5MesP1+On	CFU-GM	H-EC+10.5MesP	-58.0833	1.6887	100	-34.40	<.0001	0.05	-61.4336	-54.7330
BFU-E	E11.5MesP1+On	CFU-GM	H-EC+11.5MesP	-57.9870	1.6887	100	-34.34	<.0001	0.05	-61.3373	-54.6367
BFU-E	E11.5MesP1+On	CFU-GM	I-EC Only	-11.3658	0	100	-Infty	<.0001	.	.	.
BFU-E	E11.5MesP1+On	CFU-GM	I-EC+10.5MesP	-55.5684	1.6905	100	-32.87	<.0001	0.05	-58.9224	-52.2144
BFU-E	E11.5MesP1+On	CFU-GM	I-EC+11.5MesP	-54.8774	1.6926	100	-32.42	<.0001	0.05	-58.2354	-51.5194
BFU-E	E11.5MesP1+On	CFU-GM	L-E only	-11.3658	0	100	-Infty	<.0001	.	.	.
BFU-E	E11.5MesP1+On	CFU-GM	L-E+11.5Mesp1	-58.1462	0	100	-Infty	<.0001	.	.	.
BFU-E	E11.5MesP1-On	BFU-E	E13.5 EC Only	-14E-14	.	.	.	.	.	.	.
BFU-E	E11.5MesP1-On	BFU-E	H-EC Only	-231E-15	.	.	.	.	.	.	.
BFU-E	E11.5MesP1-On	BFU-E	H-EC+10.5MesP	-56.6721	1.6903	100	-33.53	<.0001	0.05	-60.0255	-53.3186
BFU-E	E11.5MesP1-On	BFU-E	H-EC+11.5MesP	-56.5337	1.6904	100	-33.44	<.0001	0.05	-59.8874	-53.1800
BFU-E	E11.5MesP1-On	BFU-E	I-EC Only	6.57E-14	.	.	.	.	.	.	.
BFU-E	E11.5MesP1-On	BFU-E	I-EC+10.5MesP	-37.7032	608.44	100	-0.06	0.9507	0.05	-1244.84	1169.43

The GLIMMIX Procedure

Differences of Colony_Typ*Condition Least Squares Means											
Colony_Type	Condition	_Colony_Type	_Condition	Estimate	Standard Error	DF	t Value	Pr > |t|	Alpha	Lower	Upper
BFU-E	E11.5MesP1-On	BFU-E	I-EC+11.5MesP	-37.7251	608.32	100	-0.06	0.9507	0.05	-1244.61	1169.16
BFU-E	E11.5MesP1-On	BFU-E	L-E only	2.88E-13	.	.	.	.	.	.	.
BFU-E	E11.5MesP1-On	BFU-E	L-E+11.5Mesp1	-56.7231	0	100	-Infty	<.0001	.	.	.
BFU-E	E11.5MesP1-On	CFU-GEMM	10.5EC+!0.5Me	-52.9636	1.5111	100	-35.05	<.0001	0.05	-55.9615	-49.9657
BFU-E	E11.5MesP1-On	CFU-GEMM	11.5 EC Only	-37.7364	607.65	100	-0.06	0.9506	0.05	-1243.30	1167.82
BFU-E	E11.5MesP1-On	CFU-GEMM	11.5EC+11.5Me	-52.9016	1.5108	100	-35.02	<.0001	0.05	-55.8990	-49.9042
BFU-E	E11.5MesP1-On	CFU-GEMM	13.5EC+10.5Me	-56.4613	1.6898	100	-33.41	<.0001	0.05	-59.8138	-53.1089
BFU-E	E11.5MesP1-On	CFU-GEMM	13.5EC+11.5Me	-56.0834	1.6900	100	-33.18	<.0001	0.05	-59.4364	-52.7304
BFU-E	E11.5MesP1-On	CFU-GEMM	Ao-EC Only	-0.00210	0.06423	100	-0.03	0.9740	0.05	-0.1295	0.1253
BFU-E	E11.5MesP1-On	CFU-GEMM	AoEC+10.5MesP	-56.1022	1.6900	100	-33.20	<.0001	0.05	-59.4551	-52.7493
BFU-E	E11.5MesP1-On	CFU-GEMM	AoEC+11.5MesP	-56.2631	1.6898	100	-33.29	<.0001	0.05	-59.6157	-52.9105
BFU-E	E11.5MesP1-On	CFU-GEMM	E10.5-EC Only	-0.00210	0.06423	100	-0.03	0.9740	0.05	-0.1295	0.1253
BFU-E	E11.5MesP1-On	CFU-GEMM	E10.5-MesP1+	-0.00210	0.06423	100	-0.03	0.9740	0.05	-0.1295	0.1253
BFU-E	E11.5MesP1-On	CFU-GEMM	E10.5-MesP1-	-0.00210	0.06423	100	-0.03	0.9740	0.05	-0.1295	0.1253
BFU-E	E11.5MesP1-On	CFU-GEMM	E11.5MesP1+On	-0.00210	0.06423	100	-0.03	0.9740	0.05	-0.1295	0.1253
BFU-E	E11.5MesP1-On	CFU-GEMM	E11.5MesP1-On	-0.00210	0.06423	100	-0.03	0.9740	0.05	-0.1295	0.1253
BFU-E	E11.5MesP1-On	CFU-GEMM	E13.5 EC Only	-0.00210	0.06423	100	-0.03	0.9740	0.05	-0.1295	0.1253
BFU-E	E11.5MesP1-On	CFU-GEMM	H-EC Only	-0.00210	0.06423	100	-0.03	0.9740	0.05	-0.1295	0.1253
BFU-E	E11.5MesP1-On	CFU-GEMM	H-EC+10.5MesP	-56.0336	1.6901	100	-33.15	<.0001	0.05	-59.3867	-52.6805
BFU-E	E11.5MesP1-On	CFU-GEMM	H-EC+11.5MesP	-55.9250	1.6903	100	-33.09	<.0001	0.05	-59.2784	-52.5715
BFU-E	E11.5MesP1-On	CFU-GEMM	I-EC Only	-0.00210	0.06423	100	-0.03	0.9740	0.05	-0.1295	0.1253
BFU-E	E11.5MesP1-On	CFU-GEMM	I-EC+10.5MesP	-54.7445	1.6927	100	-32.34	<.0001	0.05	-58.1027	-51.3863
BFU-E	E11.5MesP1-On	CFU-GEMM	I-EC+11.5MesP	-54.0123	1.6976	100	-31.82	<.0001	0.05	-57.3803	-50.6443
BFU-E	E11.5MesP1-On	CFU-GEMM	L-E only	-0.00210	0.06423	100	-0.03	0.9740	0.05	-0.1295	0.1253

The GLIMMIX Procedure

Differences of Colony_Typ*Condition Least Squares Means											
Colony_Type	Condition	_Colony_Type	_Condition	Estimate	Standard Error	DF	t Value	Pr > |t|	Alpha	Lower	Upper
BFU-E	E11.5MesP1-On	CFU-GEMM	L-E+11.5Mesp1	-56.2008	0.06423	100	-874.93	<.0001	0.05	-56.3282	-56.0733
BFU-E	E11.5MesP1-On	CFU-GM	10.5EC+!0.5Me	-54.4663	1.5102	100	-36.07	<.0001	0.05	-57.4625	-51.4700
BFU-E	E11.5MesP1-On	CFU-GM	11.5 EC Only	-54.9123	1.6924	100	-32.45	<.0001	0.05	-58.2701	-51.5546
BFU-E	E11.5MesP1-On	CFU-GM	11.5EC+11.5Me	-54.5162	1.5100	100	-36.10	<.0001	0.05	-57.5119	-51.5205
BFU-E	E11.5MesP1-On	CFU-GM	13.5EC+10.5Me	-57.7442	1.6887	100	-34.19	<.0001	0.05	-61.0946	-54.3938
BFU-E	E11.5MesP1-On	CFU-GM	13.5EC+11.5Me	-58.0477	1.6887	100	-34.37	<.0001	0.05	-61.3980	-54.6974
BFU-E	E11.5MesP1-On	CFU-GM	Ao-EC Only	-11.3658	0	100	-Infty	<.0001	.	.	.
BFU-E	E11.5MesP1-On	CFU-GM	AoEC+10.5MesP	-58.1017	1.6887	100	-34.41	<.0001	0.05	-61.4519	-54.7514
BFU-E	E11.5MesP1-On	CFU-GM	AoEC+11.5MesP	-58.0689	1.6887	100	-34.39	<.0001	0.05	-61.4192	-54.7186
BFU-E	E11.5MesP1-On	CFU-GM	E10.5-EC Only	-11.3658	0	100	-Infty	<.0001	.	.	.
BFU-E	E11.5MesP1-On	CFU-GM	E10.5-MesP1+	-11.3658	0	100	-Infty	<.0001	.	.	.
BFU-E	E11.5MesP1-On	CFU-GM	E10.5-MesP1-	-11.3658	0	100	-Infty	<.0001	.	.	.
BFU-E	E11.5MesP1-On	CFU-GM	E11.5MesP1+On	-11.3658	0	100	-Infty	<.0001	.	.	.
BFU-E	E11.5MesP1-On	CFU-GM	E11.5MesP1-On	-11.3658	0	100	-Infty	<.0001	.	.	.
BFU-E	E11.5MesP1-On	CFU-GM	E13.5 EC Only	-11.3658	0	100	-Infty	<.0001	.	.	.
BFU-E	E11.5MesP1-On	CFU-GM	H-EC Only	-11.3658	0	100	-Infty	<.0001	.	.	.
BFU-E	E11.5MesP1-On	CFU-GM	H-EC+10.5MesP	-58.0833	1.6887	100	-34.40	<.0001	0.05	-61.4336	-54.7330
BFU-E	E11.5MesP1-On	CFU-GM	H-EC+11.5MesP	-57.9870	1.6887	100	-34.34	<.0001	0.05	-61.3373	-54.6367
BFU-E	E11.5MesP1-On	CFU-GM	I-EC Only	-11.3658	0	100	-Infty	<.0001	.	.	.
BFU-E	E11.5MesP1-On	CFU-GM	I-EC+10.5MesP	-55.5684	1.6905	100	-32.87	<.0001	0.05	-58.9224	-52.2144
BFU-E	E11.5MesP1-On	CFU-GM	I-EC+11.5MesP	-54.8774	1.6926	100	-32.42	<.0001	0.05	-58.2354	-51.5194
BFU-E	E11.5MesP1-On	CFU-GM	L-E only	-11.3658	0	100	-Infty	<.0001	.	.	.
BFU-E	E11.5MesP1-On	CFU-GM	L-E+11.5Mesp1	-58.1462	0	100	-Infty	<.0001	.	.	.
BFU-E	E13.5 EC Only	BFU-E	H-EC Only	-906E-16	.	.	.	.	.	.	.

The GLIMMIX Procedure

Differences of Colony_Typ*Condition Least Squares Means											
Colony_Type	Condition	_Colony_Type	_Condition	Estimate	Standard Error	DF	t Value	Pr > |t|	Alpha	Lower	Upper
BFU-E	E13.5 EC Only	BFU-E	H-EC+10.5MesP	-56.6721	1.6903	100	-33.53	<.0001	0.05	-60.0255	-53.3186
BFU-E	E13.5 EC Only	BFU-E	H-EC+11.5MesP	-56.5337	1.6904	100	-33.44	<.0001	0.05	-59.8874	-53.1800
BFU-E	E13.5 EC Only	BFU-E	I-EC Only	2.06E-13	.	.	.	.	.	.	.
BFU-E	E13.5 EC Only	BFU-E	I-EC+10.5MesP	-37.7032	608.44	100	-0.06	0.9507	0.05	-1244.84	1169.43
BFU-E	E13.5 EC Only	BFU-E	I-EC+11.5MesP	-37.7251	608.32	100	-0.06	0.9507	0.05	-1244.61	1169.16
BFU-E	E13.5 EC Only	BFU-E	L-E only	4.28E-13	.	.	.	.	.	.	.
BFU-E	E13.5 EC Only	BFU-E	L-E+11.5Mesp1	-56.7231	0	100	-Infty	<.0001	.	.	.
BFU-E	E13.5 EC Only	CFU-GEMM	10.5EC+!0.5Me	-52.9636	1.5111	100	-35.05	<.0001	0.05	-55.9615	-49.9657
BFU-E	E13.5 EC Only	CFU-GEMM	11.5 EC Only	-37.7364	607.65	100	-0.06	0.9506	0.05	-1243.30	1167.82
BFU-E	E13.5 EC Only	CFU-GEMM	11.5EC+11.5Me	-52.9016	1.5108	100	-35.02	<.0001	0.05	-55.8990	-49.9042
BFU-E	E13.5 EC Only	CFU-GEMM	13.5EC+10.5Me	-56.4613	1.6898	100	-33.41	<.0001	0.05	-59.8138	-53.1089
BFU-E	E13.5 EC Only	CFU-GEMM	13.5EC+11.5Me	-56.0834	1.6900	100	-33.18	<.0001	0.05	-59.4364	-52.7304
BFU-E	E13.5 EC Only	CFU-GEMM	Ao-EC Only	-0.00210	0.06423	100	-0.03	0.9740	0.05	-0.1295	0.1253
BFU-E	E13.5 EC Only	CFU-GEMM	AoEC+10.5MesP	-56.1022	1.6900	100	-33.20	<.0001	0.05	-59.4551	-52.7493
BFU-E	E13.5 EC Only	CFU-GEMM	AoEC+11.5MesP	-56.2631	1.6898	100	-33.29	<.0001	0.05	-59.6157	-52.9105
BFU-E	E13.5 EC Only	CFU-GEMM	E10.5-EC Only	-0.00210	0.06423	100	-0.03	0.9740	0.05	-0.1295	0.1253
BFU-E	E13.5 EC Only	CFU-GEMM	E10.5-MesP1+	-0.00210	0.06423	100	-0.03	0.9740	0.05	-0.1295	0.1253
BFU-E	E13.5 EC Only	CFU-GEMM	E10.5-MesP1-	-0.00210	0.06423	100	-0.03	0.9740	0.05	-0.1295	0.1253
BFU-E	E13.5 EC Only	CFU-GEMM	E11.5MesP1+On	-0.00210	0.06423	100	-0.03	0.9740	0.05	-0.1295	0.1253
BFU-E	E13.5 EC Only	CFU-GEMM	E11.5MesP1-On	-0.00210	0.06423	100	-0.03	0.9740	0.05	-0.1295	0.1253
BFU-E	E13.5 EC Only	CFU-GEMM	E13.5 EC Only	-0.00210	0.06423	100	-0.03	0.9740	0.05	-0.1295	0.1253
BFU-E	E13.5 EC Only	CFU-GEMM	H-EC Only	-0.00210	0.06423	100	-0.03	0.9740	0.05	-0.1295	0.1253
BFU-E	E13.5 EC Only	CFU-GEMM	H-EC+10.5MesP	-56.0336	1.6901	100	-33.15	<.0001	0.05	-59.3867	-52.6805
BFU-E	E13.5 EC Only	CFU-GEMM	H-EC+11.5MesP	-55.9250	1.6903	100	-33.09	<.0001	0.05	-59.2784	-52.5715

The GLIMMIX Procedure

Differences of Colony_Typ*Condition Least Squares Means											
Colony_Type	Condition	_Colony_Type	_Condition	Estimate	Standard Error	DF	t Value	Pr > |t|	Alpha	Lower	Upper
BFU-E	E13.5 EC Only	CFU-GEMM	I-EC Only	-0.00210	0.06423	100	-0.03	0.9740	0.05	-0.1295	0.1253
BFU-E	E13.5 EC Only	CFU-GEMM	I-EC+10.5MesP	-54.7445	1.6927	100	-32.34	<.0001	0.05	-58.1027	-51.3863
BFU-E	E13.5 EC Only	CFU-GEMM	I-EC+11.5MesP	-54.0123	1.6976	100	-31.82	<.0001	0.05	-57.3803	-50.6443
BFU-E	E13.5 EC Only	CFU-GEMM	L-E only	-0.00210	0.06423	100	-0.03	0.9740	0.05	-0.1295	0.1253
BFU-E	E13.5 EC Only	CFU-GEMM	L-E+11.5Mesp1	-56.2008	0.06423	100	-874.93	<.0001	0.05	-56.3282	-56.0733
BFU-E	E13.5 EC Only	CFU-GM	10.5EC+!0.5Me	-54.4663	1.5102	100	-36.07	<.0001	0.05	-57.4625	-51.4700
BFU-E	E13.5 EC Only	CFU-GM	11.5 EC Only	-54.9123	1.6924	100	-32.45	<.0001	0.05	-58.2701	-51.5546
BFU-E	E13.5 EC Only	CFU-GM	11.5EC+11.5Me	-54.5162	1.5100	100	-36.10	<.0001	0.05	-57.5119	-51.5205
BFU-E	E13.5 EC Only	CFU-GM	13.5EC+10.5Me	-57.7442	1.6887	100	-34.19	<.0001	0.05	-61.0946	-54.3938
BFU-E	E13.5 EC Only	CFU-GM	13.5EC+11.5Me	-58.0477	1.6887	100	-34.37	<.0001	0.05	-61.3980	-54.6974
BFU-E	E13.5 EC Only	CFU-GM	Ao-EC Only	-11.3658	0	100	-Infty	<.0001	.	.	.
BFU-E	E13.5 EC Only	CFU-GM	AoEC+10.5MesP	-58.1017	1.6887	100	-34.41	<.0001	0.05	-61.4519	-54.7514
BFU-E	E13.5 EC Only	CFU-GM	AoEC+11.5MesP	-58.0689	1.6887	100	-34.39	<.0001	0.05	-61.4192	-54.7186
BFU-E	E13.5 EC Only	CFU-GM	E10.5-EC Only	-11.3658	0	100	-Infty	<.0001	.	.	.
BFU-E	E13.5 EC Only	CFU-GM	E10.5-MesP1+	-11.3658	0	100	-Infty	<.0001	.	.	.
BFU-E	E13.5 EC Only	CFU-GM	E10.5-MesP1-	-11.3658	0	100	-Infty	<.0001	.	.	.
BFU-E	E13.5 EC Only	CFU-GM	E11.5MesP1+On	-11.3658	0	100	-Infty	<.0001	.	.	.
BFU-E	E13.5 EC Only	CFU-GM	E11.5MesP1-On	-11.3658	0	100	-Infty	<.0001	.	.	.
BFU-E	E13.5 EC Only	CFU-GM	E13.5 EC Only	-11.3658	0	100	-Infty	<.0001	.	.	.
BFU-E	E13.5 EC Only	CFU-GM	H-EC Only	-11.3658	0	100	-Infty	<.0001	.	.	.
BFU-E	E13.5 EC Only	CFU-GM	H-EC+10.5MesP	-58.0833	1.6887	100	-34.40	<.0001	0.05	-61.4336	-54.7330
BFU-E	E13.5 EC Only	CFU-GM	H-EC+11.5MesP	-57.9870	1.6887	100	-34.34	<.0001	0.05	-61.3373	-54.6367
BFU-E	E13.5 EC Only	CFU-GM	I-EC Only	-11.3658	0	100	-Infty	<.0001	.	.	.
BFU-E	E13.5 EC Only	CFU-GM	I-EC+10.5MesP	-55.5684	1.6905	100	-32.87	<.0001	0.05	-58.9224	-52.2144

The GLIMMIX Procedure

Differences of Colony_Typ*Condition Least Squares Means											
Colony_Type	Condition	_Colony_Type	_Condition	Estimate	Standard Error	DF	t Value	Pr > |t|	Alpha	Lower	Upper
BFU-E	E13.5 EC Only	CFU-GM	I-EC+11.5MesP	-54.8774	1.6926	100	-32.42	<.0001	0.05	-58.2354	-51.5194
BFU-E	E13.5 EC Only	CFU-GM	L-E only	-11.3658	0	100	-Infty	<.0001	.	.	.
BFU-E	E13.5 EC Only	CFU-GM	L-E+11.5Mesp1	-58.1462	0	100	-Infty	<.0001	.	.	.
BFU-E	H-EC Only	BFU-E	H-EC+10.5MesP	-56.6721	1.6903	100	-33.53	<.0001	0.05	-60.0255	-53.3186
BFU-E	H-EC Only	BFU-E	H-EC+11.5MesP	-56.5337	1.6904	100	-33.44	<.0001	0.05	-59.8874	-53.1800
BFU-E	H-EC Only	BFU-E	I-EC Only	2.97E-13	.	.	.	.	.	.	.
BFU-E	H-EC Only	BFU-E	I-EC+10.5MesP	-37.7032	608.44	100	-0.06	0.9507	0.05	-1244.84	1169.43
BFU-E	H-EC Only	BFU-E	I-EC+11.5MesP	-37.7251	608.32	100	-0.06	0.9507	0.05	-1244.61	1169.16
BFU-E	H-EC Only	BFU-E	L-E only	5.19E-13	.	.	.	.	.	.	.
BFU-E	H-EC Only	BFU-E	L-E+11.5Mesp1	-56.7231	0	100	-Infty	<.0001	.	.	.
BFU-E	H-EC Only	CFU-GEMM	10.5EC+!0.5Me	-52.9636	1.5111	100	-35.05	<.0001	0.05	-55.9615	-49.9657
BFU-E	H-EC Only	CFU-GEMM	11.5 EC Only	-37.7364	607.65	100	-0.06	0.9506	0.05	-1243.30	1167.82
BFU-E	H-EC Only	CFU-GEMM	11.5EC+11.5Me	-52.9016	1.5108	100	-35.02	<.0001	0.05	-55.8990	-49.9042
BFU-E	H-EC Only	CFU-GEMM	13.5EC+10.5Me	-56.4613	1.6898	100	-33.41	<.0001	0.05	-59.8138	-53.1089
BFU-E	H-EC Only	CFU-GEMM	13.5EC+11.5Me	-56.0834	1.6900	100	-33.18	<.0001	0.05	-59.4364	-52.7304
BFU-E	H-EC Only	CFU-GEMM	Ao-EC Only	-0.00210	0.06423	100	-0.03	0.9740	0.05	-0.1295	0.1253
BFU-E	H-EC Only	CFU-GEMM	AoEC+10.5MesP	-56.1022	1.6900	100	-33.20	<.0001	0.05	-59.4551	-52.7493
BFU-E	H-EC Only	CFU-GEMM	AoEC+11.5MesP	-56.2631	1.6898	100	-33.29	<.0001	0.05	-59.6157	-52.9105
BFU-E	H-EC Only	CFU-GEMM	E10.5-EC Only	-0.00210	0.06423	100	-0.03	0.9740	0.05	-0.1295	0.1253
BFU-E	H-EC Only	CFU-GEMM	E10.5-MesP1+	-0.00210	0.06423	100	-0.03	0.9740	0.05	-0.1295	0.1253
BFU-E	H-EC Only	CFU-GEMM	E10.5-MesP1-	-0.00210	0.06423	100	-0.03	0.9740	0.05	-0.1295	0.1253
BFU-E	H-EC Only	CFU-GEMM	E11.5MesP1+On	-0.00210	0.06423	100	-0.03	0.9740	0.05	-0.1295	0.1253
BFU-E	H-EC Only	CFU-GEMM	E11.5MesP1-On	-0.00210	0.06423	100	-0.03	0.9740	0.05	-0.1295	0.1253
BFU-E	H-EC Only	CFU-GEMM	E13.5 EC Only	-0.00210	0.06423	100	-0.03	0.9740	0.05	-0.1295	0.1253

The GLIMMIX Procedure

Differences of Colony_Typ*Condition Least Squares Means											
Colony_Type	Condition	_Colony_Type	_Condition	Estimate	Standard Error	DF	t Value	Pr > |t|	Alpha	Lower	Upper
BFU-E	H-EC Only	CFU-GEMM	H-EC Only	-0.00210	0.06423	100	-0.03	0.9740	0.05	-0.1295	0.1253
BFU-E	H-EC Only	CFU-GEMM	H-EC+10.5MesP	-56.0336	1.6901	100	-33.15	<.0001	0.05	-59.3867	-52.6805
BFU-E	H-EC Only	CFU-GEMM	H-EC+11.5MesP	-55.9250	1.6903	100	-33.09	<.0001	0.05	-59.2784	-52.5715
BFU-E	H-EC Only	CFU-GEMM	I-EC Only	-0.00210	0.06423	100	-0.03	0.9740	0.05	-0.1295	0.1253
BFU-E	H-EC Only	CFU-GEMM	I-EC+10.5MesP	-54.7445	1.6927	100	-32.34	<.0001	0.05	-58.1027	-51.3863
BFU-E	H-EC Only	CFU-GEMM	I-EC+11.5MesP	-54.0123	1.6976	100	-31.82	<.0001	0.05	-57.3803	-50.6443
BFU-E	H-EC Only	CFU-GEMM	L-E only	-0.00210	0.06423	100	-0.03	0.9740	0.05	-0.1295	0.1253
BFU-E	H-EC Only	CFU-GEMM	L-E+11.5Mesp1	-56.2008	0.06423	100	-874.93	<.0001	0.05	-56.3282	-56.0733
BFU-E	H-EC Only	CFU-GM	10.5EC+!0.5Me	-54.4663	1.5102	100	-36.07	<.0001	0.05	-57.4625	-51.4700
BFU-E	H-EC Only	CFU-GM	11.5 EC Only	-54.9123	1.6924	100	-32.45	<.0001	0.05	-58.2701	-51.5546
BFU-E	H-EC Only	CFU-GM	11.5EC+11.5Me	-54.5162	1.5100	100	-36.10	<.0001	0.05	-57.5119	-51.5205
BFU-E	H-EC Only	CFU-GM	13.5EC+10.5Me	-57.7442	1.6887	100	-34.19	<.0001	0.05	-61.0946	-54.3938
BFU-E	H-EC Only	CFU-GM	13.5EC+11.5Me	-58.0477	1.6887	100	-34.37	<.0001	0.05	-61.3980	-54.6974
BFU-E	H-EC Only	CFU-GM	Ao-EC Only	-11.3658	0	100	-Infty	<.0001	.	.	.
BFU-E	H-EC Only	CFU-GM	AoEC+10.5MesP	-58.1017	1.6887	100	-34.41	<.0001	0.05	-61.4519	-54.7514
BFU-E	H-EC Only	CFU-GM	AoEC+11.5MesP	-58.0689	1.6887	100	-34.39	<.0001	0.05	-61.4192	-54.7186
BFU-E	H-EC Only	CFU-GM	E10.5-EC Only	-11.3658	0	100	-Infty	<.0001	.	.	.
BFU-E	H-EC Only	CFU-GM	E10.5-MesP1+	-11.3658	0	100	-Infty	<.0001	.	.	.
BFU-E	H-EC Only	CFU-GM	E10.5-MesP1-	-11.3658	0	100	-Infty	<.0001	.	.	.
BFU-E	H-EC Only	CFU-GM	E11.5MesP1+On	-11.3658	0	100	-Infty	<.0001	.	.	.
BFU-E	H-EC Only	CFU-GM	E11.5MesP1-On	-11.3658	0	100	-Infty	<.0001	.	.	.
BFU-E	H-EC Only	CFU-GM	E13.5 EC Only	-11.3658	0	100	-Infty	<.0001	.	.	.
BFU-E	H-EC Only	CFU-GM	H-EC Only	-11.3658	0	100	-Infty	<.0001	.	.	.
BFU-E	H-EC Only	CFU-GM	H-EC+10.5MesP	-58.0833	1.6887	100	-34.40	<.0001	0.05	-61.4336	-54.7330

The GLIMMIX Procedure

Differences of Colony_Typ*Condition Least Squares Means

Colony_Type	Condition	_Colony_Type	_Condition	Estimate	Standard Error	DF	t Value	Pr > |t|	Alpha	Lower	Upper
BFU-E	H-EC Only	CFU-GM	H-EC+11.5MesP	-57.9870	1.6887	100	-34.34	<.0001	0.05	-61.3373	-54.6367
BFU-E	H-EC Only	CFU-GM	I-EC Only	-11.3658	0	100	-Infty	<.0001	.	.	.
BFU-E	H-EC Only	CFU-GM	I-EC+10.5MesP	-55.5684	1.6905	100	-32.87	<.0001	0.05	-58.9224	-52.2144
BFU-E	H-EC Only	CFU-GM	I-EC+11.5MesP	-54.8774	1.6926	100	-32.42	<.0001	0.05	-58.2354	-51.5194
BFU-E	H-EC Only	CFU-GM	L-E only	-11.3658	0	100	-Infty	<.0001	.	.	.
BFU-E	H-EC Only	CFU-GM	L-E+11.5Mesp1	-58.1462	0	100	-Infty	<.0001	.	.	.
BFU-E	H-EC+10.5MesP	BFU-E	H-EC+11.5MesP	0.1384	1.6920	100	0.08	0.9350	0.05	-3.2186	3.4953
BFU-E	H-EC+10.5MesP	BFU-E	I-EC Only	56.6721	1.6903	100	33.53	<.0001	0.05	53.3186	60.0255
BFU-E	H-EC+10.5MesP	BFU-E	I-EC+10.5MesP	18.9689	608.44	100	0.03	0.9752	0.05	-1188.17	1226.11
BFU-E	H-EC+10.5MesP	BFU-E	I-EC+11.5MesP	18.9469	608.32	100	0.03	0.9752	0.05	-1187.93	1225.83
BFU-E	H-EC+10.5MesP	BFU-E	L-E only	56.6721	1.6903	100	33.53	<.0001	0.05	53.3186	60.0255
BFU-E	H-EC+10.5MesP	BFU-E	L-E+11.5Mesp1	-0.05102	1.6903	100	-0.03	0.9760	0.05	-3.4045	3.3025
BFU-E	H-EC+10.5MesP	CFU-GEMM	10.5EC+!0.5Me	3.7085	1.5128	100	2.45	0.0160	0.05	0.7071	6.7099
BFU-E	H-EC+10.5MesP	CFU-GEMM	11.5 EC Only	18.9357	607.65	100	0.03	0.9752	0.05	-1186.62	1224.50
BFU-E	H-EC+10.5MesP	CFU-GEMM	11.5EC+11.5Me	3.7704	1.5126	100	2.49	0.0143	0.05	0.7695	6.7714
BFU-E	H-EC+10.5MesP	CFU-GEMM	13.5EC+10.5Me	0.2107	1.6914	100	0.12	0.9011	0.05	-3.1450	3.5664
BFU-E	H-EC+10.5MesP	CFU-GEMM	13.5EC+11.5Me	0.5886	1.6917	100	0.35	0.7286	0.05	-2.7676	3.9448
BFU-E	H-EC+10.5MesP	CFU-GEMM	Ao-EC Only	56.6700	1.6915	100	33.50	<.0001	0.05	53.3141	60.0259
BFU-E	H-EC+10.5MesP	CFU-GEMM	AoEC+10.5MesP	0.5698	1.6916	100	0.34	0.7369	0.05	-2.7863	3.9260
BFU-E	H-EC+10.5MesP	CFU-GEMM	AoEC+11.5MesP	0.4089	1.6915	100	0.24	0.8095	0.05	-2.9469	3.7647
BFU-E	H-EC+10.5MesP	CFU-GEMM	E10.5-EC Only	56.6700	1.6915	100	33.50	<.0001	0.05	53.3141	60.0259
BFU-E	H-EC+10.5MesP	CFU-GEMM	E10.5-MesP1+	56.6700	1.6915	100	33.50	<.0001	0.05	53.3141	60.0259
BFU-E	H-EC+10.5MesP	CFU-GEMM	E10.5-MesP1-	56.6700	1.6915	100	33.50	<.0001	0.05	53.3141	60.0259
BFU-E	H-EC+10.5MesP	CFU-GEMM	E11.5MesP1+On	56.6700	1.6915	100	33.50	<.0001	0.05	53.3141	60.0259

The GLIMMIX Procedure

Differences of Colony_Typ*Condition Least Squares Means											
Colony_Type	Condition	_Colony_Type	_Condition	Estimate	Standard Error	DF	t Value	Pr > |t|	Alpha	Lower	Upper
BFU-E	H-EC+10.5MesP	CFU-GEMM	E11.5MesP1-On	56.6700	1.6915	100	33.50	<.0001	0.05	53.3141	60.0259
BFU-E	H-EC+10.5MesP	CFU-GEMM	E13.5 EC Only	56.6700	1.6915	100	33.50	<.0001	0.05	53.3141	60.0259
BFU-E	H-EC+10.5MesP	CFU-GEMM	H-EC Only	56.6700	1.6915	100	33.50	<.0001	0.05	53.3141	60.0259
BFU-E	H-EC+10.5MesP	CFU-GEMM	H-EC+10.5MesP	0.6385	0.1013	100	6.30	<.0001	0.05	0.4376	0.8394
BFU-E	H-EC+10.5MesP	CFU-GEMM	H-EC+11.5MesP	0.7471	1.6919	100	0.44	0.6598	0.05	-2.6096	4.1038
BFU-E	H-EC+10.5MesP	CFU-GEMM	I-EC Only	56.6700	1.6915	100	33.50	<.0001	0.05	53.3141	60.0259
BFU-E	H-EC+10.5MesP	CFU-GEMM	I-EC+10.5MesP	1.9276	1.6943	100	1.14	0.2580	0.05	-1.4339	5.2890
BFU-E	H-EC+10.5MesP	CFU-GEMM	I-EC+11.5MesP	2.6598	1.6992	100	1.57	0.1207	0.05	-0.7114	6.0310
BFU-E	H-EC+10.5MesP	CFU-GEMM	L-E only	56.6700	1.6915	100	33.50	<.0001	0.05	53.3141	60.0259
BFU-E	H-EC+10.5MesP	CFU-GEMM	L-E+11.5Mesp1	0.4713	1.6915	100	0.28	0.7811	0.05	-2.8846	3.8272
BFU-E	H-EC+10.5MesP	CFU-GM	10.5EC+!0.5Me	2.2058	1.5120	100	1.46	0.1477	0.05	-0.7940	5.2056
BFU-E	H-EC+10.5MesP	CFU-GM	11.5 EC Only	1.7597	1.6941	100	1.04	0.3014	0.05	-1.6012	5.1207
BFU-E	H-EC+10.5MesP	CFU-GM	11.5EC+11.5Me	2.1558	1.5117	100	1.43	0.1570	0.05	-0.8434	5.1551
BFU-E	H-EC+10.5MesP	CFU-GM	13.5EC+10.5Me	-1.0722	1.6904	100	-0.63	0.5273	0.05	-4.4258	2.2814
BFU-E	H-EC+10.5MesP	CFU-GM	13.5EC+11.5Me	-1.3756	1.6903	100	-0.81	0.4177	0.05	-4.7291	1.9779
BFU-E	H-EC+10.5MesP	CFU-GM	Ao-EC Only	45.3063	1.6903	100	26.80	<.0001	0.05	41.9528	48.6597
BFU-E	H-EC+10.5MesP	CFU-GM	AoEC+10.5MesP	-1.4296	1.6903	100	-0.85	0.3997	0.05	-4.7831	1.9239
BFU-E	H-EC+10.5MesP	CFU-GM	AoEC+11.5MesP	-1.3968	1.6903	100	-0.83	0.4105	0.05	-4.7503	1.9566
BFU-E	H-EC+10.5MesP	CFU-GM	E10.5-EC Only	45.3063	1.6903	100	26.80	<.0001	0.05	41.9528	48.6597
BFU-E	H-EC+10.5MesP	CFU-GM	E10.5-MesP1+	45.3063	1.6903	100	26.80	<.0001	0.05	41.9528	48.6597
BFU-E	H-EC+10.5MesP	CFU-GM	E10.5-MesP1-	45.3063	1.6903	100	26.80	<.0001	0.05	41.9528	48.6597
BFU-E	H-EC+10.5MesP	CFU-GM	E11.5MesP1+On	45.3063	1.6903	100	26.80	<.0001	0.05	41.9528	48.6597
BFU-E	H-EC+10.5MesP	CFU-GM	E11.5MesP1-On	45.3063	1.6903	100	26.80	<.0001	0.05	41.9528	48.6597
BFU-E	H-EC+10.5MesP	CFU-GM	E13.5 EC Only	45.3063	1.6903	100	26.80	<.0001	0.05	41.9528	48.6597

The GLIMMIX Procedure

Differences of Colony_Typ*Condition Least Squares Means											
Colony_Type	Condition	_Colony_Type	_Condition	Estimate	Standard Error	DF	t Value	Pr > |t|	Alpha	Lower	Upper
BFU-E	H-EC+10.5MesP	CFU-GM	H-EC Only	45.3063	1.6903	100	26.80	<.0001	0.05	41.9528	48.6597
BFU-E	H-EC+10.5MesP	CFU-GM	H-EC+10.5MesP	-1.4113	0.07384	100	-19.11	<.0001	0.05	-1.5578	-1.2648
BFU-E	H-EC+10.5MesP	CFU-GM	H-EC+11.5MesP	-1.3149	1.6903	100	-0.78	0.4385	0.05	-4.6685	2.0386
BFU-E	H-EC+10.5MesP	CFU-GM	I-EC Only	45.3063	1.6903	100	26.80	<.0001	0.05	41.9528	48.6597
BFU-E	H-EC+10.5MesP	CFU-GM	I-EC+10.5MesP	1.1036	1.6922	100	0.65	0.5158	0.05	-2.2536	4.4608
BFU-E	H-EC+10.5MesP	CFU-GM	I-EC+11.5MesP	1.7947	1.6942	100	1.06	0.2920	0.05	-1.5666	5.1559
BFU-E	H-EC+10.5MesP	CFU-GM	L-E only	45.3063	1.6903	100	26.80	<.0001	0.05	41.9528	48.6597
BFU-E	H-EC+10.5MesP	CFU-GM	L-E+11.5Mesp1	-1.4741	1.6903	100	-0.87	0.3852	0.05	-4.8276	1.8794
BFU-E	H-EC+11.5MesP	BFU-E	I-EC Only	56.5337	1.6904	100	33.44	<.0001	0.05	53.1800	59.8874
BFU-E	H-EC+11.5MesP	BFU-E	I-EC+10.5MesP	18.8305	608.44	100	0.03	0.9754	0.05	-1188.31	1225.97
BFU-E	H-EC+11.5MesP	BFU-E	I-EC+11.5MesP	18.8086	608.32	100	0.03	0.9754	0.05	-1188.07	1225.69
BFU-E	H-EC+11.5MesP	BFU-E	L-E only	56.5337	1.6904	100	33.44	<.0001	0.05	53.1800	59.8874
BFU-E	H-EC+11.5MesP	BFU-E	L-E+11.5Mesp1	-0.1894	1.6904	100	-0.11	0.9110	0.05	-3.5431	3.1644
BFU-E	H-EC+11.5MesP	CFU-GEMM	10.5EC+!0.5Me	3.5701	1.5130	100	2.36	0.0202	0.05	0.5683	6.5719
BFU-E	H-EC+11.5MesP	CFU-GEMM	11.5 EC Only	18.7973	607.65	100	0.03	0.9754	0.05	-1186.76	1224.36
BFU-E	H-EC+11.5MesP	CFU-GEMM	11.5EC+11.5Me	3.6321	1.5128	100	2.40	0.0182	0.05	0.6308	6.6334
BFU-E	H-EC+11.5MesP	CFU-GEMM	13.5EC+10.5Me	0.07237	1.6915	100	0.04	0.9660	0.05	-3.2836	3.4283
BFU-E	H-EC+11.5MesP	CFU-GEMM	13.5EC+11.5Me	0.4503	1.6918	100	0.27	0.7907	0.05	-2.9062	3.8068
BFU-E	H-EC+11.5MesP	CFU-GEMM	Ao-EC Only	56.5316	1.6916	100	33.42	<.0001	0.05	53.1754	59.8878
BFU-E	H-EC+11.5MesP	CFU-GEMM	AoEC+10.5MesP	0.4315	1.6918	100	0.26	0.7992	0.05	-2.9249	3.7879
BFU-E	H-EC+11.5MesP	CFU-GEMM	AoEC+11.5MesP	0.2706	1.6916	100	0.16	0.8732	0.05	-3.0855	3.6266
BFU-E	H-EC+11.5MesP	CFU-GEMM	E10.5-EC Only	56.5316	1.6916	100	33.42	<.0001	0.05	53.1754	59.8878
BFU-E	H-EC+11.5MesP	CFU-GEMM	E10.5-MesP1+	56.5316	1.6916	100	33.42	<.0001	0.05	53.1754	59.8878
BFU-E	H-EC+11.5MesP	CFU-GEMM	E10.5-MesP1-	56.5316	1.6916	100	33.42	<.0001	0.05	53.1754	59.8878

The GLIMMIX Procedure

Differences of Colony_Typ*Condition Least Squares Means											
Colony_Type	Condition	_Colony_Type	_Condition	Estimate	Standard Error	DF	t Value	Pr > |t|	Alpha	Lower	Upper
BFU-E	H-EC+11.5MesP	CFU-GEMM	E11.5MesP1+On	56.5316	1.6916	100	33.42	<.0001	0.05	53.1754	59.8878
BFU-E	H-EC+11.5MesP	CFU-GEMM	E11.5MesP1-On	56.5316	1.6916	100	33.42	<.0001	0.05	53.1754	59.8878
BFU-E	H-EC+11.5MesP	CFU-GEMM	E13.5 EC Only	56.5316	1.6916	100	33.42	<.0001	0.05	53.1754	59.8878
BFU-E	H-EC+11.5MesP	CFU-GEMM	H-EC Only	56.5316	1.6916	100	33.42	<.0001	0.05	53.1754	59.8878
BFU-E	H-EC+11.5MesP	CFU-GEMM	H-EC+10.5MesP	0.5001	1.6918	100	0.30	0.7681	0.05	-2.8564	3.8567
BFU-E	H-EC+11.5MesP	CFU-GEMM	H-EC+11.5MesP	0.6087	0.1059	100	5.75	<.0001	0.05	0.3986	0.8189
BFU-E	H-EC+11.5MesP	CFU-GEMM	I-EC Only	56.5316	1.6916	100	33.42	<.0001	0.05	53.1754	59.8878
BFU-E	H-EC+11.5MesP	CFU-GEMM	I-EC+10.5MesP	1.7892	1.6944	100	1.06	0.2935	0.05	-1.5725	5.1509
BFU-E	H-EC+11.5MesP	CFU-GEMM	I-EC+11.5MesP	2.5214	1.6994	100	1.48	0.1410	0.05	-0.8501	5.8929
BFU-E	H-EC+11.5MesP	CFU-GEMM	L-E only	56.5316	1.6916	100	33.42	<.0001	0.05	53.1754	59.8878
BFU-E	H-EC+11.5MesP	CFU-GEMM	L-E+11.5Mesp1	0.3329	1.6916	100	0.20	0.8444	0.05	-3.0232	3.6891
BFU-E	H-EC+11.5MesP	CFU-GM	10.5EC+!0.5Me	2.0674	1.5122	100	1.37	0.1746	0.05	-0.9327	5.0676
BFU-E	H-EC+11.5MesP	CFU-GM	11.5 EC Only	1.6214	1.6942	100	0.96	0.3409	0.05	-1.7399	4.9826
BFU-E	H-EC+11.5MesP	CFU-GM	11.5EC+11.5Me	2.0175	1.5119	100	1.33	0.1851	0.05	-0.9821	5.0171
BFU-E	H-EC+11.5MesP	CFU-GM	13.5EC+10.5Me	-1.2105	1.6905	100	-0.72	0.4756	0.05	-4.5644	2.1433
BFU-E	H-EC+11.5MesP	CFU-GM	13.5EC+11.5Me	-1.5140	1.6904	100	-0.90	0.3726	0.05	-4.8678	1.8398
BFU-E	H-EC+11.5MesP	CFU-GM	Ao-EC Only	45.1679	1.6904	100	26.72	<.0001	0.05	41.8142	48.5216
BFU-E	H-EC+11.5MesP	CFU-GM	AoEC+10.5MesP	-1.5680	1.6904	100	-0.93	0.3559	0.05	-4.9217	1.7858
BFU-E	H-EC+11.5MesP	CFU-GM	AoEC+11.5MesP	-1.5352	1.6904	100	-0.91	0.3660	0.05	-4.8890	1.8186
BFU-E	H-EC+11.5MesP	CFU-GM	E10.5-EC Only	45.1679	1.6904	100	26.72	<.0001	0.05	41.8142	48.5216
BFU-E	H-EC+11.5MesP	CFU-GM	E10.5-MesP1+	45.1679	1.6904	100	26.72	<.0001	0.05	41.8142	48.5216
BFU-E	H-EC+11.5MesP	CFU-GM	E10.5-MesP1-	45.1679	1.6904	100	26.72	<.0001	0.05	41.8142	48.5216
BFU-E	H-EC+11.5MesP	CFU-GM	E11.5MesP1+On	45.1679	1.6904	100	26.72	<.0001	0.05	41.8142	48.5216
BFU-E	H-EC+11.5MesP	CFU-GM	E11.5MesP1-On	45.1679	1.6904	100	26.72	<.0001	0.05	41.8142	48.5216

The GLIMMIX Procedure

Differences of Colony_Typ*Condition Least Squares Means

Colony_Type	Condition	_Colony_Type	_Condition	Estimate	Standard Error	DF	t Value	Pr > |t|	Alpha	Lower	Upper
BFU-E	H-EC+11.5MesP	CFU-GM	E13.5 EC Only	45.1679	1.6904	100	26.72	<.0001	0.05	41.8142	48.5216
BFU-E	H-EC+11.5MesP	CFU-GM	H-EC Only	45.1679	1.6904	100	26.72	<.0001	0.05	41.8142	48.5216
BFU-E	H-EC+11.5MesP	CFU-GM	H-EC+10.5MesP	-1.5496	1.6904	100	-0.92	0.3615	0.05	-4.9034	1.8041
BFU-E	H-EC+11.5MesP	CFU-GM	H-EC+11.5MesP	-1.4533	0.07651	100	-18.99	<.0001	0.05	-1.6051	-1.3015
BFU-E	H-EC+11.5MesP	CFU-GM	I-EC Only	45.1679	1.6904	100	26.72	<.0001	0.05	41.8142	48.5216
BFU-E	H-EC+11.5MesP	CFU-GM	I-EC+10.5MesP	0.9653	1.6923	100	0.57	0.5697	0.05	-2.3922	4.3227
BFU-E	H-EC+11.5MesP	CFU-GM	I-EC+11.5MesP	1.6563	1.6943	100	0.98	0.3307	0.05	-1.7052	5.0178
BFU-E	H-EC+11.5MesP	CFU-GM	L-E only	45.1679	1.6904	100	26.72	<.0001	0.05	41.8142	48.5216
BFU-E	H-EC+11.5MesP	CFU-GM	L-E+11.5Mesp1	-1.6125	1.6904	100	-0.95	0.3424	0.05	-4.9662	1.7413
BFU-E	I-EC Only	BFU-E	I-EC+10.5MesP	-37.7032	608.44	100	-0.06	0.9507	0.05	-1244.84	1169.43
BFU-E	I-EC Only	BFU-E	I-EC+11.5MesP	-37.7251	608.32	100	-0.06	0.9507	0.05	-1244.61	1169.16
BFU-E	I-EC Only	BFU-E	L-E only	2.22E-13	.	.	.	.	.	.	.
BFU-E	I-EC Only	BFU-E	L-E+11.5Mesp1	-56.7231	0	100	-Infty	<.0001	.	.	.
BFU-E	I-EC Only	CFU-GEMM	10.5EC+!0.5Me	-52.9636	1.5111	100	-35.05	<.0001	0.05	-55.9615	-49.9657
BFU-E	I-EC Only	CFU-GEMM	11.5 EC Only	-37.7364	607.65	100	-0.06	0.9506	0.05	-1243.30	1167.82
BFU-E	I-EC Only	CFU-GEMM	11.5EC+11.5Me	-52.9016	1.5108	100	-35.02	<.0001	0.05	-55.8990	-49.9042
BFU-E	I-EC Only	CFU-GEMM	13.5EC+10.5Me	-56.4613	1.6898	100	-33.41	<.0001	0.05	-59.8138	-53.1089
BFU-E	I-EC Only	CFU-GEMM	13.5EC+11.5Me	-56.0834	1.6900	100	-33.18	<.0001	0.05	-59.4364	-52.7304
BFU-E	I-EC Only	CFU-GEMM	Ao-EC Only	-0.00210	0.06423	100	-0.03	0.9740	0.05	-0.1295	0.1253
BFU-E	I-EC Only	CFU-GEMM	AoEC+10.5MesP	-56.1022	1.6900	100	-33.20	<.0001	0.05	-59.4551	-52.7493
BFU-E	I-EC Only	CFU-GEMM	AoEC+11.5MesP	-56.2631	1.6898	100	-33.29	<.0001	0.05	-59.6157	-52.9105
BFU-E	I-EC Only	CFU-GEMM	E10.5-EC Only	-0.00210	0.06423	100	-0.03	0.9740	0.05	-0.1295	0.1253
BFU-E	I-EC Only	CFU-GEMM	E10.5-MesP1+	-0.00210	0.06423	100	-0.03	0.9740	0.05	-0.1295	0.1253
BFU-E	I-EC Only	CFU-GEMM	E10.5-MesP1-	-0.00210	0.06423	100	-0.03	0.9740	0.05	-0.1295	0.1253

The GLIMMIX Procedure

Differences of Colony_Typ*Condition Least Squares Means											
Colony_Type	Condition	_Colony_Type	_Condition	Estimate	Standard Error	DF	t Value	Pr > |t|	Alpha	Lower	Upper
BFU-E	I-EC Only	CFU-GEMM	E11.5MesP1+On	-0.00210	0.06423	100	-0.03	0.9740	0.05	-0.1295	0.1253
BFU-E	I-EC Only	CFU-GEMM	E11.5MesP1-On	-0.00210	0.06423	100	-0.03	0.9740	0.05	-0.1295	0.1253
BFU-E	I-EC Only	CFU-GEMM	E13.5 EC Only	-0.00210	0.06423	100	-0.03	0.9740	0.05	-0.1295	0.1253
BFU-E	I-EC Only	CFU-GEMM	H-EC Only	-0.00210	0.06423	100	-0.03	0.9740	0.05	-0.1295	0.1253
BFU-E	I-EC Only	CFU-GEMM	H-EC+10.5MesP	-56.0336	1.6901	100	-33.15	<.0001	0.05	-59.3867	-52.6805
BFU-E	I-EC Only	CFU-GEMM	H-EC+11.5MesP	-55.9250	1.6903	100	-33.09	<.0001	0.05	-59.2784	-52.5715
BFU-E	I-EC Only	CFU-GEMM	I-EC Only	-0.00210	0.06423	100	-0.03	0.9740	0.05	-0.1295	0.1253
BFU-E	I-EC Only	CFU-GEMM	I-EC+10.5MesP	-54.7445	1.6927	100	-32.34	<.0001	0.05	-58.1027	-51.3863
BFU-E	I-EC Only	CFU-GEMM	I-EC+11.5MesP	-54.0123	1.6976	100	-31.82	<.0001	0.05	-57.3803	-50.6443
BFU-E	I-EC Only	CFU-GEMM	L-E only	-0.00210	0.06423	100	-0.03	0.9740	0.05	-0.1295	0.1253
BFU-E	I-EC Only	CFU-GEMM	L-E+11.5Mesp1	-56.2008	0.06423	100	-874.93	<.0001	0.05	-56.3282	-56.0733
BFU-E	I-EC Only	CFU-GM	10.5EC+!0.5Me	-54.4663	1.5102	100	-36.07	<.0001	0.05	-57.4625	-51.4700
BFU-E	I-EC Only	CFU-GM	11.5 EC Only	-54.9123	1.6924	100	-32.45	<.0001	0.05	-58.2701	-51.5546
BFU-E	I-EC Only	CFU-GM	11.5EC+11.5Me	-54.5162	1.5100	100	-36.10	<.0001	0.05	-57.5119	-51.5205
BFU-E	I-EC Only	CFU-GM	13.5EC+10.5Me	-57.7442	1.6887	100	-34.19	<.0001	0.05	-61.0946	-54.3938
BFU-E	I-EC Only	CFU-GM	13.5EC+11.5Me	-58.0477	1.6887	100	-34.37	<.0001	0.05	-61.3980	-54.6974
BFU-E	I-EC Only	CFU-GM	Ao-EC Only	-11.3658	0	100	-Infty	<.0001	.	.	.
BFU-E	I-EC Only	CFU-GM	AoEC+10.5MesP	-58.1017	1.6887	100	-34.41	<.0001	0.05	-61.4519	-54.7514
BFU-E	I-EC Only	CFU-GM	AoEC+11.5MesP	-58.0689	1.6887	100	-34.39	<.0001	0.05	-61.4192	-54.7186
BFU-E	I-EC Only	CFU-GM	E10.5-EC Only	-11.3658	0	100	-Infty	<.0001	.	.	.
BFU-E	I-EC Only	CFU-GM	E10.5-MesP1+	-11.3658	0	100	-Infty	<.0001	.	.	.
BFU-E	I-EC Only	CFU-GM	E10.5-MesP1-	-11.3658	0	100	-Infty	<.0001	.	.	.
BFU-E	I-EC Only	CFU-GM	E11.5MesP1+On	-11.3658	0	100	-Infty	<.0001	.	.	.
BFU-E	I-EC Only	CFU-GM	E11.5MesP1-On	-11.3658	0	100	-Infty	<.0001	.	.	.

The GLIMMIX Procedure

Differences of Colony_Typ*Condition Least Squares Means											
Colony_Type	Condition	_Colony_Type	_Condition	Estimate	Standard Error	DF	t Value	Pr > |t|	Alpha	Lower	Upper
BFU-E	I-EC Only	CFU-GM	E13.5 EC Only	-11.3658	0	100	-Infty	<.0001	.	.	.
BFU-E	I-EC Only	CFU-GM	H-EC Only	-11.3658	0	100	-Infty	<.0001	.	.	.
BFU-E	I-EC Only	CFU-GM	H-EC+10.5MesP	-58.0833	1.6887	100	-34.40	<.0001	0.05	-61.4336	-54.7330
BFU-E	I-EC Only	CFU-GM	H-EC+11.5MesP	-57.9870	1.6887	100	-34.34	<.0001	0.05	-61.3373	-54.6367
BFU-E	I-EC Only	CFU-GM	I-EC Only	-11.3658	0	100	-Infty	<.0001	.	.	.
BFU-E	I-EC Only	CFU-GM	I-EC+10.5MesP	-55.5684	1.6905	100	-32.87	<.0001	0.05	-58.9224	-52.2144
BFU-E	I-EC Only	CFU-GM	I-EC+11.5MesP	-54.8774	1.6926	100	-32.42	<.0001	0.05	-58.2354	-51.5194
BFU-E	I-EC Only	CFU-GM	L-E only	-11.3658	0	100	-Infty	<.0001	.	.	.
BFU-E	I-EC Only	CFU-GM	L-E+11.5Mesp1	-58.1462	0	100	-Infty	<.0001	.	.	.
BFU-E	I-EC+10.5MesP	BFU-E	I-EC+11.5MesP	-0.02199	860.38	100	-0.00	1.0000	0.05	-1706.99	1706.94
BFU-E	I-EC+10.5MesP	BFU-E	L-E only	37.7032	608.44	100	0.06	0.9507	0.05	-1169.43	1244.84
BFU-E	I-EC+10.5MesP	BFU-E	L-E+11.5Mesp1	-19.0199	608.44	100	-0.03	0.9751	0.05	-1226.16	1188.12
BFU-E	I-EC+10.5MesP	CFU-GEMM	10.5EC+!0.5Me	-15.2604	608.44	100	-0.03	0.9800	0.05	-1222.40	1191.88
BFU-E	I-EC+10.5MesP	CFU-GEMM	11.5 EC Only	-0.03320	859.91	100	-0.00	1.0000	0.05	-1706.06	1706.00
BFU-E	I-EC+10.5MesP	CFU-GEMM	11.5EC+11.5Me	-15.1985	608.44	100	-0.02	0.9801	0.05	-1222.33	1191.94
BFU-E	I-EC+10.5MesP	CFU-GEMM	13.5EC+10.5Me	-18.7582	608.44	100	-0.03	0.9755	0.05	-1225.90	1188.38
BFU-E	I-EC+10.5MesP	CFU-GEMM	13.5EC+11.5Me	-18.3803	608.44	100	-0.03	0.9760	0.05	-1225.52	1188.76
BFU-E	I-EC+10.5MesP	CFU-GEMM	Ao-EC Only	37.7011	608.44	100	0.06	0.9507	0.05	-1169.44	1244.84
BFU-E	I-EC+10.5MesP	CFU-GEMM	AoEC+10.5MesP	-18.3991	608.44	100	-0.03	0.9759	0.05	-1225.54	1188.74
BFU-E	I-EC+10.5MesP	CFU-GEMM	AoEC+11.5MesP	-18.5600	608.44	100	-0.03	0.9757	0.05	-1225.70	1188.58
BFU-E	I-EC+10.5MesP	CFU-GEMM	E10.5-EC Only	37.7011	608.44	100	0.06	0.9507	0.05	-1169.44	1244.84
BFU-E	I-EC+10.5MesP	CFU-GEMM	E10.5-MesP1+	37.7011	608.44	100	0.06	0.9507	0.05	-1169.44	1244.84
BFU-E	I-EC+10.5MesP	CFU-GEMM	E10.5-MesP1-	37.7011	608.44	100	0.06	0.9507	0.05	-1169.44	1244.84
BFU-E	I-EC+10.5MesP	CFU-GEMM	E11.5MesP1+On	37.7011	608.44	100	0.06	0.9507	0.05	-1169.44	1244.84

The GLIMMIX Procedure

Differences of Colony_Typ*Condition Least Squares Means											
Colony_Type	Condition	_Colony_Type	_Condition	Estimate	Standard Error	DF	t Value	Pr > |t|	Alpha	Lower	Upper
BFU-E	I-EC+10.5MesP	CFU-GEMM	E11.5MesP1-On	37.7011	608.44	100	0.06	0.9507	0.05	-1169.44	1244.84
BFU-E	I-EC+10.5MesP	CFU-GEMM	E13.5 EC Only	37.7011	608.44	100	0.06	0.9507	0.05	-1169.44	1244.84
BFU-E	I-EC+10.5MesP	CFU-GEMM	H-EC Only	37.7011	608.44	100	0.06	0.9507	0.05	-1169.44	1244.84
BFU-E	I-EC+10.5MesP	CFU-GEMM	H-EC+10.5MesP	-18.3304	608.44	100	-0.03	0.9760	0.05	-1225.47	1188.81
BFU-E	I-EC+10.5MesP	CFU-GEMM	H-EC+11.5MesP	-18.2218	608.44	100	-0.03	0.9762	0.05	-1225.36	1188.92
BFU-E	I-EC+10.5MesP	CFU-GEMM	I-EC Only	37.7011	608.44	100	0.06	0.9507	0.05	-1169.44	1244.84
BFU-E	I-EC+10.5MesP	CFU-GEMM	I-EC+10.5MesP	-17.0413	608.44	100	-0.03	0.9777	0.05	-1224.17	1190.09
BFU-E	I-EC+10.5MesP	CFU-GEMM	I-EC+11.5MesP	-16.3091	608.44	100	-0.03	0.9787	0.05	-1223.45	1190.83
BFU-E	I-EC+10.5MesP	CFU-GEMM	L-E only	37.7011	608.44	100	0.06	0.9507	0.05	-1169.44	1244.84
BFU-E	I-EC+10.5MesP	CFU-GEMM	L-E+11.5Mesp1	-18.4976	608.44	100	-0.03	0.9758	0.05	-1225.63	1188.64
BFU-E	I-EC+10.5MesP	CFU-GM	10.5EC+!0.5Me	-16.7631	608.44	100	-0.03	0.9781	0.05	-1223.90	1190.37
BFU-E	I-EC+10.5MesP	CFU-GM	11.5 EC Only	-17.2092	608.44	100	-0.03	0.9775	0.05	-1224.35	1189.93
BFU-E	I-EC+10.5MesP	CFU-GM	11.5EC+11.5Me	-16.8131	608.44	100	-0.03	0.9780	0.05	-1223.95	1190.32
BFU-E	I-EC+10.5MesP	CFU-GM	13.5EC+10.5Me	-20.0411	608.44	100	-0.03	0.9738	0.05	-1227.18	1187.10
BFU-E	I-EC+10.5MesP	CFU-GM	13.5EC+11.5Me	-20.3445	608.44	100	-0.03	0.9734	0.05	-1227.48	1186.79
BFU-E	I-EC+10.5MesP	CFU-GM	Ao-EC Only	26.3374	608.44	100	0.04	0.9656	0.05	-1180.80	1233.47
BFU-E	I-EC+10.5MesP	CFU-GM	AoEC+10.5MesP	-20.3985	608.44	100	-0.03	0.9733	0.05	-1227.54	1186.74
BFU-E	I-EC+10.5MesP	CFU-GM	AoEC+11.5MesP	-20.3657	608.44	100	-0.03	0.9734	0.05	-1227.50	1186.77
BFU-E	I-EC+10.5MesP	CFU-GM	E10.5-EC Only	26.3374	608.44	100	0.04	0.9656	0.05	-1180.80	1233.47
BFU-E	I-EC+10.5MesP	CFU-GM	E10.5-MesP1+	26.3374	608.44	100	0.04	0.9656	0.05	-1180.80	1233.47
BFU-E	I-EC+10.5MesP	CFU-GM	E10.5-MesP1-	26.3374	608.44	100	0.04	0.9656	0.05	-1180.80	1233.47
BFU-E	I-EC+10.5MesP	CFU-GM	E11.5MesP1+On	26.3374	608.44	100	0.04	0.9656	0.05	-1180.80	1233.47
BFU-E	I-EC+10.5MesP	CFU-GM	E11.5MesP1-On	26.3374	608.44	100	0.04	0.9656	0.05	-1180.80	1233.47
BFU-E	I-EC+10.5MesP	CFU-GM	E13.5 EC Only	26.3374	608.44	100	0.04	0.9656	0.05	-1180.80	1233.47

The GLIMMIX Procedure

Differences of Colony_Typ*Condition Least Squares Means											
Colony_Type	Condition	_Colony_Type	_Condition	Estimate	Standard Error	DF	t Value	Pr > |t|	Alpha	Lower	Upper
BFU-E	I-EC+10.5MesP	CFU-GM	H-EC Only	26.3374	608.44	100	0.04	0.9656	0.05	-1180.80	1233.47
BFU-E	I-EC+10.5MesP	CFU-GM	H-EC+10.5MesP	-20.3802	608.44	100	-0.03	0.9733	0.05	-1227.52	1186.76
BFU-E	I-EC+10.5MesP	CFU-GM	H-EC+11.5MesP	-20.2838	608.44	100	-0.03	0.9735	0.05	-1227.42	1186.85
BFU-E	I-EC+10.5MesP	CFU-GM	I-EC Only	26.3374	608.44	100	0.04	0.9656	0.05	-1180.80	1233.47
BFU-E	I-EC+10.5MesP	CFU-GM	I-EC+10.5MesP	-17.8653	608.44	100	-0.03	0.9766	0.05	-1225.00	1189.27
BFU-E	I-EC+10.5MesP	CFU-GM	I-EC+11.5MesP	-17.1742	608.44	100	-0.03	0.9775	0.05	-1224.31	1189.96
BFU-E	I-EC+10.5MesP	CFU-GM	L-E only	26.3374	608.44	100	0.04	0.9656	0.05	-1180.80	1233.47
BFU-E	I-EC+10.5MesP	CFU-GM	L-E+11.5Mesp1	-20.4430	608.44	100	-0.03	0.9733	0.05	-1227.58	1186.69
BFU-E	I-EC+11.5MesP	BFU-E	L-E only	37.7251	608.32	100	0.06	0.9507	0.05	-1169.16	1244.61
BFU-E	I-EC+11.5MesP	BFU-E	L-E+11.5Mesp1	-18.9979	608.32	100	-0.03	0.9751	0.05	-1225.88	1187.88
BFU-E	I-EC+11.5MesP	CFU-GEMM	10.5EC+!0.5Me	-15.2384	608.32	100	-0.03	0.9801	0.05	-1222.12	1191.64
BFU-E	I-EC+11.5MesP	CFU-GEMM	11.5 EC Only	-0.01121	859.82	100	-0.00	1.0000	0.05	-1705.86	1705.84
BFU-E	I-EC+11.5MesP	CFU-GEMM	11.5EC+11.5Me	-15.1765	608.32	100	-0.02	0.9801	0.05	-1222.06	1191.70
BFU-E	I-EC+11.5MesP	CFU-GEMM	13.5EC+10.5Me	-18.7362	608.32	100	-0.03	0.9755	0.05	-1225.62	1188.15
BFU-E	I-EC+11.5MesP	CFU-GEMM	13.5EC+11.5Me	-18.3583	608.32	100	-0.03	0.9760	0.05	-1225.24	1188.52
BFU-E	I-EC+11.5MesP	CFU-GEMM	Ao-EC Only	37.7230	608.32	100	0.06	0.9507	0.05	-1169.16	1244.60
BFU-E	I-EC+11.5MesP	CFU-GEMM	AoEC+10.5MesP	-18.3771	608.32	100	-0.03	0.9760	0.05	-1225.26	1188.50
BFU-E	I-EC+11.5MesP	CFU-GEMM	AoEC+11.5MesP	-18.5380	608.32	100	-0.03	0.9757	0.05	-1225.42	1188.34
BFU-E	I-EC+11.5MesP	CFU-GEMM	E10.5-EC Only	37.7230	608.32	100	0.06	0.9507	0.05	-1169.16	1244.60
BFU-E	I-EC+11.5MesP	CFU-GEMM	E10.5-MesP1+	37.7230	608.32	100	0.06	0.9507	0.05	-1169.16	1244.60
BFU-E	I-EC+11.5MesP	CFU-GEMM	E10.5-MesP1-	37.7230	608.32	100	0.06	0.9507	0.05	-1169.16	1244.60
BFU-E	I-EC+11.5MesP	CFU-GEMM	E11.5MesP1+On	37.7230	608.32	100	0.06	0.9507	0.05	-1169.16	1244.60
BFU-E	I-EC+11.5MesP	CFU-GEMM	E11.5MesP1-On	37.7230	608.32	100	0.06	0.9507	0.05	-1169.16	1244.60
BFU-E	I-EC+11.5MesP	CFU-GEMM	E13.5 EC Only	37.7230	608.32	100	0.06	0.9507	0.05	-1169.16	1244.60

The GLIMMIX Procedure

Differences of Colony_Typ*Condition Least Squares Means											
Colony_Type	Condition	_Colony_Type	_Condition	Estimate	Standard Error	DF	t Value	Pr > |t|	Alpha	Lower	Upper
BFU-E	I-EC+11.5MesP	CFU-GEMM	H-EC Only	37.7230	608.32	100	0.06	0.9507	0.05	-1169.16	1244.60
BFU-E	I-EC+11.5MesP	CFU-GEMM	H-EC+10.5MesP	-18.3084	608.32	100	-0.03	0.9760	0.05	-1225.19	1188.57
BFU-E	I-EC+11.5MesP	CFU-GEMM	H-EC+11.5MesP	-18.1998	608.32	100	-0.03	0.9762	0.05	-1225.08	1188.68
BFU-E	I-EC+11.5MesP	CFU-GEMM	I-EC Only	37.7230	608.32	100	0.06	0.9507	0.05	-1169.16	1244.60
BFU-E	I-EC+11.5MesP	CFU-GEMM	I-EC+10.5MesP	-17.0194	608.32	100	-0.03	0.9777	0.05	-1223.90	1189.86
BFU-E	I-EC+11.5MesP	CFU-GEMM	I-EC+11.5MesP	-16.2871	608.31	100	-0.03	0.9787	0.05	-1223.16	1190.59
BFU-E	I-EC+11.5MesP	CFU-GEMM	L-E only	37.7230	608.32	100	0.06	0.9507	0.05	-1169.16	1244.60
BFU-E	I-EC+11.5MesP	CFU-GEMM	L-E+11.5Mesp1	-18.4756	608.32	100	-0.03	0.9758	0.05	-1225.36	1188.41
BFU-E	I-EC+11.5MesP	CFU-GM	10.5EC+!0.5Me	-16.7411	608.32	100	-0.03	0.9781	0.05	-1223.62	1190.14
BFU-E	I-EC+11.5MesP	CFU-GM	11.5 EC Only	-17.1872	608.32	100	-0.03	0.9775	0.05	-1224.07	1189.69
BFU-E	I-EC+11.5MesP	CFU-GM	11.5EC+11.5Me	-16.7911	608.32	100	-0.03	0.9780	0.05	-1223.67	1190.09
BFU-E	I-EC+11.5MesP	CFU-GM	13.5EC+10.5Me	-20.0191	608.32	100	-0.03	0.9738	0.05	-1226.90	1186.86
BFU-E	I-EC+11.5MesP	CFU-GM	13.5EC+11.5Me	-20.3225	608.32	100	-0.03	0.9734	0.05	-1227.20	1186.56
BFU-E	I-EC+11.5MesP	CFU-GM	Ao-EC Only	26.3594	608.32	100	0.04	0.9655	0.05	-1180.52	1233.24
BFU-E	I-EC+11.5MesP	CFU-GM	AoEC+10.5MesP	-20.3765	608.32	100	-0.03	0.9733	0.05	-1227.26	1186.51
BFU-E	I-EC+11.5MesP	CFU-GM	AoEC+11.5MesP	-20.3438	608.32	100	-0.03	0.9734	0.05	-1227.23	1186.54
BFU-E	I-EC+11.5MesP	CFU-GM	E10.5-EC Only	26.3594	608.32	100	0.04	0.9655	0.05	-1180.52	1233.24
BFU-E	I-EC+11.5MesP	CFU-GM	E10.5-MesP1+	26.3594	608.32	100	0.04	0.9655	0.05	-1180.52	1233.24
BFU-E	I-EC+11.5MesP	CFU-GM	E10.5-MesP1-	26.3594	608.32	100	0.04	0.9655	0.05	-1180.52	1233.24
BFU-E	I-EC+11.5MesP	CFU-GM	E11.5MesP1+On	26.3594	608.32	100	0.04	0.9655	0.05	-1180.52	1233.24
BFU-E	I-EC+11.5MesP	CFU-GM	E11.5MesP1-On	26.3594	608.32	100	0.04	0.9655	0.05	-1180.52	1233.24
BFU-E	I-EC+11.5MesP	CFU-GM	E13.5 EC Only	26.3594	608.32	100	0.04	0.9655	0.05	-1180.52	1233.24
BFU-E	I-EC+11.5MesP	CFU-GM	H-EC Only	26.3594	608.32	100	0.04	0.9655	0.05	-1180.52	1233.24
BFU-E	I-EC+11.5MesP	CFU-GM	H-EC+10.5MesP	-20.3582	608.32	100	-0.03	0.9734	0.05	-1227.24	1186.52

The GLIMMIX Procedure

Differences of Colony_Typ*Condition Least Squares Means											
Colony_Type	Condition	_Colony_Type	_Condition	Estimate	Standard Error	DF	t Value	Pr > |t|	Alpha	Lower	Upper
BFU-E	I-EC+11.5MesP	CFU-GM	H-EC+11.5MesP	-20.2618	608.32	100	-0.03	0.9735	0.05	-1227.14	1186.62
BFU-E	I-EC+11.5MesP	CFU-GM	I-EC Only	26.3594	608.32	100	0.04	0.9655	0.05	-1180.52	1233.24
BFU-E	I-EC+11.5MesP	CFU-GM	I-EC+10.5MesP	-17.8433	608.32	100	-0.03	0.9767	0.05	-1224.72	1189.04
BFU-E	I-EC+11.5MesP	CFU-GM	I-EC+11.5MesP	-17.1523	608.31	100	-0.03	0.9776	0.05	-1224.03	1189.72
BFU-E	I-EC+11.5MesP	CFU-GM	L-E only	26.3594	608.32	100	0.04	0.9655	0.05	-1180.52	1233.24
BFU-E	I-EC+11.5MesP	CFU-GM	L-E+11.5Mesp1	-20.4210	608.32	100	-0.03	0.9733	0.05	-1227.30	1186.46
BFU-E	L-E only	BFU-E	L-E+11.5Mesp1	-56.7231	0	100	-Infty	<.0001	.	.	.
BFU-E	L-E only	CFU-GEMM	10.5EC+!0.5Me	-52.9636	1.5111	100	-35.05	<.0001	0.05	-55.9615	-49.9657
BFU-E	L-E only	CFU-GEMM	11.5 EC Only	-37.7364	607.65	100	-0.06	0.9506	0.05	-1243.30	1167.82
BFU-E	L-E only	CFU-GEMM	11.5EC+11.5Me	-52.9016	1.5108	100	-35.02	<.0001	0.05	-55.8990	-49.9042
BFU-E	L-E only	CFU-GEMM	13.5EC+10.5Me	-56.4613	1.6898	100	-33.41	<.0001	0.05	-59.8138	-53.1089
BFU-E	L-E only	CFU-GEMM	13.5EC+11.5Me	-56.0834	1.6900	100	-33.18	<.0001	0.05	-59.4364	-52.7304
BFU-E	L-E only	CFU-GEMM	Ao-EC Only	-0.00210	0.06423	100	-0.03	0.9740	0.05	-0.1295	0.1253
BFU-E	L-E only	CFU-GEMM	AoEC+10.5MesP	-56.1022	1.6900	100	-33.20	<.0001	0.05	-59.4551	-52.7493
BFU-E	L-E only	CFU-GEMM	AoEC+11.5MesP	-56.2631	1.6898	100	-33.29	<.0001	0.05	-59.6157	-52.9105
BFU-E	L-E only	CFU-GEMM	E10.5-EC Only	-0.00210	0.06423	100	-0.03	0.9740	0.05	-0.1295	0.1253
BFU-E	L-E only	CFU-GEMM	E10.5-MesP1+	-0.00210	0.06423	100	-0.03	0.9740	0.05	-0.1295	0.1253
BFU-E	L-E only	CFU-GEMM	E10.5-MesP1-	-0.00210	0.06423	100	-0.03	0.9740	0.05	-0.1295	0.1253
BFU-E	L-E only	CFU-GEMM	E11.5MesP1+On	-0.00210	0.06423	100	-0.03	0.9740	0.05	-0.1295	0.1253
BFU-E	L-E only	CFU-GEMM	E11.5MesP1-On	-0.00210	0.06423	100	-0.03	0.9740	0.05	-0.1295	0.1253
BFU-E	L-E only	CFU-GEMM	E13.5 EC Only	-0.00210	0.06423	100	-0.03	0.9740	0.05	-0.1295	0.1253
BFU-E	L-E only	CFU-GEMM	H-EC Only	-0.00210	0.06423	100	-0.03	0.9740	0.05	-0.1295	0.1253
BFU-E	L-E only	CFU-GEMM	H-EC+10.5MesP	-56.0336	1.6901	100	-33.15	<.0001	0.05	-59.3867	-52.6805
BFU-E	L-E only	CFU-GEMM	H-EC+11.5MesP	-55.9250	1.6903	100	-33.09	<.0001	0.05	-59.2784	-52.5715

The GLIMMIX Procedure

Differences of Colony_Typ*Condition Least Squares Means											
Colony_Type	Condition	_Colony_Type	_Condition	Estimate	Standard Error	DF	t Value	Pr > |t|	Alpha	Lower	Upper
BFU-E	L-E only	CFU-GEMM	I-EC Only	-0.00210	0.06423	100	-0.03	0.9740	0.05	-0.1295	0.1253
BFU-E	L-E only	CFU-GEMM	I-EC+10.5MesP	-54.7445	1.6927	100	-32.34	<.0001	0.05	-58.1027	-51.3863
BFU-E	L-E only	CFU-GEMM	I-EC+11.5MesP	-54.0123	1.6976	100	-31.82	<.0001	0.05	-57.3803	-50.6443
BFU-E	L-E only	CFU-GEMM	L-E only	-0.00210	0.06423	100	-0.03	0.9740	0.05	-0.1295	0.1253
BFU-E	L-E only	CFU-GEMM	L-E+11.5Mesp1	-56.2008	0.06423	100	-874.93	<.0001	0.05	-56.3282	-56.0733
BFU-E	L-E only	CFU-GM	10.5EC+!0.5Me	-54.4663	1.5102	100	-36.07	<.0001	0.05	-57.4625	-51.4700
BFU-E	L-E only	CFU-GM	11.5 EC Only	-54.9123	1.6924	100	-32.45	<.0001	0.05	-58.2701	-51.5546
BFU-E	L-E only	CFU-GM	11.5EC+11.5Me	-54.5162	1.5100	100	-36.10	<.0001	0.05	-57.5119	-51.5205
BFU-E	L-E only	CFU-GM	13.5EC+10.5Me	-57.7442	1.6887	100	-34.19	<.0001	0.05	-61.0946	-54.3938
BFU-E	L-E only	CFU-GM	13.5EC+11.5Me	-58.0477	1.6887	100	-34.37	<.0001	0.05	-61.3980	-54.6974
BFU-E	L-E only	CFU-GM	Ao-EC Only	-11.3658	0	100	-Infty	<.0001	.	.	.
BFU-E	L-E only	CFU-GM	AoEC+10.5MesP	-58.1017	1.6887	100	-34.41	<.0001	0.05	-61.4519	-54.7514
BFU-E	L-E only	CFU-GM	AoEC+11.5MesP	-58.0689	1.6887	100	-34.39	<.0001	0.05	-61.4192	-54.7186
BFU-E	L-E only	CFU-GM	E10.5-EC Only	-11.3658	0	100	-Infty	<.0001	.	.	.
BFU-E	L-E only	CFU-GM	E10.5-MesP1+	-11.3658	0	100	-Infty	<.0001	.	.	.
BFU-E	L-E only	CFU-GM	E10.5-MesP1-	-11.3658	0	100	-Infty	<.0001	.	.	.
BFU-E	L-E only	CFU-GM	E11.5MesP1+On	-11.3658	0	100	-Infty	<.0001	.	.	.
BFU-E	L-E only	CFU-GM	E11.5MesP1-On	-11.3658	0	100	-Infty	<.0001	.	.	.
BFU-E	L-E only	CFU-GM	E13.5 EC Only	-11.3658	0	100	-Infty	<.0001	.	.	.
BFU-E	L-E only	CFU-GM	H-EC Only	-11.3658	0	100	-Infty	<.0001	.	.	.
BFU-E	L-E only	CFU-GM	H-EC+10.5MesP	-58.0833	1.6887	100	-34.40	<.0001	0.05	-61.4336	-54.7330
BFU-E	L-E only	CFU-GM	H-EC+11.5MesP	-57.9870	1.6887	100	-34.34	<.0001	0.05	-61.3373	-54.6367
BFU-E	L-E only	CFU-GM	I-EC Only	-11.3658	0	100	-Infty	<.0001	.	.	.
BFU-E	L-E only	CFU-GM	I-EC+10.5MesP	-55.5684	1.6905	100	-32.87	<.0001	0.05	-58.9224	-52.2144

The GLIMMIX Procedure

Differences of Colony_Typ*Condition Least Squares Means											
Colony_Type	Condition	_Colony_Type	_Condition	Estimate	Standard Error	DF	t Value	Pr > |t|	Alpha	Lower	Upper
BFU-E	L-E only	CFU-GM	I-EC+11.5MesP	-54.8774	1.6926	100	-32.42	<.0001	0.05	-58.2354	-51.5194
BFU-E	L-E only	CFU-GM	L-E only	-11.3658	0	100	-Infty	<.0001	.	.	.
BFU-E	L-E only	CFU-GM	L-E+11.5Mesp1	-58.1462	0	100	-Infty	<.0001	.	.	.
BFU-E	L-E+11.5Mesp1	CFU-GEMM	10.5EC+!0.5Me	3.7595	1.5111	100	2.49	0.0145	0.05	0.7616	6.7574
BFU-E	L-E+11.5Mesp1	CFU-GEMM	11.5 EC Only	18.9867	607.65	100	0.03	0.9751	0.05	-1186.57	1224.55
BFU-E	L-E+11.5Mesp1	CFU-GEMM	11.5EC+11.5Me	3.8214	1.5108	100	2.53	0.0130	0.05	0.8240	6.8188
BFU-E	L-E+11.5Mesp1	CFU-GEMM	13.5EC+10.5Me	0.2617	1.6898	100	0.15	0.8772	0.05	-3.0907	3.6142
BFU-E	L-E+11.5Mesp1	CFU-GEMM	13.5EC+11.5Me	0.6397	1.6900	100	0.38	0.7059	0.05	-2.7133	3.9926
BFU-E	L-E+11.5Mesp1	CFU-GEMM	Ao-EC Only	56.7210	0.06423	100	883.03	<.0001	0.05	56.5935	56.8484
BFU-E	L-E+11.5Mesp1	CFU-GEMM	AoEC+10.5MesP	0.6208	1.6900	100	0.37	0.7141	0.05	-2.7321	3.9737
BFU-E	L-E+11.5Mesp1	CFU-GEMM	AoEC+11.5MesP	0.4599	1.6898	100	0.27	0.7860	0.05	-2.8926	3.8125
BFU-E	L-E+11.5Mesp1	CFU-GEMM	E10.5-EC Only	56.7210	0.06423	100	883.03	<.0001	0.05	56.5935	56.8484
BFU-E	L-E+11.5Mesp1	CFU-GEMM	E10.5-MesP1+	56.7210	0.06423	100	883.03	<.0001	0.05	56.5935	56.8484
BFU-E	L-E+11.5Mesp1	CFU-GEMM	E10.5-MesP1-	56.7210	0.06423	100	883.03	<.0001	0.05	56.5935	56.8484
BFU-E	L-E+11.5Mesp1	CFU-GEMM	E11.5MesP1+On	56.7210	0.06423	100	883.03	<.0001	0.05	56.5935	56.8484
BFU-E	L-E+11.5Mesp1	CFU-GEMM	E11.5MesP1-On	56.7210	0.06423	100	883.03	<.0001	0.05	56.5935	56.8484
BFU-E	L-E+11.5Mesp1	CFU-GEMM	E13.5 EC Only	56.7210	0.06423	100	883.03	<.0001	0.05	56.5935	56.8484
BFU-E	L-E+11.5Mesp1	CFU-GEMM	H-EC Only	56.7210	0.06423	100	883.03	<.0001	0.05	56.5935	56.8484
BFU-E	L-E+11.5Mesp1	CFU-GEMM	H-EC+10.5MesP	0.6895	1.6901	100	0.41	0.6842	0.05	-2.6636	4.0426
BFU-E	L-E+11.5Mesp1	CFU-GEMM	H-EC+11.5MesP	0.7981	1.6903	100	0.47	0.6378	0.05	-2.5553	4.1516
BFU-E	L-E+11.5Mesp1	CFU-GEMM	I-EC Only	56.7210	0.06423	100	883.03	<.0001	0.05	56.5935	56.8484
BFU-E	L-E+11.5Mesp1	CFU-GEMM	I-EC+10.5MesP	1.9786	1.6927	100	1.17	0.2452	0.05	-1.3796	5.3368
BFU-E	L-E+11.5Mesp1	CFU-GEMM	I-EC+11.5MesP	2.7108	1.6976	100	1.60	0.1135	0.05	-0.6572	6.0788
BFU-E	L-E+11.5Mesp1	CFU-GEMM	L-E only	56.7210	0.06423	100	883.03	<.0001	0.05	56.5935	56.8484

The GLIMMIX Procedure

Differences of Colony_Typ*Condition Least Squares Means											
Colony_Type	Condition	_Colony_Type	_Condition	Estimate	Standard Error	DF	t Value	Pr > |t|	Alpha	Lower	Upper
BFU-E	L-E+11.5Mesp1	CFU-GEMM	L-E+11.5Mesp1	0.5223	0.06423	100	8.13	<.0001	0.05	0.3949	0.6498
BFU-E	L-E+11.5Mesp1	CFU-GM	10.5EC+!0.5Me	2.2568	1.5102	100	1.49	0.1382	0.05	-0.7394	5.2530
BFU-E	L-E+11.5Mesp1	CFU-GM	11.5 EC Only	1.8107	1.6924	100	1.07	0.2872	0.05	-1.5470	5.1685
BFU-E	L-E+11.5Mesp1	CFU-GM	11.5EC+11.5Me	2.2069	1.5100	100	1.46	0.1470	0.05	-0.7888	5.2026
BFU-E	L-E+11.5Mesp1	CFU-GM	13.5EC+10.5Me	-1.0212	1.6887	100	-0.60	0.5468	0.05	-4.3716	2.3292
BFU-E	L-E+11.5Mesp1	CFU-GM	13.5EC+11.5Me	-1.3246	1.6887	100	-0.78	0.4347	0.05	-4.6749	2.0257
BFU-E	L-E+11.5Mesp1	CFU-GM	Ao-EC Only	45.3573	0	100	Infty	<.0001	.	.	.
BFU-E	L-E+11.5Mesp1	CFU-GM	AoEC+10.5MesP	-1.3786	1.6887	100	-0.82	0.4162	0.05	-4.7288	1.9717
BFU-E	L-E+11.5Mesp1	CFU-GM	AoEC+11.5MesP	-1.3458	1.6887	100	-0.80	0.4274	0.05	-4.6961	2.0044
BFU-E	L-E+11.5Mesp1	CFU-GM	E10.5-EC Only	45.3573	0	100	Infty	<.0001	.	.	.
BFU-E	L-E+11.5Mesp1	CFU-GM	E10.5-MesP1+	45.3573	0	100	Infty	<.0001	.	.	.
BFU-E	L-E+11.5Mesp1	CFU-GM	E10.5-MesP1-	45.3573	0	100	Infty	<.0001	.	.	.
BFU-E	L-E+11.5Mesp1	CFU-GM	E11.5MesP1+On	45.3573	0	100	Infty	<.0001	.	.	.
BFU-E	L-E+11.5Mesp1	CFU-GM	E11.5MesP1-On	45.3573	0	100	Infty	<.0001	.	.	.
BFU-E	L-E+11.5Mesp1	CFU-GM	E13.5 EC Only	45.3573	0	100	Infty	<.0001	.	.	.
BFU-E	L-E+11.5Mesp1	CFU-GM	H-EC Only	45.3573	0	100	Infty	<.0001	.	.	.
BFU-E	L-E+11.5Mesp1	CFU-GM	H-EC+10.5MesP	-1.3602	1.6887	100	-0.81	0.4224	0.05	-4.7105	1.9900
BFU-E	L-E+11.5Mesp1	CFU-GM	H-EC+11.5MesP	-1.2639	1.6887	100	-0.75	0.4559	0.05	-4.6142	2.0864
BFU-E	L-E+11.5Mesp1	CFU-GM	I-EC Only	45.3573	0	100	Infty	<.0001	.	.	.
BFU-E	L-E+11.5Mesp1	CFU-GM	I-EC+10.5MesP	1.1546	1.6905	100	0.68	0.4962	0.05	-2.1993	4.5086
BFU-E	L-E+11.5Mesp1	CFU-GM	I-EC+11.5MesP	1.8457	1.6926	100	1.09	0.2781	0.05	-1.5124	5.2037
BFU-E	L-E+11.5Mesp1	CFU-GM	L-E only	45.3573	0	100	Infty	<.0001	.	.	.
BFU-E	L-E+11.5Mesp1	CFU-GM	L-E+11.5Mesp1	-1.4231	0	100	-Infty	<.0001	.	.	.
CFU-GEMM	10.5EC+!0.5Me	CFU-GEMM	11.5 EC Only	15.2272	607.65	100	0.03	0.9801	0.05	-1190.33	1220.79

The GLIMMIX Procedure

Differences of Colony_Typ*Condition Least Squares Means											
Colony_Type	Condition	_Colony_Type	_Condition	Estimate	Standard Error	DF	t Value	Pr > |t|	Alpha	Lower	Upper
CFU-GEMM	10.5EC+!0.5Me	CFU-GEMM	11.5EC+11.5Me	0.06195	1.2865	100	0.05	0.9617	0.05	-2.4905	2.6144
CFU-GEMM	10.5EC+!0.5Me	CFU-GEMM	13.5EC+10.5Me	-3.4978	1.5122	100	-2.31	0.0228	0.05	-6.4979	-0.4977
CFU-GEMM	10.5EC+!0.5Me	CFU-GEMM	13.5EC+11.5Me	-3.1198	1.5126	100	-2.06	0.0417	0.05	-6.1207	-0.1190
CFU-GEMM	10.5EC+!0.5Me	CFU-GEMM	Ao-EC Only	52.9615	1.5124	100	35.02	<.0001	0.05	49.9609	55.9621
CFU-GEMM	10.5EC+!0.5Me	CFU-GEMM	AoEC+10.5MesP	-3.1387	1.5125	100	-2.08	0.0405	0.05	-6.1394	-0.1379
CFU-GEMM	10.5EC+!0.5Me	CFU-GEMM	AoEC+11.5MesP	-3.2995	1.5124	100	-2.18	0.0315	0.05	-6.3000	-0.2991
CFU-GEMM	10.5EC+!0.5Me	CFU-GEMM	E10.5-EC Only	52.9615	1.5124	100	35.02	<.0001	0.05	49.9609	55.9621
CFU-GEMM	10.5EC+!0.5Me	CFU-GEMM	E10.5-MesP1+	52.9615	1.5124	100	35.02	<.0001	0.05	49.9609	55.9621
CFU-GEMM	10.5EC+!0.5Me	CFU-GEMM	E10.5-MesP1-	52.9615	1.5124	100	35.02	<.0001	0.05	49.9609	55.9621
CFU-GEMM	10.5EC+!0.5Me	CFU-GEMM	E11.5MesP1+On	52.9615	1.5124	100	35.02	<.0001	0.05	49.9609	55.9621
CFU-GEMM	10.5EC+!0.5Me	CFU-GEMM	E11.5MesP1-On	52.9615	1.5124	100	35.02	<.0001	0.05	49.9609	55.9621
CFU-GEMM	10.5EC+!0.5Me	CFU-GEMM	E13.5 EC Only	52.9615	1.5124	100	35.02	<.0001	0.05	49.9609	55.9621
CFU-GEMM	10.5EC+!0.5Me	CFU-GEMM	H-EC Only	52.9615	1.5124	100	35.02	<.0001	0.05	49.9609	55.9621
CFU-GEMM	10.5EC+!0.5Me	CFU-GEMM	H-EC+10.5MesP	-3.0700	1.5126	100	-2.03	0.0451	0.05	-6.0710	-0.06899
CFU-GEMM	10.5EC+!0.5Me	CFU-GEMM	H-EC+11.5MesP	-2.9614	1.5129	100	-1.96	0.0531	0.05	-5.9629	0.04010
CFU-GEMM	10.5EC+!0.5Me	CFU-GEMM	I-EC Only	52.9615	1.5124	100	35.02	<.0001	0.05	49.9609	55.9621
CFU-GEMM	10.5EC+!0.5Me	CFU-GEMM	I-EC+10.5MesP	-1.7809	1.5153	100	-1.18	0.2427	0.05	-4.7873	1.2255
CFU-GEMM	10.5EC+!0.5Me	CFU-GEMM	I-EC+11.5MesP	-1.0487	1.5213	100	-0.69	0.4922	0.05	-4.0668	1.9695
CFU-GEMM	10.5EC+!0.5Me	CFU-GEMM	L-E only	52.9615	1.5124	100	35.02	<.0001	0.05	49.9609	55.9621
CFU-GEMM	10.5EC+!0.5Me	CFU-GEMM	L-E+11.5Mesp1	-3.2372	1.5124	100	-2.14	0.0348	0.05	-6.2378	-0.2366
CFU-GEMM	10.5EC+!0.5Me	CFU-GM	10.5EC+!0.5Me	-1.5027	0.05026	100	-29.90	<.0001	0.05	-1.6024	-1.4030
CFU-GEMM	10.5EC+!0.5Me	CFU-GM	11.5 EC Only	-1.9488	1.5154	100	-1.29	0.2014	0.05	-4.9553	1.0577
CFU-GEMM	10.5EC+!0.5Me	CFU-GM	11.5EC+11.5Me	-1.5526	1.2855	100	-1.21	0.2300	0.05	-4.1031	0.9978
CFU-GEMM	10.5EC+!0.5Me	CFU-GM	13.5EC+10.5Me	-4.7807	1.5110	100	-3.16	0.0021	0.05	-7.7785	-1.7829

The GLIMMIX Procedure

Differences of Colony_Typ*Condition Least Squares Means											
Colony_Type	Condition	_Colony_Type	_Condition	Estimate	Standard Error	DF	t Value	Pr > |t|	Alpha	Lower	Upper
CFU-GEMM	10.5EC+!0.5Me	CFU-GM	13.5EC+11.5Me	-5.0841	1.5110	100	-3.36	0.0011	0.05	-8.0820	-2.0862
CFU-GEMM	10.5EC+!0.5Me	CFU-GM	Ao-EC Only	41.5978	1.5111	100	27.53	<.0001	0.05	38.5999	44.5957
CFU-GEMM	10.5EC+!0.5Me	CFU-GM	AoEC+10.5MesP	-5.1381	1.5110	100	-3.40	0.0010	0.05	-8.1359	-2.1403
CFU-GEMM	10.5EC+!0.5Me	CFU-GM	AoEC+11.5MesP	-5.1053	1.5111	100	-3.38	0.0010	0.05	-8.1032	-2.1074
CFU-GEMM	10.5EC+!0.5Me	CFU-GM	E10.5-EC Only	41.5978	1.5111	100	27.53	<.0001	0.05	38.5999	44.5957
CFU-GEMM	10.5EC+!0.5Me	CFU-GM	E10.5-MesP1+	41.5978	1.5111	100	27.53	<.0001	0.05	38.5999	44.5957
CFU-GEMM	10.5EC+!0.5Me	CFU-GM	E10.5-MesP1-	41.5978	1.5111	100	27.53	<.0001	0.05	38.5999	44.5957
CFU-GEMM	10.5EC+!0.5Me	CFU-GM	E11.5MesP1+On	41.5978	1.5111	100	27.53	<.0001	0.05	38.5999	44.5957
CFU-GEMM	10.5EC+!0.5Me	CFU-GM	E11.5MesP1-On	41.5978	1.5111	100	27.53	<.0001	0.05	38.5999	44.5957
CFU-GEMM	10.5EC+!0.5Me	CFU-GM	E13.5 EC Only	41.5978	1.5111	100	27.53	<.0001	0.05	38.5999	44.5957
CFU-GEMM	10.5EC+!0.5Me	CFU-GM	H-EC Only	41.5978	1.5111	100	27.53	<.0001	0.05	38.5999	44.5957
CFU-GEMM	10.5EC+!0.5Me	CFU-GM	H-EC+10.5MesP	-5.1197	1.5110	100	-3.39	0.0010	0.05	-8.1176	-2.1219
CFU-GEMM	10.5EC+!0.5Me	CFU-GM	H-EC+11.5MesP	-5.0234	1.5111	100	-3.32	0.0012	0.05	-8.0214	-2.0254
CFU-GEMM	10.5EC+!0.5Me	CFU-GM	I-EC Only	41.5978	1.5111	100	27.53	<.0001	0.05	38.5999	44.5957
CFU-GEMM	10.5EC+!0.5Me	CFU-GM	I-EC+10.5MesP	-2.6048	1.5130	100	-1.72	0.0882	0.05	-5.6065	0.3968
CFU-GEMM	10.5EC+!0.5Me	CFU-GM	I-EC+11.5MesP	-1.9138	1.5156	100	-1.26	0.2096	0.05	-4.9208	1.0932
CFU-GEMM	10.5EC+!0.5Me	CFU-GM	L-E only	41.5978	1.5111	100	27.53	<.0001	0.05	38.5999	44.5957
CFU-GEMM	10.5EC+!0.5Me	CFU-GM	L-E+11.5Mesp1	-5.1826	1.5111	100	-3.43	0.0009	0.05	-8.1805	-2.1847
CFU-GEMM	11.5 EC Only	CFU-GEMM	11.5EC+11.5Me	-15.1653	607.65	100	-0.02	0.9801	0.05	-1220.72	1190.39
CFU-GEMM	11.5 EC Only	CFU-GEMM	13.5EC+10.5Me	-18.7250	607.65	100	-0.03	0.9755	0.05	-1224.28	1186.83
CFU-GEMM	11.5 EC Only	CFU-GEMM	13.5EC+11.5Me	-18.3471	607.65	100	-0.03	0.9760	0.05	-1223.91	1187.21
CFU-GEMM	11.5 EC Only	CFU-GEMM	Ao-EC Only	37.7343	607.65	100	0.06	0.9506	0.05	-1167.83	1243.29
CFU-GEMM	11.5 EC Only	CFU-GEMM	AoEC+10.5MesP	-18.3659	607.65	100	-0.03	0.9759	0.05	-1223.93	1187.19
CFU-GEMM	11.5 EC Only	CFU-GEMM	AoEC+11.5MesP	-18.5268	607.65	100	-0.03	0.9757	0.05	-1224.09	1187.03

The GLIMMIX Procedure

Differences of Colony_Typ*Condition Least Squares Means											
Colony_Type	Condition	_Colony_Type	_Condition	Estimate	Standard Error	DF	t Value	Pr > |t|	Alpha	Lower	Upper
CFU-GEMM	11.5 EC Only	CFU-GEMM	E10.5-EC Only	37.7343	607.65	100	0.06	0.9506	0.05	-1167.83	1243.29
CFU-GEMM	11.5 EC Only	CFU-GEMM	E10.5-MesP1+	37.7343	607.65	100	0.06	0.9506	0.05	-1167.83	1243.29
CFU-GEMM	11.5 EC Only	CFU-GEMM	E10.5-MesP1-	37.7343	607.65	100	0.06	0.9506	0.05	-1167.83	1243.29
CFU-GEMM	11.5 EC Only	CFU-GEMM	E11.5MesP1+On	37.7343	607.65	100	0.06	0.9506	0.05	-1167.83	1243.29
CFU-GEMM	11.5 EC Only	CFU-GEMM	E11.5MesP1-On	37.7343	607.65	100	0.06	0.9506	0.05	-1167.83	1243.29
CFU-GEMM	11.5 EC Only	CFU-GEMM	E13.5 EC Only	37.7343	607.65	100	0.06	0.9506	0.05	-1167.83	1243.29
CFU-GEMM	11.5 EC Only	CFU-GEMM	H-EC Only	37.7343	607.65	100	0.06	0.9506	0.05	-1167.83	1243.29
CFU-GEMM	11.5 EC Only	CFU-GEMM	H-EC+10.5MesP	-18.2972	607.65	100	-0.03	0.9760	0.05	-1223.86	1187.26
CFU-GEMM	11.5 EC Only	CFU-GEMM	H-EC+11.5MesP	-18.1886	607.65	100	-0.03	0.9762	0.05	-1223.75	1187.37
CFU-GEMM	11.5 EC Only	CFU-GEMM	I-EC Only	37.7343	607.65	100	0.06	0.9506	0.05	-1167.83	1243.29
CFU-GEMM	11.5 EC Only	CFU-GEMM	I-EC+10.5MesP	-17.0081	607.65	100	-0.03	0.9777	0.05	-1222.57	1188.55
CFU-GEMM	11.5 EC Only	CFU-GEMM	I-EC+11.5MesP	-16.2759	607.65	100	-0.03	0.9787	0.05	-1221.84	1189.28
CFU-GEMM	11.5 EC Only	CFU-GEMM	L-E only	37.7343	607.65	100	0.06	0.9506	0.05	-1167.83	1243.29
CFU-GEMM	11.5 EC Only	CFU-GEMM	L-E+11.5Mesp1	-18.4644	607.65	100	-0.03	0.9758	0.05	-1224.02	1187.10
CFU-GEMM	11.5 EC Only	CFU-GM	10.5EC+!0.5Me	-16.7299	607.65	100	-0.03	0.9781	0.05	-1222.29	1188.83
CFU-GEMM	11.5 EC Only	CFU-GM	11.5 EC Only	-17.1760	607.65	100	-0.03	0.9775	0.05	-1222.73	1188.38
CFU-GEMM	11.5 EC Only	CFU-GM	11.5EC+11.5Me	-16.7799	607.65	100	-0.03	0.9780	0.05	-1222.34	1188.78
CFU-GEMM	11.5 EC Only	CFU-GM	13.5EC+10.5Me	-20.0079	607.65	100	-0.03	0.9738	0.05	-1225.57	1185.55
CFU-GEMM	11.5 EC Only	CFU-GM	13.5EC+11.5Me	-20.3113	607.65	100	-0.03	0.9734	0.05	-1225.87	1185.25
CFU-GEMM	11.5 EC Only	CFU-GM	Ao-EC Only	26.3706	607.65	100	0.04	0.9655	0.05	-1179.19	1231.93
CFU-GEMM	11.5 EC Only	CFU-GM	AoEC+10.5MesP	-20.3653	607.65	100	-0.03	0.9733	0.05	-1225.92	1185.19
CFU-GEMM	11.5 EC Only	CFU-GM	AoEC+11.5MesP	-20.3325	607.65	100	-0.03	0.9734	0.05	-1225.89	1185.23
CFU-GEMM	11.5 EC Only	CFU-GM	E10.5-EC Only	26.3706	607.65	100	0.04	0.9655	0.05	-1179.19	1231.93
CFU-GEMM	11.5 EC Only	CFU-GM	E10.5-MesP1+	26.3706	607.65	100	0.04	0.9655	0.05	-1179.19	1231.93

The GLIMMIX Procedure

Differences of Colony_Typ*Condition Least Squares Means											
Colony_Type	Condition	_Colony_Type	_Condition	Estimate	Standard Error	DF	t Value	Pr > |t|	Alpha	Lower	Upper
CFU-GEMM	11.5 EC Only	CFU-GM	E10.5-MesP1-	26.3706	607.65	100	0.04	0.9655	0.05	-1179.19	1231.93
CFU-GEMM	11.5 EC Only	CFU-GM	E11.5MesP1+On	26.3706	607.65	100	0.04	0.9655	0.05	-1179.19	1231.93
CFU-GEMM	11.5 EC Only	CFU-GM	E11.5MesP1-On	26.3706	607.65	100	0.04	0.9655	0.05	-1179.19	1231.93
CFU-GEMM	11.5 EC Only	CFU-GM	E13.5 EC Only	26.3706	607.65	100	0.04	0.9655	0.05	-1179.19	1231.93
CFU-GEMM	11.5 EC Only	CFU-GM	H-EC Only	26.3706	607.65	100	0.04	0.9655	0.05	-1179.19	1231.93
CFU-GEMM	11.5 EC Only	CFU-GM	H-EC+10.5MesP	-20.3470	607.65	100	-0.03	0.9734	0.05	-1225.91	1185.21
CFU-GEMM	11.5 EC Only	CFU-GM	H-EC+11.5MesP	-20.2506	607.65	100	-0.03	0.9735	0.05	-1225.81	1185.31
CFU-GEMM	11.5 EC Only	CFU-GM	I-EC Only	26.3706	607.65	100	0.04	0.9655	0.05	-1179.19	1231.93
CFU-GEMM	11.5 EC Only	CFU-GM	I-EC+10.5MesP	-17.8321	607.65	100	-0.03	0.9766	0.05	-1223.39	1187.73
CFU-GEMM	11.5 EC Only	CFU-GM	I-EC+11.5MesP	-17.1410	607.65	100	-0.03	0.9776	0.05	-1222.70	1188.42
CFU-GEMM	11.5 EC Only	CFU-GM	L-E only	26.3706	607.65	100	0.04	0.9655	0.05	-1179.19	1231.93
CFU-GEMM	11.5 EC Only	CFU-GM	L-E+11.5Mesp1	-20.4098	607.65	100	-0.03	0.9733	0.05	-1225.97	1185.15
CFU-GEMM	11.5EC+11.5Me	CFU-GEMM	13.5EC+10.5Me	-3.5597	1.5119	100	-2.35	0.0205	0.05	-6.5593	-0.5601
CFU-GEMM	11.5EC+11.5Me	CFU-GEMM	13.5EC+11.5Me	-3.1818	1.5123	100	-2.10	0.0379	0.05	-6.1822	-0.1814
CFU-GEMM	11.5EC+11.5Me	CFU-GEMM	Ao-EC Only	52.8995	1.5122	100	34.98	<.0001	0.05	49.8994	55.8996
CFU-GEMM	11.5EC+11.5Me	CFU-GEMM	AoEC+10.5MesP	-3.2006	1.5123	100	-2.12	0.0368	0.05	-6.2009	-0.2003
CFU-GEMM	11.5EC+11.5Me	CFU-GEMM	AoEC+11.5MesP	-3.3615	1.5121	100	-2.22	0.0285	0.05	-6.3615	-0.3615
CFU-GEMM	11.5EC+11.5Me	CFU-GEMM	E10.5-EC Only	52.8995	1.5122	100	34.98	<.0001	0.05	49.8994	55.8996
CFU-GEMM	11.5EC+11.5Me	CFU-GEMM	E10.5-MesP1+	52.8995	1.5122	100	34.98	<.0001	0.05	49.8994	55.8996
CFU-GEMM	11.5EC+11.5Me	CFU-GEMM	E10.5-MesP1-	52.8995	1.5122	100	34.98	<.0001	0.05	49.8994	55.8996
CFU-GEMM	11.5EC+11.5Me	CFU-GEMM	E11.5MesP1+On	52.8995	1.5122	100	34.98	<.0001	0.05	49.8994	55.8996
CFU-GEMM	11.5EC+11.5Me	CFU-GEMM	E11.5MesP1-On	52.8995	1.5122	100	34.98	<.0001	0.05	49.8994	55.8996
CFU-GEMM	11.5EC+11.5Me	CFU-GEMM	E13.5 EC Only	52.8995	1.5122	100	34.98	<.0001	0.05	49.8994	55.8996
CFU-GEMM	11.5EC+11.5Me	CFU-GEMM	H-EC Only	52.8995	1.5122	100	34.98	<.0001	0.05	49.8994	55.8996

The GLIMMIX Procedure

Differences of Colony_Typ*Condition Least Squares Means											
Colony_Type	Condition	_Colony_Type	_Condition	Estimate	Standard Error	DF	t Value	Pr > |t|	Alpha	Lower	Upper
CFU-GEMM	11.5EC+11.5Me	CFU-GEMM	H-EC+10.5MesP	-3.1319	1.5124	100	-2.07	0.0409	0.05	-6.1324	-0.1314
CFU-GEMM	11.5EC+11.5Me	CFU-GEMM	H-EC+11.5MesP	-3.0233	1.5126	100	-2.00	0.0484	0.05	-6.0243	-0.02234
CFU-GEMM	11.5EC+11.5Me	CFU-GEMM	I-EC Only	52.8995	1.5122	100	34.98	<.0001	0.05	49.8994	55.8996
CFU-GEMM	11.5EC+11.5Me	CFU-GEMM	I-EC+10.5MesP	-1.8429	1.5151	100	-1.22	0.2267	0.05	-4.8488	1.1630
CFU-GEMM	11.5EC+11.5Me	CFU-GEMM	I-EC+11.5MesP	-1.1106	1.5210	100	-0.73	0.4670	0.05	-4.1283	1.9070
CFU-GEMM	11.5EC+11.5Me	CFU-GEMM	L-E only	52.8995	1.5122	100	34.98	<.0001	0.05	49.8994	55.8996
CFU-GEMM	11.5EC+11.5Me	CFU-GEMM	L-E+11.5Mesp1	-3.2991	1.5122	100	-2.18	0.0315	0.05	-6.2992	-0.2990
CFU-GEMM	11.5EC+11.5Me	CFU-GM	10.5EC+!0.5Me	-1.5646	1.2855	100	-1.22	0.2264	0.05	-4.1151	0.9858
CFU-GEMM	11.5EC+11.5Me	CFU-GM	11.5 EC Only	-2.0107	1.5151	100	-1.33	0.1875	0.05	-5.0167	0.9953
CFU-GEMM	11.5EC+11.5Me	CFU-GM	11.5EC+11.5Me	-1.6146	0.05072	100	-31.83	<.0001	0.05	-1.7152	-1.5139
CFU-GEMM	11.5EC+11.5Me	CFU-GM	13.5EC+10.5Me	-4.8426	1.5108	100	-3.21	0.0018	0.05	-7.8399	-1.8453
CFU-GEMM	11.5EC+11.5Me	CFU-GM	13.5EC+11.5Me	-5.1461	1.5108	100	-3.41	0.0009	0.05	-8.1434	-2.1487
CFU-GEMM	11.5EC+11.5Me	CFU-GM	Ao-EC Only	41.5358	1.5108	100	27.49	<.0001	0.05	38.5384	44.5332
CFU-GEMM	11.5EC+11.5Me	CFU-GM	AoEC+10.5MesP	-5.2000	1.5108	100	-3.44	0.0008	0.05	-8.1974	-2.2027
CFU-GEMM	11.5EC+11.5Me	CFU-GM	AoEC+11.5MesP	-5.1673	1.5108	100	-3.42	0.0009	0.05	-8.1647	-2.1699
CFU-GEMM	11.5EC+11.5Me	CFU-GM	E10.5-EC Only	41.5358	1.5108	100	27.49	<.0001	0.05	38.5384	44.5332
CFU-GEMM	11.5EC+11.5Me	CFU-GM	E10.5-MesP1+	41.5358	1.5108	100	27.49	<.0001	0.05	38.5384	44.5332
CFU-GEMM	11.5EC+11.5Me	CFU-GM	E10.5-MesP1-	41.5358	1.5108	100	27.49	<.0001	0.05	38.5384	44.5332
CFU-GEMM	11.5EC+11.5Me	CFU-GM	E11.5MesP1+On	41.5358	1.5108	100	27.49	<.0001	0.05	38.5384	44.5332
CFU-GEMM	11.5EC+11.5Me	CFU-GM	E11.5MesP1-On	41.5358	1.5108	100	27.49	<.0001	0.05	38.5384	44.5332
CFU-GEMM	11.5EC+11.5Me	CFU-GM	E13.5 EC Only	41.5358	1.5108	100	27.49	<.0001	0.05	38.5384	44.5332
CFU-GEMM	11.5EC+11.5Me	CFU-GM	H-EC Only	41.5358	1.5108	100	27.49	<.0001	0.05	38.5384	44.5332
CFU-GEMM	11.5EC+11.5Me	CFU-GM	H-EC+10.5MesP	-5.1817	1.5108	100	-3.43	0.0009	0.05	-8.1790	-2.1843
CFU-GEMM	11.5EC+11.5Me	CFU-GM	H-EC+11.5MesP	-5.0854	1.5108	100	-3.37	0.0011	0.05	-8.0828	-2.0879

The GLIMMIX Procedure

Differences of Colony_Typ*Condition Least Squares Means											
Colony_Type	Condition	_Colony_Type	_Condition	Estimate	Standard Error	DF	t Value	Pr > |t|	Alpha	Lower	Upper
CFU-GEMM	11.5EC+11.5Me	CFU-GM	I-EC Only	41.5358	1.5108	100	27.49	<.0001	0.05	38.5384	44.5332
CFU-GEMM	11.5EC+11.5Me	CFU-GM	I-EC+10.5MesP	-2.6668	1.5127	100	-1.76	0.0810	0.05	-5.6680	0.3344
CFU-GEMM	11.5EC+11.5Me	CFU-GM	I-EC+11.5MesP	-1.9758	1.5154	100	-1.30	0.1953	0.05	-4.9823	1.0307
CFU-GEMM	11.5EC+11.5Me	CFU-GM	L-E only	41.5358	1.5108	100	27.49	<.0001	0.05	38.5384	44.5332
CFU-GEMM	11.5EC+11.5Me	CFU-GM	L-E+11.5Mesp1	-5.2445	1.5108	100	-3.47	0.0008	0.05	-8.2419	-2.2471
CFU-GEMM	13.5EC+10.5Me	CFU-GEMM	13.5EC+11.5Me	0.3779	1.6912	100	0.22	0.8236	0.05	-2.9773	3.7331
CFU-GEMM	13.5EC+10.5Me	CFU-GEMM	Ao-EC Only	56.4592	1.6910	100	33.39	<.0001	0.05	53.1044	59.8141
CFU-GEMM	13.5EC+10.5Me	CFU-GEMM	AoEC+10.5MesP	0.3591	1.6911	100	0.21	0.8323	0.05	-2.9960	3.7142
CFU-GEMM	13.5EC+10.5Me	CFU-GEMM	AoEC+11.5MesP	0.1982	1.6910	100	0.12	0.9069	0.05	-3.1566	3.5530
CFU-GEMM	13.5EC+10.5Me	CFU-GEMM	E10.5-EC Only	56.4592	1.6910	100	33.39	<.0001	0.05	53.1044	59.8141
CFU-GEMM	13.5EC+10.5Me	CFU-GEMM	E10.5-MesP1+	56.4592	1.6910	100	33.39	<.0001	0.05	53.1044	59.8141
CFU-GEMM	13.5EC+10.5Me	CFU-GEMM	E10.5-MesP1-	56.4592	1.6910	100	33.39	<.0001	0.05	53.1044	59.8141
CFU-GEMM	13.5EC+10.5Me	CFU-GEMM	E11.5MesP1+On	56.4592	1.6910	100	33.39	<.0001	0.05	53.1044	59.8141
CFU-GEMM	13.5EC+10.5Me	CFU-GEMM	E11.5MesP1-On	56.4592	1.6910	100	33.39	<.0001	0.05	53.1044	59.8141
CFU-GEMM	13.5EC+10.5Me	CFU-GEMM	E13.5 EC Only	56.4592	1.6910	100	33.39	<.0001	0.05	53.1044	59.8141
CFU-GEMM	13.5EC+10.5Me	CFU-GEMM	H-EC Only	56.4592	1.6910	100	33.39	<.0001	0.05	53.1044	59.8141
CFU-GEMM	13.5EC+10.5Me	CFU-GEMM	H-EC+10.5MesP	0.4278	1.6912	100	0.25	0.8008	0.05	-2.9275	3.7831
CFU-GEMM	13.5EC+10.5Me	CFU-GEMM	H-EC+11.5MesP	0.5364	1.6914	100	0.32	0.7518	0.05	-2.8193	3.8920
CFU-GEMM	13.5EC+10.5Me	CFU-GEMM	I-EC Only	56.4592	1.6910	100	33.39	<.0001	0.05	53.1044	59.8141
CFU-GEMM	13.5EC+10.5Me	CFU-GEMM	I-EC+10.5MesP	1.7168	1.6938	100	1.01	0.3132	0.05	-1.6436	5.0773
CFU-GEMM	13.5EC+10.5Me	CFU-GEMM	I-EC+11.5MesP	2.4491	1.6987	100	1.44	0.1525	0.05	-0.9212	5.8193
CFU-GEMM	13.5EC+10.5Me	CFU-GEMM	L-E only	56.4592	1.6910	100	33.39	<.0001	0.05	53.1044	59.8141
CFU-GEMM	13.5EC+10.5Me	CFU-GEMM	L-E+11.5Mesp1	0.2606	1.6910	100	0.15	0.8778	0.05	-3.0943	3.6155
CFU-GEMM	13.5EC+10.5Me	CFU-GM	10.5EC+!0.5Me	1.9951	1.5113	100	1.32	0.1898	0.05	-1.0034	4.9935

The GLIMMIX Procedure

Differences of Colony_Typ*Condition Least Squares Means											
Colony_Type	Condition	_Colony_Type	_Condition	Estimate	Standard Error	DF	t Value	Pr > |t|	Alpha	Lower	Upper
CFU-GEMM	13.5EC+10.5Me	CFU-GM	11.5 EC Only	1.5490	1.6935	100	0.91	0.3626	0.05	-1.8110	4.9089
CFU-GEMM	13.5EC+10.5Me	CFU-GM	11.5EC+11.5Me	1.9451	1.5111	100	1.29	0.2010	0.05	-1.0528	4.9430
CFU-GEMM	13.5EC+10.5Me	CFU-GM	13.5EC+10.5Me	-1.2829	0.05924	100	-21.66	<.0001	0.05	-1.4004	-1.1654
CFU-GEMM	13.5EC+10.5Me	CFU-GM	13.5EC+11.5Me	-1.5864	1.6898	100	-0.94	0.3501	0.05	-4.9389	1.7661
CFU-GEMM	13.5EC+10.5Me	CFU-GM	Ao-EC Only	45.0955	1.6898	100	26.69	<.0001	0.05	41.7431	48.4480
CFU-GEMM	13.5EC+10.5Me	CFU-GM	AoEC+10.5MesP	-1.6403	1.6898	100	-0.97	0.3340	0.05	-4.9928	1.7121
CFU-GEMM	13.5EC+10.5Me	CFU-GM	AoEC+11.5MesP	-1.6076	1.6898	100	-0.95	0.3437	0.05	-4.9601	1.7449
CFU-GEMM	13.5EC+10.5Me	CFU-GM	E10.5-EC Only	45.0955	1.6898	100	26.69	<.0001	0.05	41.7431	48.4480
CFU-GEMM	13.5EC+10.5Me	CFU-GM	E10.5-MesP1+	45.0955	1.6898	100	26.69	<.0001	0.05	41.7431	48.4480
CFU-GEMM	13.5EC+10.5Me	CFU-GM	E10.5-MesP1-	45.0955	1.6898	100	26.69	<.0001	0.05	41.7431	48.4480
CFU-GEMM	13.5EC+10.5Me	CFU-GM	E11.5MesP1+On	45.0955	1.6898	100	26.69	<.0001	0.05	41.7431	48.4480
CFU-GEMM	13.5EC+10.5Me	CFU-GM	E11.5MesP1-On	45.0955	1.6898	100	26.69	<.0001	0.05	41.7431	48.4480
CFU-GEMM	13.5EC+10.5Me	CFU-GM	E13.5 EC Only	45.0955	1.6898	100	26.69	<.0001	0.05	41.7431	48.4480
CFU-GEMM	13.5EC+10.5Me	CFU-GM	H-EC Only	45.0955	1.6898	100	26.69	<.0001	0.05	41.7431	48.4480
CFU-GEMM	13.5EC+10.5Me	CFU-GM	H-EC+10.5MesP	-1.6220	1.6898	100	-0.96	0.3394	0.05	-4.9745	1.7305
CFU-GEMM	13.5EC+10.5Me	CFU-GM	H-EC+11.5MesP	-1.5257	1.6898	100	-0.90	0.3688	0.05	-4.8782	1.8269
CFU-GEMM	13.5EC+10.5Me	CFU-GM	I-EC Only	45.0955	1.6898	100	26.69	<.0001	0.05	41.7431	48.4480
CFU-GEMM	13.5EC+10.5Me	CFU-GM	I-EC+10.5MesP	0.8929	1.6917	100	0.53	0.5988	0.05	-2.4633	4.2491
CFU-GEMM	13.5EC+10.5Me	CFU-GM	I-EC+11.5MesP	1.5839	1.6937	100	0.94	0.3519	0.05	-1.7763	4.9442
CFU-GEMM	13.5EC+10.5Me	CFU-GM	L-E only	45.0955	1.6898	100	26.69	<.0001	0.05	41.7431	48.4480
CFU-GEMM	13.5EC+10.5Me	CFU-GM	L-E+11.5Mesp1	-1.6848	1.6898	100	-1.00	0.3211	0.05	-5.0373	1.6676
CFU-GEMM	13.5EC+11.5Me	CFU-GEMM	Ao-EC Only	56.0813	1.6913	100	33.16	<.0001	0.05	52.7259	59.4367
CFU-GEMM	13.5EC+11.5Me	CFU-GEMM	AoEC+10.5MesP	-0.01881	1.6914	100	-0.01	0.9911	0.05	-3.3745	3.3368
CFU-GEMM	13.5EC+11.5Me	CFU-GEMM	AoEC+11.5MesP	-0.1797	1.6912	100	-0.11	0.9156	0.05	-3.5350	3.1756

The GLIMMIX Procedure

Differences of Colony_Typ*Condition Least Squares Means											
Colony_Type	Condition	_Colony_Type	_Condition	Estimate	Standard Error	DF	t Value	Pr > |t|	Alpha	Lower	Upper
CFU-GEMM	13.5EC+11.5Me	CFU-GEMM	E10.5-EC Only	56.0813	1.6913	100	33.16	<.0001	0.05	52.7259	59.4367
CFU-GEMM	13.5EC+11.5Me	CFU-GEMM	E10.5-MesP1+	56.0813	1.6913	100	33.16	<.0001	0.05	52.7259	59.4367
CFU-GEMM	13.5EC+11.5Me	CFU-GEMM	E10.5-MesP1-	56.0813	1.6913	100	33.16	<.0001	0.05	52.7259	59.4367
CFU-GEMM	13.5EC+11.5Me	CFU-GEMM	E11.5MesP1+On	56.0813	1.6913	100	33.16	<.0001	0.05	52.7259	59.4367
CFU-GEMM	13.5EC+11.5Me	CFU-GEMM	E11.5MesP1-On	56.0813	1.6913	100	33.16	<.0001	0.05	52.7259	59.4367
CFU-GEMM	13.5EC+11.5Me	CFU-GEMM	E13.5 EC Only	56.0813	1.6913	100	33.16	<.0001	0.05	52.7259	59.4367
CFU-GEMM	13.5EC+11.5Me	CFU-GEMM	H-EC Only	56.0813	1.6913	100	33.16	<.0001	0.05	52.7259	59.4367
CFU-GEMM	13.5EC+11.5Me	CFU-GEMM	H-EC+10.5MesP	0.04986	1.6915	100	0.03	0.9765	0.05	-3.3060	3.4057
CFU-GEMM	13.5EC+11.5Me	CFU-GEMM	H-EC+11.5MesP	0.1585	1.6917	100	0.09	0.9256	0.05	-3.1977	3.5147
CFU-GEMM	13.5EC+11.5Me	CFU-GEMM	I-EC Only	56.0813	1.6913	100	33.16	<.0001	0.05	52.7259	59.4367
CFU-GEMM	13.5EC+11.5Me	CFU-GEMM	I-EC+10.5MesP	1.3389	1.6941	100	0.79	0.4312	0.05	-2.0220	4.6999
CFU-GEMM	13.5EC+11.5Me	CFU-GEMM	I-EC+11.5MesP	2.0711	1.6990	100	1.22	0.2257	0.05	-1.2996	5.4419
CFU-GEMM	13.5EC+11.5Me	CFU-GEMM	L-E only	56.0813	1.6913	100	33.16	<.0001	0.05	52.7259	59.4367
CFU-GEMM	13.5EC+11.5Me	CFU-GEMM	L-E+11.5Mesp1	-0.1173	1.6913	100	-0.07	0.9448	0.05	-3.4728	3.2381
CFU-GEMM	13.5EC+11.5Me	CFU-GM	10.5EC+!0.5Me	1.6172	1.5117	100	1.07	0.2873	0.05	-1.3821	4.6164
CFU-GEMM	13.5EC+11.5Me	CFU-GM	11.5 EC Only	1.1711	1.6938	100	0.69	0.4909	0.05	-2.1894	4.5315
CFU-GEMM	13.5EC+11.5Me	CFU-GM	11.5EC+11.5Me	1.5672	1.5115	100	1.04	0.3023	0.05	-1.4315	4.5659
CFU-GEMM	13.5EC+11.5Me	CFU-GM	13.5EC+10.5Me	-1.6608	1.6901	100	-0.98	0.3281	0.05	-5.0140	1.6923
CFU-GEMM	13.5EC+11.5Me	CFU-GM	13.5EC+11.5Me	-1.9643	0.06794	100	-28.91	<.0001	0.05	-2.0991	-1.8295
CFU-GEMM	13.5EC+11.5Me	CFU-GM	Ao-EC Only	44.7176	1.6900	100	26.46	<.0001	0.05	41.3646	48.0706
CFU-GEMM	13.5EC+11.5Me	CFU-GM	AoEC+10.5MesP	-2.0182	1.6900	100	-1.19	0.2352	0.05	-5.3712	1.3348
CFU-GEMM	13.5EC+11.5Me	CFU-GM	AoEC+11.5MesP	-1.9855	1.6901	100	-1.17	0.2429	0.05	-5.3385	1.3675
CFU-GEMM	13.5EC+11.5Me	CFU-GM	E10.5-EC Only	44.7176	1.6900	100	26.46	<.0001	0.05	41.3646	48.0706
CFU-GEMM	13.5EC+11.5Me	CFU-GM	E10.5-MesP1+	44.7176	1.6900	100	26.46	<.0001	0.05	41.3646	48.0706

The GLIMMIX Procedure

Differences of Colony_Typ*Condition Least Squares Means											
Colony_Type	Condition	_Colony_Type	_Condition	Estimate	Standard Error	DF	t Value	Pr > |t|	Alpha	Lower	Upper
CFU-GEMM	13.5EC+11.5Me	CFU-GM	E10.5-MesP1-	44.7176	1.6900	100	26.46	<.0001	0.05	41.3646	48.0706
CFU-GEMM	13.5EC+11.5Me	CFU-GM	E11.5MesP1+On	44.7176	1.6900	100	26.46	<.0001	0.05	41.3646	48.0706
CFU-GEMM	13.5EC+11.5Me	CFU-GM	E11.5MesP1-On	44.7176	1.6900	100	26.46	<.0001	0.05	41.3646	48.0706
CFU-GEMM	13.5EC+11.5Me	CFU-GM	E13.5 EC Only	44.7176	1.6900	100	26.46	<.0001	0.05	41.3646	48.0706
CFU-GEMM	13.5EC+11.5Me	CFU-GM	H-EC Only	44.7176	1.6900	100	26.46	<.0001	0.05	41.3646	48.0706
CFU-GEMM	13.5EC+11.5Me	CFU-GM	H-EC+10.5MesP	-1.9999	1.6900	100	-1.18	0.2395	0.05	-5.3529	1.3531
CFU-GEMM	13.5EC+11.5Me	CFU-GM	H-EC+11.5MesP	-1.9036	1.6901	100	-1.13	0.2627	0.05	-5.2566	1.4495
CFU-GEMM	13.5EC+11.5Me	CFU-GM	I-EC Only	44.7176	1.6900	100	26.46	<.0001	0.05	41.3646	48.0706
CFU-GEMM	13.5EC+11.5Me	CFU-GM	I-EC+10.5MesP	0.5150	1.6919	100	0.30	0.7615	0.05	-2.8417	3.8717
CFU-GEMM	13.5EC+11.5Me	CFU-GM	I-EC+11.5MesP	1.2060	1.6940	100	0.71	0.4782	0.05	-2.1547	4.5668
CFU-GEMM	13.5EC+11.5Me	CFU-GM	L-E only	44.7176	1.6900	100	26.46	<.0001	0.05	41.3646	48.0706
CFU-GEMM	13.5EC+11.5Me	CFU-GM	L-E+11.5Mesp1	-2.0627	1.6900	100	-1.22	0.2251	0.05	-5.4157	1.2903
CFU-GEMM	Ao-EC Only	CFU-GEMM	AoEC+10.5MesP	-56.1001	1.6912	100	-33.17	<.0001	0.05	-59.4555	-52.7448
CFU-GEMM	Ao-EC Only	CFU-GEMM	AoEC+11.5MesP	-56.2610	1.6911	100	-33.27	<.0001	0.05	-59.6160	-52.9060
CFU-GEMM	Ao-EC Only	CFU-GEMM	E10.5-EC Only	1.78E-15	.	.	.	.	.	.	.
CFU-GEMM	Ao-EC Only	CFU-GEMM	E10.5-MesP1+	-515E-16	.	.	.	.	.	.	.
CFU-GEMM	Ao-EC Only	CFU-GEMM	E10.5-MesP1-	-888E-17	.	.	.	.	.	.	.
CFU-GEMM	Ao-EC Only	CFU-GEMM	E11.5MesP1+On	2.49E-14	.	.	.	.	.	.	.
CFU-GEMM	Ao-EC Only	CFU-GEMM	E11.5MesP1-On	-853E-16	.	.	.	.	.	.	.
CFU-GEMM	Ao-EC Only	CFU-GEMM	E13.5 EC Only	1.12E-13	.	.	.	.	.	.	.
CFU-GEMM	Ao-EC Only	CFU-GEMM	H-EC Only	2.75E-13	.	.	.	.	.	.	.
CFU-GEMM	Ao-EC Only	CFU-GEMM	H-EC+10.5MesP	-56.0315	1.6913	100	-33.13	<.0001	0.05	-59.3870	-52.6760
CFU-GEMM	Ao-EC Only	CFU-GEMM	H-EC+11.5MesP	-55.9229	1.6915	100	-33.06	<.0001	0.05	-59.2787	-52.5670
CFU-GEMM	Ao-EC Only	CFU-GEMM	I-EC Only	9.41E-14	.	.	.	.	.	.	.

The GLIMMIX Procedure

Differences of Colony_Typ*Condition Least Squares Means

Colony_Type	Condition	_Colony_Type	_Condition	Estimate	Standard Error	DF	t Value	Pr > |t|	Alpha	Lower	Upper
CFU-GEMM	Ao-EC Only	CFU-GEMM	I-EC+10.5MesP	-54.7424	1.6939	100	-32.32	<.0001	0.05	-58.1030	-51.3818
CFU-GEMM	Ao-EC Only	CFU-GEMM	I-EC+11.5MesP	-54.0102	1.6988	100	-31.79	<.0001	0.05	-57.3806	-50.6397
CFU-GEMM	Ao-EC Only	CFU-GEMM	L-E only	8.88E-14	.	.	.	.	.	.	.
CFU-GEMM	Ao-EC Only	CFU-GEMM	L-E+11.5Mesp1	-56.1987	0	100	-Infty	<.0001	.	.	.
CFU-GEMM	Ao-EC Only	CFU-GM	10.5EC+!0.5Me	-54.4642	1.5116	100	-36.03	<.0001	0.05	-57.4631	-51.4652
CFU-GEMM	Ao-EC Only	CFU-GM	11.5 EC Only	-54.9102	1.6937	100	-32.42	<.0001	0.05	-58.2704	-51.5501
CFU-GEMM	Ao-EC Only	CFU-GM	11.5EC+11.5Me	-54.5141	1.5113	100	-36.07	<.0001	0.05	-57.5125	-51.5157
CFU-GEMM	Ao-EC Only	CFU-GM	13.5EC+10.5Me	-57.7421	1.6900	100	-34.17	<.0001	0.05	-61.0950	-54.3893
CFU-GEMM	Ao-EC Only	CFU-GM	13.5EC+11.5Me	-58.0456	1.6899	100	-34.35	<.0001	0.05	-61.3983	-54.6929
CFU-GEMM	Ao-EC Only	CFU-GM	Ao-EC Only	-11.3637	0.06423	100	-176.91	<.0001	0.05	-11.4911	-11.2363
CFU-GEMM	Ao-EC Only	CFU-GM	AoEC+10.5MesP	-58.0996	1.6899	100	-34.38	<.0001	0.05	-61.4522	-54.7469
CFU-GEMM	Ao-EC Only	CFU-GM	AoEC+11.5MesP	-58.0668	1.6899	100	-34.36	<.0001	0.05	-61.4195	-54.7141
CFU-GEMM	Ao-EC Only	CFU-GM	E10.5-EC Only	-11.3637	0.06423	100	-176.91	<.0001	0.05	-11.4911	-11.2363
CFU-GEMM	Ao-EC Only	CFU-GM	E10.5-MesP1+	-11.3637	0.06423	100	-176.91	<.0001	0.05	-11.4911	-11.2363
CFU-GEMM	Ao-EC Only	CFU-GM	E10.5-MesP1-	-11.3637	0.06423	100	-176.91	<.0001	0.05	-11.4911	-11.2363
CFU-GEMM	Ao-EC Only	CFU-GM	E11.5MesP1+On	-11.3637	0.06423	100	-176.91	<.0001	0.05	-11.4911	-11.2363
CFU-GEMM	Ao-EC Only	CFU-GM	E11.5MesP1-On	-11.3637	0.06423	100	-176.91	<.0001	0.05	-11.4911	-11.2363
CFU-GEMM	Ao-EC Only	CFU-GM	E13.5 EC Only	-11.3637	0.06423	100	-176.91	<.0001	0.05	-11.4911	-11.2363
CFU-GEMM	Ao-EC Only	CFU-GM	H-EC Only	-11.3637	0.06423	100	-176.91	<.0001	0.05	-11.4911	-11.2363
CFU-GEMM	Ao-EC Only	CFU-GM	H-EC+10.5MesP	-58.0812	1.6899	100	-34.37	<.0001	0.05	-61.4339	-54.7285
CFU-GEMM	Ao-EC Only	CFU-GM	H-EC+11.5MesP	-57.9849	1.6899	100	-34.31	<.0001	0.05	-61.3376	-54.6322
CFU-GEMM	Ao-EC Only	CFU-GM	I-EC Only	-11.3637	0.06423	100	-176.91	<.0001	0.05	-11.4911	-11.2363
CFU-GEMM	Ao-EC Only	CFU-GM	I-EC+10.5MesP	-55.5663	1.6918	100	-32.85	<.0001	0.05	-58.9227	-52.2099
CFU-GEMM	Ao-EC Only	CFU-GM	I-EC+11.5MesP	-54.8753	1.6938	100	-32.40	<.0001	0.05	-58.2357	-51.5148

The GLIMMIX Procedure

Differences of Colony_Typ*Condition Least Squares Means											
Colony_Type	Condition	_Colony_Type	_Condition	Estimate	Standard Error	DF	t Value	Pr > |t|	Alpha	Lower	Upper
CFU-GEMM	Ao-EC Only	CFU-GM	L-E only	-11.3637	0.06423	100	-176.91	<.0001	0.05	-11.4911	-11.2363
CFU-GEMM	Ao-EC Only	CFU-GM	L-E+11.5Mesp1	-58.1441	0.06423	100	-905.18	<.0001	0.05	-58.2715	-58.0166
CFU-GEMM	AoEC+10.5MesP	CFU-GEMM	AoEC+11.5MesP	-0.1609	1.6912	100	-0.10	0.9244	0.05	-3.5161	3.1944
CFU-GEMM	AoEC+10.5MesP	CFU-GEMM	E10.5-EC Only	56.1001	1.6912	100	33.17	<.0001	0.05	52.7448	59.4555
CFU-GEMM	AoEC+10.5MesP	CFU-GEMM	E10.5-MesP1+	56.1001	1.6912	100	33.17	<.0001	0.05	52.7448	59.4555
CFU-GEMM	AoEC+10.5MesP	CFU-GEMM	E10.5-MesP1-	56.1001	1.6912	100	33.17	<.0001	0.05	52.7448	59.4555
CFU-GEMM	AoEC+10.5MesP	CFU-GEMM	E11.5MesP1+On	56.1001	1.6912	100	33.17	<.0001	0.05	52.7448	59.4555
CFU-GEMM	AoEC+10.5MesP	CFU-GEMM	E11.5MesP1-On	56.1001	1.6912	100	33.17	<.0001	0.05	52.7448	59.4555
CFU-GEMM	AoEC+10.5MesP	CFU-GEMM	E13.5 EC Only	56.1001	1.6912	100	33.17	<.0001	0.05	52.7448	59.4555
CFU-GEMM	AoEC+10.5MesP	CFU-GEMM	H-EC Only	56.1001	1.6912	100	33.17	<.0001	0.05	52.7448	59.4555
CFU-GEMM	AoEC+10.5MesP	CFU-GEMM	H-EC+10.5MesP	0.06867	1.6914	100	0.04	0.9677	0.05	-3.2871	3.4244
CFU-GEMM	AoEC+10.5MesP	CFU-GEMM	H-EC+11.5MesP	0.1773	1.6916	100	0.10	0.9167	0.05	-3.1788	3.5334
CFU-GEMM	AoEC+10.5MesP	CFU-GEMM	I-EC Only	56.1001	1.6912	100	33.17	<.0001	0.05	52.7448	59.4555
CFU-GEMM	AoEC+10.5MesP	CFU-GEMM	I-EC+10.5MesP	1.3577	1.6940	100	0.80	0.4247	0.05	-2.0031	4.7186
CFU-GEMM	AoEC+10.5MesP	CFU-GEMM	I-EC+11.5MesP	2.0900	1.6989	100	1.23	0.2215	0.05	-1.2807	5.4606
CFU-GEMM	AoEC+10.5MesP	CFU-GEMM	L-E only	56.1001	1.6912	100	33.17	<.0001	0.05	52.7448	59.4555
CFU-GEMM	AoEC+10.5MesP	CFU-GEMM	L-E+11.5Mesp1	-0.09853	1.6912	100	-0.06	0.9537	0.05	-3.4539	3.2568
CFU-GEMM	AoEC+10.5MesP	CFU-GM	10.5EC+!0.5Me	1.6360	1.5117	100	1.08	0.2818	0.05	-1.3631	4.6351
CFU-GEMM	AoEC+10.5MesP	CFU-GM	11.5 EC Only	1.1899	1.6938	100	0.70	0.4840	0.05	-2.1705	4.5503
CFU-GEMM	AoEC+10.5MesP	CFU-GM	11.5EC+11.5Me	1.5860	1.5114	100	1.05	0.2965	0.05	-1.4126	4.5846
CFU-GEMM	AoEC+10.5MesP	CFU-GM	13.5EC+10.5Me	-1.6420	1.6901	100	-0.97	0.3336	0.05	-4.9951	1.7111
CFU-GEMM	AoEC+10.5MesP	CFU-GM	13.5EC+11.5Me	-1.9455	1.6900	100	-1.15	0.2524	0.05	-5.2984	1.4075
CFU-GEMM	AoEC+10.5MesP	CFU-GM	Ao-EC Only	44.7364	1.6900	100	26.47	<.0001	0.05	41.3835	48.0894
CFU-GEMM	AoEC+10.5MesP	CFU-GM	AoEC+10.5MesP	-1.9994	0.06712	100	-29.79	<.0001	0.05	-2.1326	-1.8663

The GLIMMIX Procedure

Differences of Colony_Typ*Condition Least Squares Means											
Colony_Type	Condition	_Colony_Type	_Condition	Estimate	Standard Error	DF	t Value	Pr > |t|	Alpha	Lower	Upper
CFU-GEMM	AoEC+10.5MesP	CFU-GM	AoEC+11.5MesP	-1.9667	1.6900	100	-1.16	0.2473	0.05	-5.3196	1.3863
CFU-GEMM	AoEC+10.5MesP	CFU-GM	E10.5-EC Only	44.7364	1.6900	100	26.47	<.0001	0.05	41.3835	48.0894
CFU-GEMM	AoEC+10.5MesP	CFU-GM	E10.5-MesP1+	44.7364	1.6900	100	26.47	<.0001	0.05	41.3835	48.0894
CFU-GEMM	AoEC+10.5MesP	CFU-GM	E10.5-MesP1-	44.7364	1.6900	100	26.47	<.0001	0.05	41.3835	48.0894
CFU-GEMM	AoEC+10.5MesP	CFU-GM	E11.5MesP1+On	44.7364	1.6900	100	26.47	<.0001	0.05	41.3835	48.0894
CFU-GEMM	AoEC+10.5MesP	CFU-GM	E11.5MesP1-On	44.7364	1.6900	100	26.47	<.0001	0.05	41.3835	48.0894
CFU-GEMM	AoEC+10.5MesP	CFU-GM	E13.5 EC Only	44.7364	1.6900	100	26.47	<.0001	0.05	41.3835	48.0894
CFU-GEMM	AoEC+10.5MesP	CFU-GM	H-EC Only	44.7364	1.6900	100	26.47	<.0001	0.05	41.3835	48.0894
CFU-GEMM	AoEC+10.5MesP	CFU-GM	H-EC+10.5MesP	-1.9811	1.6900	100	-1.17	0.2439	0.05	-5.3340	1.3718
CFU-GEMM	AoEC+10.5MesP	CFU-GM	H-EC+11.5MesP	-1.8848	1.6900	100	-1.12	0.2674	0.05	-5.2377	1.4682
CFU-GEMM	AoEC+10.5MesP	CFU-GM	I-EC Only	44.7364	1.6900	100	26.47	<.0001	0.05	41.3835	48.0894
CFU-GEMM	AoEC+10.5MesP	CFU-GM	I-EC+10.5MesP	0.5338	1.6919	100	0.32	0.7530	0.05	-2.8228	3.8904
CFU-GEMM	AoEC+10.5MesP	CFU-GM	I-EC+11.5MesP	1.2248	1.6939	100	0.72	0.4713	0.05	-2.1359	4.5855
CFU-GEMM	AoEC+10.5MesP	CFU-GM	L-E only	44.7364	1.6900	100	26.47	<.0001	0.05	41.3835	48.0894
CFU-GEMM	AoEC+10.5MesP	CFU-GM	L-E+11.5Mesp1	-2.0439	1.6900	100	-1.21	0.2294	0.05	-5.3968	1.3090
CFU-GEMM	AoEC+11.5MesP	CFU-GEMM	E10.5-EC Only	56.2610	1.6911	100	33.27	<.0001	0.05	52.9060	59.6160
CFU-GEMM	AoEC+11.5MesP	CFU-GEMM	E10.5-MesP1+	56.2610	1.6911	100	33.27	<.0001	0.05	52.9060	59.6160
CFU-GEMM	AoEC+11.5MesP	CFU-GEMM	E10.5-MesP1-	56.2610	1.6911	100	33.27	<.0001	0.05	52.9060	59.6160
CFU-GEMM	AoEC+11.5MesP	CFU-GEMM	E11.5MesP1+On	56.2610	1.6911	100	33.27	<.0001	0.05	52.9060	59.6160
CFU-GEMM	AoEC+11.5MesP	CFU-GEMM	E11.5MesP1-On	56.2610	1.6911	100	33.27	<.0001	0.05	52.9060	59.6160
CFU-GEMM	AoEC+11.5MesP	CFU-GEMM	E13.5 EC Only	56.2610	1.6911	100	33.27	<.0001	0.05	52.9060	59.6160
CFU-GEMM	AoEC+11.5MesP	CFU-GEMM	H-EC Only	56.2610	1.6911	100	33.27	<.0001	0.05	52.9060	59.6160
CFU-GEMM	AoEC+11.5MesP	CFU-GEMM	H-EC+10.5MesP	0.2296	1.6913	100	0.14	0.8923	0.05	-3.1259	3.5850
CFU-GEMM	AoEC+11.5MesP	CFU-GEMM	H-EC+11.5MesP	0.3382	1.6915	100	0.20	0.8419	0.05	-3.0176	3.6940

The GLIMMIX Procedure

Differences of Colony_Typ*Condition Least Squares Means											
Colony_Type	Condition	_Colony_Type	_Condition	Estimate	Standard Error	DF	t Value	Pr > |t|	Alpha	Lower	Upper
CFU-GEMM	AoEC+11.5MesP	CFU-GEMM	I-EC Only	56.2610	1.6911	100	33.27	<.0001	0.05	52.9060	59.6160
CFU-GEMM	AoEC+11.5MesP	CFU-GEMM	I-EC+10.5MesP	1.5186	1.6939	100	0.90	0.3721	0.05	-1.8419	4.8792
CFU-GEMM	AoEC+11.5MesP	CFU-GEMM	I-EC+11.5MesP	2.2509	1.6988	100	1.32	0.1882	0.05	-1.1195	5.6212
CFU-GEMM	AoEC+11.5MesP	CFU-GEMM	L-E only	56.2610	1.6911	100	33.27	<.0001	0.05	52.9060	59.6160
CFU-GEMM	AoEC+11.5MesP	CFU-GEMM	L-E+11.5Mesp1	0.06237	1.6911	100	0.04	0.9707	0.05	-3.2926	3.4174
CFU-GEMM	AoEC+11.5MesP	CFU-GM	10.5EC+!0.5Me	1.7969	1.5115	100	1.19	0.2373	0.05	-1.2020	4.7957
CFU-GEMM	AoEC+11.5MesP	CFU-GM	11.5 EC Only	1.3508	1.6936	100	0.80	0.4270	0.05	-2.0093	4.7109
CFU-GEMM	AoEC+11.5MesP	CFU-GM	11.5EC+11.5Me	1.7469	1.5113	100	1.16	0.2505	0.05	-1.2514	4.7452
CFU-GEMM	AoEC+11.5MesP	CFU-GM	13.5EC+10.5Me	-1.4811	1.6899	100	-0.88	0.3829	0.05	-4.8339	1.8716
CFU-GEMM	AoEC+11.5MesP	CFU-GM	13.5EC+11.5Me	-1.7846	1.6899	100	-1.06	0.2935	0.05	-5.1372	1.5681
CFU-GEMM	AoEC+11.5MesP	CFU-GM	Ao-EC Only	44.8973	1.6898	100	26.57	<.0001	0.05	41.5447	48.2499
CFU-GEMM	AoEC+11.5MesP	CFU-GM	AoEC+10.5MesP	-1.8385	1.6898	100	-1.09	0.2792	0.05	-5.1911	1.5141
CFU-GEMM	AoEC+11.5MesP	CFU-GM	AoEC+11.5MesP	-1.8058	0.06282	100	-28.74	<.0001	0.05	-1.9304	-1.6811
CFU-GEMM	AoEC+11.5MesP	CFU-GM	E10.5-EC Only	44.8973	1.6898	100	26.57	<.0001	0.05	41.5447	48.2499
CFU-GEMM	AoEC+11.5MesP	CFU-GM	E10.5-MesP1+	44.8973	1.6898	100	26.57	<.0001	0.05	41.5447	48.2499
CFU-GEMM	AoEC+11.5MesP	CFU-GM	E10.5-MesP1-	44.8973	1.6898	100	26.57	<.0001	0.05	41.5447	48.2499
CFU-GEMM	AoEC+11.5MesP	CFU-GM	E11.5MesP1+On	44.8973	1.6898	100	26.57	<.0001	0.05	41.5447	48.2499
CFU-GEMM	AoEC+11.5MesP	CFU-GM	E11.5MesP1-On	44.8973	1.6898	100	26.57	<.0001	0.05	41.5447	48.2499
CFU-GEMM	AoEC+11.5MesP	CFU-GM	E13.5 EC Only	44.8973	1.6898	100	26.57	<.0001	0.05	41.5447	48.2499
CFU-GEMM	AoEC+11.5MesP	CFU-GM	H-EC Only	44.8973	1.6898	100	26.57	<.0001	0.05	41.5447	48.2499
CFU-GEMM	AoEC+11.5MesP	CFU-GM	H-EC+10.5MesP	-1.8202	1.6898	100	-1.08	0.2840	0.05	-5.1728	1.5324
CFU-GEMM	AoEC+11.5MesP	CFU-GM	H-EC+11.5MesP	-1.7239	1.6899	100	-1.02	0.3101	0.05	-5.0765	1.6288
CFU-GEMM	AoEC+11.5MesP	CFU-GM	I-EC Only	44.8973	1.6898	100	26.57	<.0001	0.05	41.5447	48.2499
CFU-GEMM	AoEC+11.5MesP	CFU-GM	I-EC+10.5MesP	0.6947	1.6917	100	0.41	0.6822	0.05	-2.6616	4.0510

The GLIMMIX Procedure

Differences of Colony_Typ*Condition Least Squares Means											
**Colony_Type**	**Condition**	**_Colony_Type**	**_Condition**	**Estimate**	**Standard Error**	**DF**	**t Value**	**Pr > |t|**	**Alpha**	**Lower**	**Upper**
CFU-GEMM	AoEC+11.5MesP	CFU-GM	I-EC+11.5MesP	1.3857	1.6938	100	0.82	0.4152	0.05	-1.9746	4.7461
CFU-GEMM	AoEC+11.5MesP	CFU-GM	L-E only	44.8973	1.6898	100	26.57	<.0001	0.05	41.5447	48.2499
CFU-GEMM	AoEC+11.5MesP	CFU-GM	L-E+11.5Mesp1	-1.8830	1.6898	100	-1.11	0.2678	0.05	-5.2356	1.4696
CFU-GEMM	E10.5-EC Only	CFU-GEMM	E10.5-MesP1+	-533E-16	.	.	.	.	.	.	.
CFU-GEMM	E10.5-EC Only	CFU-GEMM	E10.5-MesP1-	-107E-16	.	.	.	.	.	.	.
CFU-GEMM	E10.5-EC Only	CFU-GEMM	E11.5MesP1+On	2.31E-14	.	.	.	.	.	.	.
CFU-GEMM	E10.5-EC Only	CFU-GEMM	E11.5MesP1-On	-87E-15	.	.	.	.	.	.	.
CFU-GEMM	E10.5-EC Only	CFU-GEMM	E13.5 EC Only	1.1E-13	.	.	.	.	.	.	.
CFU-GEMM	E10.5-EC Only	CFU-GEMM	H-EC Only	2.74E-13	.	.	.	.	.	.	.
CFU-GEMM	E10.5-EC Only	CFU-GEMM	H-EC+10.5MesP	-56.0315	1.6913	100	-33.13	<.0001	0.05	-59.3870	-52.6760
CFU-GEMM	E10.5-EC Only	CFU-GEMM	H-EC+11.5MesP	-55.9229	1.6915	100	-33.06	<.0001	0.05	-59.2787	-52.5670
CFU-GEMM	E10.5-EC Only	CFU-GEMM	I-EC Only	9.24E-14	.	.	.	.	.	.	.
CFU-GEMM	E10.5-EC Only	CFU-GEMM	I-EC+10.5MesP	-54.7424	1.6939	100	-32.32	<.0001	0.05	-58.1030	-51.3818
CFU-GEMM	E10.5-EC Only	CFU-GEMM	I-EC+11.5MesP	-54.0102	1.6988	100	-31.79	<.0001	0.05	-57.3806	-50.6397
CFU-GEMM	E10.5-EC Only	CFU-GEMM	L-E only	8.7E-14	.	.	.	.	.	.	.
CFU-GEMM	E10.5-EC Only	CFU-GEMM	L-E+11.5Mesp1	-56.1987	0	100	-Infty	<.0001	.	.	.
CFU-GEMM	E10.5-EC Only	CFU-GM	10.5EC+!0.5Me	-54.4642	1.5116	100	-36.03	<.0001	0.05	-57.4631	-51.4652
CFU-GEMM	E10.5-EC Only	CFU-GM	11.5 EC Only	-54.9102	1.6937	100	-32.42	<.0001	0.05	-58.2704	-51.5501
CFU-GEMM	E10.5-EC Only	CFU-GM	11.5EC+11.5Me	-54.5141	1.5113	100	-36.07	<.0001	0.05	-57.5125	-51.5157
CFU-GEMM	E10.5-EC Only	CFU-GM	13.5EC+10.5Me	-57.7421	1.6900	100	-34.17	<.0001	0.05	-61.0950	-54.3893
CFU-GEMM	E10.5-EC Only	CFU-GM	13.5EC+11.5Me	-58.0456	1.6899	100	-34.35	<.0001	0.05	-61.3983	-54.6929
CFU-GEMM	E10.5-EC Only	CFU-GM	Ao-EC Only	-11.3637	0.06423	100	-176.91	<.0001	0.05	-11.4911	-11.2363
CFU-GEMM	E10.5-EC Only	CFU-GM	AoEC+10.5MesP	-58.0996	1.6899	100	-34.38	<.0001	0.05	-61.4522	-54.7469
CFU-GEMM	E10.5-EC Only	CFU-GM	AoEC+11.5MesP	-58.0668	1.6899	100	-34.36	<.0001	0.05	-61.4195	-54.7141

The GLIMMIX Procedure

Differences of Colony_Typ*Condition Least Squares Means											
Colony_Type	Condition	_Colony_Type	_Condition	Estimate	Standard Error	DF	t Value	Pr > |t|	Alpha	Lower	Upper
CFU-GEMM	E10.5-EC Only	CFU-GM	E10.5-EC Only	-11.3637	0.06423	100	-176.91	<.0001	0.05	-11.4911	-11.2363
CFU-GEMM	E10.5-EC Only	CFU-GM	E10.5-MesP1+	-11.3637	0.06423	100	-176.91	<.0001	0.05	-11.4911	-11.2363
CFU-GEMM	E10.5-EC Only	CFU-GM	E10.5-MesP1-	-11.3637	0.06423	100	-176.91	<.0001	0.05	-11.4911	-11.2363
CFU-GEMM	E10.5-EC Only	CFU-GM	E11.5MesP1+On	-11.3637	0.06423	100	-176.91	<.0001	0.05	-11.4911	-11.2363
CFU-GEMM	E10.5-EC Only	CFU-GM	E11.5MesP1-On	-11.3637	0.06423	100	-176.91	<.0001	0.05	-11.4911	-11.2363
CFU-GEMM	E10.5-EC Only	CFU-GM	E13.5 EC Only	-11.3637	0.06423	100	-176.91	<.0001	0.05	-11.4911	-11.2363
CFU-GEMM	E10.5-EC Only	CFU-GM	H-EC Only	-11.3637	0.06423	100	-176.91	<.0001	0.05	-11.4911	-11.2363
CFU-GEMM	E10.5-EC Only	CFU-GM	H-EC+10.5MesP	-58.0812	1.6899	100	-34.37	<.0001	0.05	-61.4339	-54.7285
CFU-GEMM	E10.5-EC Only	CFU-GM	H-EC+11.5MesP	-57.9849	1.6899	100	-34.31	<.0001	0.05	-61.3376	-54.6322
CFU-GEMM	E10.5-EC Only	CFU-GM	I-EC Only	-11.3637	0.06423	100	-176.91	<.0001	0.05	-11.4911	-11.2363
CFU-GEMM	E10.5-EC Only	CFU-GM	I-EC+10.5MesP	-55.5663	1.6918	100	-32.85	<.0001	0.05	-58.9227	-52.2099
CFU-GEMM	E10.5-EC Only	CFU-GM	I-EC+11.5MesP	-54.8753	1.6938	100	-32.40	<.0001	0.05	-58.2357	-51.5148
CFU-GEMM	E10.5-EC Only	CFU-GM	L-E only	-11.3637	0.06423	100	-176.91	<.0001	0.05	-11.4911	-11.2363
CFU-GEMM	E10.5-EC Only	CFU-GM	L-E+11.5Mesp1	-58.1441	0.06423	100	-905.18	<.0001	0.05	-58.2715	-58.0166
CFU-GEMM	E10.5-MesP1+	CFU-GEMM	E10.5-MesP1-	4.26E-14	.	.	.	.	.	.	.
CFU-GEMM	E10.5-MesP1+	CFU-GEMM	E11.5MesP1+On	7.64E-14	.	.	.	.	.	.	.
CFU-GEMM	E10.5-MesP1+	CFU-GEMM	E11.5MesP1-On	-338E-16	.	.	.	.	.	.	.
CFU-GEMM	E10.5-MesP1+	CFU-GEMM	E13.5 EC Only	1.63E-13	.	.	.	.	.	.	.
CFU-GEMM	E10.5-MesP1+	CFU-GEMM	H-EC Only	3.27E-13	.	.	.	.	.	.	.
CFU-GEMM	E10.5-MesP1+	CFU-GEMM	H-EC+10.5MesP	-56.0315	1.6913	100	-33.13	<.0001	0.05	-59.3870	-52.6760
CFU-GEMM	E10.5-MesP1+	CFU-GEMM	H-EC+11.5MesP	-55.9229	1.6915	100	-33.06	<.0001	0.05	-59.2787	-52.5670
CFU-GEMM	E10.5-MesP1+	CFU-GEMM	I-EC Only	1.46E-13	.	.	.	.	.	.	.
CFU-GEMM	E10.5-MesP1+	CFU-GEMM	I-EC+10.5MesP	-54.7424	1.6939	100	-32.32	<.0001	0.05	-58.1030	-51.3818
CFU-GEMM	E10.5-MesP1+	CFU-GEMM	I-EC+11.5MesP	-54.0102	1.6988	100	-31.79	<.0001	0.05	-57.3806	-50.6397

The GLIMMIX Procedure

Differences of Colony_Typ*Condition Least Squares Means											
Colony_Type	Condition	_Colony_Type	_Condition	Estimate	Standard Error	DF	t Value	Pr > |t|	Alpha	Lower	Upper
CFU-GEMM	E10.5-MesP1+	CFU-GEMM	L-E only	1.4E-13	.	.	.	.	.	.	.
CFU-GEMM	E10.5-MesP1+	CFU-GEMM	L-E+11.5Mesp1	-56.1987	0	100	-Infty	<.0001	.	.	.
CFU-GEMM	E10.5-MesP1+	CFU-GM	10.5EC+!0.5Me	-54.4642	1.5116	100	-36.03	<.0001	0.05	-57.4631	-51.4652
CFU-GEMM	E10.5-MesP1+	CFU-GM	11.5 EC Only	-54.9102	1.6937	100	-32.42	<.0001	0.05	-58.2704	-51.5501
CFU-GEMM	E10.5-MesP1+	CFU-GM	11.5EC+11.5Me	-54.5141	1.5113	100	-36.07	<.0001	0.05	-57.5125	-51.5157
CFU-GEMM	E10.5-MesP1+	CFU-GM	13.5EC+10.5Me	-57.7421	1.6900	100	-34.17	<.0001	0.05	-61.0950	-54.3893
CFU-GEMM	E10.5-MesP1+	CFU-GM	13.5EC+11.5Me	-58.0456	1.6899	100	-34.35	<.0001	0.05	-61.3983	-54.6929
CFU-GEMM	E10.5-MesP1+	CFU-GM	Ao-EC Only	-11.3637	0.06423	100	-176.91	<.0001	0.05	-11.4911	-11.2363
CFU-GEMM	E10.5-MesP1+	CFU-GM	AoEC+10.5MesP	-58.0996	1.6899	100	-34.38	<.0001	0.05	-61.4522	-54.7469
CFU-GEMM	E10.5-MesP1+	CFU-GM	AoEC+11.5MesP	-58.0668	1.6899	100	-34.36	<.0001	0.05	-61.4195	-54.7141
CFU-GEMM	E10.5-MesP1+	CFU-GM	E10.5-EC Only	-11.3637	0.06423	100	-176.91	<.0001	0.05	-11.4911	-11.2363
CFU-GEMM	E10.5-MesP1+	CFU-GM	E10.5-MesP1+	-11.3637	0.06423	100	-176.91	<.0001	0.05	-11.4911	-11.2363
CFU-GEMM	E10.5-MesP1+	CFU-GM	E10.5-MesP1-	-11.3637	0.06423	100	-176.91	<.0001	0.05	-11.4911	-11.2363
CFU-GEMM	E10.5-MesP1+	CFU-GM	E11.5MesP1+On	-11.3637	0.06423	100	-176.91	<.0001	0.05	-11.4911	-11.2363
CFU-GEMM	E10.5-MesP1+	CFU-GM	E11.5MesP1-On	-11.3637	0.06423	100	-176.91	<.0001	0.05	-11.4911	-11.2363
CFU-GEMM	E10.5-MesP1+	CFU-GM	E13.5 EC Only	-11.3637	0.06423	100	-176.91	<.0001	0.05	-11.4911	-11.2363
CFU-GEMM	E10.5-MesP1+	CFU-GM	H-EC Only	-11.3637	0.06423	100	-176.91	<.0001	0.05	-11.4911	-11.2363
CFU-GEMM	E10.5-MesP1+	CFU-GM	H-EC+10.5MesP	-58.0812	1.6899	100	-34.37	<.0001	0.05	-61.4339	-54.7285
CFU-GEMM	E10.5-MesP1+	CFU-GM	H-EC+11.5MesP	-57.9849	1.6899	100	-34.31	<.0001	0.05	-61.3376	-54.6322
CFU-GEMM	E10.5-MesP1+	CFU-GM	I-EC Only	-11.3637	0.06423	100	-176.91	<.0001	0.05	-11.4911	-11.2363
CFU-GEMM	E10.5-MesP1+	CFU-GM	I-EC+10.5MesP	-55.5663	1.6918	100	-32.85	<.0001	0.05	-58.9227	-52.2099
CFU-GEMM	E10.5-MesP1+	CFU-GM	I-EC+11.5MesP	-54.8753	1.6938	100	-32.40	<.0001	0.05	-58.2357	-51.5148
CFU-GEMM	E10.5-MesP1+	CFU-GM	L-E only	-11.3637	0.06423	100	-176.91	<.0001	0.05	-11.4911	-11.2363
CFU-GEMM	E10.5-MesP1+	CFU-GM	L-E+11.5Mesp1	-58.1441	0.06423	100	-905.18	<.0001	0.05	-58.2715	-58.0166

The GLIMMIX Procedure

Differences of Colony_Typ*Condition Least Squares Means											
Colony_Type	Condition	_Colony_Type	_Condition	Estimate	Standard Error	DF	t Value	Pr > |t|	Alpha	Lower	Upper
CFU-GEMM	E10.5-MesP1-	CFU-GEMM	E11.5MesP1+On	3.38E-14	.	.	.	.	.	.	.
CFU-GEMM	E10.5-MesP1-	CFU-GEMM	E11.5MesP1-On	-764E-16	.	.	.	.	.	.	.
CFU-GEMM	E10.5-MesP1-	CFU-GEMM	E13.5 EC Only	1.21E-13	.	.	.	.	.	.	.
CFU-GEMM	E10.5-MesP1-	CFU-GEMM	H-EC Only	2.84E-13	.	.	.	.	.	.	.
CFU-GEMM	E10.5-MesP1-	CFU-GEMM	H-EC+10.5MesP	-56.0315	1.6913	100	-33.13	<.0001	0.05	-59.3870	-52.6760
CFU-GEMM	E10.5-MesP1-	CFU-GEMM	H-EC+11.5MesP	-55.9229	1.6915	100	-33.06	<.0001	0.05	-59.2787	-52.5670
CFU-GEMM	E10.5-MesP1-	CFU-GEMM	I-EC Only	1.03E-13	.	.	.	.	.	.	.
CFU-GEMM	E10.5-MesP1-	CFU-GEMM	I-EC+10.5MesP	-54.7424	1.6939	100	-32.32	<.0001	0.05	-58.1030	-51.3818
CFU-GEMM	E10.5-MesP1-	CFU-GEMM	I-EC+11.5MesP	-54.0102	1.6988	100	-31.79	<.0001	0.05	-57.3806	-50.6397
CFU-GEMM	E10.5-MesP1-	CFU-GEMM	L-E only	9.77E-14	.	.	.	.	.	.	.
CFU-GEMM	E10.5-MesP1-	CFU-GEMM	L-E+11.5Mesp1	-56.1987	0	100	-Infty	<.0001	.	.	.
CFU-GEMM	E10.5-MesP1-	CFU-GM	10.5EC+!0.5Me	-54.4642	1.5116	100	-36.03	<.0001	0.05	-57.4631	-51.4652
CFU-GEMM	E10.5-MesP1-	CFU-GM	11.5 EC Only	-54.9102	1.6937	100	-32.42	<.0001	0.05	-58.2704	-51.5501
CFU-GEMM	E10.5-MesP1-	CFU-GM	11.5EC+11.5Me	-54.5141	1.5113	100	-36.07	<.0001	0.05	-57.5125	-51.5157
CFU-GEMM	E10.5-MesP1-	CFU-GM	13.5EC+10.5Me	-57.7421	1.6900	100	-34.17	<.0001	0.05	-61.0950	-54.3893
CFU-GEMM	E10.5-MesP1-	CFU-GM	13.5EC+11.5Me	-58.0456	1.6899	100	-34.35	<.0001	0.05	-61.3983	-54.6929
CFU-GEMM	E10.5-MesP1-	CFU-GM	Ao-EC Only	-11.3637	0.06423	100	-176.91	<.0001	0.05	-11.4911	-11.2363
CFU-GEMM	E10.5-MesP1-	CFU-GM	AoEC+10.5MesP	-58.0996	1.6899	100	-34.38	<.0001	0.05	-61.4522	-54.7469
CFU-GEMM	E10.5-MesP1-	CFU-GM	AoEC+11.5MesP	-58.0668	1.6899	100	-34.36	<.0001	0.05	-61.4195	-54.7141
CFU-GEMM	E10.5-MesP1-	CFU-GM	E10.5-EC Only	-11.3637	0.06423	100	-176.91	<.0001	0.05	-11.4911	-11.2363
CFU-GEMM	E10.5-MesP1-	CFU-GM	E10.5-MesP1+	-11.3637	0.06423	100	-176.91	<.0001	0.05	-11.4911	-11.2363
CFU-GEMM	E10.5-MesP1-	CFU-GM	E10.5-MesP1-	-11.3637	0.06423	100	-176.91	<.0001	0.05	-11.4911	-11.2363
CFU-GEMM	E10.5-MesP1-	CFU-GM	E11.5MesP1+On	-11.3637	0.06423	100	-176.91	<.0001	0.05	-11.4911	-11.2363
CFU-GEMM	E10.5-MesP1-	CFU-GM	E11.5MesP1-On	-11.3637	0.06423	100	-176.91	<.0001	0.05	-11.4911	-11.2363

The GLIMMIX Procedure

Differences of Colony_Typ*Condition Least Squares Means

Colony_Type	Condition	_Colony_Type	_Condition	Estimate	Standard Error	DF	t Value	Pr > |t|	Alpha	Lower	Upper
CFU-GEMM	E10.5-MesP1-	CFU-GM	E13.5 EC Only	-11.3637	0.06423	100	-176.91	<.0001	0.05	-11.4911	-11.2363
CFU-GEMM	E10.5-MesP1-	CFU-GM	H-EC Only	-11.3637	0.06423	100	-176.91	<.0001	0.05	-11.4911	-11.2363
CFU-GEMM	E10.5-MesP1-	CFU-GM	H-EC+10.5MesP	-58.0812	1.6899	100	-34.37	<.0001	0.05	-61.4339	-54.7285
CFU-GEMM	E10.5-MesP1-	CFU-GM	H-EC+11.5MesP	-57.9849	1.6899	100	-34.31	<.0001	0.05	-61.3376	-54.6322
CFU-GEMM	E10.5-MesP1-	CFU-GM	I-EC Only	-11.3637	0.06423	100	-176.91	<.0001	0.05	-11.4911	-11.2363
CFU-GEMM	E10.5-MesP1-	CFU-GM	I-EC+10.5MesP	-55.5663	1.6918	100	-32.85	<.0001	0.05	-58.9227	-52.2099
CFU-GEMM	E10.5-MesP1-	CFU-GM	I-EC+11.5MesP	-54.8753	1.6938	100	-32.40	<.0001	0.05	-58.2357	-51.5148
CFU-GEMM	E10.5-MesP1-	CFU-GM	L-E only	-11.3637	0.06423	100	-176.91	<.0001	0.05	-11.4911	-11.2363
CFU-GEMM	E10.5-MesP1-	CFU-GM	L-E+11.5Mesp1	-58.1441	0.06423	100	-905.18	<.0001	0.05	-58.2715	-58.0166
CFU-GEMM	E11.5MesP1+On	CFU-GEMM	E11.5MesP1-On	-11E-14	.	.	.	.	.	.	.
CFU-GEMM	E11.5MesP1+On	CFU-GEMM	E13.5 EC Only	8.7E-14	.	.	.	.	.	.	.
CFU-GEMM	E11.5MesP1+On	CFU-GEMM	H-EC Only	2.5E-13	.	.	.	.	.	.	.
CFU-GEMM	E11.5MesP1+On	CFU-GEMM	H-EC+10.5MesP	-56.0315	1.6913	100	-33.13	<.0001	0.05	-59.3870	-52.6760
CFU-GEMM	E11.5MesP1+On	CFU-GEMM	H-EC+11.5MesP	-55.9229	1.6915	100	-33.06	<.0001	0.05	-59.2787	-52.5670
CFU-GEMM	E11.5MesP1+On	CFU-GEMM	I-EC Only	6.93E-14	.	.	.	.	.	.	.
CFU-GEMM	E11.5MesP1+On	CFU-GEMM	I-EC+10.5MesP	-54.7424	1.6939	100	-32.32	<.0001	0.05	-58.1030	-51.3818
CFU-GEMM	E11.5MesP1+On	CFU-GEMM	I-EC+11.5MesP	-54.0102	1.6988	100	-31.79	<.0001	0.05	-57.3806	-50.6397
CFU-GEMM	E11.5MesP1+On	CFU-GEMM	L-E only	6.39E-14	.	.	.	.	.	.	.
CFU-GEMM	E11.5MesP1+On	CFU-GEMM	L-E+11.5Mesp1	-56.1987	0	100	-Infty	<.0001	.	.	.
CFU-GEMM	E11.5MesP1+On	CFU-GM	10.5EC+!0.5Me	-54.4642	1.5116	100	-36.03	<.0001	0.05	-57.4631	-51.4652
CFU-GEMM	E11.5MesP1+On	CFU-GM	11.5 EC Only	-54.9102	1.6937	100	-32.42	<.0001	0.05	-58.2704	-51.5501
CFU-GEMM	E11.5MesP1+On	CFU-GM	11.5EC+11.5Me	-54.5141	1.5113	100	-36.07	<.0001	0.05	-57.5125	-51.5157
CFU-GEMM	E11.5MesP1+On	CFU-GM	13.5EC+10.5Me	-57.7421	1.6900	100	-34.17	<.0001	0.05	-61.0950	-54.3893
CFU-GEMM	E11.5MesP1+On	CFU-GM	13.5EC+11.5Me	-58.0456	1.6899	100	-34.35	<.0001	0.05	-61.3983	-54.6929

The GLIMMIX Procedure

Differences of Colony_Typ*Condition Least Squares Means

Colony_Type	Condition	_Colony_Type	_Condition	Estimate	Standard Error	DF	t Value	Pr > |t|	Alpha	Lower	Upper
CFU-GEMM	E11.5MesP1+On	CFU-GM	Ao-EC Only	-11.3637	0.06423	100	-176.91	<.0001	0.05	-11.4911	-11.2363
CFU-GEMM	E11.5MesP1+On	CFU-GM	AoEC+10.5MesP	-58.0996	1.6899	100	-34.38	<.0001	0.05	-61.4522	-54.7469
CFU-GEMM	E11.5MesP1+On	CFU-GM	AoEC+11.5MesP	-58.0668	1.6899	100	-34.36	<.0001	0.05	-61.4195	-54.7141
CFU-GEMM	E11.5MesP1+On	CFU-GM	E10.5-EC Only	-11.3637	0.06423	100	-176.91	<.0001	0.05	-11.4911	-11.2363
CFU-GEMM	E11.5MesP1+On	CFU-GM	E10.5-MesP1+	-11.3637	0.06423	100	-176.91	<.0001	0.05	-11.4911	-11.2363
CFU-GEMM	E11.5MesP1+On	CFU-GM	E10.5-MesP1-	-11.3637	0.06423	100	-176.91	<.0001	0.05	-11.4911	-11.2363
CFU-GEMM	E11.5MesP1+On	CFU-GM	E11.5MesP1+On	-11.3637	0.06423	100	-176.91	<.0001	0.05	-11.4911	-11.2363
CFU-GEMM	E11.5MesP1+On	CFU-GM	E11.5MesP1-On	-11.3637	0.06423	100	-176.91	<.0001	0.05	-11.4911	-11.2363
CFU-GEMM	E11.5MesP1+On	CFU-GM	E13.5 EC Only	-11.3637	0.06423	100	-176.91	<.0001	0.05	-11.4911	-11.2363
CFU-GEMM	E11.5MesP1+On	CFU-GM	H-EC Only	-11.3637	0.06423	100	-176.91	<.0001	0.05	-11.4911	-11.2363
CFU-GEMM	E11.5MesP1+On	CFU-GM	H-EC+10.5MesP	-58.0812	1.6899	100	-34.37	<.0001	0.05	-61.4339	-54.7285
CFU-GEMM	E11.5MesP1+On	CFU-GM	H-EC+11.5MesP	-57.9849	1.6899	100	-34.31	<.0001	0.05	-61.3376	-54.6322
CFU-GEMM	E11.5MesP1+On	CFU-GM	I-EC Only	-11.3637	0.06423	100	-176.91	<.0001	0.05	-11.4911	-11.2363
CFU-GEMM	E11.5MesP1+On	CFU-GM	I-EC+10.5MesP	-55.5663	1.6918	100	-32.85	<.0001	0.05	-58.9227	-52.2099
CFU-GEMM	E11.5MesP1+On	CFU-GM	I-EC+11.5MesP	-54.8753	1.6938	100	-32.40	<.0001	0.05	-58.2357	-51.5148
CFU-GEMM	E11.5MesP1+On	CFU-GM	L-E only	-11.3637	0.06423	100	-176.91	<.0001	0.05	-11.4911	-11.2363
CFU-GEMM	E11.5MesP1+On	CFU-GM	L-E+11.5Mesp1	-58.1441	0.06423	100	-905.18	<.0001	0.05	-58.2715	-58.0166
CFU-GEMM	E11.5MesP1-On	CFU-GEMM	E13.5 EC Only	1.97E-13	.	.	.	.	.	.	.
CFU-GEMM	E11.5MesP1-On	CFU-GEMM	H-EC Only	3.61E-13	.	.	.	.	.	.	.
CFU-GEMM	E11.5MesP1-On	CFU-GEMM	H-EC+10.5MesP	-56.0315	1.6913	100	-33.13	<.0001	0.05	-59.3870	-52.6760
CFU-GEMM	E11.5MesP1-On	CFU-GEMM	H-EC+11.5MesP	-55.9229	1.6915	100	-33.06	<.0001	0.05	-59.2787	-52.5670
CFU-GEMM	E11.5MesP1-On	CFU-GEMM	I-EC Only	1.79E-13	.	.	.	.	.	.	.
CFU-GEMM	E11.5MesP1-On	CFU-GEMM	I-EC+10.5MesP	-54.7424	1.6939	100	-32.32	<.0001	0.05	-58.1030	-51.3818
CFU-GEMM	E11.5MesP1-On	CFU-GEMM	I-EC+11.5MesP	-54.0102	1.6988	100	-31.79	<.0001	0.05	-57.3806	-50.6397

The GLIMMIX Procedure

Differences of Colony_Typ*Condition Least Squares Means											
Colony_Type	Condition	_Colony_Type	_Condition	Estimate	Standard Error	DF	t Value	Pr > |t|	Alpha	Lower	Upper
CFU-GEMM	E11.5MesP1-On	CFU-GEMM	L-E only	1.74E-13	.	.	.	.	.	.	.
CFU-GEMM	E11.5MesP1-On	CFU-GEMM	L-E+11.5Mesp1	-56.1987	0	100	-Infty	<.0001	.	.	.
CFU-GEMM	E11.5MesP1-On	CFU-GM	10.5EC+!0.5Me	-54.4642	1.5116	100	-36.03	<.0001	0.05	-57.4631	-51.4652
CFU-GEMM	E11.5MesP1-On	CFU-GM	11.5 EC Only	-54.9102	1.6937	100	-32.42	<.0001	0.05	-58.2704	-51.5501
CFU-GEMM	E11.5MesP1-On	CFU-GM	11.5EC+11.5Me	-54.5141	1.5113	100	-36.07	<.0001	0.05	-57.5125	-51.5157
CFU-GEMM	E11.5MesP1-On	CFU-GM	13.5EC+10.5Me	-57.7421	1.6900	100	-34.17	<.0001	0.05	-61.0950	-54.3893
CFU-GEMM	E11.5MesP1-On	CFU-GM	13.5EC+11.5Me	-58.0456	1.6899	100	-34.35	<.0001	0.05	-61.3983	-54.6929
CFU-GEMM	E11.5MesP1-On	CFU-GM	Ao-EC Only	-11.3637	0.06423	100	-176.91	<.0001	0.05	-11.4911	-11.2363
CFU-GEMM	E11.5MesP1-On	CFU-GM	AoEC+10.5MesP	-58.0996	1.6899	100	-34.38	<.0001	0.05	-61.4522	-54.7469
CFU-GEMM	E11.5MesP1-On	CFU-GM	AoEC+11.5MesP	-58.0668	1.6899	100	-34.36	<.0001	0.05	-61.4195	-54.7141
CFU-GEMM	E11.5MesP1-On	CFU-GM	E10.5-EC Only	-11.3637	0.06423	100	-176.91	<.0001	0.05	-11.4911	-11.2363
CFU-GEMM	E11.5MesP1-On	CFU-GM	E10.5-MesP1+	-11.3637	0.06423	100	-176.91	<.0001	0.05	-11.4911	-11.2363
CFU-GEMM	E11.5MesP1-On	CFU-GM	E10.5-MesP1-	-11.3637	0.06423	100	-176.91	<.0001	0.05	-11.4911	-11.2363
CFU-GEMM	E11.5MesP1-On	CFU-GM	E11.5MesP1+On	-11.3637	0.06423	100	-176.91	<.0001	0.05	-11.4911	-11.2363
CFU-GEMM	E11.5MesP1-On	CFU-GM	E11.5MesP1-On	-11.3637	0.06423	100	-176.91	<.0001	0.05	-11.4911	-11.2363
CFU-GEMM	E11.5MesP1-On	CFU-GM	E13.5 EC Only	-11.3637	0.06423	100	-176.91	<.0001	0.05	-11.4911	-11.2363
CFU-GEMM	E11.5MesP1-On	CFU-GM	H-EC Only	-11.3637	0.06423	100	-176.91	<.0001	0.05	-11.4911	-11.2363
CFU-GEMM	E11.5MesP1-On	CFU-GM	H-EC+10.5MesP	-58.0812	1.6899	100	-34.37	<.0001	0.05	-61.4339	-54.7285
CFU-GEMM	E11.5MesP1-On	CFU-GM	H-EC+11.5MesP	-57.9849	1.6899	100	-34.31	<.0001	0.05	-61.3376	-54.6322
CFU-GEMM	E11.5MesP1-On	CFU-GM	I-EC Only	-11.3637	0.06423	100	-176.91	<.0001	0.05	-11.4911	-11.2363
CFU-GEMM	E11.5MesP1-On	CFU-GM	I-EC+10.5MesP	-55.5663	1.6918	100	-32.85	<.0001	0.05	-58.9227	-52.2099
CFU-GEMM	E11.5MesP1-On	CFU-GM	I-EC+11.5MesP	-54.8753	1.6938	100	-32.40	<.0001	0.05	-58.2357	-51.5148
CFU-GEMM	E11.5MesP1-On	CFU-GM	L-E only	-11.3637	0.06423	100	-176.91	<.0001	0.05	-11.4911	-11.2363
CFU-GEMM	E11.5MesP1-On	CFU-GM	L-E+11.5Mesp1	-58.1441	0.06423	100	-905.18	<.0001	0.05	-58.2715	-58.0166

The GLIMMIX Procedure

Differences of Colony_Typ*Condition Least Squares Means											
Colony_Type	Condition	_Colony_Type	_Condition	Estimate	Standard Error	DF	t Value	Pr > |t|	Alpha	Lower	Upper
CFU-GEMM	E13.5 EC Only	CFU-GEMM	H-EC Only	1.63E-13	.	.	.	.	.	.	.
CFU-GEMM	E13.5 EC Only	CFU-GEMM	H-EC+10.5MesP	-56.0315	1.6913	100	-33.13	<.0001	0.05	-59.3870	-52.6760
CFU-GEMM	E13.5 EC Only	CFU-GEMM	H-EC+11.5MesP	-55.9229	1.6915	100	-33.06	<.0001	0.05	-59.2787	-52.5670
CFU-GEMM	E13.5 EC Only	CFU-GEMM	I-EC Only	-178E-16	.	.	.	.	.	.	.
CFU-GEMM	E13.5 EC Only	CFU-GEMM	I-EC+10.5MesP	-54.7424	1.6939	100	-32.32	<.0001	0.05	-58.1030	-51.3818
CFU-GEMM	E13.5 EC Only	CFU-GEMM	I-EC+11.5MesP	-54.0102	1.6988	100	-31.79	<.0001	0.05	-57.3806	-50.6397
CFU-GEMM	E13.5 EC Only	CFU-GEMM	L-E only	-231E-16	.	.	.	.	.	.	.
CFU-GEMM	E13.5 EC Only	CFU-GEMM	L-E+11.5Mesp1	-56.1987	0	100	-Infty	<.0001	.	.	.
CFU-GEMM	E13.5 EC Only	CFU-GM	10.5EC+!0.5Me	-54.4642	1.5116	100	-36.03	<.0001	0.05	-57.4631	-51.4652
CFU-GEMM	E13.5 EC Only	CFU-GM	11.5 EC Only	-54.9102	1.6937	100	-32.42	<.0001	0.05	-58.2704	-51.5501
CFU-GEMM	E13.5 EC Only	CFU-GM	11.5EC+11.5Me	-54.5141	1.5113	100	-36.07	<.0001	0.05	-57.5125	-51.5157
CFU-GEMM	E13.5 EC Only	CFU-GM	13.5EC+10.5Me	-57.7421	1.6900	100	-34.17	<.0001	0.05	-61.0950	-54.3893
CFU-GEMM	E13.5 EC Only	CFU-GM	13.5EC+11.5Me	-58.0456	1.6899	100	-34.35	<.0001	0.05	-61.3983	-54.6929
CFU-GEMM	E13.5 EC Only	CFU-GM	Ao-EC Only	-11.3637	0.06423	100	-176.91	<.0001	0.05	-11.4911	-11.2363
CFU-GEMM	E13.5 EC Only	CFU-GM	AoEC+10.5MesP	-58.0996	1.6899	100	-34.38	<.0001	0.05	-61.4522	-54.7469
CFU-GEMM	E13.5 EC Only	CFU-GM	AoEC+11.5MesP	-58.0668	1.6899	100	-34.36	<.0001	0.05	-61.4195	-54.7141
CFU-GEMM	E13.5 EC Only	CFU-GM	E10.5-EC Only	-11.3637	0.06423	100	-176.91	<.0001	0.05	-11.4911	-11.2363
CFU-GEMM	E13.5 EC Only	CFU-GM	E10.5-MesP1+	-11.3637	0.06423	100	-176.91	<.0001	0.05	-11.4911	-11.2363
CFU-GEMM	E13.5 EC Only	CFU-GM	E10.5-MesP1-	-11.3637	0.06423	100	-176.91	<.0001	0.05	-11.4911	-11.2363
CFU-GEMM	E13.5 EC Only	CFU-GM	E11.5MesP1+On	-11.3637	0.06423	100	-176.91	<.0001	0.05	-11.4911	-11.2363
CFU-GEMM	E13.5 EC Only	CFU-GM	E11.5MesP1-On	-11.3637	0.06423	100	-176.91	<.0001	0.05	-11.4911	-11.2363
CFU-GEMM	E13.5 EC Only	CFU-GM	E13.5 EC Only	-11.3637	0.06423	100	-176.91	<.0001	0.05	-11.4911	-11.2363
CFU-GEMM	E13.5 EC Only	CFU-GM	H-EC Only	-11.3637	0.06423	100	-176.91	<.0001	0.05	-11.4911	-11.2363
CFU-GEMM	E13.5 EC Only	CFU-GM	H-EC+10.5MesP	-58.0812	1.6899	100	-34.37	<.0001	0.05	-61.4339	-54.7285

The GLIMMIX Procedure

Differences of Colony_Typ*Condition Least Squares Means											
Colony_Type	Condition	_Colony_Type	_Condition	Estimate	Standard Error	DF	t Value	Pr > |t|	Alpha	Lower	Upper
CFU-GEMM	E13.5 EC Only	CFU-GM	H-EC+11.5MesP	-57.9849	1.6899	100	-34.31	<.0001	0.05	-61.3376	-54.6322
CFU-GEMM	E13.5 EC Only	CFU-GM	I-EC Only	-11.3637	0.06423	100	-176.91	<.0001	0.05	-11.4911	-11.2363
CFU-GEMM	E13.5 EC Only	CFU-GM	I-EC+10.5MesP	-55.5663	1.6918	100	-32.85	<.0001	0.05	-58.9227	-52.2099
CFU-GEMM	E13.5 EC Only	CFU-GM	I-EC+11.5MesP	-54.8753	1.6938	100	-32.40	<.0001	0.05	-58.2357	-51.5148
CFU-GEMM	E13.5 EC Only	CFU-GM	L-E only	-11.3637	0.06423	100	-176.91	<.0001	0.05	-11.4911	-11.2363
CFU-GEMM	E13.5 EC Only	CFU-GM	L-E+11.5Mesp1	-58.1441	0.06423	100	-905.18	<.0001	0.05	-58.2715	-58.0166
CFU-GEMM	H-EC Only	CFU-GEMM	H-EC+10.5MesP	-56.0315	1.6913	100	-33.13	<.0001	0.05	-59.3870	-52.6760
CFU-GEMM	H-EC Only	CFU-GEMM	H-EC+11.5MesP	-55.9229	1.6915	100	-33.06	<.0001	0.05	-59.2787	-52.5670
CFU-GEMM	H-EC Only	CFU-GEMM	I-EC Only	-181E-15	.	.	.	.	.	.	.
CFU-GEMM	H-EC Only	CFU-GEMM	I-EC+10.5MesP	-54.7424	1.6939	100	-32.32	<.0001	0.05	-58.1030	-51.3818
CFU-GEMM	H-EC Only	CFU-GEMM	I-EC+11.5MesP	-54.0102	1.6988	100	-31.79	<.0001	0.05	-57.3806	-50.6397
CFU-GEMM	H-EC Only	CFU-GEMM	L-E only	-187E-15	.	.	.	.	.	.	.
CFU-GEMM	H-EC Only	CFU-GEMM	L-E+11.5Mesp1	-56.1987	0	100	-Infty	<.0001	.	.	.
CFU-GEMM	H-EC Only	CFU-GM	10.5EC+!0.5Me	-54.4642	1.5116	100	-36.03	<.0001	0.05	-57.4631	-51.4652
CFU-GEMM	H-EC Only	CFU-GM	11.5 EC Only	-54.9102	1.6937	100	-32.42	<.0001	0.05	-58.2704	-51.5501
CFU-GEMM	H-EC Only	CFU-GM	11.5EC+11.5Me	-54.5141	1.5113	100	-36.07	<.0001	0.05	-57.5125	-51.5157
CFU-GEMM	H-EC Only	CFU-GM	13.5EC+10.5Me	-57.7421	1.6900	100	-34.17	<.0001	0.05	-61.0950	-54.3893
CFU-GEMM	H-EC Only	CFU-GM	13.5EC+11.5Me	-58.0456	1.6899	100	-34.35	<.0001	0.05	-61.3983	-54.6929
CFU-GEMM	H-EC Only	CFU-GM	Ao-EC Only	-11.3637	0.06423	100	-176.91	<.0001	0.05	-11.4911	-11.2363
CFU-GEMM	H-EC Only	CFU-GM	AoEC+10.5MesP	-58.0996	1.6899	100	-34.38	<.0001	0.05	-61.4522	-54.7469
CFU-GEMM	H-EC Only	CFU-GM	AoEC+11.5MesP	-58.0668	1.6899	100	-34.36	<.0001	0.05	-61.4195	-54.7141
CFU-GEMM	H-EC Only	CFU-GM	E10.5-EC Only	-11.3637	0.06423	100	-176.91	<.0001	0.05	-11.4911	-11.2363
CFU-GEMM	H-EC Only	CFU-GM	E10.5-MesP1+	-11.3637	0.06423	100	-176.91	<.0001	0.05	-11.4911	-11.2363
CFU-GEMM	H-EC Only	CFU-GM	E10.5-MesP1-	-11.3637	0.06423	100	-176.91	<.0001	0.05	-11.4911	-11.2363

The GLIMMIX Procedure

Differences of Colony_Typ*Condition Least Squares Means											
Colony_Type	Condition	_Colony_Type	_Condition	Estimate	Standard Error	DF	t Value	Pr > |t|	Alpha	Lower	Upper
CFU-GEMM	H-EC Only	CFU-GM	E11.5MesP1+On	-11.3637	0.06423	100	-176.91	<.0001	0.05	-11.4911	-11.2363
CFU-GEMM	H-EC Only	CFU-GM	E11.5MesP1-On	-11.3637	0.06423	100	-176.91	<.0001	0.05	-11.4911	-11.2363
CFU-GEMM	H-EC Only	CFU-GM	E13.5 EC Only	-11.3637	0.06423	100	-176.91	<.0001	0.05	-11.4911	-11.2363
CFU-GEMM	H-EC Only	CFU-GM	H-EC Only	-11.3637	0.06423	100	-176.91	<.0001	0.05	-11.4911	-11.2363
CFU-GEMM	H-EC Only	CFU-GM	H-EC+10.5MesP	-58.0812	1.6899	100	-34.37	<.0001	0.05	-61.4339	-54.7285
CFU-GEMM	H-EC Only	CFU-GM	H-EC+11.5MesP	-57.9849	1.6899	100	-34.31	<.0001	0.05	-61.3376	-54.6322
CFU-GEMM	H-EC Only	CFU-GM	I-EC Only	-11.3637	0.06423	100	-176.91	<.0001	0.05	-11.4911	-11.2363
CFU-GEMM	H-EC Only	CFU-GM	I-EC+10.5MesP	-55.5663	1.6918	100	-32.85	<.0001	0.05	-58.9227	-52.2099
CFU-GEMM	H-EC Only	CFU-GM	I-EC+11.5MesP	-54.8753	1.6938	100	-32.40	<.0001	0.05	-58.2357	-51.5148
CFU-GEMM	H-EC Only	CFU-GM	L-E only	-11.3637	0.06423	100	-176.91	<.0001	0.05	-11.4911	-11.2363
CFU-GEMM	H-EC Only	CFU-GM	L-E+11.5Mesp1	-58.1441	0.06423	100	-905.18	<.0001	0.05	-58.2715	-58.0166
CFU-GEMM	H-EC+10.5MesP	CFU-GEMM	H-EC+11.5MesP	0.1086	1.6917	100	0.06	0.9489	0.05	-3.2477	3.4649
CFU-GEMM	H-EC+10.5MesP	CFU-GEMM	I-EC Only	56.0315	1.6913	100	33.13	<.0001	0.05	52.6760	59.3870
CFU-GEMM	H-EC+10.5MesP	CFU-GEMM	I-EC+10.5MesP	1.2891	1.6941	100	0.76	0.4485	0.05	-2.0720	4.6501
CFU-GEMM	H-EC+10.5MesP	CFU-GEMM	I-EC+11.5MesP	2.0213	1.6990	100	1.19	0.2370	0.05	-1.3496	5.3921
CFU-GEMM	H-EC+10.5MesP	CFU-GEMM	L-E only	56.0315	1.6913	100	33.13	<.0001	0.05	52.6760	59.3870
CFU-GEMM	H-EC+10.5MesP	CFU-GEMM	L-E+11.5Mesp1	-0.1672	1.6913	100	-0.10	0.9215	0.05	-3.5227	3.1883
CFU-GEMM	H-EC+10.5MesP	CFU-GM	10.5EC+!0.5Me	1.5673	1.5118	100	1.04	0.3024	0.05	-1.4320	4.5666
CFU-GEMM	H-EC+10.5MesP	CFU-GM	11.5 EC Only	1.1212	1.6939	100	0.66	0.5095	0.05	-2.2394	4.4818
CFU-GEMM	H-EC+10.5MesP	CFU-GM	11.5EC+11.5Me	1.5174	1.5115	100	1.00	0.3179	0.05	-1.4815	4.5162
CFU-GEMM	H-EC+10.5MesP	CFU-GM	13.5EC+10.5Me	-1.7107	1.6902	100	-1.01	0.3139	0.05	-5.0639	1.6426
CFU-GEMM	H-EC+10.5MesP	CFU-GM	13.5EC+11.5Me	-2.0141	1.6901	100	-1.19	0.2362	0.05	-5.3672	1.3390
CFU-GEMM	H-EC+10.5MesP	CFU-GM	Ao-EC Only	44.6678	1.6901	100	26.43	<.0001	0.05	41.3147	48.0209
CFU-GEMM	H-EC+10.5MesP	CFU-GM	AoEC+10.5MesP	-2.0681	1.6901	100	-1.22	0.2240	0.05	-5.4212	1.2850

The GLIMMIX Procedure

Differences of Colony_Typ*Condition Least Squares Means											
Colony_Type	Condition	_Colony_Type	_Condition	Estimate	Standard Error	DF	t Value	Pr > |t|	Alpha	Lower	Upper
CFU-GEMM	H-EC+10.5MesP	CFU-GM	AoEC+11.5MesP	-2.0353	1.6901	100	-1.20	0.2313	0.05	-5.3885	1.3178
CFU-GEMM	H-EC+10.5MesP	CFU-GM	E10.5-EC Only	44.6678	1.6901	100	26.43	<.0001	0.05	41.3147	48.0209
CFU-GEMM	H-EC+10.5MesP	CFU-GM	E10.5-MesP1+	44.6678	1.6901	100	26.43	<.0001	0.05	41.3147	48.0209
CFU-GEMM	H-EC+10.5MesP	CFU-GM	E10.5-MesP1-	44.6678	1.6901	100	26.43	<.0001	0.05	41.3147	48.0209
CFU-GEMM	H-EC+10.5MesP	CFU-GM	E11.5MesP1+On	44.6678	1.6901	100	26.43	<.0001	0.05	41.3147	48.0209
CFU-GEMM	H-EC+10.5MesP	CFU-GM	E11.5MesP1-On	44.6678	1.6901	100	26.43	<.0001	0.05	41.3147	48.0209
CFU-GEMM	H-EC+10.5MesP	CFU-GM	E13.5 EC Only	44.6678	1.6901	100	26.43	<.0001	0.05	41.3147	48.0209
CFU-GEMM	H-EC+10.5MesP	CFU-GM	H-EC Only	44.6678	1.6901	100	26.43	<.0001	0.05	41.3147	48.0209
CFU-GEMM	H-EC+10.5MesP	CFU-GM	H-EC+10.5MesP	-2.0498	0.06931	100	-29.58	<.0001	0.05	-2.1873	-1.9123
CFU-GEMM	H-EC+10.5MesP	CFU-GM	H-EC+11.5MesP	-1.9534	1.6901	100	-1.16	0.2505	0.05	-5.3066	1.3997
CFU-GEMM	H-EC+10.5MesP	CFU-GM	I-EC Only	44.6678	1.6901	100	26.43	<.0001	0.05	41.3147	48.0209
CFU-GEMM	H-EC+10.5MesP	CFU-GM	I-EC+10.5MesP	0.4651	1.6920	100	0.27	0.7840	0.05	-2.8917	3.8220
CFU-GEMM	H-EC+10.5MesP	CFU-GM	I-EC+11.5MesP	1.1562	1.6940	100	0.68	0.4965	0.05	-2.2047	4.5170
CFU-GEMM	H-EC+10.5MesP	CFU-GM	L-E only	44.6678	1.6901	100	26.43	<.0001	0.05	41.3147	48.0209
CFU-GEMM	H-EC+10.5MesP	CFU-GM	L-E+11.5Mesp1	-2.1126	1.6901	100	-1.25	0.2142	0.05	-5.4657	1.2405
CFU-GEMM	H-EC+11.5MesP	CFU-GEMM	I-EC Only	55.9229	1.6915	100	33.06	<.0001	0.05	52.5670	59.2787
CFU-GEMM	H-EC+11.5MesP	CFU-GEMM	I-EC+10.5MesP	1.1805	1.6943	100	0.70	0.4876	0.05	-2.1810	4.5419
CFU-GEMM	H-EC+11.5MesP	CFU-GEMM	I-EC+11.5MesP	1.9127	1.6992	100	1.13	0.2630	0.05	-1.4585	5.2839
CFU-GEMM	H-EC+11.5MesP	CFU-GEMM	L-E only	55.9229	1.6915	100	33.06	<.0001	0.05	52.5670	59.2787
CFU-GEMM	H-EC+11.5MesP	CFU-GEMM	L-E+11.5Mesp1	-0.2758	1.6915	100	-0.16	0.8708	0.05	-3.6317	3.0801
CFU-GEMM	H-EC+11.5MesP	CFU-GM	10.5EC+!0.5Me	1.4587	1.5120	100	0.96	0.3370	0.05	-1.5411	4.4585
CFU-GEMM	H-EC+11.5MesP	CFU-GM	11.5 EC Only	1.0126	1.6940	100	0.60	0.5514	0.05	-2.3483	4.3735
CFU-GEMM	H-EC+11.5MesP	CFU-GM	11.5EC+11.5Me	1.4088	1.5118	100	0.93	0.3537	0.05	-1.5906	4.4081
CFU-GEMM	H-EC+11.5MesP	CFU-GM	13.5EC+10.5Me	-1.8193	1.6903	100	-1.08	0.2844	0.05	-5.1729	1.5343

The GLIMMIX Procedure

Differences of Colony_Typ*Condition Least Squares Means											
Colony_Type	Condition	_Colony_Type	_Condition	Estimate	Standard Error	DF	t Value	Pr > |t|	Alpha	Lower	Upper
CFU-GEMM	H-EC+11.5MesP	CFU-GM	13.5EC+11.5Me	-2.1227	1.6903	100	-1.26	0.2121	0.05	-5.4762	1.2308
CFU-GEMM	H-EC+11.5MesP	CFU-GM	Ao-EC Only	44.5592	1.6903	100	26.36	<.0001	0.05	41.2057	47.9126
CFU-GEMM	H-EC+11.5MesP	CFU-GM	AoEC+10.5MesP	-2.1767	1.6903	100	-1.29	0.2008	0.05	-5.5302	1.1768
CFU-GEMM	H-EC+11.5MesP	CFU-GM	AoEC+11.5MesP	-2.1439	1.6903	100	-1.27	0.2076	0.05	-5.4974	1.2095
CFU-GEMM	H-EC+11.5MesP	CFU-GM	E10.5-EC Only	44.5592	1.6903	100	26.36	<.0001	0.05	41.2057	47.9126
CFU-GEMM	H-EC+11.5MesP	CFU-GM	E10.5-MesP1+	44.5592	1.6903	100	26.36	<.0001	0.05	41.2057	47.9126
CFU-GEMM	H-EC+11.5MesP	CFU-GM	E10.5-MesP1-	44.5592	1.6903	100	26.36	<.0001	0.05	41.2057	47.9126
CFU-GEMM	H-EC+11.5MesP	CFU-GM	E11.5MesP1+On	44.5592	1.6903	100	26.36	<.0001	0.05	41.2057	47.9126
CFU-GEMM	H-EC+11.5MesP	CFU-GM	E11.5MesP1-On	44.5592	1.6903	100	26.36	<.0001	0.05	41.2057	47.9126
CFU-GEMM	H-EC+11.5MesP	CFU-GM	E13.5 EC Only	44.5592	1.6903	100	26.36	<.0001	0.05	41.2057	47.9126
CFU-GEMM	H-EC+11.5MesP	CFU-GM	H-EC Only	44.5592	1.6903	100	26.36	<.0001	0.05	41.2057	47.9126
CFU-GEMM	H-EC+11.5MesP	CFU-GM	H-EC+10.5MesP	-2.1584	1.6903	100	-1.28	0.2046	0.05	-5.5118	1.1951
CFU-GEMM	H-EC+11.5MesP	CFU-GM	H-EC+11.5MesP	-2.0620	0.07326	100	-28.15	<.0001	0.05	-2.2074	-1.9167
CFU-GEMM	H-EC+11.5MesP	CFU-GM	I-EC Only	44.5592	1.6903	100	26.36	<.0001	0.05	41.2057	47.9126
CFU-GEMM	H-EC+11.5MesP	CFU-GM	I-EC+10.5MesP	0.3565	1.6922	100	0.21	0.8336	0.05	-3.0006	3.7137
CFU-GEMM	H-EC+11.5MesP	CFU-GM	I-EC+11.5MesP	1.0476	1.6942	100	0.62	0.5378	0.05	-2.3137	4.4088
CFU-GEMM	H-EC+11.5MesP	CFU-GM	L-E only	44.5592	1.6903	100	26.36	<.0001	0.05	41.2057	47.9126
CFU-GEMM	H-EC+11.5MesP	CFU-GM	L-E+11.5Mesp1	-2.2212	1.6903	100	-1.31	0.1918	0.05	-5.5746	1.1323
CFU-GEMM	I-EC Only	CFU-GEMM	I-EC+10.5MesP	-54.7424	1.6939	100	-32.32	<.0001	0.05	-58.1030	-51.3818
CFU-GEMM	I-EC Only	CFU-GEMM	I-EC+11.5MesP	-54.0102	1.6988	100	-31.79	<.0001	0.05	-57.3806	-50.6397
CFU-GEMM	I-EC Only	CFU-GEMM	L-E only	-533E-17	.	.	.	.	.	.	.
CFU-GEMM	I-EC Only	CFU-GEMM	L-E+11.5Mesp1	-56.1987	0	100	-Infty	<.0001	.	.	.
CFU-GEMM	I-EC Only	CFU-GM	10.5EC+!0.5Me	-54.4642	1.5116	100	-36.03	<.0001	0.05	-57.4631	-51.4652
CFU-GEMM	I-EC Only	CFU-GM	11.5 EC Only	-54.9102	1.6937	100	-32.42	<.0001	0.05	-58.2704	-51.5501

The GLIMMIX Procedure

Differences of Colony_Typ*Condition Least Squares Means											
Colony_Type	Condition	_Colony_Type	_Condition	Estimate	Standard Error	DF	t Value	Pr > |t|	Alpha	Lower	Upper
CFU-GEMM	I-EC Only	CFU-GM	11.5EC+11.5Me	-54.5141	1.5113	100	-36.07	<.0001	0.05	-57.5125	-51.5157
CFU-GEMM	I-EC Only	CFU-GM	13.5EC+10.5Me	-57.7421	1.6900	100	-34.17	<.0001	0.05	-61.0950	-54.3893
CFU-GEMM	I-EC Only	CFU-GM	13.5EC+11.5Me	-58.0456	1.6899	100	-34.35	<.0001	0.05	-61.3983	-54.6929
CFU-GEMM	I-EC Only	CFU-GM	Ao-EC Only	-11.3637	0.06423	100	-176.91	<.0001	0.05	-11.4911	-11.2363
CFU-GEMM	I-EC Only	CFU-GM	AoEC+10.5MesP	-58.0996	1.6899	100	-34.38	<.0001	0.05	-61.4522	-54.7469
CFU-GEMM	I-EC Only	CFU-GM	AoEC+11.5MesP	-58.0668	1.6899	100	-34.36	<.0001	0.05	-61.4195	-54.7141
CFU-GEMM	I-EC Only	CFU-GM	E10.5-EC Only	-11.3637	0.06423	100	-176.91	<.0001	0.05	-11.4911	-11.2363
CFU-GEMM	I-EC Only	CFU-GM	E10.5-MesP1+	-11.3637	0.06423	100	-176.91	<.0001	0.05	-11.4911	-11.2363
CFU-GEMM	I-EC Only	CFU-GM	E10.5-MesP1-	-11.3637	0.06423	100	-176.91	<.0001	0.05	-11.4911	-11.2363
CFU-GEMM	I-EC Only	CFU-GM	E11.5MesP1+On	-11.3637	0.06423	100	-176.91	<.0001	0.05	-11.4911	-11.2363
CFU-GEMM	I-EC Only	CFU-GM	E11.5MesP1-On	-11.3637	0.06423	100	-176.91	<.0001	0.05	-11.4911	-11.2363
CFU-GEMM	I-EC Only	CFU-GM	E13.5 EC Only	-11.3637	0.06423	100	-176.91	<.0001	0.05	-11.4911	-11.2363
CFU-GEMM	I-EC Only	CFU-GM	H-EC Only	-11.3637	0.06423	100	-176.91	<.0001	0.05	-11.4911	-11.2363
CFU-GEMM	I-EC Only	CFU-GM	H-EC+10.5MesP	-58.0812	1.6899	100	-34.37	<.0001	0.05	-61.4339	-54.7285
CFU-GEMM	I-EC Only	CFU-GM	H-EC+11.5MesP	-57.9849	1.6899	100	-34.31	<.0001	0.05	-61.3376	-54.6322
CFU-GEMM	I-EC Only	CFU-GM	I-EC Only	-11.3637	0.06423	100	-176.91	<.0001	0.05	-11.4911	-11.2363
CFU-GEMM	I-EC Only	CFU-GM	I-EC+10.5MesP	-55.5663	1.6918	100	-32.85	<.0001	0.05	-58.9227	-52.2099
CFU-GEMM	I-EC Only	CFU-GM	I-EC+11.5MesP	-54.8753	1.6938	100	-32.40	<.0001	0.05	-58.2357	-51.5148
CFU-GEMM	I-EC Only	CFU-GM	L-E only	-11.3637	0.06423	100	-176.91	<.0001	0.05	-11.4911	-11.2363
CFU-GEMM	I-EC Only	CFU-GM	L-E+11.5Mesp1	-58.1441	0.06423	100	-905.18	<.0001	0.05	-58.2715	-58.0166
CFU-GEMM	I-EC+10.5MesP	CFU-GEMM	I-EC+11.5MesP	0.7322	1.7016	100	0.43	0.6679	0.05	-2.6437	4.1082
CFU-GEMM	I-EC+10.5MesP	CFU-GEMM	L-E only	54.7424	1.6939	100	32.32	<.0001	0.05	51.3818	58.1030
CFU-GEMM	I-EC+10.5MesP	CFU-GEMM	L-E+11.5Mesp1	-1.4563	1.6939	100	-0.86	0.3920	0.05	-4.8169	1.9044
CFU-GEMM	I-EC+10.5MesP	CFU-GM	10.5EC+!0.5Me	0.2782	1.5145	100	0.18	0.8546	0.05	-2.7265	3.2830

The GLIMMIX Procedure

Differences of Colony_Typ*Condition Least Squares Means											
Colony_Type	Condition	_Colony_Type	_Condition	Estimate	Standard Error	DF	t Value	Pr > |t|	Alpha	Lower	Upper
CFU-GEMM	I-EC+10.5MesP	CFU-GM	11.5 EC Only	-0.1678	1.6964	100	-0.10	0.9214	0.05	-3.5335	3.1978
CFU-GEMM	I-EC+10.5MesP	CFU-GM	11.5EC+11.5Me	0.2283	1.5143	100	0.15	0.8805	0.05	-2.7759	3.2325
CFU-GEMM	I-EC+10.5MesP	CFU-GM	13.5EC+10.5Me	-2.9997	1.6927	100	-1.77	0.0794	0.05	-6.3581	0.3586
CFU-GEMM	I-EC+10.5MesP	CFU-GM	13.5EC+11.5Me	-3.3032	1.6927	100	-1.95	0.0538	0.05	-6.6614	0.05506
CFU-GEMM	I-EC+10.5MesP	CFU-GM	Ao-EC Only	43.3787	1.6927	100	25.63	<.0001	0.05	40.0205	46.7369
CFU-GEMM	I-EC+10.5MesP	CFU-GM	AoEC+10.5MesP	-3.3572	1.6927	100	-1.98	0.0501	0.05	-6.7154	0.001073
CFU-GEMM	I-EC+10.5MesP	CFU-GM	AoEC+11.5MesP	-3.3244	1.6927	100	-1.96	0.0523	0.05	-6.6826	0.03384
CFU-GEMM	I-EC+10.5MesP	CFU-GM	E10.5-EC Only	43.3787	1.6927	100	25.63	<.0001	0.05	40.0205	46.7369
CFU-GEMM	I-EC+10.5MesP	CFU-GM	E10.5-MesP1+	43.3787	1.6927	100	25.63	<.0001	0.05	40.0205	46.7369
CFU-GEMM	I-EC+10.5MesP	CFU-GM	E10.5-MesP1-	43.3787	1.6927	100	25.63	<.0001	0.05	40.0205	46.7369
CFU-GEMM	I-EC+10.5MesP	CFU-GM	E11.5MesP1+On	43.3787	1.6927	100	25.63	<.0001	0.05	40.0205	46.7369
CFU-GEMM	I-EC+10.5MesP	CFU-GM	E11.5MesP1-On	43.3787	1.6927	100	25.63	<.0001	0.05	40.0205	46.7369
CFU-GEMM	I-EC+10.5MesP	CFU-GM	E13.5 EC Only	43.3787	1.6927	100	25.63	<.0001	0.05	40.0205	46.7369
CFU-GEMM	I-EC+10.5MesP	CFU-GM	H-EC Only	43.3787	1.6927	100	25.63	<.0001	0.05	40.0205	46.7369
CFU-GEMM	I-EC+10.5MesP	CFU-GM	H-EC+10.5MesP	-3.3388	1.6927	100	-1.97	0.0513	0.05	-6.6971	0.01942
CFU-GEMM	I-EC+10.5MesP	CFU-GM	H-EC+11.5MesP	-3.2425	1.6927	100	-1.92	0.0583	0.05	-6.6008	0.1158
CFU-GEMM	I-EC+10.5MesP	CFU-GM	I-EC Only	43.3787	1.6927	100	25.63	<.0001	0.05	40.0205	46.7369
CFU-GEMM	I-EC+10.5MesP	CFU-GM	I-EC+10.5MesP	-0.8239	0.1455	100	-5.66	<.0001	0.05	-1.1125	-0.5353
CFU-GEMM	I-EC+10.5MesP	CFU-GM	I-EC+11.5MesP	-0.1329	1.6966	100	-0.08	0.9377	0.05	-3.4989	3.2331
CFU-GEMM	I-EC+10.5MesP	CFU-GM	L-E only	43.3787	1.6927	100	25.63	<.0001	0.05	40.0205	46.7369
CFU-GEMM	I-EC+10.5MesP	CFU-GM	L-E+11.5Mesp1	-3.4017	1.6927	100	-2.01	0.0472	0.05	-6.7599	-0.04344
CFU-GEMM	I-EC+11.5MesP	CFU-GEMM	L-E only	54.0102	1.6988	100	31.79	<.0001	0.05	50.6397	57.3806
CFU-GEMM	I-EC+11.5MesP	CFU-GEMM	L-E+11.5Mesp1	-2.1885	1.6988	100	-1.29	0.2006	0.05	-5.5589	1.1819
CFU-GEMM	I-EC+11.5MesP	CFU-GM	10.5EC+!0.5Me	-0.4540	1.5204	100	-0.30	0.7659	0.05	-3.4705	2.5625

The GLIMMIX Procedure

Differences of Colony_Typ*Condition Least Squares Means											
Colony_Type	Condition	_Colony_Type	_Condition	Estimate	Standard Error	DF	t Value	Pr > |t|	Alpha	Lower	Upper
CFU-GEMM	I-EC+11.5MesP	CFU-GM	11.5 EC Only	-0.9001	1.7014	100	-0.53	0.5980	0.05	-4.2755	2.4754
CFU-GEMM	I-EC+11.5MesP	CFU-GM	11.5EC+11.5Me	-0.5039	1.5202	100	-0.33	0.7410	0.05	-3.5199	2.5120
CFU-GEMM	I-EC+11.5MesP	CFU-GM	13.5EC+10.5Me	-3.7320	1.6977	100	-2.20	0.0302	0.05	-7.1001	-0.3638
CFU-GEMM	I-EC+11.5MesP	CFU-GM	13.5EC+11.5Me	-4.0354	1.6976	100	-2.38	0.0194	0.05	-7.4035	-0.6674
CFU-GEMM	I-EC+11.5MesP	CFU-GM	Ao-EC Only	42.6465	1.6976	100	25.12	<.0001	0.05	39.2785	46.0145
CFU-GEMM	I-EC+11.5MesP	CFU-GM	AoEC+10.5MesP	-4.0894	1.6976	100	-2.41	0.0178	0.05	-7.4574	-0.7214
CFU-GEMM	I-EC+11.5MesP	CFU-GM	AoEC+11.5MesP	-4.0566	1.6976	100	-2.39	0.0187	0.05	-7.4247	-0.6886
CFU-GEMM	I-EC+11.5MesP	CFU-GM	E10.5-EC Only	42.6465	1.6976	100	25.12	<.0001	0.05	39.2785	46.0145
CFU-GEMM	I-EC+11.5MesP	CFU-GM	E10.5-MesP1+	42.6465	1.6976	100	25.12	<.0001	0.05	39.2785	46.0145
CFU-GEMM	I-EC+11.5MesP	CFU-GM	E10.5-MesP1-	42.6465	1.6976	100	25.12	<.0001	0.05	39.2785	46.0145
CFU-GEMM	I-EC+11.5MesP	CFU-GM	E11.5MesP1+On	42.6465	1.6976	100	25.12	<.0001	0.05	39.2785	46.0145
CFU-GEMM	I-EC+11.5MesP	CFU-GM	E11.5MesP1-On	42.6465	1.6976	100	25.12	<.0001	0.05	39.2785	46.0145
CFU-GEMM	I-EC+11.5MesP	CFU-GM	E13.5 EC Only	42.6465	1.6976	100	25.12	<.0001	0.05	39.2785	46.0145
CFU-GEMM	I-EC+11.5MesP	CFU-GM	H-EC Only	42.6465	1.6976	100	25.12	<.0001	0.05	39.2785	46.0145
CFU-GEMM	I-EC+11.5MesP	CFU-GM	H-EC+10.5MesP	-4.0710	1.6976	100	-2.40	0.0183	0.05	-7.4391	-0.7030
CFU-GEMM	I-EC+11.5MesP	CFU-GM	H-EC+11.5MesP	-3.9747	1.6976	100	-2.34	0.0212	0.05	-7.3428	-0.6066
CFU-GEMM	I-EC+11.5MesP	CFU-GM	I-EC Only	42.6465	1.6976	100	25.12	<.0001	0.05	39.2785	46.0145
CFU-GEMM	I-EC+11.5MesP	CFU-GM	I-EC+10.5MesP	-1.5562	1.6995	100	-0.92	0.3620	0.05	-4.9279	1.8156
CFU-GEMM	I-EC+11.5MesP	CFU-GM	I-EC+11.5MesP	-0.8651	0.2107	100	-4.11	<.0001	0.05	-1.2832	-0.4470
CFU-GEMM	I-EC+11.5MesP	CFU-GM	L-E only	42.6465	1.6976	100	25.12	<.0001	0.05	39.2785	46.0145
CFU-GEMM	I-EC+11.5MesP	CFU-GM	L-E+11.5Mesp1	-4.1339	1.6976	100	-2.44	0.0167	0.05	-7.5019	-0.7659
CFU-GEMM	L-E only	CFU-GEMM	L-E+11.5Mesp1	-56.1987	0	100	-Infty	<.0001	.	.	.
CFU-GEMM	L-E only	CFU-GM	10.5EC+!0.5Me	-54.4642	1.5116	100	-36.03	<.0001	0.05	-57.4631	-51.4652
CFU-GEMM	L-E only	CFU-GM	11.5 EC Only	-54.9102	1.6937	100	-32.42	<.0001	0.05	-58.2704	-51.5501

The GLIMMIX Procedure

Differences of Colony_Typ*Condition Least Squares Means											
Colony_Type	Condition	_Colony_Type	_Condition	Estimate	Standard Error	DF	t Value	Pr > |t|	Alpha	Lower	Upper
CFU-GEMM	L-E only	CFU-GM	11.5EC+11.5Me	-54.5141	1.5113	100	-36.07	<.0001	0.05	-57.5125	-51.5157
CFU-GEMM	L-E only	CFU-GM	13.5EC+10.5Me	-57.7421	1.6900	100	-34.17	<.0001	0.05	-61.0950	-54.3893
CFU-GEMM	L-E only	CFU-GM	13.5EC+11.5Me	-58.0456	1.6899	100	-34.35	<.0001	0.05	-61.3983	-54.6929
CFU-GEMM	L-E only	CFU-GM	Ao-EC Only	-11.3637	0.06423	100	-176.91	<.0001	0.05	-11.4911	-11.2363
CFU-GEMM	L-E only	CFU-GM	AoEC+10.5MesP	-58.0996	1.6899	100	-34.38	<.0001	0.05	-61.4522	-54.7469
CFU-GEMM	L-E only	CFU-GM	AoEC+11.5MesP	-58.0668	1.6899	100	-34.36	<.0001	0.05	-61.4195	-54.7141
CFU-GEMM	L-E only	CFU-GM	E10.5-EC Only	-11.3637	0.06423	100	-176.91	<.0001	0.05	-11.4911	-11.2363
CFU-GEMM	L-E only	CFU-GM	E10.5-MesP1+	-11.3637	0.06423	100	-176.91	<.0001	0.05	-11.4911	-11.2363
CFU-GEMM	L-E only	CFU-GM	E10.5-MesP1-	-11.3637	0.06423	100	-176.91	<.0001	0.05	-11.4911	-11.2363
CFU-GEMM	L-E only	CFU-GM	E11.5MesP1+On	-11.3637	0.06423	100	-176.91	<.0001	0.05	-11.4911	-11.2363
CFU-GEMM	L-E only	CFU-GM	E11.5MesP1-On	-11.3637	0.06423	100	-176.91	<.0001	0.05	-11.4911	-11.2363
CFU-GEMM	L-E only	CFU-GM	E13.5 EC Only	-11.3637	0.06423	100	-176.91	<.0001	0.05	-11.4911	-11.2363
CFU-GEMM	L-E only	CFU-GM	H-EC Only	-11.3637	0.06423	100	-176.91	<.0001	0.05	-11.4911	-11.2363
CFU-GEMM	L-E only	CFU-GM	H-EC+10.5MesP	-58.0812	1.6899	100	-34.37	<.0001	0.05	-61.4339	-54.7285
CFU-GEMM	L-E only	CFU-GM	H-EC+11.5MesP	-57.9849	1.6899	100	-34.31	<.0001	0.05	-61.3376	-54.6322
CFU-GEMM	L-E only	CFU-GM	I-EC Only	-11.3637	0.06423	100	-176.91	<.0001	0.05	-11.4911	-11.2363
CFU-GEMM	L-E only	CFU-GM	I-EC+10.5MesP	-55.5663	1.6918	100	-32.85	<.0001	0.05	-58.9227	-52.2099
CFU-GEMM	L-E only	CFU-GM	I-EC+11.5MesP	-54.8753	1.6938	100	-32.40	<.0001	0.05	-58.2357	-51.5148
CFU-GEMM	L-E only	CFU-GM	L-E only	-11.3637	0.06423	100	-176.91	<.0001	0.05	-11.4911	-11.2363
CFU-GEMM	L-E only	CFU-GM	L-E+11.5Mesp1	-58.1441	0.06423	100	-905.18	<.0001	0.05	-58.2715	-58.0166
CFU-GEMM	L-E+11.5Mesp1	CFU-GM	10.5EC+!0.5Me	1.7345	1.5116	100	1.15	0.2539	0.05	-1.2645	4.7334
CFU-GEMM	L-E+11.5Mesp1	CFU-GM	11.5 EC Only	1.2884	1.6937	100	0.76	0.4486	0.05	-2.0717	4.6486
CFU-GEMM	L-E+11.5Mesp1	CFU-GM	11.5EC+11.5Me	1.6845	1.5113	100	1.11	0.2677	0.05	-1.3139	4.6830
CFU-GEMM	L-E+11.5Mesp1	CFU-GM	13.5EC+10.5Me	-1.5435	1.6900	100	-0.91	0.3633	0.05	-4.8963	1.8093

The GLIMMIX Procedure

Differences of Colony_Typ*Condition Least Squares Means											
**Colony_Type**	**Condition**	**_Colony_Type**	**_Condition**	**Estimate**	**Standard Error**	**DF**	**t Value**	**Pr > |t|**	**Alpha**	**Lower**	**Upper**
CFU-GEMM	L-E+11.5Mesp1	CFU-GM	13.5EC+11.5Me	-1.8469	1.6899	100	-1.09	0.2771	0.05	-5.1996	1.5058
CFU-GEMM	L-E+11.5Mesp1	CFU-GM	Ao-EC Only	44.8350	0.06423	100	697.99	<.0001	0.05	44.7075	44.9624
CFU-GEMM	L-E+11.5Mesp1	CFU-GM	AoEC+10.5MesP	-1.9009	1.6899	100	-1.12	0.2633	0.05	-5.2536	1.4518
CFU-GEMM	L-E+11.5Mesp1	CFU-GM	AoEC+11.5MesP	-1.8681	1.6899	100	-1.11	0.2716	0.05	-5.2208	1.4846
CFU-GEMM	L-E+11.5Mesp1	CFU-GM	E10.5-EC Only	44.8350	0.06423	100	697.99	<.0001	0.05	44.7075	44.9624
CFU-GEMM	L-E+11.5Mesp1	CFU-GM	E10.5-MesP1+	44.8350	0.06423	100	697.99	<.0001	0.05	44.7075	44.9624
CFU-GEMM	L-E+11.5Mesp1	CFU-GM	E10.5-MesP1-	44.8350	0.06423	100	697.99	<.0001	0.05	44.7075	44.9624
CFU-GEMM	L-E+11.5Mesp1	CFU-GM	E11.5MesP1+On	44.8350	0.06423	100	697.99	<.0001	0.05	44.7075	44.9624
CFU-GEMM	L-E+11.5Mesp1	CFU-GM	E11.5MesP1-On	44.8350	0.06423	100	697.99	<.0001	0.05	44.7075	44.9624
CFU-GEMM	L-E+11.5Mesp1	CFU-GM	E13.5 EC Only	44.8350	0.06423	100	697.99	<.0001	0.05	44.7075	44.9624
CFU-GEMM	L-E+11.5Mesp1	CFU-GM	H-EC Only	44.8350	0.06423	100	697.99	<.0001	0.05	44.7075	44.9624
CFU-GEMM	L-E+11.5Mesp1	CFU-GM	H-EC+10.5MesP	-1.8826	1.6899	100	-1.11	0.2679	0.05	-5.2352	1.4701
CFU-GEMM	L-E+11.5Mesp1	CFU-GM	H-EC+11.5MesP	-1.7862	1.6899	100	-1.06	0.2931	0.05	-5.1390	1.5665
CFU-GEMM	L-E+11.5Mesp1	CFU-GM	I-EC Only	44.8350	0.06423	100	697.99	<.0001	0.05	44.7075	44.9624
CFU-GEMM	L-E+11.5Mesp1	CFU-GM	I-EC+10.5MesP	0.6323	1.6918	100	0.37	0.7094	0.05	-2.7241	3.9887
CFU-GEMM	L-E+11.5Mesp1	CFU-GM	I-EC+11.5MesP	1.3234	1.6938	100	0.78	0.4365	0.05	-2.0371	4.6838
CFU-GEMM	L-E+11.5Mesp1	CFU-GM	L-E only	44.8350	0.06423	100	697.99	<.0001	0.05	44.7075	44.9624
CFU-GEMM	L-E+11.5Mesp1	CFU-GM	L-E+11.5Mesp1	-1.9454	0.06423	100	-30.29	<.0001	0.05	-2.0728	-1.8180
CFU-GM	10.5EC+!0.5Me	CFU-GM	11.5 EC Only	-0.4461	1.5146	100	-0.29	0.7690	0.05	-3.4509	2.5588
CFU-GM	10.5EC+!0.5Me	CFU-GM	11.5EC+11.5Me	-0.04994	1.2845	100	-0.04	0.9691	0.05	-2.5984	2.4985
CFU-GM	10.5EC+!0.5Me	CFU-GM	13.5EC+10.5Me	-3.2780	1.5102	100	-2.17	0.0323	0.05	-6.2741	-0.2818
CFU-GM	10.5EC+!0.5Me	CFU-GM	13.5EC+11.5Me	-3.5814	1.5102	100	-2.37	0.0196	0.05	-6.5776	-0.5852
CFU-GM	10.5EC+!0.5Me	CFU-GM	Ao-EC Only	43.1005	1.5102	100	28.54	<.0001	0.05	40.1042	46.0967
CFU-GM	10.5EC+!0.5Me	CFU-GM	AoEC+10.5MesP	-3.6354	1.5102	100	-2.41	0.0179	0.05	-6.6315	-0.6392

The GLIMMIX Procedure

Differences of Colony_Typ*Condition Least Squares Means											
Colony_Type	Condition	_Colony_Type	_Condition	Estimate	Standard Error	DF	t Value	Pr > |t|	Alpha	Lower	Upper
CFU-GM	10.5EC+!0.5Me	CFU-GM	AoEC+11.5MesP	-3.6026	1.5102	100	-2.39	0.0189	0.05	-6.5989	-0.6064
CFU-GM	10.5EC+!0.5Me	CFU-GM	E10.5-EC Only	43.1005	1.5102	100	28.54	<.0001	0.05	40.1042	46.0967
CFU-GM	10.5EC+!0.5Me	CFU-GM	E10.5-MesP1+	43.1005	1.5102	100	28.54	<.0001	0.05	40.1042	46.0967
CFU-GM	10.5EC+!0.5Me	CFU-GM	E10.5-MesP1-	43.1005	1.5102	100	28.54	<.0001	0.05	40.1042	46.0967
CFU-GM	10.5EC+!0.5Me	CFU-GM	E11.5MesP1+On	43.1005	1.5102	100	28.54	<.0001	0.05	40.1042	46.0967
CFU-GM	10.5EC+!0.5Me	CFU-GM	E11.5MesP1-On	43.1005	1.5102	100	28.54	<.0001	0.05	40.1042	46.0967
CFU-GM	10.5EC+!0.5Me	CFU-GM	E13.5 EC Only	43.1005	1.5102	100	28.54	<.0001	0.05	40.1042	46.0967
CFU-GM	10.5EC+!0.5Me	CFU-GM	H-EC Only	43.1005	1.5102	100	28.54	<.0001	0.05	40.1042	46.0967
CFU-GM	10.5EC+!0.5Me	CFU-GM	H-EC+10.5MesP	-3.6170	1.5102	100	-2.40	0.0185	0.05	-6.6132	-0.6209
CFU-GM	10.5EC+!0.5Me	CFU-GM	H-EC+11.5MesP	-3.5207	1.5103	100	-2.33	0.0217	0.05	-6.5170	-0.5244
CFU-GM	10.5EC+!0.5Me	CFU-GM	I-EC Only	43.1005	1.5102	100	28.54	<.0001	0.05	40.1042	46.0967
CFU-GM	10.5EC+!0.5Me	CFU-GM	I-EC+10.5MesP	-1.1022	1.5121	100	-0.73	0.4678	0.05	-4.1022	1.8979
CFU-GM	10.5EC+!0.5Me	CFU-GM	I-EC+11.5MesP	-0.4111	1.5148	100	-0.27	0.7866	0.05	-3.4165	2.5942
CFU-GM	10.5EC+!0.5Me	CFU-GM	L-E only	43.1005	1.5102	100	28.54	<.0001	0.05	40.1042	46.0967
CFU-GM	10.5EC+!0.5Me	CFU-GM	L-E+11.5Mesp1	-3.6799	1.5102	100	-2.44	0.0166	0.05	-6.6761	-0.6837
CFU-GM	11.5 EC Only	CFU-GM	11.5EC+11.5Me	0.3961	1.5143	100	0.26	0.7942	0.05	-2.6082	3.4005
CFU-GM	11.5 EC Only	CFU-GM	13.5EC+10.5Me	-2.8319	1.6925	100	-1.67	0.0974	0.05	-6.1898	0.5260
CFU-GM	11.5 EC Only	CFU-GM	13.5EC+11.5Me	-3.1353	1.6924	100	-1.85	0.0669	0.05	-6.4931	0.2224
CFU-GM	11.5 EC Only	CFU-GM	Ao-EC Only	43.5466	1.6924	100	25.73	<.0001	0.05	40.1888	46.9043
CFU-GM	11.5 EC Only	CFU-GM	AoEC+10.5MesP	-3.1893	1.6924	100	-1.88	0.0624	0.05	-6.5471	0.1684
CFU-GM	11.5 EC Only	CFU-GM	AoEC+11.5MesP	-3.1566	1.6924	100	-1.87	0.0651	0.05	-6.5143	0.2012
CFU-GM	11.5 EC Only	CFU-GM	E10.5-EC Only	43.5466	1.6924	100	25.73	<.0001	0.05	40.1888	46.9043
CFU-GM	11.5 EC Only	CFU-GM	E10.5-MesP1+	43.5466	1.6924	100	25.73	<.0001	0.05	40.1888	46.9043
CFU-GM	11.5 EC Only	CFU-GM	E10.5-MesP1-	43.5466	1.6924	100	25.73	<.0001	0.05	40.1888	46.9043

The GLIMMIX Procedure

Differences of Colony_Typ*Condition Least Squares Means											
Colony_Type	Condition	_Colony_Type	_Condition	Estimate	Standard Error	DF	t Value	Pr > |t|	Alpha	Lower	Upper
CFU-GM	11.5 EC Only	CFU-GM	E11.5MesP1+On	43.5466	1.6924	100	25.73	<.0001	0.05	40.1888	46.9043
CFU-GM	11.5 EC Only	CFU-GM	E11.5MesP1-On	43.5466	1.6924	100	25.73	<.0001	0.05	40.1888	46.9043
CFU-GM	11.5 EC Only	CFU-GM	E13.5 EC Only	43.5466	1.6924	100	25.73	<.0001	0.05	40.1888	46.9043
CFU-GM	11.5 EC Only	CFU-GM	H-EC Only	43.5466	1.6924	100	25.73	<.0001	0.05	40.1888	46.9043
CFU-GM	11.5 EC Only	CFU-GM	H-EC+10.5MesP	-3.1710	1.6924	100	-1.87	0.0639	0.05	-6.5287	0.1868
CFU-GM	11.5 EC Only	CFU-GM	H-EC+11.5MesP	-3.0746	1.6925	100	-1.82	0.0723	0.05	-6.4324	0.2831
CFU-GM	11.5 EC Only	CFU-GM	I-EC Only	43.5466	1.6924	100	25.73	<.0001	0.05	40.1888	46.9043
CFU-GM	11.5 EC Only	CFU-GM	I-EC+10.5MesP	-0.6561	1.6943	100	-0.39	0.6994	0.05	-4.0175	2.7054
CFU-GM	11.5 EC Only	CFU-GM	I-EC+11.5MesP	0.03495	1.6963	100	0.02	0.9836	0.05	-3.3306	3.4004
CFU-GM	11.5 EC Only	CFU-GM	L-E only	43.5466	1.6924	100	25.73	<.0001	0.05	40.1888	46.9043
CFU-GM	11.5 EC Only	CFU-GM	L-E+11.5Mesp1	-3.2338	1.6924	100	-1.91	0.0589	0.05	-6.5915	0.1239
CFU-GM	11.5EC+11.5Me	CFU-GM	13.5EC+10.5Me	-3.2280	1.5099	100	-2.14	0.0350	0.05	-6.2236	-0.2324
CFU-GM	11.5EC+11.5Me	CFU-GM	13.5EC+11.5Me	-3.5315	1.5099	100	-2.34	0.0213	0.05	-6.5272	-0.5358
CFU-GM	11.5EC+11.5Me	CFU-GM	Ao-EC Only	43.1504	1.5100	100	28.58	<.0001	0.05	40.1547	46.1461
CFU-GM	11.5EC+11.5Me	CFU-GM	AoEC+10.5MesP	-3.5854	1.5099	100	-2.37	0.0195	0.05	-6.5811	-0.5898
CFU-GM	11.5EC+11.5Me	CFU-GM	AoEC+11.5MesP	-3.5527	1.5100	100	-2.35	0.0206	0.05	-6.5484	-0.5570
CFU-GM	11.5EC+11.5Me	CFU-GM	E10.5-EC Only	43.1504	1.5100	100	28.58	<.0001	0.05	40.1547	46.1461
CFU-GM	11.5EC+11.5Me	CFU-GM	E10.5-MesP1+	43.1504	1.5100	100	28.58	<.0001	0.05	40.1547	46.1461
CFU-GM	11.5EC+11.5Me	CFU-GM	E10.5-MesP1-	43.1504	1.5100	100	28.58	<.0001	0.05	40.1547	46.1461
CFU-GM	11.5EC+11.5Me	CFU-GM	E11.5MesP1+On	43.1504	1.5100	100	28.58	<.0001	0.05	40.1547	46.1461
CFU-GM	11.5EC+11.5Me	CFU-GM	E11.5MesP1-On	43.1504	1.5100	100	28.58	<.0001	0.05	40.1547	46.1461
CFU-GM	11.5EC+11.5Me	CFU-GM	E13.5 EC Only	43.1504	1.5100	100	28.58	<.0001	0.05	40.1547	46.1461
CFU-GM	11.5EC+11.5Me	CFU-GM	H-EC Only	43.1504	1.5100	100	28.58	<.0001	0.05	40.1547	46.1461
CFU-GM	11.5EC+11.5Me	CFU-GM	H-EC+10.5MesP	-3.5671	1.5099	100	-2.36	0.0201	0.05	-6.5628	-0.5714

The GLIMMIX Procedure

Differences of Colony_Typ*Condition Least Squares Means											
Colony_Type	Condition	_Colony_Type	_Condition	Estimate	Standard Error	DF	t Value	Pr > |t|	Alpha	Lower	Upper
CFU-GM	11.5EC+11.5Me	CFU-GM	H-EC+11.5MesP	-3.4708	1.5100	100	-2.30	0.0236	0.05	-6.4666	-0.4750
CFU-GM	11.5EC+11.5Me	CFU-GM	I-EC Only	43.1504	1.5100	100	28.58	<.0001	0.05	40.1547	46.1461
CFU-GM	11.5EC+11.5Me	CFU-GM	I-EC+10.5MesP	-1.0522	1.5119	100	-0.70	0.4881	0.05	-4.0517	1.9473
CFU-GM	11.5EC+11.5Me	CFU-GM	I-EC+11.5MesP	-0.3612	1.5145	100	-0.24	0.8120	0.05	-3.3660	2.6436
CFU-GM	11.5EC+11.5Me	CFU-GM	L-E only	43.1504	1.5100	100	28.58	<.0001	0.05	40.1547	46.1461
CFU-GM	11.5EC+11.5Me	CFU-GM	L-E+11.5Mesp1	-3.6299	1.5100	100	-2.40	0.0181	0.05	-6.6257	-0.6342
CFU-GM	13.5EC+10.5Me	CFU-GM	13.5EC+11.5Me	-0.3035	1.6887	100	-0.18	0.8578	0.05	-3.6539	3.0470
CFU-GM	13.5EC+10.5Me	CFU-GM	Ao-EC Only	46.3785	1.6887	100	27.46	<.0001	0.05	43.0281	49.7289
CFU-GM	13.5EC+10.5Me	CFU-GM	AoEC+10.5MesP	-0.3574	1.6887	100	-0.21	0.8328	0.05	-3.7078	2.9930
CFU-GM	13.5EC+10.5Me	CFU-GM	AoEC+11.5MesP	-0.3247	1.6887	100	-0.19	0.8479	0.05	-3.6751	3.0258
CFU-GM	13.5EC+10.5Me	CFU-GM	E10.5-EC Only	46.3785	1.6887	100	27.46	<.0001	0.05	43.0281	49.7289
CFU-GM	13.5EC+10.5Me	CFU-GM	E10.5-MesP1+	46.3785	1.6887	100	27.46	<.0001	0.05	43.0281	49.7289
CFU-GM	13.5EC+10.5Me	CFU-GM	E10.5-MesP1-	46.3785	1.6887	100	27.46	<.0001	0.05	43.0281	49.7289
CFU-GM	13.5EC+10.5Me	CFU-GM	E11.5MesP1+On	46.3785	1.6887	100	27.46	<.0001	0.05	43.0281	49.7289
CFU-GM	13.5EC+10.5Me	CFU-GM	E11.5MesP1-On	46.3785	1.6887	100	27.46	<.0001	0.05	43.0281	49.7289
CFU-GM	13.5EC+10.5Me	CFU-GM	E13.5 EC Only	46.3785	1.6887	100	27.46	<.0001	0.05	43.0281	49.7289
CFU-GM	13.5EC+10.5Me	CFU-GM	H-EC Only	46.3785	1.6887	100	27.46	<.0001	0.05	43.0281	49.7289
CFU-GM	13.5EC+10.5Me	CFU-GM	H-EC+10.5MesP	-0.3391	1.6887	100	-0.20	0.8413	0.05	-3.6895	3.0113
CFU-GM	13.5EC+10.5Me	CFU-GM	H-EC+11.5MesP	-0.2427	1.6888	100	-0.14	0.8860	0.05	-3.5932	3.1077
CFU-GM	13.5EC+10.5Me	CFU-GM	I-EC Only	46.3785	1.6887	100	27.46	<.0001	0.05	43.0281	49.7289
CFU-GM	13.5EC+10.5Me	CFU-GM	I-EC+10.5MesP	2.1758	1.6906	100	1.29	0.2011	0.05	-1.1783	5.5299
CFU-GM	13.5EC+10.5Me	CFU-GM	I-EC+11.5MesP	2.8668	1.6927	100	1.69	0.0934	0.05	-0.4913	6.2250
CFU-GM	13.5EC+10.5Me	CFU-GM	L-E only	46.3785	1.6887	100	27.46	<.0001	0.05	43.0281	49.7289
CFU-GM	13.5EC+10.5Me	CFU-GM	L-E+11.5Mesp1	-0.4019	1.6887	100	-0.24	0.8124	0.05	-3.7523	2.9485

The GLIMMIX Procedure

Differences of Colony_Typ*Condition Least Squares Means											
Colony_Type	Condition	_Colony_Type	_Condition	Estimate	Standard Error	DF	t Value	Pr > |t|	Alpha	Lower	Upper
CFU-GM	13.5EC+11.5Me	CFU-GM	Ao-EC Only	46.6819	1.6887	100	27.64	<.0001	0.05	43.3316	50.0322
CFU-GM	13.5EC+11.5Me	CFU-GM	AoEC+10.5MesP	-0.05397	1.6887	100	-0.03	0.9746	0.05	-3.4043	3.2963
CFU-GM	13.5EC+11.5Me	CFU-GM	AoEC+11.5MesP	-0.02121	1.6887	100	-0.01	0.9900	0.05	-3.3715	3.3291
CFU-GM	13.5EC+11.5Me	CFU-GM	E10.5-EC Only	46.6819	1.6887	100	27.64	<.0001	0.05	43.3316	50.0322
CFU-GM	13.5EC+11.5Me	CFU-GM	E10.5-MesP1+	46.6819	1.6887	100	27.64	<.0001	0.05	43.3316	50.0322
CFU-GM	13.5EC+11.5Me	CFU-GM	E10.5-MesP1-	46.6819	1.6887	100	27.64	<.0001	0.05	43.3316	50.0322
CFU-GM	13.5EC+11.5Me	CFU-GM	E11.5MesP1+On	46.6819	1.6887	100	27.64	<.0001	0.05	43.3316	50.0322
CFU-GM	13.5EC+11.5Me	CFU-GM	E11.5MesP1-On	46.6819	1.6887	100	27.64	<.0001	0.05	43.3316	50.0322
CFU-GM	13.5EC+11.5Me	CFU-GM	E13.5 EC Only	46.6819	1.6887	100	27.64	<.0001	0.05	43.3316	50.0322
CFU-GM	13.5EC+11.5Me	CFU-GM	H-EC Only	46.6819	1.6887	100	27.64	<.0001	0.05	43.3316	50.0322
CFU-GM	13.5EC+11.5Me	CFU-GM	H-EC+10.5MesP	-0.03563	1.6887	100	-0.02	0.9832	0.05	-3.3859	3.3147
CFU-GM	13.5EC+11.5Me	CFU-GM	H-EC+11.5MesP	0.06070	1.6887	100	0.04	0.9714	0.05	-3.2896	3.4110
CFU-GM	13.5EC+11.5Me	CFU-GM	I-EC Only	46.6819	1.6887	100	27.64	<.0001	0.05	43.3316	50.0322
CFU-GM	13.5EC+11.5Me	CFU-GM	I-EC+10.5MesP	2.4793	1.6906	100	1.47	0.1456	0.05	-0.8748	5.8333
CFU-GM	13.5EC+11.5Me	CFU-GM	I-EC+11.5MesP	3.1703	1.6926	100	1.87	0.0640	0.05	-0.1878	6.5284
CFU-GM	13.5EC+11.5Me	CFU-GM	L-E only	46.6819	1.6887	100	27.64	<.0001	0.05	43.3316	50.0322
CFU-GM	13.5EC+11.5Me	CFU-GM	L-E+11.5Mesp1	-0.09847	1.6887	100	-0.06	0.9536	0.05	-3.4487	3.2518
CFU-GM	Ao-EC Only	CFU-GM	AoEC+10.5MesP	-46.7359	1.6887	100	-27.68	<.0001	0.05	-50.0861	-43.3856
CFU-GM	Ao-EC Only	CFU-GM	AoEC+11.5MesP	-46.7031	1.6887	100	-27.66	<.0001	0.05	-50.0534	-43.3528
CFU-GM	Ao-EC Only	CFU-GM	E10.5-EC Only	1.42E-14	.	.	.	.	.	.	.
CFU-GM	Ao-EC Only	CFU-GM	E10.5-MesP1+	7.82E-14	.	.	.	.	.	.	.
CFU-GM	Ao-EC Only	CFU-GM	E10.5-MesP1-	-711E-17	.	.	.	.	.	.	.
CFU-GM	Ao-EC Only	CFU-GM	E11.5MesP1+On	1.42E-14	.	.	.	.	.	.	.
CFU-GM	Ao-EC Only	CFU-GM	E11.5MesP1-On	-853E-16	.	.	.	.	.	.	.

The GLIMMIX Procedure

Differences of Colony_Typ*Condition Least Squares Means

Colony_Type	Condition	_Colony_Type	_Condition	Estimate	Standard Error	DF	t Value	Pr > |t|	Alpha	Lower	Upper
CFU-GM	Ao-EC Only	CFU-GM	E13.5 EC Only	9.95E-14	.	.	.	.	.	.	.
CFU-GM	Ao-EC Only	CFU-GM	H-EC Only	1.42E-14	.	.	.	.	.	.	.
CFU-GM	Ao-EC Only	CFU-GM	H-EC+10.5MesP	-46.7175	1.6887	100	-27.67	<.0001	0.05	-50.0678	-43.3673
CFU-GM	Ao-EC Only	CFU-GM	H-EC+11.5MesP	-46.6212	1.6887	100	-27.61	<.0001	0.05	-49.9715	-43.2709
CFU-GM	Ao-EC Only	CFU-GM	I-EC Only	-284E-16	.	.	.	.	.	.	.
CFU-GM	Ao-EC Only	CFU-GM	I-EC+10.5MesP	-44.2026	1.6905	100	-26.15	<.0001	0.05	-47.5566	-40.8487
CFU-GM	Ao-EC Only	CFU-GM	I-EC+11.5MesP	-43.5116	1.6926	100	-25.71	<.0001	0.05	-46.8696	-40.1536
CFU-GM	Ao-EC Only	CFU-GM	L-E only	8.53E-14	.	.	.	.	.	.	.
CFU-GM	Ao-EC Only	CFU-GM	L-E+11.5Mesp1	-46.7804	0	100	-Infty	<.0001	.	.	.
CFU-GM	AoEC+10.5MesP	CFU-GM	AoEC+11.5MesP	0.03275	1.6887	100	0.02	0.9846	0.05	-3.3175	3.3830
CFU-GM	AoEC+10.5MesP	CFU-GM	E10.5-EC Only	46.7359	1.6887	100	27.68	<.0001	0.05	43.3856	50.0861
CFU-GM	AoEC+10.5MesP	CFU-GM	E10.5-MesP1+	46.7359	1.6887	100	27.68	<.0001	0.05	43.3856	50.0861
CFU-GM	AoEC+10.5MesP	CFU-GM	E10.5-MesP1-	46.7359	1.6887	100	27.68	<.0001	0.05	43.3856	50.0861
CFU-GM	AoEC+10.5MesP	CFU-GM	E11.5MesP1+On	46.7359	1.6887	100	27.68	<.0001	0.05	43.3856	50.0861
CFU-GM	AoEC+10.5MesP	CFU-GM	E11.5MesP1-On	46.7359	1.6887	100	27.68	<.0001	0.05	43.3856	50.0861
CFU-GM	AoEC+10.5MesP	CFU-GM	E13.5 EC Only	46.7359	1.6887	100	27.68	<.0001	0.05	43.3856	50.0861
CFU-GM	AoEC+10.5MesP	CFU-GM	H-EC Only	46.7359	1.6887	100	27.68	<.0001	0.05	43.3856	50.0861
CFU-GM	AoEC+10.5MesP	CFU-GM	H-EC+10.5MesP	0.01834	1.6887	100	0.01	0.9914	0.05	-3.3319	3.3686
CFU-GM	AoEC+10.5MesP	CFU-GM	H-EC+11.5MesP	0.1147	1.6887	100	0.07	0.9460	0.05	-3.2356	3.4650
CFU-GM	AoEC+10.5MesP	CFU-GM	I-EC Only	46.7359	1.6887	100	27.68	<.0001	0.05	43.3856	50.0861
CFU-GM	AoEC+10.5MesP	CFU-GM	I-EC+10.5MesP	2.5332	1.6905	100	1.50	0.1372	0.05	-0.8208	5.8872
CFU-GM	AoEC+10.5MesP	CFU-GM	I-EC+11.5MesP	3.2243	1.6926	100	1.90	0.0597	0.05	-0.1338	6.5823
CFU-GM	AoEC+10.5MesP	CFU-GM	L-E only	46.7359	1.6887	100	27.68	<.0001	0.05	43.3856	50.0861
CFU-GM	AoEC+10.5MesP	CFU-GM	L-E+11.5Mesp1	-0.04450	1.6887	100	-0.03	0.9790	0.05	-3.3948	3.3058

The GLIMMIX Procedure

Differences of Colony_Typ*Condition Least Squares Means											
Colony_Type	Condition	_Colony_Type	_Condition	Estimate	Standard Error	DF	t Value	Pr > |t|	Alpha	Lower	Upper
CFU-GM	AoEC+11.5MesP	CFU-GM	E10.5-EC Only	46.7031	1.6887	100	27.66	<.0001	0.05	43.3528	50.0534
CFU-GM	AoEC+11.5MesP	CFU-GM	E10.5-MesP1+	46.7031	1.6887	100	27.66	<.0001	0.05	43.3528	50.0534
CFU-GM	AoEC+11.5MesP	CFU-GM	E10.5-MesP1-	46.7031	1.6887	100	27.66	<.0001	0.05	43.3528	50.0534
CFU-GM	AoEC+11.5MesP	CFU-GM	E11.5MesP1+On	46.7031	1.6887	100	27.66	<.0001	0.05	43.3528	50.0534
CFU-GM	AoEC+11.5MesP	CFU-GM	E11.5MesP1-On	46.7031	1.6887	100	27.66	<.0001	0.05	43.3528	50.0534
CFU-GM	AoEC+11.5MesP	CFU-GM	E13.5 EC Only	46.7031	1.6887	100	27.66	<.0001	0.05	43.3528	50.0534
CFU-GM	AoEC+11.5MesP	CFU-GM	H-EC Only	46.7031	1.6887	100	27.66	<.0001	0.05	43.3528	50.0534
CFU-GM	AoEC+11.5MesP	CFU-GM	H-EC+10.5MesP	-0.01441	1.6887	100	-0.01	0.9932	0.05	-3.3647	3.3359
CFU-GM	AoEC+11.5MesP	CFU-GM	H-EC+11.5MesP	0.08192	1.6887	100	0.05	0.9614	0.05	-3.2684	3.4322
CFU-GM	AoEC+11.5MesP	CFU-GM	I-EC Only	46.7031	1.6887	100	27.66	<.0001	0.05	43.3528	50.0534
CFU-GM	AoEC+11.5MesP	CFU-GM	I-EC+10.5MesP	2.5005	1.6906	100	1.48	0.1423	0.05	-0.8535	5.8545
CFU-GM	AoEC+11.5MesP	CFU-GM	I-EC+11.5MesP	3.1915	1.6926	100	1.89	0.0623	0.05	-0.1666	6.5496
CFU-GM	AoEC+11.5MesP	CFU-GM	L-E only	46.7031	1.6887	100	27.66	<.0001	0.05	43.3528	50.0534
CFU-GM	AoEC+11.5MesP	CFU-GM	L-E+11.5Mesp1	-0.07725	1.6887	100	-0.05	0.9636	0.05	-3.4275	3.2730
CFU-GM	E10.5-EC Only	CFU-GM	E10.5-MesP1+	6.39E-14	.	.	.	.	.	.	.
CFU-GM	E10.5-EC Only	CFU-GM	E10.5-MesP1-	-213E-16	.	.	.	.	.	.	.
CFU-GM	E10.5-EC Only	CFU-GM	E11.5MesP1+On	0	.	.	.	.	.	.	.
CFU-GM	E10.5-EC Only	CFU-GM	E11.5MesP1-On	-995E-16	.	.	.	.	.	.	.
CFU-GM	E10.5-EC Only	CFU-GM	E13.5 EC Only	8.53E-14	.	.	.	.	.	.	.
CFU-GM	E10.5-EC Only	CFU-GM	H-EC Only	0	.	.	.	.	.	.	.
CFU-GM	E10.5-EC Only	CFU-GM	H-EC+10.5MesP	-46.7175	1.6887	100	-27.67	<.0001	0.05	-50.0678	-43.3673
CFU-GM	E10.5-EC Only	CFU-GM	H-EC+11.5MesP	-46.6212	1.6887	100	-27.61	<.0001	0.05	-49.9715	-43.2709
CFU-GM	E10.5-EC Only	CFU-GM	I-EC Only	-426E-16	.	.	.	.	.	.	.
CFU-GM	E10.5-EC Only	CFU-GM	I-EC+10.5MesP	-44.2026	1.6905	100	-26.15	<.0001	0.05	-47.5566	-40.8487

The GLIMMIX Procedure

Differences of Colony_Typ*Condition Least Squares Means											
Colony_Type	Condition	_Colony_Type	_Condition	Estimate	Standard Error	DF	t Value	Pr > |t|	Alpha	Lower	Upper
CFU-GM	E10.5-EC Only	CFU-GM	I-EC+11.5MesP	-43.5116	1.6926	100	-25.71	<.0001	0.05	-46.8696	-40.1536
CFU-GM	E10.5-EC Only	CFU-GM	L-E only	7.11E-14	.	.	.	.	.	.	.
CFU-GM	E10.5-EC Only	CFU-GM	L-E+11.5Mesp1	-46.7804	0	100	-Infty	<.0001	.	.	.
CFU-GM	E10.5-MesP1+	CFU-GM	E10.5-MesP1-	-853E-16	.	.	.	.	.	.	.
CFU-GM	E10.5-MesP1+	CFU-GM	E11.5MesP1+On	-639E-16	.	.	.	.	.	.	.
CFU-GM	E10.5-MesP1+	CFU-GM	E11.5MesP1-On	-163E-15	.	.	.	.	.	.	.
CFU-GM	E10.5-MesP1+	CFU-GM	E13.5 EC Only	2.13E-14	.	.	.	.	.	.	.
CFU-GM	E10.5-MesP1+	CFU-GM	H-EC Only	-639E-16	.	.	.	.	.	.	.
CFU-GM	E10.5-MesP1+	CFU-GM	H-EC+10.5MesP	-46.7175	1.6887	100	-27.67	<.0001	0.05	-50.0678	-43.3673
CFU-GM	E10.5-MesP1+	CFU-GM	H-EC+11.5MesP	-46.6212	1.6887	100	-27.61	<.0001	0.05	-49.9715	-43.2709
CFU-GM	E10.5-MesP1+	CFU-GM	I-EC Only	-107E-15	.	.	.	.	.	.	.
CFU-GM	E10.5-MesP1+	CFU-GM	I-EC+10.5MesP	-44.2026	1.6905	100	-26.15	<.0001	0.05	-47.5566	-40.8487
CFU-GM	E10.5-MesP1+	CFU-GM	I-EC+11.5MesP	-43.5116	1.6926	100	-25.71	<.0001	0.05	-46.8696	-40.1536
CFU-GM	E10.5-MesP1+	CFU-GM	L-E only	7.11E-15	.	.	.	.	.	.	.
CFU-GM	E10.5-MesP1+	CFU-GM	L-E+11.5Mesp1	-46.7804	0	100	-Infty	<.0001	.	.	.
CFU-GM	E10.5-MesP1-	CFU-GM	E11.5MesP1+On	2.13E-14	.	.	.	.	.	.	.
CFU-GM	E10.5-MesP1-	CFU-GM	E11.5MesP1-On	-782E-16	.	.	.	.	.	.	.
CFU-GM	E10.5-MesP1-	CFU-GM	E13.5 EC Only	1.07E-13	.	.	.	.	.	.	.
CFU-GM	E10.5-MesP1-	CFU-GM	H-EC Only	2.13E-14	.	.	.	.	.	.	.
CFU-GM	E10.5-MesP1-	CFU-GM	H-EC+10.5MesP	-46.7175	1.6887	100	-27.67	<.0001	0.05	-50.0678	-43.3673
CFU-GM	E10.5-MesP1-	CFU-GM	H-EC+11.5MesP	-46.6212	1.6887	100	-27.61	<.0001	0.05	-49.9715	-43.2709
CFU-GM	E10.5-MesP1-	CFU-GM	I-EC Only	-213E-16	.	.	.	.	.	.	.
CFU-GM	E10.5-MesP1-	CFU-GM	I-EC+10.5MesP	-44.2026	1.6905	100	-26.15	<.0001	0.05	-47.5566	-40.8487
CFU-GM	E10.5-MesP1-	CFU-GM	I-EC+11.5MesP	-43.5116	1.6926	100	-25.71	<.0001	0.05	-46.8696	-40.1536

The GLIMMIX Procedure

Differences of Colony_Typ*Condition Least Squares Means											
Colony_Type	Condition	_Colony_Type	_Condition	Estimate	Standard Error	DF	t Value	Pr > |t|	Alpha	Lower	Upper
CFU-GM	E10.5-MesP1-	CFU-GM	L-E only	9.24E-14	.	.	.	.	.	.	.
CFU-GM	E10.5-MesP1-	CFU-GM	L-E+11.5Mesp1	-46.7804	0	100	-Infty	<.0001	.	.	.
CFU-GM	E11.5MesP1+On	CFU-GM	E11.5MesP1-On	-995E-16	.	.	.	.	.	.	.
CFU-GM	E11.5MesP1+On	CFU-GM	E13.5 EC Only	8.53E-14	.	.	.	.	.	.	.
CFU-GM	E11.5MesP1+On	CFU-GM	H-EC Only	0	.	.	.	.	.	.	.
CFU-GM	E11.5MesP1+On	CFU-GM	H-EC+10.5MesP	-46.7175	1.6887	100	-27.67	<.0001	0.05	-50.0678	-43.3673
CFU-GM	E11.5MesP1+On	CFU-GM	H-EC+11.5MesP	-46.6212	1.6887	100	-27.61	<.0001	0.05	-49.9715	-43.2709
CFU-GM	E11.5MesP1+On	CFU-GM	I-EC Only	-426E-16	.	.	.	.	.	.	.
CFU-GM	E11.5MesP1+On	CFU-GM	I-EC+10.5MesP	-44.2026	1.6905	100	-26.15	<.0001	0.05	-47.5566	-40.8487
CFU-GM	E11.5MesP1+On	CFU-GM	I-EC+11.5MesP	-43.5116	1.6926	100	-25.71	<.0001	0.05	-46.8696	-40.1536
CFU-GM	E11.5MesP1+On	CFU-GM	L-E only	7.11E-14	.	.	.	.	.	.	.
CFU-GM	E11.5MesP1+On	CFU-GM	L-E+11.5Mesp1	-46.7804	0	100	-Infty	<.0001	.	.	.
CFU-GM	E11.5MesP1-On	CFU-GM	E13.5 EC Only	1.85E-13	.	.	.	.	.	.	.
CFU-GM	E11.5MesP1-On	CFU-GM	H-EC Only	9.95E-14	.	.	.	.	.	.	.
CFU-GM	E11.5MesP1-On	CFU-GM	H-EC+10.5MesP	-46.7175	1.6887	100	-27.67	<.0001	0.05	-50.0678	-43.3673
CFU-GM	E11.5MesP1-On	CFU-GM	H-EC+11.5MesP	-46.6212	1.6887	100	-27.61	<.0001	0.05	-49.9715	-43.2709
CFU-GM	E11.5MesP1-On	CFU-GM	I-EC Only	5.68E-14	.	.	.	.	.	.	.
CFU-GM	E11.5MesP1-On	CFU-GM	I-EC+10.5MesP	-44.2026	1.6905	100	-26.15	<.0001	0.05	-47.5566	-40.8487
CFU-GM	E11.5MesP1-On	CFU-GM	I-EC+11.5MesP	-43.5116	1.6926	100	-25.71	<.0001	0.05	-46.8696	-40.1536
CFU-GM	E11.5MesP1-On	CFU-GM	L-E only	1.71E-13	.	.	.	.	.	.	.
CFU-GM	E11.5MesP1-On	CFU-GM	L-E+11.5Mesp1	-46.7804	0	100	-Infty	<.0001	.	.	.
CFU-GM	E13.5 EC Only	CFU-GM	H-EC Only	-853E-16	.	.	.	.	.	.	.
CFU-GM	E13.5 EC Only	CFU-GM	H-EC+10.5MesP	-46.7175	1.6887	100	-27.67	<.0001	0.05	-50.0678	-43.3673
CFU-GM	E13.5 EC Only	CFU-GM	H-EC+11.5MesP	-46.6212	1.6887	100	-27.61	<.0001	0.05	-49.9715	-43.2709

The GLIMMIX Procedure

Differences of Colony_Typ*Condition Least Squares Means											
Colony_Type	Condition	_Colony_Type	_Condition	Estimate	Standard Error	DF	t Value	Pr > |t|	Alpha	Lower	Upper
CFU-GM	E13.5 EC Only	CFU-GM	I-EC Only	-128E-15	.	.	.	.	.	.	.
CFU-GM	E13.5 EC Only	CFU-GM	I-EC+10.5MesP	-44.2026	1.6905	100	-26.15	<.0001	0.05	-47.5566	-40.8487
CFU-GM	E13.5 EC Only	CFU-GM	I-EC+11.5MesP	-43.5116	1.6926	100	-25.71	<.0001	0.05	-46.8696	-40.1536
CFU-GM	E13.5 EC Only	CFU-GM	L-E only	-142E-16	.	.	.	.	.	.	.
CFU-GM	E13.5 EC Only	CFU-GM	L-E+11.5Mesp1	-46.7804	0	100	-Infty	<.0001	.	.	.
CFU-GM	H-EC Only	CFU-GM	H-EC+10.5MesP	-46.7175	1.6887	100	-27.67	<.0001	0.05	-50.0678	-43.3673
CFU-GM	H-EC Only	CFU-GM	H-EC+11.5MesP	-46.6212	1.6887	100	-27.61	<.0001	0.05	-49.9715	-43.2709
CFU-GM	H-EC Only	CFU-GM	I-EC Only	-426E-16	.	.	.	.	.	.	.
CFU-GM	H-EC Only	CFU-GM	I-EC+10.5MesP	-44.2026	1.6905	100	-26.15	<.0001	0.05	-47.5566	-40.8487
CFU-GM	H-EC Only	CFU-GM	I-EC+11.5MesP	-43.5116	1.6926	100	-25.71	<.0001	0.05	-46.8696	-40.1536
CFU-GM	H-EC Only	CFU-GM	L-E only	7.11E-14	.	.	.	.	.	.	.
CFU-GM	H-EC Only	CFU-GM	L-E+11.5Mesp1	-46.7804	0	100	-Infty	<.0001	.	.	.
CFU-GM	H-EC+10.5MesP	CFU-GM	H-EC+11.5MesP	0.09633	1.6887	100	0.06	0.9546	0.05	-3.2540	3.4466
CFU-GM	H-EC+10.5MesP	CFU-GM	I-EC Only	46.7175	1.6887	100	27.67	<.0001	0.05	43.3673	50.0678
CFU-GM	H-EC+10.5MesP	CFU-GM	I-EC+10.5MesP	2.5149	1.6905	100	1.49	0.1400	0.05	-0.8391	5.8689
CFU-GM	H-EC+10.5MesP	CFU-GM	I-EC+11.5MesP	3.2059	1.6926	100	1.89	0.0611	0.05	-0.1521	6.5640
CFU-GM	H-EC+10.5MesP	CFU-GM	L-E only	46.7175	1.6887	100	27.67	<.0001	0.05	43.3673	50.0678
CFU-GM	H-EC+10.5MesP	CFU-GM	L-E+11.5Mesp1	-0.06284	1.6887	100	-0.04	0.9704	0.05	-3.4131	3.2874
CFU-GM	H-EC+11.5MesP	CFU-GM	I-EC Only	46.6212	1.6887	100	27.61	<.0001	0.05	43.2709	49.9715
CFU-GM	H-EC+11.5MesP	CFU-GM	I-EC+10.5MesP	2.4186	1.6906	100	1.43	0.1557	0.05	-0.9355	5.7726
CFU-GM	H-EC+11.5MesP	CFU-GM	I-EC+11.5MesP	3.1096	1.6926	100	1.84	0.0692	0.05	-0.2485	6.4677
CFU-GM	H-EC+11.5MesP	CFU-GM	L-E only	46.6212	1.6887	100	27.61	<.0001	0.05	43.2709	49.9715
CFU-GM	H-EC+11.5MesP	CFU-GM	L-E+11.5Mesp1	-0.1592	1.6887	100	-0.09	0.9251	0.05	-3.5095	3.1911
CFU-GM	I-EC Only	CFU-GM	I-EC+10.5MesP	-44.2026	1.6905	100	-26.15	<.0001	0.05	-47.5566	-40.8487

The GLIMMIX Procedure

Differences of Colony_Typ*Condition Least Squares Means											
Colony_Type	Condition	_Colony_Type	_Condition	Estimate	Standard Error	DF	t Value	Pr > |t|	Alpha	Lower	Upper
CFU-GM	I-EC Only	CFU-GM	I-EC+11.5MesP	-43.5116	1.6926	100	-25.71	<.0001	0.05	-46.8696	-40.1536
CFU-GM	I-EC Only	CFU-GM	L-E only	1.14E-13	.	.	.	.	.	.	.
CFU-GM	I-EC Only	CFU-GM	L-E+11.5Mesp1	-46.7804	0	100	-Infty	<.0001	.	.	.
CFU-GM	I-EC+10.5MesP	CFU-GM	I-EC+11.5MesP	0.6910	1.6945	100	0.41	0.6843	0.05	-2.6707	4.0528
CFU-GM	I-EC+10.5MesP	CFU-GM	L-E only	44.2026	1.6905	100	26.15	<.0001	0.05	40.8487	47.5566
CFU-GM	I-EC+10.5MesP	CFU-GM	L-E+11.5Mesp1	-2.5777	1.6905	100	-1.52	0.1305	0.05	-5.9317	0.7763
CFU-GM	I-EC+11.5MesP	CFU-GM	L-E only	43.5116	1.6926	100	25.71	<.0001	0.05	40.1536	46.8696
CFU-GM	I-EC+11.5MesP	CFU-GM	L-E+11.5Mesp1	-3.2688	1.6926	100	-1.93	0.0563	0.05	-6.6268	0.08927
CFU-GM	L-E only	CFU-GM	L-E+11.5Mesp1	-46.7804	0	100	-Infty	<.0001	.	.	.

The GENMOD Procedure

Model Information	
**Data Set**	WORK.FIG4AIV
**Distribution**	Normal
**Link Function**	Identity
**Dependent Variable**	Percentage

**Number of Observations Read**	111
**Number of Observations Used**	111

Class Level Information		
**Class**	**Levels**	**Values**
**Condition**	21	10.5AGM Ao EC Only AoE+E10.5PSC AoE+E11.5PSC E10.5EC Only E10.5EC+E10.5PSC E11.5AGM E11.5EC Only E11.5EC+E11.5PSC E11.5PSC only E13.5EC Only E13.5EC+E10.5PSC E13.5EC+E11.5PSC HE Only HE+E10.5PSC HE+E11.5PSC IVCE Only IVCE+E10.5PSC IVCE+E11.5PSC LE Only ...

Parameter Information		
**Parameter**	**Effect**	**Condition**
**Prm1**	Intercept	
**Prm2**	Condition	10.5AGM
**Prm3**	Condition	Ao EC Only
**Prm4**	Condition	AoE+E10.5PSC
**Prm5**	Condition	AoE+E11.5PSC
**Prm6**	Condition	E10.5EC Only
**Prm7**	Condition	E10.5EC+E10.5PSC
**Prm8**	Condition	E11.5AGM
**Prm9**	Condition	E11.5EC Only
**Prm10**	Condition	E11.5EC+E11.5PSC
**Prm11**	Condition	E11.5PSC only

The GENMOD Procedure

Parameter Information		
**Parameter**	**Effect**	**Condition**
**Prm12**	Condition	E13.5EC Only
**Prm13**	Condition	E13.5EC+E10.5PSC
**Prm14**	Condition	E13.5EC+E11.5PSC
**Prm15**	Condition	HE Only
**Prm16**	Condition	HE+E10.5PSC
**Prm17**	Condition	HE+E11.5PSC
**Prm18**	Condition	IVCE Only
**Prm19**	Condition	IVCE+E10.5PSC
**Prm20**	Condition	IVCE+E11.5PSC
**Prm21**	Condition	LE Only
**Prm22**	Condition	LE+E11.5PSC

Criteria For Assessing Goodness Of Fit			
**Criterion**	**DF**	**Value**	**Value/DF**
**Deviance**	90	3665.8500	40.7317
**Scaled Deviance**	90	111.0000	1.2333
**Pearson Chi-Square**	90	3665.8500	40.7317
**Scaled Pearson X2**	90	111.0000	1.2333
**Log Likelihood**		-351.6015	
**Full Log Likelihood**		-351.6015	
**AIC (smaller is better)**		747.2030	
**AICC (smaller is better)**		758.7030	
**BIC (smaller is better)**		806.8127	

Algorithm converged.

The GENMOD Procedure

Analysis Of Maximum Likelihood Parameter Estimates								
**Parameter**		**DF**	**Estimate**	**Standard Error**	**Wald 95% Confidence Limits**		**Wald Chi-Square**	**Pr > ChiSq**
**Intercept**		1	26.2500	2.8734	20.6182	31.8818	83.46	<.0001
**Condition**	10.5AGM	1	32.5500	3.8551	24.9942	40.1058	71.29	<.0001
**Condition**	Ao EC Only	1	-26.2500	3.8551	-33.8058	-18.6942	46.37	<.0001
**Condition**	AoE+E10.5PSC	1	5.5500	3.8551	-2.0058	13.1058	2.07	0.1500
**Condition**	AoE+E11.5PSC	1	32.9500	3.8551	25.3942	40.5058	73.05	<.0001
**Condition**	E10.5EC Only	1	-26.2500	3.8551	-33.8058	-18.6942	46.37	<.0001
**Condition**	E10.5EC+E10.5PSC	1	23.1500	3.8551	15.5942	30.7058	36.06	<.0001
**Condition**	E11.5AGM	1	38.7500	3.8551	31.1942	46.3058	101.04	<.0001
**Condition**	E11.5EC Only	1	-26.2500	3.8551	-33.8058	-18.6942	46.37	<.0001
**Condition**	E11.5EC+E11.5PSC	1	-12.0500	3.3999	-18.7136	-5.3864	12.56	0.0004
**Condition**	E11.5PSC only	1	-26.2500	3.8551	-33.8058	-18.6942	46.37	<.0001
**Condition**	E13.5EC Only	1	-26.2500	3.8551	-33.8058	-18.6942	46.37	<.0001
**Condition**	E13.5EC+E10.5PSC	1	-2.4500	3.8551	-10.0058	5.1058	0.40	0.5251
**Condition**	E13.5EC+E11.5PSC	1	-12.4500	3.8551	-20.0058	-4.8942	10.43	0.0012
**Condition**	HE Only	1	-26.2500	3.7095	-33.5206	-18.9794	50.07	<.0001
**Condition**	HE+E10.5PSC	1	-4.6500	3.8551	-12.2058	2.9058	1.45	0.2277
**Condition**	HE+E11.5PSC	1	-16.7500	3.7095	-24.0206	-9.4794	20.39	<.0001
**Condition**	IVCE Only	1	-26.2500	3.8551	-33.8058	-18.6942	46.37	<.0001
**Condition**	IVCE+E10.5PSC	1	-22.4500	3.8551	-30.0058	-14.8942	33.91	<.0001
**Condition**	IVCE+E11.5PSC	1	-22.0500	3.8551	-29.6058	-14.4942	32.72	<.0001
**Condition**	LE Only	1	-26.2500	3.8551	-33.8058	-18.6942	46.37	<.0001
**Condition**	LE+E11.5PSC	0	0.0000	0.0000	0.0000	0.0000	.	.
**Scale**		1	5.7468	0.3857	5.0384	6.5547		

Note: The scale parameter was estimated by maximum likelihood.

The GENMOD Procedure

Condition Least Squares Means							
Condition	Estimate	Standard Error	z Value	Pr > |z|	Alpha	Lower	Upper
10.5AGM	58.8000	2.5700	22.88	<.0001	0.05	53.7628	63.8372
Ao EC Only	0	2.5700	0.00	1.0000	0.05	-5.0372	5.0372
AoE+E10.5PSC	31.8000	2.5700	12.37	<.0001	0.05	26.7628	36.8372
AoE+E11.5PSC	59.2000	2.5700	23.03	<.0001	0.05	54.1628	64.2372
E10.5EC Only	3.55E-15	2.5700	0.00	1.0000	0.05	-5.0372	5.0372
E10.5EC+E10.5PSC	49.4000	2.5700	19.22	<.0001	0.05	44.3628	54.4372
E11.5AGM	65.0000	2.5700	25.29	<.0001	0.05	59.9628	70.0372
E11.5EC Only	7.11E-15	2.5700	0.00	1.0000	0.05	-5.0372	5.0372
E11.5EC+E11.5PSC	14.2000	1.8173	7.81	<.0001	0.05	10.6382	17.7618
E11.5PSC only	0	2.5700	0.00	1.0000	0.05	-5.0372	5.0372
E13.5EC Only	0	2.5700	0.00	1.0000	0.05	-5.0372	5.0372
E13.5EC+E10.5PSC	23.8000	2.5700	9.26	<.0001	0.05	18.7628	28.8372
E13.5EC+E11.5PSC	13.8000	2.5700	5.37	<.0001	0.05	8.7628	18.8372
HE Only	0	2.3461	0.00	1.0000	0.05	-4.5983	4.5983
HE+E10.5PSC	21.6000	2.5700	8.40	<.0001	0.05	16.5628	26.6372
HE+E11.5PSC	9.5000	2.3461	4.05	<.0001	0.05	4.9017	14.0983
IVCE Only	0	2.5700	0.00	1.0000	0.05	-5.0372	5.0372
IVCE+E10.5PSC	3.8000	2.5700	1.48	0.1393	0.05	-1.2372	8.8372
IVCE+E11.5PSC	4.2000	2.5700	1.63	0.1022	0.05	-0.8372	9.2372
LE Only	0	2.5700	0.00	1.0000	0.05	-5.0372	5.0372
LE+E11.5PSC	26.2500	2.8734	9.14	<.0001	0.05	20.6182	31.8818

The GENMOD Procedure

Differences of Condition Least Squares Means								
Condition	_Condition	Estimate	Standard Error	z Value	Pr > |z|	Alpha	Lower	Upper
10.5AGM	Ao EC Only	58.8000	3.6346	16.18	<.0001	0.05	51.6763	65.9237
10.5AGM	AoE+E10.5PSC	27.0000	3.6346	7.43	<.0001	0.05	19.8763	34.1237
10.5AGM	AoE+E11.5PSC	-0.4000	3.6346	-0.11	0.9124	0.05	-7.5237	6.7237
10.5AGM	E10.5EC Only	58.8000	3.6346	16.18	<.0001	0.05	51.6763	65.9237
10.5AGM	E10.5EC+E10.5PSC	9.4000	3.6346	2.59	0.0097	0.05	2.2763	16.5237
10.5AGM	E11.5AGM	-6.2000	3.6346	-1.71	0.0880	0.05	-13.3237	0.9237
10.5AGM	E11.5EC Only	58.8000	3.6346	16.18	<.0001	0.05	51.6763	65.9237
10.5AGM	E11.5EC+E11.5PSC	44.6000	3.1477	14.17	<.0001	0.05	38.4307	50.7693
10.5AGM	E11.5PSC only	58.8000	3.6346	16.18	<.0001	0.05	51.6763	65.9237
10.5AGM	E13.5EC Only	58.8000	3.6346	16.18	<.0001	0.05	51.6763	65.9237
10.5AGM	E13.5EC+E10.5PSC	35.0000	3.6346	9.63	<.0001	0.05	27.8763	42.1237
10.5AGM	E13.5EC+E11.5PSC	45.0000	3.6346	12.38	<.0001	0.05	37.8763	52.1237
10.5AGM	HE Only	58.8000	3.4799	16.90	<.0001	0.05	51.9796	65.6204
10.5AGM	HE+E10.5PSC	37.2000	3.6346	10.23	<.0001	0.05	30.0763	44.3237
10.5AGM	HE+E11.5PSC	49.3000	3.4799	14.17	<.0001	0.05	42.4796	56.1204
10.5AGM	IVCE Only	58.8000	3.6346	16.18	<.0001	0.05	51.6763	65.9237
10.5AGM	IVCE+E10.5PSC	55.0000	3.6346	15.13	<.0001	0.05	47.8763	62.1237
10.5AGM	IVCE+E11.5PSC	54.6000	3.6346	15.02	<.0001	0.05	47.4763	61.7237
10.5AGM	LE Only	58.8000	3.6346	16.18	<.0001	0.05	51.6763	65.9237
10.5AGM	LE+E11.5PSC	32.5500	3.8551	8.44	<.0001	0.05	24.9942	40.1058
Ao EC Only	AoE+E10.5PSC	-31.8000	3.6346	-8.75	<.0001	0.05	-38.9237	-24.6763
Ao EC Only	AoE+E11.5PSC	-59.2000	3.6346	-16.29	<.0001	0.05	-66.3237	-52.0763
Ao EC Only	E10.5EC Only	-355E-17	3.6346	-0.00	1.0000	0.05	-7.1237	7.1237
Ao EC Only	E10.5EC+E10.5PSC	-49.4000	3.6346	-13.59	<.0001	0.05	-56.5237	-42.2763

The GENMOD Procedure

Differences of Condition Least Squares Means

Condition	_Condition	Estimate	Standard Error	z Value	Pr > |z|	Alpha	Lower	Upper
Ao EC Only	E11.5AGM	-65.0000	3.6346	-17.88	<.0001	0.05	-72.1237	-57.8763
Ao EC Only	E11.5EC Only	-711E-17	3.6346	-0.00	1.0000	0.05	-7.1237	7.1237
Ao EC Only	E11.5EC+E11.5PSC	-14.2000	3.1477	-4.51	<.0001	0.05	-20.3693	-8.0307
Ao EC Only	E11.5PSC only	0	3.6346	0.00	1.0000	0.05	-7.1237	7.1237
Ao EC Only	E13.5EC Only	0	3.6346	0.00	1.0000	0.05	-7.1237	7.1237
Ao EC Only	E13.5EC+E10.5PSC	-23.8000	3.6346	-6.55	<.0001	0.05	-30.9237	-16.6763
Ao EC Only	E13.5EC+E11.5PSC	-13.8000	3.6346	-3.80	0.0001	0.05	-20.9237	-6.6763
Ao EC Only	HE Only	0	3.4799	0.00	1.0000	0.05	-6.8204	6.8204
Ao EC Only	HE+E10.5PSC	-21.6000	3.6346	-5.94	<.0001	0.05	-28.7237	-14.4763
Ao EC Only	HE+E11.5PSC	-9.5000	3.4799	-2.73	0.0063	0.05	-16.3204	-2.6796
Ao EC Only	IVCE Only	0	3.6346	0.00	1.0000	0.05	-7.1237	7.1237
Ao EC Only	IVCE+E10.5PSC	-3.8000	3.6346	-1.05	0.2958	0.05	-10.9237	3.3237
Ao EC Only	IVCE+E11.5PSC	-4.2000	3.6346	-1.16	0.2479	0.05	-11.3237	2.9237
Ao EC Only	LE Only	0	3.6346	0.00	1.0000	0.05	-7.1237	7.1237
Ao EC Only	LE+E11.5PSC	-26.2500	3.8551	-6.81	<.0001	0.05	-33.8058	-18.6942
AoE+E10.5PSC	AoE+E11.5PSC	-27.4000	3.6346	-7.54	<.0001	0.05	-34.5237	-20.2763
AoE+E10.5PSC	E10.5EC Only	31.8000	3.6346	8.75	<.0001	0.05	24.6763	38.9237
AoE+E10.5PSC	E10.5EC+E10.5PSC	-17.6000	3.6346	-4.84	<.0001	0.05	-24.7237	-10.4763
AoE+E10.5PSC	E11.5AGM	-33.2000	3.6346	-9.13	<.0001	0.05	-40.3237	-26.0763
AoE+E10.5PSC	E11.5EC Only	31.8000	3.6346	8.75	<.0001	0.05	24.6763	38.9237
AoE+E10.5PSC	E11.5EC+E11.5PSC	17.6000	3.1477	5.59	<.0001	0.05	11.4307	23.7693
AoE+E10.5PSC	E11.5PSC only	31.8000	3.6346	8.75	<.0001	0.05	24.6763	38.9237
AoE+E10.5PSC	E13.5EC Only	31.8000	3.6346	8.75	<.0001	0.05	24.6763	38.9237
AoE+E10.5PSC	E13.5EC+E10.5PSC	8.0000	3.6346	2.20	0.0277	0.05	0.8763	15.1237

The GENMOD Procedure

Differences of Condition Least Squares Means								
Condition	_Condition	Estimate	Standard Error	z Value	Pr > |z|	Alpha	Lower	Upper
AoE+E10.5PSC	E13.5EC+E11.5PSC	18.0000	3.6346	4.95	<.0001	0.05	10.8763	25.1237
AoE+E10.5PSC	HE Only	31.8000	3.4799	9.14	<.0001	0.05	24.9796	38.6204
AoE+E10.5PSC	HE+E10.5PSC	10.2000	3.6346	2.81	0.0050	0.05	3.0763	17.3237
AoE+E10.5PSC	HE+E11.5PSC	22.3000	3.4799	6.41	<.0001	0.05	15.4796	29.1204
AoE+E10.5PSC	IVCE Only	31.8000	3.6346	8.75	<.0001	0.05	24.6763	38.9237
AoE+E10.5PSC	IVCE+E10.5PSC	28.0000	3.6346	7.70	<.0001	0.05	20.8763	35.1237
AoE+E10.5PSC	IVCE+E11.5PSC	27.6000	3.6346	7.59	<.0001	0.05	20.4763	34.7237
AoE+E10.5PSC	LE Only	31.8000	3.6346	8.75	<.0001	0.05	24.6763	38.9237
AoE+E10.5PSC	LE+E11.5PSC	5.5500	3.8551	1.44	0.1500	0.05	-2.0058	13.1058
AoE+E11.5PSC	E10.5EC Only	59.2000	3.6346	16.29	<.0001	0.05	52.0763	66.3237
AoE+E11.5PSC	E10.5EC+E10.5PSC	9.8000	3.6346	2.70	0.0070	0.05	2.6763	16.9237
AoE+E11.5PSC	E11.5AGM	-5.8000	3.6346	-1.60	0.1105	0.05	-12.9237	1.3237
AoE+E11.5PSC	E11.5EC Only	59.2000	3.6346	16.29	<.0001	0.05	52.0763	66.3237
AoE+E11.5PSC	E11.5EC+E11.5PSC	45.0000	3.1477	14.30	<.0001	0.05	38.8307	51.1693
AoE+E11.5PSC	E11.5PSC only	59.2000	3.6346	16.29	<.0001	0.05	52.0763	66.3237
AoE+E11.5PSC	E13.5EC Only	59.2000	3.6346	16.29	<.0001	0.05	52.0763	66.3237
AoE+E11.5PSC	E13.5EC+E10.5PSC	35.4000	3.6346	9.74	<.0001	0.05	28.2763	42.5237
AoE+E11.5PSC	E13.5EC+E11.5PSC	45.4000	3.6346	12.49	<.0001	0.05	38.2763	52.5237
AoE+E11.5PSC	HE Only	59.2000	3.4799	17.01	<.0001	0.05	52.3796	66.0204
AoE+E11.5PSC	HE+E10.5PSC	37.6000	3.6346	10.35	<.0001	0.05	30.4763	44.7237
AoE+E11.5PSC	HE+E11.5PSC	49.7000	3.4799	14.28	<.0001	0.05	42.8796	56.5204
AoE+E11.5PSC	IVCE Only	59.2000	3.6346	16.29	<.0001	0.05	52.0763	66.3237
AoE+E11.5PSC	IVCE+E10.5PSC	55.4000	3.6346	15.24	<.0001	0.05	48.2763	62.5237
AoE+E11.5PSC	IVCE+E11.5PSC	55.0000	3.6346	15.13	<.0001	0.05	47.8763	62.1237

The GENMOD Procedure

Differences of Condition Least Squares Means								
Condition	_Condition	Estimate	Standard Error	z Value	Pr > |z|	Alpha	Lower	Upper
AoE+E11.5PSC	LE Only	59.2000	3.6346	16.29	<.0001	0.05	52.0763	66.3237
AoE+E11.5PSC	LE+E11.5PSC	32.9500	3.8551	8.55	<.0001	0.05	25.3942	40.5058
E10.5EC Only	E10.5EC+E10.5PSC	-49.4000	3.6346	-13.59	<.0001	0.05	-56.5237	-42.2763
E10.5EC Only	E11.5AGM	-65.0000	3.6346	-17.88	<.0001	0.05	-72.1237	-57.8763
E10.5EC Only	E11.5EC Only	-355E-17	3.6346	-0.00	1.0000	0.05	-7.1237	7.1237
E10.5EC Only	E11.5EC+E11.5PSC	-14.2000	3.1477	-4.51	<.0001	0.05	-20.3693	-8.0307
E10.5EC Only	E11.5PSC only	3.55E-15	3.6346	0.00	1.0000	0.05	-7.1237	7.1237
E10.5EC Only	E13.5EC Only	3.55E-15	3.6346	0.00	1.0000	0.05	-7.1237	7.1237
E10.5EC Only	E13.5EC+E10.5PSC	-23.8000	3.6346	-6.55	<.0001	0.05	-30.9237	-16.6763
E10.5EC Only	E13.5EC+E11.5PSC	-13.8000	3.6346	-3.80	0.0001	0.05	-20.9237	-6.6763
E10.5EC Only	HE Only	3.55E-15	3.4799	0.00	1.0000	0.05	-6.8204	6.8204
E10.5EC Only	HE+E10.5PSC	-21.6000	3.6346	-5.94	<.0001	0.05	-28.7237	-14.4763
E10.5EC Only	HE+E11.5PSC	-9.5000	3.4799	-2.73	0.0063	0.05	-16.3204	-2.6796
E10.5EC Only	IVCE Only	3.55E-15	3.6346	0.00	1.0000	0.05	-7.1237	7.1237
E10.5EC Only	IVCE+E10.5PSC	-3.8000	3.6346	-1.05	0.2958	0.05	-10.9237	3.3237
E10.5EC Only	IVCE+E11.5PSC	-4.2000	3.6346	-1.16	0.2479	0.05	-11.3237	2.9237
E10.5EC Only	LE Only	3.55E-15	3.6346	0.00	1.0000	0.05	-7.1237	7.1237
E10.5EC Only	LE+E11.5PSC	-26.2500	3.8551	-6.81	<.0001	0.05	-33.8058	-18.6942
E10.5EC+E10.5PSC	E11.5AGM	-15.6000	3.6346	-4.29	<.0001	0.05	-22.7237	-8.4763
E10.5EC+E10.5PSC	E11.5EC Only	49.4000	3.6346	13.59	<.0001	0.05	42.2763	56.5237
E10.5EC+E10.5PSC	E11.5EC+E11.5PSC	35.2000	3.1477	11.18	<.0001	0.05	29.0307	41.3693
E10.5EC+E10.5PSC	E11.5PSC only	49.4000	3.6346	13.59	<.0001	0.05	42.2763	56.5237
E10.5EC+E10.5PSC	E13.5EC Only	49.4000	3.6346	13.59	<.0001	0.05	42.2763	56.5237
E10.5EC+E10.5PSC	E13.5EC+E10.5PSC	25.6000	3.6346	7.04	<.0001	0.05	18.4763	32.7237

The GENMOD Procedure

Differences of Condition Least Squares Means								
Condition	_Condition	Estimate	Standard Error	z Value	Pr > |z|	Alpha	Lower	Upper
E10.5EC+E10.5PSC	E13.5EC+E11.5PSC	35.6000	3.6346	9.79	<.0001	0.05	28.4763	42.7237
E10.5EC+E10.5PSC	HE Only	49.4000	3.4799	14.20	<.0001	0.05	42.5796	56.2204
E10.5EC+E10.5PSC	HE+E10.5PSC	27.8000	3.6346	7.65	<.0001	0.05	20.6763	34.9237
E10.5EC+E10.5PSC	HE+E11.5PSC	39.9000	3.4799	11.47	<.0001	0.05	33.0796	46.7204
E10.5EC+E10.5PSC	IVCE Only	49.4000	3.6346	13.59	<.0001	0.05	42.2763	56.5237
E10.5EC+E10.5PSC	IVCE+E10.5PSC	45.6000	3.6346	12.55	<.0001	0.05	38.4763	52.7237
E10.5EC+E10.5PSC	IVCE+E11.5PSC	45.2000	3.6346	12.44	<.0001	0.05	38.0763	52.3237
E10.5EC+E10.5PSC	LE Only	49.4000	3.6346	13.59	<.0001	0.05	42.2763	56.5237
E10.5EC+E10.5PSC	LE+E11.5PSC	23.1500	3.8551	6.01	<.0001	0.05	15.5942	30.7058
E11.5AGM	E11.5EC Only	65.0000	3.6346	17.88	<.0001	0.05	57.8763	72.1237
E11.5AGM	E11.5EC+E11.5PSC	50.8000	3.1477	16.14	<.0001	0.05	44.6307	56.9693
E11.5AGM	E11.5PSC only	65.0000	3.6346	17.88	<.0001	0.05	57.8763	72.1237
E11.5AGM	E13.5EC Only	65.0000	3.6346	17.88	<.0001	0.05	57.8763	72.1237
E11.5AGM	E13.5EC+E10.5PSC	41.2000	3.6346	11.34	<.0001	0.05	34.0763	48.3237
E11.5AGM	E13.5EC+E11.5PSC	51.2000	3.6346	14.09	<.0001	0.05	44.0763	58.3237
E11.5AGM	HE Only	65.0000	3.4799	18.68	<.0001	0.05	58.1796	71.8204
E11.5AGM	HE+E10.5PSC	43.4000	3.6346	11.94	<.0001	0.05	36.2763	50.5237
E11.5AGM	HE+E11.5PSC	55.5000	3.4799	15.95	<.0001	0.05	48.6796	62.3204
E11.5AGM	IVCE Only	65.0000	3.6346	17.88	<.0001	0.05	57.8763	72.1237
E11.5AGM	IVCE+E10.5PSC	61.2000	3.6346	16.84	<.0001	0.05	54.0763	68.3237
E11.5AGM	IVCE+E11.5PSC	60.8000	3.6346	16.73	<.0001	0.05	53.6763	67.9237
E11.5AGM	LE Only	65.0000	3.6346	17.88	<.0001	0.05	57.8763	72.1237
E11.5AGM	LE+E11.5PSC	38.7500	3.8551	10.05	<.0001	0.05	31.1942	46.3058
E11.5EC Only	E11.5EC+E11.5PSC	-14.2000	3.1477	-4.51	<.0001	0.05	-20.3693	-8.0307

The GENMOD Procedure

Differences of Condition Least Squares Means								
Condition	_Condition	Estimate	Standard Error	z Value	Pr > |z|	Alpha	Lower	Upper
E11.5EC Only	E11.5PSC only	7.11E-15	3.6346	0.00	1.0000	0.05	-7.1237	7.1237
E11.5EC Only	E13.5EC Only	7.11E-15	3.6346	0.00	1.0000	0.05	-7.1237	7.1237
E11.5EC Only	E13.5EC+E10.5PSC	-23.8000	3.6346	-6.55	<.0001	0.05	-30.9237	-16.6763
E11.5EC Only	E13.5EC+E11.5PSC	-13.8000	3.6346	-3.80	0.0001	0.05	-20.9237	-6.6763
E11.5EC Only	HE Only	7.11E-15	3.4799	0.00	1.0000	0.05	-6.8204	6.8204
E11.5EC Only	HE+E10.5PSC	-21.6000	3.6346	-5.94	<.0001	0.05	-28.7237	-14.4763
E11.5EC Only	HE+E11.5PSC	-9.5000	3.4799	-2.73	0.0063	0.05	-16.3204	-2.6796
E11.5EC Only	IVCE Only	7.11E-15	3.6346	0.00	1.0000	0.05	-7.1237	7.1237
E11.5EC Only	IVCE+E10.5PSC	-3.8000	3.6346	-1.05	0.2958	0.05	-10.9237	3.3237
E11.5EC Only	IVCE+E11.5PSC	-4.2000	3.6346	-1.16	0.2479	0.05	-11.3237	2.9237
E11.5EC Only	LE Only	7.11E-15	3.6346	0.00	1.0000	0.05	-7.1237	7.1237
E11.5EC Only	LE+E11.5PSC	-26.2500	3.8551	-6.81	<.0001	0.05	-33.8058	-18.6942
E11.5EC+E11.5PSC	E11.5PSC only	14.2000	3.1477	4.51	<.0001	0.05	8.0307	20.3693
E11.5EC+E11.5PSC	E13.5EC Only	14.2000	3.1477	4.51	<.0001	0.05	8.0307	20.3693
E11.5EC+E11.5PSC	E13.5EC+E10.5PSC	-9.6000	3.1477	-3.05	0.0023	0.05	-15.7693	-3.4307
E11.5EC+E11.5PSC	E13.5EC+E11.5PSC	0.4000	3.1477	0.13	0.8989	0.05	-5.7693	6.5693
E11.5EC+E11.5PSC	HE Only	14.2000	2.9676	4.78	<.0001	0.05	8.3835	20.0165
E11.5EC+E11.5PSC	HE+E10.5PSC	-7.4000	3.1477	-2.35	0.0187	0.05	-13.5693	-1.2307
E11.5EC+E11.5PSC	HE+E11.5PSC	4.7000	2.9676	1.58	0.1132	0.05	-1.1165	10.5165
E11.5EC+E11.5PSC	IVCE Only	14.2000	3.1477	4.51	<.0001	0.05	8.0307	20.3693
E11.5EC+E11.5PSC	IVCE+E10.5PSC	10.4000	3.1477	3.30	0.0010	0.05	4.2307	16.5693
E11.5EC+E11.5PSC	IVCE+E11.5PSC	10.0000	3.1477	3.18	0.0015	0.05	3.8307	16.1693
E11.5EC+E11.5PSC	LE Only	14.2000	3.1477	4.51	<.0001	0.05	8.0307	20.3693
E11.5EC+E11.5PSC	LE+E11.5PSC	-12.0500	3.3999	-3.54	0.0004	0.05	-18.7136	-5.3864

The GENMOD Procedure

Differences of Condition Least Squares Means								
Condition	_Condition	Estimate	Standard Error	z Value	Pr > |z|	Alpha	Lower	Upper
E11.5PSC only	E13.5EC Only	0	3.6346	0.00	1.0000	0.05	-7.1237	7.1237
E11.5PSC only	E13.5EC+E10.5PSC	-23.8000	3.6346	-6.55	<.0001	0.05	-30.9237	-16.6763
E11.5PSC only	E13.5EC+E11.5PSC	-13.8000	3.6346	-3.80	0.0001	0.05	-20.9237	-6.6763
E11.5PSC only	HE Only	0	3.4799	0.00	1.0000	0.05	-6.8204	6.8204
E11.5PSC only	HE+E10.5PSC	-21.6000	3.6346	-5.94	<.0001	0.05	-28.7237	-14.4763
E11.5PSC only	HE+E11.5PSC	-9.5000	3.4799	-2.73	0.0063	0.05	-16.3204	-2.6796
E11.5PSC only	IVCE Only	0	3.6346	0.00	1.0000	0.05	-7.1237	7.1237
E11.5PSC only	IVCE+E10.5PSC	-3.8000	3.6346	-1.05	0.2958	0.05	-10.9237	3.3237
E11.5PSC only	IVCE+E11.5PSC	-4.2000	3.6346	-1.16	0.2479	0.05	-11.3237	2.9237
E11.5PSC only	LE Only	0	3.6346	0.00	1.0000	0.05	-7.1237	7.1237
E11.5PSC only	LE+E11.5PSC	-26.2500	3.8551	-6.81	<.0001	0.05	-33.8058	-18.6942
E13.5EC Only	E13.5EC+E10.5PSC	-23.8000	3.6346	-6.55	<.0001	0.05	-30.9237	-16.6763
E13.5EC Only	E13.5EC+E11.5PSC	-13.8000	3.6346	-3.80	0.0001	0.05	-20.9237	-6.6763
E13.5EC Only	HE Only	0	3.4799	0.00	1.0000	0.05	-6.8204	6.8204
E13.5EC Only	HE+E10.5PSC	-21.6000	3.6346	-5.94	<.0001	0.05	-28.7237	-14.4763
E13.5EC Only	HE+E11.5PSC	-9.5000	3.4799	-2.73	0.0063	0.05	-16.3204	-2.6796
E13.5EC Only	IVCE Only	0	3.6346	0.00	1.0000	0.05	-7.1237	7.1237
E13.5EC Only	IVCE+E10.5PSC	-3.8000	3.6346	-1.05	0.2958	0.05	-10.9237	3.3237
E13.5EC Only	IVCE+E11.5PSC	-4.2000	3.6346	-1.16	0.2479	0.05	-11.3237	2.9237
E13.5EC Only	LE Only	0	3.6346	0.00	1.0000	0.05	-7.1237	7.1237
E13.5EC Only	LE+E11.5PSC	-26.2500	3.8551	-6.81	<.0001	0.05	-33.8058	-18.6942
E13.5EC+E10.5PSC	E13.5EC+E11.5PSC	10.0000	3.6346	2.75	0.0059	0.05	2.8763	17.1237
E13.5EC+E10.5PSC	HE Only	23.8000	3.4799	6.84	<.0001	0.05	16.9796	30.6204
E13.5EC+E10.5PSC	HE+E10.5PSC	2.2000	3.6346	0.61	0.5450	0.05	-4.9237	9.3237

The GENMOD Procedure

Differences of Condition Least Squares Means								
Condition	_Condition	Estimate	Standard Error	z Value	Pr > |z|	Alpha	Lower	Upper
E13.5EC+E10.5PSC	HE+E11.5PSC	14.3000	3.4799	4.11	<.0001	0.05	7.4796	21.1204
E13.5EC+E10.5PSC	IVCE Only	23.8000	3.6346	6.55	<.0001	0.05	16.6763	30.9237
E13.5EC+E10.5PSC	IVCE+E10.5PSC	20.0000	3.6346	5.50	<.0001	0.05	12.8763	27.1237
E13.5EC+E10.5PSC	IVCE+E11.5PSC	19.6000	3.6346	5.39	<.0001	0.05	12.4763	26.7237
E13.5EC+E10.5PSC	LE Only	23.8000	3.6346	6.55	<.0001	0.05	16.6763	30.9237
E13.5EC+E10.5PSC	LE+E11.5PSC	-2.4500	3.8551	-0.64	0.5251	0.05	-10.0058	5.1058
E13.5EC+E11.5PSC	HE Only	13.8000	3.4799	3.97	<.0001	0.05	6.9796	20.6204
E13.5EC+E11.5PSC	HE+E10.5PSC	-7.8000	3.6346	-2.15	0.0319	0.05	-14.9237	-0.6763
E13.5EC+E11.5PSC	HE+E11.5PSC	4.3000	3.4799	1.24	0.2166	0.05	-2.5204	11.1204
E13.5EC+E11.5PSC	IVCE Only	13.8000	3.6346	3.80	0.0001	0.05	6.6763	20.9237
E13.5EC+E11.5PSC	IVCE+E10.5PSC	10.0000	3.6346	2.75	0.0059	0.05	2.8763	17.1237
E13.5EC+E11.5PSC	IVCE+E11.5PSC	9.6000	3.6346	2.64	0.0083	0.05	2.4763	16.7237
E13.5EC+E11.5PSC	LE Only	13.8000	3.6346	3.80	0.0001	0.05	6.6763	20.9237
E13.5EC+E11.5PSC	LE+E11.5PSC	-12.4500	3.8551	-3.23	0.0012	0.05	-20.0058	-4.8942
HE Only	HE+E10.5PSC	-21.6000	3.4799	-6.21	<.0001	0.05	-28.4204	-14.7796
HE Only	HE+E11.5PSC	-9.5000	3.3179	-2.86	0.0042	0.05	-16.0030	-2.9970
HE Only	IVCE Only	0	3.4799	0.00	1.0000	0.05	-6.8204	6.8204
HE Only	IVCE+E10.5PSC	-3.8000	3.4799	-1.09	0.2748	0.05	-10.6204	3.0204
HE Only	IVCE+E11.5PSC	-4.2000	3.4799	-1.21	0.2275	0.05	-11.0204	2.6204
HE Only	LE Only	0	3.4799	0.00	1.0000	0.05	-6.8204	6.8204
HE Only	LE+E11.5PSC	-26.2500	3.7095	-7.08	<.0001	0.05	-33.5206	-18.9794
HE+E10.5PSC	HE+E11.5PSC	12.1000	3.4799	3.48	0.0005	0.05	5.2796	18.9204
HE+E10.5PSC	IVCE Only	21.6000	3.6346	5.94	<.0001	0.05	14.4763	28.7237
HE+E10.5PSC	IVCE+E10.5PSC	17.8000	3.6346	4.90	<.0001	0.05	10.6763	24.9237

The GENMOD Procedure

Differences of Condition Least Squares Means								
Condition	_Condition	Estimate	Standard Error	z Value	Pr > |z|	Alpha	Lower	Upper
HE+E10.5PSC	IVCE+E11.5PSC	17.4000	3.6346	4.79	<.0001	0.05	10.2763	24.5237
HE+E10.5PSC	LE Only	21.6000	3.6346	5.94	<.0001	0.05	14.4763	28.7237
HE+E10.5PSC	LE+E11.5PSC	-4.6500	3.8551	-1.21	0.2277	0.05	-12.2058	2.9058
HE+E11.5PSC	IVCE Only	9.5000	3.4799	2.73	0.0063	0.05	2.6796	16.3204
HE+E11.5PSC	IVCE+E10.5PSC	5.7000	3.4799	1.64	0.1014	0.05	-1.1204	12.5204
HE+E11.5PSC	IVCE+E11.5PSC	5.3000	3.4799	1.52	0.1277	0.05	-1.5204	12.1204
HE+E11.5PSC	LE Only	9.5000	3.4799	2.73	0.0063	0.05	2.6796	16.3204
HE+E11.5PSC	LE+E11.5PSC	-16.7500	3.7095	-4.52	<.0001	0.05	-24.0206	-9.4794
IVCE Only	IVCE+E10.5PSC	-3.8000	3.6346	-1.05	0.2958	0.05	-10.9237	3.3237
IVCE Only	IVCE+E11.5PSC	-4.2000	3.6346	-1.16	0.2479	0.05	-11.3237	2.9237
IVCE Only	LE Only	0	3.6346	0.00	1.0000	0.05	-7.1237	7.1237
IVCE Only	LE+E11.5PSC	-26.2500	3.8551	-6.81	<.0001	0.05	-33.8058	-18.6942
IVCE+E10.5PSC	IVCE+E11.5PSC	-0.4000	3.6346	-0.11	0.9124	0.05	-7.5237	6.7237
IVCE+E10.5PSC	LE Only	3.8000	3.6346	1.05	0.2958	0.05	-3.3237	10.9237
IVCE+E10.5PSC	LE+E11.5PSC	-22.4500	3.8551	-5.82	<.0001	0.05	-30.0058	-14.8942
IVCE+E11.5PSC	LE Only	4.2000	3.6346	1.16	0.2479	0.05	-2.9237	11.3237
IVCE+E11.5PSC	LE+E11.5PSC	-22.0500	3.8551	-5.72	<.0001	0.05	-29.6058	-14.4942
LE Only	LE+E11.5PSC	-26.2500	3.8551	-6.81	<.0001	0.05	-33.8058	-18.6942

The GENMOD Procedure

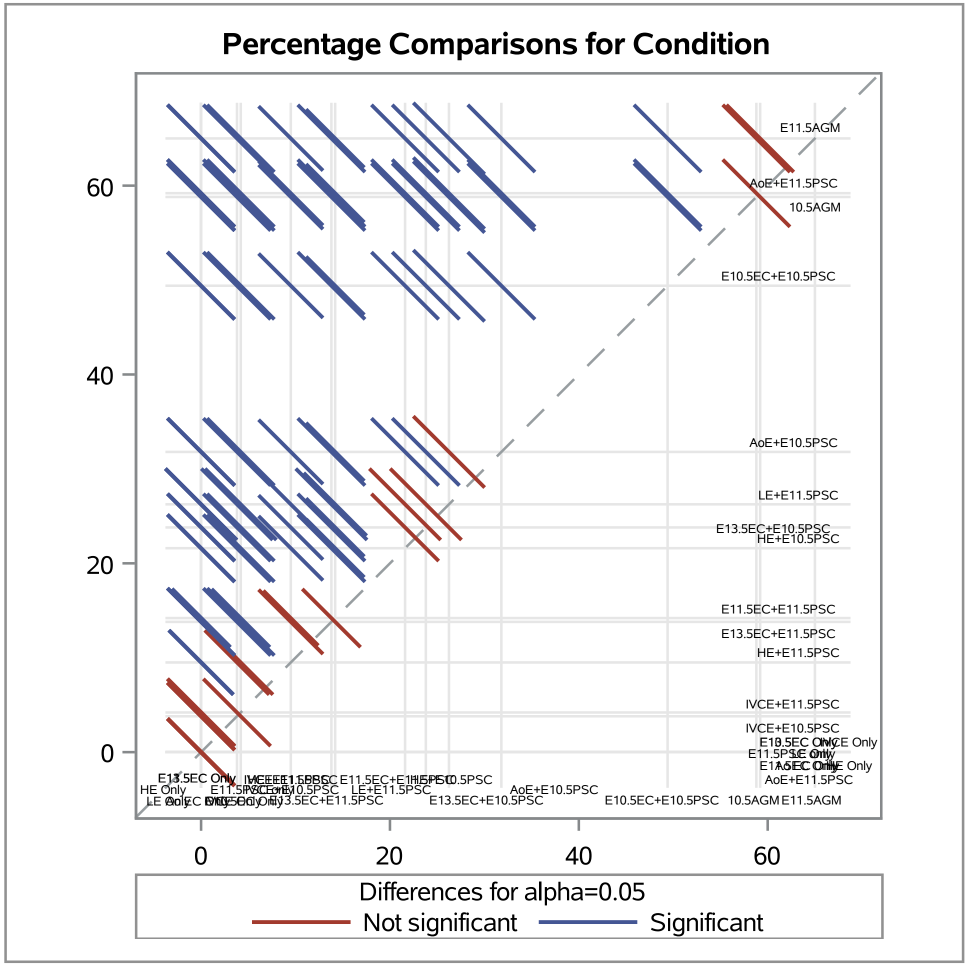

The GENMOD Procedure

Model Information	
**Data Set**	WORK.FIG4AV
**Distribution**	Normal
**Link Function**	Identity
**Dependent Variable**	Percentage

**Number of Observations Read**	24
**Number of Observations Used**	24

Class Level Information		
**Class**	**Levels**	**Values**
**Condition**	3	E11.5EC+E11.5PSC E13.5EC+E11.5PSC HE+E11.5PSC

Parameter Information		
**Parameter**	**Effect**	**Condition**
**Prm1**	Intercept	
**Prm2**	Condition	E11.5EC+E11.5PSC
**Prm3**	Condition	E13.5EC+E11.5PSC
**Prm4**	Condition	HE+E11.5PSC

Criteria For Assessing Goodness Of Fit			
**Criterion**	**DF**	**Value**	**Value/DF**
**Deviance**	21	375.9861	17.9041
**Scaled Deviance**	21	24.0000	1.1429
**Pearson Chi-Square**	21	375.9861	17.9041
**Scaled Pearson X2**	21	24.0000	1.1429
**Log Likelihood**		-67.0725	

The GENMOD Procedure

Criteria For Assessing Goodness Of Fit			
Criterion	DF	Value	Value/DF
**Full Log Likelihood**		-67.0725	
**AIC (smaller is better)**		142.1450	
**AICC (smaller is better)**		144.2503	
**BIC (smaller is better)**		146.8572	

Algorithm converged.

Analysis Of Maximum Likelihood Parameter Estimates								
Parameter		DF	Estimate	Standard Error	Wald 95% Confidence Limits		Wald Chi-Square	Pr > ChiSq
**Intercept**		1	6.0833	1.6159	2.9163	9.2504	14.17	0.0002
**Condition**	E11.5EC+E11.5PSC	1	14.8056	2.0861	10.7169	18.8942	50.37	<.0001
**Condition**	E13.5EC+E11.5PSC	1	1.8056	2.0861	-2.2831	5.8942	0.75	0.3867
**Condition**	HE+E11.5PSC	0	0.0000	0.0000	0.0000	0.0000	.	.
**Scale**		1	3.9580	0.5713	2.9828	5.2522		

Note: The scale parameter was estimated by maximum likelihood.

Condition Least Squares Means							
Condition	Estimate	Standard Error	z Value	Pr > |z|	Alpha	Lower	Upper
E11.5EC+E11.5PSC	20.8889	1.3193	15.83	<.0001	0.05	18.3030	23.4748
E13.5EC+E11.5PSC	7.8889	1.3193	5.98	<.0001	0.05	5.3030	10.4748
HE+E11.5PSC	6.0833	1.6159	3.76	0.0002	0.05	2.9163	9.2504

The GENMOD Procedure

Differences of Condition Least Squares Means								
Condition	_Condition	Estimate	Standard Error	z Value	Pr > |z|	Alpha	Lower	Upper
E11.5EC+E11.5PSC	E13.5EC+E11.5PSC	13.0000	1.8658	6.97	<.0001	0.05	9.3430	16.6570
E11.5EC+E11.5PSC	HE+E11.5PSC	14.8056	2.0861	7.10	<.0001	0.05	10.7169	18.8942
E13.5EC+E11.5PSC	HE+E11.5PSC	1.8056	2.0861	0.87	0.3867	0.05	-2.2831	5.8942

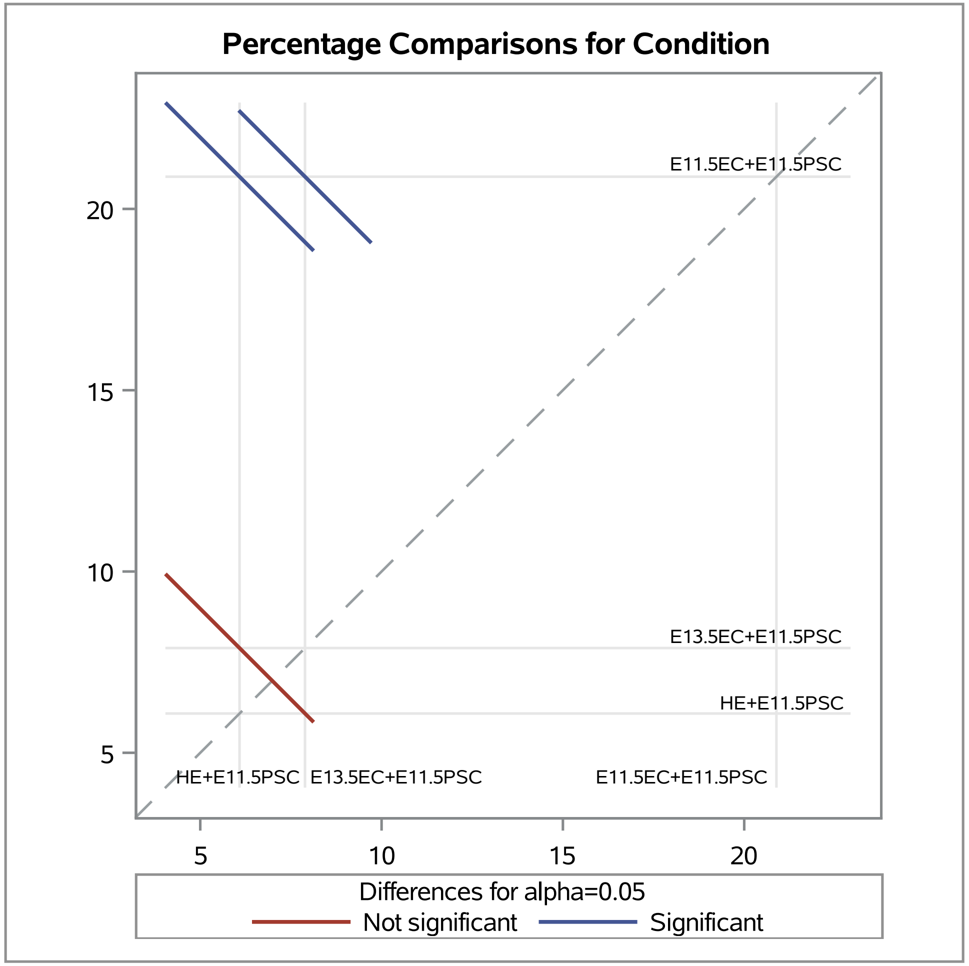

The GENMOD Procedure

Model Information	
Data Set	WORK.FIG4BII
Distribution	Normal
Link Function	Identity
Dependent Variable	Percentage

Number of Observations Read	12
Number of Observations Used	12

Class Level Information		
Class	Levels	Values
Condition	4	GFP+/VE-Cad+/CD45- GFP+VE-Cad+CD45+ GFP+VE-Cad+CD45+DsRedPSC+ GFP+VE-Cad+CD45-DsRedPSC-

Parameter Information		
Parameter	Effect	Condition
Prm1	Intercept	
Prm2	Condition	GFP+/VE-Cad+/CD45-
Prm3	Condition	GFP+VE-Cad+CD45+
Prm4	Condition	GFP+VE-Cad+CD45+DsRedPSC+
Prm5	Condition	GFP+VE-Cad+CD45-DsRedPSC-

Criteria For Assessing Goodness Of Fit			
Criterion	DF	Value	Value/DF
Deviance	8	39.2600	4.9075
Scaled Deviance	8	12.0000	1.5000
Pearson Chi-Square	8	39.2600	4.9075
Scaled Pearson X2	8	12.0000	1.5000

The GENMOD Procedure

Criteria For Assessing Goodness Of Fit			
**Criterion**	**DF**	**Value**	**Value/DF**
**Log Likelihood**		-24.1391	
**Full Log Likelihood**		-24.1391	
**AIC (smaller is better)**		58.2781	
**AICC (smaller is better)**		68.2781	
**BIC (smaller is better)**		60.7027	

Algorithm converged.

Analysis Of Maximum Likelihood Parameter Estimates								
**Parameter**		**DF**	**Estimate**	**Standard Error**	**Wald 95% Confidence Limits**		**Wald Chi-Square**	**Pr > ChiSq**
**Intercept**		1	0.0000	1.0443	-2.0468	2.0468	0.00	1.0000
**Condition**	GFP+/VE-Cad+/CD45-	1	-0.0000	1.4769	-2.8946	2.8946	0.00	1.0000
**Condition**	GFP+VE-Cad+CD45+	1	19.5000	1.4769	16.6054	22.3946	174.34	<.0001
**Condition**	GFP+VE-Cad+CD45+DsRedPSC+	1	30.7000	1.4769	27.8054	33.5946	432.11	<.0001
**Condition**	GFP+VE-Cad+CD45-DsRedPSC-	0	0.0000	0.0000	0.0000	0.0000	.	.
**Scale**		1	1.8088	0.3692	1.2124	2.6986		

Note: The scale parameter was estimated by maximum likelihood.

Condition Least Squares Means							
**Condition**	**Estimate**	**Standard Error**	**z Value**	**Pr > |z|**	**Alpha**	**Lower**	**Upper**
GFP+/VE-Cad+/CD45-	0	1.0443	0.00	1.0000	0.05	-2.0468	2.0468
GFP+VE-Cad+CD45+	19.5000	1.0443	18.67	<.0001	0.05	17.4532	21.5468
GFP+VE-Cad+CD45+DsRedPSC+	30.7000	1.0443	29.40	<.0001	0.05	28.6532	32.7468
GFP+VE-Cad+CD45-DsRedPSC-	5.33E-15	1.0443	0.00	1.0000	0.05	-2.0468	2.0468

The GENMOD Procedure

Differences of Condition Least Squares Means								
Condition	_Condition	Estimate	Standard Error	z Value	Pr > |z|	Alpha	Lower	Upper
GFP+/VE-Cad+/CD45-	GFP+VE-Cad+CD45+	-19.5000	1.4769	-13.20	<.0001	0.05	-22.3946	-16.6054
GFP+/VE-Cad+/CD45-	GFP+VE-Cad+CD45+DsRedPSC+	-30.7000	1.4769	-20.79	<.0001	0.05	-33.5946	-27.8054
GFP+/VE-Cad+/CD45-	GFP+VE-Cad+CD45-DsRedPSC-	-533E-17	1.4769	-0.00	1.0000	0.05	-2.8946	2.8946
GFP+VE-Cad+CD45+	GFP+VE-Cad+CD45+DsRedPSC+	-11.2000	1.4769	-7.58	<.0001	0.05	-14.0946	-8.3054
GFP+VE-Cad+CD45+	GFP+VE-Cad+CD45-DsRedPSC-	19.5000	1.4769	13.20	<.0001	0.05	16.6054	22.3946
GFP+VE-Cad+CD45+DsRedPSC+	GFP+VE-Cad+CD45-DsRedPSC-	30.7000	1.4769	20.79	<.0001	0.05	27.8054	33.5946

The GENMOD Procedure

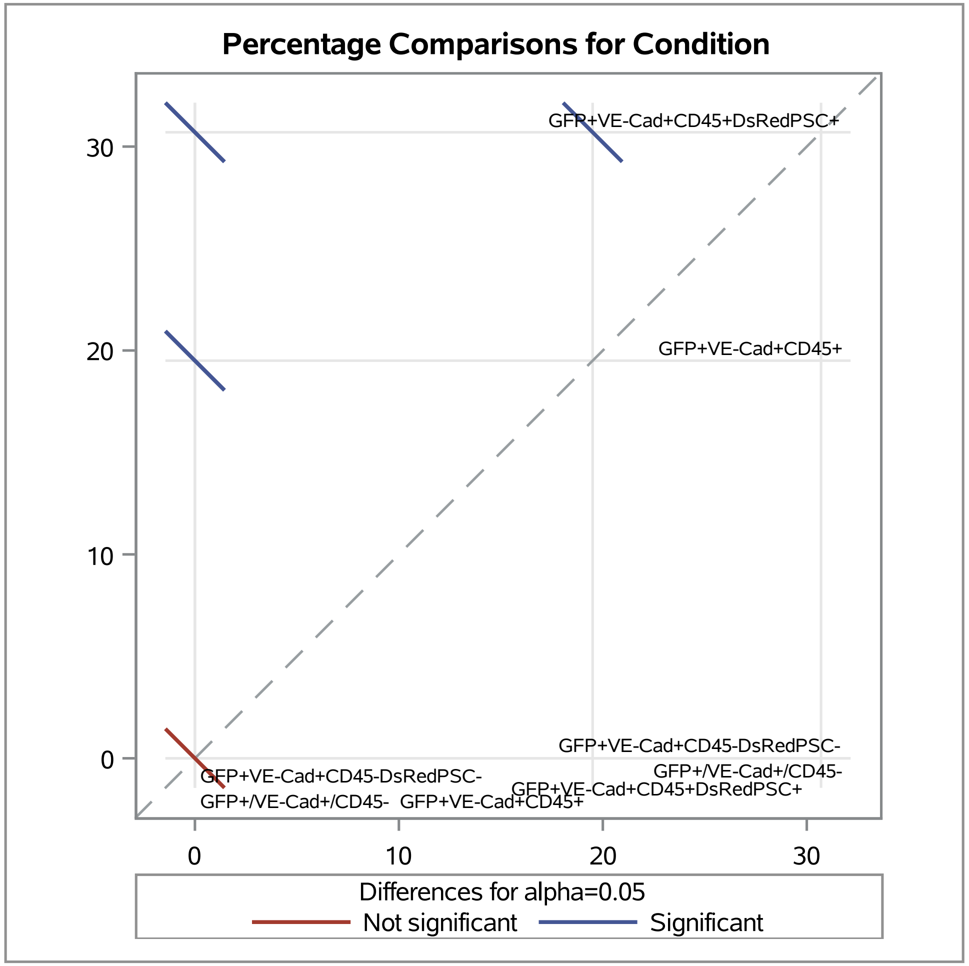

The GLIMMIX Procedure

Model Information	
**Data Set**	WORK.FIG7BII
**Response Variable**	Count
**Response Distribution**	Poisson
**Link Function**	Log
**Variance Function**	Default
**Variance Matrix Blocked By**	Dish_ID
**Estimation Technique**	Maximum Likelihood
**Likelihood Approximation**	Laplace
**Degrees of Freedom Method**	Containment

Class Level Information		
**Class**	**Levels**	**Values**
**Colony_Type**	3	BFU-E CFU-GEMM CFU-GM
**Condition**	8	E11.5EC Only MesP1+E11.5EC (APA5-0ug/ml MesP1+E11.5EC (APA5-20ug/m MesP1+E11.5EC (APA5-40ug/m MesP1+E11.5EC (APB5-0ug/ml MesP1+E11.5EC (APB5-25ug/m MesP1+E11.5EC (APB5-50ug/m MesP1DsRed only
**Dish_ID**	24	1 2 3 4 5 6 7 8 9 10 11 12 13 14 15 16 17 18 19 20 21 22 23 24

**Number of Observations Read**	72
**Number of Observations Used**	72

Dimensions	
**G-side Cov. Parameters**	1
**Columns in X**	36
**Columns in Z per Subject**	1
**Subjects (Blocks in V)**	24
**Max Obs per Subject**	3

The GLIMMIX Procedure

Optimization Information	
Optimization Technique	Dual Quasi-Newton
Parameters in Optimization	25
Lower Boundaries	1
Upper Boundaries	0
Fixed Effects	Not Profiled
Starting From	GLM estimates

Iteration History					
Iteration	Restarts	Evaluations	Objective Function	Change	Max Gradient
0	0	4	503.29730676	.	924.5673
1	0	9	502.75308603	0.54422073	82.73008
2	0	4	502.74343025	0.00965578	18.46292
3	0	4	502.74230848	0.00112176	15.52552
4	0	4	502.73490001	0.00740848	28.01471
5	0	4	502.72753358	0.00736642	8.163007
6	0	3	502.7273431	0.00019049	2.929721
7	0	4	502.7249053	0.00243780	30.16976
8	0	3	502.72358541	0.00131989	2.130787
9	0	2	502.72238203	0.00120338	4.232365
10	0	3	502.72215226	0.00022977	3.324478
11	0	2	502.72178692	0.00036534	5.641296
12	0	4	502.71978737	0.00199955	1.736043
13	0	3	502.71976435	0.00002302	1.584873
14	0	4	502.71953564	0.00022871	3.371406
15	0	3	502.71939467	0.00014097	0.438715

The GLIMMIX Procedure

Iteration History

Iteration	Restarts	Evaluations	Objective Function	Change	Max Gradient
16	0	3	502.71934922	0.00004544	0.146521
17	0	2	502.7193486	0.00000062	0.143395

Convergence criterion (GCONV=1E-8) satisfied.

Fit Statistics

-2 Log Likelihood	502.72
AIC (smaller is better)	552.72
AICC (smaller is better)	580.98
BIC (smaller is better)	582.17
CAIC (smaller is better)	607.17
HQIC (smaller is better)	560.53

Fit Statistics for Conditional Distribution

-2 log L(Count | r. effects)	457.69
Pearson Chi-Square	64.63
Pearson Chi-Square / DF	0.90

Covariance Parameter Estimates

Cov Parm	Subject	Estimate	Standard Error
Intercept	Dish_ID	0.004851	0.002260

The GLIMMIX Procedure

Solutions for Fixed Effects							
Effect	Colony_Type	Condition	Estimate	Standard Error	DF	t Value	Pr > |t|
Intercept			-5.6952	10.0046	16	-0.57	0.5771
Colony_Type	BFU-E		-0.00011	14.1066	32	-0.00	1.0000
Colony_Type	CFU-GEMM		-0.00010	14.1068	32	-0.00	1.0000
Colony_Type	CFU-GM		0	.	.	.	.
Condition		E11.5EC Only	-0.00011	14.1073	32	-0.00	1.0000
Condition		MesP1+E11.5EC (APA5-0ug/ml	12.3683	10.0047	32	1.24	0.2254
Condition		MesP1+E11.5EC (APA5-20ug/m	11.8737	10.0046	32	1.19	0.2440
Condition		MesP1+E11.5EC (APA5-40ug/m	11.3190	10.0045	32	1.13	0.2663
Condition		MesP1+E11.5EC (APB5-0ug/ml	12.3797	10.0047	32	1.24	0.2249
Condition		MesP1+E11.5EC (APB5-25ug/m	12.3473	10.0047	32	1.23	0.2261
Condition		MesP1+E11.5EC (APB5-50ug/m	12.3674	10.0047	32	1.24	0.2254
Condition		MesP1DsRed only	0	.	.	.	.
Colony_Typ*Condition	BFU-E	E11.5EC Only	-0.00004	19.9213	32	-0.00	1.0000
Colony_Typ*Condition	BFU-E	MesP1+E11.5EC (APA5-0ug/ml	-1.2368	14.1067	32	-0.09	0.9307
Colony_Typ*Condition	BFU-E	MesP1+E11.5EC (APA5-20ug/m	-1.3320	14.1067	32	-0.09	0.9254
Colony_Typ*Condition	BFU-E	MesP1+E11.5EC (APA5-40ug/m	-1.6111	14.1068	32	-0.11	0.9098
Colony_Typ*Condition	BFU-E	MesP1+E11.5EC (APB5-0ug/ml	-1.1827	14.1067	32	-0.08	0.9337
Colony_Typ*Condition	BFU-E	MesP1+E11.5EC (APB5-25ug/m	-1.2297	14.1067	32	-0.09	0.9311
Colony_Typ*Condition	BFU-E	MesP1+E11.5EC (APB5-50ug/m	-1.1883	14.1067	32	-0.08	0.9334
Colony_Typ*Condition	BFU-E	MesP1DsRed only	0	.	.	.	.
Colony_Typ*Condition	CFU-GEMM	E11.5EC Only	-0.00004	19.9213	32	-0.00	1.0000
Colony_Typ*Condition	CFU-GEMM	MesP1+E11.5EC (APA5-0ug/ml	-1.5690	14.1068	32	-0.11	0.9121
Colony_Typ*Condition	CFU-GEMM	MesP1+E11.5EC (APA5-20ug/m	-1.6525	14.1069	32	-0.12	0.9075
Colony_Typ*Condition	CFU-GEMM	MesP1+E11.5EC (APA5-40ug/m	-1.9250	14.1071	32	-0.14	0.8923

The GLIMMIX Procedure

Solutions for Fixed Effects							
**Effect**	**Colony_Type**	**Condition**	**Estimate**	**Standard Error**	**DF**	**t Value**	**Pr > |t|**
**Colony_Typ*Condition**	CFU-GEMM	MesP1+E11.5EC (APB5-0ug/ml	-1.5359	14.1068	32	-0.11	0.9140
**Colony_Typ*Condition**	CFU-GEMM	MesP1+E11.5EC (APB5-25ug/m	-1.5452	14.1068	32	-0.11	0.9135
**Colony_Typ*Condition**	CFU-GEMM	MesP1+E11.5EC (APB5-50ug/m	-1.4765	14.1068	32	-0.10	0.9173
**Colony_Typ*Condition**	CFU-GEMM	MesP1DsRed only	0	.	.	.	.
**Colony_Typ*Condition**	CFU-GM	E11.5EC Only	0	.	.	.	.
**Colony_Typ*Condition**	CFU-GM	MesP1+E11.5EC (APA5-0ug/ml	0	.	.	.	.
**Colony_Typ*Condition**	CFU-GM	MesP1+E11.5EC (APA5-20ug/m	0	.	.	.	.
**Colony_Typ*Condition**	CFU-GM	MesP1+E11.5EC (APA5-40ug/m	0	.	.	.	.
**Colony_Typ*Condition**	CFU-GM	MesP1+E11.5EC (APB5-0ug/ml	0	.	.	.	.
**Colony_Typ*Condition**	CFU-GM	MesP1+E11.5EC (APB5-25ug/m	0	.	.	.	.
**Colony_Typ*Condition**	CFU-GM	MesP1+E11.5EC (APB5-50ug/m	0	.	.	.	.
**Colony_Typ*Condition**	CFU-GM	MesP1DsRed only	0	.	.	.	.

Type III Tests of Fixed Effects				
**Effect**	**Num DF**	**Den DF**	**F Value**	**Pr > F**
**Colony_Type**	2	32	0.13	0.8760
**Condition**	7	32	73.21	<.0001
**Colony_Typ*Condition**	14	32	2.77	0.0084

The GLIMMIX Procedure

Condition Least Squares Means												
Condition	Estimate	Standard Error	DF	t Value	Pr > |t|	Alpha	Lower	Upper	Mean	Standard Error Mean	Lower Mean	Upper Mean
E11.5EC Only	-5.6954	5.7427	32	-0.99	0.3288	0.05	-17.3930	6.0021	0.003361	0.01930	2.795E-8	404.29
MesP1+E11.5EC (APA5-0ug/ml	5.7378	0.04528	32	126.73	<.0001	0.05	5.6455	5.8300	310.37	14.0525	283.02	340.35
MesP1+E11.5EC (APA5-20ug/m	5.1835	0.04884	32	106.13	<.0001	0.05	5.0841	5.2830	178.31	8.7093	161.43	196.97
MesP1+E11.5EC (APA5-40ug/m	4.4450	0.05777	32	76.94	<.0001	0.05	4.3273	4.5626	85.1961	4.9219	75.7380	95.8354
MesP1+E11.5EC (APB5-0ug/ml	5.7783	0.04505	32	128.27	<.0001	0.05	5.6865	5.8700	323.20	14.5596	294.86	354.26
MesP1+E11.5EC (APB5-25ug/m	5.7271	0.04531	32	126.41	<.0001	0.05	5.6348	5.8194	307.07	13.9120	280.00	336.76
MesP1+E11.5EC (APB5-50ug/m	5.7839	0.04498	32	128.60	<.0001	0.05	5.6922	5.8755	325.01	14.6176	296.56	356.19
MesP1DsRed only	-5.6953	5.7540	32	-0.99	0.3297	0.05	-17.4159	6.0253	0.003362	0.01934	2.731E-8	413.76

Differences of Condition Least Squares Means									
Condition	_Condition	Estimate	Standard Error	DF	t Value	Pr > |t|	Alpha	Lower	Upper
E11.5EC Only	MesP1+E11.5EC (APA5-0ug/ml	-11.4332	5.7429	32	-1.99	0.0551	0.05	-23.1311	0.2647
E11.5EC Only	MesP1+E11.5EC (APA5-20ug/m	-10.8790	5.7429	32	-1.89	0.0673	0.05	-22.5769	0.8190
E11.5EC Only	MesP1+E11.5EC (APA5-40ug/m	-10.1404	5.7430	32	-1.77	0.0870	0.05	-21.8385	1.5578
E11.5EC Only	MesP1+E11.5EC (APB5-0ug/ml	-11.4737	5.7429	32	-2.00	0.0543	0.05	-23.1716	0.2242
E11.5EC Only	MesP1+E11.5EC (APB5-25ug/m	-11.4225	5.7429	32	-1.99	0.0553	0.05	-23.1204	0.2754
E11.5EC Only	MesP1+E11.5EC (APB5-50ug/m	-11.4793	5.7429	32	-2.00	0.0542	0.05	-23.1772	0.2186
E11.5EC Only	MesP1DsRed only	-0.00013	8.1294	32	-0.00	1.0000	0.05	-16.5592	16.5590
MesP1+E11.5EC (APA5-0ug/ml	MesP1+E11.5EC (APA5-20ug/m	0.5542	0.06660	32	8.32	<.0001	0.05	0.4186	0.6899
MesP1+E11.5EC (APA5-0ug/ml	MesP1+E11.5EC (APA5-40ug/m	1.2928	0.07339	32	17.61	<.0001	0.05	1.1433	1.4423
MesP1+E11.5EC (APA5-0ug/ml	MesP1+E11.5EC (APB5-0ug/ml	-0.04051	0.06387	32	-0.63	0.5304	0.05	-0.1706	0.08959
MesP1+E11.5EC (APA5-0ug/ml	MesP1+E11.5EC (APB5-25ug/m	0.01067	0.06405	32	0.17	0.8687	0.05	-0.1198	0.1411
MesP1+E11.5EC (APA5-0ug/ml	MesP1+E11.5EC (APB5-50ug/m	-0.04610	0.06382	32	-0.72	0.4753	0.05	-0.1761	0.08389

The GLIMMIX Procedure

Differences of Condition Least Squares Means									
Condition	_Condition	Estimate	Standard Error	DF	t Value	Pr > |t|	Alpha	Lower	Upper
MesP1+E11.5EC (APA5-0ug/ml	MesP1DsRed only	11.4330	5.7542	32	1.99	0.0556	0.05	-0.2879	23.1539
MesP1+E11.5EC (APA5-20ug/m	MesP1+E11.5EC (APA5-40ug/m	0.7386	0.07564	32	9.77	<.0001	0.05	0.5845	0.8926
MesP1+E11.5EC (APA5-20ug/m	MesP1+E11.5EC (APB5-0ug/ml	-0.5947	0.06644	32	-8.95	<.0001	0.05	-0.7301	-0.4594
MesP1+E11.5EC (APA5-20ug/m	MesP1+E11.5EC (APB5-25ug/m	-0.5435	0.06662	32	-8.16	<.0001	0.05	-0.6792	-0.4078
MesP1+E11.5EC (APA5-20ug/m	MesP1+E11.5EC (APB5-50ug/m	-0.6003	0.06640	32	-9.04	<.0001	0.05	-0.7356	-0.4651
MesP1+E11.5EC (APA5-20ug/m	MesP1DsRed only	10.8788	5.7542	32	1.89	0.0678	0.05	-0.8421	22.5997
MesP1+E11.5EC (APA5-40ug/m	MesP1+E11.5EC (APB5-0ug/ml	-1.3333	0.07326	32	-18.20	<.0001	0.05	-1.4825	-1.1841
MesP1+E11.5EC (APA5-40ug/m	MesP1+E11.5EC (APB5-25ug/m	-1.2821	0.07341	32	-17.46	<.0001	0.05	-1.4317	-1.1326
MesP1+E11.5EC (APA5-40ug/m	MesP1+E11.5EC (APB5-50ug/m	-1.3389	0.07321	32	-18.29	<.0001	0.05	-1.4880	-1.1898
MesP1+E11.5EC (APA5-40ug/m	MesP1DsRed only	10.1402	5.7542	32	1.76	0.0876	0.05	-1.5806	21.8611
MesP1+E11.5EC (APB5-0ug/ml	MesP1+E11.5EC (APB5-25ug/m	0.05118	0.06389	32	0.80	0.4290	0.05	-0.07896	0.1813
MesP1+E11.5EC (APB5-0ug/ml	MesP1+E11.5EC (APB5-50ug/m	-0.00559	0.06366	32	-0.09	0.9305	0.05	-0.1353	0.1241
MesP1+E11.5EC (APB5-0ug/ml	MesP1DsRed only	11.4736	5.7542	32	1.99	0.0547	0.05	-0.2474	23.1945
MesP1+E11.5EC (APB5-25ug/m	MesP1+E11.5EC (APB5-50ug/m	-0.05677	0.06384	32	-0.89	0.3805	0.05	-0.1868	0.07326
MesP1+E11.5EC (APB5-25ug/m	MesP1DsRed only	11.4224	5.7542	32	1.99	0.0558	0.05	-0.2985	23.1433
MesP1+E11.5EC (APB5-50ug/m	MesP1DsRed only	11.4791	5.7542	32	1.99	0.0546	0.05	-0.2418	23.2001

Colony_Typ*Condition Least Squares Means													
Colony_Type	Condition	Estimate	Standard Error	DF	t Value	Pr > |t|	Alpha	Lower	Upper	Mean	Standard Error Mean	Lower Mean	Upper Mean
BFU-E	E11.5EC Only	-5.6955	9.9468	32	-0.57	0.5709	0.05	-25.9564	14.5655	0.003361	0.03343	5.34E-12	2116945
BFU-E	MesP1+E11.5EC (APA5-0ug/ml	5.4362	0.05538	32	98.16	<.0001	0.05	5.3233	5.5490	229.56	12.7132	205.07	256.97
BFU-E	MesP1+E11.5EC (APA5-20ug/m	4.8463	0.06504	32	74.51	<.0001	0.05	4.7138	4.9788	127.27	8.2778	111.48	145.30
BFU-E	MesP1+E11.5EC (APA5-40ug/m	4.0125	0.08728	32	45.97	<.0001	0.05	3.8347	4.1903	55.2843	4.8255	46.2794	66.0414

The GLIMMIX Procedure

Colony_Typ*Condition Least Squares Means													
Colony_Type	Condition	Estimate	Standard Error	DF	t Value	Pr > |t|	Alpha	Lower	Upper	Mean	Standard Error Mean	Lower Mean	Upper Mean
BFU-E	MesP1+E11.5EC (APB5-0ug/ml	5.5017	0.05455	32	100.85	<.0001	0.05	5.3906	5.6129	245.12	13.3716	219.34	273.93
BFU-E	MesP1+E11.5EC (APB5-25ug/m	5.4223	0.05557	32	97.58	<.0001	0.05	5.3091	5.5355	226.41	12.5812	202.18	253.54
BFU-E	MesP1+E11.5EC (APB5-50ug/m	5.4838	0.05478	32	100.11	<.0001	0.05	5.3722	5.5953	240.75	13.1873	215.33	269.17
BFU-E	MesP1DsRed only	-5.6953	9.9461	32	-0.57	0.5709	0.05	-25.9549	14.5642	0.003362	0.03344	5.34E-12	2114267
CFU-GEMM	E11.5EC Only	-5.6955	9.9467	32	-0.57	0.5709	0.05	-25.9563	14.5654	0.003361	0.03343	5.34E-12	2116734
CFU-GEMM	MesP1+E11.5EC (APA5-0ug/ml	5.1040	0.06032	32	84.62	<.0001	0.05	4.9812	5.2269	164.68	9.9329	145.64	186.21
CFU-GEMM	MesP1+E11.5EC (APA5-20ug/m	4.5259	0.07223	32	62.66	<.0001	0.05	4.3788	4.6730	92.3780	6.6722	79.7396	107.02
CFU-GEMM	MesP1+E11.5EC (APA5-40ug/m	3.6986	0.09913	32	37.31	<.0001	0.05	3.4967	3.9006	40.3922	4.0042	33.0067	49.4303
CFU-GEMM	MesP1+E11.5EC (APB5-0ug/ml	5.1485	0.05959	32	86.39	<.0001	0.05	5.0271	5.2699	172.18	10.2608	152.50	194.40
CFU-GEMM	MesP1+E11.5EC (APB5-25ug/m	5.1068	0.06028	32	84.72	<.0001	0.05	4.9840	5.2296	165.14	9.9544	146.06	186.71
CFU-GEMM	MesP1+E11.5EC (APB5-50ug/m	5.1956	0.05884	32	88.30	<.0001	0.05	5.0757	5.3155	180.48	10.6195	160.09	203.46
CFU-GEMM	MesP1DsRed only	-5.6953	9.9460	32	-0.57	0.5709	0.05	-25.9548	14.5641	0.003362	0.03344	5.35E-12	2114055
CFU-GM	E11.5EC Only	-5.6953	9.9461	32	-0.57	0.5709	0.05	-25.9548	14.5642	0.003362	0.03344	5.35E-12	2114145
CFU-GM	MesP1+E11.5EC (APA5-0ug/ml	6.6731	0.04515	32	147.80	<.0001	0.05	6.5811	6.7651	790.84	35.7058	721.35	867.02
CFU-GM	MesP1+E11.5EC (APA5-20ug/m	6.1784	0.04805	32	128.60	<.0001	0.05	6.0806	6.2763	482.24	23.1692	437.28	531.82
CFU-GM	MesP1+E11.5EC (APA5-40ug/m	5.6237	0.05314	32	105.83	<.0001	0.05	5.5155	5.7320	276.92	14.7159	248.51	308.58
CFU-GM	MesP1+E11.5EC (APB5-0ug/ml	6.6845	0.04510	32	148.23	<.0001	0.05	6.5927	6.7764	799.92	36.0727	729.72	876.88
CFU-GM	MesP1+E11.5EC (APB5-25ug/m	6.6521	0.04525	32	147.02	<.0001	0.05	6.5599	6.7443	774.42	35.0404	706.23	849.18
CFU-GM	MesP1+E11.5EC (APB5-50ug/m	6.6722	0.04515	32	147.77	<.0001	0.05	6.5802	6.7642	790.14	35.6769	720.71	866.26
CFU-GM	MesP1DsRed only	-5.6952	10.0046	32	-0.57	0.5732	0.05	-26.0739	14.6835	0.003362	0.03364	4.75E-12	2382050

The GLIMMIX Procedure

Differences of Colony_Typ*Condition Least Squares Means											
Colony_Type	Condition	_Colony_Type	_Condition	Estimate	Standard Error	DF	t Value	Pr > |t|	Alpha	Lower	Upper
BFU-E	E11.5EC Only	BFU-E	MesP1+E11.5EC (APA5-0ug/ml	-11.1316	9.9469	32	-1.12	0.2714	0.05	-31.3929	9.1296
BFU-E	E11.5EC Only	BFU-E	MesP1+E11.5EC (APA5-20ug/m	-10.5418	9.9470	32	-1.06	0.2972	0.05	-30.8032	9.7196
BFU-E	E11.5EC Only	BFU-E	MesP1+E11.5EC (APA5-40ug/m	-9.7080	9.9472	32	-0.98	0.3364	0.05	-29.9697	10.5538
BFU-E	E11.5EC Only	BFU-E	MesP1+E11.5EC (APB5-0ug/ml	-11.1972	9.9469	32	-1.13	0.2687	0.05	-31.4585	9.0641
BFU-E	E11.5EC Only	BFU-E	MesP1+E11.5EC (APB5-25ug/m	-11.1178	9.9469	32	-1.12	0.2720	0.05	-31.3791	9.1435
BFU-E	E11.5EC Only	BFU-E	MesP1+E11.5EC (APB5-50ug/m	-11.1792	9.9469	32	-1.12	0.2694	0.05	-31.4405	9.0820
BFU-E	E11.5EC Only	BFU-E	MesP1DsRed only	-0.00014	14.0664	32	-0.00	1.0000	0.05	-28.6525	28.6522
BFU-E	E11.5EC Only	CFU-GEMM	E11.5EC Only	-0.00001	14.0667	32	-0.00	1.0000	0.05	-28.6530	28.6530
BFU-E	E11.5EC Only	CFU-GEMM	MesP1+E11.5EC (APA5-0ug/ml	-10.7995	9.9470	32	-1.09	0.2857	0.05	-31.0608	9.4618
BFU-E	E11.5EC Only	CFU-GEMM	MesP1+E11.5EC (APA5-20ug/m	-10.2214	9.9471	32	-1.03	0.3119	0.05	-30.4829	10.0401
BFU-E	E11.5EC Only	CFU-GEMM	MesP1+E11.5EC (APA5-40ug/m	-9.3941	9.9473	32	-0.94	0.3520	0.05	-29.6561	10.8679
BFU-E	E11.5EC Only	CFU-GEMM	MesP1+E11.5EC (APB5-0ug/ml	-10.8440	9.9470	32	-1.09	0.2838	0.05	-31.1053	9.4173
BFU-E	E11.5EC Only	CFU-GEMM	MesP1+E11.5EC (APB5-25ug/m	-10.8023	9.9470	32	-1.09	0.2856	0.05	-31.0636	9.4591
BFU-E	E11.5EC Only	CFU-GEMM	MesP1+E11.5EC (APB5-50ug/m	-10.8911	9.9470	32	-1.09	0.2817	0.05	-31.1524	9.3702
BFU-E	E11.5EC Only	CFU-GEMM	MesP1DsRed only	-0.00015	14.0664	32	-0.00	1.0000	0.05	-28.6524	28.6521
BFU-E	E11.5EC Only	CFU-GM	E11.5EC Only	-0.00015	14.0663	32	-0.00	1.0000	0.05	-28.6522	28.6519
BFU-E	E11.5EC Only	CFU-GM	MesP1+E11.5EC (APA5-0ug/ml	-12.3686	9.9469	32	-1.24	0.2227	0.05	-32.6297	7.8926
BFU-E	E11.5EC Only	CFU-GM	MesP1+E11.5EC (APA5-20ug/m	-11.8739	9.9469	32	-1.19	0.2414	0.05	-32.1351	8.3873
BFU-E	E11.5EC Only	CFU-GM	MesP1+E11.5EC (APA5-40ug/m	-11.3192	9.9469	32	-1.14	0.2636	0.05	-31.5805	8.9420
BFU-E	E11.5EC Only	CFU-GM	MesP1+E11.5EC (APB5-0ug/ml	-12.3800	9.9469	32	-1.24	0.2223	0.05	-32.6411	7.8812
BFU-E	E11.5EC Only	CFU-GM	MesP1+E11.5EC (APB5-25ug/m	-12.3476	9.9469	32	-1.24	0.2235	0.05	-32.6088	7.9136
BFU-E	E11.5EC Only	CFU-GM	MesP1+E11.5EC (APB5-50ug/m	-12.3677	9.9469	32	-1.24	0.2228	0.05	-32.6289	7.8935
BFU-E	E11.5EC Only	CFU-GM	MesP1DsRed only	-0.00025	14.1078	32	-0.00	1.0000	0.05	-28.7369	28.7364
BFU-E	MesP1+E11.5EC (APA5-0ug/ml	BFU-E	MesP1+E11.5EC (APA5-20ug/m	0.5899	0.08542	32	6.91	<.0001	0.05	0.4159	0.7639

The GLIMMIX Procedure

Differences of Colony_Typ*Condition Least Squares Means											
Colony_Type	Condition	_Colony_Type	_Condition	Estimate	Standard Error	DF	t Value	Pr > |t|	Alpha	Lower	Upper
BFU-E	MesP1+E11.5EC (APA5-0ug/ml	BFU-E	MesP1+E11.5EC (APA5-40ug/m	1.4237	0.1034	32	13.77	<.0001	0.05	1.2131	1.6342
BFU-E	MesP1+E11.5EC (APA5-0ug/ml	BFU-E	MesP1+E11.5EC (APB5-0ug/ml	-0.06558	0.07774	32	-0.84	0.4051	0.05	-0.2239	0.09276
BFU-E	MesP1+E11.5EC (APA5-0ug/ml	BFU-E	MesP1+E11.5EC (APB5-25ug/m	0.01382	0.07845	32	0.18	0.8612	0.05	-0.1460	0.1736
BFU-E	MesP1+E11.5EC (APA5-0ug/ml	BFU-E	MesP1+E11.5EC (APB5-50ug/m	-0.04760	0.07789	32	-0.61	0.5455	0.05	-0.2063	0.1111
BFU-E	MesP1+E11.5EC (APA5-0ug/ml	BFU-E	MesP1DsRed only	11.1315	9.9462	32	1.12	0.2714	0.05	-9.1284	31.3913
BFU-E	MesP1+E11.5EC (APA5-0ug/ml	CFU-GEMM	E11.5EC Only	11.1316	9.9469	32	1.12	0.2714	0.05	-9.1295	31.3928
BFU-E	MesP1+E11.5EC (APA5-0ug/ml	CFU-GEMM	MesP1+E11.5EC (APA5-0ug/ml	0.3321	0.05890	32	5.64	<.0001	0.05	0.2122	0.4521
BFU-E	MesP1+E11.5EC (APA5-0ug/ml	CFU-GEMM	MesP1+E11.5EC (APA5-20ug/m	0.9103	0.09101	32	10.00	<.0001	0.05	0.7249	1.0957
BFU-E	MesP1+E11.5EC (APA5-0ug/ml	CFU-GEMM	MesP1+E11.5EC (APA5-40ug/m	1.7375	0.1135	32	15.30	<.0001	0.05	1.5062	1.9688
BFU-E	MesP1+E11.5EC (APA5-0ug/ml	CFU-GEMM	MesP1+E11.5EC (APB5-0ug/ml	0.2876	0.08135	32	3.54	0.0013	0.05	0.1219	0.4533
BFU-E	MesP1+E11.5EC (APA5-0ug/ml	CFU-GEMM	MesP1+E11.5EC (APB5-25ug/m	0.3294	0.08186	32	4.02	0.0003	0.05	0.1626	0.4961
BFU-E	MesP1+E11.5EC (APA5-0ug/ml	CFU-GEMM	MesP1+E11.5EC (APB5-50ug/m	0.2406	0.08080	32	2.98	0.0055	0.05	0.07596	0.4052
BFU-E	MesP1+E11.5EC (APA5-0ug/ml	CFU-GEMM	MesP1DsRed only	11.1315	9.9462	32	1.12	0.2714	0.05	-9.1283	31.3912
BFU-E	MesP1+E11.5EC (APA5-0ug/ml	CFU-GM	E11.5EC Only	11.1315	9.9462	32	1.12	0.2714	0.05	-9.1283	31.3913
BFU-E	MesP1+E11.5EC (APA5-0ug/ml	CFU-GM	MesP1+E11.5EC (APA5-0ug/ml	-1.2369	0.04324	32	-28.60	<.0001	0.05	-1.3250	-1.1489
BFU-E	MesP1+E11.5EC (APA5-0ug/ml	CFU-GM	MesP1+E11.5EC (APA5-20ug/m	-0.7423	0.07332	32	-10.12	<.0001	0.05	-0.8916	-0.5929
BFU-E	MesP1+E11.5EC (APA5-0ug/ml	CFU-GM	MesP1+E11.5EC (APA5-40ug/m	-0.1876	0.07675	32	-2.44	0.0202	0.05	-0.3439	-0.03126
BFU-E	MesP1+E11.5EC (APA5-0ug/ml	CFU-GM	MesP1+E11.5EC (APB5-0ug/ml	-1.2484	0.07142	32	-17.48	<.0001	0.05	-1.3938	-1.1029
BFU-E	MesP1+E11.5EC (APA5-0ug/ml	CFU-GM	MesP1+E11.5EC (APB5-25ug/m	-1.2160	0.07151	32	-17.00	<.0001	0.05	-1.3616	-1.0703
BFU-E	MesP1+E11.5EC (APA5-0ug/ml	CFU-GM	MesP1+E11.5EC (APB5-50ug/m	-1.2361	0.07145	32	-17.30	<.0001	0.05	-1.3816	-1.0905
BFU-E	MesP1+E11.5EC (APA5-0ug/ml	CFU-GM	MesP1DsRed only	11.1314	10.0047	32	1.11	0.2742	0.05	-9.2476	31.5104
BFU-E	MesP1+E11.5EC (APA5-20ug/m	BFU-E	MesP1+E11.5EC (APA5-40ug/m	0.8338	0.1088	32	7.66	<.0001	0.05	0.6121	1.0555
BFU-E	MesP1+E11.5EC (APA5-20ug/m	BFU-E	MesP1+E11.5EC (APB5-0ug/ml	-0.6554	0.08489	32	-7.72	<.0001	0.05	-0.8284	-0.4825
BFU-E	MesP1+E11.5EC (APA5-20ug/m	BFU-E	MesP1+E11.5EC (APB5-25ug/m	-0.5760	0.08555	32	-6.73	<.0001	0.05	-0.7503	-0.4018

The GLIMMIX Procedure

Differences of Colony_Typ*Condition Least Squares Means											
Colony_Type	Condition	_Colony_Type	_Condition	Estimate	Standard Error	DF	t Value	Pr > |t|	Alpha	Lower	Upper
BFU-E	MesP1+E11.5EC (APA5-20ug/m	BFU-E	MesP1+E11.5EC (APB5-50ug/m	-0.6375	0.08503	32	-7.50	<.0001	0.05	-0.8107	-0.4643
BFU-E	MesP1+E11.5EC (APA5-20ug/m	BFU-E	MesP1DsRed only	10.5416	9.9463	32	1.06	0.2971	0.05	-9.7183	30.8016
BFU-E	MesP1+E11.5EC (APA5-20ug/m	CFU-GEMM	E11.5EC Only	10.5418	9.9470	32	1.06	0.2972	0.05	-9.7195	30.8030
BFU-E	MesP1+E11.5EC (APA5-20ug/m	CFU-GEMM	MesP1+E11.5EC (APA5-0ug/ml	-0.2577	0.08870	32	-2.91	0.0066	0.05	-0.4384	-0.07703
BFU-E	MesP1+E11.5EC (APA5-20ug/m	CFU-GEMM	MesP1+E11.5EC (APA5-20ug/m	0.3204	0.07880	32	4.07	0.0003	0.05	0.1599	0.4809
BFU-E	MesP1+E11.5EC (APA5-20ug/m	CFU-GEMM	MesP1+E11.5EC (APA5-40ug/m	1.1477	0.1186	32	9.68	<.0001	0.05	0.9062	1.3891
BFU-E	MesP1+E11.5EC (APA5-20ug/m	CFU-GEMM	MesP1+E11.5EC (APB5-0ug/ml	-0.3022	0.08821	32	-3.43	0.0017	0.05	-0.4819	-0.1226
BFU-E	MesP1+E11.5EC (APA5-20ug/m	CFU-GEMM	MesP1+E11.5EC (APB5-25ug/m	-0.2605	0.08868	32	-2.94	0.0061	0.05	-0.4411	-0.07987
BFU-E	MesP1+E11.5EC (APA5-20ug/m	CFU-GEMM	MesP1+E11.5EC (APB5-50ug/m	-0.3493	0.08771	32	-3.98	0.0004	0.05	-0.5280	-0.1706
BFU-E	MesP1+E11.5EC (APA5-20ug/m	CFU-GEMM	MesP1DsRed only	10.5416	9.9463	32	1.06	0.2971	0.05	-9.7183	30.8015
BFU-E	MesP1+E11.5EC (APA5-20ug/m	CFU-GM	E11.5EC Only	10.5416	9.9463	32	1.06	0.2971	0.05	-9.7183	30.8015
BFU-E	MesP1+E11.5EC (APA5-20ug/m	CFU-GM	MesP1+E11.5EC (APA5-0ug/ml	-1.8268	0.07917	32	-23.07	<.0001	0.05	-1.9881	-1.6655
BFU-E	MesP1+E11.5EC (APA5-20ug/m	CFU-GM	MesP1+E11.5EC (APA5-20ug/m	-1.3321	0.05745	32	-23.19	<.0001	0.05	-1.4492	-1.2151
BFU-E	MesP1+E11.5EC (APA5-20ug/m	CFU-GM	MesP1+E11.5EC (APA5-40ug/m	-0.7774	0.08398	32	-9.26	<.0001	0.05	-0.9485	-0.6064
BFU-E	MesP1+E11.5EC (APA5-20ug/m	CFU-GM	MesP1+E11.5EC (APB5-0ug/ml	-1.8382	0.07915	32	-23.23	<.0001	0.05	-1.9994	-1.6770
BFU-E	MesP1+E11.5EC (APA5-20ug/m	CFU-GM	MesP1+E11.5EC (APB5-25ug/m	-1.8058	0.07923	32	-22.79	<.0001	0.05	-1.9672	-1.6444
BFU-E	MesP1+E11.5EC (APA5-20ug/m	CFU-GM	MesP1+E11.5EC (APB5-50ug/m	-1.8259	0.07918	32	-23.06	<.0001	0.05	-1.9872	-1.6646
BFU-E	MesP1+E11.5EC (APA5-20ug/m	CFU-GM	MesP1DsRed only	10.5415	10.0048	32	1.05	0.2999	0.05	-9.8375	30.9205
BFU-E	MesP1+E11.5EC (APA5-40ug/m	BFU-E	MesP1+E11.5EC (APB5-0ug/ml	-1.4892	0.1029	32	-14.47	<.0001	0.05	-1.6989	-1.2796
BFU-E	MesP1+E11.5EC (APA5-40ug/m	BFU-E	MesP1+E11.5EC (APB5-25ug/m	-1.4098	0.1035	32	-13.63	<.0001	0.05	-1.6206	-1.1991
BFU-E	MesP1+E11.5EC (APA5-40ug/m	BFU-E	MesP1+E11.5EC (APB5-50ug/m	-1.4713	0.1030	32	-14.28	<.0001	0.05	-1.6812	-1.2614
BFU-E	MesP1+E11.5EC (APA5-40ug/m	BFU-E	MesP1DsRed only	9.7078	9.9465	32	0.98	0.3364	0.05	-10.5525	29.9681
BFU-E	MesP1+E11.5EC (APA5-40ug/m	CFU-GEMM	E11.5EC Only	9.7080	9.9471	32	0.98	0.3364	0.05	-10.5537	29.9696
BFU-E	MesP1+E11.5EC (APA5-40ug/m	CFU-GEMM	MesP1+E11.5EC (APA5-0ug/ml	-1.0915	0.1061	32	-10.29	<.0001	0.05	-1.3076	-0.8754

The GLIMMIX Procedure

Differences of Colony_Typ*Condition Least Squares Means											
Colony_Type	Condition	_Colony_Type	_Condition	Estimate	Standard Error	DF	t Value	Pr > |t|	Alpha	Lower	Upper
BFU-E	MesP1+E11.5EC (APA5-40ug/m	CFU-GEMM	MesP1+E11.5EC (APA5-20ug/m	-0.5134	0.1133	32	-4.53	<.0001	0.05	-0.7441	-0.2827
BFU-E	MesP1+E11.5EC (APA5-40ug/m	CFU-GEMM	MesP1+E11.5EC (APA5-40ug/m	0.3139	0.1191	32	2.63	0.0129	0.05	0.07121	0.5565
BFU-E	MesP1+E11.5EC (APA5-40ug/m	CFU-GEMM	MesP1+E11.5EC (APB5-0ug/ml	-1.1360	0.1057	32	-10.75	<.0001	0.05	-1.3513	-0.9208
BFU-E	MesP1+E11.5EC (APA5-40ug/m	CFU-GEMM	MesP1+E11.5EC (APB5-25ug/m	-1.0943	0.1061	32	-10.32	<.0001	0.05	-1.3104	-0.8782
BFU-E	MesP1+E11.5EC (APA5-40ug/m	CFU-GEMM	MesP1+E11.5EC (APB5-50ug/m	-1.1831	0.1053	32	-11.24	<.0001	0.05	-1.3975	-0.9687
BFU-E	MesP1+E11.5EC (APA5-40ug/m	CFU-GEMM	MesP1DsRed only	9.7078	9.9464	32	0.98	0.3364	0.05	-10.5524	29.9680
BFU-E	MesP1+E11.5EC (APA5-40ug/m	CFU-GM	E11.5EC Only	9.7078	9.9465	32	0.98	0.3364	0.05	-10.5525	29.9681
BFU-E	MesP1+E11.5EC (APA5-40ug/m	CFU-GM	MesP1+E11.5EC (APA5-0ug/ml	-2.6606	0.09827	32	-27.08	<.0001	0.05	-2.8608	-2.4604
BFU-E	MesP1+E11.5EC (APA5-40ug/m	CFU-GM	MesP1+E11.5EC (APA5-20ug/m	-2.1660	0.09962	32	-21.74	<.0001	0.05	-2.3689	-1.9630
BFU-E	MesP1+E11.5EC (APA5-40ug/m	CFU-GM	MesP1+E11.5EC (APA5-40ug/m	-1.6113	0.08477	32	-19.01	<.0001	0.05	-1.7839	-1.4386
BFU-E	MesP1+E11.5EC (APA5-40ug/m	CFU-GM	MesP1+E11.5EC (APB5-0ug/ml	-2.6720	0.09824	32	-27.20	<.0001	0.05	-2.8721	-2.4719
BFU-E	MesP1+E11.5EC (APA5-40ug/m	CFU-GM	MesP1+E11.5EC (APB5-25ug/m	-2.6396	0.09831	32	-26.85	<.0001	0.05	-2.8399	-2.4394
BFU-E	MesP1+E11.5EC (APA5-40ug/m	CFU-GM	MesP1+E11.5EC (APB5-50ug/m	-2.6597	0.09827	32	-27.07	<.0001	0.05	-2.8599	-2.4596
BFU-E	MesP1+E11.5EC (APA5-40ug/m	CFU-GM	MesP1DsRed only	9.7077	10.0048	32	0.97	0.3392	0.05	-10.6713	30.0868
BFU-E	MesP1+E11.5EC (APB5-0ug/ml	BFU-E	MesP1+E11.5EC (APB5-25ug/m	0.07941	0.07787	32	1.02	0.3155	0.05	-0.07921	0.2380
BFU-E	MesP1+E11.5EC (APB5-0ug/ml	BFU-E	MesP1+E11.5EC (APB5-50ug/m	0.01798	0.07731	32	0.23	0.8175	0.05	-0.1395	0.1755
BFU-E	MesP1+E11.5EC (APB5-0ug/ml	BFU-E	MesP1DsRed only	11.1971	9.9462	32	1.13	0.2686	0.05	-9.0628	31.4569
BFU-E	MesP1+E11.5EC (APB5-0ug/ml	CFU-GEMM	E11.5EC Only	11.1972	9.9469	32	1.13	0.2687	0.05	-9.0640	31.4584
BFU-E	MesP1+E11.5EC (APB5-0ug/ml	CFU-GEMM	MesP1+E11.5EC (APA5-0ug/ml	0.3977	0.08133	32	4.89	<.0001	0.05	0.2321	0.5634
BFU-E	MesP1+E11.5EC (APB5-0ug/ml	CFU-GEMM	MesP1+E11.5EC (APA5-20ug/m	0.9758	0.09051	32	10.78	<.0001	0.05	0.7915	1.1602
BFU-E	MesP1+E11.5EC (APB5-0ug/ml	CFU-GEMM	MesP1+E11.5EC (APA5-40ug/m	1.8031	0.1131	32	15.94	<.0001	0.05	1.5726	2.0336
BFU-E	MesP1+E11.5EC (APB5-0ug/ml	CFU-GEMM	MesP1+E11.5EC (APB5-0ug/ml	0.3532	0.05738	32	6.16	<.0001	0.05	0.2363	0.4701
BFU-E	MesP1+E11.5EC (APB5-0ug/ml	CFU-GEMM	MesP1+E11.5EC (APB5-25ug/m	0.3949	0.08130	32	4.86	<.0001	0.05	0.2293	0.5605
BFU-E	MesP1+E11.5EC (APB5-0ug/ml	CFU-GEMM	MesP1+E11.5EC (APB5-50ug/m	0.3061	0.08024	32	3.82	0.0006	0.05	0.1427	0.4696

The GLIMMIX Procedure

Differences of Colony_Typ*Condition Least Squares Means											
Colony_Type	Condition	_Colony_Type	_Condition	Estimate	Standard Error	DF	t Value	Pr > |t|	Alpha	Lower	Upper
BFU-E	MesP1+E11.5EC (APB5-0ug/ml	CFU-GEMM	MesP1DsRed only	11.1971	9.9462	32	1.13	0.2686	0.05	-9.0627	31.4568
BFU-E	MesP1+E11.5EC (APB5-0ug/ml	CFU-GM	E11.5EC Only	11.1971	9.9462	32	1.13	0.2686	0.05	-9.0627	31.4569
BFU-E	MesP1+E11.5EC (APB5-0ug/ml	CFU-GM	MesP1+E11.5EC (APA5-0ug/ml	-1.1714	0.07081	32	-16.54	<.0001	0.05	-1.3156	-1.0271
BFU-E	MesP1+E11.5EC (APB5-0ug/ml	CFU-GM	MesP1+E11.5EC (APA5-20ug/m	-0.6767	0.07269	32	-9.31	<.0001	0.05	-0.8248	-0.5286
BFU-E	MesP1+E11.5EC (APB5-0ug/ml	CFU-GM	MesP1+E11.5EC (APA5-40ug/m	-0.1220	0.07615	32	-1.60	0.1190	0.05	-0.2771	0.03312
BFU-E	MesP1+E11.5EC (APB5-0ug/ml	CFU-GM	MesP1+E11.5EC (APB5-0ug/ml	-1.1828	0.04213	32	-28.07	<.0001	0.05	-1.2686	-1.0970
BFU-E	MesP1+E11.5EC (APB5-0ug/ml	CFU-GM	MesP1+E11.5EC (APB5-25ug/m	-1.1504	0.07087	32	-16.23	<.0001	0.05	-1.2947	-1.0060
BFU-E	MesP1+E11.5EC (APB5-0ug/ml	CFU-GM	MesP1+E11.5EC (APB5-50ug/m	-1.1705	0.07081	32	-16.53	<.0001	0.05	-1.3147	-1.0262
BFU-E	MesP1+E11.5EC (APB5-0ug/ml	CFU-GM	MesP1DsRed only	11.1970	10.0047	32	1.12	0.2714	0.05	-9.1820	31.5760
BFU-E	MesP1+E11.5EC (APB5-25ug/m	BFU-E	MesP1+E11.5EC (APB5-50ug/m	-0.06142	0.07803	32	-0.79	0.4370	0.05	-0.2204	0.09751
BFU-E	MesP1+E11.5EC (APB5-25ug/m	BFU-E	MesP1DsRed only	11.1177	9.9462	32	1.12	0.2720	0.05	-9.1422	31.3775
BFU-E	MesP1+E11.5EC (APB5-25ug/m	CFU-GEMM	E11.5EC Only	11.1178	9.9469	32	1.12	0.2720	0.05	-9.1434	31.3790
BFU-E	MesP1+E11.5EC (APB5-25ug/m	CFU-GEMM	MesP1+E11.5EC (APA5-0ug/ml	0.3183	0.08201	32	3.88	0.0005	0.05	0.1513	0.4854
BFU-E	MesP1+E11.5EC (APB5-25ug/m	CFU-GEMM	MesP1+E11.5EC (APA5-20ug/m	0.8964	0.09113	32	9.84	<.0001	0.05	0.7108	1.0821
BFU-E	MesP1+E11.5EC (APB5-25ug/m	CFU-GEMM	MesP1+E11.5EC (APA5-40ug/m	1.7237	0.1136	32	15.17	<.0001	0.05	1.4922	1.9552
BFU-E	MesP1+E11.5EC (APB5-25ug/m	CFU-GEMM	MesP1+E11.5EC (APB5-0ug/ml	0.2738	0.08148	32	3.36	0.0020	0.05	0.1078	0.4398
BFU-E	MesP1+E11.5EC (APB5-25ug/m	CFU-GEMM	MesP1+E11.5EC (APB5-25ug/m	0.3155	0.05905	32	5.34	<.0001	0.05	0.1953	0.4358
BFU-E	MesP1+E11.5EC (APB5-25ug/m	CFU-GEMM	MesP1+E11.5EC (APB5-50ug/m	0.2267	0.08093	32	2.80	0.0086	0.05	0.06188	0.3916
BFU-E	MesP1+E11.5EC (APB5-25ug/m	CFU-GEMM	MesP1DsRed only	11.1177	9.9462	32	1.12	0.2720	0.05	-9.1421	31.3774
BFU-E	MesP1+E11.5EC (APB5-25ug/m	CFU-GM	E11.5EC Only	11.1177	9.9462	32	1.12	0.2720	0.05	-9.1421	31.3775
BFU-E	MesP1+E11.5EC (APB5-25ug/m	CFU-GM	MesP1+E11.5EC (APA5-0ug/ml	-1.2508	0.07160	32	-17.47	<.0001	0.05	-1.3966	-1.1049
BFU-E	MesP1+E11.5EC (APB5-25ug/m	CFU-GM	MesP1+E11.5EC (APA5-20ug/m	-0.7561	0.07346	32	-10.29	<.0001	0.05	-0.9057	-0.6065
BFU-E	MesP1+E11.5EC (APB5-25ug/m	CFU-GM	MesP1+E11.5EC (APA5-40ug/m	-0.2014	0.07689	32	-2.62	0.0133	0.05	-0.3580	-0.04480
BFU-E	MesP1+E11.5EC (APB5-25ug/m	CFU-GM	MesP1+E11.5EC (APB5-0ug/ml	-1.2622	0.07156	32	-17.64	<.0001	0.05	-1.4080	-1.1164

The GLIMMIX Procedure

Differences of Colony_Typ*Condition Least Squares Means											
Colony_Type	Condition	_Colony_Type	_Condition	Estimate	Standard Error	DF	t Value	Pr > |t|	Alpha	Lower	Upper
BFU-E	MesP1+E11.5EC (APB5-25ug/m	CFU-GM	MesP1+E11.5EC (APB5-25ug/m	-1.2298	0.04359	32	-28.21	<.0001	0.05	-1.3186	-1.1410
BFU-E	MesP1+E11.5EC (APB5-25ug/m	CFU-GM	MesP1+E11.5EC (APB5-50ug/m	-1.2499	0.07160	32	-17.46	<.0001	0.05	-1.3957	-1.1040
BFU-E	MesP1+E11.5EC (APB5-25ug/m	CFU-GM	MesP1DsRed only	11.1176	10.0047	32	1.11	0.2748	0.05	-9.2614	31.4966
BFU-E	MesP1+E11.5EC (APB5-50ug/m	BFU-E	MesP1DsRed only	11.1791	9.9462	32	1.12	0.2694	0.05	-9.0807	31.4389
BFU-E	MesP1+E11.5EC (APB5-50ug/m	CFU-GEMM	E11.5EC Only	11.1792	9.9469	32	1.12	0.2694	0.05	-9.0819	31.4404
BFU-E	MesP1+E11.5EC (APB5-50ug/m	CFU-GEMM	MesP1+E11.5EC (APA5-0ug/ml	0.3797	0.08148	32	4.66	<.0001	0.05	0.2138	0.5457
BFU-E	MesP1+E11.5EC (APB5-50ug/m	CFU-GEMM	MesP1+E11.5EC (APA5-20ug/m	0.9579	0.09065	32	10.57	<.0001	0.05	0.7732	1.1425
BFU-E	MesP1+E11.5EC (APB5-50ug/m	CFU-GEMM	MesP1+E11.5EC (APA5-40ug/m	1.7851	0.1133	32	15.76	<.0001	0.05	1.5544	2.0158
BFU-E	MesP1+E11.5EC (APB5-50ug/m	CFU-GEMM	MesP1+E11.5EC (APB5-0ug/ml	0.3352	0.08094	32	4.14	0.0002	0.05	0.1703	0.5001
BFU-E	MesP1+E11.5EC (APB5-50ug/m	CFU-GEMM	MesP1+E11.5EC (APB5-25ug/m	0.3770	0.08145	32	4.63	<.0001	0.05	0.2110	0.5429
BFU-E	MesP1+E11.5EC (APB5-50ug/m	CFU-GEMM	MesP1+E11.5EC (APB5-50ug/m	0.2882	0.05682	32	5.07	<.0001	0.05	0.1724	0.4039
BFU-E	MesP1+E11.5EC (APB5-50ug/m	CFU-GEMM	MesP1DsRed only	11.1791	9.9462	32	1.12	0.2694	0.05	-9.0807	31.4388
BFU-E	MesP1+E11.5EC (APB5-50ug/m	CFU-GM	E11.5EC Only	11.1791	9.9462	32	1.12	0.2694	0.05	-9.0807	31.4389
BFU-E	MesP1+E11.5EC (APB5-50ug/m	CFU-GM	MesP1+E11.5EC (APA5-0ug/ml	-1.1893	0.07098	32	-16.75	<.0001	0.05	-1.3339	-1.0448
BFU-E	MesP1+E11.5EC (APB5-50ug/m	CFU-GM	MesP1+E11.5EC (APA5-20ug/m	-0.6947	0.07286	32	-9.53	<.0001	0.05	-0.8431	-0.5463
BFU-E	MesP1+E11.5EC (APB5-50ug/m	CFU-GM	MesP1+E11.5EC (APA5-40ug/m	-0.1400	0.07631	32	-1.83	0.0759	0.05	-0.2954	0.01546
BFU-E	MesP1+E11.5EC (APB5-50ug/m	CFU-GM	MesP1+E11.5EC (APB5-0ug/ml	-1.2008	0.07095	32	-16.92	<.0001	0.05	-1.3453	-1.0562
BFU-E	MesP1+E11.5EC (APB5-50ug/m	CFU-GM	MesP1+E11.5EC (APB5-25ug/m	-1.1684	0.07105	32	-16.44	<.0001	0.05	-1.3131	-1.0236
BFU-E	MesP1+E11.5EC (APB5-50ug/m	CFU-GM	MesP1+E11.5EC (APB5-50ug/m	-1.1885	0.04248	32	-27.98	<.0001	0.05	-1.2750	-1.1019
BFU-E	MesP1+E11.5EC (APB5-50ug/m	CFU-GM	MesP1DsRed only	11.1790	10.0047	32	1.12	0.2722	0.05	-9.2000	31.5580
BFU-E	MesP1DsRed only	CFU-GEMM	E11.5EC Only	0.000129	14.0664	32	0.00	1.0000	0.05	-28.6521	28.6524
BFU-E	MesP1DsRed only	CFU-GEMM	MesP1+E11.5EC (APA5-0ug/ml	-10.7993	9.9463	32	-1.09	0.2857	0.05	-31.0593	9.4606
BFU-E	MesP1DsRed only	CFU-GEMM	MesP1+E11.5EC (APA5-20ug/m	-10.2212	9.9464	32	-1.03	0.3118	0.05	-30.4813	10.0389
BFU-E	MesP1DsRed only	CFU-GEMM	MesP1+E11.5EC (APA5-40ug/m	-9.3940	9.9466	32	-0.94	0.3520	0.05	-29.6545	10.8666

The GLIMMIX Procedure

Differences of Colony_Typ*Condition Least Squares Means											
Colony_Type	Condition	_Colony_Type	_Condition	Estimate	Standard Error	DF	t Value	Pr > |t|	Alpha	Lower	Upper
BFU-E	MesP1DsRed only	CFU-GEMM	MesP1+E11.5EC (APB5-0ug/ml	-10.8439	9.9463	32	-1.09	0.2837	0.05	-31.1038	9.4160
BFU-E	MesP1DsRed only	CFU-GEMM	MesP1+E11.5EC (APB5-25ug/m	-10.8021	9.9463	32	-1.09	0.2856	0.05	-31.0621	9.4578
BFU-E	MesP1DsRed only	CFU-GEMM	MesP1+E11.5EC (APB5-50ug/m	-10.8909	9.9463	32	-1.09	0.2817	0.05	-31.1508	9.3690
BFU-E	MesP1DsRed only	CFU-GEMM	MesP1DsRed only	-0.00001	14.0658	32	-0.00	1.0000	0.05	-28.6510	28.6510
BFU-E	MesP1DsRed only	CFU-GM	E11.5EC Only	-5E-6	14.0659	32	-0.00	1.0000	0.05	-28.6513	28.6513
BFU-E	MesP1DsRed only	CFU-GM	MesP1+E11.5EC (APA5-0ug/ml	-12.3684	9.9462	32	-1.24	0.2227	0.05	-32.6282	7.8913
BFU-E	MesP1DsRed only	CFU-GM	MesP1+E11.5EC (APA5-20ug/m	-11.8738	9.9462	32	-1.19	0.2413	0.05	-32.1336	8.3860
BFU-E	MesP1DsRed only	CFU-GM	MesP1+E11.5EC (APA5-40ug/m	-11.3191	9.9462	32	-1.14	0.2636	0.05	-31.5789	8.9408
BFU-E	MesP1DsRed only	CFU-GM	MesP1+E11.5EC (APB5-0ug/ml	-12.3798	9.9462	32	-1.24	0.2223	0.05	-32.6396	7.8799
BFU-E	MesP1DsRed only	CFU-GM	MesP1+E11.5EC (APB5-25ug/m	-12.3474	9.9462	32	-1.24	0.2235	0.05	-32.6072	7.9123
BFU-E	MesP1DsRed only	CFU-GM	MesP1+E11.5EC (APB5-50ug/m	-12.3675	9.9462	32	-1.24	0.2227	0.05	-32.6273	7.8922
BFU-E	MesP1DsRed only	CFU-GM	MesP1DsRed only	-0.00011	14.1066	32	-0.00	1.0000	0.05	-28.7343	28.7341
CFU-GEMM	E11.5EC Only	CFU-GEMM	MesP1+E11.5EC (APA5-0ug/ml	-10.7995	9.9469	32	-1.09	0.2857	0.05	-31.0607	9.4617
CFU-GEMM	E11.5EC Only	CFU-GEMM	MesP1+E11.5EC (APA5-20ug/m	-10.2214	9.9470	32	-1.03	0.3119	0.05	-30.4827	10.0400
CFU-GEMM	E11.5EC Only	CFU-GEMM	MesP1+E11.5EC (APA5-40ug/m	-9.3941	9.9472	32	-0.94	0.3520	0.05	-29.6560	10.8678
CFU-GEMM	E11.5EC Only	CFU-GEMM	MesP1+E11.5EC (APB5-0ug/ml	-10.8440	9.9469	32	-1.09	0.2838	0.05	-31.1052	9.4172
CFU-GEMM	E11.5EC Only	CFU-GEMM	MesP1+E11.5EC (APB5-25ug/m	-10.8023	9.9469	32	-1.09	0.2856	0.05	-31.0635	9.4590
CFU-GEMM	E11.5EC Only	CFU-GEMM	MesP1+E11.5EC (APB5-50ug/m	-10.8911	9.9469	32	-1.09	0.2817	0.05	-31.1523	9.3701
CFU-GEMM	E11.5EC Only	CFU-GEMM	MesP1DsRed only	-0.00014	14.0663	32	-0.00	1.0000	0.05	-28.6523	28.6520
CFU-GEMM	E11.5EC Only	CFU-GM	E11.5EC Only	-0.00013	14.0662	32	-0.00	1.0000	0.05	-28.6521	28.6518
CFU-GEMM	E11.5EC Only	CFU-GM	MesP1+E11.5EC (APA5-0ug/ml	-12.3686	9.9468	32	-1.24	0.2227	0.05	-32.6296	7.8925
CFU-GEMM	E11.5EC Only	CFU-GM	MesP1+E11.5EC (APA5-20ug/m	-11.8739	9.9469	32	-1.19	0.2414	0.05	-32.1350	8.3872
CFU-GEMM	E11.5EC Only	CFU-GM	MesP1+E11.5EC (APA5-40ug/m	-11.3192	9.9469	32	-1.14	0.2636	0.05	-31.5803	8.9419
CFU-GEMM	E11.5EC Only	CFU-GM	MesP1+E11.5EC (APB5-0ug/ml	-12.3800	9.9468	32	-1.24	0.2223	0.05	-32.6410	7.8811

The GLIMMIX Procedure

Differences of Colony_Typ*Condition Least Squares Means											
Colony_Type	Condition	_Colony_Type	_Condition	Estimate	Standard Error	DF	t Value	Pr > |t|	Alpha	Lower	Upper
CFU-GEMM	E11.5EC Only	CFU-GM	MesP1+E11.5EC (APB5-25ug/m	-12.3476	9.9468	32	-1.24	0.2235	0.05	-32.6086	7.9135
CFU-GEMM	E11.5EC Only	CFU-GM	MesP1+E11.5EC (APB5-50ug/m	-12.3677	9.9468	32	-1.24	0.2228	0.05	-32.6287	7.8934
CFU-GEMM	E11.5EC Only	CFU-GM	MesP1DsRed only	-0.00024	14.1078	32	-0.00	1.0000	0.05	-28.7368	28.7363
CFU-GEMM	MesP1+E11.5EC (APA5-0ug/ml	CFU-GEMM	MesP1+E11.5EC (APA5-20ug/m	0.5781	0.09410	32	6.14	<.0001	0.05	0.3864	0.7698
CFU-GEMM	MesP1+E11.5EC (APA5-0ug/ml	CFU-GEMM	MesP1+E11.5EC (APA5-40ug/m	1.4054	0.1160	32	12.11	<.0001	0.05	1.1690	1.6417
CFU-GEMM	MesP1+E11.5EC (APA5-0ug/ml	CFU-GEMM	MesP1+E11.5EC (APB5-0ug/ml	-0.04453	0.08479	32	-0.53	0.6031	0.05	-0.2172	0.1282
CFU-GEMM	MesP1+E11.5EC (APA5-0ug/ml	CFU-GEMM	MesP1+E11.5EC (APB5-25ug/m	-0.00279	0.08527	32	-0.03	0.9741	0.05	-0.1765	0.1709
CFU-GEMM	MesP1+E11.5EC (APA5-0ug/ml	CFU-GEMM	MesP1+E11.5EC (APB5-50ug/m	-0.09159	0.08426	32	-1.09	0.2852	0.05	-0.2632	0.08005
CFU-GEMM	MesP1+E11.5EC (APA5-0ug/ml	CFU-GEMM	MesP1DsRed only	10.7993	9.9462	32	1.09	0.2857	0.05	-9.4604	31.0591
CFU-GEMM	MesP1+E11.5EC (APA5-0ug/ml	CFU-GM	E11.5EC Only	10.7993	9.9463	32	1.09	0.2857	0.05	-9.4605	31.0592
CFU-GEMM	MesP1+E11.5EC (APA5-0ug/ml	CFU-GM	MesP1+E11.5EC (APA5-0ug/ml	-1.5691	0.04941	32	-31.76	<.0001	0.05	-1.6697	-1.4684
CFU-GEMM	MesP1+E11.5EC (APA5-0ug/ml	CFU-GM	MesP1+E11.5EC (APA5-20ug/m	-1.0744	0.07711	32	-13.93	<.0001	0.05	-1.2315	-0.9174
CFU-GEMM	MesP1+E11.5EC (APA5-0ug/ml	CFU-GM	MesP1+E11.5EC (APA5-40ug/m	-0.5197	0.08038	32	-6.47	<.0001	0.05	-0.6835	-0.3560
CFU-GEMM	MesP1+E11.5EC (APA5-0ug/ml	CFU-GM	MesP1+E11.5EC (APB5-0ug/ml	-1.5805	0.07531	32	-20.99	<.0001	0.05	-1.7339	-1.4271
CFU-GEMM	MesP1+E11.5EC (APA5-0ug/ml	CFU-GM	MesP1+E11.5EC (APB5-25ug/m	-1.5481	0.07540	32	-20.53	<.0001	0.05	-1.7017	-1.3945
CFU-GEMM	MesP1+E11.5EC (APA5-0ug/ml	CFU-GM	MesP1+E11.5EC (APB5-50ug/m	-1.5682	0.07534	32	-20.81	<.0001	0.05	-1.7217	-1.4147
CFU-GEMM	MesP1+E11.5EC (APA5-0ug/ml	CFU-GM	MesP1DsRed only	10.7992	10.0048	32	1.08	0.2885	0.05	-9.5798	31.1783
CFU-GEMM	MesP1+E11.5EC (APA5-20ug/m	CFU-GEMM	MesP1+E11.5EC (APA5-40ug/m	0.8273	0.1226	32	6.75	<.0001	0.05	0.5774	1.0771
CFU-GEMM	MesP1+E11.5EC (APA5-20ug/m	CFU-GEMM	MesP1+E11.5EC (APB5-0ug/ml	-0.6226	0.09364	32	-6.65	<.0001	0.05	-0.8134	-0.4319
CFU-GEMM	MesP1+E11.5EC (APA5-20ug/m	CFU-GEMM	MesP1+E11.5EC (APB5-25ug/m	-0.5809	0.09408	32	-6.17	<.0001	0.05	-0.7725	-0.3893
CFU-GEMM	MesP1+E11.5EC (APA5-20ug/m	CFU-GEMM	MesP1+E11.5EC (APB5-50ug/m	-0.6697	0.09316	32	-7.19	<.0001	0.05	-0.8595	-0.4799
CFU-GEMM	MesP1+E11.5EC (APA5-20ug/m	CFU-GEMM	MesP1DsRed only	10.2212	9.9463	32	1.03	0.3118	0.05	-10.0387	30.4811
CFU-GEMM	MesP1+E11.5EC (APA5-20ug/m	CFU-GM	E11.5EC Only	10.2212	9.9463	32	1.03	0.3118	0.05	-10.0388	30.4812
CFU-GEMM	MesP1+E11.5EC (APA5-20ug/m	CFU-GM	MesP1+E11.5EC (APA5-0ug/ml	-2.1472	0.08518	32	-25.21	<.0001	0.05	-2.3207	-1.9737

The GLIMMIX Procedure

Differences of Colony_Typ*Condition Least Squares Means											
Colony_Type	Condition	_Colony_Type	_Condition	Estimate	Standard Error	DF	t Value	Pr > |t|	Alpha	Lower	Upper
CFU-GEMM	MesP1+E11.5EC (APA5-20ug/m	CFU-GM	MesP1+E11.5EC (APA5-20ug/m	-1.6526	0.06547	32	-25.24	<.0001	0.05	-1.7859	-1.5192
CFU-GEMM	MesP1+E11.5EC (APA5-20ug/m	CFU-GM	MesP1+E11.5EC (APA5-40ug/m	-1.0979	0.08966	32	-12.25	<.0001	0.05	-1.2805	-0.9152
CFU-GEMM	MesP1+E11.5EC (APA5-20ug/m	CFU-GM	MesP1+E11.5EC (APB5-0ug/ml	-2.1586	0.08515	32	-25.35	<.0001	0.05	-2.3321	-1.9852
CFU-GEMM	MesP1+E11.5EC (APA5-20ug/m	CFU-GM	MesP1+E11.5EC (APB5-25ug/m	-2.1262	0.08523	32	-24.95	<.0001	0.05	-2.2998	-1.9526
CFU-GEMM	MesP1+E11.5EC (APA5-20ug/m	CFU-GM	MesP1+E11.5EC (APB5-50ug/m	-2.1463	0.08518	32	-25.20	<.0001	0.05	-2.3198	-1.9728
CFU-GEMM	MesP1+E11.5EC (APA5-20ug/m	CFU-GM	MesP1DsRed only	10.2211	10.0048	32	1.02	0.3146	0.05	-10.1580	30.6002
CFU-GEMM	MesP1+E11.5EC (APA5-40ug/m	CFU-GEMM	MesP1+E11.5EC (APB5-0ug/ml	-1.4499	0.1157	32	-12.54	<.0001	0.05	-1.6855	-1.2143
CFU-GEMM	MesP1+E11.5EC (APA5-40ug/m	CFU-GEMM	MesP1+E11.5EC (APB5-25ug/m	-1.4082	0.1160	32	-12.14	<.0001	0.05	-1.6445	-1.1718
CFU-GEMM	MesP1+E11.5EC (APA5-40ug/m	CFU-GEMM	MesP1+E11.5EC (APB5-50ug/m	-1.4970	0.1153	32	-12.99	<.0001	0.05	-1.7318	-1.2621
CFU-GEMM	MesP1+E11.5EC (APA5-40ug/m	CFU-GEMM	MesP1DsRed only	9.3940	9.9465	32	0.94	0.3520	0.05	-10.8664	29.6544
CFU-GEMM	MesP1+E11.5EC (APA5-40ug/m	CFU-GM	E11.5EC Only	9.3940	9.9466	32	0.94	0.3520	0.05	-10.8665	29.6545
CFU-GEMM	MesP1+E11.5EC (APA5-40ug/m	CFU-GM	MesP1+E11.5EC (APA5-0ug/ml	-2.9745	0.1089	32	-27.31	<.0001	0.05	-3.1963	-2.7526
CFU-GEMM	MesP1+E11.5EC (APA5-40ug/m	CFU-GM	MesP1+E11.5EC (APA5-20ug/m	-2.4798	0.1102	32	-22.51	<.0001	0.05	-2.7042	-2.2554
CFU-GEMM	MesP1+E11.5EC (APA5-40ug/m	CFU-GM	MesP1+E11.5EC (APA5-40ug/m	-1.9251	0.09693	32	-19.86	<.0001	0.05	-2.1225	-1.7277
CFU-GEMM	MesP1+E11.5EC (APA5-40ug/m	CFU-GM	MesP1+E11.5EC (APB5-0ug/ml	-2.9859	0.1089	32	-27.42	<.0001	0.05	-3.2077	-2.7640
CFU-GEMM	MesP1+E11.5EC (APA5-40ug/m	CFU-GM	MesP1+E11.5EC (APB5-25ug/m	-2.9535	0.1090	32	-27.10	<.0001	0.05	-3.1754	-2.7315
CFU-GEMM	MesP1+E11.5EC (APA5-40ug/m	CFU-GM	MesP1+E11.5EC (APB5-50ug/m	-2.9736	0.1089	32	-27.30	<.0001	0.05	-3.1955	-2.7517
CFU-GEMM	MesP1+E11.5EC (APA5-40ug/m	CFU-GM	MesP1DsRed only	9.3939	10.0049	32	0.94	0.3548	0.05	-10.9854	29.7731
CFU-GEMM	MesP1+E11.5EC (APB5-0ug/ml	CFU-GEMM	MesP1+E11.5EC (APB5-25ug/m	0.04174	0.08476	32	0.49	0.6258	0.05	-0.1309	0.2144
CFU-GEMM	MesP1+E11.5EC (APB5-0ug/ml	CFU-GEMM	MesP1+E11.5EC (APB5-50ug/m	-0.04706	0.08375	32	-0.56	0.5781	0.05	-0.2177	0.1235
CFU-GEMM	MesP1+E11.5EC (APB5-0ug/ml	CFU-GEMM	MesP1DsRed only	10.8439	9.9462	32	1.09	0.2837	0.05	-9.4159	31.1036
CFU-GEMM	MesP1+E11.5EC (APB5-0ug/ml	CFU-GM	E11.5EC Only	10.8439	9.9463	32	1.09	0.2837	0.05	-9.4160	31.1037
CFU-GEMM	MesP1+E11.5EC (APB5-0ug/ml	CFU-GM	MesP1+E11.5EC (APA5-0ug/ml	-1.5246	0.07477	32	-20.39	<.0001	0.05	-1.6769	-1.3723
CFU-GEMM	MesP1+E11.5EC (APB5-0ug/ml	CFU-GM	MesP1+E11.5EC (APA5-20ug/m	-1.0299	0.07655	32	-13.45	<.0001	0.05	-1.1858	-0.8740

The GLIMMIX Procedure

Differences of Colony_Typ*Condition Least Squares Means											
Colony_Type	Condition	_Colony_Type	_Condition	Estimate	Standard Error	DF	t Value	Pr > |t|	Alpha	Lower	Upper
CFU-GEMM	MesP1+E11.5EC (APB5-0ug/ml	CFU-GM	MesP1+E11.5EC (APA5-40ug/m	-0.4752	0.07984	32	-5.95	<.0001	0.05	-0.6378	-0.3126
CFU-GEMM	MesP1+E11.5EC (APB5-0ug/ml	CFU-GM	MesP1+E11.5EC (APB5-0ug/ml	-1.5360	0.04848	32	-31.68	<.0001	0.05	-1.6347	-1.4372
CFU-GEMM	MesP1+E11.5EC (APB5-0ug/ml	CFU-GM	MesP1+E11.5EC (APB5-25ug/m	-1.5036	0.07482	32	-20.09	<.0001	0.05	-1.6560	-1.3512
CFU-GEMM	MesP1+E11.5EC (APB5-0ug/ml	CFU-GM	MesP1+E11.5EC (APB5-50ug/m	-1.5237	0.07477	32	-20.38	<.0001	0.05	-1.6760	-1.3714
CFU-GEMM	MesP1+E11.5EC (APB5-0ug/ml	CFU-GM	MesP1DsRed only	10.8438	10.0048	32	1.08	0.2865	0.05	-9.5353	31.2228
CFU-GEMM	MesP1+E11.5EC (APB5-25ug/m	CFU-GEMM	MesP1+E11.5EC (APB5-50ug/m	-0.08880	0.08424	32	-1.05	0.2997	0.05	-0.2604	0.08279
CFU-GEMM	MesP1+E11.5EC (APB5-25ug/m	CFU-GEMM	MesP1DsRed only	10.8021	9.9462	32	1.09	0.2856	0.05	-9.4577	31.0619
CFU-GEMM	MesP1+E11.5EC (APB5-25ug/m	CFU-GM	E11.5EC Only	10.8021	9.9463	32	1.09	0.2856	0.05	-9.4577	31.0620
CFU-GEMM	MesP1+E11.5EC (APB5-25ug/m	CFU-GM	MesP1+E11.5EC (APA5-0ug/ml	-1.5663	0.07531	32	-20.80	<.0001	0.05	-1.7197	-1.4129
CFU-GEMM	MesP1+E11.5EC (APB5-25ug/m	CFU-GM	MesP1+E11.5EC (APA5-20ug/m	-1.0716	0.07708	32	-13.90	<.0001	0.05	-1.2287	-0.9146
CFU-GEMM	MesP1+E11.5EC (APB5-25ug/m	CFU-GM	MesP1+E11.5EC (APA5-40ug/m	-0.5169	0.08035	32	-6.43	<.0001	0.05	-0.6806	-0.3533
CFU-GEMM	MesP1+E11.5EC (APB5-25ug/m	CFU-GM	MesP1+E11.5EC (APB5-0ug/ml	-1.5777	0.07528	32	-20.96	<.0001	0.05	-1.7311	-1.4244
CFU-GEMM	MesP1+E11.5EC (APB5-25ug/m	CFU-GM	MesP1+E11.5EC (APB5-25ug/m	-1.5453	0.04946	32	-31.25	<.0001	0.05	-1.6461	-1.4446
CFU-GEMM	MesP1+E11.5EC (APB5-25ug/m	CFU-GM	MesP1+E11.5EC (APB5-50ug/m	-1.5654	0.07531	32	-20.79	<.0001	0.05	-1.7188	-1.4120
CFU-GEMM	MesP1+E11.5EC (APB5-25ug/m	CFU-GM	MesP1DsRed only	10.8020	10.0048	32	1.08	0.2884	0.05	-9.5770	31.1811
CFU-GEMM	MesP1+E11.5EC (APB5-50ug/m	CFU-GEMM	MesP1DsRed only	10.8909	9.9462	32	1.09	0.2817	0.05	-9.3688	31.1507
CFU-GEMM	MesP1+E11.5EC (APB5-50ug/m	CFU-GM	E11.5EC Only	10.8909	9.9462	32	1.09	0.2817	0.05	-9.3689	31.1508
CFU-GEMM	MesP1+E11.5EC (APB5-50ug/m	CFU-GM	MesP1+E11.5EC (APA5-0ug/ml	-1.4775	0.07417	32	-19.92	<.0001	0.05	-1.6286	-1.3264
CFU-GEMM	MesP1+E11.5EC (APB5-50ug/m	CFU-GM	MesP1+E11.5EC (APA5-20ug/m	-0.9828	0.07596	32	-12.94	<.0001	0.05	-1.1376	-0.8281
CFU-GEMM	MesP1+E11.5EC (APB5-50ug/m	CFU-GM	MesP1+E11.5EC (APA5-40ug/m	-0.4281	0.07928	32	-5.40	<.0001	0.05	-0.5896	-0.2666
CFU-GEMM	MesP1+E11.5EC (APB5-50ug/m	CFU-GM	MesP1+E11.5EC (APB5-0ug/ml	-1.4889	0.07413	32	-20.08	<.0001	0.05	-1.6399	-1.3379
CFU-GEMM	MesP1+E11.5EC (APB5-50ug/m	CFU-GM	MesP1+E11.5EC (APB5-25ug/m	-1.4565	0.07423	32	-19.62	<.0001	0.05	-1.6077	-1.3053
CFU-GEMM	MesP1+E11.5EC (APB5-50ug/m	CFU-GM	MesP1+E11.5EC (APB5-50ug/m	-1.4766	0.04761	32	-31.02	<.0001	0.05	-1.5736	-1.3796
CFU-GEMM	MesP1+E11.5EC (APB5-50ug/m	CFU-GM	MesP1DsRed only	10.8908	10.0048	32	1.09	0.2845	0.05	-9.4882	31.2699

The GLIMMIX Procedure

Differences of Colony_Typ*Condition Least Squares Means											
Colony_Type	Condition	_Colony_Type	_Condition	Estimate	Standard Error	DF	t Value	Pr > |t|	Alpha	Lower	Upper
CFU-GEMM	MesP1DsRed only	CFU-GM	E11.5EC Only	5.921E-6	14.0659	32	0.00	1.0000	0.05	-28.6512	28.6512
CFU-GEMM	MesP1DsRed only	CFU-GM	MesP1+E11.5EC (APA5-0ug/ml	-12.3684	9.9462	32	-1.24	0.2227	0.05	-32.6281	7.8912
CFU-GEMM	MesP1DsRed only	CFU-GM	MesP1+E11.5EC (APA5-20ug/m	-11.8738	9.9462	32	-1.19	0.2413	0.05	-32.1334	8.3859
CFU-GEMM	MesP1DsRed only	CFU-GM	MesP1+E11.5EC (APA5-40ug/m	-11.3191	9.9462	32	-1.14	0.2636	0.05	-31.5788	8.9407
CFU-GEMM	MesP1DsRed only	CFU-GM	MesP1+E11.5EC (APB5-0ug/ml	-12.3798	9.9462	32	-1.24	0.2223	0.05	-32.6395	7.8798
CFU-GEMM	MesP1DsRed only	CFU-GM	MesP1+E11.5EC (APB5-25ug/m	-12.3474	9.9462	32	-1.24	0.2235	0.05	-32.6071	7.9122
CFU-GEMM	MesP1DsRed only	CFU-GM	MesP1+E11.5EC (APB5-50ug/m	-12.3675	9.9462	32	-1.24	0.2227	0.05	-32.6272	7.8921
CFU-GEMM	MesP1DsRed only	CFU-GM	MesP1DsRed only	-0.00010	14.1068	32	-0.00	1.0000	0.05	-28.7347	28.7345
CFU-GM	E11.5EC Only	CFU-GM	MesP1+E11.5EC (APA5-0ug/ml	-12.3684	9.9462	32	-1.24	0.2227	0.05	-32.6281	7.8913
CFU-GM	E11.5EC Only	CFU-GM	MesP1+E11.5EC (APA5-20ug/m	-11.8738	9.9462	32	-1.19	0.2413	0.05	-32.1335	8.3860
CFU-GM	E11.5EC Only	CFU-GM	MesP1+E11.5EC (APA5-40ug/m	-11.3191	9.9462	32	-1.14	0.2636	0.05	-31.5788	8.9407
CFU-GM	E11.5EC Only	CFU-GM	MesP1+E11.5EC (APB5-0ug/ml	-12.3798	9.9462	32	-1.24	0.2223	0.05	-32.6395	7.8799
CFU-GM	E11.5EC Only	CFU-GM	MesP1+E11.5EC (APB5-25ug/m	-12.3474	9.9462	32	-1.24	0.2235	0.05	-32.6071	7.9123
CFU-GM	E11.5EC Only	CFU-GM	MesP1+E11.5EC (APB5-50ug/m	-12.3675	9.9462	32	-1.24	0.2227	0.05	-32.6272	7.8922
CFU-GM	E11.5EC Only	CFU-GM	MesP1DsRed only	-0.00011	14.1073	32	-0.00	1.0000	0.05	-28.7357	28.7355
CFU-GM	MesP1+E11.5EC (APA5-0ug/ml	CFU-GM	MesP1+E11.5EC (APA5-20ug/m	0.4947	0.06593	32	7.50	<.0001	0.05	0.3604	0.6289
CFU-GM	MesP1+E11.5EC (APA5-0ug/ml	CFU-GM	MesP1+E11.5EC (APA5-40ug/m	1.0494	0.06972	32	15.05	<.0001	0.05	0.9073	1.1914
CFU-GM	MesP1+E11.5EC (APA5-0ug/ml	CFU-GM	MesP1+E11.5EC (APB5-0ug/ml	-0.01142	0.06381	32	-0.18	0.8591	0.05	-0.1414	0.1186
CFU-GM	MesP1+E11.5EC (APA5-0ug/ml	CFU-GM	MesP1+E11.5EC (APB5-25ug/m	0.02098	0.06392	32	0.33	0.7448	0.05	-0.1092	0.1512
CFU-GM	MesP1+E11.5EC (APA5-0ug/ml	CFU-GM	MesP1+E11.5EC (APB5-50ug/m	0.000882	0.06385	32	0.01	0.9891	0.05	-0.1292	0.1309
CFU-GM	MesP1+E11.5EC (APA5-0ug/ml	CFU-GM	MesP1DsRed only	12.3683	10.0047	32	1.24	0.2254	0.05	-8.0105	32.7472
CFU-GM	MesP1+E11.5EC (APA5-20ug/m	CFU-GM	MesP1+E11.5EC (APA5-40ug/m	0.5547	0.07162	32	7.74	<.0001	0.05	0.4088	0.7006
CFU-GM	MesP1+E11.5EC (APA5-20ug/m	CFU-GM	MesP1+E11.5EC (APB5-0ug/ml	-0.5061	0.06589	32	-7.68	<.0001	0.05	-0.6403	-0.3719
CFU-GM	MesP1+E11.5EC (APA5-20ug/m	CFU-GM	MesP1+E11.5EC (APB5-25ug/m	-0.4737	0.06600	32	-7.18	<.0001	0.05	-0.6081	-0.3392

The GLIMMIX Procedure

Differences of Colony_Typ*Condition Least Squares Means											
Colony_Type	Condition	_Colony_Type	_Condition	Estimate	Standard Error	DF	t Value	Pr > |t|	Alpha	Lower	Upper
CFU-GM	MesP1+E11.5EC (APA5-20ug/m	CFU-GM	MesP1+E11.5EC (APB5-50ug/m	-0.4938	0.06593	32	-7.49	<.0001	0.05	-0.6281	-0.3595
CFU-GM	MesP1+E11.5EC (APA5-20ug/m	CFU-GM	MesP1DsRed only	11.8737	10.0046	32	1.19	0.2440	0.05	-8.5051	32.2525
CFU-GM	MesP1+E11.5EC (APA5-40ug/m	CFU-GM	MesP1+E11.5EC (APB5-0ug/ml	-1.0608	0.06969	32	-15.22	<.0001	0.05	-1.2027	-0.9188
CFU-GM	MesP1+E11.5EC (APA5-40ug/m	CFU-GM	MesP1+E11.5EC (APB5-25ug/m	-1.0284	0.06979	32	-14.74	<.0001	0.05	-1.1705	-0.8862
CFU-GM	MesP1+E11.5EC (APA5-40ug/m	CFU-GM	MesP1+E11.5EC (APB5-50ug/m	-1.0485	0.06973	32	-15.04	<.0001	0.05	-1.1905	-0.9064
CFU-GM	MesP1+E11.5EC (APA5-40ug/m	CFU-GM	MesP1DsRed only	11.3190	10.0045	32	1.13	0.2663	0.05	-9.0596	31.6975
CFU-GM	MesP1+E11.5EC (APB5-0ug/ml	CFU-GM	MesP1+E11.5EC (APB5-25ug/m	0.03240	0.06388	32	0.51	0.6155	0.05	-0.09772	0.1625
CFU-GM	MesP1+E11.5EC (APB5-0ug/ml	CFU-GM	MesP1+E11.5EC (APB5-50ug/m	0.01230	0.06381	32	0.19	0.8484	0.05	-0.1177	0.1423
CFU-GM	MesP1+E11.5EC (APB5-0ug/ml	CFU-GM	MesP1DsRed only	12.3797	10.0047	32	1.24	0.2249	0.05	-7.9991	32.7586
CFU-GM	MesP1+E11.5EC (APB5-25ug/m	CFU-GM	MesP1+E11.5EC (APB5-50ug/m	-0.02010	0.06392	32	-0.31	0.7552	0.05	-0.1503	0.1101
CFU-GM	MesP1+E11.5EC (APB5-25ug/m	CFU-GM	MesP1DsRed only	12.3473	10.0047	32	1.23	0.2261	0.05	-8.0315	32.7262
CFU-GM	MesP1+E11.5EC (APB5-50ug/m	CFU-GM	MesP1DsRed only	12.3674	10.0047	32	1.24	0.2254	0.05	-8.0114	32.7463

The GENMOD Procedure

Model Information	
**Data Set**	WORK.FIG7BIII
**Distribution**	Normal
**Link Function**	Identity
**Dependent Variable**	Percentage

**Number of Observations Read**	25
**Number of Observations Used**	25

Class Level Information		
**Class**	**Levels**	**Values**
**Condition**	3	E11.5EC Only E11.5EC+E11.5PSC E11.5PSC Only
**Treatment**	3	0ug/ml APA5 20ug/ml APA5 40ug/ml APA5

Parameter Information			
**Parameter**	**Effect**	**Condition**	**Treatment**
**Prm1**	Intercept		
**Prm2**	Condition	E11.5EC Only	
**Prm3**	Condition	E11.5EC+E11.5PSC	
**Prm4**	Condition	E11.5PSC Only	
**Prm5**	Treatment		0ug/ml APA5
**Prm6**	Treatment		20ug/ml APA5
**Prm7**	Treatment		40ug/ml APA5
**Prm8**	Condition*Treatment	E11.5EC Only	0ug/ml APA5
**Prm9**	Condition*Treatment	E11.5EC+E11.5PSC	0ug/ml APA5
**Prm10**	Condition*Treatment	E11.5EC+E11.5PSC	20ug/ml APA5

The GENMOD Procedure

Parameter Information			
**Parameter**	**Effect**	**Condition**	**Treatment**
**Prm11**	Condition*Treatment	E11.5EC+E11.5PSC	40ug/ml APA5
**Prm12**	Condition*Treatment	E11.5PSC Only	0ug/ml APA5

Criteria For Assessing Goodness Of Fit			
**Criterion**	**DF**	**Value**	**Value/DF**
**Deviance**	20	134.0000	6.7000
**Scaled Deviance**	20	25.0000	1.2500
**Pearson Chi-Square**	20	134.0000	6.7000
**Scaled Pearson X2**	20	25.0000	1.2500
**Log Likelihood**		-56.4605	
**Full Log Likelihood**		-56.4605	
**AIC (smaller is better)**		124.9210	
**AICC (smaller is better)**		129.5877	
**BIC (smaller is better)**		132.2343	

Algorithm converged.

Analysis Of Maximum Likelihood Parameter Estimates									
**Parameter**			**DF**	**Estimate**	**Standard Error**	**Wald 95% Confidence Limits**		**Wald Chi-Square**	**Pr > ChiSq**
**Intercept**			1	-27.2000	1.7933	-30.7148	-23.6852	230.05	<.0001
**Condition**	E11.5EC Only		1	0.0000	1.4642	-2.8699	2.8699	0.00	1.0000
**Condition**	E11.5EC+E11.5PSC		1	33.2000	1.4642	30.3301	36.0699	514.10	<.0001
**Condition**	E11.5PSC Only		0	0.0000	0.0000	0.0000	0.0000	.	.
**Treatment**	0ug/ml APA5		1	27.2000	1.4642	24.3301	30.0699	345.07	<.0001

The GENMOD Procedure

Analysis Of Maximum Likelihood Parameter Estimates

Parameter			DF	Estimate	Standard Error	Wald 95% Confidence Limits		Wald Chi-Square	Pr > ChiSq
**Treatment**	20ug/ml APA5		1	7.4000	1.4642	4.5301	10.2699	25.54	<.0001
**Treatment**	40ug/ml APA5		0	0.0000	0.0000	0.0000	0.0000	.	.
**Condition*Treatment**	E11.5EC Only	0ug/ml APA5	0	0.0000	0.0000	0.0000	0.0000	.	.
**Condition*Treatment**	E11.5EC+E11.5PSC	0ug/ml APA5	0	0.0000	0.0000	0.0000	0.0000	.	.
**Condition*Treatment**	E11.5EC+E11.5PSC	20ug/ml APA5	0	0.0000	0.0000	0.0000	0.0000	.	.
**Condition*Treatment**	E11.5EC+E11.5PSC	40ug/ml APA5	0	0.0000	0.0000	0.0000	0.0000	.	.
**Condition*Treatment**	E11.5PSC Only	0ug/ml APA5	0	0.0000	0.0000	0.0000	0.0000	.	.
**Scale**			1	2.3152	0.3274	1.7547	3.0546		

Note: The scale parameter was estimated by maximum likelihood.

Condition*Treatment Least Squares Means

Condition	Treatment	Estimate	Standard Error	z Value	Pr > |z|	Alpha	Lower	Upper
E11.5EC Only	0ug/ml APA5	0	1.0354	0.00	1.0000	0.05	-2.0293	2.0293
E11.5EC+E11.5PSC	0ug/ml APA5	33.2000	1.0354	32.07	<.0001	0.05	31.1707	35.2293
E11.5EC+E11.5PSC	20ug/ml APA5	13.4000	1.0354	12.94	<.0001	0.05	11.3707	15.4293
E11.5EC+E11.5PSC	40ug/ml APA5	6.0000	1.0354	5.80	<.0001	0.05	3.9707	8.0293
E11.5PSC Only	0ug/ml APA5	-711E-17	1.0354	-0.00	1.0000	0.05	-2.0293	2.0293

Differences of Condition*Treatment Least Squares Means

Condition	Treatment	_Condition	_Treatment	Estimate	Standard Error	z Value	Pr > |z|	Alpha	Lower	Upper
E11.5EC Only	0ug/ml APA5	E11.5EC+E11.5PSC	0ug/ml APA5	-33.2000	1.4642	-22.67	<.0001	0.05	-36.0699	-30.3301
E11.5EC Only	0ug/ml APA5	E11.5EC+E11.5PSC	20ug/ml APA5	-13.4000	1.4642	-9.15	<.0001	0.05	-16.2699	-10.5301
E11.5EC Only	0ug/ml APA5	E11.5EC+E11.5PSC	40ug/ml APA5	-6.0000	1.4642	-4.10	<.0001	0.05	-8.8699	-3.1301
E11.5EC Only	0ug/ml APA5	E11.5PSC Only	0ug/ml APA5	7.11E-15	1.4642	0.00	1.0000	0.05	-2.8699	2.8699

The GENMOD Procedure

Differences of Condition*Treatment Least Squares Means

Condition	Treatment	_Condition	_Treatment	Estimate	Standard Error	z Value	Pr > |z|	Alpha	Lower	Upper
E11.5EC+E11.5PSC	0ug/ml APA5	E11.5EC+E11.5PSC	20ug/ml APA5	19.8000	1.4642	13.52	<.0001	0.05	16.9301	22.6699
E11.5EC+E11.5PSC	0ug/ml APA5	E11.5EC+E11.5PSC	40ug/ml APA5	27.2000	1.4642	18.58	<.0001	0.05	24.3301	30.0699
E11.5EC+E11.5PSC	0ug/ml APA5	E11.5PSC Only	0ug/ml APA5	33.2000	1.4642	22.67	<.0001	0.05	30.3301	36.0699
E11.5EC+E11.5PSC	20ug/ml APA5	E11.5EC+E11.5PSC	40ug/ml APA5	7.4000	1.4642	5.05	<.0001	0.05	4.5301	10.2699
E11.5EC+E11.5PSC	20ug/ml APA5	E11.5PSC Only	0ug/ml APA5	13.4000	1.4642	9.15	<.0001	0.05	10.5301	16.2699
E11.5EC+E11.5PSC	40ug/ml APA5	E11.5PSC Only	0ug/ml APA5	6.0000	1.4642	4.10	<.0001	0.05	3.1301	8.8699

The GENMOD Procedure

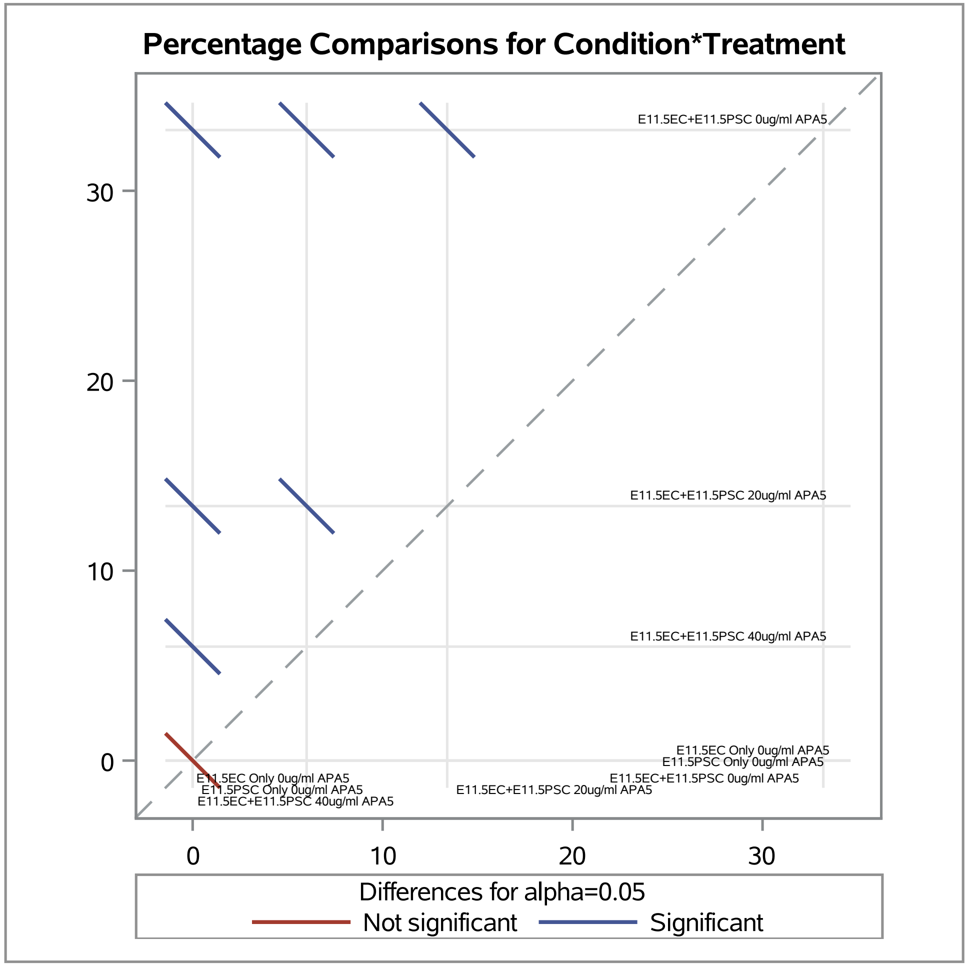

The GLIMMIX Procedure

Model Information	
Data Set	WORK.FIG7CIV
Response Variable	Count
Response Distribution	Poisson
Link Function	Log
Variance Function	Default
Variance Matrix Blocked By	Dish_ID
Estimation Technique	Maximum Likelihood
Likelihood Approximation	Laplace
Degrees of Freedom Method	Containment

Class Level Information		
Class	Levels	Values
Colony_Type	4	BFU-E CFU-GEMM CFU-GM Mix
Condition	2	APA5-0ug/ml APA5-20ug/ml
Dish_ID	6	1 2 3 4 5 6

Number of Observations Read	24
Number of Observations Used	24

Dimensions	
G-side Cov. Parameters	1
Columns in X	15
Columns in Z per Subject	1
Subjects (Blocks in V)	6
Max Obs per Subject	4

The GLIMMIX Procedure

Optimization Information	
**Optimization Technique**	Dual Quasi-Newton
**Parameters in Optimization**	9
**Lower Boundaries**	1
**Upper Boundaries**	0
**Fixed Effects**	Not Profiled
**Starting From**	GLM estimates

Iteration History					
**Iteration**	**Restarts**	**Evaluations**	**Objective Function**	**Change**	**Max Gradient**
**0**	**0**	4	241.53376081	.	1852.548
**1**	**0**	3	240.26271472	1.27104609	0.009842
**2**	**0**	4	240.26271472	0.00000000	0.008991

Convergence criterion (GCONV=1E-8) satisfied.

Estimated G matrix is not positive definite.

Fit Statistics	
**-2 Log Likelihood**	240.26
**AIC (smaller is better)**	256.26
**AICC (smaller is better)**	265.86
**BIC (smaller is better)**	254.60
**CAIC (smaller is better)**	262.60
**HQIC (smaller is better)**	249.59

The GLIMMIX Procedure

Fit Statistics for Conditional Distribution	
**-2 log L(Count | r. effects)**	240.26
**Pearson Chi-Square**	85.08
**Pearson Chi-Square / DF**	3.54

Covariance Parameter Estimates			
**Cov Parm**	**Subject**	**Estimate**	**Standard Error**
**Intercept**	Dish_ID	0	.

Solutions for Fixed Effects							
**Effect**	**Colony_Type**	**Condition**	**Estimate**	**Standard Error**	**DF**	**t Value**	**Pr > |t|**
**Intercept**			1.9924	0.2132	4	9.35	0.0007
**Colony_Type**	BFU-E		2.5579	0.2213	12	11.56	<.0001
**Colony_Type**	CFU-GEMM		2.6866	0.2203	12	12.19	<.0001
**Colony_Type**	CFU-GM		3.8077	0.2156	12	17.66	<.0001
**Colony_Type**	Mix		0	.	.	.	.
**Condition**		APA5-0ug/ml	1.0033	0.2492	12	4.03	0.0017
**Condition**		APA5-20ug/ml	0	.	.	.	.
**Colony_Typ*Condition**	BFU-E	APA5-0ug/ml	-0.2790	0.2595	12	-1.07	0.3036
**Colony_Typ*Condition**	BFU-E	APA5-20ug/ml	0	.	.	.	.
**Colony_Typ*Condition**	CFU-GEMM	APA5-0ug/ml	-0.7125	0.2599	12	-2.74	0.0179
**Colony_Typ*Condition**	CFU-GEMM	APA5-20ug/ml	0	.	.	.	.
**Colony_Typ*Condition**	CFU-GM	APA5-0ug/ml	-0.2828	0.2522	12	-1.12	0.2842
**Colony_Typ*Condition**	CFU-GM	APA5-20ug/ml	0	.	.	.	.

The GLIMMIX Procedure

Solutions for Fixed Effects							
Effect	Colony_Type	Condition	Estimate	Standard Error	DF	t Value	Pr > |t|
Colony_Typ*Condition	Mix	APA5-0ug/ml	0	.	.	.	.
Colony_Typ*Condition	Mix	APA5-20ug/ml	0	.	.	.	.

Type III Tests of Fixed Effects				
Effect	Num DF	Den DF	F Value	Pr > F
Colony_Type	3	12	751.96	<.0001
Condition	1	12	101.02	<.0001
Colony_Typ*Condition	3	12	10.08	0.0013

Condition Least Squares Means												
Condition	Estimate	Standard Error	DF	t Value	Pr > |t|	Alpha	Lower	Upper	Mean	Standard Error Mean	Lower Mean	Upper Mean
APA5-0ug/ml	4.9402	0.03638	12	135.79	<.0001	0.05	4.8609	5.0195	139.80	5.0863	129.15	151.33
APA5-20ug/ml	4.2555	0.05760	12	73.88	<.0001	0.05	4.1300	4.3810	70.4908	4.0601	62.1771	79.9162

Differences of Condition Least Squares Means									
Condition	_Condition	Estimate	Standard Error	DF	t Value	Pr > |t|	Alpha	Lower	Upper
APA5-0ug/ml	APA5-20ug/ml	0.6847	0.06813	12	10.05	<.0001	0.05	0.5363	0.8332

The GLIMMIX Procedure

Colony_Typ*Condition Least Squares Means													
Colony_Type	Condition	Estimate	Standard Error	DF	t Value	Pr > |t|	Alpha	Lower	Upper	Mean	Standard Error Mean	Lower Mean	Upper Mean
BFU-E	APA5-0ug/ml	5.2747	0.04131	12	127.69	<.0001	0.05	5.1847	5.3647	195.33	8.0691	178.52	213.73
BFU-E	APA5-20ug/ml	4.5504	0.05934	12	76.68	<.0001	0.05	4.4211	4.6796	94.6666	5.6174	83.1855	107.73
CFU-GEMM	APA5-0ug/ml	4.9698	0.04811	12	103.30	<.0001	0.05	4.8650	5.0746	144.00	6.9282	129.67	159.91
CFU-GEMM	APA5-20ug/ml	4.6790	0.05564	12	84.09	<.0001	0.05	4.5578	4.8003	107.67	5.9907	95.3741	121.54
CFU-GM	APA5-0ug/ml	6.5206	0.02216	12	294.30	<.0001	0.05	6.4723	6.5689	679.00	15.0443	647.00	712.58
CFU-GM	APA5-20ug/ml	5.8001	0.03177	12	182.59	<.0001	0.05	5.7309	5.8693	330.33	10.4933	308.24	354.01
Mix	APA5-0ug/ml	2.9957	0.1291	12	23.20	<.0001	0.05	2.7144	3.2770	20.0000	2.5820	15.0963	26.4965
Mix	APA5-20ug/ml	1.9924	0.2132	12	9.35	<.0001	0.05	1.5279	2.4570	7.3333	1.5635	4.6085	11.6692

Differences of Colony_Typ*Condition Least Squares Means											
Colony_Type	Condition	_Colony_Type	_Condition	Estimate	Standard Error	DF	t Value	Pr > |t|	Alpha	Lower	Upper
BFU-E	APA5-0ug/ml	BFU-E	APA5-20ug/ml	0.7243	0.07230	12	10.02	<.0001	0.05	0.5668	0.8819
BFU-E	APA5-0ug/ml	CFU-GEMM	APA5-0ug/ml	0.3049	0.06341	12	4.81	0.0004	0.05	0.1667	0.4431
BFU-E	APA5-0ug/ml	CFU-GEMM	APA5-20ug/ml	0.5957	0.06930	12	8.60	<.0001	0.05	0.4447	0.7467
BFU-E	APA5-0ug/ml	CFU-GM	APA5-0ug/ml	-1.2459	0.04688	12	-26.58	<.0001	0.05	-1.3480	-1.1438
BFU-E	APA5-0ug/ml	CFU-GM	APA5-20ug/ml	-0.5254	0.05211	12	-10.08	<.0001	0.05	-0.6389	-0.4119
BFU-E	APA5-0ug/ml	Mix	APA5-0ug/ml	2.2790	0.1355	12	16.81	<.0001	0.05	1.9836	2.5743
BFU-E	APA5-0ug/ml	Mix	APA5-20ug/ml	3.2823	0.2172	12	15.11	<.0001	0.05	2.8091	3.7554
BFU-E	APA5-20ug/ml	CFU-GEMM	APA5-0ug/ml	-0.4195	0.07639	12	-5.49	0.0001	0.05	-0.5859	-0.2530
BFU-E	APA5-20ug/ml	CFU-GEMM	APA5-20ug/ml	-0.1287	0.08135	12	-1.58	0.1397	0.05	-0.3059	0.04856
BFU-E	APA5-20ug/ml	CFU-GM	APA5-0ug/ml	-1.9703	0.06334	12	-31.11	<.0001	0.05	-2.1083	-1.8323
BFU-E	APA5-20ug/ml	CFU-GM	APA5-20ug/ml	-1.2497	0.06731	12	-18.57	<.0001	0.05	-1.3964	-1.1031
BFU-E	APA5-20ug/ml	Mix	APA5-0ug/ml	1.5546	0.1421	12	10.94	<.0001	0.05	1.2451	1.8642

The GLIMMIX Procedure

Differences of Colony_Typ*Condition Least Squares Means											
Colony_Type	Condition	_Colony_Type	_Condition	Estimate	Standard Error	DF	t Value	Pr > |t|	Alpha	Lower	Upper
BFU-E	APA5-20ug/ml	Mix	APA5-20ug/ml	2.5579	0.2213	12	11.56	<.0001	0.05	2.0758	3.0401
CFU-GEMM	APA5-0ug/ml	CFU-GEMM	APA5-20ug/ml	0.2908	0.07356	12	3.95	0.0019	0.05	0.1305	0.4510
CFU-GEMM	APA5-0ug/ml	CFU-GM	APA5-0ug/ml	-1.5508	0.05297	12	-29.28	<.0001	0.05	-1.6662	-1.4354
CFU-GEMM	APA5-0ug/ml	CFU-GM	APA5-20ug/ml	-0.8303	0.05765	12	-14.40	<.0001	0.05	-0.9559	-0.7047
CFU-GEMM	APA5-0ug/ml	Mix	APA5-0ug/ml	1.9741	0.1378	12	14.33	<.0001	0.05	1.6739	2.2743
CFU-GEMM	APA5-0ug/ml	Mix	APA5-20ug/ml	2.9774	0.2186	12	13.62	<.0001	0.05	2.5012	3.4536
CFU-GEMM	APA5-20ug/ml	CFU-GM	APA5-0ug/ml	-1.8416	0.05989	12	-30.75	<.0001	0.05	-1.9721	-1.7111
CFU-GEMM	APA5-20ug/ml	CFU-GM	APA5-20ug/ml	-1.1211	0.06407	12	-17.50	<.0001	0.05	-1.2607	-0.9815
CFU-GEMM	APA5-20ug/ml	Mix	APA5-0ug/ml	1.6833	0.1406	12	11.97	<.0001	0.05	1.3770	1.9896
CFU-GEMM	APA5-20ug/ml	Mix	APA5-20ug/ml	2.6866	0.2203	12	12.19	<.0001	0.05	2.2065	3.1667
CFU-GM	APA5-0ug/ml	CFU-GM	APA5-20ug/ml	0.7205	0.03873	12	18.60	<.0001	0.05	0.6361	0.8049
CFU-GM	APA5-0ug/ml	Mix	APA5-0ug/ml	3.5249	0.1310	12	26.91	<.0001	0.05	3.2395	3.8103
CFU-GM	APA5-0ug/ml	Mix	APA5-20ug/ml	4.5282	0.2143	12	21.13	<.0001	0.05	4.0612	4.9952
CFU-GM	APA5-20ug/ml	Mix	APA5-0ug/ml	2.8044	0.1329	12	21.09	<.0001	0.05	2.5147	3.0940
CFU-GM	APA5-20ug/ml	Mix	APA5-20ug/ml	3.8077	0.2156	12	17.66	<.0001	0.05	3.3380	4.2773
Mix	APA5-0ug/ml	Mix	APA5-20ug/ml	1.0033	0.2492	12	4.03	0.0017	0.05	0.4603	1.5464

The GLIMMIX Procedure

Model Information	
**Data Set**	WORK.FIG7CV
**Response Variable**	Count
**Response Distribution**	Poisson
**Link Function**	Log
**Variance Function**	Default
**Variance Matrix Blocked By**	Dish_ID
**Estimation Technique**	Maximum Likelihood
**Likelihood Approximation**	Laplace
**Degrees of Freedom Method**	Containment

Class Level Information		
**Class**	**Levels**	**Values**
**Colony_Size**	3	Large Micro Small
**Condition**	2	APA5 (0ug/ml) APA5 (20ug/ml
**Dish_ID**	3	1 2 3

**Number of Observations Read**	48
**Number of Observations Used**	48

Dimensions	
**G-side Cov. Parameters**	1
**Columns in X**	12
**Columns in Z per Subject**	1
**Subjects (Blocks in V)**	3
**Max Obs per Subject**	18

The GLIMMIX Procedure

Optimization Information	
**Optimization Technique**	Dual Quasi-Newton
**Parameters in Optimization**	7
**Lower Boundaries**	1
**Upper Boundaries**	0
**Fixed Effects**	Not Profiled
**Starting From**	GLM estimates

Iteration History					
**Iteration**	**Restarts**	**Evaluations**	**Objective Function**	**Change**	**Max Gradient**
**0**	**0**	4	327.65142551	.	1651.319
**1**	**0**	3	327.48635026	0.16507525	0.000039
**2**	**0**	1	327.48635026	-0.00000000	0.000039

Convergence criterion (GCONV=1E-8) satisfied.

Estimated G matrix is not positive definite.

Fit Statistics	
**-2 Log Likelihood**	327.49
**AIC (smaller is better)**	339.49
**AICC (smaller is better)**	341.54
**BIC (smaller is better)**	334.08
**CAIC (smaller is better)**	340.08
**HQIC (smaller is better)**	328.61

The GLIMMIX Procedure

Fit Statistics for Conditional Distribution	
-2 log L(Count | r. effects)	327.49
Pearson Chi-Square	70.70
Pearson Chi-Square / DF	1.47

Covariance Parameter Estimates			
Cov Parm	Subject	Estimate	Standard Error
Intercept	Dish_ID	0	.

Solutions for Fixed Effects							
Effect	Colony_Size	Condition	Estimate	Standard Error	DF	t Value	Pr > |t|
Intercept			3.1570	0.07293	2	43.29	0.0005
Colony_Size	Large		-0.3923	0.1149	40	-3.42	0.0015
Colony_Size	Micro		0.8571	0.08704	40	9.85	<.0001
Colony_Size	Small		0	.	.	.	.
Condition		APA5 (0ug/ml)	0.5807	0.09108	40	6.38	<.0001
Condition		APA5 (20ug/ml	0	.	.	.	.
Colony_Siz*Condition	Large	APA5 (0ug/ml)	0.1203	0.1417	40	0.85	0.4008
Colony_Siz*Condition	Large	APA5 (20ug/ml	0	.	.	.	.
Colony_Siz*Condition	Micro	APA5 (0ug/ml)	-0.4359	0.1118	40	-3.90	0.0004
Colony_Siz*Condition	Micro	APA5 (20ug/ml	0	.	.	.	.
Colony_Siz*Condition	Small	APA5 (0ug/ml)	0	.	.	.	.
Colony_Siz*Condition	Small	APA5 (20ug/ml	0	.	.	.	.

The GLIMMIX Procedure

Type III Tests of Fixed Effects				
Effect	Num DF	Den DF	F Value	Pr > F
Colony_Size	2	40	143.79	<.0001
Condition	1	40	83.78	<.0001
Colony_Siz*Condition	2	40	13.35	<.0001

Condition Least Squares Means												
Condition	Estimate	Standard Error	DF	t Value	Pr > |t|	Alpha	Lower	Upper	Mean	Standard Error Mean	Lower Mean	Upper Mean
APA5 (0ug/ml)	3.7874	0.03133	40	120.88	<.0001	0.05	3.7241	3.8508	44.1428	1.3831	41.4341	47.0285
APA5 (20ug/ml	3.3120	0.04143	40	79.94	<.0001	0.05	3.2282	3.3957	27.4388	1.1369	25.2347	29.8355

Differences of Condition Least Squares Means									
Condition	_Condition	Estimate	Standard Error	DF	t Value	Pr > |t|	Alpha	Lower	Upper
APA5 (0ug/ml)	APA5 (20ug/ml	0.4755	0.05195	40	9.15	<.0001	0.05	0.3705	0.5805

Colony_Siz*Condition Least Squares Means													
Colony_Size	Condition	Estimate	Standard Error	DF	t Value	Pr > |t|	Alpha	Lower	Upper	Mean	Standard Error Mean	Lower Mean	Upper Mean
Large	APA5 (0ug/ml)	3.4657	0.06250	40	55.45	<.0001	0.05	3.3394	3.5921	32.0000	2.0000	28.2027	36.3085
Large	APA5 (20ug/ml	2.7647	0.08874	40	31.16	<.0001	0.05	2.5854	2.9441	15.8750	1.4087	13.2687	18.9933
Micro	APA5 (0ug/ml)	4.1589	0.04419	40	94.10	<.0001	0.05	4.0696	4.2482	64.0000	2.8284	58.5314	69.9795
Micro	APA5 (20ug/ml	4.0141	0.04751	40	84.49	<.0001	0.05	3.9181	4.1102	55.3750	2.6309	50.3050	60.9560
Small	APA5 (0ug/ml)	3.7377	0.05455	40	68.51	<.0001	0.05	3.6274	3.8479	42.0000	2.2913	37.6153	46.8958
Small	APA5 (20ug/ml	3.1570	0.07293	40	43.29	<.0001	0.05	3.0096	3.3044	23.5000	1.7139	20.2793	27.2322

The GLIMMIX Procedure

Differences of Colony_Siz*Condition Least Squares Means											
Colony_Size	Condition	_Colony_Size	_Condition	Estimate	Standard Error	DF	t Value	Pr > |t|	Alpha	Lower	Upper
Large	APA5 (0ug/ml)	Large	APA5 (20ug/ml	0.7010	0.1085	40	6.46	<.0001	0.05	0.4816	0.9204
Large	APA5 (0ug/ml)	Micro	APA5 (0ug/ml)	-0.6931	0.07655	40	-9.06	<.0001	0.05	-0.8479	-0.5384
Large	APA5 (0ug/ml)	Micro	APA5 (20ug/ml	-0.5484	0.07851	40	-6.99	<.0001	0.05	-0.7071	-0.3897
Large	APA5 (0ug/ml)	Small	APA5 (0ug/ml)	-0.2719	0.08296	40	-3.28	0.0022	0.05	-0.4396	-0.1043
Large	APA5 (0ug/ml)	Small	APA5 (20ug/ml	0.3087	0.09605	40	3.21	0.0026	0.05	0.1146	0.5029
Large	APA5 (20ug/ml	Micro	APA5 (0ug/ml)	-1.3941	0.09913	40	-14.06	<.0001	0.05	-1.5945	-1.1938
Large	APA5 (20ug/ml	Micro	APA5 (20ug/ml	-1.2494	0.1007	40	-12.41	<.0001	0.05	-1.4528	-1.0460
Large	APA5 (20ug/ml	Small	APA5 (0ug/ml)	-0.9729	0.1042	40	-9.34	<.0001	0.05	-1.1834	-0.7624
Large	APA5 (20ug/ml	Small	APA5 (20ug/ml	-0.3923	0.1149	40	-3.42	0.0015	0.05	-0.6244	-0.1601
Micro	APA5 (0ug/ml)	Micro	APA5 (20ug/ml	0.1448	0.06489	40	2.23	0.0314	0.05	0.01361	0.2759
Micro	APA5 (0ug/ml)	Small	APA5 (0ug/ml)	0.4212	0.07021	40	6.00	<.0001	0.05	0.2793	0.5631
Micro	APA5 (0ug/ml)	Small	APA5 (20ug/ml	1.0019	0.08528	40	11.75	<.0001	0.05	0.8295	1.1742
Micro	APA5 (20ug/ml	Small	APA5 (0ug/ml)	0.2765	0.07234	40	3.82	0.0005	0.05	0.1302	0.4227
Micro	APA5 (20ug/ml	Small	APA5 (20ug/ml	0.8571	0.08704	40	9.85	<.0001	0.05	0.6812	1.0330
Small	APA5 (0ug/ml)	Small	APA5 (20ug/ml	0.5807	0.09108	40	6.38	<.0001	0.05	0.3966	0.7647

The GLIMMIX Procedure

Model Information	
Data Set	WORK.EXT_FIG1BII
Response Variable	Count
Response Distribution	Poisson
Link Function	Log
Variance Function	Default
Variance Matrix Blocked By	Dish_ID
Estimation Technique	Maximum Likelihood
Likelihood Approximation	Laplace
Degrees of Freedom Method	Containment

Class Level Information		
Class	Levels	Values
Colony_Size	3	Large Micro Small
Condition	5	E10. E11. E12. E13. E9.5
Dish_ID	15	1 2 3 4 5 6 7 8 9 10 11 12 13 14 15

Number of Observations Read	120
Number of Observations Used	120

Dimensions	
G-side Cov. Parameters	1
Columns in X	24
Columns in Z per Subject	1
Subjects (Blocks in V)	15
Max Obs per Subject	9

The GLIMMIX Procedure

Optimization Information	
Optimization Technique	Dual Quasi-Newton
Parameters in Optimization	16
Lower Boundaries	1
Upper Boundaries	0
Fixed Effects	Not Profiled
Starting From	GLM estimates

Iteration History

Iteration	Restarts	Evaluations	Objective Function	Change	Max Gradient
0	0	4	753.47517781	.	1772.956
1	0	3	751.09460714	2.38057067	0.075677
2	0	6	751.09460687	0.00000028	0.00503

Convergence criterion (GCONV=1E-8) satisfied.

Estimated G matrix is not positive definite.

Fit Statistics	
-2 Log Likelihood	751.09
AIC (smaller is better)	781.09
AICC (smaller is better)	785.71
BIC (smaller is better)	791.72
CAIC (smaller is better)	806.72
HQIC (smaller is better)	780.98

The GLIMMIX Procedure

Fit Statistics for Conditional Distribution	
-2 log L(Count | r. effects)	751.09
Pearson Chi-Square	109.30
Pearson Chi-Square / DF	0.91

Covariance Parameter Estimates			
Cov Parm	Subject	Estimate	Standard Error
Intercept	Dish_ID	0	.

Solutions for Fixed Effects							
Effect	Colony_Size	Condition	Estimate	Standard Error	DF	t Value	Pr > |t|
Intercept			2.1102	0.1231	10	17.14	<.0001
Colony_Size	Large		-0.4520	0.1974	95	-2.29	0.0242
Colony_Size	Micro		0.4738	0.1568	95	3.02	0.0032
Colony_Size	Small		0	.	.	.	.
Condition		E10.	1.2826	0.1391	95	9.22	<.0001
Condition		E11.	2.3280	0.1290	95	18.05	<.0001
Condition		E12.	1.5404	0.1356	95	11.36	<.0001
Condition		E13.	1.6393	0.1345	95	12.19	<.0001
Condition		E9.5	0	.	.	.	.
Colony_Siz*Condition	Large	E10.	0.5365	0.2168	95	2.47	0.0151
Colony_Siz*Condition	Large	E11.	-0.1177	0.2075	95	-0.57	0.5720
Colony_Siz*Condition	Large	E12.	0.4122	0.2135	95	1.93	0.0565
Colony_Siz*Condition	Large	E13.	0.08687	0.2148	95	0.40	0.6868
Colony_Siz*Condition	Large	E9.5	0	.	.	.	.

The GLIMMIX Procedure

Solutions for Fixed Effects							
Effect	Colony_Size	Condition	Estimate	Standard Error	DF	t Value	Pr > |t|
Colony_Siz*Condition	Micro	E10.	0.08943	0.1766	95	0.51	0.6137
Colony_Siz*Condition	Micro	E11.	-0.04160	0.1644	95	-0.25	0.8008
Colony_Siz*Condition	Micro	E12.	-0.05328	0.1731	95	-0.31	0.7589
Colony_Siz*Condition	Micro	E13.	-0.4001	0.1740	95	-2.30	0.0236
Colony_Siz*Condition	Micro	E9.5	0	.	.	.	.
Colony_Siz*Condition	Small	E10.	0	.	.	.	.
Colony_Siz*Condition	Small	E11.	0	.	.	.	.
Colony_Siz*Condition	Small	E12.	0	.	.	.	.
Colony_Siz*Condition	Small	E13.	0	.	.	.	.
Colony_Siz*Condition	Small	E9.5	0	.	.	.	.

Type III Tests of Fixed Effects				
Effect	Num DF	Den DF	F Value	Pr > F
Colony_Size	2	95	109.01	<.0001
Condition	4	95	291.70	<.0001
Colony_Siz*Condition	8	95	9.98	<.0001

Condition Least Squares Means												
Condition	Estimate	Standard Error	DF	t Value	Pr > |t|	Alpha	Lower	Upper	Mean	Standard Error Mean	Lower Mean	Upper Mean
E10.	3.6088	0.03408	95	105.88	<.0001	0.05	3.5411	3.6764	36.9200	1.2583	34.5045	39.5046
E11.	4.3924	0.02368	95	185.49	<.0001	0.05	4.3454	4.4394	80.8351	1.9142	77.1229	84.7260
E12.	3.7776	0.03119	95	121.10	<.0001	0.05	3.7157	3.8395	43.7101	1.3635	41.0854	46.5025

The GLIMMIX Procedure

Condition Least Squares Means												
Condition	Estimate	Standard Error	DF	t Value	Pr > |t|	Alpha	Lower	Upper	Mean	Standard Error Mean	Lower Mean	Upper Mean
E13.	3.6524	0.03318	95	110.06	<.0001	0.05	3.5865	3.7182	38.5657	1.2798	36.1069	41.1919
E9.5	2.1175	0.07333	95	28.88	<.0001	0.05	1.9719	2.2631	8.3102	0.6094	7.1843	9.6124

Differences of Condition Least Squares Means									
Condition	_Condition	Estimate	Standard Error	DF	t Value	Pr > |t|	Alpha	Lower	Upper
E10.	E11.	-0.7837	0.04150	95	-18.88	<.0001	0.05	-0.8660	-0.7013
E10.	E12.	-0.1688	0.04620	95	-3.65	0.0004	0.05	-0.2605	-0.07710
E10.	E13.	-0.04361	0.04757	95	-0.92	0.3616	0.05	-0.1380	0.05083
E10.	E9.5	1.4913	0.08086	95	18.44	<.0001	0.05	1.3307	1.6518
E11.	E12.	0.6148	0.03916	95	15.70	<.0001	0.05	0.5371	0.6926
E11.	E13.	0.7400	0.04077	95	18.15	<.0001	0.05	0.6591	0.8210
E11.	E9.5	2.2749	0.07706	95	29.52	<.0001	0.05	2.1220	2.4279
E12.	E13.	0.1252	0.04554	95	2.75	0.0071	0.05	0.03480	0.2156
E12.	E9.5	1.6601	0.07969	95	20.83	<.0001	0.05	1.5019	1.8183
E13.	E9.5	1.5349	0.08049	95	19.07	<.0001	0.05	1.3751	1.6947

Colony_Siz*Condition Least Squares Means													
Colony_Size	Condition	Estimate	Standard Error	DF	t Value	Pr > |t|	Alpha	Lower	Upper	Mean	Standard Error Mean	Lower Mean	Upper Mean
Large	E10.	3.4774	0.06214	95	55.96	<.0001	0.05	3.3540	3.6007	32.3751	2.0117	28.6179	36.6255
Large	E11.	3.8686	0.05110	95	75.71	<.0001	0.05	3.7672	3.9700	47.8750	2.4463	43.2567	52.9863
Large	E12.	3.6109	0.05812	95	62.12	<.0001	0.05	3.4955	3.7263	37.0000	2.1506	32.9677	41.5255
Large	E13.	3.3844	0.06509	95	51.99	<.0001	0.05	3.2552	3.5136	29.5000	1.9203	25.9238	33.5696

The GLIMMIX Procedure

Colony_Siz*Condition Least Squares Means

Colony_Size	Condition	Estimate	Standard Error	DF	t Value	Pr > |t|	Alpha	Lower	Upper	Mean	Standard Error Mean	Lower Mean	Upper Mean
Large	E9.5	1.6582	0.1543	95	10.75	<.0001	0.05	1.3519	1.9646	5.2500	0.8101	3.8648	7.1318
Micro	E10.	3.9560	0.04891	95	80.88	<.0001	0.05	3.8589	4.0531	52.2501	2.5556	47.4151	57.5782
Micro	E11.	4.8704	0.03096	95	157.29	<.0001	0.05	4.8089	4.9319	130.37	4.0369	122.60	138.64
Micro	E12.	4.0712	0.04618	95	88.17	<.0001	0.05	3.9795	4.1628	58.6250	2.7070	53.4898	64.2532
Micro	E13.	3.8232	0.05227	95	73.14	<.0001	0.05	3.7194	3.9270	45.7500	2.3914	41.2405	50.7526
Micro	E9.5	2.5840	0.09713	95	26.60	<.0001	0.05	2.3912	2.7768	13.2500	1.2870	10.9263	16.0679
Small	E10.	3.3928	0.06482	95	52.34	<.0001	0.05	3.2641	3.5215	29.7501	1.9284	26.1578	33.8357
Small	E11.	4.4382	0.03843	95	115.48	<.0001	0.05	4.3619	4.5145	84.6249	3.2524	78.4083	91.3344
Small	E12.	3.6507	0.05698	95	64.07	<.0001	0.05	3.5375	3.7638	38.5000	2.1937	34.3822	43.1110
Small	E13.	3.7495	0.05423	95	69.14	<.0001	0.05	3.6418	3.8572	42.5000	2.3049	38.1620	47.3312
Small	E9.5	2.1102	0.1231	95	17.14	<.0001	0.05	1.8658	2.3546	8.2500	1.0155	6.4614	10.5337

The GLIMMIX Procedure

Differences of Colony_Siz*Condition Least Squares Means											
Colony_Size	Condition	_Colony_Size	_Condition	Estimate	Standard Error	DF	t Value	Pr > |t|	Alpha	Lower	Upper
Large	E10.	Large	E11.	-0.3912	0.08045	95	-4.86	<.0001	0.05	-0.5509	-0.2315
Large	E10.	Large	E12.	-0.1335	0.08508	95	-1.57	0.1199	0.05	-0.3024	0.03538
Large	E10.	Large	E13.	0.09300	0.08999	95	1.03	0.3040	0.05	-0.08566	0.2717
Large	E10.	Large	E9.5	1.8192	0.1663	95	10.94	<.0001	0.05	1.4889	2.1494
Large	E10.	Micro	E10.	-0.4787	0.07908	95	-6.05	<.0001	0.05	-0.6356	-0.3217
Large	E10.	Micro	E11.	-1.3930	0.06942	95	-20.07	<.0001	0.05	-1.5308	-1.2552
Large	E10.	Micro	E12.	-0.5938	0.07742	95	-7.67	<.0001	0.05	-0.7475	-0.4401
Large	E10.	Micro	E13.	-0.3458	0.08120	95	-4.26	<.0001	0.05	-0.5070	-0.1846
Large	E10.	Micro	E9.5	0.8934	0.1153	95	7.75	<.0001	0.05	0.6645	1.1223
Large	E10.	Small	E10.	0.08456	0.08979	95	0.94	0.3487	0.05	-0.09370	0.2628
Large	E10.	Small	E11.	-0.9608	0.07306	95	-13.15	<.0001	0.05	-1.1059	-0.8158
Large	E10.	Small	E12.	-0.1733	0.08431	95	-2.06	0.0426	0.05	-0.3406	-0.00590
Large	E10.	Small	E13.	-0.2721	0.08248	95	-3.30	0.0014	0.05	-0.4358	-0.1084
Large	E10.	Small	E9.5	1.3672	0.1379	95	9.92	<.0001	0.05	1.0934	1.6409
Large	E11.	Large	E12.	0.2577	0.07739	95	3.33	0.0012	0.05	0.1040	0.4113
Large	E11.	Large	E13.	0.4842	0.08275	95	5.85	<.0001	0.05	0.3199	0.6485
Large	E11.	Large	E9.5	2.2104	0.1625	95	13.60	<.0001	0.05	1.8877	2.5331
Large	E11.	Micro	E10.	-0.08745	0.07073	95	-1.24	0.2194	0.05	-0.2279	0.05298
Large	E11.	Micro	E11.	-1.0018	0.05975	95	-16.77	<.0001	0.05	-1.1204	-0.8832
Large	E11.	Micro	E12.	-0.2026	0.06887	95	-2.94	0.0041	0.05	-0.3393	-0.06584
Large	E11.	Micro	E13.	0.04540	0.07310	95	0.62	0.5360	0.05	-0.09972	0.1905
Large	E11.	Micro	E9.5	1.2846	0.1097	95	11.70	<.0001	0.05	1.0667	1.5025
Large	E11.	Small	E10.	0.4758	0.08254	95	5.76	<.0001	0.05	0.3119	0.6396
Large	E11.	Small	E11.	-0.5696	0.06394	95	-8.91	<.0001	0.05	-0.6966	-0.4427

The GLIMMIX Procedure

Differences of Colony_Siz*Condition Least Squares Means											
Colony_Size	Condition	_Colony_Size	_Condition	Estimate	Standard Error	DF	t Value	Pr > |t|	Alpha	Lower	Upper
Large	E11.	Small	E12.	0.2179	0.07654	95	2.85	0.0054	0.05	0.06599	0.3699
Large	E11.	Small	E13.	0.1191	0.07451	95	1.60	0.1133	0.05	-0.02884	0.2670
Large	E11.	Small	E9.5	1.7584	0.1333	95	13.19	<.0001	0.05	1.4938	2.0230
Large	E12.	Large	E13.	0.2265	0.08727	95	2.60	0.0109	0.05	0.05328	0.3998
Large	E12.	Large	E9.5	1.9527	0.1649	95	11.84	<.0001	0.05	1.6253	2.2800
Large	E12.	Micro	E10.	-0.3451	0.07597	95	-4.54	<.0001	0.05	-0.4959	-0.1943
Large	E12.	Micro	E11.	-1.2595	0.06586	95	-19.12	<.0001	0.05	-1.3902	-1.1288
Large	E12.	Micro	E12.	-0.4602	0.07423	95	-6.20	<.0001	0.05	-0.6076	-0.3129
Large	E12.	Micro	E13.	-0.2123	0.07817	95	-2.72	0.0079	0.05	-0.3675	-0.05709
Large	E12.	Micro	E9.5	1.0269	0.1132	95	9.07	<.0001	0.05	0.8022	1.2516
Large	E12.	Small	E10.	0.2181	0.08706	95	2.50	0.0140	0.05	0.04525	0.3909
Large	E12.	Small	E11.	-0.8273	0.06968	95	-11.87	<.0001	0.05	-0.9656	-0.6890
Large	E12.	Small	E12.	-0.03974	0.08139	95	-0.49	0.6265	0.05	-0.2013	0.1218
Large	E12.	Small	E13.	-0.1386	0.07950	95	-1.74	0.0845	0.05	-0.2964	0.01923
Large	E12.	Small	E9.5	1.5007	0.1361	95	11.02	<.0001	0.05	1.2305	1.7709
Large	E13.	Large	E9.5	1.7262	0.1675	95	10.31	<.0001	0.05	1.3937	2.0586
Large	E13.	Micro	E10.	-0.5717	0.08142	95	-7.02	<.0001	0.05	-0.7333	-0.4100
Large	E13.	Micro	E11.	-1.4860	0.07208	95	-20.62	<.0001	0.05	-1.6291	-1.3429
Large	E13.	Micro	E12.	-0.6868	0.07981	95	-8.61	<.0001	0.05	-0.8452	-0.5283
Large	E13.	Micro	E13.	-0.4388	0.08348	95	-5.26	<.0001	0.05	-0.6045	-0.2731
Large	E13.	Micro	E9.5	0.8004	0.1169	95	6.85	<.0001	0.05	0.5683	1.0325
Large	E13.	Small	E10.	-0.00844	0.09186	95	-0.09	0.9270	0.05	-0.1908	0.1739
Large	E13.	Small	E11.	-1.0538	0.07559	95	-13.94	<.0001	0.05	-1.2039	-0.9038
Large	E13.	Small	E12.	-0.2663	0.08651	95	-3.08	0.0027	0.05	-0.4380	-0.09452

The GLIMMIX Procedure

Differences of Colony_Siz*Condition Least Squares Means

Colony_Size	Condition	_Colony_Size	_Condition	Estimate	Standard Error	DF	t Value	Pr > |t|	Alpha	Lower	Upper
Large	E13.	Small	E13.	-0.3651	0.08473	95	-4.31	<.0001	0.05	-0.5333	-0.1969
Large	E13.	Small	E9.5	1.2742	0.1392	95	9.15	<.0001	0.05	0.9977	1.5506
Large	E9.5	Micro	E10.	-2.2978	0.1619	95	-14.20	<.0001	0.05	-2.6192	-1.9765
Large	E9.5	Micro	E11.	-3.2122	0.1574	95	-20.41	<.0001	0.05	-3.5246	-2.8997
Large	E9.5	Micro	E12.	-2.4129	0.1611	95	-14.98	<.0001	0.05	-2.7327	-2.0932
Large	E9.5	Micro	E13.	-2.1650	0.1629	95	-13.29	<.0001	0.05	-2.4884	-1.8415
Large	E9.5	Micro	E9.5	-0.9258	0.1823	95	-5.08	<.0001	0.05	-1.2877	-0.5638
Large	E9.5	Small	E10.	-1.7346	0.1674	95	-10.36	<.0001	0.05	-2.0669	-1.4023
Large	E9.5	Small	E11.	-2.7800	0.1590	95	-17.48	<.0001	0.05	-3.0957	-2.4643
Large	E9.5	Small	E12.	-1.9924	0.1645	95	-12.11	<.0001	0.05	-2.3190	-1.6659
Large	E9.5	Small	E13.	-2.0913	0.1636	95	-12.79	<.0001	0.05	-2.4160	-1.7666
Large	E9.5	Small	E9.5	-0.4520	0.1974	95	-2.29	0.0242	0.05	-0.8438	-0.06013
Micro	E10.	Micro	E11.	-0.9144	0.05789	95	-15.80	<.0001	0.05	-1.0293	-0.7994
Micro	E10.	Micro	E12.	-0.1151	0.06726	95	-1.71	0.0903	0.05	-0.2487	0.01842
Micro	E10.	Micro	E13.	0.1329	0.07159	95	1.86	0.0666	0.05	-0.00927	0.2750
Micro	E10.	Micro	E9.5	1.3720	0.1087	95	12.62	<.0001	0.05	1.1562	1.5879
Micro	E10.	Small	E10.	0.5632	0.08120	95	6.94	<.0001	0.05	0.4020	0.7244
Micro	E10.	Small	E11.	-0.4822	0.06220	95	-7.75	<.0001	0.05	-0.6057	-0.3587
Micro	E10.	Small	E12.	0.3054	0.07509	95	4.07	<.0001	0.05	0.1563	0.4545
Micro	E10.	Small	E13.	0.2065	0.07303	95	2.83	0.0057	0.05	0.06155	0.3515
Micro	E10.	Small	E9.5	1.8458	0.1325	95	13.94	<.0001	0.05	1.5829	2.1088
Micro	E11.	Micro	E12.	0.7993	0.05560	95	14.38	<.0001	0.05	0.6889	0.9096
Micro	E11.	Micro	E13.	1.0472	0.06075	95	17.24	<.0001	0.05	0.9266	1.1678
Micro	E11.	Micro	E9.5	2.2864	0.1019	95	22.43	<.0001	0.05	2.0840	2.4888

The GLIMMIX Procedure

Differences of Colony_Siz*Condition Least Squares Means											
Colony_Size	Condition	_Colony_Size	_Condition	Estimate	Standard Error	DF	t Value	Pr > |t|	Alpha	Lower	Upper
Micro	E11.	Small	E10.	1.4776	0.07184	95	20.57	<.0001	0.05	1.3350	1.6202
Micro	E11.	Small	E11.	0.4322	0.04935	95	8.76	<.0001	0.05	0.3342	0.5302
Micro	E11.	Small	E12.	1.2198	0.06485	95	18.81	<.0001	0.05	1.0910	1.3485
Micro	E11.	Small	E13.	1.1209	0.06245	95	17.95	<.0001	0.05	0.9969	1.2449
Micro	E11.	Small	E9.5	2.7602	0.1269	95	21.75	<.0001	0.05	2.5082	3.0122
Micro	E12.	Micro	E13.	0.2480	0.06975	95	3.56	0.0006	0.05	0.1095	0.3864
Micro	E12.	Micro	E9.5	1.4872	0.1075	95	13.83	<.0001	0.05	1.2737	1.7007
Micro	E12.	Small	E10.	0.6783	0.07959	95	8.52	<.0001	0.05	0.5203	0.8363
Micro	E12.	Small	E11.	-0.3671	0.06008	95	-6.11	<.0001	0.05	-0.4863	-0.2478
Micro	E12.	Small	E12.	0.4205	0.07334	95	5.73	<.0001	0.05	0.2749	0.5661
Micro	E12.	Small	E13.	0.3217	0.07123	95	4.52	<.0001	0.05	0.1803	0.4631
Micro	E12.	Small	E9.5	1.9609	0.1315	95	14.92	<.0001	0.05	1.7000	2.2219
Micro	E13.	Micro	E9.5	1.2392	0.1103	95	11.23	<.0001	0.05	1.0202	1.4582
Micro	E13.	Small	E10.	0.4304	0.08327	95	5.17	<.0001	0.05	0.2650	0.5957
Micro	E13.	Small	E11.	-0.6150	0.06488	95	-9.48	<.0001	0.05	-0.7438	-0.4862
Micro	E13.	Small	E12.	0.1725	0.07732	95	2.23	0.0280	0.05	0.01903	0.3260
Micro	E13.	Small	E13.	0.07369	0.07532	95	0.98	0.3304	0.05	-0.07585	0.2232
Micro	E13.	Small	E9.5	1.7130	0.1337	95	12.81	<.0001	0.05	1.4475	1.9785
Micro	E9.5	Small	E10.	-0.8088	0.1168	95	-6.93	<.0001	0.05	-1.0407	-0.5770
Micro	E9.5	Small	E11.	-1.8542	0.1045	95	-17.75	<.0001	0.05	-2.0616	-1.6469
Micro	E9.5	Small	E12.	-1.0667	0.1126	95	-9.47	<.0001	0.05	-1.2902	-0.8431
Micro	E9.5	Small	E13.	-1.1655	0.1112	95	-10.48	<.0001	0.05	-1.3864	-0.9447
Micro	E9.5	Small	E9.5	0.4738	0.1568	95	3.02	0.0032	0.05	0.1625	0.7851
Small	E10.	Small	E11.	-1.0454	0.07536	95	-13.87	<.0001	0.05	-1.1950	-0.8958

The GLIMMIX Procedure

Differences of Colony_Siz*Condition Least Squares Means											
Colony_Size	Condition	_Colony_Size	_Condition	Estimate	Standard Error	DF	t Value	Pr > |t|	Alpha	Lower	Upper
Small	E10.	Small	E12.	-0.2578	0.08630	95	-2.99	0.0036	0.05	-0.4292	-0.08649
Small	E10.	Small	E13.	-0.3567	0.08452	95	-4.22	<.0001	0.05	-0.5245	-0.1889
Small	E10.	Small	E9.5	1.2826	0.1391	95	9.22	<.0001	0.05	1.0064	1.5588
Small	E11.	Small	E12.	0.7876	0.06873	95	11.46	<.0001	0.05	0.6511	0.9240
Small	E11.	Small	E13.	0.6887	0.06647	95	10.36	<.0001	0.05	0.5568	0.8207
Small	E11.	Small	E9.5	2.3280	0.1290	95	18.05	<.0001	0.05	2.0720	2.5840
Small	E12.	Small	E13.	-0.09885	0.07866	95	-1.26	0.2120	0.05	-0.2550	0.05732
Small	E12.	Small	E9.5	1.5404	0.1356	95	11.36	<.0001	0.05	1.2712	1.8097
Small	E13.	Small	E9.5	1.6393	0.1345	95	12.19	<.0001	0.05	1.3723	1.9063

The Mixed Procedure

Model Information	
**Data Set**	WORK.EXT_FIG2D
**Dependent Variable**	log_agg
**Covariance Structure**	Variance Components
**Subject Effect**	Replicate
**Estimation Method**	REML
**Residual Variance Method**	Profile
**Fixed Effects SE Method**	Model-Based
**Degrees of Freedom Method**	Containment

Class Level Information		
**Class**	**Levels**	**Values**
**Replicate**	9	1 2 3 4 5 6 10 11 12
**Group**	2	Pdgfra+/Nestin-GFP+/CD31-/Pdgfrb+ Pdgfra+/Nestin-GFP-/CD31-/Pdgfrb-

Dimensions	
**Covariance Parameters**	3
**Columns in X**	6
**Columns in Z per Subject**	2
**Subjects**	9
**Max Obs per Subject**	17

Number of Observations	
**Number of Observations Read**	153
**Number of Observations Used**	78
**Number of Observations Not Used**	75

The Mixed Procedure

Iteration History			
Iteration	Evaluations	-2 Res Log Like	Criterion
0	1	161.54199018	
1	3	161.52177058	0.00208677
2	2	161.50007695	0.00000829
3	1	161.49996998	0.00000000

Convergence criteria met.

Estimated G matrix is not positive definite.

Covariance Parameter Estimates		
Cov Parm	Subject	Estimate
Intercept	Replicate	0
Days	Replicate	4.724E-7
Residual		0.3512

Fit Statistics	
-2 Res Log Likelihood	161.5
AIC (Smaller is Better)	165.5
AICC (Smaller is Better)	165.7
BIC (Smaller is Better)	165.9

The Mixed Procedure

Solution for Fixed Effects						
**Effect**	**Group**	**Estimate**	**Standard Error**	**DF**	**t Value**	**Pr > |t|**
**Intercept**		3.0227	0.1718	7	17.59	<.0001
**Days**		0.1176	0.001855	7	63.42	<.0001
**Group**	Pdgfra+/Nestin-GFP+/CD31-/Pdgfrb+	0.7137	0.2467	60	2.89	0.0053
**Group**	Pdgfra+/Nestin-GFP-/CD31-/Pdgfrb-	0	.	.	.	.
**Days*Group**	Pdgfra+/Nestin-GFP+/CD31-/Pdgfrb+	-0.07303	0.004988	60	-14.64	<.0001
**Days*Group**	Pdgfra+/Nestin-GFP-/CD31-/Pdgfrb-	0	.	.	.	.

Type 3 Tests of Fixed Effects				
**Effect**	**Num DF**	**Den DF**	**F Value**	**Pr > F**
**Days**	1	7	1057.85	<.0001
**Group**	1	60	8.37	0.0053
**Days*Group**	1	60	214.36	<.0001

Estimates					
**Label**	**Estimate**	**Standard Error**	**DF**	**t Value**	**Pr > |t|**
**PLUS slope**	0.04460	0.004630	60	9.63	<.0001
**MINUS slope**	0.1176	0.001855	60	63.42	<.0001
**PLUS vs MINUS slope**	-0.07303	0.004988	60	-14.64	<.0001

The GLIMMIX Procedure

Model Information	
**Data Set**	WORK.EXT_FIG4AIV
**Response Variable**	Count
**Response Distribution**	Poisson
**Link Function**	Log
**Variance Function**	Default
**Variance Matrix Blocked By**	Dish_ID
**Estimation Technique**	Maximum Likelihood
**Likelihood Approximation**	Laplace
**Degrees of Freedom Method**	Containment

Class Level Information		
**Class**	**Levels**	**Values**
**Colony_Type**	4	BFU-E CFU-E CFU-GM Mix
**Group**	2	CreERT2 WT
**Dish_ID**	6	1 2 3 4 5 6

**Number of Observations Read**	24
**Number of Observations Used**	24

Dimensions	
**G-side Cov. Parameters**	1
**Columns in X**	15
**Columns in Z per Subject**	1
**Subjects (Blocks in V)**	6
**Max Obs per Subject**	4

The GLIMMIX Procedure

Optimization Information	
**Optimization Technique**	Dual Quasi-Newton
**Parameters in Optimization**	9
**Lower Boundaries**	1
**Upper Boundaries**	0
**Fixed Effects**	Not Profiled
**Starting From**	GLM estimates

Iteration History					
**Iteration**	**Restarts**	**Evaluations**	**Objective Function**	**Change**	**Max Gradient**
**0**	**0**	4	98.830658305	.	42.94872
**1**	**0**	5	98.587303936	0.24335437	6.733913
**2**	**0**	4	98.586162117	0.00114182	6.739685
**3**	**0**	2	98.585015352	0.00114676	3.898012
**4**	**0**	2	98.583833239	0.00118211	0.708305
**5**	**0**	3	98.583618313	0.00021493	1.247545
**6**	**0**	3	98.583481189	0.00013712	1.060891
**7**	**0**	4	98.583014892	0.00046630	1.76408
**8**	**0**	4	98.580237169	0.00277772	2.735397
**9**	**0**	4	98.555515314	0.02472186	1.438544
**10**	**0**	3	98.547120643	0.00839467	5.593176
**11**	**0**	3	98.544417311	0.00270333	0.84901
**12**	**0**	3	98.543910091	0.00050722	1.715795
**13**	**0**	2	98.543598172	0.00031192	1.874451
**14**	**0**	3	98.543381943	0.00021623	0.071001
**15**	**0**	2	98.543242917	0.00013903	0.077093

The GLIMMIX Procedure

Iteration History					
Iteration	Restarts	Evaluations	Objective Function	Change	Max Gradient
16	0	3	98.543240242	0.00000267	0.025353
17	0	2	98.543236178	0.00000406	0.011919
18	0	2	98.543234698	0.00000148	0.066051
19	0	3	98.543233918	0.00000078	0.000291
20	0	3	98.543233913	0.00000001	0.001214

Convergence criterion (GCONV=1E-8) satisfied.

Fit Statistics	
-2 Log Likelihood	98.54
AIC (smaller is better)	116.54
AICC (smaller is better)	129.40
BIC (smaller is better)	114.67
CAIC (smaller is better)	123.67
HQIC (smaller is better)	109.04

Fit Statistics for Conditional Distribution	
-2 log L(Count | r. effects)	95.01
Pearson Chi-Square	13.79
Pearson Chi-Square / DF	0.57

Covariance Parameter Estimates			
Cov Parm	Subject	Estimate	Standard Error
Intercept	Dish_ID	0.005814	0.01379

The GLIMMIX Procedure

Solutions for Fixed Effects							
Effect	Colony_Type	Group	Estimate	Standard Error	DF	t Value	Pr > |t|
Intercept			2.9931	0.1365	4	21.93	<.0001
Colony_Type	BFU-E		-0.8363	0.2348	12	-3.56	0.0039
Colony_Type	CFU-E		-22.2986	0	12	-Infty	<.0001
Colony_Type	CFU-GM		1.3987	0.1442	12	9.70	<.0001
Colony_Type	Mix		0	.	.	.	.
Group		CreERT2	-0.6292	0.2276	12	-2.76	0.0171
Group		WT	0	.	.	.	.
Colony_Type*Group	BFU-E	CreERT2	0.07856	0.3912	12	0.20	0.8442
Colony_Type*Group	BFU-E	WT	0	.	.	.	.
Colony_Type*Group	CFU-E	CreERT2	-6.1509	0	12	-Infty	<.0001
Colony_Type*Group	CFU-E	WT	0	.	.	.	.
Colony_Type*Group	CFU-GM	CreERT2	-1.2009	0.2787	12	-4.31	0.0010
Colony_Type*Group	CFU-GM	WT	0	.	.	.	.
Colony_Type*Group	Mix	CreERT2	0	.	.	.	.
Colony_Type*Group	Mix	WT	0	.	.	.	.

Type III Tests of Fixed Effects				
Effect	Num DF	Den DF	F Value	Pr > F
Colony_Type	2	12	43.34	<.0001
Group	1	12	246.47	<.0001
Colony_Type*Group	2	12	12.00	0.0014

The GLIMMIX Procedure

Group Least Squares Means

Group	Estimate	Standard Error	DF	t Value	Pr > |t|	Alpha	Lower	Upper	Mean	Standard Error Mean	Lower Mean	Upper Mean
CreERT2	-4.8884	0.1249	12	-39.15	<.0001	0.05	-5.1605	-4.6164	0.007533	0.000941	0.005739	0.009888
WT	-2.4409	0.09378	12	-26.03	<.0001	0.05	-2.6452	-2.2366	0.08708	0.008166	0.07099	0.1068

Differences of Group Least Squares Means

Group	_Group	Estimate	Standard Error	DF	t Value	Pr > |t|	Alpha	Lower	Upper
CreERT2	WT	-2.4475	0.1559	12	-15.70	<.0001	0.05	-2.7872	-2.1079

Colony_Type*Group Least Squares Means

Colony_Type	Group	Estimate	Standard Error	DF	t Value	Pr > |t|	Alpha	Lower	Upper	Mean	Standard Error Mean	Lower Mean	Upper Mean
BFU-E	CreERT2	1.6062	0.2621	12	6.13	<.0001	0.05	1.0352	2.1772	4.9839	1.3061	2.8158	8.8216
BFU-E	WT	2.1569	0.2011	12	10.73	<.0001	0.05	1.7188	2.5950	8.6441	1.7381	5.5778	13.3962
CFU-E	CreERT2	-26.0856	0.1824	12	-143.04	<.0001	0.05	-26.4829	-25.6883	4.69E-12	8.55E-13	3.15E-12	6.98E-12
CFU-E	WT	-19.3055	0.1365	12	-141.43	<.0001	0.05	-19.6029	-19.0080	4.128E-9	5.63E-10	3.066E-9	5.558E-9
CFU-GM	CreERT2	2.5617	0.1663	12	15.41	<.0001	0.05	2.1994	2.9240	12.9583	2.1547	9.0200	18.6162
CFU-GM	WT	4.3918	0.07799	12	56.31	<.0001	0.05	4.2219	4.5618	80.7895	6.3006	68.1647	95.7526
Mix	CreERT2	2.3639	0.1824	12	12.96	<.0001	0.05	1.9666	2.7613	10.6325	1.9390	7.1461	15.8197
Mix	WT	2.9931	0.1365	12	21.93	<.0001	0.05	2.6957	3.2905	19.9480	2.7230	14.8161	26.8575

The GLIMMIX Procedure

Differences of Colony_Type*Group Least Squares Means											
Colony_Type	Group	_Colony_Type	_Group	Estimate	Standard Error	DF	t Value	Pr > |t|	Alpha	Lower	Upper
BFU-E	CreERT2	BFU-E	WT	-0.5507	0.3302	12	-1.67	0.1212	0.05	-1.2701	0.1688
BFU-E	CreERT2	CFU-E	CreERT2	27.6918	0.3129	12	88.50	<.0001	0.05	27.0100	28.3736
BFU-E	CreERT2	CFU-E	WT	20.9117	0.2953	12	70.80	<.0001	0.05	20.2682	21.5552
BFU-E	CreERT2	CFU-GM	CreERT2	-0.9555	0.3038	12	-3.14	0.0085	0.05	-1.6175	-0.2935
BFU-E	CreERT2	CFU-GM	WT	-2.7856	0.2733	12	-10.19	<.0001	0.05	-3.3810	-2.1902
BFU-E	CreERT2	Mix	CreERT2	-0.7577	0.3129	12	-2.42	0.0322	0.05	-1.4395	-0.07590
BFU-E	CreERT2	Mix	WT	-1.3869	0.2953	12	-4.70	0.0005	0.05	-2.0304	-0.7434
BFU-E	WT	CFU-E	CreERT2	28.2425	0.2713	12	104.10	<.0001	0.05	27.6514	28.8336
BFU-E	WT	CFU-E	WT	21.4623	0.2348	12	91.41	<.0001	0.05	20.9508	21.9739
BFU-E	WT	CFU-GM	CreERT2	-0.4049	0.2608	12	-1.55	0.1465	0.05	-0.9730	0.1633
BFU-E	WT	CFU-GM	WT	-2.2350	0.2063	12	-10.83	<.0001	0.05	-2.6845	-1.7854
BFU-E	WT	Mix	CreERT2	-0.2070	0.2713	12	-0.76	0.4601	0.05	-0.7982	0.3841
BFU-E	WT	Mix	WT	-0.8363	0.2348	12	-3.56	0.0039	0.05	-1.3478	-0.3247
CFU-E	CreERT2	CFU-E	WT	-6.7801	0.2276	12	-29.79	<.0001	0.05	-7.2761	-6.2842
CFU-E	CreERT2	CFU-GM	CreERT2	-28.6473	0.2385	12	-120.11	<.0001	0.05	-29.1670	-28.1276
CFU-E	CreERT2	CFU-GM	WT	-30.4774	0.1981	12	-153.81	<.0001	0.05	-30.9092	-30.0457
CFU-E	CreERT2	Mix	CreERT2	-28.4495	0	12	-Infty	<.0001	.	.	.
CFU-E	CreERT2	Mix	WT	-29.0787	0.2276	12	-127.75	<.0001	0.05	-29.5747	-28.5828
CFU-E	WT	CFU-GM	CreERT2	-21.8672	0.2149	12	-101.73	<.0001	0.05	-22.3355	-21.3989
CFU-E	WT	CFU-GM	WT	-23.6973	0.1442	12	-164.38	<.0001	0.05	-24.0114	-23.3832
CFU-E	WT	Mix	CreERT2	-21.6694	0.2276	12	-95.20	<.0001	0.05	-22.1653	-21.1734
CFU-E	WT	Mix	WT	-22.2986	0	12	-Infty	<.0001	.	.	.
CFU-GM	CreERT2	CFU-GM	WT	-1.8301	0.1834	12	-9.98	<.0001	0.05	-2.2298	-1.4304
CFU-GM	CreERT2	Mix	CreERT2	0.1978	0.2385	12	0.83	0.4231	0.05	-0.3219	0.7175

The GLIMMIX Procedure

Differences of Colony_Type*Group Least Squares Means											
Colony_Type	Group	_Colony_Type	_Group	Estimate	Standard Error	DF	t Value	Pr > |t|	Alpha	Lower	Upper
CFU-GM	CreERT2	Mix	WT	-0.4314	0.2149	12	-2.01	0.0678	0.05	-0.8997	0.03694
CFU-GM	WT	Mix	CreERT2	2.0279	0.1981	12	10.23	<.0001	0.05	1.5962	2.4597
CFU-GM	WT	Mix	WT	1.3987	0.1442	12	9.70	<.0001	0.05	1.0846	1.7128
Mix	CreERT2	Mix	WT	-0.6292	0.2276	12	-2.76	0.0171	0.05	-1.1252	-0.1333

The GLIMMIX Procedure

Model Information	
**Data Set**	WORK.EXT_FIG4BII
**Response Variable**	Count
**Response Distribution**	Poisson
**Link Function**	Log
**Variance Function**	Default
**Variance Matrix Blocked By**	Dish_ID
**Estimation Technique**	Maximum Likelihood
**Likelihood Approximation**	Laplace
**Degrees of Freedom Method**	Containment

Class Level Information		
**Class**	**Levels**	**Values**
**Colony_Type**	4	BFU-E CFU-GM CFU-GMM Mix
**Group**	4	E10.5PDGFRA Het E10.5PDGFRA null E11.5PDGFRA Het E11.5PDGFRA null
**Dish_ID**	12	1 2 3 4 5 6 7 8 9 10 11 12

**Number of Observations Read**	48
**Number of Observations Used**	48

Dimensions	
**G-side Cov. Parameters**	1
**Columns in X**	25
**Columns in Z per Subject**	1
**Subjects (Blocks in V)**	12
**Max Obs per Subject**	4

The GLIMMIX Procedure

Optimization Information	
Optimization Technique	Dual Quasi-Newton
Parameters in Optimization	17
Lower Boundaries	1
Upper Boundaries	0
Fixed Effects	Not Profiled
Starting From	GLM estimates

Iteration History					
Iteration	Restarts	Evaluations	Objective Function	Change	Max Gradient
0	0	4	374.70040136	.	122.2793
1	0	6	374.67012691	0.03027445	65.32471
2	0	2	374.64778502	0.02234189	14.1855
3	0	2	374.6458932	0.00189183	7.370017
4	0	4	374.64114956	0.00474364	27.78919
5	0	4	374.63439123	0.00675833	2.203578
6	0	2	374.6340439	0.00034733	4.068261
7	0	4	374.6329273	0.00111661	1.379649
8	0	3	374.63219557	0.00073172	1.264456
9	0	4	374.63070645	0.00148912	3.033997
10	0	3	374.63052374	0.00018271	0.477147
11	0	4	374.6304037	0.00012004	0.668686
12	0	3	374.63032875	0.00007494	0.501326
13	0	2	374.63023073	0.00009803	0.537945
14	0	2	374.63007192	0.00015881	1.19656
15	0	3	374.63000585	0.00006607	0.445997

The GLIMMIX Procedure

Iteration History					
Iteration	Restarts	Evaluations	Objective Function	Change	Max Gradient
**16**	**0**	3	374.62998019	0.00002566	1.452903
**17**	**0**	6	374.62930542	0.00067477	0.4723
**18**	**0**	2	374.62928669	0.00001873	1.577695
**19**	**0**	4	374.62919954	0.00008715	0.356469
**20**	**0**	2	374.62904731	0.00015223	0.274271
**21**	**0**	3	374.62901021	0.00003710	0.187
**22**	**0**	3	374.62899997	0.00001024	0.060681
**23**	**0**	6	374.62897297	0.00002700	0.813385

Convergence criterion (GCONV=1E-8) satisfied.

Fit Statistics	
**-2 Log Likelihood**	374.63
**AIC (smaller is better)**	408.63
**AICC (smaller is better)**	429.03
**BIC (smaller is better)**	416.87
**CAIC (smaller is better)**	433.87
**HQIC (smaller is better)**	405.58

Fit Statistics for Conditional Distribution	
**-2 log L(Count | r. effects)**	349.19
**Pearson Chi-Square**	86.78
**Pearson Chi-Square / DF**	1.81

The GLIMMIX Procedure

Covariance Parameter Estimates			
Cov Parm	Subject	Estimate	Standard Error
Intercept	Dish_ID	0.007282	0.004235

Solutions for Fixed Effects							
Effect	Colony_Type	Group	Estimate	Standard Error	DF	t Value	Pr > |t|
Intercept			-5.6980	9.9456	8	-0.57	0.5824
Colony_Type	BFU-E		9.1158	9.9459	24	0.92	0.3685
Colony_Type	CFU-GM		10.3334	9.9455	24	1.04	0.3092
Colony_Type	CFU-GMM		9.6111	9.9457	24	0.97	0.3435
Colony_Type	Mix		0	.	.	.	.
Group		E10.5PDGFRA Het	9.0938	9.9463	24	0.91	0.3697
Group		E10.5PDGFRA null	0.002203	14.0672	24	0.00	0.9999
Group		E11.5PDGFRA Het	8.7570	9.9465	24	0.88	0.3874
Group		E11.5PDGFRA null	0	.	.	.	.
Colony_Type*Group	BFU-E	E10.5PDGFRA Het	-7.4738	9.9466	24	-0.75	0.4597
Colony_Type*Group	BFU-E	E10.5PDGFRA null	-1.0249	14.0684	24	-0.07	0.9425
Colony_Type*Group	BFU-E	E11.5PDGFRA Het	-7.0984	9.9468	24	-0.71	0.4823
Colony_Type*Group	BFU-E	E11.5PDGFRA null	0	.	.	.	.
Colony_Type*Group	CFU-GM	E10.5PDGFRA Het	-7.4249	9.9461	24	-0.75	0.4626
Colony_Type*Group	CFU-GM	E10.5PDGFRA null	-0.4655	14.0673	24	-0.03	0.9739
Colony_Type*Group	CFU-GM	E11.5PDGFRA Het	-6.8417	9.9463	24	-0.69	0.4981
Colony_Type*Group	CFU-GM	E11.5PDGFRA null	0	.	.	.	.
Colony_Type*Group	CFU-GMM	E10.5PDGFRA Het	-8.0152	9.9464	24	-0.81	0.4282
Colony_Type*Group	CFU-GMM	E10.5PDGFRA null	0.08341	14.0674	24	0.01	0.9953
Colony_Type*Group	CFU-GMM	E11.5PDGFRA Het	-7.5937	9.9466	24	-0.76	0.4526

The GLIMMIX Procedure

Solutions for Fixed Effects							
Effect	Colony_Type	Group	Estimate	Standard Error	DF	t Value	Pr > |t|
**Colony_Type*Group**	CFU-GMM	E11.5PDGFRA null	0	.	.	.	.
**Colony_Type*Group**	Mix	E10.5PDGFRA Het	0	.	.	.	.
**Colony_Type*Group**	Mix	E10.5PDGFRA null	0	.	.	.	.
**Colony_Type*Group**	Mix	E11.5PDGFRA Het	0	.	.	.	.
**Colony_Type*Group**	Mix	E11.5PDGFRA null	0	.	.	.	.

Type III Tests of Fixed Effects				
Effect	Num DF	Den DF	F Value	Pr > F
**Colony_Type**	3	24	298.50	<.0001
**Group**	3	24	1.36	0.2792
**Colony_Type*Group**	9	24	21.39	<.0001

Group Least Squares Means												
Group	Estimate	Standard Error	DF	t Value	Pr > |t|	Alpha	Lower	Upper	Mean	Standard Error Mean	Lower Mean	Upper Mean
E10.5PDGFRA Het	4.9325	0.05864	24	84.11	<.0001	0.05	4.8114	5.0535	138.72	8.1348	122.91	156.57
E10.5PDGFRA null	1.2176	2.4881	24	0.49	0.6290	0.05	-3.9175	6.3527	3.3789	8.4070	0.01989	574.02
E11.5PDGFRA Het	4.9406	0.06078	24	81.29	<.0001	0.05	4.8152	5.0661	139.86	8.5005	123.37	158.55
E11.5PDGFRA null	1.5671	2.4872	24	0.63	0.5346	0.05	-3.5663	6.7005	4.7927	11.9207	0.02826	812.83

The GLIMMIX Procedure

Differences of Group Least Squares Means									
Group	_Group	Estimate	Standard Error	DF	t Value	Pr > |t|	Alpha	Lower	Upper
E10.5PDGFRA Het	E10.5PDGFRA null	3.7149	2.4888	24	1.49	0.1486	0.05	-1.4216	8.8514
E10.5PDGFRA Het	E11.5PDGFRA Het	-0.00817	0.08446	24	-0.10	0.9238	0.05	-0.1825	0.1661
E10.5PDGFRA Het	E11.5PDGFRA null	3.3654	2.4879	24	1.35	0.1888	0.05	-1.7694	8.5001
E10.5PDGFRA null	E11.5PDGFRA Het	-3.7231	2.4888	24	-1.50	0.1477	0.05	-8.8597	1.4136
E10.5PDGFRA null	E11.5PDGFRA null	-0.3495	3.5180	24	-0.10	0.9217	0.05	-7.6104	6.9113
E11.5PDGFRA Het	E11.5PDGFRA null	3.3735	2.4880	24	1.36	0.1877	0.05	-1.7614	8.5084

Colony_Type*Group Least Squares Means													
Colony_Type	Group	Estimate	Standard Error	DF	t Value	Pr > |t|	Alpha	Lower	Upper	Mean	Standard Error Mean	Lower Mean	Upper Mean
BFU-E	E10.5PDGFRA Het	5.0379	0.06769	24	74.43	<.0001	0.05	4.8982	5.1776	154.14	10.4339	134.04	177.25
BFU-E	E10.5PDGFRA null	2.3951	0.1810	24	13.24	<.0001	0.05	2.0216	2.7686	10.9692	1.9850	7.5504	15.9359
BFU-E	E11.5PDGFRA Het	5.0764	0.06713	24	75.62	<.0001	0.05	4.9379	5.2150	160.20	10.7543	139.47	184.01
BFU-E	E11.5PDGFRA null	3.4178	0.1154	24	29.63	<.0001	0.05	3.1797	3.6559	30.5029	3.5190	24.0399	38.7034
CFU-GM	E10.5PDGFRA Het	6.3044	0.05511	24	114.39	<.0001	0.05	6.1906	6.4181	546.96	30.1450	488.15	612.85
CFU-GM	E10.5PDGFRA null	4.1722	0.08694	24	47.99	<.0001	0.05	3.9927	4.3516	64.8548	5.6386	54.2016	77.6019
CFU-GM	E11.5PDGFRA Het	6.5507	0.05388	24	121.57	<.0001	0.05	6.4395	6.6619	699.73	37.7043	626.08	782.04
CFU-GM	E11.5PDGFRA null	4.6354	0.07523	24	61.62	<.0001	0.05	4.4802	4.7907	103.07	7.7536	88.2497	120.38
CFU-GMM	E10.5PDGFRA Het	4.9917	0.06844	24	72.94	<.0001	0.05	4.8505	5.1330	147.19	10.0731	127.80	169.52
CFU-GMM	E10.5PDGFRA null	3.9987	0.09236	24	43.30	<.0001	0.05	3.8081	4.1894	54.5297	5.0362	45.0661	65.9807
CFU-GMM	E11.5PDGFRA Het	5.0764	0.06713	24	75.62	<.0001	0.05	4.9378	5.2149	160.19	10.7538	139.47	184.00
CFU-GMM	E11.5PDGFRA null	3.9131	0.09521	24	41.10	<.0001	0.05	3.7166	4.1096	50.0554	4.7658	41.1254	60.9243
Mix	E10.5PDGFRA Het	3.3959	0.1164	24	29.18	<.0001	0.05	3.1557	3.6360	29.8402	3.4723	23.4693	37.9405
Mix	E10.5PDGFRA null	-5.6958	9.9483	24	-0.57	0.5723	0.05	-26.2280	14.8365	0.003360	0.03343	4.07E-12	2775857

The GLIMMIX Procedure

Colony_Type*Group Least Squares Means													
Colony_Type	Group	Estimate	Standard Error	DF	t Value	Pr > |t|	Alpha	Lower	Upper	Mean	Standard Error Mean	Lower Mean	Upper Mean
Mix	E11.5PDGFRA Het	3.0590	0.1344	24	22.76	<.0001	0.05	2.7816	3.3364	21.3062	2.8632	16.1455	28.1164
Mix	E11.5PDGFRA null	-5.6980	9.9456	24	-0.57	0.5720	0.05	-26.2247	14.8288	0.003353	0.03335	4.08E-12	2754637

The GLIMMIX Procedure

Differences of Colony_Type*Group Least Squares Means											
Colony_Type	Group	_Colony_Type	_Group	Estimate	Standard Error	DF	t Value	Pr > |t|	Alpha	Lower	Upper
BFU-E	E10.5PDGFRA Het	BFU-E	E10.5PDGFRA null	2.6428	0.1932	24	13.68	<.0001	0.05	2.2440	3.0415
BFU-E	E10.5PDGFRA Het	BFU-E	E11.5PDGFRA Het	-0.03856	0.09533	24	-0.40	0.6895	0.05	-0.2353	0.1582
BFU-E	E10.5PDGFRA Het	BFU-E	E11.5PDGFRA null	1.6200	0.1337	24	12.11	<.0001	0.05	1.3440	1.8961
BFU-E	E10.5PDGFRA Het	CFU-GM	E10.5PDGFRA Het	-1.2665	0.05250	24	-24.12	<.0001	0.05	-1.3749	-1.1581
BFU-E	E10.5PDGFRA Het	CFU-GM	E10.5PDGFRA null	0.8657	0.1102	24	7.86	<.0001	0.05	0.6383	1.0931
BFU-E	E10.5PDGFRA Het	CFU-GM	E11.5PDGFRA Het	-1.5128	0.08652	24	-17.49	<.0001	0.05	-1.6914	-1.3343
BFU-E	E10.5PDGFRA Het	CFU-GM	E11.5PDGFRA null	0.4024	0.1012	24	3.98	0.0006	0.05	0.1936	0.6113
BFU-E	E10.5PDGFRA Het	CFU-GMM	E10.5PDGFRA Het	0.04615	0.06635	24	0.70	0.4934	0.05	-0.09079	0.1831
BFU-E	E10.5PDGFRA Het	CFU-GMM	E10.5PDGFRA null	1.0391	0.1145	24	9.08	<.0001	0.05	0.8028	1.2754
BFU-E	E10.5PDGFRA Het	CFU-GMM	E11.5PDGFRA Het	-0.03850	0.09533	24	-0.40	0.6899	0.05	-0.2353	0.1583
BFU-E	E10.5PDGFRA Het	CFU-GMM	E11.5PDGFRA null	1.1247	0.1168	24	9.63	<.0001	0.05	0.8837	1.3658
BFU-E	E10.5PDGFRA Het	Mix	E10.5PDGFRA Het	1.6420	0.1151	24	14.26	<.0001	0.05	1.4044	1.8797
BFU-E	E10.5PDGFRA Het	Mix	E10.5PDGFRA null	10.7336	9.9485	24	1.08	0.2913	0.05	-9.7991	31.2663
BFU-E	E10.5PDGFRA Het	Mix	E11.5PDGFRA Het	1.9789	0.1505	24	13.15	<.0001	0.05	1.6683	2.2894
BFU-E	E10.5PDGFRA Het	Mix	E11.5PDGFRA null	10.7358	9.9458	24	1.08	0.2911	0.05	-9.7913	31.2630
BFU-E	E10.5PDGFRA null	BFU-E	E11.5PDGFRA Het	-2.6813	0.1930	24	-13.89	<.0001	0.05	-3.0797	-2.2830
BFU-E	E10.5PDGFRA null	BFU-E	E11.5PDGFRA null	-1.0227	0.2146	24	-4.77	<.0001	0.05	-1.4656	-0.5798
BFU-E	E10.5PDGFRA null	CFU-GM	E10.5PDGFRA Het	-3.9093	0.1892	24	-20.67	<.0001	0.05	-4.2997	-3.5189
BFU-E	E10.5PDGFRA null	CFU-GM	E10.5PDGFRA null	-1.7771	0.1883	24	-9.44	<.0001	0.05	-2.1656	-1.3885
BFU-E	E10.5PDGFRA null	CFU-GM	E11.5PDGFRA Het	-4.1556	0.1888	24	-22.01	<.0001	0.05	-4.5453	-3.7659
BFU-E	E10.5PDGFRA null	CFU-GM	E11.5PDGFRA null	-2.2403	0.1960	24	-11.43	<.0001	0.05	-2.6448	-1.8359
BFU-E	E10.5PDGFRA null	CFU-GMM	E10.5PDGFRA Het	-2.5966	0.1935	24	-13.42	<.0001	0.05	-2.9959	-2.1973
BFU-E	E10.5PDGFRA null	CFU-GMM	E10.5PDGFRA null	-1.6037	0.1908	24	-8.40	<.0001	0.05	-1.9975	-1.2098
BFU-E	E10.5PDGFRA null	CFU-GMM	E11.5PDGFRA Het	-2.6813	0.1930	24	-13.89	<.0001	0.05	-3.0796	-2.2829

The GLIMMIX Procedure

Differences of Colony_Type*Group Least Squares Means											
Colony_Type	Group	_Colony_Type	_Group	Estimate	Standard Error	DF	t Value	Pr > |t|	Alpha	Lower	Upper
BFU-E	E10.5PDGFRA null	CFU-GMM	E11.5PDGFRA null	-1.5180	0.2045	24	-7.42	<.0001	0.05	-1.9400	-1.0960
BFU-E	E10.5PDGFRA null	Mix	E10.5PDGFRA Het	-1.0008	0.2151	24	-4.65	0.0001	0.05	-1.4448	-0.5567
BFU-E	E10.5PDGFRA null	Mix	E10.5PDGFRA null	8.0909	9.9497	24	0.81	0.4241	0.05	-12.4442	28.6259
BFU-E	E10.5PDGFRA null	Mix	E11.5PDGFRA Het	-0.6639	0.2254	24	-2.95	0.0071	0.05	-1.1291	-0.1987
BFU-E	E10.5PDGFRA null	Mix	E11.5PDGFRA null	8.0931	9.9471	24	0.81	0.4239	0.05	-12.4369	28.6230
BFU-E	E11.5PDGFRA Het	BFU-E	E11.5PDGFRA null	1.6586	0.1335	24	12.43	<.0001	0.05	1.3831	1.9341
BFU-E	E11.5PDGFRA Het	CFU-GM	E10.5PDGFRA Het	-1.2279	0.08686	24	-14.14	<.0001	0.05	-1.4072	-1.0487
BFU-E	E11.5PDGFRA Het	CFU-GM	E10.5PDGFRA null	0.9043	0.1098	24	8.23	<.0001	0.05	0.6776	1.1310
BFU-E	E11.5PDGFRA Het	CFU-GM	E11.5PDGFRA Het	-1.4743	0.05055	24	-29.17	<.0001	0.05	-1.5786	-1.3699
BFU-E	E11.5PDGFRA Het	CFU-GM	E11.5PDGFRA null	0.4410	0.1008	24	4.37	0.0002	0.05	0.2329	0.6491
BFU-E	E11.5PDGFRA Het	CFU-GMM	E10.5PDGFRA Het	0.08471	0.09586	24	0.88	0.3857	0.05	-0.1131	0.2826
BFU-E	E11.5PDGFRA Het	CFU-GMM	E10.5PDGFRA null	1.0777	0.1142	24	9.44	<.0001	0.05	0.8420	1.3133
BFU-E	E11.5PDGFRA Het	CFU-GMM	E11.5PDGFRA Het	0.000054	0.06448	24	0.00	0.9993	0.05	-0.1330	0.1331
BFU-E	E11.5PDGFRA Het	CFU-GMM	E11.5PDGFRA null	1.1633	0.1165	24	9.99	<.0001	0.05	0.9229	1.4037
BFU-E	E11.5PDGFRA Het	Mix	E10.5PDGFRA Het	1.6806	0.1343	24	12.51	<.0001	0.05	1.4033	1.9578
BFU-E	E11.5PDGFRA Het	Mix	E10.5PDGFRA null	10.7722	9.9485	24	1.08	0.2897	0.05	-9.7605	31.3049
BFU-E	E11.5PDGFRA Het	Mix	E11.5PDGFRA Het	2.0174	0.1331	24	15.16	<.0001	0.05	1.7428	2.2921
BFU-E	E11.5PDGFRA Het	Mix	E11.5PDGFRA null	10.7744	9.9458	24	1.08	0.2894	0.05	-9.7527	31.3015
BFU-E	E11.5PDGFRA null	CFU-GM	E10.5PDGFRA Het	-2.8866	0.1278	24	-22.58	<.0001	0.05	-3.1504	-2.6227
BFU-E	E11.5PDGFRA null	CFU-GM	E10.5PDGFRA null	-0.7543	0.1444	24	-5.22	<.0001	0.05	-1.0525	-0.4562
BFU-E	E11.5PDGFRA null	CFU-GM	E11.5PDGFRA Het	-3.1329	0.1273	24	-24.60	<.0001	0.05	-3.3957	-2.8701
BFU-E	E11.5PDGFRA null	CFU-GM	E11.5PDGFRA null	-1.2176	0.1187	24	-10.26	<.0001	0.05	-1.4625	-0.9727
BFU-E	E11.5PDGFRA null	CFU-GMM	E10.5PDGFRA Het	-1.5739	0.1341	24	-11.73	<.0001	0.05	-1.8507	-1.2971
BFU-E	E11.5PDGFRA null	CFU-GMM	E10.5PDGFRA null	-0.5809	0.1478	24	-3.93	0.0006	0.05	-0.8859	-0.2759

The GLIMMIX Procedure

Differences of Colony_Type*Group Least Squares Means											
Colony_Type	Group	_Colony_Type	_Group	Estimate	Standard Error	DF	t Value	Pr > |t|	Alpha	Lower	Upper
BFU-E	E11.5PDGFRA null	CFU-GMM	E11.5PDGFRA Het	-1.6586	0.1335	24	-12.43	<.0001	0.05	-1.9340	-1.3831
BFU-E	E11.5PDGFRA null	CFU-GMM	E11.5PDGFRA null	-0.4953	0.1322	24	-3.75	0.0010	0.05	-0.7682	-0.2224
BFU-E	E11.5PDGFRA null	Mix	E10.5PDGFRA Het	0.02197	0.1638	24	0.13	0.8945	0.05	-0.3162	0.3601
BFU-E	E11.5PDGFRA null	Mix	E10.5PDGFRA null	9.1136	9.9489	24	0.92	0.3688	0.05	-11.4200	29.6472
BFU-E	E11.5PDGFRA null	Mix	E11.5PDGFRA Het	0.3588	0.1771	24	2.03	0.0540	0.05	-0.00671	0.7244
BFU-E	E11.5PDGFRA null	Mix	E11.5PDGFRA null	9.1158	9.9459	24	0.92	0.3685	0.05	-11.4115	29.6430
CFU-GM	E10.5PDGFRA Het	CFU-GM	E10.5PDGFRA null	2.1322	0.1029	24	20.71	<.0001	0.05	1.9198	2.3447
CFU-GM	E10.5PDGFRA Het	CFU-GM	E11.5PDGFRA Het	-0.2463	0.07708	24	-3.20	0.0039	0.05	-0.4054	-0.08723
CFU-GM	E10.5PDGFRA Het	CFU-GM	E11.5PDGFRA null	1.6689	0.09323	24	17.90	<.0001	0.05	1.4765	1.8614
CFU-GM	E10.5PDGFRA Het	CFU-GMM	E10.5PDGFRA Het	1.3127	0.05346	24	24.55	<.0001	0.05	1.2023	1.4230
CFU-GM	E10.5PDGFRA Het	CFU-GMM	E10.5PDGFRA null	2.3056	0.1075	24	21.44	<.0001	0.05	2.0837	2.5276
CFU-GM	E10.5PDGFRA Het	CFU-GMM	E11.5PDGFRA Het	1.2280	0.08686	24	14.14	<.0001	0.05	1.0487	1.4073
CFU-GM	E10.5PDGFRA Het	CFU-GMM	E11.5PDGFRA null	2.3912	0.1100	24	21.74	<.0001	0.05	2.1642	2.6183
CFU-GM	E10.5PDGFRA Het	Mix	E10.5PDGFRA Het	2.9085	0.1082	24	26.87	<.0001	0.05	2.6851	3.1319
CFU-GM	E10.5PDGFRA Het	Mix	E10.5PDGFRA null	12.0001	9.9484	24	1.21	0.2395	0.05	-8.5324	32.5327
CFU-GM	E10.5PDGFRA Het	Mix	E11.5PDGFRA Het	3.2454	0.1452	24	22.34	<.0001	0.05	2.9456	3.5452
CFU-GM	E10.5PDGFRA Het	Mix	E11.5PDGFRA null	12.0023	9.9457	24	1.21	0.2393	0.05	-8.5247	32.5294
CFU-GM	E10.5PDGFRA null	CFU-GM	E11.5PDGFRA Het	-2.3785	0.1023	24	-23.25	<.0001	0.05	-2.5896	-2.1674
CFU-GM	E10.5PDGFRA null	CFU-GM	E11.5PDGFRA null	-0.4633	0.1150	24	-4.03	0.0005	0.05	-0.7005	-0.2260
CFU-GM	E10.5PDGFRA null	CFU-GMM	E10.5PDGFRA Het	-0.8196	0.1106	24	-7.41	<.0001	0.05	-1.0479	-0.5912
CFU-GM	E10.5PDGFRA null	CFU-GMM	E10.5PDGFRA null	0.1734	0.1060	24	1.64	0.1148	0.05	-0.04527	0.3921
CFU-GM	E10.5PDGFRA null	CFU-GMM	E11.5PDGFRA Het	-0.9042	0.1098	24	-8.23	<.0001	0.05	-1.1309	-0.6775
CFU-GM	E10.5PDGFRA null	CFU-GMM	E11.5PDGFRA null	0.2590	0.1289	24	2.01	0.0559	0.05	-0.00705	0.5251
CFU-GM	E10.5PDGFRA null	Mix	E10.5PDGFRA Het	0.7763	0.1453	24	5.34	<.0001	0.05	0.4765	1.0761

The GLIMMIX Procedure

Differences of Colony_Type*Group Least Squares Means											
Colony_Type	Group	_Colony_Type	_Group	Estimate	Standard Error	DF	t Value	Pr > |t|	Alpha	Lower	Upper
CFU-GM	E10.5PDGFRA null	Mix	E10.5PDGFRA null	9.8679	9.9484	24	0.99	0.3311	0.05	-10.6646	30.4004
CFU-GM	E10.5PDGFRA null	Mix	E11.5PDGFRA Het	1.1132	0.1601	24	6.95	<.0001	0.05	0.7828	1.4435
CFU-GM	E10.5PDGFRA null	Mix	E11.5PDGFRA null	9.8701	9.9459	24	0.99	0.3309	0.05	-10.6572	30.3975
CFU-GM	E11.5PDGFRA Het	CFU-GM	E11.5PDGFRA null	1.9153	0.09253	24	20.70	<.0001	0.05	1.7243	2.1062
CFU-GM	E11.5PDGFRA Het	CFU-GMM	E10.5PDGFRA Het	1.5590	0.08710	24	17.90	<.0001	0.05	1.3792	1.7387
CFU-GM	E11.5PDGFRA Het	CFU-GMM	E10.5PDGFRA null	2.5519	0.1069	24	23.87	<.0001	0.05	2.3313	2.7726
CFU-GM	E11.5PDGFRA Het	CFU-GMM	E11.5PDGFRA Het	1.4743	0.05055	24	29.17	<.0001	0.05	1.3700	1.5786
CFU-GM	E11.5PDGFRA Het	CFU-GMM	E11.5PDGFRA null	2.6376	0.1094	24	24.11	<.0001	0.05	2.4118	2.8633
CFU-GM	E11.5PDGFRA Het	Mix	E10.5PDGFRA Het	3.1548	0.1282	24	24.60	<.0001	0.05	2.8902	3.4195
CFU-GM	E11.5PDGFRA Het	Mix	E10.5PDGFRA null	12.2464	9.9484	24	1.23	0.2303	0.05	-8.2861	32.7790
CFU-GM	E11.5PDGFRA Het	Mix	E11.5PDGFRA Het	3.4917	0.1269	24	27.51	<.0001	0.05	3.2298	3.7536
CFU-GM	E11.5PDGFRA Het	Mix	E11.5PDGFRA null	12.2487	9.9458	24	1.23	0.2301	0.05	-8.2784	32.7757
CFU-GM	E11.5PDGFRA null	CFU-GMM	E10.5PDGFRA Het	-0.3563	0.1017	24	-3.50	0.0018	0.05	-0.5661	-0.1464
CFU-GM	E11.5PDGFRA null	CFU-GMM	E10.5PDGFRA null	0.6367	0.1191	24	5.35	<.0001	0.05	0.3909	0.8825
CFU-GM	E11.5PDGFRA null	CFU-GMM	E11.5PDGFRA Het	-0.4409	0.1008	24	-4.37	0.0002	0.05	-0.6490	-0.2329
CFU-GM	E11.5PDGFRA null	CFU-GMM	E11.5PDGFRA null	0.7223	0.09919	24	7.28	<.0001	0.05	0.5176	0.9270
CFU-GM	E11.5PDGFRA null	Mix	E10.5PDGFRA Het	1.2396	0.1385	24	8.95	<.0001	0.05	0.9536	1.5255
CFU-GM	E11.5PDGFRA null	Mix	E10.5PDGFRA null	10.3312	9.9486	24	1.04	0.3094	0.05	-10.2016	30.8640
CFU-GM	E11.5PDGFRA null	Mix	E11.5PDGFRA Het	1.5764	0.1540	24	10.24	<.0001	0.05	1.2586	1.8943
CFU-GM	E11.5PDGFRA null	Mix	E11.5PDGFRA null	10.3334	9.9455	24	1.04	0.3092	0.05	-10.1932	30.8600
CFU-GMM	E10.5PDGFRA Het	CFU-GMM	E10.5PDGFRA null	0.9930	0.1149	24	8.64	<.0001	0.05	0.7557	1.2302
CFU-GMM	E10.5PDGFRA Het	CFU-GMM	E11.5PDGFRA Het	-0.08465	0.09586	24	-0.88	0.3860	0.05	-0.2825	0.1132
CFU-GMM	E10.5PDGFRA Het	CFU-GMM	E11.5PDGFRA null	1.0786	0.1172	24	9.20	<.0001	0.05	0.8366	1.3206
CFU-GMM	E10.5PDGFRA Het	Mix	E10.5PDGFRA Het	1.5959	0.1156	24	13.81	<.0001	0.05	1.3573	1.8344

The GLIMMIX Procedure

Differences of Colony_Type*Group Least Squares Means											
Colony_Type	Group	_Colony_Type	_Group	Estimate	Standard Error	DF	t Value	Pr > |t|	Alpha	Lower	Upper
CFU-GMM	E10.5PDGFRA Het	Mix	E10.5PDGFRA null	10.6875	9.9485	24	1.07	0.2934	0.05	-9.8452	31.2202
CFU-GMM	E10.5PDGFRA Het	Mix	E11.5PDGFRA Het	1.9327	0.1508	24	12.82	<.0001	0.05	1.6215	2.2440
CFU-GMM	E10.5PDGFRA Het	Mix	E11.5PDGFRA null	10.6897	9.9458	24	1.07	0.2932	0.05	-9.8375	31.2168
CFU-GMM	E10.5PDGFRA null	CFU-GMM	E11.5PDGFRA Het	-1.0776	0.1142	24	-9.44	<.0001	0.05	-1.3133	-0.8420
CFU-GMM	E10.5PDGFRA null	CFU-GMM	E11.5PDGFRA null	0.08562	0.1326	24	0.65	0.5247	0.05	-0.1881	0.3594
CFU-GMM	E10.5PDGFRA null	Mix	E10.5PDGFRA Het	0.6029	0.1486	24	4.06	0.0005	0.05	0.2963	0.9095
CFU-GMM	E10.5PDGFRA null	Mix	E10.5PDGFRA null	9.6945	9.9485	24	0.97	0.3395	0.05	-10.8381	30.2271
CFU-GMM	E10.5PDGFRA null	Mix	E11.5PDGFRA Het	0.9397	0.1631	24	5.76	<.0001	0.05	0.6032	1.2763
CFU-GMM	E10.5PDGFRA null	Mix	E11.5PDGFRA null	9.6967	9.9460	24	0.97	0.3393	0.05	-10.8307	30.2242
CFU-GMM	E11.5PDGFRA Het	CFU-GMM	E11.5PDGFRA null	1.1632	0.1165	24	9.99	<.0001	0.05	0.9228	1.4037
CFU-GMM	E11.5PDGFRA Het	Mix	E10.5PDGFRA Het	1.6805	0.1343	24	12.51	<.0001	0.05	1.4033	1.9578
CFU-GMM	E11.5PDGFRA Het	Mix	E10.5PDGFRA null	10.7721	9.9485	24	1.08	0.2897	0.05	-9.7606	31.3048
CFU-GMM	E11.5PDGFRA Het	Mix	E11.5PDGFRA Het	2.0174	0.1331	24	15.16	<.0001	0.05	1.7427	2.2920
CFU-GMM	E11.5PDGFRA Het	Mix	E11.5PDGFRA null	10.7743	9.9458	24	1.08	0.2894	0.05	-9.7528	31.3015
CFU-GMM	E11.5PDGFRA null	Mix	E10.5PDGFRA Het	0.5173	0.1503	24	3.44	0.0021	0.05	0.2070	0.8276
CFU-GMM	E11.5PDGFRA null	Mix	E10.5PDGFRA null	9.6089	9.9487	24	0.97	0.3438	0.05	-10.9243	30.1421
CFU-GMM	E11.5PDGFRA null	Mix	E11.5PDGFRA Het	0.8541	0.1647	24	5.19	<.0001	0.05	0.5142	1.1940
CFU-GMM	E11.5PDGFRA null	Mix	E11.5PDGFRA null	9.6111	9.9457	24	0.97	0.3435	0.05	-10.9158	30.1379
Mix	E10.5PDGFRA Het	Mix	E10.5PDGFRA null	9.0916	9.9490	24	0.91	0.3699	0.05	-11.4420	29.6253
Mix	E10.5PDGFRA Het	Mix	E11.5PDGFRA Het	0.3369	0.1778	24	1.89	0.0702	0.05	-0.03002	0.7037
Mix	E10.5PDGFRA Het	Mix	E11.5PDGFRA null	9.0938	9.9463	24	0.91	0.3697	0.05	-11.4343	29.6219
Mix	E10.5PDGFRA null	Mix	E11.5PDGFRA Het	-8.7548	9.9492	24	-0.88	0.3876	0.05	-29.2889	11.7793
Mix	E10.5PDGFRA null	Mix	E11.5PDGFRA null	0.002203	14.0672	24	0.00	0.9999	0.05	-29.0310	29.0354
Mix	E11.5PDGFRA Het	Mix	E11.5PDGFRA null	8.7570	9.9465	24	0.88	0.3874	0.05	-11.7716	29.2856

The GLIMMIX Procedure

Model Information	
**Data Set**	WORK.EXT_FIG5C
**Response Variable**	Count
**Response Distribution**	Poisson
**Link Function**	Log
**Variance Function**	Default
**Variance Matrix Blocked By**	Dish_ID
**Estimation Technique**	Maximum Likelihood
**Likelihood Approximation**	Laplace
**Degrees of Freedom Method**	Containment

Class Level Information		
**Class**	**Levels**	**Values**
**Colony_Size**	3	Large Micro Small
**alpha**	2	minus plus
**Mesp1eYFP**	2	minus plus
**CD31**	1	minus
**Dish_ID**	12	1 2 3 4 5 6 7 8 9 10 11 12

**Number of Observations Read**	36
**Number of Observations Used**	36

Dimensions	
**G-side Cov. Parameters**	1
**Columns in X**	72
**Columns in Z per Subject**	1

The GLIMMIX Procedure

Dimensions	
**Subjects (Blocks in V)**	12
**Max Obs per Subject**	3

Optimization Information	
**Optimization Technique**	Dual Quasi-Newton
**Parameters in Optimization**	13
**Lower Boundaries**	1
**Upper Boundaries**	0
**Fixed Effects**	Not Profiled
**Starting From**	GLM estimates

Iteration History

Iteration	Restarts	Evaluations	Objective Function	Change	Max Gradient
**0**	**0**	4	137.91343529	.	255.8706
**1**	**0**	3	136.54322943	1.37020587	0.075613
**2**	**0**	8	136.543202	0.00002742	0.235075

Convergence criterion (GCONV=1E-8) satisfied.

Estimated G matrix is not positive definite.

Fit Statistics	
**-2 Log Likelihood**	136.54
**AIC (smaller is better)**	160.54
**AICC (smaller is better)**	174.11
**BIC (smaller is better)**	166.36

The GLIMMIX Procedure

Fit Statistics	
CAIC (smaller is better)	178.36
HQIC (smaller is better)	158.39

Fit Statistics for Conditional Distribution	
-2 log L(Count | r. effects)	136.54
Pearson Chi-Square	22.23
Pearson Chi-Square / DF	0.62

Covariance Parameter Estimates

Cov Parm	Subject	Estimate	Standard Error
Intercept	Dish_ID	0	.

The GLIMMIX Procedure

Solutions for Fixed Effects									
Effect	Colony_Size	alpha	Mesp1eYFP	CD31	Estimate	Standard Error	DF	t Value	Pr > |t|
Intercept					3.6108	0.09492	8	38.04	<.0001
Colony_Size	Large				-0.4056	0.1501	16	-2.70	0.0157
Colony_Size	Micro				0.5162	0.1199	16	4.30	0.0005
Colony_Size	Small				0	.	.	.	.
alpha		minus			-1.4140	0.2146	16	-6.59	<.0001
alpha		plus			0	.	.	.	.
Colony_Size*alpha	Large	minus			-7.4851	9.9536	16	-0.75	0.4630
Colony_Size*alpha	Large	plus			0	.	.	.	.
Colony_Size*alpha	Micro	minus			-0.4109	0.2912	16	-1.41	0.1773
Colony_Size*alpha	Micro	plus			0	.	.	.	.
Colony_Size*alpha	Small	minus			0	.	.	.	.
Colony_Size*alpha	Small	plus			0	.	.	.	.
Mesp1eYFP			minus		-0.1989	0.1414	16	-1.41	0.1789
Mesp1eYFP			plus		0	.	.	.	.
Colony_Siz*Mesp1eYFP	Large		minus		-1.6206	0.3419	16	-4.74	0.0002
Colony_Siz*Mesp1eYFP	Large		plus		0	.	.	.	.
Colony_Siz*Mesp1eYFP	Micro		minus		-0.6577	0.1950	16	-3.37	0.0039
Colony_Siz*Mesp1eYFP	Micro		plus		0	.	.	.	.
Colony_Siz*Mesp1eYFP	Small		minus		0	.	.	.	.
Colony_Siz*Mesp1eYFP	Small		plus		0	.	.	.	.
alpha*Mesp1eYFP		minus	minus		-7.6919	9.9541	16	-0.77	0.4509
alpha*Mesp1eYFP		minus	plus		0	.	.	.	.
alpha*Mesp1eYFP		plus	minus		0	.	.	.	.
alpha*Mesp1eYFP		plus	plus		0	.	.	.	.

The GLIMMIX Procedure

Solutions for Fixed Effects									
Effect	Colony_Size	alpha	Mesp1eYFP	CD31	Estimate	Standard Error	DF	t Value	Pr > |t|
Colony*alpha*Mesp1eY	Large	minus	minus		9.5108	17.2413	16	0.55	0.5888
Colony*alpha*Mesp1eY	Large	minus	plus		0	.	.	.	.
Colony*alpha*Mesp1eY	Large	plus	minus		0	.	.	.	.
Colony*alpha*Mesp1eY	Large	plus	plus		0	.	.	.	.
Colony*alpha*Mesp1eY	Micro	minus	minus		0.5522	14.0777	16	0.04	0.9692
Colony*alpha*Mesp1eY	Micro	minus	plus		0	.	.	.	.
Colony*alpha*Mesp1eY	Micro	plus	minus		0	.	.	.	.
Colony*alpha*Mesp1eY	Micro	plus	plus		0	.	.	.	.
Colony*alpha*Mesp1eY	Small	minus	minus		0	.	.	.	.
Colony*alpha*Mesp1eY	Small	minus	plus		0	.	.	.	.
Colony*alpha*Mesp1eY	Small	plus	minus		0	.	.	.	.
Colony*alpha*Mesp1eY	Small	plus	plus		0	.	.	.	.
CD31				minus	0	.	.	.	.
Colony_Size*CD31	Large			minus	0	.	.	.	.
Colony_Size*CD31	Micro			minus	0	.	.	.	.
Colony_Size*CD31	Small			minus	0	.	.	.	.
alpha*CD31		minus		minus	0	.	.	.	.
alpha*CD31		plus		minus	0	.	.	.	.
Colony_Si*alpha*CD31	Large	minus		minus	0	.	.	.	.
Colony_Si*alpha*CD31	Large	plus		minus	0	.	.	.	.
Colony_Si*alpha*CD31	Micro	minus		minus	0	.	.	.	.
Colony_Si*alpha*CD31	Micro	plus		minus	0	.	.	.	.
Colony_Si*alpha*CD31	Small	minus		minus	0	.	.	.	.
Colony_Si*alpha*CD31	Small	plus		minus	0	.	.	.	.

The GLIMMIX Procedure

Solutions for Fixed Effects									
Effect	Colony_Size	alpha	Mesp1eYFP	CD31	Estimate	Standard Error	DF	t Value	Pr > |t|
Mesp1eYFP*CD31			minus	minus	0	.	.	.	.
Mesp1eYFP*CD31			plus	minus	0	.	.	.	.
Colony_*Mesp1eY*CD31	Large		minus	minus	0	.	.	.	.
Colony_*Mesp1eY*CD31	Large		plus	minus	0	.	.	.	.
Colony_*Mesp1eY*CD31	Micro		minus	minus	0	.	.	.	.
Colony_*Mesp1eY*CD31	Micro		plus	minus	0	.	.	.	.
Colony_*Mesp1eY*CD31	Small		minus	minus	0	.	.	.	.
Colony_*Mesp1eY*CD31	Small		plus	minus	0	.	.	.	.
alpha*Mesp1eYFP*CD31		minus	minus	minus	0	.	.	.	.
alpha*Mesp1eYFP*CD31		minus	plus	minus	0	.	.	.	.
alpha*Mesp1eYFP*CD31		plus	minus	minus	0	.	.	.	.
alpha*Mesp1eYFP*CD31		plus	plus	minus	0	.	.	.	.
Colo*alph*Mesp1*CD31	Large	minus	minus	minus	0	.	.	.	.
Colo*alph*Mesp1*CD31	Large	minus	plus	minus	0	.	.	.	.
Colo*alph*Mesp1*CD31	Large	plus	minus	minus	0	.	.	.	.
Colo*alph*Mesp1*CD31	Large	plus	plus	minus	0	.	.	.	.
Colo*alph*Mesp1*CD31	Micro	minus	minus	minus	0	.	.	.	.
Colo*alph*Mesp1*CD31	Micro	minus	plus	minus	0	.	.	.	.
Colo*alph*Mesp1*CD31	Micro	plus	minus	minus	0	.	.	.	.
Colo*alph*Mesp1*CD31	Micro	plus	plus	minus	0	.	.	.	.
Colo*alph*Mesp1*CD31	Small	minus	minus	minus	0	.	.	.	.
Colo*alph*Mesp1*CD31	Small	minus	plus	minus	0	.	.	.	.
Colo*alph*Mesp1*CD31	Small	plus	minus	minus	0	.	.	.	.
Colo*alph*Mesp1*CD31	Small	plus	plus	minus	0	.	.	.	.

The GLIMMIX Procedure

Type III Tests of Fixed Effects				
**Effect**	**Num DF**	**Den DF**	**F Value**	**Pr > F**
**Colony_Size**	2	16	0.23	0.8004
**alpha**	1	16	3.51	0.0795
**Colony_Size*alpha**	2	16	0.06	0.9444
**Mesp1eYFP**	1	16	0.89	0.3600
**Colony_Siz*Mesp1eYFP**	2	16	0.09	0.9137
**alpha*Mesp1eYFP**	1	16	0.43	0.5226
**Colony*alpha*Mesp1eY**	2	16	0.17	0.8428
**CD31**	0	.	.	.
**Colony_Size*CD31**	0	.	.	.
**alpha*CD31**	0	.	.	.
**Colony_Si*alpha*CD31**	0	.	.	.
**Mesp1eYFP*CD31**	0	.	.	.
**Colony_*Mesp1eY*CD31**	0	.	.	.
**alpha*Mesp1eYFP*CD31**	0	.	.	.
**Colo*alph*Mesp1*CD31**	0	.	.	.

alpha*Mesp1eYFP*CD31 Least Squares Means														
**alpha**	**Mesp1eYFP**	**CD31**	**Estimate**	**Standard Error**	**DF**	**t Value**	**Pr > |t|**	**Alpha**	**Lower**	**Upper**	**Mean**	**Standard Error Mean**	**Lower Mean**	**Upper Mean**
minus	minus	minus	-5.6942	5.7459	16	-0.99	0.3364	0.05	-17.8751	6.4866	0.003365	0.01934	1.726E-8	656.28
minus	plus	minus	-0.3984	3.3180	16	-0.12	0.9059	0.05	-7.4323	6.6356	0.6714	2.2278	0.000592	761.72
plus	minus	minus	2.6894	0.1091	16	24.66	<.0001	0.05	2.4582	2.9206	14.7225	1.6055	11.6837	18.5517
plus	plus	minus	3.6476	0.05568	16	65.51	<.0001	0.05	3.5296	3.7657	38.3840	2.1373	34.1103	43.1931

The GLIMMIX Procedure

Differences of alpha*Mesp1eYFP*CD31 Least Squares Means													
alpha	Mesp1eYFP	CD31	_alpha	_Mesp1eYFP	_CD31	Estimate	Standard Error	DF	t Value	Pr > |t|	Alpha	Lower	Upper
minus	minus	minus	minus	plus	minus	-5.2959	6.6351	16	-0.80	0.4365	0.05	-19.3617	8.7700
minus	minus	minus	plus	minus	minus	-8.3836	5.7470	16	-1.46	0.1640	0.05	-20.5666	3.7994
minus	minus	minus	plus	plus	minus	-9.3419	5.7462	16	-1.63	0.1235	0.05	-21.5233	2.8395
minus	plus	minus	plus	minus	minus	-3.0877	3.3198	16	-0.93	0.3661	0.05	-10.1255	3.9500
minus	plus	minus	plus	plus	minus	-4.0460	3.3185	16	-1.22	0.2404	0.05	-11.0809	2.9889
plus	minus	minus	plus	plus	minus	-0.9583	0.1224	16	-7.83	<.0001	0.05	-1.2178	-0.6987

Colo*alph*Mesp1*CD31 Least Squares Means															
Colony_Size	alpha	Mesp1eYFP	CD31	Estimate	Standard Error	DF	t Value	Pr > |t|	Alpha	Lower	Upper	Mean	Standard Error Mean	Lower Mean	Upper Mean
Large	minus	minus	minus	-5.6945	9.9534	16	-0.57	0.5752	0.05	-26.7948	15.4058	0.003365	0.03349	2.31E-12	4905231
Large	minus	plus	minus	-5.6939	9.9506	16	-0.57	0.5751	0.05	-26.7882	15.4004	0.003366	0.03350	2.32E-12	4878774
Large	plus	minus	minus	1.3857	0.2888	16	4.80	0.0002	0.05	0.7736	1.9979	3.9978	1.1544	2.1676	7.3734
Large	plus	plus	minus	3.2052	0.1163	16	27.57	<.0001	0.05	2.9587	3.4516	24.6594	2.8670	19.2727	31.5518
Micro	minus	minus	minus	-5.6942	9.9522	16	-0.57	0.5752	0.05	-26.7919	15.4035	0.003365	0.03349	2.31E-12	4893925
Micro	minus	plus	minus	2.3021	0.1826	16	12.61	<.0001	0.05	1.9149	2.6892	9.9949	1.8253	6.7865	14.7201
Micro	plus	minus	minus	3.2705	0.1125	16	29.06	<.0001	0.05	3.0319	3.5090	26.3237	2.9622	20.7370	33.4155
Micro	plus	plus	minus	4.1270	0.07333	16	56.28	<.0001	0.05	3.9715	4.2824	61.9915	4.5457	53.0666	72.4174
Small	minus	minus	minus	-5.6940	9.9512	16	-0.57	0.5751	0.05	-26.7896	15.4016	0.003366	0.03350	2.32E-12	4884389
Small	minus	plus	minus	2.1968	0.1925	16	11.41	<.0001	0.05	1.7887	2.6048	8.9959	1.7316	5.9817	13.5290
Small	plus	minus	minus	3.4119	0.1048	16	32.54	<.0001	0.05	3.1896	3.6342	30.3232	3.1793	24.2799	37.8706
Small	plus	plus	minus	3.6108	0.09492	16	38.04	<.0001	0.05	3.4095	3.8120	36.9943	3.5116	30.2512	45.2404

The GLIMMIX Procedure

Differences of Colo*alph*Mesp1*CD31 Least Squares Means															
Colony_Size	alpha	Mesp1eYFP	CD31	_Colony_Size	_alpha	_Mesp1eYFP	_CD31	Estimate	Standard Error	DF	t Value	Pr > |t|	Alpha	Lower	Upper
Large	minus	minus	minus	Large	minus	plus	minus	-0.00057	14.0742	16	-0.00	1.0000	0.05	-29.8366	29.8355
Large	minus	minus	minus	Large	plus	minus	minus	-7.0802	9.9576	16	-0.71	0.4873	0.05	-28.1894	14.0289
Large	minus	minus	minus	Large	plus	plus	minus	-8.8996	9.9541	16	-0.89	0.3845	0.05	-30.0014	12.2021
Large	minus	minus	minus	Micro	minus	minus	minus	-0.00027	14.0754	16	-0.00	1.0000	0.05	-29.8388	29.8382
Large	minus	minus	minus	Micro	minus	plus	minus	-7.9965	9.9551	16	-0.80	0.4336	0.05	-29.1004	13.1073
Large	minus	minus	minus	Micro	plus	minus	minus	-8.9649	9.9540	16	-0.90	0.3811	0.05	-30.0666	12.1367
Large	minus	minus	minus	Micro	plus	plus	minus	-9.8215	9.9537	16	-0.99	0.3385	0.05	-30.9223	11.2794
Large	minus	minus	minus	Small	minus	minus	minus	-0.00045	14.0747	16	-0.00	1.0000	0.05	-29.8374	29.8365
Large	minus	minus	minus	Small	minus	plus	minus	-7.8912	9.9553	16	-0.79	0.4396	0.05	-28.9955	13.2130
Large	minus	minus	minus	Small	plus	minus	minus	-9.1064	9.9540	16	-0.91	0.3739	0.05	-30.2078	11.9951
Large	minus	minus	minus	Small	plus	plus	minus	-9.3052	9.9539	16	-0.93	0.3638	0.05	-30.4065	11.7960
Large	minus	plus	minus	Large	plus	minus	minus	-7.0796	9.9548	16	-0.71	0.4872	0.05	-28.1828	14.0235
Large	minus	plus	minus	Large	plus	plus	minus	-8.8991	9.9513	16	-0.89	0.3844	0.05	-29.9948	12.1967
Large	minus	plus	minus	Micro	minus	minus	minus	0.000302	14.0734	16	0.00	1.0000	0.05	-29.8340	29.8346
Large	minus	plus	minus	Micro	minus	plus	minus	-7.9960	9.9523	16	-0.80	0.4335	0.05	-29.0938	13.1019
Large	minus	plus	minus	Micro	plus	minus	minus	-8.9644	9.9512	16	-0.90	0.3810	0.05	-30.0600	12.1313
Large	minus	plus	minus	Micro	plus	plus	minus	-9.8209	9.9509	16	-0.99	0.3384	0.05	-30.9158	11.2740
Large	minus	plus	minus	Small	minus	minus	minus	0.000121	14.0727	16	0.00	1.0000	0.05	-29.8326	29.8329
Large	minus	plus	minus	Small	minus	plus	minus	-7.8907	9.9525	16	-0.79	0.4395	0.05	-28.9889	13.2076
Large	minus	plus	minus	Small	plus	minus	minus	-9.1058	9.9511	16	-0.92	0.3737	0.05	-30.2013	11.9897
Large	minus	plus	minus	Small	plus	plus	minus	-9.3047	9.9510	16	-0.94	0.3637	0.05	-30.3999	11.7906
Large	plus	minus	minus	Large	plus	plus	minus	-1.8194	0.3113	16	-5.84	<.0001	0.05	-2.4793	-1.1595
Large	plus	minus	minus	Micro	minus	minus	minus	7.0799	9.9564	16	0.71	0.4873	0.05	-14.0266	28.1865
Large	plus	minus	minus	Micro	minus	plus	minus	-0.9163	0.3417	16	-2.68	0.0164	0.05	-1.6406	-0.1921

The GLIMMIX Procedure

Differences of Colo*alph*Mesp1*CD31 Least Squares Means															
Colony_Size	alpha	Mesp1eYFP	CD31	_Colony_Size	_alpha	_Mesp1eYFP	_CD31	Estimate	Standard Error	DF	t Value	Pr > |t|	Alpha	Lower	Upper
Large	plus	minus	minus	Micro	plus	minus	minus	-1.8847	0.3099	16	-6.08	<.0001	0.05	-2.5417	-1.2278
Large	plus	minus	minus	Micro	plus	plus	minus	-2.7413	0.2979	16	-9.20	<.0001	0.05	-3.3728	-2.1097
Large	plus	minus	minus	Small	minus	minus	minus	7.0798	9.9554	16	0.71	0.4872	0.05	-14.0247	28.1842
Large	plus	minus	minus	Small	minus	plus	minus	-0.8110	0.3470	16	-2.34	0.0328	0.05	-1.5467	-0.07534
Large	plus	minus	minus	Small	plus	minus	minus	-2.0262	0.3072	16	-6.60	<.0001	0.05	-2.6774	-1.3749
Large	plus	minus	minus	Small	plus	plus	minus	-2.2250	0.3040	16	-7.32	<.0001	0.05	-2.8694	-1.5807
Large	plus	plus	minus	Micro	minus	minus	minus	8.8994	9.9529	16	0.89	0.3845	0.05	-12.1998	29.9985
Large	plus	plus	minus	Micro	minus	plus	minus	0.9031	0.2165	16	4.17	0.0007	0.05	0.4441	1.3620
Large	plus	plus	minus	Micro	plus	minus	minus	-0.06531	0.1618	16	-0.40	0.6918	0.05	-0.4083	0.2777
Large	plus	plus	minus	Micro	plus	plus	minus	-0.9218	0.1375	16	-6.71	<.0001	0.05	-1.2132	-0.6304
Large	plus	plus	minus	Small	minus	minus	minus	8.8992	9.9519	16	0.89	0.3845	0.05	-12.1978	29.9962
Large	plus	plus	minus	Small	minus	plus	minus	1.0084	0.2249	16	4.48	0.0004	0.05	0.5317	1.4851
Large	plus	plus	minus	Small	plus	minus	minus	-0.2068	0.1566	16	-1.32	0.2052	0.05	-0.5386	0.1251
Large	plus	plus	minus	Small	plus	plus	minus	-0.4056	0.1501	16	-2.70	0.0157	0.05	-0.7238	-0.08742
Micro	minus	minus	minus	Micro	minus	plus	minus	-7.9963	9.9539	16	-0.80	0.4336	0.05	-29.0975	13.1050
Micro	minus	minus	minus	Micro	plus	minus	minus	-8.9647	9.9528	16	-0.90	0.3811	0.05	-30.0637	12.1344
Micro	minus	minus	minus	Micro	plus	plus	minus	-9.8212	9.9525	16	-0.99	0.3384	0.05	-30.9195	11.2771
Micro	minus	minus	minus	Small	minus	minus	minus	-0.00018	14.0739	16	-0.00	1.0000	0.05	-29.8355	29.8351
Micro	minus	minus	minus	Small	minus	plus	minus	-7.8910	9.9541	16	-0.79	0.4395	0.05	-28.9926	13.2107
Micro	minus	minus	minus	Small	plus	minus	minus	-9.1061	9.9527	16	-0.91	0.3738	0.05	-30.2050	11.9928
Micro	minus	minus	minus	Small	plus	plus	minus	-9.3050	9.9526	16	-0.93	0.3637	0.05	-30.4036	11.7937
Micro	minus	plus	minus	Micro	plus	minus	minus	-0.9684	0.2145	16	-4.51	0.0004	0.05	-1.4231	-0.5137
Micro	minus	plus	minus	Micro	plus	plus	minus	-1.8249	0.1968	16	-9.27	<.0001	0.05	-2.2421	-1.4077
Micro	minus	plus	minus	Small	minus	minus	minus	7.9961	9.9529	16	0.80	0.4335	0.05	-13.1030	29.0952

The GLIMMIX Procedure

Differences of Colo*alph*Mesp1*CD31 Least Squares Means															
Colony_Size	alpha	Mesp1eYFP	CD31	_Colony_Size	_alpha	_Mesp1eYFP	_CD31	Estimate	Standard Error	DF	t Value	Pr > |t|	Alpha	Lower	Upper
Micro	minus	plus	minus	Small	minus	plus	minus	0.1053	0.2653	16	0.40	0.6967	0.05	-0.4572	0.6678
Micro	minus	plus	minus	Small	plus	minus	minus	-1.1098	0.2106	16	-5.27	<.0001	0.05	-1.5562	-0.6634
Micro	minus	plus	minus	Small	plus	plus	minus	-1.3087	0.2058	16	-6.36	<.0001	0.05	-1.7450	-0.8724
Micro	plus	minus	minus	Micro	plus	plus	minus	-0.8565	0.1343	16	-6.38	<.0001	0.05	-1.1413	-0.5718
Micro	plus	minus	minus	Small	minus	minus	minus	8.9645	9.9518	16	0.90	0.3811	0.05	-12.1324	30.0614
Micro	plus	minus	minus	Small	minus	plus	minus	1.0737	0.2230	16	4.82	0.0002	0.05	0.6010	1.5464
Micro	plus	minus	minus	Small	plus	minus	minus	-0.1414	0.1538	16	-0.92	0.3714	0.05	-0.4675	0.1846
Micro	plus	minus	minus	Small	plus	plus	minus	-0.3403	0.1472	16	-2.31	0.0345	0.05	-0.6524	-0.02821
Micro	plus	plus	minus	Small	minus	minus	minus	9.8210	9.9515	16	0.99	0.3384	0.05	-11.2751	30.9172
Micro	plus	plus	minus	Small	minus	plus	minus	1.9302	0.2060	16	9.37	<.0001	0.05	1.4936	2.3669
Micro	plus	plus	minus	Small	plus	minus	minus	0.7151	0.1279	16	5.59	<.0001	0.05	0.4439	0.9863
Micro	plus	plus	minus	Small	plus	plus	minus	0.5162	0.1199	16	4.30	0.0005	0.05	0.2620	0.7705
Small	minus	minus	minus	Small	minus	plus	minus	-7.8908	9.9531	16	-0.79	0.4395	0.05	-28.9903	13.2087
Small	minus	minus	minus	Small	plus	minus	minus	-9.1059	9.9517	16	-0.92	0.3738	0.05	-30.2027	11.9908
Small	minus	minus	minus	Small	plus	plus	minus	-9.3048	9.9516	16	-0.94	0.3637	0.05	-30.4013	11.7918
Small	minus	plus	minus	Small	plus	minus	minus	-1.2151	0.2192	16	-5.54	<.0001	0.05	-1.6798	-0.7505
Small	minus	plus	minus	Small	plus	plus	minus	-1.4140	0.2146	16	-6.59	<.0001	0.05	-1.8690	-0.9590
Small	plus	minus	minus	Small	plus	plus	minus	-0.1989	0.1414	16	-1.41	0.1789	0.05	-0.4987	0.1010

The GLIMMIX Procedure

Model Information	
Data Set	WORK.EXT_FIG5E
Response Variable	Count
Response Distribution	Poisson
Link Function	Log
Variance Function	Default
Variance Matrix Blocked By	Dish_ID
Estimation Technique	Maximum Likelihood
Likelihood Approximation	Laplace
Degrees of Freedom Method	Containment

Class Level Information		
Class	Levels	Values
Colony_Size	3	Large Micro Small
alpha	2	minus plus
Wnt1eYFP	2	minus plus
CD31	1	minus
Dish_ID	12	1 2 3 4 5 6 7 8 9 10 11 12

Number of Observations Read	36
Number of Observations Used	36

Dimensions	
G-side Cov. Parameters	1
Columns in X	72
Columns in Z per Subject	1

The GLIMMIX Procedure

Dimensions	
Subjects (Blocks in V)	12
Max Obs per Subject	3

Optimization Information	
Optimization Technique	Dual Quasi-Newton
Parameters in Optimization	13
Lower Boundaries	1
Upper Boundaries	0
Fixed Effects	Not Profiled
Starting From	GLM estimates

Iteration History

Iteration	Restarts	Evaluations	Objective Function	Change	Max Gradient
0	0	4	121.17775869	.	163.0314
1	0	3	119.70466755	1.47309114	0.132925
2	0	4	119.70465066	0.00001689	0.065615
3	0	4	119.70461432	0.00003633	0.171889
4	0	4	119.70452051	0.00009381	0.129937
5	0	6	119.70414725	0.00037326	0.129052
6	0	2	119.70394335	0.00020391	0.162332
7	0	2	119.70371279	0.00023056	0.16903
8	0	2	119.70342328	0.00028951	0.213341
9	0	2	119.70311547	0.00030781	0.164342
10	0	3	119.70290449	0.00021098	0.098451
11	0	4	119.70154142	0.00136308	0.206215

The GLIMMIX Procedure

Iteration History					
Iteration	Restarts	Evaluations	Objective Function	Change	Max Gradient
12	0	4	119.69407992	0.00746150	0.442958
13	0	3	119.69262883	0.00145109	0.173385
14	0	4	119.68749744	0.00513140	0.192131
15	0	3	119.68729799	0.00019944	0.218355
16	0	6	119.66739077	0.01990722	0.860419
17	0	3	119.66097274	0.00641803	0.784699
18	0	3	119.65793896	0.00303378	0.101767
19	0	4	119.63960362	0.01833534	0.466841
20	0	3	119.63609513	0.00350849	0.12561
21	0	4	119.62832947	0.00776566	0.451054
22	0	3	119.62644076	0.00188870	0.132547
23	0	2	119.62485402	0.00158674	0.2848
24	0	3	119.62436139	0.00049263	0.031611
25	0	2	119.62421302	0.00014837	0.081376
26	0	3	119.6241693	0.00004372	0.040828
27	0	3	119.62415702	0.00001228	0.038583
28	0	2	119.62414482	0.00001220	0.037143
29	0	4	119.62404713	0.00009769	0.037054
30	0	4	119.62381524	0.00023189	0.020257
31	0	3	119.62381298	0.00000226	0.002224
32	0	2	119.6238098	0.00000318	0.001516

Convergence criterion (GCONV=1E-8) satisfied.

Estimated G matrix is not positive definite.

The GLIMMIX Procedure

Fit Statistics	
**-2 Log Likelihood**	119.62
**AIC (smaller is better)**	143.62
**AICC (smaller is better)**	157.19
**BIC (smaller is better)**	149.44
**CAIC (smaller is better)**	161.44
**HQIC (smaller is better)**	141.47

Fit Statistics for Conditional Distribution	
**-2 log L(Count | r. effects)**	119.62
**Pearson Chi-Square**	13.64
**Pearson Chi-Square / DF**	0.38

Covariance Parameter Estimates

Cov Parm	Subject	Estimate	Standard Error
**Intercept**	Dish_ID	0	.

The GLIMMIX Procedure

Solutions for Fixed Effects									
Effect	Colony_Size	alpha	Wnt1eYFP	CD31	Estimate	Standard Error	DF	t Value	Pr > |t|
Intercept					3.1499	0.1196	8	26.34	<.0001
Colony_Size	Large				-2.0511	0.3541	16	-5.79	<.0001
Colony_Size	Micro				-1.3580	0.2643	16	-5.14	<.0001
Colony_Size	Small				0	.	.	.	.
alpha		minus			-1.7635	0.3124	16	-5.64	<.0001
alpha		plus			0	.	.	.	.
Colony_Size*alpha	Large	minus			-14.0412	901.15	16	-0.02	0.9878
Colony_Size*alpha	Large	plus			0	.	.	.	.
Colony_Size*alpha	Micro	minus			2.6798	0.4188	16	6.40	<.0001
Colony_Size*alpha	Micro	plus			0	.	.	.	.
Colony_Size*alpha	Small	minus			0	.	.	.	.
Colony_Size*alpha	Small	plus			0	.	.	.	.
Wnt1eYFP			minus		0.3159	0.1572	16	2.01	0.0616
Wnt1eYFP			plus		0	.	.	.	.
Colony_Size*Wnt1eYFP	Large		minus		1.5811	0.3905	16	4.05	0.0009
Colony_Size*Wnt1eYFP	Large		plus		0	.	.	.	.
Colony_Size*Wnt1eYFP	Micro		minus		1.8996	0.2938	16	6.47	<.0001
Colony_Size*Wnt1eYFP	Micro		plus		0	.	.	.	.
Colony_Size*Wnt1eYFP	Small		minus		0	.	.	.	.
Colony_Size*Wnt1eYFP	Small		plus		0	.	.	.	.
alpha*Wnt1eYFP		minus	minus		-20.9052	0	16	-Infty	<.0001
alpha*Wnt1eYFP		minus	plus		0	.	.	.	.
alpha*Wnt1eYFP		plus	minus		0	.	.	.	.
alpha*Wnt1eYFP		plus	plus		0	.	.	.	.

The GLIMMIX Procedure

Solutions for Fixed Effects									
Effect	Colony_Size	alpha	Wnt1eYFP	CD31	Estimate	Standard Error	DF	t Value	Pr > |t|
Colony*alpha*Wnt1eYF	Large	minus	minus		1.0275	0	16	Infty	<.0001
Colony*alpha*Wnt1eYF	Large	minus	plus		0	.	.	.	.
Colony*alpha*Wnt1eYF	Large	plus	minus		0	.	.	.	.
Colony*alpha*Wnt1eYF	Large	plus	plus		0	.	.	.	.
Colony*alpha*Wnt1eYF	Micro	minus	minus		-7.7354	0	16	-Infty	<.0001
Colony*alpha*Wnt1eYF	Micro	minus	plus		0	.	.	.	.
Colony*alpha*Wnt1eYF	Micro	plus	minus		0	.	.	.	.
Colony*alpha*Wnt1eYF	Micro	plus	plus		0	.	.	.	.
Colony*alpha*Wnt1eYF	Small	minus	minus		0	.	.	.	.
Colony*alpha*Wnt1eYF	Small	minus	plus		0	.	.	.	.
Colony*alpha*Wnt1eYF	Small	plus	minus		0	.	.	.	.
Colony*alpha*Wnt1eYF	Small	plus	plus		0	.	.	.	.
CD31				minus	0	.	.	.	.
Colony_Size*CD31	Large			minus	0	.	.	.	.
Colony_Size*CD31	Micro			minus	0	.	.	.	.
Colony_Size*CD31	Small			minus	0	.	.	.	.
alpha*CD31		minus		minus	0	.	.	.	.
alpha*CD31		plus		minus	0	.	.	.	.
Colony_Si*alpha*CD31	Large	minus		minus	0	.	.	.	.
Colony_Si*alpha*CD31	Large	plus		minus	0	.	.	.	.
Colony_Si*alpha*CD31	Micro	minus		minus	0	.	.	.	.
Colony_Si*alpha*CD31	Micro	plus		minus	0	.	.	.	.
Colony_Si*alpha*CD31	Small	minus		minus	0	.	.	.	.
Colony_Si*alpha*CD31	Small	plus		minus	0	.	.	.	.

The GLIMMIX Procedure

Solutions for Fixed Effects									
Effect	Colony_Size	alpha	Wnt1eYFP	CD31	Estimate	Standard Error	DF	t Value	Pr > |t|
Wnt1eYFP*CD31			minus	minus	0	.	.	.	.
Wnt1eYFP*CD31			plus	minus	0	.	.	.	.
Colony_*Wnt1eYF*CD31	Large		minus	minus	0	.	.	.	.
Colony_*Wnt1eYF*CD31	Large		plus	minus	0	.	.	.	.
Colony_*Wnt1eYF*CD31	Micro		minus	minus	0	.	.	.	.
Colony_*Wnt1eYF*CD31	Micro		plus	minus	0	.	.	.	.
Colony_*Wnt1eYF*CD31	Small		minus	minus	0	.	.	.	.
Colony_*Wnt1eYF*CD31	Small		plus	minus	0	.	.	.	.
alpha*Wnt1eYFP*CD31		minus	minus	minus	0	.	.	.	.
alpha*Wnt1eYFP*CD31		minus	plus	minus	0	.	.	.	.
alpha*Wnt1eYFP*CD31		plus	minus	minus	0	.	.	.	.
alpha*Wnt1eYFP*CD31		plus	plus	minus	0	.	.	.	.
Colo*alph*Wnt1e*CD31	Large	minus	minus	minus	0	.	.	.	.
Colo*alph*Wnt1e*CD31	Large	minus	plus	minus	0	.	.	.	.
Colo*alph*Wnt1e*CD31	Large	plus	minus	minus	0	.	.	.	.
Colo*alph*Wnt1e*CD31	Large	plus	plus	minus	0	.	.	.	.
Colo*alph*Wnt1e*CD31	Micro	minus	minus	minus	0	.	.	.	.
Colo*alph*Wnt1e*CD31	Micro	minus	plus	minus	0	.	.	.	.
Colo*alph*Wnt1e*CD31	Micro	plus	minus	minus	0	.	.	.	.
Colo*alph*Wnt1e*CD31	Micro	plus	plus	minus	0	.	.	.	.
Colo*alph*Wnt1e*CD31	Small	minus	minus	minus	0	.	.	.	.
Colo*alph*Wnt1e*CD31	Small	minus	plus	minus	0	.	.	.	.
Colo*alph*Wnt1e*CD31	Small	plus	minus	minus	0	.	.	.	.
Colo*alph*Wnt1e*CD31	Small	plus	plus	minus	0	.	.	.	.

The GLIMMIX Procedure

Type III Tests of Fixed Effects				
Effect	Num DF	Den DF	F Value	Pr > F
Colony_Size	2	16	16.46	0.0001
alpha	1	16	0.00	0.9553
Colony_Size*alpha	2	16	4.02	0.0384
Wnt1eYFP	1	16	4283.81	<.0001
Colony_Size*Wnt1eYFP	2	16	46.73	<.0001
alpha*Wnt1eYFP	1	16	Infty	<.0001
Colony*alpha*Wnt1eYF	2	16	Infty	<.0001
CD31	0	.	.	.
Colony_Size*CD31	0	.	.	.
alpha*CD31	0	.	.	.
Colony_Si*alpha*CD31	0	.	.	.
Wnt1eYFP*CD31	0	.	.	.
Colony_*Wnt1eYF*CD31	0	.	.	.
alpha*Wnt1eYFP*CD31	0	.	.	.
Colo*alph*Wnt1e*CD31	0	.	.	.

alpha*Wnt1eYFP*CD31 Least Squares Means														
alpha	Wnt1eYFP	CD31	Estimate	Standard Error	DF	t Value	Pr > |t|	Alpha	Lower	Upper	Mean	Standard Error Mean	Lower Mean	Upper Mean
minus	minus	minus	-25.2022	300.38	16	-0.08	0.9342	0.05	-661.99	611.58	1.13E-11	3.408E-9	318E-290	4.04E265
minus	plus	minus	-3.5372	300.38	16	-0.01	0.9908	0.05	-640.32	633.25	0.02909	8.7395	817E-281	1.03E275
plus	minus	minus	3.4896	0.06083	16	57.37	<.0001	0.05	3.3607	3.6186	32.7729	1.9935	28.8079	37.2836
plus	plus	minus	2.0135	0.1418	16	14.19	<.0001	0.05	1.7128	2.3142	7.4892	1.0623	5.5442	10.1165

The GLIMMIX Procedure

Differences of alpha*Wnt1eYFP*CD31 Least Squares Means													
alpha	Wnt1eYFP	CD31	_alpha	_Wnt1eYFP	_CD31	Estimate	Standard Error	DF	t Value	Pr > |t|	Alpha	Lower	Upper
minus	minus	minus	minus	plus	minus	-21.6650	0.1542	16	-140.47	<.0001	0.05	-21.9919	-21.3380
minus	minus	minus	plus	minus	minus	-28.6918	300.38	16	-0.10	0.9251	0.05	-665.48	608.09
minus	minus	minus	plus	plus	minus	-27.2157	300.38	16	-0.09	0.9289	0.05	-664.00	609.57
minus	plus	minus	plus	minus	minus	-7.0268	300.38	16	-0.02	0.9816	0.05	-643.81	629.76
minus	plus	minus	plus	plus	minus	-5.5507	300.38	16	-0.02	0.9855	0.05	-642.33	631.23
plus	minus	minus	plus	plus	minus	1.4761	0.1542	16	9.57	<.0001	0.05	1.1492	1.8031

Colo*alph*Wnt1e*CD31 Least Squares Means															
Colony_Size	alpha	Wnt1eYFP	CD31	Estimate	Standard Error	DF	t Value	Pr > |t|	Alpha	Lower	Upper	Mean	Standard Error Mean	Lower Mean	Upper Mean
Large	minus	minus	minus	-32.6866	901.15	16	-0.04	0.9715	0.05	-1943.04	1877.67	6.37E-15	5.74E-12	0	.
Large	minus	plus	minus	-14.7060	901.15	16	-0.02	0.9872	0.05	-1925.06	1895.65	4.105E-7	0.000370	0	.
Large	plus	minus	minus	2.9957	0.1292	16	23.19	<.0001	0.05	2.7219	3.2695	19.9999	2.5833	15.2093	26.2995
Large	plus	plus	minus	1.0987	0.3333	16	3.30	0.0046	0.05	0.3921	1.8054	3.0003	1.0001	1.4801	6.0823
Micro	minus	minus	minus	-23.7170	0.2896	16	-81.90	<.0001	0.05	-24.3308	-23.1031	5.01E-11	1.45E-11	2.71E-11	9.26E-11
Micro	minus	plus	minus	2.7080	0.1491	16	18.16	<.0001	0.05	2.3919	3.0242	14.9999	2.2369	10.9343	20.5772
Micro	plus	minus	minus	4.0073	0.07796	16	51.40	<.0001	0.05	3.8421	4.1726	54.9999	4.2879	46.6214	64.8842
Micro	plus	plus	minus	1.7918	0.2357	16	7.60	<.0001	0.05	1.2921	2.2915	6.0003	1.4145	3.6403	9.8902
Small	minus	minus	minus	-19.2030	0.3287	16	-58.42	<.0001	0.05	-19.8998	-18.5061	4.574E-9	1.503E-9	2.278E-9	9.181E-9
Small	minus	plus	minus	1.3863	0.2887	16	4.80	0.0002	0.05	0.7743	1.9983	4.0001	1.1548	2.1691	7.3768
Small	plus	minus	minus	3.4657	0.1021	16	33.93	<.0001	0.05	3.2492	3.6823	32.0003	3.2687	25.7698	39.7372
Small	plus	plus	minus	3.1499	0.1196	16	26.34	<.0001	0.05	2.8963	3.4034	23.3326	2.7905	18.1073	30.0657

The GLIMMIX Procedure

Differences of Colo*alph*Wnt1e*CD31 Least Squares Means															
Colony_Size	alpha	Wnt1eYFP	CD31	_Colony_Size	_alpha	_Wnt1eYFP	_CD31	Estimate	Standard Error	DF	t Value	Pr > |t|	Alpha	Lower	Upper
Large	minus	minus	minus	Large	minus	plus	minus	-17.9806	0.3574	16	-50.30	<.0001	0.05	-18.7384	-17.2229
Large	minus	minus	minus	Large	plus	minus	minus	-35.6824	901.15	16	-0.04	0.9689	0.05	-1946.03	1874.67
Large	minus	minus	minus	Large	plus	plus	minus	-33.7854	901.15	16	-0.04	0.9706	0.05	-1944.14	1876.57
Large	minus	minus	minus	Micro	minus	minus	minus	-8.9697	901.15	16	-0.01	0.9922	0.05	-1919.32	1901.38
Large	minus	minus	minus	Micro	minus	plus	minus	-35.3947	901.15	16	-0.04	0.9692	0.05	-1945.75	1874.96
Large	minus	minus	minus	Micro	plus	minus	minus	-36.6940	901.15	16	-0.04	0.9680	0.05	-1947.05	1873.66
Large	minus	minus	minus	Micro	plus	plus	minus	-34.4785	901.15	16	-0.04	0.9700	0.05	-1944.83	1875.87
Large	minus	minus	minus	Small	minus	minus	minus	-13.4837	901.15	16	-0.01	0.9882	0.05	-1923.84	1896.87
Large	minus	minus	minus	Small	minus	plus	minus	-34.0730	901.15	16	-0.04	0.9703	0.05	-1944.43	1876.28
Large	minus	minus	minus	Small	plus	minus	minus	-36.1524	901.15	16	-0.04	0.9685	0.05	-1946.50	1874.20
Large	minus	minus	minus	Small	plus	plus	minus	-35.8365	901.15	16	-0.04	0.9688	0.05	-1946.19	1874.52
Large	minus	plus	minus	Large	plus	minus	minus	-17.7017	901.15	16	-0.02	0.9846	0.05	-1928.05	1892.65
Large	minus	plus	minus	Large	plus	plus	minus	-15.8047	901.15	16	-0.02	0.9862	0.05	-1926.16	1894.55
Large	minus	plus	minus	Micro	minus	minus	minus	9.0110	901.15	16	0.01	0.9921	0.05	-1901.34	1919.36
Large	minus	plus	minus	Micro	minus	plus	minus	-17.4140	901.15	16	-0.02	0.9848	0.05	-1927.77	1892.94
Large	minus	plus	minus	Micro	plus	minus	minus	-18.7133	901.15	16	-0.02	0.9837	0.05	-1929.07	1891.64
Large	minus	plus	minus	Micro	plus	plus	minus	-16.4978	901.15	16	-0.02	0.9856	0.05	-1926.85	1893.85
Large	minus	plus	minus	Small	minus	minus	minus	4.4970	901.15	16	0.00	0.9961	0.05	-1905.86	1914.85
Large	minus	plus	minus	Small	minus	plus	minus	-16.0923	901.15	16	-0.02	0.9860	0.05	-1926.44	1894.26
Large	minus	plus	minus	Small	plus	minus	minus	-18.1717	901.15	16	-0.02	0.9842	0.05	-1928.52	1892.18
Large	minus	plus	minus	Small	plus	plus	minus	-17.8559	901.15	16	-0.02	0.9844	0.05	-1928.21	1892.50
Large	plus	minus	minus	Large	plus	plus	minus	1.8970	0.3574	16	5.31	<.0001	0.05	1.1393	2.6547
Large	plus	minus	minus	Micro	minus	minus	minus	26.7127	0.3170	16	84.26	<.0001	0.05	26.0407	27.3848
Large	plus	minus	minus	Micro	minus	plus	minus	0.2877	0.1972	16	1.46	0.1640	0.05	-0.1304	0.7057

The GLIMMIX Procedure

Differences of Colo*alph*Wnt1e*CD31 Least Squares Means															
Colony_Size	alpha	Wnt1eYFP	CD31	_Colony_Size	_alpha	_Wnt1eYFP	_CD31	Estimate	Standard Error	DF	t Value	Pr > |t|	Alpha	Lower	Upper
Large	plus	minus	minus	Micro	plus	minus	minus	-1.0116	0.1508	16	-6.71	<.0001	0.05	-1.3312	-0.6920
Large	plus	minus	minus	Micro	plus	plus	minus	1.2039	0.2687	16	4.48	0.0004	0.05	0.6342	1.7736
Large	plus	minus	minus	Small	minus	minus	minus	22.1987	0.3531	16	62.86	<.0001	0.05	21.4501	22.9473
Large	plus	minus	minus	Small	minus	plus	minus	1.6094	0.3162	16	5.09	0.0001	0.05	0.9390	2.2798
Large	plus	minus	minus	Small	plus	minus	minus	-0.4700	0.1646	16	-2.86	0.0114	0.05	-0.8189	-0.1211
Large	plus	minus	minus	Small	plus	plus	minus	-0.1541	0.1759	16	-0.88	0.3940	0.05	-0.5271	0.2188
Large	plus	plus	minus	Micro	minus	minus	minus	24.8157	0.4415	16	56.21	<.0001	0.05	23.8797	25.7517
Large	plus	plus	minus	Micro	minus	plus	minus	-1.6093	0.3651	16	-4.41	0.0004	0.05	-2.3834	-0.8353
Large	plus	plus	minus	Micro	plus	minus	minus	-2.9086	0.3423	16	-8.50	<.0001	0.05	-3.6342	-2.1830
Large	plus	plus	minus	Micro	plus	plus	minus	-0.6931	0.4082	16	-1.70	0.1089	0.05	-1.5585	0.1723
Large	plus	plus	minus	Small	minus	minus	minus	20.3017	0.4681	16	43.37	<.0001	0.05	19.3093	21.2940
Large	plus	plus	minus	Small	minus	plus	minus	-0.2876	0.4409	16	-0.65	0.5235	0.05	-1.2223	0.6471
Large	plus	plus	minus	Small	plus	minus	minus	-2.3670	0.3486	16	-6.79	<.0001	0.05	-3.1060	-1.6280
Large	plus	plus	minus	Small	plus	plus	minus	-2.0511	0.3541	16	-5.79	<.0001	0.05	-2.8018	-1.3005
Micro	minus	minus	minus	Micro	minus	plus	minus	-26.4250	0.2482	16	-106.46	<.0001	0.05	-26.9512	-25.8988
Micro	minus	minus	minus	Micro	plus	minus	minus	-27.7243	0.2789	16	-99.41	<.0001	0.05	-28.3155	-27.1331
Micro	minus	minus	minus	Micro	plus	plus	minus	-25.5088	0.5005	16	-50.97	<.0001	0.05	-26.5698	-24.4478
Micro	minus	minus	minus	Small	minus	minus	minus	-4.5140	0.4380	16	-10.31	<.0001	0.05	-5.4426	-3.5854
Micro	minus	minus	minus	Small	minus	plus	minus	-25.1033	0.4089	16	-61.40	<.0001	0.05	-25.9700	-24.2366
Micro	minus	minus	minus	Small	plus	minus	minus	-27.1827	0.3070	16	-88.54	<.0001	0.05	-27.8335	-26.5319
Micro	minus	minus	minus	Small	plus	plus	minus	-26.8668	0.3132	16	-85.77	<.0001	0.05	-27.5309	-26.2028
Micro	minus	plus	minus	Micro	plus	minus	minus	-1.2993	0.1682	16	-7.73	<.0001	0.05	-1.6558	-0.9428
Micro	minus	plus	minus	Micro	plus	plus	minus	0.9162	0.2789	16	3.29	0.0047	0.05	0.3250	1.5074
Micro	minus	plus	minus	Small	minus	minus	minus	21.9110	0.3609	16	60.71	<.0001	0.05	21.1459	22.6761

The GLIMMIX Procedure

Differences of Colo*alph*Wnt1e*CD31 Least Squares Means															
Colony_Size	alpha	Wnt1eYFP	CD31	_Colony_Size	_alpha	_Wnt1eYFP	_CD31	Estimate	Standard Error	DF	t Value	Pr > |t|	Alpha	Lower	Upper
Micro	minus	plus	minus	Small	minus	plus	minus	1.3217	0.3249	16	4.07	0.0009	0.05	0.6330	2.0104
Micro	minus	plus	minus	Small	plus	minus	minus	-0.7577	0.1807	16	-4.19	0.0007	0.05	-1.1407	-0.3747
Micro	minus	plus	minus	Small	plus	plus	minus	-0.4418	0.1911	16	-2.31	0.0344	0.05	-0.8469	-0.03675
Micro	plus	minus	minus	Micro	plus	plus	minus	2.2155	0.2482	16	8.93	<.0001	0.05	1.6893	2.7417
Micro	plus	minus	minus	Small	minus	minus	minus	23.2103	0.3378	16	68.71	<.0001	0.05	22.4942	23.9263
Micro	plus	minus	minus	Small	minus	plus	minus	2.6210	0.2990	16	8.77	<.0001	0.05	1.9872	3.2548
Micro	plus	minus	minus	Small	plus	minus	minus	0.5416	0.1284	16	4.22	0.0007	0.05	0.2695	0.8137
Micro	plus	minus	minus	Small	plus	plus	minus	0.8575	0.1426	16	6.01	<.0001	0.05	0.5551	1.1599
Micro	plus	plus	minus	Small	minus	minus	minus	20.9948	0.4045	16	51.91	<.0001	0.05	20.1374	21.8522
Micro	plus	plus	minus	Small	minus	plus	minus	0.4055	0.3727	16	1.09	0.2927	0.05	-0.3845	1.1955
Micro	plus	plus	minus	Small	plus	minus	minus	-1.6739	0.2568	16	-6.52	<.0001	0.05	-2.2184	-1.1294
Micro	plus	plus	minus	Small	plus	plus	minus	-1.3580	0.2643	16	-5.14	<.0001	0.05	-1.9183	-0.7978
Small	minus	minus	minus	Small	minus	plus	minus	-20.5893	0.1572	16	-131.00	<.0001	0.05	-20.9225	-20.2561
Small	minus	minus	minus	Small	plus	minus	minus	-22.6687	0.3124	16	-72.55	<.0001	0.05	-23.3310	-22.0064
Small	minus	minus	minus	Small	plus	plus	minus	-22.3528	0.3884	16	-57.54	<.0001	0.05	-23.1763	-21.5293
Small	minus	plus	minus	Small	plus	minus	minus	-2.0794	0.3062	16	-6.79	<.0001	0.05	-2.7285	-1.4303
Small	minus	plus	minus	Small	plus	plus	minus	-1.7635	0.3124	16	-5.64	<.0001	0.05	-2.4259	-1.1012
Small	plus	minus	minus	Small	plus	plus	minus	0.3159	0.1572	16	2.01	0.0616	0.05	-0.01729	0.6491

The GLIMMIX Procedure

Model Information	
**Data Set**	WORK.EXT_FIG5FIII
**Response Variable**	Count
**Response Distribution**	Poisson
**Link Function**	Log
**Variance Function**	Default
**Variance Matrix Blocked By**	Dish_ID
**Estimation Technique**	Maximum Likelihood
**Likelihood Approximation**	Laplace
**Degrees of Freedom Method**	Containment

Class Level Information		
**Class**	**Levels**	**Values**
**Colony_Size**	3	Large Micro Small
**alpha**	2	minus plus
**Sox1eYFP**	2	minus plus
**CD31**	1	minus
**Dish_ID**	12	1 2 3 4 5 6 7 8 9 10 11 12

**Number of Observations Read**	36
**Number of Observations Used**	36

Dimensions	
**G-side Cov. Parameters**	1
**Columns in X**	72
**Columns in Z per Subject**	1

The GLIMMIX Procedure

Dimensions	
Subjects (Blocks in V)	12
Max Obs per Subject	3

Optimization Information	
Optimization Technique	Dual Quasi-Newton
Parameters in Optimization	13
Lower Boundaries	1
Upper Boundaries	0
Fixed Effects	Not Profiled
Starting From	GLM estimates

Iteration History

Iteration	Restarts	Evaluations	Objective Function	Change	Max Gradient
0	0	4	122.68388621	.	285.9281
1	0	3	121.79191136	0.89197485	0.07421
2	0	6	121.79190142	0.00000994	0.103868

Convergence criterion (GCONV=1E-8) satisfied.

Estimated G matrix is not positive definite.

Fit Statistics	
-2 Log Likelihood	121.79
AIC (smaller is better)	145.79
AICC (smaller is better)	159.36
BIC (smaller is better)	151.61

The GLIMMIX Procedure

Fit Statistics	
**CAIC (smaller is better)**	163.61
**HQIC (smaller is better)**	143.64

Fit Statistics for Conditional Distribution	
**-2 log L(Count | r. effects)**	121.79
**Pearson Chi-Square**	8.34
**Pearson Chi-Square / DF**	0.23

Covariance Parameter Estimates

Cov Parm	Subject	Estimate	Standard Error
**Intercept**	Dish_ID	0	.

The GLIMMIX Procedure

Solutions for Fixed Effects									
Effect	Colony_Size	alpha	Sox1eYFP	CD31	Estimate	Standard Error	DF	t Value	Pr > |t|
Intercept					1.3863	0.2887	8	4.80	0.0014
Colony_Size	Large				-1.0986	0.5774	16	-1.90	0.0752
Colony_Size	Micro				1.3653	0.3234	16	4.22	0.0006
Colony_Size	Small				0	.	.	.	.
alpha		minus			-7.0795	9.9516	16	-0.71	0.4871
alpha		plus			0	.	.	.	.
Colony_Size*alpha	Large	minus			1.0986	14.0800	16	0.08	0.9388
Colony_Size*alpha	Large	plus			0	.	.	.	.
Colony_Size*alpha	Micro	minus			-1.3653	14.0716	16	-0.10	0.9239
Colony_Size*alpha	Micro	plus			0	.	.	.	.
Colony_Size*alpha	Small	minus			0	.	.	.	.
Colony_Size*alpha	Small	plus			0	.	.	.	.
Sox1eYFP			minus		3.1919	0.2945	16	10.84	<.0001
Sox1eYFP			plus		0	.	.	.	.
Colony_Size*Sox1eYFP	Large		minus		0.8017	0.5843	16	1.37	0.1889
Colony_Size*Sox1eYFP	Large		plus		0	.	.	.	.
Colony_Size*Sox1eYFP	Micro		minus		-1.2101	0.3331	16	-3.63	0.0022
Colony_Size*Sox1eYFP	Micro		plus		0	.	.	.	.
Colony_Size*Sox1eYFP	Small		minus		0	.	.	.	.
Colony_Size*Sox1eYFP	Small		plus		0	.	.	.	.
alpha*Sox1eYFP		minus	minus		5.2093	9.9529	16	0.52	0.6079
alpha*Sox1eYFP		minus	plus		0	.	.	.	.
alpha*Sox1eYFP		plus	minus		0	.	.	.	.
alpha*Sox1eYFP		plus	plus		0	.	.	.	.

The GLIMMIX Procedure

Solutions for Fixed Effects									
**Effect**	**Colony_Size**	**alpha**	**Sox1eYFP**	**CD31**	**Estimate**	**Standard Error**	**DF**	**t Value**	**Pr > |t|**
**Colony*alpha*Sox1eYF**	Large	minus	minus		-9.2030	17.2404	16	-0.53	0.6008
**Colony*alpha*Sox1eYF**	Large	minus	plus		0	.	.	.	.
**Colony*alpha*Sox1eYF**	Large	plus	minus		0	.	.	.	.
**Colony*alpha*Sox1eYF**	Large	plus	plus		0	.	.	.	.
**Colony*alpha*Sox1eYF**	Micro	minus	minus		0.7707	14.0739	16	0.05	0.9570
**Colony*alpha*Sox1eYF**	Micro	minus	plus		0	.	.	.	.
**Colony*alpha*Sox1eYF**	Micro	plus	minus		0	.	.	.	.
**Colony*alpha*Sox1eYF**	Micro	plus	plus		0	.	.	.	.
**Colony*alpha*Sox1eYF**	Small	minus	minus		0	.	.	.	.
**Colony*alpha*Sox1eYF**	Small	minus	plus		0	.	.	.	.
**Colony*alpha*Sox1eYF**	Small	plus	minus		0	.	.	.	.
**Colony*alpha*Sox1eYF**	Small	plus	plus		0	.	.	.	.
**CD31**				minus	0	.	.	.	.
**Colony_Size*CD31**	Large			minus	0	.	.	.	.
**Colony_Size*CD31**	Micro			minus	0	.	.	.	.
**Colony_Size*CD31**	Small			minus	0	.	.	.	.
**alpha*CD31**		minus		minus	0	.	.	.	.
**alpha*CD31**		plus		minus	0	.	.	.	.
**Colony_Si*alpha*CD31**	Large	minus		minus	0	.	.	.	.
**Colony_Si*alpha*CD31**	Large	plus		minus	0	.	.	.	.
**Colony_Si*alpha*CD31**	Micro	minus		minus	0	.	.	.	.
**Colony_Si*alpha*CD31**	Micro	plus		minus	0	.	.	.	.
**Colony_Si*alpha*CD31**	Small	minus		minus	0	.	.	.	.
**Colony_Si*alpha*CD31**	Small	plus		minus	0	.	.	.	.

The GLIMMIX Procedure

Solutions for Fixed Effects									
Effect	Colony_Size	alpha	Sox1eYFP	CD31	Estimate	Standard Error	DF	t Value	Pr > |t|
Sox1eYFP*CD31			minus	minus	0	.	.	.	.
Sox1eYFP*CD31			plus	minus	0	.	.	.	.
Colony_*Sox1eYF*CD31	Large		minus	minus	0	.	.	.	.
Colony_*Sox1eYF*CD31	Large		plus	minus	0	.	.	.	.
Colony_*Sox1eYF*CD31	Micro		minus	minus	0	.	.	.	.
Colony_*Sox1eYF*CD31	Micro		plus	minus	0	.	.	.	.
Colony_*Sox1eYF*CD31	Small		minus	minus	0	.	.	.	.
Colony_*Sox1eYF*CD31	Small		plus	minus	0	.	.	.	.
alpha*Sox1eYFP*CD31		minus	minus	minus	0	.	.	.	.
alpha*Sox1eYFP*CD31		minus	plus	minus	0	.	.	.	.
alpha*Sox1eYFP*CD31		plus	minus	minus	0	.	.	.	.
alpha*Sox1eYFP*CD31		plus	plus	minus	0	.	.	.	.
Colo*alph*Sox1e*CD31	Large	minus	minus	minus	0	.	.	.	.
Colo*alph*Sox1e*CD31	Large	minus	plus	minus	0	.	.	.	.
Colo*alph*Sox1e*CD31	Large	plus	minus	minus	0	.	.	.	.
Colo*alph*Sox1e*CD31	Large	plus	plus	minus	0	.	.	.	.
Colo*alph*Sox1e*CD31	Micro	minus	minus	minus	0	.	.	.	.
Colo*alph*Sox1e*CD31	Micro	minus	plus	minus	0	.	.	.	.
Colo*alph*Sox1e*CD31	Micro	plus	minus	minus	0	.	.	.	.
Colo*alph*Sox1e*CD31	Micro	plus	plus	minus	0	.	.	.	.
Colo*alph*Sox1e*CD31	Small	minus	minus	minus	0	.	.	.	.
Colo*alph*Sox1e*CD31	Small	minus	plus	minus	0	.	.	.	.
Colo*alph*Sox1e*CD31	Small	plus	minus	minus	0	.	.	.	.
Colo*alph*Sox1e*CD31	Small	plus	plus	minus	0	.	.	.	.

The GLIMMIX Procedure

Type III Tests of Fixed Effects				
Effect	Num DF	Den DF	F Value	Pr > F
Colony_Size	2	16	0.22	0.8059
alpha	1	16	3.24	0.0909
Colony_Size*alpha	2	16	0.08	0.9208
Sox1eYFP	1	16	1.64	0.2179
Colony_Size*Sox1eYFP	2	16	0.10	0.9058
alpha*Sox1eYFP	1	16	0.13	0.7225
Colony*alpha*Sox1eYF	2	16	0.19	0.8312
CD31	0	.	.	.
Colony_Size*CD31	0	.	.	.
alpha*CD31	0	.	.	.
Colony_Si*alpha*CD31	0	.	.	.
Sox1eYFP*CD31	0	.	.	.
Colony_*Sox1eYF*CD31	0	.	.	.
alpha*Sox1eYFP*CD31	0	.	.	.
Colo*alph*Sox1e*CD31	0	.	.	.

alpha*Sox1eYFP*CD31 Least Squares Means														
alpha	Sox1eYFP	CD31	Estimate	Standard Error	DF	t Value	Pr > |t|	Alpha	Lower	Upper	Mean	Standard Error Mean	Lower Mean	Upper Mean
minus	minus	minus	-0.2389	3.3169	16	-0.07	0.9435	0.05	-7.2704	6.7926	0.7875	2.6120	0.000696	891.18
minus	plus	minus	-5.6933	5.7432	16	-0.99	0.3363	0.05	-17.8684	6.4818	0.003368	0.01935	1.737E-8	653.15
plus	minus	minus	4.5309	0.03491	16	129.81	<.0001	0.05	4.4569	4.6049	92.8435	3.2407	86.2215	99.9741
plus	plus	minus	1.4751	0.1985	16	7.43	<.0001	0.05	1.0543	1.8959	4.3716	0.8678	2.8701	6.6588

The GLIMMIX Procedure

Differences of alpha*Sox1eYFP*CD31 Least Squares Means													
alpha	Sox1eYFP	CD31	_alpha	_Sox1eYFP	_CD31	Estimate	Standard Error	DF	t Value	Pr > |t|	Alpha	Lower	Upper
minus	minus	minus	minus	plus	minus	5.4544	6.6322	16	0.82	0.4229	0.05	-8.6053	19.5141
minus	minus	minus	plus	minus	minus	-4.7698	3.3171	16	-1.44	0.1697	0.05	-11.8017	2.2620
minus	minus	minus	plus	plus	minus	-1.7141	3.3228	16	-0.52	0.6130	0.05	-8.7581	5.3300
minus	plus	minus	plus	minus	minus	-10.2242	5.7433	16	-1.78	0.0940	0.05	-22.3995	1.9511
minus	plus	minus	plus	plus	minus	-7.1684	5.7467	16	-1.25	0.2302	0.05	-19.3508	5.0139
plus	minus	minus	plus	plus	minus	3.0558	0.2015	16	15.16	<.0001	0.05	2.6285	3.4830

Colo*alph*Sox1e*CD31 Least Squares Means															
Colony_Size	alpha	Sox1eYFP	CD31	Estimate	Standard Error	DF	t Value	Pr > |t|	Alpha	Lower	Upper	Mean	Standard Error Mean	Lower Mean	Upper Mean
Large	minus	minus	minus	-5.6933	9.9478	16	-0.57	0.5751	0.05	-26.7817	15.3950	0.003368	0.03351	2.34E-12	4852659
Large	minus	plus	minus	-5.6933	9.9479	16	-0.57	0.5751	0.05	-26.7819	15.3952	0.003368	0.03351	2.34E-12	4853573
Large	plus	minus	minus	4.2813	0.06788	16	63.07	<.0001	0.05	4.1374	4.4252	72.3317	4.9102	62.6368	83.5272
Large	plus	plus	minus	0.2876	0.5000	16	0.58	0.5731	0.05	-0.7724	1.3476	1.3333	0.6666	0.4619	3.8482
Micro	minus	minus	minus	2.2686	0.1857	16	12.22	<.0001	0.05	1.8749	2.6623	9.6660	1.7950	6.5205	14.3290
Micro	minus	plus	minus	-5.6933	9.9476	16	-0.57	0.5751	0.05	-26.7811	15.3946	0.003369	0.03351	2.34E-12	4850523
Micro	plus	minus	minus	4.7333	0.05415	16	87.41	<.0001	0.05	4.6185	4.8481	113.67	6.1555	101.35	127.50
Micro	plus	plus	minus	2.7515	0.1459	16	18.86	<.0001	0.05	2.4423	3.0607	15.6664	2.2852	11.4994	21.3433
Small	minus	minus	minus	2.7080	0.1491	16	18.16	<.0001	0.05	2.3919	3.0240	14.9986	2.2359	10.9346	20.5732
Small	minus	plus	minus	-5.6933	9.9474	16	-0.57	0.5750	0.05	-26.7808	15.3943	0.003369	0.03351	2.34E-12	4849108
Small	plus	minus	minus	4.5782	0.05852	16	78.23	<.0001	0.05	4.4541	4.7022	97.3349	5.6960	85.9788	110.19
Small	plus	plus	minus	1.3863	0.2887	16	4.80	0.0002	0.05	0.7743	1.9982	3.9999	1.1547	2.1691	7.3760

The GLIMMIX Procedure

Differences of Colo*alph*Sox1e*CD31 Least Squares Means															
Colony_Size	alpha	Sox1eYFP	CD31	_Colony_Size	_alpha	_Sox1eYFP	_CD31	Estimate	Standard Error	DF	t Value	Pr > |t|	Alpha	Lower	Upper
Large	minus	minus	minus	Large	minus	plus	minus	-7.38E-6	14.0683	16	-0.00	1.0000	0.05	-29.8234	29.8234
Large	minus	minus	minus	Large	plus	minus	minus	-9.9746	9.9480	16	-1.00	0.3309	0.05	-31.0635	11.1143
Large	minus	minus	minus	Large	plus	plus	minus	-5.9810	9.9604	16	-0.60	0.5566	0.05	-27.0960	15.1340
Large	minus	minus	minus	Micro	minus	minus	minus	-7.9620	9.9495	16	-0.80	0.4353	0.05	-29.0540	13.1301
Large	minus	minus	minus	Micro	minus	plus	minus	-0.00007	14.0681	16	-0.00	1.0000	0.05	-29.8232	29.8230
Large	minus	minus	minus	Micro	plus	minus	minus	-10.4267	9.9479	16	-1.05	0.3102	0.05	-31.5154	10.6620
Large	minus	minus	minus	Micro	plus	plus	minus	-8.4449	9.9489	16	-0.85	0.4085	0.05	-29.5355	12.6458
Large	minus	minus	minus	Small	minus	minus	minus	-8.4013	9.9489	16	-0.84	0.4109	0.05	-29.4921	12.6894
Large	minus	minus	minus	Small	minus	plus	minus	-0.00008	14.0681	16	-0.00	1.0000	0.05	-29.8232	29.8230
Large	minus	minus	minus	Small	plus	minus	minus	-10.2715	9.9480	16	-1.03	0.3172	0.05	-31.3602	10.8172
Large	minus	minus	minus	Small	plus	plus	minus	-7.0796	9.9520	16	-0.71	0.4871	0.05	-28.1769	14.0176
Large	minus	plus	minus	Large	plus	minus	minus	-9.9746	9.9481	16	-1.00	0.3309	0.05	-31.0637	11.1145
Large	minus	plus	minus	Large	plus	plus	minus	-5.9810	9.9604	16	-0.60	0.5566	0.05	-27.0961	15.1342
Large	minus	plus	minus	Micro	minus	minus	minus	-7.9620	9.9496	16	-0.80	0.4353	0.05	-29.0542	13.1303
Large	minus	plus	minus	Micro	minus	plus	minus	-0.00006	14.0682	16	-0.00	1.0000	0.05	-29.8233	29.8232
Large	minus	plus	minus	Micro	plus	minus	minus	-10.4267	9.9480	16	-1.05	0.3102	0.05	-31.5155	10.6622
Large	minus	plus	minus	Micro	plus	plus	minus	-8.4449	9.9489	16	-0.85	0.4085	0.05	-29.5357	12.6460
Large	minus	plus	minus	Small	minus	minus	minus	-8.4013	9.9490	16	-0.84	0.4109	0.05	-29.4922	12.6896
Large	minus	plus	minus	Small	minus	plus	minus	-0.00008	14.0682	16	-0.00	1.0000	0.05	-29.8232	29.8231
Large	minus	plus	minus	Small	plus	minus	minus	-10.2715	9.9481	16	-1.03	0.3172	0.05	-31.3604	10.8174
Large	minus	plus	minus	Small	plus	plus	minus	-7.0796	9.9521	16	-0.71	0.4871	0.05	-28.1770	14.0178
Large	plus	minus	minus	Large	plus	plus	minus	3.9936	0.5046	16	7.91	<.0001	0.05	2.9239	5.0633
Large	plus	minus	minus	Micro	minus	minus	minus	2.0126	0.1977	16	10.18	<.0001	0.05	1.5935	2.4318
Large	plus	minus	minus	Micro	minus	plus	minus	9.9745	9.9478	16	1.00	0.3309	0.05	-11.1138	31.0629

The GLIMMIX Procedure

Differences of Colo*alph*Sox1e*CD31 Least Squares Means															
Colony_Size	alpha	Sox1eYFP	CD31	_Colony_Size	_alpha	_Sox1eYFP	_CD31	Estimate	Standard Error	DF	t Value	Pr > |t|	Alpha	Lower	Upper
Large	plus	minus	minus	Micro	plus	minus	minus	-0.4521	0.08684	16	-5.21	<.0001	0.05	-0.6362	-0.2680
Large	plus	minus	minus	Micro	plus	plus	minus	1.5297	0.1609	16	9.51	<.0001	0.05	1.1887	1.8708
Large	plus	minus	minus	Small	minus	minus	minus	1.5733	0.1638	16	9.60	<.0001	0.05	1.2261	1.9206
Large	plus	minus	minus	Small	minus	plus	minus	9.9745	9.9476	16	1.00	0.3309	0.05	-11.1135	31.0626
Large	plus	minus	minus	Small	plus	minus	minus	-0.2969	0.08963	16	-3.31	0.0044	0.05	-0.4869	-0.1069
Large	plus	minus	minus	Small	plus	plus	minus	2.8950	0.2966	16	9.76	<.0001	0.05	2.2663	3.5237
Large	plus	plus	minus	Micro	minus	minus	minus	-1.9810	0.5334	16	-3.71	0.0019	0.05	-3.1117	-0.8503
Large	plus	plus	minus	Micro	minus	plus	minus	5.9809	9.9601	16	0.60	0.5566	0.05	-15.1336	27.0954
Large	plus	plus	minus	Micro	plus	minus	minus	-4.4457	0.5029	16	-8.84	<.0001	0.05	-5.5119	-3.3795
Large	plus	plus	minus	Micro	plus	plus	minus	-2.4639	0.5209	16	-4.73	0.0002	0.05	-3.5681	-1.3597
Large	plus	plus	minus	Small	minus	minus	minus	-2.4203	0.5218	16	-4.64	0.0003	0.05	-3.5264	-1.3142
Large	plus	plus	minus	Small	minus	plus	minus	5.9809	9.9600	16	0.60	0.5566	0.05	-15.1333	27.0951
Large	plus	plus	minus	Small	plus	minus	minus	-4.2905	0.5034	16	-8.52	<.0001	0.05	-5.3577	-3.2233
Large	plus	plus	minus	Small	plus	plus	minus	-1.0986	0.5774	16	-1.90	0.0752	0.05	-2.3226	0.1253
Micro	minus	minus	minus	Micro	minus	plus	minus	7.9619	9.9493	16	0.80	0.4353	0.05	-13.1297	29.0535
Micro	minus	minus	minus	Micro	plus	minus	minus	-2.4647	0.1934	16	-12.74	<.0001	0.05	-2.8748	-2.0546
Micro	minus	minus	minus	Micro	plus	plus	minus	-0.4829	0.2361	16	-2.04	0.0577	0.05	-0.9835	0.01769
Micro	minus	minus	minus	Small	minus	minus	minus	-0.4393	0.2381	16	-1.84	0.0836	0.05	-0.9442	0.06548
Micro	minus	minus	minus	Small	minus	plus	minus	7.9619	9.9492	16	0.80	0.4353	0.05	-13.1294	29.0532
Micro	minus	minus	minus	Small	plus	minus	minus	-2.3095	0.1947	16	-11.86	<.0001	0.05	-2.7223	-1.8968
Micro	minus	minus	minus	Small	plus	plus	minus	0.8823	0.3432	16	2.57	0.0205	0.05	0.1547	1.6100
Micro	minus	plus	minus	Micro	plus	minus	minus	-10.4266	9.9477	16	-1.05	0.3101	0.05	-31.5148	10.6616
Micro	minus	plus	minus	Micro	plus	plus	minus	-8.4448	9.9486	16	-0.85	0.4085	0.05	-29.5349	12.6453
Micro	minus	plus	minus	Small	minus	minus	minus	-8.4012	9.9487	16	-0.84	0.4109	0.05	-29.4915	12.6890

The GLIMMIX Procedure

Differences of Colo*alph*Sox1e*CD31 Least Squares Means

Colony_Size	alpha	Sox1eYFP	CD31	_Colony_Size	_alpha	_Sox1eYFP	_CD31	Estimate	Standard Error	DF	t Value	Pr > |t|	Alpha	Lower	Upper
Micro	minus	plus	minus	Small	minus	plus	minus	-0.00001	14.0679	16	-0.00	1.0000	0.05	-29.8227	29.8227
Micro	minus	plus	minus	Small	plus	minus	minus	-10.2714	9.9477	16	-1.03	0.3172	0.05	-31.3597	10.8168
Micro	minus	plus	minus	Small	plus	plus	minus	-7.0795	9.9517	16	-0.71	0.4871	0.05	-28.1763	14.0172
Micro	plus	minus	minus	Micro	plus	plus	minus	1.9818	0.1556	16	12.74	<.0001	0.05	1.6520	2.3117
Micro	plus	minus	minus	Small	minus	minus	minus	2.0254	0.1586	16	12.77	<.0001	0.05	1.6891	2.3616
Micro	plus	minus	minus	Small	minus	plus	minus	10.4266	9.9476	16	1.05	0.3101	0.05	-10.6613	31.5145
Micro	plus	minus	minus	Small	plus	minus	minus	0.1552	0.07973	16	1.95	0.0694	0.05	-0.01385	0.3242
Micro	plus	minus	minus	Small	plus	plus	minus	3.3471	0.2937	16	11.40	<.0001	0.05	2.7244	3.9697
Micro	plus	plus	minus	Small	minus	minus	minus	0.04356	0.2086	16	0.21	0.8372	0.05	-0.3986	0.4857
Micro	plus	plus	minus	Small	minus	plus	minus	8.4448	9.9485	16	0.85	0.4085	0.05	-12.6451	29.5346
Micro	plus	plus	minus	Small	plus	minus	minus	-1.8266	0.1572	16	-11.62	<.0001	0.05	-2.1598	-1.4935
Micro	plus	plus	minus	Small	plus	plus	minus	1.3653	0.3234	16	4.22	0.0006	0.05	0.6796	2.0509
Small	minus	minus	minus	Small	minus	plus	minus	8.4012	9.9486	16	0.84	0.4109	0.05	-12.6888	29.4912
Small	minus	minus	minus	Small	plus	minus	minus	-1.8702	0.1602	16	-11.68	<.0001	0.05	-2.2097	-1.5307
Small	minus	minus	minus	Small	plus	plus	minus	1.3217	0.3249	16	4.07	0.0009	0.05	0.6329	2.0104
Small	minus	plus	minus	Small	plus	minus	minus	-10.2714	9.9476	16	-1.03	0.3172	0.05	-31.3593	10.8165
Small	minus	plus	minus	Small	plus	plus	minus	-7.0795	9.9516	16	-0.71	0.4871	0.05	-28.1760	14.0169
Small	plus	minus	minus	Small	plus	plus	minus	3.1919	0.2945	16	10.84	<.0001	0.05	2.5675	3.8163

The GLIMMIX Procedure

Model Information	
**Data Set**	WORK.EXT_FIG8AII
**Response Variable**	Count
**Response Distribution**	Poisson
**Link Function**	Log
**Variance Function**	Default
**Variance Matrix Blocked By**	Dish_ID
**Estimation Technique**	Maximum Likelihood
**Likelihood Approximation**	Laplace
**Degrees of Freedom Method**	Containment

Class Level Information		
**Class**	**Levels**	**Values**
**Colony_Type**	3	BFU-E CFU-GEMM CFU-GM
**Condition**	5	E11.5 Endo+E11.5 Mesp1-PSC E11.5 Endo+E11.5 Mesp1-PSC+Cytokines E11.5 Endo+E11.5 Mesp1-PSC+PDGF-AA E11.5 Endo+E11.5 Wnt1-PSC+PDGF-AA E11.5AGM+Cytokines
**Dish_ID**	18	1 2 3 4 5 6 7 8 9 10 11 12 13 14 15 16 17 18

**Number of Observations Read**	54
**Number of Observations Used**	54

Dimensions	
**G-side Cov. Parameters**	1
**Columns in X**	24
**Columns in Z per Subject**	1
**Subjects (Blocks in V)**	18
**Max Obs per Subject**	3

The GLIMMIX Procedure

Optimization Information	
Optimization Technique	Dual Quasi-Newton
Parameters in Optimization	16
Lower Boundaries	1
Upper Boundaries	0
Fixed Effects	Not Profiled
Starting From	GLM estimates

Iteration History					
Iteration	Restarts	Evaluations	Objective Function	Change	Max Gradient
0	0	4	565.40174527	.	226.4414
1	0	4	561.51650756	3.88523771	25.20755
2	0	4	561.08022725	0.43628031	36.28161
3	0	2	560.44570545	0.63452180	27.01405
4	0	3	560.33656742	0.10913803	16.88273
5	0	2	560.21972379	0.11684362	7.94803
6	0	2	560.13952101	0.08020279	11.19042
7	0	2	560.05651221	0.08300880	5.520488
8	0	2	560.03494658	0.02156562	17.48087
9	0	4	559.96859843	0.06634816	1.884319
10	0	3	559.95809285	0.01050558	0.458764
11	0	3	559.95735131	0.00074154	1.319587
12	0	4	559.94833323	0.00901808	1.129353
13	0	3	559.94499909	0.00333414	0.5008
14	0	3	559.94446647	0.00053262	0.335419
15	0	3	559.94426867	0.00019780	0.136786

The GLIMMIX Procedure

Iteration History					
Iteration	Restarts	Evaluations	Objective Function	Change	Max Gradient
16	0	3	559.94413543	0.00013324	0.117395
17	0	3	559.94411426	0.00002117	0.063458
18	0	6	559.94356749	0.00054677	0.582386
19	0	2	559.94321999	0.00034750	0.323531
20	0	3	559.94303989	0.00018010	0.072575
21	0	4	559.94038084	0.00265905	0.940658
22	0	2	559.9387782	0.00160264	0.803598
23	0	2	559.93712754	0.00165066	0.422038
24	0	4	559.93054906	0.00657847	1.445704
25	0	4	559.90779299	0.02275607	1.004129
26	0	2	559.8888905	0.01890249	1.994095
27	0	3	559.88258102	0.00630948	0.158608
28	0	3	559.88181471	0.00076631	0.317786
29	0	2	559.88142672	0.00038800	0.500277
30	0	2	559.88095895	0.00046777	0.039278
31	0	3	559.8807394	0.00021955	0.080327
32	0	3	559.8806936	0.00004580	0.046025
33	0	2	559.88066309	0.00003051	0.107599
34	0	2	559.88062891	0.00003418	0.042273
35	0	2	559.88060274	0.00002617	0.081826
36	0	3	559.88059244	0.00001030	0.000787
37	0	3	559.8805922	0.00000024	0.00253

Convergence criterion (GCONV=1E-8) satisfied.

The GLIMMIX Procedure

Fit Statistics	
-2 Log Likelihood	559.88
AIC (smaller is better)	591.88
AICC (smaller is better)	606.58
BIC (smaller is better)	606.13
CAIC (smaller is better)	622.13
HQIC (smaller is better)	593.84

Fit Statistics for Conditional Distribution	
-2 log L(Count | r. effects)	474.87
Pearson Chi-Square	147.17
Pearson Chi-Square / DF	2.73

Covariance Parameter Estimates

Cov Parm	Subject	Estimate	Standard Error
Intercept	Dish_ID	0.1206	.

Solutions for Fixed Effects

Effect	Colony_Type	Condition	Estimate	Standard Error	DF	t Value	Pr > |t|
Intercept			6.6046	0	13	Infty	<.0001
Colony_Type	BFU-E		-1.1937	0	26	-Infty	<.0001
Colony_Type	CFU-GEMM		-1.3461	0	26	-Infty	<.0001
Colony_Type	CFU-GM		0	.	.	.	.
Condition		E11.5 Endo+E11.5 Mesp1-PSC	0.01150	0	26	Infty	<.0001
Condition		E11.5 Endo+E11.5 Mesp1-PSC+Cytokines	-0.5714	0	26	-Infty	<.0001

The GLIMMIX Procedure

Solutions for Fixed Effects							
**Effect**	**Colony_Type**	**Condition**	**Estimate**	**Standard Error**	**DF**	**t Value**	**Pr > |t|**
**Condition**		E11.5 Endo+E11.5 Mesp1-PSC+PDGF-AA	-0.09160	0	26	-Infty	<.0001
**Condition**		E11.5 Endo+E11.5 Wnt1-PSC+PDGF-AA	-22.0646	1275.89	26	-0.02	0.9863
**Condition**		E11.5AGM+Cytokines	0	.	.	.	.
**Colony_Typ*Condition**	BFU-E	E11.5 Endo+E11.5 Mesp1-PSC	-0.1706	0	26	-Infty	<.0001
**Colony_Typ*Condition**	BFU-E	E11.5 Endo+E11.5 Mesp1-PSC+Cytokines	0.04300	0	26	Infty	<.0001
**Colony_Typ*Condition**	BFU-E	E11.5 Endo+E11.5 Mesp1-PSC+PDGF-AA	-0.2171	0	26	-Infty	<.0001
**Colony_Typ*Condition**	BFU-E	E11.5 Endo+E11.5 Wnt1-PSC+PDGF-AA	-2.0580	6609.45	26	-0.00	0.9998
**Colony_Typ*Condition**	BFU-E	E11.5AGM+Cytokines	0	.	.	.	.
**Colony_Typ*Condition**	CFU-GEMM	E11.5 Endo+E11.5 Mesp1-PSC	-0.1846	0	26	-Infty	<.0001
**Colony_Typ*Condition**	CFU-GEMM	E11.5 Endo+E11.5 Mesp1-PSC+Cytokines	0.02856	0	26	Infty	<.0001
**Colony_Typ*Condition**	CFU-GEMM	E11.5 Endo+E11.5 Mesp1-PSC+PDGF-AA	-0.2783	0	26	-Infty	<.0001
**Colony_Typ*Condition**	CFU-GEMM	E11.5 Endo+E11.5 Wnt1-PSC+PDGF-AA	-1.9056	6609.42	26	-0.00	0.9998
**Colony_Typ*Condition**	CFU-GEMM	E11.5AGM+Cytokines	0	.	.	.	.
**Colony_Typ*Condition**	CFU-GM	E11.5 Endo+E11.5 Mesp1-PSC	0	.	.	.	.
**Colony_Typ*Condition**	CFU-GM	E11.5 Endo+E11.5 Mesp1-PSC+Cytokines	0	.	.	.	.
**Colony_Typ*Condition**	CFU-GM	E11.5 Endo+E11.5 Mesp1-PSC+PDGF-AA	0	.	.	.	.
**Colony_Typ*Condition**	CFU-GM	E11.5 Endo+E11.5 Wnt1-PSC+PDGF-AA	0	.	.	.	.
**Colony_Typ*Condition**	CFU-GM	E11.5AGM+Cytokines	0	.	.	.	.

Type III Tests of Fixed Effects				
**Effect**	**Num DF**	**Den DF**	**F Value**	**Pr > F**
**Colony_Type**	2	26	0.00	1.0000
**Condition**	1	26	0.00	0.9940
**Colony_Typ*Condition**	2	26	0.00	1.0000

The GLIMMIX Procedure

Condition Least Squares Means												
Condition	Estimate	Standard Error	DF	t Value	Pr > |t|	Alpha	Lower	Upper	Mean	Standard Error Mean	Lower Mean	Upper Mean
E11.5 Endo+E11.5 Mesp1-PSC	5.6511	0	26	Infty	<.0001	.	.	.	284.60	0	.	.
E11.5 Endo+E11.5 Mesp1-PSC+Cytokines	5.2105	0	26	Infty	<.0001	.	.	.	183.18	0	.	.
E11.5 Endo+E11.5 Mesp1-PSC+PDGF-AA	5.5012	0	26	Infty	<.0001	.	.	.	245.00	0	.	.
E11.5 Endo+E11.5 Wnt1-PSC+PDGF-AA	-17.6278	3086.55	26	-0.01	0.9955	0.05	-6362.12	6326.86	2.21E-8	0.000068	0	.
E11.5AGM+Cytokines	5.7580	0	26	Infty	<.0001	.	.	.	316.71	0	.	.

Differences of Condition Least Squares Means									
Condition	_Condition	Estimate	Standard Error	DF	t Value	Pr > |t|	Alpha	Lower	Upper
E11.5 Endo+E11.5 Mesp1-PSC	E11.5 Endo+E11.5 Mesp1-PSC+Cytokines	0.4406	0	26	Infty	<.0001	.	.	.
E11.5 Endo+E11.5 Mesp1-PSC	E11.5 Endo+E11.5 Mesp1-PSC+PDGF-AA	0.1498	0	26	Infty	<.0001	.	.	.
E11.5 Endo+E11.5 Mesp1-PSC	E11.5 Endo+E11.5 Wnt1-PSC+PDGF-AA	23.2789	3086.55	26	0.01	0.9940	0.05	-6321.21	6367.77
E11.5 Endo+E11.5 Mesp1-PSC	E11.5AGM+Cytokines	-0.1069	0	26	-Infty	<.0001	.	.	.
E11.5 Endo+E11.5 Mesp1-PSC+Cytokines	E11.5 Endo+E11.5 Mesp1-PSC+PDGF-AA	-0.2908	0	26	-Infty	<.0001	.	.	.
E11.5 Endo+E11.5 Mesp1-PSC+Cytokines	E11.5 Endo+E11.5 Wnt1-PSC+PDGF-AA	22.8383	3086.55	26	0.01	0.9942	0.05	-6321.65	6367.33
E11.5 Endo+E11.5 Mesp1-PSC+Cytokines	E11.5AGM+Cytokines	-0.5475	0	26	-Infty	<.0001	.	.	.
E11.5 Endo+E11.5 Mesp1-PSC+PDGF-AA	E11.5 Endo+E11.5 Wnt1-PSC+PDGF-AA	23.1290	3086.55	26	0.01	0.9941	0.05	-6321.36	6367.62
E11.5 Endo+E11.5 Mesp1-PSC+PDGF-AA	E11.5AGM+Cytokines	-0.2567	0	26	-Infty	<.0001	.	.	.
E11.5 Endo+E11.5 Wnt1-PSC+PDGF-AA	E11.5AGM+Cytokines	-23.3858	3086.55	26	-0.01	0.9940	0.05	-6367.88	6321.10

The GLIMMIX Procedure

Colony_Typ*Condition Least Squares Means														
Colony_Type	Condition	Estimate	Standard Error	DF	t Value	Pr > |t|	Alpha	Lower	Upper	Mean	Standard Error Mean	Lower Mean	Upper Mean
BFU-E	E11.5 Endo+E11.5 Mesp1-PSC	5.2518	0	26	Infty	<.0001	.	.	.	190.91	0	.	.
BFU-E	E11.5 Endo+E11.5 Mesp1-PSC+Cytokines	4.8825	0	26	Infty	<.0001	.	.	.	131.96	0	.	.
BFU-E	E11.5 Endo+E11.5 Mesp1-PSC+PDGF-AA	5.1022	0	26	Infty	<.0001	.	.	.	164.38	0	.	.
BFU-E	E11.5 Endo+E11.5 Wnt1-PSC+PDGF-AA	-18.7117	6485.12	26	-0.00	0.9977	0.05	-13349	13312	7.475E-9	0.000048	0	.
BFU-E	E11.5AGM+Cytokines	5.4109	0	26	Infty	<.0001	.	.	.	223.84	0	.	.
CFU-GEMM	E11.5 Endo+E11.5 Mesp1-PSC	5.0854	0	26	Infty	<.0001	.	.	.	161.64	0	.	.
CFU-GEMM	E11.5 Endo+E11.5 Mesp1-PSC+Cytokines	4.7157	0	26	Infty	<.0001	.	.	.	111.68	0	.	.
CFU-GEMM	E11.5 Endo+E11.5 Mesp1-PSC+PDGF-AA	4.8886	0	26	Infty	<.0001	.	.	.	132.77	0	.	.
CFU-GEMM	E11.5 Endo+E11.5 Wnt1-PSC+PDGF-AA	-18.7117	6485.10	26	-0.00	0.9977	0.05	-13349	13312	7.475E-9	0.000048	0	.
CFU-GEMM	E11.5AGM+Cytokines	5.2585	0	26	Infty	<.0001	.	.	.	192.19	0	.	.
CFU-GM	E11.5 Endo+E11.5 Mesp1-PSC	6.6161	0	26	Infty	<.0001	.	.	.	747.00	0	.	.
CFU-GM	E11.5 Endo+E11.5 Mesp1-PSC+Cytokines	6.0332	0	26	Infty	<.0001	.	.	.	417.04	0	.	.
CFU-GM	E11.5 Endo+E11.5 Mesp1-PSC+PDGF-AA	6.5130	0	26	Infty	<.0001	.	.	.	673.82	0	.	.
CFU-GM	E11.5 Endo+E11.5 Wnt1-PSC+PDGF-AA	-15.4600	1275.89	26	-0.01	0.9904	0.05	-2638.09	2607.17	1.931E-7	0.000246	0	.
CFU-GM	E11.5AGM+Cytokines	6.6046	0	26	Infty	<.0001	.	.	.	738.46	0	.	.

The GLIMMIX Procedure

Differences of Colony_Typ*Condition Least Squares Means											
Colony_Type	Condition	_Colony_Type	_Condition	Estimate	Standard Error	DF	t Value	Pr > |t|	Alpha	Lower	Upper
BFU-E	E11.5 Endo+E11.5 Mesp1-PSC	BFU-E	E11.5 Endo+E11.5 Mesp1-PSC+Cytokines	0.3693	0	26	Infty	<.0001	.	.	.
BFU-E	E11.5 Endo+E11.5 Mesp1-PSC	BFU-E	E11.5 Endo+E11.5 Mesp1-PSC+PDGF-AA	0.1496	0	26	Infty	<.0001	.	.	.
BFU-E	E11.5 Endo+E11.5 Mesp1-PSC	BFU-E	E11.5 Endo+E11.5 Wnt1-PSC+PDGF-AA	23.9635	6485.12	26	0.00	0.9971	0.05	-13306	13354
BFU-E	E11.5 Endo+E11.5 Mesp1-PSC	BFU-E	E11.5AGM+Cytokines	-0.1591	0	26	-Infty	<.0001	.	.	.
BFU-E	E11.5 Endo+E11.5 Mesp1-PSC	CFU-GEMM	E11.5 Endo+E11.5 Mesp1-PSC	0.1664	0	26	Infty	<.0001	.	.	.
BFU-E	E11.5 Endo+E11.5 Mesp1-PSC	CFU-GEMM	E11.5 Endo+E11.5 Mesp1-PSC+Cytokines	0.5361	0	26	Infty	<.0001	.	.	.
BFU-E	E11.5 Endo+E11.5 Mesp1-PSC	CFU-GEMM	E11.5 Endo+E11.5 Mesp1-PSC+PDGF-AA	0.3632	0	26	Infty	<.0001	.	.	.
BFU-E	E11.5 Endo+E11.5 Mesp1-PSC	CFU-GEMM	E11.5 Endo+E11.5 Wnt1-PSC+PDGF-AA	23.9635	6485.10	26	0.00	0.9971	0.05	-13306	13354
BFU-E	E11.5 Endo+E11.5 Mesp1-PSC	CFU-GEMM	E11.5AGM+Cytokines	-0.00671	0	26	-Infty	<.0001	.	.	.
BFU-E	E11.5 Endo+E11.5 Mesp1-PSC	CFU-GM	E11.5 Endo+E11.5 Mesp1-PSC	-1.3643	0	26	-Infty	<.0001	.	.	.
BFU-E	E11.5 Endo+E11.5 Mesp1-PSC	CFU-GM	E11.5 Endo+E11.5 Mesp1-PSC+Cytokines	-0.7814	0	26	-Infty	<.0001	.	.	.
BFU-E	E11.5 Endo+E11.5 Mesp1-PSC	CFU-GM	E11.5 Endo+E11.5 Mesp1-PSC+PDGF-AA	-1.2612	0	26	-Infty	<.0001	.	.	.
BFU-E	E11.5 Endo+E11.5 Mesp1-PSC	CFU-GM	E11.5 Endo+E11.5 Wnt1-PSC+PDGF-AA	20.7118	1275.89	26	0.02	0.9872	0.05	-2601.92	2643.34
BFU-E	E11.5 Endo+E11.5 Mesp1-PSC	CFU-GM	E11.5AGM+Cytokines	-1.3528	0	26	-Infty	<.0001	.	.	.
BFU-E	E11.5 Endo+E11.5 Mesp1-PSC+Cytokines	BFU-E	E11.5 Endo+E11.5 Mesp1-PSC+PDGF-AA	-0.2197	0	26	-Infty	<.0001	.	.	.
BFU-E	E11.5 Endo+E11.5 Mesp1-PSC+Cytokines	BFU-E	E11.5 Endo+E11.5 Wnt1-PSC+PDGF-AA	23.5942	6485.12	26	0.00	0.9971	0.05	-13307	13354
BFU-E	E11.5 Endo+E11.5 Mesp1-PSC+Cytokines	BFU-E	E11.5AGM+Cytokines	-0.5284	0	26	-Infty	<.0001	.	.	.
BFU-E	E11.5 Endo+E11.5 Mesp1-PSC+Cytokines	CFU-GEMM	E11.5 Endo+E11.5 Mesp1-PSC	-0.2028	0	26	-Infty	<.0001	.	.	.
BFU-E	E11.5 Endo+E11.5 Mesp1-PSC+Cytokines	CFU-GEMM	E11.5 Endo+E11.5 Mesp1-PSC+Cytokines	0.1669	0	26	Infty	<.0001	.	.	.
BFU-E	E11.5 Endo+E11.5 Mesp1-PSC+Cytokines	CFU-GEMM	E11.5 Endo+E11.5 Mesp1-PSC+PDGF-AA	-0.00608	0	26	-Infty	<.0001	.	.	.
BFU-E	E11.5 Endo+E11.5 Mesp1-PSC+Cytokines	CFU-GEMM	E11.5 Endo+E11.5 Wnt1-PSC+PDGF-AA	23.5942	6485.10	26	0.00	0.9971	0.05	-13307	13354
BFU-E	E11.5 Endo+E11.5 Mesp1-PSC+Cytokines	CFU-GEMM	E11.5AGM+Cytokines	-0.3760	0	26	-Infty	<.0001	.	.	.
BFU-E	E11.5 Endo+E11.5 Mesp1-PSC+Cytokines	CFU-GM	E11.5 Endo+E11.5 Mesp1-PSC	-1.7335	0	26	-Infty	<.0001	.	.	.
BFU-E	E11.5 Endo+E11.5 Mesp1-PSC+Cytokines	CFU-GM	E11.5 Endo+E11.5 Mesp1-PSC+Cytokines	-1.1507	0	26	-Infty	<.0001	.	.	.

The GLIMMIX Procedure

Differences of Colony_Typ*Condition Least Squares Means											
Colony_Type	Condition	_Colony_Type	_Condition	Estimate	Standard Error	DF	t Value	Pr > |t|	Alpha	Lower	Upper
BFU-E	E11.5 Endo+E11.5 Mesp1-PSC+Cytokines	CFU-GM	E11.5 Endo+E11.5 Mesp1-PSC+PDGF-AA	-1.6304	0	26	-Infty	<.0001	.	.	.
BFU-E	E11.5 Endo+E11.5 Mesp1-PSC+Cytokines	CFU-GM	E11.5 Endo+E11.5 Wnt1-PSC+PDGF-AA	20.3425	1275.89	26	0.02	0.9874	0.05	-2602.29	2642.97
BFU-E	E11.5 Endo+E11.5 Mesp1-PSC+Cytokines	CFU-GM	E11.5AGM+Cytokines	-1.7220	0	26	-Infty	<.0001	.	.	.
BFU-E	E11.5 Endo+E11.5 Mesp1-PSC+PDGF-AA	BFU-E	E11.5 Endo+E11.5 Wnt1-PSC+PDGF-AA	23.8139	6485.12	26	0.00	0.9971	0.05	-13307	13354
BFU-E	E11.5 Endo+E11.5 Mesp1-PSC+PDGF-AA	BFU-E	E11.5AGM+Cytokines	-0.3087	0	26	-Infty	<.0001	.	.	.
BFU-E	E11.5 Endo+E11.5 Mesp1-PSC+PDGF-AA	CFU-GEMM	E11.5 Endo+E11.5 Mesp1-PSC	0.01681	0	26	Infty	<.0001	.	.	.
BFU-E	E11.5 Endo+E11.5 Mesp1-PSC+PDGF-AA	CFU-GEMM	E11.5 Endo+E11.5 Mesp1-PSC+Cytokines	0.3865	0	26	Infty	<.0001	.	.	.
BFU-E	E11.5 Endo+E11.5 Mesp1-PSC+PDGF-AA	CFU-GEMM	E11.5 Endo+E11.5 Mesp1-PSC+PDGF-AA	0.2136	0	26	Infty	<.0001	.	.	.
BFU-E	E11.5 Endo+E11.5 Mesp1-PSC+PDGF-AA	CFU-GEMM	E11.5 Endo+E11.5 Wnt1-PSC+PDGF-AA	23.8139	6485.10	26	0.00	0.9971	0.05	-13306	13354
BFU-E	E11.5 Endo+E11.5 Mesp1-PSC+PDGF-AA	CFU-GEMM	E11.5AGM+Cytokines	-0.1563	0	26	-Infty	<.0001	.	.	.
BFU-E	E11.5 Endo+E11.5 Mesp1-PSC+PDGF-AA	CFU-GM	E11.5 Endo+E11.5 Mesp1-PSC	-1.5139	0	26	-Infty	<.0001	.	.	.
BFU-E	E11.5 Endo+E11.5 Mesp1-PSC+PDGF-AA	CFU-GM	E11.5 Endo+E11.5 Mesp1-PSC+Cytokines	-0.9310	0	26	-Infty	<.0001	.	.	.
BFU-E	E11.5 Endo+E11.5 Mesp1-PSC+PDGF-AA	CFU-GM	E11.5 Endo+E11.5 Mesp1-PSC+PDGF-AA	-1.4108	0	26	-Infty	<.0001	.	.	.
BFU-E	E11.5 Endo+E11.5 Mesp1-PSC+PDGF-AA	CFU-GM	E11.5 Endo+E11.5 Wnt1-PSC+PDGF-AA	20.5622	1275.89	26	0.02	0.9873	0.05	-2602.07	2643.19
BFU-E	E11.5 Endo+E11.5 Mesp1-PSC+PDGF-AA	CFU-GM	E11.5AGM+Cytokines	-1.5024	0	26	-Infty	<.0001	.	.	.
BFU-E	E11.5 Endo+E11.5 Wnt1-PSC+PDGF-AA	BFU-E	E11.5AGM+Cytokines	-24.1226	6485.12	26	-0.00	0.9971	0.05	-13354	13306
BFU-E	E11.5 Endo+E11.5 Wnt1-PSC+PDGF-AA	CFU-GEMM	E11.5 Endo+E11.5 Mesp1-PSC	-23.7971	6485.12	26	-0.00	0.9971	0.05	-13354	13307
BFU-E	E11.5 Endo+E11.5 Wnt1-PSC+PDGF-AA	CFU-GEMM	E11.5 Endo+E11.5 Mesp1-PSC+Cytokines	-23.4274	6485.12	26	-0.00	0.9971	0.05	-13354	13307
BFU-E	E11.5 Endo+E11.5 Wnt1-PSC+PDGF-AA	CFU-GEMM	E11.5 Endo+E11.5 Mesp1-PSC+PDGF-AA	-23.6003	6485.12	26	-0.00	0.9971	0.05	-13354	13307
BFU-E	E11.5 Endo+E11.5 Wnt1-PSC+PDGF-AA	CFU-GEMM	E11.5 Endo+E11.5 Wnt1-PSC+PDGF-AA	-8.26E-6	9171.33	26	-0.00	1.0000	0.05	-18852	18852
BFU-E	E11.5 Endo+E11.5 Wnt1-PSC+PDGF-AA	CFU-GEMM	E11.5AGM+Cytokines	-23.9702	6485.12	26	-0.00	0.9971	0.05	-13354	13306
BFU-E	E11.5 Endo+E11.5 Wnt1-PSC+PDGF-AA	CFU-GM	E11.5 Endo+E11.5 Mesp1-PSC	-25.3278	6485.12	26	-0.00	0.9969	0.05	-13356	13305
BFU-E	E11.5 Endo+E11.5 Wnt1-PSC+PDGF-AA	CFU-GM	E11.5 Endo+E11.5 Mesp1-PSC+Cytokines	-24.7449	6485.12	26	-0.00	0.9970	0.05	-13355	13306
BFU-E	E11.5 Endo+E11.5 Wnt1-PSC+PDGF-AA	CFU-GM	E11.5 Endo+E11.5 Mesp1-PSC+PDGF-AA	-25.2247	6485.12	26	-0.00	0.9969	0.05	-13356	13305

The GLIMMIX Procedure

Differences of Colony_Typ*Condition Least Squares Means											
Colony_Type	Condition	_Colony_Type	_Condition	Estimate	Standard Error	DF	t Value	Pr > |t|	Alpha	Lower	Upper
BFU-E	E11.5 Endo+E11.5 Wnt1-PSC+PDGF-AA	CFU-GM	E11.5 Endo+E11.5 Wnt1-PSC+PDGF-AA	-3.2517	6609.45	26	-0.00	0.9996	0.05	-13589	13583
BFU-E	E11.5 Endo+E11.5 Wnt1-PSC+PDGF-AA	CFU-GM	E11.5AGM+Cytokines	-25.3163	6485.12	26	-0.00	0.9969	0.05	-13356	13305
BFU-E	E11.5AGM+Cytokines	CFU-GEMM	E11.5 Endo+E11.5 Mesp1-PSC	0.3255	0	26	Infty	<.0001	.	.	.
BFU-E	E11.5AGM+Cytokines	CFU-GEMM	E11.5 Endo+E11.5 Mesp1-PSC+Cytokines	0.6952	0	26	Infty	<.0001	.	.	.
BFU-E	E11.5AGM+Cytokines	CFU-GEMM	E11.5 Endo+E11.5 Mesp1-PSC+PDGF-AA	0.5223	0	26	Infty	<.0001	.	.	.
BFU-E	E11.5AGM+Cytokines	CFU-GEMM	E11.5 Endo+E11.5 Wnt1-PSC+PDGF-AA	24.1226	6485.10	26	0.00	0.9971	0.05	-13306	13354
BFU-E	E11.5AGM+Cytokines	CFU-GEMM	E11.5AGM+Cytokines	0.1524	0	26	Infty	<.0001	.	.	.
BFU-E	E11.5AGM+Cytokines	CFU-GM	E11.5 Endo+E11.5 Mesp1-PSC	-1.2052	0	26	-Infty	<.0001	.	.	.
BFU-E	E11.5AGM+Cytokines	CFU-GM	E11.5 Endo+E11.5 Mesp1-PSC+Cytokines	-0.6223	0	26	-Infty	<.0001	.	.	.
BFU-E	E11.5AGM+Cytokines	CFU-GM	E11.5 Endo+E11.5 Mesp1-PSC+PDGF-AA	-1.1020	0	26	-Infty	<.0001	.	.	.
BFU-E	E11.5AGM+Cytokines	CFU-GM	E11.5 Endo+E11.5 Wnt1-PSC+PDGF-AA	20.8709	1275.89	26	0.02	0.9871	0.05	-2601.76	2643.50
BFU-E	E11.5AGM+Cytokines	CFU-GM	E11.5AGM+Cytokines	-1.1937	0	26	-Infty	<.0001	.	.	.
CFU-GEMM	E11.5 Endo+E11.5 Mesp1-PSC	CFU-GEMM	E11.5 Endo+E11.5 Mesp1-PSC+Cytokines	0.3697	0	26	Infty	<.0001	.	.	.
CFU-GEMM	E11.5 Endo+E11.5 Mesp1-PSC	CFU-GEMM	E11.5 Endo+E11.5 Mesp1-PSC+PDGF-AA	0.1968	0	26	Infty	<.0001	.	.	.
CFU-GEMM	E11.5 Endo+E11.5 Mesp1-PSC	CFU-GEMM	E11.5 Endo+E11.5 Wnt1-PSC+PDGF-AA	23.7971	6485.10	26	0.00	0.9971	0.05	-13307	13354
CFU-GEMM	E11.5 Endo+E11.5 Mesp1-PSC	CFU-GEMM	E11.5AGM+Cytokines	-0.1731	0	26	-Infty	<.0001	.	.	.
CFU-GEMM	E11.5 Endo+E11.5 Mesp1-PSC	CFU-GM	E11.5 Endo+E11.5 Mesp1-PSC	-1.5307	0	26	-Infty	<.0001	.	.	.
CFU-GEMM	E11.5 Endo+E11.5 Mesp1-PSC	CFU-GM	E11.5 Endo+E11.5 Mesp1-PSC+Cytokines	-0.9478	0	26	-Infty	<.0001	.	.	.
CFU-GEMM	E11.5 Endo+E11.5 Mesp1-PSC	CFU-GM	E11.5 Endo+E11.5 Mesp1-PSC+PDGF-AA	-1.4276	0	26	-Infty	<.0001	.	.	.
CFU-GEMM	E11.5 Endo+E11.5 Mesp1-PSC	CFU-GM	E11.5 Endo+E11.5 Wnt1-PSC+PDGF-AA	20.5454	1275.89	26	0.02	0.9873	0.05	-2602.09	2643.18
CFU-GEMM	E11.5 Endo+E11.5 Mesp1-PSC	CFU-GM	E11.5AGM+Cytokines	-1.5192	0	26	-Infty	<.0001	.	.	.
CFU-GEMM	E11.5 Endo+E11.5 Mesp1-PSC+Cytokines	CFU-GEMM	E11.5 Endo+E11.5 Mesp1-PSC+PDGF-AA	-0.1729	0	26	-Infty	<.0001	.	.	.
CFU-GEMM	E11.5 Endo+E11.5 Mesp1-PSC+Cytokines	CFU-GEMM	E11.5 Endo+E11.5 Wnt1-PSC+PDGF-AA	23.4274	6485.10	26	0.00	0.9971	0.05	-13307	13354
CFU-GEMM	E11.5 Endo+E11.5 Mesp1-PSC+Cytokines	CFU-GEMM	E11.5AGM+Cytokines	-0.5428	0	26	-Infty	<.0001	.	.	.

The GLIMMIX Procedure

Differences of Colony_Typ*Condition Least Squares Means											
Colony_Type	Condition	_Colony_Type	_Condition	Estimate	Standard Error	DF	t Value	Pr > |t|	Alpha	Lower	Upper
CFU-GEMM	E11.5 Endo+E11.5 Mesp1-PSC+Cytokines	CFU-GM	E11.5 Endo+E11.5 Mesp1-PSC	-1.9004	0	26	-Infty	<.0001	.	.	.
CFU-GEMM	E11.5 Endo+E11.5 Mesp1-PSC+Cytokines	CFU-GM	E11.5 Endo+E11.5 Mesp1-PSC+Cytokines	-1.3175	0	26	-Infty	<.0001	.	.	.
CFU-GEMM	E11.5 Endo+E11.5 Mesp1-PSC+Cytokines	CFU-GM	E11.5 Endo+E11.5 Mesp1-PSC+PDGF-AA	-1.7973	0	26	-Infty	<.0001	.	.	.
CFU-GEMM	E11.5 Endo+E11.5 Mesp1-PSC+Cytokines	CFU-GM	E11.5 Endo+E11.5 Wnt1-PSC+PDGF-AA	20.1757	1275.89	26	0.02	0.9875	0.05	-2602.46	2642.81
CFU-GEMM	E11.5 Endo+E11.5 Mesp1-PSC+Cytokines	CFU-GM	E11.5AGM+Cytokines	-1.8889	0	26	-Infty	<.0001	.	.	.
CFU-GEMM	E11.5 Endo+E11.5 Mesp1-PSC+PDGF-AA	CFU-GEMM	E11.5 Endo+E11.5 Wnt1-PSC+PDGF-AA	23.6003	6485.10	26	0.00	0.9971	0.05	-13307	13354
CFU-GEMM	E11.5 Endo+E11.5 Mesp1-PSC+PDGF-AA	CFU-GEMM	E11.5AGM+Cytokines	-0.3699	0	26	-Infty	<.0001	.	.	.
CFU-GEMM	E11.5 Endo+E11.5 Mesp1-PSC+PDGF-AA	CFU-GM	E11.5 Endo+E11.5 Mesp1-PSC	-1.7275	0	26	-Infty	<.0001	.	.	.
CFU-GEMM	E11.5 Endo+E11.5 Mesp1-PSC+PDGF-AA	CFU-GM	E11.5 Endo+E11.5 Mesp1-PSC+Cytokines	-1.1446	0	26	-Infty	<.0001	.	.	.
CFU-GEMM	E11.5 Endo+E11.5 Mesp1-PSC+PDGF-AA	CFU-GM	E11.5 Endo+E11.5 Mesp1-PSC+PDGF-AA	-1.6244	0	26	-Infty	<.0001	.	.	.
CFU-GEMM	E11.5 Endo+E11.5 Mesp1-PSC+PDGF-AA	CFU-GM	E11.5 Endo+E11.5 Wnt1-PSC+PDGF-AA	20.3486	1275.89	26	0.02	0.9874	0.05	-2602.28	2642.98
CFU-GEMM	E11.5 Endo+E11.5 Mesp1-PSC+PDGF-AA	CFU-GM	E11.5AGM+Cytokines	-1.7160	0	26	-Infty	<.0001	.	.	.
CFU-GEMM	E11.5 Endo+E11.5 Wnt1-PSC+PDGF-AA	CFU-GEMM	E11.5AGM+Cytokines	-23.9702	6485.10	26	-0.00	0.9971	0.05	-13354	13306
CFU-GEMM	E11.5 Endo+E11.5 Wnt1-PSC+PDGF-AA	CFU-GM	E11.5 Endo+E11.5 Mesp1-PSC	-25.3278	6485.10	26	-0.00	0.9969	0.05	-13356	13305
CFU-GEMM	E11.5 Endo+E11.5 Wnt1-PSC+PDGF-AA	CFU-GM	E11.5 Endo+E11.5 Mesp1-PSC+Cytokines	-24.7449	6485.10	26	-0.00	0.9970	0.05	-13355	13306
CFU-GEMM	E11.5 Endo+E11.5 Wnt1-PSC+PDGF-AA	CFU-GM	E11.5 Endo+E11.5 Mesp1-PSC+PDGF-AA	-25.2247	6485.10	26	-0.00	0.9969	0.05	-13356	13305
CFU-GEMM	E11.5 Endo+E11.5 Wnt1-PSC+PDGF-AA	CFU-GM	E11.5 Endo+E11.5 Wnt1-PSC+PDGF-AA	-3.2517	6609.42	26	-0.00	0.9996	0.05	-13589	13583
CFU-GEMM	E11.5 Endo+E11.5 Wnt1-PSC+PDGF-AA	CFU-GM	E11.5AGM+Cytokines	-25.3163	6485.10	26	-0.00	0.9969	0.05	-13356	13305
CFU-GEMM	E11.5AGM+Cytokines	CFU-GM	E11.5 Endo+E11.5 Mesp1-PSC	-1.3576	0	26	-Infty	<.0001	.	.	.
CFU-GEMM	E11.5AGM+Cytokines	CFU-GM	E11.5 Endo+E11.5 Mesp1-PSC+Cytokines	-0.7747	0	26	-Infty	<.0001	.	.	.
CFU-GEMM	E11.5AGM+Cytokines	CFU-GM	E11.5 Endo+E11.5 Mesp1-PSC+PDGF-AA	-1.2545	0	26	-Infty	<.0001	.	.	.
CFU-GEMM	E11.5AGM+Cytokines	CFU-GM	E11.5 Endo+E11.5 Wnt1-PSC+PDGF-AA	20.7185	1275.89	26	0.02	0.9872	0.05	-2601.91	2643.35
CFU-GEMM	E11.5AGM+Cytokines	CFU-GM	E11.5AGM+Cytokines	-1.3461	0	26	-Infty	<.0001	.	.	.
CFU-GM	E11.5 Endo+E11.5 Mesp1-PSC	CFU-GM	E11.5 Endo+E11.5 Mesp1-PSC+Cytokines	0.5829	0	26	Infty	<.0001	.	.	.

The GLIMMIX Procedure

Differences of Colony_Typ*Condition Least Squares Means											
Colony_Type	Condition	_Colony_Type	_Condition	Estimate	Standard Error	DF	t Value	Pr > |t|	Alpha	Lower	Upper
CFU-GM	E11.5 Endo+E11.5 Mesp1-PSC	CFU-GM	E11.5 Endo+E11.5 Mesp1-PSC+PDGF-AA	0.1031	0	26	Infty	<.0001	.	.	.
CFU-GM	E11.5 Endo+E11.5 Mesp1-PSC	CFU-GM	E11.5 Endo+E11.5 Wnt1-PSC+PDGF-AA	22.0761	1275.89	26	0.02	0.9863	0.05	-2600.55	2644.71
CFU-GM	E11.5 Endo+E11.5 Mesp1-PSC	CFU-GM	E11.5AGM+Cytokines	0.01150	0	26	Infty	<.0001	.	.	.
CFU-GM	E11.5 Endo+E11.5 Mesp1-PSC+Cytokines	CFU-GM	E11.5 Endo+E11.5 Mesp1-PSC+PDGF-AA	-0.4798	0	26	-Infty	<.0001	.	.	.
CFU-GM	E11.5 Endo+E11.5 Mesp1-PSC+Cytokines	CFU-GM	E11.5 Endo+E11.5 Wnt1-PSC+PDGF-AA	21.4932	1275.89	26	0.02	0.9867	0.05	-2601.14	2644.12
CFU-GM	E11.5 Endo+E11.5 Mesp1-PSC+Cytokines	CFU-GM	E11.5AGM+Cytokines	-0.5714	0	26	-Infty	<.0001	.	.	.
CFU-GM	E11.5 Endo+E11.5 Mesp1-PSC+PDGF-AA	CFU-GM	E11.5 Endo+E11.5 Wnt1-PSC+PDGF-AA	21.9730	1275.89	26	0.02	0.9864	0.05	-2600.66	2644.60
CFU-GM	E11.5 Endo+E11.5 Mesp1-PSC+PDGF-AA	CFU-GM	E11.5AGM+Cytokines	-0.09160	0	26	-Infty	<.0001	.	.	.
CFU-GM	E11.5 Endo+E11.5 Wnt1-PSC+PDGF-AA	CFU-GM	E11.5AGM+Cytokines	-22.0646	1275.89	26	-0.02	0.9863	0.05	-2644.70	2600.57

The GENMOD Procedure

Model Information	
**Data Set**	WORK.EXT_FIG8AIII
**Distribution**	Normal
**Link Function**	Identity
**Dependent Variable**	Percentage

**Number of Observations Read**	25
**Number of Observations Used**	25

Class Level Information		
**Class**	**Levels**	**Values**
**Condition**	2	E11.5AGM E11.5EC+E11.5PSC
**Treatment**	4	With Cyto+PDGF-AA With PDGF-AA Only no Cyto with Cyto

Parameter Information			
**Parameter**	**Effect**	**Condition**	**Treatment**
**Prm1**	Intercept		
**Prm2**	Condition	E11.5AGM	
**Prm3**	Condition	E11.5EC+E11.5PSC	
**Prm4**	Treatment		With Cyto+PDGF-AA
**Prm5**	Treatment		With PDGF-AA Only
**Prm6**	Treatment		no Cyto
**Prm7**	Treatment		with Cyto
**Prm8**	Condition*Treatment	E11.5AGM	with Cyto
**Prm9**	Condition*Treatment	E11.5EC+E11.5PSC	With Cyto+PDGF-AA
**Prm10**	Condition*Treatment	E11.5EC+E11.5PSC	With PDGF-AA Only

The GENMOD Procedure

Parameter Information			
**Parameter**	**Effect**	**Condition**	**Treatment**
**Prm11**	Condition*Treatment	E11.5EC+E11.5PSC	no Cyto
**Prm12**	Condition*Treatment	E11.5EC+E11.5PSC	with Cyto

Criteria For Assessing Goodness Of Fit			
**Criterion**	**DF**	**Value**	**Value/DF**
**Deviance**	20	688.4000	34.4200
**Scaled Deviance**	20	25.0000	1.2500
**Pearson Chi-Square**	20	688.4000	34.4200
**Scaled Pearson X2**	20	25.0000	1.2500
**Log Likelihood**		-76.9171	
**Full Log Likelihood**		-76.9171	
**AIC (smaller is better)**		165.8343	
**AICC (smaller is better)**		170.5009	
**BIC (smaller is better)**		173.1475	

Algorithm converged.

Analysis Of Maximum Likelihood Parameter Estimates									
**Parameter**			**DF**	**Estimate**	**Standard Error**	**Wald 95% Confidence Limits**		**Wald Chi-Square**	**Pr > ChiSq**
**Intercept**			1	33.8000	2.3467	29.2005	38.3995	207.44	<.0001
**Condition**	E11.5AGM		1	33.6000	3.3188	27.0953	40.1047	102.50	<.0001
**Condition**	E11.5EC+E11.5PSC		0	0.0000	0.0000	0.0000	0.0000	.	.
**Treatment**	With Cyto+PDGF-AA		1	7.6000	3.3188	1.0953	14.1047	5.24	0.0220
**Treatment**	With PDGF-AA Only		1	-23.4000	3.3188	-29.9047	-16.8953	49.71	<.0001

The GENMOD Procedure

Analysis Of Maximum Likelihood Parameter Estimates									
Parameter			DF	Estimate	Standard Error	Wald 95% Confidence Limits		Wald Chi-Square	Pr > ChiSq
Treatment	no Cyto		1	-33.8000	3.3188	-40.3047	-27.2953	103.72	<.0001
Treatment	with Cyto		0	0.0000	0.0000	0.0000	0.0000	.	.
Condition*Treatment	E11.5AGM	with Cyto	0	0.0000	0.0000	0.0000	0.0000	.	.
Condition*Treatment	E11.5EC+E11.5PSC	With Cyto+PDGF-AA	0	0.0000	0.0000	0.0000	0.0000	.	.
Condition*Treatment	E11.5EC+E11.5PSC	With PDGF-AA Only	0	0.0000	0.0000	0.0000	0.0000	.	.
Condition*Treatment	E11.5EC+E11.5PSC	no Cyto	0	0.0000	0.0000	0.0000	0.0000	.	.
Condition*Treatment	E11.5EC+E11.5PSC	with Cyto	0	0.0000	0.0000	0.0000	0.0000	.	.
Scale			1	5.2475	0.7421	3.9772	6.9235		

Note: The scale parameter was estimated by maximum likelihood.

Condition*Treatment Least Squares Means								
Condition	Treatment	Estimate	Standard Error	z Value	Pr > |z|	Alpha	Lower	Upper
E11.5AGM	with Cyto	67.4000	2.3467	28.72	<.0001	0.05	62.8005	71.9995
E11.5EC+E11.5PSC	With Cyto+PDGF-AA	41.4000	2.3467	17.64	<.0001	0.05	36.8005	45.9995
E11.5EC+E11.5PSC	With PDGF-AA Only	10.4000	2.3467	4.43	<.0001	0.05	5.8005	14.9995
E11.5EC+E11.5PSC	no Cyto	0	2.3467	0.00	1.0000	0.05	-4.5995	4.5995
E11.5EC+E11.5PSC	with Cyto	33.8000	2.3467	14.40	<.0001	0.05	29.2005	38.3995

Differences of Condition*Treatment Least Squares Means										
Condition	Treatment	_Condition	_Treatment	Estimate	Standard Error	z Value	Pr > |z|	Alpha	Lower	Upper
E11.5AGM	with Cyto	E11.5EC+E11.5PSC	With Cyto+PDGF-AA	26.0000	3.3188	7.83	<.0001	0.05	19.4953	32.5047
E11.5AGM	with Cyto	E11.5EC+E11.5PSC	With PDGF-AA Only	57.0000	3.3188	17.17	<.0001	0.05	50.4953	63.5047
E11.5AGM	with Cyto	E11.5EC+E11.5PSC	no Cyto	67.4000	3.3188	20.31	<.0001	0.05	60.8953	73.9047
E11.5AGM	with Cyto	E11.5EC+E11.5PSC	with Cyto	33.6000	3.3188	10.12	<.0001	0.05	27.0953	40.1047

The GENMOD Procedure

Differences of Condition*Treatment Least Squares Means										
Condition	Treatment	_Condition	_Treatment	Estimate	Standard Error	z Value	Pr > |z|	Alpha	Lower	Upper
E11.5EC+E11.5PSC	With Cyto+PDGF-AA	E11.5EC+E11.5PSC	With PDGF-AA Only	31.0000	3.3188	9.34	<.0001	0.05	24.4953	37.5047
E11.5EC+E11.5PSC	With Cyto+PDGF-AA	E11.5EC+E11.5PSC	no Cyto	41.4000	3.3188	12.47	<.0001	0.05	34.8953	47.9047
E11.5EC+E11.5PSC	With Cyto+PDGF-AA	E11.5EC+E11.5PSC	with Cyto	7.6000	3.3188	2.29	0.0220	0.05	1.0953	14.1047
E11.5EC+E11.5PSC	With PDGF-AA Only	E11.5EC+E11.5PSC	no Cyto	10.4000	3.3188	3.13	0.0017	0.05	3.8953	16.9047
E11.5EC+E11.5PSC	With PDGF-AA Only	E11.5EC+E11.5PSC	with Cyto	-23.4000	3.3188	-7.05	<.0001	0.05	-29.9047	-16.8953
E11.5EC+E11.5PSC	no Cyto	E11.5EC+E11.5PSC	with Cyto	-33.8000	3.3188	-10.18	<.0001	0.05	-40.3047	-27.2953

The GENMOD Procedure

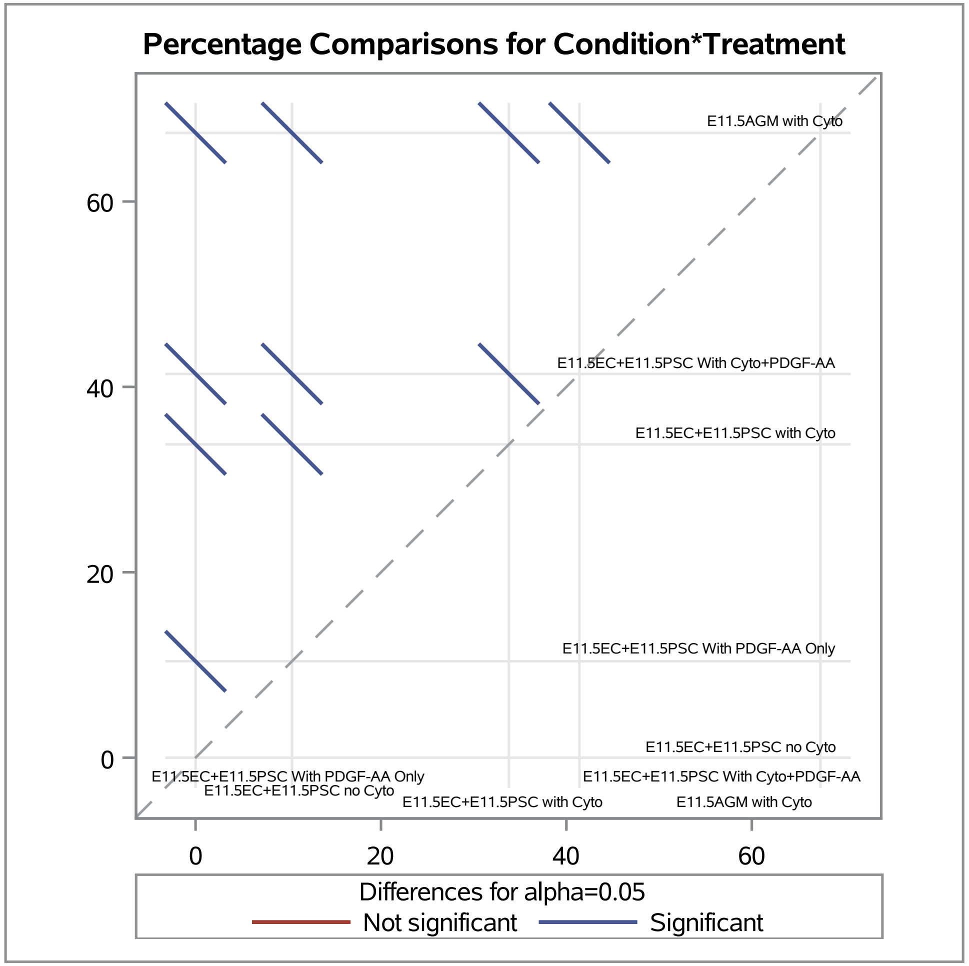

The GLIMMIX Procedure

Model Information	
**Data Set**	WORK.EXT_FIG8DII
**Response Variable**	Count
**Response Distribution**	Poisson
**Link Function**	Log
**Variance Function**	Default
**Variance Matrix Blocked By**	Dish_ID
**Estimation Technique**	Maximum Likelihood
**Likelihood Approximation**	Laplace
**Degrees of Freedom Method**	Containment

Class Level Information		
**Class**	**Levels**	**Values**
**Colony_Size**	3	Large Micro Small
**Condition**	3	AoD AoV Whole AGM
**Dish_ID**	9	1 2 3 4 5 6 7 8 9

**Number of Observations Read**	27
**Number of Observations Used**	27

Dimensions	
**G-side Cov. Parameters**	1
**Columns in X**	16
**Columns in Z per Subject**	1
**Subjects (Blocks in V)**	9
**Max Obs per Subject**	3

The GLIMMIX Procedure

Optimization Information	
**Optimization Technique**	Dual Quasi-Newton
**Parameters in Optimization**	10
**Lower Boundaries**	1
**Upper Boundaries**	0
**Fixed Effects**	Not Profiled
**Starting From**	GLM estimates

Iteration History					
**Iteration**	**Restarts**	**Evaluations**	**Objective Function**	**Change**	**Max Gradient**
**0**	**0**	4	164.21560755	.	61.4012
**1**	**0**	5	163.9767917	0.23881586	10.66601
**2**	**0**	4	163.96576045	0.01103125	10.63736
**3**	**0**	3	163.9648266	0.00093384	9.821369
**4**	**0**	2	163.9633534	0.00147321	7.47076
**5**	**0**	2	163.96114738	0.00220601	1.802888
**6**	**0**	3	163.96093293	0.00021445	0.847951
**7**	**0**	3	163.96085436	0.00007857	0.328883
**8**	**0**	2	163.96078681	0.00006755	0.347201
**9**	**0**	3	163.96076988	0.00001693	0.315556
**10**	**0**	4	163.96071678	0.00005310	0.035415
**11**	**0**	4	163.96058183	0.00013494	0.645624
**12**	**0**	6	163.94837752	0.01220432	0.217803
**13**	**0**	3	163.94724036	0.00113715	0.038251
**14**	**0**	2	163.94589294	0.00134742	0.930331
**15**	**0**	4	163.94232055	0.00357239	0.585592

The GLIMMIX Procedure

Iteration History					
**Iteration**	**Restarts**	**Evaluations**	**Objective Function**	**Change**	**Max Gradient**
**16**	**0**	3	163.94129963	0.00102091	0.730211
**17**	**0**	3	163.94091948	0.00038016	0.129547
**18**	**0**	2	163.94070799	0.00021149	0.462181
**19**	**0**	3	163.94064227	0.00006572	0.009998
**20**	**0**	2	163.9406347	0.00000757	0.068321

Convergence criterion (GCONV=1E-8) satisfied.

Fit Statistics	
**-2 Log Likelihood**	163.94
**AIC (smaller is better)**	183.94
**AICC (smaller is better)**	197.69
**BIC (smaller is better)**	185.91
**CAIC (smaller is better)**	195.91
**HQIC (smaller is better)**	179.68

Fit Statistics for Conditional Distribution	
**-2 log L(Count | r. effects)**	151.83
**Pearson Chi-Square**	23.23
**Pearson Chi-Square / DF**	0.86

Covariance Parameter Estimates			
**Cov Parm**	**Subject**	**Estimate**	**Standard Error**
**Intercept**	Dish_ID	0.01278	.

The GLIMMIX Procedure

Solutions for Fixed Effects

Effect	Colony_Size	Condition	Estimate	Standard Error	DF	t Value	Pr > |t|
**Intercept**			3.6755	0.1126	6	32.64	<.0001
**Colony_Size**	Large		-0.09698	0.1329	12	-0.73	0.4796
**Colony_Size**	Micro		0.6008	0.1141	12	5.27	0.0002
**Colony_Size**	Small		0	.	.	.	.
**Condition**		AoD	-0.6868	0.1834	12	-3.74	0.0028
**Condition**		AoV	-0.4375	0.1731	12	-2.53	0.0265
**Condition**		Whole AGM	0	.	.	.	.
**Colony_Siz*Condition**	Large	AoD	-17.3731	802.58	12	-0.02	0.9831
**Colony_Siz*Condition**	Large	AoV	0.1840	0.2063	12	0.89	0.3900
**Colony_Siz*Condition**	Large	Whole AGM	0	.	.	.	.
**Colony_Siz*Condition**	Micro	AoD	0.1089	0.1946	12	0.56	0.5862
**Colony_Siz*Condition**	Micro	AoV	-0.1997	0.1863	12	-1.07	0.3048
**Colony_Siz*Condition**	Micro	Whole AGM	0	.	.	.	.
**Colony_Siz*Condition**	Small	AoD	0	.	.	.	.
**Colony_Siz*Condition**	Small	AoV	0	.	.	.	.
**Colony_Siz*Condition**	Small	Whole AGM	0	.	.	.	.

Type III Tests of Fixed Effects

Effect	Num DF	Den DF	F Value	Pr > F
**Colony_Size**	2	12	24.59	<.0001
**Condition**	2	12	6.64	0.0115
**Colony_Siz*Condition**	4	12	1.34	0.3128

The GLIMMIX Procedure

Condition Least Squares Means												
Condition	Estimate	Standard Error	DF	t Value	Pr > |t|	Alpha	Lower	Upper	Mean	Standard Error Mean	Lower Mean	Upper Mean
AoD	-2.5980	267.53	12	-0.01	0.9924	0.05	-585.49	580.29	0.07442	19.9092	532E-257	1.04E252
AoV	3.4008	0.08964	12	37.94	<.0001	0.05	3.2055	3.5961	29.9872	2.6882	24.6667	36.4553
Whole AGM	3.8435	0.08217	12	46.78	<.0001	0.05	3.6644	4.0225	46.6870	3.8361	39.0343	55.8400

Differences of Condition Least Squares Means									
Condition	_Condition	Estimate	Standard Error	DF	t Value	Pr > |t|	Alpha	Lower	Upper
AoD	AoV	-5.9988	267.53	12	-0.02	0.9825	0.05	-588.89	576.89
AoD	Whole AGM	-6.4415	267.53	12	-0.02	0.9812	0.05	-589.33	576.45
AoV	Whole AGM	-0.4427	0.1215	12	-3.64	0.0034	0.05	-0.7074	-0.1779

Colony_Siz*Condition Least Squares Means													
Colony_Size	Condition	Estimate	Standard Error	DF	t Value	Pr > |t|	Alpha	Lower	Upper	Mean	Standard Error Mean	Lower Mean	Upper Mean
Large	AoD	-14.4813	802.58	12	-0.02	0.9859	0.05	-1763.15	1734.19	5.139E-7	0.000412	0	.
Large	AoV	3.3251	0.1273	12	26.12	<.0001	0.05	3.0477	3.6025	27.8009	3.5394	21.0663	36.6884
Large	Whole AGM	3.5786	0.1163	12	30.76	<.0001	0.05	3.3251	3.8320	35.8219	4.1676	27.8009	46.1570
Micro	AoD	3.6984	0.1118	12	33.07	<.0001	0.05	3.4547	3.9421	40.3836	4.5166	31.6501	51.5272
Micro	AoV	3.6392	0.1140	12	31.92	<.0001	0.05	3.3907	3.8876	38.0603	4.3395	29.6882	48.7933
Micro	Whole AGM	4.2763	0.09426	12	45.37	<.0001	0.05	4.0709	4.4817	71.9740	6.7843	58.6114	88.3831
Small	AoD	2.9888	0.1448	12	20.64	<.0001	0.05	2.6732	3.3044	19.8615	2.8767	14.4863	27.2310
Small	AoV	3.2381	0.1315	12	24.63	<.0001	0.05	2.9516	3.5246	25.4846	3.3511	19.1360	33.9394
Small	Whole AGM	3.6755	0.1126	12	32.64	<.0001	0.05	3.4302	3.9209	39.4698	4.4445	30.8825	50.4449

The GLIMMIX Procedure

Differences of Colony_Siz*Condition Least Squares Means											
Colony_Size	Condition	_Colony_Size	_Condition	Estimate	Standard Error	DF	t Value	Pr > |t|	Alpha	Lower	Upper
Large	AoD	Large	AoV	-17.8064	802.58	12	-0.02	0.9827	0.05	-1766.47	1730.86
Large	AoD	Large	Whole AGM	-18.0599	802.58	12	-0.02	0.9824	0.05	-1766.73	1730.61
Large	AoD	Micro	AoD	-18.1797	802.58	12	-0.02	0.9823	0.05	-1766.85	1730.49
Large	AoD	Micro	AoV	-18.1205	802.58	12	-0.02	0.9824	0.05	-1766.79	1730.55
Large	AoD	Micro	Whole AGM	-18.7576	802.58	12	-0.02	0.9817	0.05	-1767.43	1729.91
Large	AoD	Small	AoD	-17.4701	802.58	12	-0.02	0.9830	0.05	-1766.14	1731.20
Large	AoD	Small	AoV	-17.7194	802.58	12	-0.02	0.9827	0.05	-1766.39	1730.95
Large	AoD	Small	Whole AGM	-18.1568	802.58	12	-0.02	0.9823	0.05	-1766.83	1730.51
Large	AoV	Large	Whole AGM	-0.2535	0.1724	12	-1.47	0.1672	0.05	-0.6291	0.1221
Large	AoV	Micro	AoD	-0.3734	0.1693	12	-2.21	0.0477	0.05	-0.7423	-0.00445
Large	AoV	Micro	AoV	-0.3141	0.1435	12	-2.19	0.0491	0.05	-0.6268	-0.00138
Large	AoV	Micro	Whole AGM	-0.9512	0.1583	12	-6.01	<.0001	0.05	-1.2962	-0.6062
Large	AoV	Small	AoD	0.3363	0.1927	12	1.75	0.1065	0.05	-0.08359	0.7562
Large	AoV	Small	AoV	0.08699	0.1578	12	0.55	0.5915	0.05	-0.2568	0.4307
Large	AoV	Small	Whole AGM	-0.3505	0.1699	12	-2.06	0.0615	0.05	-0.7206	0.01971
Large	Whole AGM	Micro	AoD	-0.1199	0.1613	12	-0.74	0.4717	0.05	-0.4713	0.2316
Large	Whole AGM	Micro	AoV	-0.06061	0.1628	12	-0.37	0.7162	0.05	-0.4154	0.2942
Large	Whole AGM	Micro	Whole AGM	-0.6977	0.1178	12	-5.93	<.0001	0.05	-0.9543	-0.4412
Large	Whole AGM	Small	AoD	0.5898	0.1857	12	3.18	0.0080	0.05	0.1851	0.9944
Large	Whole AGM	Small	AoV	0.3405	0.1755	12	1.94	0.0762	0.05	-0.04192	0.7229
Large	Whole AGM	Small	Whole AGM	-0.09698	0.1329	12	-0.73	0.4796	0.05	-0.3865	0.1926
Micro	AoD	Micro	AoV	0.05925	0.1596	12	0.37	0.7168	0.05	-0.2884	0.4069
Micro	AoD	Micro	Whole AGM	-0.5779	0.1462	12	-3.95	0.0019	0.05	-0.8964	-0.2594
Micro	AoD	Small	AoD	0.7096	0.1577	12	4.50	0.0007	0.05	0.3661	1.0532

The GLIMMIX Procedure

Differences of Colony_Siz*Condition Least Squares Means											
Colony_Size	Condition	_Colony_Size	_Condition	Estimate	Standard Error	DF	t Value	Pr > |t|	Alpha	Lower	Upper
Micro	AoD	Small	AoV	0.4604	0.1725	12	2.67	0.0204	0.05	0.08455	0.8362
Micro	AoD	Small	Whole AGM	0.02289	0.1586	12	0.14	0.8877	0.05	-0.3227	0.3685
Micro	AoV	Micro	Whole AGM	-0.6371	0.1479	12	-4.31	0.0010	0.05	-0.9593	-0.3150
Micro	AoV	Small	AoD	0.6504	0.1842	12	3.53	0.0041	0.05	0.2491	1.0517
Micro	AoV	Small	AoV	0.4011	0.1472	12	2.72	0.0185	0.05	0.08027	0.7219
Micro	AoV	Small	Whole AGM	-0.03636	0.1602	12	-0.23	0.8242	0.05	-0.3854	0.3126
Micro	Whole AGM	Small	AoD	1.2875	0.1727	12	7.45	<.0001	0.05	0.9112	1.6639
Micro	Whole AGM	Small	AoV	1.0382	0.1617	12	6.42	<.0001	0.05	0.6859	1.3906
Micro	Whole AGM	Small	Whole AGM	0.6008	0.1141	12	5.27	0.0002	0.05	0.3522	0.8493
Small	AoD	Small	AoV	-0.2493	0.1955	12	-1.28	0.2264	0.05	-0.6752	0.1767
Small	AoD	Small	Whole AGM	-0.6868	0.1834	12	-3.74	0.0028	0.05	-1.0863	-0.2872
Small	AoV	Small	Whole AGM	-0.4375	0.1731	12	-2.53	0.0265	0.05	-0.8145	-0.06041

The Mixed Procedure

Model Information	
**Data Set**	WORK.EXT_FIG8DIII
**Dependent Variable**	log_agg
**Covariance Structure**	Variance Components
**Subject Effect**	Replicate
**Estimation Method**	REML
**Residual Variance Method**	Profile
**Fixed Effects SE Method**	Model-Based
**Degrees of Freedom Method**	Containment

Class Level Information		
**Class**	**Levels**	**Values**
**Replicate**	3	1 2 3
**Group**	3	AoD AoV Whole AGM

Dimensions	
**Covariance Parameters**	3
**Columns in X**	8
**Columns in Z per Subject**	2
**Subjects**	3
**Max Obs per Subject**	30

Number of Observations	
**Number of Observations Read**	117
**Number of Observations Used**	90
**Number of Observations Not Used**	27

The Mixed Procedure

Iteration History			
Iteration	Evaluations	-2 Res Log Like	Criterion
0	1	120.69048920	
1	3	119.98227291	0.00003769
2	1	119.98160404	0.00000009
3	1	119.98160244	0.00000000

Convergence criteria met.

Estimated G matrix is not positive definite.

Covariance Parameter Estimates		
Cov Parm	Subject	Estimate
Intercept	Replicate	0
Days	Replicate	1.119E-6
Residual		0.1502

Fit Statistics	
-2 Res Log Likelihood	120.0
AIC (Smaller is Better)	124.0
AICC (Smaller is Better)	124.1
BIC (Smaller is Better)	122.2

The Mixed Procedure

Solution for Fixed Effects						
Effect	Group	Estimate	Standard Error	DF	t Value	Pr > |t|
Intercept		3.8115	0.1247	2	30.56	0.0011
Days		0.1053	0.001760	2	59.85	0.0003
Group	AoD	0.06520	0.2250	80	0.29	0.7727
Group	AoV	-0.1245	0.1764	80	-0.71	0.4822
Group	Whole AGM	0	.	.	.	.
Days*Group	AoD	-0.06702	0.008501	80	-7.88	<.0001
Days*Group	AoV	-0.00832	0.002334	80	-3.56	0.0006
Days*Group	Whole AGM	0	.	.	.	.

Type 3 Tests of Fixed Effects				
Effect	Num DF	Den DF	F Value	Pr > F
Days	1	2	738.97	0.0014
Group	2	80	0.44	0.6463
Days*Group	2	80	34.21	<.0001

Estimates					
Label	Estimate	Standard Error	DF	t Value	Pr > |t|
AoD slope	0.03830	0.008362	80	4.58	<.0001
AoV slope	0.09701	0.001760	80	55.12	<.0001
Whole AGM slope	0.1053	0.001760	80	59.85	<.0001
AoD vs AoV slope	-0.05870	0.008501	80	-6.91	<.0001
AoD vs Whole AGM slope	-0.06702	0.008501	80	-7.88	<.0001
AoV vs Whole AGM slope	-0.00832	0.002334	80	-3.56	0.0006

The GLIMMIX Procedure

Model Information	
**Data Set**	WORK.EXT_FIG8DIV
**Response Variable**	Count
**Response Distribution**	Poisson
**Link Function**	Log
**Variance Function**	Default
**Variance Matrix Blocked By**	Dish_ID
**Estimation Technique**	Maximum Likelihood
**Likelihood Approximation**	Laplace
**Degrees of Freedom Method**	Containment

Class Level Information		
**Class**	**Levels**	**Values**
**Colony_Type**	3	BFU-E CFU-GEMM CFU-GM
**Condition**	6	Adult (H) Endo+AoD Adult (H) Endo+Aov Adult Endo (H) only E11.5 Endo only E11.5 endo+AoD E11.5 endo+AoV
**Dish_ID**	18	1 2 3 4 5 6 7 8 9 10 11 12 13 14 15 16 17 18

**Number of Observations Read**	54
**Number of Observations Used**	54

Dimensions	
**G-side Cov. Parameters**	1
**Columns in X**	28
**Columns in Z per Subject**	1
**Subjects (Blocks in V)**	18
**Max Obs per Subject**	3

The GLIMMIX Procedure

Optimization Information	
Optimization Technique	Dual Quasi-Newton
Parameters in Optimization	19
Lower Boundaries	1
Upper Boundaries	0
Fixed Effects	Not Profiled
Starting From	GLM estimates

Iteration History

Iteration	Restarts	Evaluations	Objective Function	Change	Max Gradient
0	0	4	165.23171281	.	289.245
1	0	5	165.00602995	0.22568287	72.74238
2	0	3	164.99817724	0.00785270	4.228174
3	0	4	164.99768365	0.00049359	16.8258
4	0	4	164.9954778	0.00220585	0.914944
5	0	2	164.99547285	0.00000495	0.566896
6	0	4	164.99546283	0.00001002	2.260324
7	0	4	164.99535015	0.00011268	3.556296
8	0	2	164.99517817	0.00017198	1.092975
9	0	3	164.99513548	0.00004269	0.995472
10	0	3	164.99513194	0.00000354	0.249329
11	0	4	164.99508374	0.00004820	0.848611

Convergence criterion (GCONV=1E-8) satisfied.

The GLIMMIX Procedure

Fit Statistics	
-2 Log Likelihood	165.00
AIC (smaller is better)	203.00
AICC (smaller is better)	225.35
BIC (smaller is better)	219.91
CAIC (smaller is better)	238.91
HQIC (smaller is better)	205.33

Fit Statistics for Conditional Distribution	
-2 log L(Count | r. effects)	154.04
Pearson Chi-Square	26.36
Pearson Chi-Square / DF	0.49

Covariance Parameter Estimates

Cov Parm	Subject	Estimate	Standard Error
Intercept	Dish_ID	0.002690	0.002265

Solutions for Fixed Effects

Effect	Colony_Type	Condition	Estimate	Standard Error	DF	t Value	Pr > |t|
Intercept			6.4404	0.03780	12	170.38	<.0001
Colony_Type	BFU-E		-1.2830	0.04947	24	-25.94	<.0001
Colony_Type	CFU-GEMM		-1.4886	0.05371	24	-27.72	<.0001
Colony_Type	CFU-GM		0	.	.	.	.
Condition		Adult (H) Endo+AoD	-12.1353	9.9493	24	-1.22	0.2344
Condition		Adult (H) Endo+Aov	-0.2058	0.05457	24	-3.77	0.0009

The GLIMMIX Procedure

Solutions for Fixed Effects							
**Effect**	**Colony_Type**	**Condition**	**Estimate**	**Standard Error**	**DF**	**t Value**	**Pr > |t|**
**Condition**		Adult Endo (H) only	-12.1353	9.9493	24	-1.22	0.2344
**Condition**		E11.5 Endo only	-12.1353	9.9493	24	-1.22	0.2344
**Condition**		E11.5 endo+AoD	-12.1353	9.9493	24	-1.22	0.2344
**Condition**		E11.5 endo+AoV	0	.	.	.	.
**Colony_Typ*Condition**	BFU-E	Adult (H) Endo+AoD	1.2829	14.0710	24	0.09	0.9281
**Colony_Typ*Condition**	BFU-E	Adult (H) Endo+Aov	-0.2460	0.07818	24	-3.15	0.0044
**Colony_Typ*Condition**	BFU-E	Adult Endo (H) only	1.2829	14.0710	24	0.09	0.9281
**Colony_Typ*Condition**	BFU-E	E11.5 Endo only	1.2829	14.0710	24	0.09	0.9281
**Colony_Typ*Condition**	BFU-E	E11.5 endo+AoD	1.2829	14.0710	24	0.09	0.9281
**Colony_Typ*Condition**	BFU-E	E11.5 endo+AoV	0	.	.	.	.
**Colony_Typ*Condition**	CFU-GEMM	Adult (H) Endo+AoD	1.4885	14.0711	24	0.11	0.9166
**Colony_Typ*Condition**	CFU-GEMM	Adult (H) Endo+Aov	-0.8621	0.1019	24	-8.46	<.0001
**Colony_Typ*Condition**	CFU-GEMM	Adult Endo (H) only	1.4885	14.0711	24	0.11	0.9166
**Colony_Typ*Condition**	CFU-GEMM	E11.5 Endo only	1.4885	14.0711	24	0.11	0.9166
**Colony_Typ*Condition**	CFU-GEMM	E11.5 endo+AoD	1.4885	14.0711	24	0.11	0.9166
**Colony_Typ*Condition**	CFU-GEMM	E11.5 endo+AoV	0	.	.	.	.
**Colony_Typ*Condition**	CFU-GM	Adult (H) Endo+AoD	0	.	.	.	.
**Colony_Typ*Condition**	CFU-GM	Adult (H) Endo+Aov	0	.	.	.	.
**Colony_Typ*Condition**	CFU-GM	Adult Endo (H) only	0	.	.	.	.
**Colony_Typ*Condition**	CFU-GM	E11.5 Endo only	0	.	.	.	.
**Colony_Typ*Condition**	CFU-GM	E11.5 endo+AoD	0	.	.	.	.
**Colony_Typ*Condition**	CFU-GM	E11.5 endo+AoV	0	.	.	.	.

The GLIMMIX Procedure

Type III Tests of Fixed Effects				
Effect	Num DF	Den DF	F Value	Pr > F
Colony_Type	2	24	0.01	0.9901
Condition	5	24	21.85	<.0001
Colony_Typ*Condition	10	24	7.53	<.0001

Condition Least Squares Means												
Condition	Estimate	Standard Error	DF	t Value	Pr > |t|	Alpha	Lower	Upper	Mean	Standard Error Mean	Lower Mean	Upper Mean
Adult (H) Endo+AoD	-5.6950	5.7445	24	-0.99	0.3314	0.05	-17.5512	6.1611	0.003363	0.01932	2.386E-8	473.93
Adult (H) Endo+Aov	4.9413	0.04545	24	108.72	<.0001	0.05	4.8475	5.0351	139.95	6.3605	127.42	153.71
Adult Endo (H) only	-5.6950	5.7445	24	-0.99	0.3314	0.05	-17.5512	6.1611	0.003363	0.01932	2.386E-8	473.93
E11.5 Endo only	-5.6950	5.7445	24	-0.99	0.3314	0.05	-17.5512	6.1611	0.003363	0.01932	2.386E-8	473.93
E11.5 endo+AoD	-5.6950	5.7445	24	-0.99	0.3314	0.05	-17.5512	6.1611	0.003363	0.01932	2.386E-8	473.93
E11.5 endo+AoV	5.5165	0.03783	24	145.82	<.0001	0.05	5.4384	5.5945	248.75	9.4105	230.07	268.95

Differences of Condition Least Squares Means									
Condition	_Condition	Estimate	Standard Error	DF	t Value	Pr > |t|	Alpha	Lower	Upper
Adult (H) Endo+AoD	Adult (H) Endo+Aov	-10.6363	5.7447	24	-1.85	0.0764	0.05	-22.4928	1.2202
Adult (H) Endo+AoD	Adult Endo (H) only	6E-15	8.1240	24	0.00	1.0000	0.05	-16.7671	16.7671
Adult (H) Endo+AoD	E11.5 Endo only	6.88E-15	8.1240	24	0.00	1.0000	0.05	-16.7671	16.7671
Adult (H) Endo+AoD	E11.5 endo+AoD	5.05E-15	8.1240	24	0.00	1.0000	0.05	-16.7671	16.7671
Adult (H) Endo+AoD	E11.5 endo+AoV	-11.2115	5.7446	24	-1.95	0.0627	0.05	-23.0679	0.6449
Adult (H) Endo+Aov	Adult Endo (H) only	10.6363	5.7447	24	1.85	0.0764	0.05	-1.2202	22.4928
Adult (H) Endo+Aov	E11.5 Endo only	10.6363	5.7447	24	1.85	0.0764	0.05	-1.2202	22.4928
Adult (H) Endo+Aov	E11.5 endo+AoD	10.6363	5.7447	24	1.85	0.0764	0.05	-1.2202	22.4928

The GLIMMIX Procedure

Differences of Condition Least Squares Means									
Condition	_Condition	Estimate	Standard Error	DF	t Value	Pr > |t|	Alpha	Lower	Upper
Adult (H) Endo+Aov	E11.5 endo+AoV	-0.5752	0.05913	24	-9.73	<.0001	0.05	-0.6972	-0.4532
Adult Endo (H) only	E11.5 Endo only	8.88E-16	8.1240	24	0.00	1.0000	0.05	-16.7671	16.7671
Adult Endo (H) only	E11.5 endo+AoD	-944E-18	8.1240	24	-0.00	1.0000	0.05	-16.7671	16.7671
Adult Endo (H) only	E11.5 endo+AoV	-11.2115	5.7446	24	-1.95	0.0627	0.05	-23.0679	0.6449
E11.5 Endo only	E11.5 endo+AoD	-183E-17	8.1240	24	-0.00	1.0000	0.05	-16.7671	16.7671
E11.5 Endo only	E11.5 endo+AoV	-11.2115	5.7446	24	-1.95	0.0627	0.05	-23.0679	0.6449
E11.5 endo+AoD	E11.5 endo+AoV	-11.2115	5.7446	24	-1.95	0.0627	0.05	-23.0679	0.6449

Colony_Typ*Condition Least Squares Means													
Colony_Type	Condition	Estimate	Standard Error	DF	t Value	Pr > |t|	Alpha	Lower	Upper	Mean	Standard Error Mean	Lower Mean	Upper Mean
BFU-E	Adult (H) Endo+AoD	-5.6951	9.9501	24	-0.57	0.5724	0.05	-26.2310	14.8408	0.003362	0.03346	4.06E-12	2788023
BFU-E	Adult (H) Endo+Aov	4.7054	0.06252	24	75.26	<.0001	0.05	4.5764	4.8345	110.55	6.9118	97.1633	125.77
BFU-E	Adult Endo (H) only	-5.6951	9.9501	24	-0.57	0.5724	0.05	-26.2310	14.8408	0.003362	0.03346	4.06E-12	2788023
BFU-E	E11.5 Endo only	-5.6951	9.9501	24	-0.57	0.5724	0.05	-26.2310	14.8408	0.003362	0.03346	4.06E-12	2788023
BFU-E	E11.5 endo+AoD	-5.6951	9.9501	24	-0.57	0.5724	0.05	-26.2310	14.8408	0.003362	0.03346	4.06E-12	2788023
BFU-E	E11.5 endo+AoV	5.1573	0.05304	24	97.23	<.0001	0.05	5.0478	5.2668	173.70	9.2136	155.69	193.79
CFU-GEMM	Adult (H) Endo+AoD	-5.6951	9.9502	24	-0.57	0.5724	0.05	-26.2313	14.8411	0.003362	0.03346	4.05E-12	2788602
CFU-GEMM	Adult (H) Endo+Aov	3.8838	0.08802	24	44.13	<.0001	0.05	3.7021	4.0655	48.6088	4.2784	40.5343	58.2919
CFU-GEMM	Adult Endo (H) only	-5.6951	9.9502	24	-0.57	0.5724	0.05	-26.2313	14.8411	0.003362	0.03346	4.05E-12	2788602
CFU-GEMM	E11.5 Endo only	-5.6951	9.9502	24	-0.57	0.5724	0.05	-26.2313	14.8411	0.003362	0.03346	4.05E-12	2788602
CFU-GEMM	E11.5 endo+AoD	-5.6951	9.9502	24	-0.57	0.5724	0.05	-26.2313	14.8411	0.003362	0.03346	4.05E-12	2788602
CFU-GEMM	E11.5 endo+AoV	4.9517	0.05702	24	86.85	<.0001	0.05	4.8340	5.0694	141.42	8.0633	125.72	159.08
CFU-GM	Adult (H) Endo+AoD	-5.6949	9.9492	24	-0.57	0.5724	0.05	-26.2291	14.8392	0.003363	0.03346	4.06E-12	2783462

The GLIMMIX Procedure

Colony_Typ*Condition Least Squares Means													
Colony_Type	Condition	Estimate	Standard Error	DF	t Value	Pr > |t|	Alpha	Lower	Upper	Mean	Standard Error Mean	Lower Mean	Upper Mean
CFU-GM	Adult (H) Endo+Aov	6.2345	0.03937	24	158.35	<.0001	0.05	6.1533	6.3158	510.06	20.0826	470.25	553.24
CFU-GM	Adult Endo (H) only	-5.6949	9.9492	24	-0.57	0.5724	0.05	-26.2291	14.8392	0.003363	0.03346	4.06E-12	2783462
CFU-GM	E11.5 Endo only	-5.6949	9.9492	24	-0.57	0.5724	0.05	-26.2291	14.8392	0.003363	0.03346	4.06E-12	2783462
CFU-GM	E11.5 endo+AoD	-5.6949	9.9492	24	-0.57	0.5724	0.05	-26.2291	14.8392	0.003363	0.03346	4.06E-12	2783462
CFU-GM	E11.5 endo+AoV	6.4404	0.03780	24	170.38	<.0001	0.05	6.3623	6.5184	626.63	23.6868	579.60	677.48

The GLIMMIX Procedure

Differences of Colony_Typ*Condition Least Squares Means											
Colony_Type	Condition	_Colony_Type	_Condition	Estimate	Standard Error	DF	t Value	Pr > |t|	Alpha	Lower	Upper
BFU-E	Adult (H) Endo+AoD	BFU-E	Adult (H) Endo+Aov	-10.4005	9.9503	24	-1.05	0.3063	0.05	-30.9369	10.1358
BFU-E	Adult (H) Endo+AoD	BFU-E	Adult Endo (H) only	5.77E-15	14.0715	24	0.00	1.0000	0.05	-29.0422	29.0422
BFU-E	Adult (H) Endo+AoD	BFU-E	E11.5 Endo only	6.66E-15	14.0715	24	0.00	1.0000	0.05	-29.0422	29.0422
BFU-E	Adult (H) Endo+AoD	BFU-E	E11.5 endo+AoD	4E-15	14.0715	24	0.00	1.0000	0.05	-29.0422	29.0422
BFU-E	Adult (H) Endo+AoD	BFU-E	E11.5 endo+AoV	-10.8524	9.9502	24	-1.09	0.2862	0.05	-31.3886	9.6838
BFU-E	Adult (H) Endo+AoD	CFU-GEMM	Adult (H) Endo+AoD	0.000023	14.0715	24	0.00	1.0000	0.05	-29.0422	29.0422
BFU-E	Adult (H) Endo+AoD	CFU-GEMM	Adult (H) Endo+Aov	-9.5789	9.9505	24	-0.96	0.3453	0.05	-30.1156	10.9578
BFU-E	Adult (H) Endo+AoD	CFU-GEMM	Adult Endo (H) only	0.000023	14.0716	24	0.00	1.0000	0.05	-29.0423	29.0424
BFU-E	Adult (H) Endo+AoD	CFU-GEMM	E11.5 Endo only	0.000023	14.0716	24	0.00	1.0000	0.05	-29.0423	29.0424
BFU-E	Adult (H) Endo+AoD	CFU-GEMM	E11.5 endo+AoD	0.000023	14.0716	24	0.00	1.0000	0.05	-29.0423	29.0424
BFU-E	Adult (H) Endo+AoD	CFU-GEMM	E11.5 endo+AoV	-10.6468	9.9502	24	-1.07	0.2953	0.05	-31.1831	9.8895
BFU-E	Adult (H) Endo+AoD	CFU-GM	Adult (H) Endo+AoD	-0.00014	14.0710	24	-0.00	1.0000	0.05	-29.0412	29.0409
BFU-E	Adult (H) Endo+AoD	CFU-GM	Adult (H) Endo+Aov	-11.9296	9.9501	24	-1.20	0.2423	0.05	-32.4657	8.6065
BFU-E	Adult (H) Endo+AoD	CFU-GM	Adult Endo (H) only	-0.00014	14.0709	24	-0.00	1.0000	0.05	-29.0411	29.0408
BFU-E	Adult (H) Endo+AoD	CFU-GM	E11.5 Endo only	-0.00014	14.0709	24	-0.00	1.0000	0.05	-29.0411	29.0408
BFU-E	Adult (H) Endo+AoD	CFU-GM	E11.5 endo+AoD	-0.00014	14.0709	24	-0.00	1.0000	0.05	-29.0411	29.0408
BFU-E	Adult (H) Endo+AoD	CFU-GM	E11.5 endo+AoV	-12.1354	9.9501	24	-1.22	0.2345	0.05	-32.6715	8.4006
BFU-E	Adult (H) Endo+Aov	BFU-E	Adult Endo (H) only	10.4005	9.9503	24	1.05	0.3063	0.05	-10.1358	30.9369
BFU-E	Adult (H) Endo+Aov	BFU-E	E11.5 Endo only	10.4005	9.9503	24	1.05	0.3063	0.05	-10.1358	30.9369
BFU-E	Adult (H) Endo+Aov	BFU-E	E11.5 endo+AoD	10.4005	9.9503	24	1.05	0.3063	0.05	-10.1358	30.9369
BFU-E	Adult (H) Endo+Aov	BFU-E	E11.5 endo+AoV	-0.4519	0.08199	24	-5.51	<.0001	0.05	-0.6211	-0.2827
BFU-E	Adult (H) Endo+Aov	CFU-GEMM	Adult (H) Endo+AoD	10.4005	9.9504	24	1.05	0.3063	0.05	-10.1360	30.9371
BFU-E	Adult (H) Endo+Aov	CFU-GEMM	Adult (H) Endo+Aov	0.8216	0.09930	24	8.27	<.0001	0.05	0.6167	1.0266
BFU-E	Adult (H) Endo+Aov	CFU-GEMM	Adult Endo (H) only	10.4005	9.9504	24	1.05	0.3063	0.05	-10.1360	30.9371

The GLIMMIX Procedure

Differences of Colony_Typ*Condition Least Squares Means											
Colony_Type	Condition	_Colony_Type	_Condition	Estimate	Standard Error	DF	t Value	Pr > |t|	Alpha	Lower	Upper
BFU-E	Adult (H) Endo+Aov	CFU-GEMM	E11.5 Endo only	10.4005	9.9504	24	1.05	0.3063	0.05	-10.1360	30.9371
BFU-E	Adult (H) Endo+Aov	CFU-GEMM	E11.5 endo+AoD	10.4005	9.9504	24	1.05	0.3063	0.05	-10.1360	30.9371
BFU-E	Adult (H) Endo+Aov	CFU-GEMM	E11.5 endo+AoV	-0.2463	0.08461	24	-2.91	0.0077	0.05	-0.4209	-0.07164
BFU-E	Adult (H) Endo+Aov	CFU-GM	Adult (H) Endo+AoD	10.4004	9.9494	24	1.05	0.3063	0.05	-10.1342	30.9349
BFU-E	Adult (H) Endo+Aov	CFU-GM	Adult (H) Endo+Aov	-1.5291	0.06054	24	-25.26	<.0001	0.05	-1.6540	-1.4042
BFU-E	Adult (H) Endo+Aov	CFU-GM	Adult Endo (H) only	10.4004	9.9494	24	1.05	0.3063	0.05	-10.1342	30.9349
BFU-E	Adult (H) Endo+Aov	CFU-GM	E11.5 Endo only	10.4004	9.9494	24	1.05	0.3063	0.05	-10.1342	30.9349
BFU-E	Adult (H) Endo+Aov	CFU-GM	E11.5 endo+AoD	10.4004	9.9494	24	1.05	0.3063	0.05	-10.1342	30.9349
BFU-E	Adult (H) Endo+Aov	CFU-GM	E11.5 endo+AoV	-1.7349	0.07306	24	-23.75	<.0001	0.05	-1.8857	-1.5841
BFU-E	Adult Endo (H) only	BFU-E	E11.5 Endo only	8.88E-16	14.0715	24	0.00	1.0000	0.05	-29.0422	29.0422
BFU-E	Adult Endo (H) only	BFU-E	E11.5 endo+AoD	-178E-17	14.0715	24	-0.00	1.0000	0.05	-29.0422	29.0422
BFU-E	Adult Endo (H) only	BFU-E	E11.5 endo+AoV	-10.8524	9.9502	24	-1.09	0.2862	0.05	-31.3886	9.6838
BFU-E	Adult Endo (H) only	CFU-GEMM	Adult (H) Endo+AoD	0.000023	14.0716	24	0.00	1.0000	0.05	-29.0423	29.0424
BFU-E	Adult Endo (H) only	CFU-GEMM	Adult (H) Endo+Aov	-9.5789	9.9505	24	-0.96	0.3453	0.05	-30.1156	10.9578
BFU-E	Adult Endo (H) only	CFU-GEMM	Adult Endo (H) only	0.000023	14.0715	24	0.00	1.0000	0.05	-29.0422	29.0422
BFU-E	Adult Endo (H) only	CFU-GEMM	E11.5 Endo only	0.000023	14.0716	24	0.00	1.0000	0.05	-29.0423	29.0424
BFU-E	Adult Endo (H) only	CFU-GEMM	E11.5 endo+AoD	0.000023	14.0716	24	0.00	1.0000	0.05	-29.0423	29.0424
BFU-E	Adult Endo (H) only	CFU-GEMM	E11.5 endo+AoV	-10.6468	9.9502	24	-1.07	0.2953	0.05	-31.1831	9.8895
BFU-E	Adult Endo (H) only	CFU-GM	Adult (H) Endo+AoD	-0.00014	14.0709	24	-0.00	1.0000	0.05	-29.0411	29.0408
BFU-E	Adult Endo (H) only	CFU-GM	Adult (H) Endo+Aov	-11.9296	9.9501	24	-1.20	0.2423	0.05	-32.4657	8.6065
BFU-E	Adult Endo (H) only	CFU-GM	Adult Endo (H) only	-0.00014	14.0710	24	-0.00	1.0000	0.05	-29.0412	29.0409
BFU-E	Adult Endo (H) only	CFU-GM	E11.5 Endo only	-0.00014	14.0709	24	-0.00	1.0000	0.05	-29.0411	29.0408
BFU-E	Adult Endo (H) only	CFU-GM	E11.5 endo+AoD	-0.00014	14.0709	24	-0.00	1.0000	0.05	-29.0411	29.0408
BFU-E	Adult Endo (H) only	CFU-GM	E11.5 endo+AoV	-12.1354	9.9501	24	-1.22	0.2345	0.05	-32.6715	8.4006

The GLIMMIX Procedure

Differences of Colony_Typ*Condition Least Squares Means											
Colony_Type	Condition	_Colony_Type	_Condition	Estimate	Standard Error	DF	t Value	Pr > |t|	Alpha	Lower	Upper
BFU-E	E11.5 Endo only	BFU-E	E11.5 endo+AoD	-266E-17	14.0715	24	-0.00	1.0000	0.05	-29.0422	29.0422
BFU-E	E11.5 Endo only	BFU-E	E11.5 endo+AoV	-10.8524	9.9502	24	-1.09	0.2862	0.05	-31.3886	9.6838
BFU-E	E11.5 Endo only	CFU-GEMM	Adult (H) Endo+AoD	0.000023	14.0716	24	0.00	1.0000	0.05	-29.0423	29.0424
BFU-E	E11.5 Endo only	CFU-GEMM	Adult (H) Endo+Aov	-9.5789	9.9505	24	-0.96	0.3453	0.05	-30.1156	10.9578
BFU-E	E11.5 Endo only	CFU-GEMM	Adult Endo (H) only	0.000023	14.0716	24	0.00	1.0000	0.05	-29.0423	29.0424
BFU-E	E11.5 Endo only	CFU-GEMM	E11.5 Endo only	0.000023	14.0715	24	0.00	1.0000	0.05	-29.0422	29.0422
BFU-E	E11.5 Endo only	CFU-GEMM	E11.5 endo+AoD	0.000023	14.0716	24	0.00	1.0000	0.05	-29.0423	29.0424
BFU-E	E11.5 Endo only	CFU-GEMM	E11.5 endo+AoV	-10.6468	9.9502	24	-1.07	0.2953	0.05	-31.1831	9.8895
BFU-E	E11.5 Endo only	CFU-GM	Adult (H) Endo+AoD	-0.00014	14.0709	24	-0.00	1.0000	0.05	-29.0411	29.0408
BFU-E	E11.5 Endo only	CFU-GM	Adult (H) Endo+Aov	-11.9296	9.9501	24	-1.20	0.2423	0.05	-32.4657	8.6065
BFU-E	E11.5 Endo only	CFU-GM	Adult Endo (H) only	-0.00014	14.0709	24	-0.00	1.0000	0.05	-29.0411	29.0408
BFU-E	E11.5 Endo only	CFU-GM	E11.5 Endo only	-0.00014	14.0710	24	-0.00	1.0000	0.05	-29.0412	29.0409
BFU-E	E11.5 Endo only	CFU-GM	E11.5 endo+AoD	-0.00014	14.0709	24	-0.00	1.0000	0.05	-29.0411	29.0408
BFU-E	E11.5 Endo only	CFU-GM	E11.5 endo+AoV	-12.1354	9.9501	24	-1.22	0.2345	0.05	-32.6715	8.4006
BFU-E	E11.5 endo+AoD	BFU-E	E11.5 endo+AoV	-10.8524	9.9502	24	-1.09	0.2862	0.05	-31.3886	9.6838
BFU-E	E11.5 endo+AoD	CFU-GEMM	Adult (H) Endo+AoD	0.000023	14.0716	24	0.00	1.0000	0.05	-29.0423	29.0424
BFU-E	E11.5 endo+AoD	CFU-GEMM	Adult (H) Endo+Aov	-9.5789	9.9505	24	-0.96	0.3453	0.05	-30.1156	10.9578
BFU-E	E11.5 endo+AoD	CFU-GEMM	Adult Endo (H) only	0.000023	14.0716	24	0.00	1.0000	0.05	-29.0423	29.0424
BFU-E	E11.5 endo+AoD	CFU-GEMM	E11.5 Endo only	0.000023	14.0716	24	0.00	1.0000	0.05	-29.0423	29.0424
BFU-E	E11.5 endo+AoD	CFU-GEMM	E11.5 endo+AoD	0.000023	14.0715	24	0.00	1.0000	0.05	-29.0422	29.0422
BFU-E	E11.5 endo+AoD	CFU-GEMM	E11.5 endo+AoV	-10.6468	9.9502	24	-1.07	0.2953	0.05	-31.1831	9.8895
BFU-E	E11.5 endo+AoD	CFU-GM	Adult (H) Endo+AoD	-0.00014	14.0709	24	-0.00	1.0000	0.05	-29.0411	29.0408
BFU-E	E11.5 endo+AoD	CFU-GM	Adult (H) Endo+Aov	-11.9296	9.9501	24	-1.20	0.2423	0.05	-32.4657	8.6065
BFU-E	E11.5 endo+AoD	CFU-GM	Adult Endo (H) only	-0.00014	14.0709	24	-0.00	1.0000	0.05	-29.0411	29.0408

The GLIMMIX Procedure

Differences of Colony_Typ*Condition Least Squares Means											
Colony_Type	Condition	_Colony_Type	_Condition	Estimate	Standard Error	DF	t Value	Pr > |t|	Alpha	Lower	Upper
BFU-E	E11.5 endo+AoD	CFU-GM	E11.5 Endo only	-0.00014	14.0709	24	-0.00	1.0000	0.05	-29.0411	29.0408
BFU-E	E11.5 endo+AoD	CFU-GM	E11.5 endo+AoD	-0.00014	14.0710	24	-0.00	1.0000	0.05	-29.0412	29.0409
BFU-E	E11.5 endo+AoD	CFU-GM	E11.5 endo+AoV	-12.1354	9.9501	24	-1.22	0.2345	0.05	-32.6715	8.4006
BFU-E	E11.5 endo+AoV	CFU-GEMM	Adult (H) Endo+AoD	10.8524	9.9503	24	1.09	0.2863	0.05	-9.6840	31.3889
BFU-E	E11.5 endo+AoV	CFU-GEMM	Adult (H) Endo+Aov	1.2735	0.1028	24	12.39	<.0001	0.05	1.0614	1.4856
BFU-E	E11.5 endo+AoV	CFU-GEMM	Adult Endo (H) only	10.8524	9.9503	24	1.09	0.2863	0.05	-9.6840	31.3889
BFU-E	E11.5 endo+AoV	CFU-GEMM	E11.5 Endo only	10.8524	9.9503	24	1.09	0.2863	0.05	-9.6840	31.3889
BFU-E	E11.5 endo+AoV	CFU-GEMM	E11.5 endo+AoD	10.8524	9.9503	24	1.09	0.2863	0.05	-9.6840	31.3889
BFU-E	E11.5 endo+AoV	CFU-GEMM	E11.5 endo+AoV	0.2056	0.06534	24	3.15	0.0044	0.05	0.07075	0.3405
BFU-E	E11.5 endo+AoV	CFU-GM	Adult (H) Endo+AoD	10.8523	9.9493	24	1.09	0.2862	0.05	-9.6822	31.3867
BFU-E	E11.5 endo+AoV	CFU-GM	Adult (H) Endo+Aov	-1.0772	0.06605	24	-16.31	<.0001	0.05	-1.2135	-0.9409
BFU-E	E11.5 endo+AoV	CFU-GM	Adult Endo (H) only	10.8523	9.9493	24	1.09	0.2862	0.05	-9.6822	31.3867
BFU-E	E11.5 endo+AoV	CFU-GM	E11.5 Endo only	10.8523	9.9493	24	1.09	0.2862	0.05	-9.6822	31.3867
BFU-E	E11.5 endo+AoV	CFU-GM	E11.5 endo+AoD	10.8523	9.9493	24	1.09	0.2862	0.05	-9.6822	31.3867
BFU-E	E11.5 endo+AoV	CFU-GM	E11.5 endo+AoV	-1.2830	0.04947	24	-25.94	<.0001	0.05	-1.3851	-1.1809
CFU-GEMM	Adult (H) Endo+AoD	CFU-GEMM	Adult (H) Endo+Aov	-9.5789	9.9506	24	-0.96	0.3453	0.05	-30.1159	10.9580
CFU-GEMM	Adult (H) Endo+AoD	CFU-GEMM	Adult Endo (H) only	5.11E-15	14.0717	24	0.00	1.0000	0.05	-29.0425	29.0425
CFU-GEMM	Adult (H) Endo+AoD	CFU-GEMM	E11.5 Endo only	6.88E-15	14.0717	24	0.00	1.0000	0.05	-29.0425	29.0425
CFU-GEMM	Adult (H) Endo+AoD	CFU-GEMM	E11.5 endo+AoD	5.77E-15	14.0717	24	0.00	1.0000	0.05	-29.0425	29.0425
CFU-GEMM	Adult (H) Endo+AoD	CFU-GEMM	E11.5 endo+AoV	-10.6468	9.9503	24	-1.07	0.2953	0.05	-31.1833	9.8897
CFU-GEMM	Adult (H) Endo+AoD	CFU-GM	Adult (H) Endo+AoD	-0.00016	14.0710	24	-0.00	1.0000	0.05	-29.0414	29.0410
CFU-GEMM	Adult (H) Endo+AoD	CFU-GM	Adult (H) Endo+Aov	-11.9296	9.9503	24	-1.20	0.2423	0.05	-32.4660	8.6067
CFU-GEMM	Adult (H) Endo+AoD	CFU-GM	Adult Endo (H) only	-0.00016	14.0710	24	-0.00	1.0000	0.05	-29.0413	29.0409
CFU-GEMM	Adult (H) Endo+AoD	CFU-GM	E11.5 Endo only	-0.00016	14.0710	24	-0.00	1.0000	0.05	-29.0413	29.0409

The GLIMMIX Procedure

Differences of Colony_Typ*Condition Least Squares Means											
Colony_Type	Condition	_Colony_Type	_Condition	Estimate	Standard Error	DF	t Value	Pr > |t|	Alpha	Lower	Upper
CFU-GEMM	Adult (H) Endo+AoD	CFU-GM	E11.5 endo+AoD	-0.00016	14.0710	24	-0.00	1.0000	0.05	-29.0413	29.0409
CFU-GEMM	Adult (H) Endo+AoD	CFU-GM	E11.5 endo+AoV	-12.1355	9.9503	24	-1.22	0.2345	0.05	-32.6718	8.4008
CFU-GEMM	Adult (H) Endo+Aov	CFU-GEMM	Adult Endo (H) only	9.5789	9.9506	24	0.96	0.3453	0.05	-10.9580	30.1159
CFU-GEMM	Adult (H) Endo+Aov	CFU-GEMM	E11.5 Endo only	9.5789	9.9506	24	0.96	0.3453	0.05	-10.9580	30.1159
CFU-GEMM	Adult (H) Endo+Aov	CFU-GEMM	E11.5 endo+AoD	9.5789	9.9506	24	0.96	0.3453	0.05	-10.9580	30.1159
CFU-GEMM	Adult (H) Endo+Aov	CFU-GEMM	E11.5 endo+AoV	-1.0679	0.1049	24	-10.18	<.0001	0.05	-1.2843	-0.8515
CFU-GEMM	Adult (H) Endo+Aov	CFU-GM	Adult (H) Endo+AoD	9.5788	9.9496	24	0.96	0.3453	0.05	-10.9562	30.1137
CFU-GEMM	Adult (H) Endo+Aov	CFU-GM	Adult (H) Endo+Aov	-2.3507	0.08662	24	-27.14	<.0001	0.05	-2.5295	-2.1720
CFU-GEMM	Adult (H) Endo+Aov	CFU-GM	Adult Endo (H) only	9.5788	9.9496	24	0.96	0.3453	0.05	-10.9562	30.1137
CFU-GEMM	Adult (H) Endo+Aov	CFU-GM	E11.5 Endo only	9.5788	9.9496	24	0.96	0.3453	0.05	-10.9562	30.1137
CFU-GEMM	Adult (H) Endo+Aov	CFU-GM	E11.5 endo+AoD	9.5788	9.9496	24	0.96	0.3453	0.05	-10.9562	30.1137
CFU-GEMM	Adult (H) Endo+Aov	CFU-GM	E11.5 endo+AoV	-2.5566	0.09579	24	-26.69	<.0001	0.05	-2.7542	-2.3589
CFU-GEMM	Adult Endo (H) only	CFU-GEMM	E11.5 Endo only	1.78E-15	14.0717	24	0.00	1.0000	0.05	-29.0425	29.0425
CFU-GEMM	Adult Endo (H) only	CFU-GEMM	E11.5 endo+AoD	6.66E-16	14.0717	24	0.00	1.0000	0.05	-29.0425	29.0425
CFU-GEMM	Adult Endo (H) only	CFU-GEMM	E11.5 endo+AoV	-10.6468	9.9503	24	-1.07	0.2953	0.05	-31.1833	9.8897
CFU-GEMM	Adult Endo (H) only	CFU-GM	Adult (H) Endo+AoD	-0.00016	14.0710	24	-0.00	1.0000	0.05	-29.0413	29.0409
CFU-GEMM	Adult Endo (H) only	CFU-GM	Adult (H) Endo+Aov	-11.9296	9.9503	24	-1.20	0.2423	0.05	-32.4660	8.6067
CFU-GEMM	Adult Endo (H) only	CFU-GM	Adult Endo (H) only	-0.00016	14.0710	24	-0.00	1.0000	0.05	-29.0414	29.0410
CFU-GEMM	Adult Endo (H) only	CFU-GM	E11.5 Endo only	-0.00016	14.0710	24	-0.00	1.0000	0.05	-29.0413	29.0409
CFU-GEMM	Adult Endo (H) only	CFU-GM	E11.5 endo+AoD	-0.00016	14.0710	24	-0.00	1.0000	0.05	-29.0413	29.0409
CFU-GEMM	Adult Endo (H) only	CFU-GM	E11.5 endo+AoV	-12.1355	9.9503	24	-1.22	0.2345	0.05	-32.6718	8.4008
CFU-GEMM	E11.5 Endo only	CFU-GEMM	E11.5 endo+AoD	-111E-17	14.0717	24	-0.00	1.0000	0.05	-29.0425	29.0425
CFU-GEMM	E11.5 Endo only	CFU-GEMM	E11.5 endo+AoV	-10.6468	9.9503	24	-1.07	0.2953	0.05	-31.1833	9.8897
CFU-GEMM	E11.5 Endo only	CFU-GM	Adult (H) Endo+AoD	-0.00016	14.0710	24	-0.00	1.0000	0.05	-29.0413	29.0409

The GLIMMIX Procedure

Differences of Colony_Typ*Condition Least Squares Means											
Colony_Type	Condition	_Colony_Type	_Condition	Estimate	Standard Error	DF	t Value	Pr > |t|	Alpha	Lower	Upper
CFU-GEMM	E11.5 Endo only	CFU-GM	Adult (H) Endo+Aov	-11.9296	9.9503	24	-1.20	0.2423	0.05	-32.4660	8.6067
CFU-GEMM	E11.5 Endo only	CFU-GM	Adult Endo (H) only	-0.00016	14.0710	24	-0.00	1.0000	0.05	-29.0413	29.0409
CFU-GEMM	E11.5 Endo only	CFU-GM	E11.5 Endo only	-0.00016	14.0710	24	-0.00	1.0000	0.05	-29.0414	29.0410
CFU-GEMM	E11.5 Endo only	CFU-GM	E11.5 endo+AoD	-0.00016	14.0710	24	-0.00	1.0000	0.05	-29.0413	29.0409
CFU-GEMM	E11.5 Endo only	CFU-GM	E11.5 endo+AoV	-12.1355	9.9503	24	-1.22	0.2345	0.05	-32.6718	8.4008
CFU-GEMM	E11.5 endo+AoD	CFU-GEMM	E11.5 endo+AoV	-10.6468	9.9503	24	-1.07	0.2953	0.05	-31.1833	9.8897
CFU-GEMM	E11.5 endo+AoD	CFU-GM	Adult (H) Endo+AoD	-0.00016	14.0710	24	-0.00	1.0000	0.05	-29.0413	29.0409
CFU-GEMM	E11.5 endo+AoD	CFU-GM	Adult (H) Endo+Aov	-11.9296	9.9503	24	-1.20	0.2423	0.05	-32.4660	8.6067
CFU-GEMM	E11.5 endo+AoD	CFU-GM	Adult Endo (H) only	-0.00016	14.0710	24	-0.00	1.0000	0.05	-29.0413	29.0409
CFU-GEMM	E11.5 endo+AoD	CFU-GM	E11.5 Endo only	-0.00016	14.0710	24	-0.00	1.0000	0.05	-29.0413	29.0409
CFU-GEMM	E11.5 endo+AoD	CFU-GM	E11.5 endo+AoD	-0.00016	14.0710	24	-0.00	1.0000	0.05	-29.0414	29.0410
CFU-GEMM	E11.5 endo+AoD	CFU-GM	E11.5 endo+AoV	-12.1355	9.9503	24	-1.22	0.2345	0.05	-32.6718	8.4008
CFU-GEMM	E11.5 endo+AoV	CFU-GM	Adult (H) Endo+AoD	10.6467	9.9494	24	1.07	0.2952	0.05	-9.8878	31.1811
CFU-GEMM	E11.5 endo+AoV	CFU-GM	Adult (H) Endo+Aov	-1.2828	0.06929	24	-18.51	<.0001	0.05	-1.4258	-1.1398
CFU-GEMM	E11.5 endo+AoV	CFU-GM	Adult Endo (H) only	10.6467	9.9494	24	1.07	0.2952	0.05	-9.8878	31.1811
CFU-GEMM	E11.5 endo+AoV	CFU-GM	E11.5 Endo only	10.6467	9.9494	24	1.07	0.2952	0.05	-9.8878	31.1811
CFU-GEMM	E11.5 endo+AoV	CFU-GM	E11.5 endo+AoD	10.6467	9.9494	24	1.07	0.2952	0.05	-9.8878	31.1811
CFU-GEMM	E11.5 endo+AoV	CFU-GM	E11.5 endo+AoV	-1.4886	0.05371	24	-27.72	<.0001	0.05	-1.5995	-1.3778
CFU-GM	Adult (H) Endo+AoD	CFU-GM	Adult (H) Endo+Aov	-11.9295	9.9493	24	-1.20	0.2422	0.05	-32.4638	8.6048
CFU-GM	Adult (H) Endo+AoD	CFU-GM	Adult Endo (H) only	7.11E-15	14.0703	24	0.00	1.0000	0.05	-29.0397	29.0397
CFU-GM	Adult (H) Endo+AoD	CFU-GM	E11.5 Endo only	7.11E-15	14.0703	24	0.00	1.0000	0.05	-29.0397	29.0397
CFU-GM	Adult (H) Endo+AoD	CFU-GM	E11.5 endo+AoD	5.33E-15	14.0703	24	0.00	1.0000	0.05	-29.0397	29.0397
CFU-GM	Adult (H) Endo+AoD	CFU-GM	E11.5 endo+AoV	-12.1353	9.9493	24	-1.22	0.2344	0.05	-32.6696	8.3990
CFU-GM	Adult (H) Endo+Aov	CFU-GM	Adult Endo (H) only	11.9295	9.9493	24	1.20	0.2422	0.05	-8.6048	32.4638

The GLIMMIX Procedure

Differences of Colony_Typ*Condition Least Squares Means											
Colony_Type	Condition	_Colony_Type	_Condition	Estimate	Standard Error	DF	t Value	Pr > |t|	Alpha	Lower	Upper
CFU-GM	Adult (H) Endo+Aov	CFU-GM	E11.5 Endo only	11.9295	9.9493	24	1.20	0.2422	0.05	-8.6048	32.4638
CFU-GM	Adult (H) Endo+Aov	CFU-GM	E11.5 endo+AoD	11.9295	9.9493	24	1.20	0.2422	0.05	-8.6048	32.4638
CFU-GM	Adult (H) Endo+Aov	CFU-GM	E11.5 endo+AoV	-0.2058	0.05457	24	-3.77	0.0009	0.05	-0.3185	-0.09320
CFU-GM	Adult Endo (H) only	CFU-GM	E11.5 Endo only	0	14.0703	24	0.00	1.0000	0.05	-29.0397	29.0397
CFU-GM	Adult Endo (H) only	CFU-GM	E11.5 endo+AoD	-178E-17	14.0703	24	-0.00	1.0000	0.05	-29.0397	29.0397
CFU-GM	Adult Endo (H) only	CFU-GM	E11.5 endo+AoV	-12.1353	9.9493	24	-1.22	0.2344	0.05	-32.6696	8.3990
CFU-GM	E11.5 Endo only	CFU-GM	E11.5 endo+AoD	-178E-17	14.0703	24	-0.00	1.0000	0.05	-29.0397	29.0397
CFU-GM	E11.5 Endo only	CFU-GM	E11.5 endo+AoV	-12.1353	9.9493	24	-1.22	0.2344	0.05	-32.6696	8.3990
CFU-GM	E11.5 endo+AoD	CFU-GM	E11.5 endo+AoV	-12.1353	9.9493	24	-1.22	0.2344	0.05	-32.6696	8.3990

The GENMOD Procedure

Model Information	
**Data Set**	WORK.EXT_FIG8DV
**Distribution**	Normal
**Link Function**	Identity
**Dependent Variable**	Percentage

**Number of Observations Read**	30
**Number of Observations Used**	30

Class Level Information		
**Class**	**Levels**	**Values**
**Condition**	6	AoE+AoDPSC AoE+AoVPSC E11.5EC Only HE Only HE+AoDPSC HE+AoVPSC

Parameter Information		
**Parameter**	**Effect**	**Condition**
**Prm1**	Intercept	
**Prm2**	Condition	AoE+AoDPSC
**Prm3**	Condition	AoE+AoVPSC
**Prm4**	Condition	E11.5EC Only
**Prm5**	Condition	HE Only
**Prm6**	Condition	HE+AoDPSC
**Prm7**	Condition	HE+AoVPSC

The GENMOD Procedure

Criteria For Assessing Goodness Of Fit			
**Criterion**	**DF**	**Value**	**Value/DF**
**Deviance**	24	110.0000	4.5833
**Scaled Deviance**	24	30.0000	1.2500
**Pearson Chi-Square**	24	110.0000	4.5833
**Scaled Pearson X2**	24	30.0000	1.2500
**Log Likelihood**		-62.0574	
**Full Log Likelihood**		-62.0574	
**AIC (smaller is better)**		138.1148	
**AICC (smaller is better)**		143.2057	
**BIC (smaller is better)**		147.9232	

Algorithm converged.

Analysis Of Maximum Likelihood Parameter Estimates								
**Parameter**		**DF**	**Estimate**	**Standard Error**	**Wald 95% Confidence Limits**		**Wald Chi-Square**	**Pr > ChiSq**
**Intercept**		1	8.6000	0.8563	6.9216	10.2784	100.85	<.0001
**Condition**	AoE+AoDPSC	1	-8.6000	1.2111	-10.9736	-6.2264	50.43	<.0001
**Condition**	AoE+AoVPSC	1	13.6000	1.2111	11.2264	15.9736	126.11	<.0001
**Condition**	E11.5EC Only	1	-8.6000	1.2111	-10.9736	-6.2264	50.43	<.0001
**Condition**	HE Only	1	-8.6000	1.2111	-10.9736	-6.2264	50.43	<.0001
**Condition**	HE+AoDPSC	1	-8.6000	1.2111	-10.9736	-6.2264	50.43	<.0001
**Condition**	HE+AoVPSC	0	0.0000	0.0000	0.0000	0.0000	.	.
**Scale**		1	1.9149	0.2472	1.4868	2.4662		

Note: The scale parameter was estimated by maximum likelihood.

The GENMOD Procedure

Condition Least Squares Means							
Condition	Estimate	Standard Error	z Value	Pr > |z|	Alpha	Lower	Upper
AoE+AoDPSC	0	0.8563	0.00	1.0000	0.05	-1.6784	1.6784
AoE+AoVPSC	22.2000	0.8563	25.92	<.0001	0.05	20.5216	23.8784
E11.5EC Only	0	0.8563	0.00	1.0000	0.05	-1.6784	1.6784
HE Only	0	0.8563	0.00	1.0000	0.05	-1.6784	1.6784
HE+AoDPSC	0	0.8563	0.00	1.0000	0.05	-1.6784	1.6784
HE+AoVPSC	8.6000	0.8563	10.04	<.0001	0.05	6.9216	10.2784

Differences of Condition Least Squares Means								
Condition	_Condition	Estimate	Standard Error	z Value	Pr > |z|	Alpha	Lower	Upper
AoE+AoDPSC	AoE+AoVPSC	-22.2000	1.2111	-18.33	<.0001	0.05	-24.5736	-19.8264
AoE+AoDPSC	E11.5EC Only	0	1.2111	0.00	1.0000	0.05	-2.3736	2.3736
AoE+AoDPSC	HE Only	0	1.2111	0.00	1.0000	0.05	-2.3736	2.3736
AoE+AoDPSC	HE+AoDPSC	0	1.2111	0.00	1.0000	0.05	-2.3736	2.3736
AoE+AoDPSC	HE+AoVPSC	-8.6000	1.2111	-7.10	<.0001	0.05	-10.9736	-6.2264
AoE+AoVPSC	E11.5EC Only	22.2000	1.2111	18.33	<.0001	0.05	19.8264	24.5736
AoE+AoVPSC	HE Only	22.2000	1.2111	18.33	<.0001	0.05	19.8264	24.5736
AoE+AoVPSC	HE+AoDPSC	22.2000	1.2111	18.33	<.0001	0.05	19.8264	24.5736
AoE+AoVPSC	HE+AoVPSC	13.6000	1.2111	11.23	<.0001	0.05	11.2264	15.9736
E11.5EC Only	HE Only	0	1.2111	0.00	1.0000	0.05	-2.3736	2.3736
E11.5EC Only	HE+AoDPSC	0	1.2111	0.00	1.0000	0.05	-2.3736	2.3736
E11.5EC Only	HE+AoVPSC	-8.6000	1.2111	-7.10	<.0001	0.05	-10.9736	-6.2264
HE Only	HE+AoDPSC	0	1.2111	0.00	1.0000	0.05	-2.3736	2.3736
HE Only	HE+AoVPSC	-8.6000	1.2111	-7.10	<.0001	0.05	-10.9736	-6.2264
HE+AoDPSC	HE+AoVPSC	-8.6000	1.2111	-7.10	<.0001	0.05	-10.9736	-6.2264

The GENMOD Procedure

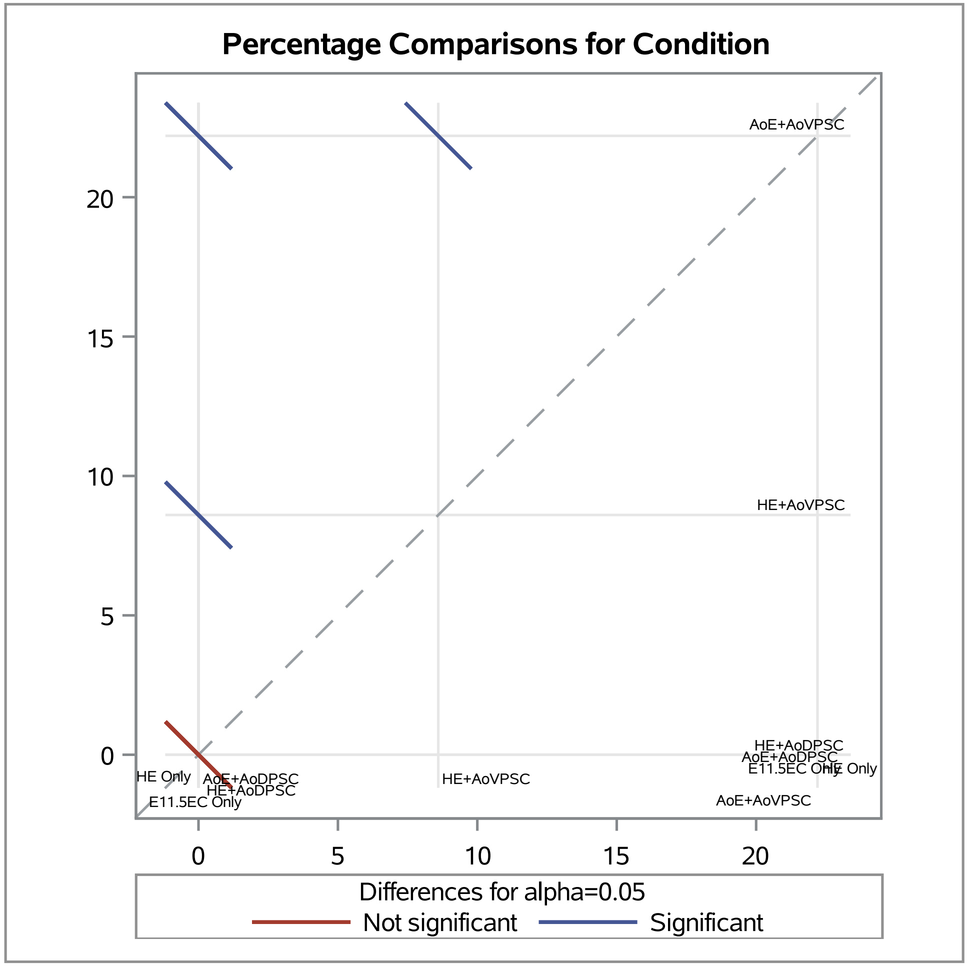

The GLIMMIX Procedure

Model Information	
**Data Set**	WORK.EXT_FIG10BI_E10DRAXIN
**Response Variable**	Count
**Response Distribution**	Poisson
**Link Function**	Log
**Variance Function**	Default
**Variance Matrix Blocked By**	Dish_ID
**Estimation Technique**	Maximum Likelihood
**Likelihood Approximation**	Laplace
**Degrees of Freedom Method**	Containment

Class Level Information		
**Class**	**Levels**	**Values**
**Colony_Type**	3	BFU-E CFU-GEMM CFU-GM
**Condition**	3	AoE+PSCs E10.5AGM HE+PSCs
**Treatment**	3	0ug/ml 2ug/ml 6ug/ml
**Dish_ID**	27	1 2 3 4 5 6 7 8 9 10 11 12 13 14 15 16 17 18 19 20 21 22 23 24 25 26 27

**Number of Observations Read**	81
**Number of Observations Used**	81

Dimensions	
**G-side Cov. Parameters**	1
**Columns in X**	64
**Columns in Z per Subject**	1
**Subjects (Blocks in V)**	27
**Max Obs per Subject**	3

The GLIMMIX Procedure

Optimization Information	
Optimization Technique	Dual Quasi-Newton
Parameters in Optimization	28
Lower Boundaries	1
Upper Boundaries	0
Fixed Effects	Not Profiled
Starting From	GLM estimates

Iteration History					
Iteration	Restarts	Evaluations	Objective Function	Change	Max Gradient
0	0	4	699.68419739	.	1286.361
1	0	8	698.44403704	1.24016035	338.5573
2	0	4	697.73145885	0.71257819	271.2121
3	0	2	697.60348141	0.12797744	124.8529
4	0	2	697.5324929	0.07098852	32.44105
5	0	2	697.52404553	0.00844736	14.53688
6	0	4	697.50603907	0.01800647	36.80343
7	0	3	697.50091443	0.00512463	3.935246
8	0	3	697.49997955	0.00093488	6.790517
9	0	4	697.49361261	0.00636694	2.264431
10	0	4	697.49016827	0.00344434	15.60495
11	0	2	697.48734826	0.00282001	10.72464
12	0	3	697.48584113	0.00150712	2.839936
13	0	3	697.4848143	0.00102683	1.966603
14	0	4	697.48270456	0.00210974	12.46788
15	0	3	697.48196055	0.00074401	0.778828

The GLIMMIX Procedure

Iteration History					
**Iteration**	**Restarts**	**Evaluations**	**Objective Function**	**Change**	**Max Gradient**
**16**	**0**	2	697.48173606	0.00022448	1.974582
**17**	**0**	3	697.48161089	0.00012517	0.481112
**18**	**0**	4	697.48143345	0.00017744	0.771938
**19**	**0**	3	697.48138534	0.00004812	0.506687
**20**	**0**	4	697.48093089	0.00045445	0.786114
**21**	**0**	3	697.48091818	0.00001271	0.433097
**22**	**0**	4	697.48078537	0.00013281	0.691671
**23**	**0**	2	697.4806196	0.00016577	1.211089
**24**	**0**	2	697.48037651	0.00024309	1.960113
**25**	**0**	3	697.48028495	0.00009156	0.345907
**26**	**0**	3	697.48026608	0.00001887	1.09012
**27**	**0**	4	697.48011398	0.00015210	0.223978
**28**	**0**	2	697.4800493	0.00006469	0.299229
**29**	**0**	3	697.48002707	0.00002222	0.148417
**30**	**0**	2	697.4800205	0.00000657	0.212593

Convergence criterion (GCONV=1E-8) satisfied.

Fit Statistics	
**-2 Log Likelihood**	697.48
**AIC (smaller is better)**	753.48
**AICC (smaller is better)**	784.71
**BIC (smaller is better)**	789.76
**CAIC (smaller is better)**	817.76
**HQIC (smaller is better)**	764.27

The GLIMMIX Procedure

Fit Statistics for Conditional Distribution	
**-2 log L(Count | r. effects)**	632.17
**Pearson Chi-Square**	111.07
**Pearson Chi-Square / DF**	1.37

Covariance Parameter Estimates			
**Cov Parm**	**Subject**	**Estimate**	**Standard Error**
**Intercept**	Dish_ID	0.01011	0.003877

The GLIMMIX Procedure

Solutions for Fixed Effects								
Effect	Colony_Type	Condition	Treatment	Estimate	Standard Error	DF	t Value	Pr > |t|
Intercept				4.4694	0.08477	18	52.73	<.0001
Colony_Type	BFU-E			-1.2993	0.1329	36	-9.77	<.0001
Colony_Type	CFU-GEMM			-1.2584	0.1308	36	-9.62	<.0001
Colony_Type	CFU-GM			0	.	.	.	.
Condition		AoE+PSCs		0.4052	0.1144	36	3.54	0.0011
Condition		E10.5AGM		0.8713	0.1102	36	7.91	<.0001
Condition		HE+PSCs		0	.	.	.	.
Colony_Typ*Condition	BFU-E	AoE+PSCs		-0.8755	0.2060	36	-4.25	0.0001
Colony_Typ*Condition	BFU-E	E10.5AGM		-0.3198	0.1651	36	-1.94	0.0606
Colony_Typ*Condition	BFU-E	HE+PSCs		0	.	.	.	.
Colony_Typ*Condition	CFU-GEMM	AoE+PSCs		-0.6798	0.1928	36	-3.53	0.0012
Colony_Typ*Condition	CFU-GEMM	E10.5AGM		0.06869	0.1546	36	0.44	0.6595
Colony_Typ*Condition	CFU-GEMM	HE+PSCs		0	.	.	.	.
Colony_Typ*Condition	CFU-GM	AoE+PSCs		0	.	.	.	.
Colony_Typ*Condition	CFU-GM	E10.5AGM		0	.	.	.	.
Colony_Typ*Condition	CFU-GM	HE+PSCs		0	.	.	.	.
Treatment			0ug/ml	1.6356	0.1063	36	15.39	<.0001
Treatment			2ug/ml	0.7601	0.1111	36	6.84	<.0001
Treatment			6ug/ml	0	.	.	.	.
Colony_Typ*Treatment	BFU-E		0ug/ml	-0.2463	0.1479	36	-1.67	0.1045
Colony_Typ*Treatment	BFU-E		2ug/ml	0.03732	0.1604	36	0.23	0.8174
Colony_Typ*Treatment	BFU-E		6ug/ml	0	.	.	.	.
Colony_Typ*Treatment	CFU-GEMM		0ug/ml	-0.3743	0.1471	36	-2.54	0.0154
Colony_Typ*Treatment	CFU-GEMM		2ug/ml	-0.4980	0.1709	36	-2.91	0.0061

The GLIMMIX Procedure

Solutions for Fixed Effects								
**Effect**	**Colony_Type**	**Condition**	**Treatment**	**Estimate**	**Standard Error**	**DF**	**t Value**	**Pr > |t|**
**Colony_Typ*Treatment**	CFU-GEMM		6ug/ml	0	.	.	.	.
**Colony_Typ*Treatment**	CFU-GM		0ug/ml	0	.	.	.	.
**Colony_Typ*Treatment**	CFU-GM		2ug/ml	0	.	.	.	.
**Colony_Typ*Treatment**	CFU-GM		6ug/ml	0	.	.	.	.
**Condition*Treatment**		AoE+PSCs	0ug/ml	-0.3165	0.1458	36	-2.17	0.0366
**Condition*Treatment**		AoE+PSCs	2ug/ml	-0.2135	0.1520	36	-1.40	0.1687
**Condition*Treatment**		AoE+PSCs	6ug/ml	0	.	.	.	.
**Condition*Treatment**		E10.5AGM	0ug/ml	-0.3377	0.1417	36	-2.38	0.0225
**Condition*Treatment**		E10.5AGM	2ug/ml	-0.07547	0.1466	36	-0.51	0.6097
**Condition*Treatment**		E10.5AGM	6ug/ml	0	.	.	.	.
**Condition*Treatment**		HE+PSCs	0ug/ml	0	.	.	.	.
**Condition*Treatment**		HE+PSCs	2ug/ml	0	.	.	.	.
**Condition*Treatment**		HE+PSCs	6ug/ml	0	.	.	.	.
**Colony*Condit*Treatm**	BFU-E	AoE+PSCs	0ug/ml	0.9973	0.2239	36	4.45	<.0001
**Colony*Condit*Treatm**	BFU-E	AoE+PSCs	2ug/ml	0.4696	0.2443	36	1.92	0.0626
**Colony*Condit*Treatm**	BFU-E	AoE+PSCs	6ug/ml	0	.	.	.	.
**Colony*Condit*Treatm**	BFU-E	E10.5AGM	0ug/ml	0.5155	0.1833	36	2.81	0.0079
**Colony*Condit*Treatm**	BFU-E	E10.5AGM	2ug/ml	0.3818	0.1970	36	1.94	0.0605
**Colony*Condit*Treatm**	BFU-E	E10.5AGM	6ug/ml	0	.	.	.	.
**Colony*Condit*Treatm**	BFU-E	HE+PSCs	0ug/ml	0	.	.	.	.
**Colony*Condit*Treatm**	BFU-E	HE+PSCs	2ug/ml	0	.	.	.	.
**Colony*Condit*Treatm**	BFU-E	HE+PSCs	6ug/ml	0	.	.	.	.
**Colony*Condit*Treatm**	CFU-GEMM	AoE+PSCs	0ug/ml	0.7430	0.2137	36	3.48	0.0013
**Colony*Condit*Treatm**	CFU-GEMM	AoE+PSCs	2ug/ml	1.0501	0.2379	36	4.41	<.0001

The GLIMMIX Procedure

Solutions for Fixed Effects								
Effect	Colony_Type	Condition	Treatment	Estimate	Standard Error	DF	t Value	Pr > |t|
**Colony*Condit*Treatm**	CFU-GEMM	AoE+PSCs	6ug/ml	0	.	.	.	.
**Colony*Condit*Treatm**	CFU-GEMM	E10.5AGM	0ug/ml	0.1059	0.1753	36	0.60	0.5498
**Colony*Condit*Treatm**	CFU-GEMM	E10.5AGM	2ug/ml	0.7176	0.1973	36	3.64	0.0009
**Colony*Condit*Treatm**	CFU-GEMM	E10.5AGM	6ug/ml	0	.	.	.	.
**Colony*Condit*Treatm**	CFU-GEMM	HE+PSCs	0ug/ml	0	.	.	.	.
**Colony*Condit*Treatm**	CFU-GEMM	HE+PSCs	2ug/ml	0	.	.	.	.
**Colony*Condit*Treatm**	CFU-GEMM	HE+PSCs	6ug/ml	0	.	.	.	.
**Colony*Condit*Treatm**	CFU-GM	AoE+PSCs	0ug/ml	0	.	.	.	.
**Colony*Condit*Treatm**	CFU-GM	AoE+PSCs	2ug/ml	0	.	.	.	.
**Colony*Condit*Treatm**	CFU-GM	AoE+PSCs	6ug/ml	0	.	.	.	.
**Colony*Condit*Treatm**	CFU-GM	E10.5AGM	0ug/ml	0	.	.	.	.
**Colony*Condit*Treatm**	CFU-GM	E10.5AGM	2ug/ml	0	.	.	.	.
**Colony*Condit*Treatm**	CFU-GM	E10.5AGM	6ug/ml	0	.	.	.	.
**Colony*Condit*Treatm**	CFU-GM	HE+PSCs	0ug/ml	0	.	.	.	.
**Colony*Condit*Treatm**	CFU-GM	HE+PSCs	2ug/ml	0	.	.	.	.
**Colony*Condit*Treatm**	CFU-GM	HE+PSCs	6ug/ml	0	.	.	.	.

Type III Tests of Fixed Effects				
Effect	Num DF	Den DF	F Value	Pr > F
**Colony_Type**	2	36	1904.24	<.0001
**Condition**	2	36	137.98	<.0001
**Colony_Typ*Condition**	4	36	17.47	<.0001
**Treatment**	2	36	306.05	<.0001
**Colony_Typ*Treatment**	4	36	5.56	0.0014

The GLIMMIX Procedure

Type III Tests of Fixed Effects				
Effect	Num DF	Den DF	F Value	Pr > F
Condition*Treatment	4	36	4.36	0.0056
Colony*Condit*Treatm	8	36	7.42	<.0001

Condition*Treatment Least Squares Means													
Condition	Treatment	Estimate	Standard Error	DF	t Value	Pr > |t|	Alpha	Lower	Upper	Mean	Standard Error Mean	Lower Mean	Upper Mean
AoE+PSCs	0ug/ml	5.1960	0.06422	36	80.91	<.0001	0.05	5.0657	5.3262	180.55	11.5940	158.50	205.66
AoE+PSCs	2ug/ml	4.4033	0.07112	36	61.92	<.0001	0.05	4.2591	4.5476	81.7228	5.8118	70.7464	94.4021
AoE+PSCs	6ug/ml	3.5036	0.08995	36	38.95	<.0001	0.05	3.3212	3.6861	33.2362	2.9897	27.6938	39.8878
E10.5AGM	0ug/ml	5.7026	0.06176	36	92.33	<.0001	0.05	5.5774	5.8279	299.66	18.5076	264.38	339.65
E10.5AGM	2ug/ml	5.3020	0.06320	36	83.90	<.0001	0.05	5.1738	5.4302	200.74	12.6863	176.59	228.19
E10.5AGM	6ug/ml	4.4045	0.07091	36	62.12	<.0001	0.05	4.2606	4.5483	81.8144	5.8012	70.8559	94.4678
HE+PSCs	0ug/ml	5.0456	0.06530	36	77.27	<.0001	0.05	4.9131	5.1780	155.33	10.1431	136.07	177.33
HE+PSCs	2ug/ml	4.2234	0.07360	36	57.39	<.0001	0.05	4.0741	4.3727	68.2658	5.0242	58.8004	79.2551
HE+PSCs	6ug/ml	3.6168	0.08272	36	43.72	<.0001	0.05	3.4491	3.7846	37.2197	3.0789	31.4711	44.0183

Differences of Condition*Treatment Least Squares Means											
Condition	Treatment	_Condition	_Treatment	Estimate	Standard Error	DF	t Value	Pr > |t|	Alpha	Lower	Upper
AoE+PSCs	0ug/ml	AoE+PSCs	2ug/ml	0.7927	0.09582	36	8.27	<.0001	0.05	0.5983	0.9870
AoE+PSCs	0ug/ml	AoE+PSCs	6ug/ml	1.6923	0.1105	36	15.31	<.0001	0.05	1.4682	1.9165
AoE+PSCs	0ug/ml	E10.5AGM	0ug/ml	-0.5067	0.08910	36	-5.69	<.0001	0.05	-0.6874	-0.3260
AoE+PSCs	0ug/ml	E10.5AGM	2ug/ml	-0.1060	0.09010	36	-1.18	0.2470	0.05	-0.2888	0.07670
AoE+PSCs	0ug/ml	E10.5AGM	6ug/ml	0.7915	0.09566	36	8.27	<.0001	0.05	0.5975	0.9855
AoE+PSCs	0ug/ml	HE+PSCs	0ug/ml	0.1504	0.09158	36	1.64	0.1092	0.05	-0.03533	0.3362

The GLIMMIX Procedure

Differences of Condition*Treatment Least Squares Means											
Condition	Treatment	_Condition	_Treatment	Estimate	Standard Error	DF	t Value	Pr > |t|	Alpha	Lower	Upper
AoE+PSCs	0ug/ml	HE+PSCs	2ug/ml	0.9726	0.09767	36	9.96	<.0001	0.05	0.7745	1.1707
AoE+PSCs	0ug/ml	HE+PSCs	6ug/ml	1.5791	0.1047	36	15.08	<.0001	0.05	1.3668	1.7915
AoE+PSCs	2ug/ml	AoE+PSCs	6ug/ml	0.8997	0.1146	36	7.85	<.0001	0.05	0.6672	1.1322
AoE+PSCs	2ug/ml	E10.5AGM	0ug/ml	-1.2993	0.09419	36	-13.79	<.0001	0.05	-1.4903	-1.1083
AoE+PSCs	2ug/ml	E10.5AGM	2ug/ml	-0.8987	0.09514	36	-9.45	<.0001	0.05	-1.0916	-0.7057
AoE+PSCs	2ug/ml	E10.5AGM	6ug/ml	-0.00112	0.1004	36	-0.01	0.9912	0.05	-0.2048	0.2025
AoE+PSCs	2ug/ml	HE+PSCs	0ug/ml	-0.6422	0.09654	36	-6.65	<.0001	0.05	-0.8380	-0.4464
AoE+PSCs	2ug/ml	HE+PSCs	2ug/ml	0.1799	0.1023	36	1.76	0.0872	0.05	-0.02762	0.3875
AoE+PSCs	2ug/ml	HE+PSCs	6ug/ml	0.7865	0.1091	36	7.21	<.0001	0.05	0.5653	1.0077
AoE+PSCs	6ug/ml	E10.5AGM	0ug/ml	-2.1990	0.1091	36	-20.15	<.0001	0.05	-2.4203	-1.9777
AoE+PSCs	6ug/ml	E10.5AGM	2ug/ml	-1.7984	0.1099	36	-16.36	<.0001	0.05	-2.0213	-1.5754
AoE+PSCs	6ug/ml	E10.5AGM	6ug/ml	-0.9008	0.1145	36	-7.87	<.0001	0.05	-1.1331	-0.6686
AoE+PSCs	6ug/ml	HE+PSCs	0ug/ml	-1.5419	0.1111	36	-13.87	<.0001	0.05	-1.7673	-1.3165
AoE+PSCs	6ug/ml	HE+PSCs	2ug/ml	-0.7198	0.1162	36	-6.19	<.0001	0.05	-0.9555	-0.4841
AoE+PSCs	6ug/ml	HE+PSCs	6ug/ml	-0.1132	0.1222	36	-0.93	0.3603	0.05	-0.3610	0.1346
E10.5AGM	0ug/ml	E10.5AGM	2ug/ml	0.4006	0.08837	36	4.53	<.0001	0.05	0.2214	0.5798
E10.5AGM	0ug/ml	E10.5AGM	6ug/ml	1.2982	0.09403	36	13.81	<.0001	0.05	1.1075	1.4889
E10.5AGM	0ug/ml	HE+PSCs	0ug/ml	0.6571	0.08988	36	7.31	<.0001	0.05	0.4748	0.8394
E10.5AGM	0ug/ml	HE+PSCs	2ug/ml	1.4792	0.09608	36	15.40	<.0001	0.05	1.2844	1.6741
E10.5AGM	0ug/ml	HE+PSCs	6ug/ml	2.0858	0.1032	36	20.20	<.0001	0.05	1.8764	2.2952
E10.5AGM	2ug/ml	E10.5AGM	6ug/ml	0.8976	0.09498	36	9.45	<.0001	0.05	0.7049	1.0902
E10.5AGM	2ug/ml	HE+PSCs	0ug/ml	0.2564	0.09087	36	2.82	0.0077	0.05	0.07215	0.4407
E10.5AGM	2ug/ml	HE+PSCs	2ug/ml	1.0786	0.09701	36	11.12	<.0001	0.05	0.8819	1.2753
E10.5AGM	2ug/ml	HE+PSCs	6ug/ml	1.6852	0.1041	36	16.19	<.0001	0.05	1.4741	1.8963

The GLIMMIX Procedure

Differences of Condition*Treatment Least Squares Means											
Condition	Treatment	_Condition	_Treatment	Estimate	Standard Error	DF	t Value	Pr > |t|	Alpha	Lower	Upper
E10.5AGM	6ug/ml	HE+PSCs	0ug/ml	-0.6411	0.09639	36	-6.65	<.0001	0.05	-0.8366	-0.4456
E10.5AGM	6ug/ml	HE+PSCs	2ug/ml	0.1810	0.1022	36	1.77	0.0849	0.05	-0.02621	0.3883
E10.5AGM	6ug/ml	HE+PSCs	6ug/ml	0.7876	0.1089	36	7.23	<.0001	0.05	0.5667	1.0085
HE+PSCs	0ug/ml	HE+PSCs	2ug/ml	0.8222	0.09839	36	8.36	<.0001	0.05	0.6226	1.0217
HE+PSCs	0ug/ml	HE+PSCs	6ug/ml	1.4287	0.1054	36	13.56	<.0001	0.05	1.2150	1.6424
HE+PSCs	2ug/ml	HE+PSCs	6ug/ml	0.6066	0.1107	36	5.48	<.0001	0.05	0.3820	0.8311

Colony*Condit*Treatm Least Squares Means														
Colony_Type	Condition	Treatment	Estimate	Standard Error	DF	t Value	Pr > |t|	Alpha	Lower	Upper	Mean	Standard Error Mean	Lower Mean	Upper Mean
BFU-E	AoE+PSCs	0ug/ml	4.7699	0.07872	36	60.60	<.0001	0.05	4.6103	4.9296	117.91	9.2815	100.51	138.32
BFU-E	AoE+PSCs	2ug/ml	3.7534	0.1055	36	35.57	<.0001	0.05	3.5394	3.9674	42.6668	4.5022	34.4468	52.8482
BFU-E	AoE+PSCs	6ug/ml	2.6998	0.1601	36	16.87	<.0001	0.05	2.3752	3.0244	14.8771	2.3813	10.7532	20.5827
BFU-E	E10.5AGM	0ug/ml	5.2887	0.07108	36	74.41	<.0001	0.05	5.1446	5.4329	198.09	14.0795	171.49	228.80
BFU-E	E10.5AGM	2ug/ml	4.8254	0.07772	36	62.09	<.0001	0.05	4.6677	4.9830	124.63	9.6857	106.46	145.91
BFU-E	E10.5AGM	6ug/ml	3.7216	0.1067	36	34.88	<.0001	0.05	3.5052	3.9380	41.3300	4.4101	33.2875	51.3157
BFU-E	HE+PSCs	0ug/ml	4.5594	0.08275	36	55.10	<.0001	0.05	4.3915	4.7272	95.5225	7.9043	80.7647	112.98
BFU-E	HE+PSCs	2ug/ml	3.9676	0.09830	36	40.36	<.0001	0.05	3.7682	4.1669	52.8550	5.1954	43.3020	64.5154
BFU-E	HE+PSCs	6ug/ml	3.1701	0.1315	36	24.11	<.0001	0.05	2.9035	3.4367	23.8097	3.1300	18.2376	31.0843
CFU-GEMM	AoE+PSCs	0ug/ml	4.6243	0.08148	36	56.75	<.0001	0.05	4.4590	4.7895	101.93	8.3052	86.4026	120.24
CFU-GEMM	AoE+PSCs	2ug/ml	4.0353	0.09608	36	42.00	<.0001	0.05	3.8404	4.2301	56.5577	5.4339	46.5444	68.7252
CFU-GEMM	AoE+PSCs	6ug/ml	2.9365	0.1447	36	20.29	<.0001	0.05	2.6430	3.2299	18.8490	2.7273	14.0554	25.2774
CFU-GEMM	E10.5AGM	0ug/ml	5.1806	0.07242	36	71.54	<.0001	0.05	5.0337	5.3274	177.78	12.8743	153.50	205.91
CFU-GEMM	E10.5AGM	2ug/ml	5.0553	0.07411	36	68.22	<.0001	0.05	4.9050	5.2056	156.85	11.6238	134.96	182.29

The GLIMMIX Procedure

Colony*Condit*Treatm Least Squares Means														
Colony_Type	Condition	Treatment	Estimate	Standard Error	DF	t Value	Pr > |t|	Alpha	Lower	Upper	Mean	Standard Error Mean	Lower Mean	Upper Mean
CFU-GEMM	E10.5AGM	6ug/ml	4.1511	0.09269	36	44.78	<.0001	0.05	3.9631	4.3390	63.5010	5.8861	52.6184	76.6343
CFU-GEMM	HE+PSCs	0ug/ml	4.4724	0.08463	36	52.84	<.0001	0.05	4.3007	4.6440	87.5628	7.4108	73.7522	103.96
CFU-GEMM	HE+PSCs	2ug/ml	3.4732	0.1170	36	29.69	<.0001	0.05	3.2359	3.7104	32.2382	3.7710	25.4298	40.8695
CFU-GEMM	HE+PSCs	6ug/ml	3.2110	0.1293	36	24.83	<.0001	0.05	2.9488	3.4733	24.8047	3.2078	19.0822	32.2434
CFU-GM	AoE+PSCs	0ug/ml	6.1938	0.06365	36	97.31	<.0001	0.05	6.0647	6.3228	489.68	31.1686	430.38	557.16
CFU-GM	AoE+PSCs	2ug/ml	5.4213	0.06962	36	77.87	<.0001	0.05	5.2801	5.5625	226.18	15.7455	196.40	260.47
CFU-GM	AoE+PSCs	6ug/ml	4.8746	0.07692	36	63.37	<.0001	0.05	4.7186	5.0306	130.93	10.0714	112.01	153.03
CFU-GM	E10.5AGM	0ug/ml	6.6387	0.06170	36	107.59	<.0001	0.05	6.5135	6.7638	764.07	47.1441	674.19	865.92
CFU-GM	E10.5AGM	2ug/ml	6.0254	0.06462	36	93.24	<.0001	0.05	5.8943	6.1564	413.80	26.7410	362.97	471.75
CFU-GM	E10.5AGM	6ug/ml	5.3407	0.07049	36	75.77	<.0001	0.05	5.1978	5.4837	208.66	14.7086	180.87	240.73
CFU-GM	HE+PSCs	0ug/ml	6.1050	0.06415	36	95.17	<.0001	0.05	5.9749	6.2351	448.09	28.7449	393.42	510.35
CFU-GM	HE+PSCs	2ug/ml	5.2295	0.07180	36	72.84	<.0001	0.05	5.0839	5.3751	186.70	13.4047	161.40	215.97
CFU-GM	HE+PSCs	6ug/ml	4.4694	0.08477	36	52.73	<.0001	0.05	4.2975	4.6413	87.3032	7.4003	73.5139	103.68

The GLIMMIX Procedure

Differences of Colony*Condit*Treatm Least Squares Means													
Colony_Type	Condition	Treatment	_Colony_Type	_Condition	_Treatment	Estimate	Standard Error	DF	t Value	Pr > |t|	Alpha	Lower	Upper
BFU-E	AoE+PSCs	0ug/ml	BFU-E	AoE+PSCs	2ug/ml	1.0165	0.1316	36	7.72	<.0001	0.05	0.7495	1.2835
BFU-E	AoE+PSCs	0ug/ml	BFU-E	AoE+PSCs	6ug/ml	2.0701	0.1784	36	11.61	<.0001	0.05	1.7084	2.4319
BFU-E	AoE+PSCs	0ug/ml	BFU-E	E10.5AGM	0ug/ml	-0.5188	0.1061	36	-4.89	<.0001	0.05	-0.7339	-0.3037
BFU-E	AoE+PSCs	0ug/ml	BFU-E	E10.5AGM	2ug/ml	-0.05542	0.1106	36	-0.50	0.6194	0.05	-0.2798	0.1689
BFU-E	AoE+PSCs	0ug/ml	BFU-E	E10.5AGM	6ug/ml	1.0483	0.1326	36	7.91	<.0001	0.05	0.7794	1.3173
BFU-E	AoE+PSCs	0ug/ml	BFU-E	HE+PSCs	0ug/ml	0.2106	0.1142	36	1.84	0.0735	0.05	-0.02105	0.4422
BFU-E	AoE+PSCs	0ug/ml	BFU-E	HE+PSCs	2ug/ml	0.8024	0.1259	36	6.37	<.0001	0.05	0.5470	1.0578
BFU-E	AoE+PSCs	0ug/ml	BFU-E	HE+PSCs	6ug/ml	1.5998	0.1532	36	10.44	<.0001	0.05	1.2891	1.9106
BFU-E	AoE+PSCs	0ug/ml	CFU-GEMM	AoE+PSCs	0ug/ml	0.1457	0.07806	36	1.87	0.0702	0.05	-0.01265	0.3040
BFU-E	AoE+PSCs	0ug/ml	CFU-GEMM	AoE+PSCs	2ug/ml	0.7347	0.1242	36	5.91	<.0001	0.05	0.4828	0.9866
BFU-E	AoE+PSCs	0ug/ml	CFU-GEMM	AoE+PSCs	6ug/ml	1.8335	0.1647	36	11.13	<.0001	0.05	1.4994	2.1675
BFU-E	AoE+PSCs	0ug/ml	CFU-GEMM	E10.5AGM	0ug/ml	-0.4106	0.1070	36	-3.84	0.0005	0.05	-0.6276	-0.1937
BFU-E	AoE+PSCs	0ug/ml	CFU-GEMM	E10.5AGM	2ug/ml	-0.2854	0.1081	36	-2.64	0.0122	0.05	-0.5046	-0.06611
BFU-E	AoE+PSCs	0ug/ml	CFU-GEMM	E10.5AGM	6ug/ml	0.6189	0.1216	36	5.09	<.0001	0.05	0.3722	0.8655
BFU-E	AoE+PSCs	0ug/ml	CFU-GEMM	HE+PSCs	0ug/ml	0.2976	0.1156	36	2.57	0.0143	0.05	0.06316	0.5320
BFU-E	AoE+PSCs	0ug/ml	CFU-GEMM	HE+PSCs	2ug/ml	1.2968	0.1410	36	9.20	<.0001	0.05	1.0108	1.5827
BFU-E	AoE+PSCs	0ug/ml	CFU-GEMM	HE+PSCs	6ug/ml	1.5589	0.1514	36	10.30	<.0001	0.05	1.2519	1.8659
BFU-E	AoE+PSCs	0ug/ml	CFU-GM	AoE+PSCs	0ug/ml	-1.4238	0.05921	36	-24.05	<.0001	0.05	-1.5439	-1.3038
BFU-E	AoE+PSCs	0ug/ml	CFU-GM	AoE+PSCs	2ug/ml	-0.6514	0.1051	36	-6.20	<.0001	0.05	-0.8645	-0.4383
BFU-E	AoE+PSCs	0ug/ml	CFU-GM	AoE+PSCs	6ug/ml	-0.1047	0.1101	36	-0.95	0.3478	0.05	-0.3279	0.1185
BFU-E	AoE+PSCs	0ug/ml	CFU-GM	E10.5AGM	0ug/ml	-1.8687	0.1000	36	-18.68	<.0001	0.05	-2.0716	-1.6659
BFU-E	AoE+PSCs	0ug/ml	CFU-GM	E10.5AGM	2ug/ml	-1.2555	0.1018	36	-12.33	<.0001	0.05	-1.4620	-1.0489
BFU-E	AoE+PSCs	0ug/ml	CFU-GM	E10.5AGM	6ug/ml	-0.5708	0.1057	36	-5.40	<.0001	0.05	-0.7851	-0.3565
BFU-E	AoE+PSCs	0ug/ml	CFU-GM	HE+PSCs	0ug/ml	-1.3351	0.1015	36	-13.15	<.0001	0.05	-1.5410	-1.1291

The GLIMMIX Procedure

Differences of Colony*Condit*Treatm Least Squares Means													
Colony_Type	Condition	Treatment	_Colony_Type	_Condition	_Treatment	Estimate	Standard Error	DF	t Value	Pr > |t|	Alpha	Lower	Upper
BFU-E	AoE+PSCs	0ug/ml	CFU-GM	HE+PSCs	2ug/ml	-0.4596	0.1065	36	-4.31	0.0001	0.05	-0.6757	-0.2435
BFU-E	AoE+PSCs	0ug/ml	CFU-GM	HE+PSCs	6ug/ml	0.3005	0.1157	36	2.60	0.0135	0.05	0.06594	0.5351
BFU-E	AoE+PSCs	2ug/ml	BFU-E	AoE+PSCs	6ug/ml	1.0536	0.1917	36	5.50	<.0001	0.05	0.6648	1.4424
BFU-E	AoE+PSCs	2ug/ml	BFU-E	E10.5AGM	0ug/ml	-1.5353	0.1272	36	-12.07	<.0001	0.05	-1.7933	-1.2773
BFU-E	AoE+PSCs	2ug/ml	BFU-E	E10.5AGM	2ug/ml	-1.0719	0.1310	36	-8.18	<.0001	0.05	-1.3377	-0.8062
BFU-E	AoE+PSCs	2ug/ml	BFU-E	E10.5AGM	6ug/ml	0.03183	0.1501	36	0.21	0.8332	0.05	-0.2725	0.3362
BFU-E	AoE+PSCs	2ug/ml	BFU-E	HE+PSCs	0ug/ml	-0.8059	0.1341	36	-6.01	<.0001	0.05	-1.0779	-0.5340
BFU-E	AoE+PSCs	2ug/ml	BFU-E	HE+PSCs	2ug/ml	-0.2141	0.1442	36	-1.48	0.1463	0.05	-0.5066	0.07833
BFU-E	AoE+PSCs	2ug/ml	BFU-E	HE+PSCs	6ug/ml	0.5833	0.1686	36	3.46	0.0014	0.05	0.2415	0.9252
BFU-E	AoE+PSCs	2ug/ml	CFU-GEMM	AoE+PSCs	0ug/ml	-0.8708	0.1333	36	-6.53	<.0001	0.05	-1.1412	-0.6005
BFU-E	AoE+PSCs	2ug/ml	CFU-GEMM	AoE+PSCs	2ug/ml	-0.2818	0.1166	36	-2.42	0.0208	0.05	-0.5183	-0.04536
BFU-E	AoE+PSCs	2ug/ml	CFU-GEMM	AoE+PSCs	6ug/ml	0.8170	0.1791	36	4.56	<.0001	0.05	0.4538	1.1801
BFU-E	AoE+PSCs	2ug/ml	CFU-GEMM	E10.5AGM	0ug/ml	-1.4271	0.1280	36	-11.15	<.0001	0.05	-1.6867	-1.1676
BFU-E	AoE+PSCs	2ug/ml	CFU-GEMM	E10.5AGM	2ug/ml	-1.3019	0.1289	36	-10.10	<.0001	0.05	-1.5634	-1.0404
BFU-E	AoE+PSCs	2ug/ml	CFU-GEMM	E10.5AGM	6ug/ml	-0.3976	0.1404	36	-2.83	0.0075	0.05	-0.6825	-0.1128
BFU-E	AoE+PSCs	2ug/ml	CFU-GEMM	HE+PSCs	0ug/ml	-0.7189	0.1353	36	-5.32	<.0001	0.05	-0.9933	-0.4446
BFU-E	AoE+PSCs	2ug/ml	CFU-GEMM	HE+PSCs	2ug/ml	0.2803	0.1575	36	1.78	0.0837	0.05	-0.03922	0.5998
BFU-E	AoE+PSCs	2ug/ml	CFU-GEMM	HE+PSCs	6ug/ml	0.5424	0.1669	36	3.25	0.0025	0.05	0.2039	0.8809
BFU-E	AoE+PSCs	2ug/ml	CFU-GM	AoE+PSCs	0ug/ml	-2.4403	0.1232	36	-19.80	<.0001	0.05	-2.6903	-2.1904
BFU-E	AoE+PSCs	2ug/ml	CFU-GM	AoE+PSCs	2ug/ml	-1.6679	0.09598	36	-17.38	<.0001	0.05	-1.8626	-1.4732
BFU-E	AoE+PSCs	2ug/ml	CFU-GM	AoE+PSCs	6ug/ml	-1.1212	0.1306	36	-8.59	<.0001	0.05	-1.3860	-0.8564
BFU-E	AoE+PSCs	2ug/ml	CFU-GM	E10.5AGM	0ug/ml	-2.8852	0.1222	36	-23.60	<.0001	0.05	-3.1331	-2.6373
BFU-E	AoE+PSCs	2ug/ml	CFU-GM	E10.5AGM	2ug/ml	-2.2720	0.1237	36	-18.36	<.0001	0.05	-2.5229	-2.0210
BFU-E	AoE+PSCs	2ug/ml	CFU-GM	E10.5AGM	6ug/ml	-1.5873	0.1269	36	-12.51	<.0001	0.05	-1.8446	-1.3300

The GLIMMIX Procedure

Differences of Colony*Condit*Treatm Least Squares Means													
Colony_Type	Condition	Treatment	_Colony_Type	_Condition	_Treatment	Estimate	Standard Error	DF	t Value	Pr > |t|	Alpha	Lower	Upper
BFU-E	AoE+PSCs	2ug/ml	CFU-GM	HE+PSCs	0ug/ml	-2.3516	0.1235	36	-19.04	<.0001	0.05	-2.6020	-2.1011
BFU-E	AoE+PSCs	2ug/ml	CFU-GM	HE+PSCs	2ug/ml	-1.4761	0.1276	36	-11.57	<.0001	0.05	-1.7349	-1.2173
BFU-E	AoE+PSCs	2ug/ml	CFU-GM	HE+PSCs	6ug/ml	-0.7160	0.1353	36	-5.29	<.0001	0.05	-0.9904	-0.4415
BFU-E	AoE+PSCs	6ug/ml	BFU-E	E10.5AGM	0ug/ml	-2.5889	0.1751	36	-14.78	<.0001	0.05	-2.9441	-2.2337
BFU-E	AoE+PSCs	6ug/ml	BFU-E	E10.5AGM	2ug/ml	-2.1255	0.1779	36	-11.95	<.0001	0.05	-2.4864	-1.7647
BFU-E	AoE+PSCs	6ug/ml	BFU-E	E10.5AGM	6ug/ml	-1.0218	0.1924	36	-5.31	<.0001	0.05	-1.4119	-0.6317
BFU-E	AoE+PSCs	6ug/ml	BFU-E	HE+PSCs	0ug/ml	-1.8595	0.1802	36	-10.32	<.0001	0.05	-2.2250	-1.4941
BFU-E	AoE+PSCs	6ug/ml	BFU-E	HE+PSCs	2ug/ml	-1.2677	0.1878	36	-6.75	<.0001	0.05	-1.6487	-0.8868
BFU-E	AoE+PSCs	6ug/ml	BFU-E	HE+PSCs	6ug/ml	-0.4703	0.2071	36	-2.27	0.0292	0.05	-0.8903	-0.05024
BFU-E	AoE+PSCs	6ug/ml	CFU-GEMM	AoE+PSCs	0ug/ml	-1.9244	0.1796	36	-10.71	<.0001	0.05	-2.2887	-1.5602
BFU-E	AoE+PSCs	6ug/ml	CFU-GEMM	AoE+PSCs	2ug/ml	-1.3354	0.1867	36	-7.15	<.0001	0.05	-1.7140	-0.9568
BFU-E	AoE+PSCs	6ug/ml	CFU-GEMM	AoE+PSCs	6ug/ml	-0.2366	0.1994	36	-1.19	0.2432	0.05	-0.6411	0.1678
BFU-E	AoE+PSCs	6ug/ml	CFU-GEMM	E10.5AGM	0ug/ml	-2.4807	0.1757	36	-14.12	<.0001	0.05	-2.8370	-2.1244
BFU-E	AoE+PSCs	6ug/ml	CFU-GEMM	E10.5AGM	2ug/ml	-2.3555	0.1764	36	-13.35	<.0001	0.05	-2.7132	-1.9978
BFU-E	AoE+PSCs	6ug/ml	CFU-GEMM	E10.5AGM	6ug/ml	-1.4512	0.1849	36	-7.85	<.0001	0.05	-1.8263	-1.0761
BFU-E	AoE+PSCs	6ug/ml	CFU-GEMM	HE+PSCs	0ug/ml	-1.7725	0.1811	36	-9.79	<.0001	0.05	-2.1397	-1.4053
BFU-E	AoE+PSCs	6ug/ml	CFU-GEMM	HE+PSCs	2ug/ml	-0.7733	0.1982	36	-3.90	0.0004	0.05	-1.1754	-0.3713
BFU-E	AoE+PSCs	6ug/ml	CFU-GEMM	HE+PSCs	6ug/ml	-0.5112	0.2058	36	-2.48	0.0178	0.05	-0.9285	-0.09392
BFU-E	AoE+PSCs	6ug/ml	CFU-GM	AoE+PSCs	0ug/ml	-3.4939	0.1723	36	-20.28	<.0001	0.05	-3.8433	-3.1446
BFU-E	AoE+PSCs	6ug/ml	CFU-GM	AoE+PSCs	2ug/ml	-2.7215	0.1745	36	-15.59	<.0001	0.05	-3.0755	-2.3675
BFU-E	AoE+PSCs	6ug/ml	CFU-GM	AoE+PSCs	6ug/ml	-2.1748	0.1573	36	-13.82	<.0001	0.05	-2.4939	-1.8557
BFU-E	AoE+PSCs	6ug/ml	CFU-GM	E10.5AGM	0ug/ml	-3.9388	0.1715	36	-22.96	<.0001	0.05	-4.2867	-3.5909
BFU-E	AoE+PSCs	6ug/ml	CFU-GM	E10.5AGM	2ug/ml	-3.3256	0.1726	36	-19.27	<.0001	0.05	-3.6756	-2.9755
BFU-E	AoE+PSCs	6ug/ml	CFU-GM	E10.5AGM	6ug/ml	-2.6409	0.1749	36	-15.10	<.0001	0.05	-2.9956	-2.2862

The GLIMMIX Procedure

Differences of Colony*Condit*Treatm Least Squares Means													
Colony_Type	Condition	Treatment	_Colony_Type	_Condition	_Treatment	Estimate	Standard Error	DF	t Value	Pr > |t|	Alpha	Lower	Upper
BFU-E	AoE+PSCs	6ug/ml	CFU-GM	HE+PSCs	0ug/ml	-3.4052	0.1724	36	-19.75	<.0001	0.05	-3.7549	-3.0555
BFU-E	AoE+PSCs	6ug/ml	CFU-GM	HE+PSCs	2ug/ml	-2.5297	0.1754	36	-14.42	<.0001	0.05	-2.8855	-2.1739
BFU-E	AoE+PSCs	6ug/ml	CFU-GM	HE+PSCs	6ug/ml	-1.7696	0.1811	36	-9.77	<.0001	0.05	-2.1368	-1.4023
BFU-E	E10.5AGM	0ug/ml	BFU-E	E10.5AGM	2ug/ml	0.4634	0.1053	36	4.40	<.0001	0.05	0.2498	0.6769
BFU-E	E10.5AGM	0ug/ml	BFU-E	E10.5AGM	6ug/ml	1.5671	0.1282	36	12.22	<.0001	0.05	1.3071	1.8271
BFU-E	E10.5AGM	0ug/ml	BFU-E	HE+PSCs	0ug/ml	0.7293	0.1091	36	6.69	<.0001	0.05	0.5081	0.9506
BFU-E	E10.5AGM	0ug/ml	BFU-E	HE+PSCs	2ug/ml	1.3212	0.1213	36	10.89	<.0001	0.05	1.0751	1.5672
BFU-E	E10.5AGM	0ug/ml	BFU-E	HE+PSCs	6ug/ml	2.1186	0.1494	36	14.18	<.0001	0.05	1.8155	2.4217
BFU-E	E10.5AGM	0ug/ml	CFU-GEMM	AoE+PSCs	0ug/ml	0.6644	0.1081	36	6.15	<.0001	0.05	0.4451	0.8837
BFU-E	E10.5AGM	0ug/ml	CFU-GEMM	AoE+PSCs	2ug/ml	1.2534	0.1195	36	10.49	<.0001	0.05	1.0111	1.4958
BFU-E	E10.5AGM	0ug/ml	CFU-GEMM	AoE+PSCs	6ug/ml	2.3522	0.1612	36	14.59	<.0001	0.05	2.0253	2.6792
BFU-E	E10.5AGM	0ug/ml	CFU-GEMM	E10.5AGM	0ug/ml	0.1081	0.05961	36	1.81	0.0780	0.05	-0.01276	0.2290
BFU-E	E10.5AGM	0ug/ml	CFU-GEMM	E10.5AGM	2ug/ml	0.2334	0.1027	36	2.27	0.0291	0.05	0.02515	0.4417
BFU-E	E10.5AGM	0ug/ml	CFU-GEMM	E10.5AGM	6ug/ml	1.1376	0.1168	36	9.74	<.0001	0.05	0.9008	1.3745
BFU-E	E10.5AGM	0ug/ml	CFU-GEMM	HE+PSCs	0ug/ml	0.8163	0.1105	36	7.39	<.0001	0.05	0.5922	1.0405
BFU-E	E10.5AGM	0ug/ml	CFU-GEMM	HE+PSCs	2ug/ml	1.8156	0.1369	36	13.26	<.0001	0.05	1.5380	2.0931
BFU-E	E10.5AGM	0ug/ml	CFU-GEMM	HE+PSCs	6ug/ml	2.0777	0.1476	36	14.08	<.0001	0.05	1.7784	2.3770
BFU-E	E10.5AGM	0ug/ml	CFU-GM	AoE+PSCs	0ug/ml	-0.9051	0.09541	36	-9.49	<.0001	0.05	-1.0986	-0.7116
BFU-E	E10.5AGM	0ug/ml	CFU-GM	AoE+PSCs	2ug/ml	-0.1326	0.09949	36	-1.33	0.1909	0.05	-0.3344	0.06916
BFU-E	E10.5AGM	0ug/ml	CFU-GM	AoE+PSCs	6ug/ml	0.4141	0.1047	36	3.95	0.0003	0.05	0.2017	0.6265
BFU-E	E10.5AGM	0ug/ml	CFU-GM	E10.5AGM	0ug/ml	-1.3500	0.04600	36	-29.34	<.0001	0.05	-1.4433	-1.2566
BFU-E	E10.5AGM	0ug/ml	CFU-GM	E10.5AGM	2ug/ml	-0.7367	0.09606	36	-7.67	<.0001	0.05	-0.9315	-0.5419
BFU-E	E10.5AGM	0ug/ml	CFU-GM	E10.5AGM	6ug/ml	-0.05201	0.1001	36	-0.52	0.6065	0.05	-0.2550	0.1510
BFU-E	E10.5AGM	0ug/ml	CFU-GM	HE+PSCs	0ug/ml	-0.8163	0.09575	36	-8.53	<.0001	0.05	-1.0105	-0.6221

The GLIMMIX Procedure

Differences of Colony*Condit*Treatm Least Squares Means													
Colony_Type	Condition	Treatment	_Colony_Type	_Condition	_Treatment	Estimate	Standard Error	DF	t Value	Pr > |t|	Alpha	Lower	Upper
BFU-E	E10.5AGM	0ug/ml	CFU-GM	HE+PSCs	2ug/ml	0.05918	0.1010	36	0.59	0.5617	0.05	-0.1457	0.2641
BFU-E	E10.5AGM	0ug/ml	CFU-GM	HE+PSCs	6ug/ml	0.8193	0.1106	36	7.41	<.0001	0.05	0.5950	1.0437
BFU-E	E10.5AGM	2ug/ml	BFU-E	E10.5AGM	6ug/ml	1.1038	0.1320	36	8.36	<.0001	0.05	0.8360	1.3715
BFU-E	E10.5AGM	2ug/ml	BFU-E	HE+PSCs	0ug/ml	0.2660	0.1135	36	2.34	0.0248	0.05	0.03576	0.4962
BFU-E	E10.5AGM	2ug/ml	BFU-E	HE+PSCs	2ug/ml	0.8578	0.1253	36	6.85	<.0001	0.05	0.6037	1.1119
BFU-E	E10.5AGM	2ug/ml	BFU-E	HE+PSCs	6ug/ml	1.6553	0.1527	36	10.84	<.0001	0.05	1.3456	1.9650
BFU-E	E10.5AGM	2ug/ml	CFU-GEMM	AoE+PSCs	0ug/ml	0.2011	0.1126	36	1.79	0.0826	0.05	-0.02728	0.4294
BFU-E	E10.5AGM	2ug/ml	CFU-GEMM	AoE+PSCs	2ug/ml	0.7901	0.1236	36	6.39	<.0001	0.05	0.5395	1.0407
BFU-E	E10.5AGM	2ug/ml	CFU-GEMM	AoE+PSCs	6ug/ml	1.8889	0.1642	36	11.50	<.0001	0.05	1.5558	2.2220
BFU-E	E10.5AGM	2ug/ml	CFU-GEMM	E10.5AGM	0ug/ml	-0.3552	0.1062	36	-3.34	0.0019	0.05	-0.5706	-0.1398
BFU-E	E10.5AGM	2ug/ml	CFU-GEMM	E10.5AGM	2ug/ml	-0.2299	0.06918	36	-3.32	0.0020	0.05	-0.3702	-0.08965
BFU-E	E10.5AGM	2ug/ml	CFU-GEMM	E10.5AGM	6ug/ml	0.6743	0.1210	36	5.57	<.0001	0.05	0.4290	0.9196
BFU-E	E10.5AGM	2ug/ml	CFU-GEMM	HE+PSCs	0ug/ml	0.3530	0.1149	36	3.07	0.0040	0.05	0.1200	0.5860
BFU-E	E10.5AGM	2ug/ml	CFU-GEMM	HE+PSCs	2ug/ml	1.3522	0.1404	36	9.63	<.0001	0.05	1.0674	1.6370
BFU-E	E10.5AGM	2ug/ml	CFU-GEMM	HE+PSCs	6ug/ml	1.6143	0.1509	36	10.70	<.0001	0.05	1.3083	1.9203
BFU-E	E10.5AGM	2ug/ml	CFU-GM	AoE+PSCs	0ug/ml	-1.3684	0.1005	36	-13.62	<.0001	0.05	-1.5721	-1.1647
BFU-E	E10.5AGM	2ug/ml	CFU-GM	AoE+PSCs	2ug/ml	-0.5960	0.1043	36	-5.71	<.0001	0.05	-0.8076	-0.3844
BFU-E	E10.5AGM	2ug/ml	CFU-GM	AoE+PSCs	6ug/ml	-0.04928	0.1093	36	-0.45	0.6549	0.05	-0.2710	0.1725
BFU-E	E10.5AGM	2ug/ml	CFU-GM	E10.5AGM	0ug/ml	-1.8133	0.09923	36	-18.27	<.0001	0.05	-2.0146	-1.6121
BFU-E	E10.5AGM	2ug/ml	CFU-GM	E10.5AGM	2ug/ml	-1.2000	0.05891	36	-20.37	<.0001	0.05	-1.3195	-1.0806
BFU-E	E10.5AGM	2ug/ml	CFU-GM	E10.5AGM	6ug/ml	-0.5154	0.1049	36	-4.91	<.0001	0.05	-0.7281	-0.3026
BFU-E	E10.5AGM	2ug/ml	CFU-GM	HE+PSCs	0ug/ml	-1.2796	0.1008	36	-12.70	<.0001	0.05	-1.4840	-1.0753
BFU-E	E10.5AGM	2ug/ml	CFU-GM	HE+PSCs	2ug/ml	-0.4042	0.1058	36	-3.82	0.0005	0.05	-0.6187	-0.1896
BFU-E	E10.5AGM	2ug/ml	CFU-GM	HE+PSCs	6ug/ml	0.3560	0.1150	36	3.10	0.0038	0.05	0.1227	0.5892

The GLIMMIX Procedure

Differences of Colony*Condit*Treatm Least Squares Means													
Colony_Type	Condition	Treatment	_Colony_Type	_Condition	_Treatment	Estimate	Standard Error	DF	t Value	Pr > |t|	Alpha	Lower	Upper
BFU-E	E10.5AGM	6ug/ml	BFU-E	HE+PSCs	0ug/ml	-0.8378	0.1350	36	-6.20	<.0001	0.05	-1.1116	-0.5639
BFU-E	E10.5AGM	6ug/ml	BFU-E	HE+PSCs	2ug/ml	-0.2460	0.1451	36	-1.70	0.0986	0.05	-0.5402	0.04826
BFU-E	E10.5AGM	6ug/ml	BFU-E	HE+PSCs	6ug/ml	0.5515	0.1693	36	3.26	0.0025	0.05	0.2082	0.8948
BFU-E	E10.5AGM	6ug/ml	CFU-GEMM	AoE+PSCs	0ug/ml	-0.9027	0.1343	36	-6.72	<.0001	0.05	-1.1750	-0.6304
BFU-E	E10.5AGM	6ug/ml	CFU-GEMM	AoE+PSCs	2ug/ml	-0.3137	0.1436	36	-2.18	0.0355	0.05	-0.6048	-0.02249
BFU-E	E10.5AGM	6ug/ml	CFU-GEMM	AoE+PSCs	6ug/ml	0.7851	0.1798	36	4.37	0.0001	0.05	0.4205	1.1497
BFU-E	E10.5AGM	6ug/ml	CFU-GEMM	E10.5AGM	0ug/ml	-1.4590	0.1290	36	-11.31	<.0001	0.05	-1.7205	-1.1974
BFU-E	E10.5AGM	6ug/ml	CFU-GEMM	E10.5AGM	2ug/ml	-1.3337	0.1299	36	-10.27	<.0001	0.05	-1.5972	-1.0702
BFU-E	E10.5AGM	6ug/ml	CFU-GEMM	E10.5AGM	6ug/ml	-0.4295	0.1149	36	-3.74	0.0006	0.05	-0.6626	-0.1964
BFU-E	E10.5AGM	6ug/ml	CFU-GEMM	HE+PSCs	0ug/ml	-0.7508	0.1362	36	-5.51	<.0001	0.05	-1.0270	-0.4746
BFU-E	E10.5AGM	6ug/ml	CFU-GEMM	HE+PSCs	2ug/ml	0.2484	0.1583	36	1.57	0.1254	0.05	-0.07266	0.5695
BFU-E	E10.5AGM	6ug/ml	CFU-GEMM	HE+PSCs	6ug/ml	0.5106	0.1676	36	3.05	0.0043	0.05	0.1706	0.8505
BFU-E	E10.5AGM	6ug/ml	CFU-GM	AoE+PSCs	0ug/ml	-2.4722	0.1242	36	-19.90	<.0001	0.05	-2.7242	-2.2202
BFU-E	E10.5AGM	6ug/ml	CFU-GM	AoE+PSCs	2ug/ml	-1.6997	0.1274	36	-13.34	<.0001	0.05	-1.9581	-1.4414
BFU-E	E10.5AGM	6ug/ml	CFU-GM	AoE+PSCs	6ug/ml	-1.1530	0.1315	36	-8.77	<.0001	0.05	-1.4198	-0.8863
BFU-E	E10.5AGM	6ug/ml	CFU-GM	E10.5AGM	0ug/ml	-2.9171	0.1233	36	-23.67	<.0001	0.05	-3.1670	-2.6671
BFU-E	E10.5AGM	6ug/ml	CFU-GM	E10.5AGM	2ug/ml	-2.3038	0.1247	36	-18.47	<.0001	0.05	-2.5568	-2.0508
BFU-E	E10.5AGM	6ug/ml	CFU-GM	E10.5AGM	6ug/ml	-1.6191	0.09791	36	-16.54	<.0001	0.05	-1.8177	-1.4205
BFU-E	E10.5AGM	6ug/ml	CFU-GM	HE+PSCs	0ug/ml	-2.3834	0.1245	36	-19.14	<.0001	0.05	-2.6359	-2.1309
BFU-E	E10.5AGM	6ug/ml	CFU-GM	HE+PSCs	2ug/ml	-1.5079	0.1286	36	-11.73	<.0001	0.05	-1.7688	-1.2471
BFU-E	E10.5AGM	6ug/ml	CFU-GM	HE+PSCs	6ug/ml	-0.7478	0.1363	36	-5.49	<.0001	0.05	-1.0241	-0.4715
BFU-E	HE+PSCs	0ug/ml	BFU-E	HE+PSCs	2ug/ml	0.5918	0.1285	36	4.61	<.0001	0.05	0.3312	0.8524
BFU-E	HE+PSCs	0ug/ml	BFU-E	HE+PSCs	6ug/ml	1.3893	0.1553	36	8.94	<.0001	0.05	1.0743	1.7043
BFU-E	HE+PSCs	0ug/ml	CFU-GEMM	AoE+PSCs	0ug/ml	-0.06491	0.1161	36	-0.56	0.5797	0.05	-0.3004	0.1706

The GLIMMIX Procedure

Differences of Colony*Condit*Treatm Least Squares Means													
Colony_Type	Condition	Treatment	_Colony_Type	_Condition	_Treatment	Estimate	Standard Error	DF	t Value	Pr > |t|	Alpha	Lower	Upper
BFU-E	HE+PSCs	0ug/ml	CFU-GEMM	AoE+PSCs	2ug/ml	0.5241	0.1268	36	4.13	0.0002	0.05	0.2669	0.7813
BFU-E	HE+PSCs	0ug/ml	CFU-GEMM	AoE+PSCs	6ug/ml	1.6229	0.1667	36	9.74	<.0001	0.05	1.2849	1.9609
BFU-E	HE+PSCs	0ug/ml	CFU-GEMM	E10.5AGM	0ug/ml	-0.6212	0.1100	36	-5.65	<.0001	0.05	-0.8442	-0.3982
BFU-E	HE+PSCs	0ug/ml	CFU-GEMM	E10.5AGM	2ug/ml	-0.4959	0.1111	36	-4.46	<.0001	0.05	-0.7212	-0.2707
BFU-E	HE+PSCs	0ug/ml	CFU-GEMM	E10.5AGM	6ug/ml	0.4083	0.1242	36	3.29	0.0023	0.05	0.1563	0.6603
BFU-E	HE+PSCs	0ug/ml	CFU-GEMM	HE+PSCs	0ug/ml	0.08700	0.08521	36	1.02	0.3140	0.05	-0.08580	0.2598
BFU-E	HE+PSCs	0ug/ml	CFU-GEMM	HE+PSCs	2ug/ml	1.0862	0.1433	36	7.58	<.0001	0.05	0.7956	1.3768
BFU-E	HE+PSCs	0ug/ml	CFU-GEMM	HE+PSCs	6ug/ml	1.3483	0.1535	36	8.78	<.0001	0.05	1.0370	1.6597
BFU-E	HE+PSCs	0ug/ml	CFU-GM	AoE+PSCs	0ug/ml	-1.6344	0.1044	36	-15.66	<.0001	0.05	-1.8461	-1.4227
BFU-E	HE+PSCs	0ug/ml	CFU-GM	AoE+PSCs	2ug/ml	-0.8620	0.1081	36	-7.97	<.0001	0.05	-1.0813	-0.6427
BFU-E	HE+PSCs	0ug/ml	CFU-GM	AoE+PSCs	6ug/ml	-0.3153	0.1130	36	-2.79	0.0084	0.05	-0.5444	-0.08616
BFU-E	HE+PSCs	0ug/ml	CFU-GM	E10.5AGM	0ug/ml	-2.0793	0.1032	36	-20.14	<.0001	0.05	-2.2886	-1.8700
BFU-E	HE+PSCs	0ug/ml	CFU-GM	E10.5AGM	2ug/ml	-1.4660	0.1050	36	-13.96	<.0001	0.05	-1.6790	-1.2531
BFU-E	HE+PSCs	0ug/ml	CFU-GM	E10.5AGM	6ug/ml	-0.7814	0.1087	36	-7.19	<.0001	0.05	-1.0018	-0.5609
BFU-E	HE+PSCs	0ug/ml	CFU-GM	HE+PSCs	0ug/ml	-1.5456	0.06490	36	-23.81	<.0001	0.05	-1.6773	-1.4140
BFU-E	HE+PSCs	0ug/ml	CFU-GM	HE+PSCs	2ug/ml	-0.6702	0.1096	36	-6.12	<.0001	0.05	-0.8923	-0.4480
BFU-E	HE+PSCs	0ug/ml	CFU-GM	HE+PSCs	6ug/ml	0.08997	0.1184	36	0.76	0.4524	0.05	-0.1502	0.3302
BFU-E	HE+PSCs	2ug/ml	BFU-E	HE+PSCs	6ug/ml	0.7975	0.1641	36	4.86	<.0001	0.05	0.4646	1.1303
BFU-E	HE+PSCs	2ug/ml	CFU-GEMM	AoE+PSCs	0ug/ml	-0.6567	0.1277	36	-5.14	<.0001	0.05	-0.9157	-0.3978
BFU-E	HE+PSCs	2ug/ml	CFU-GEMM	AoE+PSCs	2ug/ml	-0.06771	0.1374	36	-0.49	0.6253	0.05	-0.3465	0.2110
BFU-E	HE+PSCs	2ug/ml	CFU-GEMM	AoE+PSCs	6ug/ml	1.0311	0.1749	36	5.89	<.0001	0.05	0.6763	1.3858
BFU-E	HE+PSCs	2ug/ml	CFU-GEMM	E10.5AGM	0ug/ml	-1.2130	0.1221	36	-9.94	<.0001	0.05	-1.4606	-0.9654
BFU-E	HE+PSCs	2ug/ml	CFU-GEMM	E10.5AGM	2ug/ml	-1.0877	0.1231	36	-8.84	<.0001	0.05	-1.3374	-0.8381
BFU-E	HE+PSCs	2ug/ml	CFU-GEMM	E10.5AGM	6ug/ml	-0.1835	0.1351	36	-1.36	0.1828	0.05	-0.4575	0.09049

The GLIMMIX Procedure

Differences of Colony*Condit*Treatm Least Squares Means													
Colony_Type	Condition	Treatment	_Colony_Type	_Condition	_Treatment	Estimate	Standard Error	DF	t Value	Pr > |t|	Alpha	Lower	Upper
BFU-E	HE+PSCs	2ug/ml	CFU-GEMM	HE+PSCs	0ug/ml	-0.5048	0.1297	36	-3.89	0.0004	0.05	-0.7679	-0.2417
BFU-E	HE+PSCs	2ug/ml	CFU-GEMM	HE+PSCs	2ug/ml	0.4944	0.1288	36	3.84	0.0005	0.05	0.2331	0.7557
BFU-E	HE+PSCs	2ug/ml	CFU-GEMM	HE+PSCs	6ug/ml	0.7565	0.1624	36	4.66	<.0001	0.05	0.4271	1.0859
BFU-E	HE+PSCs	2ug/ml	CFU-GM	AoE+PSCs	0ug/ml	-2.2262	0.1171	36	-19.01	<.0001	0.05	-2.4637	-1.9887
BFU-E	HE+PSCs	2ug/ml	CFU-GM	AoE+PSCs	2ug/ml	-1.4538	0.1204	36	-12.07	<.0001	0.05	-1.6980	-1.2095
BFU-E	HE+PSCs	2ug/ml	CFU-GM	AoE+PSCs	6ug/ml	-0.9071	0.1248	36	-7.27	<.0001	0.05	-1.1602	-0.6540
BFU-E	HE+PSCs	2ug/ml	CFU-GM	E10.5AGM	0ug/ml	-2.6711	0.1161	36	-23.02	<.0001	0.05	-2.9065	-2.4357
BFU-E	HE+PSCs	2ug/ml	CFU-GM	E10.5AGM	2ug/ml	-2.0578	0.1176	36	-17.49	<.0001	0.05	-2.2964	-1.8193
BFU-E	HE+PSCs	2ug/ml	CFU-GM	E10.5AGM	6ug/ml	-1.3732	0.1210	36	-11.35	<.0001	0.05	-1.6185	-1.1279
BFU-E	HE+PSCs	2ug/ml	CFU-GM	HE+PSCs	0ug/ml	-2.1374	0.1174	36	-18.21	<.0001	0.05	-2.3755	-1.8994
BFU-E	HE+PSCs	2ug/ml	CFU-GM	HE+PSCs	2ug/ml	-1.2620	0.08982	36	-14.05	<.0001	0.05	-1.4441	-1.0798
BFU-E	HE+PSCs	2ug/ml	CFU-GM	HE+PSCs	6ug/ml	-0.5018	0.1298	36	-3.87	0.0004	0.05	-0.7651	-0.2386
BFU-E	HE+PSCs	6ug/ml	CFU-GEMM	AoE+PSCs	0ug/ml	-1.4542	0.1547	36	-9.40	<.0001	0.05	-1.7678	-1.1405
BFU-E	HE+PSCs	6ug/ml	CFU-GEMM	AoE+PSCs	2ug/ml	-0.8652	0.1628	36	-5.31	<.0001	0.05	-1.1954	-0.5350
BFU-E	HE+PSCs	6ug/ml	CFU-GEMM	AoE+PSCs	6ug/ml	0.2336	0.1955	36	1.20	0.2398	0.05	-0.1628	0.6301
BFU-E	HE+PSCs	6ug/ml	CFU-GEMM	E10.5AGM	0ug/ml	-2.0105	0.1501	36	-13.40	<.0001	0.05	-2.3149	-1.7061
BFU-E	HE+PSCs	6ug/ml	CFU-GEMM	E10.5AGM	2ug/ml	-1.8852	0.1509	36	-12.49	<.0001	0.05	-2.1913	-1.5792
BFU-E	HE+PSCs	6ug/ml	CFU-GEMM	E10.5AGM	6ug/ml	-0.9810	0.1608	36	-6.10	<.0001	0.05	-1.3071	-0.6548
BFU-E	HE+PSCs	6ug/ml	CFU-GEMM	HE+PSCs	0ug/ml	-1.3023	0.1563	36	-8.33	<.0001	0.05	-1.6193	-0.9852
BFU-E	HE+PSCs	6ug/ml	CFU-GEMM	HE+PSCs	2ug/ml	-0.3031	0.1760	36	-1.72	0.0936	0.05	-0.6599	0.05380
BFU-E	HE+PSCs	6ug/ml	CFU-GEMM	HE+PSCs	6ug/ml	-0.04094	0.1649	36	-0.25	0.8054	0.05	-0.3755	0.2936
BFU-E	HE+PSCs	6ug/ml	CFU-GM	AoE+PSCs	0ug/ml	-3.0237	0.1461	36	-20.70	<.0001	0.05	-3.3199	-2.7274
BFU-E	HE+PSCs	6ug/ml	CFU-GM	AoE+PSCs	2ug/ml	-2.2512	0.1487	36	-15.14	<.0001	0.05	-2.5529	-1.9496
BFU-E	HE+PSCs	6ug/ml	CFU-GM	AoE+PSCs	6ug/ml	-1.7045	0.1523	36	-11.19	<.0001	0.05	-2.0134	-1.3957

The GLIMMIX Procedure

Differences of Colony*Condit*Treatm Least Squares Means													
Colony_Type	Condition	Treatment	_Colony_Type	_Condition	_Treatment	Estimate	Standard Error	DF	t Value	Pr > |t|	Alpha	Lower	Upper
BFU-E	HE+PSCs	6ug/ml	CFU-GM	E10.5AGM	0ug/ml	-3.4686	0.1452	36	-23.89	<.0001	0.05	-3.7631	-3.1740
BFU-E	HE+PSCs	6ug/ml	CFU-GM	E10.5AGM	2ug/ml	-2.8553	0.1465	36	-19.49	<.0001	0.05	-3.1524	-2.5582
BFU-E	HE+PSCs	6ug/ml	CFU-GM	E10.5AGM	6ug/ml	-2.1706	0.1491	36	-14.55	<.0001	0.05	-2.4731	-1.8681
BFU-E	HE+PSCs	6ug/ml	CFU-GM	HE+PSCs	0ug/ml	-2.9349	0.1463	36	-20.07	<.0001	0.05	-3.2315	-2.6383
BFU-E	HE+PSCs	6ug/ml	CFU-GM	HE+PSCs	2ug/ml	-2.0594	0.1498	36	-13.75	<.0001	0.05	-2.3632	-1.7557
BFU-E	HE+PSCs	6ug/ml	CFU-GM	HE+PSCs	6ug/ml	-1.2993	0.1329	36	-9.77	<.0001	0.05	-1.5689	-1.0297
CFU-GEMM	AoE+PSCs	0ug/ml	CFU-GEMM	AoE+PSCs	2ug/ml	0.5890	0.1260	36	4.68	<.0001	0.05	0.3335	0.8445
CFU-GEMM	AoE+PSCs	0ug/ml	CFU-GEMM	AoE+PSCs	6ug/ml	1.6878	0.1661	36	10.16	<.0001	0.05	1.3510	2.0246
CFU-GEMM	AoE+PSCs	0ug/ml	CFU-GEMM	E10.5AGM	0ug/ml	-0.5563	0.1090	36	-5.10	<.0001	0.05	-0.7774	-0.3352
CFU-GEMM	AoE+PSCs	0ug/ml	CFU-GEMM	E10.5AGM	2ug/ml	-0.4310	0.1101	36	-3.91	0.0004	0.05	-0.6544	-0.2077
CFU-GEMM	AoE+PSCs	0ug/ml	CFU-GEMM	E10.5AGM	6ug/ml	0.4732	0.1234	36	3.83	0.0005	0.05	0.2229	0.7235
CFU-GEMM	AoE+PSCs	0ug/ml	CFU-GEMM	HE+PSCs	0ug/ml	0.1519	0.1175	36	1.29	0.2042	0.05	-0.08635	0.3902
CFU-GEMM	AoE+PSCs	0ug/ml	CFU-GEMM	HE+PSCs	2ug/ml	1.1511	0.1426	36	8.07	<.0001	0.05	0.8620	1.4402
CFU-GEMM	AoE+PSCs	0ug/ml	CFU-GEMM	HE+PSCs	6ug/ml	1.4132	0.1529	36	9.25	<.0001	0.05	1.1032	1.7232
CFU-GEMM	AoE+PSCs	0ug/ml	CFU-GM	AoE+PSCs	0ug/ml	-1.5695	0.06283	36	-24.98	<.0001	0.05	-1.6969	-1.4421
CFU-GEMM	AoE+PSCs	0ug/ml	CFU-GM	AoE+PSCs	2ug/ml	-0.7970	0.1072	36	-7.44	<.0001	0.05	-1.0144	-0.5797
CFU-GEMM	AoE+PSCs	0ug/ml	CFU-GM	AoE+PSCs	6ug/ml	-0.2504	0.1121	36	-2.23	0.0318	0.05	-0.4776	-0.02311
CFU-GEMM	AoE+PSCs	0ug/ml	CFU-GM	E10.5AGM	0ug/ml	-2.0144	0.1022	36	-19.71	<.0001	0.05	-2.2217	-1.8071
CFU-GEMM	AoE+PSCs	0ug/ml	CFU-GM	E10.5AGM	2ug/ml	-1.4011	0.1040	36	-13.47	<.0001	0.05	-1.6120	-1.1902
CFU-GEMM	AoE+PSCs	0ug/ml	CFU-GM	E10.5AGM	6ug/ml	-0.7164	0.1077	36	-6.65	<.0001	0.05	-0.9350	-0.4979
CFU-GEMM	AoE+PSCs	0ug/ml	CFU-GM	HE+PSCs	0ug/ml	-1.4807	0.1037	36	-14.28	<.0001	0.05	-1.6910	-1.2704
CFU-GEMM	AoE+PSCs	0ug/ml	CFU-GM	HE+PSCs	2ug/ml	-0.6053	0.1086	36	-5.57	<.0001	0.05	-0.8255	-0.3850
CFU-GEMM	AoE+PSCs	0ug/ml	CFU-GM	HE+PSCs	6ug/ml	0.1549	0.1176	36	1.32	0.1961	0.05	-0.08358	0.3933
CFU-GEMM	AoE+PSCs	2ug/ml	CFU-GEMM	AoE+PSCs	6ug/ml	1.0988	0.1737	36	6.33	<.0001	0.05	0.7466	1.4510

The GLIMMIX Procedure

Differences of Colony*Condit*Treatm Least Squares Means													
Colony_Type	Condition	Treatment	_Colony_Type	_Condition	_Treatment	Estimate	Standard Error	DF	t Value	Pr > |t|	Alpha	Lower	Upper
CFU-GEMM	AoE+PSCs	2ug/ml	CFU-GEMM	E10.5AGM	0ug/ml	-1.1453	0.1203	36	-9.52	<.0001	0.05	-1.3893	-0.9013
CFU-GEMM	AoE+PSCs	2ug/ml	CFU-GEMM	E10.5AGM	2ug/ml	-1.0200	0.1213	36	-8.41	<.0001	0.05	-1.2661	-0.7740
CFU-GEMM	AoE+PSCs	2ug/ml	CFU-GEMM	E10.5AGM	6ug/ml	-0.1158	0.1335	36	-0.87	0.3914	0.05	-0.3865	0.1549
CFU-GEMM	AoE+PSCs	2ug/ml	CFU-GEMM	HE+PSCs	0ug/ml	-0.4371	0.1280	36	-3.41	0.0016	0.05	-0.6968	-0.1774
CFU-GEMM	AoE+PSCs	2ug/ml	CFU-GEMM	HE+PSCs	2ug/ml	0.5621	0.1514	36	3.71	0.0007	0.05	0.2551	0.8691
CFU-GEMM	AoE+PSCs	2ug/ml	CFU-GEMM	HE+PSCs	6ug/ml	0.8242	0.1611	36	5.12	<.0001	0.05	0.4975	1.1509
CFU-GEMM	AoE+PSCs	2ug/ml	CFU-GM	AoE+PSCs	0ug/ml	-2.1585	0.1152	36	-18.73	<.0001	0.05	-2.3922	-1.9248
CFU-GEMM	AoE+PSCs	2ug/ml	CFU-GM	AoE+PSCs	2ug/ml	-1.3861	0.08549	36	-16.21	<.0001	0.05	-1.5594	-1.2127
CFU-GEMM	AoE+PSCs	2ug/ml	CFU-GM	AoE+PSCs	6ug/ml	-0.8394	0.1231	36	-6.82	<.0001	0.05	-1.0889	-0.5898
CFU-GEMM	AoE+PSCs	2ug/ml	CFU-GM	E10.5AGM	0ug/ml	-2.6034	0.1142	36	-22.80	<.0001	0.05	-2.8350	-2.3718
CFU-GEMM	AoE+PSCs	2ug/ml	CFU-GM	E10.5AGM	2ug/ml	-1.9901	0.1158	36	-17.19	<.0001	0.05	-2.2250	-1.7553
CFU-GEMM	AoE+PSCs	2ug/ml	CFU-GM	E10.5AGM	6ug/ml	-1.3055	0.1191	36	-10.96	<.0001	0.05	-1.5471	-1.0638
CFU-GEMM	AoE+PSCs	2ug/ml	CFU-GM	HE+PSCs	0ug/ml	-2.0697	0.1155	36	-17.92	<.0001	0.05	-2.3040	-1.8354
CFU-GEMM	AoE+PSCs	2ug/ml	CFU-GM	HE+PSCs	2ug/ml	-1.1943	0.1199	36	-9.96	<.0001	0.05	-1.4375	-0.9510
CFU-GEMM	AoE+PSCs	2ug/ml	CFU-GM	HE+PSCs	6ug/ml	-0.4341	0.1281	36	-3.39	0.0017	0.05	-0.6939	-0.1743
CFU-GEMM	AoE+PSCs	6ug/ml	CFU-GEMM	E10.5AGM	0ug/ml	-2.2441	0.1618	36	-13.87	<.0001	0.05	-2.5723	-1.9160
CFU-GEMM	AoE+PSCs	6ug/ml	CFU-GEMM	E10.5AGM	2ug/ml	-2.1188	0.1626	36	-13.03	<.0001	0.05	-2.4485	-1.7891
CFU-GEMM	AoE+PSCs	6ug/ml	CFU-GEMM	E10.5AGM	6ug/ml	-1.2146	0.1718	36	-7.07	<.0001	0.05	-1.5631	-0.8661
CFU-GEMM	AoE+PSCs	6ug/ml	CFU-GEMM	HE+PSCs	0ug/ml	-1.5359	0.1676	36	-9.16	<.0001	0.05	-1.8759	-1.1959
CFU-GEMM	AoE+PSCs	6ug/ml	CFU-GEMM	HE+PSCs	2ug/ml	-0.5367	0.1861	36	-2.88	0.0066	0.05	-0.9140	-0.1594
CFU-GEMM	AoE+PSCs	6ug/ml	CFU-GEMM	HE+PSCs	6ug/ml	-0.2746	0.1940	36	-1.42	0.1656	0.05	-0.6681	0.1190
CFU-GEMM	AoE+PSCs	6ug/ml	CFU-GM	AoE+PSCs	0ug/ml	-3.2573	0.1581	36	-20.61	<.0001	0.05	-3.5779	-2.9367
CFU-GEMM	AoE+PSCs	6ug/ml	CFU-GM	AoE+PSCs	2ug/ml	-2.4849	0.1606	36	-15.48	<.0001	0.05	-2.8105	-2.1592
CFU-GEMM	AoE+PSCs	6ug/ml	CFU-GM	AoE+PSCs	6ug/ml	-1.9382	0.1417	36	-13.68	<.0001	0.05	-2.2255	-1.6509

The GLIMMIX Procedure

Differences of Colony*Condit*Treatm Least Squares Means													
Colony_Type	Condition	Treatment	_Colony_Type	_Condition	_Treatment	Estimate	Standard Error	DF	t Value	Pr > |t|	Alpha	Lower	Upper
CFU-GEMM	AoE+PSCs	6ug/ml	CFU-GM	E10.5AGM	0ug/ml	-3.7022	0.1573	36	-23.54	<.0001	0.05	-4.0212	-3.3832
CFU-GEMM	AoE+PSCs	6ug/ml	CFU-GM	E10.5AGM	2ug/ml	-3.0889	0.1585	36	-19.49	<.0001	0.05	-3.4103	-2.7675
CFU-GEMM	AoE+PSCs	6ug/ml	CFU-GM	E10.5AGM	6ug/ml	-2.4043	0.1609	36	-14.94	<.0001	0.05	-2.7306	-2.0779
CFU-GEMM	AoE+PSCs	6ug/ml	CFU-GM	HE+PSCs	0ug/ml	-3.1685	0.1583	36	-20.02	<.0001	0.05	-3.4895	-2.8475
CFU-GEMM	AoE+PSCs	6ug/ml	CFU-GM	HE+PSCs	2ug/ml	-2.2931	0.1615	36	-14.20	<.0001	0.05	-2.6206	-1.9655
CFU-GEMM	AoE+PSCs	6ug/ml	CFU-GM	HE+PSCs	6ug/ml	-1.5329	0.1677	36	-9.14	<.0001	0.05	-1.8730	-1.1929
CFU-GEMM	E10.5AGM	0ug/ml	CFU-GEMM	E10.5AGM	2ug/ml	0.1253	0.1036	36	1.21	0.2345	0.05	-0.08487	0.3354
CFU-GEMM	E10.5AGM	0ug/ml	CFU-GEMM	E10.5AGM	6ug/ml	1.0295	0.1176	36	8.75	<.0001	0.05	0.7910	1.2681
CFU-GEMM	E10.5AGM	0ug/ml	CFU-GEMM	HE+PSCs	0ug/ml	0.7082	0.1114	36	6.36	<.0001	0.05	0.4823	0.9341
CFU-GEMM	E10.5AGM	0ug/ml	CFU-GEMM	HE+PSCs	2ug/ml	1.7074	0.1376	36	12.41	<.0001	0.05	1.4284	1.9864
CFU-GEMM	E10.5AGM	0ug/ml	CFU-GEMM	HE+PSCs	6ug/ml	1.9695	0.1482	36	13.29	<.0001	0.05	1.6689	2.2701
CFU-GEMM	E10.5AGM	0ug/ml	CFU-GM	AoE+PSCs	0ug/ml	-1.0132	0.09641	36	-10.51	<.0001	0.05	-1.2087	-0.8177
CFU-GEMM	E10.5AGM	0ug/ml	CFU-GM	AoE+PSCs	2ug/ml	-0.2407	0.1005	36	-2.40	0.0219	0.05	-0.4445	-0.03702
CFU-GEMM	E10.5AGM	0ug/ml	CFU-GM	AoE+PSCs	6ug/ml	0.3059	0.1056	36	2.90	0.0064	0.05	0.09167	0.5202
CFU-GEMM	E10.5AGM	0ug/ml	CFU-GM	E10.5AGM	0ug/ml	-1.4581	0.04805	36	-30.35	<.0001	0.05	-1.5555	-1.3606
CFU-GEMM	E10.5AGM	0ug/ml	CFU-GM	E10.5AGM	2ug/ml	-0.8448	0.09706	36	-8.70	<.0001	0.05	-1.0417	-0.6480
CFU-GEMM	E10.5AGM	0ug/ml	CFU-GM	E10.5AGM	6ug/ml	-0.1601	0.1011	36	-1.58	0.1218	0.05	-0.3651	0.04481
CFU-GEMM	E10.5AGM	0ug/ml	CFU-GM	HE+PSCs	0ug/ml	-0.9244	0.09674	36	-9.56	<.0001	0.05	-1.1206	-0.7282
CFU-GEMM	E10.5AGM	0ug/ml	CFU-GM	HE+PSCs	2ug/ml	-0.04896	0.1020	36	-0.48	0.6341	0.05	-0.2558	0.1579
CFU-GEMM	E10.5AGM	0ug/ml	CFU-GM	HE+PSCs	6ug/ml	0.7112	0.1115	36	6.38	<.0001	0.05	0.4851	0.9373
CFU-GEMM	E10.5AGM	2ug/ml	CFU-GEMM	E10.5AGM	6ug/ml	0.9042	0.1187	36	7.62	<.0001	0.05	0.6636	1.1449
CFU-GEMM	E10.5AGM	2ug/ml	CFU-GEMM	HE+PSCs	0ug/ml	0.5829	0.1125	36	5.18	<.0001	0.05	0.3548	0.8111
CFU-GEMM	E10.5AGM	2ug/ml	CFU-GEMM	HE+PSCs	2ug/ml	1.5821	0.1385	36	11.43	<.0001	0.05	1.3013	1.8630
CFU-GEMM	E10.5AGM	2ug/ml	CFU-GEMM	HE+PSCs	6ug/ml	1.8443	0.1490	36	12.37	<.0001	0.05	1.5420	2.1465

The GLIMMIX Procedure

Differences of Colony*Condit*Treatm Least Squares Means													
Colony_Type	Condition	Treatment	_Colony_Type	_Condition	_Treatment	Estimate	Standard Error	DF	t Value	Pr > |t|	Alpha	Lower	Upper
CFU-GEMM	E10.5AGM	2ug/ml	CFU-GM	AoE+PSCs	0ug/ml	-1.1385	0.09769	36	-11.65	<.0001	0.05	-1.3366	-0.9403
CFU-GEMM	E10.5AGM	2ug/ml	CFU-GM	AoE+PSCs	2ug/ml	-0.3660	0.1017	36	-3.60	0.0010	0.05	-0.5722	-0.1598
CFU-GEMM	E10.5AGM	2ug/ml	CFU-GM	AoE+PSCs	6ug/ml	0.1807	0.1068	36	1.69	0.0994	0.05	-0.03595	0.3973
CFU-GEMM	E10.5AGM	2ug/ml	CFU-GM	E10.5AGM	0ug/ml	-1.5834	0.09643	36	-16.42	<.0001	0.05	-1.7789	-1.3878
CFU-GEMM	E10.5AGM	2ug/ml	CFU-GM	E10.5AGM	2ug/ml	-0.9701	0.05406	36	-17.95	<.0001	0.05	-1.0797	-0.8605
CFU-GEMM	E10.5AGM	2ug/ml	CFU-GM	E10.5AGM	6ug/ml	-0.2854	0.1023	36	-2.79	0.0084	0.05	-0.4928	-0.07800
CFU-GEMM	E10.5AGM	2ug/ml	CFU-GM	HE+PSCs	0ug/ml	-1.0497	0.09801	36	-10.71	<.0001	0.05	-1.2485	-0.8509
CFU-GEMM	E10.5AGM	2ug/ml	CFU-GM	HE+PSCs	2ug/ml	-0.1742	0.1032	36	-1.69	0.1000	0.05	-0.3835	0.03503
CFU-GEMM	E10.5AGM	2ug/ml	CFU-GM	HE+PSCs	6ug/ml	0.5859	0.1126	36	5.20	<.0001	0.05	0.3576	0.8142
CFU-GEMM	E10.5AGM	6ug/ml	CFU-GEMM	HE+PSCs	0ug/ml	-0.3213	0.1255	36	-2.56	0.0148	0.05	-0.5759	-0.06675
CFU-GEMM	E10.5AGM	6ug/ml	CFU-GEMM	HE+PSCs	2ug/ml	0.6779	0.1492	36	4.54	<.0001	0.05	0.3752	0.9806
CFU-GEMM	E10.5AGM	6ug/ml	CFU-GEMM	HE+PSCs	6ug/ml	0.9400	0.1591	36	5.91	<.0001	0.05	0.6174	1.2627
CFU-GEMM	E10.5AGM	6ug/ml	CFU-GM	AoE+PSCs	0ug/ml	-2.0427	0.1124	36	-18.17	<.0001	0.05	-2.2707	-1.8147
CFU-GEMM	E10.5AGM	6ug/ml	CFU-GM	AoE+PSCs	2ug/ml	-1.2703	0.1159	36	-10.96	<.0001	0.05	-1.5053	-1.0352
CFU-GEMM	E10.5AGM	6ug/ml	CFU-GM	AoE+PSCs	6ug/ml	-0.7236	0.1204	36	-6.01	<.0001	0.05	-0.9678	-0.4793
CFU-GEMM	E10.5AGM	6ug/ml	CFU-GM	E10.5AGM	0ug/ml	-2.4876	0.1114	36	-22.34	<.0001	0.05	-2.7134	-2.2618
CFU-GEMM	E10.5AGM	6ug/ml	CFU-GM	E10.5AGM	2ug/ml	-1.8743	0.1130	36	-16.59	<.0001	0.05	-2.1035	-1.6452
CFU-GEMM	E10.5AGM	6ug/ml	CFU-GM	E10.5AGM	6ug/ml	-1.1897	0.08242	36	-14.43	<.0001	0.05	-1.3568	-1.0225
CFU-GEMM	E10.5AGM	6ug/ml	CFU-GM	HE+PSCs	0ug/ml	-1.9539	0.1127	36	-17.33	<.0001	0.05	-2.1825	-1.7253
CFU-GEMM	E10.5AGM	6ug/ml	CFU-GM	HE+PSCs	2ug/ml	-1.0785	0.1172	36	-9.20	<.0001	0.05	-1.3162	-0.8407
CFU-GEMM	E10.5AGM	6ug/ml	CFU-GM	HE+PSCs	6ug/ml	-0.3183	0.1256	36	-2.53	0.0157	0.05	-0.5730	-0.06364
CFU-GEMM	HE+PSCs	0ug/ml	CFU-GEMM	HE+PSCs	2ug/ml	0.9992	0.1444	36	6.92	<.0001	0.05	0.7064	1.2920
CFU-GEMM	HE+PSCs	0ug/ml	CFU-GEMM	HE+PSCs	6ug/ml	1.2613	0.1545	36	8.16	<.0001	0.05	0.9479	1.5748
CFU-GEMM	HE+PSCs	0ug/ml	CFU-GM	AoE+PSCs	0ug/ml	-1.7214	0.1059	36	-16.26	<.0001	0.05	-1.9362	-1.5066

The GLIMMIX Procedure

Differences of Colony*Condit*Treatm Least Squares Means													
Colony_Type	Condition	Treatment	_Colony_Type	_Condition	_Treatment	Estimate	Standard Error	DF	t Value	Pr > |t|	Alpha	Lower	Upper
CFU-GEMM	HE+PSCs	0ug/ml	CFU-GM	AoE+PSCs	2ug/ml	-0.9490	0.1096	36	-8.66	<.0001	0.05	-1.1712	-0.7267
CFU-GEMM	HE+PSCs	0ug/ml	CFU-GM	AoE+PSCs	6ug/ml	-0.4023	0.1144	36	-3.52	0.0012	0.05	-0.6342	-0.1703
CFU-GEMM	HE+PSCs	0ug/ml	CFU-GM	E10.5AGM	0ug/ml	-2.1663	0.1047	36	-20.68	<.0001	0.05	-2.3787	-1.9539
CFU-GEMM	HE+PSCs	0ug/ml	CFU-GM	E10.5AGM	2ug/ml	-1.5530	0.1065	36	-14.58	<.0001	0.05	-1.7690	-1.3371
CFU-GEMM	HE+PSCs	0ug/ml	CFU-GM	E10.5AGM	6ug/ml	-0.8684	0.1101	36	-7.88	<.0001	0.05	-1.0917	-0.6450
CFU-GEMM	HE+PSCs	0ug/ml	CFU-GM	HE+PSCs	0ug/ml	-1.6326	0.06729	36	-24.26	<.0001	0.05	-1.7691	-1.4962
CFU-GEMM	HE+PSCs	0ug/ml	CFU-GM	HE+PSCs	2ug/ml	-0.7572	0.1110	36	-6.82	<.0001	0.05	-0.9823	-0.5321
CFU-GEMM	HE+PSCs	0ug/ml	CFU-GM	HE+PSCs	6ug/ml	0.002970	0.1198	36	0.02	0.9804	0.05	-0.2399	0.2459
CFU-GEMM	HE+PSCs	2ug/ml	CFU-GEMM	HE+PSCs	6ug/ml	0.2621	0.1744	36	1.50	0.1415	0.05	-0.09152	0.6158
CFU-GEMM	HE+PSCs	2ug/ml	CFU-GM	AoE+PSCs	0ug/ml	-2.7206	0.1332	36	-20.43	<.0001	0.05	-2.9907	-2.4505
CFU-GEMM	HE+PSCs	2ug/ml	CFU-GM	AoE+PSCs	2ug/ml	-1.9482	0.1361	36	-14.31	<.0001	0.05	-2.2242	-1.6721
CFU-GEMM	HE+PSCs	2ug/ml	CFU-GM	AoE+PSCs	6ug/ml	-1.4015	0.1400	36	-10.01	<.0001	0.05	-1.6854	-1.1176
CFU-GEMM	HE+PSCs	2ug/ml	CFU-GM	E10.5AGM	0ug/ml	-3.1655	0.1322	36	-23.94	<.0001	0.05	-3.4337	-2.8973
CFU-GEMM	HE+PSCs	2ug/ml	CFU-GM	E10.5AGM	2ug/ml	-2.5522	0.1336	36	-19.10	<.0001	0.05	-2.8233	-2.2812
CFU-GEMM	HE+PSCs	2ug/ml	CFU-GM	E10.5AGM	6ug/ml	-1.8676	0.1366	36	-13.68	<.0001	0.05	-2.1445	-1.5906
CFU-GEMM	HE+PSCs	2ug/ml	CFU-GM	HE+PSCs	0ug/ml	-2.6318	0.1334	36	-19.73	<.0001	0.05	-2.9024	-2.3613
CFU-GEMM	HE+PSCs	2ug/ml	CFU-GM	HE+PSCs	2ug/ml	-1.7564	0.1099	36	-15.98	<.0001	0.05	-1.9793	-1.5334
CFU-GEMM	HE+PSCs	2ug/ml	CFU-GM	HE+PSCs	6ug/ml	-0.9962	0.1444	36	-6.90	<.0001	0.05	-1.2892	-0.7033
CFU-GEMM	HE+PSCs	6ug/ml	CFU-GM	AoE+PSCs	0ug/ml	-2.9827	0.1441	36	-20.69	<.0001	0.05	-3.2751	-2.6904
CFU-GEMM	HE+PSCs	6ug/ml	CFU-GM	AoE+PSCs	2ug/ml	-2.2103	0.1468	36	-15.05	<.0001	0.05	-2.5081	-1.9125
CFU-GEMM	HE+PSCs	6ug/ml	CFU-GM	AoE+PSCs	6ug/ml	-1.6636	0.1504	36	-11.06	<.0001	0.05	-1.9687	-1.3585
CFU-GEMM	HE+PSCs	6ug/ml	CFU-GM	E10.5AGM	0ug/ml	-3.4276	0.1433	36	-23.92	<.0001	0.05	-3.7182	-3.1370
CFU-GEMM	HE+PSCs	6ug/ml	CFU-GM	E10.5AGM	2ug/ml	-2.8144	0.1446	36	-19.47	<.0001	0.05	-3.1075	-2.5212
CFU-GEMM	HE+PSCs	6ug/ml	CFU-GM	E10.5AGM	6ug/ml	-2.1297	0.1473	36	-14.46	<.0001	0.05	-2.4283	-1.8310

The GLIMMIX Procedure

Differences of Colony*Condit*Treatm Least Squares Means													
Colony_Type	Condition	Treatment	_Colony_Type	_Condition	_Treatment	Estimate	Standard Error	DF	t Value	Pr > |t|	Alpha	Lower	Upper
CFU-GEMM	HE+PSCs	6ug/ml	CFU-GM	HE+PSCs	0ug/ml	-2.8940	0.1444	36	-20.05	<.0001	0.05	-3.1867	-2.6012
CFU-GEMM	HE+PSCs	6ug/ml	CFU-GM	HE+PSCs	2ug/ml	-2.0185	0.1479	36	-13.65	<.0001	0.05	-2.3185	-1.7185
CFU-GEMM	HE+PSCs	6ug/ml	CFU-GM	HE+PSCs	6ug/ml	-1.2584	0.1308	36	-9.62	<.0001	0.05	-1.5236	-0.9931
CFU-GM	AoE+PSCs	0ug/ml	CFU-GM	AoE+PSCs	2ug/ml	0.7724	0.09433	36	8.19	<.0001	0.05	0.5811	0.9637
CFU-GM	AoE+PSCs	0ug/ml	CFU-GM	AoE+PSCs	6ug/ml	1.3191	0.09984	36	13.21	<.0001	0.05	1.1166	1.5216
CFU-GM	AoE+PSCs	0ug/ml	CFU-GM	E10.5AGM	0ug/ml	-0.4449	0.08865	36	-5.02	<.0001	0.05	-0.6247	-0.2651
CFU-GM	AoE+PSCs	0ug/ml	CFU-GM	E10.5AGM	2ug/ml	0.1684	0.09071	36	1.86	0.0716	0.05	-0.01559	0.3523
CFU-GM	AoE+PSCs	0ug/ml	CFU-GM	E10.5AGM	6ug/ml	0.8530	0.09497	36	8.98	<.0001	0.05	0.6604	1.0457
CFU-GM	AoE+PSCs	0ug/ml	CFU-GM	HE+PSCs	0ug/ml	0.08877	0.09037	36	0.98	0.3325	0.05	-0.09451	0.2720
CFU-GM	AoE+PSCs	0ug/ml	CFU-GM	HE+PSCs	2ug/ml	0.9642	0.09595	36	10.05	<.0001	0.05	0.7696	1.1588
CFU-GM	AoE+PSCs	0ug/ml	CFU-GM	HE+PSCs	6ug/ml	1.7244	0.1060	36	16.27	<.0001	0.05	1.5094	1.9394
CFU-GM	AoE+PSCs	2ug/ml	CFU-GM	AoE+PSCs	6ug/ml	0.5467	0.1037	36	5.27	<.0001	0.05	0.3363	0.7570
CFU-GM	AoE+PSCs	2ug/ml	CFU-GM	E10.5AGM	0ug/ml	-1.2173	0.09302	36	-13.09	<.0001	0.05	-1.4060	-1.0287
CFU-GM	AoE+PSCs	2ug/ml	CFU-GM	E10.5AGM	2ug/ml	-0.6041	0.09498	36	-6.36	<.0001	0.05	-0.7967	-0.4114
CFU-GM	AoE+PSCs	2ug/ml	CFU-GM	E10.5AGM	6ug/ml	0.08060	0.09905	36	0.81	0.4212	0.05	-0.1203	0.2815
CFU-GM	AoE+PSCs	2ug/ml	CFU-GM	HE+PSCs	0ug/ml	-0.6837	0.09466	36	-7.22	<.0001	0.05	-0.8757	-0.4917
CFU-GM	AoE+PSCs	2ug/ml	CFU-GM	HE+PSCs	2ug/ml	0.1918	0.10000	36	1.92	0.0631	0.05	-0.01102	0.3946
CFU-GM	AoE+PSCs	2ug/ml	CFU-GM	HE+PSCs	6ug/ml	0.9519	0.1097	36	8.68	<.0001	0.05	0.7295	1.1743
CFU-GM	AoE+PSCs	6ug/ml	CFU-GM	E10.5AGM	0ug/ml	-1.7640	0.09861	36	-17.89	<.0001	0.05	-1.9640	-1.5640
CFU-GM	AoE+PSCs	6ug/ml	CFU-GM	E10.5AGM	2ug/ml	-1.1508	0.1005	36	-11.45	<.0001	0.05	-1.3545	-0.9470
CFU-GM	AoE+PSCs	6ug/ml	CFU-GM	E10.5AGM	6ug/ml	-0.4661	0.1043	36	-4.47	<.0001	0.05	-0.6776	-0.2545
CFU-GM	AoE+PSCs	6ug/ml	CFU-GM	HE+PSCs	0ug/ml	-1.2304	0.1002	36	-12.28	<.0001	0.05	-1.4335	-1.0272
CFU-GM	AoE+PSCs	6ug/ml	CFU-GM	HE+PSCs	2ug/ml	-0.3549	0.1052	36	-3.37	0.0018	0.05	-0.5683	-0.1415
CFU-GM	AoE+PSCs	6ug/ml	CFU-GM	HE+PSCs	6ug/ml	0.4052	0.1144	36	3.54	0.0011	0.05	0.1732	0.6373

The GLIMMIX Procedure

Differences of Colony*Condit*Treatm Least Squares Means													
Colony_Type	Condition	Treatment	_Colony_Type	_Condition	_Treatment	Estimate	Standard Error	DF	t Value	Pr > |t|	Alpha	Lower	Upper
CFU-GM	E10.5AGM	0ug/ml	CFU-GM	E10.5AGM	2ug/ml	0.6133	0.08935	36	6.86	<.0001	0.05	0.4321	0.7945
CFU-GM	E10.5AGM	0ug/ml	CFU-GM	E10.5AGM	6ug/ml	1.2979	0.09368	36	13.86	<.0001	0.05	1.1079	1.4879
CFU-GM	E10.5AGM	0ug/ml	CFU-GM	HE+PSCs	0ug/ml	0.5337	0.08901	36	6.00	<.0001	0.05	0.3531	0.7142
CFU-GM	E10.5AGM	0ug/ml	CFU-GM	HE+PSCs	2ug/ml	1.4091	0.09467	36	14.89	<.0001	0.05	1.2171	1.6011
CFU-GM	E10.5AGM	0ug/ml	CFU-GM	HE+PSCs	6ug/ml	2.1693	0.1048	36	20.69	<.0001	0.05	1.9566	2.3819
CFU-GM	E10.5AGM	2ug/ml	CFU-GM	E10.5AGM	6ug/ml	0.6847	0.09562	36	7.16	<.0001	0.05	0.4907	0.8786
CFU-GM	E10.5AGM	2ug/ml	CFU-GM	HE+PSCs	0ug/ml	-0.07960	0.09105	36	-0.87	0.3878	0.05	-0.2643	0.1051
CFU-GM	E10.5AGM	2ug/ml	CFU-GM	HE+PSCs	2ug/ml	0.7959	0.09659	36	8.24	<.0001	0.05	0.6000	0.9918
CFU-GM	E10.5AGM	2ug/ml	CFU-GM	HE+PSCs	6ug/ml	1.5560	0.1066	36	14.60	<.0001	0.05	1.3398	1.7722
CFU-GM	E10.5AGM	6ug/ml	CFU-GM	HE+PSCs	0ug/ml	-0.7643	0.09530	36	-8.02	<.0001	0.05	-0.9576	-0.5710
CFU-GM	E10.5AGM	6ug/ml	CFU-GM	HE+PSCs	2ug/ml	0.1112	0.1006	36	1.11	0.2764	0.05	-0.09285	0.3152
CFU-GM	E10.5AGM	6ug/ml	CFU-GM	HE+PSCs	6ug/ml	0.8713	0.1102	36	7.91	<.0001	0.05	0.6478	1.0949
CFU-GM	HE+PSCs	0ug/ml	CFU-GM	HE+PSCs	2ug/ml	0.8755	0.09628	36	9.09	<.0001	0.05	0.6802	1.0707
CFU-GM	HE+PSCs	0ug/ml	CFU-GM	HE+PSCs	6ug/ml	1.6356	0.1063	36	15.39	<.0001	0.05	1.4200	1.8512
CFU-GM	HE+PSCs	2ug/ml	CFU-GM	HE+PSCs	6ug/ml	0.7601	0.1111	36	6.84	<.0001	0.05	0.5349	0.9854

The GENMOD Procedure

Model Information	
Data Set	WORK.EXT_FIG10BI_E10DRAXIN_TRANS
Distribution	Normal
Link Function	Identity
Dependent Variable	Percentage

Number of Observations Read	45
Number of Observations Used	45

Class Level Information		
Class	Levels	Values
Condition	3	AoE+PSCs E10.5AGM HE+PSCs
Treatment	3	0ug/ml 2ug/ml 6ug/ml

Parameter Information			
Parameter	Effect	Condition	Treatment
Prm1	Intercept		
Prm2	Condition	AoE+PSCs	
Prm3	Condition	E10.5AGM	
Prm4	Condition	HE+PSCs	
Prm5	Treatment		0ug/ml
Prm6	Treatment		2ug/ml
Prm7	Treatment		6ug/ml
Prm8	Condition*Treatment	AoE+PSCs	0ug/ml
Prm9	Condition*Treatment	AoE+PSCs	2ug/ml
Prm10	Condition*Treatment	AoE+PSCs	6ug/ml
Prm11	Condition*Treatment	E10.5AGM	0ug/ml

The GENMOD Procedure

Parameter Information			
**Parameter**	**Effect**	**Condition**	**Treatment**
**Prm12**	Condition*Treatment	E10.5AGM	2ug/ml
**Prm13**	Condition*Treatment	E10.5AGM	6ug/ml
**Prm14**	Condition*Treatment	HE+PSCs	0ug/ml
**Prm15**	Condition*Treatment	HE+PSCs	2ug/ml
**Prm16**	Condition*Treatment	HE+PSCs	6ug/ml

Criteria For Assessing Goodness Of Fit			
**Criterion**	**DF**	**Value**	**Value/DF**
**Deviance**	36	535.2000	14.8667
**Scaled Deviance**	36	45.0000	1.2500
**Pearson Chi-Square**	36	535.2000	14.8667
**Scaled Pearson X2**	36	45.0000	1.2500
**Log Likelihood**		-119.5617	
**Full Log Likelihood**		-119.5617	
**AIC (smaller is better)**		259.1235	
**AICC (smaller is better)**		265.5941	
**BIC (smaller is better)**		277.1901	

Algorithm converged.

The GENMOD Procedure

Analysis Of Maximum Likelihood Parameter Estimates									
Parameter			DF	Estimate	Standard Error	Wald 95% Confidence Limits		Wald Chi-Square	Pr > ChiSq
Intercept			1	4.8000	1.5423	1.7772	7.8228	9.69	0.0019
Condition	AoE+PSCs		1	3.0000	2.1811	-1.2749	7.2749	1.89	0.1690
Condition	E10.5AGM		1	11.8000	2.1811	7.5251	16.0749	29.27	<.0001
Condition	HE+PSCs		0	0.0000	0.0000	0.0000	0.0000	.	.
Treatment	0ug/ml		1	21.4000	2.1811	17.1251	25.6749	96.26	<.0001
Treatment	2ug/ml		1	10.8000	2.1811	6.5251	15.0749	24.52	<.0001
Treatment	6ug/ml		0	0.0000	0.0000	0.0000	0.0000	.	.
Condition*Treatment	AoE+PSCs	0ug/ml	1	2.6000	3.0846	-3.4457	8.6457	0.71	0.3993
Condition*Treatment	AoE+PSCs	2ug/ml	1	0.4000	3.0846	-5.6457	6.4457	0.02	0.8968
Condition*Treatment	AoE+PSCs	6ug/ml	0	0.0000	0.0000	0.0000	0.0000	.	.
Condition*Treatment	E10.5AGM	0ug/ml	1	18.8000	3.0846	12.7543	24.8457	37.15	<.0001
Condition*Treatment	E10.5AGM	2ug/ml	1	12.4000	3.0846	6.3543	18.4457	16.16	<.0001
Condition*Treatment	E10.5AGM	6ug/ml	0	0.0000	0.0000	0.0000	0.0000	.	.
Condition*Treatment	HE+PSCs	0ug/ml	0	0.0000	0.0000	0.0000	0.0000	.	.
Condition*Treatment	HE+PSCs	2ug/ml	0	0.0000	0.0000	0.0000	0.0000	.	.
Condition*Treatment	HE+PSCs	6ug/ml	0	0.0000	0.0000	0.0000	0.0000	.	.
Scale			1	3.4487	0.3635	2.8050	4.2401		

Note: The scale parameter was estimated by maximum likelihood.

The GENMOD Procedure

Condition*Treatment Least Squares Means								
Condition	Treatment	Estimate	Standard Error	z Value	Pr > |z|	Alpha	Lower	Upper
AoE+PSCs	0ug/ml	31.8000	1.5423	20.62	<.0001	0.05	28.7772	34.8228
AoE+PSCs	2ug/ml	19.0000	1.5423	12.32	<.0001	0.05	15.9772	22.0228
AoE+PSCs	6ug/ml	7.8000	1.5423	5.06	<.0001	0.05	4.7772	10.8228
E10.5AGM	0ug/ml	56.8000	1.5423	36.83	<.0001	0.05	53.7772	59.8228
E10.5AGM	2ug/ml	39.8000	1.5423	25.81	<.0001	0.05	36.7772	42.8228
E10.5AGM	6ug/ml	16.6000	1.5423	10.76	<.0001	0.05	13.5772	19.6228
HE+PSCs	0ug/ml	26.2000	1.5423	16.99	<.0001	0.05	23.1772	29.2228
HE+PSCs	2ug/ml	15.6000	1.5423	10.11	<.0001	0.05	12.5772	18.6228
HE+PSCs	6ug/ml	4.8000	1.5423	3.11	0.0019	0.05	1.7772	7.8228

Differences of Condition*Treatment Least Squares Means										
Condition	Treatment	_Condition	_Treatment	Estimate	Standard Error	z Value	Pr > |z|	Alpha	Lower	Upper
AoE+PSCs	0ug/ml	AoE+PSCs	2ug/ml	12.8000	2.1811	5.87	<.0001	0.05	8.5251	17.0749
AoE+PSCs	0ug/ml	AoE+PSCs	6ug/ml	24.0000	2.1811	11.00	<.0001	0.05	19.7251	28.2749
AoE+PSCs	0ug/ml	E10.5AGM	0ug/ml	-25.0000	2.1811	-11.46	<.0001	0.05	-29.2749	-20.7251
AoE+PSCs	0ug/ml	E10.5AGM	2ug/ml	-8.0000	2.1811	-3.67	0.0002	0.05	-12.2749	-3.7251
AoE+PSCs	0ug/ml	E10.5AGM	6ug/ml	15.2000	2.1811	6.97	<.0001	0.05	10.9251	19.4749
AoE+PSCs	0ug/ml	HE+PSCs	0ug/ml	5.6000	2.1811	2.57	0.0102	0.05	1.3251	9.8749
AoE+PSCs	0ug/ml	HE+PSCs	2ug/ml	16.2000	2.1811	7.43	<.0001	0.05	11.9251	20.4749
AoE+PSCs	0ug/ml	HE+PSCs	6ug/ml	27.0000	2.1811	12.38	<.0001	0.05	22.7251	31.2749
AoE+PSCs	2ug/ml	AoE+PSCs	6ug/ml	11.2000	2.1811	5.13	<.0001	0.05	6.9251	15.4749
AoE+PSCs	2ug/ml	E10.5AGM	0ug/ml	-37.8000	2.1811	-17.33	<.0001	0.05	-42.0749	-33.5251
AoE+PSCs	2ug/ml	E10.5AGM	2ug/ml	-20.8000	2.1811	-9.54	<.0001	0.05	-25.0749	-16.5251
AoE+PSCs	2ug/ml	E10.5AGM	6ug/ml	2.4000	2.1811	1.10	0.2712	0.05	-1.8749	6.6749

The GENMOD Procedure

Differences of Condition*Treatment Least Squares Means										
Condition	Treatment	_Condition	_Treatment	Estimate	Standard Error	z Value	Pr > |z|	Alpha	Lower	Upper
AoE+PSCs	2ug/ml	HE+PSCs	0ug/ml	-7.2000	2.1811	-3.30	0.0010	0.05	-11.4749	-2.9251
AoE+PSCs	2ug/ml	HE+PSCs	2ug/ml	3.4000	2.1811	1.56	0.1190	0.05	-0.8749	7.6749
AoE+PSCs	2ug/ml	HE+PSCs	6ug/ml	14.2000	2.1811	6.51	<.0001	0.05	9.9251	18.4749
AoE+PSCs	6ug/ml	E10.5AGM	0ug/ml	-49.0000	2.1811	-22.47	<.0001	0.05	-53.2749	-44.7251
AoE+PSCs	6ug/ml	E10.5AGM	2ug/ml	-32.0000	2.1811	-14.67	<.0001	0.05	-36.2749	-27.7251
AoE+PSCs	6ug/ml	E10.5AGM	6ug/ml	-8.8000	2.1811	-4.03	<.0001	0.05	-13.0749	-4.5251
AoE+PSCs	6ug/ml	HE+PSCs	0ug/ml	-18.4000	2.1811	-8.44	<.0001	0.05	-22.6749	-14.1251
AoE+PSCs	6ug/ml	HE+PSCs	2ug/ml	-7.8000	2.1811	-3.58	0.0003	0.05	-12.0749	-3.5251
AoE+PSCs	6ug/ml	HE+PSCs	6ug/ml	3.0000	2.1811	1.38	0.1690	0.05	-1.2749	7.2749
E10.5AGM	0ug/ml	E10.5AGM	2ug/ml	17.0000	2.1811	7.79	<.0001	0.05	12.7251	21.2749
E10.5AGM	0ug/ml	E10.5AGM	6ug/ml	40.2000	2.1811	18.43	<.0001	0.05	35.9251	44.4749
E10.5AGM	0ug/ml	HE+PSCs	0ug/ml	30.6000	2.1811	14.03	<.0001	0.05	26.3251	34.8749
E10.5AGM	0ug/ml	HE+PSCs	2ug/ml	41.2000	2.1811	18.89	<.0001	0.05	36.9251	45.4749
E10.5AGM	0ug/ml	HE+PSCs	6ug/ml	52.0000	2.1811	23.84	<.0001	0.05	47.7251	56.2749
E10.5AGM	2ug/ml	E10.5AGM	6ug/ml	23.2000	2.1811	10.64	<.0001	0.05	18.9251	27.4749
E10.5AGM	2ug/ml	HE+PSCs	0ug/ml	13.6000	2.1811	6.24	<.0001	0.05	9.3251	17.8749
E10.5AGM	2ug/ml	HE+PSCs	2ug/ml	24.2000	2.1811	11.10	<.0001	0.05	19.9251	28.4749
E10.5AGM	2ug/ml	HE+PSCs	6ug/ml	35.0000	2.1811	16.05	<.0001	0.05	30.7251	39.2749
E10.5AGM	6ug/ml	HE+PSCs	0ug/ml	-9.6000	2.1811	-4.40	<.0001	0.05	-13.8749	-5.3251
E10.5AGM	6ug/ml	HE+PSCs	2ug/ml	1.0000	2.1811	0.46	0.6466	0.05	-3.2749	5.2749
E10.5AGM	6ug/ml	HE+PSCs	6ug/ml	11.8000	2.1811	5.41	<.0001	0.05	7.5251	16.0749
HE+PSCs	0ug/ml	HE+PSCs	2ug/ml	10.6000	2.1811	4.86	<.0001	0.05	6.3251	14.8749
HE+PSCs	0ug/ml	HE+PSCs	6ug/ml	21.4000	2.1811	9.81	<.0001	0.05	17.1251	25.6749
HE+PSCs	2ug/ml	HE+PSCs	6ug/ml	10.8000	2.1811	4.95	<.0001	0.05	6.5251	15.0749

The GENMOD Procedure

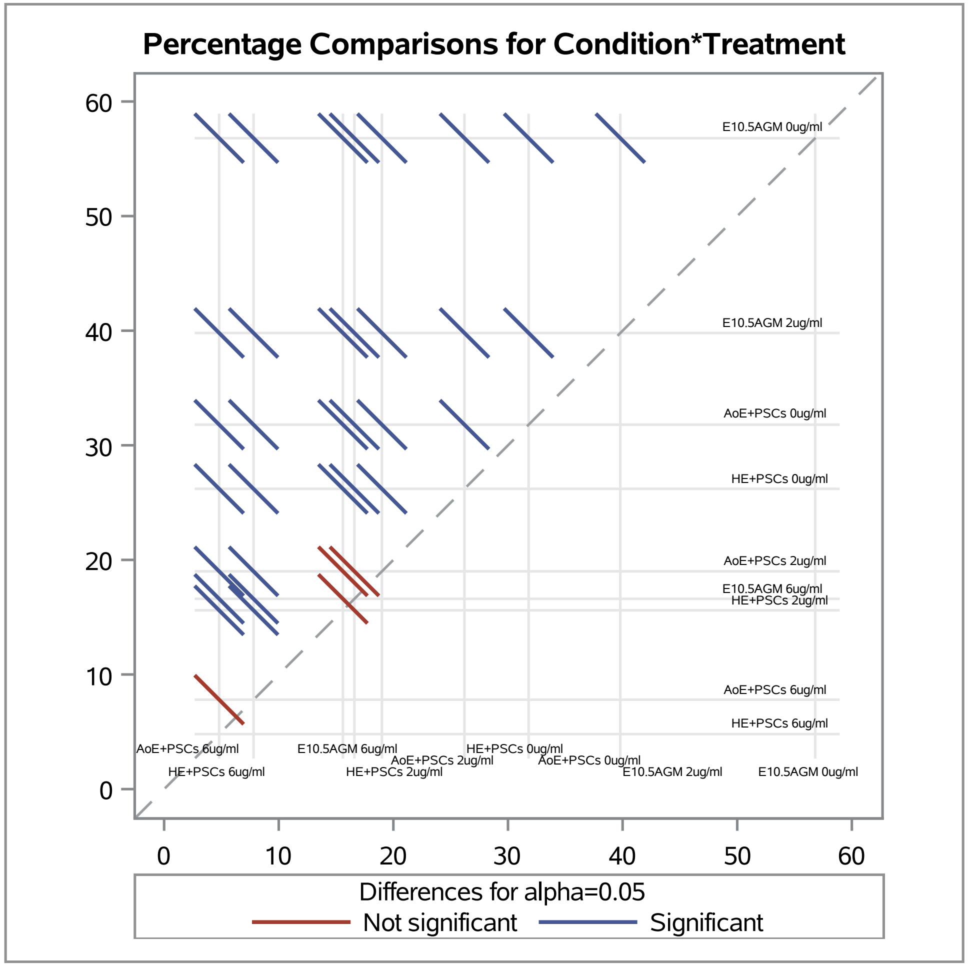

The GENMOD Procedure

Model Information	
**Data Set**	WORK.EXT_FIG10BI_E10LY45_TRANS
**Distribution**	Normal
**Link Function**	Identity
**Dependent Variable**	Percentage

**Number of Observations Read**	45
**Number of Observations Used**	45

Class Level Information		
**Class**	**Levels**	**Values**
**Condition**	3	AoE+PSCs E10.5AGM HE+PSCs
**Treatment**	3	0nM 15nM 45nM

Parameter Information			
**Parameter**	**Effect**	**Condition**	**Treatment**
**Prm1**	Intercept		
**Prm2**	Condition	AoE+PSCs	
**Prm3**	Condition	E10.5AGM	
**Prm4**	Condition	HE+PSCs	
**Prm5**	Treatment		0nM
**Prm6**	Treatment		15nM
**Prm7**	Treatment		45nM
**Prm8**	Condition*Treatment	AoE+PSCs	0nM
**Prm9**	Condition*Treatment	AoE+PSCs	15nM
**Prm10**	Condition*Treatment	AoE+PSCs	45nM
**Prm11**	Condition*Treatment	E10.5AGM	0nM

The GENMOD Procedure

Parameter Information			
**Parameter**	**Effect**	**Condition**	**Treatment**
**Prm12**	Condition*Treatment	E10.5AGM	15nM
**Prm13**	Condition*Treatment	E10.5AGM	45nM
**Prm14**	Condition*Treatment	HE+PSCs	0nM
**Prm15**	Condition*Treatment	HE+PSCs	15nM
**Prm16**	Condition*Treatment	HE+PSCs	45nM

Criteria For Assessing Goodness Of Fit			
**Criterion**	**DF**	**Value**	**Value/DF**
**Deviance**	36	498.8000	13.8556
**Scaled Deviance**	36	45.0000	1.2500
**Pearson Chi-Square**	36	498.8000	13.8556
**Scaled Pearson X2**	36	45.0000	1.2500
**Log Likelihood**		-117.9769	
**Full Log Likelihood**		-117.9769	
**AIC (smaller is better)**		255.9539	
**AICC (smaller is better)**		262.4245	
**BIC (smaller is better)**		274.0205	

Algorithm converged.

The GENMOD Procedure

Analysis Of Maximum Likelihood Parameter Estimates									
Parameter			DF	Estimate	Standard Error	Wald 95% Confidence Limits		Wald Chi-Square	Pr > ChiSq
Intercept			1	4.4000	1.4889	1.4818	7.3182	8.73	0.0031
Condition	AoE+PSCs		1	0.8000	2.1057	-3.3270	4.9270	0.14	0.7040
Condition	E10.5AGM		1	10.4000	2.1057	6.2730	14.5270	24.39	<.0001
Condition	HE+PSCs		0	0.0000	0.0000	0.0000	0.0000	.	.
Treatment	0nM		1	21.8000	2.1057	17.6730	25.9270	107.19	<.0001
Treatment	15nM		1	9.2000	2.1057	5.0730	13.3270	19.09	<.0001
Treatment	45nM		0	0.0000	0.0000	0.0000	0.0000	.	.
Condition*Treatment	AoE+PSCs	0nM	1	4.8000	2.9778	-1.0365	10.6365	2.60	0.1070
Condition*Treatment	AoE+PSCs	15nM	1	0.2000	2.9778	-5.6365	6.0365	0.00	0.9465
Condition*Treatment	AoE+PSCs	45nM	0	0.0000	0.0000	0.0000	0.0000	.	.
Condition*Treatment	E10.5AGM	0nM	1	20.2000	2.9778	14.3635	26.0365	46.01	<.0001
Condition*Treatment	E10.5AGM	15nM	1	5.4000	2.9778	-0.4365	11.2365	3.29	0.0698
Condition*Treatment	E10.5AGM	45nM	0	0.0000	0.0000	0.0000	0.0000	.	.
Condition*Treatment	HE+PSCs	0nM	0	0.0000	0.0000	0.0000	0.0000	.	.
Condition*Treatment	HE+PSCs	15nM	0	0.0000	0.0000	0.0000	0.0000	.	.
Condition*Treatment	HE+PSCs	45nM	0	0.0000	0.0000	0.0000	0.0000	.	.
Scale			1	3.3293	0.3509	2.7079	4.0934		

Note: The scale parameter was estimated by maximum likelihood.

The GENMOD Procedure

Condition*Treatment Least Squares Means								
Condition	Treatment	Estimate	Standard Error	z Value	Pr > |z|	Alpha	Lower	Upper
AoE+PSCs	0nM	31.8000	1.4889	21.36	<.0001	0.05	28.8818	34.7182
AoE+PSCs	15nM	14.6000	1.4889	9.81	<.0001	0.05	11.6818	17.5182
AoE+PSCs	45nM	5.2000	1.4889	3.49	0.0005	0.05	2.2818	8.1182
E10.5AGM	0nM	56.8000	1.4889	38.15	<.0001	0.05	53.8818	59.7182
E10.5AGM	15nM	29.4000	1.4889	19.75	<.0001	0.05	26.4818	32.3182
E10.5AGM	45nM	14.8000	1.4889	9.94	<.0001	0.05	11.8818	17.7182
HE+PSCs	0nM	26.2000	1.4889	17.60	<.0001	0.05	23.2818	29.1182
HE+PSCs	15nM	13.6000	1.4889	9.13	<.0001	0.05	10.6818	16.5182
HE+PSCs	45nM	4.4000	1.4889	2.96	0.0031	0.05	1.4818	7.3182

Differences of Condition*Treatment Least Squares Means										
Condition	Treatment	_Condition	_Treatment	Estimate	Standard Error	z Value	Pr > |z|	Alpha	Lower	Upper
AoE+PSCs	0nM	AoE+PSCs	15nM	17.2000	2.1057	8.17	<.0001	0.05	13.0730	21.3270
AoE+PSCs	0nM	AoE+PSCs	45nM	26.6000	2.1057	12.63	<.0001	0.05	22.4730	30.7270
AoE+PSCs	0nM	E10.5AGM	0nM	-25.0000	2.1057	-11.87	<.0001	0.05	-29.1270	-20.8730
AoE+PSCs	0nM	E10.5AGM	15nM	2.4000	2.1057	1.14	0.2544	0.05	-1.7270	6.5270
AoE+PSCs	0nM	E10.5AGM	45nM	17.0000	2.1057	8.07	<.0001	0.05	12.8730	21.1270
AoE+PSCs	0nM	HE+PSCs	0nM	5.6000	2.1057	2.66	0.0078	0.05	1.4730	9.7270
AoE+PSCs	0nM	HE+PSCs	15nM	18.2000	2.1057	8.64	<.0001	0.05	14.0730	22.3270
AoE+PSCs	0nM	HE+PSCs	45nM	27.4000	2.1057	13.01	<.0001	0.05	23.2730	31.5270
AoE+PSCs	15nM	AoE+PSCs	45nM	9.4000	2.1057	4.46	<.0001	0.05	5.2730	13.5270
AoE+PSCs	15nM	E10.5AGM	0nM	-42.2000	2.1057	-20.04	<.0001	0.05	-46.3270	-38.0730
AoE+PSCs	15nM	E10.5AGM	15nM	-14.8000	2.1057	-7.03	<.0001	0.05	-18.9270	-10.6730
AoE+PSCs	15nM	E10.5AGM	45nM	-0.2000	2.1057	-0.09	0.9243	0.05	-4.3270	3.9270

The GENMOD Procedure

Differences of Condition*Treatment Least Squares Means										
Condition	Treatment	_Condition	_Treatment	Estimate	Standard Error	z Value	Pr > |z|	Alpha	Lower	Upper
AoE+PSCs	15nM	HE+PSCs	0nM	-11.6000	2.1057	-5.51	<.0001	0.05	-15.7270	-7.4730
AoE+PSCs	15nM	HE+PSCs	15nM	1.0000	2.1057	0.47	0.6348	0.05	-3.1270	5.1270
AoE+PSCs	15nM	HE+PSCs	45nM	10.2000	2.1057	4.84	<.0001	0.05	6.0730	14.3270
AoE+PSCs	45nM	E10.5AGM	0nM	-51.6000	2.1057	-24.51	<.0001	0.05	-55.7270	-47.4730
AoE+PSCs	45nM	E10.5AGM	15nM	-24.2000	2.1057	-11.49	<.0001	0.05	-28.3270	-20.0730
AoE+PSCs	45nM	E10.5AGM	45nM	-9.6000	2.1057	-4.56	<.0001	0.05	-13.7270	-5.4730
AoE+PSCs	45nM	HE+PSCs	0nM	-21.0000	2.1057	-9.97	<.0001	0.05	-25.1270	-16.8730
AoE+PSCs	45nM	HE+PSCs	15nM	-8.4000	2.1057	-3.99	<.0001	0.05	-12.5270	-4.2730
AoE+PSCs	45nM	HE+PSCs	45nM	0.8000	2.1057	0.38	0.7040	0.05	-3.3270	4.9270
E10.5AGM	0nM	E10.5AGM	15nM	27.4000	2.1057	13.01	<.0001	0.05	23.2730	31.5270
E10.5AGM	0nM	E10.5AGM	45nM	42.0000	2.1057	19.95	<.0001	0.05	37.8730	46.1270
E10.5AGM	0nM	HE+PSCs	0nM	30.6000	2.1057	14.53	<.0001	0.05	26.4730	34.7270
E10.5AGM	0nM	HE+PSCs	15nM	43.2000	2.1057	20.52	<.0001	0.05	39.0730	47.3270
E10.5AGM	0nM	HE+PSCs	45nM	52.4000	2.1057	24.89	<.0001	0.05	48.2730	56.5270
E10.5AGM	15nM	E10.5AGM	45nM	14.6000	2.1057	6.93	<.0001	0.05	10.4730	18.7270
E10.5AGM	15nM	HE+PSCs	0nM	3.2000	2.1057	1.52	0.1286	0.05	-0.9270	7.3270
E10.5AGM	15nM	HE+PSCs	15nM	15.8000	2.1057	7.50	<.0001	0.05	11.6730	19.9270
E10.5AGM	15nM	HE+PSCs	45nM	25.0000	2.1057	11.87	<.0001	0.05	20.8730	29.1270
E10.5AGM	45nM	HE+PSCs	0nM	-11.4000	2.1057	-5.41	<.0001	0.05	-15.5270	-7.2730
E10.5AGM	45nM	HE+PSCs	15nM	1.2000	2.1057	0.57	0.5687	0.05	-2.9270	5.3270
E10.5AGM	45nM	HE+PSCs	45nM	10.4000	2.1057	4.94	<.0001	0.05	6.2730	14.5270
HE+PSCs	0nM	HE+PSCs	15nM	12.6000	2.1057	5.98	<.0001	0.05	8.4730	16.7270
HE+PSCs	0nM	HE+PSCs	45nM	21.8000	2.1057	10.35	<.0001	0.05	17.6730	25.9270
HE+PSCs	15nM	HE+PSCs	45nM	9.2000	2.1057	4.37	<.0001	0.05	5.0730	13.3270

The GENMOD Procedure

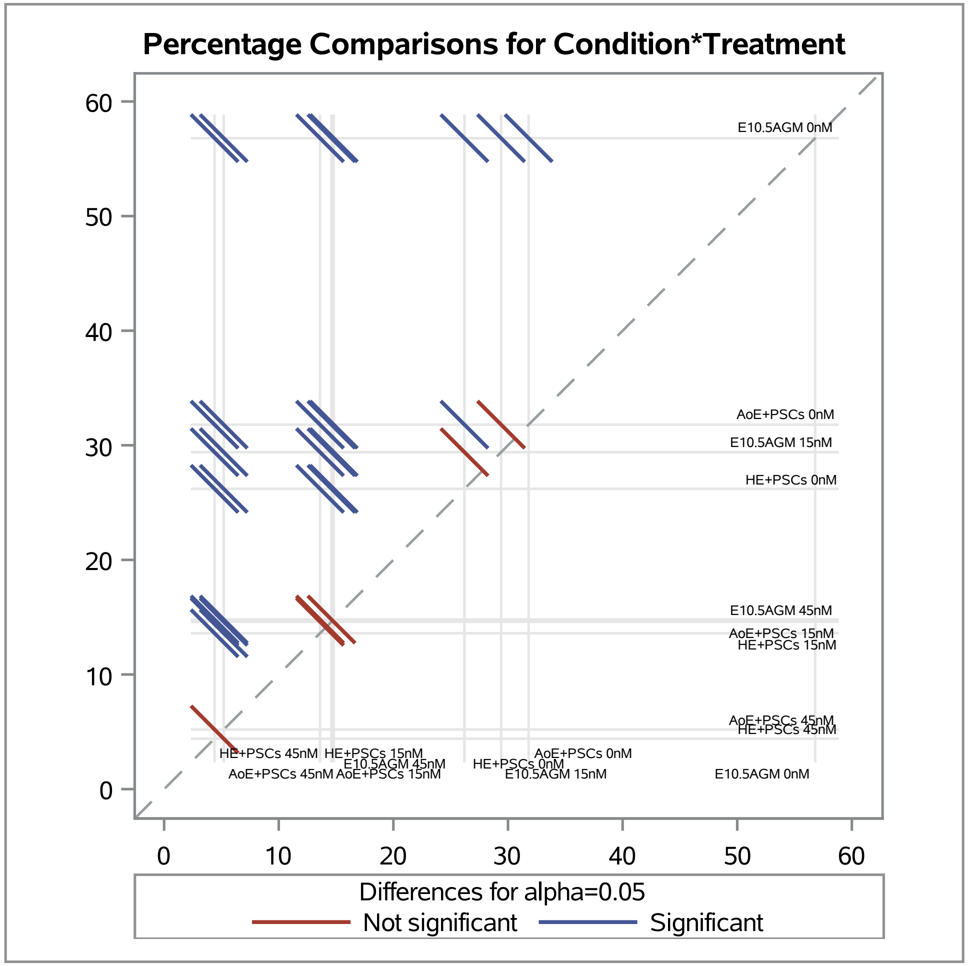

The GLIMMIX Procedure

Model Information	
**Data Set**	WORK.EXT_FIG10BI_E10WIF1
**Response Variable**	Count
**Response Distribution**	Poisson
**Link Function**	Log
**Variance Function**	Default
**Variance Matrix Blocked By**	Dish_ID
**Estimation Technique**	Maximum Likelihood
**Likelihood Approximation**	Laplace
**Degrees of Freedom Method**	Containment

Class Level Information		
**Class**	**Levels**	**Values**
**Colony_Type**	3	BFU-E CFU-GEMM CFU-GM
**Condition**	3	AoE+PSCs E10.5AGM HE+PSCs
**Treatment**	3	0ug/ml 2.5ug/ml 7.5ug/ml
**Dish_ID**	27	1 2 3 4 5 6 7 8 9 10 11 12 13 14 15 16 17 18 19 20 21 22 23 24 25 26 27

**Number of Observations Read**	81
**Number of Observations Used**	81

Dimensions	
**G-side Cov. Parameters**	1
**Columns in X**	64
**Columns in Z per Subject**	1
**Subjects (Blocks in V)**	27
**Max Obs per Subject**	3

The GLIMMIX Procedure

Optimization Information	
Optimization Technique	Dual Quasi-Newton
Parameters in Optimization	28
Lower Boundaries	1
Upper Boundaries	0
Fixed Effects	Not Profiled
Starting From	GLM estimates

Iteration History

Iteration	Restarts	Evaluations	Objective Function	Change	Max Gradient
0	0	4	717.64400565	.	512.4832
1	0	7	717.36334078	0.28066486	107.5497
2	0	2	717.3412023	0.02213849	33.02827
3	0	2	717.33781879	0.00338350	15.65809
4	0	4	717.32988008	0.00793871	39.24529
5	0	4	717.32129828	0.00858180	6.815119
6	0	3	717.32106376	0.00023452	1.53951
7	0	4	717.31903151	0.00203224	25.53964
8	0	3	717.31771309	0.00131843	1.911414
9	0	2	717.31637871	0.00133437	1.730365
10	0	3	717.31609416	0.00028455	2.27353
11	0	2	717.31566517	0.00042899	3.177562
12	0	4	717.31165636	0.00400881	6.378231
13	0	3	717.3110878	0.00056855	0.993935
14	0	3	717.3110497	0.00003810	0.97232
15	0	4	717.31061363	0.00043607	3.375314

The GLIMMIX Procedure

Iteration History					
**Iteration**	**Restarts**	**Evaluations**	**Objective Function**	**Change**	**Max Gradient**
**16**	**0**	3	717.31056731	0.00004632	0.61235
**17**	**0**	2	717.31052862	0.00003870	0.834995

Convergence criterion (GCONV=1E-8) satisfied.

Fit Statistics	
**-2 Log Likelihood**	717.31
**AIC (smaller is better)**	773.31
**AICC (smaller is better)**	804.54
**BIC (smaller is better)**	809.59
**CAIC (smaller is better)**	837.59
**HQIC (smaller is better)**	784.10

Fit Statistics for Conditional Distribution	
**-2 log L(Count | r. effects)**	664.26
**Pearson Chi-Square**	142.85
**Pearson Chi-Square / DF**	1.76

Covariance Parameter Estimates			
**Cov Parm**	**Subject**	**Estimate**	**Standard Error**
**Intercept**	Dish_ID	0.006090	0.002675

The GLIMMIX Procedure

Solutions for Fixed Effects								
Effect	Colony_Type	Condition	Treatment	Estimate	Standard Error	DF	t Value	Pr > |t|
Intercept				4.5800	0.07382	18	62.05	<.0001
Colony_Type	BFU-E			-1.9463	0.1649	36	-11.80	<.0001
Colony_Type	CFU-GEMM			-1.7923	0.1542	36	-11.62	<.0001
Colony_Type	CFU-GM			0	.	.	.	.
Condition		AoE+PSCs		0.2451	0.1007	36	2.43	0.0201
Condition		E10.5AGM		0.8663	0.09439	36	9.18	<.0001
Condition		HE+PSCs		0	.	.	.	.
Colony_Typ*Condition	BFU-E	AoE+PSCs		-0.1741	0.2282	36	-0.76	0.4505
Colony_Typ*Condition	BFU-E	E10.5AGM		0.5554	0.1854	36	3.00	0.0049
Colony_Typ*Condition	BFU-E	HE+PSCs		0	.	.	.	.
Colony_Typ*Condition	CFU-GEMM	AoE+PSCs		0.008172	0.2058	36	0.04	0.9685
Colony_Typ*Condition	CFU-GEMM	E10.5AGM		0.3481	0.1769	36	1.97	0.0568
Colony_Typ*Condition	CFU-GEMM	HE+PSCs		0	.	.	.	.
Colony_Typ*Condition	CFU-GM	AoE+PSCs		0	.	.	.	.
Colony_Typ*Condition	CFU-GM	E10.5AGM		0	.	.	.	.
Colony_Typ*Condition	CFU-GM	HE+PSCs		0	.	.	.	.
Treatment			0ug/ml	1.5261	0.09066	36	16.83	<.0001
Treatment			2.5ug/ml	0.7346	0.09545	36	7.70	<.0001
Treatment			7.5ug/ml	0	.	.	.	.
Colony_Typ*Treatment	BFU-E		0ug/ml	0.4004	0.1772	36	2.26	0.0300
Colony_Typ*Treatment	BFU-E		2.5ug/ml	0.3894	0.1912	36	2.04	0.0492
Colony_Typ*Treatment	BFU-E		7.5ug/ml	0	.	.	.	.
Colony_Typ*Treatment	CFU-GEMM		0ug/ml	0.1592	0.1683	36	0.95	0.3505
Colony_Typ*Treatment	CFU-GEMM		2.5ug/ml	0.1203	0.1847	36	0.65	0.5191

The GLIMMIX Procedure

Solutions for Fixed Effects								
Effect	Colony_Type	Condition	Treatment	Estimate	Standard Error	DF	t Value	Pr > |t|
**Colony_Typ*Treatment**	CFU-GEMM		7.5ug/ml	0	.	.	.	.
**Colony_Typ*Treatment**	CFU-GM		0ug/ml	0	.	.	.	.
**Colony_Typ*Treatment**	CFU-GM		2.5ug/ml	0	.	.	.	.
**Colony_Typ*Treatment**	CFU-GM		7.5ug/ml	0	.	.	.	.
**Condition*Treatment**		AoE+PSCs	0ug/ml	-0.1571	0.1250	36	-1.26	0.2170
**Condition*Treatment**		AoE+PSCs	2.5ug/ml	-0.1549	0.1317	36	-1.18	0.2472
**Condition*Treatment**		AoE+PSCs	7.5ug/ml	0	.	.	.	.
**Condition*Treatment**		E10.5AGM	0ug/ml	-0.3340	0.1189	36	-2.81	0.0080
**Condition*Treatment**		E10.5AGM	2.5ug/ml	-0.1061	0.1240	36	-0.86	0.3978
**Condition*Treatment**		E10.5AGM	7.5ug/ml	0	.	.	.	.
**Condition*Treatment**		HE+PSCs	0ug/ml	0	.	.	.	.
**Condition*Treatment**		HE+PSCs	2.5ug/ml	0	.	.	.	.
**Condition*Treatment**		HE+PSCs	7.5ug/ml	0	.	.	.	.
**Colony*Condit*Treatm**	BFU-E	AoE+PSCs	0ug/ml	0.2965	0.2446	36	1.21	0.2333
**Colony*Condit*Treatm**	BFU-E	AoE+PSCs	2.5ug/ml	0.1952	0.2644	36	0.74	0.4651
**Colony*Condit*Treatm**	BFU-E	AoE+PSCs	7.5ug/ml	0	.	.	.	.
**Colony*Condit*Treatm**	BFU-E	E10.5AGM	0ug/ml	-0.3593	0.2017	36	-1.78	0.0833
**Colony*Condit*Treatm**	BFU-E	E10.5AGM	2.5ug/ml	-0.1453	0.2166	36	-0.67	0.5065
**Colony*Condit*Treatm**	BFU-E	E10.5AGM	7.5ug/ml	0	.	.	.	.
**Colony*Condit*Treatm**	BFU-E	HE+PSCs	0ug/ml	0	.	.	.	.
**Colony*Condit*Treatm**	BFU-E	HE+PSCs	2.5ug/ml	0	.	.	.	.
**Colony*Condit*Treatm**	BFU-E	HE+PSCs	7.5ug/ml	0	.	.	.	.
**Colony*Condit*Treatm**	CFU-GEMM	AoE+PSCs	0ug/ml	0.05544	0.2254	36	0.25	0.8071
**Colony*Condit*Treatm**	CFU-GEMM	AoE+PSCs	2.5ug/ml	0.2142	0.2461	36	0.87	0.3897

The GLIMMIX Procedure

Solutions for Fixed Effects								
Effect	Colony_Type	Condition	Treatment	Estimate	Standard Error	DF	t Value	Pr > |t|
**Colony*Condit*Treatm**	CFU-GEMM	AoE+PSCs	7.5ug/ml	0	.	.	.	.
**Colony*Condit*Treatm**	CFU-GEMM	E10.5AGM	0ug/ml	-0.1733	0.1952	36	-0.89	0.3807
**Colony*Condit*Treatm**	CFU-GEMM	E10.5AGM	2.5ug/ml	0.1887	0.2116	36	0.89	0.3783
**Colony*Condit*Treatm**	CFU-GEMM	E10.5AGM	7.5ug/ml	0	.	.	.	.
**Colony*Condit*Treatm**	CFU-GEMM	HE+PSCs	0ug/ml	0	.	.	.	.
**Colony*Condit*Treatm**	CFU-GEMM	HE+PSCs	2.5ug/ml	0	.	.	.	.
**Colony*Condit*Treatm**	CFU-GEMM	HE+PSCs	7.5ug/ml	0	.	.	.	.
**Colony*Condit*Treatm**	CFU-GM	AoE+PSCs	0ug/ml	0	.	.	.	.
**Colony*Condit*Treatm**	CFU-GM	AoE+PSCs	2.5ug/ml	0	.	.	.	.
**Colony*Condit*Treatm**	CFU-GM	AoE+PSCs	7.5ug/ml	0	.	.	.	.
**Colony*Condit*Treatm**	CFU-GM	E10.5AGM	0ug/ml	0	.	.	.	.
**Colony*Condit*Treatm**	CFU-GM	E10.5AGM	2.5ug/ml	0	.	.	.	.
**Colony*Condit*Treatm**	CFU-GM	E10.5AGM	7.5ug/ml	0	.	.	.	.
**Colony*Condit*Treatm**	CFU-GM	HE+PSCs	0ug/ml	0	.	.	.	.
**Colony*Condit*Treatm**	CFU-GM	HE+PSCs	2.5ug/ml	0	.	.	.	.
**Colony*Condit*Treatm**	CFU-GM	HE+PSCs	7.5ug/ml	0	.	.	.	.

Type III Tests of Fixed Effects				
Effect	Num DF	Den DF	F Value	Pr > F
**Colony_Type**	2	36	1971.71	<.0001
**Condition**	2	36	229.00	<.0001
**Colony_Typ*Condition**	4	36	15.43	<.0001
**Treatment**	2	36	419.26	<.0001
**Colony_Typ*Treatment**	4	36	6.93	0.0003

The GLIMMIX Procedure

Type III Tests of Fixed Effects				
Effect	Num DF	Den DF	F Value	Pr > F
Condition*Treatment	4	36	7.48	0.0002
Colony*Condit*Treatm	8	36	2.65	0.0213

Condition*Treatment Least Squares Means													
Condition	Treatment	Estimate	Standard Error	DF	t Value	Pr > |t|	Alpha	Lower	Upper	Mean	Standard Error Mean	Lower Mean	Upper Mean
AoE+PSCs	0ug/ml	5.1963	0.05275	36	98.50	<.0001	0.05	5.0893	5.3033	180.61	9.5276	162.28	201.00
AoE+PSCs	2.5ug/ml	4.4096	0.06065	36	72.70	<.0001	0.05	4.2866	4.5326	82.2397	4.9880	72.7210	93.0044
AoE+PSCs	7.5ug/ml	3.5235	0.08103	36	43.48	<.0001	0.05	3.3592	3.6879	33.9042	2.7473	28.7662	39.9600
E10.5AGM	0ug/ml	5.7023	0.04974	36	114.65	<.0001	0.05	5.6015	5.8032	299.57	14.8997	270.82	331.36
E10.5AGM	2.5ug/ml	5.3142	0.05147	36	103.26	<.0001	0.05	5.2098	5.4185	203.19	10.4574	183.05	225.55
E10.5AGM	7.5ug/ml	4.5013	0.05923	36	75.99	<.0001	0.05	4.3812	4.6214	90.1337	5.3389	79.9310	101.64
HE+PSCs	0ug/ml	5.0464	0.05406	36	93.34	<.0001	0.05	4.9367	5.1560	155.46	8.4043	139.31	173.47
HE+PSCs	2.5ug/ml	4.2383	0.06359	36	66.65	<.0001	0.05	4.1093	4.3673	69.2894	4.4059	60.9061	78.8266
HE+PSCs	7.5ug/ml	3.3338	0.08566	36	38.92	<.0001	0.05	3.1601	3.5075	28.0446	2.4024	23.5721	33.3657

Differences of Condition*Treatment Least Squares Means											
Condition	Treatment	_Condition	_Treatment	Estimate	Standard Error	DF	t Value	Pr > |t|	Alpha	Lower	Upper
AoE+PSCs	0ug/ml	AoE+PSCs	2.5ug/ml	0.7867	0.08038	36	9.79	<.0001	0.05	0.6237	0.9497
AoE+PSCs	0ug/ml	AoE+PSCs	7.5ug/ml	1.6728	0.09669	36	17.30	<.0001	0.05	1.4767	1.8689
AoE+PSCs	0ug/ml	E10.5AGM	0ug/ml	-0.5060	0.07250	36	-6.98	<.0001	0.05	-0.6530	-0.3590
AoE+PSCs	0ug/ml	E10.5AGM	2.5ug/ml	-0.1178	0.07370	36	-1.60	0.1186	0.05	-0.2673	0.03164
AoE+PSCs	0ug/ml	E10.5AGM	7.5ug/ml	0.6950	0.07932	36	8.76	<.0001	0.05	0.5342	0.8559
AoE+PSCs	0ug/ml	HE+PSCs	0ug/ml	0.1500	0.07554	36	1.99	0.0548	0.05	-0.00323	0.3032

The GLIMMIX Procedure

Differences of Condition*Treatment Least Squares Means											
Condition	Treatment	_Condition	_Treatment	Estimate	Standard Error	DF	t Value	Pr > |t|	Alpha	Lower	Upper
AoE+PSCs	0ug/ml	HE+PSCs	2.5ug/ml	0.9580	0.08262	36	11.60	<.0001	0.05	0.7905	1.1256
AoE+PSCs	0ug/ml	HE+PSCs	7.5ug/ml	1.8625	0.1006	36	18.51	<.0001	0.05	1.6585	2.0666
AoE+PSCs	2.5ug/ml	AoE+PSCs	7.5ug/ml	0.8861	0.1012	36	8.75	<.0001	0.05	0.6808	1.0914
AoE+PSCs	2.5ug/ml	E10.5AGM	0ug/ml	-1.2927	0.07844	36	-16.48	<.0001	0.05	-1.4518	-1.1336
AoE+PSCs	2.5ug/ml	E10.5AGM	2.5ug/ml	-0.9045	0.07954	36	-11.37	<.0001	0.05	-1.0658	-0.7432
AoE+PSCs	2.5ug/ml	E10.5AGM	7.5ug/ml	-0.09166	0.08477	36	-1.08	0.2868	0.05	-0.2636	0.08027
AoE+PSCs	2.5ug/ml	HE+PSCs	0ug/ml	-0.6367	0.08124	36	-7.84	<.0001	0.05	-0.8015	-0.4720
AoE+PSCs	2.5ug/ml	HE+PSCs	2.5ug/ml	0.1713	0.08787	36	1.95	0.0590	0.05	-0.00686	0.3496
AoE+PSCs	2.5ug/ml	HE+PSCs	7.5ug/ml	1.0758	0.1049	36	10.25	<.0001	0.05	0.8630	1.2887
AoE+PSCs	7.5ug/ml	E10.5AGM	0ug/ml	-2.1788	0.09508	36	-22.92	<.0001	0.05	-2.3716	-1.9860
AoE+PSCs	7.5ug/ml	E10.5AGM	2.5ug/ml	-1.7906	0.09599	36	-18.65	<.0001	0.05	-1.9853	-1.5959
AoE+PSCs	7.5ug/ml	E10.5AGM	7.5ug/ml	-0.9778	0.1004	36	-9.74	<.0001	0.05	-1.1813	-0.7742
AoE+PSCs	7.5ug/ml	HE+PSCs	0ug/ml	-1.5228	0.09740	36	-15.63	<.0001	0.05	-1.7204	-1.3253
AoE+PSCs	7.5ug/ml	HE+PSCs	2.5ug/ml	-0.7148	0.1030	36	-6.94	<.0001	0.05	-0.9236	-0.5059
AoE+PSCs	7.5ug/ml	HE+PSCs	7.5ug/ml	0.1897	0.1179	36	1.61	0.1163	0.05	-0.04936	0.4289
E10.5AGM	0ug/ml	E10.5AGM	2.5ug/ml	0.3882	0.07157	36	5.42	<.0001	0.05	0.2430	0.5333
E10.5AGM	0ug/ml	E10.5AGM	7.5ug/ml	1.2010	0.07734	36	15.53	<.0001	0.05	1.0442	1.3579
E10.5AGM	0ug/ml	HE+PSCs	0ug/ml	0.6560	0.07346	36	8.93	<.0001	0.05	0.5070	0.8049
E10.5AGM	0ug/ml	HE+PSCs	2.5ug/ml	1.4640	0.08073	36	18.14	<.0001	0.05	1.3003	1.6278
E10.5AGM	0ug/ml	HE+PSCs	7.5ug/ml	2.3685	0.09905	36	23.91	<.0001	0.05	2.1677	2.5694
E10.5AGM	2.5ug/ml	E10.5AGM	7.5ug/ml	0.8129	0.07846	36	10.36	<.0001	0.05	0.6537	0.9720
E10.5AGM	2.5ug/ml	HE+PSCs	0ug/ml	0.2678	0.07464	36	3.59	0.0010	0.05	0.1164	0.4192
E10.5AGM	2.5ug/ml	HE+PSCs	2.5ug/ml	1.0759	0.08180	36	13.15	<.0001	0.05	0.9100	1.2418
E10.5AGM	2.5ug/ml	HE+PSCs	7.5ug/ml	1.9804	0.09993	36	19.82	<.0001	0.05	1.7777	2.1830

The GLIMMIX Procedure

Differences of Condition*Treatment Least Squares Means											
Condition	Treatment	_Condition	_Treatment	Estimate	Standard Error	DF	t Value	Pr > |t|	Alpha	Lower	Upper
E10.5AGM	7.5ug/ml	HE+PSCs	0ug/ml	-0.5451	0.08018	36	-6.80	<.0001	0.05	-0.7077	-0.3825
E10.5AGM	7.5ug/ml	HE+PSCs	2.5ug/ml	0.2630	0.08689	36	3.03	0.0045	0.05	0.08678	0.4392
E10.5AGM	7.5ug/ml	HE+PSCs	7.5ug/ml	1.1675	0.1041	36	11.21	<.0001	0.05	0.9563	1.3787
HE+PSCs	0ug/ml	HE+PSCs	2.5ug/ml	0.8081	0.08345	36	9.68	<.0001	0.05	0.6388	0.9773
HE+PSCs	0ug/ml	HE+PSCs	7.5ug/ml	1.7126	0.1013	36	16.91	<.0001	0.05	1.5072	1.9180
HE+PSCs	2.5ug/ml	HE+PSCs	7.5ug/ml	0.9045	0.1067	36	8.48	<.0001	0.05	0.6882	1.1208

Colony*Condit*Treatm Least Squares Means														
Colony_Type	Condition	Treatment	Estimate	Standard Error	DF	t Value	Pr > |t|	Alpha	Lower	Upper	Mean	Standard Error Mean	Lower Mean	Upper Mean
BFU-E	AoE+PSCs	0ug/ml	4.7705	0.06967	36	68.47	<.0001	0.05	4.6292	4.9118	117.98	8.2199	102.43	135.88
BFU-E	AoE+PSCs	2.5ug/ml	3.8690	0.09474	36	40.84	<.0001	0.05	3.6768	4.0611	47.8927	4.5374	39.5206	58.0384
BFU-E	AoE+PSCs	7.5ug/ml	2.7046	0.1558	36	17.36	<.0001	0.05	2.3886	3.0206	14.9488	2.3291	10.8987	20.5038
BFU-E	E10.5AGM	0ug/ml	5.2886	0.06092	36	86.81	<.0001	0.05	5.1650	5.4121	198.06	12.0657	175.05	224.11
BFU-E	E10.5AGM	2.5ug/ml	4.9280	0.06664	36	73.95	<.0001	0.05	4.7929	5.0631	138.10	9.2025	120.65	158.09
BFU-E	E10.5AGM	7.5ug/ml	4.0555	0.08823	36	45.96	<.0001	0.05	3.8765	4.2344	57.7114	5.0921	48.2555	69.0203
BFU-E	HE+PSCs	0ug/ml	4.5602	0.07420	36	61.45	<.0001	0.05	4.4097	4.7106	95.5984	7.0938	82.2417	111.12
BFU-E	HE+PSCs	2.5ug/ml	3.7577	0.09894	36	37.98	<.0001	0.05	3.5570	3.9583	42.8490	4.2395	35.0587	52.3704
BFU-E	HE+PSCs	7.5ug/ml	2.6337	0.1607	36	16.38	<.0001	0.05	2.3077	2.9597	13.9252	2.2384	10.0513	19.2923
CFU-GEMM	AoE+PSCs	0ug/ml	4.6245	0.07279	36	63.53	<.0001	0.05	4.4769	4.7721	101.95	7.4211	87.9589	118.17
CFU-GEMM	AoE+PSCs	2.5ug/ml	3.9552	0.09166	36	43.15	<.0001	0.05	3.7692	4.1411	52.2035	4.7852	43.3474	62.8691
CFU-GEMM	AoE+PSCs	7.5ug/ml	3.0409	0.1339	36	22.71	<.0001	0.05	2.7694	3.3124	20.9245	2.8013	15.9492	27.4518
CFU-GEMM	E10.5AGM	0ug/ml	5.1800	0.06248	36	82.91	<.0001	0.05	5.0533	5.3068	177.69	11.1021	156.54	201.70
CFU-GEMM	E10.5AGM	2.5ug/ml	4.9396	0.06643	36	74.36	<.0001	0.05	4.8049	5.0743	139.72	9.2810	122.11	159.87

The GLIMMIX Procedure

Colony*Condit*Treatm Least Squares Means														
Colony_Type	Condition	Treatment	Estimate	Standard Error	DF	t Value	Pr > |t|	Alpha	Lower	Upper	Mean	Standard Error Mean	Lower Mean	Upper Mean
CFU-GEMM	E10.5AGM	7.5ug/ml	4.0021	0.09000	36	44.47	<.0001	0.05	3.8196	4.1846	54.7126	4.9242	45.5843	65.6689
CFU-GEMM	HE+PSCs	0ug/ml	4.4729	0.07631	36	58.62	<.0001	0.05	4.3182	4.6277	87.6113	6.6854	75.0497	102.28
CFU-GEMM	HE+PSCs	2.5ug/ml	3.6426	0.1036	36	35.16	<.0001	0.05	3.4324	3.8527	38.1900	3.9569	30.9520	47.1204
CFU-GEMM	HE+PSCs	7.5ug/ml	2.7877	0.1498	36	18.61	<.0001	0.05	2.4838	3.0915	16.2433	2.4335	11.9873	22.0105
CFU-GM	AoE+PSCs	0ug/ml	6.1940	0.05206	36	118.97	<.0001	0.05	6.0884	6.2996	489.81	25.5012	440.73	544.36
CFU-GM	AoE+PSCs	2.5ug/ml	5.4048	0.05940	36	90.99	<.0001	0.05	5.2843	5.5253	222.47	13.2144	197.22	250.95
CFU-GM	AoE+PSCs	7.5ug/ml	4.8251	0.06857	36	70.37	<.0001	0.05	4.6860	4.9641	124.60	8.5429	108.42	143.18
CFU-GM	E10.5AGM	0ug/ml	6.6384	0.04966	36	133.67	<.0001	0.05	6.5377	6.7391	763.85	37.9343	690.66	844.79
CFU-GM	E10.5AGM	2.5ug/ml	6.0749	0.05288	36	114.87	<.0001	0.05	5.9676	6.1821	434.79	22.9926	390.57	484.01
CFU-GM	E10.5AGM	7.5ug/ml	5.4463	0.05889	36	92.49	<.0001	0.05	5.3269	5.5658	231.91	13.6563	205.80	261.32
CFU-GM	HE+PSCs	0ug/ml	6.1061	0.05267	36	115.94	<.0001	0.05	5.9992	6.2129	448.57	23.6238	403.12	499.13
CFU-GM	HE+PSCs	2.5ug/ml	5.3146	0.06056	36	87.76	<.0001	0.05	5.1918	5.4374	203.29	12.3114	179.79	229.85
CFU-GM	HE+PSCs	7.5ug/ml	4.5800	0.07382	36	62.05	<.0001	0.05	4.4303	4.7297	97.5148	7.1981	83.9565	113.26

The GLIMMIX Procedure

Differences of Colony*Condit*Treatm Least Squares Means													
Colony_Type	Condition	Treatment	_Colony_Type	_Condition	_Treatment	Estimate	Standard Error	DF	t Value	Pr > |t|	Alpha	Lower	Upper
BFU-E	AoE+PSCs	0ug/ml	BFU-E	AoE+PSCs	2.5ug/ml	0.9015	0.1176	36	7.67	<.0001	0.05	0.6630	1.1400
BFU-E	AoE+PSCs	0ug/ml	BFU-E	AoE+PSCs	7.5ug/ml	2.0658	0.1707	36	12.10	<.0001	0.05	1.7197	2.4120
BFU-E	AoE+PSCs	0ug/ml	BFU-E	E10.5AGM	0ug/ml	-0.5181	0.09255	36	-5.60	<.0001	0.05	-0.7058	-0.3304
BFU-E	AoE+PSCs	0ug/ml	BFU-E	E10.5AGM	2.5ug/ml	-0.1575	0.09641	36	-1.63	0.1110	0.05	-0.3530	0.03801
BFU-E	AoE+PSCs	0ug/ml	BFU-E	E10.5AGM	7.5ug/ml	0.7150	0.1124	36	6.36	<.0001	0.05	0.4870	0.9430
BFU-E	AoE+PSCs	0ug/ml	BFU-E	HE+PSCs	0ug/ml	0.2103	0.1018	36	2.07	0.0461	0.05	0.003886	0.4168
BFU-E	AoE+PSCs	0ug/ml	BFU-E	HE+PSCs	2.5ug/ml	1.0128	0.1210	36	8.37	<.0001	0.05	0.7674	1.2582
BFU-E	AoE+PSCs	0ug/ml	BFU-E	HE+PSCs	7.5ug/ml	2.1368	0.1752	36	12.20	<.0001	0.05	1.7815	2.4921
BFU-E	AoE+PSCs	0ug/ml	CFU-GEMM	AoE+PSCs	0ug/ml	0.1460	0.07805	36	1.87	0.0696	0.05	-0.01232	0.3043
BFU-E	AoE+PSCs	0ug/ml	CFU-GEMM	AoE+PSCs	2.5ug/ml	0.8153	0.1151	36	7.08	<.0001	0.05	0.5818	1.0488
BFU-E	AoE+PSCs	0ug/ml	CFU-GEMM	AoE+PSCs	7.5ug/ml	1.7296	0.1509	36	11.46	<.0001	0.05	1.4235	2.0356
BFU-E	AoE+PSCs	0ug/ml	CFU-GEMM	E10.5AGM	0ug/ml	-0.4096	0.09359	36	-4.38	<.0001	0.05	-0.5994	-0.2198
BFU-E	AoE+PSCs	0ug/ml	CFU-GEMM	E10.5AGM	2.5ug/ml	-0.1692	0.09627	36	-1.76	0.0874	0.05	-0.3644	0.02608
BFU-E	AoE+PSCs	0ug/ml	CFU-GEMM	E10.5AGM	7.5ug/ml	0.7684	0.1138	36	6.75	<.0001	0.05	0.5375	0.9992
BFU-E	AoE+PSCs	0ug/ml	CFU-GEMM	HE+PSCs	0ug/ml	0.2976	0.1033	36	2.88	0.0067	0.05	0.08800	0.5071
BFU-E	AoE+PSCs	0ug/ml	CFU-GEMM	HE+PSCs	2.5ug/ml	1.1279	0.1249	36	9.03	<.0001	0.05	0.8747	1.3811
BFU-E	AoE+PSCs	0ug/ml	CFU-GEMM	HE+PSCs	7.5ug/ml	1.9828	0.1652	36	12.00	<.0001	0.05	1.6477	2.3179
BFU-E	AoE+PSCs	0ug/ml	CFU-GM	AoE+PSCs	0ug/ml	-1.4236	0.05920	36	-24.05	<.0001	0.05	-1.5436	-1.3035
BFU-E	AoE+PSCs	0ug/ml	CFU-GM	AoE+PSCs	2.5ug/ml	-0.6343	0.09156	36	-6.93	<.0001	0.05	-0.8200	-0.4486
BFU-E	AoE+PSCs	0ug/ml	CFU-GM	AoE+PSCs	7.5ug/ml	-0.05459	0.09775	36	-0.56	0.5800	0.05	-0.2528	0.1437
BFU-E	AoE+PSCs	0ug/ml	CFU-GM	E10.5AGM	0ug/ml	-1.8679	0.08556	36	-21.83	<.0001	0.05	-2.0414	-1.6944
BFU-E	AoE+PSCs	0ug/ml	CFU-GM	E10.5AGM	2.5ug/ml	-1.3044	0.08747	36	-14.91	<.0001	0.05	-1.4818	-1.1270
BFU-E	AoE+PSCs	0ug/ml	CFU-GM	E10.5AGM	7.5ug/ml	-0.6759	0.09123	36	-7.41	<.0001	0.05	-0.8609	-0.4908
BFU-E	AoE+PSCs	0ug/ml	CFU-GM	HE+PSCs	0ug/ml	-1.3356	0.08734	36	-15.29	<.0001	0.05	-1.5127	-1.1584

The GLIMMIX Procedure

Differences of Colony*Condit*Treatm Least Squares Means													
Colony_Type	Condition	Treatment	_Colony_Type	_Condition	_Treatment	Estimate	Standard Error	DF	t Value	Pr > |t|	Alpha	Lower	Upper
BFU-E	AoE+PSCs	0ug/ml	CFU-GM	HE+PSCs	2.5ug/ml	-0.5441	0.09232	36	-5.89	<.0001	0.05	-0.7314	-0.3569
BFU-E	AoE+PSCs	0ug/ml	CFU-GM	HE+PSCs	7.5ug/ml	0.1905	0.1015	36	1.88	0.0687	0.05	-0.01539	0.3963
BFU-E	AoE+PSCs	2.5ug/ml	BFU-E	AoE+PSCs	7.5ug/ml	1.1643	0.1823	36	6.39	<.0001	0.05	0.7945	1.5341
BFU-E	AoE+PSCs	2.5ug/ml	BFU-E	E10.5AGM	0ug/ml	-1.4196	0.1126	36	-12.60	<.0001	0.05	-1.6481	-1.1912
BFU-E	AoE+PSCs	2.5ug/ml	BFU-E	E10.5AGM	2.5ug/ml	-1.0590	0.1158	36	-9.14	<.0001	0.05	-1.2939	-0.8241
BFU-E	AoE+PSCs	2.5ug/ml	BFU-E	E10.5AGM	7.5ug/ml	-0.1865	0.1295	36	-1.44	0.1584	0.05	-0.4490	0.07606
BFU-E	AoE+PSCs	2.5ug/ml	BFU-E	HE+PSCs	0ug/ml	-0.6912	0.1203	36	-5.74	<.0001	0.05	-0.9352	-0.4471
BFU-E	AoE+PSCs	2.5ug/ml	BFU-E	HE+PSCs	2.5ug/ml	0.1113	0.1370	36	0.81	0.4219	0.05	-0.1665	0.3891
BFU-E	AoE+PSCs	2.5ug/ml	BFU-E	HE+PSCs	7.5ug/ml	1.2353	0.1866	36	6.62	<.0001	0.05	0.8569	1.6137
BFU-E	AoE+PSCs	2.5ug/ml	CFU-GEMM	AoE+PSCs	0ug/ml	-0.7555	0.1195	36	-6.32	<.0001	0.05	-0.9978	-0.5132
BFU-E	AoE+PSCs	2.5ug/ml	CFU-GEMM	AoE+PSCs	2.5ug/ml	-0.08619	0.1154	36	-0.75	0.4599	0.05	-0.3202	0.1478
BFU-E	AoE+PSCs	2.5ug/ml	CFU-GEMM	AoE+PSCs	7.5ug/ml	0.8280	0.1640	36	5.05	<.0001	0.05	0.4954	1.1607
BFU-E	AoE+PSCs	2.5ug/ml	CFU-GEMM	E10.5AGM	0ug/ml	-1.3111	0.1135	36	-11.55	<.0001	0.05	-1.5412	-1.0809
BFU-E	AoE+PSCs	2.5ug/ml	CFU-GEMM	E10.5AGM	2.5ug/ml	-1.0707	0.1157	36	-9.25	<.0001	0.05	-1.3053	-0.8360
BFU-E	AoE+PSCs	2.5ug/ml	CFU-GEMM	E10.5AGM	7.5ug/ml	-0.1331	0.1307	36	-1.02	0.3151	0.05	-0.3981	0.1319
BFU-E	AoE+PSCs	2.5ug/ml	CFU-GEMM	HE+PSCs	0ug/ml	-0.6039	0.1216	36	-4.96	<.0001	0.05	-0.8507	-0.3572
BFU-E	AoE+PSCs	2.5ug/ml	CFU-GEMM	HE+PSCs	2.5ug/ml	0.2264	0.1404	36	1.61	0.1156	0.05	-0.05834	0.5111
BFU-E	AoE+PSCs	2.5ug/ml	CFU-GEMM	HE+PSCs	7.5ug/ml	1.0813	0.1772	36	6.10	<.0001	0.05	0.7218	1.4408
BFU-E	AoE+PSCs	2.5ug/ml	CFU-GM	AoE+PSCs	0ug/ml	-2.3251	0.1081	36	-21.51	<.0001	0.05	-2.5443	-2.1058
BFU-E	AoE+PSCs	2.5ug/ml	CFU-GM	AoE+PSCs	2.5ug/ml	-1.5358	0.09185	36	-16.72	<.0001	0.05	-1.7221	-1.3496
BFU-E	AoE+PSCs	2.5ug/ml	CFU-GM	AoE+PSCs	7.5ug/ml	-0.9561	0.1169	36	-8.18	<.0001	0.05	-1.1933	-0.7189
BFU-E	AoE+PSCs	2.5ug/ml	CFU-GM	E10.5AGM	0ug/ml	-2.7694	0.1070	36	-25.89	<.0001	0.05	-2.9863	-2.5525
BFU-E	AoE+PSCs	2.5ug/ml	CFU-GM	E10.5AGM	2.5ug/ml	-2.2059	0.1085	36	-20.33	<.0001	0.05	-2.4259	-1.9858
BFU-E	AoE+PSCs	2.5ug/ml	CFU-GM	E10.5AGM	7.5ug/ml	-1.5774	0.1115	36	-14.14	<.0001	0.05	-1.8036	-1.3511

The GLIMMIX Procedure

Differences of Colony*Condit*Treatm Least Squares Means													
Colony_Type	Condition	Treatment	_Colony_Type	_Condition	_Treatment	Estimate	Standard Error	DF	t Value	Pr > |t|	Alpha	Lower	Upper
BFU-E	AoE+PSCs	2.5ug/ml	CFU-GM	HE+PSCs	0ug/ml	-2.2371	0.1084	36	-20.64	<.0001	0.05	-2.4569	-2.0173
BFU-E	AoE+PSCs	2.5ug/ml	CFU-GM	HE+PSCs	2.5ug/ml	-1.4457	0.1124	36	-12.86	<.0001	0.05	-1.6737	-1.2176
BFU-E	AoE+PSCs	2.5ug/ml	CFU-GM	HE+PSCs	7.5ug/ml	-0.7110	0.1201	36	-5.92	<.0001	0.05	-0.9546	-0.4675
BFU-E	AoE+PSCs	7.5ug/ml	BFU-E	E10.5AGM	0ug/ml	-2.5840	0.1673	36	-15.45	<.0001	0.05	-2.9232	-2.2447
BFU-E	AoE+PSCs	7.5ug/ml	BFU-E	E10.5AGM	2.5ug/ml	-2.2234	0.1695	36	-13.12	<.0001	0.05	-2.5670	-1.8797
BFU-E	AoE+PSCs	7.5ug/ml	BFU-E	E10.5AGM	7.5ug/ml	-1.3508	0.1790	36	-7.54	<.0001	0.05	-1.7140	-0.9877
BFU-E	AoE+PSCs	7.5ug/ml	BFU-E	HE+PSCs	0ug/ml	-1.8555	0.1726	36	-10.75	<.0001	0.05	-2.2055	-1.5055
BFU-E	AoE+PSCs	7.5ug/ml	BFU-E	HE+PSCs	2.5ug/ml	-1.0531	0.1846	36	-5.71	<.0001	0.05	-1.4274	-0.6788
BFU-E	AoE+PSCs	7.5ug/ml	BFU-E	HE+PSCs	7.5ug/ml	0.07093	0.2238	36	0.32	0.7532	0.05	-0.3831	0.5249
BFU-E	AoE+PSCs	7.5ug/ml	CFU-GEMM	AoE+PSCs	0ug/ml	-1.9199	0.1720	36	-11.16	<.0001	0.05	-2.2686	-1.5711
BFU-E	AoE+PSCs	7.5ug/ml	CFU-GEMM	AoE+PSCs	2.5ug/ml	-1.2505	0.1808	36	-6.92	<.0001	0.05	-1.6171	-0.8839
BFU-E	AoE+PSCs	7.5ug/ml	CFU-GEMM	AoE+PSCs	7.5ug/ml	-0.3363	0.1953	36	-1.72	0.0936	0.05	-0.7323	0.05974
BFU-E	AoE+PSCs	7.5ug/ml	CFU-GEMM	E10.5AGM	0ug/ml	-2.4754	0.1679	36	-14.75	<.0001	0.05	-2.8159	-2.1350
BFU-E	AoE+PSCs	7.5ug/ml	CFU-GEMM	E10.5AGM	2.5ug/ml	-2.2350	0.1694	36	-13.20	<.0001	0.05	-2.5785	-1.8915
BFU-E	AoE+PSCs	7.5ug/ml	CFU-GEMM	E10.5AGM	7.5ug/ml	-1.2975	0.1799	36	-7.21	<.0001	0.05	-1.6624	-0.9326
BFU-E	AoE+PSCs	7.5ug/ml	CFU-GEMM	HE+PSCs	0ug/ml	-1.7683	0.1735	36	-10.19	<.0001	0.05	-2.1201	-1.4164
BFU-E	AoE+PSCs	7.5ug/ml	CFU-GEMM	HE+PSCs	2.5ug/ml	-0.9379	0.1871	36	-5.01	<.0001	0.05	-1.3174	-0.5585
BFU-E	AoE+PSCs	7.5ug/ml	CFU-GEMM	HE+PSCs	7.5ug/ml	-0.08306	0.2161	36	-0.38	0.7030	0.05	-0.5214	0.3553
BFU-E	AoE+PSCs	7.5ug/ml	CFU-GM	AoE+PSCs	0ug/ml	-3.4894	0.1643	36	-21.24	<.0001	0.05	-3.8226	-3.1562
BFU-E	AoE+PSCs	7.5ug/ml	CFU-GM	AoE+PSCs	2.5ug/ml	-2.7002	0.1667	36	-16.19	<.0001	0.05	-3.0383	-2.3620
BFU-E	AoE+PSCs	7.5ug/ml	CFU-GM	AoE+PSCs	7.5ug/ml	-2.1204	0.1578	36	-13.44	<.0001	0.05	-2.4405	-1.8003
BFU-E	AoE+PSCs	7.5ug/ml	CFU-GM	E10.5AGM	0ug/ml	-3.9337	0.1635	36	-24.06	<.0001	0.05	-4.2654	-3.6021
BFU-E	AoE+PSCs	7.5ug/ml	CFU-GM	E10.5AGM	2.5ug/ml	-3.3702	0.1645	36	-20.48	<.0001	0.05	-3.7039	-3.0365
BFU-E	AoE+PSCs	7.5ug/ml	CFU-GM	E10.5AGM	7.5ug/ml	-2.7417	0.1666	36	-16.46	<.0001	0.05	-3.0795	-2.4039

The GLIMMIX Procedure

Differences of Colony*Condit*Treatm Least Squares Means													
Colony_Type	Condition	Treatment	_Colony_Type	_Condition	_Treatment	Estimate	Standard Error	DF	t Value	Pr > |t|	Alpha	Lower	Upper
BFU-E	AoE+PSCs	7.5ug/ml	CFU-GM	HE+PSCs	0ug/ml	-3.4014	0.1645	36	-20.68	<.0001	0.05	-3.7350	-3.0679
BFU-E	AoE+PSCs	7.5ug/ml	CFU-GM	HE+PSCs	2.5ug/ml	-2.6100	0.1672	36	-15.61	<.0001	0.05	-2.9490	-2.2710
BFU-E	AoE+PSCs	7.5ug/ml	CFU-GM	HE+PSCs	7.5ug/ml	-1.8754	0.1724	36	-10.88	<.0001	0.05	-2.2250	-1.5257
BFU-E	E10.5AGM	0ug/ml	BFU-E	E10.5AGM	2.5ug/ml	0.3606	0.09028	36	3.99	0.0003	0.05	0.1775	0.5437
BFU-E	E10.5AGM	0ug/ml	BFU-E	E10.5AGM	7.5ug/ml	1.2331	0.1072	36	11.50	<.0001	0.05	1.0157	1.4506
BFU-E	E10.5AGM	0ug/ml	BFU-E	HE+PSCs	0ug/ml	0.7284	0.09601	36	7.59	<.0001	0.05	0.5337	0.9231
BFU-E	E10.5AGM	0ug/ml	BFU-E	HE+PSCs	2.5ug/ml	1.5309	0.1162	36	13.18	<.0001	0.05	1.2953	1.7666
BFU-E	E10.5AGM	0ug/ml	BFU-E	HE+PSCs	7.5ug/ml	2.6549	0.1719	36	15.44	<.0001	0.05	2.3063	3.0035
BFU-E	E10.5AGM	0ug/ml	CFU-GEMM	AoE+PSCs	0ug/ml	0.6641	0.09492	36	7.00	<.0001	0.05	0.4716	0.8566
BFU-E	E10.5AGM	0ug/ml	CFU-GEMM	AoE+PSCs	2.5ug/ml	1.3334	0.1101	36	12.12	<.0001	0.05	1.1102	1.5567
BFU-E	E10.5AGM	0ug/ml	CFU-GEMM	AoE+PSCs	7.5ug/ml	2.2477	0.1471	36	15.28	<.0001	0.05	1.9494	2.5460
BFU-E	E10.5AGM	0ug/ml	CFU-GEMM	E10.5AGM	0ug/ml	0.1085	0.05961	36	1.82	0.0769	0.05	-0.01235	0.2294
BFU-E	E10.5AGM	0ug/ml	CFU-GEMM	E10.5AGM	2.5ug/ml	0.3490	0.09013	36	3.87	0.0004	0.05	0.1662	0.5318
BFU-E	E10.5AGM	0ug/ml	CFU-GEMM	E10.5AGM	7.5ug/ml	1.2865	0.1087	36	11.84	<.0001	0.05	1.0661	1.5069
BFU-E	E10.5AGM	0ug/ml	CFU-GEMM	HE+PSCs	0ug/ml	0.8157	0.09764	36	8.35	<.0001	0.05	0.6177	1.0137
BFU-E	E10.5AGM	0ug/ml	CFU-GEMM	HE+PSCs	2.5ug/ml	1.6460	0.1202	36	13.70	<.0001	0.05	1.4023	1.8898
BFU-E	E10.5AGM	0ug/ml	CFU-GEMM	HE+PSCs	7.5ug/ml	2.5009	0.1617	36	15.46	<.0001	0.05	2.1729	2.8289
BFU-E	E10.5AGM	0ug/ml	CFU-GM	AoE+PSCs	0ug/ml	-0.9054	0.08013	36	-11.30	<.0001	0.05	-1.0680	-0.7429
BFU-E	E10.5AGM	0ug/ml	CFU-GM	AoE+PSCs	2.5ug/ml	-0.1162	0.08508	36	-1.37	0.1805	0.05	-0.2888	0.05635
BFU-E	E10.5AGM	0ug/ml	CFU-GM	AoE+PSCs	7.5ug/ml	0.4635	0.09172	36	5.05	<.0001	0.05	0.2775	0.6495
BFU-E	E10.5AGM	0ug/ml	CFU-GM	E10.5AGM	0ug/ml	-1.3498	0.04600	36	-29.34	<.0001	0.05	-1.4431	-1.2565
BFU-E	E10.5AGM	0ug/ml	CFU-GM	E10.5AGM	2.5ug/ml	-0.7863	0.08067	36	-9.75	<.0001	0.05	-0.9499	-0.6227
BFU-E	E10.5AGM	0ug/ml	CFU-GM	E10.5AGM	7.5ug/ml	-0.1577	0.08472	36	-1.86	0.0708	0.05	-0.3296	0.01409
BFU-E	E10.5AGM	0ug/ml	CFU-GM	HE+PSCs	0ug/ml	-0.8175	0.08053	36	-10.15	<.0001	0.05	-0.9808	-0.6541

The GLIMMIX Procedure

Differences of Colony*Condit*Treatm Least Squares Means													
Colony_Type	Condition	Treatment	_Colony_Type	_Condition	_Treatment	Estimate	Standard Error	DF	t Value	Pr > |t|	Alpha	Lower	Upper
BFU-E	E10.5AGM	0ug/ml	CFU-GM	HE+PSCs	2.5ug/ml	-0.02603	0.08590	36	-0.30	0.7636	0.05	-0.2002	0.1482
BFU-E	E10.5AGM	0ug/ml	CFU-GM	HE+PSCs	7.5ug/ml	0.7086	0.09570	36	7.40	<.0001	0.05	0.5145	0.9027
BFU-E	E10.5AGM	2.5ug/ml	BFU-E	E10.5AGM	7.5ug/ml	0.8725	0.1106	36	7.89	<.0001	0.05	0.6483	1.0968
BFU-E	E10.5AGM	2.5ug/ml	BFU-E	HE+PSCs	0ug/ml	0.3678	0.09973	36	3.69	0.0007	0.05	0.1656	0.5701
BFU-E	E10.5AGM	2.5ug/ml	BFU-E	HE+PSCs	2.5ug/ml	1.1703	0.1193	36	9.81	<.0001	0.05	0.9284	1.4122
BFU-E	E10.5AGM	2.5ug/ml	BFU-E	HE+PSCs	7.5ug/ml	2.2943	0.1740	36	13.19	<.0001	0.05	1.9414	2.6472
BFU-E	E10.5AGM	2.5ug/ml	CFU-GEMM	AoE+PSCs	0ug/ml	0.3035	0.09868	36	3.08	0.0040	0.05	0.1034	0.5036
BFU-E	E10.5AGM	2.5ug/ml	CFU-GEMM	AoE+PSCs	2.5ug/ml	0.9728	0.1133	36	8.58	<.0001	0.05	0.7430	1.2027
BFU-E	E10.5AGM	2.5ug/ml	CFU-GEMM	AoE+PSCs	7.5ug/ml	1.8871	0.1495	36	12.62	<.0001	0.05	1.5838	2.1904
BFU-E	E10.5AGM	2.5ug/ml	CFU-GEMM	E10.5AGM	0ug/ml	-0.2521	0.09134	36	-2.76	0.0090	0.05	-0.4373	-0.06679
BFU-E	E10.5AGM	2.5ug/ml	CFU-GEMM	E10.5AGM	2.5ug/ml	-0.01163	0.06921	36	-0.17	0.8675	0.05	-0.1520	0.1287
BFU-E	E10.5AGM	2.5ug/ml	CFU-GEMM	E10.5AGM	7.5ug/ml	0.9259	0.1120	36	8.27	<.0001	0.05	0.6988	1.1530
BFU-E	E10.5AGM	2.5ug/ml	CFU-GEMM	HE+PSCs	0ug/ml	0.4551	0.1013	36	4.49	<.0001	0.05	0.2496	0.6605
BFU-E	E10.5AGM	2.5ug/ml	CFU-GEMM	HE+PSCs	2.5ug/ml	1.2854	0.1232	36	10.43	<.0001	0.05	1.0356	1.5353
BFU-E	E10.5AGM	2.5ug/ml	CFU-GEMM	HE+PSCs	7.5ug/ml	2.1403	0.1640	36	13.05	<.0001	0.05	1.8078	2.4728
BFU-E	E10.5AGM	2.5ug/ml	CFU-GM	AoE+PSCs	0ug/ml	-1.2660	0.08456	36	-14.97	<.0001	0.05	-1.4375	-1.0945
BFU-E	E10.5AGM	2.5ug/ml	CFU-GM	AoE+PSCs	2.5ug/ml	-0.4768	0.08926	36	-5.34	<.0001	0.05	-0.6578	-0.2958
BFU-E	E10.5AGM	2.5ug/ml	CFU-GM	AoE+PSCs	7.5ug/ml	0.1029	0.09561	36	1.08	0.2888	0.05	-0.09098	0.2968
BFU-E	E10.5AGM	2.5ug/ml	CFU-GM	E10.5AGM	0ug/ml	-1.7104	0.08311	36	-20.58	<.0001	0.05	-1.8789	-1.5418
BFU-E	E10.5AGM	2.5ug/ml	CFU-GM	E10.5AGM	2.5ug/ml	-1.1469	0.05634	36	-20.36	<.0001	0.05	-1.2611	-1.0326
BFU-E	E10.5AGM	2.5ug/ml	CFU-GM	E10.5AGM	7.5ug/ml	-0.5183	0.08892	36	-5.83	<.0001	0.05	-0.6987	-0.3380
BFU-E	E10.5AGM	2.5ug/ml	CFU-GM	HE+PSCs	0ug/ml	-1.1781	0.08493	36	-13.87	<.0001	0.05	-1.3503	-1.0058
BFU-E	E10.5AGM	2.5ug/ml	CFU-GM	HE+PSCs	2.5ug/ml	-0.3866	0.09004	36	-4.29	0.0001	0.05	-0.5692	-0.2040
BFU-E	E10.5AGM	2.5ug/ml	CFU-GM	HE+PSCs	7.5ug/ml	0.3480	0.09944	36	3.50	0.0013	0.05	0.1463	0.5497

The GLIMMIX Procedure

Differences of Colony*Condit*Treatm Least Squares Means													
Colony_Type	Condition	Treatment	_Colony_Type	_Condition	_Treatment	Estimate	Standard Error	DF	t Value	Pr > |t|	Alpha	Lower	Upper
BFU-E	E10.5AGM	7.5ug/ml	BFU-E	HE+PSCs	0ug/ml	-0.5047	0.1153	36	-4.38	<.0001	0.05	-0.7385	-0.2709
BFU-E	E10.5AGM	7.5ug/ml	BFU-E	HE+PSCs	2.5ug/ml	0.2978	0.1326	36	2.25	0.0309	0.05	0.02893	0.5666
BFU-E	E10.5AGM	7.5ug/ml	BFU-E	HE+PSCs	7.5ug/ml	1.4218	0.1833	36	7.75	<.0001	0.05	1.0499	1.7936
BFU-E	E10.5AGM	7.5ug/ml	CFU-GEMM	AoE+PSCs	0ug/ml	-0.5690	0.1144	36	-4.97	<.0001	0.05	-0.8010	-0.3371
BFU-E	E10.5AGM	7.5ug/ml	CFU-GEMM	AoE+PSCs	2.5ug/ml	0.1003	0.1272	36	0.79	0.4356	0.05	-0.1577	0.3583
BFU-E	E10.5AGM	7.5ug/ml	CFU-GEMM	AoE+PSCs	7.5ug/ml	1.0145	0.1603	36	6.33	<.0001	0.05	0.6894	1.3397
BFU-E	E10.5AGM	7.5ug/ml	CFU-GEMM	E10.5AGM	0ug/ml	-1.1246	0.1081	36	-10.40	<.0001	0.05	-1.3439	-0.9053
BFU-E	E10.5AGM	7.5ug/ml	CFU-GEMM	E10.5AGM	2.5ug/ml	-0.8842	0.1104	36	-8.01	<.0001	0.05	-1.1082	-0.6602
BFU-E	E10.5AGM	7.5ug/ml	CFU-GEMM	E10.5AGM	7.5ug/ml	0.05336	0.1087	36	0.49	0.6264	0.05	-0.1670	0.2738
BFU-E	E10.5AGM	7.5ug/ml	CFU-GEMM	HE+PSCs	0ug/ml	-0.4175	0.1166	36	-3.58	0.0010	0.05	-0.6540	-0.1809
BFU-E	E10.5AGM	7.5ug/ml	CFU-GEMM	HE+PSCs	2.5ug/ml	0.4129	0.1361	36	3.03	0.0045	0.05	0.1369	0.6889
BFU-E	E10.5AGM	7.5ug/ml	CFU-GEMM	HE+PSCs	7.5ug/ml	1.2678	0.1738	36	7.29	<.0001	0.05	0.9152	1.6203
BFU-E	E10.5AGM	7.5ug/ml	CFU-GM	AoE+PSCs	0ug/ml	-2.1386	0.1024	36	-20.87	<.0001	0.05	-2.3463	-1.9308
BFU-E	E10.5AGM	7.5ug/ml	CFU-GM	AoE+PSCs	2.5ug/ml	-1.3493	0.1064	36	-12.69	<.0001	0.05	-1.5650	-1.1336
BFU-E	E10.5AGM	7.5ug/ml	CFU-GM	AoE+PSCs	7.5ug/ml	-0.7696	0.1117	36	-6.89	<.0001	0.05	-0.9962	-0.5430
BFU-E	E10.5AGM	7.5ug/ml	CFU-GM	E10.5AGM	0ug/ml	-2.5829	0.1012	36	-25.51	<.0001	0.05	-2.7883	-2.3776
BFU-E	E10.5AGM	7.5ug/ml	CFU-GM	E10.5AGM	2.5ug/ml	-2.0194	0.1029	36	-19.63	<.0001	0.05	-2.2280	-1.8108
BFU-E	E10.5AGM	7.5ug/ml	CFU-GM	E10.5AGM	7.5ug/ml	-1.3909	0.08472	36	-16.42	<.0001	0.05	-1.5627	-1.2190
BFU-E	E10.5AGM	7.5ug/ml	CFU-GM	HE+PSCs	0ug/ml	-2.0506	0.1027	36	-19.96	<.0001	0.05	-2.2590	-1.8422
BFU-E	E10.5AGM	7.5ug/ml	CFU-GM	HE+PSCs	2.5ug/ml	-1.2592	0.1070	36	-11.77	<.0001	0.05	-1.4762	-1.0421
BFU-E	E10.5AGM	7.5ug/ml	CFU-GM	HE+PSCs	7.5ug/ml	-0.5245	0.1150	36	-4.56	<.0001	0.05	-0.7578	-0.2913
BFU-E	HE+PSCs	0ug/ml	BFU-E	HE+PSCs	2.5ug/ml	0.8025	0.1237	36	6.49	<.0001	0.05	0.5517	1.0533
BFU-E	HE+PSCs	0ug/ml	BFU-E	HE+PSCs	7.5ug/ml	1.9265	0.1770	36	10.88	<.0001	0.05	1.5674	2.2855
BFU-E	HE+PSCs	0ug/ml	CFU-GEMM	AoE+PSCs	0ug/ml	-0.06434	0.1039	36	-0.62	0.5398	0.05	-0.2752	0.1465

The GLIMMIX Procedure

Differences of Colony*Condit*Treatm Least Squares Means													
Colony_Type	Condition	Treatment	_Colony_Type	_Condition	_Treatment	Estimate	Standard Error	DF	t Value	Pr > |t|	Alpha	Lower	Upper
BFU-E	HE+PSCs	0ug/ml	CFU-GEMM	AoE+PSCs	2.5ug/ml	0.6050	0.1179	36	5.13	<.0001	0.05	0.3658	0.8442
BFU-E	HE+PSCs	0ug/ml	CFU-GEMM	AoE+PSCs	7.5ug/ml	1.5192	0.1531	36	9.93	<.0001	0.05	1.2088	1.8297
BFU-E	HE+PSCs	0ug/ml	CFU-GEMM	E10.5AGM	0ug/ml	-0.6199	0.09700	36	-6.39	<.0001	0.05	-0.8166	-0.4232
BFU-E	HE+PSCs	0ug/ml	CFU-GEMM	E10.5AGM	2.5ug/ml	-0.3795	0.09959	36	-3.81	0.0005	0.05	-0.5815	-0.1775
BFU-E	HE+PSCs	0ug/ml	CFU-GEMM	E10.5AGM	7.5ug/ml	0.5581	0.1166	36	4.78	<.0001	0.05	0.3215	0.7946
BFU-E	HE+PSCs	0ug/ml	CFU-GEMM	HE+PSCs	0ug/ml	0.08725	0.08522	36	1.02	0.3128	0.05	-0.08558	0.2601
BFU-E	HE+PSCs	0ug/ml	CFU-GEMM	HE+PSCs	2.5ug/ml	0.9176	0.1274	36	7.20	<.0001	0.05	0.6591	1.1760
BFU-E	HE+PSCs	0ug/ml	CFU-GEMM	HE+PSCs	7.5ug/ml	1.7725	0.1672	36	10.60	<.0001	0.05	1.4334	2.1115
BFU-E	HE+PSCs	0ug/ml	CFU-GM	AoE+PSCs	0ug/ml	-1.6339	0.09065	36	-18.02	<.0001	0.05	-1.8177	-1.4500
BFU-E	HE+PSCs	0ug/ml	CFU-GM	AoE+PSCs	2.5ug/ml	-0.8446	0.09505	36	-8.89	<.0001	0.05	-1.0374	-0.6519
BFU-E	HE+PSCs	0ug/ml	CFU-GM	AoE+PSCs	7.5ug/ml	-0.2649	0.1010	36	-2.62	0.0127	0.05	-0.4698	-0.06002
BFU-E	HE+PSCs	0ug/ml	CFU-GM	E10.5AGM	0ug/ml	-2.0782	0.08929	36	-23.28	<.0001	0.05	-2.2593	-1.8971
BFU-E	HE+PSCs	0ug/ml	CFU-GM	E10.5AGM	2.5ug/ml	-1.5147	0.09112	36	-16.62	<.0001	0.05	-1.6995	-1.3299
BFU-E	HE+PSCs	0ug/ml	CFU-GM	E10.5AGM	7.5ug/ml	-0.8862	0.09472	36	-9.36	<.0001	0.05	-1.0783	-0.6941
BFU-E	HE+PSCs	0ug/ml	CFU-GM	HE+PSCs	0ug/ml	-1.5459	0.06491	36	-23.82	<.0001	0.05	-1.6775	-1.4143
BFU-E	HE+PSCs	0ug/ml	CFU-GM	HE+PSCs	2.5ug/ml	-0.7545	0.09577	36	-7.88	<.0001	0.05	-0.9487	-0.5602
BFU-E	HE+PSCs	0ug/ml	CFU-GM	HE+PSCs	7.5ug/ml	-0.01985	0.1046	36	-0.19	0.8506	0.05	-0.2321	0.1924
BFU-E	HE+PSCs	2.5ug/ml	BFU-E	HE+PSCs	7.5ug/ml	1.1240	0.1887	36	5.96	<.0001	0.05	0.7412	1.5068
BFU-E	HE+PSCs	2.5ug/ml	CFU-GEMM	AoE+PSCs	0ug/ml	-0.8668	0.1228	36	-7.06	<.0001	0.05	-1.1159	-0.6177
BFU-E	HE+PSCs	2.5ug/ml	CFU-GEMM	AoE+PSCs	2.5ug/ml	-0.1975	0.1349	36	-1.46	0.1518	0.05	-0.4710	0.07607
BFU-E	HE+PSCs	2.5ug/ml	CFU-GEMM	AoE+PSCs	7.5ug/ml	0.7168	0.1665	36	4.31	0.0001	0.05	0.3792	1.0544
BFU-E	HE+PSCs	2.5ug/ml	CFU-GEMM	E10.5AGM	0ug/ml	-1.4224	0.1170	36	-12.16	<.0001	0.05	-1.6597	-1.1850
BFU-E	HE+PSCs	2.5ug/ml	CFU-GEMM	E10.5AGM	2.5ug/ml	-1.1819	0.1192	36	-9.92	<.0001	0.05	-1.4236	-0.9403
BFU-E	HE+PSCs	2.5ug/ml	CFU-GEMM	E10.5AGM	7.5ug/ml	-0.2444	0.1337	36	-1.83	0.0759	0.05	-0.5157	0.02683

The GLIMMIX Procedure

Differences of Colony*Condit*Treatm Least Squares Means													
Colony_Type	Condition	Treatment	_Colony_Type	_Condition	_Treatment	Estimate	Standard Error	DF	t Value	Pr > |t|	Alpha	Lower	Upper
BFU-E	HE+PSCs	2.5ug/ml	CFU-GEMM	HE+PSCs	0ug/ml	-0.7152	0.1249	36	-5.72	<.0001	0.05	-0.9686	-0.4618
BFU-E	HE+PSCs	2.5ug/ml	CFU-GEMM	HE+PSCs	2.5ug/ml	0.1151	0.1283	36	0.90	0.3755	0.05	-0.1451	0.3753
BFU-E	HE+PSCs	2.5ug/ml	CFU-GEMM	HE+PSCs	7.5ug/ml	0.9700	0.1795	36	5.40	<.0001	0.05	0.6059	1.3341
BFU-E	HE+PSCs	2.5ug/ml	CFU-GM	AoE+PSCs	0ug/ml	-2.4363	0.1118	36	-21.79	<.0001	0.05	-2.6631	-2.2096
BFU-E	HE+PSCs	2.5ug/ml	CFU-GM	AoE+PSCs	2.5ug/ml	-1.6471	0.1154	36	-14.27	<.0001	0.05	-1.8812	-1.4131
BFU-E	HE+PSCs	2.5ug/ml	CFU-GM	AoE+PSCs	7.5ug/ml	-1.0674	0.1204	36	-8.87	<.0001	0.05	-1.3115	-0.8233
BFU-E	HE+PSCs	2.5ug/ml	CFU-GM	E10.5AGM	0ug/ml	-2.8807	0.1107	36	-26.02	<.0001	0.05	-3.1052	-2.6562
BFU-E	HE+PSCs	2.5ug/ml	CFU-GM	E10.5AGM	2.5ug/ml	-2.3172	0.1122	36	-20.66	<.0001	0.05	-2.5447	-2.0897
BFU-E	HE+PSCs	2.5ug/ml	CFU-GM	E10.5AGM	7.5ug/ml	-1.6887	0.1151	36	-14.67	<.0001	0.05	-1.9221	-1.4552
BFU-E	HE+PSCs	2.5ug/ml	CFU-GM	HE+PSCs	0ug/ml	-2.3484	0.1121	36	-20.95	<.0001	0.05	-2.5757	-2.1211
BFU-E	HE+PSCs	2.5ug/ml	CFU-GM	HE+PSCs	2.5ug/ml	-1.5569	0.09691	36	-16.07	<.0001	0.05	-1.7535	-1.3604
BFU-E	HE+PSCs	2.5ug/ml	CFU-GM	HE+PSCs	7.5ug/ml	-0.8223	0.1234	36	-6.66	<.0001	0.05	-1.0726	-0.5720
BFU-E	HE+PSCs	7.5ug/ml	CFU-GEMM	AoE+PSCs	0ug/ml	-1.9908	0.1765	36	-11.28	<.0001	0.05	-2.3487	-1.6329
BFU-E	HE+PSCs	7.5ug/ml	CFU-GEMM	AoE+PSCs	2.5ug/ml	-1.3214	0.1850	36	-7.14	<.0001	0.05	-1.6967	-0.9462
BFU-E	HE+PSCs	7.5ug/ml	CFU-GEMM	AoE+PSCs	7.5ug/ml	-0.4072	0.2092	36	-1.95	0.0594	0.05	-0.8315	0.01702
BFU-E	HE+PSCs	7.5ug/ml	CFU-GEMM	E10.5AGM	0ug/ml	-2.5463	0.1725	36	-14.77	<.0001	0.05	-2.8961	-2.1966
BFU-E	HE+PSCs	7.5ug/ml	CFU-GEMM	E10.5AGM	2.5ug/ml	-2.3059	0.1739	36	-13.26	<.0001	0.05	-2.6587	-1.9532
BFU-E	HE+PSCs	7.5ug/ml	CFU-GEMM	E10.5AGM	7.5ug/ml	-1.3684	0.1842	36	-7.43	<.0001	0.05	-1.7420	-0.9948
BFU-E	HE+PSCs	7.5ug/ml	CFU-GEMM	HE+PSCs	0ug/ml	-1.8392	0.1779	36	-10.34	<.0001	0.05	-2.2001	-1.4784
BFU-E	HE+PSCs	7.5ug/ml	CFU-GEMM	HE+PSCs	2.5ug/ml	-1.0089	0.1912	36	-5.28	<.0001	0.05	-1.3967	-0.6210
BFU-E	HE+PSCs	7.5ug/ml	CFU-GEMM	HE+PSCs	7.5ug/ml	-0.1540	0.2102	36	-0.73	0.4685	0.05	-0.5803	0.2723
BFU-E	HE+PSCs	7.5ug/ml	CFU-GM	AoE+PSCs	0ug/ml	-3.5603	0.1690	36	-21.07	<.0001	0.05	-3.9030	-3.2176
BFU-E	HE+PSCs	7.5ug/ml	CFU-GM	AoE+PSCs	2.5ug/ml	-2.7711	0.1714	36	-16.17	<.0001	0.05	-3.1186	-2.4236
BFU-E	HE+PSCs	7.5ug/ml	CFU-GM	AoE+PSCs	7.5ug/ml	-2.1914	0.1747	36	-12.54	<.0001	0.05	-2.5458	-1.8370

The GLIMMIX Procedure

Differences of Colony*Condit*Treatm Least Squares Means													
Colony_Type	Condition	Treatment	_Colony_Type	_Condition	_Treatment	Estimate	Standard Error	DF	t Value	Pr > |t|	Alpha	Lower	Upper
BFU-E	HE+PSCs	7.5ug/ml	CFU-GM	E10.5AGM	0ug/ml	-4.0047	0.1682	36	-23.80	<.0001	0.05	-4.3459	-3.6635
BFU-E	HE+PSCs	7.5ug/ml	CFU-GM	E10.5AGM	2.5ug/ml	-3.4412	0.1692	36	-20.34	<.0001	0.05	-3.7843	-3.0980
BFU-E	HE+PSCs	7.5ug/ml	CFU-GM	E10.5AGM	7.5ug/ml	-2.8126	0.1712	36	-16.43	<.0001	0.05	-3.1598	-2.4655
BFU-E	HE+PSCs	7.5ug/ml	CFU-GM	HE+PSCs	0ug/ml	-3.4724	0.1691	36	-20.53	<.0001	0.05	-3.8154	-3.1293
BFU-E	HE+PSCs	7.5ug/ml	CFU-GM	HE+PSCs	2.5ug/ml	-2.6809	0.1718	36	-15.61	<.0001	0.05	-3.0293	-2.3326
BFU-E	HE+PSCs	7.5ug/ml	CFU-GM	HE+PSCs	7.5ug/ml	-1.9463	0.1649	36	-11.80	<.0001	0.05	-2.2807	-1.6119
CFU-GEMM	AoE+PSCs	0ug/ml	CFU-GEMM	AoE+PSCs	2.5ug/ml	0.6693	0.1171	36	5.72	<.0001	0.05	0.4320	0.9067
CFU-GEMM	AoE+PSCs	0ug/ml	CFU-GEMM	AoE+PSCs	7.5ug/ml	1.5836	0.1524	36	10.39	<.0001	0.05	1.2745	1.8926
CFU-GEMM	AoE+PSCs	0ug/ml	CFU-GEMM	E10.5AGM	0ug/ml	-0.5556	0.09593	36	-5.79	<.0001	0.05	-0.7501	-0.3610
CFU-GEMM	AoE+PSCs	0ug/ml	CFU-GEMM	E10.5AGM	2.5ug/ml	-0.3151	0.09854	36	-3.20	0.0029	0.05	-0.5150	-0.1153
CFU-GEMM	AoE+PSCs	0ug/ml	CFU-GEMM	E10.5AGM	7.5ug/ml	0.6224	0.1158	36	5.38	<.0001	0.05	0.3876	0.8572
CFU-GEMM	AoE+PSCs	0ug/ml	CFU-GEMM	HE+PSCs	0ug/ml	0.1516	0.1055	36	1.44	0.1592	0.05	-0.06229	0.3655
CFU-GEMM	AoE+PSCs	0ug/ml	CFU-GEMM	HE+PSCs	2.5ug/ml	0.9819	0.1266	36	7.75	<.0001	0.05	0.7251	1.2387
CFU-GEMM	AoE+PSCs	0ug/ml	CFU-GEMM	HE+PSCs	7.5ug/ml	1.8368	0.1666	36	11.03	<.0001	0.05	1.4990	2.1746
CFU-GEMM	AoE+PSCs	0ug/ml	CFU-GM	AoE+PSCs	0ug/ml	-1.5695	0.06284	36	-24.98	<.0001	0.05	-1.6970	-1.4421
CFU-GEMM	AoE+PSCs	0ug/ml	CFU-GM	AoE+PSCs	2.5ug/ml	-0.7803	0.09395	36	-8.31	<.0001	0.05	-0.9708	-0.5898
CFU-GEMM	AoE+PSCs	0ug/ml	CFU-GM	AoE+PSCs	7.5ug/ml	-0.2006	0.10000	36	-2.01	0.0524	0.05	-0.4034	0.002231
CFU-GEMM	AoE+PSCs	0ug/ml	CFU-GM	E10.5AGM	0ug/ml	-2.0139	0.08812	36	-22.85	<.0001	0.05	-2.1926	-1.8352
CFU-GEMM	AoE+PSCs	0ug/ml	CFU-GM	E10.5AGM	2.5ug/ml	-1.4504	0.08997	36	-16.12	<.0001	0.05	-1.6328	-1.2679
CFU-GEMM	AoE+PSCs	0ug/ml	CFU-GM	E10.5AGM	7.5ug/ml	-0.8218	0.09363	36	-8.78	<.0001	0.05	-1.0117	-0.6320
CFU-GEMM	AoE+PSCs	0ug/ml	CFU-GM	HE+PSCs	0ug/ml	-1.4816	0.08985	36	-16.49	<.0001	0.05	-1.6638	-1.2993
CFU-GEMM	AoE+PSCs	0ug/ml	CFU-GM	HE+PSCs	2.5ug/ml	-0.6901	0.09469	36	-7.29	<.0001	0.05	-0.8822	-0.4981
CFU-GEMM	AoE+PSCs	0ug/ml	CFU-GM	HE+PSCs	7.5ug/ml	0.04449	0.1037	36	0.43	0.6704	0.05	-0.1658	0.2547
CFU-GEMM	AoE+PSCs	2.5ug/ml	CFU-GEMM	AoE+PSCs	7.5ug/ml	0.9142	0.1622	36	5.63	<.0001	0.05	0.5852	1.2433

The GLIMMIX Procedure

Differences of Colony*Condit*Treatm Least Squares Means													
Colony_Type	Condition	Treatment	_Colony_Type	_Condition	_Treatment	Estimate	Standard Error	DF	t Value	Pr > |t|	Alpha	Lower	Upper
CFU-GEMM	AoE+PSCs	2.5ug/ml	CFU-GEMM	E10.5AGM	0ug/ml	-1.2249	0.1109	36	-11.04	<.0001	0.05	-1.4499	-0.9999
CFU-GEMM	AoE+PSCs	2.5ug/ml	CFU-GEMM	E10.5AGM	2.5ug/ml	-0.9845	0.1132	36	-8.70	<.0001	0.05	-1.2141	-0.7549
CFU-GEMM	AoE+PSCs	2.5ug/ml	CFU-GEMM	E10.5AGM	7.5ug/ml	-0.04694	0.1285	36	-0.37	0.7169	0.05	-0.3075	0.2136
CFU-GEMM	AoE+PSCs	2.5ug/ml	CFU-GEMM	HE+PSCs	0ug/ml	-0.5178	0.1193	36	-4.34	0.0001	0.05	-0.7596	-0.2759
CFU-GEMM	AoE+PSCs	2.5ug/ml	CFU-GEMM	HE+PSCs	2.5ug/ml	0.3126	0.1383	36	2.26	0.0300	0.05	0.03202	0.5931
CFU-GEMM	AoE+PSCs	2.5ug/ml	CFU-GEMM	HE+PSCs	7.5ug/ml	1.1675	0.1756	36	6.65	<.0001	0.05	0.8113	1.5236
CFU-GEMM	AoE+PSCs	2.5ug/ml	CFU-GM	AoE+PSCs	0ug/ml	-2.2389	0.1054	36	-21.24	<.0001	0.05	-2.4527	-2.0251
CFU-GEMM	AoE+PSCs	2.5ug/ml	CFU-GM	AoE+PSCs	2.5ug/ml	-1.4497	0.08867	36	-16.35	<.0001	0.05	-1.6295	-1.2698
CFU-GEMM	AoE+PSCs	2.5ug/ml	CFU-GM	AoE+PSCs	7.5ug/ml	-0.8699	0.1145	36	-7.60	<.0001	0.05	-1.1021	-0.6378
CFU-GEMM	AoE+PSCs	2.5ug/ml	CFU-GM	E10.5AGM	0ug/ml	-2.6832	0.1043	36	-25.74	<.0001	0.05	-2.8947	-2.4718
CFU-GEMM	AoE+PSCs	2.5ug/ml	CFU-GM	E10.5AGM	2.5ug/ml	-2.1197	0.1058	36	-20.03	<.0001	0.05	-2.3343	-1.9051
CFU-GEMM	AoE+PSCs	2.5ug/ml	CFU-GM	E10.5AGM	7.5ug/ml	-1.4912	0.1089	36	-13.69	<.0001	0.05	-1.7121	-1.2702
CFU-GEMM	AoE+PSCs	2.5ug/ml	CFU-GM	HE+PSCs	0ug/ml	-2.1509	0.1057	36	-20.35	<.0001	0.05	-2.3653	-1.9365
CFU-GEMM	AoE+PSCs	2.5ug/ml	CFU-GM	HE+PSCs	2.5ug/ml	-1.3595	0.1099	36	-12.37	<.0001	0.05	-1.5823	-1.1367
CFU-GEMM	AoE+PSCs	2.5ug/ml	CFU-GM	HE+PSCs	7.5ug/ml	-0.6249	0.1177	36	-5.31	<.0001	0.05	-0.8635	-0.3862
CFU-GEMM	AoE+PSCs	7.5ug/ml	CFU-GEMM	E10.5AGM	0ug/ml	-2.1391	0.1477	36	-14.48	<.0001	0.05	-2.4387	-1.8395
CFU-GEMM	AoE+PSCs	7.5ug/ml	CFU-GEMM	E10.5AGM	2.5ug/ml	-1.8987	0.1494	36	-12.70	<.0001	0.05	-2.2018	-1.5956
CFU-GEMM	AoE+PSCs	7.5ug/ml	CFU-GEMM	E10.5AGM	7.5ug/ml	-0.9612	0.1613	36	-5.96	<.0001	0.05	-1.2883	-0.6340
CFU-GEMM	AoE+PSCs	7.5ug/ml	CFU-GEMM	HE+PSCs	0ug/ml	-1.4320	0.1541	36	-9.29	<.0001	0.05	-1.7445	-1.1195
CFU-GEMM	AoE+PSCs	7.5ug/ml	CFU-GEMM	HE+PSCs	2.5ug/ml	-0.6017	0.1693	36	-3.55	0.0011	0.05	-0.9450	-0.2583
CFU-GEMM	AoE+PSCs	7.5ug/ml	CFU-GEMM	HE+PSCs	7.5ug/ml	0.2532	0.2009	36	1.26	0.2156	0.05	-0.1542	0.6607
CFU-GEMM	AoE+PSCs	7.5ug/ml	CFU-GM	AoE+PSCs	0ug/ml	-3.1531	0.1436	36	-21.95	<.0001	0.05	-3.4444	-2.8618
CFU-GEMM	AoE+PSCs	7.5ug/ml	CFU-GM	AoE+PSCs	2.5ug/ml	-2.3639	0.1465	36	-16.14	<.0001	0.05	-2.6609	-2.0669
CFU-GEMM	AoE+PSCs	7.5ug/ml	CFU-GM	AoE+PSCs	7.5ug/ml	-1.7841	0.1362	36	-13.10	<.0001	0.05	-2.0604	-1.5079

The GLIMMIX Procedure

Differences of Colony*Condit*Treatm Least Squares Means													
Colony_Type	Condition	Treatment	_Colony_Type	_Condition	_Treatment	Estimate	Standard Error	DF	t Value	Pr > |t|	Alpha	Lower	Upper
CFU-GEMM	AoE+PSCs	7.5ug/ml	CFU-GM	E10.5AGM	0ug/ml	-3.5975	0.1428	36	-25.19	<.0001	0.05	-3.8870	-3.3079
CFU-GEMM	AoE+PSCs	7.5ug/ml	CFU-GM	E10.5AGM	2.5ug/ml	-3.0339	0.1439	36	-21.08	<.0001	0.05	-3.3259	-2.7420
CFU-GEMM	AoE+PSCs	7.5ug/ml	CFU-GM	E10.5AGM	7.5ug/ml	-2.4054	0.1462	36	-16.45	<.0001	0.05	-2.7020	-2.1088
CFU-GEMM	AoE+PSCs	7.5ug/ml	CFU-GM	HE+PSCs	0ug/ml	-3.0651	0.1439	36	-21.31	<.0001	0.05	-3.3569	-2.7734
CFU-GEMM	AoE+PSCs	7.5ug/ml	CFU-GM	HE+PSCs	2.5ug/ml	-2.2737	0.1469	36	-15.47	<.0001	0.05	-2.5717	-1.9757
CFU-GEMM	AoE+PSCs	7.5ug/ml	CFU-GM	HE+PSCs	7.5ug/ml	-1.5391	0.1529	36	-10.07	<.0001	0.05	-1.8491	-1.2291
CFU-GEMM	E10.5AGM	0ug/ml	CFU-GEMM	E10.5AGM	2.5ug/ml	0.2404	0.09119	36	2.64	0.0123	0.05	0.05547	0.4254
CFU-GEMM	E10.5AGM	0ug/ml	CFU-GEMM	E10.5AGM	7.5ug/ml	1.1780	0.1096	36	10.75	<.0001	0.05	0.9558	1.4002
CFU-GEMM	E10.5AGM	0ug/ml	CFU-GEMM	HE+PSCs	0ug/ml	0.7071	0.09862	36	7.17	<.0001	0.05	0.5071	0.9072
CFU-GEMM	E10.5AGM	0ug/ml	CFU-GEMM	HE+PSCs	2.5ug/ml	1.5375	0.1210	36	12.71	<.0001	0.05	1.2921	1.7829
CFU-GEMM	E10.5AGM	0ug/ml	CFU-GEMM	HE+PSCs	7.5ug/ml	2.3924	0.1623	36	14.74	<.0001	0.05	2.0632	2.7216
CFU-GEMM	E10.5AGM	0ug/ml	CFU-GM	AoE+PSCs	0ug/ml	-1.0140	0.08133	36	-12.47	<.0001	0.05	-1.1789	-0.8490
CFU-GEMM	E10.5AGM	0ug/ml	CFU-GM	AoE+PSCs	2.5ug/ml	-0.2248	0.08621	36	-2.61	0.0132	0.05	-0.3996	-0.04992
CFU-GEMM	E10.5AGM	0ug/ml	CFU-GM	AoE+PSCs	7.5ug/ml	0.3550	0.09276	36	3.83	0.0005	0.05	0.1669	0.5431
CFU-GEMM	E10.5AGM	0ug/ml	CFU-GM	E10.5AGM	0ug/ml	-1.4583	0.04805	36	-30.35	<.0001	0.05	-1.5558	-1.3609
CFU-GEMM	E10.5AGM	0ug/ml	CFU-GM	E10.5AGM	2.5ug/ml	-0.8948	0.08185	36	-10.93	<.0001	0.05	-1.0608	-0.7288
CFU-GEMM	E10.5AGM	0ug/ml	CFU-GM	E10.5AGM	7.5ug/ml	-0.2663	0.08585	36	-3.10	0.0037	0.05	-0.4404	-0.09216
CFU-GEMM	E10.5AGM	0ug/ml	CFU-GM	HE+PSCs	0ug/ml	-0.9260	0.08171	36	-11.33	<.0001	0.05	-1.0917	-0.7603
CFU-GEMM	E10.5AGM	0ug/ml	CFU-GM	HE+PSCs	2.5ug/ml	-0.1346	0.08701	36	-1.55	0.1307	0.05	-0.3110	0.04190
CFU-GEMM	E10.5AGM	0ug/ml	CFU-GM	HE+PSCs	7.5ug/ml	0.6000	0.09670	36	6.21	<.0001	0.05	0.4039	0.7962
CFU-GEMM	E10.5AGM	2.5ug/ml	CFU-GEMM	E10.5AGM	7.5ug/ml	0.9375	0.1119	36	8.38	<.0001	0.05	0.7107	1.1644
CFU-GEMM	E10.5AGM	2.5ug/ml	CFU-GEMM	HE+PSCs	0ug/ml	0.4667	0.1012	36	4.61	<.0001	0.05	0.2615	0.6719
CFU-GEMM	E10.5AGM	2.5ug/ml	CFU-GEMM	HE+PSCs	2.5ug/ml	1.2971	0.1231	36	10.54	<.0001	0.05	1.0475	1.5467
CFU-GEMM	E10.5AGM	2.5ug/ml	CFU-GEMM	HE+PSCs	7.5ug/ml	2.1519	0.1639	36	13.13	<.0001	0.05	1.8196	2.4843

The GLIMMIX Procedure

Differences of Colony*Condit*Treatm Least Squares Means													
Colony_Type	Condition	Treatment	_Colony_Type	_Condition	_Treatment	Estimate	Standard Error	DF	t Value	Pr > |t|	Alpha	Lower	Upper
CFU-GEMM	E10.5AGM	2.5ug/ml	CFU-GM	AoE+PSCs	0ug/ml	-1.2544	0.08440	36	-14.86	<.0001	0.05	-1.4256	-1.0832
CFU-GEMM	E10.5AGM	2.5ug/ml	CFU-GM	AoE+PSCs	2.5ug/ml	-0.4652	0.08911	36	-5.22	<.0001	0.05	-0.6459	-0.2845
CFU-GEMM	E10.5AGM	2.5ug/ml	CFU-GM	AoE+PSCs	7.5ug/ml	0.1146	0.09546	36	1.20	0.2380	0.05	-0.07905	0.3082
CFU-GEMM	E10.5AGM	2.5ug/ml	CFU-GM	E10.5AGM	0ug/ml	-1.6987	0.08294	36	-20.48	<.0001	0.05	-1.8669	-1.5305
CFU-GEMM	E10.5AGM	2.5ug/ml	CFU-GM	E10.5AGM	2.5ug/ml	-1.1352	0.05609	36	-20.24	<.0001	0.05	-1.2490	-1.0215
CFU-GEMM	E10.5AGM	2.5ug/ml	CFU-GM	E10.5AGM	7.5ug/ml	-0.5067	0.08877	36	-5.71	<.0001	0.05	-0.6867	-0.3267
CFU-GEMM	E10.5AGM	2.5ug/ml	CFU-GM	HE+PSCs	0ug/ml	-1.1664	0.08477	36	-13.76	<.0001	0.05	-1.3383	-0.9945
CFU-GEMM	E10.5AGM	2.5ug/ml	CFU-GM	HE+PSCs	2.5ug/ml	-0.3750	0.08989	36	-4.17	0.0002	0.05	-0.5573	-0.1927
CFU-GEMM	E10.5AGM	2.5ug/ml	CFU-GM	HE+PSCs	7.5ug/ml	0.3596	0.09930	36	3.62	0.0009	0.05	0.1582	0.5610
CFU-GEMM	E10.5AGM	7.5ug/ml	CFU-GEMM	HE+PSCs	0ug/ml	-0.4708	0.1180	36	-3.99	0.0003	0.05	-0.7101	-0.2315
CFU-GEMM	E10.5AGM	7.5ug/ml	CFU-GEMM	HE+PSCs	2.5ug/ml	0.3595	0.1372	36	2.62	0.0128	0.05	0.08120	0.6378
CFU-GEMM	E10.5AGM	7.5ug/ml	CFU-GEMM	HE+PSCs	7.5ug/ml	1.2144	0.1747	36	6.95	<.0001	0.05	0.8600	1.5688
CFU-GEMM	E10.5AGM	7.5ug/ml	CFU-GM	AoE+PSCs	0ug/ml	-2.1919	0.1040	36	-21.08	<.0001	0.05	-2.4028	-1.9811
CFU-GEMM	E10.5AGM	7.5ug/ml	CFU-GM	AoE+PSCs	2.5ug/ml	-1.4027	0.1078	36	-13.01	<.0001	0.05	-1.6214	-1.1840
CFU-GEMM	E10.5AGM	7.5ug/ml	CFU-GM	AoE+PSCs	7.5ug/ml	-0.8230	0.1131	36	-7.27	<.0001	0.05	-1.0524	-0.5935
CFU-GEMM	E10.5AGM	7.5ug/ml	CFU-GM	E10.5AGM	0ug/ml	-2.6363	0.1028	36	-25.65	<.0001	0.05	-2.8447	-2.4278
CFU-GEMM	E10.5AGM	7.5ug/ml	CFU-GM	E10.5AGM	2.5ug/ml	-2.0728	0.1044	36	-19.86	<.0001	0.05	-2.2845	-1.8611
CFU-GEMM	E10.5AGM	7.5ug/ml	CFU-GM	E10.5AGM	7.5ug/ml	-1.4442	0.08656	36	-16.68	<.0001	0.05	-1.6198	-1.2687
CFU-GEMM	E10.5AGM	7.5ug/ml	CFU-GM	HE+PSCs	0ug/ml	-2.1040	0.1043	36	-20.18	<.0001	0.05	-2.3154	-1.8925
CFU-GEMM	E10.5AGM	7.5ug/ml	CFU-GM	HE+PSCs	2.5ug/ml	-1.3125	0.1085	36	-12.10	<.0001	0.05	-1.5325	-1.0925
CFU-GEMM	E10.5AGM	7.5ug/ml	CFU-GM	HE+PSCs	7.5ug/ml	-0.5779	0.1164	36	-4.97	<.0001	0.05	-0.8139	-0.3419
CFU-GEMM	HE+PSCs	0ug/ml	CFU-GEMM	HE+PSCs	2.5ug/ml	0.8303	0.1287	36	6.45	<.0001	0.05	0.5694	1.0913
CFU-GEMM	HE+PSCs	0ug/ml	CFU-GEMM	HE+PSCs	7.5ug/ml	1.6852	0.1681	36	10.02	<.0001	0.05	1.3443	2.0262
CFU-GEMM	HE+PSCs	0ug/ml	CFU-GM	AoE+PSCs	0ug/ml	-1.7211	0.09238	36	-18.63	<.0001	0.05	-1.9085	-1.5338

The GLIMMIX Procedure

Differences of Colony*Condit*Treatm Least Squares Means													
Colony_Type	Condition	Treatment	_Colony_Type	_Condition	_Treatment	Estimate	Standard Error	DF	t Value	Pr > |t|	Alpha	Lower	Upper
CFU-GEMM	HE+PSCs	0ug/ml	CFU-GM	AoE+PSCs	2.5ug/ml	-0.9319	0.09670	36	-9.64	<.0001	0.05	-1.1280	-0.7358
CFU-GEMM	HE+PSCs	0ug/ml	CFU-GM	AoE+PSCs	7.5ug/ml	-0.3522	0.1026	36	-3.43	0.0015	0.05	-0.5602	-0.1441
CFU-GEMM	HE+PSCs	0ug/ml	CFU-GM	E10.5AGM	0ug/ml	-2.1655	0.09104	36	-23.79	<.0001	0.05	-2.3501	-1.9808
CFU-GEMM	HE+PSCs	0ug/ml	CFU-GM	E10.5AGM	2.5ug/ml	-1.6019	0.09284	36	-17.26	<.0001	0.05	-1.7902	-1.4137
CFU-GEMM	HE+PSCs	0ug/ml	CFU-GM	E10.5AGM	7.5ug/ml	-0.9734	0.09638	36	-10.10	<.0001	0.05	-1.1689	-0.7780
CFU-GEMM	HE+PSCs	0ug/ml	CFU-GM	HE+PSCs	0ug/ml	-1.6331	0.06730	36	-24.27	<.0001	0.05	-1.7696	-1.4967
CFU-GEMM	HE+PSCs	0ug/ml	CFU-GM	HE+PSCs	2.5ug/ml	-0.8417	0.09741	36	-8.64	<.0001	0.05	-1.0393	-0.6441
CFU-GEMM	HE+PSCs	0ug/ml	CFU-GM	HE+PSCs	7.5ug/ml	-0.1071	0.1062	36	-1.01	0.3198	0.05	-0.3224	0.1082
CFU-GEMM	HE+PSCs	2.5ug/ml	CFU-GEMM	HE+PSCs	7.5ug/ml	0.8549	0.1821	36	4.69	<.0001	0.05	0.4855	1.2243
CFU-GEMM	HE+PSCs	2.5ug/ml	CFU-GM	AoE+PSCs	0ug/ml	-2.5515	0.1160	36	-22.00	<.0001	0.05	-2.7866	-2.3163
CFU-GEMM	HE+PSCs	2.5ug/ml	CFU-GM	AoE+PSCs	2.5ug/ml	-1.7622	0.1194	36	-14.76	<.0001	0.05	-2.0044	-1.5200
CFU-GEMM	HE+PSCs	2.5ug/ml	CFU-GM	AoE+PSCs	7.5ug/ml	-1.1825	0.1242	36	-9.52	<.0001	0.05	-1.4345	-0.9305
CFU-GEMM	HE+PSCs	2.5ug/ml	CFU-GM	E10.5AGM	0ug/ml	-2.9958	0.1149	36	-26.07	<.0001	0.05	-3.2288	-2.7628
CFU-GEMM	HE+PSCs	2.5ug/ml	CFU-GM	E10.5AGM	2.5ug/ml	-2.4323	0.1163	36	-20.91	<.0001	0.05	-2.6682	-2.1964
CFU-GEMM	HE+PSCs	2.5ug/ml	CFU-GM	E10.5AGM	7.5ug/ml	-1.8038	0.1192	36	-15.14	<.0001	0.05	-2.0454	-1.5621
CFU-GEMM	HE+PSCs	2.5ug/ml	CFU-GM	HE+PSCs	0ug/ml	-2.4635	0.1162	36	-21.20	<.0001	0.05	-2.6992	-2.2278
CFU-GEMM	HE+PSCs	2.5ug/ml	CFU-GM	HE+PSCs	2.5ug/ml	-1.6720	0.1017	36	-16.45	<.0001	0.05	-1.8783	-1.4658
CFU-GEMM	HE+PSCs	2.5ug/ml	CFU-GM	HE+PSCs	7.5ug/ml	-0.9374	0.1272	36	-7.37	<.0001	0.05	-1.1954	-0.6795
CFU-GEMM	HE+PSCs	7.5ug/ml	CFU-GM	AoE+PSCs	0ug/ml	-3.4063	0.1586	36	-21.48	<.0001	0.05	-3.7280	-3.0847
CFU-GEMM	HE+PSCs	7.5ug/ml	CFU-GM	AoE+PSCs	2.5ug/ml	-2.6171	0.1611	36	-16.24	<.0001	0.05	-2.9439	-2.2903
CFU-GEMM	HE+PSCs	7.5ug/ml	CFU-GM	AoE+PSCs	7.5ug/ml	-2.0374	0.1647	36	-12.37	<.0001	0.05	-2.3715	-1.7033
CFU-GEMM	HE+PSCs	7.5ug/ml	CFU-GM	E10.5AGM	0ug/ml	-3.8507	0.1578	36	-24.40	<.0001	0.05	-4.1708	-3.5306
CFU-GEMM	HE+PSCs	7.5ug/ml	CFU-GM	E10.5AGM	2.5ug/ml	-3.2872	0.1589	36	-20.69	<.0001	0.05	-3.6094	-2.9650
CFU-GEMM	HE+PSCs	7.5ug/ml	CFU-GM	E10.5AGM	7.5ug/ml	-2.6587	0.1609	36	-16.52	<.0001	0.05	-2.9851	-2.3322

The GLIMMIX Procedure

Colony_Type	Condition	Treatment	_Colony_Type	_Condition	_Treatment	Estimate	Standard Error	DF	t Value	Pr > |t|	Alpha	Lower	Upper
CFU-GEMM	HE+PSCs	7.5ug/ml	CFU-GM	HE+PSCs	0ug/ml	-3.3184	0.1588	36	-20.90	<.0001	0.05	-3.6404	-2.9963
CFU-GEMM	HE+PSCs	7.5ug/ml	CFU-GM	HE+PSCs	2.5ug/ml	-2.5269	0.1616	36	-15.64	<.0001	0.05	-2.8546	-2.1992
CFU-GEMM	HE+PSCs	7.5ug/ml	CFU-GM	HE+PSCs	7.5ug/ml	-1.7923	0.1542	36	-11.62	<.0001	0.05	-2.1051	-1.4795
CFU-GM	AoE+PSCs	0ug/ml	CFU-GM	AoE+PSCs	2.5ug/ml	0.7892	0.07899	36	9.99	<.0001	0.05	0.6290	0.9494
CFU-GM	AoE+PSCs	0ug/ml	CFU-GM	AoE+PSCs	7.5ug/ml	1.3690	0.08609	36	15.90	<.0001	0.05	1.1944	1.5436
CFU-GM	AoE+PSCs	0ug/ml	CFU-GM	E10.5AGM	0ug/ml	-0.4443	0.07195	36	-6.18	<.0001	0.05	-0.5903	-0.2984
CFU-GM	AoE+PSCs	0ug/ml	CFU-GM	E10.5AGM	2.5ug/ml	0.1192	0.07421	36	1.61	0.1170	0.05	-0.03133	0.2697
CFU-GM	AoE+PSCs	0ug/ml	CFU-GM	E10.5AGM	7.5ug/ml	0.7477	0.07860	36	9.51	<.0001	0.05	0.5883	0.9071
CFU-GM	AoE+PSCs	0ug/ml	CFU-GM	HE+PSCs	0ug/ml	0.08797	0.07406	36	1.19	0.2426	0.05	-0.06222	0.2382
CFU-GM	AoE+PSCs	0ug/ml	CFU-GM	HE+PSCs	2.5ug/ml	0.8794	0.07986	36	11.01	<.0001	0.05	0.7174	1.0414
CFU-GM	AoE+PSCs	0ug/ml	CFU-GM	HE+PSCs	7.5ug/ml	1.6140	0.09033	36	17.87	<.0001	0.05	1.4308	1.7972
CFU-GM	AoE+PSCs	2.5ug/ml	CFU-GM	AoE+PSCs	7.5ug/ml	0.5797	0.09071	36	6.39	<.0001	0.05	0.3958	0.7637
CFU-GM	AoE+PSCs	2.5ug/ml	CFU-GM	E10.5AGM	0ug/ml	-1.2336	0.07742	36	-15.93	<.0001	0.05	-1.3906	-1.0766
CFU-GM	AoE+PSCs	2.5ug/ml	CFU-GM	E10.5AGM	2.5ug/ml	-0.6700	0.07953	36	-8.43	<.0001	0.05	-0.8313	-0.5088
CFU-GM	AoE+PSCs	2.5ug/ml	CFU-GM	E10.5AGM	7.5ug/ml	-0.04153	0.08363	36	-0.50	0.6225	0.05	-0.2111	0.1281
CFU-GM	AoE+PSCs	2.5ug/ml	CFU-GM	HE+PSCs	0ug/ml	-0.7013	0.07938	36	-8.83	<.0001	0.05	-0.8622	-0.5403
CFU-GM	AoE+PSCs	2.5ug/ml	CFU-GM	HE+PSCs	2.5ug/ml	0.09018	0.08482	36	1.06	0.2948	0.05	-0.08185	0.2622
CFU-GM	AoE+PSCs	2.5ug/ml	CFU-GM	HE+PSCs	7.5ug/ml	0.8248	0.09473	36	8.71	<.0001	0.05	0.6327	1.0169
CFU-GM	AoE+PSCs	7.5ug/ml	CFU-GM	E10.5AGM	0ug/ml	-1.8133	0.08466	36	-21.42	<.0001	0.05	-1.9850	-1.6416
CFU-GM	AoE+PSCs	7.5ug/ml	CFU-GM	E10.5AGM	2.5ug/ml	-1.2498	0.08659	36	-14.43	<.0001	0.05	-1.4254	-1.0742
CFU-GM	AoE+PSCs	7.5ug/ml	CFU-GM	E10.5AGM	7.5ug/ml	-0.6213	0.09037	36	-6.87	<.0001	0.05	-0.8045	-0.4380
CFU-GM	AoE+PSCs	7.5ug/ml	CFU-GM	HE+PSCs	0ug/ml	-1.2810	0.08645	36	-14.82	<.0001	0.05	-1.4563	-1.1057
CFU-GM	AoE+PSCs	7.5ug/ml	CFU-GM	HE+PSCs	2.5ug/ml	-0.4896	0.09147	36	-5.35	<.0001	0.05	-0.6751	-0.3040
CFU-GM	AoE+PSCs	7.5ug/ml	CFU-GM	HE+PSCs	7.5ug/ml	0.2451	0.1007	36	2.43	0.0201	0.05	0.04079	0.4493

Differences of Colony*Condit*Treatm Least Squares Means

The GLIMMIX Procedure

Differences of Colony*Condit*Treatm Least Squares Means

Colony_Type	Condition	Treatment	_Colony_Type	_Condition	_Treatment	Estimate	Standard Error	DF	t Value	Pr > |t|	Alpha	Lower	Upper
CFU-GM	E10.5AGM	0ug/ml	CFU-GM	E10.5AGM	2.5ug/ml	0.5635	0.07254	36	7.77	<.0001	0.05	0.4164	0.7106
CFU-GM	E10.5AGM	0ug/ml	CFU-GM	E10.5AGM	7.5ug/ml	1.1920	0.07703	36	15.48	<.0001	0.05	1.0358	1.3483
CFU-GM	E10.5AGM	0ug/ml	CFU-GM	HE+PSCs	0ug/ml	0.5323	0.07239	36	7.35	<.0001	0.05	0.3855	0.6791
CFU-GM	E10.5AGM	0ug/ml	CFU-GM	HE+PSCs	2.5ug/ml	1.3238	0.07832	36	16.90	<.0001	0.05	1.1649	1.4826
CFU-GM	E10.5AGM	0ug/ml	CFU-GM	HE+PSCs	7.5ug/ml	2.0584	0.08896	36	23.14	<.0001	0.05	1.8779	2.2388
CFU-GM	E10.5AGM	2.5ug/ml	CFU-GM	E10.5AGM	7.5ug/ml	0.6285	0.07914	36	7.94	<.0001	0.05	0.4680	0.7890
CFU-GM	E10.5AGM	2.5ug/ml	CFU-GM	HE+PSCs	0ug/ml	-0.03120	0.07463	36	-0.42	0.6784	0.05	-0.1826	0.1202
CFU-GM	E10.5AGM	2.5ug/ml	CFU-GM	HE+PSCs	2.5ug/ml	0.7602	0.08040	36	9.46	<.0001	0.05	0.5972	0.9233
CFU-GM	E10.5AGM	2.5ug/ml	CFU-GM	HE+PSCs	7.5ug/ml	1.4948	0.09080	36	16.46	<.0001	0.05	1.3107	1.6790
CFU-GM	E10.5AGM	7.5ug/ml	CFU-GM	HE+PSCs	0ug/ml	-0.6597	0.07899	36	-8.35	<.0001	0.05	-0.8199	-0.4995
CFU-GM	E10.5AGM	7.5ug/ml	CFU-GM	HE+PSCs	2.5ug/ml	0.1317	0.08446	36	1.56	0.1276	0.05	-0.03958	0.3030
CFU-GM	E10.5AGM	7.5ug/ml	CFU-GM	HE+PSCs	7.5ug/ml	0.8663	0.09439	36	9.18	<.0001	0.05	0.6749	1.0578
CFU-GM	HE+PSCs	0ug/ml	CFU-GM	HE+PSCs	2.5ug/ml	0.7914	0.08025	36	9.86	<.0001	0.05	0.6287	0.9542
CFU-GM	HE+PSCs	0ug/ml	CFU-GM	HE+PSCs	7.5ug/ml	1.5261	0.09066	36	16.83	<.0001	0.05	1.3422	1.7099
CFU-GM	HE+PSCs	2.5ug/ml	CFU-GM	HE+PSCs	7.5ug/ml	0.7346	0.09545	36	7.70	<.0001	0.05	0.5410	0.9282

The GENMOD Procedure

Model Information	
Data Set	WORK.EXT_FIG10BI_E10WIF15_TRANS
Distribution	Normal
Link Function	Identity
Dependent Variable	Percentage

Number of Observations Read	45
Number of Observations Used	45

Class Level Information		
Class	Levels	Values
Condition	3	AoE+PSCs E10.5AGM HE+PSCs
Treatment	3	0ug/ml 2.5ug/ml 7.5ug/ml

Parameter Information			
Parameter	Effect	Condition	Treatment
Prm1	Intercept		
Prm2	Condition	AoE+PSCs	
Prm3	Condition	E10.5AGM	
Prm4	Condition	HE+PSCs	
Prm5	Treatment		0ug/ml
Prm6	Treatment		2.5ug/ml
Prm7	Treatment		7.5ug/ml
Prm8	Condition*Treatment	AoE+PSCs	0ug/ml
Prm9	Condition*Treatment	AoE+PSCs	2.5ug/ml
Prm10	Condition*Treatment	AoE+PSCs	7.5ug/ml
Prm11	Condition*Treatment	E10.5AGM	0ug/ml

The GENMOD Procedure

Parameter Information			
**Parameter**	**Effect**	**Condition**	**Treatment**
**Prm12**	Condition*Treatment	E10.5AGM	2.5ug/ml
**Prm13**	Condition*Treatment	E10.5AGM	7.5ug/ml
**Prm14**	Condition*Treatment	HE+PSCs	0ug/ml
**Prm15**	Condition*Treatment	HE+PSCs	2.5ug/ml
**Prm16**	Condition*Treatment	HE+PSCs	7.5ug/ml

Criteria For Assessing Goodness Of Fit			
**Criterion**	**DF**	**Value**	**Value/DF**
**Deviance**	36	451.2000	12.5333
**Scaled Deviance**	36	45.0000	1.2500
**Pearson Chi-Square**	36	451.2000	12.5333
**Scaled Pearson X2**	36	45.0000	1.2500
**Log Likelihood**		-115.7203	
**Full Log Likelihood**		-115.7203	
**AIC (smaller is better)**		251.4406	
**AICC (smaller is better)**		257.9112	
**BIC (smaller is better)**		269.5073	

Algorithm converged.

The GENMOD Procedure

Analysis Of Maximum Likelihood Parameter Estimates									
Parameter			DF	Estimate	Standard Error	Wald 95% Confidence Limits		Wald Chi-Square	Pr > ChiSq
Intercept			1	5.0000	1.4161	2.2245	7.7755	12.47	0.0004
Condition	AoE+PSCs		1	1.8000	2.0027	-2.1252	5.7252	0.81	0.3688
Condition	E10.5AGM		1	10.6000	2.0027	6.6748	14.5252	28.02	<.0001
Condition	HE+PSCs		0	0.0000	0.0000	0.0000	0.0000	.	.
Treatment	0ug/ml		1	21.2000	2.0027	17.2748	25.1252	112.06	<.0001
Treatment	2.5ug/ml		1	10.8000	2.0027	6.8748	14.7252	29.08	<.0001
Treatment	7.5ug/ml		0	0.0000	0.0000	0.0000	0.0000	.	.
Condition*Treatment	AoE+PSCs	0ug/ml	1	3.8000	2.8322	-1.7510	9.3510	1.80	0.1797
Condition*Treatment	AoE+PSCs	2.5ug/ml	1	3.4000	2.8322	-2.1510	8.9510	1.44	0.2300
Condition*Treatment	AoE+PSCs	7.5ug/ml	0	0.0000	0.0000	0.0000	0.0000	.	.
Condition*Treatment	E10.5AGM	0ug/ml	1	20.0000	2.8322	14.4490	25.5510	49.87	<.0001
Condition*Treatment	E10.5AGM	2.5ug/ml	1	15.6000	2.8322	10.0490	21.1510	30.34	<.0001
Condition*Treatment	E10.5AGM	7.5ug/ml	0	0.0000	0.0000	0.0000	0.0000	.	.
Condition*Treatment	HE+PSCs	0ug/ml	0	0.0000	0.0000	0.0000	0.0000	.	.
Condition*Treatment	HE+PSCs	2.5ug/ml	0	0.0000	0.0000	0.0000	0.0000	.	.
Condition*Treatment	HE+PSCs	7.5ug/ml	0	0.0000	0.0000	0.0000	0.0000	.	.
Scale			1	3.1665	0.3338	2.5755	3.8932		

Note: The scale parameter was estimated by maximum likelihood.

The GENMOD Procedure

Condition*Treatment Least Squares Means								
Condition	Treatment	Estimate	Standard Error	z Value	Pr > |z|	Alpha	Lower	Upper
AoE+PSCs	0ug/ml	31.8000	1.4161	22.46	<.0001	0.05	29.0245	34.5755
AoE+PSCs	2.5ug/ml	21.0000	1.4161	14.83	<.0001	0.05	18.2245	23.7755
AoE+PSCs	7.5ug/ml	6.8000	1.4161	4.80	<.0001	0.05	4.0245	9.5755
E10.5AGM	0ug/ml	56.8000	1.4161	40.11	<.0001	0.05	54.0245	59.5755
E10.5AGM	2.5ug/ml	42.0000	1.4161	29.66	<.0001	0.05	39.2245	44.7755
E10.5AGM	7.5ug/ml	15.6000	1.4161	11.02	<.0001	0.05	12.8245	18.3755
HE+PSCs	0ug/ml	26.2000	1.4161	18.50	<.0001	0.05	23.4245	28.9755
HE+PSCs	2.5ug/ml	15.8000	1.4161	11.16	<.0001	0.05	13.0245	18.5755
HE+PSCs	7.5ug/ml	5.0000	1.4161	3.53	0.0004	0.05	2.2245	7.7755

Differences of Condition*Treatment Least Squares Means										
Condition	Treatment	_Condition	_Treatment	Estimate	Standard Error	z Value	Pr > |z|	Alpha	Lower	Upper
AoE+PSCs	0ug/ml	AoE+PSCs	2.5ug/ml	10.8000	2.0027	5.39	<.0001	0.05	6.8748	14.7252
AoE+PSCs	0ug/ml	AoE+PSCs	7.5ug/ml	25.0000	2.0027	12.48	<.0001	0.05	21.0748	28.9252
AoE+PSCs	0ug/ml	E10.5AGM	0ug/ml	-25.0000	2.0027	-12.48	<.0001	0.05	-28.9252	-21.0748
AoE+PSCs	0ug/ml	E10.5AGM	2.5ug/ml	-10.2000	2.0027	-5.09	<.0001	0.05	-14.1252	-6.2748
AoE+PSCs	0ug/ml	E10.5AGM	7.5ug/ml	16.2000	2.0027	8.09	<.0001	0.05	12.2748	20.1252
AoE+PSCs	0ug/ml	HE+PSCs	0ug/ml	5.6000	2.0027	2.80	0.0052	0.05	1.6748	9.5252
AoE+PSCs	0ug/ml	HE+PSCs	2.5ug/ml	16.0000	2.0027	7.99	<.0001	0.05	12.0748	19.9252
AoE+PSCs	0ug/ml	HE+PSCs	7.5ug/ml	26.8000	2.0027	13.38	<.0001	0.05	22.8748	30.7252
AoE+PSCs	2.5ug/ml	AoE+PSCs	7.5ug/ml	14.2000	2.0027	7.09	<.0001	0.05	10.2748	18.1252
AoE+PSCs	2.5ug/ml	E10.5AGM	0ug/ml	-35.8000	2.0027	-17.88	<.0001	0.05	-39.7252	-31.8748
AoE+PSCs	2.5ug/ml	E10.5AGM	2.5ug/ml	-21.0000	2.0027	-10.49	<.0001	0.05	-24.9252	-17.0748
AoE+PSCs	2.5ug/ml	E10.5AGM	7.5ug/ml	5.4000	2.0027	2.70	0.0070	0.05	1.4748	9.3252

The GENMOD Procedure

Differences of Condition*Treatment Least Squares Means										
Condition	Treatment	_Condition	_Treatment	Estimate	Standard Error	z Value	Pr > |z|	Alpha	Lower	Upper
AoE+PSCs	2.5ug/ml	HE+PSCs	0ug/ml	-5.2000	2.0027	-2.60	0.0094	0.05	-9.1252	-1.2748
AoE+PSCs	2.5ug/ml	HE+PSCs	2.5ug/ml	5.2000	2.0027	2.60	0.0094	0.05	1.2748	9.1252
AoE+PSCs	2.5ug/ml	HE+PSCs	7.5ug/ml	16.0000	2.0027	7.99	<.0001	0.05	12.0748	19.9252
AoE+PSCs	7.5ug/ml	E10.5AGM	0ug/ml	-50.0000	2.0027	-24.97	<.0001	0.05	-53.9252	-46.0748
AoE+PSCs	7.5ug/ml	E10.5AGM	2.5ug/ml	-35.2000	2.0027	-17.58	<.0001	0.05	-39.1252	-31.2748
AoE+PSCs	7.5ug/ml	E10.5AGM	7.5ug/ml	-8.8000	2.0027	-4.39	<.0001	0.05	-12.7252	-4.8748
AoE+PSCs	7.5ug/ml	HE+PSCs	0ug/ml	-19.4000	2.0027	-9.69	<.0001	0.05	-23.3252	-15.4748
AoE+PSCs	7.5ug/ml	HE+PSCs	2.5ug/ml	-9.0000	2.0027	-4.49	<.0001	0.05	-12.9252	-5.0748
AoE+PSCs	7.5ug/ml	HE+PSCs	7.5ug/ml	1.8000	2.0027	0.90	0.3688	0.05	-2.1252	5.7252
E10.5AGM	0ug/ml	E10.5AGM	2.5ug/ml	14.8000	2.0027	7.39	<.0001	0.05	10.8748	18.7252
E10.5AGM	0ug/ml	E10.5AGM	7.5ug/ml	41.2000	2.0027	20.57	<.0001	0.05	37.2748	45.1252
E10.5AGM	0ug/ml	HE+PSCs	0ug/ml	30.6000	2.0027	15.28	<.0001	0.05	26.6748	34.5252
E10.5AGM	0ug/ml	HE+PSCs	2.5ug/ml	41.0000	2.0027	20.47	<.0001	0.05	37.0748	44.9252
E10.5AGM	0ug/ml	HE+PSCs	7.5ug/ml	51.8000	2.0027	25.87	<.0001	0.05	47.8748	55.7252
E10.5AGM	2.5ug/ml	E10.5AGM	7.5ug/ml	26.4000	2.0027	13.18	<.0001	0.05	22.4748	30.3252
E10.5AGM	2.5ug/ml	HE+PSCs	0ug/ml	15.8000	2.0027	7.89	<.0001	0.05	11.8748	19.7252
E10.5AGM	2.5ug/ml	HE+PSCs	2.5ug/ml	26.2000	2.0027	13.08	<.0001	0.05	22.2748	30.1252
E10.5AGM	2.5ug/ml	HE+PSCs	7.5ug/ml	37.0000	2.0027	18.48	<.0001	0.05	33.0748	40.9252
E10.5AGM	7.5ug/ml	HE+PSCs	0ug/ml	-10.6000	2.0027	-5.29	<.0001	0.05	-14.5252	-6.6748
E10.5AGM	7.5ug/ml	HE+PSCs	2.5ug/ml	-0.2000	2.0027	-0.10	0.9204	0.05	-4.1252	3.7252
E10.5AGM	7.5ug/ml	HE+PSCs	7.5ug/ml	10.6000	2.0027	5.29	<.0001	0.05	6.6748	14.5252
HE+PSCs	0ug/ml	HE+PSCs	2.5ug/ml	10.4000	2.0027	5.19	<.0001	0.05	6.4748	14.3252
HE+PSCs	0ug/ml	HE+PSCs	7.5ug/ml	21.2000	2.0027	10.59	<.0001	0.05	17.2748	25.1252
HE+PSCs	2.5ug/ml	HE+PSCs	7.5ug/ml	10.8000	2.0027	5.39	<.0001	0.05	6.8748	14.7252

The GENMOD Procedure

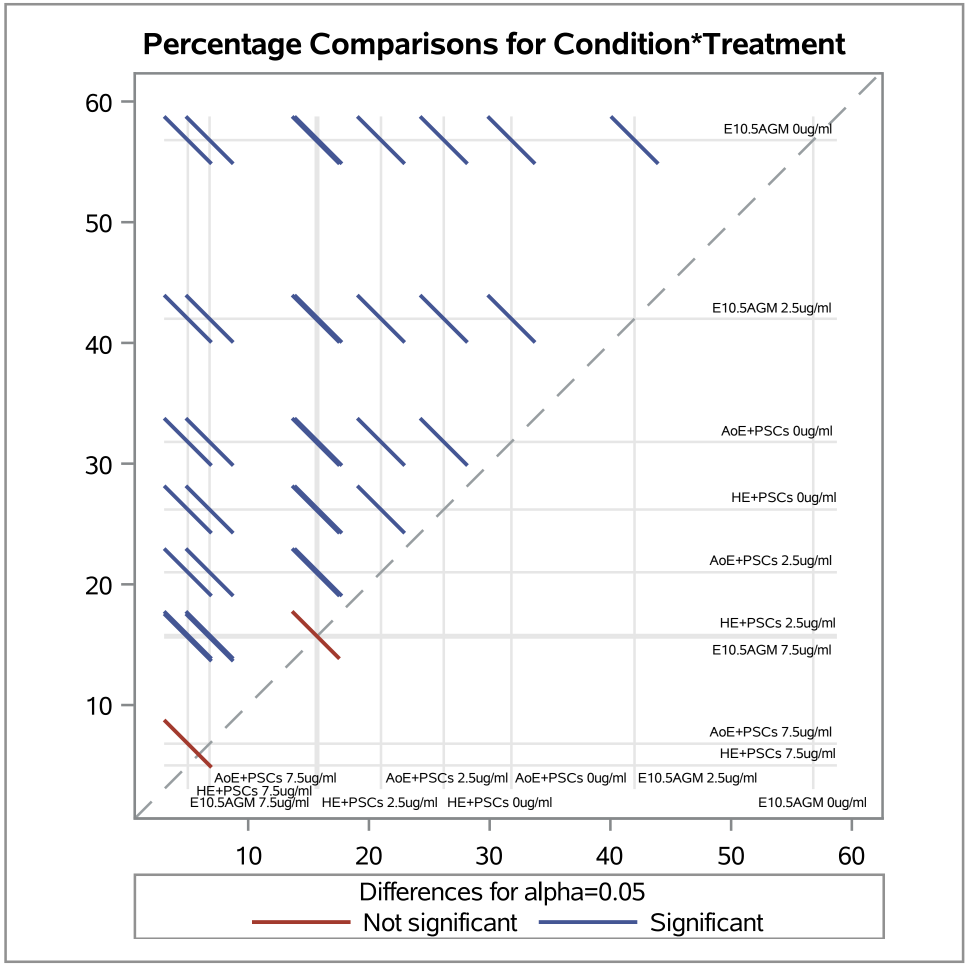

The GLIMMIX Procedure

Model Information	
Data Set	WORK.EXT_FIG10BI_E11DRAXIN
Response Variable	Count
Response Distribution	Poisson
Link Function	Log
Variance Function	Default
Variance Matrix Blocked By	Dish_ID
Estimation Technique	Maximum Likelihood
Likelihood Approximation	Laplace
Degrees of Freedom Method	Containment

Class Level Information		
Class	Levels	Values
Colony_Type	3	BFU-E CFU-GEMM CFU-GM
Condition	3	AoE+PSCs E11.5AGM HE+PSCs
Treatment	3	0ug/ml 2ug/ml 6ug/ml
Dish_ID	27	1 2 3 4 5 6 7 8 9 10 11 12 13 14 15 16 17 18 19 20 21 22 23 24 25 26 27

Number of Observations Read	81
Number of Observations Used	81

Dimensions	
G-side Cov. Parameters	1
Columns in X	64
Columns in Z per Subject	1
Subjects (Blocks in V)	27
Max Obs per Subject	3

The GLIMMIX Procedure

Optimization Information	
Optimization Technique	Dual Quasi-Newton
Parameters in Optimization	28
Lower Boundaries	1
Upper Boundaries	0
Fixed Effects	Not Profiled
Starting From	GLM estimates

Iteration History

Iteration	Restarts	Evaluations	Objective Function	Change	Max Gradient
0	0	4	823.17979438	.	2424.21
1	0	9	814.14461119	9.03518319	117.8846
2	0	4	813.89201734	0.25259385	51.54377
3	0	2	813.85446321	0.03755413	16.80489
4	0	4	813.80469358	0.04976964	23.91615
5	0	2	813.79477622	0.00991735	4.97863
6	0	3	813.79060941	0.00416681	8.799402
7	0	4	813.77892323	0.01168618	3.610637
8	0	3	813.77248914	0.00643409	3.093295
9	0	4	813.75729687	0.01519227	1.985022
10	0	3	813.75596126	0.00133561	2.272758
11	0	4	813.74576975	0.01019151	1.815805
12	0	3	813.74467564	0.00109411	3.082919
13	0	3	813.74432444	0.00035120	0.838516
14	0	4	813.74293968	0.00138477	0.840142
15	0	3	813.74278925	0.00015043	0.465236

The GLIMMIX Procedure

Iteration History					
**Iteration**	**Restarts**	**Evaluations**	**Objective Function**	**Change**	**Max Gradient**
**16**	**0**	4	813.74226814	0.00052111	1.370968
**17**	**0**	2	813.74150709	0.00076105	0.888084
**18**	**0**	2	813.74019459	0.00131249	1.285485
**19**	**0**	2	813.738965	0.00122959	0.985413
**20**	**0**	3	813.73846571	0.00049929	0.616019
**21**	**0**	3	813.738231	0.00023471	0.769973
**22**	**0**	4	813.73718179	0.00104922	0.622338
**23**	**0**	3	813.73703807	0.00014372	0.579588
**24**	**0**	4	813.73463121	0.00240686	0.695739
**25**	**0**	3	813.73385084	0.00078037	2.287961
**26**	**0**	3	813.73337237	0.00047847	2.577843
**27**	**0**	4	813.7319071	0.00146527	0.421931
**28**	**0**	2	813.73166963	0.00023746	0.861401
**29**	**0**	4	813.73076048	0.00090915	0.813403
**30**	**0**	3	813.73062416	0.00013632	0.609386
**31**	**0**	4	813.72978987	0.00083430	0.459345
**32**	**0**	3	813.72975195	0.00003792	0.359891
**33**	**0**	4	813.72936577	0.00038618	0.217266
**34**	**0**	3	813.72935713	0.00000864	0.284646

Convergence criterion (GCONV=1E-8) satisfied.

The GLIMMIX Procedure

Fit Statistics	
**-2 Log Likelihood**	813.73
**AIC (smaller is better)**	869.73
**AICC (smaller is better)**	900.96
**BIC (smaller is better)**	906.01
**CAIC (smaller is better)**	934.01
**HQIC (smaller is better)**	880.52

Fit Statistics for Conditional Distribution	
**-2 log L(Count | r. effects)**	730.28
**Pearson Chi-Square**	206.21
**Pearson Chi-Square / DF**	2.55

Covariance Parameter Estimates

Cov Parm	Subject	Estimate	Standard Error
**Intercept**	Dish_ID	0.02066	0.007119

The GLIMMIX Procedure

Solutions for Fixed Effects								
**Effect**	**Colony_Type**	**Condition**	**Treatment**	**Estimate**	**Standard Error**	**DF**	**t Value**	**Pr > |t|**
**Intercept**				4.2562	0.1078	18	39.49	<.0001
**Colony_Type**	BFU-E			-0.9874	0.1302	36	-7.58	<.0001
**Colony_Type**	CFU-GEMM			-1.0124	0.1314	36	-7.70	<.0001
**Colony_Type**	CFU-GM			0	.	.	.	.
**Condition**		AoE+PSCs		0.4847	0.1463	36	3.31	0.0021
**Condition**		E11.5AGM		1.1885	0.1412	36	8.42	<.0001
**Condition**		HE+PSCs		0	.	.	.	.
**Colony_Typ*Condition**	BFU-E	AoE+PSCs		-0.6015	0.1841	36	-3.27	0.0024
**Colony_Typ*Condition**	BFU-E	E11.5AGM		-0.7315	0.1622	36	-4.51	<.0001
**Colony_Typ*Condition**	BFU-E	HE+PSCs		0	.	.	.	.
**Colony_Typ*Condition**	CFU-GEMM	AoE+PSCs		-0.5488	0.1840	36	-2.98	0.0051
**Colony_Typ*Condition**	CFU-GEMM	E11.5AGM		-0.6375	0.1616	36	-3.95	0.0004
**Colony_Typ*Condition**	CFU-GEMM	HE+PSCs		0	.	.	.	.
**Colony_Typ*Condition**	CFU-GM	AoE+PSCs		0	.	.	.	.
**Colony_Typ*Condition**	CFU-GM	E11.5AGM		0	.	.	.	.
**Colony_Typ*Condition**	CFU-GM	HE+PSCs		0	.	.	.	.
**Treatment**			0ug/ml	1.8003	0.1389	36	12.96	<.0001
**Treatment**			2ug/ml	0.9410	0.1426	36	6.60	<.0001
**Treatment**			6ug/ml	0	.	.	.	.
**Colony_Typ*Treatment**	BFU-E		0ug/ml	-0.5953	0.1467	36	-4.06	0.0003
**Colony_Typ*Treatment**	BFU-E		2ug/ml	-0.4150	0.1618	36	-2.56	0.0146
**Colony_Typ*Treatment**	BFU-E		6ug/ml	0	.	.	.	.
**Colony_Typ*Treatment**	CFU-GEMM		0ug/ml	-0.4902	0.1468	36	-3.34	0.0020
**Colony_Typ*Treatment**	CFU-GEMM		2ug/ml	-0.7628	0.1728	36	-4.42	<.0001

The GLIMMIX Procedure

Solutions for Fixed Effects								
**Effect**	**Colony_Type**	**Condition**	**Treatment**	**Estimate**	**Standard Error**	**DF**	**t Value**	**Pr > |t|**
**Colony_Typ*Treatment**	CFU-GEMM		6ug/ml	0	.	.	.	.
**Colony_Typ*Treatment**	CFU-GM		0ug/ml	0	.	.	.	.
**Colony_Typ*Treatment**	CFU-GM		2ug/ml	0	.	.	.	.
**Colony_Typ*Treatment**	CFU-GM		6ug/ml	0	.	.	.	.
**Condition*Treatment**		AoE+PSCs	0ug/ml	-0.2902	0.1913	36	-1.52	0.1380
**Condition*Treatment**		AoE+PSCs	2ug/ml	-0.1802	0.1959	36	-0.92	0.3637
**Condition*Treatment**		AoE+PSCs	6ug/ml	0	.	.	.	.
**Condition*Treatment**		E11.5AGM	0ug/ml	-0.5761	0.1868	36	-3.08	0.0039
**Condition*Treatment**		E11.5AGM	2ug/ml	-0.3944	0.1907	36	-2.07	0.0459
**Condition*Treatment**		E11.5AGM	6ug/ml	0	.	.	.	.
**Condition*Treatment**		HE+PSCs	0ug/ml	0	.	.	.	.
**Condition*Treatment**		HE+PSCs	2ug/ml	0	.	.	.	.
**Condition*Treatment**		HE+PSCs	6ug/ml	0	.	.	.	.
**Colony*Condit*Treatm**	BFU-E	AoE+PSCs	0ug/ml	0.4201	0.2070	36	2.03	0.0498
**Colony*Condit*Treatm**	BFU-E	AoE+PSCs	2ug/ml	0.2479	0.2285	36	1.08	0.2853
**Colony*Condit*Treatm**	BFU-E	AoE+PSCs	6ug/ml	0	.	.	.	.
**Colony*Condit*Treatm**	BFU-E	E11.5AGM	0ug/ml	0.9919	0.1814	36	5.47	<.0001
**Colony*Condit*Treatm**	BFU-E	E11.5AGM	2ug/ml	1.0300	0.1972	36	5.22	<.0001
**Colony*Condit*Treatm**	BFU-E	E11.5AGM	6ug/ml	0	.	.	.	.
**Colony*Condit*Treatm**	BFU-E	HE+PSCs	0ug/ml	0	.	.	.	.
**Colony*Condit*Treatm**	BFU-E	HE+PSCs	2ug/ml	0	.	.	.	.
**Colony*Condit*Treatm**	BFU-E	HE+PSCs	6ug/ml	0	.	.	.	.
**Colony*Condit*Treatm**	CFU-GEMM	AoE+PSCs	0ug/ml	0.3800	0.2053	36	1.85	0.0724
**Colony*Condit*Treatm**	CFU-GEMM	AoE+PSCs	2ug/ml	0.8260	0.2318	36	3.56	0.0011

The GLIMMIX Procedure

Solutions for Fixed Effects								
**Effect**	**Colony_Type**	**Condition**	**Treatment**	**Estimate**	**Standard Error**	**DF**	**t Value**	**Pr > |t|**
**Colony*Condit*Treatm**	CFU-GEMM	AoE+PSCs	6ug/ml	0	.	.	.	.
**Colony*Condit*Treatm**	CFU-GEMM	E11.5AGM	0ug/ml	0.6983	0.1805	36	3.87	0.0004
**Colony*Condit*Treatm**	CFU-GEMM	E11.5AGM	2ug/ml	1.4425	0.2042	36	7.06	<.0001
**Colony*Condit*Treatm**	CFU-GEMM	E11.5AGM	6ug/ml	0	.	.	.	.
**Colony*Condit*Treatm**	CFU-GEMM	HE+PSCs	0ug/ml	0	.	.	.	.
**Colony*Condit*Treatm**	CFU-GEMM	HE+PSCs	2ug/ml	0	.	.	.	.
**Colony*Condit*Treatm**	CFU-GEMM	HE+PSCs	6ug/ml	0	.	.	.	.
**Colony*Condit*Treatm**	CFU-GM	AoE+PSCs	0ug/ml	0	.	.	.	.
**Colony*Condit*Treatm**	CFU-GM	AoE+PSCs	2ug/ml	0	.	.	.	.
**Colony*Condit*Treatm**	CFU-GM	AoE+PSCs	6ug/ml	0	.	.	.	.
**Colony*Condit*Treatm**	CFU-GM	E11.5AGM	0ug/ml	0	.	.	.	.
**Colony*Condit*Treatm**	CFU-GM	E11.5AGM	2ug/ml	0	.	.	.	.
**Colony*Condit*Treatm**	CFU-GM	E11.5AGM	6ug/ml	0	.	.	.	.
**Colony*Condit*Treatm**	CFU-GM	HE+PSCs	0ug/ml	0	.	.	.	.
**Colony*Condit*Treatm**	CFU-GM	HE+PSCs	2ug/ml	0	.	.	.	.
**Colony*Condit*Treatm**	CFU-GM	HE+PSCs	6ug/ml	0	.	.	.	.

Type III Tests of Fixed Effects				
**Effect**	**Num DF**	**Den DF**	**F Value**	**Pr > F**
**Colony_Type**	2	36	1907.40	<.0001
**Condition**	2	36	78.50	<.0001
**Colony_Typ*Condition**	4	36	8.28	<.0001
**Treatment**	2	36	175.42	<.0001
**Colony_Typ*Treatment**	4	36	2.51	0.0585

The GLIMMIX Procedure

Type III Tests of Fixed Effects				
Effect	Num DF	Den DF	F Value	Pr > F
Condition*Treatment	4	36	1.94	0.1254
Colony*Condit*Treatm	8	36	10.21	<.0001

Condition*Treatment Least Squares Means													
Condition	Treatment	Estimate	Standard Error	DF	t Value	Pr > |t|	Alpha	Lower	Upper	Mean	Standard Error Mean	Lower Mean	Upper Mean
AoE+PSCs	0ug/ml	5.1058	0.08805	36	57.98	<.0001	0.05	4.9272	5.2844	164.98	14.5273	138.00	197.24
AoE+PSCs	2ug/ml	4.4171	0.09267	36	47.66	<.0001	0.05	4.2292	4.6051	82.8569	7.6787	68.6597	99.9897
AoE+PSCs	6ug/ml	3.6909	0.1016	36	36.31	<.0001	0.05	3.4848	3.8971	40.0816	4.0739	32.6153	49.2572
E11.5AGM	0ug/ml	5.7474	0.08549	36	67.23	<.0001	0.05	5.5740	5.9208	313.38	26.7905	263.50	372.71
E11.5AGM	2ug/ml	5.3000	0.08662	36	61.19	<.0001	0.05	5.1243	5.4756	200.33	17.3526	168.06	238.80
E11.5AGM	6ug/ml	4.3217	0.09365	36	46.15	<.0001	0.05	4.1318	4.5117	75.3202	7.0534	62.2916	91.0739
HE+PSCs	0ug/ml	5.0281	0.08823	36	56.98	<.0001	0.05	4.8491	5.2070	152.64	13.4679	127.63	182.55
HE+PSCs	2ug/ml	4.1381	0.09555	36	43.31	<.0001	0.05	3.9443	4.3318	62.6808	5.9894	51.6382	76.0847
HE+PSCs	6ug/ml	3.5896	0.1015	36	35.36	<.0001	0.05	3.3838	3.7955	36.2205	3.6765	29.4816	44.4998

Differences of Condition*Treatment Least Squares Means											
Condition	Treatment	_Condition	_Treatment	Estimate	Standard Error	DF	t Value	Pr > |t|	Alpha	Lower	Upper
AoE+PSCs	0ug/ml	AoE+PSCs	2ug/ml	0.6887	0.1278	36	5.39	<.0001	0.05	0.4295	0.9480
AoE+PSCs	0ug/ml	AoE+PSCs	6ug/ml	1.4149	0.1345	36	10.52	<.0001	0.05	1.1422	1.6876
AoE+PSCs	0ug/ml	E11.5AGM	0ug/ml	-0.6416	0.1227	36	-5.23	<.0001	0.05	-0.8905	-0.3927
AoE+PSCs	0ug/ml	E11.5AGM	2ug/ml	-0.1941	0.1235	36	-1.57	0.1248	0.05	-0.4446	0.05637
AoE+PSCs	0ug/ml	E11.5AGM	6ug/ml	0.7841	0.1285	36	6.10	<.0001	0.05	0.5234	1.0448
AoE+PSCs	0ug/ml	HE+PSCs	0ug/ml	0.07776	0.1247	36	0.62	0.5367	0.05	-0.1751	0.3306

The GLIMMIX Procedure

Differences of Condition*Treatment Least Squares Means

Condition	Treatment	_Condition	_Treatment	Estimate	Standard Error	DF	t Value	Pr > |t|	Alpha	Lower	Upper
AoE+PSCs	0ug/ml	HE+PSCs	2ug/ml	0.9678	0.1299	36	7.45	<.0001	0.05	0.7043	1.2313
AoE+PSCs	0ug/ml	HE+PSCs	6ug/ml	1.5162	0.1344	36	11.28	<.0001	0.05	1.2437	1.7887
AoE+PSCs	2ug/ml	AoE+PSCs	6ug/ml	0.7262	0.1375	36	5.28	<.0001	0.05	0.4473	1.0051
AoE+PSCs	2ug/ml	E11.5AGM	0ug/ml	-1.3303	0.1261	36	-10.55	<.0001	0.05	-1.5860	-1.0746
AoE+PSCs	2ug/ml	E11.5AGM	2ug/ml	-0.8829	0.1269	36	-6.96	<.0001	0.05	-1.1401	-0.6256
AoE+PSCs	2ug/ml	E11.5AGM	6ug/ml	0.09537	0.1317	36	0.72	0.4738	0.05	-0.1718	0.3625
AoE+PSCs	2ug/ml	HE+PSCs	0ug/ml	-0.6109	0.1280	36	-4.77	<.0001	0.05	-0.8705	-0.3514
AoE+PSCs	2ug/ml	HE+PSCs	2ug/ml	0.2791	0.1331	36	2.10	0.0431	0.05	0.009119	0.5490
AoE+PSCs	2ug/ml	HE+PSCs	6ug/ml	0.8275	0.1374	36	6.02	<.0001	0.05	0.5488	1.1061
AoE+PSCs	6ug/ml	E11.5AGM	0ug/ml	-2.0565	0.1328	36	-15.48	<.0001	0.05	-2.3259	-1.7871
AoE+PSCs	6ug/ml	E11.5AGM	2ug/ml	-1.6090	0.1335	36	-12.05	<.0001	0.05	-1.8799	-1.3382
AoE+PSCs	6ug/ml	E11.5AGM	6ug/ml	-0.6308	0.1382	36	-4.57	<.0001	0.05	-0.9111	-0.3506
AoE+PSCs	6ug/ml	HE+PSCs	0ug/ml	-1.3371	0.1346	36	-9.93	<.0001	0.05	-1.6101	-1.0642
AoE+PSCs	6ug/ml	HE+PSCs	2ug/ml	-0.4471	0.1395	36	-3.21	0.0028	0.05	-0.7300	-0.1642
AoE+PSCs	6ug/ml	HE+PSCs	6ug/ml	0.1013	0.1436	36	0.71	0.4850	0.05	-0.1899	0.3925
E11.5AGM	0ug/ml	E11.5AGM	2ug/ml	0.4474	0.1217	36	3.68	0.0008	0.05	0.2006	0.6943
E11.5AGM	0ug/ml	E11.5AGM	6ug/ml	1.4257	0.1268	36	11.24	<.0001	0.05	1.1685	1.6828
E11.5AGM	0ug/ml	HE+PSCs	0ug/ml	0.7194	0.1229	36	5.86	<.0001	0.05	0.4702	0.9685
E11.5AGM	0ug/ml	HE+PSCs	2ug/ml	1.6094	0.1282	36	12.55	<.0001	0.05	1.3493	1.8694
E11.5AGM	0ug/ml	HE+PSCs	6ug/ml	2.1578	0.1327	36	16.26	<.0001	0.05	1.8886	2.4269
E11.5AGM	2ug/ml	E11.5AGM	6ug/ml	0.9782	0.1276	36	7.67	<.0001	0.05	0.7195	1.2369
E11.5AGM	2ug/ml	HE+PSCs	0ug/ml	0.2719	0.1236	36	2.20	0.0344	0.05	0.02114	0.5227
E11.5AGM	2ug/ml	HE+PSCs	2ug/ml	1.1619	0.1290	36	9.01	<.0001	0.05	0.9003	1.4235
E11.5AGM	2ug/ml	HE+PSCs	6ug/ml	1.7103	0.1334	36	12.82	<.0001	0.05	1.4397	1.9810

The GLIMMIX Procedure

Differences of Condition*Treatment Least Squares Means											
Condition	Treatment	_Condition	_Treatment	Estimate	Standard Error	DF	t Value	Pr > |t|	Alpha	Lower	Upper
E11.5AGM	6ug/ml	HE+PSCs	0ug/ml	-0.7063	0.1287	36	-5.49	<.0001	0.05	-0.9673	-0.4454
E11.5AGM	6ug/ml	HE+PSCs	2ug/ml	0.1837	0.1338	36	1.37	0.1782	0.05	-0.08762	0.4550
E11.5AGM	6ug/ml	HE+PSCs	6ug/ml	0.7321	0.1381	36	5.30	<.0001	0.05	0.4521	1.0121
HE+PSCs	0ug/ml	HE+PSCs	2ug/ml	0.8900	0.1301	36	6.84	<.0001	0.05	0.6262	1.1538
HE+PSCs	0ug/ml	HE+PSCs	6ug/ml	1.4384	0.1345	36	10.70	<.0001	0.05	1.1657	1.7112
HE+PSCs	2ug/ml	HE+PSCs	6ug/ml	0.5484	0.1394	36	3.94	0.0004	0.05	0.2658	0.8311

Colony*Condit*Treatm Least Squares Means														
Colony_Type	Condition	Treatment	Estimate	Standard Error	DF	t Value	Pr > |t|	Alpha	Lower	Upper	Mean	Standard Error Mean	Lower Mean	Upper Mean
BFU-E	AoE+PSCs	0ug/ml	4.4869	0.1031	36	43.54	<.0001	0.05	4.2779	4.6959	88.8465	9.1567	72.0883	109.50
BFU-E	AoE+PSCs	2ug/ml	3.7457	0.1211	36	30.93	<.0001	0.05	3.5002	3.9913	42.3407	5.1272	33.1207	54.1273
BFU-E	AoE+PSCs	6ug/ml	3.1521	0.1449	36	21.75	<.0001	0.05	2.8581	3.4460	23.3842	3.3890	17.4291	31.3741
BFU-E	E11.5AGM	0ug/ml	5.3465	0.09205	36	58.08	<.0001	0.05	5.1598	5.5332	209.87	19.3190	174.13	252.95
BFU-E	E11.5AGM	2ug/ml	4.8875	0.09693	36	50.42	<.0001	0.05	4.6909	5.0841	132.62	12.8546	108.95	161.43
BFU-E	E11.5AGM	6ug/ml	3.7258	0.1218	36	30.58	<.0001	0.05	3.4788	3.9729	41.5060	5.0567	32.4194	53.1396
BFU-E	HE+PSCs	0ug/ml	4.4738	0.1033	36	43.30	<.0001	0.05	4.2642	4.6834	87.6900	9.0612	71.1108	108.13
BFU-E	HE+PSCs	2ug/ml	3.7949	0.1197	36	31.72	<.0001	0.05	3.5522	4.0375	44.4729	5.3212	34.8906	56.6868
BFU-E	HE+PSCs	6ug/ml	3.2689	0.1392	36	23.49	<.0001	0.05	2.9866	3.5511	26.2814	3.6575	19.8186	34.8518
CFU-GEMM	AoE+PSCs	0ug/ml	4.5796	0.1014	36	45.14	<.0001	0.05	4.3738	4.7853	97.4723	9.8883	79.3465	119.74
CFU-GEMM	AoE+PSCs	2ug/ml	4.0038	0.1136	36	35.25	<.0001	0.05	3.7735	4.2342	54.8081	6.2252	43.5315	69.0058
CFU-GEMM	AoE+PSCs	6ug/ml	3.1798	0.1436	36	22.14	<.0001	0.05	2.8885	3.4710	24.0409	3.4521	17.9670	32.1681
CFU-GEMM	E11.5AGM	0ug/ml	5.2269	0.09314	36	56.12	<.0001	0.05	5.0380	5.4158	186.22	17.3444	154.16	224.94
CFU-GEMM	E11.5AGM	2ug/ml	5.0211	0.09530	36	52.69	<.0001	0.05	4.8278	5.2144	151.58	14.4450	124.94	183.90

The GLIMMIX Procedure

Colony*Condit*Treatm Least Squares Means														
Colony_Type	Condition	Treatment	Estimate	Standard Error	DF	t Value	Pr > |t|	Alpha	Lower	Upper	Mean	Standard Error Mean	Lower Mean	Upper Mean
CFU-GEMM	E11.5AGM	6ug/ml	3.7947	0.1196	36	31.72	<.0001	0.05	3.5521	4.0374	44.4661	5.3200	34.8858	56.6773
CFU-GEMM	HE+PSCs	0ug/ml	4.5539	0.1019	36	44.68	<.0001	0.05	4.3472	4.7606	94.9998	9.6817	77.2608	116.81
CFU-GEMM	HE+PSCs	2ug/ml	3.4220	0.1329	36	25.75	<.0001	0.05	3.1525	3.6916	30.6319	4.0710	23.3947	40.1080
CFU-GEMM	HE+PSCs	6ug/ml	3.2438	0.1403	36	23.12	<.0001	0.05	2.9593	3.5283	25.6309	3.5957	19.2841	34.0665
CFU-GM	AoE+PSCs	0ug/ml	6.2510	0.08677	36	72.04	<.0001	0.05	6.0750	6.4270	518.53	44.9940	434.86	618.31
CFU-GM	AoE+PSCs	2ug/ml	5.5018	0.09084	36	60.56	<.0001	0.05	5.3175	5.6860	245.12	22.2672	203.88	294.71
CFU-GM	AoE+PSCs	6ug/ml	4.7409	0.09899	36	47.89	<.0001	0.05	4.5402	4.9417	114.54	11.3390	93.7064	140.01
CFU-GM	E11.5AGM	0ug/ml	6.6688	0.08549	36	78.00	<.0001	0.05	6.4954	6.8422	787.47	67.3244	662.11	936.56
CFU-GM	E11.5AGM	2ug/ml	5.9913	0.08786	36	68.19	<.0001	0.05	5.8131	6.1695	399.94	35.1391	334.66	477.95
CFU-GM	E11.5AGM	6ug/ml	5.4447	0.09127	36	59.66	<.0001	0.05	5.2596	5.6298	231.52	21.1300	192.40	278.60
CFU-GM	HE+PSCs	0ug/ml	6.0565	0.08757	36	69.17	<.0001	0.05	5.8789	6.2341	426.88	37.3801	357.42	509.84
CFU-GM	HE+PSCs	2ug/ml	5.1972	0.09345	36	55.62	<.0001	0.05	5.0077	5.3868	180.77	16.8929	149.56	218.49
CFU-GM	HE+PSCs	6ug/ml	4.2562	0.1078	36	39.49	<.0001	0.05	4.0376	4.4748	70.5425	7.6024	56.6928	87.7755

The GLIMMIX Procedure

Differences of Colony*Condit*Treatm Least Squares Means													
Colony_Type	Condition	Treatment	_Colony_Type	_Condition	_Treatment	Estimate	Standard Error	DF	t Value	Pr > |t|	Alpha	Lower	Upper
BFU-E	AoE+PSCs	0ug/ml	BFU-E	AoE+PSCs	2ug/ml	0.7412	0.1590	36	4.66	<.0001	0.05	0.4187	1.0636
BFU-E	AoE+PSCs	0ug/ml	BFU-E	AoE+PSCs	6ug/ml	1.3348	0.1778	36	7.51	<.0001	0.05	0.9742	1.6955
BFU-E	AoE+PSCs	0ug/ml	BFU-E	E11.5AGM	0ug/ml	-0.8596	0.1382	36	-6.22	<.0001	0.05	-1.1398	-0.5793
BFU-E	AoE+PSCs	0ug/ml	BFU-E	E11.5AGM	2ug/ml	-0.4006	0.1415	36	-2.83	0.0075	0.05	-0.6875	-0.1136
BFU-E	AoE+PSCs	0ug/ml	BFU-E	E11.5AGM	6ug/ml	0.7611	0.1596	36	4.77	<.0001	0.05	0.4374	1.0847
BFU-E	AoE+PSCs	0ug/ml	BFU-E	HE+PSCs	0ug/ml	0.01310	0.1459	36	0.09	0.9290	0.05	-0.2829	0.3091
BFU-E	AoE+PSCs	0ug/ml	BFU-E	HE+PSCs	2ug/ml	0.6920	0.1579	36	4.38	<.0001	0.05	0.3718	1.0123
BFU-E	AoE+PSCs	0ug/ml	BFU-E	HE+PSCs	6ug/ml	1.2180	0.1732	36	7.03	<.0001	0.05	0.8669	1.5692
BFU-E	AoE+PSCs	0ug/ml	CFU-GEMM	AoE+PSCs	0ug/ml	-0.09266	0.08446	36	-1.10	0.2799	0.05	-0.2639	0.07863
BFU-E	AoE+PSCs	0ug/ml	CFU-GEMM	AoE+PSCs	2ug/ml	0.4831	0.1534	36	3.15	0.0033	0.05	0.1720	0.7941
BFU-E	AoE+PSCs	0ug/ml	CFU-GEMM	AoE+PSCs	6ug/ml	1.3072	0.1767	36	7.40	<.0001	0.05	0.9487	1.6656
BFU-E	AoE+PSCs	0ug/ml	CFU-GEMM	E11.5AGM	0ug/ml	-0.7400	0.1389	36	-5.33	<.0001	0.05	-1.0217	-0.4583
BFU-E	AoE+PSCs	0ug/ml	CFU-GEMM	E11.5AGM	2ug/ml	-0.5342	0.1404	36	-3.81	0.0005	0.05	-0.8189	-0.2495
BFU-E	AoE+PSCs	0ug/ml	CFU-GEMM	E11.5AGM	6ug/ml	0.6922	0.1579	36	4.38	<.0001	0.05	0.3719	1.0124
BFU-E	AoE+PSCs	0ug/ml	CFU-GEMM	HE+PSCs	0ug/ml	-0.06696	0.1449	36	-0.46	0.6469	0.05	-0.3609	0.2270
BFU-E	AoE+PSCs	0ug/ml	CFU-GEMM	HE+PSCs	2ug/ml	1.0649	0.1682	36	6.33	<.0001	0.05	0.7238	1.4059
BFU-E	AoE+PSCs	0ug/ml	CFU-GEMM	HE+PSCs	6ug/ml	1.2431	0.1741	36	7.14	<.0001	0.05	0.8901	1.5961
BFU-E	AoE+PSCs	0ug/ml	CFU-GM	AoE+PSCs	0ug/ml	-1.7641	0.06611	36	-26.68	<.0001	0.05	-1.8982	-1.6300
BFU-E	AoE+PSCs	0ug/ml	CFU-GM	AoE+PSCs	2ug/ml	-1.0148	0.1374	36	-7.39	<.0001	0.05	-1.2935	-0.7362
BFU-E	AoE+PSCs	0ug/ml	CFU-GM	AoE+PSCs	6ug/ml	-0.2540	0.1429	36	-1.78	0.0839	0.05	-0.5438	0.03578
BFU-E	AoE+PSCs	0ug/ml	CFU-GM	E11.5AGM	0ug/ml	-2.1819	0.1339	36	-16.29	<.0001	0.05	-2.4535	-1.9103
BFU-E	AoE+PSCs	0ug/ml	CFU-GM	E11.5AGM	2ug/ml	-1.5044	0.1354	36	-11.11	<.0001	0.05	-1.7791	-1.2297
BFU-E	AoE+PSCs	0ug/ml	CFU-GM	E11.5AGM	6ug/ml	-0.9578	0.1377	36	-6.96	<.0001	0.05	-1.2370	-0.6786
BFU-E	AoE+PSCs	0ug/ml	CFU-GM	HE+PSCs	0ug/ml	-1.5696	0.1352	36	-11.61	<.0001	0.05	-1.8439	-1.2953

The GLIMMIX Procedure

Differences of Colony*Condit*Treatm Least Squares Means													
Colony_Type	Condition	Treatment	_Colony_Type	_Condition	_Treatment	Estimate	Standard Error	DF	t Value	Pr > |t|	Alpha	Lower	Upper
BFU-E	AoE+PSCs	0ug/ml	CFU-GM	HE+PSCs	2ug/ml	-0.7103	0.1391	36	-5.11	<.0001	0.05	-0.9925	-0.4282
BFU-E	AoE+PSCs	0ug/ml	CFU-GM	HE+PSCs	6ug/ml	0.2307	0.1491	36	1.55	0.1306	0.05	-0.07170	0.5331
BFU-E	AoE+PSCs	2ug/ml	BFU-E	AoE+PSCs	6ug/ml	0.5937	0.1888	36	3.14	0.0033	0.05	0.2107	0.9767
BFU-E	AoE+PSCs	2ug/ml	BFU-E	E11.5AGM	0ug/ml	-1.6007	0.1521	36	-10.52	<.0001	0.05	-1.9092	-1.2923
BFU-E	AoE+PSCs	2ug/ml	BFU-E	E11.5AGM	2ug/ml	-1.1417	0.1551	36	-7.36	<.0001	0.05	-1.4563	-0.8272
BFU-E	AoE+PSCs	2ug/ml	BFU-E	E11.5AGM	6ug/ml	0.01991	0.1718	36	0.12	0.9084	0.05	-0.3284	0.3683
BFU-E	AoE+PSCs	2ug/ml	BFU-E	HE+PSCs	0ug/ml	-0.7281	0.1592	36	-4.57	<.0001	0.05	-1.0509	-0.4052
BFU-E	AoE+PSCs	2ug/ml	BFU-E	HE+PSCs	2ug/ml	-0.04913	0.1702	36	-0.29	0.7745	0.05	-0.3944	0.2961
BFU-E	AoE+PSCs	2ug/ml	BFU-E	HE+PSCs	6ug/ml	0.4769	0.1844	36	2.59	0.0139	0.05	0.1028	0.8509
BFU-E	AoE+PSCs	2ug/ml	CFU-GEMM	AoE+PSCs	0ug/ml	-0.8338	0.1580	36	-5.28	<.0001	0.05	-1.1542	-0.5134
BFU-E	AoE+PSCs	2ug/ml	CFU-GEMM	AoE+PSCs	2ug/ml	-0.2581	0.1172	36	-2.20	0.0342	0.05	-0.4958	-0.02038
BFU-E	AoE+PSCs	2ug/ml	CFU-GEMM	AoE+PSCs	6ug/ml	0.5660	0.1878	36	3.01	0.0047	0.05	0.1851	0.9469
BFU-E	AoE+PSCs	2ug/ml	CFU-GEMM	E11.5AGM	0ug/ml	-1.4812	0.1528	36	-9.70	<.0001	0.05	-1.7910	-1.1713
BFU-E	AoE+PSCs	2ug/ml	CFU-GEMM	E11.5AGM	2ug/ml	-1.2754	0.1541	36	-8.28	<.0001	0.05	-1.5879	-0.9628
BFU-E	AoE+PSCs	2ug/ml	CFU-GEMM	E11.5AGM	6ug/ml	-0.04898	0.1702	36	-0.29	0.7752	0.05	-0.3942	0.2962
BFU-E	AoE+PSCs	2ug/ml	CFU-GEMM	HE+PSCs	0ug/ml	-0.8081	0.1583	36	-5.11	<.0001	0.05	-1.1291	-0.4871
BFU-E	AoE+PSCs	2ug/ml	CFU-GEMM	HE+PSCs	2ug/ml	0.3237	0.1798	36	1.80	0.0802	0.05	-0.04091	0.6883
BFU-E	AoE+PSCs	2ug/ml	CFU-GEMM	HE+PSCs	6ug/ml	0.5020	0.1853	36	2.71	0.0103	0.05	0.1262	0.8777
BFU-E	AoE+PSCs	2ug/ml	CFU-GM	AoE+PSCs	0ug/ml	-2.5053	0.1490	36	-16.82	<.0001	0.05	-2.8074	-2.2031
BFU-E	AoE+PSCs	2ug/ml	CFU-GM	AoE+PSCs	2ug/ml	-1.7560	0.09534	36	-18.42	<.0001	0.05	-1.9494	-1.5627
BFU-E	AoE+PSCs	2ug/ml	CFU-GM	AoE+PSCs	6ug/ml	-0.9952	0.1564	36	-6.36	<.0001	0.05	-1.3124	-0.6780
BFU-E	AoE+PSCs	2ug/ml	CFU-GM	E11.5AGM	0ug/ml	-2.9231	0.1482	36	-19.72	<.0001	0.05	-3.2237	-2.6224
BFU-E	AoE+PSCs	2ug/ml	CFU-GM	E11.5AGM	2ug/ml	-2.2456	0.1496	36	-15.01	<.0001	0.05	-2.5490	-1.9421
BFU-E	AoE+PSCs	2ug/ml	CFU-GM	E11.5AGM	6ug/ml	-1.6989	0.1516	36	-11.20	<.0001	0.05	-2.0064	-1.3914

The GLIMMIX Procedure

Differences of Colony*Condit*Treatm Least Squares Means													
Colony_Type	Condition	Treatment	_Colony_Type	_Condition	_Treatment	Estimate	Standard Error	DF	t Value	Pr > |t|	Alpha	Lower	Upper
BFU-E	AoE+PSCs	2ug/ml	CFU-GM	HE+PSCs	0ug/ml	-2.3108	0.1494	36	-15.46	<.0001	0.05	-2.6138	-2.0077
BFU-E	AoE+PSCs	2ug/ml	CFU-GM	HE+PSCs	2ug/ml	-1.4515	0.1529	36	-9.49	<.0001	0.05	-1.7617	-1.1413
BFU-E	AoE+PSCs	2ug/ml	CFU-GM	HE+PSCs	6ug/ml	-0.5105	0.1621	36	-3.15	0.0033	0.05	-0.8391	-0.1818
BFU-E	AoE+PSCs	6ug/ml	BFU-E	E11.5AGM	0ug/ml	-2.1944	0.1717	36	-12.78	<.0001	0.05	-2.5426	-1.8462
BFU-E	AoE+PSCs	6ug/ml	BFU-E	E11.5AGM	2ug/ml	-1.7354	0.1744	36	-9.95	<.0001	0.05	-2.0890	-1.3818
BFU-E	AoE+PSCs	6ug/ml	BFU-E	E11.5AGM	6ug/ml	-0.5738	0.1893	36	-3.03	0.0045	0.05	-0.9577	-0.1898
BFU-E	AoE+PSCs	6ug/ml	BFU-E	HE+PSCs	0ug/ml	-1.3217	0.1780	36	-7.43	<.0001	0.05	-1.6827	-0.9608
BFU-E	AoE+PSCs	6ug/ml	BFU-E	HE+PSCs	2ug/ml	-0.6428	0.1879	36	-3.42	0.0016	0.05	-1.0239	-0.2617
BFU-E	AoE+PSCs	6ug/ml	BFU-E	HE+PSCs	6ug/ml	-0.1168	0.2009	36	-0.58	0.5646	0.05	-0.5242	0.2906
BFU-E	AoE+PSCs	6ug/ml	CFU-GEMM	AoE+PSCs	0ug/ml	-1.4275	0.1769	36	-8.07	<.0001	0.05	-1.7863	-1.0687
BFU-E	AoE+PSCs	6ug/ml	CFU-GEMM	AoE+PSCs	2ug/ml	-0.8518	0.1841	36	-4.63	<.0001	0.05	-1.2252	-0.4784
BFU-E	AoE+PSCs	6ug/ml	CFU-GEMM	AoE+PSCs	6ug/ml	-0.02770	0.1666	36	-0.17	0.8689	0.05	-0.3657	0.3103
BFU-E	AoE+PSCs	6ug/ml	CFU-GEMM	E11.5AGM	0ug/ml	-2.0749	0.1723	36	-12.04	<.0001	0.05	-2.4242	-1.7255
BFU-E	AoE+PSCs	6ug/ml	CFU-GEMM	E11.5AGM	2ug/ml	-1.8690	0.1734	36	-10.78	<.0001	0.05	-2.2208	-1.5173
BFU-E	AoE+PSCs	6ug/ml	CFU-GEMM	E11.5AGM	6ug/ml	-0.6427	0.1879	36	-3.42	0.0016	0.05	-1.0238	-0.2616
BFU-E	AoE+PSCs	6ug/ml	CFU-GEMM	HE+PSCs	0ug/ml	-1.4018	0.1772	36	-7.91	<.0001	0.05	-1.7611	-1.0425
BFU-E	AoE+PSCs	6ug/ml	CFU-GEMM	HE+PSCs	2ug/ml	-0.2700	0.1966	36	-1.37	0.1782	0.05	-0.6688	0.1288
BFU-E	AoE+PSCs	6ug/ml	CFU-GEMM	HE+PSCs	6ug/ml	-0.09174	0.2017	36	-0.45	0.6519	0.05	-0.5007	0.3172
BFU-E	AoE+PSCs	6ug/ml	CFU-GM	AoE+PSCs	0ug/ml	-3.0989	0.1689	36	-18.35	<.0001	0.05	-3.4415	-2.7564
BFU-E	AoE+PSCs	6ug/ml	CFU-GM	AoE+PSCs	2ug/ml	-2.3497	0.1710	36	-13.74	<.0001	0.05	-2.6966	-2.0028
BFU-E	AoE+PSCs	6ug/ml	CFU-GM	AoE+PSCs	6ug/ml	-1.5889	0.1302	36	-12.20	<.0001	0.05	-1.8529	-1.3248
BFU-E	AoE+PSCs	6ug/ml	CFU-GM	E11.5AGM	0ug/ml	-3.5168	0.1683	36	-20.90	<.0001	0.05	-3.8580	-3.1755
BFU-E	AoE+PSCs	6ug/ml	CFU-GM	E11.5AGM	2ug/ml	-2.8392	0.1695	36	-16.75	<.0001	0.05	-3.1830	-2.4955
BFU-E	AoE+PSCs	6ug/ml	CFU-GM	E11.5AGM	6ug/ml	-2.2926	0.1713	36	-13.39	<.0001	0.05	-2.6399	-1.9453

The GLIMMIX Procedure

Differences of Colony*Condit*Treatm Least Squares Means													
Colony_Type	Condition	Treatment	_Colony_Type	_Condition	_Treatment	Estimate	Standard Error	DF	t Value	Pr > |t|	Alpha	Lower	Upper
BFU-E	AoE+PSCs	6ug/ml	CFU-GM	HE+PSCs	0ug/ml	-2.9044	0.1693	36	-17.15	<.0001	0.05	-3.2479	-2.5610
BFU-E	AoE+PSCs	6ug/ml	CFU-GM	HE+PSCs	2ug/ml	-2.0452	0.1724	36	-11.86	<.0001	0.05	-2.3949	-1.6955
BFU-E	AoE+PSCs	6ug/ml	CFU-GM	HE+PSCs	6ug/ml	-1.1042	0.1805	36	-6.12	<.0001	0.05	-1.4703	-0.7380
BFU-E	E11.5AGM	0ug/ml	BFU-E	E11.5AGM	2ug/ml	0.4590	0.1337	36	3.43	0.0015	0.05	0.1879	0.7301
BFU-E	E11.5AGM	0ug/ml	BFU-E	E11.5AGM	6ug/ml	1.6207	0.1527	36	10.61	<.0001	0.05	1.3110	1.9303
BFU-E	E11.5AGM	0ug/ml	BFU-E	HE+PSCs	0ug/ml	0.8727	0.1384	36	6.31	<.0001	0.05	0.5920	1.1534
BFU-E	E11.5AGM	0ug/ml	BFU-E	HE+PSCs	2ug/ml	1.5516	0.1510	36	10.28	<.0001	0.05	1.2454	1.8578
BFU-E	E11.5AGM	0ug/ml	BFU-E	HE+PSCs	6ug/ml	2.0776	0.1669	36	12.45	<.0001	0.05	1.7392	2.4160
BFU-E	E11.5AGM	0ug/ml	CFU-GEMM	AoE+PSCs	0ug/ml	0.7669	0.1370	36	5.60	<.0001	0.05	0.4891	1.0447
BFU-E	E11.5AGM	0ug/ml	CFU-GEMM	AoE+PSCs	2ug/ml	1.3427	0.1462	36	9.18	<.0001	0.05	1.0462	1.6392
BFU-E	E11.5AGM	0ug/ml	CFU-GEMM	AoE+PSCs	6ug/ml	2.1667	0.1706	36	12.70	<.0001	0.05	1.8208	2.5127
BFU-E	E11.5AGM	0ug/ml	CFU-GEMM	E11.5AGM	0ug/ml	0.1196	0.05810	36	2.06	0.0469	0.05	0.001738	0.2374
BFU-E	E11.5AGM	0ug/ml	CFU-GEMM	E11.5AGM	2ug/ml	0.3254	0.1325	36	2.46	0.0190	0.05	0.05668	0.5941
BFU-E	E11.5AGM	0ug/ml	CFU-GEMM	E11.5AGM	6ug/ml	1.5518	0.1510	36	10.28	<.0001	0.05	1.2456	1.8579
BFU-E	E11.5AGM	0ug/ml	CFU-GEMM	HE+PSCs	0ug/ml	0.7926	0.1373	36	5.77	<.0001	0.05	0.5141	1.0711
BFU-E	E11.5AGM	0ug/ml	CFU-GEMM	HE+PSCs	2ug/ml	1.9245	0.1617	36	11.90	<.0001	0.05	1.5966	2.2523
BFU-E	E11.5AGM	0ug/ml	CFU-GEMM	HE+PSCs	6ug/ml	2.1027	0.1678	36	12.53	<.0001	0.05	1.7624	2.4430
BFU-E	E11.5AGM	0ug/ml	CFU-GM	AoE+PSCs	0ug/ml	-0.9045	0.1265	36	-7.15	<.0001	0.05	-1.1611	-0.6479
BFU-E	E11.5AGM	0ug/ml	CFU-GM	AoE+PSCs	2ug/ml	-0.1553	0.1293	36	-1.20	0.2378	0.05	-0.4175	0.1070
BFU-E	E11.5AGM	0ug/ml	CFU-GM	AoE+PSCs	6ug/ml	0.6056	0.1352	36	4.48	<.0001	0.05	0.3314	0.8797
BFU-E	E11.5AGM	0ug/ml	CFU-GM	E11.5AGM	0ug/ml	-1.3223	0.04484	36	-29.49	<.0001	0.05	-1.4133	-1.2314
BFU-E	E11.5AGM	0ug/ml	CFU-GM	E11.5AGM	2ug/ml	-0.6448	0.1273	36	-5.07	<.0001	0.05	-0.9029	-0.3867
BFU-E	E11.5AGM	0ug/ml	CFU-GM	E11.5AGM	6ug/ml	-0.09818	0.1296	36	-0.76	0.4537	0.05	-0.3611	0.1647
BFU-E	E11.5AGM	0ug/ml	CFU-GM	HE+PSCs	0ug/ml	-0.7100	0.1270	36	-5.59	<.0001	0.05	-0.9677	-0.4523

The GLIMMIX Procedure

Differences of Colony*Condit*Treatm Least Squares Means													
Colony_Type	Condition	Treatment	_Colony_Type	_Condition	_Treatment	Estimate	Standard Error	DF	t Value	Pr > |t|	Alpha	Lower	Upper
BFU-E	E11.5AGM	0ug/ml	CFU-GM	HE+PSCs	2ug/ml	0.1493	0.1312	36	1.14	0.2627	0.05	-0.1168	0.4153
BFU-E	E11.5AGM	0ug/ml	CFU-GM	HE+PSCs	6ug/ml	1.0903	0.1417	36	7.69	<.0001	0.05	0.8028	1.3777
BFU-E	E11.5AGM	2ug/ml	BFU-E	E11.5AGM	6ug/ml	1.1616	0.1557	36	7.46	<.0001	0.05	0.8459	1.4774
BFU-E	E11.5AGM	2ug/ml	BFU-E	HE+PSCs	0ug/ml	0.4137	0.1417	36	2.92	0.0060	0.05	0.1263	0.7010
BFU-E	E11.5AGM	2ug/ml	BFU-E	HE+PSCs	2ug/ml	1.0926	0.1540	36	7.10	<.0001	0.05	0.7803	1.4049
BFU-E	E11.5AGM	2ug/ml	BFU-E	HE+PSCs	6ug/ml	1.6186	0.1696	36	9.54	<.0001	0.05	1.2747	1.9626
BFU-E	E11.5AGM	2ug/ml	CFU-GEMM	AoE+PSCs	0ug/ml	0.3079	0.1403	36	2.19	0.0347	0.05	0.02336	0.5925
BFU-E	E11.5AGM	2ug/ml	CFU-GEMM	AoE+PSCs	2ug/ml	0.8837	0.1493	36	5.92	<.0001	0.05	0.5808	1.1865
BFU-E	E11.5AGM	2ug/ml	CFU-GEMM	AoE+PSCs	6ug/ml	1.7077	0.1732	36	9.86	<.0001	0.05	1.3564	2.0591
BFU-E	E11.5AGM	2ug/ml	CFU-GEMM	E11.5AGM	0ug/ml	-0.3394	0.1344	36	-2.53	0.0161	0.05	-0.6121	-0.06680
BFU-E	E11.5AGM	2ug/ml	CFU-GEMM	E11.5AGM	2ug/ml	-0.1336	0.06855	36	-1.95	0.0591	0.05	-0.2726	0.005415
BFU-E	E11.5AGM	2ug/ml	CFU-GEMM	E11.5AGM	6ug/ml	1.0928	0.1540	36	7.10	<.0001	0.05	0.7805	1.4050
BFU-E	E11.5AGM	2ug/ml	CFU-GEMM	HE+PSCs	0ug/ml	0.3336	0.1406	36	2.37	0.0232	0.05	0.04837	0.6189
BFU-E	E11.5AGM	2ug/ml	CFU-GEMM	HE+PSCs	2ug/ml	1.4654	0.1645	36	8.91	<.0001	0.05	1.1318	1.7991
BFU-E	E11.5AGM	2ug/ml	CFU-GEMM	HE+PSCs	6ug/ml	1.6437	0.1705	36	9.64	<.0001	0.05	1.2979	1.9895
BFU-E	E11.5AGM	2ug/ml	CFU-GM	AoE+PSCs	0ug/ml	-1.3635	0.1301	36	-10.48	<.0001	0.05	-1.6274	-1.0997
BFU-E	E11.5AGM	2ug/ml	CFU-GM	AoE+PSCs	2ug/ml	-0.6143	0.1328	36	-4.62	<.0001	0.05	-0.8837	-0.3449
BFU-E	E11.5AGM	2ug/ml	CFU-GM	AoE+PSCs	6ug/ml	0.1466	0.1385	36	1.06	0.2972	0.05	-0.1344	0.4275
BFU-E	E11.5AGM	2ug/ml	CFU-GM	E11.5AGM	0ug/ml	-1.7813	0.1292	36	-13.78	<.0001	0.05	-2.0435	-1.5192
BFU-E	E11.5AGM	2ug/ml	CFU-GM	E11.5AGM	2ug/ml	-1.1038	0.05777	36	-19.11	<.0001	0.05	-1.2210	-0.9866
BFU-E	E11.5AGM	2ug/ml	CFU-GM	E11.5AGM	6ug/ml	-0.5572	0.1331	36	-4.19	0.0002	0.05	-0.8272	-0.2872
BFU-E	E11.5AGM	2ug/ml	CFU-GM	HE+PSCs	0ug/ml	-1.1690	0.1306	36	-8.95	<.0001	0.05	-1.4339	-0.9041
BFU-E	E11.5AGM	2ug/ml	CFU-GM	HE+PSCs	2ug/ml	-0.3098	0.1346	36	-2.30	0.0273	0.05	-0.5828	-0.03669
BFU-E	E11.5AGM	2ug/ml	CFU-GM	HE+PSCs	6ug/ml	0.6313	0.1449	36	4.36	0.0001	0.05	0.3373	0.9252

The GLIMMIX Procedure

Differences of Colony*Condit*Treatm Least Squares Means													
Colony_Type	Condition	Treatment	_Colony_Type	_Condition	_Treatment	Estimate	Standard Error	DF	t Value	Pr > |t|	Alpha	Lower	Upper
BFU-E	E11.5AGM	6ug/ml	BFU-E	HE+PSCs	0ug/ml	-0.7480	0.1598	36	-4.68	<.0001	0.05	-1.0720	-0.4240
BFU-E	E11.5AGM	6ug/ml	BFU-E	HE+PSCs	2ug/ml	-0.06904	0.1707	36	-0.40	0.6884	0.05	-0.4153	0.2773
BFU-E	E11.5AGM	6ug/ml	BFU-E	HE+PSCs	6ug/ml	0.4570	0.1849	36	2.47	0.0183	0.05	0.08194	0.8320
BFU-E	E11.5AGM	6ug/ml	CFU-GEMM	AoE+PSCs	0ug/ml	-0.8537	0.1585	36	-5.39	<.0001	0.05	-1.1753	-0.5322
BFU-E	E11.5AGM	6ug/ml	CFU-GEMM	AoE+PSCs	2ug/ml	-0.2780	0.1666	36	-1.67	0.1038	0.05	-0.6158	0.05979
BFU-E	E11.5AGM	6ug/ml	CFU-GEMM	AoE+PSCs	6ug/ml	0.5461	0.1883	36	2.90	0.0063	0.05	0.1642	0.9280
BFU-E	E11.5AGM	6ug/ml	CFU-GEMM	E11.5AGM	0ug/ml	-1.5011	0.1534	36	-9.79	<.0001	0.05	-1.8121	-1.1901
BFU-E	E11.5AGM	6ug/ml	CFU-GEMM	E11.5AGM	2ug/ml	-1.2953	0.1547	36	-8.37	<.0001	0.05	-1.6090	-0.9816
BFU-E	E11.5AGM	6ug/ml	CFU-GEMM	E11.5AGM	6ug/ml	-0.06889	0.1239	36	-0.56	0.5816	0.05	-0.3201	0.1823
BFU-E	E11.5AGM	6ug/ml	CFU-GEMM	HE+PSCs	0ug/ml	-0.8280	0.1588	36	-5.21	<.0001	0.05	-1.1502	-0.5059
BFU-E	E11.5AGM	6ug/ml	CFU-GEMM	HE+PSCs	2ug/ml	0.3038	0.1803	36	1.69	0.1006	0.05	-0.06183	0.6694
BFU-E	E11.5AGM	6ug/ml	CFU-GEMM	HE+PSCs	6ug/ml	0.4820	0.1858	36	2.59	0.0136	0.05	0.1053	0.8588
BFU-E	E11.5AGM	6ug/ml	CFU-GM	AoE+PSCs	0ug/ml	-2.5252	0.1496	36	-16.88	<.0001	0.05	-2.8285	-2.2218
BFU-E	E11.5AGM	6ug/ml	CFU-GM	AoE+PSCs	2ug/ml	-1.7759	0.1520	36	-11.69	<.0001	0.05	-2.0841	-1.4677
BFU-E	E11.5AGM	6ug/ml	CFU-GM	AoE+PSCs	6ug/ml	-1.0151	0.1570	36	-6.47	<.0001	0.05	-1.3334	-0.6968
BFU-E	E11.5AGM	6ug/ml	CFU-GM	E11.5AGM	0ug/ml	-2.9430	0.1488	36	-19.77	<.0001	0.05	-3.2448	-2.6411
BFU-E	E11.5AGM	6ug/ml	CFU-GM	E11.5AGM	2ug/ml	-2.2655	0.1502	36	-15.08	<.0001	0.05	-2.5701	-1.9608
BFU-E	E11.5AGM	6ug/ml	CFU-GM	E11.5AGM	6ug/ml	-1.7188	0.09674	36	-17.77	<.0001	0.05	-1.9150	-1.5226
BFU-E	E11.5AGM	6ug/ml	CFU-GM	HE+PSCs	0ug/ml	-2.3307	0.1500	36	-15.53	<.0001	0.05	-2.6350	-2.0264
BFU-E	E11.5AGM	6ug/ml	CFU-GM	HE+PSCs	2ug/ml	-1.4714	0.1535	36	-9.58	<.0001	0.05	-1.7828	-1.1600
BFU-E	E11.5AGM	6ug/ml	CFU-GM	HE+PSCs	6ug/ml	-0.5304	0.1626	36	-3.26	0.0024	0.05	-0.8602	-0.2006
BFU-E	HE+PSCs	0ug/ml	BFU-E	HE+PSCs	2ug/ml	0.6789	0.1581	36	4.29	0.0001	0.05	0.3583	0.9996
BFU-E	HE+PSCs	0ug/ml	BFU-E	HE+PSCs	6ug/ml	1.2049	0.1733	36	6.95	<.0001	0.05	0.8534	1.5565
BFU-E	HE+PSCs	0ug/ml	CFU-GEMM	AoE+PSCs	0ug/ml	-0.1058	0.1448	36	-0.73	0.4699	0.05	-0.3994	0.1879

The GLIMMIX Procedure

Differences of Colony*Condit*Treatm Least Squares Means													
Colony_Type	Condition	Treatment	_Colony_Type	_Condition	_Treatment	Estimate	Standard Error	DF	t Value	Pr > |t|	Alpha	Lower	Upper
BFU-E	HE+PSCs	0ug/ml	CFU-GEMM	AoE+PSCs	2ug/ml	0.4700	0.1536	36	3.06	0.0042	0.05	0.1586	0.7814
BFU-E	HE+PSCs	0ug/ml	CFU-GEMM	AoE+PSCs	6ug/ml	1.2941	0.1769	36	7.31	<.0001	0.05	0.9353	1.6528
BFU-E	HE+PSCs	0ug/ml	CFU-GEMM	E11.5AGM	0ug/ml	-0.7531	0.1391	36	-5.41	<.0001	0.05	-1.0352	-0.4710
BFU-E	HE+PSCs	0ug/ml	CFU-GEMM	E11.5AGM	2ug/ml	-0.5473	0.1406	36	-3.89	0.0004	0.05	-0.8324	-0.2622
BFU-E	HE+PSCs	0ug/ml	CFU-GEMM	E11.5AGM	6ug/ml	0.6791	0.1581	36	4.30	0.0001	0.05	0.3585	0.9997
BFU-E	HE+PSCs	0ug/ml	CFU-GEMM	HE+PSCs	0ug/ml	-0.08007	0.08535	36	-0.94	0.3544	0.05	-0.2532	0.09303
BFU-E	HE+PSCs	0ug/ml	CFU-GEMM	HE+PSCs	2ug/ml	1.0518	0.1683	36	6.25	<.0001	0.05	0.7103	1.3932
BFU-E	HE+PSCs	0ug/ml	CFU-GEMM	HE+PSCs	6ug/ml	1.2300	0.1742	36	7.06	<.0001	0.05	0.8766	1.5834
BFU-E	HE+PSCs	0ug/ml	CFU-GM	AoE+PSCs	0ug/ml	-1.7772	0.1349	36	-13.17	<.0001	0.05	-2.0508	-1.5035
BFU-E	HE+PSCs	0ug/ml	CFU-GM	AoE+PSCs	2ug/ml	-1.0279	0.1376	36	-7.47	<.0001	0.05	-1.3070	-0.7489
BFU-E	HE+PSCs	0ug/ml	CFU-GM	AoE+PSCs	6ug/ml	-0.2671	0.1431	36	-1.87	0.0701	0.05	-0.5573	0.02309
BFU-E	HE+PSCs	0ug/ml	CFU-GM	E11.5AGM	0ug/ml	-2.1950	0.1341	36	-16.37	<.0001	0.05	-2.4670	-1.9230
BFU-E	HE+PSCs	0ug/ml	CFU-GM	E11.5AGM	2ug/ml	-1.5175	0.1356	36	-11.19	<.0001	0.05	-1.7926	-1.2424
BFU-E	HE+PSCs	0ug/ml	CFU-GM	E11.5AGM	6ug/ml	-0.9709	0.1379	36	-7.04	<.0001	0.05	-1.2505	-0.6913
BFU-E	HE+PSCs	0ug/ml	CFU-GM	HE+PSCs	0ug/ml	-1.5827	0.06757	36	-23.42	<.0001	0.05	-1.7197	-1.4457
BFU-E	HE+PSCs	0ug/ml	CFU-GM	HE+PSCs	2ug/ml	-0.7234	0.1393	36	-5.19	<.0001	0.05	-1.0060	-0.4409
BFU-E	HE+PSCs	0ug/ml	CFU-GM	HE+PSCs	6ug/ml	0.2176	0.1493	36	1.46	0.1537	0.05	-0.08521	0.5204
BFU-E	HE+PSCs	2ug/ml	BFU-E	HE+PSCs	6ug/ml	0.5260	0.1835	36	2.87	0.0069	0.05	0.1539	0.8982
BFU-E	HE+PSCs	2ug/ml	CFU-GEMM	AoE+PSCs	0ug/ml	-0.7847	0.1569	36	-5.00	<.0001	0.05	-1.1028	-0.4666
BFU-E	HE+PSCs	2ug/ml	CFU-GEMM	AoE+PSCs	2ug/ml	-0.2090	0.1650	36	-1.27	0.2134	0.05	-0.5435	0.1256
BFU-E	HE+PSCs	2ug/ml	CFU-GEMM	AoE+PSCs	6ug/ml	0.6151	0.1869	36	3.29	0.0022	0.05	0.2361	0.9942
BFU-E	HE+PSCs	2ug/ml	CFU-GEMM	E11.5AGM	0ug/ml	-1.4320	0.1516	36	-9.44	<.0001	0.05	-1.7396	-1.1245
BFU-E	HE+PSCs	2ug/ml	CFU-GEMM	E11.5AGM	2ug/ml	-1.2262	0.1530	36	-8.02	<.0001	0.05	-1.5364	-0.9160
BFU-E	HE+PSCs	2ug/ml	CFU-GEMM	E11.5AGM	6ug/ml	0.000154	0.1692	36	0.00	0.9993	0.05	-0.3430	0.3433

The GLIMMIX Procedure

Differences of Colony*Condit*Treatm Least Squares Means													
Colony_Type	Condition	Treatment	_Colony_Type	_Condition	_Treatment	Estimate	Standard Error	DF	t Value	Pr > |t|	Alpha	Lower	Upper
BFU-E	HE+PSCs	2ug/ml	CFU-GEMM	HE+PSCs	0ug/ml	-0.7590	0.1572	36	-4.83	<.0001	0.05	-1.0777	-0.4402
BFU-E	HE+PSCs	2ug/ml	CFU-GEMM	HE+PSCs	2ug/ml	0.3728	0.1347	36	2.77	0.0089	0.05	0.09956	0.6461
BFU-E	HE+PSCs	2ug/ml	CFU-GEMM	HE+PSCs	6ug/ml	0.5511	0.1843	36	2.99	0.0050	0.05	0.1772	0.9250
BFU-E	HE+PSCs	2ug/ml	CFU-GM	AoE+PSCs	0ug/ml	-2.4561	0.1478	36	-16.62	<.0001	0.05	-2.7559	-2.1564
BFU-E	HE+PSCs	2ug/ml	CFU-GM	AoE+PSCs	2ug/ml	-1.7069	0.1502	36	-11.36	<.0001	0.05	-2.0115	-1.4022
BFU-E	HE+PSCs	2ug/ml	CFU-GM	AoE+PSCs	6ug/ml	-0.9461	0.1553	36	-6.09	<.0001	0.05	-1.2610	-0.6311
BFU-E	HE+PSCs	2ug/ml	CFU-GM	E11.5AGM	0ug/ml	-2.8739	0.1471	36	-19.54	<.0001	0.05	-3.1722	-2.5757
BFU-E	HE+PSCs	2ug/ml	CFU-GM	E11.5AGM	2ug/ml	-2.1964	0.1484	36	-14.80	<.0001	0.05	-2.4975	-1.8954
BFU-E	HE+PSCs	2ug/ml	CFU-GM	E11.5AGM	6ug/ml	-1.6498	0.1505	36	-10.96	<.0001	0.05	-1.9550	-1.3446
BFU-E	HE+PSCs	2ug/ml	CFU-GM	HE+PSCs	0ug/ml	-2.2616	0.1483	36	-15.25	<.0001	0.05	-2.5623	-1.9609
BFU-E	HE+PSCs	2ug/ml	CFU-GM	HE+PSCs	2ug/ml	-1.4024	0.09606	36	-14.60	<.0001	0.05	-1.5972	-1.2075
BFU-E	HE+PSCs	2ug/ml	CFU-GM	HE+PSCs	6ug/ml	-0.4613	0.1610	36	-2.87	0.0069	0.05	-0.7878	-0.1348
BFU-E	HE+PSCs	6ug/ml	CFU-GEMM	AoE+PSCs	0ug/ml	-1.3107	0.1722	36	-7.61	<.0001	0.05	-1.6600	-0.9615
BFU-E	HE+PSCs	6ug/ml	CFU-GEMM	AoE+PSCs	2ug/ml	-0.7350	0.1796	36	-4.09	0.0002	0.05	-1.0992	-0.3707
BFU-E	HE+PSCs	6ug/ml	CFU-GEMM	AoE+PSCs	6ug/ml	0.08911	0.1999	36	0.45	0.6585	0.05	-0.3163	0.4946
BFU-E	HE+PSCs	6ug/ml	CFU-GEMM	E11.5AGM	0ug/ml	-1.9581	0.1675	36	-11.69	<.0001	0.05	-2.2977	-1.6184
BFU-E	HE+PSCs	6ug/ml	CFU-GEMM	E11.5AGM	2ug/ml	-1.7522	0.1687	36	-10.39	<.0001	0.05	-2.0943	-1.4102
BFU-E	HE+PSCs	6ug/ml	CFU-GEMM	E11.5AGM	6ug/ml	-0.5259	0.1835	36	-2.87	0.0069	0.05	-0.8980	-0.1537
BFU-E	HE+PSCs	6ug/ml	CFU-GEMM	HE+PSCs	0ug/ml	-1.2850	0.1725	36	-7.45	<.0001	0.05	-1.6348	-0.9352
BFU-E	HE+PSCs	6ug/ml	CFU-GEMM	HE+PSCs	2ug/ml	-0.1532	0.1924	36	-0.80	0.4312	0.05	-0.5434	0.2370
BFU-E	HE+PSCs	6ug/ml	CFU-GEMM	HE+PSCs	6ug/ml	0.02506	0.1582	36	0.16	0.8750	0.05	-0.2958	0.3459
BFU-E	HE+PSCs	6ug/ml	CFU-GM	AoE+PSCs	0ug/ml	-2.9821	0.1640	36	-18.19	<.0001	0.05	-3.3147	-2.6496
BFU-E	HE+PSCs	6ug/ml	CFU-GM	AoE+PSCs	2ug/ml	-2.2329	0.1662	36	-13.44	<.0001	0.05	-2.5699	-1.8959
BFU-E	HE+PSCs	6ug/ml	CFU-GM	AoE+PSCs	6ug/ml	-1.4721	0.1707	36	-8.62	<.0001	0.05	-1.8183	-1.1258

The GLIMMIX Procedure

Differences of Colony*Condit*Treatm Least Squares Means													
Colony_Type	Condition	Treatment	_Colony_Type	_Condition	_Treatment	Estimate	Standard Error	DF	t Value	Pr > |t|	Alpha	Lower	Upper
BFU-E	HE+PSCs	6ug/ml	CFU-GM	E11.5AGM	0ug/ml	-3.4000	0.1633	36	-20.82	<.0001	0.05	-3.7312	-3.0687
BFU-E	HE+PSCs	6ug/ml	CFU-GM	E11.5AGM	2ug/ml	-2.7224	0.1646	36	-16.54	<.0001	0.05	-3.0562	-2.3887
BFU-E	HE+PSCs	6ug/ml	CFU-GM	E11.5AGM	6ug/ml	-2.1758	0.1664	36	-13.08	<.0001	0.05	-2.5133	-1.8384
BFU-E	HE+PSCs	6ug/ml	CFU-GM	HE+PSCs	0ug/ml	-2.7876	0.1644	36	-16.95	<.0001	0.05	-3.1211	-2.4542
BFU-E	HE+PSCs	6ug/ml	CFU-GM	HE+PSCs	2ug/ml	-1.9284	0.1676	36	-11.51	<.0001	0.05	-2.2683	-1.5885
BFU-E	HE+PSCs	6ug/ml	CFU-GM	HE+PSCs	6ug/ml	-0.9874	0.1302	36	-7.58	<.0001	0.05	-1.2515	-0.7232
CFU-GEMM	AoE+PSCs	0ug/ml	CFU-GEMM	AoE+PSCs	2ug/ml	0.5757	0.1523	36	3.78	0.0006	0.05	0.2669	0.8846
CFU-GEMM	AoE+PSCs	0ug/ml	CFU-GEMM	AoE+PSCs	6ug/ml	1.3998	0.1758	36	7.96	<.0001	0.05	1.0433	1.7564
CFU-GEMM	AoE+PSCs	0ug/ml	CFU-GEMM	E11.5AGM	0ug/ml	-0.6474	0.1377	36	-4.70	<.0001	0.05	-0.9267	-0.3680
CFU-GEMM	AoE+PSCs	0ug/ml	CFU-GEMM	E11.5AGM	2ug/ml	-0.4415	0.1392	36	-3.17	0.0031	0.05	-0.7238	-0.1593
CFU-GEMM	AoE+PSCs	0ug/ml	CFU-GEMM	E11.5AGM	6ug/ml	0.7848	0.1569	36	5.00	<.0001	0.05	0.4667	1.1030
CFU-GEMM	AoE+PSCs	0ug/ml	CFU-GEMM	HE+PSCs	0ug/ml	0.02569	0.1438	36	0.18	0.8592	0.05	-0.2659	0.3173
CFU-GEMM	AoE+PSCs	0ug/ml	CFU-GEMM	HE+PSCs	2ug/ml	1.1575	0.1672	36	6.92	<.0001	0.05	0.8184	1.4966
CFU-GEMM	AoE+PSCs	0ug/ml	CFU-GEMM	HE+PSCs	6ug/ml	1.3358	0.1731	36	7.72	<.0001	0.05	0.9847	1.6869
CFU-GEMM	AoE+PSCs	0ug/ml	CFU-GM	AoE+PSCs	0ug/ml	-1.6714	0.06357	36	-26.29	<.0001	0.05	-1.8004	-1.5425
CFU-GEMM	AoE+PSCs	0ug/ml	CFU-GM	AoE+PSCs	2ug/ml	-0.9222	0.1362	36	-6.77	<.0001	0.05	-1.1984	-0.6460
CFU-GEMM	AoE+PSCs	0ug/ml	CFU-GM	AoE+PSCs	6ug/ml	-0.1614	0.1417	36	-1.14	0.2624	0.05	-0.4488	0.1261
CFU-GEMM	AoE+PSCs	0ug/ml	CFU-GM	E11.5AGM	0ug/ml	-2.0893	0.1327	36	-15.75	<.0001	0.05	-2.3583	-1.8202
CFU-GEMM	AoE+PSCs	0ug/ml	CFU-GM	E11.5AGM	2ug/ml	-1.4117	0.1342	36	-10.52	<.0001	0.05	-1.6839	-1.1396
CFU-GEMM	AoE+PSCs	0ug/ml	CFU-GM	E11.5AGM	6ug/ml	-0.8651	0.1365	36	-6.34	<.0001	0.05	-1.1419	-0.5884
CFU-GEMM	AoE+PSCs	0ug/ml	CFU-GM	HE+PSCs	0ug/ml	-1.4769	0.1340	36	-11.02	<.0001	0.05	-1.7487	-1.2052
CFU-GEMM	AoE+PSCs	0ug/ml	CFU-GM	HE+PSCs	2ug/ml	-0.6177	0.1379	36	-4.48	<.0001	0.05	-0.8974	-0.3380
CFU-GEMM	AoE+PSCs	0ug/ml	CFU-GM	HE+PSCs	6ug/ml	0.3234	0.1480	36	2.18	0.0355	0.05	0.02321	0.6235
CFU-GEMM	AoE+PSCs	2ug/ml	CFU-GEMM	AoE+PSCs	6ug/ml	0.8241	0.1831	36	4.50	<.0001	0.05	0.4528	1.1954

The GLIMMIX Procedure

Differences of Colony*Condit*Treatm Least Squares Means													
Colony_Type	Condition	Treatment	_Colony_Type	_Condition	_Treatment	Estimate	Standard Error	DF	t Value	Pr > |t|	Alpha	Lower	Upper
CFU-GEMM	AoE+PSCs	2ug/ml	CFU-GEMM	E11.5AGM	0ug/ml	-1.2231	0.1469	36	-8.33	<.0001	0.05	-1.5210	-0.9252
CFU-GEMM	AoE+PSCs	2ug/ml	CFU-GEMM	E11.5AGM	2ug/ml	-1.0173	0.1483	36	-6.86	<.0001	0.05	-1.3180	-0.7166
CFU-GEMM	AoE+PSCs	2ug/ml	CFU-GEMM	E11.5AGM	6ug/ml	0.2091	0.1650	36	1.27	0.2131	0.05	-0.1254	0.5437
CFU-GEMM	AoE+PSCs	2ug/ml	CFU-GEMM	HE+PSCs	0ug/ml	-0.5500	0.1526	36	-3.60	0.0009	0.05	-0.8595	-0.2405
CFU-GEMM	AoE+PSCs	2ug/ml	CFU-GEMM	HE+PSCs	2ug/ml	0.5818	0.1748	36	3.33	0.0020	0.05	0.2273	0.9363
CFU-GEMM	AoE+PSCs	2ug/ml	CFU-GEMM	HE+PSCs	6ug/ml	0.7600	0.1805	36	4.21	0.0002	0.05	0.3940	1.1260
CFU-GEMM	AoE+PSCs	2ug/ml	CFU-GM	AoE+PSCs	0ug/ml	-2.2472	0.1429	36	-15.72	<.0001	0.05	-2.5370	-1.9573
CFU-GEMM	AoE+PSCs	2ug/ml	CFU-GM	AoE+PSCs	2ug/ml	-1.4979	0.08559	36	-17.50	<.0001	0.05	-1.6715	-1.3243
CFU-GEMM	AoE+PSCs	2ug/ml	CFU-GM	AoE+PSCs	6ug/ml	-0.7371	0.1507	36	-4.89	<.0001	0.05	-1.0426	-0.4316
CFU-GEMM	AoE+PSCs	2ug/ml	CFU-GM	E11.5AGM	0ug/ml	-2.6650	0.1422	36	-18.75	<.0001	0.05	-2.9533	-2.3767
CFU-GEMM	AoE+PSCs	2ug/ml	CFU-GM	E11.5AGM	2ug/ml	-1.9875	0.1436	36	-13.84	<.0001	0.05	-2.2787	-1.6962
CFU-GEMM	AoE+PSCs	2ug/ml	CFU-GM	E11.5AGM	6ug/ml	-1.4408	0.1457	36	-9.89	<.0001	0.05	-1.7363	-1.1454
CFU-GEMM	AoE+PSCs	2ug/ml	CFU-GM	HE+PSCs	0ug/ml	-2.0527	0.1434	36	-14.31	<.0001	0.05	-2.3435	-1.7618
CFU-GEMM	AoE+PSCs	2ug/ml	CFU-GM	HE+PSCs	2ug/ml	-1.1934	0.1471	36	-8.11	<.0001	0.05	-1.4917	-0.8951
CFU-GEMM	AoE+PSCs	2ug/ml	CFU-GM	HE+PSCs	6ug/ml	-0.2524	0.1565	36	-1.61	0.1156	0.05	-0.5698	0.06508
CFU-GEMM	AoE+PSCs	6ug/ml	CFU-GEMM	E11.5AGM	0ug/ml	-2.0472	0.1712	36	-11.96	<.0001	0.05	-2.3943	-1.7000
CFU-GEMM	AoE+PSCs	6ug/ml	CFU-GEMM	E11.5AGM	2ug/ml	-1.8413	0.1723	36	-10.68	<.0001	0.05	-2.1909	-1.4918
CFU-GEMM	AoE+PSCs	6ug/ml	CFU-GEMM	E11.5AGM	6ug/ml	-0.6150	0.1869	36	-3.29	0.0022	0.05	-0.9940	-0.2359
CFU-GEMM	AoE+PSCs	6ug/ml	CFU-GEMM	HE+PSCs	0ug/ml	-1.3741	0.1761	36	-7.80	<.0001	0.05	-1.7312	-1.0170
CFU-GEMM	AoE+PSCs	6ug/ml	CFU-GEMM	HE+PSCs	2ug/ml	-0.2423	0.1956	36	-1.24	0.2236	0.05	-0.6391	0.1545
CFU-GEMM	AoE+PSCs	6ug/ml	CFU-GEMM	HE+PSCs	6ug/ml	-0.06404	0.2007	36	-0.32	0.7515	0.05	-0.4711	0.3430
CFU-GEMM	AoE+PSCs	6ug/ml	CFU-GM	AoE+PSCs	0ug/ml	-3.0712	0.1678	36	-18.31	<.0001	0.05	-3.4115	-2.7310
CFU-GEMM	AoE+PSCs	6ug/ml	CFU-GM	AoE+PSCs	2ug/ml	-2.3220	0.1699	36	-13.67	<.0001	0.05	-2.6666	-1.9774
CFU-GEMM	AoE+PSCs	6ug/ml	CFU-GM	AoE+PSCs	6ug/ml	-1.5612	0.1287	36	-12.13	<.0001	0.05	-1.8222	-1.3001

The GLIMMIX Procedure

Differences of Colony*Condit*Treatm Least Squares Means

Colony_Type	Condition	Treatment	_Colony_Type	_Condition	_Treatment	Estimate	Standard Error	DF	t Value	Pr > |t|	Alpha	Lower	Upper
CFU-GEMM	AoE+PSCs	6ug/ml	CFU-GM	E11.5AGM	0ug/ml	-3.4891	0.1671	36	-20.88	<.0001	0.05	-3.8280	-3.1501
CFU-GEMM	AoE+PSCs	6ug/ml	CFU-GM	E11.5AGM	2ug/ml	-2.8115	0.1683	36	-16.70	<.0001	0.05	-3.1530	-2.4701
CFU-GEMM	AoE+PSCs	6ug/ml	CFU-GM	E11.5AGM	6ug/ml	-2.2649	0.1701	36	-13.31	<.0001	0.05	-2.6100	-1.9199
CFU-GEMM	AoE+PSCs	6ug/ml	CFU-GM	HE+PSCs	0ug/ml	-2.8768	0.1682	36	-17.10	<.0001	0.05	-3.2178	-2.5357
CFU-GEMM	AoE+PSCs	6ug/ml	CFU-GM	HE+PSCs	2ug/ml	-2.0175	0.1713	36	-11.78	<.0001	0.05	-2.3649	-1.6701
CFU-GEMM	AoE+PSCs	6ug/ml	CFU-GM	HE+PSCs	6ug/ml	-1.0765	0.1795	36	-6.00	<.0001	0.05	-1.4405	-0.7125
CFU-GEMM	E11.5AGM	0ug/ml	CFU-GEMM	E11.5AGM	2ug/ml	0.2058	0.1333	36	1.54	0.1312	0.05	-0.06444	0.4761
CFU-GEMM	E11.5AGM	0ug/ml	CFU-GEMM	E11.5AGM	6ug/ml	1.4322	0.1516	36	9.45	<.0001	0.05	1.1247	1.7397
CFU-GEMM	E11.5AGM	0ug/ml	CFU-GEMM	HE+PSCs	0ug/ml	0.6730	0.1381	36	4.87	<.0001	0.05	0.3930	0.9530
CFU-GEMM	E11.5AGM	0ug/ml	CFU-GEMM	HE+PSCs	2ug/ml	1.8049	0.1623	36	11.12	<.0001	0.05	1.4757	2.1340
CFU-GEMM	E11.5AGM	0ug/ml	CFU-GEMM	HE+PSCs	6ug/ml	1.9831	0.1684	36	11.78	<.0001	0.05	1.6416	2.3246
CFU-GEMM	E11.5AGM	0ug/ml	CFU-GM	AoE+PSCs	0ug/ml	-1.0241	0.1273	36	-8.04	<.0001	0.05	-1.2823	-0.7659
CFU-GEMM	E11.5AGM	0ug/ml	CFU-GM	AoE+PSCs	2ug/ml	-0.2748	0.1301	36	-2.11	0.0417	0.05	-0.5387	-0.01097
CFU-GEMM	E11.5AGM	0ug/ml	CFU-GM	AoE+PSCs	6ug/ml	0.4860	0.1359	36	3.58	0.0010	0.05	0.2103	0.7616
CFU-GEMM	E11.5AGM	0ug/ml	CFU-GM	E11.5AGM	0ug/ml	-1.4419	0.04703	36	-30.66	<.0001	0.05	-1.5373	-1.3465
CFU-GEMM	E11.5AGM	0ug/ml	CFU-GM	E11.5AGM	2ug/ml	-0.7644	0.1280	36	-5.97	<.0001	0.05	-1.0241	-0.5047
CFU-GEMM	E11.5AGM	0ug/ml	CFU-GM	E11.5AGM	6ug/ml	-0.2178	0.1304	36	-1.67	0.1036	0.05	-0.4822	0.04671
CFU-GEMM	E11.5AGM	0ug/ml	CFU-GM	HE+PSCs	0ug/ml	-0.8296	0.1278	36	-6.49	<.0001	0.05	-1.0889	-0.5703
CFU-GEMM	E11.5AGM	0ug/ml	CFU-GM	HE+PSCs	2ug/ml	0.02968	0.1319	36	0.22	0.8233	0.05	-0.2379	0.2973
CFU-GEMM	E11.5AGM	0ug/ml	CFU-GM	HE+PSCs	6ug/ml	0.9707	0.1424	36	6.81	<.0001	0.05	0.6818	1.2596
CFU-GEMM	E11.5AGM	2ug/ml	CFU-GEMM	E11.5AGM	6ug/ml	1.2264	0.1530	36	8.02	<.0001	0.05	0.9162	1.5366
CFU-GEMM	E11.5AGM	2ug/ml	CFU-GEMM	HE+PSCs	0ug/ml	0.4672	0.1395	36	3.35	0.0019	0.05	0.1843	0.7502
CFU-GEMM	E11.5AGM	2ug/ml	CFU-GEMM	HE+PSCs	2ug/ml	1.5991	0.1635	36	9.78	<.0001	0.05	1.2674	1.9307
CFU-GEMM	E11.5AGM	2ug/ml	CFU-GEMM	HE+PSCs	6ug/ml	1.7773	0.1696	36	10.48	<.0001	0.05	1.4334	2.1213

The GLIMMIX Procedure

Differences of Colony*Condit*Treatm Least Squares Means													
Colony_Type	Condition	Treatment	_Colony_Type	_Condition	_Treatment	Estimate	Standard Error	DF	t Value	Pr > |t|	Alpha	Lower	Upper
CFU-GEMM	E11.5AGM	2ug/ml	CFU-GM	AoE+PSCs	0ug/ml	-1.2299	0.1289	36	-9.54	<.0001	0.05	-1.4913	-0.9685
CFU-GEMM	E11.5AGM	2ug/ml	CFU-GM	AoE+PSCs	2ug/ml	-0.4807	0.1317	36	-3.65	0.0008	0.05	-0.7477	-0.2136
CFU-GEMM	E11.5AGM	2ug/ml	CFU-GM	AoE+PSCs	6ug/ml	0.2802	0.1374	36	2.04	0.0489	0.05	0.001486	0.5588
CFU-GEMM	E11.5AGM	2ug/ml	CFU-GM	E11.5AGM	0ug/ml	-1.6477	0.1280	36	-12.87	<.0001	0.05	-1.9074	-1.3881
CFU-GEMM	E11.5AGM	2ug/ml	CFU-GM	E11.5AGM	2ug/ml	-0.9702	0.05499	36	-17.64	<.0001	0.05	-1.0817	-0.8587
CFU-GEMM	E11.5AGM	2ug/ml	CFU-GM	E11.5AGM	6ug/ml	-0.4236	0.1320	36	-3.21	0.0028	0.05	-0.6912	-0.1560
CFU-GEMM	E11.5AGM	2ug/ml	CFU-GM	HE+PSCs	0ug/ml	-1.0354	0.1294	36	-8.00	<.0001	0.05	-1.2979	-0.7729
CFU-GEMM	E11.5AGM	2ug/ml	CFU-GM	HE+PSCs	2ug/ml	-0.1761	0.1335	36	-1.32	0.1953	0.05	-0.4468	0.09455
CFU-GEMM	E11.5AGM	2ug/ml	CFU-GM	HE+PSCs	6ug/ml	0.7649	0.1439	36	5.32	<.0001	0.05	0.4731	1.0567
CFU-GEMM	E11.5AGM	6ug/ml	CFU-GEMM	HE+PSCs	0ug/ml	-0.7591	0.1572	36	-4.83	<.0001	0.05	-1.0779	-0.4404
CFU-GEMM	E11.5AGM	6ug/ml	CFU-GEMM	HE+PSCs	2ug/ml	0.3727	0.1788	36	2.08	0.0443	0.05	0.01004	0.7353
CFU-GEMM	E11.5AGM	6ug/ml	CFU-GEMM	HE+PSCs	6ug/ml	0.5509	0.1843	36	2.99	0.0050	0.05	0.1771	0.9248
CFU-GEMM	E11.5AGM	6ug/ml	CFU-GM	AoE+PSCs	0ug/ml	-2.4563	0.1478	36	-16.62	<.0001	0.05	-2.7560	-2.1565
CFU-GEMM	E11.5AGM	6ug/ml	CFU-GM	AoE+PSCs	2ug/ml	-1.7070	0.1502	36	-11.36	<.0001	0.05	-2.0117	-1.4024
CFU-GEMM	E11.5AGM	6ug/ml	CFU-GM	AoE+PSCs	6ug/ml	-0.9462	0.1553	36	-6.09	<.0001	0.05	-1.2611	-0.6313
CFU-GEMM	E11.5AGM	6ug/ml	CFU-GM	E11.5AGM	0ug/ml	-2.8741	0.1471	36	-19.55	<.0001	0.05	-3.1723	-2.5759
CFU-GEMM	E11.5AGM	6ug/ml	CFU-GM	E11.5AGM	2ug/ml	-2.1966	0.1484	36	-14.80	<.0001	0.05	-2.4976	-1.8955
CFU-GEMM	E11.5AGM	6ug/ml	CFU-GM	E11.5AGM	6ug/ml	-1.6500	0.09397	36	-17.56	<.0001	0.05	-1.8405	-1.4594
CFU-GEMM	E11.5AGM	6ug/ml	CFU-GM	HE+PSCs	0ug/ml	-2.2618	0.1483	36	-15.26	<.0001	0.05	-2.5625	-1.9611
CFU-GEMM	E11.5AGM	6ug/ml	CFU-GM	HE+PSCs	2ug/ml	-1.4025	0.1518	36	-9.24	<.0001	0.05	-1.7104	-1.0946
CFU-GEMM	E11.5AGM	6ug/ml	CFU-GM	HE+PSCs	6ug/ml	-0.4615	0.1610	36	-2.87	0.0069	0.05	-0.7880	-0.1350
CFU-GEMM	HE+PSCs	0ug/ml	CFU-GEMM	HE+PSCs	2ug/ml	1.1318	0.1675	36	6.76	<.0001	0.05	0.7922	1.4715
CFU-GEMM	HE+PSCs	0ug/ml	CFU-GEMM	HE+PSCs	6ug/ml	1.3101	0.1734	36	7.56	<.0001	0.05	0.9584	1.6617
CFU-GEMM	HE+PSCs	0ug/ml	CFU-GM	AoE+PSCs	0ug/ml	-1.6971	0.1338	36	-12.68	<.0001	0.05	-1.9686	-1.4257

The GLIMMIX Procedure

Differences of Colony*Condit*Treatm Least Squares Means													
Colony_Type	Condition	Treatment	_Colony_Type	_Condition	_Treatment	Estimate	Standard Error	DF	t Value	Pr > |t|	Alpha	Lower	Upper
CFU-GEMM	HE+PSCs	0ug/ml	CFU-GM	AoE+PSCs	2ug/ml	-0.9479	0.1365	36	-6.94	<.0001	0.05	-1.2248	-0.6710
CFU-GEMM	HE+PSCs	0ug/ml	CFU-GM	AoE+PSCs	6ug/ml	-0.1871	0.1421	36	-1.32	0.1963	0.05	-0.4752	0.1011
CFU-GEMM	HE+PSCs	0ug/ml	CFU-GM	E11.5AGM	0ug/ml	-2.1150	0.1330	36	-15.90	<.0001	0.05	-2.3847	-1.8452
CFU-GEMM	HE+PSCs	0ug/ml	CFU-GM	E11.5AGM	2ug/ml	-1.4374	0.1346	36	-10.68	<.0001	0.05	-1.7103	-1.1645
CFU-GEMM	HE+PSCs	0ug/ml	CFU-GM	E11.5AGM	6ug/ml	-0.8908	0.1368	36	-6.51	<.0001	0.05	-1.1683	-0.6134
CFU-GEMM	HE+PSCs	0ug/ml	CFU-GM	HE+PSCs	0ug/ml	-1.5026	0.06538	36	-22.98	<.0001	0.05	-1.6352	-1.3700
CFU-GEMM	HE+PSCs	0ug/ml	CFU-GM	HE+PSCs	2ug/ml	-0.6434	0.1383	36	-4.65	<.0001	0.05	-0.9238	-0.3629
CFU-GEMM	HE+PSCs	0ug/ml	CFU-GM	HE+PSCs	6ug/ml	0.2977	0.1483	36	2.01	0.0523	0.05	-0.00316	0.5985
CFU-GEMM	HE+PSCs	2ug/ml	CFU-GEMM	HE+PSCs	6ug/ml	0.1782	0.1932	36	0.92	0.3624	0.05	-0.2136	0.5701
CFU-GEMM	HE+PSCs	2ug/ml	CFU-GM	AoE+PSCs	0ug/ml	-2.8290	0.1587	36	-17.82	<.0001	0.05	-3.1508	-2.5071
CFU-GEMM	HE+PSCs	2ug/ml	CFU-GM	AoE+PSCs	2ug/ml	-2.0797	0.1610	36	-12.92	<.0001	0.05	-2.4062	-1.7533
CFU-GEMM	HE+PSCs	2ug/ml	CFU-GM	AoE+PSCs	6ug/ml	-1.3189	0.1657	36	-7.96	<.0001	0.05	-1.6550	-0.9828
CFU-GEMM	HE+PSCs	2ug/ml	CFU-GM	E11.5AGM	0ug/ml	-3.2468	0.1580	36	-20.55	<.0001	0.05	-3.5673	-2.9263
CFU-GEMM	HE+PSCs	2ug/ml	CFU-GM	E11.5AGM	2ug/ml	-2.5693	0.1593	36	-16.13	<.0001	0.05	-2.8924	-2.2462
CFU-GEMM	HE+PSCs	2ug/ml	CFU-GM	E11.5AGM	6ug/ml	-2.0226	0.1612	36	-12.55	<.0001	0.05	-2.3496	-1.6957
CFU-GEMM	HE+PSCs	2ug/ml	CFU-GM	HE+PSCs	0ug/ml	-2.6345	0.1592	36	-16.55	<.0001	0.05	-2.9572	-2.3117
CFU-GEMM	HE+PSCs	2ug/ml	CFU-GM	HE+PSCs	2ug/ml	-1.7752	0.1121	36	-15.83	<.0001	0.05	-2.0026	-1.5478
CFU-GEMM	HE+PSCs	2ug/ml	CFU-GM	HE+PSCs	6ug/ml	-0.8342	0.1711	36	-4.88	<.0001	0.05	-1.1811	-0.4872
CFU-GEMM	HE+PSCs	6ug/ml	CFU-GM	AoE+PSCs	0ug/ml	-3.0072	0.1649	36	-18.23	<.0001	0.05	-3.3417	-2.6727
CFU-GEMM	HE+PSCs	6ug/ml	CFU-GM	AoE+PSCs	2ug/ml	-2.2580	0.1671	36	-13.51	<.0001	0.05	-2.5968	-1.9191
CFU-GEMM	HE+PSCs	6ug/ml	CFU-GM	AoE+PSCs	6ug/ml	-1.4971	0.1716	36	-8.72	<.0001	0.05	-1.8452	-1.1490
CFU-GEMM	HE+PSCs	6ug/ml	CFU-GM	E11.5AGM	0ug/ml	-3.4250	0.1643	36	-20.85	<.0001	0.05	-3.7582	-3.0918
CFU-GEMM	HE+PSCs	6ug/ml	CFU-GM	E11.5AGM	2ug/ml	-2.7475	0.1655	36	-16.60	<.0001	0.05	-3.0832	-2.4118
CFU-GEMM	HE+PSCs	6ug/ml	CFU-GM	E11.5AGM	6ug/ml	-2.2009	0.1673	36	-13.15	<.0001	0.05	-2.5402	-1.8615

The GLIMMIX Procedure

Differences of Colony*Condit*Treatm Least Squares Means													
Colony_Type	Condition	Treatment	_Colony_Type	_Condition	_Treatment	Estimate	Standard Error	DF	t Value	Pr > |t|	Alpha	Lower	Upper
CFU-GEMM	HE+PSCs	6ug/ml	CFU-GM	HE+PSCs	0ug/ml	-2.8127	0.1654	36	-17.01	<.0001	0.05	-3.1481	-2.4773
CFU-GEMM	HE+PSCs	6ug/ml	CFU-GM	HE+PSCs	2ug/ml	-1.9534	0.1685	36	-11.59	<.0001	0.05	-2.2952	-1.6117
CFU-GEMM	HE+PSCs	6ug/ml	CFU-GM	HE+PSCs	6ug/ml	-1.0124	0.1314	36	-7.70	<.0001	0.05	-1.2790	-0.7459
CFU-GM	AoE+PSCs	0ug/ml	CFU-GM	AoE+PSCs	2ug/ml	0.7492	0.1256	36	5.96	<.0001	0.05	0.4945	1.0040
CFU-GM	AoE+PSCs	0ug/ml	CFU-GM	AoE+PSCs	6ug/ml	1.5101	0.1316	36	11.47	<.0001	0.05	1.2431	1.7770
CFU-GM	AoE+PSCs	0ug/ml	CFU-GM	E11.5AGM	0ug/ml	-0.4178	0.1218	36	-3.43	0.0015	0.05	-0.6649	-0.1708
CFU-GM	AoE+PSCs	0ug/ml	CFU-GM	E11.5AGM	2ug/ml	0.2597	0.1235	36	2.10	0.0425	0.05	0.009252	0.5101
CFU-GM	AoE+PSCs	0ug/ml	CFU-GM	E11.5AGM	6ug/ml	0.8063	0.1259	36	6.40	<.0001	0.05	0.5509	1.0617
CFU-GM	AoE+PSCs	0ug/ml	CFU-GM	HE+PSCs	0ug/ml	0.1945	0.1233	36	1.58	0.1234	0.05	-0.05552	0.4445
CFU-GM	AoE+PSCs	0ug/ml	CFU-GM	HE+PSCs	2ug/ml	1.0538	0.1275	36	8.26	<.0001	0.05	0.7951	1.3124
CFU-GM	AoE+PSCs	0ug/ml	CFU-GM	HE+PSCs	6ug/ml	1.9948	0.1383	36	14.42	<.0001	0.05	1.7142	2.2754
CFU-GM	AoE+PSCs	2ug/ml	CFU-GM	AoE+PSCs	6ug/ml	0.7608	0.1343	36	5.66	<.0001	0.05	0.4884	1.0333
CFU-GM	AoE+PSCs	2ug/ml	CFU-GM	E11.5AGM	0ug/ml	-1.1671	0.1247	36	-9.36	<.0001	0.05	-1.4201	-0.9141
CFU-GM	AoE+PSCs	2ug/ml	CFU-GM	E11.5AGM	2ug/ml	-0.4895	0.1264	36	-3.87	0.0004	0.05	-0.7459	-0.2332
CFU-GM	AoE+PSCs	2ug/ml	CFU-GM	E11.5AGM	6ug/ml	0.05708	0.1288	36	0.44	0.6602	0.05	-0.2041	0.3182
CFU-GM	AoE+PSCs	2ug/ml	CFU-GM	HE+PSCs	0ug/ml	-0.5548	0.1262	36	-4.40	<.0001	0.05	-0.8106	-0.2989
CFU-GM	AoE+PSCs	2ug/ml	CFU-GM	HE+PSCs	2ug/ml	0.3045	0.1303	36	2.34	0.0251	0.05	0.04023	0.5688
CFU-GM	AoE+PSCs	2ug/ml	CFU-GM	HE+PSCs	6ug/ml	1.2455	0.1409	36	8.84	<.0001	0.05	0.9598	1.5313
CFU-GM	AoE+PSCs	6ug/ml	CFU-GM	E11.5AGM	0ug/ml	-1.9279	0.1308	36	-14.74	<.0001	0.05	-2.1932	-1.6626
CFU-GM	AoE+PSCs	6ug/ml	CFU-GM	E11.5AGM	2ug/ml	-1.2504	0.1324	36	-9.45	<.0001	0.05	-1.5188	-0.9819
CFU-GM	AoE+PSCs	6ug/ml	CFU-GM	E11.5AGM	6ug/ml	-0.7037	0.1346	36	-5.23	<.0001	0.05	-0.9768	-0.4307
CFU-GM	AoE+PSCs	6ug/ml	CFU-GM	HE+PSCs	0ug/ml	-1.3156	0.1322	36	-9.95	<.0001	0.05	-1.5836	-1.0475
CFU-GM	AoE+PSCs	6ug/ml	CFU-GM	HE+PSCs	2ug/ml	-0.4563	0.1361	36	-3.35	0.0019	0.05	-0.7324	-0.1802
CFU-GM	AoE+PSCs	6ug/ml	CFU-GM	HE+PSCs	6ug/ml	0.4847	0.1463	36	3.31	0.0021	0.05	0.1881	0.7814

The GLIMMIX Procedure

Differences of Colony*Condit*Treatm Least Squares Means													
Colony_Type	Condition	Treatment	_Colony_Type	_Condition	_Treatment	Estimate	Standard Error	DF	t Value	Pr > |t|	Alpha	Lower	Upper
CFU-GM	E11.5AGM	0ug/ml	CFU-GM	E11.5AGM	2ug/ml	0.6775	0.1226	36	5.53	<.0001	0.05	0.4289	0.9262
CFU-GM	E11.5AGM	0ug/ml	CFU-GM	E11.5AGM	6ug/ml	1.2241	0.1251	36	9.79	<.0001	0.05	0.9705	1.4778
CFU-GM	E11.5AGM	0ug/ml	CFU-GM	HE+PSCs	0ug/ml	0.6123	0.1224	36	5.00	<.0001	0.05	0.3641	0.8605
CFU-GM	E11.5AGM	0ug/ml	CFU-GM	HE+PSCs	2ug/ml	1.4716	0.1267	36	11.62	<.0001	0.05	1.2147	1.7285
CFU-GM	E11.5AGM	0ug/ml	CFU-GM	HE+PSCs	6ug/ml	2.4126	0.1376	36	17.54	<.0001	0.05	2.1336	2.6916
CFU-GM	E11.5AGM	2ug/ml	CFU-GM	E11.5AGM	6ug/ml	0.5466	0.1267	36	4.31	0.0001	0.05	0.2897	0.8036
CFU-GM	E11.5AGM	2ug/ml	CFU-GM	HE+PSCs	0ug/ml	-0.06520	0.1240	36	-0.53	0.6024	0.05	-0.3168	0.1864
CFU-GM	E11.5AGM	2ug/ml	CFU-GM	HE+PSCs	2ug/ml	0.7941	0.1283	36	6.19	<.0001	0.05	0.5339	1.0542
CFU-GM	E11.5AGM	2ug/ml	CFU-GM	HE+PSCs	6ug/ml	1.7351	0.1390	36	12.48	<.0001	0.05	1.4531	2.0171
CFU-GM	E11.5AGM	6ug/ml	CFU-GM	HE+PSCs	0ug/ml	-0.6118	0.1265	36	-4.84	<.0001	0.05	-0.8683	-0.3553
CFU-GM	E11.5AGM	6ug/ml	CFU-GM	HE+PSCs	2ug/ml	0.2474	0.1306	36	1.89	0.0662	0.05	-0.01745	0.5123
CFU-GM	E11.5AGM	6ug/ml	CFU-GM	HE+PSCs	6ug/ml	1.1885	0.1412	36	8.42	<.0001	0.05	0.9021	1.4748
CFU-GM	HE+PSCs	0ug/ml	CFU-GM	HE+PSCs	2ug/ml	0.8593	0.1281	36	6.71	<.0001	0.05	0.5995	1.1190
CFU-GM	HE+PSCs	0ug/ml	CFU-GM	HE+PSCs	6ug/ml	1.8003	0.1389	36	12.96	<.0001	0.05	1.5187	2.0819
CFU-GM	HE+PSCs	2ug/ml	CFU-GM	HE+PSCs	6ug/ml	0.9410	0.1426	36	6.60	<.0001	0.05	0.6518	1.2302

The GENMOD Procedure

Model Information	
Data Set	WORK.EXT_FIG10BI_E11DRAXIN_TRANS
Distribution	Normal
Link Function	Identity
Dependent Variable	Percentage

Number of Observations Read	45
Number of Observations Used	45

Class Level Information		
Class	Levels	Values
Condition	3	AoE+PSCs E11.5AGM HE+PSCs
Treatment	3	0ug/ml 2ug/ml 6ug/ml

Parameter Information			
Parameter	Effect	Condition	Treatment
Prm1	Intercept		
Prm2	Condition	AoE+PSCs	
Prm3	Condition	E11.5AGM	
Prm4	Condition	HE+PSCs	
Prm5	Treatment		0ug/ml
Prm6	Treatment		2ug/ml
Prm7	Treatment		6ug/ml
Prm8	Condition*Treatment	AoE+PSCs	0ug/ml
Prm9	Condition*Treatment	AoE+PSCs	2ug/ml
Prm10	Condition*Treatment	AoE+PSCs	6ug/ml
Prm11	Condition*Treatment	E11.5AGM	0ug/ml

The GENMOD Procedure

Parameter Information			
**Parameter**	**Effect**	**Condition**	**Treatment**
**Prm12**	Condition*Treatment	E11.5AGM	2ug/ml
**Prm13**	Condition*Treatment	E11.5AGM	6ug/ml
**Prm14**	Condition*Treatment	HE+PSCs	0ug/ml
**Prm15**	Condition*Treatment	HE+PSCs	2ug/ml
**Prm16**	Condition*Treatment	HE+PSCs	6ug/ml

Criteria For Assessing Goodness Of Fit			
**Criterion**	**DF**	**Value**	**Value/DF**
**Deviance**	36	652.4000	18.1222
**Scaled Deviance**	36	45.0000	1.2500
**Pearson Chi-Square**	36	652.4000	18.1222
**Scaled Pearson X2**	36	45.0000	1.2500
**Log Likelihood**		-124.0171	
**Full Log Likelihood**		-124.0171	
**AIC (smaller is better)**		268.0343	
**AICC (smaller is better)**		274.5048	
**BIC (smaller is better)**		286.1009	

Algorithm converged.

The GENMOD Procedure

Analysis Of Maximum Likelihood Parameter Estimates

Parameter			DF	Estimate	Standard Error	Wald 95% Confidence Limits		Wald Chi-Square	Pr > ChiSq
**Intercept**			1	3.0000	1.7028	-0.3374	6.3374	3.10	0.0781
**Condition**	AoE+PSCs		1	4.2000	2.4081	-0.5199	8.9199	3.04	0.0811
**Condition**	E11.5AGM		1	15.8000	2.4081	11.0801	20.5199	43.05	<.0001
**Condition**	HE+PSCs		0	0.0000	0.0000	0.0000	0.0000	.	.
**Treatment**	0ug/ml		1	17.4000	2.4081	12.6801	22.1199	52.21	<.0001
**Treatment**	2ug/ml		1	2.0000	2.4081	-2.7199	6.7199	0.69	0.4062
**Treatment**	6ug/ml		0	0.0000	0.0000	0.0000	0.0000	.	.
**Condition*Treatment**	AoE+PSCs	0ug/ml	1	4.4000	3.4056	-2.2749	11.0749	1.67	0.1964
**Condition*Treatment**	AoE+PSCs	2ug/ml	1	6.4000	3.4056	-0.2749	13.0749	3.53	0.0602
**Condition*Treatment**	AoE+PSCs	6ug/ml	0	0.0000	0.0000	0.0000	0.0000	.	.
**Condition*Treatment**	E11.5AGM	0ug/ml	1	22.4000	3.4056	15.7251	29.0749	43.26	<.0001
**Condition*Treatment**	E11.5AGM	2ug/ml	1	18.8000	3.4056	12.1251	25.4749	30.47	<.0001
**Condition*Treatment**	E11.5AGM	6ug/ml	0	0.0000	0.0000	0.0000	0.0000	.	.
**Condition*Treatment**	HE+PSCs	0ug/ml	0	0.0000	0.0000	0.0000	0.0000	.	.
**Condition*Treatment**	HE+PSCs	2ug/ml	0	0.0000	0.0000	0.0000	0.0000	.	.
**Condition*Treatment**	HE+PSCs	6ug/ml	0	0.0000	0.0000	0.0000	0.0000	.	.
**Scale**			1	3.8076	0.4014	3.0969	4.6814		

Note: The scale parameter was estimated by maximum likelihood.

The GENMOD Procedure

Condition*Treatment Least Squares Means								
Condition	Treatment	Estimate	Standard Error	z Value	Pr > |z|	Alpha	Lower	Upper
AoE+PSCs	0ug/ml	29.0000	1.7028	17.03	<.0001	0.05	25.6626	32.3374
AoE+PSCs	2ug/ml	15.6000	1.7028	9.16	<.0001	0.05	12.2626	18.9374
AoE+PSCs	6ug/ml	7.2000	1.7028	4.23	<.0001	0.05	3.8626	10.5374
E11.5AGM	0ug/ml	58.6000	1.7028	34.41	<.0001	0.05	55.2626	61.9374
E11.5AGM	2ug/ml	39.6000	1.7028	23.26	<.0001	0.05	36.2626	42.9374
E11.5AGM	6ug/ml	18.8000	1.7028	11.04	<.0001	0.05	15.4626	22.1374
HE+PSCs	0ug/ml	20.4000	1.7028	11.98	<.0001	0.05	17.0626	23.7374
HE+PSCs	2ug/ml	5.0000	1.7028	2.94	0.0033	0.05	1.6626	8.3374
HE+PSCs	6ug/ml	3.0000	1.7028	1.76	0.0781	0.05	-0.3374	6.3374

Differences of Condition*Treatment Least Squares Means										
Condition	Treatment	_Condition	_Treatment	Estimate	Standard Error	z Value	Pr > |z|	Alpha	Lower	Upper
AoE+PSCs	0ug/ml	AoE+PSCs	2ug/ml	13.4000	2.4081	5.56	<.0001	0.05	8.6801	18.1199
AoE+PSCs	0ug/ml	AoE+PSCs	6ug/ml	21.8000	2.4081	9.05	<.0001	0.05	17.0801	26.5199
AoE+PSCs	0ug/ml	E11.5AGM	0ug/ml	-29.6000	2.4081	-12.29	<.0001	0.05	-34.3199	-24.8801
AoE+PSCs	0ug/ml	E11.5AGM	2ug/ml	-10.6000	2.4081	-4.40	<.0001	0.05	-15.3199	-5.8801
AoE+PSCs	0ug/ml	E11.5AGM	6ug/ml	10.2000	2.4081	4.24	<.0001	0.05	5.4801	14.9199
AoE+PSCs	0ug/ml	HE+PSCs	0ug/ml	8.6000	2.4081	3.57	0.0004	0.05	3.8801	13.3199
AoE+PSCs	0ug/ml	HE+PSCs	2ug/ml	24.0000	2.4081	9.97	<.0001	0.05	19.2801	28.7199
AoE+PSCs	0ug/ml	HE+PSCs	6ug/ml	26.0000	2.4081	10.80	<.0001	0.05	21.2801	30.7199
AoE+PSCs	2ug/ml	AoE+PSCs	6ug/ml	8.4000	2.4081	3.49	0.0005	0.05	3.6801	13.1199
AoE+PSCs	2ug/ml	E11.5AGM	0ug/ml	-43.0000	2.4081	-17.86	<.0001	0.05	-47.7199	-38.2801
AoE+PSCs	2ug/ml	E11.5AGM	2ug/ml	-24.0000	2.4081	-9.97	<.0001	0.05	-28.7199	-19.2801
AoE+PSCs	2ug/ml	E11.5AGM	6ug/ml	-3.2000	2.4081	-1.33	0.1839	0.05	-7.9199	1.5199

The GENMOD Procedure

Differences of Condition*Treatment Least Squares Means										
Condition	Treatment	_Condition	_Treatment	Estimate	Standard Error	z Value	Pr > |z|	Alpha	Lower	Upper
AoE+PSCs	2ug/ml	HE+PSCs	0ug/ml	-4.8000	2.4081	-1.99	0.0462	0.05	-9.5199	-0.08014
AoE+PSCs	2ug/ml	HE+PSCs	2ug/ml	10.6000	2.4081	4.40	<.0001	0.05	5.8801	15.3199
AoE+PSCs	2ug/ml	HE+PSCs	6ug/ml	12.6000	2.4081	5.23	<.0001	0.05	7.8801	17.3199
AoE+PSCs	6ug/ml	E11.5AGM	0ug/ml	-51.4000	2.4081	-21.34	<.0001	0.05	-56.1199	-46.6801
AoE+PSCs	6ug/ml	E11.5AGM	2ug/ml	-32.4000	2.4081	-13.45	<.0001	0.05	-37.1199	-27.6801
AoE+PSCs	6ug/ml	E11.5AGM	6ug/ml	-11.6000	2.4081	-4.82	<.0001	0.05	-16.3199	-6.8801
AoE+PSCs	6ug/ml	HE+PSCs	0ug/ml	-13.2000	2.4081	-5.48	<.0001	0.05	-17.9199	-8.4801
AoE+PSCs	6ug/ml	HE+PSCs	2ug/ml	2.2000	2.4081	0.91	0.3609	0.05	-2.5199	6.9199
AoE+PSCs	6ug/ml	HE+PSCs	6ug/ml	4.2000	2.4081	1.74	0.0811	0.05	-0.5199	8.9199
E11.5AGM	0ug/ml	E11.5AGM	2ug/ml	19.0000	2.4081	7.89	<.0001	0.05	14.2801	23.7199
E11.5AGM	0ug/ml	E11.5AGM	6ug/ml	39.8000	2.4081	16.53	<.0001	0.05	35.0801	44.5199
E11.5AGM	0ug/ml	HE+PSCs	0ug/ml	38.2000	2.4081	15.86	<.0001	0.05	33.4801	42.9199
E11.5AGM	0ug/ml	HE+PSCs	2ug/ml	53.6000	2.4081	22.26	<.0001	0.05	48.8801	58.3199
E11.5AGM	0ug/ml	HE+PSCs	6ug/ml	55.6000	2.4081	23.09	<.0001	0.05	50.8801	60.3199
E11.5AGM	2ug/ml	E11.5AGM	6ug/ml	20.8000	2.4081	8.64	<.0001	0.05	16.0801	25.5199
E11.5AGM	2ug/ml	HE+PSCs	0ug/ml	19.2000	2.4081	7.97	<.0001	0.05	14.4801	23.9199
E11.5AGM	2ug/ml	HE+PSCs	2ug/ml	34.6000	2.4081	14.37	<.0001	0.05	29.8801	39.3199
E11.5AGM	2ug/ml	HE+PSCs	6ug/ml	36.6000	2.4081	15.20	<.0001	0.05	31.8801	41.3199
E11.5AGM	6ug/ml	HE+PSCs	0ug/ml	-1.6000	2.4081	-0.66	0.5064	0.05	-6.3199	3.1199
E11.5AGM	6ug/ml	HE+PSCs	2ug/ml	13.8000	2.4081	5.73	<.0001	0.05	9.0801	18.5199
E11.5AGM	6ug/ml	HE+PSCs	6ug/ml	15.8000	2.4081	6.56	<.0001	0.05	11.0801	20.5199
HE+PSCs	0ug/ml	HE+PSCs	2ug/ml	15.4000	2.4081	6.39	<.0001	0.05	10.6801	20.1199
HE+PSCs	0ug/ml	HE+PSCs	6ug/ml	17.4000	2.4081	7.23	<.0001	0.05	12.6801	22.1199
HE+PSCs	2ug/ml	HE+PSCs	6ug/ml	2.0000	2.4081	0.83	0.4062	0.05	-2.7199	6.7199

The GENMOD Procedure

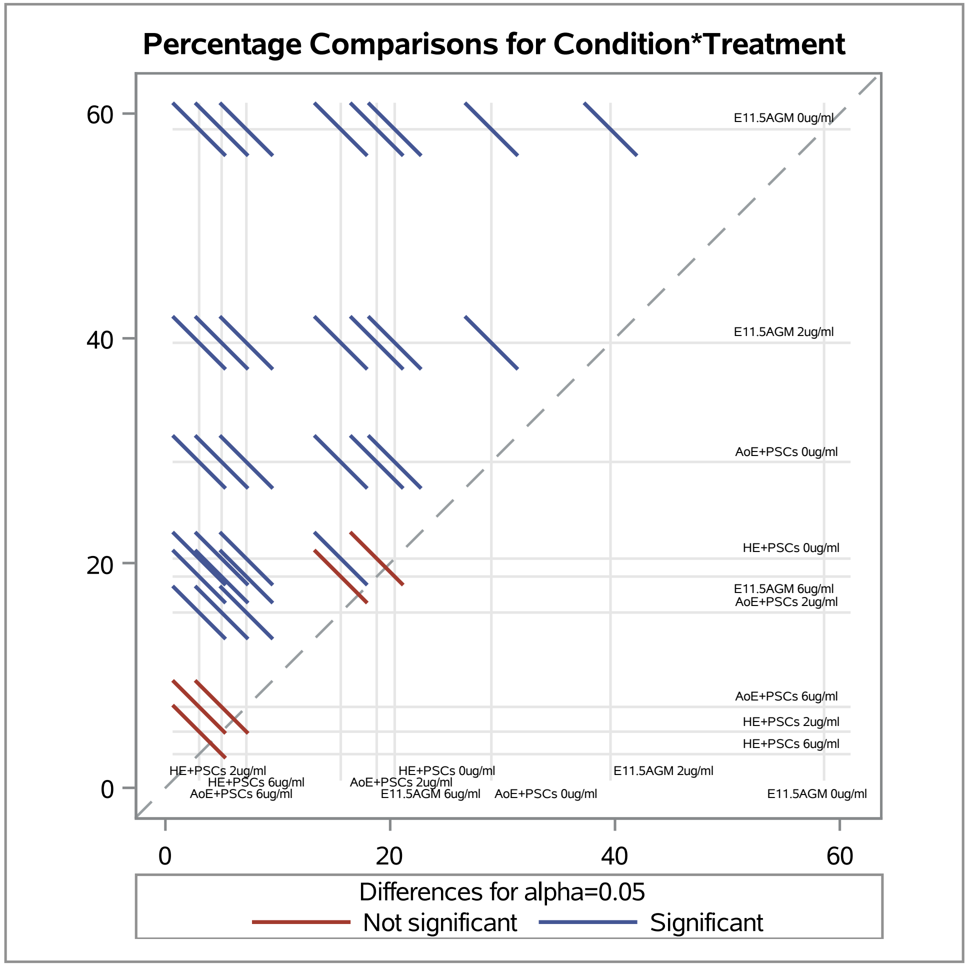

The GLIMMIX Procedure

Model Information	
Data Set	WORK.EXT_FIG10BI_E11WIF1
Response Variable	Count
Response Distribution	Poisson
Link Function	Log
Variance Function	Default
Variance Matrix Blocked By	Dish_ID
Estimation Technique	Maximum Likelihood
Likelihood Approximation	Laplace
Degrees of Freedom Method	Containment

Class Level Information		
Class	Levels	Values
Colony_Type	3	BFU-E CFU-GEMM CFU-GM
Condition	3	AoE+PSCs E11.5AGM HE+PSCs
Treatment	3	0ug/ml 2.5ug/ml 7.5ug/ml
Dish_ID	27	1 2 3 4 5 6 7 8 9 10 11 12 13 14 15 16 17 18 19 20 21 22 23 24 25 26 27

Number of Observations Read	81
Number of Observations Used	81

Dimensions	
G-side Cov. Parameters	1
Columns in X	64
Columns in Z per Subject	1
Subjects (Blocks in V)	27
Max Obs per Subject	3

The GLIMMIX Procedure

Optimization Information	
**Optimization Technique**	Dual Quasi-Newton
**Parameters in Optimization**	28
**Lower Boundaries**	1
**Upper Boundaries**	0
**Fixed Effects**	Not Profiled
**Starting From**	GLM estimates

Iteration History					
**Iteration**	**Restarts**	**Evaluations**	**Objective Function**	**Change**	**Max Gradient**
**0**	**0**	4	812.60767879	.	2693.958
**1**	**0**	8	800.06665561	12.54102317	166.0883
**2**	**0**	4	798.19104488	1.87561073	113.2874
**3**	**0**	3	797.96463544	0.22640944	16.1015
**4**	**0**	4	797.89027771	0.07435772	20.8052
**5**	**0**	4	797.86929936	0.02097836	6.076376
**6**	**0**	2	797.85052737	0.01877199	4.962983
**7**	**0**	2	797.82710509	0.02342228	3.207996
**8**	**0**	3	797.81331484	0.01379025	2.615331
**9**	**0**	3	797.80935934	0.00395550	2.23695
**10**	**0**	4	797.7844555	0.02490384	7.07948
**11**	**0**	3	797.78238854	0.00206696	1.77573
**12**	**0**	3	797.78114529	0.00124325	2.430008
**13**	**0**	2	797.78034079	0.00080451	2.169779
**14**	**0**	2	797.77985559	0.00048520	1.761011
**15**	**0**	2	797.77922192	0.00063367	0.654785

The GLIMMIX Procedure

Iteration History					
Iteration	Restarts	Evaluations	Objective Function	Change	Max Gradient
16	0	4	797.77661205	0.00260987	1.32972
17	0	2	797.77529858	0.00131347	2.46143
18	0	2	797.77373766	0.00156092	0.819707
19	0	2	797.77229536	0.00144230	0.7512
20	0	3	797.77169534	0.00060002	0.477997
21	0	3	797.77143615	0.00025918	0.634641
22	0	4	797.77003728	0.00139887	0.915765
23	0	3	797.76957261	0.00046467	0.776013
24	0	2	797.768903	0.00066962	0.403521
25	0	3	797.76876756	0.00013544	0.228475
26	0	3	797.768717	0.00005055	0.219876
27	0	4	797.76830565	0.00041136	0.569408
28	0	3	797.76821978	0.00008587	0.23842
29	0	4	797.76800843	0.00021135	0.17044
30	0	3	797.76795691	0.00005151	0.14073
31	0	4	797.76785047	0.00010644	0.68095
32	0	2	797.76768736	0.00016311	0.185638
33	0	3	797.7676499	0.00003745	0.23547
34	0	4	797.76737087	0.00027903	0.909748
35	0	3	797.76720466	0.00016621	0.162819
36	0	2	797.76713444	0.00007022	0.209191
37	0	2	797.7670797	0.00005474	0.126827
38	0	3	797.76704442	0.00003527	0.165309

The GLIMMIX Procedure

Iteration History					
**Iteration**	**Restarts**	**Evaluations**	**Objective Function**	**Change**	**Max Gradient**
**39**	**0**	4	797.76674864	0.00029578	0.087063
**40**	**0**	4	797.76666358	0.00008506	0.68387

Convergence criterion (GCONV=1E-8) satisfied.

Fit Statistics	
**-2 Log Likelihood**	797.77
**AIC (smaller is better)**	853.77
**AICC (smaller is better)**	885.00
**BIC (smaller is better)**	890.05
**CAIC (smaller is better)**	918.05
**HQIC (smaller is better)**	864.56

Fit Statistics for Conditional Distribution	
**-2 log L(Count | r. effects)**	704.44
**Pearson Chi-Square**	180.96
**Pearson Chi-Square / DF**	2.23

Covariance Parameter Estimates			
**Cov Parm**	**Subject**	**Estimate**	**Standard Error**
**Intercept**	Dish_ID	0.03037	0.009814

The GLIMMIX Procedure

Solutions for Fixed Effects								
Effect	Colony_Type	Condition	Treatment	Estimate	Standard Error	DF	t Value	Pr > |t|
Intercept				4.4459	0.1186	18	37.50	<.0001
Colony_Type	BFU-E			-1.4175	0.1393	36	-10.17	<.0001
Colony_Type	CFU-GEMM			-1.6251	0.1517	36	-10.71	<.0001
Colony_Type	CFU-GM			0	.	.	.	.
Condition		AoE+PSCs		0.2494	0.1650	36	1.51	0.1394
Condition		E11.5AGM		1.0882	0.1597	36	6.81	<.0001
Condition		HE+PSCs		0	.	.	.	.
Colony_Typ*Condition	BFU-E	AoE+PSCs		-0.3471	0.1990	36	-1.74	0.0896
Colony_Typ*Condition	BFU-E	E11.5AGM		-0.3862	0.1693	36	-2.28	0.0286
Colony_Typ*Condition	BFU-E	HE+PSCs		0	.	.	.	.
Colony_Typ*Condition	CFU-GEMM	AoE+PSCs		-0.1572	0.2086	36	-0.75	0.4560
Colony_Typ*Condition	CFU-GEMM	E11.5AGM		-0.1787	0.1796	36	-0.99	0.3265
Colony_Typ*Condition	CFU-GEMM	HE+PSCs		0	.	.	.	.
Colony_Typ*Condition	CFU-GM	AoE+PSCs		0	.	.	.	.
Colony_Typ*Condition	CFU-GM	E11.5AGM		0	.	.	.	.
Colony_Typ*Condition	CFU-GM	HE+PSCs		0	.	.	.	.
Treatment			0ug/ml	1.6097	0.1580	36	10.19	<.0001
Treatment			2.5ug/ml	0.7871	0.1611	36	4.89	<.0001
Treatment			7.5ug/ml	0	.	.	.	.
Colony_Typ*Treatment	BFU-E		0ug/ml	-0.1651	0.1549	36	-1.07	0.2935
Colony_Typ*Treatment	BFU-E		2.5ug/ml	0.1805	0.1646	36	1.10	0.2800
Colony_Typ*Treatment	BFU-E		7.5ug/ml	0	.	.	.	.
Colony_Typ*Treatment	CFU-GEMM		0ug/ml	0.1225	0.1652	36	0.74	0.4634
Colony_Typ*Treatment	CFU-GEMM		2.5ug/ml	-0.2813	0.1907	36	-1.48	0.1489

The GLIMMIX Procedure

Solutions for Fixed Effects								
**Effect**	**Colony_Type**	**Condition**	**Treatment**	**Estimate**	**Standard Error**	**DF**	**t Value**	**Pr > |t|**
**Colony_Typ*Treatment**	CFU-GEMM		7.5ug/ml	0	.	.	.	.
**Colony_Typ*Treatment**	CFU-GM		0ug/ml	0	.	.	.	.
**Colony_Typ*Treatment**	CFU-GM		2.5ug/ml	0	.	.	.	.
**Colony_Typ*Treatment**	CFU-GM		7.5ug/ml	0	.	.	.	.
**Condition*Treatment**		AoE+PSCs	0ug/ml	-0.05330	0.2211	36	-0.24	0.8109
**Condition*Treatment**		AoE+PSCs	2.5ug/ml	-0.05749	0.2252	36	-0.26	0.8000
**Condition*Treatment**		AoE+PSCs	7.5ug/ml	0	.	.	.	.
**Condition*Treatment**		E11.5AGM	0ug/ml	-0.4751	0.2167	36	-2.19	0.0349
**Condition*Treatment**		E11.5AGM	2.5ug/ml	-0.3763	0.2200	36	-1.71	0.0957
**Condition*Treatment**		E11.5AGM	7.5ug/ml	0	.	.	.	.
**Condition*Treatment**		HE+PSCs	0ug/ml	0	.	.	.	.
**Condition*Treatment**		HE+PSCs	2.5ug/ml	0	.	.	.	.
**Condition*Treatment**		HE+PSCs	7.5ug/ml	0	.	.	.	.
**Colony*Condit*Treatm**	BFU-E	AoE+PSCs	0ug/ml	0.1656	0.2203	36	0.75	0.4570
**Colony*Condit*Treatm**	BFU-E	AoE+PSCs	2.5ug/ml	0.1119	0.2345	36	0.48	0.6361
**Colony*Condit*Treatm**	BFU-E	AoE+PSCs	7.5ug/ml	0	.	.	.	.
**Colony*Condit*Treatm**	BFU-E	E11.5AGM	0ug/ml	0.6465	0.1877	36	3.44	0.0015
**Colony*Condit*Treatm**	BFU-E	E11.5AGM	2.5ug/ml	0.5079	0.1996	36	2.54	0.0154
**Colony*Condit*Treatm**	BFU-E	E11.5AGM	7.5ug/ml	0	.	.	.	.
**Colony*Condit*Treatm**	BFU-E	HE+PSCs	0ug/ml	0	.	.	.	.
**Colony*Condit*Treatm**	BFU-E	HE+PSCs	2.5ug/ml	0	.	.	.	.
**Colony*Condit*Treatm**	BFU-E	HE+PSCs	7.5ug/ml	0	.	.	.	.
**Colony*Condit*Treatm**	CFU-GEMM	AoE+PSCs	0ug/ml	-0.01164	0.2276	36	-0.05	0.9595
**Colony*Condit*Treatm**	CFU-GEMM	AoE+PSCs	2.5ug/ml	0.4708	0.2557	36	1.84	0.0738

The GLIMMIX Procedure

Solutions for Fixed Effects								
Effect	Colony_Type	Condition	Treatment	Estimate	Standard Error	DF	t Value	Pr > |t|
Colony*Condit*Treatm	CFU-GEMM	AoE+PSCs	7.5ug/ml	0	.	.	.	.
Colony*Condit*Treatm	CFU-GEMM	E11.5AGM	0ug/ml	0.2393	0.1969	36	1.22	0.2321
Colony*Condit*Treatm	CFU-GEMM	E11.5AGM	2.5ug/ml	1.0164	0.2214	36	4.59	<.0001
Colony*Condit*Treatm	CFU-GEMM	E11.5AGM	7.5ug/ml	0	.	.	.	.
Colony*Condit*Treatm	CFU-GEMM	HE+PSCs	0ug/ml	0	.	.	.	.
Colony*Condit*Treatm	CFU-GEMM	HE+PSCs	2.5ug/ml	0	.	.	.	.
Colony*Condit*Treatm	CFU-GEMM	HE+PSCs	7.5ug/ml	0	.	.	.	.
Colony*Condit*Treatm	CFU-GM	AoE+PSCs	0ug/ml	0	.	.	.	.
Colony*Condit*Treatm	CFU-GM	AoE+PSCs	2.5ug/ml	0	.	.	.	.
Colony*Condit*Treatm	CFU-GM	AoE+PSCs	7.5ug/ml	0	.	.	.	.
Colony*Condit*Treatm	CFU-GM	E11.5AGM	0ug/ml	0	.	.	.	.
Colony*Condit*Treatm	CFU-GM	E11.5AGM	2.5ug/ml	0	.	.	.	.
Colony*Condit*Treatm	CFU-GM	E11.5AGM	7.5ug/ml	0	.	.	.	.
Colony*Condit*Treatm	CFU-GM	HE+PSCs	0ug/ml	0	.	.	.	.
Colony*Condit*Treatm	CFU-GM	HE+PSCs	2.5ug/ml	0	.	.	.	.
Colony*Condit*Treatm	CFU-GM	HE+PSCs	7.5ug/ml	0	.	.	.	.

Type III Tests of Fixed Effects				
Effect	Num DF	Den DF	F Value	Pr > F
Colony_Type	2	36	1991.95	<.0001
Condition	2	36	58.95	<.0001
Colony_Typ*Condition	4	36	6.87	0.0003
Treatment	2	36	143.95	<.0001
Colony_Typ*Treatment	4	36	8.93	<.0001

The GLIMMIX Procedure

Type III Tests of Fixed Effects				
Effect	Num DF	Den DF	F Value	Pr > F
Condition*Treatment	4	36	0.61	0.6607
Colony*Condit*Treatm	8	36	6.08	<.0001

Condition*Treatment Least Squares Means													
Condition	Treatment	Estimate	Standard Error	DF	t Value	Pr > |t|	Alpha	Lower	Upper	Mean	Standard Error Mean	Lower Mean	Upper Mean
AoE+PSCs	0ug/ml	5.1065	0.1048	36	48.71	<.0001	0.05	4.8939	5.3191	165.09	17.3078	133.47	204.21
AoE+PSCs	2.5ug/ml	4.4033	0.1086	36	40.53	<.0001	0.05	4.1830	4.6237	81.7219	8.8781	65.5619	101.87
AoE+PSCs	7.5ug/ml	3.5130	0.1202	36	29.23	<.0001	0.05	3.2693	3.7567	33.5493	4.0318	26.2927	42.8088
E11.5AGM	0ug/ml	5.7472	0.1027	36	55.97	<.0001	0.05	5.5389	5.9554	313.30	32.1734	254.40	385.84
E11.5AGM	2.5ug/ml	5.2169	0.1039	36	50.20	<.0001	0.05	5.0061	5.4277	184.36	19.1594	149.32	227.61
E11.5AGM	7.5ug/ml	4.3316	0.1097	36	39.47	<.0001	0.05	4.1090	4.5542	76.0660	8.3477	60.8877	95.0279
HE+PSCs	0ug/ml	5.0272	0.1050	36	47.88	<.0001	0.05	4.8142	5.2401	152.50	16.0111	123.25	188.69
HE+PSCs	2.5ug/ml	4.1853	0.1111	36	37.69	<.0001	0.05	3.9600	4.4105	65.7112	7.2976	52.4594	82.3106
HE+PSCs	7.5ug/ml	3.4317	0.1207	36	28.43	<.0001	0.05	3.1869	3.6765	30.9299	3.7330	24.2144	39.5079

Differences of Condition*Treatment Least Squares Means											
Condition	Treatment	_Condition	_Treatment	Estimate	Standard Error	DF	t Value	Pr > |t|	Alpha	Lower	Upper
AoE+PSCs	0ug/ml	AoE+PSCs	2.5ug/ml	0.7032	0.1510	36	4.66	<.0001	0.05	0.3970	1.0094
AoE+PSCs	0ug/ml	AoE+PSCs	7.5ug/ml	1.5935	0.1595	36	9.99	<.0001	0.05	1.2701	1.9169
AoE+PSCs	0ug/ml	E11.5AGM	0ug/ml	-0.6407	0.1468	36	-4.37	0.0001	0.05	-0.9383	-0.3430
AoE+PSCs	0ug/ml	E11.5AGM	2.5ug/ml	-0.1104	0.1476	36	-0.75	0.4595	0.05	-0.4098	0.1890
AoE+PSCs	0ug/ml	E11.5AGM	7.5ug/ml	0.7749	0.1518	36	5.11	<.0001	0.05	0.4671	1.0827
AoE+PSCs	0ug/ml	HE+PSCs	0ug/ml	0.07933	0.1484	36	0.53	0.5962	0.05	-0.2216	0.3802

The GLIMMIX Procedure

Differences of Condition*Treatment Least Squares Means											
Condition	Treatment	_Condition	_Treatment	Estimate	Standard Error	DF	t Value	Pr > |t|	Alpha	Lower	Upper
AoE+PSCs	0ug/ml	HE+PSCs	2.5ug/ml	0.9212	0.1527	36	6.03	<.0001	0.05	0.6115	1.2310
AoE+PSCs	0ug/ml	HE+PSCs	7.5ug/ml	1.6748	0.1599	36	10.48	<.0001	0.05	1.3506	1.9990
AoE+PSCs	2.5ug/ml	AoE+PSCs	7.5ug/ml	0.8903	0.1620	36	5.50	<.0001	0.05	0.5618	1.2188
AoE+PSCs	2.5ug/ml	E11.5AGM	0ug/ml	-1.3438	0.1495	36	-8.99	<.0001	0.05	-1.6470	-1.0407
AoE+PSCs	2.5ug/ml	E11.5AGM	2.5ug/ml	-0.8136	0.1503	36	-5.41	<.0001	0.05	-1.1185	-0.5087
AoE+PSCs	2.5ug/ml	E11.5AGM	7.5ug/ml	0.07172	0.1544	36	0.46	0.6451	0.05	-0.2415	0.3849
AoE+PSCs	2.5ug/ml	HE+PSCs	0ug/ml	-0.6239	0.1511	36	-4.13	0.0002	0.05	-0.9303	-0.3175
AoE+PSCs	2.5ug/ml	HE+PSCs	2.5ug/ml	0.2181	0.1553	36	1.40	0.1690	0.05	-0.09700	0.5331
AoE+PSCs	2.5ug/ml	HE+PSCs	7.5ug/ml	0.9716	0.1624	36	5.98	<.0001	0.05	0.6423	1.3009
AoE+PSCs	7.5ug/ml	E11.5AGM	0ug/ml	-2.2342	0.1581	36	-14.13	<.0001	0.05	-2.5547	-1.9136
AoE+PSCs	7.5ug/ml	E11.5AGM	2.5ug/ml	-1.7039	0.1589	36	-10.72	<.0001	0.05	-2.0261	-1.3817
AoE+PSCs	7.5ug/ml	E11.5AGM	7.5ug/ml	-0.8186	0.1627	36	-5.03	<.0001	0.05	-1.1486	-0.4885
AoE+PSCs	7.5ug/ml	HE+PSCs	0ug/ml	-1.5142	0.1596	36	-9.49	<.0001	0.05	-1.8378	-1.1905
AoE+PSCs	7.5ug/ml	HE+PSCs	2.5ug/ml	-0.6723	0.1636	36	-4.11	0.0002	0.05	-1.0040	-0.3405
AoE+PSCs	7.5ug/ml	HE+PSCs	7.5ug/ml	0.08129	0.1702	36	0.48	0.6358	0.05	-0.2639	0.4265
E11.5AGM	0ug/ml	E11.5AGM	2.5ug/ml	0.5303	0.1461	36	3.63	0.0009	0.05	0.2340	0.8266
E11.5AGM	0ug/ml	E11.5AGM	7.5ug/ml	1.4156	0.1503	36	9.42	<.0001	0.05	1.1108	1.7204
E11.5AGM	0ug/ml	HE+PSCs	0ug/ml	0.7200	0.1469	36	4.90	<.0001	0.05	0.4221	1.0178
E11.5AGM	0ug/ml	HE+PSCs	2.5ug/ml	1.5619	0.1513	36	10.33	<.0001	0.05	1.2551	1.8687
E11.5AGM	0ug/ml	HE+PSCs	7.5ug/ml	2.3154	0.1585	36	14.61	<.0001	0.05	1.9941	2.6368
E11.5AGM	2.5ug/ml	E11.5AGM	7.5ug/ml	0.8853	0.1511	36	5.86	<.0001	0.05	0.5788	1.1918
E11.5AGM	2.5ug/ml	HE+PSCs	0ug/ml	0.1897	0.1477	36	1.28	0.2073	0.05	-0.1099	0.4893
E11.5AGM	2.5ug/ml	HE+PSCs	2.5ug/ml	1.0316	0.1521	36	6.78	<.0001	0.05	0.7232	1.3401
E11.5AGM	2.5ug/ml	HE+PSCs	7.5ug/ml	1.7852	0.1593	36	11.21	<.0001	0.05	1.4622	2.1082

The GLIMMIX Procedure

Differences of Condition*Treatment Least Squares Means											
Condition	Treatment	_Condition	_Treatment	Estimate	Standard Error	DF	t Value	Pr > |t|	Alpha	Lower	Upper
E11.5AGM	7.5ug/ml	HE+PSCs	0ug/ml	-0.6956	0.1519	36	-4.58	<.0001	0.05	-1.0036	-0.3876
E11.5AGM	7.5ug/ml	HE+PSCs	2.5ug/ml	0.1463	0.1561	36	0.94	0.3549	0.05	-0.1703	0.4630
E11.5AGM	7.5ug/ml	HE+PSCs	7.5ug/ml	0.8999	0.1631	36	5.52	<.0001	0.05	0.5691	1.2307
HE+PSCs	0ug/ml	HE+PSCs	2.5ug/ml	0.8419	0.1528	36	5.51	<.0001	0.05	0.5320	1.1519
HE+PSCs	0ug/ml	HE+PSCs	7.5ug/ml	1.5955	0.1600	36	9.97	<.0001	0.05	1.2710	1.9199
HE+PSCs	2.5ug/ml	HE+PSCs	7.5ug/ml	0.7535	0.1640	36	4.60	<.0001	0.05	0.4210	1.0861

Colony*Condit*Treatm Least Squares Means														
Colony_Type	Condition	Treatment	Estimate	Standard Error	DF	t Value	Pr > |t|	Alpha	Lower	Upper	Mean	Standard Error Mean	Lower Mean	Upper Mean
BFU-E	AoE+PSCs	0ug/ml	4.4876	0.1177	36	38.12	<.0001	0.05	4.2488	4.7263	88.9036	10.4660	70.0212	112.88
BFU-E	AoE+PSCs	2.5ug/ml	3.9527	0.1282	36	30.82	<.0001	0.05	3.6927	4.2128	52.0779	6.6782	40.1519	67.5463
BFU-E	AoE+PSCs	7.5ug/ml	2.9306	0.1658	36	17.68	<.0001	0.05	2.5944	3.2668	18.7394	3.1066	13.3888	26.2283
BFU-E	E11.5AGM	0ug/ml	5.3463	0.1082	36	49.40	<.0001	0.05	5.1268	5.5657	209.82	22.7060	168.48	261.32
BFU-E	E11.5AGM	2.5ug/ml	4.8296	0.1130	36	42.73	<.0001	0.05	4.6004	5.0588	125.16	14.1463	99.5200	157.40
BFU-E	E11.5AGM	7.5ug/ml	3.7304	0.1344	36	27.75	<.0001	0.05	3.4577	4.0030	41.6945	5.6050	31.7448	54.7626
BFU-E	HE+PSCs	0ug/ml	4.4730	0.1180	36	37.92	<.0001	0.05	4.2337	4.7122	87.6153	10.3353	68.9731	111.30
BFU-E	HE+PSCs	2.5ug/ml	3.9961	0.1270	36	31.45	<.0001	0.05	3.7384	4.2537	54.3838	6.9091	42.0312	70.3668
BFU-E	HE+PSCs	7.5ug/ml	3.0284	0.1609	36	18.82	<.0001	0.05	2.7020	3.3548	20.6637	3.3256	14.9092	28.6391
CFU-GEMM	AoE+PSCs	0ug/ml	4.5803	0.1163	36	39.38	<.0001	0.05	4.3444	4.8162	97.5419	11.3452	77.0452	123.49
CFU-GEMM	AoE+PSCs	2.5ug/ml	3.8323	0.1313	36	29.18	<.0001	0.05	3.5659	4.0986	46.1666	6.0634	35.3710	60.2571
CFU-GEMM	AoE+PSCs	7.5ug/ml	2.9131	0.1667	36	17.48	<.0001	0.05	2.5750	3.2512	18.4138	3.0695	13.1317	25.8205
CFU-GEMM	E11.5AGM	0ug/ml	5.2266	0.1091	36	47.89	<.0001	0.05	5.0053	5.4480	186.17	20.3188	149.20	232.29
CFU-GEMM	E11.5AGM	2.5ug/ml	4.8762	0.1125	36	43.35	<.0001	0.05	4.6481	5.1043	131.13	14.7512	104.38	164.74

The GLIMMIX Procedure

Colony*Condit*Treatm Least Squares Means														
Colony_Type	Condition	Treatment	Estimate	Standard Error	DF	t Value	Pr > |t|	Alpha	Lower	Upper	Mean	Standard Error Mean	Lower Mean	Upper Mean
CFU-GEMM	E11.5AGM	7.5ug/ml	3.7304	0.1344	36	27.75	<.0001	0.05	3.4577	4.0030	41.6941	5.6050	31.7445	54.7622
CFU-GEMM	HE+PSCs	0ug/ml	4.5530	0.1167	36	39.01	<.0001	0.05	4.3163	4.7897	94.9160	11.0786	74.9089	120.27
CFU-GEMM	HE+PSCs	2.5ug/ml	3.3267	0.1477	36	22.53	<.0001	0.05	3.0272	3.6262	27.8457	4.1125	20.6384	37.5699
CFU-GEMM	HE+PSCs	7.5ug/ml	2.8209	0.1718	36	16.42	<.0001	0.05	2.4725	3.1692	16.7914	2.8843	11.8521	23.7893
CFU-GM	AoE+PSCs	0ug/ml	6.2517	0.1038	36	60.25	<.0001	0.05	6.0413	6.4621	518.89	53.8409	420.42	640.42
CFU-GM	AoE+PSCs	2.5ug/ml	5.4250	0.1077	36	50.37	<.0001	0.05	5.2066	5.6434	227.00	24.4466	182.47	282.42
CFU-GM	AoE+PSCs	7.5ug/ml	4.6953	0.1149	36	40.86	<.0001	0.05	4.4623	4.9284	109.43	12.5739	86.6864	138.15
CFU-GM	E11.5AGM	0ug/ml	6.6686	0.1027	36	64.94	<.0001	0.05	6.4603	6.8769	787.29	80.8517	639.27	969.60
CFU-GM	E11.5AGM	2.5ug/ml	5.9449	0.1049	36	56.69	<.0001	0.05	5.7322	6.1575	381.79	40.0365	308.65	472.27
CFU-GM	E11.5AGM	7.5ug/ml	5.5341	0.1070	36	51.74	<.0001	0.05	5.3171	5.7510	253.17	27.0809	203.80	314.51
CFU-GM	HE+PSCs	0ug/ml	6.0556	0.1044	36	57.99	<.0001	0.05	5.8438	6.2674	426.49	44.5373	345.09	527.09
CFU-GM	HE+PSCs	2.5ug/ml	5.2331	0.1092	36	47.94	<.0001	0.05	5.0117	5.4545	187.37	20.4530	150.16	233.80
CFU-GM	HE+PSCs	7.5ug/ml	4.4459	0.1186	36	37.50	<.0001	0.05	4.2054	4.6864	85.2787	10.1117	67.0507	108.46

The GLIMMIX Procedure

Differences of Colony*Condit*Treatm Least Squares Means													
Colony_Type	Condition	Treatment	_Colony_Type	_Condition	_Treatment	Estimate	Standard Error	DF	t Value	Pr > |t|	Alpha	Lower	Upper
BFU-E	AoE+PSCs	0ug/ml	BFU-E	AoE+PSCs	2.5ug/ml	0.5348	0.1741	36	3.07	0.0040	0.05	0.1818	0.8879
BFU-E	AoE+PSCs	0ug/ml	BFU-E	AoE+PSCs	7.5ug/ml	1.5569	0.2033	36	7.66	<.0001	0.05	1.1446	1.9693
BFU-E	AoE+PSCs	0ug/ml	BFU-E	E11.5AGM	0ug/ml	-0.8587	0.1599	36	-5.37	<.0001	0.05	-1.1830	-0.5344
BFU-E	AoE+PSCs	0ug/ml	BFU-E	E11.5AGM	2.5ug/ml	-0.3420	0.1632	36	-2.10	0.0432	0.05	-0.6730	-0.01105
BFU-E	AoE+PSCs	0ug/ml	BFU-E	E11.5AGM	7.5ug/ml	0.7572	0.1787	36	4.24	0.0001	0.05	0.3948	1.1196
BFU-E	AoE+PSCs	0ug/ml	BFU-E	HE+PSCs	0ug/ml	0.01460	0.1667	36	0.09	0.9307	0.05	-0.3234	0.3526
BFU-E	AoE+PSCs	0ug/ml	BFU-E	HE+PSCs	2.5ug/ml	0.4915	0.1732	36	2.84	0.0074	0.05	0.1402	0.8428
BFU-E	AoE+PSCs	0ug/ml	BFU-E	HE+PSCs	7.5ug/ml	1.4592	0.1994	36	7.32	<.0001	0.05	1.0548	1.8636
BFU-E	AoE+PSCs	0ug/ml	CFU-GEMM	AoE+PSCs	0ug/ml	-0.09273	0.08446	36	-1.10	0.2795	0.05	-0.2640	0.07856
BFU-E	AoE+PSCs	0ug/ml	CFU-GEMM	AoE+PSCs	2.5ug/ml	0.6553	0.1764	36	3.72	0.0007	0.05	0.2976	1.0130
BFU-E	AoE+PSCs	0ug/ml	CFU-GEMM	AoE+PSCs	7.5ug/ml	1.5745	0.2041	36	7.72	<.0001	0.05	1.1606	1.9883
BFU-E	AoE+PSCs	0ug/ml	CFU-GEMM	E11.5AGM	0ug/ml	-0.7391	0.1605	36	-4.60	<.0001	0.05	-1.0647	-0.4135
BFU-E	AoE+PSCs	0ug/ml	CFU-GEMM	E11.5AGM	2.5ug/ml	-0.3886	0.1628	36	-2.39	0.0224	0.05	-0.7189	-0.05842
BFU-E	AoE+PSCs	0ug/ml	CFU-GEMM	E11.5AGM	7.5ug/ml	0.7572	0.1787	36	4.24	0.0001	0.05	0.3948	1.1196
BFU-E	AoE+PSCs	0ug/ml	CFU-GEMM	HE+PSCs	0ug/ml	-0.06544	0.1658	36	-0.39	0.6954	0.05	-0.4017	0.2708
BFU-E	AoE+PSCs	0ug/ml	CFU-GEMM	HE+PSCs	2.5ug/ml	1.1609	0.1889	36	6.15	<.0001	0.05	0.7778	1.5439
BFU-E	AoE+PSCs	0ug/ml	CFU-GEMM	HE+PSCs	7.5ug/ml	1.6667	0.2082	36	8.00	<.0001	0.05	1.2444	2.0890
BFU-E	AoE+PSCs	0ug/ml	CFU-GM	AoE+PSCs	0ug/ml	-1.7641	0.06611	36	-26.68	<.0001	0.05	-1.8982	-1.6301
BFU-E	AoE+PSCs	0ug/ml	CFU-GM	AoE+PSCs	2.5ug/ml	-0.9374	0.1596	36	-5.88	<.0001	0.05	-1.2610	-0.6138
BFU-E	AoE+PSCs	0ug/ml	CFU-GM	AoE+PSCs	7.5ug/ml	-0.2078	0.1645	36	-1.26	0.2147	0.05	-0.5414	0.1258
BFU-E	AoE+PSCs	0ug/ml	CFU-GM	E11.5AGM	0ug/ml	-2.1811	0.1562	36	-13.96	<.0001	0.05	-2.4979	-1.8642
BFU-E	AoE+PSCs	0ug/ml	CFU-GM	E11.5AGM	2.5ug/ml	-1.4573	0.1577	36	-9.24	<.0001	0.05	-1.7771	-1.1376
BFU-E	AoE+PSCs	0ug/ml	CFU-GM	E11.5AGM	7.5ug/ml	-1.0465	0.1591	36	-6.58	<.0001	0.05	-1.3691	-0.7239
BFU-E	AoE+PSCs	0ug/ml	CFU-GM	HE+PSCs	0ug/ml	-1.5680	0.1574	36	-9.96	<.0001	0.05	-1.8872	-1.2489

The GLIMMIX Procedure

Differences of Colony*Condit*Treatm Least Squares Means													
Colony_Type	Condition	Treatment	_Colony_Type	_Condition	_Treatment	Estimate	Standard Error	DF	t Value	Pr > |t|	Alpha	Lower	Upper
BFU-E	AoE+PSCs	0ug/ml	CFU-GM	HE+PSCs	2.5ug/ml	-0.7455	0.1605	36	-4.64	<.0001	0.05	-1.0711	-0.4199
BFU-E	AoE+PSCs	0ug/ml	CFU-GM	HE+PSCs	7.5ug/ml	0.04163	0.1671	36	0.25	0.8047	0.05	-0.2972	0.3805
BFU-E	AoE+PSCs	2.5ug/ml	BFU-E	AoE+PSCs	7.5ug/ml	1.0221	0.2096	36	4.88	<.0001	0.05	0.5971	1.4471
BFU-E	AoE+PSCs	2.5ug/ml	BFU-E	E11.5AGM	0ug/ml	-1.3935	0.1678	36	-8.30	<.0001	0.05	-1.7338	-1.0532
BFU-E	AoE+PSCs	2.5ug/ml	BFU-E	E11.5AGM	2.5ug/ml	-0.8768	0.1709	36	-5.13	<.0001	0.05	-1.2235	-0.5302
BFU-E	AoE+PSCs	2.5ug/ml	BFU-E	E11.5AGM	7.5ug/ml	0.2224	0.1858	36	1.20	0.2391	0.05	-0.1544	0.5992
BFU-E	AoE+PSCs	2.5ug/ml	BFU-E	HE+PSCs	0ug/ml	-0.5202	0.1742	36	-2.99	0.0051	0.05	-0.8736	-0.1668
BFU-E	AoE+PSCs	2.5ug/ml	BFU-E	HE+PSCs	2.5ug/ml	-0.04332	0.1805	36	-0.24	0.8117	0.05	-0.4094	0.3227
BFU-E	AoE+PSCs	2.5ug/ml	BFU-E	HE+PSCs	7.5ug/ml	0.9244	0.2058	36	4.49	<.0001	0.05	0.5071	1.3417
BFU-E	AoE+PSCs	2.5ug/ml	CFU-GEMM	AoE+PSCs	0ug/ml	-0.6275	0.1731	36	-3.62	0.0009	0.05	-0.9787	-0.2764
BFU-E	AoE+PSCs	2.5ug/ml	CFU-GEMM	AoE+PSCs	2.5ug/ml	0.1205	0.1157	36	1.04	0.3046	0.05	-0.1141	0.3551
BFU-E	AoE+PSCs	2.5ug/ml	CFU-GEMM	AoE+PSCs	7.5ug/ml	1.0396	0.2103	36	4.94	<.0001	0.05	0.6131	1.4661
BFU-E	AoE+PSCs	2.5ug/ml	CFU-GEMM	E11.5AGM	0ug/ml	-1.2739	0.1684	36	-7.56	<.0001	0.05	-1.6154	-0.9324
BFU-E	AoE+PSCs	2.5ug/ml	CFU-GEMM	E11.5AGM	2.5ug/ml	-0.9235	0.1706	36	-5.41	<.0001	0.05	-1.2694	-0.5775
BFU-E	AoE+PSCs	2.5ug/ml	CFU-GEMM	E11.5AGM	7.5ug/ml	0.2224	0.1858	36	1.20	0.2391	0.05	-0.1544	0.5992
BFU-E	AoE+PSCs	2.5ug/ml	CFU-GEMM	HE+PSCs	0ug/ml	-0.6003	0.1734	36	-3.46	0.0014	0.05	-0.9519	-0.2486
BFU-E	AoE+PSCs	2.5ug/ml	CFU-GEMM	HE+PSCs	2.5ug/ml	0.6261	0.1956	36	3.20	0.0029	0.05	0.2294	1.0227
BFU-E	AoE+PSCs	2.5ug/ml	CFU-GEMM	HE+PSCs	7.5ug/ml	1.1319	0.2143	36	5.28	<.0001	0.05	0.6972	1.5666
BFU-E	AoE+PSCs	2.5ug/ml	CFU-GM	AoE+PSCs	0ug/ml	-2.2989	0.1650	36	-13.94	<.0001	0.05	-2.6335	-1.9644
BFU-E	AoE+PSCs	2.5ug/ml	CFU-GM	AoE+PSCs	2.5ug/ml	-1.4722	0.08793	36	-16.74	<.0001	0.05	-1.6506	-1.2939
BFU-E	AoE+PSCs	2.5ug/ml	CFU-GM	AoE+PSCs	7.5ug/ml	-0.7426	0.1722	36	-4.31	0.0001	0.05	-1.0917	-0.3934
BFU-E	AoE+PSCs	2.5ug/ml	CFU-GM	E11.5AGM	0ug/ml	-2.7159	0.1643	36	-16.53	<.0001	0.05	-3.0491	-2.3827
BFU-E	AoE+PSCs	2.5ug/ml	CFU-GM	E11.5AGM	2.5ug/ml	-1.9921	0.1657	36	-12.03	<.0001	0.05	-2.3281	-1.6562
BFU-E	AoE+PSCs	2.5ug/ml	CFU-GM	E11.5AGM	7.5ug/ml	-1.5813	0.1670	36	-9.47	<.0001	0.05	-1.9200	-1.2427

The GLIMMIX Procedure

Differences of Colony*Condit*Treatm Least Squares Means													
Colony_Type	Condition	Treatment	_Colony_Type	_Condition	_Treatment	Estimate	Standard Error	DF	t Value	Pr > |t|	Alpha	Lower	Upper
BFU-E	AoE+PSCs	2.5ug/ml	CFU-GM	HE+PSCs	0ug/ml	-2.1028	0.1654	36	-12.72	<.0001	0.05	-2.4382	-1.7674
BFU-E	AoE+PSCs	2.5ug/ml	CFU-GM	HE+PSCs	2.5ug/ml	-1.2803	0.1684	36	-7.60	<.0001	0.05	-1.6218	-0.9388
BFU-E	AoE+PSCs	2.5ug/ml	CFU-GM	HE+PSCs	7.5ug/ml	-0.4932	0.1746	36	-2.82	0.0077	0.05	-0.8473	-0.1390
BFU-E	AoE+PSCs	7.5ug/ml	BFU-E	E11.5AGM	0ug/ml	-2.4156	0.1980	36	-12.20	<.0001	0.05	-2.8171	-2.0141
BFU-E	AoE+PSCs	7.5ug/ml	BFU-E	E11.5AGM	2.5ug/ml	-1.8990	0.2006	36	-9.46	<.0001	0.05	-2.3059	-1.4920
BFU-E	AoE+PSCs	7.5ug/ml	BFU-E	E11.5AGM	7.5ug/ml	-0.7997	0.2134	36	-3.75	0.0006	0.05	-1.2326	-0.3669
BFU-E	AoE+PSCs	7.5ug/ml	BFU-E	HE+PSCs	0ug/ml	-1.5423	0.2035	36	-7.58	<.0001	0.05	-1.9550	-1.1297
BFU-E	AoE+PSCs	7.5ug/ml	BFU-E	HE+PSCs	2.5ug/ml	-1.0654	0.2088	36	-5.10	<.0001	0.05	-1.4889	-0.6419
BFU-E	AoE+PSCs	7.5ug/ml	BFU-E	HE+PSCs	7.5ug/ml	-0.09775	0.2310	36	-0.42	0.6747	0.05	-0.5662	0.3707
BFU-E	AoE+PSCs	7.5ug/ml	CFU-GEMM	AoE+PSCs	0ug/ml	-1.6497	0.2025	36	-8.15	<.0001	0.05	-2.0604	-1.2389
BFU-E	AoE+PSCs	7.5ug/ml	CFU-GEMM	AoE+PSCs	2.5ug/ml	-0.9016	0.2115	36	-4.26	0.0001	0.05	-1.3305	-0.4727
BFU-E	AoE+PSCs	7.5ug/ml	CFU-GEMM	AoE+PSCs	7.5ug/ml	0.01753	0.1865	36	0.09	0.9256	0.05	-0.3606	0.3957
BFU-E	AoE+PSCs	7.5ug/ml	CFU-GEMM	E11.5AGM	0ug/ml	-2.2960	0.1985	36	-11.57	<.0001	0.05	-2.6985	-1.8935
BFU-E	AoE+PSCs	7.5ug/ml	CFU-GEMM	E11.5AGM	2.5ug/ml	-1.9456	0.2003	36	-9.71	<.0001	0.05	-2.3519	-1.5393
BFU-E	AoE+PSCs	7.5ug/ml	CFU-GEMM	E11.5AGM	7.5ug/ml	-0.7997	0.2134	36	-3.75	0.0006	0.05	-1.2326	-0.3669
BFU-E	AoE+PSCs	7.5ug/ml	CFU-GEMM	HE+PSCs	0ug/ml	-1.6224	0.2027	36	-8.00	<.0001	0.05	-2.0335	-1.2112
BFU-E	AoE+PSCs	7.5ug/ml	CFU-GEMM	HE+PSCs	2.5ug/ml	-0.3961	0.2220	36	-1.78	0.0828	0.05	-0.8463	0.05416
BFU-E	AoE+PSCs	7.5ug/ml	CFU-GEMM	HE+PSCs	7.5ug/ml	0.1098	0.2386	36	0.46	0.6483	0.05	-0.3742	0.5938
BFU-E	AoE+PSCs	7.5ug/ml	CFU-GM	AoE+PSCs	0ug/ml	-3.3211	0.1956	36	-16.98	<.0001	0.05	-3.7177	-2.9244
BFU-E	AoE+PSCs	7.5ug/ml	CFU-GM	AoE+PSCs	2.5ug/ml	-2.4943	0.1977	36	-12.62	<.0001	0.05	-2.8952	-2.0935
BFU-E	AoE+PSCs	7.5ug/ml	CFU-GM	AoE+PSCs	7.5ug/ml	-1.7647	0.1421	36	-12.42	<.0001	0.05	-2.0528	-1.4766
BFU-E	AoE+PSCs	7.5ug/ml	CFU-GM	E11.5AGM	0ug/ml	-3.7380	0.1950	36	-19.17	<.0001	0.05	-4.1335	-3.3425
BFU-E	AoE+PSCs	7.5ug/ml	CFU-GM	E11.5AGM	2.5ug/ml	-3.0142	0.1962	36	-15.37	<.0001	0.05	-3.4121	-2.6164
BFU-E	AoE+PSCs	7.5ug/ml	CFU-GM	E11.5AGM	7.5ug/ml	-2.6034	0.1973	36	-13.20	<.0001	0.05	-3.0036	-2.2033

The GLIMMIX Procedure

Differences of Colony*Condit*Treatm Least Squares Means													
Colony_Type	Condition	Treatment	_Colony_Type	_Condition	_Treatment	Estimate	Standard Error	DF	t Value	Pr > |t|	Alpha	Lower	Upper
BFU-E	AoE+PSCs	7.5ug/ml	CFU-GM	HE+PSCs	0ug/ml	-3.1250	0.1959	36	-15.95	<.0001	0.05	-3.5223	-2.7276
BFU-E	AoE+PSCs	7.5ug/ml	CFU-GM	HE+PSCs	2.5ug/ml	-2.3024	0.1984	36	-11.60	<.0001	0.05	-2.7049	-1.9000
BFU-E	AoE+PSCs	7.5ug/ml	CFU-GM	HE+PSCs	7.5ug/ml	-1.5153	0.2037	36	-7.44	<.0001	0.05	-1.9285	-1.1021
BFU-E	E11.5AGM	0ug/ml	BFU-E	E11.5AGM	2.5ug/ml	0.5167	0.1565	36	3.30	0.0022	0.05	0.1993	0.8340
BFU-E	E11.5AGM	0ug/ml	BFU-E	E11.5AGM	7.5ug/ml	1.6159	0.1726	36	9.36	<.0001	0.05	1.2659	1.9659
BFU-E	E11.5AGM	0ug/ml	BFU-E	HE+PSCs	0ug/ml	0.8733	0.1601	36	5.46	<.0001	0.05	0.5486	1.1980
BFU-E	E11.5AGM	0ug/ml	BFU-E	HE+PSCs	2.5ug/ml	1.3502	0.1669	36	8.09	<.0001	0.05	1.0117	1.6887
BFU-E	E11.5AGM	0ug/ml	BFU-E	HE+PSCs	7.5ug/ml	2.3179	0.1939	36	11.95	<.0001	0.05	1.9246	2.7112
BFU-E	E11.5AGM	0ug/ml	CFU-GEMM	AoE+PSCs	0ug/ml	0.7660	0.1589	36	4.82	<.0001	0.05	0.4438	1.0882
BFU-E	E11.5AGM	0ug/ml	CFU-GEMM	AoE+PSCs	2.5ug/ml	1.5140	0.1702	36	8.90	<.0001	0.05	1.1689	1.8591
BFU-E	E11.5AGM	0ug/ml	CFU-GEMM	AoE+PSCs	7.5ug/ml	2.4332	0.1987	36	12.24	<.0001	0.05	2.0301	2.8362
BFU-E	E11.5AGM	0ug/ml	CFU-GEMM	E11.5AGM	0ug/ml	0.1196	0.05810	36	2.06	0.0468	0.05	0.001783	0.2375
BFU-E	E11.5AGM	0ug/ml	CFU-GEMM	E11.5AGM	2.5ug/ml	0.4701	0.1561	36	3.01	0.0047	0.05	0.1535	0.7866
BFU-E	E11.5AGM	0ug/ml	CFU-GEMM	E11.5AGM	7.5ug/ml	1.6159	0.1726	36	9.36	<.0001	0.05	1.2659	1.9659
BFU-E	E11.5AGM	0ug/ml	CFU-GEMM	HE+PSCs	0ug/ml	0.7933	0.1592	36	4.98	<.0001	0.05	0.4705	1.1161
BFU-E	E11.5AGM	0ug/ml	CFU-GEMM	HE+PSCs	2.5ug/ml	2.0196	0.1831	36	11.03	<.0001	0.05	1.6483	2.3909
BFU-E	E11.5AGM	0ug/ml	CFU-GEMM	HE+PSCs	7.5ug/ml	2.5254	0.2030	36	12.44	<.0001	0.05	2.1137	2.9371
BFU-E	E11.5AGM	0ug/ml	CFU-GM	AoE+PSCs	0ug/ml	-0.9054	0.1499	36	-6.04	<.0001	0.05	-1.2095	-0.6014
BFU-E	E11.5AGM	0ug/ml	CFU-GM	AoE+PSCs	2.5ug/ml	-0.07871	0.1527	36	-0.52	0.6093	0.05	-0.3883	0.2309
BFU-E	E11.5AGM	0ug/ml	CFU-GM	AoE+PSCs	7.5ug/ml	0.6509	0.1578	36	4.12	0.0002	0.05	0.3308	0.9710
BFU-E	E11.5AGM	0ug/ml	CFU-GM	E11.5AGM	0ug/ml	-1.3223	0.04484	36	-29.49	<.0001	0.05	-1.4133	-1.2314
BFU-E	E11.5AGM	0ug/ml	CFU-GM	E11.5AGM	2.5ug/ml	-0.5986	0.1507	36	-3.97	0.0003	0.05	-0.9042	-0.2930
BFU-E	E11.5AGM	0ug/ml	CFU-GM	E11.5AGM	7.5ug/ml	-0.1878	0.1522	36	-1.23	0.2251	0.05	-0.4964	0.1208
BFU-E	E11.5AGM	0ug/ml	CFU-GM	HE+PSCs	0ug/ml	-0.7093	0.1504	36	-4.72	<.0001	0.05	-1.0143	-0.4043

The GLIMMIX Procedure

Differences of Colony*Condit*Treatm Least Squares Means													
Colony_Type	Condition	Treatment	_Colony_Type	_Condition	_Treatment	Estimate	Standard Error	DF	t Value	Pr > |t|	Alpha	Lower	Upper
BFU-E	E11.5AGM	0ug/ml	CFU-GM	HE+PSCs	2.5ug/ml	0.1132	0.1537	36	0.74	0.4662	0.05	-0.1985	0.4249
BFU-E	E11.5AGM	0ug/ml	CFU-GM	HE+PSCs	7.5ug/ml	0.9003	0.1605	36	5.61	<.0001	0.05	0.5748	1.2259
BFU-E	E11.5AGM	2.5ug/ml	BFU-E	E11.5AGM	7.5ug/ml	1.0992	0.1756	36	6.26	<.0001	0.05	0.7430	1.4554
BFU-E	E11.5AGM	2.5ug/ml	BFU-E	HE+PSCs	0ug/ml	0.3566	0.1634	36	2.18	0.0356	0.05	0.02530	0.6880
BFU-E	E11.5AGM	2.5ug/ml	BFU-E	HE+PSCs	2.5ug/ml	0.8335	0.1700	36	4.90	<.0001	0.05	0.4887	1.1784
BFU-E	E11.5AGM	2.5ug/ml	BFU-E	HE+PSCs	7.5ug/ml	1.8012	0.1967	36	9.16	<.0001	0.05	1.4024	2.2000
BFU-E	E11.5AGM	2.5ug/ml	CFU-GEMM	AoE+PSCs	0ug/ml	0.2493	0.1622	36	1.54	0.1330	0.05	-0.07962	0.5782
BFU-E	E11.5AGM	2.5ug/ml	CFU-GEMM	AoE+PSCs	2.5ug/ml	0.9973	0.1733	36	5.76	<.0001	0.05	0.6459	1.3487
BFU-E	E11.5AGM	2.5ug/ml	CFU-GEMM	AoE+PSCs	7.5ug/ml	1.9165	0.2014	36	9.52	<.0001	0.05	1.5080	2.3249
BFU-E	E11.5AGM	2.5ug/ml	CFU-GEMM	E11.5AGM	0ug/ml	-0.3971	0.1571	36	-2.53	0.0160	0.05	-0.7157	-0.07839
BFU-E	E11.5AGM	2.5ug/ml	CFU-GEMM	E11.5AGM	2.5ug/ml	-0.04661	0.07191	36	-0.65	0.5210	0.05	-0.1924	0.09922
BFU-E	E11.5AGM	2.5ug/ml	CFU-GEMM	E11.5AGM	7.5ug/ml	1.0992	0.1756	36	6.26	<.0001	0.05	0.7430	1.4554
BFU-E	E11.5AGM	2.5ug/ml	CFU-GEMM	HE+PSCs	0ug/ml	0.2766	0.1625	36	1.70	0.0973	0.05	-0.05292	0.6061
BFU-E	E11.5AGM	2.5ug/ml	CFU-GEMM	HE+PSCs	2.5ug/ml	1.5029	0.1860	36	8.08	<.0001	0.05	1.1257	1.8801
BFU-E	E11.5AGM	2.5ug/ml	CFU-GEMM	HE+PSCs	7.5ug/ml	2.0087	0.2056	36	9.77	<.0001	0.05	1.5917	2.4257
BFU-E	E11.5AGM	2.5ug/ml	CFU-GM	AoE+PSCs	0ug/ml	-1.4221	0.1534	36	-9.27	<.0001	0.05	-1.7333	-1.1109
BFU-E	E11.5AGM	2.5ug/ml	CFU-GM	AoE+PSCs	2.5ug/ml	-0.5954	0.1561	36	-3.81	0.0005	0.05	-0.9120	-0.2788
BFU-E	E11.5AGM	2.5ug/ml	CFU-GM	AoE+PSCs	7.5ug/ml	0.1343	0.1612	36	0.83	0.4103	0.05	-0.1926	0.4611
BFU-E	E11.5AGM	2.5ug/ml	CFU-GM	E11.5AGM	0ug/ml	-1.8390	0.1527	36	-12.04	<.0001	0.05	-2.1487	-1.5293
BFU-E	E11.5AGM	2.5ug/ml	CFU-GM	E11.5AGM	2.5ug/ml	-1.1153	0.05927	36	-18.82	<.0001	0.05	-1.2355	-0.9951
BFU-E	E11.5AGM	2.5ug/ml	CFU-GM	E11.5AGM	7.5ug/ml	-0.7045	0.1556	36	-4.53	<.0001	0.05	-1.0201	-0.3889
BFU-E	E11.5AGM	2.5ug/ml	CFU-GM	HE+PSCs	0ug/ml	-1.2260	0.1539	36	-7.97	<.0001	0.05	-1.5381	-0.9139
BFU-E	E11.5AGM	2.5ug/ml	CFU-GM	HE+PSCs	2.5ug/ml	-0.4035	0.1571	36	-2.57	0.0145	0.05	-0.7222	-0.08480
BFU-E	E11.5AGM	2.5ug/ml	CFU-GM	HE+PSCs	7.5ug/ml	0.3837	0.1638	36	2.34	0.0248	0.05	0.05145	0.7159

The GLIMMIX Procedure

Colony_Type	Condition	Treatment	_Colony_Type	_Condition	_Treatment	Estimate	Standard Error	DF	t Value	Pr > |t|	Alpha	Lower	Upper
Differences of Colony*Condit*Treatm Least Squares Means													
BFU-E	E11.5AGM	7.5ug/ml	BFU-E	HE+PSCs	0ug/ml	-0.7426	0.1788	36	-4.15	0.0002	0.05	-1.1053	-0.3799
BFU-E	E11.5AGM	7.5ug/ml	BFU-E	HE+PSCs	2.5ug/ml	-0.2657	0.1850	36	-1.44	0.1595	0.05	-0.6408	0.1094
BFU-E	E11.5AGM	7.5ug/ml	BFU-E	HE+PSCs	7.5ug/ml	0.7020	0.2097	36	3.35	0.0019	0.05	0.2767	1.1273
BFU-E	E11.5AGM	7.5ug/ml	CFU-GEMM	AoE+PSCs	0ug/ml	-0.8499	0.1778	36	-4.78	<.0001	0.05	-1.2104	-0.4894
BFU-E	E11.5AGM	7.5ug/ml	CFU-GEMM	AoE+PSCs	2.5ug/ml	-0.1019	0.1879	36	-0.54	0.5911	0.05	-0.4830	0.2793
BFU-E	E11.5AGM	7.5ug/ml	CFU-GEMM	AoE+PSCs	7.5ug/ml	0.8173	0.2141	36	3.82	0.0005	0.05	0.3830	1.2516
BFU-E	E11.5AGM	7.5ug/ml	CFU-GEMM	E11.5AGM	0ug/ml	-1.4963	0.1732	36	-8.64	<.0001	0.05	-1.8475	-1.1451
BFU-E	E11.5AGM	7.5ug/ml	CFU-GEMM	E11.5AGM	2.5ug/ml	-1.1458	0.1753	36	-6.54	<.0001	0.05	-1.5013	-0.7903
BFU-E	E11.5AGM	7.5ug/ml	CFU-GEMM	E11.5AGM	7.5ug/ml	9.127E-6	0.1260	36	0.00	0.9999	0.05	-0.2555	0.2555
BFU-E	E11.5AGM	7.5ug/ml	CFU-GEMM	HE+PSCs	0ug/ml	-0.8226	0.1780	36	-4.62	<.0001	0.05	-1.1837	-0.4616
BFU-E	E11.5AGM	7.5ug/ml	CFU-GEMM	HE+PSCs	2.5ug/ml	0.4037	0.1997	36	2.02	0.0507	0.05	-0.00133	0.8087
BFU-E	E11.5AGM	7.5ug/ml	CFU-GEMM	HE+PSCs	7.5ug/ml	0.9095	0.2181	36	4.17	0.0002	0.05	0.4671	1.3519
BFU-E	E11.5AGM	7.5ug/ml	CFU-GM	AoE+PSCs	0ug/ml	-2.5213	0.1698	36	-14.85	<.0001	0.05	-2.8657	-2.1769
BFU-E	E11.5AGM	7.5ug/ml	CFU-GM	AoE+PSCs	2.5ug/ml	-1.6946	0.1722	36	-9.84	<.0001	0.05	-2.0439	-1.3453
BFU-E	E11.5AGM	7.5ug/ml	CFU-GM	AoE+PSCs	7.5ug/ml	-0.9650	0.1768	36	-5.46	<.0001	0.05	-1.3236	-0.6063
BFU-E	E11.5AGM	7.5ug/ml	CFU-GM	E11.5AGM	0ug/ml	-2.9382	0.1692	36	-17.37	<.0001	0.05	-3.2813	-2.5951
BFU-E	E11.5AGM	7.5ug/ml	CFU-GM	E11.5AGM	2.5ug/ml	-2.2145	0.1705	36	-12.99	<.0001	0.05	-2.5603	-1.8687
BFU-E	E11.5AGM	7.5ug/ml	CFU-GM	E11.5AGM	7.5ug/ml	-1.8037	0.09615	36	-18.76	<.0001	0.05	-1.9987	-1.6087
BFU-E	E11.5AGM	7.5ug/ml	CFU-GM	HE+PSCs	0ug/ml	-2.3252	0.1702	36	-13.66	<.0001	0.05	-2.6705	-1.9800
BFU-E	E11.5AGM	7.5ug/ml	CFU-GM	HE+PSCs	2.5ug/ml	-1.5027	0.1732	36	-8.68	<.0001	0.05	-1.8539	-1.1515
BFU-E	E11.5AGM	7.5ug/ml	CFU-GM	HE+PSCs	7.5ug/ml	-0.7156	0.1792	36	-3.99	0.0003	0.05	-1.0791	-0.3520
BFU-E	HE+PSCs	0ug/ml	BFU-E	HE+PSCs	2.5ug/ml	0.4769	0.1734	36	2.75	0.0092	0.05	0.1253	0.8285
BFU-E	HE+PSCs	0ug/ml	BFU-E	HE+PSCs	7.5ug/ml	1.4446	0.1995	36	7.24	<.0001	0.05	1.0399	1.8493
BFU-E	HE+PSCs	0ug/ml	CFU-GEMM	AoE+PSCs	0ug/ml	-0.1073	0.1657	36	-0.65	0.5212	0.05	-0.4433	0.2286

The GLIMMIX Procedure

Differences of Colony*Condit*Treatm Least Squares Means													
Colony_Type	Condition	Treatment	_Colony_Type	_Condition	_Treatment	Estimate	Standard Error	DF	t Value	Pr > |t|	Alpha	Lower	Upper
BFU-E	HE+PSCs	0ug/ml	CFU-GEMM	AoE+PSCs	2.5ug/ml	0.6407	0.1765	36	3.63	0.0009	0.05	0.2827	0.9987
BFU-E	HE+PSCs	0ug/ml	CFU-GEMM	AoE+PSCs	7.5ug/ml	1.5599	0.2042	36	7.64	<.0001	0.05	1.1457	1.9740
BFU-E	HE+PSCs	0ug/ml	CFU-GEMM	E11.5AGM	0ug/ml	-0.7537	0.1607	36	-4.69	<.0001	0.05	-1.0796	-0.4277
BFU-E	HE+PSCs	0ug/ml	CFU-GEMM	E11.5AGM	2.5ug/ml	-0.4032	0.1630	36	-2.47	0.0182	0.05	-0.7338	-0.07266
BFU-E	HE+PSCs	0ug/ml	CFU-GEMM	E11.5AGM	7.5ug/ml	0.7426	0.1788	36	4.15	0.0002	0.05	0.3799	1.1053
BFU-E	HE+PSCs	0ug/ml	CFU-GEMM	HE+PSCs	0ug/ml	-0.08004	0.08535	36	-0.94	0.3546	0.05	-0.2531	0.09306
BFU-E	HE+PSCs	0ug/ml	CFU-GEMM	HE+PSCs	2.5ug/ml	1.1463	0.1890	36	6.06	<.0001	0.05	0.7629	1.5296
BFU-E	HE+PSCs	0ug/ml	CFU-GEMM	HE+PSCs	7.5ug/ml	1.6521	0.2084	36	7.93	<.0001	0.05	1.2295	2.0747
BFU-E	HE+PSCs	0ug/ml	CFU-GM	AoE+PSCs	0ug/ml	-1.7787	0.1571	36	-11.32	<.0001	0.05	-2.0974	-1.4601
BFU-E	HE+PSCs	0ug/ml	CFU-GM	AoE+PSCs	2.5ug/ml	-0.9520	0.1597	36	-5.96	<.0001	0.05	-1.2760	-0.6281
BFU-E	HE+PSCs	0ug/ml	CFU-GM	AoE+PSCs	7.5ug/ml	-0.2224	0.1647	36	-1.35	0.1853	0.05	-0.5563	0.1116
BFU-E	HE+PSCs	0ug/ml	CFU-GM	E11.5AGM	0ug/ml	-2.1956	0.1564	36	-14.04	<.0001	0.05	-2.5128	-1.8785
BFU-E	HE+PSCs	0ug/ml	CFU-GM	E11.5AGM	2.5ug/ml	-1.4719	0.1578	36	-9.33	<.0001	0.05	-1.7920	-1.1518
BFU-E	HE+PSCs	0ug/ml	CFU-GM	E11.5AGM	7.5ug/ml	-1.0611	0.1592	36	-6.66	<.0001	0.05	-1.3841	-0.7382
BFU-E	HE+PSCs	0ug/ml	CFU-GM	HE+PSCs	0ug/ml	-1.5826	0.06757	36	-23.42	<.0001	0.05	-1.7197	-1.4456
BFU-E	HE+PSCs	0ug/ml	CFU-GM	HE+PSCs	2.5ug/ml	-0.7601	0.1607	36	-4.73	<.0001	0.05	-1.0861	-0.4342
BFU-E	HE+PSCs	0ug/ml	CFU-GM	HE+PSCs	7.5ug/ml	0.02703	0.1673	36	0.16	0.8725	0.05	-0.3122	0.3662
BFU-E	HE+PSCs	2.5ug/ml	BFU-E	HE+PSCs	7.5ug/ml	0.9677	0.2050	36	4.72	<.0001	0.05	0.5519	1.3834
BFU-E	HE+PSCs	2.5ug/ml	CFU-GEMM	AoE+PSCs	0ug/ml	-0.5842	0.1722	36	-3.39	0.0017	0.05	-0.9335	-0.2349
BFU-E	HE+PSCs	2.5ug/ml	CFU-GEMM	AoE+PSCs	2.5ug/ml	0.1638	0.1827	36	0.90	0.3759	0.05	-0.2068	0.5344
BFU-E	HE+PSCs	2.5ug/ml	CFU-GEMM	AoE+PSCs	7.5ug/ml	1.0830	0.2095	36	5.17	<.0001	0.05	0.6580	1.5080
BFU-E	HE+PSCs	2.5ug/ml	CFU-GEMM	E11.5AGM	0ug/ml	-1.2306	0.1675	36	-7.35	<.0001	0.05	-1.5703	-0.8909
BFU-E	HE+PSCs	2.5ug/ml	CFU-GEMM	E11.5AGM	2.5ug/ml	-0.8801	0.1697	36	-5.19	<.0001	0.05	-1.2243	-0.5360
BFU-E	HE+PSCs	2.5ug/ml	CFU-GEMM	E11.5AGM	7.5ug/ml	0.2657	0.1850	36	1.44	0.1595	0.05	-0.1094	0.6408

The GLIMMIX Procedure

Differences of Colony*Condit*Treatm Least Squares Means													
Colony_Type	Condition	Treatment	_Colony_Type	_Condition	_Treatment	Estimate	Standard Error	DF	t Value	Pr > |t|	Alpha	Lower	Upper
BFU-E	HE+PSCs	2.5ug/ml	CFU-GEMM	HE+PSCs	0ug/ml	-0.5569	0.1725	36	-3.23	0.0027	0.05	-0.9068	-0.2070
BFU-E	HE+PSCs	2.5ug/ml	CFU-GEMM	HE+PSCs	2.5ug/ml	0.6694	0.1326	36	5.05	<.0001	0.05	0.4005	0.9382
BFU-E	HE+PSCs	2.5ug/ml	CFU-GEMM	HE+PSCs	7.5ug/ml	1.1752	0.2136	36	5.50	<.0001	0.05	0.7420	1.6084
BFU-E	HE+PSCs	2.5ug/ml	CFU-GM	AoE+PSCs	0ug/ml	-2.2556	0.1640	36	-13.75	<.0001	0.05	-2.5883	-1.9230
BFU-E	HE+PSCs	2.5ug/ml	CFU-GM	AoE+PSCs	2.5ug/ml	-1.4289	0.1665	36	-8.58	<.0001	0.05	-1.7667	-1.0912
BFU-E	HE+PSCs	2.5ug/ml	CFU-GM	AoE+PSCs	7.5ug/ml	-0.6993	0.1712	36	-4.08	0.0002	0.05	-1.0466	-0.3520
BFU-E	HE+PSCs	2.5ug/ml	CFU-GM	E11.5AGM	0ug/ml	-2.6725	0.1634	36	-16.36	<.0001	0.05	-3.0038	-2.3412
BFU-E	HE+PSCs	2.5ug/ml	CFU-GM	E11.5AGM	2.5ug/ml	-1.9488	0.1647	36	-11.83	<.0001	0.05	-2.2829	-1.6147
BFU-E	HE+PSCs	2.5ug/ml	CFU-GM	E11.5AGM	7.5ug/ml	-1.5380	0.1661	36	-9.26	<.0001	0.05	-1.8748	-1.2012
BFU-E	HE+PSCs	2.5ug/ml	CFU-GM	HE+PSCs	0ug/ml	-2.0595	0.1645	36	-12.52	<.0001	0.05	-2.3930	-1.7260
BFU-E	HE+PSCs	2.5ug/ml	CFU-GM	HE+PSCs	2.5ug/ml	-1.2370	0.08763	36	-14.12	<.0001	0.05	-1.4147	-1.0593
BFU-E	HE+PSCs	2.5ug/ml	CFU-GM	HE+PSCs	7.5ug/ml	-0.4499	0.1737	36	-2.59	0.0138	0.05	-0.8022	-0.09752
BFU-E	HE+PSCs	7.5ug/ml	CFU-GEMM	AoE+PSCs	0ug/ml	-1.5519	0.1986	36	-7.82	<.0001	0.05	-1.9546	-1.1492
BFU-E	HE+PSCs	7.5ug/ml	CFU-GEMM	AoE+PSCs	2.5ug/ml	-0.8039	0.2077	36	-3.87	0.0004	0.05	-1.2251	-0.3826
BFU-E	HE+PSCs	7.5ug/ml	CFU-GEMM	AoE+PSCs	7.5ug/ml	0.1153	0.2316	36	0.50	0.6217	0.05	-0.3545	0.5851
BFU-E	HE+PSCs	7.5ug/ml	CFU-GEMM	E11.5AGM	0ug/ml	-2.1983	0.1945	36	-11.30	<.0001	0.05	-2.5926	-1.8039
BFU-E	HE+PSCs	7.5ug/ml	CFU-GEMM	E11.5AGM	2.5ug/ml	-1.8478	0.1964	36	-9.41	<.0001	0.05	-2.2460	-1.4496
BFU-E	HE+PSCs	7.5ug/ml	CFU-GEMM	E11.5AGM	7.5ug/ml	-0.7020	0.2097	36	-3.35	0.0019	0.05	-1.1273	-0.2767
BFU-E	HE+PSCs	7.5ug/ml	CFU-GEMM	HE+PSCs	0ug/ml	-1.5246	0.1988	36	-7.67	<.0001	0.05	-1.9278	-1.1214
BFU-E	HE+PSCs	7.5ug/ml	CFU-GEMM	HE+PSCs	2.5ug/ml	-0.2983	0.2184	36	-1.37	0.1804	0.05	-0.7412	0.1446
BFU-E	HE+PSCs	7.5ug/ml	CFU-GEMM	HE+PSCs	7.5ug/ml	0.2075	0.1867	36	1.11	0.2738	0.05	-0.1712	0.5862
BFU-E	HE+PSCs	7.5ug/ml	CFU-GM	AoE+PSCs	0ug/ml	-3.2233	0.1915	36	-16.83	<.0001	0.05	-3.6117	-2.8350
BFU-E	HE+PSCs	7.5ug/ml	CFU-GM	AoE+PSCs	2.5ug/ml	-2.3966	0.1936	36	-12.38	<.0001	0.05	-2.7893	-2.0039
BFU-E	HE+PSCs	7.5ug/ml	CFU-GM	AoE+PSCs	7.5ug/ml	-1.6669	0.1977	36	-8.43	<.0001	0.05	-2.0678	-1.2661

The GLIMMIX Procedure

Differences of Colony*Condit*Treatm Least Squares Means													
Colony_Type	Condition	Treatment	_Colony_Type	_Condition	_Treatment	Estimate	Standard Error	DF	t Value	Pr > |t|	Alpha	Lower	Upper
BFU-E	HE+PSCs	7.5ug/ml	CFU-GM	E11.5AGM	0ug/ml	-3.6402	0.1909	36	-19.07	<.0001	0.05	-4.0274	-3.2530
BFU-E	HE+PSCs	7.5ug/ml	CFU-GM	E11.5AGM	2.5ug/ml	-2.9165	0.1921	36	-15.18	<.0001	0.05	-3.3061	-2.5269
BFU-E	HE+PSCs	7.5ug/ml	CFU-GM	E11.5AGM	7.5ug/ml	-2.5057	0.1932	36	-12.97	<.0001	0.05	-2.8976	-2.1138
BFU-E	HE+PSCs	7.5ug/ml	CFU-GM	HE+PSCs	0ug/ml	-3.0272	0.1918	36	-15.78	<.0001	0.05	-3.4163	-2.6381
BFU-E	HE+PSCs	7.5ug/ml	CFU-GM	HE+PSCs	2.5ug/ml	-2.2047	0.1944	36	-11.34	<.0001	0.05	-2.5990	-1.8104
BFU-E	HE+PSCs	7.5ug/ml	CFU-GM	HE+PSCs	7.5ug/ml	-1.4175	0.1393	36	-10.17	<.0001	0.05	-1.7001	-1.1350
CFU-GEMM	AoE+PSCs	0ug/ml	CFU-GEMM	AoE+PSCs	2.5ug/ml	0.7480	0.1754	36	4.26	0.0001	0.05	0.3922	1.1038
CFU-GEMM	AoE+PSCs	0ug/ml	CFU-GEMM	AoE+PSCs	7.5ug/ml	1.6672	0.2033	36	8.20	<.0001	0.05	1.2550	2.0794
CFU-GEMM	AoE+PSCs	0ug/ml	CFU-GEMM	E11.5AGM	0ug/ml	-0.6464	0.1595	36	-4.05	0.0003	0.05	-0.9698	-0.3229
CFU-GEMM	AoE+PSCs	0ug/ml	CFU-GEMM	E11.5AGM	2.5ug/ml	-0.2959	0.1618	36	-1.83	0.0757	0.05	-0.6241	0.03225
CFU-GEMM	AoE+PSCs	0ug/ml	CFU-GEMM	E11.5AGM	7.5ug/ml	0.8499	0.1778	36	4.78	<.0001	0.05	0.4894	1.2104
CFU-GEMM	AoE+PSCs	0ug/ml	CFU-GEMM	HE+PSCs	0ug/ml	0.02729	0.1648	36	0.17	0.8694	0.05	-0.3069	0.3615
CFU-GEMM	AoE+PSCs	0ug/ml	CFU-GEMM	HE+PSCs	2.5ug/ml	1.2536	0.1880	36	6.67	<.0001	0.05	0.8723	1.6349
CFU-GEMM	AoE+PSCs	0ug/ml	CFU-GEMM	HE+PSCs	7.5ug/ml	1.7594	0.2074	36	8.48	<.0001	0.05	1.3387	2.1801
CFU-GEMM	AoE+PSCs	0ug/ml	CFU-GM	AoE+PSCs	0ug/ml	-1.6714	0.06357	36	-26.29	<.0001	0.05	-1.8003	-1.5425
CFU-GEMM	AoE+PSCs	0ug/ml	CFU-GM	AoE+PSCs	2.5ug/ml	-0.8447	0.1585	36	-5.33	<.0001	0.05	-1.1662	-0.5232
CFU-GEMM	AoE+PSCs	0ug/ml	CFU-GM	AoE+PSCs	7.5ug/ml	-0.1150	0.1635	36	-0.70	0.4862	0.05	-0.4466	0.2165
CFU-GEMM	AoE+PSCs	0ug/ml	CFU-GM	E11.5AGM	0ug/ml	-2.0883	0.1552	36	-13.46	<.0001	0.05	-2.4030	-1.7736
CFU-GEMM	AoE+PSCs	0ug/ml	CFU-GM	E11.5AGM	2.5ug/ml	-1.3646	0.1566	36	-8.71	<.0001	0.05	-1.6822	-1.0470
CFU-GEMM	AoE+PSCs	0ug/ml	CFU-GM	E11.5AGM	7.5ug/ml	-0.9538	0.1580	36	-6.04	<.0001	0.05	-1.2743	-0.6333
CFU-GEMM	AoE+PSCs	0ug/ml	CFU-GM	HE+PSCs	0ug/ml	-1.4753	0.1563	36	-9.44	<.0001	0.05	-1.7923	-1.1583
CFU-GEMM	AoE+PSCs	0ug/ml	CFU-GM	HE+PSCs	2.5ug/ml	-0.6528	0.1595	36	-4.09	0.0002	0.05	-0.9763	-0.3293
CFU-GEMM	AoE+PSCs	0ug/ml	CFU-GM	HE+PSCs	7.5ug/ml	0.1344	0.1661	36	0.81	0.4239	0.05	-0.2025	0.4712
CFU-GEMM	AoE+PSCs	2.5ug/ml	CFU-GEMM	AoE+PSCs	7.5ug/ml	0.9192	0.2122	36	4.33	0.0001	0.05	0.4888	1.3495

The GLIMMIX Procedure

Differences of Colony*Condit*Treatm Least Squares Means													
Colony_Type	Condition	Treatment	_Colony_Type	_Condition	_Treatment	Estimate	Standard Error	DF	t Value	Pr > |t|	Alpha	Lower	Upper
CFU-GEMM	AoE+PSCs	2.5ug/ml	CFU-GEMM	E11.5AGM	0ug/ml	-1.3944	0.1708	36	-8.17	<.0001	0.05	-1.7407	-1.0480
CFU-GEMM	AoE+PSCs	2.5ug/ml	CFU-GEMM	E11.5AGM	2.5ug/ml	-1.0439	0.1729	36	-6.04	<.0001	0.05	-1.3947	-0.6932
CFU-GEMM	AoE+PSCs	2.5ug/ml	CFU-GEMM	E11.5AGM	7.5ug/ml	0.1019	0.1879	36	0.54	0.5910	0.05	-0.2793	0.4831
CFU-GEMM	AoE+PSCs	2.5ug/ml	CFU-GEMM	HE+PSCs	0ug/ml	-0.7207	0.1757	36	-4.10	0.0002	0.05	-1.0771	-0.3644
CFU-GEMM	AoE+PSCs	2.5ug/ml	CFU-GEMM	HE+PSCs	2.5ug/ml	0.5056	0.1976	36	2.56	0.0149	0.05	0.1048	0.9064
CFU-GEMM	AoE+PSCs	2.5ug/ml	CFU-GEMM	HE+PSCs	7.5ug/ml	1.0114	0.2162	36	4.68	<.0001	0.05	0.5729	1.4499
CFU-GEMM	AoE+PSCs	2.5ug/ml	CFU-GM	AoE+PSCs	0ug/ml	-2.4194	0.1674	36	-14.45	<.0001	0.05	-2.7589	-2.0800
CFU-GEMM	AoE+PSCs	2.5ug/ml	CFU-GM	AoE+PSCs	2.5ug/ml	-1.5927	0.09240	36	-17.24	<.0001	0.05	-1.7801	-1.4053
CFU-GEMM	AoE+PSCs	2.5ug/ml	CFU-GM	AoE+PSCs	7.5ug/ml	-0.8631	0.1745	36	-4.95	<.0001	0.05	-1.2169	-0.5092
CFU-GEMM	AoE+PSCs	2.5ug/ml	CFU-GM	E11.5AGM	0ug/ml	-2.8363	0.1667	36	-17.01	<.0001	0.05	-3.1745	-2.4982
CFU-GEMM	AoE+PSCs	2.5ug/ml	CFU-GM	E11.5AGM	2.5ug/ml	-2.1126	0.1681	36	-12.57	<.0001	0.05	-2.4535	-1.7718
CFU-GEMM	AoE+PSCs	2.5ug/ml	CFU-GM	E11.5AGM	7.5ug/ml	-1.7018	0.1694	36	-10.05	<.0001	0.05	-2.0453	-1.3583
CFU-GEMM	AoE+PSCs	2.5ug/ml	CFU-GM	HE+PSCs	0ug/ml	-2.2233	0.1678	36	-13.25	<.0001	0.05	-2.5636	-1.8830
CFU-GEMM	AoE+PSCs	2.5ug/ml	CFU-GM	HE+PSCs	2.5ug/ml	-1.4008	0.1708	36	-8.20	<.0001	0.05	-1.7471	-1.0545
CFU-GEMM	AoE+PSCs	2.5ug/ml	CFU-GM	HE+PSCs	7.5ug/ml	-0.6137	0.1769	36	-3.47	0.0014	0.05	-0.9725	-0.2549
CFU-GEMM	AoE+PSCs	7.5ug/ml	CFU-GEMM	E11.5AGM	0ug/ml	-2.3135	0.1992	36	-11.61	<.0001	0.05	-2.7176	-1.9094
CFU-GEMM	AoE+PSCs	7.5ug/ml	CFU-GEMM	E11.5AGM	2.5ug/ml	-1.9631	0.2011	36	-9.76	<.0001	0.05	-2.3709	-1.5553
CFU-GEMM	AoE+PSCs	7.5ug/ml	CFU-GEMM	E11.5AGM	7.5ug/ml	-0.8173	0.2141	36	-3.82	0.0005	0.05	-1.2516	-0.3830
CFU-GEMM	AoE+PSCs	7.5ug/ml	CFU-GEMM	HE+PSCs	0ug/ml	-1.6399	0.2035	36	-8.06	<.0001	0.05	-2.0526	-1.2272
CFU-GEMM	AoE+PSCs	7.5ug/ml	CFU-GEMM	HE+PSCs	2.5ug/ml	-0.4136	0.2227	36	-1.86	0.0715	0.05	-0.8652	0.03802
CFU-GEMM	AoE+PSCs	7.5ug/ml	CFU-GEMM	HE+PSCs	7.5ug/ml	0.09223	0.2393	36	0.39	0.7022	0.05	-0.3931	0.5775
CFU-GEMM	AoE+PSCs	7.5ug/ml	CFU-GM	AoE+PSCs	0ug/ml	-3.3386	0.1963	36	-17.00	<.0001	0.05	-3.7368	-2.9404
CFU-GEMM	AoE+PSCs	7.5ug/ml	CFU-GM	AoE+PSCs	2.5ug/ml	-2.5119	0.1984	36	-12.66	<.0001	0.05	-2.9143	-2.1094
CFU-GEMM	AoE+PSCs	7.5ug/ml	CFU-GM	AoE+PSCs	7.5ug/ml	-1.7822	0.1431	36	-12.45	<.0001	0.05	-2.0725	-1.4919

The GLIMMIX Procedure

Differences of Colony*Condit*Treatm Least Squares Means													
Colony_Type	Condition	Treatment	_Colony_Type	_Condition	_Treatment	Estimate	Standard Error	DF	t Value	Pr > |t|	Alpha	Lower	Upper
CFU-GEMM	AoE+PSCs	7.5ug/ml	CFU-GM	E11.5AGM	0ug/ml	-3.7555	0.1958	36	-19.18	<.0001	0.05	-4.1526	-3.3584
CFU-GEMM	AoE+PSCs	7.5ug/ml	CFU-GM	E11.5AGM	2.5ug/ml	-3.0318	0.1969	36	-15.40	<.0001	0.05	-3.4312	-2.6324
CFU-GEMM	AoE+PSCs	7.5ug/ml	CFU-GM	E11.5AGM	7.5ug/ml	-2.6210	0.1981	36	-13.23	<.0001	0.05	-3.0226	-2.2193
CFU-GEMM	AoE+PSCs	7.5ug/ml	CFU-GM	HE+PSCs	0ug/ml	-3.1425	0.1967	36	-15.98	<.0001	0.05	-3.5414	-2.7436
CFU-GEMM	AoE+PSCs	7.5ug/ml	CFU-GM	HE+PSCs	2.5ug/ml	-2.3200	0.1992	36	-11.65	<.0001	0.05	-2.7240	-1.9159
CFU-GEMM	AoE+PSCs	7.5ug/ml	CFU-GM	HE+PSCs	7.5ug/ml	-1.5328	0.2045	36	-7.50	<.0001	0.05	-1.9475	-1.1181
CFU-GEMM	E11.5AGM	0ug/ml	CFU-GEMM	E11.5AGM	2.5ug/ml	0.3504	0.1567	36	2.24	0.0317	0.05	0.03256	0.6683
CFU-GEMM	E11.5AGM	0ug/ml	CFU-GEMM	E11.5AGM	7.5ug/ml	1.4963	0.1732	36	8.64	<.0001	0.05	1.1451	1.8475
CFU-GEMM	E11.5AGM	0ug/ml	CFU-GEMM	HE+PSCs	0ug/ml	0.6736	0.1598	36	4.22	0.0002	0.05	0.3496	0.9977
CFU-GEMM	E11.5AGM	0ug/ml	CFU-GEMM	HE+PSCs	2.5ug/ml	1.9000	0.1836	36	10.35	<.0001	0.05	1.5275	2.2724
CFU-GEMM	E11.5AGM	0ug/ml	CFU-GEMM	HE+PSCs	7.5ug/ml	2.4058	0.2035	36	11.82	<.0001	0.05	1.9930	2.8185
CFU-GEMM	E11.5AGM	0ug/ml	CFU-GM	AoE+PSCs	0ug/ml	-1.0251	0.1506	36	-6.81	<.0001	0.05	-1.3305	-0.7196
CFU-GEMM	E11.5AGM	0ug/ml	CFU-GM	AoE+PSCs	2.5ug/ml	-0.1983	0.1533	36	-1.29	0.2041	0.05	-0.5093	0.1126
CFU-GEMM	E11.5AGM	0ug/ml	CFU-GM	AoE+PSCs	7.5ug/ml	0.5313	0.1585	36	3.35	0.0019	0.05	0.2099	0.8527
CFU-GEMM	E11.5AGM	0ug/ml	CFU-GM	E11.5AGM	0ug/ml	-1.4420	0.04703	36	-30.66	<.0001	0.05	-1.5373	-1.3466
CFU-GEMM	E11.5AGM	0ug/ml	CFU-GM	E11.5AGM	2.5ug/ml	-0.7182	0.1514	36	-4.75	<.0001	0.05	-1.0252	-0.4113
CFU-GEMM	E11.5AGM	0ug/ml	CFU-GM	E11.5AGM	7.5ug/ml	-0.3074	0.1528	36	-2.01	0.0518	0.05	-0.6174	0.002495
CFU-GEMM	E11.5AGM	0ug/ml	CFU-GM	HE+PSCs	0ug/ml	-0.8289	0.1511	36	-5.49	<.0001	0.05	-1.1353	-0.5226
CFU-GEMM	E11.5AGM	0ug/ml	CFU-GM	HE+PSCs	2.5ug/ml	-0.00643	0.1544	36	-0.04	0.9670	0.05	-0.3195	0.3066
CFU-GEMM	E11.5AGM	0ug/ml	CFU-GM	HE+PSCs	7.5ug/ml	0.7807	0.1612	36	4.84	<.0001	0.05	0.4539	1.1076
CFU-GEMM	E11.5AGM	2.5ug/ml	CFU-GEMM	E11.5AGM	7.5ug/ml	1.1458	0.1753	36	6.54	<.0001	0.05	0.7903	1.5013
CFU-GEMM	E11.5AGM	2.5ug/ml	CFU-GEMM	HE+PSCs	0ug/ml	0.3232	0.1621	36	1.99	0.0538	0.05	-0.00556	0.6520
CFU-GEMM	E11.5AGM	2.5ug/ml	CFU-GEMM	HE+PSCs	2.5ug/ml	1.5495	0.1856	36	8.35	<.0001	0.05	1.1730	1.9260
CFU-GEMM	E11.5AGM	2.5ug/ml	CFU-GEMM	HE+PSCs	7.5ug/ml	2.0553	0.2053	36	10.01	<.0001	0.05	1.6389	2.4717

The GLIMMIX Procedure

Differences of Colony*Condit*Treatm Least Squares Means													
Colony_Type	Condition	Treatment	_Colony_Type	_Condition	_Treatment	Estimate	Standard Error	DF	t Value	Pr > |t|	Alpha	Lower	Upper
CFU-GEMM	E11.5AGM	2.5ug/ml	CFU-GM	AoE+PSCs	0ug/ml	-1.3755	0.1530	36	-8.99	<.0001	0.05	-1.6859	-1.0651
CFU-GEMM	E11.5AGM	2.5ug/ml	CFU-GM	AoE+PSCs	2.5ug/ml	-0.5488	0.1557	36	-3.52	0.0012	0.05	-0.8646	-0.2329
CFU-GEMM	E11.5AGM	2.5ug/ml	CFU-GM	AoE+PSCs	7.5ug/ml	0.1809	0.1608	36	1.12	0.2681	0.05	-0.1452	0.5070
CFU-GEMM	E11.5AGM	2.5ug/ml	CFU-GM	E11.5AGM	0ug/ml	-1.7924	0.1523	36	-11.77	<.0001	0.05	-2.1013	-1.4835
CFU-GEMM	E11.5AGM	2.5ug/ml	CFU-GM	E11.5AGM	2.5ug/ml	-1.0687	0.05824	36	-18.35	<.0001	0.05	-1.1868	-0.9505
CFU-GEMM	E11.5AGM	2.5ug/ml	CFU-GM	E11.5AGM	7.5ug/ml	-0.6579	0.1552	36	-4.24	0.0001	0.05	-0.9727	-0.3431
CFU-GEMM	E11.5AGM	2.5ug/ml	CFU-GM	HE+PSCs	0ug/ml	-1.1794	0.1535	36	-7.68	<.0001	0.05	-1.4907	-0.8681
CFU-GEMM	E11.5AGM	2.5ug/ml	CFU-GM	HE+PSCs	2.5ug/ml	-0.3569	0.1567	36	-2.28	0.0289	0.05	-0.6748	-0.03897
CFU-GEMM	E11.5AGM	2.5ug/ml	CFU-GM	HE+PSCs	7.5ug/ml	0.4303	0.1634	36	2.63	0.0124	0.05	0.09881	0.7617
CFU-GEMM	E11.5AGM	7.5ug/ml	CFU-GEMM	HE+PSCs	0ug/ml	-0.8226	0.1780	36	-4.62	<.0001	0.05	-1.1837	-0.4616
CFU-GEMM	E11.5AGM	7.5ug/ml	CFU-GEMM	HE+PSCs	2.5ug/ml	0.4037	0.1997	36	2.02	0.0507	0.05	-0.00134	0.8087
CFU-GEMM	E11.5AGM	7.5ug/ml	CFU-GEMM	HE+PSCs	7.5ug/ml	0.9095	0.2181	36	4.17	0.0002	0.05	0.4671	1.3518
CFU-GEMM	E11.5AGM	7.5ug/ml	CFU-GM	AoE+PSCs	0ug/ml	-2.5213	0.1698	36	-14.85	<.0001	0.05	-2.8657	-2.1769
CFU-GEMM	E11.5AGM	7.5ug/ml	CFU-GM	AoE+PSCs	2.5ug/ml	-1.6946	0.1722	36	-9.84	<.0001	0.05	-2.0439	-1.3453
CFU-GEMM	E11.5AGM	7.5ug/ml	CFU-GM	AoE+PSCs	7.5ug/ml	-0.9650	0.1768	36	-5.46	<.0001	0.05	-1.3236	-0.6063
CFU-GEMM	E11.5AGM	7.5ug/ml	CFU-GM	E11.5AGM	0ug/ml	-2.9382	0.1692	36	-17.37	<.0001	0.05	-3.2813	-2.5952
CFU-GEMM	E11.5AGM	7.5ug/ml	CFU-GM	E11.5AGM	2.5ug/ml	-2.2145	0.1705	36	-12.99	<.0001	0.05	-2.5603	-1.8687
CFU-GEMM	E11.5AGM	7.5ug/ml	CFU-GM	E11.5AGM	7.5ug/ml	-1.8037	0.09615	36	-18.76	<.0001	0.05	-1.9987	-1.6087
CFU-GEMM	E11.5AGM	7.5ug/ml	CFU-GM	HE+PSCs	0ug/ml	-2.3252	0.1702	36	-13.66	<.0001	0.05	-2.6705	-1.9800
CFU-GEMM	E11.5AGM	7.5ug/ml	CFU-GM	HE+PSCs	2.5ug/ml	-1.5027	0.1732	36	-8.68	<.0001	0.05	-1.8539	-1.1515
CFU-GEMM	E11.5AGM	7.5ug/ml	CFU-GM	HE+PSCs	7.5ug/ml	-0.7156	0.1792	36	-3.99	0.0003	0.05	-1.0791	-0.3520
CFU-GEMM	HE+PSCs	0ug/ml	CFU-GEMM	HE+PSCs	2.5ug/ml	1.2263	0.1882	36	6.51	<.0001	0.05	0.8445	1.6081
CFU-GEMM	HE+PSCs	0ug/ml	CFU-GEMM	HE+PSCs	7.5ug/ml	1.7321	0.2077	36	8.34	<.0001	0.05	1.3109	2.1533
CFU-GEMM	HE+PSCs	0ug/ml	CFU-GM	AoE+PSCs	0ug/ml	-1.6987	0.1562	36	-10.88	<.0001	0.05	-2.0154	-1.3820

The GLIMMIX Procedure

Differences of Colony*Condit*Treatm Least Squares Means													
Colony_Type	Condition	Treatment	_Colony_Type	_Condition	_Treatment	Estimate	Standard Error	DF	t Value	Pr > |t|	Alpha	Lower	Upper
CFU-GEMM	HE+PSCs	0ug/ml	CFU-GM	AoE+PSCs	2.5ug/ml	-0.8720	0.1588	36	-5.49	<.0001	0.05	-1.1941	-0.5499
CFU-GEMM	HE+PSCs	0ug/ml	CFU-GM	AoE+PSCs	7.5ug/ml	-0.1423	0.1638	36	-0.87	0.3906	0.05	-0.4745	0.1898
CFU-GEMM	HE+PSCs	0ug/ml	CFU-GM	E11.5AGM	0ug/ml	-2.1156	0.1555	36	-13.61	<.0001	0.05	-2.4309	-1.8003
CFU-GEMM	HE+PSCs	0ug/ml	CFU-GM	E11.5AGM	2.5ug/ml	-1.3919	0.1569	36	-8.87	<.0001	0.05	-1.7101	-1.0737
CFU-GEMM	HE+PSCs	0ug/ml	CFU-GM	E11.5AGM	7.5ug/ml	-0.9811	0.1583	36	-6.20	<.0001	0.05	-1.3022	-0.6600
CFU-GEMM	HE+PSCs	0ug/ml	CFU-GM	HE+PSCs	0ug/ml	-1.5026	0.06538	36	-22.98	<.0001	0.05	-1.6352	-1.3700
CFU-GEMM	HE+PSCs	0ug/ml	CFU-GM	HE+PSCs	2.5ug/ml	-0.6801	0.1598	36	-4.26	0.0001	0.05	-1.0042	-0.3560
CFU-GEMM	HE+PSCs	0ug/ml	CFU-GM	HE+PSCs	7.5ug/ml	0.1071	0.1664	36	0.64	0.5240	0.05	-0.2304	0.4445
CFU-GEMM	HE+PSCs	2.5ug/ml	CFU-GEMM	HE+PSCs	7.5ug/ml	0.5058	0.2265	36	2.23	0.0318	0.05	0.04646	0.9652
CFU-GEMM	HE+PSCs	2.5ug/ml	CFU-GM	AoE+PSCs	0ug/ml	-2.9250	0.1805	36	-16.21	<.0001	0.05	-3.2911	-2.5590
CFU-GEMM	HE+PSCs	2.5ug/ml	CFU-GM	AoE+PSCs	2.5ug/ml	-2.0983	0.1828	36	-11.48	<.0001	0.05	-2.4690	-1.7276
CFU-GEMM	HE+PSCs	2.5ug/ml	CFU-GM	AoE+PSCs	7.5ug/ml	-1.3686	0.1871	36	-7.32	<.0001	0.05	-1.7481	-0.9892
CFU-GEMM	HE+PSCs	2.5ug/ml	CFU-GM	E11.5AGM	0ug/ml	-3.3419	0.1799	36	-18.58	<.0001	0.05	-3.7067	-2.9771
CFU-GEMM	HE+PSCs	2.5ug/ml	CFU-GM	E11.5AGM	2.5ug/ml	-2.6182	0.1811	36	-14.45	<.0001	0.05	-2.9855	-2.2508
CFU-GEMM	HE+PSCs	2.5ug/ml	CFU-GM	E11.5AGM	7.5ug/ml	-2.2074	0.1824	36	-12.11	<.0001	0.05	-2.5772	-1.8376
CFU-GEMM	HE+PSCs	2.5ug/ml	CFU-GM	HE+PSCs	0ug/ml	-2.7289	0.1809	36	-15.09	<.0001	0.05	-3.0957	-2.3621
CFU-GEMM	HE+PSCs	2.5ug/ml	CFU-GM	HE+PSCs	2.5ug/ml	-1.9064	0.1155	36	-16.50	<.0001	0.05	-2.1407	-1.6720
CFU-GEMM	HE+PSCs	2.5ug/ml	CFU-GM	HE+PSCs	7.5ug/ml	-1.1192	0.1894	36	-5.91	<.0001	0.05	-1.5033	-0.7352
CFU-GEMM	HE+PSCs	7.5ug/ml	CFU-GM	AoE+PSCs	0ug/ml	-3.4308	0.2007	36	-17.10	<.0001	0.05	-3.8378	-3.0238
CFU-GEMM	HE+PSCs	7.5ug/ml	CFU-GM	AoE+PSCs	2.5ug/ml	-2.6041	0.2027	36	-12.85	<.0001	0.05	-3.0152	-2.1930
CFU-GEMM	HE+PSCs	7.5ug/ml	CFU-GM	AoE+PSCs	7.5ug/ml	-1.8745	0.2066	36	-9.07	<.0001	0.05	-2.2934	-1.4555
CFU-GEMM	HE+PSCs	7.5ug/ml	CFU-GM	E11.5AGM	0ug/ml	-3.8477	0.2001	36	-19.23	<.0001	0.05	-4.2536	-3.4419
CFU-GEMM	HE+PSCs	7.5ug/ml	CFU-GM	E11.5AGM	2.5ug/ml	-3.1240	0.2012	36	-15.52	<.0001	0.05	-3.5321	-2.7159
CFU-GEMM	HE+PSCs	7.5ug/ml	CFU-GM	E11.5AGM	7.5ug/ml	-2.7132	0.2023	36	-13.41	<.0001	0.05	-3.1236	-2.3028

The GLIMMIX Procedure

Differences of Colony*Condit*Treatm Least Squares Means													
Colony_Type	Condition	Treatment	_Colony_Type	_Condition	_Treatment	Estimate	Standard Error	DF	t Value	Pr > |t|	Alpha	Lower	Upper
CFU-GEMM	HE+PSCs	7.5ug/ml	CFU-GM	HE+PSCs	0ug/ml	-3.2347	0.2010	36	-16.09	<.0001	0.05	-3.6424	-2.8270
CFU-GEMM	HE+PSCs	7.5ug/ml	CFU-GM	HE+PSCs	2.5ug/ml	-2.4122	0.2035	36	-11.85	<.0001	0.05	-2.8249	-1.9995
CFU-GEMM	HE+PSCs	7.5ug/ml	CFU-GM	HE+PSCs	7.5ug/ml	-1.6251	0.1517	36	-10.71	<.0001	0.05	-1.9328	-1.3174
CFU-GM	AoE+PSCs	0ug/ml	CFU-GM	AoE+PSCs	2.5ug/ml	0.8267	0.1495	36	5.53	<.0001	0.05	0.5234	1.1300
CFU-GM	AoE+PSCs	0ug/ml	CFU-GM	AoE+PSCs	7.5ug/ml	1.5564	0.1548	36	10.05	<.0001	0.05	1.2424	1.8703
CFU-GM	AoE+PSCs	0ug/ml	CFU-GM	E11.5AGM	0ug/ml	-0.4169	0.1460	36	-2.86	0.0071	0.05	-0.7130	-0.1208
CFU-GM	AoE+PSCs	0ug/ml	CFU-GM	E11.5AGM	2.5ug/ml	0.3068	0.1475	36	2.08	0.0447	0.05	0.007626	0.6060
CFU-GM	AoE+PSCs	0ug/ml	CFU-GM	E11.5AGM	7.5ug/ml	0.7176	0.1490	36	4.82	<.0001	0.05	0.4154	1.0198
CFU-GM	AoE+PSCs	0ug/ml	CFU-GM	HE+PSCs	0ug/ml	0.1961	0.1472	36	1.33	0.1912	0.05	-0.1025	0.4947
CFU-GM	AoE+PSCs	0ug/ml	CFU-GM	HE+PSCs	2.5ug/ml	1.0186	0.1506	36	6.76	<.0001	0.05	0.7132	1.3241
CFU-GM	AoE+PSCs	0ug/ml	CFU-GM	HE+PSCs	7.5ug/ml	1.8058	0.1576	36	11.46	<.0001	0.05	1.4862	2.1253
CFU-GM	AoE+PSCs	2.5ug/ml	CFU-GM	AoE+PSCs	7.5ug/ml	0.7296	0.1575	36	4.63	<.0001	0.05	0.4103	1.0490
CFU-GM	AoE+PSCs	2.5ug/ml	CFU-GM	E11.5AGM	0ug/ml	-1.2436	0.1488	36	-8.36	<.0001	0.05	-1.5454	-0.9418
CFU-GM	AoE+PSCs	2.5ug/ml	CFU-GM	E11.5AGM	2.5ug/ml	-0.5199	0.1503	36	-3.46	0.0014	0.05	-0.8248	-0.2151
CFU-GM	AoE+PSCs	2.5ug/ml	CFU-GM	E11.5AGM	7.5ug/ml	-0.1091	0.1518	36	-0.72	0.4769	0.05	-0.4169	0.1987
CFU-GM	AoE+PSCs	2.5ug/ml	CFU-GM	HE+PSCs	0ug/ml	-0.6306	0.1500	36	-4.20	0.0002	0.05	-0.9348	-0.3264
CFU-GM	AoE+PSCs	2.5ug/ml	CFU-GM	HE+PSCs	2.5ug/ml	0.1919	0.1533	36	1.25	0.2188	0.05	-0.1191	0.5029
CFU-GM	AoE+PSCs	2.5ug/ml	CFU-GM	HE+PSCs	7.5ug/ml	0.9790	0.1602	36	6.11	<.0001	0.05	0.6542	1.3038
CFU-GM	AoE+PSCs	7.5ug/ml	CFU-GM	E11.5AGM	0ug/ml	-1.9733	0.1541	36	-12.80	<.0001	0.05	-2.2858	-1.6607
CFU-GM	AoE+PSCs	7.5ug/ml	CFU-GM	E11.5AGM	2.5ug/ml	-1.2495	0.1556	36	-8.03	<.0001	0.05	-1.5650	-0.9341
CFU-GM	AoE+PSCs	7.5ug/ml	CFU-GM	E11.5AGM	7.5ug/ml	-0.8388	0.1570	36	-5.34	<.0001	0.05	-1.1571	-0.5204
CFU-GM	AoE+PSCs	7.5ug/ml	CFU-GM	HE+PSCs	0ug/ml	-1.3603	0.1553	36	-8.76	<.0001	0.05	-1.6751	-1.0454
CFU-GM	AoE+PSCs	7.5ug/ml	CFU-GM	HE+PSCs	2.5ug/ml	-0.5377	0.1584	36	-3.39	0.0017	0.05	-0.8591	-0.2164
CFU-GM	AoE+PSCs	7.5ug/ml	CFU-GM	HE+PSCs	7.5ug/ml	0.2494	0.1650	36	1.51	0.1394	0.05	-0.08525	0.5841

The GLIMMIX Procedure

Differences of Colony*Condit*Treatm Least Squares Means													
Colony_Type	Condition	Treatment	_Colony_Type	_Condition	_Treatment	Estimate	Standard Error	DF	t Value	Pr > |t|	Alpha	Lower	Upper
CFU-GM	E11.5AGM	0ug/ml	CFU-GM	E11.5AGM	2.5ug/ml	0.7237	0.1468	36	4.93	<.0001	0.05	0.4261	1.0214
CFU-GM	E11.5AGM	0ug/ml	CFU-GM	E11.5AGM	7.5ug/ml	1.1345	0.1483	36	7.65	<.0001	0.05	0.8338	1.4353
CFU-GM	E11.5AGM	0ug/ml	CFU-GM	HE+PSCs	0ug/ml	0.6130	0.1465	36	4.19	0.0002	0.05	0.3160	0.9101
CFU-GM	E11.5AGM	0ug/ml	CFU-GM	HE+PSCs	2.5ug/ml	1.4355	0.1499	36	9.58	<.0001	0.05	1.1316	1.7395
CFU-GM	E11.5AGM	0ug/ml	CFU-GM	HE+PSCs	7.5ug/ml	2.2227	0.1569	36	14.17	<.0001	0.05	1.9045	2.5408
CFU-GM	E11.5AGM	2.5ug/ml	CFU-GM	E11.5AGM	7.5ug/ml	0.4108	0.1498	36	2.74	0.0094	0.05	0.1070	0.7146
CFU-GM	E11.5AGM	2.5ug/ml	CFU-GM	HE+PSCs	0ug/ml	-0.1107	0.1480	36	-0.75	0.4593	0.05	-0.4109	0.1894
CFU-GM	E11.5AGM	2.5ug/ml	CFU-GM	HE+PSCs	2.5ug/ml	0.7118	0.1514	36	4.70	<.0001	0.05	0.4048	1.0188
CFU-GM	E11.5AGM	2.5ug/ml	CFU-GM	HE+PSCs	7.5ug/ml	1.4989	0.1583	36	9.47	<.0001	0.05	1.1779	1.8200
CFU-GM	E11.5AGM	7.5ug/ml	CFU-GM	HE+PSCs	0ug/ml	-0.5215	0.1495	36	-3.49	0.0013	0.05	-0.8247	-0.2183
CFU-GM	E11.5AGM	7.5ug/ml	CFU-GM	HE+PSCs	2.5ug/ml	0.3010	0.1528	36	1.97	0.0566	0.05	-0.00894	0.6110
CFU-GM	E11.5AGM	7.5ug/ml	CFU-GM	HE+PSCs	7.5ug/ml	1.0882	0.1597	36	6.81	<.0001	0.05	0.7643	1.4120
CFU-GM	HE+PSCs	0ug/ml	CFU-GM	HE+PSCs	2.5ug/ml	0.8225	0.1511	36	5.44	<.0001	0.05	0.5162	1.1289
CFU-GM	HE+PSCs	0ug/ml	CFU-GM	HE+PSCs	7.5ug/ml	1.6097	0.1580	36	10.19	<.0001	0.05	1.2892	1.9301
CFU-GM	HE+PSCs	2.5ug/ml	CFU-GM	HE+PSCs	7.5ug/ml	0.7871	0.1611	36	4.89	<.0001	0.05	0.4604	1.1139

The GENMOD Procedure

Model Information	
**Data Set**	WORK.EXT_FIG10BI_E11WIF1_TRANS
**Distribution**	Normal
**Link Function**	Identity
**Dependent Variable**	Percentage

**Number of Observations Read**	45
**Number of Observations Used**	45

Class Level Information		
**Class**	**Levels**	**Values**
**Condition**	3	AoE+PSCs E11.5AGM HE+PSCs
**Treatment**	3	0ug/ml 2.5ug/ml 7.5ug/ml

Parameter Information			
**Parameter**	**Effect**	**Condition**	**Treatment**
**Prm1**	Intercept		
**Prm2**	Condition	AoE+PSCs	
**Prm3**	Condition	E11.5AGM	
**Prm4**	Condition	HE+PSCs	
**Prm5**	Treatment		0ug/ml
**Prm6**	Treatment		2.5ug/ml
**Prm7**	Treatment		7.5ug/ml
**Prm8**	Condition*Treatment	AoE+PSCs	0ug/ml
**Prm9**	Condition*Treatment	AoE+PSCs	2.5ug/ml
**Prm10**	Condition*Treatment	AoE+PSCs	7.5ug/ml
**Prm11**	Condition*Treatment	E11.5AGM	0ug/ml

The GENMOD Procedure

Parameter Information			
**Parameter**	**Effect**	**Condition**	**Treatment**
**Prm12**	Condition*Treatment	E11.5AGM	2.5ug/ml
**Prm13**	Condition*Treatment	E11.5AGM	7.5ug/ml
**Prm14**	Condition*Treatment	HE+PSCs	0ug/ml
**Prm15**	Condition*Treatment	HE+PSCs	2.5ug/ml
**Prm16**	Condition*Treatment	HE+PSCs	7.5ug/ml

Criteria For Assessing Goodness Of Fit			
**Criterion**	**DF**	**Value**	**Value/DF**
**Deviance**	36	660.4000	18.3444
**Scaled Deviance**	36	45.0000	1.2500
**Pearson Chi-Square**	36	660.4000	18.3444
**Scaled Pearson X2**	36	45.0000	1.2500
**Log Likelihood**		-124.2914	
**Full Log Likelihood**		-124.2914	
**AIC (smaller is better)**		268.5827	
**AICC (smaller is better)**		275.0533	
**BIC (smaller is better)**		286.6493	

Algorithm converged.

The GENMOD Procedure

Analysis Of Maximum Likelihood Parameter Estimates									
Parameter			DF	Estimate	Standard Error	Wald 95% Confidence Limits		Wald Chi-Square	Pr > ChiSq
Intercept			1	5.4000	1.7132	2.0422	8.7578	9.93	0.0016
Condition	AoE+PSCs		1	-0.2000	2.4229	-4.9487	4.5487	0.01	0.9342
Condition	E11.5AGM		1	12.2000	2.4229	7.4513	16.9487	25.36	<.0001
Condition	HE+PSCs		0	0.0000	0.0000	0.0000	0.0000	.	.
Treatment	0ug/ml		1	15.0000	2.4229	10.2513	19.7487	38.33	<.0001
Treatment	2.5ug/ml		1	6.4000	2.4229	1.6513	11.1487	6.98	0.0083
Treatment	7.5ug/ml		0	0.0000	0.0000	0.0000	0.0000	.	.
Condition*Treatment	AoE+PSCs	0ug/ml	1	8.8000	3.4264	2.0843	15.5157	6.60	0.0102
Condition*Treatment	AoE+PSCs	2.5ug/ml	1	3.2000	3.4264	-3.5157	9.9157	0.87	0.3503
Condition*Treatment	AoE+PSCs	7.5ug/ml	0	0.0000	0.0000	0.0000	0.0000	.	.
Condition*Treatment	E11.5AGM	0ug/ml	1	26.0000	3.4264	19.2843	32.7157	57.58	<.0001
Condition*Treatment	E11.5AGM	2.5ug/ml	1	18.6000	3.4264	11.8843	25.3157	29.47	<.0001
Condition*Treatment	E11.5AGM	7.5ug/ml	0	0.0000	0.0000	0.0000	0.0000	.	.
Condition*Treatment	HE+PSCs	0ug/ml	0	0.0000	0.0000	0.0000	0.0000	.	.
Condition*Treatment	HE+PSCs	2.5ug/ml	0	0.0000	0.0000	0.0000	0.0000	.	.
Condition*Treatment	HE+PSCs	7.5ug/ml	0	0.0000	0.0000	0.0000	0.0000	.	.
Scale			1	3.8309	0.4038	3.1158	4.7100		

Note: The scale parameter was estimated by maximum likelihood.

The GENMOD Procedure

Condition*Treatment Least Squares Means								
Condition	Treatment	Estimate	Standard Error	z Value	Pr > |z|	Alpha	Lower	Upper
AoE+PSCs	0ug/ml	29.0000	1.7132	16.93	<.0001	0.05	25.6422	32.3578
AoE+PSCs	2.5ug/ml	14.8000	1.7132	8.64	<.0001	0.05	11.4422	18.1578
AoE+PSCs	7.5ug/ml	5.2000	1.7132	3.04	0.0024	0.05	1.8422	8.5578
E11.5AGM	0ug/ml	58.6000	1.7132	34.20	<.0001	0.05	55.2422	61.9578
E11.5AGM	2.5ug/ml	42.6000	1.7132	24.87	<.0001	0.05	39.2422	45.9578
E11.5AGM	7.5ug/ml	17.6000	1.7132	10.27	<.0001	0.05	14.2422	20.9578
HE+PSCs	0ug/ml	20.4000	1.7132	11.91	<.0001	0.05	17.0422	23.7578
HE+PSCs	2.5ug/ml	11.8000	1.7132	6.89	<.0001	0.05	8.4422	15.1578
HE+PSCs	7.5ug/ml	5.4000	1.7132	3.15	0.0016	0.05	2.0422	8.7578

Differences of Condition*Treatment Least Squares Means										
Condition	Treatment	_Condition	_Treatment	Estimate	Standard Error	z Value	Pr > |z|	Alpha	Lower	Upper
AoE+PSCs	0ug/ml	AoE+PSCs	2.5ug/ml	14.2000	2.4229	5.86	<.0001	0.05	9.4513	18.9487
AoE+PSCs	0ug/ml	AoE+PSCs	7.5ug/ml	23.8000	2.4229	9.82	<.0001	0.05	19.0513	28.5487
AoE+PSCs	0ug/ml	E11.5AGM	0ug/ml	-29.6000	2.4229	-12.22	<.0001	0.05	-34.3487	-24.8513
AoE+PSCs	0ug/ml	E11.5AGM	2.5ug/ml	-13.6000	2.4229	-5.61	<.0001	0.05	-18.3487	-8.8513
AoE+PSCs	0ug/ml	E11.5AGM	7.5ug/ml	11.4000	2.4229	4.71	<.0001	0.05	6.6513	16.1487
AoE+PSCs	0ug/ml	HE+PSCs	0ug/ml	8.6000	2.4229	3.55	0.0004	0.05	3.8513	13.3487
AoE+PSCs	0ug/ml	HE+PSCs	2.5ug/ml	17.2000	2.4229	7.10	<.0001	0.05	12.4513	21.9487
AoE+PSCs	0ug/ml	HE+PSCs	7.5ug/ml	23.6000	2.4229	9.74	<.0001	0.05	18.8513	28.3487
AoE+PSCs	2.5ug/ml	AoE+PSCs	7.5ug/ml	9.6000	2.4229	3.96	<.0001	0.05	4.8513	14.3487
AoE+PSCs	2.5ug/ml	E11.5AGM	0ug/ml	-43.8000	2.4229	-18.08	<.0001	0.05	-48.5487	-39.0513
AoE+PSCs	2.5ug/ml	E11.5AGM	2.5ug/ml	-27.8000	2.4229	-11.47	<.0001	0.05	-32.5487	-23.0513
AoE+PSCs	2.5ug/ml	E11.5AGM	7.5ug/ml	-2.8000	2.4229	-1.16	0.2478	0.05	-7.5487	1.9487

The GENMOD Procedure

Differences of Condition*Treatment Least Squares Means										
Condition	Treatment	_Condition	_Treatment	Estimate	Standard Error	z Value	Pr > |z|	Alpha	Lower	Upper
AoE+PSCs	2.5ug/ml	HE+PSCs	0ug/ml	-5.6000	2.4229	-2.31	0.0208	0.05	-10.3487	-0.8513
AoE+PSCs	2.5ug/ml	HE+PSCs	2.5ug/ml	3.0000	2.4229	1.24	0.2156	0.05	-1.7487	7.7487
AoE+PSCs	2.5ug/ml	HE+PSCs	7.5ug/ml	9.4000	2.4229	3.88	0.0001	0.05	4.6513	14.1487
AoE+PSCs	7.5ug/ml	E11.5AGM	0ug/ml	-53.4000	2.4229	-22.04	<.0001	0.05	-58.1487	-48.6513
AoE+PSCs	7.5ug/ml	E11.5AGM	2.5ug/ml	-37.4000	2.4229	-15.44	<.0001	0.05	-42.1487	-32.6513
AoE+PSCs	7.5ug/ml	E11.5AGM	7.5ug/ml	-12.4000	2.4229	-5.12	<.0001	0.05	-17.1487	-7.6513
AoE+PSCs	7.5ug/ml	HE+PSCs	0ug/ml	-15.2000	2.4229	-6.27	<.0001	0.05	-19.9487	-10.4513
AoE+PSCs	7.5ug/ml	HE+PSCs	2.5ug/ml	-6.6000	2.4229	-2.72	0.0064	0.05	-11.3487	-1.8513
AoE+PSCs	7.5ug/ml	HE+PSCs	7.5ug/ml	-0.2000	2.4229	-0.08	0.9342	0.05	-4.9487	4.5487
E11.5AGM	0ug/ml	E11.5AGM	2.5ug/ml	16.0000	2.4229	6.60	<.0001	0.05	11.2513	20.7487
E11.5AGM	0ug/ml	E11.5AGM	7.5ug/ml	41.0000	2.4229	16.92	<.0001	0.05	36.2513	45.7487
E11.5AGM	0ug/ml	HE+PSCs	0ug/ml	38.2000	2.4229	15.77	<.0001	0.05	33.4513	42.9487
E11.5AGM	0ug/ml	HE+PSCs	2.5ug/ml	46.8000	2.4229	19.32	<.0001	0.05	42.0513	51.5487
E11.5AGM	0ug/ml	HE+PSCs	7.5ug/ml	53.2000	2.4229	21.96	<.0001	0.05	48.4513	57.9487
E11.5AGM	2.5ug/ml	E11.5AGM	7.5ug/ml	25.0000	2.4229	10.32	<.0001	0.05	20.2513	29.7487
E11.5AGM	2.5ug/ml	HE+PSCs	0ug/ml	22.2000	2.4229	9.16	<.0001	0.05	17.4513	26.9487
E11.5AGM	2.5ug/ml	HE+PSCs	2.5ug/ml	30.8000	2.4229	12.71	<.0001	0.05	26.0513	35.5487
E11.5AGM	2.5ug/ml	HE+PSCs	7.5ug/ml	37.2000	2.4229	15.35	<.0001	0.05	32.4513	41.9487
E11.5AGM	7.5ug/ml	HE+PSCs	0ug/ml	-2.8000	2.4229	-1.16	0.2478	0.05	-7.5487	1.9487
E11.5AGM	7.5ug/ml	HE+PSCs	2.5ug/ml	5.8000	2.4229	2.39	0.0167	0.05	1.0513	10.5487
E11.5AGM	7.5ug/ml	HE+PSCs	7.5ug/ml	12.2000	2.4229	5.04	<.0001	0.05	7.4513	16.9487
HE+PSCs	0ug/ml	HE+PSCs	2.5ug/ml	8.6000	2.4229	3.55	0.0004	0.05	3.8513	13.3487
HE+PSCs	0ug/ml	HE+PSCs	7.5ug/ml	15.0000	2.4229	6.19	<.0001	0.05	10.2513	19.7487
HE+PSCs	2.5ug/ml	HE+PSCs	7.5ug/ml	6.4000	2.4229	2.64	0.0083	0.05	1.6513	11.1487

The GENMOD Procedure

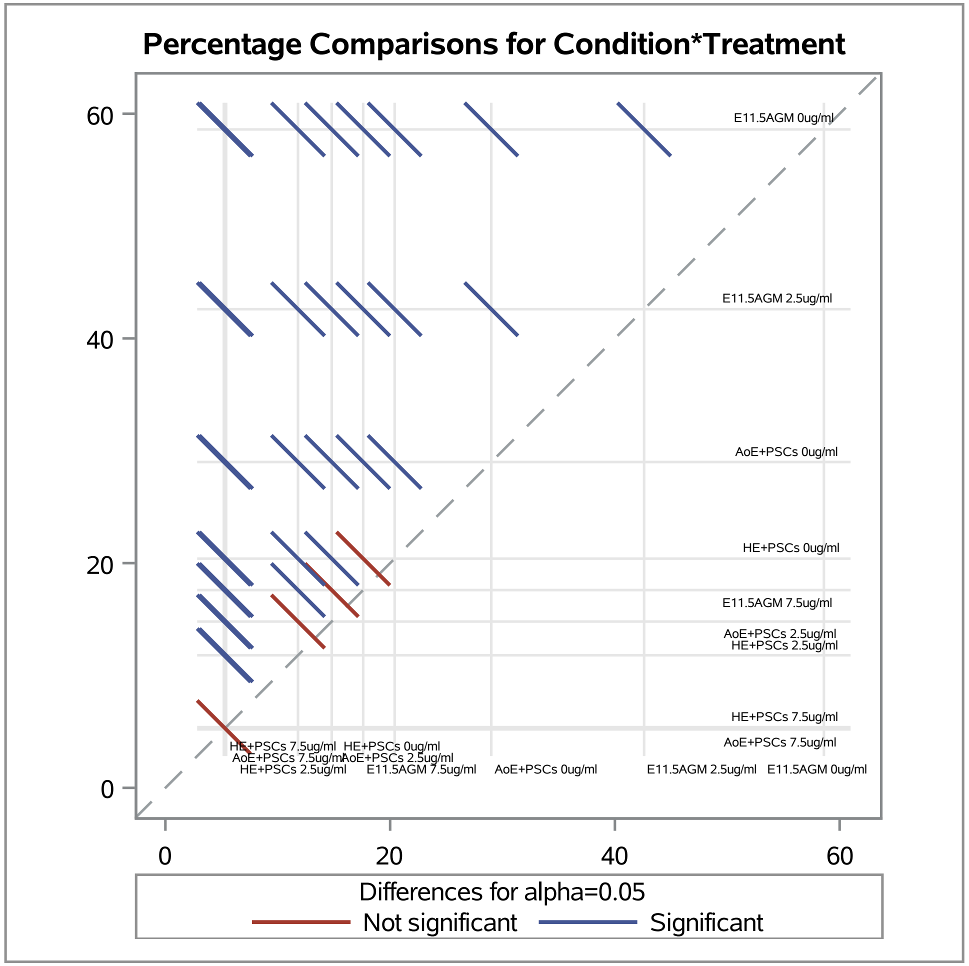

The GLIMMIX Procedure

Model Information	
**Data Set**	WORK.EXT_FIG10BII_E10LY45
**Response Variable**	Count
**Response Distribution**	Poisson
**Link Function**	Log
**Variance Function**	Default
**Variance Matrix Blocked By**	Dish_ID
**Estimation Technique**	Maximum Likelihood
**Likelihood Approximation**	Laplace
**Degrees of Freedom Method**	Containment

Class Level Information		
**Class**	**Levels**	**Values**
**Colony_Type**	3	BFU-E CFU-GEMM CFU-GM
**Condition**	3	AoE+PSCs E10.5AGM HE+PSCs
**Treatment**	3	0nM 15nM 45nM
**Dish_ID**	27	1 2 3 4 5 6 7 8 9 10 11 12 13 14 15 16 17 18 19 20 21 22 23 24 25 26 27

**Number of Observations Read**	81
**Number of Observations Used**	81

Dimensions	
**G-side Cov. Parameters**	1
**Columns in X**	64
**Columns in Z per Subject**	1
**Subjects (Blocks in V)**	27
**Max Obs per Subject**	3

The GLIMMIX Procedure

Optimization Information	
Optimization Technique	Dual Quasi-Newton
Parameters in Optimization	28
Lower Boundaries	1
Upper Boundaries	0
Fixed Effects	Not Profiled
Starting From	GLM estimates

Iteration History					
Iteration	Restarts	Evaluations	Objective Function	Change	Max Gradient
0	0	4	731.91404084	.	771.6973
1	0	8	731.21479627	0.69924456	84.07326
2	0	4	731.19561369	0.01918259	21.26228
3	0	4	731.19101734	0.00459635	22.17263
4	0	4	731.16901672	0.02200062	2.730521
5	0	4	731.16858398	0.00043274	9.077907
6	0	4	731.16338443	0.00519954	2.178605
7	0	3	731.16322517	0.00015927	4.54836
8	0	6	731.15976625	0.00345891	2.450752
9	0	3	731.15964277	0.00012348	3.038649
10	0	2	731.15945866	0.00018411	1.051621
11	0	2	731.15920787	0.00025079	4.220848
12	0	4	731.15817525	0.00103262	1.290134
13	0	3	731.1579276	0.00024765	1.409857
14	0	3	731.15788369	0.00004391	0.901424

The GLIMMIX Procedure

Iteration History					
**Iteration**	**Restarts**	**Evaluations**	**Objective Function**	**Change**	**Max Gradient**
**15**	**0**	4	731.1576505	0.00023318	0.290164
**16**	**0**	3	731.1576435	0.00000700	0.677298

Convergence criterion (GCONV=1E-8) satisfied.

Fit Statistics	
**-2 Log Likelihood**	731.16
**AIC (smaller is better)**	787.16
**AICC (smaller is better)**	818.39
**BIC (smaller is better)**	823.44
**CAIC (smaller is better)**	851.44
**HQIC (smaller is better)**	797.95

Fit Statistics for Conditional Distribution	
**-2 log L(Count | r. effects)**	675.13
**Pearson Chi-Square**	157.97
**Pearson Chi-Square / DF**	1.95

Covariance Parameter Estimates			
**Cov Parm**	**Subject**	**Estimate**	**Standard Error**
**Intercept**	Dish_ID	0.007385	0.003011

The GLIMMIX Procedure

Solutions for Fixed Effects								
**Effect**	**Colony_Type**	**Condition**	**Treatment**	**Estimate**	**Standard Error**	**DF**	**t Value**	**Pr > |t|**
**Intercept**				4.4061	0.08074	18	54.57	<.0001
**Colony_Type**	BFU-E			-1.7030	0.1622	36	-10.50	<.0001
**Colony_Type**	CFU-GEMM			-1.0323	0.1242	36	-8.31	<.0001
**Colony_Type**	CFU-GM			0	.	.	.	.
**Condition**		AoE+PSCs		0.2442	0.1103	36	2.21	0.0332
**Condition**		E10.5AGM		0.8926	0.1032	36	8.65	<.0001
**Condition**		HE+PSCs		0	.	.	.	.
**Colony_Typ*Condition**	BFU-E	AoE+PSCs		-0.04229	0.2183	36	-0.19	0.8475
**Colony_Typ*Condition**	BFU-E	E10.5AGM		-0.1647	0.1968	36	-0.84	0.4081
**Colony_Typ*Condition**	BFU-E	HE+PSCs		0	.	.	.	.
**Colony_Typ*Condition**	CFU-GEMM	AoE+PSCs		-0.7313	0.1927	36	-3.80	0.0005
**Colony_Typ*Condition**	CFU-GEMM	E10.5AGM		-0.7430	0.1640	36	-4.53	<.0001
**Colony_Typ*Condition**	CFU-GEMM	HE+PSCs		0	.	.	.	.
**Colony_Typ*Condition**	CFU-GM	AoE+PSCs		0	.	.	.	.
**Colony_Typ*Condition**	CFU-GM	E10.5AGM		0	.	.	.	.
**Colony_Typ*Condition**	CFU-GM	HE+PSCs		0	.	.	.	.
**Treatment**			0nM	1.6997	0.09861	36	17.24	<.0001
**Treatment**			15nM	0.8514	0.1035	36	8.22	<.0001
**Treatment**			45nM	0	.	.	.	.
**Colony_Typ*Treatment**	BFU-E		0nM	0.1570	0.1747	36	0.90	0.3747
**Colony_Typ*Treatment**	BFU-E		15nM	-0.09590	0.1961	36	-0.49	0.6277
**Colony_Typ*Treatment**	BFU-E		45nM	0	.	.	.	.
**Colony_Typ*Treatment**	CFU-GEMM		0nM	-0.6006	0.1413	36	-4.25	0.0001
**Colony_Typ*Treatment**	CFU-GEMM		15nM	-0.5107	0.1588	36	-3.22	0.0027

The GLIMMIX Procedure

Solutions for Fixed Effects								
**Effect**	**Colony_Type**	**Condition**	**Treatment**	**Estimate**	**Standard Error**	**DF**	**t Value**	**Pr > |t|**
**Colony_Typ*Treatment**	CFU-GEMM		45nM	0	.	.	.	.
**Colony_Typ*Treatment**	CFU-GM		0nM	0	.	.	.	.
**Colony_Typ*Treatment**	CFU-GM		15nM	0	.	.	.	.
**Colony_Typ*Treatment**	CFU-GM		45nM	0	.	.	.	.
**Condition*Treatment**		AoE+PSCs	0nM	-0.1564	0.1360	36	-1.15	0.2579
**Condition*Treatment**		AoE+PSCs	15nM	-0.1092	0.1426	36	-0.77	0.4487
**Condition*Treatment**		AoE+PSCs	45nM	0	.	.	.	.
**Condition*Treatment**		E10.5AGM	0nM	-0.3599	0.1294	36	-2.78	0.0086
**Condition*Treatment**		E10.5AGM	15nM	-0.1640	0.1347	36	-1.22	0.2313
**Condition*Treatment**		E10.5AGM	45nM	0	.	.	.	.
**Condition*Treatment**		HE+PSCs	0nM	0	.	.	.	.
**Condition*Treatment**		HE+PSCs	15nM	0	.	.	.	.
**Condition*Treatment**		HE+PSCs	45nM	0	.	.	.	.
**Colony*Condit*Treatm**	BFU-E	AoE+PSCs	0nM	0.1647	0.2353	36	0.70	0.4884
**Colony*Condit*Treatm**	BFU-E	AoE+PSCs	15nM	0.08074	0.2647	36	0.30	0.7621
**Colony*Condit*Treatm**	BFU-E	AoE+PSCs	45nM	0	.	.	.	.
**Colony*Condit*Treatm**	BFU-E	E10.5AGM	0nM	0.3606	0.2122	36	1.70	0.0979
**Colony*Condit*Treatm**	BFU-E	E10.5AGM	15nM	0.7900	0.2332	36	3.39	0.0017
**Colony*Condit*Treatm**	BFU-E	E10.5AGM	45nM	0	.	.	.	.
**Colony*Condit*Treatm**	BFU-E	HE+PSCs	0nM	0	.	.	.	.
**Colony*Condit*Treatm**	BFU-E	HE+PSCs	15nM	0	.	.	.	.
**Colony*Condit*Treatm**	BFU-E	HE+PSCs	45nM	0	.	.	.	.
**Colony*Condit*Treatm**	CFU-GEMM	AoE+PSCs	0nM	0.7950	0.2135	36	3.72	0.0007
**Colony*Condit*Treatm**	CFU-GEMM	AoE+PSCs	15nM	0.6452	0.2369	36	2.72	0.0099

The GLIMMIX Procedure

Solutions for Fixed Effects								
Effect	Colony_Type	Condition	Treatment	Estimate	Standard Error	DF	t Value	Pr > |t|
**Colony*Condit*Treatm**	CFU-GEMM	AoE+PSCs	45nM	0	.	.	.	.
**Colony*Condit*Treatm**	CFU-GEMM	E10.5AGM	0nM	0.9178	0.1837	36	5.00	<.0001
**Colony*Condit*Treatm**	CFU-GEMM	E10.5AGM	15nM	1.2028	0.2000	36	6.02	<.0001
**Colony*Condit*Treatm**	CFU-GEMM	E10.5AGM	45nM	0	.	.	.	.
**Colony*Condit*Treatm**	CFU-GEMM	HE+PSCs	0nM	0	.	.	.	.
**Colony*Condit*Treatm**	CFU-GEMM	HE+PSCs	15nM	0	.	.	.	.
**Colony*Condit*Treatm**	CFU-GEMM	HE+PSCs	45nM	0	.	.	.	.
**Colony*Condit*Treatm**	CFU-GM	AoE+PSCs	0nM	0	.	.	.	.
**Colony*Condit*Treatm**	CFU-GM	AoE+PSCs	15nM	0	.	.	.	.
**Colony*Condit*Treatm**	CFU-GM	AoE+PSCs	45nM	0	.	.	.	.
**Colony*Condit*Treatm**	CFU-GM	E10.5AGM	0nM	0	.	.	.	.
**Colony*Condit*Treatm**	CFU-GM	E10.5AGM	15nM	0	.	.	.	.
**Colony*Condit*Treatm**	CFU-GM	E10.5AGM	45nM	0	.	.	.	.
**Colony*Condit*Treatm**	CFU-GM	HE+PSCs	0nM	0	.	.	.	.
**Colony*Condit*Treatm**	CFU-GM	HE+PSCs	15nM	0	.	.	.	.
**Colony*Condit*Treatm**	CFU-GM	HE+PSCs	45nM	0	.	.	.	.

Type III Tests of Fixed Effects				
Effect	Num DF	Den DF	F Value	Pr > F
**Colony_Type**	2	36	1876.64	<.0001
**Condition**	2	36	134.35	<.0001
**Colony_Typ*Condition**	4	36	4.92	0.0029
**Treatment**	2	36	437.99	<.0001
**Colony_Typ*Treatment**	4	36	5.90	0.0009

The GLIMMIX Procedure

Type III Tests of Fixed Effects				
Effect	Num DF	Den DF	F Value	Pr > F
Condition*Treatment	4	36	6.25	0.0006
Colony*Condit*Treatm	8	36	7.71	<.0001

Condition*Treatment Least Squares Means													
Condition	Treatment	Estimate	Standard Error	DF	t Value	Pr > |t|	Alpha	Lower	Upper	Mean	Standard Error Mean	Lower Mean	Upper Mean
AoE+PSCs	0nM	5.1960	0.05670	36	91.65	<.0001	0.05	5.0810	5.3110	180.55	10.2361	160.93	202.55
AoE+PSCs	15nM	4.2626	0.06684	36	63.78	<.0001	0.05	4.1271	4.3982	70.9976	4.7453	61.9974	81.3043
AoE+PSCs	45nM	3.4807	0.08307	36	41.90	<.0001	0.05	3.3122	3.6492	32.4822	2.6983	27.4460	38.4425
E10.5AGM	0nM	5.7024	0.05390	36	105.79	<.0001	0.05	5.5931	5.8117	299.58	16.1477	268.55	334.18
E10.5AGM	15nM	5.2338	0.05595	36	93.54	<.0001	0.05	5.1203	5.3473	187.50	10.4915	167.39	210.04
E10.5AGM	45nM	4.0844	0.07025	36	58.14	<.0001	0.05	3.9419	4.2268	59.4041	4.1732	51.5158	68.5003
HE+PSCs	0nM	5.0461	0.05792	36	87.13	<.0001	0.05	4.9286	5.1636	155.42	9.0013	138.19	174.79
HE+PSCs	15nM	4.1435	0.06871	36	60.30	<.0001	0.05	4.0042	4.2829	63.0242	4.3307	54.8257	72.4487
HE+PSCs	45nM	3.4943	0.08157	36	42.84	<.0001	0.05	3.3289	3.6598	32.9279	2.6860	27.9072	38.8518

Differences of Condition*Treatment Least Squares Means											
Condition	Treatment	_Condition	_Treatment	Estimate	Standard Error	DF	t Value	Pr > |t|	Alpha	Lower	Upper
AoE+PSCs	0nM	AoE+PSCs	15nM	0.9333	0.08764	36	10.65	<.0001	0.05	0.7556	1.1111
AoE+PSCs	0nM	AoE+PSCs	45nM	1.7153	0.1006	36	17.06	<.0001	0.05	1.5113	1.9193
AoE+PSCs	0nM	E10.5AGM	0nM	-0.5064	0.07823	36	-6.47	<.0001	0.05	-0.6650	-0.3477
AoE+PSCs	0nM	E10.5AGM	15nM	-0.03782	0.07966	36	-0.47	0.6378	0.05	-0.1994	0.1237
AoE+PSCs	0nM	E10.5AGM	45nM	1.1116	0.09027	36	12.31	<.0001	0.05	0.9285	1.2947
AoE+PSCs	0nM	HE+PSCs	0nM	0.1499	0.08105	36	1.85	0.0727	0.05	-0.01450	0.3142

The GLIMMIX Procedure

Differences of Condition*Treatment Least Squares Means

Condition	Treatment	_Condition	_Treatment	Estimate	Standard Error	DF	t Value	Pr > |t|	Alpha	Lower	Upper
AoE+PSCs	0nM	HE+PSCs	15nM	1.0525	0.08908	36	11.81	<.0001	0.05	0.8718	1.2331
AoE+PSCs	0nM	HE+PSCs	45nM	1.7017	0.09934	36	17.13	<.0001	0.05	1.5002	1.9031
AoE+PSCs	15nM	AoE+PSCs	45nM	0.7820	0.1066	36	7.34	<.0001	0.05	0.5658	0.9982
AoE+PSCs	15nM	E10.5AGM	0nM	-1.4397	0.08586	36	-16.77	<.0001	0.05	-1.6139	-1.2656
AoE+PSCs	15nM	E10.5AGM	15nM	-0.9712	0.08716	36	-11.14	<.0001	0.05	-1.1479	-0.7944
AoE+PSCs	15nM	E10.5AGM	45nM	0.1783	0.09696	36	1.84	0.0742	0.05	-0.01835	0.3749
AoE+PSCs	15nM	HE+PSCs	0nM	-0.7835	0.08843	36	-8.86	<.0001	0.05	-0.9628	-0.6041
AoE+PSCs	15nM	HE+PSCs	15nM	0.1191	0.09583	36	1.24	0.2219	0.05	-0.07523	0.3135
AoE+PSCs	15nM	HE+PSCs	45nM	0.7683	0.1054	36	7.29	<.0001	0.05	0.5545	0.9822
AoE+PSCs	45nM	E10.5AGM	0nM	-2.2217	0.09902	36	-22.44	<.0001	0.05	-2.4225	-2.0209
AoE+PSCs	45nM	E10.5AGM	15nM	-1.7531	0.1002	36	-17.50	<.0001	0.05	-1.9562	-1.5500
AoE+PSCs	45nM	E10.5AGM	45nM	-0.6037	0.1088	36	-5.55	<.0001	0.05	-0.8243	-0.3830
AoE+PSCs	45nM	HE+PSCs	0nM	-1.5654	0.1013	36	-15.46	<.0001	0.05	-1.7708	-1.3601
AoE+PSCs	45nM	HE+PSCs	15nM	-0.6628	0.1078	36	-6.15	<.0001	0.05	-0.8814	-0.4442
AoE+PSCs	45nM	HE+PSCs	45nM	-0.01363	0.1164	36	-0.12	0.9075	0.05	-0.2497	0.2225
E10.5AGM	0nM	E10.5AGM	15nM	0.4686	0.07769	36	6.03	<.0001	0.05	0.3110	0.6261
E10.5AGM	0nM	E10.5AGM	45nM	1.6180	0.08855	36	18.27	<.0001	0.05	1.4384	1.7976
E10.5AGM	0nM	HE+PSCs	0nM	0.6563	0.07912	36	8.29	<.0001	0.05	0.4958	0.8167
E10.5AGM	0nM	HE+PSCs	15nM	1.5588	0.08733	36	17.85	<.0001	0.05	1.3817	1.7360
E10.5AGM	0nM	HE+PSCs	45nM	2.2080	0.09777	36	22.58	<.0001	0.05	2.0098	2.4063
E10.5AGM	15nM	E10.5AGM	45nM	1.1494	0.08981	36	12.80	<.0001	0.05	0.9673	1.3316
E10.5AGM	15nM	HE+PSCs	0nM	0.1877	0.08053	36	2.33	0.0255	0.05	0.02437	0.3510
E10.5AGM	15nM	HE+PSCs	15nM	1.0903	0.08861	36	12.30	<.0001	0.05	0.9106	1.2700
E10.5AGM	15nM	HE+PSCs	45nM	1.7395	0.09891	36	17.59	<.0001	0.05	1.5389	1.9401

The GLIMMIX Procedure

Differences of Condition*Treatment Least Squares Means											
Condition	Treatment	_Condition	_Treatment	Estimate	Standard Error	DF	t Value	Pr > |t|	Alpha	Lower	Upper
E10.5AGM	45nM	HE+PSCs	0nM	-0.9617	0.09104	36	-10.56	<.0001	0.05	-1.1464	-0.7771
E10.5AGM	45nM	HE+PSCs	15nM	-0.05916	0.09826	36	-0.60	0.5509	0.05	-0.2584	0.1401
E10.5AGM	45nM	HE+PSCs	45nM	0.5900	0.1076	36	5.48	<.0001	0.05	0.3717	0.8084
HE+PSCs	0nM	HE+PSCs	15nM	0.9026	0.08986	36	10.04	<.0001	0.05	0.7204	1.0848
HE+PSCs	0nM	HE+PSCs	45nM	1.5518	0.1000	36	15.51	<.0001	0.05	1.3489	1.7547
HE+PSCs	15nM	HE+PSCs	45nM	0.6492	0.1066	36	6.09	<.0001	0.05	0.4329	0.8655

Colony*Condit*Treatm Least Squares Means														
Colony_Type	Condition	Treatment	Estimate	Standard Error	DF	t Value	Pr > |t|	Alpha	Lower	Upper	Mean	Standard Error Mean	Lower Mean	Upper Mean
BFU-E	AoE+PSCs	0nM	4.7700	0.07271	36	65.60	<.0001	0.05	4.6225	4.9175	117.92	8.5737	101.75	136.65
BFU-E	AoE+PSCs	15nM	3.6320	0.1060	36	34.25	<.0001	0.05	3.4170	3.8471	37.7888	4.0069	30.4768	46.8550
BFU-E	AoE+PSCs	45nM	2.9050	0.1437	36	20.22	<.0001	0.05	2.6136	3.1964	18.2654	2.6246	13.6480	24.4451
BFU-E	E10.5AGM	0nM	5.2884	0.06437	36	82.16	<.0001	0.05	5.1579	5.4189	198.02	12.7460	173.79	225.64
BFU-E	E10.5AGM	15nM	4.8124	0.07187	36	66.96	<.0001	0.05	4.6667	4.9582	123.03	8.8425	106.34	142.34
BFU-E	E10.5AGM	45nM	3.4310	0.1150	36	29.84	<.0001	0.05	3.1978	3.6641	30.9070	3.5533	24.4790	39.0229
BFU-E	HE+PSCs	0nM	4.5598	0.07706	36	59.17	<.0001	0.05	4.4035	4.7161	95.5609	7.3640	81.7346	111.73
BFU-E	HE+PSCs	15nM	3.4586	0.1135	36	30.46	<.0001	0.05	3.2283	3.6888	31.7713	3.6074	25.2364	39.9984
BFU-E	HE+PSCs	45nM	2.7031	0.1572	36	17.19	<.0001	0.05	2.3842	3.0219	14.9255	2.3466	10.8505	20.5308
CFU-GEMM	AoE+PSCs	0nM	4.6244	0.07569	36	61.09	<.0001	0.05	4.4709	4.7779	101.94	7.7159	87.4320	118.85
CFU-GEMM	AoE+PSCs	15nM	3.7634	0.1008	36	37.33	<.0001	0.05	3.5590	3.9679	43.0964	4.3445	35.1276	52.8729
CFU-GEMM	AoE+PSCs	45nM	2.8867	0.1449	36	19.93	<.0001	0.05	2.5930	3.1805	17.9348	2.5979	13.3694	24.0592
CFU-GEMM	E10.5AGM	0nM	5.1803	0.06584	36	78.68	<.0001	0.05	5.0468	5.3138	177.73	11.7019	155.52	203.12
CFU-GEMM	E10.5AGM	15nM	4.9029	0.07023	36	69.81	<.0001	0.05	4.7605	5.0453	134.68	9.4581	116.80	155.29

The GLIMMIX Procedure

Colony*Condit*Treatm Least Squares Means														
Colony_Type	Condition	Treatment	Estimate	Standard Error	DF	t Value	Pr > |t|	Alpha	Lower	Upper	Mean	Standard Error Mean	Lower Mean	Upper Mean
CFU-GEMM	E10.5AGM	45nM	3.5234	0.1108	36	31.81	<.0001	0.05	3.2988	3.7480	33.8998	3.7548	27.0794	42.4382
CFU-GEMM	HE+PSCs	0nM	4.4728	0.07908	36	56.56	<.0001	0.05	4.3124	4.6332	87.6026	6.9278	74.6213	102.84
CFU-GEMM	HE+PSCs	15nM	3.7145	0.1027	36	36.18	<.0001	0.05	3.5063	3.9227	41.0385	4.2132	33.3246	50.5379
CFU-GEMM	HE+PSCs	45nM	3.3738	0.1177	36	28.67	<.0001	0.05	3.1352	3.6124	29.1889	3.4345	22.9922	37.0558
CFU-GM	AoE+PSCs	0nM	6.1936	0.05606	36	110.49	<.0001	0.05	6.0799	6.3073	489.59	27.4442	436.98	548.54
CFU-GM	AoE+PSCs	15nM	5.3925	0.06308	36	85.49	<.0001	0.05	5.2646	5.5204	219.75	13.8612	193.36	249.74
CFU-GM	AoE+PSCs	45nM	4.6503	0.07513	36	61.89	<.0001	0.05	4.4979	4.8027	104.62	7.8603	89.8323	121.84
CFU-GM	E10.5AGM	0nM	6.6384	0.05383	36	123.32	<.0001	0.05	6.5293	6.7476	763.90	41.1220	684.89	852.02
CFU-GM	E10.5AGM	15nM	5.9861	0.05744	36	104.22	<.0001	0.05	5.8696	6.1026	397.85	22.8517	354.11	447.01
CFU-GM	E10.5AGM	45nM	5.2987	0.06425	36	82.47	<.0001	0.05	5.1684	5.4290	200.08	12.8544	175.63	227.92
CFU-GM	HE+PSCs	0nM	6.1058	0.05662	36	107.84	<.0001	0.05	5.9909	6.2206	448.43	25.3887	399.79	503.00
CFU-GM	HE+PSCs	15nM	5.2575	0.06481	36	81.12	<.0001	0.05	5.1260	5.3889	192.00	12.4437	168.35	218.97
CFU-GM	HE+PSCs	45nM	4.4061	0.08074	36	54.57	<.0001	0.05	4.2423	4.5699	81.9489	6.6169	69.5704	96.5300

The GLIMMIX Procedure

Differences of Colony*Condit*Treatm Least Squares Means													
Colony_Type	Condition	Treatment	_Colony_Type	_Condition	_Treatment	Estimate	Standard Error	DF	t Value	Pr > |t|	Alpha	Lower	Upper
BFU-E	AoE+PSCs	0nM	BFU-E	AoE+PSCs	15nM	1.1380	0.1286	36	8.85	<.0001	0.05	0.8772	1.3987
BFU-E	AoE+PSCs	0nM	BFU-E	AoE+PSCs	45nM	1.8650	0.1610	36	11.58	<.0001	0.05	1.5384	2.1916
BFU-E	AoE+PSCs	0nM	BFU-E	E10.5AGM	0nM	-0.5184	0.09711	36	-5.34	<.0001	0.05	-0.7153	-0.3215
BFU-E	AoE+PSCs	0nM	BFU-E	E10.5AGM	15nM	-0.04243	0.1022	36	-0.42	0.6806	0.05	-0.2498	0.1649
BFU-E	AoE+PSCs	0nM	BFU-E	E10.5AGM	45nM	1.3390	0.1360	36	9.84	<.0001	0.05	1.0631	1.6149
BFU-E	AoE+PSCs	0nM	BFU-E	HE+PSCs	0nM	0.2102	0.1059	36	1.98	0.0549	0.05	-0.00463	0.4251
BFU-E	AoE+PSCs	0nM	BFU-E	HE+PSCs	15nM	1.3114	0.1348	36	9.73	<.0001	0.05	1.0380	1.5849
BFU-E	AoE+PSCs	0nM	BFU-E	HE+PSCs	45nM	2.0669	0.1732	36	11.93	<.0001	0.05	1.7156	2.4182
BFU-E	AoE+PSCs	0nM	CFU-GEMM	AoE+PSCs	0nM	0.1456	0.07805	36	1.87	0.0702	0.05	-0.01267	0.3039
BFU-E	AoE+PSCs	0nM	CFU-GEMM	AoE+PSCs	15nM	1.0066	0.1243	36	8.10	<.0001	0.05	0.7545	1.2586
BFU-E	AoE+PSCs	0nM	CFU-GEMM	AoE+PSCs	45nM	1.8833	0.1621	36	11.62	<.0001	0.05	1.5546	2.2120
BFU-E	AoE+PSCs	0nM	CFU-GEMM	E10.5AGM	0nM	-0.4103	0.09809	36	-4.18	0.0002	0.05	-0.6092	-0.2113
BFU-E	AoE+PSCs	0nM	CFU-GEMM	E10.5AGM	15nM	-0.1329	0.1011	36	-1.31	0.1970	0.05	-0.3379	0.07213
BFU-E	AoE+PSCs	0nM	CFU-GEMM	E10.5AGM	45nM	1.2466	0.1325	36	9.41	<.0001	0.05	0.9779	1.5153
BFU-E	AoE+PSCs	0nM	CFU-GEMM	HE+PSCs	0nM	0.2972	0.1074	36	2.77	0.0089	0.05	0.07932	0.5151
BFU-E	AoE+PSCs	0nM	CFU-GEMM	HE+PSCs	15nM	1.0555	0.1258	36	8.39	<.0001	0.05	0.8003	1.3106
BFU-E	AoE+PSCs	0nM	CFU-GEMM	HE+PSCs	45nM	1.3962	0.1383	36	10.09	<.0001	0.05	1.1157	1.6767
BFU-E	AoE+PSCs	0nM	CFU-GM	AoE+PSCs	0nM	-1.4236	0.05920	36	-24.05	<.0001	0.05	-1.5436	-1.3035
BFU-E	AoE+PSCs	0nM	CFU-GM	AoE+PSCs	15nM	-0.6225	0.09626	36	-6.47	<.0001	0.05	-0.8177	-0.4273
BFU-E	AoE+PSCs	0nM	CFU-GM	AoE+PSCs	45nM	0.1197	0.1046	36	1.14	0.2599	0.05	-0.09236	0.3317
BFU-E	AoE+PSCs	0nM	CFU-GM	E10.5AGM	0nM	-1.8684	0.09047	36	-20.65	<.0001	0.05	-2.0519	-1.6850
BFU-E	AoE+PSCs	0nM	CFU-GM	E10.5AGM	15nM	-1.2161	0.09266	36	-13.12	<.0001	0.05	-1.4040	-1.0282
BFU-E	AoE+PSCs	0nM	CFU-GM	E10.5AGM	45nM	-0.5287	0.09703	36	-5.45	<.0001	0.05	-0.7255	-0.3319
BFU-E	AoE+PSCs	0nM	CFU-GM	HE+PSCs	0nM	-1.3358	0.09215	36	-14.50	<.0001	0.05	-1.5227	-1.1489

The GLIMMIX Procedure

Differences of Colony*Condit*Treatm Least Squares Means													
Colony_Type	Condition	Treatment	_Colony_Type	_Condition	_Treatment	Estimate	Standard Error	DF	t Value	Pr > |t|	Alpha	Lower	Upper
BFU-E	AoE+PSCs	0nM	CFU-GM	HE+PSCs	15nM	-0.4875	0.09740	36	-5.00	<.0001	0.05	-0.6850	-0.2899
BFU-E	AoE+PSCs	0nM	CFU-GM	HE+PSCs	45nM	0.3639	0.1087	36	3.35	0.0019	0.05	0.1435	0.5843
BFU-E	AoE+PSCs	15nM	BFU-E	AoE+PSCs	45nM	0.7270	0.1786	36	4.07	0.0002	0.05	0.3648	1.0892
BFU-E	AoE+PSCs	15nM	BFU-E	E10.5AGM	0nM	-1.6564	0.1240	36	-13.35	<.0001	0.05	-1.9079	-1.4048
BFU-E	AoE+PSCs	15nM	BFU-E	E10.5AGM	15nM	-1.1804	0.1281	36	-9.22	<.0001	0.05	-1.4402	-0.9206
BFU-E	AoE+PSCs	15nM	BFU-E	E10.5AGM	45nM	0.2010	0.1564	36	1.29	0.2069	0.05	-0.1162	0.5182
BFU-E	AoE+PSCs	15nM	BFU-E	HE+PSCs	0nM	-0.9278	0.1311	36	-7.08	<.0001	0.05	-1.1936	-0.6619
BFU-E	AoE+PSCs	15nM	BFU-E	HE+PSCs	15nM	0.1734	0.1553	36	1.12	0.2716	0.05	-0.1416	0.4885
BFU-E	AoE+PSCs	15nM	BFU-E	HE+PSCs	45nM	0.9289	0.1896	36	4.90	<.0001	0.05	0.5444	1.3135
BFU-E	AoE+PSCs	15nM	CFU-GEMM	AoE+PSCs	0nM	-0.9924	0.1303	36	-7.62	<.0001	0.05	-1.2566	-0.7281
BFU-E	AoE+PSCs	15nM	CFU-GEMM	AoE+PSCs	15nM	-0.1314	0.1283	36	-1.02	0.3126	0.05	-0.3917	0.1288
BFU-E	AoE+PSCs	15nM	CFU-GEMM	AoE+PSCs	45nM	0.7453	0.1795	36	4.15	0.0002	0.05	0.3812	1.1093
BFU-E	AoE+PSCs	15nM	CFU-GEMM	E10.5AGM	0nM	-1.5483	0.1248	36	-12.41	<.0001	0.05	-1.8014	-1.2951
BFU-E	AoE+PSCs	15nM	CFU-GEMM	E10.5AGM	15nM	-1.2709	0.1272	36	-9.99	<.0001	0.05	-1.5288	-1.0129
BFU-E	AoE+PSCs	15nM	CFU-GEMM	E10.5AGM	45nM	0.1086	0.1533	36	0.71	0.4833	0.05	-0.2024	0.4196
BFU-E	AoE+PSCs	15nM	CFU-GEMM	HE+PSCs	0nM	-0.8408	0.1323	36	-6.36	<.0001	0.05	-1.1091	-0.5725
BFU-E	AoE+PSCs	15nM	CFU-GEMM	HE+PSCs	15nM	-0.08250	0.1476	36	-0.56	0.5796	0.05	-0.3818	0.2168
BFU-E	AoE+PSCs	15nM	CFU-GEMM	HE+PSCs	45nM	0.2582	0.1584	36	1.63	0.1117	0.05	-0.06299	0.5794
BFU-E	AoE+PSCs	15nM	CFU-GM	AoE+PSCs	0nM	-2.5616	0.1199	36	-21.36	<.0001	0.05	-2.8048	-2.3183
BFU-E	AoE+PSCs	15nM	CFU-GM	AoE+PSCs	15nM	-1.7605	0.1014	36	-17.36	<.0001	0.05	-1.9661	-1.5548
BFU-E	AoE+PSCs	15nM	CFU-GM	AoE+PSCs	45nM	-1.0183	0.1299	36	-7.84	<.0001	0.05	-1.2818	-0.7548
BFU-E	AoE+PSCs	15nM	CFU-GM	E10.5AGM	0nM	-3.0064	0.1189	36	-25.28	<.0001	0.05	-3.2476	-2.7653
BFU-E	AoE+PSCs	15nM	CFU-GM	E10.5AGM	15nM	-2.3541	0.1206	36	-19.52	<.0001	0.05	-2.5986	-2.1095
BFU-E	AoE+PSCs	15nM	CFU-GM	E10.5AGM	45nM	-1.6667	0.1240	36	-13.44	<.0001	0.05	-1.9181	-1.4153

The GLIMMIX Procedure

Differences of Colony*Condit*Treatm Least Squares Means													
Colony_Type	Condition	Treatment	_Colony_Type	_Condition	_Treatment	Estimate	Standard Error	DF	t Value	Pr > |t|	Alpha	Lower	Upper
BFU-E	AoE+PSCs	15nM	CFU-GM	HE+PSCs	0nM	-2.4737	0.1202	36	-20.58	<.0001	0.05	-2.7175	-2.2300
BFU-E	AoE+PSCs	15nM	CFU-GM	HE+PSCs	15nM	-1.6255	0.1243	36	-13.08	<.0001	0.05	-1.8775	-1.3735
BFU-E	AoE+PSCs	15nM	CFU-GM	HE+PSCs	45nM	-0.7741	0.1333	36	-5.81	<.0001	0.05	-1.0444	-0.5038
BFU-E	AoE+PSCs	45nM	BFU-E	E10.5AGM	0nM	-2.3834	0.1574	36	-15.14	<.0001	0.05	-2.7027	-2.0641
BFU-E	AoE+PSCs	45nM	BFU-E	E10.5AGM	15nM	-1.9074	0.1607	36	-11.87	<.0001	0.05	-2.2333	-1.5816
BFU-E	AoE+PSCs	45nM	BFU-E	E10.5AGM	45nM	-0.5260	0.1840	36	-2.86	0.0070	0.05	-0.8992	-0.1528
BFU-E	AoE+PSCs	45nM	BFU-E	HE+PSCs	0nM	-1.6548	0.1630	36	-10.15	<.0001	0.05	-1.9854	-1.3241
BFU-E	AoE+PSCs	45nM	BFU-E	HE+PSCs	15nM	-0.5536	0.1831	36	-3.02	0.0046	0.05	-0.9249	-0.1822
BFU-E	AoE+PSCs	45nM	BFU-E	HE+PSCs	45nM	0.2019	0.2130	36	0.95	0.3494	0.05	-0.2300	0.6339
BFU-E	AoE+PSCs	45nM	CFU-GEMM	AoE+PSCs	0nM	-1.7194	0.1624	36	-10.59	<.0001	0.05	-2.0487	-1.3900
BFU-E	AoE+PSCs	45nM	CFU-GEMM	AoE+PSCs	15nM	-0.8584	0.1755	36	-4.89	<.0001	0.05	-1.2144	-0.5025
BFU-E	AoE+PSCs	45nM	CFU-GEMM	AoE+PSCs	45nM	0.01827	0.1915	36	0.10	0.9245	0.05	-0.3702	0.4067
BFU-E	AoE+PSCs	45nM	CFU-GEMM	E10.5AGM	0nM	-2.2753	0.1581	36	-14.40	<.0001	0.05	-2.5958	-1.9547
BFU-E	AoE+PSCs	45nM	CFU-GEMM	E10.5AGM	15nM	-1.9979	0.1599	36	-12.49	<.0001	0.05	-2.3222	-1.6735
BFU-E	AoE+PSCs	45nM	CFU-GEMM	E10.5AGM	45nM	-0.6184	0.1814	36	-3.41	0.0016	0.05	-0.9863	-0.2505
BFU-E	AoE+PSCs	45nM	CFU-GEMM	HE+PSCs	0nM	-1.5678	0.1640	36	-9.56	<.0001	0.05	-1.9004	-1.2352
BFU-E	AoE+PSCs	45nM	CFU-GEMM	HE+PSCs	15nM	-0.8095	0.1766	36	-4.58	<.0001	0.05	-1.1676	-0.4514
BFU-E	AoE+PSCs	45nM	CFU-GEMM	HE+PSCs	45nM	-0.4688	0.1857	36	-2.52	0.0161	0.05	-0.8454	-0.09214
BFU-E	AoE+PSCs	45nM	CFU-GM	AoE+PSCs	0nM	-3.2886	0.1542	36	-21.32	<.0001	0.05	-3.6014	-2.9758
BFU-E	AoE+PSCs	45nM	CFU-GM	AoE+PSCs	15nM	-2.4875	0.1569	36	-15.85	<.0001	0.05	-2.8057	-2.1692
BFU-E	AoE+PSCs	45nM	CFU-GM	AoE+PSCs	45nM	-1.7453	0.1461	36	-11.94	<.0001	0.05	-2.0416	-1.4490
BFU-E	AoE+PSCs	45nM	CFU-GM	E10.5AGM	0nM	-3.7334	0.1534	36	-24.33	<.0001	0.05	-4.0446	-3.4222
BFU-E	AoE+PSCs	45nM	CFU-GM	E10.5AGM	15nM	-3.0811	0.1547	36	-19.91	<.0001	0.05	-3.3949	-2.7672
BFU-E	AoE+PSCs	45nM	CFU-GM	E10.5AGM	45nM	-2.3937	0.1574	36	-15.21	<.0001	0.05	-2.7129	-2.0745

The GLIMMIX Procedure

Differences of Colony*Condit*Treatm Least Squares Means													
Colony_Type	Condition	Treatment	_Colony_Type	_Condition	_Treatment	Estimate	Standard Error	DF	t Value	Pr > |t|	Alpha	Lower	Upper
BFU-E	AoE+PSCs	45nM	CFU-GM	HE+PSCs	0nM	-3.2007	0.1544	36	-20.73	<.0001	0.05	-3.5140	-2.8875
BFU-E	AoE+PSCs	45nM	CFU-GM	HE+PSCs	15nM	-2.3525	0.1576	36	-14.93	<.0001	0.05	-2.6721	-2.0328
BFU-E	AoE+PSCs	45nM	CFU-GM	HE+PSCs	45nM	-1.5011	0.1648	36	-9.11	<.0001	0.05	-1.8353	-1.1668
BFU-E	E10.5AGM	0nM	BFU-E	E10.5AGM	15nM	0.4760	0.09648	36	4.93	<.0001	0.05	0.2803	0.6716
BFU-E	E10.5AGM	0nM	BFU-E	E10.5AGM	45nM	1.8574	0.1318	36	14.10	<.0001	0.05	1.5902	2.1246
BFU-E	E10.5AGM	0nM	BFU-E	HE+PSCs	0nM	0.7286	0.1004	36	7.26	<.0001	0.05	0.5250	0.9323
BFU-E	E10.5AGM	0nM	BFU-E	HE+PSCs	15nM	1.8298	0.1305	36	14.02	<.0001	0.05	1.5651	2.0945
BFU-E	E10.5AGM	0nM	BFU-E	HE+PSCs	45nM	2.5853	0.1699	36	15.22	<.0001	0.05	2.2408	2.9299
BFU-E	E10.5AGM	0nM	CFU-GEMM	AoE+PSCs	0nM	0.6640	0.09936	36	6.68	<.0001	0.05	0.4625	0.8655
BFU-E	E10.5AGM	0nM	CFU-GEMM	AoE+PSCs	15nM	1.5250	0.1196	36	12.75	<.0001	0.05	1.2824	1.7675
BFU-E	E10.5AGM	0nM	CFU-GEMM	AoE+PSCs	45nM	2.4017	0.1585	36	15.15	<.0001	0.05	2.0802	2.7231
BFU-E	E10.5AGM	0nM	CFU-GEMM	E10.5AGM	0nM	0.1081	0.05961	36	1.81	0.0781	0.05	-0.01279	0.2290
BFU-E	E10.5AGM	0nM	CFU-GEMM	E10.5AGM	15nM	0.3855	0.09526	36	4.05	0.0003	0.05	0.1923	0.5787
BFU-E	E10.5AGM	0nM	CFU-GEMM	E10.5AGM	45nM	1.7650	0.1281	36	13.78	<.0001	0.05	1.5052	2.0248
BFU-E	E10.5AGM	0nM	CFU-GEMM	HE+PSCs	0nM	0.8156	0.1020	36	8.00	<.0001	0.05	0.6088	1.0224
BFU-E	E10.5AGM	0nM	CFU-GEMM	HE+PSCs	15nM	1.5739	0.1212	36	12.99	<.0001	0.05	1.3281	1.8196
BFU-E	E10.5AGM	0nM	CFU-GEMM	HE+PSCs	45nM	1.9146	0.1341	36	14.28	<.0001	0.05	1.6426	2.1866
BFU-E	E10.5AGM	0nM	CFU-GM	AoE+PSCs	0nM	-0.9052	0.08535	36	-10.61	<.0001	0.05	-1.0783	-0.7321
BFU-E	E10.5AGM	0nM	CFU-GM	AoE+PSCs	15nM	-0.1041	0.09012	36	-1.16	0.2557	0.05	-0.2869	0.07868
BFU-E	E10.5AGM	0nM	CFU-GM	AoE+PSCs	45nM	0.6381	0.09893	36	6.45	<.0001	0.05	0.4374	0.8387
BFU-E	E10.5AGM	0nM	CFU-GM	E10.5AGM	0nM	-1.3500	0.04601	36	-29.34	<.0001	0.05	-1.4433	-1.2567
BFU-E	E10.5AGM	0nM	CFU-GM	E10.5AGM	15nM	-0.6977	0.08627	36	-8.09	<.0001	0.05	-0.8726	-0.5227
BFU-E	E10.5AGM	0nM	CFU-GM	E10.5AGM	45nM	-0.01031	0.09094	36	-0.11	0.9104	0.05	-0.1947	0.1741
BFU-E	E10.5AGM	0nM	CFU-GM	HE+PSCs	0nM	-0.8174	0.08572	36	-9.54	<.0001	0.05	-0.9912	-0.6435

The GLIMMIX Procedure

Differences of Colony*Condit*Treatm Least Squares Means													
Colony_Type	Condition	Treatment	_Colony_Type	_Condition	_Treatment	Estimate	Standard Error	DF	t Value	Pr > |t|	Alpha	Lower	Upper
BFU-E	E10.5AGM	0nM	CFU-GM	HE+PSCs	15nM	0.03091	0.09134	36	0.34	0.7370	0.05	-0.1543	0.2162
BFU-E	E10.5AGM	0nM	CFU-GM	HE+PSCs	45nM	0.8823	0.1033	36	8.54	<.0001	0.05	0.6729	1.0917
BFU-E	E10.5AGM	15nM	BFU-E	E10.5AGM	45nM	1.3815	0.1356	36	10.19	<.0001	0.05	1.1065	1.6564
BFU-E	E10.5AGM	15nM	BFU-E	HE+PSCs	0nM	0.2527	0.1054	36	2.40	0.0218	0.05	0.03896	0.4664
BFU-E	E10.5AGM	15nM	BFU-E	HE+PSCs	15nM	1.3539	0.1344	36	10.08	<.0001	0.05	1.0813	1.6264
BFU-E	E10.5AGM	15nM	BFU-E	HE+PSCs	45nM	2.1094	0.1729	36	12.20	<.0001	0.05	1.7588	2.4600
BFU-E	E10.5AGM	15nM	CFU-GEMM	AoE+PSCs	0nM	0.1881	0.1044	36	1.80	0.0800	0.05	-0.02363	0.3997
BFU-E	E10.5AGM	15nM	CFU-GEMM	AoE+PSCs	15nM	1.0490	0.1238	36	8.47	<.0001	0.05	0.7979	1.3001
BFU-E	E10.5AGM	15nM	CFU-GEMM	AoE+PSCs	45nM	1.9257	0.1617	36	11.91	<.0001	0.05	1.5977	2.2536
BFU-E	E10.5AGM	15nM	CFU-GEMM	E10.5AGM	0nM	-0.3678	0.09747	36	-3.77	0.0006	0.05	-0.5655	-0.1702
BFU-E	E10.5AGM	15nM	CFU-GEMM	E10.5AGM	15nM	-0.09045	0.07191	36	-1.26	0.2165	0.05	-0.2363	0.05538
BFU-E	E10.5AGM	15nM	CFU-GEMM	E10.5AGM	45nM	1.2890	0.1320	36	9.76	<.0001	0.05	1.0212	1.5568
BFU-E	E10.5AGM	15nM	CFU-GEMM	HE+PSCs	0nM	0.3396	0.1069	36	3.18	0.0030	0.05	0.1229	0.5563
BFU-E	E10.5AGM	15nM	CFU-GEMM	HE+PSCs	15nM	1.0979	0.1253	36	8.76	<.0001	0.05	0.8438	1.3521
BFU-E	E10.5AGM	15nM	CFU-GEMM	HE+PSCs	45nM	1.4386	0.1379	36	10.43	<.0001	0.05	1.1590	1.7183
BFU-E	E10.5AGM	15nM	CFU-GM	AoE+PSCs	0nM	-1.3811	0.09115	36	-15.15	<.0001	0.05	-1.5660	-1.1963
BFU-E	E10.5AGM	15nM	CFU-GM	AoE+PSCs	15nM	-0.5801	0.09562	36	-6.07	<.0001	0.05	-0.7740	-0.3861
BFU-E	E10.5AGM	15nM	CFU-GM	AoE+PSCs	45nM	0.1621	0.1040	36	1.56	0.1277	0.05	-0.04875	0.3730
BFU-E	E10.5AGM	15nM	CFU-GM	E10.5AGM	0nM	-1.8260	0.08980	36	-20.33	<.0001	0.05	-2.0081	-1.6439
BFU-E	E10.5AGM	15nM	CFU-GM	E10.5AGM	15nM	-1.1737	0.05948	36	-19.73	<.0001	0.05	-1.2943	-1.0530
BFU-E	E10.5AGM	15nM	CFU-GM	E10.5AGM	45nM	-0.4863	0.09640	36	-5.04	<.0001	0.05	-0.6818	-0.2908
BFU-E	E10.5AGM	15nM	CFU-GM	HE+PSCs	0nM	-1.2933	0.09149	36	-14.14	<.0001	0.05	-1.4789	-1.1078
BFU-E	E10.5AGM	15nM	CFU-GM	HE+PSCs	15nM	-0.4451	0.09677	36	-4.60	<.0001	0.05	-0.6413	-0.2488
BFU-E	E10.5AGM	15nM	CFU-GM	HE+PSCs	45nM	0.4063	0.1081	36	3.76	0.0006	0.05	0.1871	0.6256

The GLIMMIX Procedure

Differences of Colony*Condit*Treatm Least Squares Means													
Colony_Type	Condition	Treatment	_Colony_Type	_Condition	_Treatment	Estimate	Standard Error	DF	t Value	Pr > |t|	Alpha	Lower	Upper
BFU-E	E10.5AGM	45nM	BFU-E	HE+PSCs	0nM	-1.1288	0.1384	36	-8.16	<.0001	0.05	-1.4095	-0.8481
BFU-E	E10.5AGM	45nM	BFU-E	HE+PSCs	15nM	-0.02758	0.1616	36	-0.17	0.8654	0.05	-0.3553	0.3001
BFU-E	E10.5AGM	45nM	BFU-E	HE+PSCs	45nM	0.7279	0.1948	36	3.74	0.0006	0.05	0.3329	1.1229
BFU-E	E10.5AGM	45nM	CFU-GEMM	AoE+PSCs	0nM	-1.1934	0.1376	36	-8.67	<.0001	0.05	-1.4726	-0.9142
BFU-E	E10.5AGM	45nM	CFU-GEMM	AoE+PSCs	15nM	-0.3325	0.1529	36	-2.17	0.0363	0.05	-0.6426	-0.02236
BFU-E	E10.5AGM	45nM	CFU-GEMM	AoE+PSCs	45nM	0.5442	0.1849	36	2.94	0.0057	0.05	0.1692	0.9193
BFU-E	E10.5AGM	45nM	CFU-GEMM	E10.5AGM	0nM	-1.7493	0.1325	36	-13.20	<.0001	0.05	-2.0180	-1.4806
BFU-E	E10.5AGM	45nM	CFU-GEMM	E10.5AGM	15nM	-1.4719	0.1347	36	-10.93	<.0001	0.05	-1.7451	-1.1987
BFU-E	E10.5AGM	45nM	CFU-GEMM	E10.5AGM	45nM	-0.09243	0.1434	36	-0.64	0.5232	0.05	-0.3832	0.1983
BFU-E	E10.5AGM	45nM	CFU-GEMM	HE+PSCs	0nM	-1.0418	0.1395	36	-7.47	<.0001	0.05	-1.3248	-0.7588
BFU-E	E10.5AGM	45nM	CFU-GEMM	HE+PSCs	15nM	-0.2835	0.1541	36	-1.84	0.0741	0.05	-0.5961	0.02906
BFU-E	E10.5AGM	45nM	CFU-GEMM	HE+PSCs	45nM	0.05719	0.1645	36	0.35	0.7301	0.05	-0.2764	0.3908
BFU-E	E10.5AGM	45nM	CFU-GM	AoE+PSCs	0nM	-2.7626	0.1279	36	-21.60	<.0001	0.05	-3.0220	-2.5032
BFU-E	E10.5AGM	45nM	CFU-GM	AoE+PSCs	15nM	-1.9615	0.1311	36	-14.96	<.0001	0.05	-2.2274	-1.6956
BFU-E	E10.5AGM	45nM	CFU-GM	AoE+PSCs	45nM	-1.2193	0.1373	36	-8.88	<.0001	0.05	-1.4979	-0.9408
BFU-E	E10.5AGM	45nM	CFU-GM	E10.5AGM	0nM	-3.2075	0.1269	36	-25.27	<.0001	0.05	-3.4649	-2.9500
BFU-E	E10.5AGM	45nM	CFU-GM	E10.5AGM	15nM	-2.5551	0.1285	36	-19.88	<.0001	0.05	-2.8157	-2.2945
BFU-E	E10.5AGM	45nM	CFU-GM	E10.5AGM	45nM	-1.8677	0.1114	36	-16.76	<.0001	0.05	-2.0937	-1.6418
BFU-E	E10.5AGM	45nM	CFU-GM	HE+PSCs	0nM	-2.6748	0.1281	36	-20.87	<.0001	0.05	-2.9347	-2.4149
BFU-E	E10.5AGM	45nM	CFU-GM	HE+PSCs	15nM	-1.8265	0.1320	36	-13.84	<.0001	0.05	-2.0941	-1.5589
BFU-E	E10.5AGM	45nM	CFU-GM	HE+PSCs	45nM	-0.9751	0.1405	36	-6.94	<.0001	0.05	-1.2600	-0.6902
BFU-E	HE+PSCs	0nM	BFU-E	HE+PSCs	15nM	1.1012	0.1372	36	8.03	<.0001	0.05	0.8229	1.3795
BFU-E	HE+PSCs	0nM	BFU-E	HE+PSCs	45nM	1.8567	0.1751	36	10.60	<.0001	0.05	1.5016	2.2118
BFU-E	HE+PSCs	0nM	CFU-GEMM	AoE+PSCs	0nM	-0.06461	0.1080	36	-0.60	0.5535	0.05	-0.2837	0.1545

The GLIMMIX Procedure

Differences of Colony*Condit*Treatm Least Squares Means													
Colony_Type	Condition	Treatment	_Colony_Type	_Condition	_Treatment	Estimate	Standard Error	DF	t Value	Pr > |t|	Alpha	Lower	Upper
BFU-E	HE+PSCs	0nM	CFU-GEMM	AoE+PSCs	15nM	0.7963	0.1269	36	6.28	<.0001	0.05	0.5390	1.0537
BFU-E	HE+PSCs	0nM	CFU-GEMM	AoE+PSCs	45nM	1.6730	0.1641	36	10.20	<.0001	0.05	1.3403	2.0058
BFU-E	HE+PSCs	0nM	CFU-GEMM	E10.5AGM	0nM	-0.6205	0.1014	36	-6.12	<.0001	0.05	-0.8261	-0.4150
BFU-E	HE+PSCs	0nM	CFU-GEMM	E10.5AGM	15nM	-0.3431	0.1043	36	-3.29	0.0022	0.05	-0.5546	-0.1317
BFU-E	HE+PSCs	0nM	CFU-GEMM	E10.5AGM	45nM	1.0364	0.1349	36	7.68	<.0001	0.05	0.7627	1.3100
BFU-E	HE+PSCs	0nM	CFU-GEMM	HE+PSCs	0nM	0.08695	0.08522	36	1.02	0.3144	0.05	-0.08587	0.2598
BFU-E	HE+PSCs	0nM	CFU-GEMM	HE+PSCs	15nM	0.8453	0.1284	36	6.58	<.0001	0.05	0.5849	1.1056
BFU-E	HE+PSCs	0nM	CFU-GEMM	HE+PSCs	45nM	1.1860	0.1406	36	8.43	<.0001	0.05	0.9007	1.4712
BFU-E	HE+PSCs	0nM	CFU-GM	AoE+PSCs	0nM	-1.6338	0.09529	36	-17.15	<.0001	0.05	-1.8271	-1.4405
BFU-E	HE+PSCs	0nM	CFU-GM	AoE+PSCs	15nM	-0.8327	0.09958	36	-8.36	<.0001	0.05	-1.0347	-0.6308
BFU-E	HE+PSCs	0nM	CFU-GM	AoE+PSCs	45nM	-0.09056	0.1076	36	-0.84	0.4056	0.05	-0.3088	0.1277
BFU-E	HE+PSCs	0nM	CFU-GM	E10.5AGM	0nM	-2.0787	0.09400	36	-22.11	<.0001	0.05	-2.2693	-1.8880
BFU-E	HE+PSCs	0nM	CFU-GM	E10.5AGM	15nM	-1.4263	0.09611	36	-14.84	<.0001	0.05	-1.6212	-1.2314
BFU-E	HE+PSCs	0nM	CFU-GM	E10.5AGM	45nM	-0.7389	0.1003	36	-7.37	<.0001	0.05	-0.9424	-0.5355
BFU-E	HE+PSCs	0nM	CFU-GM	HE+PSCs	0nM	-1.5460	0.06491	36	-23.82	<.0001	0.05	-1.6776	-1.4144
BFU-E	HE+PSCs	0nM	CFU-GM	HE+PSCs	15nM	-0.6977	0.1007	36	-6.93	<.0001	0.05	-0.9019	-0.4935
BFU-E	HE+PSCs	0nM	CFU-GM	HE+PSCs	45nM	0.1537	0.1116	36	1.38	0.1771	0.05	-0.07269	0.3800
BFU-E	HE+PSCs	15nM	BFU-E	HE+PSCs	45nM	0.7555	0.1939	36	3.90	0.0004	0.05	0.3622	1.1488
BFU-E	HE+PSCs	15nM	CFU-GEMM	AoE+PSCs	0nM	-1.1658	0.1365	36	-8.54	<.0001	0.05	-1.4426	-0.8891
BFU-E	HE+PSCs	15nM	CFU-GEMM	AoE+PSCs	15nM	-0.3049	0.1518	36	-2.01	0.0522	0.05	-0.6128	0.003031
BFU-E	HE+PSCs	15nM	CFU-GEMM	AoE+PSCs	45nM	0.5718	0.1840	36	3.11	0.0037	0.05	0.1986	0.9451
BFU-E	HE+PSCs	15nM	CFU-GEMM	E10.5AGM	0nM	-1.7217	0.1312	36	-13.12	<.0001	0.05	-1.9879	-1.4555
BFU-E	HE+PSCs	15nM	CFU-GEMM	E10.5AGM	15nM	-1.4443	0.1335	36	-10.82	<.0001	0.05	-1.7151	-1.1736
BFU-E	HE+PSCs	15nM	CFU-GEMM	E10.5AGM	45nM	-0.06485	0.1586	36	-0.41	0.6851	0.05	-0.3865	0.2568

The GLIMMIX Procedure

Differences of Colony*Condit*Treatm Least Squares Means													
Colony_Type	Condition	Treatment	_Colony_Type	_Condition	_Treatment	Estimate	Standard Error	DF	t Value	Pr > |t|	Alpha	Lower	Upper
BFU-E	HE+PSCs	15nM	CFU-GEMM	HE+PSCs	0nM	-1.0142	0.1384	36	-7.33	<.0001	0.05	-1.2949	-0.7336
BFU-E	HE+PSCs	15nM	CFU-GEMM	HE+PSCs	15nM	-0.2559	0.1359	36	-1.88	0.0678	0.05	-0.5316	0.01974
BFU-E	HE+PSCs	15nM	CFU-GEMM	HE+PSCs	45nM	0.08477	0.1635	36	0.52	0.6073	0.05	-0.2468	0.4164
BFU-E	HE+PSCs	15nM	CFU-GM	AoE+PSCs	0nM	-2.7350	0.1266	36	-21.60	<.0001	0.05	-2.9918	-2.4782
BFU-E	HE+PSCs	15nM	CFU-GM	AoE+PSCs	15nM	-1.9339	0.1299	36	-14.89	<.0001	0.05	-2.1973	-1.6705
BFU-E	HE+PSCs	15nM	CFU-GM	AoE+PSCs	45nM	-1.1918	0.1361	36	-8.75	<.0001	0.05	-1.4679	-0.9157
BFU-E	HE+PSCs	15nM	CFU-GM	E10.5AGM	0nM	-3.1799	0.1257	36	-25.31	<.0001	0.05	-3.4347	-2.9250
BFU-E	HE+PSCs	15nM	CFU-GM	E10.5AGM	15nM	-2.5275	0.1272	36	-19.86	<.0001	0.05	-2.7856	-2.2695
BFU-E	HE+PSCs	15nM	CFU-GM	E10.5AGM	45nM	-1.8401	0.1305	36	-14.11	<.0001	0.05	-2.1047	-1.5756
BFU-E	HE+PSCs	15nM	CFU-GM	HE+PSCs	0nM	-2.6472	0.1269	36	-20.87	<.0001	0.05	-2.9045	-2.3899
BFU-E	HE+PSCs	15nM	CFU-GM	HE+PSCs	15nM	-1.7989	0.1102	36	-16.33	<.0001	0.05	-2.0224	-1.5755
BFU-E	HE+PSCs	15nM	CFU-GM	HE+PSCs	45nM	-0.9475	0.1393	36	-6.80	<.0001	0.05	-1.2301	-0.6650
BFU-E	HE+PSCs	45nM	CFU-GEMM	AoE+PSCs	0nM	-1.9213	0.1745	36	-11.01	<.0001	0.05	-2.2752	-1.5674
BFU-E	HE+PSCs	45nM	CFU-GEMM	AoE+PSCs	15nM	-1.0604	0.1868	36	-5.68	<.0001	0.05	-1.4391	-0.6816
BFU-E	HE+PSCs	45nM	CFU-GEMM	AoE+PSCs	45nM	-0.1837	0.2138	36	-0.86	0.3959	0.05	-0.6172	0.2499
BFU-E	HE+PSCs	45nM	CFU-GEMM	E10.5AGM	0nM	-2.4772	0.1704	36	-14.53	<.0001	0.05	-2.8229	-2.1315
BFU-E	HE+PSCs	45nM	CFU-GEMM	E10.5AGM	15nM	-2.1998	0.1722	36	-12.78	<.0001	0.05	-2.5490	-1.8506
BFU-E	HE+PSCs	45nM	CFU-GEMM	E10.5AGM	45nM	-0.8203	0.1923	36	-4.27	0.0001	0.05	-1.2104	-0.4303
BFU-E	HE+PSCs	45nM	CFU-GEMM	HE+PSCs	0nM	-1.7697	0.1760	36	-10.06	<.0001	0.05	-2.1267	-1.4128
BFU-E	HE+PSCs	45nM	CFU-GEMM	HE+PSCs	15nM	-1.0114	0.1878	36	-5.39	<.0001	0.05	-1.3922	-0.6306
BFU-E	HE+PSCs	45nM	CFU-GEMM	HE+PSCs	45nM	-0.6707	0.1834	36	-3.66	0.0008	0.05	-1.0426	-0.2988
BFU-E	HE+PSCs	45nM	CFU-GM	AoE+PSCs	0nM	-3.4905	0.1669	36	-20.91	<.0001	0.05	-3.8290	-3.1520
BFU-E	HE+PSCs	45nM	CFU-GM	AoE+PSCs	15nM	-2.6894	0.1694	36	-15.88	<.0001	0.05	-3.0330	-2.3459
BFU-E	HE+PSCs	45nM	CFU-GM	AoE+PSCs	45nM	-1.9472	0.1742	36	-11.18	<.0001	0.05	-2.3006	-1.5939

The GLIMMIX Procedure

Differences of Colony*Condit*Treatm Least Squares Means													
Colony_Type	Condition	Treatment	_Colony_Type	_Condition	_Treatment	Estimate	Standard Error	DF	t Value	Pr > |t|	Alpha	Lower	Upper
BFU-E	HE+PSCs	45nM	CFU-GM	E10.5AGM	0nM	-3.9354	0.1662	36	-23.68	<.0001	0.05	-4.2724	-3.5983
BFU-E	HE+PSCs	45nM	CFU-GM	E10.5AGM	15nM	-3.2830	0.1674	36	-19.61	<.0001	0.05	-3.6225	-2.9435
BFU-E	HE+PSCs	45nM	CFU-GM	E10.5AGM	45nM	-2.5956	0.1698	36	-15.28	<.0001	0.05	-2.9401	-2.2512
BFU-E	HE+PSCs	45nM	CFU-GM	HE+PSCs	0nM	-3.4027	0.1671	36	-20.36	<.0001	0.05	-3.7416	-3.0638
BFU-E	HE+PSCs	45nM	CFU-GM	HE+PSCs	15nM	-2.5544	0.1700	36	-15.02	<.0001	0.05	-2.8993	-2.2096
BFU-E	HE+PSCs	45nM	CFU-GM	HE+PSCs	45nM	-1.7030	0.1622	36	-10.50	<.0001	0.05	-2.0319	-1.3741
CFU-GEMM	AoE+PSCs	0nM	CFU-GEMM	AoE+PSCs	15nM	0.8609	0.1261	36	6.83	<.0001	0.05	0.6053	1.1166
CFU-GEMM	AoE+PSCs	0nM	CFU-GEMM	AoE+PSCs	45nM	1.7376	0.1634	36	10.63	<.0001	0.05	1.4062	2.0691
CFU-GEMM	AoE+PSCs	0nM	CFU-GEMM	E10.5AGM	0nM	-0.5559	0.1003	36	-5.54	<.0001	0.05	-0.7594	-0.3524
CFU-GEMM	AoE+PSCs	0nM	CFU-GEMM	E10.5AGM	15nM	-0.2785	0.1033	36	-2.70	0.0106	0.05	-0.4879	-0.06911
CFU-GEMM	AoE+PSCs	0nM	CFU-GEMM	E10.5AGM	45nM	1.1010	0.1342	36	8.21	<.0001	0.05	0.8289	1.3730
CFU-GEMM	AoE+PSCs	0nM	CFU-GEMM	HE+PSCs	0nM	0.1516	0.1095	36	1.38	0.1747	0.05	-0.07045	0.3736
CFU-GEMM	AoE+PSCs	0nM	CFU-GEMM	HE+PSCs	15nM	0.9099	0.1276	36	7.13	<.0001	0.05	0.6512	1.1685
CFU-GEMM	AoE+PSCs	0nM	CFU-GEMM	HE+PSCs	45nM	1.2506	0.1399	36	8.94	<.0001	0.05	0.9668	1.5343
CFU-GEMM	AoE+PSCs	0nM	CFU-GM	AoE+PSCs	0nM	-1.5692	0.06283	36	-24.98	<.0001	0.05	-1.6966	-1.4418
CFU-GEMM	AoE+PSCs	0nM	CFU-GM	AoE+PSCs	15nM	-0.7681	0.09853	36	-7.80	<.0001	0.05	-0.9679	-0.5683
CFU-GEMM	AoE+PSCs	0nM	CFU-GM	AoE+PSCs	45nM	-0.02595	0.1066	36	-0.24	0.8091	0.05	-0.2422	0.1903
CFU-GEMM	AoE+PSCs	0nM	CFU-GM	E10.5AGM	0nM	-2.0141	0.09288	36	-21.68	<.0001	0.05	-2.2024	-1.8257
CFU-GEMM	AoE+PSCs	0nM	CFU-GM	E10.5AGM	15nM	-1.3617	0.09502	36	-14.33	<.0001	0.05	-1.5544	-1.1690
CFU-GEMM	AoE+PSCs	0nM	CFU-GM	E10.5AGM	45nM	-0.6743	0.09928	36	-6.79	<.0001	0.05	-0.8757	-0.4730
CFU-GEMM	AoE+PSCs	0nM	CFU-GM	HE+PSCs	0nM	-1.4814	0.09452	36	-15.67	<.0001	0.05	-1.6731	-1.2897
CFU-GEMM	AoE+PSCs	0nM	CFU-GM	HE+PSCs	15nM	-0.6331	0.09965	36	-6.35	<.0001	0.05	-0.8352	-0.4310
CFU-GEMM	AoE+PSCs	0nM	CFU-GM	HE+PSCs	45nM	0.2183	0.1107	36	1.97	0.0563	0.05	-0.00618	0.4427
CFU-GEMM	AoE+PSCs	15nM	CFU-GEMM	AoE+PSCs	45nM	0.8767	0.1765	36	4.97	<.0001	0.05	0.5188	1.2346

The GLIMMIX Procedure

Differences of Colony*Condit*Treatm Least Squares Means

Colony_Type	Condition	Treatment	_Colony_Type	_Condition	_Treatment	Estimate	Standard Error	DF	t Value	Pr > |t|	Alpha	Lower	Upper
CFU-GEMM	AoE+PSCs	15nM	CFU-GEMM	E10.5AGM	0nM	-1.4168	0.1204	36	-11.77	<.0001	0.05	-1.6610	-1.1726
CFU-GEMM	AoE+PSCs	15nM	CFU-GEMM	E10.5AGM	15nM	-1.1394	0.1229	36	-9.27	<.0001	0.05	-1.3886	-0.8903
CFU-GEMM	AoE+PSCs	15nM	CFU-GEMM	E10.5AGM	45nM	0.2400	0.1498	36	1.60	0.1177	0.05	-0.06371	0.5438
CFU-GEMM	AoE+PSCs	15nM	CFU-GEMM	HE+PSCs	0nM	-0.7094	0.1281	36	-5.54	<.0001	0.05	-0.9692	-0.4495
CFU-GEMM	AoE+PSCs	15nM	CFU-GEMM	HE+PSCs	15nM	0.04893	0.1439	36	0.34	0.7358	0.05	-0.2428	0.3407
CFU-GEMM	AoE+PSCs	15nM	CFU-GEMM	HE+PSCs	45nM	0.3896	0.1549	36	2.51	0.0165	0.05	0.07543	0.7039
CFU-GEMM	AoE+PSCs	15nM	CFU-GM	AoE+PSCs	0nM	-2.4301	0.1153	36	-21.07	<.0001	0.05	-2.6641	-2.1962
CFU-GEMM	AoE+PSCs	15nM	CFU-GM	AoE+PSCs	15nM	-1.6290	0.09592	36	-16.98	<.0001	0.05	-1.8236	-1.4345
CFU-GEMM	AoE+PSCs	15nM	CFU-GM	AoE+PSCs	45nM	-0.8869	0.1257	36	-7.05	<.0001	0.05	-1.1418	-0.6319
CFU-GEMM	AoE+PSCs	15nM	CFU-GM	E10.5AGM	0nM	-2.8750	0.1143	36	-25.16	<.0001	0.05	-3.1068	-2.6432
CFU-GEMM	AoE+PSCs	15nM	CFU-GM	E10.5AGM	15nM	-2.2226	0.1160	36	-19.16	<.0001	0.05	-2.4579	-1.9873
CFU-GEMM	AoE+PSCs	15nM	CFU-GM	E10.5AGM	45nM	-1.5353	0.1195	36	-12.84	<.0001	0.05	-1.7777	-1.2928
CFU-GEMM	AoE+PSCs	15nM	CFU-GM	HE+PSCs	0nM	-2.3423	0.1156	36	-20.26	<.0001	0.05	-2.5768	-2.1078
CFU-GEMM	AoE+PSCs	15nM	CFU-GM	HE+PSCs	15nM	-1.4940	0.1198	36	-12.47	<.0001	0.05	-1.7371	-1.2510
CFU-GEMM	AoE+PSCs	15nM	CFU-GM	HE+PSCs	45nM	-0.6427	0.1291	36	-4.98	<.0001	0.05	-0.9046	-0.3807
CFU-GEMM	AoE+PSCs	45nM	CFU-GEMM	E10.5AGM	0nM	-2.2935	0.1591	36	-14.41	<.0001	0.05	-2.6162	-1.9708
CFU-GEMM	AoE+PSCs	45nM	CFU-GEMM	E10.5AGM	15nM	-2.0161	0.1610	36	-12.52	<.0001	0.05	-2.3426	-1.6897
CFU-GEMM	AoE+PSCs	45nM	CFU-GEMM	E10.5AGM	45nM	-0.6367	0.1823	36	-3.49	0.0013	0.05	-1.0065	-0.2669
CFU-GEMM	AoE+PSCs	45nM	CFU-GEMM	HE+PSCs	0nM	-1.5861	0.1650	36	-9.61	<.0001	0.05	-1.9208	-1.2514
CFU-GEMM	AoE+PSCs	45nM	CFU-GEMM	HE+PSCs	15nM	-0.8278	0.1775	36	-4.66	<.0001	0.05	-1.1878	-0.4677
CFU-GEMM	AoE+PSCs	45nM	CFU-GEMM	HE+PSCs	45nM	-0.4870	0.1866	36	-2.61	0.0131	0.05	-0.8655	-0.1086
CFU-GEMM	AoE+PSCs	45nM	CFU-GM	AoE+PSCs	0nM	-3.3068	0.1553	36	-21.29	<.0001	0.05	-3.6218	-2.9918
CFU-GEMM	AoE+PSCs	45nM	CFU-GM	AoE+PSCs	15nM	-2.5057	0.1580	36	-15.86	<.0001	0.05	-2.8261	-2.1853
CFU-GEMM	AoE+PSCs	45nM	CFU-GM	AoE+PSCs	45nM	-1.7636	0.1473	36	-11.98	<.0001	0.05	-2.0622	-1.4649

The GLIMMIX Procedure

Differences of Colony*Condit*Treatm Least Squares Means													
Colony_Type	Condition	Treatment	_Colony_Type	_Condition	_Treatment	Estimate	Standard Error	DF	t Value	Pr > |t|	Alpha	Lower	Upper
CFU-GEMM	AoE+PSCs	45nM	CFU-GM	E10.5AGM	0nM	-3.7517	0.1545	36	-24.28	<.0001	0.05	-4.0651	-3.4383
CFU-GEMM	AoE+PSCs	45nM	CFU-GM	E10.5AGM	15nM	-3.0993	0.1558	36	-19.89	<.0001	0.05	-3.4154	-2.7833
CFU-GEMM	AoE+PSCs	45nM	CFU-GM	E10.5AGM	45nM	-2.4120	0.1585	36	-15.22	<.0001	0.05	-2.7333	-2.0906
CFU-GEMM	AoE+PSCs	45nM	CFU-GM	HE+PSCs	0nM	-3.2190	0.1555	36	-20.70	<.0001	0.05	-3.5344	-2.9036
CFU-GEMM	AoE+PSCs	45nM	CFU-GM	HE+PSCs	15nM	-2.3707	0.1587	36	-14.94	<.0001	0.05	-2.6926	-2.0489
CFU-GEMM	AoE+PSCs	45nM	CFU-GM	HE+PSCs	45nM	-1.5194	0.1658	36	-9.16	<.0001	0.05	-1.8557	-1.1830
CFU-GEMM	E10.5AGM	0nM	CFU-GEMM	E10.5AGM	15nM	0.2774	0.09626	36	2.88	0.0066	0.05	0.08216	0.4726
CFU-GEMM	E10.5AGM	0nM	CFU-GEMM	E10.5AGM	45nM	1.6569	0.1289	36	12.86	<.0001	0.05	1.3955	1.9182
CFU-GEMM	E10.5AGM	0nM	CFU-GEMM	HE+PSCs	0nM	0.7075	0.1029	36	6.88	<.0001	0.05	0.4988	0.9162
CFU-GEMM	E10.5AGM	0nM	CFU-GEMM	HE+PSCs	15nM	1.4658	0.1220	36	12.02	<.0001	0.05	1.2184	1.7131
CFU-GEMM	E10.5AGM	0nM	CFU-GEMM	HE+PSCs	45nM	1.8065	0.1348	36	13.40	<.0001	0.05	1.5330	2.0799
CFU-GEMM	E10.5AGM	0nM	CFU-GM	AoE+PSCs	0nM	-1.0133	0.08647	36	-11.72	<.0001	0.05	-1.1887	-0.8379
CFU-GEMM	E10.5AGM	0nM	CFU-GM	AoE+PSCs	15nM	-0.2122	0.09118	36	-2.33	0.0257	0.05	-0.3971	-0.02729
CFU-GEMM	E10.5AGM	0nM	CFU-GM	AoE+PSCs	45nM	0.5300	0.09990	36	5.31	<.0001	0.05	0.3274	0.7326
CFU-GEMM	E10.5AGM	0nM	CFU-GM	E10.5AGM	0nM	-1.4582	0.04805	36	-30.35	<.0001	0.05	-1.5556	-1.3607
CFU-GEMM	E10.5AGM	0nM	CFU-GM	E10.5AGM	15nM	-0.8058	0.08737	36	-9.22	<.0001	0.05	-0.9830	-0.6286
CFU-GEMM	E10.5AGM	0nM	CFU-GM	E10.5AGM	45nM	-0.1184	0.09199	36	-1.29	0.2062	0.05	-0.3050	0.06815
CFU-GEMM	E10.5AGM	0nM	CFU-GM	HE+PSCs	0nM	-0.9255	0.08683	36	-10.66	<.0001	0.05	-1.1016	-0.7494
CFU-GEMM	E10.5AGM	0nM	CFU-GM	HE+PSCs	15nM	-0.07721	0.09238	36	-0.84	0.4088	0.05	-0.2646	0.1102
CFU-GEMM	E10.5AGM	0nM	CFU-GM	HE+PSCs	45nM	0.7742	0.1042	36	7.43	<.0001	0.05	0.5629	0.9855
CFU-GEMM	E10.5AGM	15nM	CFU-GEMM	E10.5AGM	45nM	1.3795	0.1311	36	10.52	<.0001	0.05	1.1135	1.6455
CFU-GEMM	E10.5AGM	15nM	CFU-GEMM	HE+PSCs	0nM	0.4301	0.1058	36	4.07	0.0002	0.05	0.2156	0.6446
CFU-GEMM	E10.5AGM	15nM	CFU-GEMM	HE+PSCs	15nM	1.1884	0.1244	36	9.55	<.0001	0.05	0.9361	1.4406
CFU-GEMM	E10.5AGM	15nM	CFU-GEMM	HE+PSCs	45nM	1.5291	0.1370	36	11.16	<.0001	0.05	1.2512	1.8070

The GLIMMIX Procedure

Differences of Colony*Condit*Treatm Least Squares Means													
Colony_Type	Condition	Treatment	_Colony_Type	_Condition	_Treatment	Estimate	Standard Error	DF	t Value	Pr > |t|	Alpha	Lower	Upper
CFU-GEMM	E10.5AGM	15nM	CFU-GM	AoE+PSCs	0nM	-1.2907	0.08986	36	-14.36	<.0001	0.05	-1.4729	-1.1084
CFU-GEMM	E10.5AGM	15nM	CFU-GM	AoE+PSCs	15nM	-0.4896	0.09439	36	-5.19	<.0001	0.05	-0.6810	-0.2982
CFU-GEMM	E10.5AGM	15nM	CFU-GM	AoE+PSCs	45nM	0.2526	0.1028	36	2.46	0.0190	0.05	0.04400	0.4611
CFU-GEMM	E10.5AGM	15nM	CFU-GM	E10.5AGM	0nM	-1.7355	0.08849	36	-19.61	<.0001	0.05	-1.9150	-1.5561
CFU-GEMM	E10.5AGM	15nM	CFU-GM	E10.5AGM	15nM	-1.0832	0.05748	36	-18.84	<.0001	0.05	-1.1998	-0.9666
CFU-GEMM	E10.5AGM	15nM	CFU-GM	E10.5AGM	45nM	-0.3958	0.09518	36	-4.16	0.0002	0.05	-0.5888	-0.2028
CFU-GEMM	E10.5AGM	15nM	CFU-GM	HE+PSCs	0nM	-1.2029	0.09020	36	-13.33	<.0001	0.05	-1.3858	-1.0199
CFU-GEMM	E10.5AGM	15nM	CFU-GM	HE+PSCs	15nM	-0.3546	0.09556	36	-3.71	0.0007	0.05	-0.5484	-0.1608
CFU-GEMM	E10.5AGM	15nM	CFU-GM	HE+PSCs	45nM	0.4968	0.1070	36	4.64	<.0001	0.05	0.2798	0.7138
CFU-GEMM	E10.5AGM	45nM	CFU-GEMM	HE+PSCs	0nM	-0.9494	0.1361	36	-6.98	<.0001	0.05	-1.2254	-0.6734
CFU-GEMM	E10.5AGM	45nM	CFU-GEMM	HE+PSCs	15nM	-0.1911	0.1510	36	-1.27	0.2139	0.05	-0.4974	0.1152
CFU-GEMM	E10.5AGM	45nM	CFU-GEMM	HE+PSCs	45nM	0.1496	0.1616	36	0.93	0.3607	0.05	-0.1781	0.4773
CFU-GEMM	E10.5AGM	45nM	CFU-GM	AoE+PSCs	0nM	-2.6702	0.1241	36	-21.51	<.0001	0.05	-2.9219	-2.4184
CFU-GEMM	E10.5AGM	45nM	CFU-GM	AoE+PSCs	15nM	-1.8691	0.1275	36	-14.66	<.0001	0.05	-2.1276	-1.6106
CFU-GEMM	E10.5AGM	45nM	CFU-GM	AoE+PSCs	45nM	-1.1269	0.1338	36	-8.42	<.0001	0.05	-1.3983	-0.8555
CFU-GEMM	E10.5AGM	45nM	CFU-GM	E10.5AGM	0nM	-3.1150	0.1232	36	-25.29	<.0001	0.05	-3.3648	-2.8653
CFU-GEMM	E10.5AGM	45nM	CFU-GM	E10.5AGM	15nM	-2.4627	0.1248	36	-19.74	<.0001	0.05	-2.7157	-2.2096
CFU-GEMM	E10.5AGM	45nM	CFU-GM	E10.5AGM	45nM	-1.7753	0.1071	36	-16.58	<.0001	0.05	-1.9924	-1.5582
CFU-GEMM	E10.5AGM	45nM	CFU-GM	HE+PSCs	0nM	-2.5823	0.1244	36	-20.76	<.0001	0.05	-2.8346	-2.3301
CFU-GEMM	E10.5AGM	45nM	CFU-GM	HE+PSCs	15nM	-1.7341	0.1283	36	-13.51	<.0001	0.05	-1.9943	-1.4738
CFU-GEMM	E10.5AGM	45nM	CFU-GM	HE+PSCs	45nM	-0.8827	0.1371	36	-6.44	<.0001	0.05	-1.1607	-0.6047
CFU-GEMM	HE+PSCs	0nM	CFU-GEMM	HE+PSCs	15nM	0.7583	0.1296	36	5.85	<.0001	0.05	0.4955	1.0211
CFU-GEMM	HE+PSCs	0nM	CFU-GEMM	HE+PSCs	45nM	1.0990	0.1418	36	7.75	<.0001	0.05	0.8115	1.3865
CFU-GEMM	HE+PSCs	0nM	CFU-GM	AoE+PSCs	0nM	-1.7208	0.09693	36	-17.75	<.0001	0.05	-1.9173	-1.5242

The GLIMMIX Procedure

Differences of Colony*Condit*Treatm Least Squares Means

Colony_Type	Condition	Treatment	_Colony_Type	_Condition	_Treatment	Estimate	Standard Error	DF	t Value	Pr > |t|	Alpha	Lower	Upper
CFU-GEMM	HE+PSCs	0nM	CFU-GM	AoE+PSCs	15nM	-0.9197	0.1011	36	-9.09	<.0001	0.05	-1.1248	-0.7145
CFU-GEMM	HE+PSCs	0nM	CFU-GM	AoE+PSCs	45nM	-0.1775	0.1091	36	-1.63	0.1124	0.05	-0.3987	0.04370
CFU-GEMM	HE+PSCs	0nM	CFU-GM	E10.5AGM	0nM	-2.1656	0.09566	36	-22.64	<.0001	0.05	-2.3596	-1.9716
CFU-GEMM	HE+PSCs	0nM	CFU-GM	E10.5AGM	15nM	-1.5133	0.09774	36	-15.48	<.0001	0.05	-1.7115	-1.3151
CFU-GEMM	HE+PSCs	0nM	CFU-GM	E10.5AGM	45nM	-0.8259	0.1019	36	-8.11	<.0001	0.05	-1.0325	-0.6193
CFU-GEMM	HE+PSCs	0nM	CFU-GM	HE+PSCs	0nM	-1.6329	0.06730	36	-24.27	<.0001	0.05	-1.7694	-1.4965
CFU-GEMM	HE+PSCs	0nM	CFU-GM	HE+PSCs	15nM	-0.7847	0.1022	36	-7.68	<.0001	0.05	-0.9920	-0.5773
CFU-GEMM	HE+PSCs	0nM	CFU-GM	HE+PSCs	45nM	0.06671	0.1130	36	0.59	0.5587	0.05	-0.1625	0.2959
CFU-GEMM	HE+PSCs	15nM	CFU-GEMM	HE+PSCs	45nM	0.3407	0.1561	36	2.18	0.0357	0.05	0.02404	0.6574
CFU-GEMM	HE+PSCs	15nM	CFU-GM	AoE+PSCs	0nM	-2.4791	0.1170	36	-21.19	<.0001	0.05	-2.7163	-2.2418
CFU-GEMM	HE+PSCs	15nM	CFU-GM	AoE+PSCs	15nM	-1.6780	0.1205	36	-13.93	<.0001	0.05	-1.9223	-1.4336
CFU-GEMM	HE+PSCs	15nM	CFU-GM	AoE+PSCs	45nM	-0.9358	0.1272	36	-7.36	<.0001	0.05	-1.1938	-0.6778
CFU-GEMM	HE+PSCs	15nM	CFU-GM	E10.5AGM	0nM	-2.9239	0.1159	36	-25.22	<.0001	0.05	-3.1590	-2.6888
CFU-GEMM	HE+PSCs	15nM	CFU-GM	E10.5AGM	15nM	-2.2716	0.1176	36	-19.31	<.0001	0.05	-2.5101	-2.0330
CFU-GEMM	HE+PSCs	15nM	CFU-GM	E10.5AGM	45nM	-1.5842	0.1211	36	-13.08	<.0001	0.05	-1.8298	-1.3386
CFU-GEMM	HE+PSCs	15nM	CFU-GM	HE+PSCs	0nM	-2.3913	0.1172	36	-20.40	<.0001	0.05	-2.6290	-2.1535
CFU-GEMM	HE+PSCs	15nM	CFU-GM	HE+PSCs	15nM	-1.5430	0.09893	36	-15.60	<.0001	0.05	-1.7436	-1.3423
CFU-GEMM	HE+PSCs	15nM	CFU-GM	HE+PSCs	45nM	-0.6916	0.1306	36	-5.30	<.0001	0.05	-0.9565	-0.4267
CFU-GEMM	HE+PSCs	45nM	CFU-GM	AoE+PSCs	0nM	-2.8198	0.1303	36	-21.63	<.0001	0.05	-3.0841	-2.5555
CFU-GEMM	HE+PSCs	45nM	CFU-GM	AoE+PSCs	15nM	-2.0187	0.1335	36	-15.12	<.0001	0.05	-2.2894	-1.7480
CFU-GEMM	HE+PSCs	45nM	CFU-GM	AoE+PSCs	45nM	-1.2765	0.1396	36	-9.14	<.0001	0.05	-1.5596	-0.9934
CFU-GEMM	HE+PSCs	45nM	CFU-GM	E10.5AGM	0nM	-3.2646	0.1294	36	-25.23	<.0001	0.05	-3.5271	-3.0022
CFU-GEMM	HE+PSCs	45nM	CFU-GM	E10.5AGM	15nM	-2.6123	0.1309	36	-19.95	<.0001	0.05	-2.8778	-2.3468
CFU-GEMM	HE+PSCs	45nM	CFU-GM	E10.5AGM	45nM	-1.9249	0.1341	36	-14.36	<.0001	0.05	-2.1968	-1.6530

The GLIMMIX Procedure

Differences of Colony*Condit*Treatm Least Squares Means													
Colony_Type	Condition	Treatment	_Colony_Type	_Condition	_Treatment	Estimate	Standard Error	DF	t Value	Pr > |t|	Alpha	Lower	Upper
CFU-GEMM	HE+PSCs	45nM	CFU-GM	HE+PSCs	0nM	-2.7320	0.1306	36	-20.92	<.0001	0.05	-2.9968	-2.4672
CFU-GEMM	HE+PSCs	45nM	CFU-GM	HE+PSCs	15nM	-1.8837	0.1343	36	-14.02	<.0001	0.05	-2.1561	-1.6113
CFU-GEMM	HE+PSCs	45nM	CFU-GM	HE+PSCs	45nM	-1.0323	0.1242	36	-8.31	<.0001	0.05	-1.2842	-0.7804
CFU-GM	AoE+PSCs	0nM	CFU-GM	AoE+PSCs	15nM	0.8011	0.08438	36	9.49	<.0001	0.05	0.6299	0.9722
CFU-GM	AoE+PSCs	0nM	CFU-GM	AoE+PSCs	45nM	1.5432	0.09374	36	16.46	<.0001	0.05	1.3531	1.7334
CFU-GM	AoE+PSCs	0nM	CFU-GM	E10.5AGM	0nM	-0.4449	0.07772	36	-5.72	<.0001	0.05	-0.6025	-0.2872
CFU-GM	AoE+PSCs	0nM	CFU-GM	E10.5AGM	15nM	0.2075	0.08026	36	2.59	0.0139	0.05	0.04472	0.3703
CFU-GM	AoE+PSCs	0nM	CFU-GM	E10.5AGM	45nM	0.8949	0.08526	36	10.50	<.0001	0.05	0.7220	1.0678
CFU-GM	AoE+PSCs	0nM	CFU-GM	HE+PSCs	0nM	0.08781	0.07967	36	1.10	0.2777	0.05	-0.07377	0.2494
CFU-GM	AoE+PSCs	0nM	CFU-GM	HE+PSCs	15nM	0.9361	0.08569	36	10.92	<.0001	0.05	0.7623	1.1099
CFU-GM	AoE+PSCs	0nM	CFU-GM	HE+PSCs	45nM	1.7875	0.09829	36	18.18	<.0001	0.05	1.5881	1.9868
CFU-GM	AoE+PSCs	15nM	CFU-GM	AoE+PSCs	45nM	0.7422	0.09808	36	7.57	<.0001	0.05	0.5432	0.9411
CFU-GM	AoE+PSCs	15nM	CFU-GM	E10.5AGM	0nM	-1.2459	0.08292	36	-15.03	<.0001	0.05	-1.4141	-1.0778
CFU-GM	AoE+PSCs	15nM	CFU-GM	E10.5AGM	15nM	-0.5936	0.08530	36	-6.96	<.0001	0.05	-0.7666	-0.4206
CFU-GM	AoE+PSCs	15nM	CFU-GM	E10.5AGM	45nM	0.09379	0.09003	36	1.04	0.3045	0.05	-0.08879	0.2764
CFU-GM	AoE+PSCs	15nM	CFU-GM	HE+PSCs	0nM	-0.7133	0.08475	36	-8.42	<.0001	0.05	-0.8852	-0.5414
CFU-GM	AoE+PSCs	15nM	CFU-GM	HE+PSCs	15nM	0.1350	0.09041	36	1.49	0.1441	0.05	-0.04836	0.3184
CFU-GM	AoE+PSCs	15nM	CFU-GM	HE+PSCs	45nM	0.9864	0.1024	36	9.63	<.0001	0.05	0.7786	1.1942
CFU-GM	AoE+PSCs	45nM	CFU-GM	E10.5AGM	0nM	-1.9881	0.09243	36	-21.51	<.0001	0.05	-2.1756	-1.8007
CFU-GM	AoE+PSCs	45nM	CFU-GM	E10.5AGM	15nM	-1.3358	0.09457	36	-14.12	<.0001	0.05	-1.5276	-1.1440
CFU-GM	AoE+PSCs	45nM	CFU-GM	E10.5AGM	45nM	-0.6484	0.09885	36	-6.56	<.0001	0.05	-0.8488	-0.4479
CFU-GM	AoE+PSCs	45nM	CFU-GM	HE+PSCs	0nM	-1.4554	0.09407	36	-15.47	<.0001	0.05	-1.6462	-1.2647
CFU-GM	AoE+PSCs	45nM	CFU-GM	HE+PSCs	15nM	-0.6072	0.09920	36	-6.12	<.0001	0.05	-0.8084	-0.4060
CFU-GM	AoE+PSCs	45nM	CFU-GM	HE+PSCs	45nM	0.2442	0.1103	36	2.21	0.0332	0.05	0.02057	0.4679

The GLIMMIX Procedure

Differences of Colony*Condit*Treatm Least Squares Means													
Colony_Type	Condition	Treatment	_Colony_Type	_Condition	_Treatment	Estimate	Standard Error	DF	t Value	Pr > |t|	Alpha	Lower	Upper
CFU-GM	E10.5AGM	0nM	CFU-GM	E10.5AGM	15nM	0.6524	0.07872	36	8.29	<.0001	0.05	0.4927	0.8120
CFU-GM	E10.5AGM	0nM	CFU-GM	E10.5AGM	45nM	1.3397	0.08382	36	15.98	<.0001	0.05	1.1697	1.5097
CFU-GM	E10.5AGM	0nM	CFU-GM	HE+PSCs	0nM	0.5327	0.07812	36	6.82	<.0001	0.05	0.3742	0.6911
CFU-GM	E10.5AGM	0nM	CFU-GM	HE+PSCs	15nM	1.3810	0.08425	36	16.39	<.0001	0.05	1.2101	1.5518
CFU-GM	E10.5AGM	0nM	CFU-GM	HE+PSCs	45nM	2.2323	0.09704	36	23.00	<.0001	0.05	2.0355	2.4292
CFU-GM	E10.5AGM	15nM	CFU-GM	E10.5AGM	45nM	0.6874	0.08618	36	7.98	<.0001	0.05	0.5126	0.8622
CFU-GM	E10.5AGM	15nM	CFU-GM	HE+PSCs	0nM	-0.1197	0.08065	36	-1.48	0.1465	0.05	-0.2832	0.04389
CFU-GM	E10.5AGM	15nM	CFU-GM	HE+PSCs	15nM	0.7286	0.08659	36	8.41	<.0001	0.05	0.5530	0.9042
CFU-GM	E10.5AGM	15nM	CFU-GM	HE+PSCs	45nM	1.5800	0.09909	36	15.95	<.0001	0.05	1.3790	1.7809
CFU-GM	E10.5AGM	45nM	CFU-GM	HE+PSCs	0nM	-0.8071	0.08563	36	-9.43	<.0001	0.05	-0.9807	-0.6334
CFU-GM	E10.5AGM	45nM	CFU-GM	HE+PSCs	15nM	0.04121	0.09125	36	0.45	0.6542	0.05	-0.1438	0.2263
CFU-GM	E10.5AGM	45nM	CFU-GM	HE+PSCs	45nM	0.8926	0.1032	36	8.65	<.0001	0.05	0.6833	1.1019
CFU-GM	HE+PSCs	0nM	CFU-GM	HE+PSCs	15nM	0.8483	0.08605	36	9.86	<.0001	0.05	0.6738	1.0228
CFU-GM	HE+PSCs	0nM	CFU-GM	HE+PSCs	45nM	1.6997	0.09861	36	17.24	<.0001	0.05	1.4997	1.8997
CFU-GM	HE+PSCs	15nM	CFU-GM	HE+PSCs	45nM	0.8514	0.1035	36	8.22	<.0001	0.05	0.6414	1.0613

The GLIMMIX Procedure

Model Information	
**Data Set**	WORK.EXT_FIG10BII_E10MK07
**Response Variable**	Count
**Response Distribution**	Poisson
**Link Function**	Log
**Variance Function**	Default
**Variance Matrix Blocked By**	Dish_ID
**Estimation Technique**	Maximum Likelihood
**Likelihood Approximation**	Laplace
**Degrees of Freedom Method**	Containment

Class Level Information		
**Class**	**Levels**	**Values**
**Colony_Type**	3	BFU-E CFU-GEMM CFU-GM
**Condition**	3	AoE+PSCs E10.5AGM HE+PSCs
**Treatment**	3	0nM 15nM 5nM
**Dish_ID**	27	1 2 3 4 5 6 7 8 9 10 11 12 13 14 15 16 17 18 19 20 21 22 23 24 25 26 27

**Number of Observations Read**	81
**Number of Observations Used**	81

Dimensions	
**G-side Cov. Parameters**	1
**Columns in X**	64
**Columns in Z per Subject**	1
**Subjects (Blocks in V)**	27
**Max Obs per Subject**	3

The GLIMMIX Procedure

Optimization Information	
Optimization Technique	Dual Quasi-Newton
Parameters in Optimization	28
Lower Boundaries	1
Upper Boundaries	0
Fixed Effects	Not Profiled
Starting From	GLM estimates

Iteration History					
Iteration	Restarts	Evaluations	Objective Function	Change	Max Gradient
0	0	4	961.61553545	.	900.8916
1	0	5	950.7199362	10.89559925	69.10147
2	0	4	947.92214788	2.79778832	88.79598
3	0	3	947.19475114	0.72739675	15.53923
4	0	4	946.38896796	0.80578317	18.22464
5	0	2	946.22377636	0.16519160	24.06978
6	0	4	945.76912637	0.45465000	6.432152
7	0	3	945.63374789	0.13537848	4.152557
8	0	3	945.59186299	0.04188490	9.900364
9	0	4	945.42119131	0.17067167	11.87262
10	0	2	945.21906408	0.20212724	3.598006
11	0	3	945.20139553	0.01766855	1.50651
12	0	3	945.19286429	0.00853124	1.702236
13	0	4	945.15961853	0.03324576	2.859765
14	0	3	945.14041835	0.01920018	1.910978
15	0	2	945.11087716	0.02954119	2.080451

The GLIMMIX Procedure

Iteration History					
Iteration	Restarts	Evaluations	Objective Function	Change	Max Gradient
16	0	3	945.09224903	0.01862813	1.595883
17	0	3	945.09015861	0.00209043	0.547223
18	0	3	945.0889038	0.00125481	0.652233
19	0	3	945.0885286	0.00037520	0.691594
20	0	4	945.08758778	0.00094082	0.297277
21	0	3	945.08702792	0.00055986	0.207804
22	0	2	945.08634793	0.00067999	0.497755
23	0	3	945.08601878	0.00032915	0.193248
24	0	2	945.08557129	0.00044748	0.425957
25	0	4	945.08343421	0.00213708	0.387494
26	0	3	945.08299645	0.00043777	0.195654
27	0	2	945.08232578	0.00067067	0.2123
28	0	3	945.08214122	0.00018455	0.141628
29	0	4	945.08169935	0.00044187	0.314993
30	0	3	945.08160787	0.00009149	0.068969
31	0	2	945.08155634	0.00005153	0.083905
32	0	3	945.08152102	0.00003532	0.058494
33	0	2	945.08147509	0.00004593	0.046142

Convergence criterion (GCONV=1E-8) satisfied.

The GLIMMIX Procedure

Fit Statistics	
**-2 Log Likelihood**	945.08
**AIC (smaller is better)**	1001.08
**AICC (smaller is better)**	1032.31
**BIC (smaller is better)**	1037.36
**CAIC (smaller is better)**	1065.36
**HQIC (smaller is better)**	1011.87

Fit Statistics for Conditional Distribution	
**-2 log L(Count | r. effects)**	825.53
**Pearson Chi-Square**	324.66
**Pearson Chi-Square / DF**	4.01

Covariance Parameter Estimates			
**Cov Parm**	**Subject**	**Estimate**	**Standard Error**
**Intercept**	Dish_ID	0.07908	0.02250

The GLIMMIX Procedure

Solutions for Fixed Effects								
Effect	Colony_Type	Condition	Treatment	Estimate	Standard Error	DF	t Value	Pr > |t|
Intercept				5.1397	0.1683	18	30.55	<.0001
Colony_Type	BFU-E			-1.2916	0.09509	36	-13.58	<.0001
Colony_Type	CFU-GEMM			-1.5040	0.1035	36	-14.53	<.0001
Colony_Type	CFU-GM			0	.	.	.	.
Condition		AoE+PSCs		0.1481	0.2374	36	0.62	0.5366
Condition		E10.5AGM		0.8031	0.2357	36	3.41	0.0016
Condition		HE+PSCs		0	.	.	.	.
Colony_Typ*Condition	BFU-E	AoE+PSCs		-0.2663	0.1363	36	-1.95	0.0585
Colony_Typ*Condition	BFU-E	E10.5AGM		0.04664	0.1138	36	0.41	0.6843
Colony_Typ*Condition	BFU-E	HE+PSCs		0	.	.	.	.
Colony_Typ*Condition	CFU-GEMM	AoE+PSCs		0.2261	0.1354	36	1.67	0.1035
Colony_Typ*Condition	CFU-GEMM	E10.5AGM		0.5074	0.1181	36	4.29	0.0001
Colony_Typ*Condition	CFU-GEMM	HE+PSCs		0	.	.	.	.
Colony_Typ*Condition	CFU-GM	AoE+PSCs		0	.	.	.	.
Colony_Typ*Condition	CFU-GM	E10.5AGM		0	.	.	.	.
Colony_Typ*Condition	CFU-GM	HE+PSCs		0	.	.	.	.
Treatment			0nM	0.9639	0.2354	36	4.09	0.0002
Treatment			15nM	-0.8714	0.2436	36	-3.58	0.0010
Treatment			5nM	0	.	.	.	.
Colony_Typ*Treatment	BFU-E		0nM	-0.2542	0.1151	36	-2.21	0.0337
Colony_Typ*Treatment	BFU-E		15nM	0.04048	0.1717	36	0.24	0.8149
Colony_Typ*Treatment	BFU-E		5nM	0	.	.	.	.
Colony_Typ*Treatment	CFU-GEMM		0nM	-0.1284	0.1235	36	-1.04	0.3052
Colony_Typ*Treatment	CFU-GEMM		15nM	0.3148	0.1738	36	1.81	0.0784

The GLIMMIX Procedure

Solutions for Fixed Effects								
Effect	Colony_Type	Condition	Treatment	Estimate	Standard Error	DF	t Value	Pr > |t|
**Colony_Typ*Treatment**	CFU-GEMM		5nM	0	.	.	.	.
**Colony_Typ*Treatment**	CFU-GM		0nM	0	.	.	.	.
**Colony_Typ*Treatment**	CFU-GM		15nM	0	.	.	.	.
**Colony_Typ*Treatment**	CFU-GM		5nM	0	.	.	.	.
**Condition*Treatment**		AoE+PSCs	0nM	-0.05800	0.3324	36	-0.17	0.8625
**Condition*Treatment**		AoE+PSCs	15nM	1.0147	0.3398	36	2.99	0.0051
**Condition*Treatment**		AoE+PSCs	5nM	0	.	.	.	.
**Condition*Treatment**		E10.5AGM	0nM	-0.2685	0.3308	36	-0.81	0.4224
**Condition*Treatment**		E10.5AGM	15nM	0.1664	0.3387	36	0.49	0.6262
**Condition*Treatment**		E10.5AGM	5nM	0	.	.	.	.
**Condition*Treatment**		HE+PSCs	0nM	0	.	.	.	.
**Condition*Treatment**		HE+PSCs	15nM	0	.	.	.	.
**Condition*Treatment**		HE+PSCs	5nM	0	.	.	.	.
**Colony*Condit*Treatm**	BFU-E	AoE+PSCs	0nM	0.3884	0.1622	36	2.40	0.0219
**Colony*Condit*Treatm**	BFU-E	AoE+PSCs	15nM	-1.0506	0.2323	36	-4.52	<.0001
**Colony*Condit*Treatm**	BFU-E	AoE+PSCs	5nM	0	.	.	.	.
**Colony*Condit*Treatm**	BFU-E	E10.5AGM	0nM	0.1492	0.1388	36	1.07	0.2898
**Colony*Condit*Treatm**	BFU-E	E10.5AGM	15nM	-0.03769	0.2030	36	-0.19	0.8538
**Colony*Condit*Treatm**	BFU-E	E10.5AGM	5nM	0	.	.	.	.
**Colony*Condit*Treatm**	BFU-E	HE+PSCs	0nM	0	.	.	.	.
**Colony*Condit*Treatm**	BFU-E	HE+PSCs	15nM	0	.	.	.	.
**Colony*Condit*Treatm**	BFU-E	HE+PSCs	5nM	0	.	.	.	.
**Colony*Condit*Treatm**	CFU-GEMM	AoE+PSCs	0nM	-0.1632	0.1637	36	-1.00	0.3256
**Colony*Condit*Treatm**	CFU-GEMM	AoE+PSCs	15nM	-1.5641	0.2285	36	-6.85	<.0001

The GLIMMIX Procedure

Solutions for Fixed Effects								
Effect	Colony_Type	Condition	Treatment	Estimate	Standard Error	DF	t Value	Pr > |t|
Colony*Condit*Treatm	CFU-GEMM	AoE+PSCs	5nM	0	.	.	.	.
Colony*Condit*Treatm	CFU-GEMM	E10.5AGM	0nM	-0.3330	0.1442	36	-2.31	0.0268
Colony*Condit*Treatm	CFU-GEMM	E10.5AGM	15nM	-0.8240	0.2077	36	-3.97	0.0003
Colony*Condit*Treatm	CFU-GEMM	E10.5AGM	5nM	0	.	.	.	.
Colony*Condit*Treatm	CFU-GEMM	HE+PSCs	0nM	0	.	.	.	.
Colony*Condit*Treatm	CFU-GEMM	HE+PSCs	15nM	0	.	.	.	.
Colony*Condit*Treatm	CFU-GEMM	HE+PSCs	5nM	0	.	.	.	.
Colony*Condit*Treatm	CFU-GM	AoE+PSCs	0nM	0	.	.	.	.
Colony*Condit*Treatm	CFU-GM	AoE+PSCs	15nM	0	.	.	.	.
Colony*Condit*Treatm	CFU-GM	AoE+PSCs	5nM	0	.	.	.	.
Colony*Condit*Treatm	CFU-GM	E10.5AGM	0nM	0	.	.	.	.
Colony*Condit*Treatm	CFU-GM	E10.5AGM	15nM	0	.	.	.	.
Colony*Condit*Treatm	CFU-GM	E10.5AGM	5nM	0	.	.	.	.
Colony*Condit*Treatm	CFU-GM	HE+PSCs	0nM	0	.	.	.	.
Colony*Condit*Treatm	CFU-GM	HE+PSCs	15nM	0	.	.	.	.
Colony*Condit*Treatm	CFU-GM	HE+PSCs	5nM	0	.	.	.	.

Type III Tests of Fixed Effects				
Effect	Num DF	Den DF	F Value	Pr > F
Colony_Type	2	36	2058.35	<.0001
Condition	2	36	20.84	<.0001
Colony_Typ*Condition	4	36	26.48	<.0001
Treatment	2	36	57.85	<.0001
Colony_Typ*Treatment	4	36	11.89	<.0001

The GLIMMIX Procedure

Type III Tests of Fixed Effects				
Effect	Num DF	Den DF	F Value	Pr > F
Condition*Treatment	4	36	0.40	0.8041
Colony*Condit*Treatm	8	36	14.83	<.0001

Condition*Treatment Least Squares Means													
Condition	Treatment	Estimate	Standard Error	DF	t Value	Pr > |t|	Alpha	Lower	Upper	Mean	Standard Error Mean	Lower Mean	Upper Mean
AoE+PSCs	0nM	5.1960	0.1647	36	31.56	<.0001	0.05	4.8621	5.5300	180.56	29.7307	129.30	252.14
AoE+PSCs	15nM	3.7327	0.1737	36	21.48	<.0001	0.05	3.3803	4.0850	41.7902	7.2608	29.3794	59.4438
AoE+PSCs	5nM	4.3426	0.1677	36	25.90	<.0001	0.05	4.0025	4.6826	76.9051	12.8942	54.7367	108.05
E10.5AGM	0nM	5.7023	0.1637	36	34.83	<.0001	0.05	5.3702	6.0343	299.55	49.0413	214.91	417.51
E10.5AGM	15nM	4.3218	0.1677	36	25.77	<.0001	0.05	3.9817	4.6619	75.3230	12.6321	53.6061	105.84
E10.5AGM	5nM	5.1956	0.1645	36	31.59	<.0001	0.05	4.8620	5.5292	180.48	29.6884	129.28	251.95
HE+PSCs	0nM	5.0442	0.1651	36	30.55	<.0001	0.05	4.7094	5.3790	155.13	25.6095	110.99	216.82
HE+PSCs	15nM	3.4548	0.1744	36	19.81	<.0001	0.05	3.1011	3.8086	31.6526	5.5212	22.2214	45.0865
HE+PSCs	5nM	4.2078	0.1684	36	24.99	<.0001	0.05	3.8664	4.5493	67.2104	11.3151	47.7697	94.5627

Differences of Condition*Treatment Least Squares Means											
Condition	Treatment	_Condition	_Treatment	Estimate	Standard Error	DF	t Value	Pr > |t|	Alpha	Lower	Upper
AoE+PSCs	0nM	AoE+PSCs	15nM	1.4634	0.2394	36	6.11	<.0001	0.05	0.9779	1.9489
AoE+PSCs	0nM	AoE+PSCs	5nM	0.8535	0.2350	36	3.63	0.0009	0.05	0.3769	1.3301
AoE+PSCs	0nM	E10.5AGM	0nM	-0.5062	0.2322	36	-2.18	0.0359	0.05	-0.9771	-0.03529
AoE+PSCs	0nM	E10.5AGM	15nM	0.8743	0.2350	36	3.72	0.0007	0.05	0.3976	1.3509
AoE+PSCs	0nM	E10.5AGM	5nM	0.000420	0.2327	36	0.00	0.9986	0.05	-0.4716	0.4725
AoE+PSCs	0nM	HE+PSCs	0nM	0.1518	0.2332	36	0.65	0.5191	0.05	-0.3211	0.6247

The GLIMMIX Procedure

Differences of Condition*Treatment Least Squares Means											
Condition	Treatment	_Condition	_Treatment	Estimate	Standard Error	DF	t Value	Pr > |t|	Alpha	Lower	Upper
AoE+PSCs	0nM	HE+PSCs	15nM	1.7412	0.2399	36	7.26	<.0001	0.05	1.2547	2.2277
AoE+PSCs	0nM	HE+PSCs	5nM	0.9882	0.2355	36	4.20	0.0002	0.05	0.5106	1.4658
AoE+PSCs	15nM	AoE+PSCs	5nM	-0.6099	0.2414	36	-2.53	0.0161	0.05	-1.0996	-0.1203
AoE+PSCs	15nM	E10.5AGM	0nM	-1.9696	0.2387	36	-8.25	<.0001	0.05	-2.4538	-1.4854
AoE+PSCs	15nM	E10.5AGM	15nM	-0.5891	0.2415	36	-2.44	0.0198	0.05	-1.0789	-0.09939
AoE+PSCs	15nM	E10.5AGM	5nM	-1.4630	0.2393	36	-6.11	<.0001	0.05	-1.9482	-0.9777
AoE+PSCs	15nM	HE+PSCs	0nM	-1.3116	0.2397	36	-5.47	<.0001	0.05	-1.7976	-0.8255
AoE+PSCs	15nM	HE+PSCs	15nM	0.2778	0.2462	36	1.13	0.2665	0.05	-0.2214	0.7771
AoE+PSCs	15nM	HE+PSCs	5nM	-0.4752	0.2419	36	-1.96	0.0573	0.05	-0.9658	0.01549
AoE+PSCs	5nM	E10.5AGM	0nM	-1.3597	0.2343	36	-5.80	<.0001	0.05	-1.8350	-0.8844
AoE+PSCs	5nM	E10.5AGM	15nM	0.02079	0.2371	36	0.09	0.9306	0.05	-0.4602	0.5017
AoE+PSCs	5nM	E10.5AGM	5nM	-0.8531	0.2349	36	-3.63	0.0009	0.05	-1.3294	-0.3767
AoE+PSCs	5nM	HE+PSCs	0nM	-0.7017	0.2353	36	-2.98	0.0051	0.05	-1.1789	-0.2245
AoE+PSCs	5nM	HE+PSCs	15nM	0.8878	0.2419	36	3.67	0.0008	0.05	0.3971	1.3784
AoE+PSCs	5nM	HE+PSCs	5nM	0.1347	0.2376	36	0.57	0.5742	0.05	-0.3471	0.6166
E10.5AGM	0nM	E10.5AGM	15nM	1.3805	0.2344	36	5.89	<.0001	0.05	0.9052	1.8558
E10.5AGM	0nM	E10.5AGM	5nM	0.5066	0.2321	36	2.18	0.0356	0.05	0.03595	0.9773
E10.5AGM	0nM	HE+PSCs	0nM	0.6580	0.2325	36	2.83	0.0076	0.05	0.1865	1.1296
E10.5AGM	0nM	HE+PSCs	15nM	2.2474	0.2392	36	9.39	<.0001	0.05	1.7623	2.7326
E10.5AGM	0nM	HE+PSCs	5nM	1.4944	0.2348	36	6.36	<.0001	0.05	1.0182	1.9707
E10.5AGM	15nM	E10.5AGM	5nM	-0.8738	0.2349	36	-3.72	0.0007	0.05	-1.3503	-0.3974
E10.5AGM	15nM	HE+PSCs	0nM	-0.7224	0.2353	36	-3.07	0.0041	0.05	-1.1997	-0.2452
E10.5AGM	15nM	HE+PSCs	15nM	0.8670	0.2420	36	3.58	0.0010	0.05	0.3762	1.3577
E10.5AGM	15nM	HE+PSCs	5nM	0.1140	0.2376	36	0.48	0.6344	0.05	-0.3680	0.5959

The GLIMMIX Procedure

Differences of Condition*Treatment Least Squares Means											
Condition	Treatment	_Condition	_Treatment	Estimate	Standard Error	DF	t Value	Pr > |t|	Alpha	Lower	Upper
E10.5AGM	5nM	HE+PSCs	0nM	0.1514	0.2331	36	0.65	0.5201	0.05	-0.3213	0.6240
E10.5AGM	5nM	HE+PSCs	15nM	1.7408	0.2398	36	7.26	<.0001	0.05	1.2546	2.2271
E10.5AGM	5nM	HE+PSCs	5nM	0.9878	0.2354	36	4.20	0.0002	0.05	0.5104	1.4652
HE+PSCs	0nM	HE+PSCs	15nM	1.5894	0.2402	36	6.62	<.0001	0.05	1.1023	2.0765
HE+PSCs	0nM	HE+PSCs	5nM	0.8364	0.2358	36	3.55	0.0011	0.05	0.3582	1.3146
HE+PSCs	15nM	HE+PSCs	5nM	-0.7530	0.2424	36	-3.11	0.0037	0.05	-1.2447	-0.2614

Colony*Condit*Treatm Least Squares Means														
Colony_Type	Condition	Treatment	Estimate	Standard Error	DF	t Value	Pr > |t|	Alpha	Lower	Upper	Mean	Standard Error Mean	Lower Mean	Upper Mean
BFU-E	AoE+PSCs	0nM	4.7701	0.1708	36	27.92	<.0001	0.05	4.4236	5.1166	117.93	20.1471	83.3991	166.76
BFU-E	AoE+PSCs	15nM	2.8631	0.2023	36	14.15	<.0001	0.05	2.4527	3.2734	17.5150	3.5435	11.6202	26.4001
BFU-E	AoE+PSCs	5nM	3.7300	0.1851	36	20.15	<.0001	0.05	3.3545	4.1054	41.6771	7.7148	28.6322	60.6653
BFU-E	E10.5AGM	0nM	5.2884	0.1675	36	31.58	<.0001	0.05	4.9487	5.6280	198.02	33.1591	141.00	278.10
BFU-E	E10.5AGM	15nM	3.9956	0.1802	36	22.18	<.0001	0.05	3.6302	4.3611	54.3611	9.7952	37.7209	78.3421
BFU-E	E10.5AGM	5nM	4.6979	0.1714	36	27.40	<.0001	0.05	4.3502	5.0456	109.72	18.8101	77.4937	155.34
BFU-E	HE+PSCs	0nM	4.5579	0.1727	36	26.39	<.0001	0.05	4.2076	4.9082	95.3846	16.4765	67.1944	135.40
BFU-E	HE+PSCs	15nM	3.0172	0.2059	36	14.66	<.0001	0.05	2.5997	3.4347	20.4333	4.2064	13.4592	31.0211
BFU-E	HE+PSCs	5nM	3.8481	0.1829	36	21.04	<.0001	0.05	3.4772	4.2191	46.9055	8.5795	32.3683	67.9718
CFU-GEMM	AoE+PSCs	0nM	4.6243	0.1721	36	26.86	<.0001	0.05	4.2752	4.9734	101.93	17.5448	71.8921	144.51
CFU-GEMM	AoE+PSCs	15nM	2.9039	0.2009	36	14.45	<.0001	0.05	2.4963	3.3114	18.2444	3.6660	12.1379	27.4229
CFU-GEMM	AoE+PSCs	5nM	4.0099	0.1798	36	22.30	<.0001	0.05	3.6452	4.3747	55.1430	9.9169	38.2903	79.4131
CFU-GEMM	E10.5AGM	0nM	5.1802	0.1680	36	30.83	<.0001	0.05	4.8394	5.5209	177.71	29.8605	126.39	249.87
CFU-GEMM	E10.5AGM	15nM	3.7319	0.1852	36	20.15	<.0001	0.05	3.3563	4.1076	41.7595	7.7348	28.6823	60.7990

The GLIMMIX Procedure

Colony*Condit*Treatm Least Squares Means

Colony_Type	Condition	Treatment	Estimate	Standard Error	DF	t Value	Pr > |t|	Alpha	Lower	Upper	Mean	Standard Error Mean	Lower Mean	Upper Mean
CFU-GEMM	E10.5AGM	5nM	4.9462	0.1695	36	29.18	<.0001	0.05	4.6024	5.2899	140.64	23.8361	99.7264	198.33
CFU-GEMM	HE+PSCs	0nM	4.4711	0.1736	36	25.75	<.0001	0.05	4.1190	4.8233	87.4568	15.1865	61.4961	124.38
CFU-GEMM	HE+PSCs	15nM	3.0790	0.2035	36	15.13	<.0001	0.05	2.6663	3.4918	21.7375	4.4243	14.3860	32.8457
CFU-GEMM	HE+PSCs	5nM	3.6357	0.1874	36	19.40	<.0001	0.05	3.2555	4.0158	37.9265	7.1091	25.9325	55.4678
CFU-GM	AoE+PSCs	0nM	6.1938	0.1644	36	37.67	<.0001	0.05	5.8603	6.5273	489.69	80.5253	350.82	683.54
CFU-GM	AoE+PSCs	15nM	5.4311	0.1677	36	32.39	<.0001	0.05	5.0911	5.7711	228.39	38.2922	162.56	320.89
CFU-GM	AoE+PSCs	5nM	5.2878	0.1675	36	31.57	<.0001	0.05	4.9482	5.6275	197.91	33.1463	140.92	277.97
CFU-GM	E10.5AGM	0nM	6.6383	0.1637	36	40.55	<.0001	0.05	6.3063	6.9703	763.78	125.03	548.01	1064.51
CFU-GM	E10.5AGM	15nM	5.2378	0.1677	36	31.23	<.0001	0.05	4.8976	5.5779	188.25	31.5742	133.97	264.53
CFU-GM	E10.5AGM	5nM	5.9428	0.1650	36	36.01	<.0001	0.05	5.6081	6.2775	381.01	62.8778	272.63	532.47
CFU-GM	HE+PSCs	0nM	6.1036	0.1646	36	37.07	<.0001	0.05	5.7697	6.4375	447.48	73.6726	320.45	624.87
CFU-GM	HE+PSCs	15nM	4.2683	0.1762	36	24.23	<.0001	0.05	3.9109	4.6256	71.3967	12.5794	49.9450	102.06
CFU-GM	HE+PSCs	5nM	5.1397	0.1683	36	30.55	<.0001	0.05	4.7984	5.4810	170.66	28.7166	121.32	240.07

The GLIMMIX Procedure

Differences of Colony*Condit*Treatm Least Squares Means													
Colony_Type	Condition	Treatment	_Colony_Type	_Condition	_Treatment	Estimate	Standard Error	DF	t Value	Pr > |t|	Alpha	Lower	Upper
BFU-E	AoE+PSCs	0nM	BFU-E	AoE+PSCs	15nM	1.9071	0.2648	36	7.20	<.0001	0.05	1.3700	2.4441
BFU-E	AoE+PSCs	0nM	BFU-E	AoE+PSCs	5nM	1.0402	0.2519	36	4.13	0.0002	0.05	0.5293	1.5510
BFU-E	AoE+PSCs	0nM	BFU-E	E10.5AGM	0nM	-0.5182	0.2392	36	-2.17	0.0370	0.05	-1.0034	-0.03308
BFU-E	AoE+PSCs	0nM	BFU-E	E10.5AGM	15nM	0.7745	0.2483	36	3.12	0.0036	0.05	0.2709	1.2780
BFU-E	AoE+PSCs	0nM	BFU-E	E10.5AGM	5nM	0.07221	0.2420	36	0.30	0.7671	0.05	-0.4186	0.5631
BFU-E	AoE+PSCs	0nM	BFU-E	HE+PSCs	0nM	0.2122	0.2429	36	0.87	0.3882	0.05	-0.2805	0.7049
BFU-E	AoE+PSCs	0nM	BFU-E	HE+PSCs	15nM	1.7529	0.2675	36	6.55	<.0001	0.05	1.2104	2.2955
BFU-E	AoE+PSCs	0nM	BFU-E	HE+PSCs	5nM	0.9220	0.2503	36	3.68	0.0008	0.05	0.4144	1.4296
BFU-E	AoE+PSCs	0nM	CFU-GEMM	AoE+PSCs	0nM	0.1458	0.07806	36	1.87	0.0699	0.05	-0.01246	0.3042
BFU-E	AoE+PSCs	0nM	CFU-GEMM	AoE+PSCs	15nM	1.8663	0.2637	36	7.08	<.0001	0.05	1.3314	2.4012
BFU-E	AoE+PSCs	0nM	CFU-GEMM	AoE+PSCs	5nM	0.7602	0.2480	36	3.06	0.0041	0.05	0.2571	1.2632
BFU-E	AoE+PSCs	0nM	CFU-GEMM	E10.5AGM	0nM	-0.4101	0.2396	36	-1.71	0.0956	0.05	-0.8960	0.07592
BFU-E	AoE+PSCs	0nM	CFU-GEMM	E10.5AGM	15nM	1.0382	0.2520	36	4.12	0.0002	0.05	0.5272	1.5492
BFU-E	AoE+PSCs	0nM	CFU-GEMM	E10.5AGM	5nM	-0.1761	0.2406	36	-0.73	0.4691	0.05	-0.6641	0.3120
BFU-E	AoE+PSCs	0nM	CFU-GEMM	HE+PSCs	0nM	0.2990	0.2436	36	1.23	0.2277	0.05	-0.1951	0.7930
BFU-E	AoE+PSCs	0nM	CFU-GEMM	HE+PSCs	15nM	1.6911	0.2657	36	6.36	<.0001	0.05	1.1522	2.2300
BFU-E	AoE+PSCs	0nM	CFU-GEMM	HE+PSCs	5nM	1.1345	0.2536	36	4.47	<.0001	0.05	0.6201	1.6488
BFU-E	AoE+PSCs	0nM	CFU-GM	AoE+PSCs	0nM	-1.4237	0.05920	36	-24.05	<.0001	0.05	-1.5437	-1.3036
BFU-E	AoE+PSCs	0nM	CFU-GM	AoE+PSCs	15nM	-0.6610	0.2394	36	-2.76	0.0090	0.05	-1.1464	-0.1755
BFU-E	AoE+PSCs	0nM	CFU-GM	AoE+PSCs	5nM	-0.5177	0.2392	36	-2.16	0.0372	0.05	-1.0029	-0.03253
BFU-E	AoE+PSCs	0nM	CFU-GM	E10.5AGM	0nM	-1.8682	0.2366	36	-7.90	<.0001	0.05	-2.3480	-1.3883
BFU-E	AoE+PSCs	0nM	CFU-GM	E10.5AGM	15nM	-0.4677	0.2394	36	-1.95	0.0586	0.05	-0.9532	0.01787
BFU-E	AoE+PSCs	0nM	CFU-GM	E10.5AGM	5nM	-1.1727	0.2375	36	-4.94	<.0001	0.05	-1.6544	-0.6910
BFU-E	AoE+PSCs	0nM	CFU-GM	HE+PSCs	0nM	-1.3335	0.2373	36	-5.62	<.0001	0.05	-1.8147	-0.8523

The GLIMMIX Procedure

Differences of Colony*Condit*Treatm Least Squares Means													
Colony_Type	Condition	Treatment	_Colony_Type	_Condition	_Treatment	Estimate	Standard Error	DF	t Value	Pr > |t|	Alpha	Lower	Upper
BFU-E	AoE+PSCs	0nM	CFU-GM	HE+PSCs	15nM	0.5019	0.2454	36	2.04	0.0482	0.05	0.004133	0.9996
BFU-E	AoE+PSCs	0nM	CFU-GM	HE+PSCs	5nM	-0.3696	0.2398	36	-1.54	0.1320	0.05	-0.8559	0.1167
BFU-E	AoE+PSCs	15nM	BFU-E	AoE+PSCs	5nM	-0.8669	0.2742	36	-3.16	0.0032	0.05	-1.4230	-0.3108
BFU-E	AoE+PSCs	15nM	BFU-E	E10.5AGM	0nM	-2.4253	0.2626	36	-9.23	<.0001	0.05	-2.9579	-1.8927
BFU-E	AoE+PSCs	15nM	BFU-E	E10.5AGM	15nM	-1.1326	0.2709	36	-4.18	0.0002	0.05	-1.6820	-0.5831
BFU-E	AoE+PSCs	15nM	BFU-E	E10.5AGM	5nM	-1.8348	0.2652	36	-6.92	<.0001	0.05	-2.3727	-1.2970
BFU-E	AoE+PSCs	15nM	BFU-E	HE+PSCs	0nM	-1.6949	0.2660	36	-6.37	<.0001	0.05	-2.2344	-1.1553
BFU-E	AoE+PSCs	15nM	BFU-E	HE+PSCs	15nM	-0.1541	0.2886	36	-0.53	0.5966	0.05	-0.7394	0.4312
BFU-E	AoE+PSCs	15nM	BFU-E	HE+PSCs	5nM	-0.9851	0.2727	36	-3.61	0.0009	0.05	-1.5382	-0.4319
BFU-E	AoE+PSCs	15nM	CFU-GEMM	AoE+PSCs	0nM	-1.7612	0.2656	36	-6.63	<.0001	0.05	-2.2999	-1.2225
BFU-E	AoE+PSCs	15nM	CFU-GEMM	AoE+PSCs	15nM	-0.04080	0.1650	36	-0.25	0.8061	0.05	-0.3754	0.2938
BFU-E	AoE+PSCs	15nM	CFU-GEMM	AoE+PSCs	5nM	-1.1469	0.2707	36	-4.24	0.0002	0.05	-1.6958	-0.5979
BFU-E	AoE+PSCs	15nM	CFU-GEMM	E10.5AGM	0nM	-2.3171	0.2630	36	-8.81	<.0001	0.05	-2.8505	-1.7837
BFU-E	AoE+PSCs	15nM	CFU-GEMM	E10.5AGM	15nM	-0.8689	0.2743	36	-3.17	0.0031	0.05	-1.4252	-0.3126
BFU-E	AoE+PSCs	15nM	CFU-GEMM	E10.5AGM	5nM	-2.0831	0.2639	36	-7.89	<.0001	0.05	-2.6184	-1.5478
BFU-E	AoE+PSCs	15nM	CFU-GEMM	HE+PSCs	0nM	-1.6081	0.2666	36	-6.03	<.0001	0.05	-2.1488	-1.0674
BFU-E	AoE+PSCs	15nM	CFU-GEMM	HE+PSCs	15nM	-0.2160	0.2869	36	-0.75	0.4565	0.05	-0.7979	0.3660
BFU-E	AoE+PSCs	15nM	CFU-GEMM	HE+PSCs	5nM	-0.7726	0.2758	36	-2.80	0.0081	0.05	-1.3320	-0.2132
BFU-E	AoE+PSCs	15nM	CFU-GM	AoE+PSCs	0nM	-3.3307	0.2607	36	-12.78	<.0001	0.05	-3.8595	-2.8020
BFU-E	AoE+PSCs	15nM	CFU-GM	AoE+PSCs	15nM	-2.5680	0.1223	36	-21.00	<.0001	0.05	-2.8160	-2.3200
BFU-E	AoE+PSCs	15nM	CFU-GM	AoE+PSCs	5nM	-2.4248	0.2626	36	-9.23	<.0001	0.05	-2.9574	-1.8921
BFU-E	AoE+PSCs	15nM	CFU-GM	E10.5AGM	0nM	-3.7752	0.2602	36	-14.51	<.0001	0.05	-4.3030	-3.2474
BFU-E	AoE+PSCs	15nM	CFU-GM	E10.5AGM	15nM	-2.3747	0.2628	36	-9.04	<.0001	0.05	-2.9077	-1.8418
BFU-E	AoE+PSCs	15nM	CFU-GM	E10.5AGM	5nM	-3.0798	0.2611	36	-11.80	<.0001	0.05	-3.6093	-2.5503

The GLIMMIX Procedure

Differences of Colony*Condit*Treatm Least Squares Means													
Colony_Type	Condition	Treatment	_Colony_Type	_Condition	_Treatment	Estimate	Standard Error	DF	t Value	Pr > |t|	Alpha	Lower	Upper
BFU-E	AoE+PSCs	15nM	CFU-GM	HE+PSCs	0nM	-3.2406	0.2608	36	-12.42	<.0001	0.05	-3.7696	-2.7116
BFU-E	AoE+PSCs	15nM	CFU-GM	HE+PSCs	15nM	-1.4052	0.2682	36	-5.24	<.0001	0.05	-1.9492	-0.8612
BFU-E	AoE+PSCs	15nM	CFU-GM	HE+PSCs	5nM	-2.2766	0.2631	36	-8.65	<.0001	0.05	-2.8103	-1.7430
BFU-E	AoE+PSCs	5nM	BFU-E	E10.5AGM	0nM	-1.5584	0.2496	36	-6.24	<.0001	0.05	-2.0646	-1.0522
BFU-E	AoE+PSCs	5nM	BFU-E	E10.5AGM	15nM	-0.2657	0.2583	36	-1.03	0.3106	0.05	-0.7896	0.2582
BFU-E	AoE+PSCs	5nM	BFU-E	E10.5AGM	5nM	-0.9679	0.2523	36	-3.84	0.0005	0.05	-1.4796	-0.4562
BFU-E	AoE+PSCs	5nM	BFU-E	HE+PSCs	0nM	-0.8280	0.2532	36	-3.27	0.0024	0.05	-1.3415	-0.3145
BFU-E	AoE+PSCs	5nM	BFU-E	HE+PSCs	15nM	0.7128	0.2768	36	2.57	0.0143	0.05	0.1513	1.2742
BFU-E	AoE+PSCs	5nM	BFU-E	HE+PSCs	5nM	-0.1182	0.2602	36	-0.45	0.6524	0.05	-0.6460	0.4096
BFU-E	AoE+PSCs	5nM	CFU-GEMM	AoE+PSCs	0nM	-0.8943	0.2528	36	-3.54	0.0011	0.05	-1.4070	-0.3817
BFU-E	AoE+PSCs	5nM	CFU-GEMM	AoE+PSCs	15nM	0.8261	0.2732	36	3.02	0.0046	0.05	0.2720	1.3802
BFU-E	AoE+PSCs	5nM	CFU-GEMM	AoE+PSCs	5nM	-0.2800	0.1176	36	-2.38	0.0227	0.05	-0.5185	-0.04150
BFU-E	AoE+PSCs	5nM	CFU-GEMM	E10.5AGM	0nM	-1.4502	0.2500	36	-5.80	<.0001	0.05	-1.9572	-0.9432
BFU-E	AoE+PSCs	5nM	CFU-GEMM	E10.5AGM	15nM	-0.00198	0.2619	36	-0.01	0.9940	0.05	-0.5331	0.5291
BFU-E	AoE+PSCs	5nM	CFU-GEMM	E10.5AGM	5nM	-1.2162	0.2510	36	-4.85	<.0001	0.05	-1.7252	-0.7072
BFU-E	AoE+PSCs	5nM	CFU-GEMM	HE+PSCs	0nM	-0.7412	0.2538	36	-2.92	0.0060	0.05	-1.2559	-0.2264
BFU-E	AoE+PSCs	5nM	CFU-GEMM	HE+PSCs	15nM	0.6509	0.2751	36	2.37	0.0235	0.05	0.09295	1.2089
BFU-E	AoE+PSCs	5nM	CFU-GEMM	HE+PSCs	5nM	0.09430	0.2634	36	0.36	0.7225	0.05	-0.4400	0.6286
BFU-E	AoE+PSCs	5nM	CFU-GM	AoE+PSCs	0nM	-2.4638	0.2476	36	-9.95	<.0001	0.05	-2.9660	-1.9617
BFU-E	AoE+PSCs	5nM	CFU-GM	AoE+PSCs	15nM	-1.7011	0.2497	36	-6.81	<.0001	0.05	-2.2076	-1.1946
BFU-E	AoE+PSCs	5nM	CFU-GM	AoE+PSCs	5nM	-1.5579	0.09764	36	-15.96	<.0001	0.05	-1.7559	-1.3599
BFU-E	AoE+PSCs	5nM	CFU-GM	E10.5AGM	0nM	-2.9083	0.2471	36	-11.77	<.0001	0.05	-3.4095	-2.4072
BFU-E	AoE+PSCs	5nM	CFU-GM	E10.5AGM	15nM	-1.5078	0.2498	36	-6.04	<.0001	0.05	-2.0144	-1.0012
BFU-E	AoE+PSCs	5nM	CFU-GM	E10.5AGM	5nM	-2.2129	0.2480	36	-8.92	<.0001	0.05	-2.7158	-1.7099

The GLIMMIX Procedure

Differences of Colony*Condit*Treatm Least Squares Means													
Colony_Type	Condition	Treatment	_Colony_Type	_Condition	_Treatment	Estimate	Standard Error	DF	t Value	Pr > |t|	Alpha	Lower	Upper
BFU-E	AoE+PSCs	5nM	CFU-GM	HE+PSCs	0nM	-2.3737	0.2477	36	-9.58	<.0001	0.05	-2.8761	-1.8713
BFU-E	AoE+PSCs	5nM	CFU-GM	HE+PSCs	15nM	-0.5383	0.2556	36	-2.11	0.0422	0.05	-1.0566	-0.02002
BFU-E	AoE+PSCs	5nM	CFU-GM	HE+PSCs	5nM	-1.4097	0.2502	36	-5.64	<.0001	0.05	-1.9171	-0.9024
BFU-E	E10.5AGM	0nM	BFU-E	E10.5AGM	15nM	1.2927	0.2460	36	5.26	<.0001	0.05	0.7938	1.7916
BFU-E	E10.5AGM	0nM	BFU-E	E10.5AGM	5nM	0.5905	0.2397	36	2.46	0.0187	0.05	0.1044	1.0765
BFU-E	E10.5AGM	0nM	BFU-E	HE+PSCs	0nM	0.7304	0.2406	36	3.04	0.0044	0.05	0.2425	1.2184
BFU-E	E10.5AGM	0nM	BFU-E	HE+PSCs	15nM	2.2712	0.2654	36	8.56	<.0001	0.05	1.7330	2.8094
BFU-E	E10.5AGM	0nM	BFU-E	HE+PSCs	5nM	1.4402	0.2480	36	5.81	<.0001	0.05	0.9373	1.9432
BFU-E	E10.5AGM	0nM	CFU-GEMM	AoE+PSCs	0nM	0.6641	0.2401	36	2.77	0.0089	0.05	0.1771	1.1511
BFU-E	E10.5AGM	0nM	CFU-GEMM	AoE+PSCs	15nM	2.3845	0.2616	36	9.12	<.0001	0.05	1.8540	2.9150
BFU-E	E10.5AGM	0nM	CFU-GEMM	AoE+PSCs	5nM	1.2784	0.2457	36	5.20	<.0001	0.05	0.7801	1.7768
BFU-E	E10.5AGM	0nM	CFU-GEMM	E10.5AGM	0nM	0.1082	0.05961	36	1.82	0.0779	0.05	-0.01270	0.2291
BFU-E	E10.5AGM	0nM	CFU-GEMM	E10.5AGM	15nM	1.5564	0.2497	36	6.23	<.0001	0.05	1.0500	2.0628
BFU-E	E10.5AGM	0nM	CFU-GEMM	E10.5AGM	5nM	0.3422	0.2383	36	1.44	0.1596	0.05	-0.1410	0.8254
BFU-E	E10.5AGM	0nM	CFU-GEMM	HE+PSCs	0nM	0.8172	0.2412	36	3.39	0.0017	0.05	0.3280	1.3065
BFU-E	E10.5AGM	0nM	CFU-GEMM	HE+PSCs	15nM	2.2093	0.2636	36	8.38	<.0001	0.05	1.6748	2.7439
BFU-E	E10.5AGM	0nM	CFU-GEMM	HE+PSCs	5nM	1.6527	0.2513	36	6.58	<.0001	0.05	1.1429	2.1625
BFU-E	E10.5AGM	0nM	CFU-GM	AoE+PSCs	0nM	-0.9054	0.2347	36	-3.86	0.0005	0.05	-1.3814	-0.4294
BFU-E	E10.5AGM	0nM	CFU-GM	AoE+PSCs	15nM	-0.1427	0.2370	36	-0.60	0.5507	0.05	-0.6233	0.3379
BFU-E	E10.5AGM	0nM	CFU-GM	AoE+PSCs	5nM	0.000519	0.2368	36	0.00	0.9983	0.05	-0.4798	0.4808
BFU-E	E10.5AGM	0nM	CFU-GM	E10.5AGM	0nM	-1.3499	0.04600	36	-29.34	<.0001	0.05	-1.4432	-1.2566
BFU-E	E10.5AGM	0nM	CFU-GM	E10.5AGM	15nM	0.05057	0.2370	36	0.21	0.8322	0.05	-0.4301	0.5312
BFU-E	E10.5AGM	0nM	CFU-GM	E10.5AGM	5nM	-0.6545	0.2351	36	-2.78	0.0085	0.05	-1.1313	-0.1776
BFU-E	E10.5AGM	0nM	CFU-GM	HE+PSCs	0nM	-0.8153	0.2348	36	-3.47	0.0014	0.05	-1.2915	-0.3390

The GLIMMIX Procedure

Differences of Colony*Condit*Treatm Least Squares Means													
Colony_Type	Condition	Treatment	_Colony_Type	_Condition	_Treatment	Estimate	Standard Error	DF	t Value	Pr > |t|	Alpha	Lower	Upper
BFU-E	E10.5AGM	0nM	CFU-GM	HE+PSCs	15nM	1.0201	0.2431	36	4.20	0.0002	0.05	0.5271	1.5131
BFU-E	E10.5AGM	0nM	CFU-GM	HE+PSCs	5nM	0.1487	0.2374	36	0.63	0.5351	0.05	-0.3328	0.6301
BFU-E	E10.5AGM	15nM	BFU-E	E10.5AGM	5nM	-0.7023	0.2487	36	-2.82	0.0077	0.05	-1.2067	-0.1978
BFU-E	E10.5AGM	15nM	BFU-E	HE+PSCs	0nM	-0.5623	0.2496	36	-2.25	0.0305	0.05	-1.0685	-0.05603
BFU-E	E10.5AGM	15nM	BFU-E	HE+PSCs	15nM	0.9785	0.2736	36	3.58	0.0010	0.05	0.4236	1.5333
BFU-E	E10.5AGM	15nM	BFU-E	HE+PSCs	5nM	0.1475	0.2568	36	0.57	0.5692	0.05	-0.3732	0.6682
BFU-E	E10.5AGM	15nM	CFU-GEMM	AoE+PSCs	0nM	-0.6286	0.2492	36	-2.52	0.0162	0.05	-1.1340	-0.1232
BFU-E	E10.5AGM	15nM	CFU-GEMM	AoE+PSCs	15nM	1.0918	0.2699	36	4.05	0.0003	0.05	0.5444	1.6392
BFU-E	E10.5AGM	15nM	CFU-GEMM	AoE+PSCs	5nM	-0.01428	0.2546	36	-0.06	0.9556	0.05	-0.5306	0.5020
BFU-E	E10.5AGM	15nM	CFU-GEMM	E10.5AGM	0nM	-1.1845	0.2464	36	-4.81	<.0001	0.05	-1.6842	-0.6848
BFU-E	E10.5AGM	15nM	CFU-GEMM	E10.5AGM	15nM	0.2637	0.1185	36	2.23	0.0324	0.05	0.02346	0.5040
BFU-E	E10.5AGM	15nM	CFU-GEMM	E10.5AGM	5nM	-0.9505	0.2474	36	-3.84	0.0005	0.05	-1.4522	-0.4488
BFU-E	E10.5AGM	15nM	CFU-GEMM	HE+PSCs	0nM	-0.4755	0.2502	36	-1.90	0.0654	0.05	-0.9830	0.03202
BFU-E	E10.5AGM	15nM	CFU-GEMM	HE+PSCs	15nM	0.9166	0.2718	36	3.37	0.0018	0.05	0.3653	1.4679
BFU-E	E10.5AGM	15nM	CFU-GEMM	HE+PSCs	5nM	0.3600	0.2600	36	1.38	0.1747	0.05	-0.1673	0.8873
BFU-E	E10.5AGM	15nM	CFU-GM	AoE+PSCs	0nM	-2.1981	0.2439	36	-9.01	<.0001	0.05	-2.6929	-1.7034
BFU-E	E10.5AGM	15nM	CFU-GM	AoE+PSCs	15nM	-1.4354	0.2461	36	-5.83	<.0001	0.05	-1.9346	-0.9363
BFU-E	E10.5AGM	15nM	CFU-GM	AoE+PSCs	5nM	-1.2922	0.2460	36	-5.25	<.0001	0.05	-1.7911	-0.7933
BFU-E	E10.5AGM	15nM	CFU-GM	E10.5AGM	0nM	-2.6426	0.2434	36	-10.86	<.0001	0.05	-3.1364	-2.1489
BFU-E	E10.5AGM	15nM	CFU-GM	E10.5AGM	15nM	-1.2421	0.08864	36	-14.01	<.0001	0.05	-1.4219	-1.0624
BFU-E	E10.5AGM	15nM	CFU-GM	E10.5AGM	5nM	-1.9472	0.2443	36	-7.97	<.0001	0.05	-2.4427	-1.4516
BFU-E	E10.5AGM	15nM	CFU-GM	HE+PSCs	0nM	-2.1080	0.2441	36	-8.64	<.0001	0.05	-2.6030	-1.6130
BFU-E	E10.5AGM	15nM	CFU-GM	HE+PSCs	15nM	-0.2726	0.2520	36	-1.08	0.2866	0.05	-0.7837	0.2385
BFU-E	E10.5AGM	15nM	CFU-GM	HE+PSCs	5nM	-1.1440	0.2465	36	-4.64	<.0001	0.05	-1.6440	-0.6440

The GLIMMIX Procedure

Differences of Colony*Condit*Treatm Least Squares Means													
Colony_Type	Condition	Treatment	_Colony_Type	_Condition	_Treatment	Estimate	Standard Error	DF	t Value	Pr > |t|	Alpha	Lower	Upper
BFU-E	E10.5AGM	5nM	BFU-E	HE+PSCs	0nM	0.1400	0.2434	36	0.58	0.5688	0.05	-0.3536	0.6336
BFU-E	E10.5AGM	5nM	BFU-E	HE+PSCs	15nM	1.6807	0.2679	36	6.27	<.0001	0.05	1.1374	2.2241
BFU-E	E10.5AGM	5nM	BFU-E	HE+PSCs	5nM	0.8498	0.2507	36	3.39	0.0017	0.05	0.3413	1.3582
BFU-E	E10.5AGM	5nM	CFU-GEMM	AoE+PSCs	0nM	0.07364	0.2429	36	0.30	0.7636	0.05	-0.4191	0.5663
BFU-E	E10.5AGM	5nM	CFU-GEMM	AoE+PSCs	15nM	1.7940	0.2641	36	6.79	<.0001	0.05	1.2583	2.3297
BFU-E	E10.5AGM	5nM	CFU-GEMM	AoE+PSCs	5nM	0.6880	0.2485	36	2.77	0.0088	0.05	0.1841	1.1919
BFU-E	E10.5AGM	5nM	CFU-GEMM	E10.5AGM	0nM	-0.4823	0.2401	36	-2.01	0.0521	0.05	-0.9691	0.004589
BFU-E	E10.5AGM	5nM	CFU-GEMM	E10.5AGM	15nM	0.9660	0.2524	36	3.83	0.0005	0.05	0.4541	1.4778
BFU-E	E10.5AGM	5nM	CFU-GEMM	E10.5AGM	5nM	-0.2483	0.07345	36	-3.38	0.0018	0.05	-0.3972	-0.09932
BFU-E	E10.5AGM	5nM	CFU-GEMM	HE+PSCs	0nM	0.2268	0.2440	36	0.93	0.3590	0.05	-0.2681	0.7216
BFU-E	E10.5AGM	5nM	CFU-GEMM	HE+PSCs	15nM	1.6189	0.2661	36	6.08	<.0001	0.05	1.0792	2.1586
BFU-E	E10.5AGM	5nM	CFU-GEMM	HE+PSCs	5nM	1.0622	0.2540	36	4.18	0.0002	0.05	0.5471	1.5774
BFU-E	E10.5AGM	5nM	CFU-GM	AoE+PSCs	0nM	-1.4959	0.2376	36	-6.30	<.0001	0.05	-1.9777	-1.0141
BFU-E	E10.5AGM	5nM	CFU-GM	AoE+PSCs	15nM	-0.7332	0.2398	36	-3.06	0.0042	0.05	-1.2195	-0.2469
BFU-E	E10.5AGM	5nM	CFU-GM	AoE+PSCs	5nM	-0.5899	0.2397	36	-2.46	0.0188	0.05	-1.0760	-0.1039
BFU-E	E10.5AGM	5nM	CFU-GM	E10.5AGM	0nM	-1.9404	0.2370	36	-8.19	<.0001	0.05	-2.4211	-1.4596
BFU-E	E10.5AGM	5nM	CFU-GM	E10.5AGM	15nM	-0.5399	0.2398	36	-2.25	0.0306	0.05	-1.0263	-0.05346
BFU-E	E10.5AGM	5nM	CFU-GM	E10.5AGM	5nM	-1.2449	0.06247	36	-19.93	<.0001	0.05	-1.3716	-1.1182
BFU-E	E10.5AGM	5nM	CFU-GM	HE+PSCs	0nM	-1.4057	0.2377	36	-5.91	<.0001	0.05	-1.8878	-0.9237
BFU-E	E10.5AGM	5nM	CFU-GM	HE+PSCs	15nM	0.4296	0.2458	36	1.75	0.0890	0.05	-0.06893	0.9282
BFU-E	E10.5AGM	5nM	CFU-GM	HE+PSCs	5nM	-0.4418	0.2402	36	-1.84	0.0742	0.05	-0.9290	0.04539
BFU-E	HE+PSCs	0nM	BFU-E	HE+PSCs	15nM	1.5407	0.2687	36	5.73	<.0001	0.05	0.9957	2.0858
BFU-E	HE+PSCs	0nM	BFU-E	HE+PSCs	5nM	0.7098	0.2516	36	2.82	0.0077	0.05	0.1995	1.2200
BFU-E	HE+PSCs	0nM	CFU-GEMM	AoE+PSCs	0nM	-0.06634	0.2439	36	-0.27	0.7871	0.05	-0.5609	0.4282

The GLIMMIX Procedure

Differences of Colony*Condit*Treatm Least Squares Means													
Colony_Type	Condition	Treatment	_Colony_Type	_Condition	_Treatment	Estimate	Standard Error	DF	t Value	Pr > |t|	Alpha	Lower	Upper
BFU-E	HE+PSCs	0nM	CFU-GEMM	AoE+PSCs	15nM	1.6541	0.2650	36	6.24	<.0001	0.05	1.1167	2.1915
BFU-E	HE+PSCs	0nM	CFU-GEMM	AoE+PSCs	5nM	0.5480	0.2494	36	2.20	0.0345	0.05	0.04226	1.0537
BFU-E	HE+PSCs	0nM	CFU-GEMM	E10.5AGM	0nM	-0.6222	0.2410	36	-2.58	0.0140	0.05	-1.1110	-0.1335
BFU-E	HE+PSCs	0nM	CFU-GEMM	E10.5AGM	15nM	0.8260	0.2533	36	3.26	0.0024	0.05	0.3123	1.3396
BFU-E	HE+PSCs	0nM	CFU-GEMM	E10.5AGM	5nM	-0.3883	0.2420	36	-1.60	0.1174	0.05	-0.8791	0.1025
BFU-E	HE+PSCs	0nM	CFU-GEMM	HE+PSCs	0nM	0.08677	0.08520	36	1.02	0.3153	0.05	-0.08603	0.2596
BFU-E	HE+PSCs	0nM	CFU-GEMM	HE+PSCs	15nM	1.4789	0.2670	36	5.54	<.0001	0.05	0.9375	2.0203
BFU-E	HE+PSCs	0nM	CFU-GEMM	HE+PSCs	5nM	0.9223	0.2549	36	3.62	0.0009	0.05	0.4053	1.4392
BFU-E	HE+PSCs	0nM	CFU-GM	AoE+PSCs	0nM	-1.6359	0.2385	36	-6.86	<.0001	0.05	-2.1195	-1.1522
BFU-E	HE+PSCs	0nM	CFU-GM	AoE+PSCs	15nM	-0.8732	0.2407	36	-3.63	0.0009	0.05	-1.3614	-0.3850
BFU-E	HE+PSCs	0nM	CFU-GM	AoE+PSCs	5nM	-0.7299	0.2406	36	-3.03	0.0045	0.05	-1.2179	-0.2420
BFU-E	HE+PSCs	0nM	CFU-GM	E10.5AGM	0nM	-2.0804	0.2380	36	-8.74	<.0001	0.05	-2.5630	-1.5977
BFU-E	HE+PSCs	0nM	CFU-GM	E10.5AGM	15nM	-0.6799	0.2408	36	-2.82	0.0077	0.05	-1.1682	-0.1916
BFU-E	HE+PSCs	0nM	CFU-GM	E10.5AGM	5nM	-1.3849	0.2389	36	-5.80	<.0001	0.05	-1.8694	-0.9004
BFU-E	HE+PSCs	0nM	CFU-GM	HE+PSCs	0nM	-1.5457	0.06491	36	-23.81	<.0001	0.05	-1.6774	-1.4141
BFU-E	HE+PSCs	0nM	CFU-GM	HE+PSCs	15nM	0.2897	0.2467	36	1.17	0.2481	0.05	-0.2107	0.7901
BFU-E	HE+PSCs	0nM	CFU-GM	HE+PSCs	5nM	-0.5818	0.2411	36	-2.41	0.0211	0.05	-1.0708	-0.09271
BFU-E	HE+PSCs	15nM	BFU-E	HE+PSCs	5nM	-0.8310	0.2754	36	-3.02	0.0047	0.05	-1.3895	-0.2725
BFU-E	HE+PSCs	15nM	CFU-GEMM	AoE+PSCs	0nM	-1.6071	0.2683	36	-5.99	<.0001	0.05	-2.1513	-1.0629
BFU-E	HE+PSCs	15nM	CFU-GEMM	AoE+PSCs	15nM	0.1133	0.2876	36	0.39	0.6959	0.05	-0.4700	0.6967
BFU-E	HE+PSCs	15nM	CFU-GEMM	AoE+PSCs	5nM	-0.9928	0.2733	36	-3.63	0.0009	0.05	-1.5471	-0.4384
BFU-E	HE+PSCs	15nM	CFU-GEMM	E10.5AGM	0nM	-2.1630	0.2657	36	-8.14	<.0001	0.05	-2.7019	-1.6241
BFU-E	HE+PSCs	15nM	CFU-GEMM	E10.5AGM	15nM	-0.7148	0.2769	36	-2.58	0.0141	0.05	-1.2764	-0.1531
BFU-E	HE+PSCs	15nM	CFU-GEMM	E10.5AGM	5nM	-1.9290	0.2667	36	-7.23	<.0001	0.05	-2.4698	-1.3882

The GLIMMIX Procedure

Differences of Colony*Condit*Treatm Least Squares Means													
Colony_Type	Condition	Treatment	_Colony_Type	_Condition	_Treatment	Estimate	Standard Error	DF	t Value	Pr > |t|	Alpha	Lower	Upper
BFU-E	HE+PSCs	15nM	CFU-GEMM	HE+PSCs	0nM	-1.4540	0.2693	36	-5.40	<.0001	0.05	-2.0002	-0.9078
BFU-E	HE+PSCs	15nM	CFU-GEMM	HE+PSCs	15nM	-0.06187	0.1755	36	-0.35	0.7265	0.05	-0.4178	0.2941
BFU-E	HE+PSCs	15nM	CFU-GEMM	HE+PSCs	5nM	-0.6185	0.2784	36	-2.22	0.0327	0.05	-1.1831	-0.05384
BFU-E	HE+PSCs	15nM	CFU-GM	AoE+PSCs	0nM	-3.1766	0.2635	36	-12.06	<.0001	0.05	-3.7110	-2.6423
BFU-E	HE+PSCs	15nM	CFU-GM	AoE+PSCs	15nM	-2.4139	0.2655	36	-9.09	<.0001	0.05	-2.9523	-1.8755
BFU-E	HE+PSCs	15nM	CFU-GM	AoE+PSCs	5nM	-2.2707	0.2654	36	-8.56	<.0001	0.05	-2.8089	-1.7325
BFU-E	HE+PSCs	15nM	CFU-GM	E10.5AGM	0nM	-3.6211	0.2630	36	-13.77	<.0001	0.05	-4.1545	-3.0877
BFU-E	HE+PSCs	15nM	CFU-GM	E10.5AGM	15nM	-2.2206	0.2655	36	-8.36	<.0001	0.05	-2.7591	-1.6821
BFU-E	HE+PSCs	15nM	CFU-GM	E10.5AGM	5nM	-2.9257	0.2638	36	-11.09	<.0001	0.05	-3.4608	-2.3906
BFU-E	HE+PSCs	15nM	CFU-GM	HE+PSCs	0nM	-3.0865	0.2636	36	-11.71	<.0001	0.05	-3.6211	-2.5519
BFU-E	HE+PSCs	15nM	CFU-GM	HE+PSCs	15nM	-1.2511	0.1429	36	-8.75	<.0001	0.05	-1.5409	-0.9612
BFU-E	HE+PSCs	15nM	CFU-GM	HE+PSCs	5nM	-2.1225	0.2659	36	-7.98	<.0001	0.05	-2.6618	-1.5833
BFU-E	HE+PSCs	5nM	CFU-GEMM	AoE+PSCs	0nM	-0.7761	0.2512	36	-3.09	0.0038	0.05	-1.2855	-0.2667
BFU-E	HE+PSCs	5nM	CFU-GEMM	AoE+PSCs	15nM	0.9443	0.2717	36	3.48	0.0013	0.05	0.3932	1.4954
BFU-E	HE+PSCs	5nM	CFU-GEMM	AoE+PSCs	5nM	-0.1618	0.2565	36	-0.63	0.5322	0.05	-0.6820	0.3584
BFU-E	HE+PSCs	5nM	CFU-GEMM	E10.5AGM	0nM	-1.3320	0.2484	36	-5.36	<.0001	0.05	-1.8358	-0.8283
BFU-E	HE+PSCs	5nM	CFU-GEMM	E10.5AGM	15nM	0.1162	0.2603	36	0.45	0.6580	0.05	-0.4117	0.6441
BFU-E	HE+PSCs	5nM	CFU-GEMM	E10.5AGM	5nM	-1.0980	0.2494	36	-4.40	<.0001	0.05	-1.6038	-0.5923
BFU-E	HE+PSCs	5nM	CFU-GEMM	HE+PSCs	0nM	-0.6230	0.2522	36	-2.47	0.0184	0.05	-1.1345	-0.1115
BFU-E	HE+PSCs	5nM	CFU-GEMM	HE+PSCs	15nM	0.7691	0.2736	36	2.81	0.0079	0.05	0.2141	1.3241
BFU-E	HE+PSCs	5nM	CFU-GEMM	HE+PSCs	5nM	0.2125	0.1260	36	1.69	0.1002	0.05	-0.04296	0.4679
BFU-E	HE+PSCs	5nM	CFU-GM	AoE+PSCs	0nM	-2.3456	0.2460	36	-9.54	<.0001	0.05	-2.8445	-1.8468
BFU-E	HE+PSCs	5nM	CFU-GM	AoE+PSCs	15nM	-1.5829	0.2481	36	-6.38	<.0001	0.05	-2.0862	-1.0797
BFU-E	HE+PSCs	5nM	CFU-GM	AoE+PSCs	5nM	-1.4397	0.2480	36	-5.81	<.0001	0.05	-1.9427	-0.9367

The GLIMMIX Procedure

Differences of Colony*Condit*Treatm Least Squares Means													
Colony_Type	Condition	Treatment	_Colony_Type	_Condition	_Treatment	Estimate	Standard Error	DF	t Value	Pr > |t|	Alpha	Lower	Upper
BFU-E	HE+PSCs	5nM	CFU-GM	E10.5AGM	0nM	-2.7901	0.2455	36	-11.37	<.0001	0.05	-3.2880	-2.2923
BFU-E	HE+PSCs	5nM	CFU-GM	E10.5AGM	15nM	-1.3896	0.2482	36	-5.60	<.0001	0.05	-1.8930	-0.8863
BFU-E	HE+PSCs	5nM	CFU-GM	E10.5AGM	5nM	-2.0947	0.2464	36	-8.50	<.0001	0.05	-2.5943	-1.5951
BFU-E	HE+PSCs	5nM	CFU-GM	HE+PSCs	0nM	-2.2555	0.2461	36	-9.17	<.0001	0.05	-2.7546	-1.7564
BFU-E	HE+PSCs	5nM	CFU-GM	HE+PSCs	15nM	-0.4201	0.2540	36	-1.65	0.1068	0.05	-0.9352	0.09495
BFU-E	HE+PSCs	5nM	CFU-GM	HE+PSCs	5nM	-1.2916	0.09509	36	-13.58	<.0001	0.05	-1.4844	-1.0987
CFU-GEMM	AoE+PSCs	0nM	CFU-GEMM	AoE+PSCs	15nM	1.7204	0.2646	36	6.50	<.0001	0.05	1.1838	2.2570
CFU-GEMM	AoE+PSCs	0nM	CFU-GEMM	AoE+PSCs	5nM	0.6143	0.2489	36	2.47	0.0185	0.05	0.1095	1.1192
CFU-GEMM	AoE+PSCs	0nM	CFU-GEMM	E10.5AGM	0nM	-0.5559	0.2405	36	-2.31	0.0267	0.05	-1.0437	-0.06805
CFU-GEMM	AoE+PSCs	0nM	CFU-GEMM	E10.5AGM	15nM	0.8923	0.2529	36	3.53	0.0012	0.05	0.3795	1.4052
CFU-GEMM	AoE+PSCs	0nM	CFU-GEMM	E10.5AGM	5nM	-0.3219	0.2416	36	-1.33	0.1910	0.05	-0.8118	0.1680
CFU-GEMM	AoE+PSCs	0nM	CFU-GEMM	HE+PSCs	0nM	0.1531	0.2445	36	0.63	0.5351	0.05	-0.3428	0.6490
CFU-GEMM	AoE+PSCs	0nM	CFU-GEMM	HE+PSCs	15nM	1.5452	0.2666	36	5.80	<.0001	0.05	1.0046	2.0858
CFU-GEMM	AoE+PSCs	0nM	CFU-GEMM	HE+PSCs	5nM	0.9886	0.2545	36	3.88	0.0004	0.05	0.4725	1.5047
CFU-GEMM	AoE+PSCs	0nM	CFU-GM	AoE+PSCs	0nM	-1.5695	0.06284	36	-24.98	<.0001	0.05	-1.6970	-1.4421
CFU-GEMM	AoE+PSCs	0nM	CFU-GM	AoE+PSCs	15nM	-0.8068	0.2403	36	-3.36	0.0019	0.05	-1.2941	-0.3195
CFU-GEMM	AoE+PSCs	0nM	CFU-GM	AoE+PSCs	5nM	-0.6636	0.2402	36	-2.76	0.0090	0.05	-1.1506	-0.1765
CFU-GEMM	AoE+PSCs	0nM	CFU-GM	E10.5AGM	0nM	-2.0140	0.2375	36	-8.48	<.0001	0.05	-2.4958	-1.5323
CFU-GEMM	AoE+PSCs	0nM	CFU-GM	E10.5AGM	15nM	-0.6135	0.2403	36	-2.55	0.0151	0.05	-1.1009	-0.1261
CFU-GEMM	AoE+PSCs	0nM	CFU-GM	E10.5AGM	5nM	-1.3186	0.2385	36	-5.53	<.0001	0.05	-1.8022	-0.8349
CFU-GEMM	AoE+PSCs	0nM	CFU-GM	HE+PSCs	0nM	-1.4794	0.2382	36	-6.21	<.0001	0.05	-1.9624	-0.9963
CFU-GEMM	AoE+PSCs	0nM	CFU-GM	HE+PSCs	15nM	0.3560	0.2463	36	1.45	0.1570	0.05	-0.1435	0.8556
CFU-GEMM	AoE+PSCs	0nM	CFU-GM	HE+PSCs	5nM	-0.5154	0.2407	36	-2.14	0.0391	0.05	-1.0036	-0.02725
CFU-GEMM	AoE+PSCs	15nM	CFU-GEMM	AoE+PSCs	5nM	-1.1061	0.2697	36	-4.10	0.0002	0.05	-1.6530	-0.5592

The GLIMMIX Procedure

Differences of Colony*Condit*Treatm Least Squares Means													
Colony_Type	Condition	Treatment	_Colony_Type	_Condition	_Treatment	Estimate	Standard Error	DF	t Value	Pr > |t|	Alpha	Lower	Upper
CFU-GEMM	AoE+PSCs	15nM	CFU-GEMM	E10.5AGM	0nM	-2.2763	0.2619	36	-8.69	<.0001	0.05	-2.8075	-1.7451
CFU-GEMM	AoE+PSCs	15nM	CFU-GEMM	E10.5AGM	15nM	-0.8281	0.2733	36	-3.03	0.0045	0.05	-1.3823	-0.2738
CFU-GEMM	AoE+PSCs	15nM	CFU-GEMM	E10.5AGM	5nM	-2.0423	0.2629	36	-7.77	<.0001	0.05	-2.5754	-1.5092
CFU-GEMM	AoE+PSCs	15nM	CFU-GEMM	HE+PSCs	0nM	-1.5673	0.2656	36	-5.90	<.0001	0.05	-2.1059	-1.0287
CFU-GEMM	AoE+PSCs	15nM	CFU-GEMM	HE+PSCs	15nM	-0.1752	0.2860	36	-0.61	0.5440	0.05	-0.7552	0.4048
CFU-GEMM	AoE+PSCs	15nM	CFU-GEMM	HE+PSCs	5nM	-0.7318	0.2748	36	-2.66	0.0115	0.05	-1.2891	-0.1745
CFU-GEMM	AoE+PSCs	15nM	CFU-GM	AoE+PSCs	0nM	-3.2899	0.2596	36	-12.67	<.0001	0.05	-3.8165	-2.7633
CFU-GEMM	AoE+PSCs	15nM	CFU-GM	AoE+PSCs	15nM	-2.5272	0.1200	36	-21.06	<.0001	0.05	-2.7706	-2.2839
CFU-GEMM	AoE+PSCs	15nM	CFU-GM	AoE+PSCs	5nM	-2.3840	0.2616	36	-9.11	<.0001	0.05	-2.9145	-1.8535
CFU-GEMM	AoE+PSCs	15nM	CFU-GM	E10.5AGM	0nM	-3.7344	0.2592	36	-14.41	<.0001	0.05	-4.2601	-3.2088
CFU-GEMM	AoE+PSCs	15nM	CFU-GM	E10.5AGM	15nM	-2.3339	0.2617	36	-8.92	<.0001	0.05	-2.8647	-1.8031
CFU-GEMM	AoE+PSCs	15nM	CFU-GM	E10.5AGM	5nM	-3.0390	0.2600	36	-11.69	<.0001	0.05	-3.5663	-2.5116
CFU-GEMM	AoE+PSCs	15nM	CFU-GM	HE+PSCs	0nM	-3.1998	0.2598	36	-12.32	<.0001	0.05	-3.7266	-2.6729
CFU-GEMM	AoE+PSCs	15nM	CFU-GM	HE+PSCs	15nM	-1.3644	0.2672	36	-5.11	<.0001	0.05	-1.9063	-0.8225
CFU-GEMM	AoE+PSCs	15nM	CFU-GM	HE+PSCs	5nM	-2.2358	0.2621	36	-8.53	<.0001	0.05	-2.7674	-1.7043
CFU-GEMM	AoE+PSCs	5nM	CFU-GEMM	E10.5AGM	0nM	-1.1702	0.2461	36	-4.75	<.0001	0.05	-1.6694	-0.6711
CFU-GEMM	AoE+PSCs	5nM	CFU-GEMM	E10.5AGM	15nM	0.2780	0.2582	36	1.08	0.2887	0.05	-0.2456	0.8016
CFU-GEMM	AoE+PSCs	5nM	CFU-GEMM	E10.5AGM	5nM	-0.9362	0.2471	36	-3.79	0.0006	0.05	-1.4374	-0.4351
CFU-GEMM	AoE+PSCs	5nM	CFU-GEMM	HE+PSCs	0nM	-0.4612	0.2500	36	-1.84	0.0733	0.05	-0.9682	0.04579
CFU-GEMM	AoE+PSCs	5nM	CFU-GEMM	HE+PSCs	15nM	0.9309	0.2716	36	3.43	0.0015	0.05	0.3801	1.4817
CFU-GEMM	AoE+PSCs	5nM	CFU-GEMM	HE+PSCs	5nM	0.3743	0.2598	36	1.44	0.1583	0.05	-0.1525	0.9011
CFU-GEMM	AoE+PSCs	5nM	CFU-GM	AoE+PSCs	0nM	-2.1838	0.2437	36	-8.96	<.0001	0.05	-2.6781	-1.6896
CFU-GEMM	AoE+PSCs	5nM	CFU-GM	AoE+PSCs	15nM	-1.4211	0.2459	36	-5.78	<.0001	0.05	-1.9198	-0.9225
CFU-GEMM	AoE+PSCs	5nM	CFU-GM	AoE+PSCs	5nM	-1.2779	0.08724	36	-14.65	<.0001	0.05	-1.4548	-1.1010

The GLIMMIX Procedure

Differences of Colony*Condit*Treatm Least Squares Means													
Colony_Type	Condition	Treatment	_Colony_Type	_Condition	_Treatment	Estimate	Standard Error	DF	t Value	Pr > |t|	Alpha	Lower	Upper
CFU-GEMM	AoE+PSCs	5nM	CFU-GM	E10.5AGM	0nM	-2.6283	0.2432	36	-10.81	<.0001	0.05	-3.1216	-2.1351
CFU-GEMM	AoE+PSCs	5nM	CFU-GM	E10.5AGM	15nM	-1.2279	0.2459	36	-4.99	<.0001	0.05	-1.7266	-0.7291
CFU-GEMM	AoE+PSCs	5nM	CFU-GM	E10.5AGM	5nM	-1.9329	0.2441	36	-7.92	<.0001	0.05	-2.4279	-1.4379
CFU-GEMM	AoE+PSCs	5nM	CFU-GM	HE+PSCs	0nM	-2.0937	0.2438	36	-8.59	<.0001	0.05	-2.5882	-1.5992
CFU-GEMM	AoE+PSCs	5nM	CFU-GM	HE+PSCs	15nM	-0.2583	0.2518	36	-1.03	0.3117	0.05	-0.7689	0.2523
CFU-GEMM	AoE+PSCs	5nM	CFU-GM	HE+PSCs	5nM	-1.1298	0.2463	36	-4.59	<.0001	0.05	-1.6293	-0.6303
CFU-GEMM	E10.5AGM	0nM	CFU-GEMM	E10.5AGM	15nM	1.4482	0.2501	36	5.79	<.0001	0.05	0.9410	1.9554
CFU-GEMM	E10.5AGM	0nM	CFU-GEMM	E10.5AGM	5nM	0.2340	0.2387	36	0.98	0.3334	0.05	-0.2500	0.7180
CFU-GEMM	E10.5AGM	0nM	CFU-GEMM	HE+PSCs	0nM	0.7090	0.2416	36	2.93	0.0058	0.05	0.2190	1.1991
CFU-GEMM	E10.5AGM	0nM	CFU-GEMM	HE+PSCs	15nM	2.1011	0.2639	36	7.96	<.0001	0.05	1.5658	2.6364
CFU-GEMM	E10.5AGM	0nM	CFU-GEMM	HE+PSCs	5nM	1.5445	0.2517	36	6.14	<.0001	0.05	1.0340	2.0550
CFU-GEMM	E10.5AGM	0nM	CFU-GM	AoE+PSCs	0nM	-1.0136	0.2351	36	-4.31	0.0001	0.05	-1.4904	-0.5368
CFU-GEMM	E10.5AGM	0nM	CFU-GM	AoE+PSCs	15nM	-0.2509	0.2374	36	-1.06	0.2975	0.05	-0.7323	0.2305
CFU-GEMM	E10.5AGM	0nM	CFU-GM	AoE+PSCs	5nM	-0.1077	0.2372	36	-0.45	0.6527	0.05	-0.5888	0.3735
CFU-GEMM	E10.5AGM	0nM	CFU-GM	E10.5AGM	0nM	-1.4581	0.04805	36	-30.35	<.0001	0.05	-1.5556	-1.3607
CFU-GEMM	E10.5AGM	0nM	CFU-GM	E10.5AGM	15nM	-0.05762	0.2374	36	-0.24	0.8096	0.05	-0.5391	0.4239
CFU-GEMM	E10.5AGM	0nM	CFU-GM	E10.5AGM	5nM	-0.7627	0.2355	36	-3.24	0.0026	0.05	-1.2403	-0.2850
CFU-GEMM	E10.5AGM	0nM	CFU-GM	HE+PSCs	0nM	-0.9235	0.2352	36	-3.93	0.0004	0.05	-1.4006	-0.4464
CFU-GEMM	E10.5AGM	0nM	CFU-GM	HE+PSCs	15nM	0.9119	0.2435	36	3.75	0.0006	0.05	0.4181	1.4057
CFU-GEMM	E10.5AGM	0nM	CFU-GM	HE+PSCs	5nM	0.04047	0.2378	36	0.17	0.8658	0.05	-0.4418	0.5227
CFU-GEMM	E10.5AGM	15nM	CFU-GEMM	E10.5AGM	5nM	-1.2142	0.2511	36	-4.84	<.0001	0.05	-1.7234	-0.7051
CFU-GEMM	E10.5AGM	15nM	CFU-GEMM	HE+PSCs	0nM	-0.7392	0.2539	36	-2.91	0.0061	0.05	-1.2541	-0.2243
CFU-GEMM	E10.5AGM	15nM	CFU-GEMM	HE+PSCs	15nM	0.6529	0.2752	36	2.37	0.0231	0.05	0.09477	1.2110
CFU-GEMM	E10.5AGM	15nM	CFU-GEMM	HE+PSCs	5nM	0.09628	0.2635	36	0.37	0.7170	0.05	-0.4382	0.6307

The GLIMMIX Procedure

Differences of Colony*Condit*Treatm Least Squares Means													
Colony_Type	Condition	Treatment	_Colony_Type	_Condition	_Treatment	Estimate	Standard Error	DF	t Value	Pr > |t|	Alpha	Lower	Upper
CFU-GEMM	E10.5AGM	15nM	CFU-GM	AoE+PSCs	0nM	-2.4619	0.2477	36	-9.94	<.0001	0.05	-2.9642	-1.9595
CFU-GEMM	E10.5AGM	15nM	CFU-GM	AoE+PSCs	15nM	-1.6992	0.2498	36	-6.80	<.0001	0.05	-2.2058	-1.1925
CFU-GEMM	E10.5AGM	15nM	CFU-GM	AoE+PSCs	5nM	-1.5559	0.2497	36	-6.23	<.0001	0.05	-2.0623	-1.0495
CFU-GEMM	E10.5AGM	15nM	CFU-GM	E10.5AGM	0nM	-2.9064	0.2472	36	-11.76	<.0001	0.05	-3.4077	-2.4050
CFU-GEMM	E10.5AGM	15nM	CFU-GM	E10.5AGM	15nM	-1.5059	0.09848	36	-15.29	<.0001	0.05	-1.7056	-1.3061
CFU-GEMM	E10.5AGM	15nM	CFU-GM	E10.5AGM	5nM	-2.2109	0.2481	36	-8.91	<.0001	0.05	-2.7140	-1.7078
CFU-GEMM	E10.5AGM	15nM	CFU-GM	HE+PSCs	0nM	-2.3717	0.2478	36	-9.57	<.0001	0.05	-2.8743	-1.8691
CFU-GEMM	E10.5AGM	15nM	CFU-GM	HE+PSCs	15nM	-0.5363	0.2556	36	-2.10	0.0430	0.05	-1.0548	-0.01787
CFU-GEMM	E10.5AGM	15nM	CFU-GM	HE+PSCs	5nM	-1.4078	0.2502	36	-5.63	<.0001	0.05	-1.9153	-0.9003
CFU-GEMM	E10.5AGM	5nM	CFU-GEMM	HE+PSCs	0nM	0.4750	0.2427	36	1.96	0.0581	0.05	-0.01709	0.9671
CFU-GEMM	E10.5AGM	5nM	CFU-GEMM	HE+PSCs	15nM	1.8671	0.2649	36	7.05	<.0001	0.05	1.3300	2.4043
CFU-GEMM	E10.5AGM	5nM	CFU-GEMM	HE+PSCs	5nM	1.3105	0.2527	36	5.19	<.0001	0.05	0.7980	1.8230
CFU-GEMM	E10.5AGM	5nM	CFU-GM	AoE+PSCs	0nM	-1.2476	0.2362	36	-5.28	<.0001	0.05	-1.7265	-0.7687
CFU-GEMM	E10.5AGM	5nM	CFU-GM	AoE+PSCs	15nM	-0.4849	0.2384	36	-2.03	0.0494	0.05	-0.9684	-0.00141
CFU-GEMM	E10.5AGM	5nM	CFU-GM	AoE+PSCs	5nM	-0.3417	0.2383	36	-1.43	0.1602	0.05	-0.8249	0.1416
CFU-GEMM	E10.5AGM	5nM	CFU-GM	E10.5AGM	0nM	-1.6921	0.2356	36	-7.18	<.0001	0.05	-2.1700	-1.2142
CFU-GEMM	E10.5AGM	5nM	CFU-GM	E10.5AGM	15nM	-0.2916	0.2384	36	-1.22	0.2293	0.05	-0.7752	0.1920
CFU-GEMM	E10.5AGM	5nM	CFU-GM	E10.5AGM	5nM	-0.9967	0.05689	36	-17.52	<.0001	0.05	-1.1120	-0.8813
CFU-GEMM	E10.5AGM	5nM	CFU-GM	HE+PSCs	0nM	-1.1575	0.2363	36	-4.90	<.0001	0.05	-1.6367	-0.6782
CFU-GEMM	E10.5AGM	5nM	CFU-GM	HE+PSCs	15nM	0.6779	0.2445	36	2.77	0.0087	0.05	0.1821	1.1737
CFU-GEMM	E10.5AGM	5nM	CFU-GM	HE+PSCs	5nM	-0.1935	0.2388	36	-0.81	0.4231	0.05	-0.6779	0.2908
CFU-GEMM	HE+PSCs	0nM	CFU-GEMM	HE+PSCs	15nM	1.3921	0.2675	36	5.20	<.0001	0.05	0.8495	1.9347
CFU-GEMM	HE+PSCs	0nM	CFU-GEMM	HE+PSCs	5nM	0.8355	0.2555	36	3.27	0.0024	0.05	0.3173	1.3537
CFU-GEMM	HE+PSCs	0nM	CFU-GM	AoE+PSCs	0nM	-1.7226	0.2392	36	-7.20	<.0001	0.05	-2.2077	-1.2376

The GLIMMIX Procedure

Differences of Colony*Condit*Treatm Least Squares Means													
Colony_Type	Condition	Treatment	_Colony_Type	_Condition	_Treatment	Estimate	Standard Error	DF	t Value	Pr > |t|	Alpha	Lower	Upper
CFU-GEMM	HE+PSCs	0nM	CFU-GM	AoE+PSCs	15nM	-0.9599	0.2414	36	-3.98	0.0003	0.05	-1.4495	-0.4704
CFU-GEMM	HE+PSCs	0nM	CFU-GM	AoE+PSCs	5nM	-0.8167	0.2413	36	-3.39	0.0017	0.05	-1.3060	-0.3274
CFU-GEMM	HE+PSCs	0nM	CFU-GM	E10.5AGM	0nM	-2.1671	0.2386	36	-9.08	<.0001	0.05	-2.6511	-1.6831
CFU-GEMM	HE+PSCs	0nM	CFU-GM	E10.5AGM	15nM	-0.7666	0.2414	36	-3.18	0.0031	0.05	-1.2563	-0.2770
CFU-GEMM	HE+PSCs	0nM	CFU-GM	E10.5AGM	5nM	-1.4717	0.2396	36	-6.14	<.0001	0.05	-1.9575	-0.9858
CFU-GEMM	HE+PSCs	0nM	CFU-GM	HE+PSCs	0nM	-1.6325	0.06729	36	-24.26	<.0001	0.05	-1.7690	-1.4960
CFU-GEMM	HE+PSCs	0nM	CFU-GM	HE+PSCs	15nM	0.2029	0.2474	36	0.82	0.4175	0.05	-0.2988	0.7046
CFU-GEMM	HE+PSCs	0nM	CFU-GM	HE+PSCs	5nM	-0.6686	0.2418	36	-2.76	0.0089	0.05	-1.1589	-0.1782
CFU-GEMM	HE+PSCs	15nM	CFU-GEMM	HE+PSCs	5nM	-0.5566	0.2767	36	-2.01	0.0518	0.05	-1.1178	0.004553
CFU-GEMM	HE+PSCs	15nM	CFU-GM	AoE+PSCs	0nM	-3.1147	0.2617	36	-11.90	<.0001	0.05	-3.6454	-2.5841
CFU-GEMM	HE+PSCs	15nM	CFU-GM	AoE+PSCs	15nM	-2.3520	0.2637	36	-8.92	<.0001	0.05	-2.8868	-1.8173
CFU-GEMM	HE+PSCs	15nM	CFU-GM	AoE+PSCs	5nM	-2.2088	0.2636	36	-8.38	<.0001	0.05	-2.7434	-1.6742
CFU-GEMM	HE+PSCs	15nM	CFU-GM	E10.5AGM	0nM	-3.5592	0.2612	36	-13.63	<.0001	0.05	-4.0890	-3.0295
CFU-GEMM	HE+PSCs	15nM	CFU-GM	E10.5AGM	15nM	-2.1587	0.2637	36	-8.19	<.0001	0.05	-2.6936	-1.6239
CFU-GEMM	HE+PSCs	15nM	CFU-GM	E10.5AGM	5nM	-2.8638	0.2620	36	-10.93	<.0001	0.05	-3.3952	-2.3324
CFU-GEMM	HE+PSCs	15nM	CFU-GM	HE+PSCs	0nM	-3.0246	0.2618	36	-11.55	<.0001	0.05	-3.5555	-2.4937
CFU-GEMM	HE+PSCs	15nM	CFU-GM	HE+PSCs	15nM	-1.1892	0.1395	36	-8.52	<.0001	0.05	-1.4722	-0.9062
CFU-GEMM	HE+PSCs	15nM	CFU-GM	HE+PSCs	5nM	-2.0607	0.2641	36	-7.80	<.0001	0.05	-2.5962	-1.5251
CFU-GEMM	HE+PSCs	5nM	CFU-GM	AoE+PSCs	0nM	-2.5581	0.2494	36	-10.26	<.0001	0.05	-3.0638	-2.0524
CFU-GEMM	HE+PSCs	5nM	CFU-GM	AoE+PSCs	15nM	-1.7954	0.2515	36	-7.14	<.0001	0.05	-2.3055	-1.2854
CFU-GEMM	HE+PSCs	5nM	CFU-GM	AoE+PSCs	5nM	-1.6522	0.2514	36	-6.57	<.0001	0.05	-2.1620	-1.1424
CFU-GEMM	HE+PSCs	5nM	CFU-GM	E10.5AGM	0nM	-3.0026	0.2489	36	-12.07	<.0001	0.05	-3.5073	-2.4979
CFU-GEMM	HE+PSCs	5nM	CFU-GM	E10.5AGM	15nM	-1.6021	0.2515	36	-6.37	<.0001	0.05	-2.1123	-1.0920
CFU-GEMM	HE+PSCs	5nM	CFU-GM	E10.5AGM	5nM	-2.3072	0.2497	36	-9.24	<.0001	0.05	-2.8137	-1.8007

The GLIMMIX Procedure

Differences of Colony*Condit*Treatm Least Squares Means													
Colony_Type	Condition	Treatment	_Colony_Type	_Condition	_Treatment	Estimate	Standard Error	DF	t Value	Pr > |t|	Alpha	Lower	Upper
CFU-GEMM	HE+PSCs	5nM	CFU-GM	HE+PSCs	0nM	-2.4680	0.2495	36	-9.89	<.0001	0.05	-2.9740	-1.9620
CFU-GEMM	HE+PSCs	5nM	CFU-GM	HE+PSCs	15nM	-0.6326	0.2573	36	-2.46	0.0189	0.05	-1.1543	-0.1109
CFU-GEMM	HE+PSCs	5nM	CFU-GM	HE+PSCs	5nM	-1.5040	0.1035	36	-14.53	<.0001	0.05	-1.7140	-1.2941
CFU-GM	AoE+PSCs	0nM	CFU-GM	AoE+PSCs	15nM	0.7627	0.2348	36	3.25	0.0025	0.05	0.2864	1.2390
CFU-GM	AoE+PSCs	0nM	CFU-GM	AoE+PSCs	5nM	0.9059	0.2347	36	3.86	0.0005	0.05	0.4299	1.3820
CFU-GM	AoE+PSCs	0nM	CFU-GM	E10.5AGM	0nM	-0.4445	0.2320	36	-1.92	0.0634	0.05	-0.9151	0.02607
CFU-GM	AoE+PSCs	0nM	CFU-GM	E10.5AGM	15nM	0.9560	0.2349	36	4.07	0.0002	0.05	0.4796	1.4324
CFU-GM	AoE+PSCs	0nM	CFU-GM	E10.5AGM	5nM	0.2510	0.2330	36	1.08	0.2886	0.05	-0.2215	0.7234
CFU-GM	AoE+PSCs	0nM	CFU-GM	HE+PSCs	0nM	0.09014	0.2327	36	0.39	0.7007	0.05	-0.3818	0.5621
CFU-GM	AoE+PSCs	0nM	CFU-GM	HE+PSCs	15nM	1.9255	0.2410	36	7.99	<.0001	0.05	1.4367	2.4143
CFU-GM	AoE+PSCs	0nM	CFU-GM	HE+PSCs	5nM	1.0541	0.2353	36	4.48	<.0001	0.05	0.5769	1.5312
CFU-GM	AoE+PSCs	15nM	CFU-GM	AoE+PSCs	5nM	0.1432	0.2370	36	0.60	0.5493	0.05	-0.3373	0.6238
CFU-GM	AoE+PSCs	15nM	CFU-GM	E10.5AGM	0nM	-1.2072	0.2343	36	-5.15	<.0001	0.05	-1.6824	-0.7320
CFU-GM	AoE+PSCs	15nM	CFU-GM	E10.5AGM	15nM	0.1933	0.2371	36	0.82	0.4204	0.05	-0.2877	0.6742
CFU-GM	AoE+PSCs	15nM	CFU-GM	E10.5AGM	5nM	-0.5117	0.2353	36	-2.18	0.0363	0.05	-0.9889	-0.03463
CFU-GM	AoE+PSCs	15nM	CFU-GM	HE+PSCs	0nM	-0.6726	0.2350	36	-2.86	0.0070	0.05	-1.1491	-0.1960
CFU-GM	AoE+PSCs	15nM	CFU-GM	HE+PSCs	15nM	1.1628	0.2432	36	4.78	<.0001	0.05	0.6697	1.6560
CFU-GM	AoE+PSCs	15nM	CFU-GM	HE+PSCs	5nM	0.2914	0.2375	36	1.23	0.2279	0.05	-0.1904	0.7731
CFU-GM	AoE+PSCs	5nM	CFU-GM	E10.5AGM	0nM	-1.3504	0.2342	36	-5.77	<.0001	0.05	-1.8254	-0.8755
CFU-GM	AoE+PSCs	5nM	CFU-GM	E10.5AGM	15nM	0.05005	0.2370	36	0.21	0.8339	0.05	-0.4307	0.5308
CFU-GM	AoE+PSCs	5nM	CFU-GM	E10.5AGM	5nM	-0.6550	0.2351	36	-2.79	0.0085	0.05	-1.1318	-0.1781
CFU-GM	AoE+PSCs	5nM	CFU-GM	HE+PSCs	0nM	-0.8158	0.2349	36	-3.47	0.0014	0.05	-1.2921	-0.3395
CFU-GM	AoE+PSCs	5nM	CFU-GM	HE+PSCs	15nM	1.0196	0.2431	36	4.19	0.0002	0.05	0.5266	1.5126
CFU-GM	AoE+PSCs	5nM	CFU-GM	HE+PSCs	5nM	0.1481	0.2374	36	0.62	0.5366	0.05	-0.3333	0.6296

The GLIMMIX Procedure

Differences of Colony*Condit*Treatm Least Squares Means													
Colony_Type	Condition	Treatment	_Colony_Type	_Condition	_Treatment	Estimate	Standard Error	DF	t Value	Pr > |t|	Alpha	Lower	Upper
CFU-GM	E10.5AGM	0nM	CFU-GM	E10.5AGM	15nM	1.4005	0.2344	36	5.98	<.0001	0.05	0.9252	1.8758
CFU-GM	E10.5AGM	0nM	CFU-GM	E10.5AGM	5nM	0.6955	0.2324	36	2.99	0.0050	0.05	0.2240	1.1669
CFU-GM	E10.5AGM	0nM	CFU-GM	HE+PSCs	0nM	0.5346	0.2322	36	2.30	0.0272	0.05	0.06379	1.0055
CFU-GM	E10.5AGM	0nM	CFU-GM	HE+PSCs	15nM	2.3700	0.2405	36	9.85	<.0001	0.05	1.8823	2.8578
CFU-GM	E10.5AGM	0nM	CFU-GM	HE+PSCs	5nM	1.4986	0.2348	36	6.38	<.0001	0.05	1.0225	1.9747
CFU-GM	E10.5AGM	15nM	CFU-GM	E10.5AGM	5nM	-0.7050	0.2353	36	-3.00	0.0049	0.05	-1.1822	-0.2278
CFU-GM	E10.5AGM	15nM	CFU-GM	HE+PSCs	0nM	-0.8659	0.2350	36	-3.68	0.0007	0.05	-1.3425	-0.3892
CFU-GM	E10.5AGM	15nM	CFU-GM	HE+PSCs	15nM	0.9695	0.2433	36	3.99	0.0003	0.05	0.4762	1.4629
CFU-GM	E10.5AGM	15nM	CFU-GM	HE+PSCs	5nM	0.09809	0.2376	36	0.41	0.6822	0.05	-0.3837	0.5799
CFU-GM	E10.5AGM	5nM	CFU-GM	HE+PSCs	0nM	-0.1608	0.2331	36	-0.69	0.4947	0.05	-0.6336	0.3120
CFU-GM	E10.5AGM	5nM	CFU-GM	HE+PSCs	15nM	1.6746	0.2414	36	6.94	<.0001	0.05	1.1850	2.1642
CFU-GM	E10.5AGM	5nM	CFU-GM	HE+PSCs	5nM	0.8031	0.2357	36	3.41	0.0016	0.05	0.3251	1.2811
CFU-GM	HE+PSCs	0nM	CFU-GM	HE+PSCs	15nM	1.8354	0.2411	36	7.61	<.0001	0.05	1.3463	2.3244
CFU-GM	HE+PSCs	0nM	CFU-GM	HE+PSCs	5nM	0.9639	0.2354	36	4.09	0.0002	0.05	0.4865	1.4414
CFU-GM	HE+PSCs	15nM	CFU-GM	HE+PSCs	5nM	-0.8714	0.2436	36	-3.58	0.0010	0.05	-1.3656	-0.3773

The GENMOD Procedure

Model Information	
Data Set	WORK.EXT_FIG10BII_E10MK07_TRANS
Distribution	Normal
Link Function	Identity
Dependent Variable	Percentage

Number of Observations Read	45
Number of Observations Used	45

Class Level Information		
Class	Levels	Values
Condition	3	AoE+PSCs E10.5AGM HE+PSCs
Treatment	3	0nM 15nM 5nM

Parameter Information			
Parameter	Effect	Condition	Treatment
Prm1	Intercept		
Prm2	Condition	AoE+PSCs	
Prm3	Condition	E10.5AGM	
Prm4	Condition	HE+PSCs	
Prm5	Treatment		0nM
Prm6	Treatment		15nM
Prm7	Treatment		5nM
Prm8	Condition*Treatment	AoE+PSCs	0nM
Prm9	Condition*Treatment	AoE+PSCs	15nM
Prm10	Condition*Treatment	AoE+PSCs	5nM
Prm11	Condition*Treatment	E10.5AGM	0nM

The GENMOD Procedure

Parameter Information			
**Parameter**	**Effect**	**Condition**	**Treatment**
**Prm12**	Condition*Treatment	E10.5AGM	15nM
**Prm13**	Condition*Treatment	E10.5AGM	5nM
**Prm14**	Condition*Treatment	HE+PSCs	0nM
**Prm15**	Condition*Treatment	HE+PSCs	15nM
**Prm16**	Condition*Treatment	HE+PSCs	5nM

Criteria For Assessing Goodness Of Fit			
**Criterion**	**DF**	**Value**	**Value/DF**
**Deviance**	36	570.8000	15.8556
**Scaled Deviance**	36	45.0000	1.2500
**Pearson Chi-Square**	36	570.8000	15.8556
**Scaled Pearson X2**	36	45.0000	1.2500
**Log Likelihood**		-121.0107	
**Full Log Likelihood**		-121.0107	
**AIC (smaller is better)**		262.0214	
**AICC (smaller is better)**		268.4920	
**BIC (smaller is better)**		280.0880	

Algorithm converged.

The GENMOD Procedure

Analysis Of Maximum Likelihood Parameter Estimates									
Parameter			DF	Estimate	Standard Error	Wald 95% Confidence Limits		Wald Chi-Square	Pr > ChiSq
**Intercept**			1	12.6000	1.5928	9.4782	15.7218	62.58	<.0001
**Condition**	AoE+PSCs		1	1.4000	2.2525	-3.0148	5.8148	0.39	0.5343
**Condition**	E10.5AGM		1	25.6000	2.2525	21.1852	30.0148	129.17	<.0001
**Condition**	HE+PSCs		0	0.0000	0.0000	0.0000	0.0000	.	.
**Treatment**	0nM		1	13.6000	2.2525	9.1852	18.0148	36.45	<.0001
**Treatment**	15nM		1	-9.2000	2.2525	-13.6148	-4.7852	16.68	<.0001
**Treatment**	5nM		0	0.0000	0.0000	0.0000	0.0000	.	.
**Condition*Treatment**	AoE+PSCs	0nM	1	4.2000	3.1855	-2.0435	10.4435	1.74	0.1873
**Condition*Treatment**	AoE+PSCs	15nM	1	1.6000	3.1855	-4.6435	7.8435	0.25	0.6155
**Condition*Treatment**	AoE+PSCs	5nM	0	0.0000	0.0000	0.0000	0.0000	.	.
**Condition*Treatment**	E10.5AGM	0nM	1	5.0000	3.1855	-1.2435	11.2435	2.46	0.1165
**Condition*Treatment**	E10.5AGM	15nM	1	-16.0000	3.1855	-22.2435	-9.7565	25.23	<.0001
**Condition*Treatment**	E10.5AGM	5nM	0	0.0000	0.0000	0.0000	0.0000	.	.
**Condition*Treatment**	HE+PSCs	0nM	0	0.0000	0.0000	0.0000	0.0000	.	.
**Condition*Treatment**	HE+PSCs	15nM	0	0.0000	0.0000	0.0000	0.0000	.	.
**Condition*Treatment**	HE+PSCs	5nM	0	0.0000	0.0000	0.0000	0.0000	.	.
**Scale**			1	3.5615	0.3754	2.8968	4.3789		

Note: The scale parameter was estimated by maximum likelihood.

Condition*Treatment Least Squares Means								
Condition	Treatment	Estimate	Standard Error	z Value	Pr > |z|	Alpha	Lower	Upper
AoE+PSCs	0nM	31.8000	1.5928	19.97	<.0001	0.05	28.6782	34.9218
AoE+PSCs	15nM	6.4000	1.5928	4.02	<.0001	0.05	3.2782	9.5218
AoE+PSCs	5nM	14.0000	1.5928	8.79	<.0001	0.05	10.8782	17.1218
E10.5AGM	0nM	56.8000	1.5928	35.66	<.0001	0.05	53.6782	59.9218

The GENMOD Procedure

Condition*Treatment Least Squares Means								
Condition	Treatment	Estimate	Standard Error	z Value	Pr > |z|	Alpha	Lower	Upper
E10.5AGM	15nM	13.0000	1.5928	8.16	<.0001	0.05	9.8782	16.1218
E10.5AGM	5nM	38.2000	1.5928	23.98	<.0001	0.05	35.0782	41.3218
HE+PSCs	0nM	26.2000	1.5928	16.45	<.0001	0.05	23.0782	29.3218
HE+PSCs	15nM	3.4000	1.5928	2.13	0.0328	0.05	0.2782	6.5218
HE+PSCs	5nM	12.6000	1.5928	7.91	<.0001	0.05	9.4782	15.7218

Differences of Condition*Treatment Least Squares Means										
Condition	Treatment	_Condition	_Treatment	Estimate	Standard Error	z Value	Pr > |z|	Alpha	Lower	Upper
AoE+PSCs	0nM	AoE+PSCs	15nM	25.4000	2.2525	11.28	<.0001	0.05	20.9852	29.8148
AoE+PSCs	0nM	AoE+PSCs	5nM	17.8000	2.2525	7.90	<.0001	0.05	13.3852	22.2148
AoE+PSCs	0nM	E10.5AGM	0nM	-25.0000	2.2525	-11.10	<.0001	0.05	-29.4148	-20.5852
AoE+PSCs	0nM	E10.5AGM	15nM	18.8000	2.2525	8.35	<.0001	0.05	14.3852	23.2148
AoE+PSCs	0nM	E10.5AGM	5nM	-6.4000	2.2525	-2.84	0.0045	0.05	-10.8148	-1.9852
AoE+PSCs	0nM	HE+PSCs	0nM	5.6000	2.2525	2.49	0.0129	0.05	1.1852	10.0148
AoE+PSCs	0nM	HE+PSCs	15nM	28.4000	2.2525	12.61	<.0001	0.05	23.9852	32.8148
AoE+PSCs	0nM	HE+PSCs	5nM	19.2000	2.2525	8.52	<.0001	0.05	14.7852	23.6148
AoE+PSCs	15nM	AoE+PSCs	5nM	-7.6000	2.2525	-3.37	0.0007	0.05	-12.0148	-3.1852
AoE+PSCs	15nM	E10.5AGM	0nM	-50.4000	2.2525	-22.38	<.0001	0.05	-54.8148	-45.9852
AoE+PSCs	15nM	E10.5AGM	15nM	-6.6000	2.2525	-2.93	0.0034	0.05	-11.0148	-2.1852
AoE+PSCs	15nM	E10.5AGM	5nM	-31.8000	2.2525	-14.12	<.0001	0.05	-36.2148	-27.3852
AoE+PSCs	15nM	HE+PSCs	0nM	-19.8000	2.2525	-8.79	<.0001	0.05	-24.2148	-15.3852
AoE+PSCs	15nM	HE+PSCs	15nM	3.0000	2.2525	1.33	0.1829	0.05	-1.4148	7.4148
AoE+PSCs	15nM	HE+PSCs	5nM	-6.2000	2.2525	-2.75	0.0059	0.05	-10.6148	-1.7852
AoE+PSCs	5nM	E10.5AGM	0nM	-42.8000	2.2525	-19.00	<.0001	0.05	-47.2148	-38.3852

The GENMOD Procedure

Differences of Condition*Treatment Least Squares Means										
Condition	Treatment	_Condition	_Treatment	Estimate	Standard Error	z Value	Pr > |z|	Alpha	Lower	Upper
AoE+PSCs	5nM	E10.5AGM	15nM	1.0000	2.2525	0.44	0.6571	0.05	-3.4148	5.4148
AoE+PSCs	5nM	E10.5AGM	5nM	-24.2000	2.2525	-10.74	<.0001	0.05	-28.6148	-19.7852
AoE+PSCs	5nM	HE+PSCs	0nM	-12.2000	2.2525	-5.42	<.0001	0.05	-16.6148	-7.7852
AoE+PSCs	5nM	HE+PSCs	15nM	10.6000	2.2525	4.71	<.0001	0.05	6.1852	15.0148
AoE+PSCs	5nM	HE+PSCs	5nM	1.4000	2.2525	0.62	0.5343	0.05	-3.0148	5.8148
E10.5AGM	0nM	E10.5AGM	15nM	43.8000	2.2525	19.45	<.0001	0.05	39.3852	48.2148
E10.5AGM	0nM	E10.5AGM	5nM	18.6000	2.2525	8.26	<.0001	0.05	14.1852	23.0148
E10.5AGM	0nM	HE+PSCs	0nM	30.6000	2.2525	13.58	<.0001	0.05	26.1852	35.0148
E10.5AGM	0nM	HE+PSCs	15nM	53.4000	2.2525	23.71	<.0001	0.05	48.9852	57.8148
E10.5AGM	0nM	HE+PSCs	5nM	44.2000	2.2525	19.62	<.0001	0.05	39.7852	48.6148
E10.5AGM	15nM	E10.5AGM	5nM	-25.2000	2.2525	-11.19	<.0001	0.05	-29.6148	-20.7852
E10.5AGM	15nM	HE+PSCs	0nM	-13.2000	2.2525	-5.86	<.0001	0.05	-17.6148	-8.7852
E10.5AGM	15nM	HE+PSCs	15nM	9.6000	2.2525	4.26	<.0001	0.05	5.1852	14.0148
E10.5AGM	15nM	HE+PSCs	5nM	0.4000	2.2525	0.18	0.8591	0.05	-4.0148	4.8148
E10.5AGM	5nM	HE+PSCs	0nM	12.0000	2.2525	5.33	<.0001	0.05	7.5852	16.4148
E10.5AGM	5nM	HE+PSCs	15nM	34.8000	2.2525	15.45	<.0001	0.05	30.3852	39.2148
E10.5AGM	5nM	HE+PSCs	5nM	25.6000	2.2525	11.37	<.0001	0.05	21.1852	30.0148
HE+PSCs	0nM	HE+PSCs	15nM	22.8000	2.2525	10.12	<.0001	0.05	18.3852	27.2148
HE+PSCs	0nM	HE+PSCs	5nM	13.6000	2.2525	6.04	<.0001	0.05	9.1852	18.0148
HE+PSCs	15nM	HE+PSCs	5nM	-9.2000	2.2525	-4.08	<.0001	0.05	-13.6148	-4.7852

The GENMOD Procedure

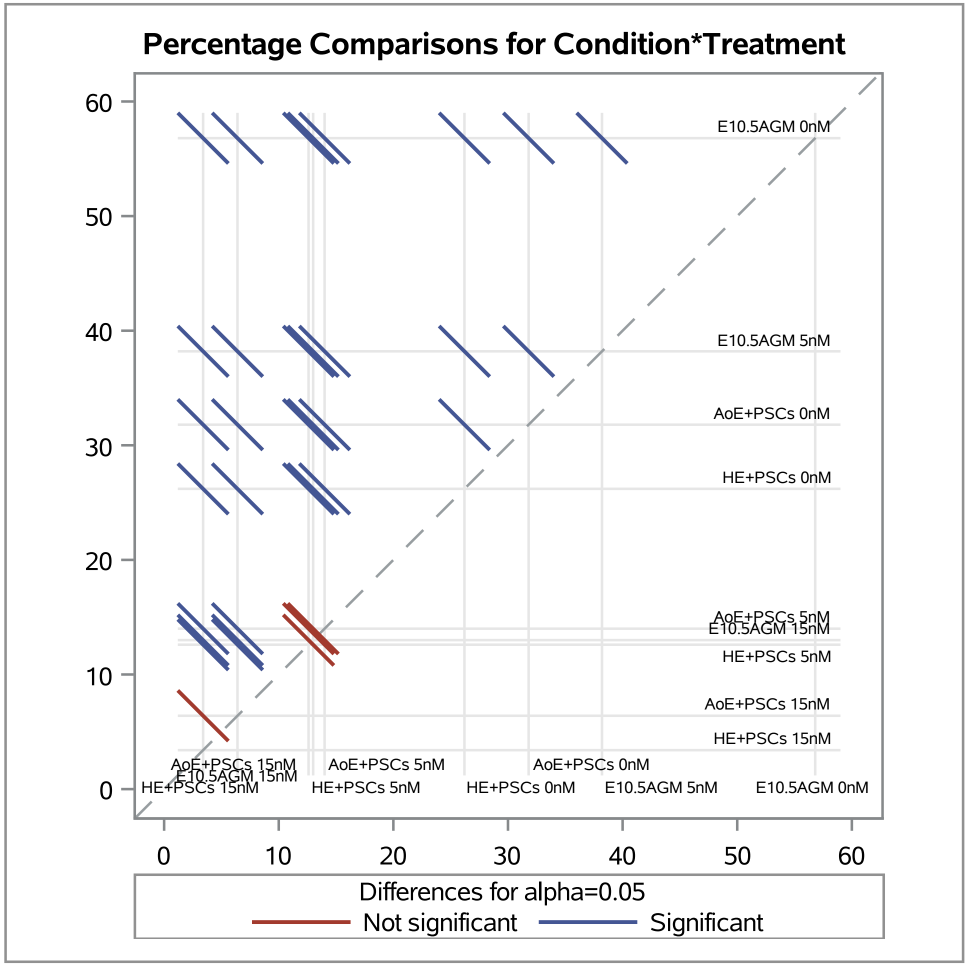

The GLIMMIX Procedure

Model Information	
**Data Set**	WORK.EXT_FIG10BII_E11LY45
**Response Variable**	Count
**Response Distribution**	Poisson
**Link Function**	Log
**Variance Function**	Default
**Variance Matrix Blocked By**	Dish_ID
**Estimation Technique**	Maximum Likelihood
**Likelihood Approximation**	Laplace
**Degrees of Freedom Method**	Containment

Class Level Information		
**Class**	**Levels**	**Values**
**Colony_Type**	3	BFU-E CFU-GEMM CFU-GM
**Condition**	3	AoE+PSCs E11.5AGM HE+PSCs
**Treatment**	3	0nM 15nM 45nM
**Dish_ID**	27	1 2 3 4 5 6 7 8 9 10 11 12 13 14 15 16 17 18 19 20 21 22 23 24 25 26 27

**Number of Observations Read**	81
**Number of Observations Used**	81

Dimensions	
**G-side Cov. Parameters**	1
**Columns in X**	64
**Columns in Z per Subject**	1
**Subjects (Blocks in V)**	27
**Max Obs per Subject**	3

The GLIMMIX Procedure

Optimization Information	
**Optimization Technique**	Dual Quasi-Newton
**Parameters in Optimization**	28
**Lower Boundaries**	1
**Upper Boundaries**	0
**Fixed Effects**	Not Profiled
**Starting From**	GLM estimates

Iteration History

Iteration	Restarts	Evaluations	Objective Function	Change	Max Gradient
**0**	**0**	4	874.41700439	.	1744.01
**1**	**0**	8	865.59627833	8.82072605	104.7053
**2**	**0**	4	865.23586598	0.36041236	66.82824
**3**	**0**	2	865.14846131	0.08740467	17.76756
**4**	**0**	4	865.09386399	0.05459732	25.26233
**5**	**0**	4	865.05287644	0.04098755	4.64876
**6**	**0**	3	865.0455937	0.00728274	5.181019
**7**	**0**	4	865.0304242	0.01516950	6.005505
**8**	**0**	2	865.01411937	0.01630483	2.323509
**9**	**0**	2	865.00384846	0.01027091	2.422635
**10**	**0**	3	864.99916117	0.00468729	2.247473
**11**	**0**	4	864.9776821	0.02147907	4.270924
**12**	**0**	3	864.97502689	0.00265521	1.448312
**13**	**0**	2	864.97374238	0.00128451	3.753434
**14**	**0**	2	864.9719554	0.00178698	1.298604
**15**	**0**	2	864.97035402	0.00160138	2.055392

The GLIMMIX Procedure

Iteration History					
Iteration	Restarts	Evaluations	Objective Function	Change	Max Gradient
16	0	3	864.96978818	0.00056584	0.690842
17	0	4	864.96865469	0.00113349	1.48421
18	0	2	864.9674143	0.00124038	0.965085
19	0	4	864.96427422	0.00314008	1.321285
20	0	2	864.96087334	0.00340088	0.891097
21	0	3	864.96044577	0.00042757	0.69641
22	0	4	864.95955793	0.00088783	0.414055
23	0	3	864.95930567	0.00025227	0.413846
24	0	2	864.95905509	0.00025057	0.711541
25	0	4	864.95851002	0.00054507	0.623936
26	0	3	864.95834459	0.00016543	0.706249
27	0	4	864.95774272	0.00060187	0.168351
28	0	3	864.95772032	0.00002240	0.178888
29	0	4	864.95735694	0.00036337	0.171883
30	0	3	864.95729151	0.00006543	0.165507
31	0	2	864.95722579	0.00006572	0.865262
32	0	2	864.9571425	0.00008330	0.185786
33	0	2	864.95710098	0.00004151	0.603478
34	0	2	864.95704243	0.00005855	0.102273
35	0	3	864.95702525	0.00001719	0.118813
36	0	4	864.95695815	0.00006710	0.296388

Convergence criterion (GCONV=1E-8) satisfied.

The GLIMMIX Procedure

Fit Statistics	
-2 Log Likelihood	864.96
AIC (smaller is better)	920.96
AICC (smaller is better)	952.19
BIC (smaller is better)	957.24
CAIC (smaller is better)	985.24
HQIC (smaller is better)	931.75

Fit Statistics for Conditional Distribution	
-2 log L(Count | r. effects)	773.68
Pearson Chi-Square	263.13
Pearson Chi-Square / DF	3.25

Covariance Parameter Estimates

Cov Parm	Subject	Estimate	Standard Error
Intercept	Dish_ID	0.02976	0.009425

The GLIMMIX Procedure

Solutions for Fixed Effects								
Effect	Colony_Type	Condition	Treatment	Estimate	Standard Error	DF	t Value	Pr > |t|
Intercept				4.2701	0.1207	18	35.37	<.0001
Colony_Type	BFU-E			-1.9550	0.1919	36	-10.19	<.0001
Colony_Type	CFU-GEMM			-1.2456	0.1430	36	-8.71	<.0001
Colony_Type	CFU-GM			0	.	.	.	.
Condition		AoE+PSCs		0.3051	0.1671	36	1.83	0.0763
Condition		E11.5AGM		0.9202	0.1624	36	5.67	<.0001
Condition		HE+PSCs		0	.	.	.	.
Colony_Typ*Condition	BFU-E	AoE+PSCs		0.4146	0.2369	36	1.75	0.0886
Colony_Typ*Condition	BFU-E	E11.5AGM		0.4663	0.2158	36	2.16	0.0375
Colony_Typ*Condition	BFU-E	HE+PSCs		0	.	.	.	.
Colony_Typ*Condition	CFU-GEMM	AoE+PSCs		-0.2948	0.1993	36	-1.48	0.1478
Colony_Typ*Condition	CFU-GEMM	E11.5AGM		-0.2432	0.1737	36	-1.40	0.1702
Colony_Typ*Condition	CFU-GEMM	HE+PSCs		0	.	.	.	.
Colony_Typ*Condition	CFU-GM	AoE+PSCs		0	.	.	.	.
Colony_Typ*Condition	CFU-GM	E11.5AGM		0	.	.	.	.
Colony_Typ*Condition	CFU-GM	HE+PSCs		0	.	.	.	.
Treatment			0nM	1.7865	0.1590	36	11.24	<.0001
Treatment			15nM	0.8537	0.1628	36	5.24	<.0001
Treatment			45nM	0	.	.	.	.
Colony_Typ*Treatment	BFU-E		0nM	0.3725	0.2035	36	1.83	0.0755
Colony_Typ*Treatment	BFU-E		15nM	0.1689	0.2242	36	0.75	0.4560
Colony_Typ*Treatment	BFU-E		45nM	0	.	.	.	.
Colony_Typ*Treatment	CFU-GEMM		0nM	-0.2568	0.1572	36	-1.63	0.1111
Colony_Typ*Treatment	CFU-GEMM		15nM	-0.4737	0.1820	36	-2.60	0.0133

The GLIMMIX Procedure

Solutions for Fixed Effects								
Effect	Colony_Type	Condition	Treatment	Estimate	Standard Error	DF	t Value	Pr > |t|
**Colony_Typ*Treatment**	CFU-GEMM		45nM	0	.	.	.	.
**Colony_Typ*Treatment**	CFU-GM		0nM	0	.	.	.	.
**Colony_Typ*Treatment**	CFU-GM		15nM	0	.	.	.	.
**Colony_Typ*Treatment**	CFU-GM		45nM	0	.	.	.	.
**Condition*Treatment**		AoE+PSCs	0nM	-0.1105	0.2218	36	-0.50	0.6213
**Condition*Treatment**		AoE+PSCs	15nM	0.06206	0.2262	36	0.27	0.7854
**Condition*Treatment**		AoE+PSCs	45nM	0	.	.	.	.
**Condition*Treatment**		E11.5AGM	0nM	-0.3089	0.2177	36	-1.42	0.1647
**Condition*Treatment**		E11.5AGM	15nM	0.002241	0.2214	36	0.01	0.9920
**Condition*Treatment**		E11.5AGM	45nM	0	.	.	.	.
**Condition*Treatment**		HE+PSCs	0nM	0	.	.	.	.
**Condition*Treatment**		HE+PSCs	15nM	0	.	.	.	.
**Condition*Treatment**		HE+PSCs	45nM	0	.	.	.	.
**Colony*Condit*Treatm**	BFU-E	AoE+PSCs	0nM	-0.5961	0.2550	36	-2.34	0.0251
**Colony*Condit*Treatm**	BFU-E	AoE+PSCs	15nM	-0.2588	0.2790	36	-0.93	0.3598
**Colony*Condit*Treatm**	BFU-E	AoE+PSCs	45nM	0	.	.	.	.
**Colony*Condit*Treatm**	BFU-E	E11.5AGM	0nM	-0.2062	0.2305	36	-0.89	0.3771
**Colony*Condit*Treatm**	BFU-E	E11.5AGM	15nM	0.1487	0.2515	36	0.59	0.5580
**Colony*Condit*Treatm**	BFU-E	E11.5AGM	45nM	0	.	.	.	.
**Colony*Condit*Treatm**	BFU-E	HE+PSCs	0nM	0	.	.	.	.
**Colony*Condit*Treatm**	BFU-E	HE+PSCs	15nM	0	.	.	.	.
**Colony*Condit*Treatm**	BFU-E	HE+PSCs	45nM	0	.	.	.	.
**Colony*Condit*Treatm**	CFU-GEMM	AoE+PSCs	0nM	0.1257	0.2192	36	0.57	0.5698
**Colony*Condit*Treatm**	CFU-GEMM	AoE+PSCs	15nM	0.4892	0.2450	36	2.00	0.0534

The GLIMMIX Procedure

Solutions for Fixed Effects								
**Effect**	**Colony_Type**	**Condition**	**Treatment**	**Estimate**	**Standard Error**	**DF**	**t Value**	**Pr > |t|**
**Colony*Condit*Treatm**	CFU-GEMM	AoE+PSCs	45nM	0	.	.	.	.
**Colony*Condit*Treatm**	CFU-GEMM	E11.5AGM	0nM	0.3035	0.1915	36	1.59	0.1217
**Colony*Condit*Treatm**	CFU-GEMM	E11.5AGM	15nM	0.8777	0.2143	36	4.10	0.0002
**Colony*Condit*Treatm**	CFU-GEMM	E11.5AGM	45nM	0	.	.	.	.
**Colony*Condit*Treatm**	CFU-GEMM	HE+PSCs	0nM	0	.	.	.	.
**Colony*Condit*Treatm**	CFU-GEMM	HE+PSCs	15nM	0	.	.	.	.
**Colony*Condit*Treatm**	CFU-GEMM	HE+PSCs	45nM	0	.	.	.	.
**Colony*Condit*Treatm**	CFU-GM	AoE+PSCs	0nM	0	.	.	.	.
**Colony*Condit*Treatm**	CFU-GM	AoE+PSCs	15nM	0	.	.	.	.
**Colony*Condit*Treatm**	CFU-GM	AoE+PSCs	45nM	0	.	.	.	.
**Colony*Condit*Treatm**	CFU-GM	E11.5AGM	0nM	0	.	.	.	.
**Colony*Condit*Treatm**	CFU-GM	E11.5AGM	15nM	0	.	.	.	.
**Colony*Condit*Treatm**	CFU-GM	E11.5AGM	45nM	0	.	.	.	.
**Colony*Condit*Treatm**	CFU-GM	HE+PSCs	0nM	0	.	.	.	.
**Colony*Condit*Treatm**	CFU-GM	HE+PSCs	15nM	0	.	.	.	.
**Colony*Condit*Treatm**	CFU-GM	HE+PSCs	45nM	0	.	.	.	.

Type III Tests of Fixed Effects				
**Effect**	**Num DF**	**Den DF**	**F Value**	**Pr > F**
**Colony_Type**	2	36	1747.40	<.0001
**Condition**	2	36	69.76	<.0001
**Colony_Typ*Condition**	4	36	10.66	<.0001
**Treatment**	2	36	165.06	<.0001
**Colony_Typ*Treatment**	4	36	1.43	0.2450

The GLIMMIX Procedure

Type III Tests of Fixed Effects				
Effect	Num DF	Den DF	F Value	Pr > F
Condition*Treatment	4	36	2.29	0.0791
Colony*Condit*Treatm	8	36	3.92	0.0021

Condition*Treatment Least Squares Means													
Condition	Treatment	Estimate	Standard Error	DF	t Value	Pr > |t|	Alpha	Lower	Upper	Mean	Standard Error Mean	Lower Mean	Upper Mean
AoE+PSCs	0nM	5.1061	0.1039	36	49.16	<.0001	0.05	4.8954	5.3167	165.02	17.1407	133.67	203.71
AoE+PSCs	15nM	4.4392	0.1075	36	41.29	<.0001	0.05	4.2212	4.6572	84.7076	9.1062	68.1139	105.34
AoE+PSCs	45nM	3.5483	0.1177	36	30.14	<.0001	0.05	3.3095	3.7871	34.7534	4.0920	27.3710	44.1269
E11.5AGM	0nM	5.7465	0.1017	36	56.50	<.0001	0.05	5.5403	5.9528	313.10	31.8443	254.74	384.83
E11.5AGM	15nM	5.2943	0.1027	36	51.53	<.0001	0.05	5.0859	5.5027	199.20	20.4654	161.73	245.34
E11.5AGM	45nM	4.1979	0.1094	36	38.36	<.0001	0.05	3.9759	4.4198	66.5433	7.2814	53.2997	83.0775
HE+PSCs	0nM	5.0284	0.1040	36	48.34	<.0001	0.05	4.8174	5.2393	152.68	15.8829	123.64	188.55
HE+PSCs	15nM	3.9553	0.1126	36	35.11	<.0001	0.05	3.7269	4.1838	52.2135	5.8816	41.5494	65.6146
HE+PSCs	45nM	3.2033	0.1260	36	25.43	<.0001	0.05	2.9478	3.4587	24.6131	3.1004	19.0641	31.7772

Differences of Condition*Treatment Least Squares Means											
Condition	Treatment	_Condition	_Treatment	Estimate	Standard Error	DF	t Value	Pr > |t|	Alpha	Lower	Upper
AoE+PSCs	0nM	AoE+PSCs	15nM	0.6669	0.1495	36	4.46	<.0001	0.05	0.3637	0.9700
AoE+PSCs	0nM	AoE+PSCs	45nM	1.5578	0.1570	36	9.92	<.0001	0.05	1.2393	1.8762
AoE+PSCs	0nM	E11.5AGM	0nM	-0.6405	0.1454	36	-4.41	<.0001	0.05	-0.9353	-0.3456
AoE+PSCs	0nM	E11.5AGM	15nM	-0.1882	0.1461	36	-1.29	0.2058	0.05	-0.4845	0.1081
AoE+PSCs	0nM	E11.5AGM	45nM	0.9082	0.1509	36	6.02	<.0001	0.05	0.6022	1.2142
AoE+PSCs	0nM	HE+PSCs	0nM	0.07769	0.1470	36	0.53	0.6004	0.05	-0.2205	0.3758

The GLIMMIX Procedure

Differences of Condition*Treatment Least Squares Means											
Condition	Treatment	_Condition	_Treatment	Estimate	Standard Error	DF	t Value	Pr > |t|	Alpha	Lower	Upper
AoE+PSCs	0nM	HE+PSCs	15nM	1.1507	0.1532	36	7.51	<.0001	0.05	0.8400	1.4615
AoE+PSCs	0nM	HE+PSCs	45nM	1.9028	0.1633	36	11.65	<.0001	0.05	1.5717	2.2339
AoE+PSCs	15nM	AoE+PSCs	45nM	0.8909	0.1594	36	5.59	<.0001	0.05	0.5676	1.2143
AoE+PSCs	15nM	E11.5AGM	0nM	-1.3073	0.1480	36	-8.83	<.0001	0.05	-1.6075	-1.0072
AoE+PSCs	15nM	E11.5AGM	15nM	-0.8551	0.1487	36	-5.75	<.0001	0.05	-1.1567	-0.5535
AoE+PSCs	15nM	E11.5AGM	45nM	0.2414	0.1534	36	1.57	0.1243	0.05	-0.06973	0.5524
AoE+PSCs	15nM	HE+PSCs	0nM	-0.5892	0.1496	36	-3.94	0.0004	0.05	-0.8926	-0.2858
AoE+PSCs	15nM	HE+PSCs	15nM	0.4839	0.1557	36	3.11	0.0037	0.05	0.1681	0.7996
AoE+PSCs	15nM	HE+PSCs	45nM	1.2359	0.1656	36	7.46	<.0001	0.05	0.9001	1.5718
AoE+PSCs	45nM	E11.5AGM	0nM	-2.1983	0.1556	36	-14.13	<.0001	0.05	-2.5138	-1.8827
AoE+PSCs	45nM	E11.5AGM	15nM	-1.7460	0.1563	36	-11.17	<.0001	0.05	-2.0629	-1.4291
AoE+PSCs	45nM	E11.5AGM	45nM	-0.6496	0.1607	36	-4.04	0.0003	0.05	-0.9755	-0.3236
AoE+PSCs	45nM	HE+PSCs	0nM	-1.4801	0.1571	36	-9.42	<.0001	0.05	-1.7987	-1.1615
AoE+PSCs	45nM	HE+PSCs	15nM	-0.4071	0.1629	36	-2.50	0.0172	0.05	-0.7375	-0.07662
AoE+PSCs	45nM	HE+PSCs	45nM	0.3450	0.1724	36	2.00	0.0530	0.05	-0.00466	0.6947
E11.5AGM	0nM	E11.5AGM	15nM	0.4522	0.1446	36	3.13	0.0035	0.05	0.1590	0.7454
E11.5AGM	0nM	E11.5AGM	45nM	1.5487	0.1494	36	10.37	<.0001	0.05	1.2457	1.8516
E11.5AGM	0nM	HE+PSCs	0nM	0.7182	0.1455	36	4.94	<.0001	0.05	0.4231	1.0132
E11.5AGM	0nM	HE+PSCs	15nM	1.7912	0.1518	36	11.80	<.0001	0.05	1.4834	2.0990
E11.5AGM	0nM	HE+PSCs	45nM	2.5433	0.1619	36	15.71	<.0001	0.05	2.2149	2.8716
E11.5AGM	15nM	E11.5AGM	45nM	1.0964	0.1501	36	7.31	<.0001	0.05	0.7920	1.4008
E11.5AGM	15nM	HE+PSCs	0nM	0.2659	0.1462	36	1.82	0.0773	0.05	-0.03060	0.5624
E11.5AGM	15nM	HE+PSCs	15nM	1.3390	0.1525	36	8.78	<.0001	0.05	1.0298	1.6481
E11.5AGM	15nM	HE+PSCs	45nM	2.0910	0.1625	36	12.86	<.0001	0.05	1.7614	2.4207

The GLIMMIX Procedure

Differences of Condition*Treatment Least Squares Means											
Condition	Treatment	_Condition	_Treatment	Estimate	Standard Error	DF	t Value	Pr > |t|	Alpha	Lower	Upper
E11.5AGM	45nM	HE+PSCs	0nM	-0.8305	0.1510	36	-5.50	<.0001	0.05	-1.1367	-0.5243
E11.5AGM	45nM	HE+PSCs	15nM	0.2425	0.1570	36	1.54	0.1312	0.05	-0.07590	0.5609
E11.5AGM	45nM	HE+PSCs	45nM	0.9946	0.1668	36	5.96	<.0001	0.05	0.6563	1.3329
HE+PSCs	0nM	HE+PSCs	15nM	1.0730	0.1533	36	7.00	<.0001	0.05	0.7621	1.3840
HE+PSCs	0nM	HE+PSCs	45nM	1.8251	0.1634	36	11.17	<.0001	0.05	1.4938	2.1564
HE+PSCs	15nM	HE+PSCs	45nM	0.7521	0.1689	36	4.45	<.0001	0.05	0.4094	1.0947

Colony*Condit*Treatm Least Squares Means														
Colony_Type	Condition	Treatment	Estimate	Standard Error	DF	t Value	Pr > |t|	Alpha	Lower	Upper	Mean	Standard Error Mean	Lower Mean	Upper Mean
BFU-E	AoE+PSCs	0nM	4.4873	0.1169	36	38.40	<.0001	0.05	4.2502	4.7243	88.8772	10.3864	70.1228	112.65
BFU-E	AoE+PSCs	15nM	3.8607	0.1299	36	29.71	<.0001	0.05	3.5971	4.1242	47.4974	6.1718	36.4938	61.8187
BFU-E	AoE+PSCs	45nM	3.0348	0.1607	36	18.88	<.0001	0.05	2.7089	3.3608	20.7972	3.3423	15.0125	28.8108
BFU-E	E11.5AGM	0nM	5.3456	0.1073	36	49.83	<.0001	0.05	5.1281	5.5632	209.69	22.4957	168.69	260.66
BFU-E	E11.5AGM	15nM	4.8752	0.1115	36	43.71	<.0001	0.05	4.6490	5.1014	131.00	14.6119	104.48	164.26
BFU-E	E11.5AGM	45nM	3.7016	0.1340	36	27.63	<.0001	0.05	3.4299	3.9733	40.5133	5.4279	30.8739	53.1623
BFU-E	HE+PSCs	0nM	4.4742	0.1171	36	38.21	<.0001	0.05	4.2367	4.7117	87.7245	10.2727	69.1797	111.24
BFU-E	HE+PSCs	15nM	3.3378	0.1466	36	22.76	<.0001	0.05	3.0404	3.6351	28.1559	4.1282	20.9136	37.9062
BFU-E	HE+PSCs	45nM	2.3152	0.2056	36	11.26	<.0001	0.05	1.8981	2.7322	10.1266	2.0823	6.6734	15.3666
CFU-GEMM	AoE+PSCs	0nM	4.5797	0.1154	36	39.67	<.0001	0.05	4.3456	4.8138	97.4867	11.2542	77.1371	123.20
CFU-GEMM	AoE+PSCs	15nM	3.9660	0.1272	36	31.17	<.0001	0.05	3.7080	4.2241	52.7756	6.7151	40.7720	68.3132
CFU-GEMM	AoE+PSCs	45nM	3.0348	0.1607	36	18.88	<.0001	0.05	2.7089	3.3608	20.7975	3.3424	15.0127	28.8113
CFU-GEMM	E11.5AGM	0nM	5.2260	0.1082	36	48.29	<.0001	0.05	5.0065	5.4454	186.04	20.1326	149.38	231.70
CFU-GEMM	E11.5AGM	15nM	4.9614	0.1106	36	44.86	<.0001	0.05	4.7371	5.1858	142.80	15.7948	114.10	178.71

The GLIMMIX Procedure

Colony*Condit*Treatm Least Squares Means														
Colony_Type	Condition	Treatment	Estimate	Standard Error	DF	t Value	Pr > |t|	Alpha	Lower	Upper	Mean	Standard Error Mean	Lower Mean	Upper Mean
CFU-GEMM	E11.5AGM	45nM	3.7016	0.1340	36	27.63	<.0001	0.05	3.4299	3.9733	40.5117	5.4277	30.8726	53.1603
CFU-GEMM	HE+PSCs	0nM	4.5542	0.1159	36	39.31	<.0001	0.05	4.3193	4.7892	95.0348	11.0099	75.1350	120.21
CFU-GEMM	HE+PSCs	15nM	3.4045	0.1441	36	23.63	<.0001	0.05	3.1123	3.6967	30.0988	4.3362	22.4727	40.3127
CFU-GEMM	HE+PSCs	45nM	3.0245	0.1609	36	18.80	<.0001	0.05	2.6982	3.3508	20.5846	3.3119	14.8535	28.5269
CFU-GM	AoE+PSCs	0nM	6.2512	0.1028	36	60.82	<.0001	0.05	6.0427	6.4597	518.63	53.3085	421.04	638.84
CFU-GM	AoE+PSCs	15nM	5.4909	0.1063	36	51.65	<.0001	0.05	5.2753	5.7065	242.47	25.7750	195.45	300.81
CFU-GM	AoE+PSCs	45nM	4.5752	0.1156	36	39.58	<.0001	0.05	4.3408	4.8096	97.0458	11.2170	76.7664	122.68
CFU-GM	E11.5AGM	0nM	6.6680	0.1017	36	65.56	<.0001	0.05	6.4617	6.8743	786.83	80.0282	640.17	967.08
CFU-GM	E11.5AGM	15nM	6.0462	0.1035	36	58.42	<.0001	0.05	5.8363	6.2561	422.52	43.7303	342.52	521.20
CFU-GM	E11.5AGM	45nM	5.1903	0.1086	36	47.78	<.0001	0.05	4.9700	5.4107	179.53	19.5032	144.03	223.78
CFU-GM	HE+PSCs	0nM	6.0567	0.1035	36	58.54	<.0001	0.05	5.8468	6.2665	426.95	44.1714	346.14	526.62
CFU-GM	HE+PSCs	15nM	5.1238	0.1092	36	46.91	<.0001	0.05	4.9023	5.3453	167.97	18.3464	134.59	209.62
CFU-GM	HE+PSCs	45nM	4.2701	0.1207	36	35.37	<.0001	0.05	4.0253	4.5150	71.5309	8.6368	55.9945	91.3781

The GLIMMIX Procedure

Differences of Colony*Condit*Treatm Least Squares Means													
Colony_Type	Condition	Treatment	_Colony_Type	_Condition	_Treatment	Estimate	Standard Error	DF	t Value	Pr > |t|	Alpha	Lower	Upper
BFU-E	AoE+PSCs	0nM	BFU-E	AoE+PSCs	15nM	0.6266	0.1748	36	3.59	0.0010	0.05	0.2722	0.9810
BFU-E	AoE+PSCs	0nM	BFU-E	AoE+PSCs	45nM	1.4524	0.1987	36	7.31	<.0001	0.05	1.0494	1.8554
BFU-E	AoE+PSCs	0nM	BFU-E	E11.5AGM	0nM	-0.8584	0.1586	36	-5.41	<.0001	0.05	-1.1801	-0.5366
BFU-E	AoE+PSCs	0nM	BFU-E	E11.5AGM	15nM	-0.3880	0.1615	36	-2.40	0.0216	0.05	-0.7156	-0.06032
BFU-E	AoE+PSCs	0nM	BFU-E	E11.5AGM	45nM	0.7856	0.1778	36	4.42	<.0001	0.05	0.4251	1.1462
BFU-E	AoE+PSCs	0nM	BFU-E	HE+PSCs	0nM	0.01305	0.1654	36	0.08	0.9375	0.05	-0.3225	0.3486
BFU-E	AoE+PSCs	0nM	BFU-E	HE+PSCs	15nM	1.1495	0.1875	36	6.13	<.0001	0.05	0.7692	1.5297
BFU-E	AoE+PSCs	0nM	BFU-E	HE+PSCs	45nM	2.1721	0.2365	36	9.18	<.0001	0.05	1.6924	2.6518
BFU-E	AoE+PSCs	0nM	CFU-GEMM	AoE+PSCs	0nM	-0.09246	0.08446	36	-1.09	0.2809	0.05	-0.2637	0.07882
BFU-E	AoE+PSCs	0nM	CFU-GEMM	AoE+PSCs	15nM	0.5212	0.1728	36	3.02	0.0047	0.05	0.1708	0.8716
BFU-E	AoE+PSCs	0nM	CFU-GEMM	AoE+PSCs	45nM	1.4524	0.1987	36	7.31	<.0001	0.05	1.0494	1.8554
BFU-E	AoE+PSCs	0nM	CFU-GEMM	E11.5AGM	0nM	-0.7387	0.1593	36	-4.64	<.0001	0.05	-1.0617	-0.4157
BFU-E	AoE+PSCs	0nM	CFU-GEMM	E11.5AGM	15nM	-0.4742	0.1609	36	-2.95	0.0056	0.05	-0.8005	-0.1479
BFU-E	AoE+PSCs	0nM	CFU-GEMM	E11.5AGM	45nM	0.7857	0.1778	36	4.42	<.0001	0.05	0.4251	1.1462
BFU-E	AoE+PSCs	0nM	CFU-GEMM	HE+PSCs	0nM	-0.06699	0.1646	36	-0.41	0.6864	0.05	-0.4007	0.2667
BFU-E	AoE+PSCs	0nM	CFU-GEMM	HE+PSCs	15nM	1.0828	0.1855	36	5.84	<.0001	0.05	0.7066	1.4590
BFU-E	AoE+PSCs	0nM	CFU-GEMM	HE+PSCs	45nM	1.4627	0.1989	36	7.36	<.0001	0.05	1.0594	1.8660
BFU-E	AoE+PSCs	0nM	CFU-GM	AoE+PSCs	0nM	-1.7639	0.06611	36	-26.68	<.0001	0.05	-1.8980	-1.6299
BFU-E	AoE+PSCs	0nM	CFU-GM	AoE+PSCs	15nM	-1.0036	0.1580	36	-6.35	<.0001	0.05	-1.3240	-0.6832
BFU-E	AoE+PSCs	0nM	CFU-GM	AoE+PSCs	45nM	-0.08793	0.1644	36	-0.53	0.5960	0.05	-0.4213	0.2454
BFU-E	AoE+PSCs	0nM	CFU-GM	E11.5AGM	0nM	-2.1808	0.1549	36	-14.08	<.0001	0.05	-2.4950	-1.8666
BFU-E	AoE+PSCs	0nM	CFU-GM	E11.5AGM	15nM	-1.5590	0.1561	36	-9.99	<.0001	0.05	-1.8756	-1.2424
BFU-E	AoE+PSCs	0nM	CFU-GM	E11.5AGM	45nM	-0.7031	0.1596	36	-4.41	<.0001	0.05	-1.0267	-0.3795
BFU-E	AoE+PSCs	0nM	CFU-GM	HE+PSCs	0nM	-1.5694	0.1561	36	-10.06	<.0001	0.05	-1.8860	-1.2529

The GLIMMIX Procedure

Differences of Colony*Condit*Treatm Least Squares Means													
Colony_Type	Condition	Treatment	_Colony_Type	_Condition	_Treatment	Estimate	Standard Error	DF	t Value	Pr > |t|	Alpha	Lower	Upper
BFU-E	AoE+PSCs	0nM	CFU-GM	HE+PSCs	15nM	-0.6365	0.1600	36	-3.98	0.0003	0.05	-0.9609	-0.3121
BFU-E	AoE+PSCs	0nM	CFU-GM	HE+PSCs	45nM	0.2171	0.1680	36	1.29	0.2045	0.05	-0.1237	0.5579
BFU-E	AoE+PSCs	15nM	BFU-E	AoE+PSCs	45nM	0.8259	0.2067	36	4.00	0.0003	0.05	0.4067	1.2450
BFU-E	AoE+PSCs	15nM	BFU-E	E11.5AGM	0nM	-1.4850	0.1685	36	-8.81	<.0001	0.05	-1.8267	-1.1432
BFU-E	AoE+PSCs	15nM	BFU-E	E11.5AGM	15nM	-1.0145	0.1712	36	-5.92	<.0001	0.05	-1.3618	-0.6672
BFU-E	AoE+PSCs	15nM	BFU-E	E11.5AGM	45nM	0.1590	0.1866	36	0.85	0.3997	0.05	-0.2195	0.5375
BFU-E	AoE+PSCs	15nM	BFU-E	HE+PSCs	0nM	-0.6135	0.1749	36	-3.51	0.0012	0.05	-0.9683	-0.2588
BFU-E	AoE+PSCs	15nM	BFU-E	HE+PSCs	15nM	0.5229	0.1959	36	2.67	0.0113	0.05	0.1256	0.9202
BFU-E	AoE+PSCs	15nM	BFU-E	HE+PSCs	45nM	1.5455	0.2432	36	6.35	<.0001	0.05	1.0522	2.0388
BFU-E	AoE+PSCs	15nM	CFU-GEMM	AoE+PSCs	0nM	-0.7190	0.1738	36	-4.14	0.0002	0.05	-1.0716	-0.3665
BFU-E	AoE+PSCs	15nM	CFU-GEMM	AoE+PSCs	15nM	-0.1054	0.1149	36	-0.92	0.3651	0.05	-0.3383	0.1276
BFU-E	AoE+PSCs	15nM	CFU-GEMM	AoE+PSCs	45nM	0.8258	0.2067	36	4.00	0.0003	0.05	0.4067	1.2450
BFU-E	AoE+PSCs	15nM	CFU-GEMM	E11.5AGM	0nM	-1.3653	0.1691	36	-8.07	<.0001	0.05	-1.7082	-1.0223
BFU-E	AoE+PSCs	15nM	CFU-GEMM	E11.5AGM	15nM	-1.1008	0.1706	36	-6.45	<.0001	0.05	-1.4468	-0.7547
BFU-E	AoE+PSCs	15nM	CFU-GEMM	E11.5AGM	45nM	0.1591	0.1866	36	0.85	0.3996	0.05	-0.2194	0.5376
BFU-E	AoE+PSCs	15nM	CFU-GEMM	HE+PSCs	0nM	-0.6936	0.1741	36	-3.98	0.0003	0.05	-1.0466	-0.3405
BFU-E	AoE+PSCs	15nM	CFU-GEMM	HE+PSCs	15nM	0.4562	0.1940	36	2.35	0.0243	0.05	0.06274	0.8496
BFU-E	AoE+PSCs	15nM	CFU-GEMM	HE+PSCs	45nM	0.8361	0.2068	36	4.04	0.0003	0.05	0.4167	1.2555
BFU-E	AoE+PSCs	15nM	CFU-GM	AoE+PSCs	0nM	-2.3905	0.1657	36	-14.43	<.0001	0.05	-2.7265	-2.0545
BFU-E	AoE+PSCs	15nM	CFU-GM	AoE+PSCs	15nM	-1.6302	0.09114	36	-17.89	<.0001	0.05	-1.8150	-1.4454
BFU-E	AoE+PSCs	15nM	CFU-GM	AoE+PSCs	45nM	-0.7145	0.1739	36	-4.11	0.0002	0.05	-1.0672	-0.3618
BFU-E	AoE+PSCs	15nM	CFU-GM	E11.5AGM	0nM	-2.8073	0.1650	36	-17.01	<.0001	0.05	-3.1420	-2.4727
BFU-E	AoE+PSCs	15nM	CFU-GM	E11.5AGM	15nM	-2.1856	0.1661	36	-13.16	<.0001	0.05	-2.5225	-1.8486
BFU-E	AoE+PSCs	15nM	CFU-GM	E11.5AGM	45nM	-1.3297	0.1694	36	-7.85	<.0001	0.05	-1.6731	-0.9862

The GLIMMIX Procedure

Differences of Colony*Condit*Treatm Least Squares Means													
Colony_Type	Condition	Treatment	_Colony_Type	_Condition	_Treatment	Estimate	Standard Error	DF	t Value	Pr > |t|	Alpha	Lower	Upper
BFU-E	AoE+PSCs	15nM	CFU-GM	HE+PSCs	0nM	-2.1960	0.1661	36	-13.22	<.0001	0.05	-2.5329	-1.8591
BFU-E	AoE+PSCs	15nM	CFU-GM	HE+PSCs	15nM	-1.2631	0.1697	36	-7.44	<.0001	0.05	-1.6074	-0.9189
BFU-E	AoE+PSCs	15nM	CFU-GM	HE+PSCs	45nM	-0.4095	0.1774	36	-2.31	0.0268	0.05	-0.7692	-0.04974
BFU-E	AoE+PSCs	45nM	BFU-E	E11.5AGM	0nM	-2.3108	0.1932	36	-11.96	<.0001	0.05	-2.7027	-1.9189
BFU-E	AoE+PSCs	45nM	BFU-E	E11.5AGM	15nM	-1.8404	0.1956	36	-9.41	<.0001	0.05	-2.2371	-1.4436
BFU-E	AoE+PSCs	45nM	BFU-E	E11.5AGM	45nM	-0.6668	0.2092	36	-3.19	0.0030	0.05	-1.0911	-0.2425
BFU-E	AoE+PSCs	45nM	BFU-E	HE+PSCs	0nM	-1.4394	0.1988	36	-7.24	<.0001	0.05	-1.8427	-1.0361
BFU-E	AoE+PSCs	45nM	BFU-E	HE+PSCs	15nM	-0.3029	0.2175	36	-1.39	0.1723	0.05	-0.7441	0.1382
BFU-E	AoE+PSCs	45nM	BFU-E	HE+PSCs	45nM	0.7197	0.2610	36	2.76	0.0091	0.05	0.1904	1.2489
BFU-E	AoE+PSCs	45nM	CFU-GEMM	AoE+PSCs	0nM	-1.5449	0.1979	36	-7.81	<.0001	0.05	-1.9462	-1.1436
BFU-E	AoE+PSCs	45nM	CFU-GEMM	AoE+PSCs	15nM	-0.9312	0.2050	36	-4.54	<.0001	0.05	-1.3470	-0.5155
BFU-E	AoE+PSCs	45nM	CFU-GEMM	AoE+PSCs	45nM	-0.00002	0.1781	36	-0.00	0.9999	0.05	-0.3613	0.3613
BFU-E	AoE+PSCs	45nM	CFU-GEMM	E11.5AGM	0nM	-2.1911	0.1937	36	-11.31	<.0001	0.05	-2.5841	-1.7982
BFU-E	AoE+PSCs	45nM	CFU-GEMM	E11.5AGM	15nM	-1.9266	0.1951	36	-9.88	<.0001	0.05	-2.3223	-1.5310
BFU-E	AoE+PSCs	45nM	CFU-GEMM	E11.5AGM	45nM	-0.6668	0.2092	36	-3.19	0.0030	0.05	-1.0911	-0.2425
BFU-E	AoE+PSCs	45nM	CFU-GEMM	HE+PSCs	0nM	-1.5194	0.1981	36	-7.67	<.0001	0.05	-1.9212	-1.1176
BFU-E	AoE+PSCs	45nM	CFU-GEMM	HE+PSCs	15nM	-0.3697	0.2158	36	-1.71	0.0953	0.05	-0.8074	0.06804
BFU-E	AoE+PSCs	45nM	CFU-GEMM	HE+PSCs	45nM	0.01027	0.2274	36	0.05	0.9642	0.05	-0.4509	0.4715
BFU-E	AoE+PSCs	45nM	CFU-GM	AoE+PSCs	0nM	-3.2164	0.1908	36	-16.86	<.0001	0.05	-3.6033	-2.8295
BFU-E	AoE+PSCs	45nM	CFU-GM	AoE+PSCs	15nM	-2.4561	0.1927	36	-12.75	<.0001	0.05	-2.8469	-2.0653
BFU-E	AoE+PSCs	45nM	CFU-GM	AoE+PSCs	45nM	-1.5404	0.1388	36	-11.10	<.0001	0.05	-1.8219	-1.2588
BFU-E	AoE+PSCs	45nM	CFU-GM	E11.5AGM	0nM	-3.6332	0.1902	36	-19.10	<.0001	0.05	-4.0189	-3.2475
BFU-E	AoE+PSCs	45nM	CFU-GM	E11.5AGM	15nM	-3.0114	0.1912	36	-15.75	<.0001	0.05	-3.3991	-2.6237
BFU-E	AoE+PSCs	45nM	CFU-GM	E11.5AGM	45nM	-2.1555	0.1940	36	-11.11	<.0001	0.05	-2.5489	-1.7621

The GLIMMIX Procedure

Differences of Colony*Condit*Treatm Least Squares Means													
Colony_Type	Condition	Treatment	_Colony_Type	_Condition	_Treatment	Estimate	Standard Error	DF	t Value	Pr > |t|	Alpha	Lower	Upper
BFU-E	AoE+PSCs	45nM	CFU-GM	HE+PSCs	0nM	-3.0218	0.1911	36	-15.81	<.0001	0.05	-3.4095	-2.6342
BFU-E	AoE+PSCs	45nM	CFU-GM	HE+PSCs	15nM	-2.0890	0.1943	36	-10.75	<.0001	0.05	-2.4830	-1.6949
BFU-E	AoE+PSCs	45nM	CFU-GM	HE+PSCs	45nM	-1.2353	0.2010	36	-6.15	<.0001	0.05	-1.6430	-0.8277
BFU-E	E11.5AGM	0nM	BFU-E	E11.5AGM	15nM	0.4704	0.1548	36	3.04	0.0044	0.05	0.1566	0.7843
BFU-E	E11.5AGM	0nM	BFU-E	E11.5AGM	45nM	1.6440	0.1716	36	9.58	<.0001	0.05	1.2959	1.9921
BFU-E	E11.5AGM	0nM	BFU-E	HE+PSCs	0nM	0.8714	0.1588	36	5.49	<.0001	0.05	0.5493	1.1935
BFU-E	E11.5AGM	0nM	BFU-E	HE+PSCs	15nM	2.0079	0.1817	36	11.05	<.0001	0.05	1.6394	2.3763
BFU-E	E11.5AGM	0nM	BFU-E	HE+PSCs	45nM	3.0305	0.2319	36	13.07	<.0001	0.05	2.5601	3.5008
BFU-E	E11.5AGM	0nM	CFU-GEMM	AoE+PSCs	0nM	0.7659	0.1576	36	4.86	<.0001	0.05	0.4463	1.0855
BFU-E	E11.5AGM	0nM	CFU-GEMM	AoE+PSCs	15nM	1.3796	0.1664	36	8.29	<.0001	0.05	1.0420	1.7171
BFU-E	E11.5AGM	0nM	CFU-GEMM	AoE+PSCs	45nM	2.3108	0.1932	36	11.96	<.0001	0.05	1.9189	2.7027
BFU-E	E11.5AGM	0nM	CFU-GEMM	E11.5AGM	0nM	0.1197	0.05811	36	2.06	0.0467	0.05	0.001827	0.2375
BFU-E	E11.5AGM	0nM	CFU-GEMM	E11.5AGM	15nM	0.3842	0.1541	36	2.49	0.0174	0.05	0.07168	0.6967
BFU-E	E11.5AGM	0nM	CFU-GEMM	E11.5AGM	45nM	1.6440	0.1716	36	9.58	<.0001	0.05	1.2959	1.9921
BFU-E	E11.5AGM	0nM	CFU-GEMM	HE+PSCs	0nM	0.7914	0.1579	36	5.01	<.0001	0.05	0.4712	1.1116
BFU-E	E11.5AGM	0nM	CFU-GEMM	HE+PSCs	15nM	1.9411	0.1796	36	10.81	<.0001	0.05	1.5769	2.3054
BFU-E	E11.5AGM	0nM	CFU-GEMM	HE+PSCs	45nM	2.3211	0.1934	36	12.00	<.0001	0.05	1.9289	2.7133
BFU-E	E11.5AGM	0nM	CFU-GM	AoE+PSCs	0nM	-0.9056	0.1486	36	-6.10	<.0001	0.05	-1.2069	-0.6042
BFU-E	E11.5AGM	0nM	CFU-GM	AoE+PSCs	15nM	-0.1453	0.1510	36	-0.96	0.3425	0.05	-0.4516	0.1610
BFU-E	E11.5AGM	0nM	CFU-GM	AoE+PSCs	45nM	0.7704	0.1577	36	4.89	<.0001	0.05	0.4506	1.0903
BFU-E	E11.5AGM	0nM	CFU-GM	E11.5AGM	0nM	-1.3224	0.04484	36	-29.49	<.0001	0.05	-1.4133	-1.2314
BFU-E	E11.5AGM	0nM	CFU-GM	E11.5AGM	15nM	-0.7006	0.1491	36	-4.70	<.0001	0.05	-1.0029	-0.3983
BFU-E	E11.5AGM	0nM	CFU-GM	E11.5AGM	45nM	0.1553	0.1527	36	1.02	0.3159	0.05	-0.1543	0.4649
BFU-E	E11.5AGM	0nM	CFU-GM	HE+PSCs	0nM	-0.7110	0.1490	36	-4.77	<.0001	0.05	-1.0133	-0.4088

The GLIMMIX Procedure

Differences of Colony*Condit*Treatm Least Squares Means													
Colony_Type	Condition	Treatment	_Colony_Type	_Condition	_Treatment	Estimate	Standard Error	DF	t Value	Pr > |t|	Alpha	Lower	Upper
BFU-E	E11.5AGM	0nM	CFU-GM	HE+PSCs	15nM	0.2218	0.1531	36	1.45	0.1560	0.05	-0.08865	0.5323
BFU-E	E11.5AGM	0nM	CFU-GM	HE+PSCs	45nM	1.0755	0.1615	36	6.66	<.0001	0.05	0.7479	1.4031
BFU-E	E11.5AGM	15nM	BFU-E	E11.5AGM	45nM	1.1736	0.1743	36	6.73	<.0001	0.05	0.8200	1.5271
BFU-E	E11.5AGM	15nM	BFU-E	HE+PSCs	0nM	0.4010	0.1617	36	2.48	0.0180	0.05	0.07302	0.7290
BFU-E	E11.5AGM	15nM	BFU-E	HE+PSCs	15nM	1.5374	0.1842	36	8.35	<.0001	0.05	1.1638	1.9111
BFU-E	E11.5AGM	15nM	BFU-E	HE+PSCs	45nM	2.5600	0.2339	36	10.94	<.0001	0.05	2.0856	3.0345
BFU-E	E11.5AGM	15nM	CFU-GEMM	AoE+PSCs	0nM	0.2955	0.1605	36	1.84	0.0739	0.05	-0.03007	0.6210
BFU-E	E11.5AGM	15nM	CFU-GEMM	AoE+PSCs	15nM	0.9092	0.1692	36	5.37	<.0001	0.05	0.5660	1.2523
BFU-E	E11.5AGM	15nM	CFU-GEMM	AoE+PSCs	45nM	1.8404	0.1956	36	9.41	<.0001	0.05	1.4436	2.2371
BFU-E	E11.5AGM	15nM	CFU-GEMM	E11.5AGM	0nM	-0.3507	0.1554	36	-2.26	0.0302	0.05	-0.6659	-0.03556
BFU-E	E11.5AGM	15nM	CFU-GEMM	E11.5AGM	15nM	-0.08624	0.06932	36	-1.24	0.2215	0.05	-0.2268	0.05435
BFU-E	E11.5AGM	15nM	CFU-GEMM	E11.5AGM	45nM	1.1736	0.1743	36	6.73	<.0001	0.05	0.8201	1.5272
BFU-E	E11.5AGM	15nM	CFU-GEMM	HE+PSCs	0nM	0.3210	0.1608	36	2.00	0.0536	0.05	-0.00520	0.6471
BFU-E	E11.5AGM	15nM	CFU-GEMM	HE+PSCs	15nM	1.4707	0.1822	36	8.07	<.0001	0.05	1.1012	1.8402
BFU-E	E11.5AGM	15nM	CFU-GEMM	HE+PSCs	45nM	1.8507	0.1958	36	9.45	<.0001	0.05	1.4536	2.2477
BFU-E	E11.5AGM	15nM	CFU-GM	AoE+PSCs	0nM	-1.3760	0.1517	36	-9.07	<.0001	0.05	-1.6836	-1.0684
BFU-E	E11.5AGM	15nM	CFU-GM	AoE+PSCs	15nM	-0.6157	0.1541	36	-4.00	0.0003	0.05	-0.9282	-0.3032
BFU-E	E11.5AGM	15nM	CFU-GM	AoE+PSCs	45nM	0.3000	0.1606	36	1.87	0.0699	0.05	-0.02574	0.6258
BFU-E	E11.5AGM	15nM	CFU-GM	E11.5AGM	0nM	-1.7928	0.1510	36	-11.88	<.0001	0.05	-2.0989	-1.4867
BFU-E	E11.5AGM	15nM	CFU-GM	E11.5AGM	15nM	-1.1710	0.05730	36	-20.44	<.0001	0.05	-1.2872	-1.0548
BFU-E	E11.5AGM	15nM	CFU-GM	E11.5AGM	45nM	-0.3151	0.1557	36	-2.02	0.0504	0.05	-0.6309	0.000628
BFU-E	E11.5AGM	15nM	CFU-GM	HE+PSCs	0nM	-1.1815	0.1521	36	-7.77	<.0001	0.05	-1.4900	-0.8729
BFU-E	E11.5AGM	15nM	CFU-GM	HE+PSCs	15nM	-0.2486	0.1561	36	-1.59	0.1200	0.05	-0.5652	0.06802
BFU-E	E11.5AGM	15nM	CFU-GM	HE+PSCs	45nM	0.6051	0.1644	36	3.68	0.0008	0.05	0.2717	0.9384

The GLIMMIX Procedure

Differences of Colony*Condit*Treatm Least Squares Means													
Colony_Type	Condition	Treatment	_Colony_Type	_Condition	_Treatment	Estimate	Standard Error	DF	t Value	Pr > |t|	Alpha	Lower	Upper
BFU-E	E11.5AGM	45nM	BFU-E	HE+PSCs	0nM	-0.7726	0.1779	36	-4.34	0.0001	0.05	-1.1335	-0.4117
BFU-E	E11.5AGM	45nM	BFU-E	HE+PSCs	15nM	0.3639	0.1986	36	1.83	0.0752	0.05	-0.03886	0.7666
BFU-E	E11.5AGM	45nM	BFU-E	HE+PSCs	45nM	1.3865	0.2454	36	5.65	<.0001	0.05	0.8888	1.8841
BFU-E	E11.5AGM	45nM	CFU-GEMM	AoE+PSCs	0nM	-0.8781	0.1768	36	-4.97	<.0001	0.05	-1.2368	-0.5194
BFU-E	E11.5AGM	45nM	CFU-GEMM	AoE+PSCs	15nM	-0.2644	0.1848	36	-1.43	0.1610	0.05	-0.6391	0.1103
BFU-E	E11.5AGM	45nM	CFU-GEMM	AoE+PSCs	45nM	0.6668	0.2092	36	3.19	0.0030	0.05	0.2425	1.0911
BFU-E	E11.5AGM	45nM	CFU-GEMM	E11.5AGM	0nM	-1.5243	0.1722	36	-8.85	<.0001	0.05	-1.8736	-1.1750
BFU-E	E11.5AGM	45nM	CFU-GEMM	E11.5AGM	15nM	-1.2598	0.1737	36	-7.25	<.0001	0.05	-1.6121	-0.9075
BFU-E	E11.5AGM	45nM	CFU-GEMM	E11.5AGM	45nM	0.000040	0.1260	36	0.00	0.9997	0.05	-0.2555	0.2556
BFU-E	E11.5AGM	45nM	CFU-GEMM	HE+PSCs	0nM	-0.8526	0.1771	36	-4.81	<.0001	0.05	-1.2118	-0.4934
BFU-E	E11.5AGM	45nM	CFU-GEMM	HE+PSCs	15nM	0.2971	0.1967	36	1.51	0.1396	0.05	-0.1018	0.6961
BFU-E	E11.5AGM	45nM	CFU-GEMM	HE+PSCs	45nM	0.6771	0.2093	36	3.23	0.0026	0.05	0.2525	1.1016
BFU-E	E11.5AGM	45nM	CFU-GM	AoE+PSCs	0nM	-2.5496	0.1689	36	-15.10	<.0001	0.05	-2.8920	-2.2071
BFU-E	E11.5AGM	45nM	CFU-GM	AoE+PSCs	15nM	-1.7893	0.1710	36	-10.46	<.0001	0.05	-2.1361	-1.4424
BFU-E	E11.5AGM	45nM	CFU-GM	AoE+PSCs	45nM	-0.8736	0.1769	36	-4.94	<.0001	0.05	-1.2324	-0.5147
BFU-E	E11.5AGM	45nM	CFU-GM	E11.5AGM	0nM	-2.9664	0.1682	36	-17.64	<.0001	0.05	-3.3075	-2.6252
BFU-E	E11.5AGM	45nM	CFU-GM	E11.5AGM	15nM	-2.3446	0.1693	36	-13.85	<.0001	0.05	-2.6879	-2.0013
BFU-E	E11.5AGM	45nM	CFU-GM	E11.5AGM	45nM	-1.4887	0.09865	36	-15.09	<.0001	0.05	-1.6888	-1.2886
BFU-E	E11.5AGM	45nM	CFU-GM	HE+PSCs	0nM	-2.3550	0.1693	36	-13.91	<.0001	0.05	-2.6983	-2.0117
BFU-E	E11.5AGM	45nM	CFU-GM	HE+PSCs	15nM	-1.4222	0.1728	36	-8.23	<.0001	0.05	-1.7726	-1.0717
BFU-E	E11.5AGM	45nM	CFU-GM	HE+PSCs	45nM	-0.5685	0.1803	36	-3.15	0.0033	0.05	-0.9342	-0.2028
BFU-E	HE+PSCs	0nM	BFU-E	HE+PSCs	15nM	1.1364	0.1876	36	6.06	<.0001	0.05	0.7559	1.5170
BFU-E	HE+PSCs	0nM	BFU-E	HE+PSCs	45nM	2.1590	0.2366	36	9.12	<.0001	0.05	1.6791	2.6390
BFU-E	HE+PSCs	0nM	CFU-GEMM	AoE+PSCs	0nM	-0.1055	0.1644	36	-0.64	0.5252	0.05	-0.4390	0.2280

The GLIMMIX Procedure

Differences of Colony*Condit*Treatm Least Squares Means													
Colony_Type	Condition	Treatment	_Colony_Type	_Condition	_Treatment	Estimate	Standard Error	DF	t Value	Pr > |t|	Alpha	Lower	Upper
BFU-E	HE+PSCs	0nM	CFU-GEMM	AoE+PSCs	15nM	0.5082	0.1729	36	2.94	0.0057	0.05	0.1574	0.8589
BFU-E	HE+PSCs	0nM	CFU-GEMM	AoE+PSCs	45nM	1.4394	0.1988	36	7.24	<.0001	0.05	1.0361	1.8427
BFU-E	HE+PSCs	0nM	CFU-GEMM	E11.5AGM	0nM	-0.7518	0.1594	36	-4.71	<.0001	0.05	-1.0751	-0.4284
BFU-E	HE+PSCs	0nM	CFU-GEMM	E11.5AGM	15nM	-0.4872	0.1611	36	-3.02	0.0046	0.05	-0.8139	-0.1606
BFU-E	HE+PSCs	0nM	CFU-GEMM	E11.5AGM	45nM	0.7726	0.1779	36	4.34	0.0001	0.05	0.4117	1.1335
BFU-E	HE+PSCs	0nM	CFU-GEMM	HE+PSCs	0nM	-0.08004	0.08534	36	-0.94	0.3546	0.05	-0.2531	0.09304
BFU-E	HE+PSCs	0nM	CFU-GEMM	HE+PSCs	15nM	1.0697	0.1857	36	5.76	<.0001	0.05	0.6932	1.4462
BFU-E	HE+PSCs	0nM	CFU-GEMM	HE+PSCs	45nM	1.4497	0.1990	36	7.28	<.0001	0.05	1.0461	1.8532
BFU-E	HE+PSCs	0nM	CFU-GM	AoE+PSCs	0nM	-1.7770	0.1558	36	-11.40	<.0001	0.05	-2.0930	-1.4610
BFU-E	HE+PSCs	0nM	CFU-GM	AoE+PSCs	15nM	-1.0167	0.1582	36	-6.43	<.0001	0.05	-1.3374	-0.6959
BFU-E	HE+PSCs	0nM	CFU-GM	AoE+PSCs	45nM	-0.1010	0.1645	36	-0.61	0.5433	0.05	-0.4347	0.2327
BFU-E	HE+PSCs	0nM	CFU-GM	E11.5AGM	0nM	-2.1938	0.1551	36	-14.14	<.0001	0.05	-2.5084	-1.8792
BFU-E	HE+PSCs	0nM	CFU-GM	E11.5AGM	15nM	-1.5720	0.1563	36	-10.06	<.0001	0.05	-1.8890	-1.2551
BFU-E	HE+PSCs	0nM	CFU-GM	E11.5AGM	45nM	-0.7161	0.1597	36	-4.48	<.0001	0.05	-1.0401	-0.3922
BFU-E	HE+PSCs	0nM	CFU-GM	HE+PSCs	0nM	-1.5825	0.06757	36	-23.42	<.0001	0.05	-1.7195	-1.4454
BFU-E	HE+PSCs	0nM	CFU-GM	HE+PSCs	15nM	-0.6496	0.1601	36	-4.06	0.0003	0.05	-0.9744	-0.3248
BFU-E	HE+PSCs	0nM	CFU-GM	HE+PSCs	45nM	0.2041	0.1682	36	1.21	0.2329	0.05	-0.1371	0.5452
BFU-E	HE+PSCs	15nM	BFU-E	HE+PSCs	45nM	1.0226	0.2525	36	4.05	0.0003	0.05	0.5105	1.5347
BFU-E	HE+PSCs	15nM	CFU-GEMM	AoE+PSCs	0nM	-1.2420	0.1866	36	-6.66	<.0001	0.05	-1.6204	-0.8635
BFU-E	HE+PSCs	15nM	CFU-GEMM	AoE+PSCs	15nM	-0.6283	0.1941	36	-3.24	0.0026	0.05	-1.0220	-0.2346
BFU-E	HE+PSCs	15nM	CFU-GEMM	AoE+PSCs	45nM	0.3029	0.2175	36	1.39	0.1723	0.05	-0.1383	0.7441
BFU-E	HE+PSCs	15nM	CFU-GEMM	E11.5AGM	0nM	-1.8882	0.1822	36	-10.36	<.0001	0.05	-2.2578	-1.5186
BFU-E	HE+PSCs	15nM	CFU-GEMM	E11.5AGM	15nM	-1.6237	0.1837	36	-8.84	<.0001	0.05	-1.9962	-1.2512
BFU-E	HE+PSCs	15nM	CFU-GEMM	E11.5AGM	45nM	-0.3638	0.1986	36	-1.83	0.0752	0.05	-0.7666	0.03890

The GLIMMIX Procedure

Differences of Colony*Condit*Treatm Least Squares Means													
Colony_Type	Condition	Treatment	_Colony_Type	_Condition	_Treatment	Estimate	Standard Error	DF	t Value	Pr > |t|	Alpha	Lower	Upper
BFU-E	HE+PSCs	15nM	CFU-GEMM	HE+PSCs	0nM	-1.2165	0.1869	36	-6.51	<.0001	0.05	-1.5955	-0.8375
BFU-E	HE+PSCs	15nM	CFU-GEMM	HE+PSCs	15nM	-0.06673	0.1492	36	-0.45	0.6573	0.05	-0.3692	0.2358
BFU-E	HE+PSCs	15nM	CFU-GEMM	HE+PSCs	45nM	0.3132	0.2176	36	1.44	0.1588	0.05	-0.1282	0.7546
BFU-E	HE+PSCs	15nM	CFU-GM	AoE+PSCs	0nM	-2.9134	0.1791	36	-16.27	<.0001	0.05	-3.2766	-2.5503
BFU-E	HE+PSCs	15nM	CFU-GM	AoE+PSCs	15nM	-2.1531	0.1811	36	-11.89	<.0001	0.05	-2.5204	-1.7859
BFU-E	HE+PSCs	15nM	CFU-GM	AoE+PSCs	45nM	-1.2374	0.1867	36	-6.63	<.0001	0.05	-1.6160	-0.8588
BFU-E	HE+PSCs	15nM	CFU-GM	E11.5AGM	0nM	-3.3303	0.1784	36	-18.66	<.0001	0.05	-3.6921	-2.9684
BFU-E	HE+PSCs	15nM	CFU-GM	E11.5AGM	15nM	-2.7085	0.1795	36	-15.09	<.0001	0.05	-3.0724	-2.3445
BFU-E	HE+PSCs	15nM	CFU-GM	E11.5AGM	45nM	-1.8526	0.1824	36	-10.15	<.0001	0.05	-2.2226	-1.4826
BFU-E	HE+PSCs	15nM	CFU-GM	HE+PSCs	0nM	-2.7189	0.1794	36	-15.15	<.0001	0.05	-3.0828	-2.3550
BFU-E	HE+PSCs	15nM	CFU-GM	HE+PSCs	15nM	-1.7860	0.1158	36	-15.42	<.0001	0.05	-2.0210	-1.5511
BFU-E	HE+PSCs	15nM	CFU-GM	HE+PSCs	45nM	-0.9324	0.1899	36	-4.91	<.0001	0.05	-1.3175	-0.5472
BFU-E	HE+PSCs	45nM	CFU-GEMM	AoE+PSCs	0nM	-2.2646	0.2358	36	-9.60	<.0001	0.05	-2.7428	-1.7863
BFU-E	HE+PSCs	45nM	CFU-GEMM	AoE+PSCs	15nM	-1.6509	0.2418	36	-6.83	<.0001	0.05	-2.1413	-1.1605
BFU-E	HE+PSCs	45nM	CFU-GEMM	AoE+PSCs	45nM	-0.7197	0.2610	36	-2.76	0.0091	0.05	-1.2489	-0.1904
BFU-E	HE+PSCs	45nM	CFU-GEMM	E11.5AGM	0nM	-2.9108	0.2324	36	-12.53	<.0001	0.05	-3.3820	-2.4395
BFU-E	HE+PSCs	45nM	CFU-GEMM	E11.5AGM	15nM	-2.6463	0.2335	36	-11.33	<.0001	0.05	-3.1198	-2.1728
BFU-E	HE+PSCs	45nM	CFU-GEMM	E11.5AGM	45nM	-1.3864	0.2454	36	-5.65	<.0001	0.05	-1.8841	-0.8888
BFU-E	HE+PSCs	45nM	CFU-GEMM	HE+PSCs	0nM	-2.2391	0.2360	36	-9.49	<.0001	0.05	-2.7177	-1.7604
BFU-E	HE+PSCs	45nM	CFU-GEMM	HE+PSCs	15nM	-1.0893	0.2510	36	-4.34	0.0001	0.05	-1.5985	-0.5802
BFU-E	HE+PSCs	45nM	CFU-GEMM	HE+PSCs	45nM	-0.7094	0.2194	36	-3.23	0.0026	0.05	-1.1544	-0.2644
BFU-E	HE+PSCs	45nM	CFU-GM	AoE+PSCs	0nM	-3.9360	0.2299	36	-17.12	<.0001	0.05	-4.4023	-3.4698
BFU-E	HE+PSCs	45nM	CFU-GM	AoE+PSCs	15nM	-3.1757	0.2315	36	-13.72	<.0001	0.05	-3.6452	-2.7063
BFU-E	HE+PSCs	45nM	CFU-GM	AoE+PSCs	45nM	-2.2600	0.2359	36	-9.58	<.0001	0.05	-2.7384	-1.7816

The GLIMMIX Procedure

Differences of Colony*Condit*Treatm Least Squares Means													
Colony_Type	Condition	Treatment	_Colony_Type	_Condition	_Treatment	Estimate	Standard Error	DF	t Value	Pr > |t|	Alpha	Lower	Upper
BFU-E	HE+PSCs	45nM	CFU-GM	E11.5AGM	0nM	-4.3528	0.2294	36	-18.97	<.0001	0.05	-4.8181	-3.8876
BFU-E	HE+PSCs	45nM	CFU-GM	E11.5AGM	15nM	-3.7311	0.2302	36	-16.21	<.0001	0.05	-4.1979	-3.2642
BFU-E	HE+PSCs	45nM	CFU-GM	E11.5AGM	45nM	-2.8752	0.2325	36	-12.37	<.0001	0.05	-3.3468	-2.4036
BFU-E	HE+PSCs	45nM	CFU-GM	HE+PSCs	0nM	-3.7415	0.2302	36	-16.25	<.0001	0.05	-4.2083	-3.2747
BFU-E	HE+PSCs	45nM	CFU-GM	HE+PSCs	15nM	-2.8086	0.2328	36	-12.06	<.0001	0.05	-3.2808	-2.3365
BFU-E	HE+PSCs	45nM	CFU-GM	HE+PSCs	45nM	-1.9550	0.1919	36	-10.19	<.0001	0.05	-2.3442	-1.5657
CFU-GEMM	AoE+PSCs	0nM	CFU-GEMM	AoE+PSCs	15nM	0.6137	0.1718	36	3.57	0.0010	0.05	0.2652	0.9621
CFU-GEMM	AoE+PSCs	0nM	CFU-GEMM	AoE+PSCs	45nM	1.5449	0.1979	36	7.81	<.0001	0.05	1.1436	1.9462
CFU-GEMM	AoE+PSCs	0nM	CFU-GEMM	E11.5AGM	0nM	-0.6462	0.1582	36	-4.08	0.0002	0.05	-0.9672	-0.3253
CFU-GEMM	AoE+PSCs	0nM	CFU-GEMM	E11.5AGM	15nM	-0.3817	0.1599	36	-2.39	0.0223	0.05	-0.7060	-0.05748
CFU-GEMM	AoE+PSCs	0nM	CFU-GEMM	E11.5AGM	45nM	0.8781	0.1769	36	4.97	<.0001	0.05	0.5195	1.2368
CFU-GEMM	AoE+PSCs	0nM	CFU-GEMM	HE+PSCs	0nM	0.02547	0.1636	36	0.16	0.8771	0.05	-0.3062	0.3572
CFU-GEMM	AoE+PSCs	0nM	CFU-GEMM	HE+PSCs	15nM	1.1752	0.1846	36	6.37	<.0001	0.05	0.8008	1.5496
CFU-GEMM	AoE+PSCs	0nM	CFU-GEMM	HE+PSCs	45nM	1.5552	0.1980	36	7.85	<.0001	0.05	1.1536	1.9568
CFU-GEMM	AoE+PSCs	0nM	CFU-GM	AoE+PSCs	0nM	-1.6715	0.06357	36	-26.29	<.0001	0.05	-1.8004	-1.5426
CFU-GEMM	AoE+PSCs	0nM	CFU-GM	AoE+PSCs	15nM	-0.9112	0.1569	36	-5.81	<.0001	0.05	-1.2294	-0.5929
CFU-GEMM	AoE+PSCs	0nM	CFU-GM	AoE+PSCs	45nM	0.004532	0.1634	36	0.03	0.9780	0.05	-0.3268	0.3358
CFU-GEMM	AoE+PSCs	0nM	CFU-GM	E11.5AGM	0nM	-2.0883	0.1539	36	-13.57	<.0001	0.05	-2.4003	-1.7763
CFU-GEMM	AoE+PSCs	0nM	CFU-GM	E11.5AGM	15nM	-1.4665	0.1550	36	-9.46	<.0001	0.05	-1.7810	-1.1521
CFU-GEMM	AoE+PSCs	0nM	CFU-GM	E11.5AGM	45nM	-0.6106	0.1585	36	-3.85	0.0005	0.05	-0.9321	-0.2891
CFU-GEMM	AoE+PSCs	0nM	CFU-GM	HE+PSCs	0nM	-1.4770	0.1550	36	-9.53	<.0001	0.05	-1.7913	-1.1626
CFU-GEMM	AoE+PSCs	0nM	CFU-GM	HE+PSCs	15nM	-0.5441	0.1589	36	-3.42	0.0016	0.05	-0.8664	-0.2218
CFU-GEMM	AoE+PSCs	0nM	CFU-GM	HE+PSCs	45nM	0.3096	0.1670	36	1.85	0.0721	0.05	-0.02920	0.6484
CFU-GEMM	AoE+PSCs	15nM	CFU-GEMM	AoE+PSCs	45nM	0.9312	0.2050	36	4.54	<.0001	0.05	0.5155	1.3469

The GLIMMIX Procedure

Colony_Type	Condition	Treatment	_Colony_Type	_Condition	_Treatment	Estimate	Standard Error	DF	t Value	Pr > |t|	Alpha	Lower	Upper
CFU-GEMM	AoE+PSCs	15nM	CFU-GEMM	E11.5AGM	0nM	-1.2599	0.1670	36	-7.54	<.0001	0.05	-1.5987	-0.9211
CFU-GEMM	AoE+PSCs	15nM	CFU-GEMM	E11.5AGM	15nM	-0.9954	0.1686	36	-5.90	<.0001	0.05	-1.3373	-0.6535
CFU-GEMM	AoE+PSCs	15nM	CFU-GEMM	E11.5AGM	45nM	0.2645	0.1848	36	1.43	0.1610	0.05	-0.1103	0.6392
CFU-GEMM	AoE+PSCs	15nM	CFU-GEMM	HE+PSCs	0nM	-0.5882	0.1721	36	-3.42	0.0016	0.05	-0.9372	-0.2392
CFU-GEMM	AoE+PSCs	15nM	CFU-GEMM	HE+PSCs	15nM	0.5616	0.1922	36	2.92	0.0060	0.05	0.1718	0.9514
CFU-GEMM	AoE+PSCs	15nM	CFU-GEMM	HE+PSCs	45nM	0.9415	0.2051	36	4.59	<.0001	0.05	0.5255	1.3575
CFU-GEMM	AoE+PSCs	15nM	CFU-GM	AoE+PSCs	0nM	-2.2851	0.1636	36	-13.97	<.0001	0.05	-2.6169	-1.9534
CFU-GEMM	AoE+PSCs	15nM	CFU-GM	AoE+PSCs	15nM	-1.5248	0.08724	36	-17.48	<.0001	0.05	-1.7018	-1.3479
CFU-GEMM	AoE+PSCs	15nM	CFU-GM	AoE+PSCs	45nM	-0.6091	0.1719	36	-3.54	0.0011	0.05	-0.9578	-0.2605
CFU-GEMM	AoE+PSCs	15nM	CFU-GM	E11.5AGM	0nM	-2.7020	0.1629	36	-16.59	<.0001	0.05	-3.0323	-2.3716
CFU-GEMM	AoE+PSCs	15nM	CFU-GM	E11.5AGM	15nM	-2.0802	0.1640	36	-12.68	<.0001	0.05	-2.4128	-1.7475
CFU-GEMM	AoE+PSCs	15nM	CFU-GM	E11.5AGM	45nM	-1.2243	0.1673	36	-7.32	<.0001	0.05	-1.5636	-0.8850
CFU-GEMM	AoE+PSCs	15nM	CFU-GM	HE+PSCs	0nM	-2.0906	0.1640	36	-12.75	<.0001	0.05	-2.4232	-1.7580
CFU-GEMM	AoE+PSCs	15nM	CFU-GM	HE+PSCs	15nM	-1.1577	0.1677	36	-6.90	<.0001	0.05	-1.4978	-0.8177
CFU-GEMM	AoE+PSCs	15nM	CFU-GM	HE+PSCs	45nM	-0.3041	0.1754	36	-1.73	0.0915	0.05	-0.6598	0.05165
CFU-GEMM	AoE+PSCs	45nM	CFU-GEMM	E11.5AGM	0nM	-2.1911	0.1937	36	-11.31	<.0001	0.05	-2.5841	-1.7982
CFU-GEMM	AoE+PSCs	45nM	CFU-GEMM	E11.5AGM	15nM	-1.9266	0.1951	36	-9.88	<.0001	0.05	-2.3223	-1.5309
CFU-GEMM	AoE+PSCs	45nM	CFU-GEMM	E11.5AGM	45nM	-0.6668	0.2092	36	-3.19	0.0030	0.05	-1.0911	-0.2424
CFU-GEMM	AoE+PSCs	45nM	CFU-GEMM	HE+PSCs	0nM	-1.5194	0.1981	36	-7.67	<.0001	0.05	-1.9212	-1.1176
CFU-GEMM	AoE+PSCs	45nM	CFU-GEMM	HE+PSCs	15nM	-0.3697	0.2158	36	-1.71	0.0954	0.05	-0.8074	0.06805
CFU-GEMM	AoE+PSCs	45nM	CFU-GEMM	HE+PSCs	45nM	0.01029	0.2274	36	0.05	0.9642	0.05	-0.4509	0.4715
CFU-GEMM	AoE+PSCs	45nM	CFU-GM	AoE+PSCs	0nM	-3.2164	0.1908	36	-16.86	<.0001	0.05	-3.6033	-2.8295
CFU-GEMM	AoE+PSCs	45nM	CFU-GM	AoE+PSCs	15nM	-2.4561	0.1927	36	-12.75	<.0001	0.05	-2.8468	-2.0653
CFU-GEMM	AoE+PSCs	45nM	CFU-GM	AoE+PSCs	45nM	-1.5404	0.1388	36	-11.10	<.0001	0.05	-1.8219	-1.2588

Table title: Differences of Colony*Condit*Treatm Least Squares Means

The GLIMMIX Procedure

Differences of Colony*Condit*Treatm Least Squares Means													
Colony_Type	Condition	Treatment	_Colony_Type	_Condition	_Treatment	Estimate	Standard Error	DF	t Value	Pr > |t|	Alpha	Lower	Upper
CFU-GEMM	AoE+PSCs	45nM	CFU-GM	E11.5AGM	0nM	-3.6332	0.1902	36	-19.10	<.0001	0.05	-4.0189	-3.2475
CFU-GEMM	AoE+PSCs	45nM	CFU-GM	E11.5AGM	15nM	-3.0114	0.1912	36	-15.75	<.0001	0.05	-3.3991	-2.6237
CFU-GEMM	AoE+PSCs	45nM	CFU-GM	E11.5AGM	45nM	-2.1555	0.1940	36	-11.11	<.0001	0.05	-2.5489	-1.7621
CFU-GEMM	AoE+PSCs	45nM	CFU-GM	HE+PSCs	0nM	-3.0218	0.1911	36	-15.81	<.0001	0.05	-3.4095	-2.6342
CFU-GEMM	AoE+PSCs	45nM	CFU-GM	HE+PSCs	15nM	-2.0889	0.1943	36	-10.75	<.0001	0.05	-2.4830	-1.6949
CFU-GEMM	AoE+PSCs	45nM	CFU-GM	HE+PSCs	45nM	-1.2353	0.2010	36	-6.15	<.0001	0.05	-1.6429	-0.8277
CFU-GEMM	E11.5AGM	0nM	CFU-GEMM	E11.5AGM	15nM	0.2645	0.1547	36	1.71	0.0960	0.05	-0.04932	0.5783
CFU-GEMM	E11.5AGM	0nM	CFU-GEMM	E11.5AGM	45nM	1.5244	0.1722	36	8.85	<.0001	0.05	1.1751	1.8736
CFU-GEMM	E11.5AGM	0nM	CFU-GEMM	HE+PSCs	0nM	0.6717	0.1585	36	4.24	0.0002	0.05	0.3502	0.9932
CFU-GEMM	E11.5AGM	0nM	CFU-GEMM	HE+PSCs	15nM	1.8215	0.1802	36	10.11	<.0001	0.05	1.4560	2.1869
CFU-GEMM	E11.5AGM	0nM	CFU-GEMM	HE+PSCs	45nM	2.2014	0.1939	36	11.35	<.0001	0.05	1.8082	2.5947
CFU-GEMM	E11.5AGM	0nM	CFU-GM	AoE+PSCs	0nM	-1.0252	0.1493	36	-6.87	<.0001	0.05	-1.3279	-0.7225
CFU-GEMM	E11.5AGM	0nM	CFU-GM	AoE+PSCs	15nM	-0.2649	0.1517	36	-1.75	0.0892	0.05	-0.5726	0.04271
CFU-GEMM	E11.5AGM	0nM	CFU-GM	AoE+PSCs	45nM	0.6508	0.1583	36	4.11	0.0002	0.05	0.3296	0.9719
CFU-GEMM	E11.5AGM	0nM	CFU-GM	E11.5AGM	0nM	-1.4421	0.04703	36	-30.66	<.0001	0.05	-1.5374	-1.3467
CFU-GEMM	E11.5AGM	0nM	CFU-GM	E11.5AGM	15nM	-0.8203	0.1497	36	-5.48	<.0001	0.05	-1.1240	-0.5166
CFU-GEMM	E11.5AGM	0nM	CFU-GM	E11.5AGM	45nM	0.03562	0.1533	36	0.23	0.8176	0.05	-0.2754	0.3466
CFU-GEMM	E11.5AGM	0nM	CFU-GM	HE+PSCs	0nM	-0.8307	0.1497	36	-5.55	<.0001	0.05	-1.1343	-0.5271
CFU-GEMM	E11.5AGM	0nM	CFU-GM	HE+PSCs	15nM	0.1022	0.1538	36	0.66	0.5106	0.05	-0.2097	0.4140
CFU-GEMM	E11.5AGM	0nM	CFU-GM	HE+PSCs	45nM	0.9558	0.1621	36	5.90	<.0001	0.05	0.6270	1.2847
CFU-GEMM	E11.5AGM	15nM	CFU-GEMM	E11.5AGM	45nM	1.2599	0.1737	36	7.25	<.0001	0.05	0.9075	1.6122
CFU-GEMM	E11.5AGM	15nM	CFU-GEMM	HE+PSCs	0nM	0.4072	0.1602	36	2.54	0.0155	0.05	0.08235	0.7320
CFU-GEMM	E11.5AGM	15nM	CFU-GEMM	HE+PSCs	15nM	1.5570	0.1816	36	8.57	<.0001	0.05	1.1886	1.9253
CFU-GEMM	E11.5AGM	15nM	CFU-GEMM	HE+PSCs	45nM	1.9369	0.1952	36	9.92	<.0001	0.05	1.5409	2.3329

The GLIMMIX Procedure

Differences of Colony*Condit*Treatm Least Squares Means													
Colony_Type	Condition	Treatment	_Colony_Type	_Condition	_Treatment	Estimate	Standard Error	DF	t Value	Pr > |t|	Alpha	Lower	Upper
CFU-GEMM	E11.5AGM	15nM	CFU-GM	AoE+PSCs	0nM	-1.2898	0.1510	36	-8.54	<.0001	0.05	-1.5960	-0.9835
CFU-GEMM	E11.5AGM	15nM	CFU-GM	AoE+PSCs	15nM	-0.5295	0.1534	36	-3.45	0.0014	0.05	-0.8406	-0.2183
CFU-GEMM	E11.5AGM	15nM	CFU-GM	AoE+PSCs	45nM	0.3863	0.1600	36	2.41	0.0210	0.05	0.06181	0.7107
CFU-GEMM	E11.5AGM	15nM	CFU-GM	E11.5AGM	0nM	-1.7066	0.1503	36	-11.36	<.0001	0.05	-2.0113	-1.4018
CFU-GEMM	E11.5AGM	15nM	CFU-GM	E11.5AGM	15nM	-1.0848	0.05546	36	-19.56	<.0001	0.05	-1.1973	-0.9723
CFU-GEMM	E11.5AGM	15nM	CFU-GM	E11.5AGM	45nM	-0.2289	0.1550	36	-1.48	0.1485	0.05	-0.5433	0.08551
CFU-GEMM	E11.5AGM	15nM	CFU-GM	HE+PSCs	0nM	-1.0952	0.1515	36	-7.23	<.0001	0.05	-1.4024	-0.7881
CFU-GEMM	E11.5AGM	15nM	CFU-GM	HE+PSCs	15nM	-0.1623	0.1554	36	-1.04	0.3033	0.05	-0.4776	0.1529
CFU-GEMM	E11.5AGM	15nM	CFU-GM	HE+PSCs	45nM	0.6913	0.1637	36	4.22	0.0002	0.05	0.3592	1.0234
CFU-GEMM	E11.5AGM	45nM	CFU-GEMM	HE+PSCs	0nM	-0.8527	0.1771	36	-4.81	<.0001	0.05	-1.2119	-0.4934
CFU-GEMM	E11.5AGM	45nM	CFU-GEMM	HE+PSCs	15nM	0.2971	0.1967	36	1.51	0.1397	0.05	-0.1018	0.6960
CFU-GEMM	E11.5AGM	45nM	CFU-GEMM	HE+PSCs	45nM	0.6770	0.2093	36	3.23	0.0026	0.05	0.2525	1.1016
CFU-GEMM	E11.5AGM	45nM	CFU-GM	AoE+PSCs	0nM	-2.5496	0.1689	36	-15.10	<.0001	0.05	-2.8921	-2.2071
CFU-GEMM	E11.5AGM	45nM	CFU-GM	AoE+PSCs	15nM	-1.7893	0.1710	36	-10.46	<.0001	0.05	-2.1361	-1.4425
CFU-GEMM	E11.5AGM	45nM	CFU-GM	AoE+PSCs	45nM	-0.8736	0.1769	36	-4.94	<.0001	0.05	-1.2324	-0.5148
CFU-GEMM	E11.5AGM	45nM	CFU-GM	E11.5AGM	0nM	-2.9664	0.1682	36	-17.64	<.0001	0.05	-3.3076	-2.6253
CFU-GEMM	E11.5AGM	45nM	CFU-GM	E11.5AGM	15nM	-2.3446	0.1693	36	-13.85	<.0001	0.05	-2.6880	-2.0013
CFU-GEMM	E11.5AGM	45nM	CFU-GM	E11.5AGM	45nM	-1.4887	0.09865	36	-15.09	<.0001	0.05	-1.6888	-1.2887
CFU-GEMM	E11.5AGM	45nM	CFU-GM	HE+PSCs	0nM	-2.3551	0.1693	36	-13.91	<.0001	0.05	-2.6984	-2.0118
CFU-GEMM	E11.5AGM	45nM	CFU-GM	HE+PSCs	15nM	-1.4222	0.1728	36	-8.23	<.0001	0.05	-1.7727	-1.0717
CFU-GEMM	E11.5AGM	45nM	CFU-GM	HE+PSCs	45nM	-0.5685	0.1803	36	-3.15	0.0033	0.05	-0.9342	-0.2029
CFU-GEMM	HE+PSCs	0nM	CFU-GEMM	HE+PSCs	15nM	1.1498	0.1849	36	6.22	<.0001	0.05	0.7748	1.5247
CFU-GEMM	HE+PSCs	0nM	CFU-GEMM	HE+PSCs	45nM	1.5297	0.1983	36	7.72	<.0001	0.05	1.1276	1.9318
CFU-GEMM	HE+PSCs	0nM	CFU-GM	AoE+PSCs	0nM	-1.6970	0.1549	36	-10.96	<.0001	0.05	-2.0111	-1.3829

The GLIMMIX Procedure

Differences of Colony*Condit*Treatm Least Squares Means													
Colony_Type	Condition	Treatment	_Colony_Type	_Condition	_Treatment	Estimate	Standard Error	DF	t Value	Pr > |t|	Alpha	Lower	Upper
CFU-GEMM	HE+PSCs	0nM	CFU-GM	AoE+PSCs	15nM	-0.9366	0.1572	36	-5.96	<.0001	0.05	-1.2555	-0.6178
CFU-GEMM	HE+PSCs	0nM	CFU-GM	AoE+PSCs	45nM	-0.02094	0.1637	36	-0.13	0.8989	0.05	-0.3528	0.3110
CFU-GEMM	HE+PSCs	0nM	CFU-GM	E11.5AGM	0nM	-2.1138	0.1542	36	-13.71	<.0001	0.05	-2.4264	-1.8011
CFU-GEMM	HE+PSCs	0nM	CFU-GM	E11.5AGM	15nM	-1.4920	0.1554	36	-9.60	<.0001	0.05	-1.8071	-1.1769
CFU-GEMM	HE+PSCs	0nM	CFU-GM	E11.5AGM	45nM	-0.6361	0.1588	36	-4.01	0.0003	0.05	-0.9582	-0.3140
CFU-GEMM	HE+PSCs	0nM	CFU-GM	HE+PSCs	0nM	-1.5024	0.06538	36	-22.98	<.0001	0.05	-1.6350	-1.3698
CFU-GEMM	HE+PSCs	0nM	CFU-GM	HE+PSCs	15nM	-0.5695	0.1592	36	-3.58	0.0010	0.05	-0.8925	-0.2466
CFU-GEMM	HE+PSCs	0nM	CFU-GM	HE+PSCs	45nM	0.2841	0.1673	36	1.70	0.0982	0.05	-0.05526	0.6235
CFU-GEMM	HE+PSCs	15nM	CFU-GEMM	HE+PSCs	45nM	0.3799	0.2159	36	1.76	0.0870	0.05	-0.05799	0.8179
CFU-GEMM	HE+PSCs	15nM	CFU-GM	AoE+PSCs	0nM	-2.8467	0.1770	36	-16.09	<.0001	0.05	-3.2056	-2.4878
CFU-GEMM	HE+PSCs	15nM	CFU-GM	AoE+PSCs	15nM	-2.0864	0.1790	36	-11.65	<.0001	0.05	-2.4495	-1.7233
CFU-GEMM	HE+PSCs	15nM	CFU-GM	AoE+PSCs	45nM	-1.1707	0.1847	36	-6.34	<.0001	0.05	-1.5453	-0.7961
CFU-GEMM	HE+PSCs	15nM	CFU-GM	E11.5AGM	0nM	-3.2635	0.1763	36	-18.51	<.0001	0.05	-3.6212	-2.9059
CFU-GEMM	HE+PSCs	15nM	CFU-GM	E11.5AGM	15nM	-2.6417	0.1774	36	-14.89	<.0001	0.05	-3.0015	-2.2820
CFU-GEMM	HE+PSCs	15nM	CFU-GM	E11.5AGM	45nM	-1.7859	0.1804	36	-9.90	<.0001	0.05	-2.1517	-1.4200
CFU-GEMM	HE+PSCs	15nM	CFU-GM	HE+PSCs	0nM	-2.6522	0.1774	36	-14.95	<.0001	0.05	-3.0119	-2.2925
CFU-GEMM	HE+PSCs	15nM	CFU-GM	HE+PSCs	15nM	-1.7193	0.1126	36	-15.27	<.0001	0.05	-1.9477	-1.4909
CFU-GEMM	HE+PSCs	15nM	CFU-GM	HE+PSCs	45nM	-0.8656	0.1879	36	-4.61	<.0001	0.05	-1.2468	-0.4845
CFU-GEMM	HE+PSCs	45nM	CFU-GM	AoE+PSCs	0nM	-3.2267	0.1909	36	-16.90	<.0001	0.05	-3.6139	-2.8395
CFU-GEMM	HE+PSCs	45nM	CFU-GM	AoE+PSCs	15nM	-2.4663	0.1928	36	-12.79	<.0001	0.05	-2.8574	-2.0753
CFU-GEMM	HE+PSCs	45nM	CFU-GM	AoE+PSCs	45nM	-1.5506	0.1981	36	-7.83	<.0001	0.05	-1.9524	-1.1489
CFU-GEMM	HE+PSCs	45nM	CFU-GM	E11.5AGM	0nM	-3.6435	0.1903	36	-19.14	<.0001	0.05	-4.0295	-3.2574
CFU-GEMM	HE+PSCs	45nM	CFU-GM	E11.5AGM	15nM	-3.0217	0.1913	36	-15.80	<.0001	0.05	-3.4097	-2.6337
CFU-GEMM	HE+PSCs	45nM	CFU-GM	E11.5AGM	45nM	-2.1658	0.1941	36	-11.16	<.0001	0.05	-2.5594	-1.7722

The GLIMMIX Procedure

Differences of Colony*Condit*Treatm Least Squares Means													
Colony_Type	Condition	Treatment	_Colony_Type	_Condition	_Treatment	Estimate	Standard Error	DF	t Value	Pr > |t|	Alpha	Lower	Upper
CFU-GEMM	HE+PSCs	45nM	CFU-GM	HE+PSCs	0nM	-3.0321	0.1913	36	-15.85	<.0001	0.05	-3.4201	-2.6442
CFU-GEMM	HE+PSCs	45nM	CFU-GM	HE+PSCs	15nM	-2.0992	0.1944	36	-10.80	<.0001	0.05	-2.4936	-1.7049
CFU-GEMM	HE+PSCs	45nM	CFU-GM	HE+PSCs	45nM	-1.2456	0.1430	36	-8.71	<.0001	0.05	-1.5356	-0.9556
CFU-GM	AoE+PSCs	0nM	CFU-GM	AoE+PSCs	15nM	0.7603	0.1479	36	5.14	<.0001	0.05	0.4604	1.0602
CFU-GM	AoE+PSCs	0nM	CFU-GM	AoE+PSCs	45nM	1.6760	0.1547	36	10.84	<.0001	0.05	1.3623	1.9897
CFU-GM	AoE+PSCs	0nM	CFU-GM	E11.5AGM	0nM	-0.4168	0.1446	36	-2.88	0.0066	0.05	-0.7101	-0.1235
CFU-GM	AoE+PSCs	0nM	CFU-GM	E11.5AGM	15nM	0.2050	0.1459	36	1.41	0.1685	0.05	-0.09086	0.5008
CFU-GM	AoE+PSCs	0nM	CFU-GM	E11.5AGM	45nM	1.0609	0.1496	36	7.09	<.0001	0.05	0.7576	1.3642
CFU-GM	AoE+PSCs	0nM	CFU-GM	HE+PSCs	0nM	0.1945	0.1458	36	1.33	0.1906	0.05	-0.1012	0.4903
CFU-GM	AoE+PSCs	0nM	CFU-GM	HE+PSCs	15nM	1.1274	0.1500	36	7.52	<.0001	0.05	0.8232	1.4316
CFU-GM	AoE+PSCs	0nM	CFU-GM	HE+PSCs	45nM	1.9811	0.1586	36	12.49	<.0001	0.05	1.6595	2.3026
CFU-GM	AoE+PSCs	15nM	CFU-GM	AoE+PSCs	45nM	0.9157	0.1570	36	5.83	<.0001	0.05	0.5972	1.2342
CFU-GM	AoE+PSCs	15nM	CFU-GM	E11.5AGM	0nM	-1.1771	0.1471	36	-8.00	<.0001	0.05	-1.4755	-0.8787
CFU-GM	AoE+PSCs	15nM	CFU-GM	E11.5AGM	15nM	-0.5553	0.1484	36	-3.74	0.0006	0.05	-0.8562	-0.2544
CFU-GM	AoE+PSCs	15nM	CFU-GM	E11.5AGM	45nM	0.3006	0.1520	36	1.98	0.0557	0.05	-0.00767	0.6088
CFU-GM	AoE+PSCs	15nM	CFU-GM	HE+PSCs	0nM	-0.5658	0.1483	36	-3.81	0.0005	0.05	-0.8666	-0.2649
CFU-GM	AoE+PSCs	15nM	CFU-GM	HE+PSCs	15nM	0.3671	0.1524	36	2.41	0.0212	0.05	0.05802	0.6762
CFU-GM	AoE+PSCs	15nM	CFU-GM	HE+PSCs	45nM	1.2208	0.1609	36	7.59	<.0001	0.05	0.8945	1.5470
CFU-GM	AoE+PSCs	45nM	CFU-GM	E11.5AGM	0nM	-2.0928	0.1540	36	-13.59	<.0001	0.05	-2.4051	-1.7806
CFU-GM	AoE+PSCs	45nM	CFU-GM	E11.5AGM	15nM	-1.4710	0.1551	36	-9.48	<.0001	0.05	-1.7857	-1.1564
CFU-GM	AoE+PSCs	45nM	CFU-GM	E11.5AGM	45nM	-0.6152	0.1586	36	-3.88	0.0004	0.05	-0.9368	-0.2935
CFU-GM	AoE+PSCs	45nM	CFU-GM	HE+PSCs	0nM	-1.4815	0.1551	36	-9.55	<.0001	0.05	-1.7961	-1.1669
CFU-GM	AoE+PSCs	45nM	CFU-GM	HE+PSCs	15nM	-0.5486	0.1590	36	-3.45	0.0014	0.05	-0.8711	-0.2261
CFU-GM	AoE+PSCs	45nM	CFU-GM	HE+PSCs	45nM	0.3051	0.1671	36	1.83	0.0763	0.05	-0.03390	0.6440

The GLIMMIX Procedure

Differences of Colony*Condit*Treatm Least Squares Means													
Colony_Type	Condition	Treatment	_Colony_Type	_Condition	_Treatment	Estimate	Standard Error	DF	t Value	Pr > |t|	Alpha	Lower	Upper
CFU-GM	E11.5AGM	0nM	CFU-GM	E11.5AGM	15nM	0.6218	0.1451	36	4.28	0.0001	0.05	0.3275	0.9161
CFU-GM	E11.5AGM	0nM	CFU-GM	E11.5AGM	45nM	1.4777	0.1488	36	9.93	<.0001	0.05	1.1759	1.7795
CFU-GM	E11.5AGM	0nM	CFU-GM	HE+PSCs	0nM	0.6113	0.1451	36	4.21	0.0002	0.05	0.3171	0.9056
CFU-GM	E11.5AGM	0nM	CFU-GM	HE+PSCs	15nM	1.5442	0.1492	36	10.35	<.0001	0.05	1.2415	1.8469
CFU-GM	E11.5AGM	0nM	CFU-GM	HE+PSCs	45nM	2.3979	0.1579	36	15.19	<.0001	0.05	2.0777	2.7181
CFU-GM	E11.5AGM	15nM	CFU-GM	E11.5AGM	45nM	0.8559	0.1500	36	5.70	<.0001	0.05	0.5516	1.1602
CFU-GM	E11.5AGM	15nM	CFU-GM	HE+PSCs	0nM	-0.01044	0.1463	36	-0.07	0.9435	0.05	-0.3072	0.2864
CFU-GM	E11.5AGM	15nM	CFU-GM	HE+PSCs	15nM	0.9224	0.1505	36	6.13	<.0001	0.05	0.6173	1.2276
CFU-GM	E11.5AGM	15nM	CFU-GM	HE+PSCs	45nM	1.7761	0.1590	36	11.17	<.0001	0.05	1.4536	2.0986
CFU-GM	E11.5AGM	45nM	CFU-GM	HE+PSCs	0nM	-0.8663	0.1500	36	-5.77	<.0001	0.05	-1.1706	-0.5621
CFU-GM	E11.5AGM	45nM	CFU-GM	HE+PSCs	15nM	0.06655	0.1540	36	0.43	0.6682	0.05	-0.2458	0.3789
CFU-GM	E11.5AGM	45nM	CFU-GM	HE+PSCs	45nM	0.9202	0.1624	36	5.67	<.0001	0.05	0.5909	1.2495
CFU-GM	HE+PSCs	0nM	CFU-GM	HE+PSCs	15nM	0.9329	0.1504	36	6.20	<.0001	0.05	0.6278	1.2380
CFU-GM	HE+PSCs	0nM	CFU-GM	HE+PSCs	45nM	1.7865	0.1590	36	11.24	<.0001	0.05	1.4641	2.1090
CFU-GM	HE+PSCs	15nM	CFU-GM	HE+PSCs	45nM	0.8537	0.1628	36	5.24	<.0001	0.05	0.5235	1.1838

The GENMOD Procedure

Model Information	
Data Set	WORK.EXT_FIG10BII_E11LY45_TRANS
Distribution	Normal
Link Function	Identity
Dependent Variable	Percentage

Number of Observations Read	45
Number of Observations Used	45

Class Level Information		
Class	Levels	Values
Condition	3	AoE+PSCs E10.5AGM HE+PSCs
Treatment	3	0nM 15nM 45nM

Parameter Information			
Parameter	Effect	Condition	Treatment
Prm1	Intercept		
Prm2	Condition	AoE+PSCs	
Prm3	Condition	E10.5AGM	
Prm4	Condition	HE+PSCs	
Prm5	Treatment		0nM
Prm6	Treatment		15nM
Prm7	Treatment		45nM
Prm8	Condition*Treatment	AoE+PSCs	0nM
Prm9	Condition*Treatment	AoE+PSCs	15nM
Prm10	Condition*Treatment	AoE+PSCs	45nM
Prm11	Condition*Treatment	E10.5AGM	0nM

The GENMOD Procedure

Parameter Information			
**Parameter**	**Effect**	**Condition**	**Treatment**
**Prm12**	Condition*Treatment	E10.5AGM	15nM
**Prm13**	Condition*Treatment	E10.5AGM	45nM
**Prm14**	Condition*Treatment	HE+PSCs	0nM
**Prm15**	Condition*Treatment	HE+PSCs	15nM
**Prm16**	Condition*Treatment	HE+PSCs	45nM

Criteria For Assessing Goodness Of Fit			
**Criterion**	**DF**	**Value**	**Value/DF**
**Deviance**	36	546.4000	15.1778
**Scaled Deviance**	36	45.0000	1.2500
**Pearson Chi-Square**	36	546.4000	15.1778
**Scaled Pearson X2**	36	45.0000	1.2500
**Log Likelihood**		-120.0277	
**Full Log Likelihood**		-120.0277	
**AIC (smaller is better)**		260.0555	
**AICC (smaller is better)**		266.5261	
**BIC (smaller is better)**		278.1221	

Algorithm converged.

The GENMOD Procedure

Analysis Of Maximum Likelihood Parameter Estimates									
Parameter			DF	Estimate	Standard Error	Wald 95% Confidence Limits		Wald Chi-Square	Pr > ChiSq
Intercept			1	2.4000	1.5583	-0.6543	5.4543	2.37	0.1235
Condition	AoE+PSCs		1	3.6000	2.2038	-0.7194	7.9194	2.67	0.1024
Condition	E10.5AGM		1	13.6000	2.2038	9.2806	17.9194	38.08	<.0001
Condition	HE+PSCs		0	0.0000	0.0000	0.0000	0.0000	.	.
Treatment	0nM		1	18.0000	2.2038	13.6806	22.3194	66.71	<.0001
Treatment	15nM		1	6.8000	2.2038	2.4806	11.1194	9.52	0.0020
Treatment	45nM		0	0.0000	0.0000	0.0000	0.0000	.	.
Condition*Treatment	AoE+PSCs	0nM	1	5.0000	3.1167	-1.1086	11.1086	2.57	0.1087
Condition*Treatment	AoE+PSCs	15nM	1	-1.2000	3.1167	-7.3086	4.9086	0.15	0.7002
Condition*Treatment	AoE+PSCs	45nM	0	0.0000	0.0000	0.0000	0.0000	.	.
Condition*Treatment	E10.5AGM	0nM	1	24.6000	3.1167	18.4914	30.7086	62.30	<.0001
Condition*Treatment	E10.5AGM	15nM	1	6.4000	3.1167	0.2914	12.5086	4.22	0.0400
Condition*Treatment	E10.5AGM	45nM	0	0.0000	0.0000	0.0000	0.0000	.	.
Condition*Treatment	HE+PSCs	0nM	0	0.0000	0.0000	0.0000	0.0000	.	.
Condition*Treatment	HE+PSCs	15nM	0	0.0000	0.0000	0.0000	0.0000	.	.
Condition*Treatment	HE+PSCs	45nM	0	0.0000	0.0000	0.0000	0.0000	.	.
Scale			1	3.4846	0.3673	2.8342	4.2842		

Note: The scale parameter was estimated by maximum likelihood.

The GENMOD Procedure

Condition*Treatment Least Squares Means								
Condition	Treatment	Estimate	Standard Error	z Value	Pr > |z|	Alpha	Lower	Upper
AoE+PSCs	0nM	29.0000	1.5583	18.61	<.0001	0.05	25.9457	32.0543
AoE+PSCs	15nM	11.6000	1.5583	7.44	<.0001	0.05	8.5457	14.6543
AoE+PSCs	45nM	6.0000	1.5583	3.85	0.0001	0.05	2.9457	9.0543
E10.5AGM	0nM	58.6000	1.5583	37.60	<.0001	0.05	55.5457	61.6543
E10.5AGM	15nM	29.2000	1.5583	18.74	<.0001	0.05	26.1457	32.2543
E10.5AGM	45nM	16.0000	1.5583	10.27	<.0001	0.05	12.9457	19.0543
HE+PSCs	0nM	20.4000	1.5583	13.09	<.0001	0.05	17.3457	23.4543
HE+PSCs	15nM	9.2000	1.5583	5.90	<.0001	0.05	6.1457	12.2543
HE+PSCs	45nM	2.4000	1.5583	1.54	0.1235	0.05	-0.6543	5.4543

Differences of Condition*Treatment Least Squares Means										
Condition	Treatment	_Condition	_Treatment	Estimate	Standard Error	z Value	Pr > |z|	Alpha	Lower	Upper
AoE+PSCs	0nM	AoE+PSCs	15nM	17.4000	2.2038	7.90	<.0001	0.05	13.0806	21.7194
AoE+PSCs	0nM	AoE+PSCs	45nM	23.0000	2.2038	10.44	<.0001	0.05	18.6806	27.3194
AoE+PSCs	0nM	E10.5AGM	0nM	-29.6000	2.2038	-13.43	<.0001	0.05	-33.9194	-25.2806
AoE+PSCs	0nM	E10.5AGM	15nM	-0.2000	2.2038	-0.09	0.9277	0.05	-4.5194	4.1194
AoE+PSCs	0nM	E10.5AGM	45nM	13.0000	2.2038	5.90	<.0001	0.05	8.6806	17.3194
AoE+PSCs	0nM	HE+PSCs	0nM	8.6000	2.2038	3.90	<.0001	0.05	4.2806	12.9194
AoE+PSCs	0nM	HE+PSCs	15nM	19.8000	2.2038	8.98	<.0001	0.05	15.4806	24.1194
AoE+PSCs	0nM	HE+PSCs	45nM	26.6000	2.2038	12.07	<.0001	0.05	22.2806	30.9194
AoE+PSCs	15nM	AoE+PSCs	45nM	5.6000	2.2038	2.54	0.0111	0.05	1.2806	9.9194
AoE+PSCs	15nM	E10.5AGM	0nM	-47.0000	2.2038	-21.33	<.0001	0.05	-51.3194	-42.6806
AoE+PSCs	15nM	E10.5AGM	15nM	-17.6000	2.2038	-7.99	<.0001	0.05	-21.9194	-13.2806
AoE+PSCs	15nM	E10.5AGM	45nM	-4.4000	2.2038	-2.00	0.0459	0.05	-8.7194	-0.08056

The GENMOD Procedure

Differences of Condition*Treatment Least Squares Means

Condition	Treatment	_Condition	_Treatment	Estimate	Standard Error	z Value	Pr > |z|	Alpha	Lower	Upper
AoE+PSCs	15nM	HE+PSCs	0nM	-8.8000	2.2038	-3.99	<.0001	0.05	-13.1194	-4.4806
AoE+PSCs	15nM	HE+PSCs	15nM	2.4000	2.2038	1.09	0.2761	0.05	-1.9194	6.7194
AoE+PSCs	15nM	HE+PSCs	45nM	9.2000	2.2038	4.17	<.0001	0.05	4.8806	13.5194
AoE+PSCs	45nM	E10.5AGM	0nM	-52.6000	2.2038	-23.87	<.0001	0.05	-56.9194	-48.2806
AoE+PSCs	45nM	E10.5AGM	15nM	-23.2000	2.2038	-10.53	<.0001	0.05	-27.5194	-18.8806
AoE+PSCs	45nM	E10.5AGM	45nM	-10.0000	2.2038	-4.54	<.0001	0.05	-14.3194	-5.6806
AoE+PSCs	45nM	HE+PSCs	0nM	-14.4000	2.2038	-6.53	<.0001	0.05	-18.7194	-10.0806
AoE+PSCs	45nM	HE+PSCs	15nM	-3.2000	2.2038	-1.45	0.1465	0.05	-7.5194	1.1194
AoE+PSCs	45nM	HE+PSCs	45nM	3.6000	2.2038	1.63	0.1024	0.05	-0.7194	7.9194
E10.5AGM	0nM	E10.5AGM	15nM	29.4000	2.2038	13.34	<.0001	0.05	25.0806	33.7194
E10.5AGM	0nM	E10.5AGM	45nM	42.6000	2.2038	19.33	<.0001	0.05	38.2806	46.9194
E10.5AGM	0nM	HE+PSCs	0nM	38.2000	2.2038	17.33	<.0001	0.05	33.8806	42.5194
E10.5AGM	0nM	HE+PSCs	15nM	49.4000	2.2038	22.42	<.0001	0.05	45.0806	53.7194
E10.5AGM	0nM	HE+PSCs	45nM	56.2000	2.2038	25.50	<.0001	0.05	51.8806	60.5194
E10.5AGM	15nM	E10.5AGM	45nM	13.2000	2.2038	5.99	<.0001	0.05	8.8806	17.5194
E10.5AGM	15nM	HE+PSCs	0nM	8.8000	2.2038	3.99	<.0001	0.05	4.4806	13.1194
E10.5AGM	15nM	HE+PSCs	15nM	20.0000	2.2038	9.08	<.0001	0.05	15.6806	24.3194
E10.5AGM	15nM	HE+PSCs	45nM	26.8000	2.2038	12.16	<.0001	0.05	22.4806	31.1194
E10.5AGM	45nM	HE+PSCs	0nM	-4.4000	2.2038	-2.00	0.0459	0.05	-8.7194	-0.08056
E10.5AGM	45nM	HE+PSCs	15nM	6.8000	2.2038	3.09	0.0020	0.05	2.4806	11.1194
E10.5AGM	45nM	HE+PSCs	45nM	13.6000	2.2038	6.17	<.0001	0.05	9.2806	17.9194
HE+PSCs	0nM	HE+PSCs	15nM	11.2000	2.2038	5.08	<.0001	0.05	6.8806	15.5194
HE+PSCs	0nM	HE+PSCs	45nM	18.0000	2.2038	8.17	<.0001	0.05	13.6806	22.3194
HE+PSCs	15nM	HE+PSCs	45nM	6.8000	2.2038	3.09	0.0020	0.05	2.4806	11.1194

The GENMOD Procedure

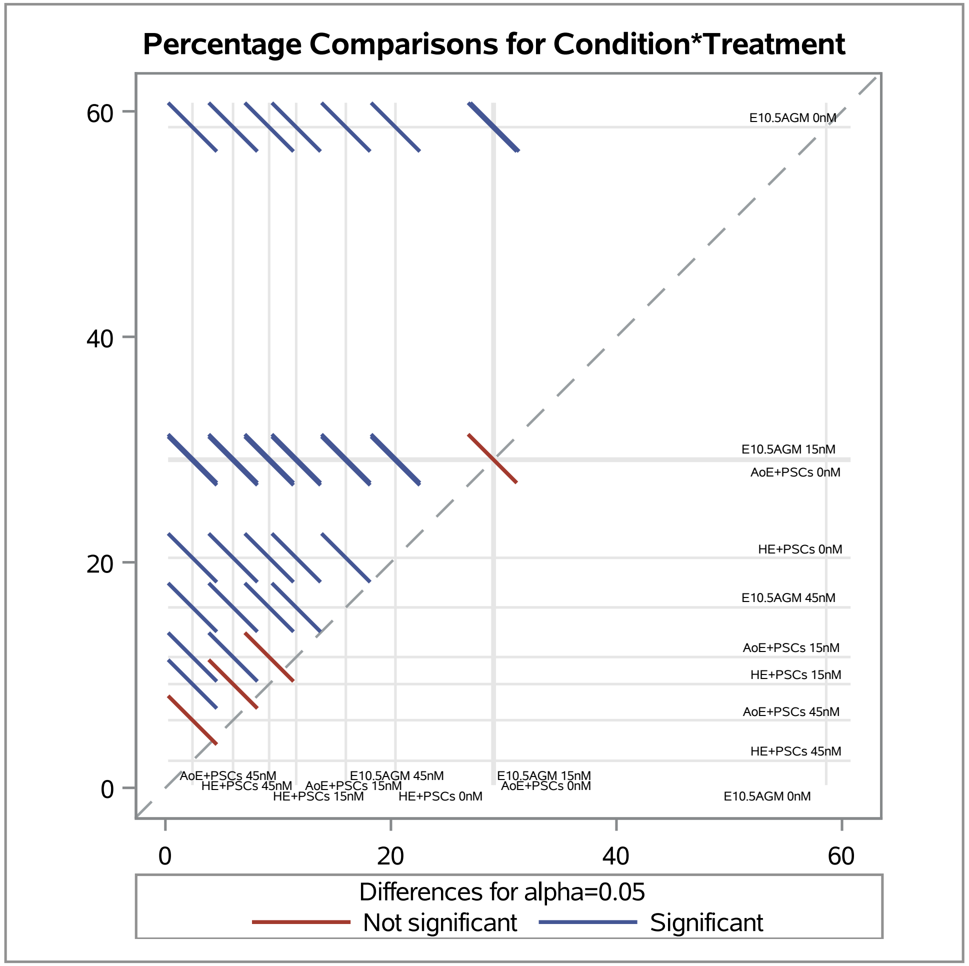

The GLIMMIX Procedure

Model Information	
Data Set	WORK.EXT_FIG10BII_E11MK07
Response Variable	Count
Response Distribution	Poisson
Link Function	Log
Variance Function	Default
Variance Matrix Blocked By	Dish_ID
Estimation Technique	Maximum Likelihood
Likelihood Approximation	Laplace
Degrees of Freedom Method	Containment

Class Level Information		
Class	Levels	Values
Colony_Type	3	BFU-E CFU-GEMM CFU-GM
Condition	3	AoE+PSCs E11.5AGM HE+PSCs
Treatment	3	0nM 15nM 5nM
Dish_ID	27	1 2 3 4 5 6 7 8 9 10 11 12 13 14 15 16 17 18 19 20 21 22 23 24 25 26 27

Number of Observations Read	81
Number of Observations Used	81

Dimensions	
G-side Cov. Parameters	1
Columns in X	64
Columns in Z per Subject	1
Subjects (Blocks in V)	27
Max Obs per Subject	3

The GLIMMIX Procedure

Optimization Information	
Optimization Technique	Dual Quasi-Newton
Parameters in Optimization	28
Lower Boundaries	1
Upper Boundaries	0
Fixed Effects	Not Profiled
Starting From	GLM estimates

Iteration History					
Iteration	Restarts	Evaluations	Objective Function	Change	Max Gradient
0	0	4	849.50878611	.	2810.759
1	0	8	838.00237584	11.50641026	188.1552
2	0	4	835.43919175	2.56318409	131.0276
3	0	3	835.18489063	0.25430112	15.11923
4	0	4	835.10825695	0.07663368	26.87413
5	0	4	835.07691063	0.03134632	6.108781
6	0	3	835.06455971	0.01235092	4.385313
7	0	2	835.04958313	0.01497658	3.505363
8	0	3	835.04224159	0.00734154	0.836001
9	0	3	835.04111863	0.00112296	1.202833
10	0	4	835.03680723	0.00431141	1.509064
11	0	3	835.03631463	0.00049260	2.962905
12	0	4	835.03356494	0.00274969	1.183766
13	0	3	835.03301736	0.00054758	0.599796
14	0	2	835.03246208	0.00055529	1.72715
15	0	2	835.0318859	0.00057618	0.816643

The GLIMMIX Procedure

Iteration History					
Iteration	Restarts	Evaluations	Objective Function	Change	Max Gradient
**16**	**0**	3	835.0317	0.00018590	0.524281
**17**	**0**	4	835.03010835	0.00159165	1.085615
**18**	**0**	3	835.02951332	0.00059502	0.83217
**19**	**0**	4	835.02681103	0.00270229	0.446377
**20**	**0**	2	835.02643868	0.00037235	1.987287
**21**	**0**	2	835.02586293	0.00057576	0.624092
**22**	**0**	4	835.02424504	0.00161788	4.298129
**23**	**0**	2	835.02217017	0.00207487	0.616843
**24**	**0**	2	835.02167735	0.00049283	0.866622
**25**	**0**	2	835.02091229	0.00076505	0.292083
**26**	**0**	3	835.0206485	0.00026380	0.247662
**27**	**0**	3	835.02062197	0.00002653	0.22365
**28**	**0**	4	835.0204947	0.00012727	0.153519
**29**	**0**	2	835.02045692	0.00003778	0.295958
**30**	**0**	4	835.02035903	0.00009789	0.093053
**31**	**0**	3	835.02030406	0.00005498	0.088828
**32**	**0**	4	835.02014394	0.00016011	0.18722

Convergence criterion (GCONV=1E-8) satisfied.

Fit Statistics	
**-2 Log Likelihood**	835.02
**AIC (smaller is better)**	891.02
**AICC (smaller is better)**	922.25
**BIC (smaller is better)**	927.30

The GLIMMIX Procedure

Fit Statistics	
CAIC (smaller is better)	955.30
HQIC (smaller is better)	901.81

Fit Statistics for Conditional Distribution	
-2 log L(Count | r. effects)	746.56
Pearson Chi-Square	230.62
Pearson Chi-Square / DF	2.85

Covariance Parameter Estimates

Cov Parm	Subject	Estimate	Standard Error
Intercept	Dish_ID	0.02780	0.009148

The GLIMMIX Procedure

Solutions for Fixed Effects								
Effect	Colony_Type	Condition	Treatment	Estimate	Standard Error	DF	t Value	Pr > |t|
Intercept				5.0902	0.1065	18	47.82	<.0001
Colony_Type	BFU-E			-1.5292	0.1062	36	-14.40	<.0001
Colony_Type	CFU-GEMM			-1.3673	0.09943	36	-13.75	<.0001
Colony_Type	CFU-GM			0	.	.	.	.
Condition		AoE+PSCs		0.3093	0.1487	36	2.08	0.0447
Condition		E11.5AGM		0.7926	0.1467	36	5.40	<.0001
Condition		HE+PSCs		0	.	.	.	.
Colony_Typ*Condition	BFU-E	AoE+PSCs		0.2349	0.1350	36	1.74	0.0905
Colony_Typ*Condition	BFU-E	E11.5AGM		0.4345	0.1223	36	3.55	0.0011
Colony_Typ*Condition	BFU-E	HE+PSCs		0	.	.	.	.
Colony_Typ*Condition	CFU-GEMM	AoE+PSCs		-0.3000	0.1390	36	-2.16	0.0376
Colony_Typ*Condition	CFU-GEMM	E11.5AGM		0.3207	0.1159	36	2.77	0.0089
Colony_Typ*Condition	CFU-GEMM	HE+PSCs		0	.	.	.	.
Colony_Typ*Condition	CFU-GM	AoE+PSCs		0	.	.	.	.
Colony_Typ*Condition	CFU-GM	E11.5AGM		0	.	.	.	.
Colony_Typ*Condition	CFU-GM	HE+PSCs		0	.	.	.	.
Treatment			0nM	0.9652	0.1462	36	6.60	<.0001
Treatment			15nM	-0.7160	0.1575	36	-4.55	<.0001
Treatment			5nM	0	.	.	.	.
Colony_Typ*Treatment	BFU-E		0nM	-0.05308	0.1258	36	-0.42	0.6757
Colony_Typ*Treatment	BFU-E		15nM	-0.3796	0.2077	36	-1.83	0.0759
Colony_Typ*Treatment	BFU-E		5nM	0	.	.	.	.
Colony_Typ*Treatment	CFU-GEMM		0nM	-0.1350	0.1190	36	-1.13	0.2640
Colony_Typ*Treatment	CFU-GEMM		15nM	-0.1931	0.1833	36	-1.05	0.2991

The GLIMMIX Procedure

Solutions for Fixed Effects								
Effect	Colony_Type	Condition	Treatment	Estimate	Standard Error	DF	t Value	Pr > |t|
Colony_Typ*Treatment	CFU-GEMM		5nM	0	.	.	.	.
Colony_Typ*Treatment	CFU-GM		0nM	0	.	.	.	.
Colony_Typ*Treatment	CFU-GM		15nM	0	.	.	.	.
Colony_Typ*Treatment	CFU-GM		5nM	0	.	.	.	.
Condition*Treatment		AoE+PSCs	0nM	-0.1130	0.2051	36	-0.55	0.5852
Condition*Treatment		AoE+PSCs	15nM	-0.3946	0.2222	36	-1.78	0.0842
Condition*Treatment		AoE+PSCs	5nM	0	.	.	.	.
Condition*Treatment		E11.5AGM	0nM	-0.1795	0.2031	36	-0.88	0.3827
Condition*Treatment		E11.5AGM	15nM	-0.5216	0.2179	36	-2.39	0.0220
Condition*Treatment		E11.5AGM	5nM	0	.	.	.	.
Condition*Treatment		HE+PSCs	0nM	0	.	.	.	.
Condition*Treatment		HE+PSCs	15nM	0	.	.	.	.
Condition*Treatment		HE+PSCs	5nM	0	.	.	.	.
Colony*Condit*Treatm	BFU-E	AoE+PSCs	0nM	-0.4168	0.1648	36	-2.53	0.0160
Colony*Condit*Treatm	BFU-E	AoE+PSCs	15nM	0.7989	0.2556	36	3.13	0.0035
Colony*Condit*Treatm	BFU-E	AoE+PSCs	5nM	0	.	.	.	.
Colony*Condit*Treatm	BFU-E	E11.5AGM	0nM	-0.1746	0.1467	36	-1.19	0.2417
Colony*Condit*Treatm	BFU-E	E11.5AGM	15nM	0.5985	0.2394	36	2.50	0.0171
Colony*Condit*Treatm	BFU-E	E11.5AGM	5nM	0	.	.	.	.
Colony*Condit*Treatm	BFU-E	HE+PSCs	0nM	0	.	.	.	.
Colony*Condit*Treatm	BFU-E	HE+PSCs	15nM	0	.	.	.	.
Colony*Condit*Treatm	BFU-E	HE+PSCs	5nM	0	.	.	.	.
Colony*Condit*Treatm	CFU-GEMM	AoE+PSCs	0nM	0.1307	0.1662	36	0.79	0.4368
Colony*Condit*Treatm	CFU-GEMM	AoE+PSCs	15nM	1.6349	0.2305	36	7.09	<.0001

The GLIMMIX Procedure

Solutions for Fixed Effects								
**Effect**	**Colony_Type**	**Condition**	**Treatment**	**Estimate**	**Standard Error**	**DF**	**t Value**	**Pr > |t|**
**Colony*Condit*Treatm**	CFU-GEMM	AoE+PSCs	5nM	0	.	.	.	.
**Colony*Condit*Treatm**	CFU-GEMM	E11.5AGM	0nM	-0.2603	0.1411	36	-1.84	0.0733
**Colony*Condit*Treatm**	CFU-GEMM	E11.5AGM	15nM	-0.07575	0.2274	36	-0.33	0.7410
**Colony*Condit*Treatm**	CFU-GEMM	E11.5AGM	5nM	0	.	.	.	.
**Colony*Condit*Treatm**	CFU-GEMM	HE+PSCs	0nM	0	.	.	.	.
**Colony*Condit*Treatm**	CFU-GEMM	HE+PSCs	15nM	0	.	.	.	.
**Colony*Condit*Treatm**	CFU-GEMM	HE+PSCs	5nM	0	.	.	.	.
**Colony*Condit*Treatm**	CFU-GM	AoE+PSCs	0nM	0	.	.	.	.
**Colony*Condit*Treatm**	CFU-GM	AoE+PSCs	15nM	0	.	.	.	.
**Colony*Condit*Treatm**	CFU-GM	AoE+PSCs	5nM	0	.	.	.	.
**Colony*Condit*Treatm**	CFU-GM	E11.5AGM	0nM	0	.	.	.	.
**Colony*Condit*Treatm**	CFU-GM	E11.5AGM	15nM	0	.	.	.	.
**Colony*Condit*Treatm**	CFU-GM	E11.5AGM	5nM	0	.	.	.	.
**Colony*Condit*Treatm**	CFU-GM	HE+PSCs	0nM	0	.	.	.	.
**Colony*Condit*Treatm**	CFU-GM	HE+PSCs	15nM	0	.	.	.	.
**Colony*Condit*Treatm**	CFU-GM	HE+PSCs	5nM	0	.	.	.	.

Type III Tests of Fixed Effects				
**Effect**	**Num DF**	**Den DF**	**F Value**	**Pr > F**
**Colony_Type**	2	36	1389.88	<.0001
**Condition**	2	36	45.06	<.0001
**Colony_Typ*Condition**	4	36	14.31	<.0001
**Treatment**	2	36	169.75	<.0001
**Colony_Typ*Treatment**	4	36	14.46	<.0001

The GLIMMIX Procedure

Type III Tests of Fixed Effects				
Effect	Num DF	Den DF	F Value	Pr > F
Condition*Treatment	4	36	4.60	0.0042
Colony*Condit*Treatm	8	36	15.78	<.0001

Condition*Treatment Least Squares Means													
Condition	Treatment	Estimate	Standard Error	DF	t Value	Pr > |t|	Alpha	Lower	Upper	Mean	Standard Error Mean	Lower Mean	Upper Mean
AoE+PSCs	0nM	5.1065	0.1007	36	50.72	<.0001	0.05	4.9023	5.3107	165.09	16.6208	134.60	202.49
AoE+PSCs	15nM	3.9221	0.1079	36	36.34	<.0001	0.05	3.7032	4.1410	50.5065	5.4518	40.5763	62.8667
AoE+PSCs	5nM	4.4123	0.1046	36	42.20	<.0001	0.05	4.2003	4.6244	82.4601	8.6217	66.7040	101.94
E11.5AGM	0nM	5.7470	0.09844	36	58.38	<.0001	0.05	5.5474	5.9467	313.26	30.8374	256.57	382.48
E11.5AGM	15nM	3.9148	0.1088	36	35.97	<.0001	0.05	3.6941	4.1355	50.1401	5.4563	40.2102	62.5222
E11.5AGM	5nM	5.1690	0.09988	36	51.75	<.0001	0.05	4.9665	5.3716	175.75	17.5537	143.52	215.21
HE+PSCs	0nM	5.0272	0.1008	36	49.86	<.0001	0.05	4.8227	5.2317	152.51	15.3783	124.30	187.12
HE+PSCs	15nM	3.2179	0.1228	36	26.20	<.0001	0.05	2.9688	3.4669	24.9753	3.0671	19.4691	32.0388
HE+PSCs	5nM	4.1247	0.1070	36	38.54	<.0001	0.05	3.9077	4.3418	61.8521	6.6192	49.7847	76.8445

Differences of Condition*Treatment Least Squares Means											
Condition	Treatment	_Condition	_Treatment	Estimate	Standard Error	DF	t Value	Pr > |t|	Alpha	Lower	Upper
AoE+PSCs	0nM	AoE+PSCs	15nM	1.1844	0.1476	36	8.02	<.0001	0.05	0.8850	1.4838
AoE+PSCs	0nM	AoE+PSCs	5nM	0.6942	0.1451	36	4.78	<.0001	0.05	0.3998	0.9886
AoE+PSCs	0nM	E11.5AGM	0nM	-0.6405	0.1408	36	-4.55	<.0001	0.05	-0.9261	-0.3550
AoE+PSCs	0nM	E11.5AGM	15nM	1.1917	0.1482	36	8.04	<.0001	0.05	0.8910	1.4923
AoE+PSCs	0nM	E11.5AGM	5nM	-0.06255	0.1418	36	-0.44	0.6618	0.05	-0.3502	0.2251
AoE+PSCs	0nM	HE+PSCs	0nM	0.07927	0.1425	36	0.56	0.5814	0.05	-0.2097	0.3682

The GLIMMIX Procedure

Differences of Condition*Treatment Least Squares Means											
Condition	Treatment	_Condition	_Treatment	Estimate	Standard Error	DF	t Value	Pr > |t|	Alpha	Lower	Upper
AoE+PSCs	0nM	HE+PSCs	15nM	1.8886	0.1588	36	11.89	<.0001	0.05	1.5666	2.2107
AoE+PSCs	0nM	HE+PSCs	5nM	0.9818	0.1469	36	6.68	<.0001	0.05	0.6838	1.2797
AoE+PSCs	15nM	AoE+PSCs	5nM	-0.4902	0.1503	36	-3.26	0.0024	0.05	-0.7950	-0.1854
AoE+PSCs	15nM	E11.5AGM	0nM	-1.8249	0.1461	36	-12.49	<.0001	0.05	-2.1212	-1.5287
AoE+PSCs	15nM	E11.5AGM	15nM	0.007280	0.1532	36	0.05	0.9624	0.05	-0.3035	0.3180
AoE+PSCs	15nM	E11.5AGM	5nM	-1.2469	0.1471	36	-8.48	<.0001	0.05	-1.5452	-0.9487
AoE+PSCs	15nM	HE+PSCs	0nM	-1.1051	0.1477	36	-7.48	<.0001	0.05	-1.4047	-0.8056
AoE+PSCs	15nM	HE+PSCs	15nM	0.7042	0.1635	36	4.31	0.0001	0.05	0.3727	1.0357
AoE+PSCs	15nM	HE+PSCs	5nM	-0.2026	0.1520	36	-1.33	0.1908	0.05	-0.5109	0.1056
AoE+PSCs	5nM	E11.5AGM	0nM	-1.3347	0.1436	36	-9.29	<.0001	0.05	-1.6260	-1.0435
AoE+PSCs	5nM	E11.5AGM	15nM	0.4975	0.1509	36	3.30	0.0022	0.05	0.1915	0.8035
AoE+PSCs	5nM	E11.5AGM	5nM	-0.7567	0.1446	36	-5.23	<.0001	0.05	-1.0500	-0.4635
AoE+PSCs	5nM	HE+PSCs	0nM	-0.6149	0.1453	36	-4.23	0.0002	0.05	-0.9095	-0.3203
AoE+PSCs	5nM	HE+PSCs	15nM	1.1944	0.1613	36	7.41	<.0001	0.05	0.8673	1.5215
AoE+PSCs	5nM	HE+PSCs	5nM	0.2876	0.1496	36	1.92	0.0625	0.05	-0.01585	0.5910
E11.5AGM	0nM	E11.5AGM	15nM	1.8322	0.1467	36	12.49	<.0001	0.05	1.5346	2.1298
E11.5AGM	0nM	E11.5AGM	5nM	0.5780	0.1402	36	4.12	0.0002	0.05	0.2936	0.8624
E11.5AGM	0nM	HE+PSCs	0nM	0.7198	0.1409	36	5.11	<.0001	0.05	0.4340	1.0056
E11.5AGM	0nM	HE+PSCs	15nM	2.5292	0.1574	36	16.07	<.0001	0.05	2.2100	2.8484
E11.5AGM	0nM	HE+PSCs	5nM	1.6223	0.1454	36	11.16	<.0001	0.05	1.3274	1.9172
E11.5AGM	15nM	E11.5AGM	5nM	-1.2542	0.1477	36	-8.49	<.0001	0.05	-1.5538	-0.9547
E11.5AGM	15nM	HE+PSCs	0nM	-1.1124	0.1483	36	-7.50	<.0001	0.05	-1.4133	-0.8115
E11.5AGM	15nM	HE+PSCs	15nM	0.6969	0.1640	36	4.25	0.0001	0.05	0.3643	1.0295
E11.5AGM	15nM	HE+PSCs	5nM	-0.2099	0.1526	36	-1.38	0.1773	0.05	-0.5194	0.09950

The GLIMMIX Procedure

Differences of Condition*Treatment Least Squares Means											
Condition	Treatment	_Condition	_Treatment	Estimate	Standard Error	DF	t Value	Pr > |t|	Alpha	Lower	Upper
E11.5AGM	5nM	HE+PSCs	0nM	0.1418	0.1419	36	1.00	0.3244	0.05	-0.1460	0.4297
E11.5AGM	5nM	HE+PSCs	15nM	1.9512	0.1583	36	12.33	<.0001	0.05	1.6301	2.2722
E11.5AGM	5nM	HE+PSCs	5nM	1.0443	0.1464	36	7.13	<.0001	0.05	0.7474	1.3412
HE+PSCs	0nM	HE+PSCs	15nM	1.8093	0.1589	36	11.39	<.0001	0.05	1.4871	2.1316
HE+PSCs	0nM	HE+PSCs	5nM	0.9025	0.1470	36	6.14	<.0001	0.05	0.6043	1.2007
HE+PSCs	15nM	HE+PSCs	5nM	-0.9069	0.1629	36	-5.57	<.0001	0.05	-1.2371	-0.5766

Colony*Condit*Treatm Least Squares Means														
Colony_Type	Condition	Treatment	Estimate	Standard Error	DF	t Value	Pr > |t|	Alpha	Lower	Upper	Mean	Standard Error Mean	Lower Mean	Upper Mean
BFU-E	AoE+PSCs	0nM	4.4876	0.1140	36	39.35	<.0001	0.05	4.2563	4.7188	88.9058	10.1383	70.5489	112.04
BFU-E	AoE+PSCs	15nM	3.4139	0.1417	36	24.09	<.0001	0.05	3.1264	3.7014	30.3832	4.3065	22.7924	40.5020
BFU-E	AoE+PSCs	5nM	4.1052	0.1214	36	33.81	<.0001	0.05	3.8589	4.3514	60.6537	7.3640	47.4155	77.5879
BFU-E	E11.5AGM	0nM	5.3461	0.1042	36	51.31	<.0001	0.05	5.1348	5.5574	209.78	21.8572	169.82	259.14
BFU-E	E11.5AGM	15nM	3.7694	0.1296	36	29.08	<.0001	0.05	3.5065	4.0323	43.3548	5.6206	33.3311	56.3929
BFU-E	E11.5AGM	5nM	4.7881	0.1097	36	43.65	<.0001	0.05	4.5656	5.0105	120.07	13.1696	96.1234	149.98
BFU-E	HE+PSCs	0nM	4.4731	0.1143	36	39.14	<.0001	0.05	4.2414	4.7049	87.6317	10.0142	69.5038	110.49
BFU-E	HE+PSCs	15nM	2.4655	0.1927	36	12.80	<.0001	0.05	2.0747	2.8562	11.7691	2.2677	7.9622	17.3961
BFU-E	HE+PSCs	5nM	3.5610	0.1363	36	26.12	<.0001	0.05	3.2845	3.8375	35.1993	4.7990	26.6961	46.4109
CFU-GEMM	AoE+PSCs	0nM	4.5802	0.1126	36	40.68	<.0001	0.05	4.3518	4.8085	97.5294	10.9797	77.6208	122.54
CFU-GEMM	AoE+PSCs	15nM	4.0635	0.1223	36	33.23	<.0001	0.05	3.8155	4.3115	58.1755	7.1135	45.3983	74.5487
CFU-GEMM	AoE+PSCs	5nM	3.7322	0.1312	36	28.45	<.0001	0.05	3.4662	3.9983	41.7720	5.4799	32.0138	54.5045
CFU-GEMM	E11.5AGM	0nM	5.2266	0.1052	36	49.70	<.0001	0.05	5.0133	5.4398	186.15	19.5744	150.40	230.40
CFU-GEMM	E11.5AGM	15nM	3.3298	0.1446	36	23.03	<.0001	0.05	3.0366	3.6230	27.9335	4.0380	20.8353	37.4499

The GLIMMIX Procedure

Colony*Condit*Treatm Least Squares Means														
Colony_Type	Condition	Treatment	Estimate	Standard Error	DF	t Value	Pr > |t|	Alpha	Lower	Upper	Mean	Standard Error Mean	Lower Mean	Upper Mean
CFU-GEMM	E11.5AGM	5nM	4.8363	0.1091	36	44.33	<.0001	0.05	4.6150	5.0575	126.00	13.7451	100.99	157.20
CFU-GEMM	HE+PSCs	0nM	4.5531	0.1130	36	40.29	<.0001	0.05	4.3240	4.7823	94.9286	10.7264	75.4870	119.38
CFU-GEMM	HE+PSCs	15nM	2.8139	0.1702	36	16.54	<.0001	0.05	2.4688	3.1590	16.6749	2.8375	11.8081	23.5475
CFU-GEMM	HE+PSCs	5nM	3.7230	0.1312	36	28.38	<.0001	0.05	3.4570	3.9890	41.3870	5.4284	31.7204	53.9995
CFU-GM	AoE+PSCs	0nM	6.2518	0.09956	36	62.80	<.0001	0.05	6.0499	6.4537	518.94	51.6629	424.06	635.04
CFU-GM	AoE+PSCs	15nM	4.2890	0.1175	36	36.49	<.0001	0.05	4.0506	4.5273	72.8899	8.5681	57.4291	92.5130
CFU-GM	AoE+PSCs	5nM	5.3995	0.1038	36	52.02	<.0001	0.05	5.1890	5.6101	221.30	22.9716	179.29	273.16
CFU-GM	E11.5AGM	0nM	6.6685	0.09844	36	67.74	<.0001	0.05	6.4688	6.8681	787.20	77.4957	644.73	961.16
CFU-GM	E11.5AGM	15nM	4.6452	0.1117	36	41.57	<.0001	0.05	4.4186	4.8718	104.09	11.6299	82.9813	130.56
CFU-GM	E11.5AGM	5nM	5.8828	0.1010	36	58.26	<.0001	0.05	5.6780	6.0876	358.82	36.2343	292.37	440.37
CFU-GM	HE+PSCs	0nM	6.0554	0.1002	36	60.40	<.0001	0.05	5.8521	6.2588	426.43	42.7491	347.97	522.57
CFU-GM	HE+PSCs	15nM	4.3743	0.1161	36	37.68	<.0001	0.05	4.1388	4.6097	79.3828	9.2158	62.7297	100.46
CFU-GM	HE+PSCs	5nM	5.0902	0.1065	36	47.82	<.0001	0.05	4.8743	5.3061	162.43	17.2910	130.89	201.57

The GLIMMIX Procedure

Differences of Colony*Condit*Treatm Least Squares Means													
Colony_Type	Condition	Treatment	_Colony_Type	_Condition	_Treatment	Estimate	Standard Error	DF	t Value	Pr > |t|	Alpha	Lower	Upper
BFU-E	AoE+PSCs	0nM	BFU-E	AoE+PSCs	15nM	1.0737	0.1819	36	5.90	<.0001	0.05	0.7047	1.4426
BFU-E	AoE+PSCs	0nM	BFU-E	AoE+PSCs	5nM	0.3824	0.1666	36	2.30	0.0276	0.05	0.04459	0.7202
BFU-E	AoE+PSCs	0nM	BFU-E	E11.5AGM	0nM	-0.8585	0.1545	36	-5.56	<.0001	0.05	-1.1718	-0.5452
BFU-E	AoE+PSCs	0nM	BFU-E	E11.5AGM	15nM	0.7182	0.1727	36	4.16	0.0002	0.05	0.3680	1.0683
BFU-E	AoE+PSCs	0nM	BFU-E	E11.5AGM	5nM	-0.3005	0.1582	36	-1.90	0.0656	0.05	-0.6214	0.02039
BFU-E	AoE+PSCs	0nM	BFU-E	HE+PSCs	0nM	0.01443	0.1614	36	0.09	0.9293	0.05	-0.3130	0.3418
BFU-E	AoE+PSCs	0nM	BFU-E	HE+PSCs	15nM	2.0221	0.2239	36	9.03	<.0001	0.05	1.5680	2.4762
BFU-E	AoE+PSCs	0nM	BFU-E	HE+PSCs	5nM	0.9266	0.1777	36	5.21	<.0001	0.05	0.5661	1.2870
BFU-E	AoE+PSCs	0nM	CFU-GEMM	AoE+PSCs	0nM	-0.09258	0.08446	36	-1.10	0.2803	0.05	-0.2639	0.07872
BFU-E	AoE+PSCs	0nM	CFU-GEMM	AoE+PSCs	15nM	0.4241	0.1672	36	2.54	0.0157	0.05	0.08502	0.7632
BFU-E	AoE+PSCs	0nM	CFU-GEMM	AoE+PSCs	5nM	0.7554	0.1738	36	4.35	0.0001	0.05	0.4028	1.1079
BFU-E	AoE+PSCs	0nM	CFU-GEMM	E11.5AGM	0nM	-0.7390	0.1551	36	-4.76	<.0001	0.05	-1.0536	-0.4244
BFU-E	AoE+PSCs	0nM	CFU-GEMM	E11.5AGM	15nM	1.1578	0.1841	36	6.29	<.0001	0.05	0.7843	1.5312
BFU-E	AoE+PSCs	0nM	CFU-GEMM	E11.5AGM	5nM	-0.3487	0.1578	36	-2.21	0.0336	0.05	-0.6687	-0.02863
BFU-E	AoE+PSCs	0nM	CFU-GEMM	HE+PSCs	0nM	-0.06555	0.1605	36	-0.41	0.6855	0.05	-0.3911	0.2600
BFU-E	AoE+PSCs	0nM	CFU-GEMM	HE+PSCs	15nM	1.6737	0.2048	36	8.17	<.0001	0.05	1.2582	2.0891
BFU-E	AoE+PSCs	0nM	CFU-GEMM	HE+PSCs	5nM	0.7646	0.1738	36	4.40	<.0001	0.05	0.4121	1.1171
BFU-E	AoE+PSCs	0nM	CFU-GM	AoE+PSCs	0nM	-1.7642	0.06611	36	-26.68	<.0001	0.05	-1.8983	-1.6301
BFU-E	AoE+PSCs	0nM	CFU-GM	AoE+PSCs	15nM	0.1986	0.1638	36	1.21	0.2331	0.05	-0.1335	0.5308
BFU-E	AoE+PSCs	0nM	CFU-GM	AoE+PSCs	5nM	-0.9120	0.1542	36	-5.91	<.0001	0.05	-1.2247	-0.5992
BFU-E	AoE+PSCs	0nM	CFU-GM	E11.5AGM	0nM	-2.1809	0.1506	36	-14.48	<.0001	0.05	-2.4864	-1.8754
BFU-E	AoE+PSCs	0nM	CFU-GM	E11.5AGM	15nM	-0.1576	0.1596	36	-0.99	0.3300	0.05	-0.4814	0.1661
BFU-E	AoE+PSCs	0nM	CFU-GM	E11.5AGM	5nM	-1.3952	0.1523	36	-9.16	<.0001	0.05	-1.7041	-1.0863
BFU-E	AoE+PSCs	0nM	CFU-GM	HE+PSCs	0nM	-1.5679	0.1518	36	-10.33	<.0001	0.05	-1.8758	-1.2599

The GLIMMIX Procedure

Differences of Colony*Condit*Treatm Least Squares Means													
Colony_Type	Condition	Treatment	_Colony_Type	_Condition	_Treatment	Estimate	Standard Error	DF	t Value	Pr > |t|	Alpha	Lower	Upper
BFU-E	AoE+PSCs	0nM	CFU-GM	HE+PSCs	15nM	0.1133	0.1627	36	0.70	0.4908	0.05	-0.2167	0.4433
BFU-E	AoE+PSCs	0nM	CFU-GM	HE+PSCs	5nM	-0.6027	0.1560	36	-3.86	0.0004	0.05	-0.9190	-0.2863
BFU-E	AoE+PSCs	15nM	BFU-E	AoE+PSCs	5nM	-0.6913	0.1866	36	-3.70	0.0007	0.05	-1.0698	-0.3128
BFU-E	AoE+PSCs	15nM	BFU-E	E11.5AGM	0nM	-1.9322	0.1759	36	-10.98	<.0001	0.05	-2.2889	-1.5754
BFU-E	AoE+PSCs	15nM	BFU-E	E11.5AGM	15nM	-0.3555	0.1920	36	-1.85	0.0723	0.05	-0.7450	0.03395
BFU-E	AoE+PSCs	15nM	BFU-E	E11.5AGM	5nM	-1.3742	0.1792	36	-7.67	<.0001	0.05	-1.7377	-1.0107
BFU-E	AoE+PSCs	15nM	BFU-E	HE+PSCs	0nM	-1.0593	0.1821	36	-5.82	<.0001	0.05	-1.4285	-0.6900
BFU-E	AoE+PSCs	15nM	BFU-E	HE+PSCs	15nM	0.9484	0.2392	36	3.97	0.0003	0.05	0.4634	1.4335
BFU-E	AoE+PSCs	15nM	BFU-E	HE+PSCs	5nM	-0.1471	0.1966	36	-0.75	0.4592	0.05	-0.5460	0.2517
BFU-E	AoE+PSCs	15nM	CFU-GEMM	AoE+PSCs	0nM	-1.1663	0.1810	36	-6.44	<.0001	0.05	-1.5334	-0.7992
BFU-E	AoE+PSCs	15nM	CFU-GEMM	AoE+PSCs	15nM	-0.6496	0.1280	36	-5.08	<.0001	0.05	-0.9091	-0.3901
BFU-E	AoE+PSCs	15nM	CFU-GEMM	AoE+PSCs	5nM	-0.3183	0.1931	36	-1.65	0.1080	0.05	-0.7100	0.07335
BFU-E	AoE+PSCs	15nM	CFU-GEMM	E11.5AGM	0nM	-1.8127	0.1765	36	-10.27	<.0001	0.05	-2.1706	-1.4547
BFU-E	AoE+PSCs	15nM	CFU-GEMM	E11.5AGM	15nM	0.08406	0.2024	36	0.42	0.6804	0.05	-0.3264	0.4946
BFU-E	AoE+PSCs	15nM	CFU-GEMM	E11.5AGM	5nM	-1.4224	0.1789	36	-7.95	<.0001	0.05	-1.7851	-1.0596
BFU-E	AoE+PSCs	15nM	CFU-GEMM	HE+PSCs	0nM	-1.1392	0.1813	36	-6.28	<.0001	0.05	-1.5069	-0.7716
BFU-E	AoE+PSCs	15nM	CFU-GEMM	HE+PSCs	15nM	0.6000	0.2214	36	2.71	0.0103	0.05	0.1509	1.0491
BFU-E	AoE+PSCs	15nM	CFU-GEMM	HE+PSCs	5nM	-0.3091	0.1931	36	-1.60	0.1182	0.05	-0.7007	0.08253
BFU-E	AoE+PSCs	15nM	CFU-GM	AoE+PSCs	0nM	-2.8379	0.1732	36	-16.38	<.0001	0.05	-3.1892	-2.4866
BFU-E	AoE+PSCs	15nM	CFU-GM	AoE+PSCs	15nM	-0.8751	0.1235	36	-7.09	<.0001	0.05	-1.1254	-0.6247
BFU-E	AoE+PSCs	15nM	CFU-GM	AoE+PSCs	5nM	-1.9857	0.1757	36	-11.30	<.0001	0.05	-2.3419	-1.6294
BFU-E	AoE+PSCs	15nM	CFU-GM	E11.5AGM	0nM	-3.2546	0.1726	36	-18.86	<.0001	0.05	-3.6046	-2.9046
BFU-E	AoE+PSCs	15nM	CFU-GM	E11.5AGM	15nM	-1.2313	0.1804	36	-6.82	<.0001	0.05	-1.5973	-0.8654
BFU-E	AoE+PSCs	15nM	CFU-GM	E11.5AGM	5nM	-2.4689	0.1740	36	-14.19	<.0001	0.05	-2.8219	-2.1160

The GLIMMIX Procedure

Differences of Colony*Condit*Treatm Least Squares Means													
Colony_Type	Condition	Treatment	_Colony_Type	_Condition	_Treatment	Estimate	Standard Error	DF	t Value	Pr > |t|	Alpha	Lower	Upper
BFU-E	AoE+PSCs	15nM	CFU-GM	HE+PSCs	0nM	-2.6415	0.1736	36	-15.22	<.0001	0.05	-2.9936	-2.2895
BFU-E	AoE+PSCs	15nM	CFU-GM	HE+PSCs	15nM	-0.9604	0.1832	36	-5.24	<.0001	0.05	-1.3319	-0.5889
BFU-E	AoE+PSCs	15nM	CFU-GM	HE+PSCs	5nM	-1.6764	0.1772	36	-9.46	<.0001	0.05	-2.0358	-1.3169
BFU-E	AoE+PSCs	5nM	BFU-E	E11.5AGM	0nM	-1.2409	0.1600	36	-7.76	<.0001	0.05	-1.5654	-0.9164
BFU-E	AoE+PSCs	5nM	BFU-E	E11.5AGM	15nM	0.3358	0.1776	36	1.89	0.0668	0.05	-0.02444	0.6960
BFU-E	AoE+PSCs	5nM	BFU-E	E11.5AGM	5nM	-0.6829	0.1636	36	-4.17	0.0002	0.05	-1.0147	-0.3511
BFU-E	AoE+PSCs	5nM	BFU-E	HE+PSCs	0nM	-0.3680	0.1667	36	-2.21	0.0338	0.05	-0.7061	-0.02982
BFU-E	AoE+PSCs	5nM	BFU-E	HE+PSCs	15nM	1.6397	0.2277	36	7.20	<.0001	0.05	1.1778	2.1016
BFU-E	AoE+PSCs	5nM	BFU-E	HE+PSCs	5nM	0.5442	0.1826	36	2.98	0.0051	0.05	0.1739	0.9144
BFU-E	AoE+PSCs	5nM	CFU-GEMM	AoE+PSCs	0nM	-0.4750	0.1656	36	-2.87	0.0069	0.05	-0.8108	-0.1392
BFU-E	AoE+PSCs	5nM	CFU-GEMM	AoE+PSCs	15nM	0.04172	0.1723	36	0.24	0.8101	0.05	-0.3077	0.3912
BFU-E	AoE+PSCs	5nM	CFU-GEMM	AoE+PSCs	5nM	0.3730	0.1157	36	3.22	0.0027	0.05	0.1382	0.6077
BFU-E	AoE+PSCs	5nM	CFU-GEMM	E11.5AGM	0nM	-1.1214	0.1606	36	-6.98	<.0001	0.05	-1.4471	-0.7956
BFU-E	AoE+PSCs	5nM	CFU-GEMM	E11.5AGM	15nM	0.7754	0.1888	36	4.11	0.0002	0.05	0.3925	1.1582
BFU-E	AoE+PSCs	5nM	CFU-GEMM	E11.5AGM	5nM	-0.7311	0.1632	36	-4.48	<.0001	0.05	-1.0621	-0.4001
BFU-E	AoE+PSCs	5nM	CFU-GEMM	HE+PSCs	0nM	-0.4479	0.1659	36	-2.70	0.0105	0.05	-0.7843	-0.1116
BFU-E	AoE+PSCs	5nM	CFU-GEMM	HE+PSCs	15nM	1.2913	0.2090	36	6.18	<.0001	0.05	0.8673	1.7152
BFU-E	AoE+PSCs	5nM	CFU-GEMM	HE+PSCs	5nM	0.3822	0.1787	36	2.14	0.0393	0.05	0.01974	0.7447
BFU-E	AoE+PSCs	5nM	CFU-GM	AoE+PSCs	0nM	-2.1466	0.1570	36	-13.67	<.0001	0.05	-2.4650	-1.8282
BFU-E	AoE+PSCs	5nM	CFU-GM	AoE+PSCs	15nM	-0.1838	0.1690	36	-1.09	0.2840	0.05	-0.5265	0.1589
BFU-E	AoE+PSCs	5nM	CFU-GM	AoE+PSCs	5nM	-1.2944	0.08343	36	-15.51	<.0001	0.05	-1.4636	-1.1252
BFU-E	AoE+PSCs	5nM	CFU-GM	E11.5AGM	0nM	-2.5633	0.1563	36	-16.40	<.0001	0.05	-2.8803	-2.2463
BFU-E	AoE+PSCs	5nM	CFU-GM	E11.5AGM	15nM	-0.5400	0.1650	36	-3.27	0.0024	0.05	-0.8747	-0.2054
BFU-E	AoE+PSCs	5nM	CFU-GM	E11.5AGM	5nM	-1.7776	0.1579	36	-11.26	<.0001	0.05	-2.0979	-1.4574

The GLIMMIX Procedure

Differences of Colony*Condit*Treatm Least Squares Means													
Colony_Type	Condition	Treatment	_Colony_Type	_Condition	_Treatment	Estimate	Standard Error	DF	t Value	Pr > |t|	Alpha	Lower	Upper
BFU-E	AoE+PSCs	5nM	CFU-GM	HE+PSCs	0nM	-1.9503	0.1574	36	-12.39	<.0001	0.05	-2.2696	-1.6309
BFU-E	AoE+PSCs	5nM	CFU-GM	HE+PSCs	15nM	-0.2691	0.1680	36	-1.60	0.1179	0.05	-0.6098	0.07157
BFU-E	AoE+PSCs	5nM	CFU-GM	HE+PSCs	5nM	-0.9851	0.1615	36	-6.10	<.0001	0.05	-1.3125	-0.6576
BFU-E	E11.5AGM	0nM	BFU-E	E11.5AGM	15nM	1.5766	0.1663	36	9.48	<.0001	0.05	1.2393	1.9140
BFU-E	E11.5AGM	0nM	BFU-E	E11.5AGM	5nM	0.5580	0.1513	36	3.69	0.0007	0.05	0.2512	0.8648
BFU-E	E11.5AGM	0nM	BFU-E	HE+PSCs	0nM	0.8729	0.1546	36	5.64	<.0001	0.05	0.5593	1.1866
BFU-E	E11.5AGM	0nM	BFU-E	HE+PSCs	15nM	2.8806	0.2190	36	13.15	<.0001	0.05	2.4363	3.3248
BFU-E	E11.5AGM	0nM	BFU-E	HE+PSCs	5nM	1.7850	0.1716	36	10.40	<.0001	0.05	1.4370	2.1330
BFU-E	E11.5AGM	0nM	CFU-GEMM	AoE+PSCs	0nM	0.7659	0.1534	36	4.99	<.0001	0.05	0.4548	1.0770
BFU-E	E11.5AGM	0nM	CFU-GEMM	AoE+PSCs	15nM	1.2826	0.1606	36	7.98	<.0001	0.05	0.9568	1.6084
BFU-E	E11.5AGM	0nM	CFU-GEMM	AoE+PSCs	5nM	1.6138	0.1675	36	9.63	<.0001	0.05	1.2741	1.9536
BFU-E	E11.5AGM	0nM	CFU-GEMM	E11.5AGM	0nM	0.1195	0.05810	36	2.06	0.0470	0.05	0.001657	0.2373
BFU-E	E11.5AGM	0nM	CFU-GEMM	E11.5AGM	15nM	2.0162	0.1782	36	11.31	<.0001	0.05	1.6549	2.3776
BFU-E	E11.5AGM	0nM	CFU-GEMM	E11.5AGM	5nM	0.5098	0.1509	36	3.38	0.0018	0.05	0.2039	0.8157
BFU-E	E11.5AGM	0nM	CFU-GEMM	HE+PSCs	0nM	0.7929	0.1537	36	5.16	<.0001	0.05	0.4812	1.1047
BFU-E	E11.5AGM	0nM	CFU-GEMM	HE+PSCs	15nM	2.5322	0.1995	36	12.69	<.0001	0.05	2.1275	2.9368
BFU-E	E11.5AGM	0nM	CFU-GEMM	HE+PSCs	5nM	1.6231	0.1675	36	9.69	<.0001	0.05	1.2834	1.9628
BFU-E	E11.5AGM	0nM	CFU-GM	AoE+PSCs	0nM	-0.9057	0.1441	36	-6.28	<.0001	0.05	-1.1980	-0.6134
BFU-E	E11.5AGM	0nM	CFU-GM	AoE+PSCs	15nM	1.0571	0.1571	36	6.73	<.0001	0.05	0.7386	1.3757
BFU-E	E11.5AGM	0nM	CFU-GM	AoE+PSCs	5nM	-0.05347	0.1471	36	-0.36	0.7183	0.05	-0.3517	0.2448
BFU-E	E11.5AGM	0nM	CFU-GM	E11.5AGM	0nM	-1.3224	0.04484	36	-29.49	<.0001	0.05	-1.4134	-1.2315
BFU-E	E11.5AGM	0nM	CFU-GM	E11.5AGM	15nM	0.7008	0.1528	36	4.59	<.0001	0.05	0.3910	1.0107
BFU-E	E11.5AGM	0nM	CFU-GM	E11.5AGM	5nM	-0.5367	0.1451	36	-3.70	0.0007	0.05	-0.8310	-0.2425
BFU-E	E11.5AGM	0nM	CFU-GM	HE+PSCs	0nM	-0.7094	0.1446	36	-4.91	<.0001	0.05	-1.0026	-0.4161

The GLIMMIX Procedure

Differences of Colony*Condit*Treatm Least Squares Means													
Colony_Type	Condition	Treatment	_Colony_Type	_Condition	_Treatment	Estimate	Standard Error	DF	t Value	Pr > |t|	Alpha	Lower	Upper
BFU-E	E11.5AGM	0nM	CFU-GM	HE+PSCs	15nM	0.9718	0.1560	36	6.23	<.0001	0.05	0.6554	1.2881
BFU-E	E11.5AGM	0nM	CFU-GM	HE+PSCs	5nM	0.2558	0.1490	36	1.72	0.0945	0.05	-0.04627	0.5579
BFU-E	E11.5AGM	15nM	BFU-E	E11.5AGM	5nM	-1.0187	0.1698	36	-6.00	<.0001	0.05	-1.3631	-0.6743
BFU-E	E11.5AGM	15nM	BFU-E	HE+PSCs	0nM	-0.7037	0.1728	36	-4.07	0.0002	0.05	-1.0542	-0.3532
BFU-E	E11.5AGM	15nM	BFU-E	HE+PSCs	15nM	1.3039	0.2322	36	5.62	<.0001	0.05	0.8331	1.7748
BFU-E	E11.5AGM	15nM	BFU-E	HE+PSCs	5nM	0.2084	0.1881	36	1.11	0.2752	0.05	-0.1731	0.5899
BFU-E	E11.5AGM	15nM	CFU-GEMM	AoE+PSCs	0nM	-0.8107	0.1717	36	-4.72	<.0001	0.05	-1.1590	-0.4625
BFU-E	E11.5AGM	15nM	CFU-GEMM	AoE+PSCs	15nM	-0.2940	0.1782	36	-1.65	0.1075	0.05	-0.6554	0.06728
BFU-E	E11.5AGM	15nM	CFU-GEMM	AoE+PSCs	5nM	0.03719	0.1844	36	0.20	0.8413	0.05	-0.3368	0.4112
BFU-E	E11.5AGM	15nM	CFU-GEMM	E11.5AGM	0nM	-1.4571	0.1669	36	-8.73	<.0001	0.05	-1.7957	-1.1186
BFU-E	E11.5AGM	15nM	CFU-GEMM	E11.5AGM	15nM	0.4396	0.1375	36	3.20	0.0029	0.05	0.1607	0.7185
BFU-E	E11.5AGM	15nM	CFU-GEMM	E11.5AGM	5nM	-1.0668	0.1694	36	-6.30	<.0001	0.05	-1.4105	-0.7232
BFU-E	E11.5AGM	15nM	CFU-GEMM	HE+PSCs	0nM	-0.7837	0.1720	36	-4.56	<.0001	0.05	-1.1325	-0.4349
BFU-E	E11.5AGM	15nM	CFU-GEMM	HE+PSCs	15nM	0.9555	0.2139	36	4.47	<.0001	0.05	0.5218	1.3892
BFU-E	E11.5AGM	15nM	CFU-GEMM	HE+PSCs	5nM	0.04645	0.1844	36	0.25	0.8025	0.05	-0.3275	0.4204
BFU-E	E11.5AGM	15nM	CFU-GM	AoE+PSCs	0nM	-2.4824	0.1635	36	-15.19	<.0001	0.05	-2.8139	-2.1509
BFU-E	E11.5AGM	15nM	CFU-GM	AoE+PSCs	15nM	-0.5195	0.1749	36	-2.97	0.0053	0.05	-0.8743	-0.1647
BFU-E	E11.5AGM	15nM	CFU-GM	AoE+PSCs	5nM	-1.6301	0.1661	36	-9.82	<.0001	0.05	-1.9669	-1.2933
BFU-E	E11.5AGM	15nM	CFU-GM	E11.5AGM	0nM	-2.8991	0.1628	36	-17.81	<.0001	0.05	-3.2292	-2.5689
BFU-E	E11.5AGM	15nM	CFU-GM	E11.5AGM	15nM	-0.8758	0.1024	36	-8.55	<.0001	0.05	-1.0836	-0.6680
BFU-E	E11.5AGM	15nM	CFU-GM	E11.5AGM	5nM	-2.1134	0.1643	36	-12.86	<.0001	0.05	-2.4467	-1.7801
BFU-E	E11.5AGM	15nM	CFU-GM	HE+PSCs	0nM	-2.2860	0.1639	36	-13.95	<.0001	0.05	-2.6184	-1.9537
BFU-E	E11.5AGM	15nM	CFU-GM	HE+PSCs	15nM	-0.6049	0.1739	36	-3.48	0.0013	0.05	-0.9576	-0.2521
BFU-E	E11.5AGM	15nM	CFU-GM	HE+PSCs	5nM	-1.3208	0.1677	36	-7.88	<.0001	0.05	-1.6609	-0.9807

The GLIMMIX Procedure

Differences of Colony*Condit*Treatm Least Squares Means													
Colony_Type	Condition	Treatment	_Colony_Type	_Condition	_Treatment	Estimate	Standard Error	DF	t Value	Pr > |t|	Alpha	Lower	Upper
BFU-E	E11.5AGM	5nM	BFU-E	HE+PSCs	0nM	0.3149	0.1584	36	1.99	0.0544	0.05	-0.00630	0.6362
BFU-E	E11.5AGM	5nM	BFU-E	HE+PSCs	15nM	2.3226	0.2217	36	10.48	<.0001	0.05	1.8730	2.7722
BFU-E	E11.5AGM	5nM	BFU-E	HE+PSCs	5nM	1.2271	0.1750	36	7.01	<.0001	0.05	0.8722	1.5819
BFU-E	E11.5AGM	5nM	CFU-GEMM	AoE+PSCs	0nM	0.2079	0.1572	36	1.32	0.1942	0.05	-0.1108	0.5267
BFU-E	E11.5AGM	5nM	CFU-GEMM	AoE+PSCs	15nM	0.7246	0.1643	36	4.41	<.0001	0.05	0.3915	1.0577
BFU-E	E11.5AGM	5nM	CFU-GEMM	AoE+PSCs	5nM	1.0559	0.1710	36	6.17	<.0001	0.05	0.7091	1.4027
BFU-E	E11.5AGM	5nM	CFU-GEMM	E11.5AGM	0nM	-0.4385	0.1519	36	-2.89	0.0066	0.05	-0.7466	-0.1303
BFU-E	E11.5AGM	5nM	CFU-GEMM	E11.5AGM	15nM	1.4583	0.1815	36	8.04	<.0001	0.05	1.0902	1.8263
BFU-E	E11.5AGM	5nM	CFU-GEMM	E11.5AGM	5nM	-0.04818	0.07334	36	-0.66	0.5154	0.05	-0.1969	0.1006
BFU-E	E11.5AGM	5nM	CFU-GEMM	HE+PSCs	0nM	0.2350	0.1575	36	1.49	0.1444	0.05	-0.08442	0.5543
BFU-E	E11.5AGM	5nM	CFU-GEMM	HE+PSCs	15nM	1.9742	0.2024	36	9.75	<.0001	0.05	1.5636	2.3848
BFU-E	E11.5AGM	5nM	CFU-GEMM	HE+PSCs	5nM	1.0651	0.1710	36	6.23	<.0001	0.05	0.7184	1.4119
BFU-E	E11.5AGM	5nM	CFU-GM	AoE+PSCs	0nM	-1.4637	0.1481	36	-9.88	<.0001	0.05	-1.7641	-1.1633
BFU-E	E11.5AGM	5nM	CFU-GM	AoE+PSCs	15nM	0.4991	0.1608	36	3.10	0.0037	0.05	0.1731	0.8252
BFU-E	E11.5AGM	5nM	CFU-GM	AoE+PSCs	5nM	-0.6115	0.1510	36	-4.05	0.0003	0.05	-0.9177	-0.3052
BFU-E	E11.5AGM	5nM	CFU-GM	E11.5AGM	0nM	-1.8804	0.1474	36	-12.76	<.0001	0.05	-2.1793	-1.5815
BFU-E	E11.5AGM	5nM	CFU-GM	E11.5AGM	15nM	0.1429	0.1566	36	0.91	0.3676	0.05	-0.1747	0.4604
BFU-E	E11.5AGM	5nM	CFU-GM	E11.5AGM	5nM	-1.0947	0.06063	36	-18.06	<.0001	0.05	-1.2177	-0.9718
BFU-E	E11.5AGM	5nM	CFU-GM	HE+PSCs	0nM	-1.2674	0.1486	36	-8.53	<.0001	0.05	-1.5687	-0.9660
BFU-E	E11.5AGM	5nM	CFU-GM	HE+PSCs	15nM	0.4138	0.1597	36	2.59	0.0137	0.05	0.08989	0.7377
BFU-E	E11.5AGM	5nM	CFU-GM	HE+PSCs	5nM	-0.3022	0.1528	36	-1.98	0.0557	0.05	-0.6121	0.007819
BFU-E	HE+PSCs	0nM	BFU-E	HE+PSCs	15nM	2.0077	0.2240	36	8.96	<.0001	0.05	1.5533	2.4620
BFU-E	HE+PSCs	0nM	BFU-E	HE+PSCs	5nM	0.9121	0.1779	36	5.13	<.0001	0.05	0.5513	1.2729
BFU-E	HE+PSCs	0nM	CFU-GEMM	AoE+PSCs	0nM	-0.1070	0.1604	36	-0.67	0.5090	0.05	-0.4323	0.2183

The GLIMMIX Procedure

Differences of Colony*Condit*Treatm Least Squares Means													
Colony_Type	Condition	Treatment	_Colony_Type	_Condition	_Treatment	Estimate	Standard Error	DF	t Value	Pr > abs(t)	Alpha	Lower	Upper
BFU-E	HE+PSCs	0nM	CFU-GEMM	AoE+PSCs	15nM	0.4097	0.1674	36	2.45	0.0194	0.05	0.07026	0.7491
BFU-E	HE+PSCs	0nM	CFU-GEMM	AoE+PSCs	5nM	0.7409	0.1740	36	4.26	0.0001	0.05	0.3881	1.0938
BFU-E	HE+PSCs	0nM	CFU-GEMM	E11.5AGM	0nM	-0.7534	0.1553	36	-4.85	<.0001	0.05	-1.0684	-0.4385
BFU-E	HE+PSCs	0nM	CFU-GEMM	E11.5AGM	15nM	1.1433	0.1843	36	6.20	<.0001	0.05	0.7696	1.5170
BFU-E	HE+PSCs	0nM	CFU-GEMM	E11.5AGM	5nM	-0.3631	0.1580	36	-2.30	0.0275	0.05	-0.6835	-0.04271
BFU-E	HE+PSCs	0nM	CFU-GEMM	HE+PSCs	0nM	-0.07998	0.08534	36	-0.94	0.3549	0.05	-0.2531	0.09310
BFU-E	HE+PSCs	0nM	CFU-GEMM	HE+PSCs	15nM	1.6592	0.2050	36	8.09	<.0001	0.05	1.2435	2.0749
BFU-E	HE+PSCs	0nM	CFU-GEMM	HE+PSCs	5nM	0.7502	0.1740	36	4.31	0.0001	0.05	0.3974	1.1030
BFU-E	HE+PSCs	0nM	CFU-GM	AoE+PSCs	0nM	-1.7786	0.1516	36	-11.74	<.0001	0.05	-2.0860	-1.4713
BFU-E	HE+PSCs	0nM	CFU-GM	AoE+PSCs	15nM	0.1842	0.1639	36	1.12	0.2686	0.05	-0.1483	0.5167
BFU-E	HE+PSCs	0nM	CFU-GM	AoE+PSCs	5nM	-0.9264	0.1544	36	-6.00	<.0001	0.05	-1.2395	-0.6133
BFU-E	HE+PSCs	0nM	CFU-GM	E11.5AGM	0nM	-2.1953	0.1508	36	-14.55	<.0001	0.05	-2.5012	-1.8894
BFU-E	HE+PSCs	0nM	CFU-GM	E11.5AGM	15nM	-0.1721	0.1598	36	-1.08	0.2888	0.05	-0.4962	0.1520
BFU-E	HE+PSCs	0nM	CFU-GM	E11.5AGM	5nM	-1.4097	0.1525	36	-9.24	<.0001	0.05	-1.7189	-1.1004
BFU-E	HE+PSCs	0nM	CFU-GM	HE+PSCs	0nM	-1.5823	0.06757	36	-23.42	<.0001	0.05	-1.7193	-1.4453
BFU-E	HE+PSCs	0nM	CFU-GM	HE+PSCs	15nM	0.09886	0.1629	36	0.61	0.5477	0.05	-0.2315	0.4292
BFU-E	HE+PSCs	0nM	CFU-GM	HE+PSCs	5nM	-0.6171	0.1562	36	-3.95	0.0003	0.05	-0.9338	-0.3004
BFU-E	HE+PSCs	15nM	BFU-E	HE+PSCs	5nM	-1.0956	0.2360	36	-4.64	<.0001	0.05	-1.5742	-0.6169
BFU-E	HE+PSCs	15nM	CFU-GEMM	AoE+PSCs	0nM	-2.1147	0.2232	36	-9.48	<.0001	0.05	-2.5673	-1.6621
BFU-E	HE+PSCs	15nM	CFU-GEMM	AoE+PSCs	15nM	-1.5980	0.2282	36	-7.00	<.0001	0.05	-2.0607	-1.1352
BFU-E	HE+PSCs	15nM	CFU-GEMM	AoE+PSCs	5nM	-1.2667	0.2331	36	-5.43	<.0001	0.05	-1.7395	-0.7940
BFU-E	HE+PSCs	15nM	CFU-GEMM	E11.5AGM	0nM	-2.7611	0.2195	36	-12.58	<.0001	0.05	-3.2063	-2.3159
BFU-E	HE+PSCs	15nM	CFU-GEMM	E11.5AGM	15nM	-0.8643	0.2408	36	-3.59	0.0010	0.05	-1.3528	-0.3759
BFU-E	HE+PSCs	15nM	CFU-GEMM	E11.5AGM	5nM	-2.3708	0.2214	36	-10.71	<.0001	0.05	-2.8198	-1.9217

The GLIMMIX Procedure

Differences of Colony*Condit*Treatm Least Squares Means													
Colony_Type	Condition	Treatment	_Colony_Type	_Condition	_Treatment	Estimate	Standard Error	DF	t Value	Pr > |t|	Alpha	Lower	Upper
BFU-E	HE+PSCs	15nM	CFU-GEMM	HE+PSCs	0nM	-2.0876	0.2234	36	-9.35	<.0001	0.05	-2.5407	-1.6346
BFU-E	HE+PSCs	15nM	CFU-GEMM	HE+PSCs	15nM	-0.3484	0.2176	36	-1.60	0.1181	0.05	-0.7898	0.09293
BFU-E	HE+PSCs	15nM	CFU-GEMM	HE+PSCs	5nM	-1.2575	0.2331	36	-5.40	<.0001	0.05	-1.7302	-0.7848
BFU-E	HE+PSCs	15nM	CFU-GM	AoE+PSCs	0nM	-3.7863	0.2169	36	-17.46	<.0001	0.05	-4.2262	-3.3465
BFU-E	HE+PSCs	15nM	CFU-GM	AoE+PSCs	15nM	-1.8235	0.2257	36	-8.08	<.0001	0.05	-2.2812	-1.3658
BFU-E	HE+PSCs	15nM	CFU-GM	AoE+PSCs	5nM	-2.9341	0.2189	36	-13.41	<.0001	0.05	-3.3779	-2.4902
BFU-E	HE+PSCs	15nM	CFU-GM	E11.5AGM	0nM	-4.2030	0.2164	36	-19.43	<.0001	0.05	-4.6418	-3.7642
BFU-E	HE+PSCs	15nM	CFU-GM	E11.5AGM	15nM	-2.1797	0.2227	36	-9.79	<.0001	0.05	-2.6313	-1.7281
BFU-E	HE+PSCs	15nM	CFU-GM	E11.5AGM	5nM	-3.4173	0.2175	36	-15.71	<.0001	0.05	-3.8585	-2.9761
BFU-E	HE+PSCs	15nM	CFU-GM	HE+PSCs	0nM	-3.5900	0.2172	36	-16.53	<.0001	0.05	-4.0305	-3.1495
BFU-E	HE+PSCs	15nM	CFU-GM	HE+PSCs	15nM	-1.9088	0.1786	36	-10.69	<.0001	0.05	-2.2709	-1.5467
BFU-E	HE+PSCs	15nM	CFU-GM	HE+PSCs	5nM	-2.6248	0.2201	36	-11.93	<.0001	0.05	-3.0712	-2.1784
BFU-E	HE+PSCs	5nM	CFU-GEMM	AoE+PSCs	0nM	-1.0191	0.1768	36	-5.76	<.0001	0.05	-1.3777	-0.6605
BFU-E	HE+PSCs	5nM	CFU-GEMM	AoE+PSCs	15nM	-0.5024	0.1831	36	-2.74	0.0094	0.05	-0.8738	-0.1311
BFU-E	HE+PSCs	5nM	CFU-GEMM	AoE+PSCs	5nM	-0.1712	0.1892	36	-0.90	0.3716	0.05	-0.5549	0.2125
BFU-E	HE+PSCs	5nM	CFU-GEMM	E11.5AGM	0nM	-1.6655	0.1722	36	-9.67	<.0001	0.05	-2.0147	-1.3163
BFU-E	HE+PSCs	5nM	CFU-GEMM	E11.5AGM	15nM	0.2312	0.1987	36	1.16	0.2522	0.05	-0.1717	0.6341
BFU-E	HE+PSCs	5nM	CFU-GEMM	E11.5AGM	5nM	-1.2752	0.1746	36	-7.30	<.0001	0.05	-1.6294	-0.9211
BFU-E	HE+PSCs	5nM	CFU-GEMM	HE+PSCs	0nM	-0.9921	0.1771	36	-5.60	<.0001	0.05	-1.3512	-0.6330
BFU-E	HE+PSCs	5nM	CFU-GEMM	HE+PSCs	15nM	0.7471	0.2180	36	3.43	0.0015	0.05	0.3050	1.1893
BFU-E	HE+PSCs	5nM	CFU-GEMM	HE+PSCs	5nM	-0.1619	0.1309	36	-1.24	0.2241	0.05	-0.4275	0.1036
BFU-E	HE+PSCs	5nM	CFU-GM	AoE+PSCs	0nM	-2.6908	0.1688	36	-15.94	<.0001	0.05	-3.0331	-2.3484
BFU-E	HE+PSCs	5nM	CFU-GM	AoE+PSCs	15nM	-0.7279	0.1800	36	-4.04	0.0003	0.05	-1.0930	-0.3629
BFU-E	HE+PSCs	5nM	CFU-GM	AoE+PSCs	5nM	-1.8385	0.1714	36	-10.73	<.0001	0.05	-2.1860	-1.4910

The GLIMMIX Procedure

Differences of Colony*Condit*Treatm Least Squares Means													
Colony_Type	Condition	Treatment	_Colony_Type	_Condition	_Treatment	Estimate	Standard Error	DF	t Value	Pr > |t|	Alpha	Lower	Upper
BFU-E	HE+PSCs	5nM	CFU-GM	E11.5AGM	0nM	-3.1075	0.1682	36	-18.48	<.0001	0.05	-3.4485	-2.7664
BFU-E	HE+PSCs	5nM	CFU-GM	E11.5AGM	15nM	-1.0842	0.1762	36	-6.15	<.0001	0.05	-1.4416	-0.7268
BFU-E	HE+PSCs	5nM	CFU-GM	E11.5AGM	5nM	-2.3218	0.1697	36	-13.68	<.0001	0.05	-2.6659	-1.9777
BFU-E	HE+PSCs	5nM	CFU-GM	HE+PSCs	0nM	-2.4944	0.1692	36	-14.74	<.0001	0.05	-2.8376	-2.1512
BFU-E	HE+PSCs	5nM	CFU-GM	HE+PSCs	15nM	-0.8133	0.1790	36	-4.54	<.0001	0.05	-1.1764	-0.4502
BFU-E	HE+PSCs	5nM	CFU-GM	HE+PSCs	5nM	-1.5292	0.1062	36	-14.40	<.0001	0.05	-1.7445	-1.3139
CFU-GEMM	AoE+PSCs	0nM	CFU-GEMM	AoE+PSCs	15nM	0.5167	0.1662	36	3.11	0.0037	0.05	0.1796	0.8538
CFU-GEMM	AoE+PSCs	0nM	CFU-GEMM	AoE+PSCs	5nM	0.8479	0.1729	36	4.91	<.0001	0.05	0.4973	1.1985
CFU-GEMM	AoE+PSCs	0nM	CFU-GEMM	E11.5AGM	0nM	-0.6464	0.1540	36	-4.20	0.0002	0.05	-0.9588	-0.3340
CFU-GEMM	AoE+PSCs	0nM	CFU-GEMM	E11.5AGM	15nM	1.2503	0.1832	36	6.82	<.0001	0.05	0.8787	1.6219
CFU-GEMM	AoE+PSCs	0nM	CFU-GEMM	E11.5AGM	5nM	-0.2561	0.1568	36	-1.63	0.1110	0.05	-0.5740	0.06182
CFU-GEMM	AoE+PSCs	0nM	CFU-GEMM	HE+PSCs	0nM	0.02703	0.1595	36	0.17	0.8664	0.05	-0.2965	0.3505
CFU-GEMM	AoE+PSCs	0nM	CFU-GEMM	HE+PSCs	15nM	1.7662	0.2040	36	8.66	<.0001	0.05	1.3524	2.1801
CFU-GEMM	AoE+PSCs	0nM	CFU-GEMM	HE+PSCs	5nM	0.8572	0.1728	36	4.96	<.0001	0.05	0.5066	1.2077
CFU-GEMM	AoE+PSCs	0nM	CFU-GM	AoE+PSCs	0nM	-1.6716	0.06357	36	-26.30	<.0001	0.05	-1.8006	-1.5427
CFU-GEMM	AoE+PSCs	0nM	CFU-GM	AoE+PSCs	15nM	0.2912	0.1628	36	1.79	0.0820	0.05	-0.03889	0.6213
CFU-GEMM	AoE+PSCs	0nM	CFU-GM	AoE+PSCs	5nM	-0.8194	0.1531	36	-5.35	<.0001	0.05	-1.1299	-0.5088
CFU-GEMM	AoE+PSCs	0nM	CFU-GM	E11.5AGM	0nM	-2.0883	0.1495	36	-13.96	<.0001	0.05	-2.3916	-1.7850
CFU-GEMM	AoE+PSCs	0nM	CFU-GM	E11.5AGM	15nM	-0.06507	0.1586	36	-0.41	0.6841	0.05	-0.3867	0.2566
CFU-GEMM	AoE+PSCs	0nM	CFU-GM	E11.5AGM	5nM	-1.3027	0.1512	36	-8.61	<.0001	0.05	-1.6094	-0.9959
CFU-GEMM	AoE+PSCs	0nM	CFU-GM	HE+PSCs	0nM	-1.4753	0.1507	36	-9.79	<.0001	0.05	-1.7810	-1.1696
CFU-GEMM	AoE+PSCs	0nM	CFU-GM	HE+PSCs	15nM	0.2059	0.1617	36	1.27	0.2112	0.05	-0.1221	0.5338
CFU-GEMM	AoE+PSCs	0nM	CFU-GM	HE+PSCs	5nM	-0.5101	0.1549	36	-3.29	0.0022	0.05	-0.8243	-0.1959
CFU-GEMM	AoE+PSCs	15nM	CFU-GEMM	AoE+PSCs	5nM	0.3312	0.1793	36	1.85	0.0730	0.05	-0.03246	0.6949

The GLIMMIX Procedure

Differences of Colony*Condit*Treatm Least Squares Means													
Colony_Type	Condition	Treatment	_Colony_Type	_Condition	_Treatment	Estimate	Standard Error	DF	t Value	Pr > |t|	Alpha	Lower	Upper
CFU-GEMM	AoE+PSCs	15nM	CFU-GEMM	E11.5AGM	0nM	-1.1631	0.1613	36	-7.21	<.0001	0.05	-1.4902	-0.8360
CFU-GEMM	AoE+PSCs	15nM	CFU-GEMM	E11.5AGM	15nM	0.7336	0.1893	36	3.88	0.0004	0.05	0.3497	1.1175
CFU-GEMM	AoE+PSCs	15nM	CFU-GEMM	E11.5AGM	5nM	-0.7728	0.1639	36	-4.72	<.0001	0.05	-1.1051	-0.4405
CFU-GEMM	AoE+PSCs	15nM	CFU-GEMM	HE+PSCs	0nM	-0.4897	0.1665	36	-2.94	0.0057	0.05	-0.8273	-0.1520
CFU-GEMM	AoE+PSCs	15nM	CFU-GEMM	HE+PSCs	15nM	1.2496	0.2095	36	5.96	<.0001	0.05	0.8246	1.6745
CFU-GEMM	AoE+PSCs	15nM	CFU-GEMM	HE+PSCs	5nM	0.3405	0.1793	36	1.90	0.0656	0.05	-0.02313	0.7041
CFU-GEMM	AoE+PSCs	15nM	CFU-GM	AoE+PSCs	0nM	-2.1883	0.1577	36	-13.88	<.0001	0.05	-2.5081	-1.8685
CFU-GEMM	AoE+PSCs	15nM	CFU-GM	AoE+PSCs	15nM	-0.2255	0.1005	36	-2.24	0.0311	0.05	-0.4293	-0.02165
CFU-GEMM	AoE+PSCs	15nM	CFU-GM	AoE+PSCs	5nM	-1.3361	0.1604	36	-8.33	<.0001	0.05	-1.6614	-1.0108
CFU-GEMM	AoE+PSCs	15nM	CFU-GM	E11.5AGM	0nM	-2.6050	0.1570	36	-16.59	<.0001	0.05	-2.9234	-2.2867
CFU-GEMM	AoE+PSCs	15nM	CFU-GM	E11.5AGM	15nM	-0.5818	0.1656	36	-3.51	0.0012	0.05	-0.9176	-0.2459
CFU-GEMM	AoE+PSCs	15nM	CFU-GM	E11.5AGM	5nM	-1.8193	0.1586	36	-11.47	<.0001	0.05	-2.1410	-1.4977
CFU-GEMM	AoE+PSCs	15nM	CFU-GM	HE+PSCs	0nM	-1.9920	0.1581	36	-12.60	<.0001	0.05	-2.3126	-1.6713
CFU-GEMM	AoE+PSCs	15nM	CFU-GM	HE+PSCs	15nM	-0.3108	0.1686	36	-1.84	0.0734	0.05	-0.6527	0.03106
CFU-GEMM	AoE+PSCs	15nM	CFU-GM	HE+PSCs	5nM	-1.0268	0.1621	36	-6.33	<.0001	0.05	-1.3555	-0.6980
CFU-GEMM	AoE+PSCs	5nM	CFU-GEMM	E11.5AGM	0nM	-1.4943	0.1681	36	-8.89	<.0001	0.05	-1.8353	-1.1534
CFU-GEMM	AoE+PSCs	5nM	CFU-GEMM	E11.5AGM	15nM	0.4024	0.1952	36	2.06	0.0465	0.05	0.006512	0.7983
CFU-GEMM	AoE+PSCs	5nM	CFU-GEMM	E11.5AGM	5nM	-1.1040	0.1706	36	-6.47	<.0001	0.05	-1.4501	-0.7580
CFU-GEMM	AoE+PSCs	5nM	CFU-GEMM	HE+PSCs	0nM	-0.8209	0.1731	36	-4.74	<.0001	0.05	-1.1720	-0.4698
CFU-GEMM	AoE+PSCs	5nM	CFU-GEMM	HE+PSCs	15nM	0.9183	0.2149	36	4.27	0.0001	0.05	0.4826	1.3541
CFU-GEMM	AoE+PSCs	5nM	CFU-GEMM	HE+PSCs	5nM	0.009258	0.1855	36	0.05	0.9605	0.05	-0.3670	0.3855
CFU-GEMM	AoE+PSCs	5nM	CFU-GM	AoE+PSCs	0nM	-2.5196	0.1647	36	-15.30	<.0001	0.05	-2.8535	-2.1856
CFU-GEMM	AoE+PSCs	5nM	CFU-GM	AoE+PSCs	15nM	-0.5567	0.1761	36	-3.16	0.0032	0.05	-0.9140	-0.1995
CFU-GEMM	AoE+PSCs	5nM	CFU-GM	AoE+PSCs	5nM	-1.6673	0.09711	36	-17.17	<.0001	0.05	-1.8643	-1.4704

The GLIMMIX Procedure

Differences of Colony*Condit*Treatm Least Squares Means													
Colony_Type	Condition	Treatment	_Colony_Type	_Condition	_Treatment	Estimate	Standard Error	DF	t Value	Pr > |t|	Alpha	Lower	Upper
CFU-GEMM	AoE+PSCs	5nM	CFU-GM	E11.5AGM	0nM	-2.9363	0.1640	36	-17.90	<.0001	0.05	-3.2689	-2.6036
CFU-GEMM	AoE+PSCs	5nM	CFU-GM	E11.5AGM	15nM	-0.9130	0.1723	36	-5.30	<.0001	0.05	-1.2625	-0.5635
CFU-GEMM	AoE+PSCs	5nM	CFU-GM	E11.5AGM	5nM	-2.1506	0.1656	36	-12.99	<.0001	0.05	-2.4863	-1.8148
CFU-GEMM	AoE+PSCs	5nM	CFU-GM	HE+PSCs	0nM	-2.3232	0.1651	36	-14.07	<.0001	0.05	-2.6581	-1.9884
CFU-GEMM	AoE+PSCs	5nM	CFU-GM	HE+PSCs	15nM	-0.6421	0.1752	36	-3.67	0.0008	0.05	-0.9973	-0.2868
CFU-GEMM	AoE+PSCs	5nM	CFU-GM	HE+PSCs	5nM	-1.3580	0.1689	36	-8.04	<.0001	0.05	-1.7006	-1.0154
CFU-GEMM	E11.5AGM	0nM	CFU-GEMM	E11.5AGM	15nM	1.8967	0.1788	36	10.61	<.0001	0.05	1.5342	2.2593
CFU-GEMM	E11.5AGM	0nM	CFU-GEMM	E11.5AGM	5nM	0.3903	0.1515	36	2.58	0.0142	0.05	0.08301	0.6976
CFU-GEMM	E11.5AGM	0nM	CFU-GEMM	HE+PSCs	0nM	0.6734	0.1544	36	4.36	0.0001	0.05	0.3604	0.9865
CFU-GEMM	E11.5AGM	0nM	CFU-GEMM	HE+PSCs	15nM	2.4127	0.2000	36	12.06	<.0001	0.05	2.0070	2.8184
CFU-GEMM	E11.5AGM	0nM	CFU-GEMM	HE+PSCs	5nM	1.5036	0.1681	36	8.94	<.0001	0.05	1.1627	1.8445
CFU-GEMM	E11.5AGM	0nM	CFU-GM	AoE+PSCs	0nM	-1.0252	0.1448	36	-7.08	<.0001	0.05	-1.3189	-0.7315
CFU-GEMM	E11.5AGM	0nM	CFU-GM	AoE+PSCs	15nM	0.9376	0.1577	36	5.94	<.0001	0.05	0.6178	1.2575
CFU-GEMM	E11.5AGM	0nM	CFU-GM	AoE+PSCs	5nM	-0.1730	0.1478	36	-1.17	0.2494	0.05	-0.4726	0.1267
CFU-GEMM	E11.5AGM	0nM	CFU-GM	E11.5AGM	0nM	-1.4419	0.04703	36	-30.66	<.0001	0.05	-1.5373	-1.3465
CFU-GEMM	E11.5AGM	0nM	CFU-GM	E11.5AGM	15nM	0.5813	0.1534	36	3.79	0.0006	0.05	0.2702	0.8925
CFU-GEMM	E11.5AGM	0nM	CFU-GM	E11.5AGM	5nM	-0.6562	0.1458	36	-4.50	<.0001	0.05	-0.9519	-0.3606
CFU-GEMM	E11.5AGM	0nM	CFU-GM	HE+PSCs	0nM	-0.8289	0.1453	36	-5.71	<.0001	0.05	-1.1235	-0.5342
CFU-GEMM	E11.5AGM	0nM	CFU-GM	HE+PSCs	15nM	0.8523	0.1566	36	5.44	<.0001	0.05	0.5346	1.1700
CFU-GEMM	E11.5AGM	0nM	CFU-GM	HE+PSCs	5nM	0.1363	0.1496	36	0.91	0.3683	0.05	-0.1671	0.4398
CFU-GEMM	E11.5AGM	15nM	CFU-GEMM	E11.5AGM	5nM	-1.5064	0.1811	36	-8.32	<.0001	0.05	-1.8737	-1.1392
CFU-GEMM	E11.5AGM	15nM	CFU-GEMM	HE+PSCs	0nM	-1.2233	0.1835	36	-6.67	<.0001	0.05	-1.5954	-0.8512
CFU-GEMM	E11.5AGM	15nM	CFU-GEMM	HE+PSCs	15nM	0.5159	0.2232	36	2.31	0.0267	0.05	0.06321	0.9686
CFU-GEMM	E11.5AGM	15nM	CFU-GEMM	HE+PSCs	5nM	-0.3931	0.1952	36	-2.01	0.0515	0.05	-0.7889	0.002643

The GLIMMIX Procedure

Differences of Colony*Condit*Treatm Least Squares Means													
Colony_Type	Condition	Treatment	_Colony_Type	_Condition	_Treatment	Estimate	Standard Error	DF	t Value	Pr > |t|	Alpha	Lower	Upper
CFU-GEMM	E11.5AGM	15nM	CFU-GM	AoE+PSCs	0nM	-2.9220	0.1755	36	-16.65	<.0001	0.05	-3.2779	-2.5660
CFU-GEMM	E11.5AGM	15nM	CFU-GM	AoE+PSCs	15nM	-0.9591	0.1863	36	-5.15	<.0001	0.05	-1.3369	-0.5813
CFU-GEMM	E11.5AGM	15nM	CFU-GM	AoE+PSCs	5nM	-2.0697	0.1780	36	-11.63	<.0001	0.05	-2.4306	-1.7088
CFU-GEMM	E11.5AGM	15nM	CFU-GM	E11.5AGM	0nM	-3.3387	0.1749	36	-19.09	<.0001	0.05	-3.6934	-2.9840
CFU-GEMM	E11.5AGM	15nM	CFU-GM	E11.5AGM	15nM	-1.3154	0.1208	36	-10.89	<.0001	0.05	-1.5603	-1.0705
CFU-GEMM	E11.5AGM	15nM	CFU-GM	E11.5AGM	5nM	-2.5530	0.1763	36	-14.48	<.0001	0.05	-2.9106	-2.1954
CFU-GEMM	E11.5AGM	15nM	CFU-GM	HE+PSCs	0nM	-2.7256	0.1759	36	-15.49	<.0001	0.05	-3.0824	-2.3688
CFU-GEMM	E11.5AGM	15nM	CFU-GM	HE+PSCs	15nM	-1.0445	0.1853	36	-5.64	<.0001	0.05	-1.4203	-0.6686
CFU-GEMM	E11.5AGM	15nM	CFU-GM	HE+PSCs	5nM	-1.7604	0.1795	36	-9.81	<.0001	0.05	-2.1244	-1.3964
CFU-GEMM	E11.5AGM	5nM	CFU-GEMM	HE+PSCs	0nM	0.2831	0.1571	36	1.80	0.0798	0.05	-0.03540	0.6017
CFU-GEMM	E11.5AGM	5nM	CFU-GEMM	HE+PSCs	15nM	2.0224	0.2021	36	10.01	<.0001	0.05	1.6124	2.4323
CFU-GEMM	E11.5AGM	5nM	CFU-GEMM	HE+PSCs	5nM	1.1133	0.1706	36	6.53	<.0001	0.05	0.7673	1.4593
CFU-GEMM	E11.5AGM	5nM	CFU-GM	AoE+PSCs	0nM	-1.4155	0.1477	36	-9.58	<.0001	0.05	-1.7150	-1.1160
CFU-GEMM	E11.5AGM	5nM	CFU-GM	AoE+PSCs	15nM	0.5473	0.1604	36	3.41	0.0016	0.05	0.2221	0.8726
CFU-GEMM	E11.5AGM	5nM	CFU-GM	AoE+PSCs	5nM	-0.5633	0.1506	36	-3.74	0.0006	0.05	-0.8687	-0.2579
CFU-GEMM	E11.5AGM	5nM	CFU-GM	E11.5AGM	0nM	-1.8322	0.1469	36	-12.47	<.0001	0.05	-2.1302	-1.5342
CFU-GEMM	E11.5AGM	5nM	CFU-GM	E11.5AGM	15nM	0.1910	0.1562	36	1.22	0.2291	0.05	-0.1256	0.5077
CFU-GEMM	E11.5AGM	5nM	CFU-GM	E11.5AGM	5nM	-1.0465	0.05955	36	-17.57	<.0001	0.05	-1.1673	-0.9258
CFU-GEMM	E11.5AGM	5nM	CFU-GM	HE+PSCs	0nM	-1.2192	0.1482	36	-8.23	<.0001	0.05	-1.5197	-0.9187
CFU-GEMM	E11.5AGM	5nM	CFU-GM	HE+PSCs	15nM	0.4620	0.1593	36	2.90	0.0063	0.05	0.1389	0.7851
CFU-GEMM	E11.5AGM	5nM	CFU-GM	HE+PSCs	5nM	-0.2540	0.1524	36	-1.67	0.1043	0.05	-0.5631	0.05514
CFU-GEMM	HE+PSCs	0nM	CFU-GEMM	HE+PSCs	15nM	1.7392	0.2043	36	8.51	<.0001	0.05	1.3250	2.1535
CFU-GEMM	HE+PSCs	0nM	CFU-GEMM	HE+PSCs	5nM	0.8302	0.1731	36	4.80	<.0001	0.05	0.4791	1.1813
CFU-GEMM	HE+PSCs	0nM	CFU-GM	AoE+PSCs	0nM	-1.6987	0.1506	36	-11.28	<.0001	0.05	-2.0041	-1.3932

The GLIMMIX Procedure

Differences of Colony*Condit*Treatm Least Squares Means													
Colony_Type	Condition	Treatment	_Colony_Type	_Condition	_Treatment	Estimate	Standard Error	DF	t Value	Pr > |t|	Alpha	Lower	Upper
CFU-GEMM	HE+PSCs	0nM	CFU-GM	AoE+PSCs	15nM	0.2642	0.1630	36	1.62	0.1139	0.05	-0.06650	0.5948
CFU-GEMM	HE+PSCs	0nM	CFU-GM	AoE+PSCs	5nM	-0.8464	0.1534	36	-5.52	<.0001	0.05	-1.1576	-0.5352
CFU-GEMM	HE+PSCs	0nM	CFU-GM	E11.5AGM	0nM	-2.1154	0.1499	36	-14.12	<.0001	0.05	-2.4193	-1.8114
CFU-GEMM	HE+PSCs	0nM	CFU-GM	E11.5AGM	15nM	-0.09210	0.1589	36	-0.58	0.5658	0.05	-0.4144	0.2302
CFU-GEMM	HE+PSCs	0nM	CFU-GM	E11.5AGM	5nM	-1.3297	0.1515	36	-8.77	<.0001	0.05	-1.6370	-1.0223
CFU-GEMM	HE+PSCs	0nM	CFU-GM	HE+PSCs	0nM	-1.5023	0.06538	36	-22.98	<.0001	0.05	-1.6349	-1.3697
CFU-GEMM	HE+PSCs	0nM	CFU-GM	HE+PSCs	15nM	0.1788	0.1620	36	1.10	0.2769	0.05	-0.1497	0.5074
CFU-GEMM	HE+PSCs	0nM	CFU-GM	HE+PSCs	5nM	-0.5371	0.1552	36	-3.46	0.0014	0.05	-0.8520	-0.2223
CFU-GEMM	HE+PSCs	15nM	CFU-GEMM	HE+PSCs	5nM	-0.9091	0.2148	36	-4.23	0.0002	0.05	-1.3447	-0.4734
CFU-GEMM	HE+PSCs	15nM	CFU-GM	AoE+PSCs	0nM	-3.4379	0.1971	36	-17.44	<.0001	0.05	-3.8377	-3.0380
CFU-GEMM	HE+PSCs	15nM	CFU-GM	AoE+PSCs	15nM	-1.4750	0.2068	36	-7.13	<.0001	0.05	-1.8944	-1.0557
CFU-GEMM	HE+PSCs	15nM	CFU-GM	AoE+PSCs	5nM	-2.5856	0.1993	36	-12.97	<.0001	0.05	-2.9899	-2.1814
CFU-GEMM	HE+PSCs	15nM	CFU-GM	E11.5AGM	0nM	-3.8546	0.1966	36	-19.61	<.0001	0.05	-4.2533	-3.4559
CFU-GEMM	HE+PSCs	15nM	CFU-GM	E11.5AGM	15nM	-1.8313	0.2035	36	-9.00	<.0001	0.05	-2.2440	-1.4186
CFU-GEMM	HE+PSCs	15nM	CFU-GM	E11.5AGM	5nM	-3.0689	0.1979	36	-15.51	<.0001	0.05	-3.4702	-2.6676
CFU-GEMM	HE+PSCs	15nM	CFU-GM	HE+PSCs	0nM	-3.2415	0.1975	36	-16.41	<.0001	0.05	-3.6421	-2.8410
CFU-GEMM	HE+PSCs	15nM	CFU-GM	HE+PSCs	15nM	-1.5604	0.1540	36	-10.13	<.0001	0.05	-1.8727	-1.2481
CFU-GEMM	HE+PSCs	15nM	CFU-GM	HE+PSCs	5nM	-2.2763	0.2007	36	-11.34	<.0001	0.05	-2.6834	-1.8693
CFU-GEMM	HE+PSCs	5nM	CFU-GM	AoE+PSCs	0nM	-2.5288	0.1647	36	-15.36	<.0001	0.05	-2.8628	-2.1949
CFU-GEMM	HE+PSCs	5nM	CFU-GM	AoE+PSCs	15nM	-0.5660	0.1761	36	-3.21	0.0028	0.05	-0.9231	-0.2088
CFU-GEMM	HE+PSCs	5nM	CFU-GM	AoE+PSCs	5nM	-1.6766	0.1673	36	-10.02	<.0001	0.05	-2.0158	-1.3374
CFU-GEMM	HE+PSCs	5nM	CFU-GM	E11.5AGM	0nM	-2.9455	0.1640	36	-17.96	<.0001	0.05	-3.2781	-2.6129
CFU-GEMM	HE+PSCs	5nM	CFU-GM	E11.5AGM	15nM	-0.9223	0.1723	36	-5.35	<.0001	0.05	-1.2716	-0.5729
CFU-GEMM	HE+PSCs	5nM	CFU-GM	E11.5AGM	5nM	-2.1598	0.1655	36	-13.05	<.0001	0.05	-2.4955	-1.8241

The GLIMMIX Procedure

Differences of Colony*Condit*Treatm Least Squares Means													
Colony_Type	Condition	Treatment	_Colony_Type	_Condition	_Treatment	Estimate	Standard Error	DF	t Value	Pr > |t|	Alpha	Lower	Upper
CFU-GEMM	HE+PSCs	5nM	CFU-GM	HE+PSCs	0nM	-2.3325	0.1651	36	-14.13	<.0001	0.05	-2.6673	-1.9977
CFU-GEMM	HE+PSCs	5nM	CFU-GM	HE+PSCs	15nM	-0.6513	0.1751	36	-3.72	0.0007	0.05	-1.0065	-0.2961
CFU-GEMM	HE+PSCs	5nM	CFU-GM	HE+PSCs	5nM	-1.3673	0.09943	36	-13.75	<.0001	0.05	-1.5689	-1.1656
CFU-GM	AoE+PSCs	0nM	CFU-GM	AoE+PSCs	15nM	1.9628	0.1540	36	12.74	<.0001	0.05	1.6504	2.2752
CFU-GM	AoE+PSCs	0nM	CFU-GM	AoE+PSCs	5nM	0.8522	0.1438	36	5.93	<.0001	0.05	0.5605	1.1439
CFU-GM	AoE+PSCs	0nM	CFU-GM	E11.5AGM	0nM	-0.4167	0.1400	36	-2.98	0.0052	0.05	-0.7007	-0.1328
CFU-GM	AoE+PSCs	0nM	CFU-GM	E11.5AGM	15nM	1.6066	0.1496	36	10.74	<.0001	0.05	1.3031	1.9101
CFU-GM	AoE+PSCs	0nM	CFU-GM	E11.5AGM	5nM	0.3690	0.1418	36	2.60	0.0134	0.05	0.08138	0.6566
CFU-GM	AoE+PSCs	0nM	CFU-GM	HE+PSCs	0nM	0.1963	0.1413	36	1.39	0.1732	0.05	-0.09020	0.4829
CFU-GM	AoE+PSCs	0nM	CFU-GM	HE+PSCs	15nM	1.8775	0.1529	36	12.28	<.0001	0.05	1.5673	2.1877
CFU-GM	AoE+PSCs	0nM	CFU-GM	HE+PSCs	5nM	1.1615	0.1457	36	7.97	<.0001	0.05	0.8659	1.4571
CFU-GM	AoE+PSCs	15nM	CFU-GM	AoE+PSCs	5nM	-1.1106	0.1568	36	-7.08	<.0001	0.05	-1.4286	-0.7926
CFU-GM	AoE+PSCs	15nM	CFU-GM	E11.5AGM	0nM	-2.3795	0.1533	36	-15.52	<.0001	0.05	-2.6905	-2.0686
CFU-GM	AoE+PSCs	15nM	CFU-GM	E11.5AGM	15nM	-0.3563	0.1621	36	-2.20	0.0345	0.05	-0.6851	-0.02747
CFU-GM	AoE+PSCs	15nM	CFU-GM	E11.5AGM	5nM	-1.5939	0.1550	36	-10.29	<.0001	0.05	-1.9081	-1.2796
CFU-GM	AoE+PSCs	15nM	CFU-GM	HE+PSCs	0nM	-1.7665	0.1545	36	-11.43	<.0001	0.05	-2.0798	-1.4532
CFU-GM	AoE+PSCs	15nM	CFU-GM	HE+PSCs	15nM	-0.08533	0.1652	36	-0.52	0.6086	0.05	-0.4203	0.2497
CFU-GM	AoE+PSCs	15nM	CFU-GM	HE+PSCs	5nM	-0.8013	0.1586	36	-5.05	<.0001	0.05	-1.1229	-0.4797
CFU-GM	AoE+PSCs	5nM	CFU-GM	E11.5AGM	0nM	-1.2689	0.1431	36	-8.87	<.0001	0.05	-1.5591	-0.9788
CFU-GM	AoE+PSCs	5nM	CFU-GM	E11.5AGM	15nM	0.7543	0.1525	36	4.95	<.0001	0.05	0.4450	1.0636
CFU-GM	AoE+PSCs	5nM	CFU-GM	E11.5AGM	5nM	-0.4833	0.1448	36	-3.34	0.0020	0.05	-0.7770	-0.1896
CFU-GM	AoE+PSCs	5nM	CFU-GM	HE+PSCs	0nM	-0.6559	0.1443	36	-4.55	<.0001	0.05	-0.9486	-0.3632
CFU-GM	AoE+PSCs	5nM	CFU-GM	HE+PSCs	15nM	1.0253	0.1557	36	6.58	<.0001	0.05	0.7094	1.3411
CFU-GM	AoE+PSCs	5nM	CFU-GM	HE+PSCs	5nM	0.3093	0.1487	36	2.08	0.0447	0.05	0.007762	0.6108

The GLIMMIX Procedure

Differences of Colony*Condit*Treatm Least Squares Means													
Colony_Type	Condition	Treatment	_Colony_Type	_Condition	_Treatment	Estimate	Standard Error	DF	t Value	Pr > |t|	Alpha	Lower	Upper
CFU-GM	E11.5AGM	0nM	CFU-GM	E11.5AGM	15nM	2.0233	0.1489	36	13.59	<.0001	0.05	1.7213	2.3253
CFU-GM	E11.5AGM	0nM	CFU-GM	E11.5AGM	5nM	0.7857	0.1410	36	5.57	<.0001	0.05	0.4997	1.0717
CFU-GM	E11.5AGM	0nM	CFU-GM	HE+PSCs	0nM	0.6131	0.1405	36	4.36	0.0001	0.05	0.3281	0.8980
CFU-GM	E11.5AGM	0nM	CFU-GM	HE+PSCs	15nM	2.2942	0.1522	36	15.07	<.0001	0.05	1.9855	2.6029
CFU-GM	E11.5AGM	0nM	CFU-GM	HE+PSCs	5nM	1.5782	0.1450	36	10.88	<.0001	0.05	1.2842	1.8723
CFU-GM	E11.5AGM	15nM	CFU-GM	E11.5AGM	5nM	-1.2376	0.1506	36	-8.22	<.0001	0.05	-1.5430	-0.9322
CFU-GM	E11.5AGM	15nM	CFU-GM	HE+PSCs	0nM	-1.4102	0.1501	36	-9.39	<.0001	0.05	-1.7146	-1.1058
CFU-GM	E11.5AGM	15nM	CFU-GM	HE+PSCs	15nM	0.2709	0.1610	36	1.68	0.1012	0.05	-0.05568	0.5976
CFU-GM	E11.5AGM	15nM	CFU-GM	HE+PSCs	5nM	-0.4450	0.1543	36	-2.88	0.0066	0.05	-0.7579	-0.1321
CFU-GM	E11.5AGM	5nM	CFU-GM	HE+PSCs	0nM	-0.1726	0.1423	36	-1.21	0.2330	0.05	-0.4612	0.1160
CFU-GM	E11.5AGM	5nM	CFU-GM	HE+PSCs	15nM	1.5085	0.1539	36	9.80	<.0001	0.05	1.1965	1.8206
CFU-GM	E11.5AGM	5nM	CFU-GM	HE+PSCs	5nM	0.7926	0.1467	36	5.40	<.0001	0.05	0.4950	1.0901
CFU-GM	HE+PSCs	0nM	CFU-GM	HE+PSCs	15nM	1.6812	0.1534	36	10.96	<.0001	0.05	1.3701	1.9922
CFU-GM	HE+PSCs	0nM	CFU-GM	HE+PSCs	5nM	0.9652	0.1462	36	6.60	<.0001	0.05	0.6686	1.2617
CFU-GM	HE+PSCs	15nM	CFU-GM	HE+PSCs	5nM	-0.7160	0.1575	36	-4.55	<.0001	0.05	-1.0353	-0.3966

The GENMOD Procedure

Model Information	
Data Set	WORK.EXT_FIG10BII_E11MK07_TRANS
Distribution	Normal
Link Function	Identity
Dependent Variable	Percentage

Number of Observations Read	45
Number of Observations Used	45

Class Level Information		
Class	Levels	Values
Condition	3	AoE+PSCs E11.5AGM HE+PSCs
Treatment	3	0nM 15nM 5nM

Parameter Information			
Parameter	Effect	Condition	Treatment
Prm1	Intercept		
Prm2	Condition	AoE+PSCs	
Prm3	Condition	E11.5AGM	
Prm4	Condition	HE+PSCs	
Prm5	Treatment		0nM
Prm6	Treatment		15nM
Prm7	Treatment		5nM
Prm8	Condition*Treatment	AoE+PSCs	0nM
Prm9	Condition*Treatment	AoE+PSCs	15nM
Prm10	Condition*Treatment	AoE+PSCs	5nM
Prm11	Condition*Treatment	E11.5AGM	0nM

The GENMOD Procedure

Parameter Information			
**Parameter**	**Effect**	**Condition**	**Treatment**
**Prm12**	Condition*Treatment	E11.5AGM	15nM
**Prm13**	Condition*Treatment	E11.5AGM	5nM
**Prm14**	Condition*Treatment	HE+PSCs	0nM
**Prm15**	Condition*Treatment	HE+PSCs	15nM
**Prm16**	Condition*Treatment	HE+PSCs	5nM

Criteria For Assessing Goodness Of Fit			
**Criterion**	**DF**	**Value**	**Value/DF**
**Deviance**	36	550.8000	15.3000
**Scaled Deviance**	36	45.0000	1.2500
**Pearson Chi-Square**	36	550.8000	15.3000
**Scaled Pearson X2**	36	45.0000	1.2500
**Log Likelihood**		-120.2082	
**Full Log Likelihood**		-120.2082	
**AIC (smaller is better)**		260.4164	
**AICC (smaller is better)**		266.8870	
**BIC (smaller is better)**		278.4830	

Algorithm converged.

The GENMOD Procedure

Analysis Of Maximum Likelihood Parameter Estimates									
Parameter			DF	Estimate	Standard Error	Wald 95% Confidence Limits		Wald Chi-Square	Pr > ChiSq
Intercept			1	9.0000	1.5646	5.9334	12.0666	33.09	<.0001
Condition	AoE+PSCs		1	5.4000	2.2127	1.0632	9.7368	5.96	0.0147
Condition	E11.5AGM		1	20.2000	2.2127	15.8632	24.5368	83.34	<.0001
Condition	HE+PSCs		0	0.0000	0.0000	0.0000	0.0000	.	.
Treatment	0nM		1	11.4000	2.2127	7.0632	15.7368	26.54	<.0001
Treatment	15nM		1	-6.6000	2.2127	-10.9368	-2.2632	8.90	0.0029
Treatment	5nM		0	0.0000	0.0000	0.0000	0.0000	.	.
Condition*Treatment	AoE+PSCs	0nM	1	3.2000	3.1292	-2.9332	9.3332	1.05	0.3065
Condition*Treatment	AoE+PSCs	15nM	1	-0.8000	3.1292	-6.9332	5.3332	0.07	0.7982
Condition*Treatment	AoE+PSCs	5nM	0	0.0000	0.0000	0.0000	0.0000	.	.
Condition*Treatment	E11.5AGM	0nM	1	18.0000	3.1292	11.8668	24.1332	33.09	<.0001
Condition*Treatment	E11.5AGM	15nM	1	-7.0000	3.1292	-13.1332	-0.8668	5.00	0.0253
Condition*Treatment	E11.5AGM	5nM	0	0.0000	0.0000	0.0000	0.0000	.	.
Condition*Treatment	HE+PSCs	0nM	0	0.0000	0.0000	0.0000	0.0000	.	.
Condition*Treatment	HE+PSCs	15nM	0	0.0000	0.0000	0.0000	0.0000	.	.
Condition*Treatment	HE+PSCs	5nM	0	0.0000	0.0000	0.0000	0.0000	.	.
Scale			1	3.4986	0.3688	2.8455	4.3015		

Note: The scale parameter was estimated by maximum likelihood.

Condition*Treatment Least Squares Means								
Condition	Treatment	Estimate	Standard Error	z Value	Pr > |z|	Alpha	Lower	Upper
AoE+PSCs	0nM	29.0000	1.5646	18.53	<.0001	0.05	25.9334	32.0666
AoE+PSCs	15nM	7.0000	1.5646	4.47	<.0001	0.05	3.9334	10.0666
AoE+PSCs	5nM	14.4000	1.5646	9.20	<.0001	0.05	11.3334	17.4666
E11.5AGM	0nM	58.6000	1.5646	37.45	<.0001	0.05	55.5334	61.6666

The GENMOD Procedure

Condition*Treatment Least Squares Means								
Condition	Treatment	Estimate	Standard Error	z Value	Pr > |z|	Alpha	Lower	Upper
E11.5AGM	15nM	15.6000	1.5646	9.97	<.0001	0.05	12.5334	18.6666
E11.5AGM	5nM	29.2000	1.5646	18.66	<.0001	0.05	26.1334	32.2666
HE+PSCs	0nM	20.4000	1.5646	13.04	<.0001	0.05	17.3334	23.4666
HE+PSCs	15nM	2.4000	1.5646	1.53	0.1250	0.05	-0.6666	5.4666
HE+PSCs	5nM	9.0000	1.5646	5.75	<.0001	0.05	5.9334	12.0666

Differences of Condition*Treatment Least Squares Means										
Condition	Treatment	_Condition	_Treatment	Estimate	Standard Error	z Value	Pr > |z|	Alpha	Lower	Upper
AoE+PSCs	0nM	AoE+PSCs	15nM	22.0000	2.2127	9.94	<.0001	0.05	17.6632	26.3368
AoE+PSCs	0nM	AoE+PSCs	5nM	14.6000	2.2127	6.60	<.0001	0.05	10.2632	18.9368
AoE+PSCs	0nM	E11.5AGM	0nM	-29.6000	2.2127	-13.38	<.0001	0.05	-33.9368	-25.2632
AoE+PSCs	0nM	E11.5AGM	15nM	13.4000	2.2127	6.06	<.0001	0.05	9.0632	17.7368
AoE+PSCs	0nM	E11.5AGM	5nM	-0.2000	2.2127	-0.09	0.9280	0.05	-4.5368	4.1368
AoE+PSCs	0nM	HE+PSCs	0nM	8.6000	2.2127	3.89	0.0001	0.05	4.2632	12.9368
AoE+PSCs	0nM	HE+PSCs	15nM	26.6000	2.2127	12.02	<.0001	0.05	22.2632	30.9368
AoE+PSCs	0nM	HE+PSCs	5nM	20.0000	2.2127	9.04	<.0001	0.05	15.6632	24.3368
AoE+PSCs	15nM	AoE+PSCs	5nM	-7.4000	2.2127	-3.34	0.0008	0.05	-11.7368	-3.0632
AoE+PSCs	15nM	E11.5AGM	0nM	-51.6000	2.2127	-23.32	<.0001	0.05	-55.9368	-47.2632
AoE+PSCs	15nM	E11.5AGM	15nM	-8.6000	2.2127	-3.89	0.0001	0.05	-12.9368	-4.2632
AoE+PSCs	15nM	E11.5AGM	5nM	-22.2000	2.2127	-10.03	<.0001	0.05	-26.5368	-17.8632
AoE+PSCs	15nM	HE+PSCs	0nM	-13.4000	2.2127	-6.06	<.0001	0.05	-17.7368	-9.0632
AoE+PSCs	15nM	HE+PSCs	15nM	4.6000	2.2127	2.08	0.0376	0.05	0.2632	8.9368
AoE+PSCs	15nM	HE+PSCs	5nM	-2.0000	2.2127	-0.90	0.3661	0.05	-6.3368	2.3368
AoE+PSCs	5nM	E11.5AGM	0nM	-44.2000	2.2127	-19.98	<.0001	0.05	-48.5368	-39.8632

The GENMOD Procedure

Differences of Condition*Treatment Least Squares Means

Condition	Treatment	_Condition	_Treatment	Estimate	Standard Error	z Value	Pr > |z|	Alpha	Lower	Upper
AoE+PSCs	5nM	E11.5AGM	15nM	-1.2000	2.2127	-0.54	0.5876	0.05	-5.5368	3.1368
AoE+PSCs	5nM	E11.5AGM	5nM	-14.8000	2.2127	-6.69	<.0001	0.05	-19.1368	-10.4632
AoE+PSCs	5nM	HE+PSCs	0nM	-6.0000	2.2127	-2.71	0.0067	0.05	-10.3368	-1.6632
AoE+PSCs	5nM	HE+PSCs	15nM	12.0000	2.2127	5.42	<.0001	0.05	7.6632	16.3368
AoE+PSCs	5nM	HE+PSCs	5nM	5.4000	2.2127	2.44	0.0147	0.05	1.0632	9.7368
E11.5AGM	0nM	E11.5AGM	15nM	43.0000	2.2127	19.43	<.0001	0.05	38.6632	47.3368
E11.5AGM	0nM	E11.5AGM	5nM	29.4000	2.2127	13.29	<.0001	0.05	25.0632	33.7368
E11.5AGM	0nM	HE+PSCs	0nM	38.2000	2.2127	17.26	<.0001	0.05	33.8632	42.5368
E11.5AGM	0nM	HE+PSCs	15nM	56.2000	2.2127	25.40	<.0001	0.05	51.8632	60.5368
E11.5AGM	0nM	HE+PSCs	5nM	49.6000	2.2127	22.42	<.0001	0.05	45.2632	53.9368
E11.5AGM	15nM	E11.5AGM	5nM	-13.6000	2.2127	-6.15	<.0001	0.05	-17.9368	-9.2632
E11.5AGM	15nM	HE+PSCs	0nM	-4.8000	2.2127	-2.17	0.0301	0.05	-9.1368	-0.4632
E11.5AGM	15nM	HE+PSCs	15nM	13.2000	2.2127	5.97	<.0001	0.05	8.8632	17.5368
E11.5AGM	15nM	HE+PSCs	5nM	6.6000	2.2127	2.98	0.0029	0.05	2.2632	10.9368
E11.5AGM	5nM	HE+PSCs	0nM	8.8000	2.2127	3.98	<.0001	0.05	4.4632	13.1368
E11.5AGM	5nM	HE+PSCs	15nM	26.8000	2.2127	12.11	<.0001	0.05	22.4632	31.1368
E11.5AGM	5nM	HE+PSCs	5nM	20.2000	2.2127	9.13	<.0001	0.05	15.8632	24.5368
HE+PSCs	0nM	HE+PSCs	15nM	18.0000	2.2127	8.13	<.0001	0.05	13.6632	22.3368
HE+PSCs	0nM	HE+PSCs	5nM	11.4000	2.2127	5.15	<.0001	0.05	7.0632	15.7368
HE+PSCs	15nM	HE+PSCs	5nM	-6.6000	2.2127	-2.98	0.0029	0.05	-10.9368	-2.2632

The GENMOD Procedure

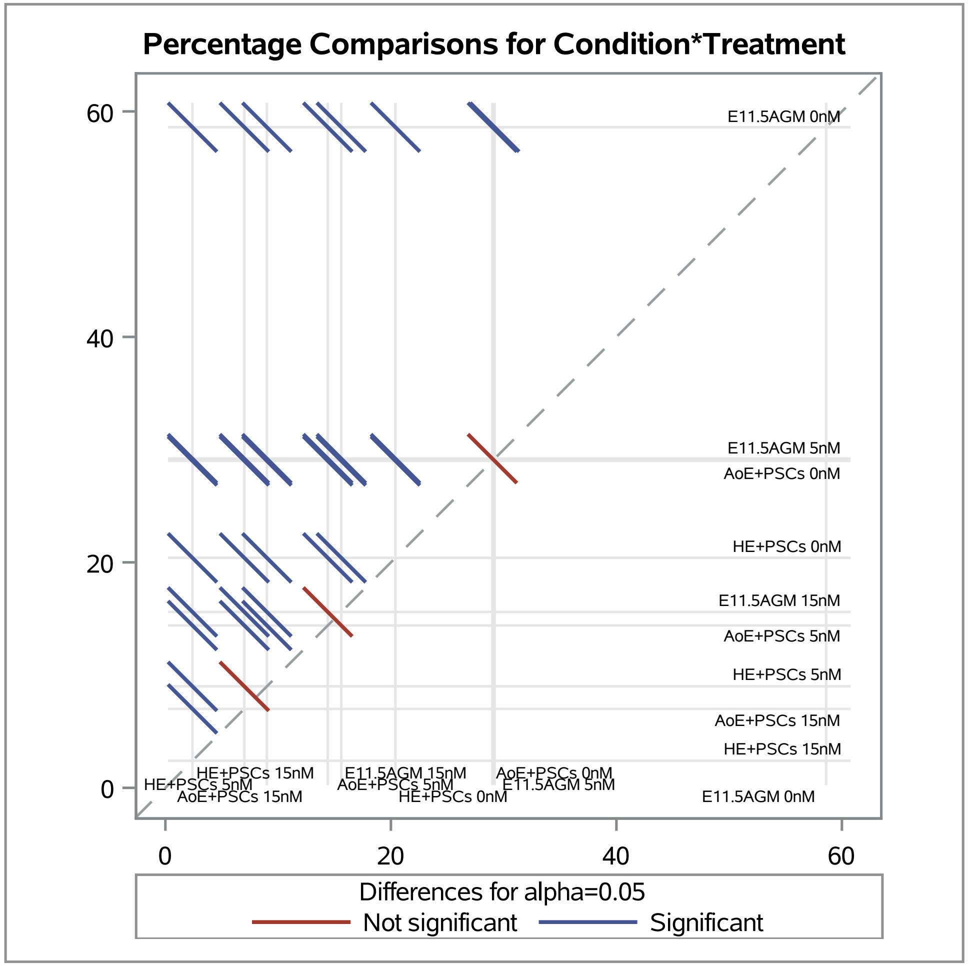

The GLIMMIX Procedure

Model Information	
**Data Set**	WORK.EXT_FIG10BIII_E10USAG
**Response Variable**	Count
**Response Distribution**	Poisson
**Link Function**	Log
**Variance Function**	Default
**Variance Matrix Blocked By**	Dish_ID
**Estimation Technique**	Maximum Likelihood
**Likelihood Approximation**	Laplace
**Degrees of Freedom Method**	Containment

Class Level Information		
**Class**	**Levels**	**Values**
**Colony_Type**	3	BFU-E CFU-GEMM CFU-GM
**Condition**	3	AoE+PSCs E10.5AGM HE+PSCs
**Treatment**	3	0ug/ml 1ug/ml 5ug/ml
**Dish_ID**	27	1 2 3 4 5 6 7 8 9 10 11 12 13 14 15 16 17 18 19 20 21 22 23 24 25 26 27

**Number of Observations Read**	81
**Number of Observations Used**	81

Dimensions	
**G-side Cov. Parameters**	1
**Columns in X**	64
**Columns in Z per Subject**	1
**Subjects (Blocks in V)**	27
**Max Obs per Subject**	3

The GLIMMIX Procedure

Optimization Information	
**Optimization Technique**	Dual Quasi-Newton
**Parameters in Optimization**	28
**Lower Boundaries**	1
**Upper Boundaries**	0
**Fixed Effects**	Not Profiled
**Starting From**	GLM estimates

Iteration History					
**Iteration**	**Restarts**	**Evaluations**	**Objective Function**	**Change**	**Max Gradient**
**0**	**0**	4	742.77526309	.	112.5163
**1**	**0**	6	742.75873397	0.01652912	16.90547
**2**	**0**	4	742.75536619	0.00336778	32.9664
**3**	**0**	4	742.73861976	0.01674643	1.778316
**4**	**0**	6	742.73658336	0.00203640	17.87056
**5**	**0**	2	742.73549365	0.00108972	4.181801
**6**	**0**	3	742.73513639	0.00035726	4.833719
**7**	**0**	2	742.73472146	0.00041493	3.345056
**8**	**0**	4	742.7330658	0.00165566	6.014422
**9**	**0**	3	742.73261071	0.00045509	0.917566
**10**	**0**	2	742.73252269	0.00008802	3.540188
**11**	**0**	4	742.7322652	0.00025750	0.670709
**12**	**0**	2	742.73213305	0.00013215	0.895953
**13**	**0**	3	742.73204997	0.00008308	0.549537
**14**	**0**	2	742.73196613	0.00008385	0.211282

The GLIMMIX Procedure

Iteration History					
Iteration	Restarts	Evaluations	Objective Function	Change	Max Gradient
15	0	3	742.73194772	0.00001841	0.177584
16	0	4	742.73184471	0.00010301	4.399931

Convergence criterion (GCONV=1E-8) satisfied.

Fit Statistics	
-2 Log Likelihood	742.73
AIC (smaller is better)	798.73
AICC (smaller is better)	829.96
BIC (smaller is better)	835.02
CAIC (smaller is better)	863.02
HQIC (smaller is better)	809.52

Fit Statistics for Conditional Distribution	
-2 log L(Count | r. effects)	692.07
Pearson Chi-Square	163.77
Pearson Chi-Square / DF	2.02

Covariance Parameter Estimates			
Cov Parm	Subject	Estimate	Standard Error
Intercept	Dish_ID	0.005512	0.002319

The GLIMMIX Procedure

Solutions for Fixed Effects								
**Effect**	**Colony_Type**	**Condition**	**Treatment**	**Estimate**	**Standard Error**	**DF**	**t Value**	**Pr > |t|**
**Intercept**				4.4736	0.07506	18	59.60	<.0001
**Colony_Type**	BFU-E			-1.3865	0.1376	36	-10.08	<.0001
**Colony_Type**	CFU-GEMM			-1.1103	0.1236	36	-8.98	<.0001
**Colony_Type**	CFU-GM			0	.	.	.	.
**Condition**		AoE+PSCs		0.1592	0.1035	36	1.54	0.1326
**Condition**		E10.5AGM		0.8658	0.09522	36	9.09	<.0001
**Condition**		HE+PSCs		0	.	.	.	.
**Colony_Typ*Condition**	BFU-E	AoE+PSCs		0.5130	0.1730	36	2.97	0.0053
**Colony_Typ*Condition**	BFU-E	E10.5AGM		-0.1058	0.1662	36	-0.64	0.5284
**Colony_Typ*Condition**	BFU-E	HE+PSCs		0	.	.	.	.
**Colony_Typ*Condition**	CFU-GEMM	AoE+PSCs		-0.05874	0.1701	36	-0.35	0.7319
**Colony_Typ*Condition**	CFU-GEMM	E10.5AGM		-0.2188	0.1513	36	-1.45	0.1568
**Colony_Typ*Condition**	CFU-GEMM	HE+PSCs		0	.	.	.	.
**Colony_Typ*Condition**	CFU-GM	AoE+PSCs		0	.	.	.	.
**Colony_Typ*Condition**	CFU-GM	E10.5AGM		0	.	.	.	.
**Colony_Typ*Condition**	CFU-GM	HE+PSCs		0	.	.	.	.
**Treatment**			0ug/ml	1.6325	0.09062	36	18.01	<.0001
**Treatment**			1ug/ml	0.7657	0.09609	36	7.97	<.0001
**Treatment**			5ug/ml	0	.	.	.	.
**Colony_Typ*Treatment**	BFU-E		0ug/ml	-0.1593	0.1521	36	-1.05	0.3021
**Colony_Typ*Treatment**	BFU-E		1ug/ml	-0.1851	0.1708	36	-1.08	0.2856
**Colony_Typ*Treatment**	BFU-E		5ug/ml	0	.	.	.	.
**Colony_Typ*Treatment**	CFU-GEMM		0ug/ml	-0.5225	0.1407	36	-3.71	0.0007
**Colony_Typ*Treatment**	CFU-GEMM		1ug/ml	-0.4198	0.1587	36	-2.65	0.0120

The GLIMMIX Procedure

Solutions for Fixed Effects								
Effect	Colony_Type	Condition	Treatment	Estimate	Standard Error	DF	t Value	Pr > |t|
**Colony_Typ*Treatment**	CFU-GEMM		5ug/ml	0	.	.	.	.
**Colony_Typ*Treatment**	CFU-GM		0ug/ml	0	.	.	.	.
**Colony_Typ*Treatment**	CFU-GM		1ug/ml	0	.	.	.	.
**Colony_Typ*Treatment**	CFU-GM		5ug/ml	0	.	.	.	.
**Condition*Treatment**		AoE+PSCs	0ug/ml	-0.07169	0.1257	36	-0.57	0.5721
**Condition*Treatment**		AoE+PSCs	1ug/ml	-0.04920	0.1332	36	-0.37	0.7139
**Condition*Treatment**		AoE+PSCs	5ug/ml	0	.	.	.	.
**Condition*Treatment**		E10.5AGM	0ug/ml	-0.3334	0.1180	36	-2.83	0.0076
**Condition*Treatment**		E10.5AGM	1ug/ml	-0.1145	0.1239	36	-0.92	0.3615
**Condition*Treatment**		E10.5AGM	5ug/ml	0	.	.	.	.
**Condition*Treatment**		HE+PSCs	0ug/ml	0	.	.	.	.
**Condition*Treatment**		HE+PSCs	1ug/ml	0	.	.	.	.
**Condition*Treatment**		HE+PSCs	5ug/ml	0	.	.	.	.
**Colony*Condit*Treatm**	BFU-E	AoE+PSCs	0ug/ml	-0.3908	0.1940	36	-2.01	0.0515
**Colony*Condit*Treatm**	BFU-E	AoE+PSCs	1ug/ml	-0.1501	0.2169	36	-0.69	0.4933
**Colony*Condit*Treatm**	BFU-E	AoE+PSCs	5ug/ml	0	.	.	.	.
**Colony*Condit*Treatm**	BFU-E	E10.5AGM	0ug/ml	0.3018	0.1843	36	1.64	0.1102
**Colony*Condit*Treatm**	BFU-E	E10.5AGM	1ug/ml	0.6130	0.2027	36	3.02	0.0046
**Colony*Condit*Treatm**	BFU-E	E10.5AGM	5ug/ml	0	.	.	.	.
**Colony*Condit*Treatm**	BFU-E	HE+PSCs	0ug/ml	0	.	.	.	.
**Colony*Condit*Treatm**	BFU-E	HE+PSCs	1ug/ml	0	.	.	.	.
**Colony*Condit*Treatm**	BFU-E	HE+PSCs	5ug/ml	0	.	.	.	.
**Colony*Condit*Treatm**	CFU-GEMM	AoE+PSCs	0ug/ml	0.1223	0.1934	36	0.63	0.5313
**Colony*Condit*Treatm**	CFU-GEMM	AoE+PSCs	1ug/ml	0.5044	0.2123	36	2.38	0.0230

The GLIMMIX Procedure

Solutions for Fixed Effects								
**Effect**	**Colony_Type**	**Condition**	**Treatment**	**Estimate**	**Standard Error**	**DF**	**t Value**	**Pr > |t|**
**Colony*Condit*Treatm**	CFU-GEMM	AoE+PSCs	5ug/ml	0	.	.	.	.
**Colony*Condit*Treatm**	CFU-GEMM	E10.5AGM	0ug/ml	0.3936	0.1724	36	2.28	0.0285
**Colony*Condit*Treatm**	CFU-GEMM	E10.5AGM	1ug/ml	0.6377	0.1901	36	3.35	0.0019
**Colony*Condit*Treatm**	CFU-GEMM	E10.5AGM	5ug/ml	0	.	.	.	.
**Colony*Condit*Treatm**	CFU-GEMM	HE+PSCs	0ug/ml	0	.	.	.	.
**Colony*Condit*Treatm**	CFU-GEMM	HE+PSCs	1ug/ml	0	.	.	.	.
**Colony*Condit*Treatm**	CFU-GEMM	HE+PSCs	5ug/ml	0	.	.	.	.
**Colony*Condit*Treatm**	CFU-GM	AoE+PSCs	0ug/ml	0	.	.	.	.
**Colony*Condit*Treatm**	CFU-GM	AoE+PSCs	1ug/ml	0	.	.	.	.
**Colony*Condit*Treatm**	CFU-GM	AoE+PSCs	5ug/ml	0	.	.	.	.
**Colony*Condit*Treatm**	CFU-GM	E10.5AGM	0ug/ml	0	.	.	.	.
**Colony*Condit*Treatm**	CFU-GM	E10.5AGM	1ug/ml	0	.	.	.	.
**Colony*Condit*Treatm**	CFU-GM	E10.5AGM	5ug/ml	0	.	.	.	.
**Colony*Condit*Treatm**	CFU-GM	HE+PSCs	0ug/ml	0	.	.	.	.
**Colony*Condit*Treatm**	CFU-GM	HE+PSCs	1ug/ml	0	.	.	.	.
**Colony*Condit*Treatm**	CFU-GM	HE+PSCs	5ug/ml	0	.	.	.	.

Type III Tests of Fixed Effects				
**Effect**	**Num DF**	**Den DF**	**F Value**	**Pr > F**
**Colony_Type**	2	36	1733.86	<.0001
**Condition**	2	36	165.35	<.0001
**Colony_Typ*Condition**	4	36	4.99	0.0026
**Treatment**	2	36	387.01	<.0001
**Colony_Typ*Treatment**	4	36	11.28	<.0001

The GLIMMIX Procedure

Type III Tests of Fixed Effects

Effect	Num DF	Den DF	F Value	Pr > F
Condition*Treatment	4	36	4.09	0.0078
Colony*Condit*Treatm	8	36	4.85	0.0004

Condition*Treatment Least Squares Means

Condition	Treatment	Estimate	Standard Error	DF	t Value	Pr > |t|	Alpha	Lower	Upper	Mean	Standard Error Mean	Lower Mean	Upper Mean
AoE+PSCs	0ug/ml	5.1960	0.05089	36	102.10	<.0001	0.05	5.0928	5.2992	180.55	9.1886	162.84	200.18
AoE+PSCs	1ug/ml	4.5849	0.05588	36	82.04	<.0001	0.05	4.4716	4.6983	97.9951	5.4763	87.4948	109.76
AoE+PSCs	5ug/ml	3.9520	0.06494	36	60.85	<.0001	0.05	3.8203	4.0837	52.0390	3.3796	45.6171	59.3650
E10.5AGM	0ug/ml	5.7026	0.04776	36	119.40	<.0001	0.05	5.6057	5.7994	299.64	14.3105	271.98	330.11
E10.5AGM	1ug/ml	5.2654	0.04981	36	105.72	<.0001	0.05	5.1644	5.3665	193.53	9.6390	174.94	214.10
E10.5AGM	5ug/ml	4.3990	0.05896	36	74.61	<.0001	0.05	4.2794	4.5185	81.3665	4.7970	72.1969	91.7008
HE+PSCs	0ug/ml	5.0465	0.05225	36	96.59	<.0001	0.05	4.9406	5.1525	155.48	8.1234	139.85	172.86
HE+PSCs	1ug/ml	4.2054	0.06233	36	67.47	<.0001	0.05	4.0790	4.3318	67.0465	4.1788	59.0853	76.0804
HE+PSCs	5ug/ml	3.6413	0.07231	36	50.36	<.0001	0.05	3.4947	3.7880	38.1433	2.7580	32.9407	44.1676

Differences of Condition*Treatment Least Squares Means

Condition	Treatment	_Condition	_Treatment	Estimate	Standard Error	DF	t Value	Pr > |t|	Alpha	Lower	Upper
AoE+PSCs	0ug/ml	AoE+PSCs	1ug/ml	0.6111	0.07558	36	8.08	<.0001	0.05	0.4578	0.7644
AoE+PSCs	0ug/ml	AoE+PSCs	5ug/ml	1.2440	0.08251	36	15.08	<.0001	0.05	1.0767	1.4113
AoE+PSCs	0ug/ml	E10.5AGM	0ug/ml	-0.5066	0.06979	36	-7.26	<.0001	0.05	-0.6481	-0.3650
AoE+PSCs	0ug/ml	E10.5AGM	1ug/ml	-0.06944	0.07121	36	-0.98	0.3360	0.05	-0.2139	0.07498
AoE+PSCs	0ug/ml	E10.5AGM	5ug/ml	0.7970	0.07788	36	10.23	<.0001	0.05	0.6391	0.9550
AoE+PSCs	0ug/ml	HE+PSCs	0ug/ml	0.1495	0.07294	36	2.05	0.0478	0.05	0.001537	0.2974

The GLIMMIX Procedure

Differences of Condition*Treatment Least Squares Means											
Condition	Treatment	_Condition	_Treatment	Estimate	Standard Error	DF	t Value	Pr > |t|	Alpha	Lower	Upper
AoE+PSCs	0ug/ml	HE+PSCs	1ug/ml	0.9906	0.08046	36	12.31	<.0001	0.05	0.8274	1.1538
AoE+PSCs	0ug/ml	HE+PSCs	5ug/ml	1.5547	0.08842	36	17.58	<.0001	0.05	1.3753	1.7340
AoE+PSCs	1ug/ml	AoE+PSCs	5ug/ml	0.6329	0.08567	36	7.39	<.0001	0.05	0.4592	0.8067
AoE+PSCs	1ug/ml	E10.5AGM	0ug/ml	-1.1177	0.07351	36	-15.20	<.0001	0.05	-1.2667	-0.9686
AoE+PSCs	1ug/ml	E10.5AGM	1ug/ml	-0.6805	0.07485	36	-9.09	<.0001	0.05	-0.8323	-0.5287
AoE+PSCs	1ug/ml	E10.5AGM	5ug/ml	0.1860	0.08123	36	2.29	0.0280	0.05	0.02122	0.3507
AoE+PSCs	1ug/ml	HE+PSCs	0ug/ml	-0.4616	0.07650	36	-6.03	<.0001	0.05	-0.6168	-0.3065
AoE+PSCs	1ug/ml	HE+PSCs	1ug/ml	0.3795	0.08370	36	4.53	<.0001	0.05	0.2098	0.5493
AoE+PSCs	1ug/ml	HE+PSCs	5ug/ml	0.9436	0.09137	36	10.33	<.0001	0.05	0.7583	1.1289
AoE+PSCs	5ug/ml	E10.5AGM	0ug/ml	-1.7506	0.08061	36	-21.72	<.0001	0.05	-1.9141	-1.5871
AoE+PSCs	5ug/ml	E10.5AGM	1ug/ml	-1.3134	0.08184	36	-16.05	<.0001	0.05	-1.4794	-1.1475
AoE+PSCs	5ug/ml	E10.5AGM	5ug/ml	-0.4470	0.08771	36	-5.10	<.0001	0.05	-0.6249	-0.2691
AoE+PSCs	5ug/ml	HE+PSCs	0ug/ml	-1.0945	0.08335	36	-13.13	<.0001	0.05	-1.2636	-0.9255
AoE+PSCs	5ug/ml	HE+PSCs	1ug/ml	-0.2534	0.09001	36	-2.82	0.0079	0.05	-0.4359	-0.07085
AoE+PSCs	5ug/ml	HE+PSCs	5ug/ml	0.3106	0.09718	36	3.20	0.0029	0.05	0.1135	0.5077
E10.5AGM	0ug/ml	E10.5AGM	1ug/ml	0.4371	0.06900	36	6.33	<.0001	0.05	0.2972	0.5771
E10.5AGM	0ug/ml	E10.5AGM	5ug/ml	1.3036	0.07587	36	17.18	<.0001	0.05	1.1497	1.4575
E10.5AGM	0ug/ml	HE+PSCs	0ug/ml	0.6560	0.07078	36	9.27	<.0001	0.05	0.5125	0.7996
E10.5AGM	0ug/ml	HE+PSCs	1ug/ml	1.4972	0.07852	36	19.07	<.0001	0.05	1.3379	1.6564
E10.5AGM	0ug/ml	HE+PSCs	5ug/ml	2.0612	0.08665	36	23.79	<.0001	0.05	1.8855	2.2370
E10.5AGM	1ug/ml	E10.5AGM	5ug/ml	0.8665	0.07717	36	11.23	<.0001	0.05	0.7100	1.0230
E10.5AGM	1ug/ml	HE+PSCs	0ug/ml	0.2189	0.07218	36	3.03	0.0045	0.05	0.07251	0.3653
E10.5AGM	1ug/ml	HE+PSCs	1ug/ml	1.0601	0.07977	36	13.29	<.0001	0.05	0.8983	1.2218
E10.5AGM	1ug/ml	HE+PSCs	5ug/ml	1.6241	0.08779	36	18.50	<.0001	0.05	1.4460	1.8021

The GLIMMIX Procedure

Differences of Condition*Treatment Least Squares Means

Condition	Treatment	_Condition	_Treatment	Estimate	Standard Error	DF	t Value	Pr > |t|	Alpha	Lower	Upper
E10.5AGM	5ug/ml	HE+PSCs	0ug/ml	-0.6476	0.07877	36	-8.22	<.0001	0.05	-0.8073	-0.4878
E10.5AGM	5ug/ml	HE+PSCs	1ug/ml	0.1936	0.08578	36	2.26	0.0302	0.05	0.01960	0.3676
E10.5AGM	5ug/ml	HE+PSCs	5ug/ml	0.7576	0.09328	36	8.12	<.0001	0.05	0.5684	0.9468
HE+PSCs	0ug/ml	HE+PSCs	1ug/ml	0.8412	0.08132	36	10.34	<.0001	0.05	0.6762	1.0061
HE+PSCs	0ug/ml	HE+PSCs	5ug/ml	1.4052	0.08919	36	15.75	<.0001	0.05	1.2243	1.5861
HE+PSCs	1ug/ml	HE+PSCs	5ug/ml	0.5640	0.09544	36	5.91	<.0001	0.05	0.3705	0.7576

Colony*Condit*Treatm Least Squares Means

Colony_Type	Condition	Treatment	Estimate	Standard Error	DF	t Value	Pr > |t|	Alpha	Lower	Upper	Mean	Standard Error Mean	Lower Mean	Upper Mean
BFU-E	AoE+PSCs	0ug/ml	4.7701	0.06828	36	69.86	<.0001	0.05	4.6316	4.9085	117.93	8.0519	102.68	135.44
BFU-E	AoE+PSCs	1ug/ml	4.1406	0.08445	36	49.03	<.0001	0.05	3.9693	4.3119	62.8406	5.3066	52.9495	74.5794
BFU-E	AoE+PSCs	5ug/ml	3.7593	0.09794	36	38.38	<.0001	0.05	3.5607	3.9580	42.9196	4.2035	35.1877	52.3504
BFU-E	E10.5AGM	0ug/ml	5.2887	0.05932	36	89.16	<.0001	0.05	5.1684	5.4090	198.08	11.7493	175.63	223.40
BFU-E	E10.5AGM	1ug/ml	4.9262	0.06519	36	75.57	<.0001	0.05	4.7940	5.0585	137.86	8.9871	120.79	157.35
BFU-E	E10.5AGM	5ug/ml	3.8471	0.09452	36	40.70	<.0001	0.05	3.6554	4.0388	46.8577	4.4290	38.6838	56.7587
BFU-E	HE+PSCs	0ug/ml	4.5603	0.07289	36	62.56	<.0001	0.05	4.4125	4.7081	95.6106	6.9692	82.4715	110.84
BFU-E	HE+PSCs	1ug/ml	3.6677	0.1016	36	36.10	<.0001	0.05	3.4616	3.8737	39.1607	3.9784	31.8691	48.1207
BFU-E	HE+PSCs	5ug/ml	3.0871	0.1304	36	23.68	<.0001	0.05	2.8227	3.3515	21.9136	2.8568	16.8224	28.5456
CFU-GEMM	AoE+PSCs	0ug/ml	4.6243	0.07145	36	64.72	<.0001	0.05	4.4794	4.7692	101.93	7.2833	88.1832	117.83
CFU-GEMM	AoE+PSCs	1ug/ml	4.2648	0.08070	36	52.85	<.0001	0.05	4.1012	4.4285	71.1525	5.7422	60.4098	83.8055
CFU-GEMM	AoE+PSCs	5ug/ml	3.4638	0.1107	36	31.29	<.0001	0.05	3.2393	3.6884	31.9384	3.5361	25.5150	39.9789
CFU-GEMM	E10.5AGM	0ug/ml	5.1805	0.06091	36	85.05	<.0001	0.05	5.0570	5.3041	177.78	10.8288	157.12	201.15
CFU-GEMM	E10.5AGM	1ug/ml	4.8794	0.06607	36	73.85	<.0001	0.05	4.7454	5.0134	131.56	8.6919	115.06	150.42

The GLIMMIX Procedure

Colony*Condit*Treatm Least Squares Means														
Colony_Type	Condition	Treatment	Estimate	Standard Error	DF	t Value	Pr > |t|	Alpha	Lower	Upper	Mean	Standard Error Mean	Lower Mean	Upper Mean
CFU-GEMM	E10.5AGM	5ug/ml	4.0103	0.08869	36	45.22	<.0001	0.05	3.8305	4.1902	55.1655	4.8926	46.0841	66.0365
CFU-GEMM	HE+PSCs	0ug/ml	4.4733	0.07503	36	59.62	<.0001	0.05	4.3211	4.6254	87.6422	6.5755	75.2715	102.05
CFU-GEMM	HE+PSCs	1ug/ml	3.7092	0.09988	36	37.14	<.0001	0.05	3.5066	3.9117	40.8202	4.0772	33.3351	49.9861
CFU-GEMM	HE+PSCs	5ug/ml	3.3633	0.1155	36	29.12	<.0001	0.05	3.1291	3.5976	28.8850	3.3361	22.8530	36.5090
CFU-GM	AoE+PSCs	0ug/ml	6.1936	0.05018	36	123.43	<.0001	0.05	6.0919	6.2954	489.61	24.5680	442.24	542.06
CFU-GM	AoE+PSCs	1ug/ml	5.3493	0.05848	36	91.47	<.0001	0.05	5.2307	5.4679	210.47	12.3085	186.93	236.97
CFU-GM	AoE+PSCs	5ug/ml	4.6328	0.07124	36	65.03	<.0001	0.05	4.4884	4.7773	102.81	7.3243	88.9749	118.79
CFU-GM	E10.5AGM	0ug/ml	6.6385	0.04768	36	139.23	<.0001	0.05	6.5418	6.7352	763.96	36.4265	693.54	841.52
CFU-GM	E10.5AGM	1ug/ml	5.9906	0.05168	36	115.91	<.0001	0.05	5.8858	6.0955	399.67	20.6562	359.90	443.84
CFU-GM	E10.5AGM	5ug/ml	5.3394	0.05862	36	91.09	<.0001	0.05	5.2206	5.4583	208.40	12.2154	185.04	234.70
CFU-GM	HE+PSCs	0ug/ml	6.1061	0.05080	36	120.20	<.0001	0.05	6.0031	6.2091	448.58	22.7884	404.66	497.26
CFU-GM	HE+PSCs	1ug/ml	5.2393	0.06004	36	87.27	<.0001	0.05	5.1176	5.3611	188.54	11.3190	166.93	212.95
CFU-GM	HE+PSCs	5ug/ml	4.4736	0.07506	36	59.60	<.0001	0.05	4.3214	4.6258	87.6736	6.5805	75.2939	102.09

The GLIMMIX Procedure

Differences of Colony*Condit*Treatm Least Squares Means													
Colony_Type	Condition	Treatment	_Colony_Type	_Condition	_Treatment	Estimate	Standard Error	DF	t Value	Pr > |t|	Alpha	Lower	Upper
BFU-E	AoE+PSCs	0ug/ml	BFU-E	AoE+PSCs	1ug/ml	0.6295	0.1086	36	5.80	<.0001	0.05	0.4092	0.8497
BFU-E	AoE+PSCs	0ug/ml	BFU-E	AoE+PSCs	5ug/ml	1.0107	0.1194	36	8.47	<.0001	0.05	0.7686	1.2529
BFU-E	AoE+PSCs	0ug/ml	BFU-E	E10.5AGM	0ug/ml	-0.5186	0.09045	36	-5.73	<.0001	0.05	-0.7021	-0.3352
BFU-E	AoE+PSCs	0ug/ml	BFU-E	E10.5AGM	1ug/ml	-0.1562	0.09440	36	-1.65	0.1067	0.05	-0.3476	0.03527
BFU-E	AoE+PSCs	0ug/ml	BFU-E	E10.5AGM	5ug/ml	0.9229	0.1166	36	7.92	<.0001	0.05	0.6865	1.1594
BFU-E	AoE+PSCs	0ug/ml	BFU-E	HE+PSCs	0ug/ml	0.2098	0.09988	36	2.10	0.0428	0.05	0.007215	0.4123
BFU-E	AoE+PSCs	0ug/ml	BFU-E	HE+PSCs	1ug/ml	1.1024	0.1224	36	9.01	<.0001	0.05	0.8541	1.3506
BFU-E	AoE+PSCs	0ug/ml	BFU-E	HE+PSCs	5ug/ml	1.6829	0.1472	36	11.44	<.0001	0.05	1.3845	1.9814
BFU-E	AoE+PSCs	0ug/ml	CFU-GEMM	AoE+PSCs	0ug/ml	0.1457	0.07805	36	1.87	0.0700	0.05	-0.01257	0.3040
BFU-E	AoE+PSCs	0ug/ml	CFU-GEMM	AoE+PSCs	1ug/ml	0.5052	0.1057	36	4.78	<.0001	0.05	0.2908	0.7196
BFU-E	AoE+PSCs	0ug/ml	CFU-GEMM	AoE+PSCs	5ug/ml	1.3062	0.1301	36	10.04	<.0001	0.05	1.0424	1.5701
BFU-E	AoE+PSCs	0ug/ml	CFU-GEMM	E10.5AGM	0ug/ml	-0.4105	0.09150	36	-4.49	<.0001	0.05	-0.5960	-0.2249
BFU-E	AoE+PSCs	0ug/ml	CFU-GEMM	E10.5AGM	1ug/ml	-0.1094	0.09501	36	-1.15	0.2572	0.05	-0.3021	0.08331
BFU-E	AoE+PSCs	0ug/ml	CFU-GEMM	E10.5AGM	5ug/ml	0.7597	0.1119	36	6.79	<.0001	0.05	0.5327	0.9867
BFU-E	AoE+PSCs	0ug/ml	CFU-GEMM	HE+PSCs	0ug/ml	0.2968	0.1014	36	2.93	0.0059	0.05	0.09106	0.5025
BFU-E	AoE+PSCs	0ug/ml	CFU-GEMM	HE+PSCs	1ug/ml	1.0609	0.1210	36	8.77	<.0001	0.05	0.8155	1.3063
BFU-E	AoE+PSCs	0ug/ml	CFU-GEMM	HE+PSCs	5ug/ml	1.4067	0.1342	36	10.48	<.0001	0.05	1.1346	1.6788
BFU-E	AoE+PSCs	0ug/ml	CFU-GM	AoE+PSCs	0ug/ml	-1.4236	0.05920	36	-24.05	<.0001	0.05	-1.5436	-1.3035
BFU-E	AoE+PSCs	0ug/ml	CFU-GM	AoE+PSCs	1ug/ml	-0.5793	0.08990	36	-6.44	<.0001	0.05	-0.7616	-0.3969
BFU-E	AoE+PSCs	0ug/ml	CFU-GM	AoE+PSCs	5ug/ml	0.1372	0.09868	36	1.39	0.1729	0.05	-0.06292	0.3373
BFU-E	AoE+PSCs	0ug/ml	CFU-GM	E10.5AGM	0ug/ml	-1.8685	0.08328	36	-22.44	<.0001	0.05	-2.0374	-1.6996
BFU-E	AoE+PSCs	0ug/ml	CFU-GM	E10.5AGM	1ug/ml	-1.2206	0.08563	36	-14.25	<.0001	0.05	-1.3943	-1.0469
BFU-E	AoE+PSCs	0ug/ml	CFU-GM	E10.5AGM	5ug/ml	-0.5694	0.08999	36	-6.33	<.0001	0.05	-0.7519	-0.3869
BFU-E	AoE+PSCs	0ug/ml	CFU-GM	HE+PSCs	0ug/ml	-1.3360	0.08510	36	-15.70	<.0001	0.05	-1.5086	-1.1634

The GLIMMIX Procedure

Differences of Colony*Condit*Treatm Least Squares Means													
Colony_Type	Condition	Treatment	_Colony_Type	_Condition	_Treatment	Estimate	Standard Error	DF	t Value	Pr > |t|	Alpha	Lower	Upper
BFU-E	AoE+PSCs	0ug/ml	CFU-GM	HE+PSCs	1ug/ml	-0.4693	0.09092	36	-5.16	<.0001	0.05	-0.6536	-0.2849
BFU-E	AoE+PSCs	0ug/ml	CFU-GM	HE+PSCs	5ug/ml	0.2964	0.1015	36	2.92	0.0060	0.05	0.09065	0.5022
BFU-E	AoE+PSCs	1ug/ml	BFU-E	AoE+PSCs	5ug/ml	0.3813	0.1293	36	2.95	0.0056	0.05	0.1190	0.6435
BFU-E	AoE+PSCs	1ug/ml	BFU-E	E10.5AGM	0ug/ml	-1.1481	0.1032	36	-11.13	<.0001	0.05	-1.3574	-0.9388
BFU-E	AoE+PSCs	1ug/ml	BFU-E	E10.5AGM	1ug/ml	-0.7856	0.1067	36	-7.36	<.0001	0.05	-1.0020	-0.5693
BFU-E	AoE+PSCs	1ug/ml	BFU-E	E10.5AGM	5ug/ml	0.2935	0.1267	36	2.32	0.0264	0.05	0.03644	0.5505
BFU-E	AoE+PSCs	1ug/ml	BFU-E	HE+PSCs	0ug/ml	-0.4197	0.1116	36	-3.76	0.0006	0.05	-0.6459	-0.1934
BFU-E	AoE+PSCs	1ug/ml	BFU-E	HE+PSCs	1ug/ml	0.4729	0.1321	36	3.58	0.0010	0.05	0.2050	0.7408
BFU-E	AoE+PSCs	1ug/ml	BFU-E	HE+PSCs	5ug/ml	1.0535	0.1553	36	6.78	<.0001	0.05	0.7385	1.3685
BFU-E	AoE+PSCs	1ug/ml	CFU-GEMM	AoE+PSCs	0ug/ml	-0.4837	0.1106	36	-4.37	0.0001	0.05	-0.7081	-0.2594
BFU-E	AoE+PSCs	1ug/ml	CFU-GEMM	AoE+PSCs	1ug/ml	-0.1242	0.09982	36	-1.24	0.2213	0.05	-0.3267	0.07821
BFU-E	AoE+PSCs	1ug/ml	CFU-GEMM	AoE+PSCs	5ug/ml	0.6768	0.1392	36	4.86	<.0001	0.05	0.3944	0.9592
BFU-E	AoE+PSCs	1ug/ml	CFU-GEMM	E10.5AGM	0ug/ml	-1.0399	0.1041	36	-9.99	<.0001	0.05	-1.2511	-0.8288
BFU-E	AoE+PSCs	1ug/ml	CFU-GEMM	E10.5AGM	1ug/ml	-0.7388	0.1072	36	-6.89	<.0001	0.05	-0.9563	-0.5214
BFU-E	AoE+PSCs	1ug/ml	CFU-GEMM	E10.5AGM	5ug/ml	0.1303	0.1225	36	1.06	0.2945	0.05	-0.1181	0.3786
BFU-E	AoE+PSCs	1ug/ml	CFU-GEMM	HE+PSCs	0ug/ml	-0.3327	0.1130	36	-2.95	0.0056	0.05	-0.5617	-0.1036
BFU-E	AoE+PSCs	1ug/ml	CFU-GEMM	HE+PSCs	1ug/ml	0.4314	0.1308	36	3.30	0.0022	0.05	0.1662	0.6967
BFU-E	AoE+PSCs	1ug/ml	CFU-GEMM	HE+PSCs	5ug/ml	0.7773	0.1431	36	5.43	<.0001	0.05	0.4871	1.0674
BFU-E	AoE+PSCs	1ug/ml	CFU-GM	AoE+PSCs	0ug/ml	-2.0530	0.09823	36	-20.90	<.0001	0.05	-2.2522	-1.8538
BFU-E	AoE+PSCs	1ug/ml	CFU-GM	AoE+PSCs	1ug/ml	-1.2087	0.08289	36	-14.58	<.0001	0.05	-1.3768	-1.0406
BFU-E	AoE+PSCs	1ug/ml	CFU-GM	AoE+PSCs	5ug/ml	-0.4922	0.1105	36	-4.46	<.0001	0.05	-0.7163	-0.2682
BFU-E	AoE+PSCs	1ug/ml	CFU-GM	E10.5AGM	0ug/ml	-2.4979	0.09698	36	-25.76	<.0001	0.05	-2.6946	-2.3012
BFU-E	AoE+PSCs	1ug/ml	CFU-GM	E10.5AGM	1ug/ml	-1.8500	0.09900	36	-18.69	<.0001	0.05	-2.0508	-1.6493
BFU-E	AoE+PSCs	1ug/ml	CFU-GM	E10.5AGM	5ug/ml	-1.1988	0.1028	36	-11.66	<.0001	0.05	-1.4073	-0.9904

The GLIMMIX Procedure

Differences of Colony*Condit*Treatm Least Squares Means													
Colony_Type	Condition	Treatment	_Colony_Type	_Condition	_Treatment	Estimate	Standard Error	DF	t Value	Pr > |t|	Alpha	Lower	Upper
BFU-E	AoE+PSCs	1ug/ml	CFU-GM	HE+PSCs	0ug/ml	-1.9655	0.09854	36	-19.95	<.0001	0.05	-2.1653	-1.7656
BFU-E	AoE+PSCs	1ug/ml	CFU-GM	HE+PSCs	1ug/ml	-1.0987	0.1036	36	-10.60	<.0001	0.05	-1.3088	-0.8886
BFU-E	AoE+PSCs	1ug/ml	CFU-GM	HE+PSCs	5ug/ml	-0.3330	0.1130	36	-2.95	0.0056	0.05	-0.5621	-0.1039
BFU-E	AoE+PSCs	5ug/ml	BFU-E	E10.5AGM	0ug/ml	-1.5293	0.1145	36	-13.36	<.0001	0.05	-1.7616	-1.2971
BFU-E	AoE+PSCs	5ug/ml	BFU-E	E10.5AGM	1ug/ml	-1.1669	0.1176	36	-9.92	<.0001	0.05	-1.4055	-0.9283
BFU-E	AoE+PSCs	5ug/ml	BFU-E	E10.5AGM	5ug/ml	-0.08779	0.1361	36	-0.64	0.5230	0.05	-0.3638	0.1883
BFU-E	AoE+PSCs	5ug/ml	BFU-E	HE+PSCs	0ug/ml	-0.8010	0.1221	36	-6.56	<.0001	0.05	-1.0486	-0.5534
BFU-E	AoE+PSCs	5ug/ml	BFU-E	HE+PSCs	1ug/ml	0.09165	0.1411	36	0.65	0.5201	0.05	-0.1945	0.3778
BFU-E	AoE+PSCs	5ug/ml	BFU-E	HE+PSCs	5ug/ml	0.6722	0.1631	36	4.12	0.0002	0.05	0.3415	1.0029
BFU-E	AoE+PSCs	5ug/ml	CFU-GEMM	AoE+PSCs	0ug/ml	-0.8650	0.1212	36	-7.14	<.0001	0.05	-1.1109	-0.6191
BFU-E	AoE+PSCs	5ug/ml	CFU-GEMM	AoE+PSCs	1ug/ml	-0.5055	0.1269	36	-3.98	0.0003	0.05	-0.7629	-0.2481
BFU-E	AoE+PSCs	5ug/ml	CFU-GEMM	AoE+PSCs	5ug/ml	0.2955	0.1348	36	2.19	0.0349	0.05	0.02212	0.5689
BFU-E	AoE+PSCs	5ug/ml	CFU-GEMM	E10.5AGM	0ug/ml	-1.4212	0.1153	36	-12.32	<.0001	0.05	-1.6551	-1.1873
BFU-E	AoE+PSCs	5ug/ml	CFU-GEMM	E10.5AGM	1ug/ml	-1.1201	0.1181	36	-9.48	<.0001	0.05	-1.3597	-0.8805
BFU-E	AoE+PSCs	5ug/ml	CFU-GEMM	E10.5AGM	5ug/ml	-0.2510	0.1321	36	-1.90	0.0655	0.05	-0.5190	0.01695
BFU-E	AoE+PSCs	5ug/ml	CFU-GEMM	HE+PSCs	0ug/ml	-0.7139	0.1234	36	-5.79	<.0001	0.05	-0.9641	-0.4637
BFU-E	AoE+PSCs	5ug/ml	CFU-GEMM	HE+PSCs	1ug/ml	0.05015	0.1399	36	0.36	0.7221	0.05	-0.2335	0.3338
BFU-E	AoE+PSCs	5ug/ml	CFU-GEMM	HE+PSCs	5ug/ml	0.3960	0.1514	36	2.62	0.0129	0.05	0.08890	0.7031
BFU-E	AoE+PSCs	5ug/ml	CFU-GM	AoE+PSCs	0ug/ml	-2.4343	0.1100	36	-22.12	<.0001	0.05	-2.6575	-2.2111
BFU-E	AoE+PSCs	5ug/ml	CFU-GM	AoE+PSCs	1ug/ml	-1.5900	0.1141	36	-13.94	<.0001	0.05	-1.8213	-1.3587
BFU-E	AoE+PSCs	5ug/ml	CFU-GM	AoE+PSCs	5ug/ml	-0.8735	0.1048	36	-8.33	<.0001	0.05	-1.0861	-0.6609
BFU-E	AoE+PSCs	5ug/ml	CFU-GM	E10.5AGM	0ug/ml	-2.8792	0.1089	36	-26.43	<.0001	0.05	-3.1001	-2.6583
BFU-E	AoE+PSCs	5ug/ml	CFU-GM	E10.5AGM	1ug/ml	-2.2313	0.1107	36	-20.15	<.0001	0.05	-2.4559	-2.0067
BFU-E	AoE+PSCs	5ug/ml	CFU-GM	E10.5AGM	5ug/ml	-1.5801	0.1141	36	-13.84	<.0001	0.05	-1.8116	-1.3486

The GLIMMIX Procedure

Differences of Colony*Condit*Treatm Least Squares Means													
Colony_Type	Condition	Treatment	_Colony_Type	_Condition	_Treatment	Estimate	Standard Error	DF	t Value	Pr > |t|	Alpha	Lower	Upper
BFU-E	AoE+PSCs	5ug/ml	CFU-GM	HE+PSCs	0ug/ml	-2.3468	0.1103	36	-21.27	<.0001	0.05	-2.5705	-2.1230
BFU-E	AoE+PSCs	5ug/ml	CFU-GM	HE+PSCs	1ug/ml	-1.4800	0.1149	36	-12.88	<.0001	0.05	-1.7129	-1.2470
BFU-E	AoE+PSCs	5ug/ml	CFU-GM	HE+PSCs	5ug/ml	-0.7143	0.1234	36	-5.79	<.0001	0.05	-0.9645	-0.4641
BFU-E	E10.5AGM	0ug/ml	BFU-E	E10.5AGM	1ug/ml	0.3624	0.08814	36	4.11	0.0002	0.05	0.1837	0.5412
BFU-E	E10.5AGM	0ug/ml	BFU-E	E10.5AGM	5ug/ml	1.4416	0.1116	36	12.92	<.0001	0.05	1.2152	1.6679
BFU-E	E10.5AGM	0ug/ml	BFU-E	HE+PSCs	0ug/ml	0.7284	0.09398	36	7.75	<.0001	0.05	0.5378	0.9190
BFU-E	E10.5AGM	0ug/ml	BFU-E	HE+PSCs	1ug/ml	1.6210	0.1176	36	13.78	<.0001	0.05	1.3824	1.8596
BFU-E	E10.5AGM	0ug/ml	BFU-E	HE+PSCs	5ug/ml	2.2016	0.1432	36	15.37	<.0001	0.05	1.9111	2.4920
BFU-E	E10.5AGM	0ug/ml	CFU-GEMM	AoE+PSCs	0ug/ml	0.6644	0.09286	36	7.15	<.0001	0.05	0.4760	0.8527
BFU-E	E10.5AGM	0ug/ml	CFU-GEMM	AoE+PSCs	1ug/ml	1.0239	0.1002	36	10.22	<.0001	0.05	0.8207	1.2270
BFU-E	E10.5AGM	0ug/ml	CFU-GEMM	AoE+PSCs	5ug/ml	1.8249	0.1256	36	14.53	<.0001	0.05	1.5701	2.0796
BFU-E	E10.5AGM	0ug/ml	CFU-GEMM	E10.5AGM	0ug/ml	0.1081	0.05961	36	1.81	0.0780	0.05	-0.01274	0.2290
BFU-E	E10.5AGM	0ug/ml	CFU-GEMM	E10.5AGM	1ug/ml	0.4092	0.08879	36	4.61	<.0001	0.05	0.2292	0.5893
BFU-E	E10.5AGM	0ug/ml	CFU-GEMM	E10.5AGM	5ug/ml	1.2783	0.1067	36	11.98	<.0001	0.05	1.0620	1.4947
BFU-E	E10.5AGM	0ug/ml	CFU-GEMM	HE+PSCs	0ug/ml	0.8154	0.09564	36	8.53	<.0001	0.05	0.6214	1.0094
BFU-E	E10.5AGM	0ug/ml	CFU-GEMM	HE+PSCs	1ug/ml	1.5795	0.1162	36	13.60	<.0001	0.05	1.3439	1.8151
BFU-E	E10.5AGM	0ug/ml	CFU-GEMM	HE+PSCs	5ug/ml	1.9254	0.1298	36	14.83	<.0001	0.05	1.6620	2.1887
BFU-E	E10.5AGM	0ug/ml	CFU-GM	AoE+PSCs	0ug/ml	-0.9049	0.07769	36	-11.65	<.0001	0.05	-1.0625	-0.7474
BFU-E	E10.5AGM	0ug/ml	CFU-GM	AoE+PSCs	1ug/ml	-0.06065	0.08330	36	-0.73	0.4713	0.05	-0.2296	0.1083
BFU-E	E10.5AGM	0ug/ml	CFU-GM	AoE+PSCs	5ug/ml	0.6558	0.09270	36	7.07	<.0001	0.05	0.4678	0.8438
BFU-E	E10.5AGM	0ug/ml	CFU-GM	E10.5AGM	0ug/ml	-1.3498	0.04600	36	-29.34	<.0001	0.05	-1.4431	-1.2565
BFU-E	E10.5AGM	0ug/ml	CFU-GM	E10.5AGM	1ug/ml	-0.7020	0.07867	36	-8.92	<.0001	0.05	-0.8615	-0.5424
BFU-E	E10.5AGM	0ug/ml	CFU-GM	E10.5AGM	5ug/ml	-0.05076	0.08339	36	-0.61	0.5465	0.05	-0.2199	0.1184
BFU-E	E10.5AGM	0ug/ml	CFU-GM	HE+PSCs	0ug/ml	-0.8174	0.07810	36	-10.47	<.0001	0.05	-0.9758	-0.6590

The GLIMMIX Procedure

Differences of Colony*Condit*Treatm Least Squares Means													
Colony_Type	Condition	Treatment	_Colony_Type	_Condition	_Treatment	Estimate	Standard Error	DF	t Value	Pr > |t|	Alpha	Lower	Upper
BFU-E	E10.5AGM	0ug/ml	CFU-GM	HE+PSCs	1ug/ml	0.04937	0.08439	36	0.58	0.5622	0.05	-0.1218	0.2205
BFU-E	E10.5AGM	0ug/ml	CFU-GM	HE+PSCs	5ug/ml	0.8151	0.09566	36	8.52	<.0001	0.05	0.6210	1.0091
BFU-E	E10.5AGM	1ug/ml	BFU-E	E10.5AGM	5ug/ml	1.0791	0.1148	36	9.40	<.0001	0.05	0.8463	1.3120
BFU-E	E10.5AGM	1ug/ml	BFU-E	HE+PSCs	0ug/ml	0.3660	0.09779	36	3.74	0.0006	0.05	0.1676	0.5643
BFU-E	E10.5AGM	1ug/ml	BFU-E	HE+PSCs	1ug/ml	1.2586	0.1207	36	10.43	<.0001	0.05	1.0138	1.5034
BFU-E	E10.5AGM	1ug/ml	BFU-E	HE+PSCs	5ug/ml	1.8391	0.1458	36	12.62	<.0001	0.05	1.5435	2.1347
BFU-E	E10.5AGM	1ug/ml	CFU-GEMM	AoE+PSCs	0ug/ml	0.3019	0.09672	36	3.12	0.0035	0.05	0.1058	0.4981
BFU-E	E10.5AGM	1ug/ml	CFU-GEMM	AoE+PSCs	1ug/ml	0.6614	0.1037	36	6.38	<.0001	0.05	0.4510	0.8718
BFU-E	E10.5AGM	1ug/ml	CFU-GEMM	AoE+PSCs	5ug/ml	1.4624	0.1285	36	11.38	<.0001	0.05	1.2019	1.7230
BFU-E	E10.5AGM	1ug/ml	CFU-GEMM	E10.5AGM	0ug/ml	-0.2543	0.08922	36	-2.85	0.0072	0.05	-0.4352	-0.07334
BFU-E	E10.5AGM	1ug/ml	CFU-GEMM	E10.5AGM	1ug/ml	0.04681	0.07025	36	0.67	0.5095	0.05	-0.09567	0.1893
BFU-E	E10.5AGM	1ug/ml	CFU-GEMM	E10.5AGM	5ug/ml	0.9159	0.1101	36	8.32	<.0001	0.05	0.6927	1.1391
BFU-E	E10.5AGM	1ug/ml	CFU-GEMM	HE+PSCs	0ug/ml	0.4530	0.09939	36	4.56	<.0001	0.05	0.2514	0.6545
BFU-E	E10.5AGM	1ug/ml	CFU-GEMM	HE+PSCs	1ug/ml	1.2171	0.1193	36	10.20	<.0001	0.05	0.9752	1.4589
BFU-E	E10.5AGM	1ug/ml	CFU-GEMM	HE+PSCs	5ug/ml	1.5629	0.1326	36	11.79	<.0001	0.05	1.2940	1.8319
BFU-E	E10.5AGM	1ug/ml	CFU-GM	AoE+PSCs	0ug/ml	-1.2674	0.08226	36	-15.41	<.0001	0.05	-1.4342	-1.1005
BFU-E	E10.5AGM	1ug/ml	CFU-GM	AoE+PSCs	1ug/ml	-0.4231	0.08757	36	-4.83	<.0001	0.05	-0.6007	-0.2455
BFU-E	E10.5AGM	1ug/ml	CFU-GM	AoE+PSCs	5ug/ml	0.2934	0.09657	36	3.04	0.0044	0.05	0.09755	0.4892
BFU-E	E10.5AGM	1ug/ml	CFU-GM	E10.5AGM	0ug/ml	-1.7123	0.08077	36	-21.20	<.0001	0.05	-1.8761	-1.5485
BFU-E	E10.5AGM	1ug/ml	CFU-GM	E10.5AGM	1ug/ml	-1.0644	0.05693	36	-18.70	<.0001	0.05	-1.1799	-0.9489
BFU-E	E10.5AGM	1ug/ml	CFU-GM	E10.5AGM	5ug/ml	-0.4132	0.08766	36	-4.71	<.0001	0.05	-0.5910	-0.2354
BFU-E	E10.5AGM	1ug/ml	CFU-GM	HE+PSCs	0ug/ml	-1.1798	0.08264	36	-14.28	<.0001	0.05	-1.3474	-1.0122
BFU-E	E10.5AGM	1ug/ml	CFU-GM	HE+PSCs	1ug/ml	-0.3131	0.08862	36	-3.53	0.0011	0.05	-0.4928	-0.1333
BFU-E	E10.5AGM	1ug/ml	CFU-GM	HE+PSCs	5ug/ml	0.4526	0.09941	36	4.55	<.0001	0.05	0.2510	0.6542

The GLIMMIX Procedure

Differences of Colony*Condit*Treatm Least Squares Means

Colony_Type	Condition	Treatment	_Colony_Type	_Condition	_Treatment	Estimate	Standard Error	DF	t Value	Pr > |t|	Alpha	Lower	Upper
BFU-E	E10.5AGM	5ug/ml	BFU-E	HE+PSCs	0ug/ml	-0.7132	0.1194	36	-5.98	<.0001	0.05	-0.9552	-0.4711
BFU-E	E10.5AGM	5ug/ml	BFU-E	HE+PSCs	1ug/ml	0.1794	0.1388	36	1.29	0.2042	0.05	-0.1020	0.4608
BFU-E	E10.5AGM	5ug/ml	BFU-E	HE+PSCs	5ug/ml	0.7600	0.1610	36	4.72	<.0001	0.05	0.4334	1.0866
BFU-E	E10.5AGM	5ug/ml	CFU-GEMM	AoE+PSCs	0ug/ml	-0.7772	0.1185	36	-6.56	<.0001	0.05	-1.0175	-0.5369
BFU-E	E10.5AGM	5ug/ml	CFU-GEMM	AoE+PSCs	1ug/ml	-0.4177	0.1243	36	-3.36	0.0019	0.05	-0.6698	-0.1657
BFU-E	E10.5AGM	5ug/ml	CFU-GEMM	AoE+PSCs	5ug/ml	0.3833	0.1456	36	2.63	0.0124	0.05	0.08807	0.6785
BFU-E	E10.5AGM	5ug/ml	CFU-GEMM	E10.5AGM	0ug/ml	-1.3334	0.1124	36	-11.86	<.0001	0.05	-1.5615	-1.1054
BFU-E	E10.5AGM	5ug/ml	CFU-GEMM	E10.5AGM	1ug/ml	-1.0323	0.1153	36	-8.95	<.0001	0.05	-1.2662	-0.7984
BFU-E	E10.5AGM	5ug/ml	CFU-GEMM	E10.5AGM	5ug/ml	-0.1632	0.1145	36	-1.43	0.1627	0.05	-0.3955	0.06906
BFU-E	E10.5AGM	5ug/ml	CFU-GEMM	HE+PSCs	0ug/ml	-0.6261	0.1207	36	-5.19	<.0001	0.05	-0.8709	-0.3814
BFU-E	E10.5AGM	5ug/ml	CFU-GEMM	HE+PSCs	1ug/ml	0.1379	0.1375	36	1.00	0.3225	0.05	-0.1409	0.4168
BFU-E	E10.5AGM	5ug/ml	CFU-GEMM	HE+PSCs	5ug/ml	0.4838	0.1492	36	3.24	0.0026	0.05	0.1811	0.7865
BFU-E	E10.5AGM	5ug/ml	CFU-GM	AoE+PSCs	0ug/ml	-2.3465	0.1070	36	-21.93	<.0001	0.05	-2.5635	-2.1295
BFU-E	E10.5AGM	5ug/ml	CFU-GM	AoE+PSCs	1ug/ml	-1.5022	0.1111	36	-13.52	<.0001	0.05	-1.7276	-1.2768
BFU-E	E10.5AGM	5ug/ml	CFU-GM	AoE+PSCs	5ug/ml	-0.7857	0.1184	36	-6.64	<.0001	0.05	-1.0258	-0.5457
BFU-E	E10.5AGM	5ug/ml	CFU-GM	E10.5AGM	0ug/ml	-2.7914	0.1059	36	-26.37	<.0001	0.05	-3.0061	-2.5767
BFU-E	E10.5AGM	5ug/ml	CFU-GM	E10.5AGM	1ug/ml	-2.1435	0.1077	36	-19.90	<.0001	0.05	-2.3620	-1.9251
BFU-E	E10.5AGM	5ug/ml	CFU-GM	E10.5AGM	5ug/ml	-1.4923	0.09321	36	-16.01	<.0001	0.05	-1.6814	-1.3033
BFU-E	E10.5AGM	5ug/ml	CFU-GM	HE+PSCs	0ug/ml	-2.2590	0.1073	36	-21.05	<.0001	0.05	-2.4766	-2.0414
BFU-E	E10.5AGM	5ug/ml	CFU-GM	HE+PSCs	1ug/ml	-1.3922	0.1120	36	-12.43	<.0001	0.05	-1.6193	-1.1651
BFU-E	E10.5AGM	5ug/ml	CFU-GM	HE+PSCs	5ug/ml	-0.6265	0.1207	36	-5.19	<.0001	0.05	-0.8713	-0.3817
BFU-E	HE+PSCs	0ug/ml	BFU-E	HE+PSCs	1ug/ml	0.8926	0.1250	36	7.14	<.0001	0.05	0.6390	1.1462
BFU-E	HE+PSCs	0ug/ml	BFU-E	HE+PSCs	5ug/ml	1.4732	0.1494	36	9.86	<.0001	0.05	1.1703	1.7761
BFU-E	HE+PSCs	0ug/ml	CFU-GEMM	AoE+PSCs	0ug/ml	-0.06404	0.1021	36	-0.63	0.5343	0.05	-0.2710	0.1430

The GLIMMIX Procedure

Differences of Colony*Condit*Treatm Least Squares Means													
Colony_Type	Condition	Treatment	_Colony_Type	_Condition	_Treatment	Estimate	Standard Error	DF	t Value	Pr > |t|	Alpha	Lower	Upper
BFU-E	HE+PSCs	0ug/ml	CFU-GEMM	AoE+PSCs	1ug/ml	0.2955	0.1087	36	2.72	0.0101	0.05	0.07492	0.5160
BFU-E	HE+PSCs	0ug/ml	CFU-GEMM	AoE+PSCs	5ug/ml	1.0965	0.1326	36	8.27	<.0001	0.05	0.8276	1.3653
BFU-E	HE+PSCs	0ug/ml	CFU-GEMM	E10.5AGM	0ug/ml	-0.6202	0.09499	36	-6.53	<.0001	0.05	-0.8129	-0.4276
BFU-E	HE+PSCs	0ug/ml	CFU-GEMM	E10.5AGM	1ug/ml	-0.3192	0.09838	36	-3.24	0.0025	0.05	-0.5187	-0.1196
BFU-E	HE+PSCs	0ug/ml	CFU-GEMM	E10.5AGM	5ug/ml	0.5499	0.1148	36	4.79	<.0001	0.05	0.3171	0.7828
BFU-E	HE+PSCs	0ug/ml	CFU-GEMM	HE+PSCs	0ug/ml	0.08702	0.08521	36	1.02	0.3140	0.05	-0.08579	0.2598
BFU-E	HE+PSCs	0ug/ml	CFU-GEMM	HE+PSCs	1ug/ml	0.8511	0.1236	36	6.88	<.0001	0.05	0.6003	1.1019
BFU-E	HE+PSCs	0ug/ml	CFU-GEMM	HE+PSCs	5ug/ml	1.1970	0.1366	36	8.76	<.0001	0.05	0.9200	1.4739
BFU-E	HE+PSCs	0ug/ml	CFU-GM	AoE+PSCs	0ug/ml	-1.6333	0.08849	36	-18.46	<.0001	0.05	-1.8128	-1.4539
BFU-E	HE+PSCs	0ug/ml	CFU-GM	AoE+PSCs	1ug/ml	-0.7890	0.09345	36	-8.44	<.0001	0.05	-0.9786	-0.5995
BFU-E	HE+PSCs	0ug/ml	CFU-GM	AoE+PSCs	5ug/ml	-0.07256	0.1019	36	-0.71	0.4811	0.05	-0.2793	0.1342
BFU-E	HE+PSCs	0ug/ml	CFU-GM	E10.5AGM	0ug/ml	-2.0782	0.08710	36	-23.86	<.0001	0.05	-2.2549	-1.9016
BFU-E	HE+PSCs	0ug/ml	CFU-GM	E10.5AGM	1ug/ml	-1.4304	0.08935	36	-16.01	<.0001	0.05	-1.6116	-1.2491
BFU-E	HE+PSCs	0ug/ml	CFU-GM	E10.5AGM	5ug/ml	-0.7792	0.09353	36	-8.33	<.0001	0.05	-0.9688	-0.5895
BFU-E	HE+PSCs	0ug/ml	CFU-GM	HE+PSCs	0ug/ml	-1.5458	0.06491	36	-23.82	<.0001	0.05	-1.6774	-1.4142
BFU-E	HE+PSCs	0ug/ml	CFU-GM	HE+PSCs	1ug/ml	-0.6790	0.09442	36	-7.19	<.0001	0.05	-0.8705	-0.4875
BFU-E	HE+PSCs	0ug/ml	CFU-GM	HE+PSCs	5ug/ml	0.08666	0.1046	36	0.83	0.4129	0.05	-0.1255	0.2988
BFU-E	HE+PSCs	1ug/ml	BFU-E	HE+PSCs	5ug/ml	0.5806	0.1653	36	3.51	0.0012	0.05	0.2454	0.9157
BFU-E	HE+PSCs	1ug/ml	CFU-GEMM	AoE+PSCs	0ug/ml	-0.9567	0.1242	36	-7.70	<.0001	0.05	-1.2085	-0.7048
BFU-E	HE+PSCs	1ug/ml	CFU-GEMM	AoE+PSCs	1ug/ml	-0.5971	0.1297	36	-4.60	<.0001	0.05	-0.8603	-0.3340
BFU-E	HE+PSCs	1ug/ml	CFU-GEMM	AoE+PSCs	5ug/ml	0.2039	0.1503	36	1.36	0.1833	0.05	-0.1009	0.5086
BFU-E	HE+PSCs	1ug/ml	CFU-GEMM	E10.5AGM	0ug/ml	-1.5129	0.1185	36	-12.77	<.0001	0.05	-1.7531	-1.2726
BFU-E	HE+PSCs	1ug/ml	CFU-GEMM	E10.5AGM	1ug/ml	-1.2118	0.1212	36	-10.00	<.0001	0.05	-1.4575	-0.9660
BFU-E	HE+PSCs	1ug/ml	CFU-GEMM	E10.5AGM	5ug/ml	-0.3427	0.1349	36	-2.54	0.0155	0.05	-0.6162	-0.06917

The GLIMMIX Procedure

Differences of Colony*Condit*Treatm Least Squares Means													
Colony_Type	Condition	Treatment	_Colony_Type	_Condition	_Treatment	Estimate	Standard Error	DF	t Value	Pr > |t|	Alpha	Lower	Upper
BFU-E	HE+PSCs	1ug/ml	CFU-GEMM	HE+PSCs	0ug/ml	-0.8056	0.1263	36	-6.38	<.0001	0.05	-1.0617	-0.5495
BFU-E	HE+PSCs	1ug/ml	CFU-GEMM	HE+PSCs	1ug/ml	-0.04150	0.1289	36	-0.32	0.7493	0.05	-0.3029	0.2199
BFU-E	HE+PSCs	1ug/ml	CFU-GEMM	HE+PSCs	5ug/ml	0.3044	0.1538	36	1.98	0.0555	0.05	-0.00758	0.6163
BFU-E	HE+PSCs	1ug/ml	CFU-GM	AoE+PSCs	0ug/ml	-2.5259	0.1133	36	-22.29	<.0001	0.05	-2.7557	-2.2961
BFU-E	HE+PSCs	1ug/ml	CFU-GM	AoE+PSCs	1ug/ml	-1.6817	0.1172	36	-14.35	<.0001	0.05	-1.9194	-1.4439
BFU-E	HE+PSCs	1ug/ml	CFU-GM	AoE+PSCs	5ug/ml	-0.9652	0.1241	36	-7.78	<.0001	0.05	-1.2168	-0.7135
BFU-E	HE+PSCs	1ug/ml	CFU-GM	E10.5AGM	0ug/ml	-2.9708	0.1122	36	-26.47	<.0001	0.05	-3.1984	-2.7432
BFU-E	HE+PSCs	1ug/ml	CFU-GM	E10.5AGM	1ug/ml	-2.3230	0.1140	36	-20.38	<.0001	0.05	-2.5541	-2.0918
BFU-E	HE+PSCs	1ug/ml	CFU-GM	E10.5AGM	5ug/ml	-1.6718	0.1173	36	-14.25	<.0001	0.05	-1.9096	-1.4339
BFU-E	HE+PSCs	1ug/ml	CFU-GM	HE+PSCs	0ug/ml	-2.4384	0.1136	36	-21.47	<.0001	0.05	-2.6688	-2.2081
BFU-E	HE+PSCs	1ug/ml	CFU-GM	HE+PSCs	1ug/ml	-1.5716	0.1012	36	-15.53	<.0001	0.05	-1.7768	-1.3664
BFU-E	HE+PSCs	1ug/ml	CFU-GM	HE+PSCs	5ug/ml	-0.8059	0.1263	36	-6.38	<.0001	0.05	-1.0621	-0.5498
BFU-E	HE+PSCs	5ug/ml	CFU-GEMM	AoE+PSCs	0ug/ml	-1.5372	0.1487	36	-10.34	<.0001	0.05	-1.8387	-1.2357
BFU-E	HE+PSCs	5ug/ml	CFU-GEMM	AoE+PSCs	1ug/ml	-1.1777	0.1533	36	-7.68	<.0001	0.05	-1.4887	-0.8668
BFU-E	HE+PSCs	5ug/ml	CFU-GEMM	AoE+PSCs	5ug/ml	-0.3767	0.1710	36	-2.20	0.0341	0.05	-0.7236	-0.02983
BFU-E	HE+PSCs	5ug/ml	CFU-GEMM	E10.5AGM	0ug/ml	-2.0934	0.1439	36	-14.55	<.0001	0.05	-2.3853	-1.8016
BFU-E	HE+PSCs	5ug/ml	CFU-GEMM	E10.5AGM	1ug/ml	-1.7923	0.1461	36	-12.26	<.0001	0.05	-2.0887	-1.4959
BFU-E	HE+PSCs	5ug/ml	CFU-GEMM	E10.5AGM	5ug/ml	-0.9232	0.1577	36	-5.86	<.0001	0.05	-1.2430	-0.6035
BFU-E	HE+PSCs	5ug/ml	CFU-GEMM	HE+PSCs	0ug/ml	-1.3862	0.1504	36	-9.22	<.0001	0.05	-1.6912	-1.0811
BFU-E	HE+PSCs	5ug/ml	CFU-GEMM	HE+PSCs	1ug/ml	-0.6221	0.1642	36	-3.79	0.0006	0.05	-0.9551	-0.2890
BFU-E	HE+PSCs	5ug/ml	CFU-GEMM	HE+PSCs	5ug/ml	-0.2762	0.1632	36	-1.69	0.0992	0.05	-0.6072	0.05481
BFU-E	HE+PSCs	5ug/ml	CFU-GM	AoE+PSCs	0ug/ml	-3.1065	0.1397	36	-22.24	<.0001	0.05	-3.3898	-2.8232
BFU-E	HE+PSCs	5ug/ml	CFU-GM	AoE+PSCs	1ug/ml	-2.2622	0.1429	36	-15.83	<.0001	0.05	-2.5520	-1.9725
BFU-E	HE+PSCs	5ug/ml	CFU-GM	AoE+PSCs	5ug/ml	-1.5457	0.1486	36	-10.40	<.0001	0.05	-1.8470	-1.2444

The GLIMMIX Procedure

Differences of Colony*Condit*Treatm Least Squares Means													
Colony_Type	Condition	Treatment	_Colony_Type	_Condition	_Treatment	Estimate	Standard Error	DF	t Value	Pr > |t|	Alpha	Lower	Upper
BFU-E	HE+PSCs	5ug/ml	CFU-GM	E10.5AGM	0ug/ml	-3.5514	0.1388	36	-25.58	<.0001	0.05	-3.8329	-3.2699
BFU-E	HE+PSCs	5ug/ml	CFU-GM	E10.5AGM	1ug/ml	-2.9035	0.1402	36	-20.71	<.0001	0.05	-3.1879	-2.6191
BFU-E	HE+PSCs	5ug/ml	CFU-GM	E10.5AGM	5ug/ml	-2.2523	0.1429	36	-15.76	<.0001	0.05	-2.5422	-1.9625
BFU-E	HE+PSCs	5ug/ml	CFU-GM	HE+PSCs	0ug/ml	-3.0190	0.1399	36	-21.58	<.0001	0.05	-3.3027	-2.7352
BFU-E	HE+PSCs	5ug/ml	CFU-GM	HE+PSCs	1ug/ml	-2.1522	0.1435	36	-15.00	<.0001	0.05	-2.4433	-1.8611
BFU-E	HE+PSCs	5ug/ml	CFU-GM	HE+PSCs	5ug/ml	-1.3865	0.1376	36	-10.08	<.0001	0.05	-1.6656	-1.1074
CFU-GEMM	AoE+PSCs	0ug/ml	CFU-GEMM	AoE+PSCs	1ug/ml	0.3595	0.1078	36	3.34	0.0020	0.05	0.1409	0.5781
CFU-GEMM	AoE+PSCs	0ug/ml	CFU-GEMM	AoE+PSCs	5ug/ml	1.1605	0.1318	36	8.81	<.0001	0.05	0.8933	1.4278
CFU-GEMM	AoE+PSCs	0ug/ml	CFU-GEMM	E10.5AGM	0ug/ml	-0.5562	0.09389	36	-5.92	<.0001	0.05	-0.7466	-0.3658
CFU-GEMM	AoE+PSCs	0ug/ml	CFU-GEMM	E10.5AGM	1ug/ml	-0.2551	0.09732	36	-2.62	0.0127	0.05	-0.4525	-0.05775
CFU-GEMM	AoE+PSCs	0ug/ml	CFU-GEMM	E10.5AGM	5ug/ml	0.6140	0.1139	36	5.39	<.0001	0.05	0.3830	0.8450
CFU-GEMM	AoE+PSCs	0ug/ml	CFU-GEMM	HE+PSCs	0ug/ml	0.1511	0.1036	36	1.46	0.1535	0.05	-0.05906	0.3612
CFU-GEMM	AoE+PSCs	0ug/ml	CFU-GEMM	HE+PSCs	1ug/ml	0.9151	0.1228	36	7.45	<.0001	0.05	0.6661	1.1642
CFU-GEMM	AoE+PSCs	0ug/ml	CFU-GEMM	HE+PSCs	5ug/ml	1.2610	0.1358	36	9.29	<.0001	0.05	0.9856	1.5364
CFU-GEMM	AoE+PSCs	0ug/ml	CFU-GM	AoE+PSCs	0ug/ml	-1.5693	0.06283	36	-24.98	<.0001	0.05	-1.6967	-1.4419
CFU-GEMM	AoE+PSCs	0ug/ml	CFU-GM	AoE+PSCs	1ug/ml	-0.7250	0.09233	36	-7.85	<.0001	0.05	-0.9123	-0.5377
CFU-GEMM	AoE+PSCs	0ug/ml	CFU-GM	AoE+PSCs	5ug/ml	-0.00852	0.1009	36	-0.08	0.9332	0.05	-0.2132	0.1961
CFU-GEMM	AoE+PSCs	0ug/ml	CFU-GM	E10.5AGM	0ug/ml	-2.0142	0.08590	36	-23.45	<.0001	0.05	-2.1884	-1.8400
CFU-GEMM	AoE+PSCs	0ug/ml	CFU-GM	E10.5AGM	1ug/ml	-1.3663	0.08818	36	-15.49	<.0001	0.05	-1.5452	-1.1875
CFU-GEMM	AoE+PSCs	0ug/ml	CFU-GM	E10.5AGM	5ug/ml	-0.7151	0.09242	36	-7.74	<.0001	0.05	-0.9025	-0.5277
CFU-GEMM	AoE+PSCs	0ug/ml	CFU-GM	HE+PSCs	0ug/ml	-1.4818	0.08767	36	-16.90	<.0001	0.05	-1.6596	-1.3040
CFU-GEMM	AoE+PSCs	0ug/ml	CFU-GM	HE+PSCs	1ug/ml	-0.6150	0.09332	36	-6.59	<.0001	0.05	-0.8043	-0.4257
CFU-GEMM	AoE+PSCs	0ug/ml	CFU-GM	HE+PSCs	5ug/ml	0.1507	0.1036	36	1.45	0.1545	0.05	-0.05946	0.3609
CFU-GEMM	AoE+PSCs	1ug/ml	CFU-GEMM	AoE+PSCs	5ug/ml	0.8010	0.1370	36	5.85	<.0001	0.05	0.5232	1.0789

The GLIMMIX Procedure

Differences of Colony*Condit*Treatm Least Squares Means													
Colony_Type	Condition	Treatment	_Colony_Type	_Condition	_Treatment	Estimate	Standard Error	DF	t Value	Pr > |t|	Alpha	Lower	Upper
CFU-GEMM	AoE+PSCs	1ug/ml	CFU-GEMM	E10.5AGM	0ug/ml	-0.9157	0.1011	36	-9.06	<.0001	0.05	-1.1208	-0.7106
CFU-GEMM	AoE+PSCs	1ug/ml	CFU-GEMM	E10.5AGM	1ug/ml	-0.6146	0.1043	36	-5.89	<.0001	0.05	-0.8261	-0.4031
CFU-GEMM	AoE+PSCs	1ug/ml	CFU-GEMM	E10.5AGM	5ug/ml	0.2545	0.1199	36	2.12	0.0408	0.05	0.01130	0.4977
CFU-GEMM	AoE+PSCs	1ug/ml	CFU-GEMM	HE+PSCs	0ug/ml	-0.2084	0.1102	36	-1.89	0.0666	0.05	-0.4319	0.01503
CFU-GEMM	AoE+PSCs	1ug/ml	CFU-GEMM	HE+PSCs	1ug/ml	0.5556	0.1284	36	4.33	0.0001	0.05	0.2952	0.8161
CFU-GEMM	AoE+PSCs	1ug/ml	CFU-GEMM	HE+PSCs	5ug/ml	0.9015	0.1409	36	6.40	<.0001	0.05	0.6158	1.1872
CFU-GEMM	AoE+PSCs	1ug/ml	CFU-GM	AoE+PSCs	0ug/ml	-1.9288	0.09503	36	-20.30	<.0001	0.05	-2.1215	-1.7361
CFU-GEMM	AoE+PSCs	1ug/ml	CFU-GM	AoE+PSCs	1ug/ml	-1.0845	0.07907	36	-13.72	<.0001	0.05	-1.2449	-0.9241
CFU-GEMM	AoE+PSCs	1ug/ml	CFU-GM	AoE+PSCs	5ug/ml	-0.3680	0.1076	36	-3.42	0.0016	0.05	-0.5863	-0.1497
CFU-GEMM	AoE+PSCs	1ug/ml	CFU-GM	E10.5AGM	0ug/ml	-2.3737	0.09374	36	-25.32	<.0001	0.05	-2.5638	-2.1836
CFU-GEMM	AoE+PSCs	1ug/ml	CFU-GM	E10.5AGM	1ug/ml	-1.7258	0.09583	36	-18.01	<.0001	0.05	-1.9202	-1.5315
CFU-GEMM	AoE+PSCs	1ug/ml	CFU-GM	E10.5AGM	5ug/ml	-1.0746	0.09974	36	-10.77	<.0001	0.05	-1.2769	-0.8723
CFU-GEMM	AoE+PSCs	1ug/ml	CFU-GM	HE+PSCs	0ug/ml	-1.8413	0.09536	36	-19.31	<.0001	0.05	-2.0347	-1.6479
CFU-GEMM	AoE+PSCs	1ug/ml	CFU-GM	HE+PSCs	1ug/ml	-0.9745	0.1006	36	-9.69	<.0001	0.05	-1.1785	-0.7705
CFU-GEMM	AoE+PSCs	1ug/ml	CFU-GM	HE+PSCs	5ug/ml	-0.2088	0.1102	36	-1.89	0.0662	0.05	-0.4323	0.01470
CFU-GEMM	AoE+PSCs	5ug/ml	CFU-GEMM	E10.5AGM	0ug/ml	-1.7167	0.1264	36	-13.59	<.0001	0.05	-1.9730	-1.4604
CFU-GEMM	AoE+PSCs	5ug/ml	CFU-GEMM	E10.5AGM	1ug/ml	-1.4156	0.1289	36	-10.98	<.0001	0.05	-1.6771	-1.1541
CFU-GEMM	AoE+PSCs	5ug/ml	CFU-GEMM	E10.5AGM	5ug/ml	-0.5465	0.1419	36	-3.85	0.0005	0.05	-0.8342	-0.2588
CFU-GEMM	AoE+PSCs	5ug/ml	CFU-GEMM	HE+PSCs	0ug/ml	-1.0095	0.1337	36	-7.55	<.0001	0.05	-1.2807	-0.7382
CFU-GEMM	AoE+PSCs	5ug/ml	CFU-GEMM	HE+PSCs	1ug/ml	-0.2454	0.1491	36	-1.65	0.1086	0.05	-0.5478	0.05704
CFU-GEMM	AoE+PSCs	5ug/ml	CFU-GEMM	HE+PSCs	5ug/ml	0.1005	0.1600	36	0.63	0.5339	0.05	-0.2240	0.4250
CFU-GEMM	AoE+PSCs	5ug/ml	CFU-GM	AoE+PSCs	0ug/ml	-2.7298	0.1216	36	-22.46	<.0001	0.05	-2.9763	-2.4833
CFU-GEMM	AoE+PSCs	5ug/ml	CFU-GM	AoE+PSCs	1ug/ml	-1.8855	0.1252	36	-15.06	<.0001	0.05	-2.1395	-1.6316
CFU-GEMM	AoE+PSCs	5ug/ml	CFU-GM	AoE+PSCs	5ug/ml	-1.1690	0.1169	36	-10.00	<.0001	0.05	-1.4060	-0.9320

The GLIMMIX Procedure

Differences of Colony*Condit*Treatm Least Squares Means													
Colony_Type	Condition	Treatment	_Colony_Type	_Condition	_Treatment	Estimate	Standard Error	DF	t Value	Pr > |t|	Alpha	Lower	Upper
CFU-GEMM	AoE+PSCs	5ug/ml	CFU-GM	E10.5AGM	0ug/ml	-3.1747	0.1205	36	-26.34	<.0001	0.05	-3.4192	-2.9302
CFU-GEMM	AoE+PSCs	5ug/ml	CFU-GM	E10.5AGM	1ug/ml	-2.5268	0.1222	36	-20.68	<.0001	0.05	-2.7746	-2.2790
CFU-GEMM	AoE+PSCs	5ug/ml	CFU-GM	E10.5AGM	5ug/ml	-1.8756	0.1253	36	-14.97	<.0001	0.05	-2.1297	-1.6216
CFU-GEMM	AoE+PSCs	5ug/ml	CFU-GM	HE+PSCs	0ug/ml	-2.6423	0.1218	36	-21.69	<.0001	0.05	-2.8893	-2.3952
CFU-GEMM	AoE+PSCs	5ug/ml	CFU-GM	HE+PSCs	1ug/ml	-1.7755	0.1259	36	-14.10	<.0001	0.05	-2.0309	-1.5201
CFU-GEMM	AoE+PSCs	5ug/ml	CFU-GM	HE+PSCs	5ug/ml	-1.0098	0.1338	36	-7.55	<.0001	0.05	-1.2811	-0.7385
CFU-GEMM	E10.5AGM	0ug/ml	CFU-GEMM	E10.5AGM	1ug/ml	0.3011	0.08986	36	3.35	0.0019	0.05	0.1188	0.4833
CFU-GEMM	E10.5AGM	0ug/ml	CFU-GEMM	E10.5AGM	5ug/ml	1.1702	0.1076	36	10.88	<.0001	0.05	0.9520	1.3884
CFU-GEMM	E10.5AGM	0ug/ml	CFU-GEMM	HE+PSCs	0ug/ml	0.7073	0.09664	36	7.32	<.0001	0.05	0.5113	0.9033
CFU-GEMM	E10.5AGM	0ug/ml	CFU-GEMM	HE+PSCs	1ug/ml	1.4714	0.1170	36	12.58	<.0001	0.05	1.2341	1.7086
CFU-GEMM	E10.5AGM	0ug/ml	CFU-GEMM	HE+PSCs	5ug/ml	1.8172	0.1306	36	13.92	<.0001	0.05	1.5524	2.0820
CFU-GEMM	E10.5AGM	0ug/ml	CFU-GM	AoE+PSCs	0ug/ml	-1.0131	0.07892	36	-12.84	<.0001	0.05	-1.1731	-0.8530
CFU-GEMM	E10.5AGM	0ug/ml	CFU-GM	AoE+PSCs	1ug/ml	-0.1688	0.08444	36	-2.00	0.0532	0.05	-0.3400	0.002458
CFU-GEMM	E10.5AGM	0ug/ml	CFU-GM	AoE+PSCs	5ug/ml	0.5477	0.09373	36	5.84	<.0001	0.05	0.3576	0.7378
CFU-GEMM	E10.5AGM	0ug/ml	CFU-GM	E10.5AGM	0ug/ml	-1.4580	0.04804	36	-30.35	<.0001	0.05	-1.5554	-1.3605
CFU-GEMM	E10.5AGM	0ug/ml	CFU-GM	E10.5AGM	1ug/ml	-0.8101	0.07988	36	-10.14	<.0001	0.05	-0.9721	-0.6481
CFU-GEMM	E10.5AGM	0ug/ml	CFU-GM	E10.5AGM	5ug/ml	-0.1589	0.08453	36	-1.88	0.0682	0.05	-0.3304	0.01253
CFU-GEMM	E10.5AGM	0ug/ml	CFU-GM	HE+PSCs	0ug/ml	-0.9256	0.07931	36	-11.67	<.0001	0.05	-1.0864	-0.7647
CFU-GEMM	E10.5AGM	0ug/ml	CFU-GM	HE+PSCs	1ug/ml	-0.05878	0.08552	36	-0.69	0.4963	0.05	-0.2322	0.1147
CFU-GEMM	E10.5AGM	0ug/ml	CFU-GM	HE+PSCs	5ug/ml	0.7069	0.09666	36	7.31	<.0001	0.05	0.5109	0.9029
CFU-GEMM	E10.5AGM	1ug/ml	CFU-GEMM	E10.5AGM	5ug/ml	0.8691	0.1106	36	7.86	<.0001	0.05	0.6448	1.0934
CFU-GEMM	E10.5AGM	1ug/ml	CFU-GEMM	HE+PSCs	0ug/ml	0.4062	0.09997	36	4.06	0.0003	0.05	0.2034	0.6089
CFU-GEMM	E10.5AGM	1ug/ml	CFU-GEMM	HE+PSCs	1ug/ml	1.1703	0.1197	36	9.77	<.0001	0.05	0.9274	1.4131
CFU-GEMM	E10.5AGM	1ug/ml	CFU-GEMM	HE+PSCs	5ug/ml	1.5161	0.1331	36	11.39	<.0001	0.05	1.2463	1.7860

The GLIMMIX Procedure

Differences of Colony*Condit*Treatm Least Squares Means													
Colony_Type	Condition	Treatment	_Colony_Type	_Condition	_Treatment	Estimate	Standard Error	DF	t Value	Pr > |t|	Alpha	Lower	Upper
CFU-GEMM	E10.5AGM	1ug/ml	CFU-GM	AoE+PSCs	0ug/ml	-1.3142	0.08296	36	-15.84	<.0001	0.05	-1.4824	-1.1459
CFU-GEMM	E10.5AGM	1ug/ml	CFU-GM	AoE+PSCs	1ug/ml	-0.4699	0.08823	36	-5.33	<.0001	0.05	-0.6488	-0.2909
CFU-GEMM	E10.5AGM	1ug/ml	CFU-GM	AoE+PSCs	5ug/ml	0.2466	0.09716	36	2.54	0.0156	0.05	0.04954	0.4436
CFU-GEMM	E10.5AGM	1ug/ml	CFU-GM	E10.5AGM	0ug/ml	-1.7591	0.08148	36	-21.59	<.0001	0.05	-1.9243	-1.5938
CFU-GEMM	E10.5AGM	1ug/ml	CFU-GM	E10.5AGM	1ug/ml	-1.1112	0.05793	36	-19.18	<.0001	0.05	-1.2287	-0.9937
CFU-GEMM	E10.5AGM	1ug/ml	CFU-GM	E10.5AGM	5ug/ml	-0.4600	0.08832	36	-5.21	<.0001	0.05	-0.6391	-0.2809
CFU-GEMM	E10.5AGM	1ug/ml	CFU-GM	HE+PSCs	0ug/ml	-1.2266	0.08334	36	-14.72	<.0001	0.05	-1.3957	-1.0576
CFU-GEMM	E10.5AGM	1ug/ml	CFU-GM	HE+PSCs	1ug/ml	-0.3599	0.08926	36	-4.03	0.0003	0.05	-0.5409	-0.1788
CFU-GEMM	E10.5AGM	1ug/ml	CFU-GM	HE+PSCs	5ug/ml	0.4058	0.09998	36	4.06	0.0003	0.05	0.2030	0.6086
CFU-GEMM	E10.5AGM	5ug/ml	CFU-GEMM	HE+PSCs	0ug/ml	-0.4629	0.1162	36	-3.99	0.0003	0.05	-0.6985	-0.2273
CFU-GEMM	E10.5AGM	5ug/ml	CFU-GEMM	HE+PSCs	1ug/ml	0.3012	0.1336	36	2.25	0.0303	0.05	0.03027	0.5720
CFU-GEMM	E10.5AGM	5ug/ml	CFU-GEMM	HE+PSCs	5ug/ml	0.6470	0.1456	36	4.44	<.0001	0.05	0.3517	0.9423
CFU-GEMM	E10.5AGM	5ug/ml	CFU-GM	AoE+PSCs	0ug/ml	-2.1833	0.1019	36	-21.43	<.0001	0.05	-2.3899	-1.9766
CFU-GEMM	E10.5AGM	5ug/ml	CFU-GM	AoE+PSCs	1ug/ml	-1.3390	0.1062	36	-12.60	<.0001	0.05	-1.5544	-1.1235
CFU-GEMM	E10.5AGM	5ug/ml	CFU-GM	AoE+PSCs	5ug/ml	-0.6225	0.1138	36	-5.47	<.0001	0.05	-0.8532	-0.3918
CFU-GEMM	E10.5AGM	5ug/ml	CFU-GM	E10.5AGM	0ug/ml	-2.6282	0.1007	36	-26.10	<.0001	0.05	-2.8324	-2.4240
CFU-GEMM	E10.5AGM	5ug/ml	CFU-GM	E10.5AGM	1ug/ml	-1.9803	0.1026	36	-19.29	<.0001	0.05	-2.1885	-1.7721
CFU-GEMM	E10.5AGM	5ug/ml	CFU-GM	E10.5AGM	5ug/ml	-1.3291	0.08729	36	-15.23	<.0001	0.05	-1.5061	-1.1521
CFU-GEMM	E10.5AGM	5ug/ml	CFU-GM	HE+PSCs	0ug/ml	-2.0957	0.1022	36	-20.51	<.0001	0.05	-2.3030	-1.8885
CFU-GEMM	E10.5AGM	5ug/ml	CFU-GM	HE+PSCs	1ug/ml	-1.2290	0.1071	36	-11.48	<.0001	0.05	-1.4462	-1.0118
CFU-GEMM	E10.5AGM	5ug/ml	CFU-GM	HE+PSCs	5ug/ml	-0.4633	0.1162	36	-3.99	0.0003	0.05	-0.6989	-0.2277
CFU-GEMM	HE+PSCs	0ug/ml	CFU-GEMM	HE+PSCs	1ug/ml	0.7641	0.1249	36	6.12	<.0001	0.05	0.5107	1.0174
CFU-GEMM	HE+PSCs	0ug/ml	CFU-GEMM	HE+PSCs	5ug/ml	1.1099	0.1377	36	8.06	<.0001	0.05	0.8306	1.3892
CFU-GEMM	HE+PSCs	0ug/ml	CFU-GM	AoE+PSCs	0ug/ml	-1.7204	0.09026	36	-19.06	<.0001	0.05	-1.9034	-1.5373

The GLIMMIX Procedure

Differences of Colony*Condit*Treatm Least Squares Means													
Colony_Type	Condition	Treatment	_Colony_Type	_Condition	_Treatment	Estimate	Standard Error	DF	t Value	Pr > |t|	Alpha	Lower	Upper
CFU-GEMM	HE+PSCs	0ug/ml	CFU-GM	AoE+PSCs	1ug/ml	-0.8761	0.09512	36	-9.21	<.0001	0.05	-1.0690	-0.6831
CFU-GEMM	HE+PSCs	0ug/ml	CFU-GM	AoE+PSCs	5ug/ml	-0.1596	0.1035	36	-1.54	0.1317	0.05	-0.3694	0.05025
CFU-GEMM	HE+PSCs	0ug/ml	CFU-GM	E10.5AGM	0ug/ml	-2.1652	0.08889	36	-24.36	<.0001	0.05	-2.3455	-1.9850
CFU-GEMM	HE+PSCs	0ug/ml	CFU-GM	E10.5AGM	1ug/ml	-1.5174	0.09110	36	-16.66	<.0001	0.05	-1.7021	-1.3326
CFU-GEMM	HE+PSCs	0ug/ml	CFU-GM	E10.5AGM	5ug/ml	-0.8662	0.09520	36	-9.10	<.0001	0.05	-1.0593	-0.6731
CFU-GEMM	HE+PSCs	0ug/ml	CFU-GM	HE+PSCs	0ug/ml	-1.6328	0.06729	36	-24.26	<.0001	0.05	-1.7693	-1.4963
CFU-GEMM	HE+PSCs	0ug/ml	CFU-GM	HE+PSCs	1ug/ml	-0.7660	0.09608	36	-7.97	<.0001	0.05	-0.9609	-0.5712
CFU-GEMM	HE+PSCs	0ug/ml	CFU-GM	HE+PSCs	5ug/ml	-0.00036	0.1061	36	-0.00	0.9973	0.05	-0.2156	0.2149
CFU-GEMM	HE+PSCs	1ug/ml	CFU-GEMM	HE+PSCs	5ug/ml	0.3459	0.1527	36	2.27	0.0296	0.05	0.03620	0.6555
CFU-GEMM	HE+PSCs	1ug/ml	CFU-GM	AoE+PSCs	0ug/ml	-2.4844	0.1118	36	-22.23	<.0001	0.05	-2.7111	-2.2577
CFU-GEMM	HE+PSCs	1ug/ml	CFU-GM	AoE+PSCs	1ug/ml	-1.6401	0.1157	36	-14.17	<.0001	0.05	-1.8749	-1.4054
CFU-GEMM	HE+PSCs	1ug/ml	CFU-GM	AoE+PSCs	5ug/ml	-0.9237	0.1227	36	-7.53	<.0001	0.05	-1.1725	-0.6749
CFU-GEMM	HE+PSCs	1ug/ml	CFU-GM	E10.5AGM	0ug/ml	-2.9293	0.1107	36	-26.47	<.0001	0.05	-3.1538	-2.7049
CFU-GEMM	HE+PSCs	1ug/ml	CFU-GM	E10.5AGM	1ug/ml	-2.2815	0.1125	36	-20.29	<.0001	0.05	-2.5095	-2.0534
CFU-GEMM	HE+PSCs	1ug/ml	CFU-GM	E10.5AGM	5ug/ml	-1.6303	0.1158	36	-14.08	<.0001	0.05	-1.8651	-1.3954
CFU-GEMM	HE+PSCs	1ug/ml	CFU-GM	HE+PSCs	0ug/ml	-2.3969	0.1121	36	-21.39	<.0001	0.05	-2.6242	-2.1697
CFU-GEMM	HE+PSCs	1ug/ml	CFU-GM	HE+PSCs	1ug/ml	-1.5301	0.09947	36	-15.38	<.0001	0.05	-1.7319	-1.3284
CFU-GEMM	HE+PSCs	1ug/ml	CFU-GM	HE+PSCs	5ug/ml	-0.7644	0.1249	36	-6.12	<.0001	0.05	-1.0178	-0.5111
CFU-GEMM	HE+PSCs	5ug/ml	CFU-GM	AoE+PSCs	0ug/ml	-2.8303	0.1259	36	-22.48	<.0001	0.05	-3.0857	-2.5749
CFU-GEMM	HE+PSCs	5ug/ml	CFU-GM	AoE+PSCs	1ug/ml	-1.9860	0.1295	36	-15.34	<.0001	0.05	-2.2485	-1.7235
CFU-GEMM	HE+PSCs	5ug/ml	CFU-GM	AoE+PSCs	5ug/ml	-1.2695	0.1357	36	-9.36	<.0001	0.05	-1.5447	-0.9943
CFU-GEMM	HE+PSCs	5ug/ml	CFU-GM	E10.5AGM	0ug/ml	-3.2752	0.1250	36	-26.21	<.0001	0.05	-3.5286	-3.0218
CFU-GEMM	HE+PSCs	5ug/ml	CFU-GM	E10.5AGM	1ug/ml	-2.6273	0.1265	36	-20.76	<.0001	0.05	-2.8839	-2.3707
CFU-GEMM	HE+PSCs	5ug/ml	CFU-GM	E10.5AGM	5ug/ml	-1.9761	0.1295	36	-15.26	<.0001	0.05	-2.2388	-1.7135

The GLIMMIX Procedure

Differences of Colony*Condit*Treatm Least Squares Means													
Colony_Type	Condition	Treatment	_Colony_Type	_Condition	_Treatment	Estimate	Standard Error	DF	t Value	Pr > |t|	Alpha	Lower	Upper
CFU-GEMM	HE+PSCs	5ug/ml	CFU-GM	HE+PSCs	0ug/ml	-2.7428	0.1262	36	-21.74	<.0001	0.05	-2.9986	-2.4869
CFU-GEMM	HE+PSCs	5ug/ml	CFU-GM	HE+PSCs	1ug/ml	-1.8760	0.1302	36	-14.41	<.0001	0.05	-2.1399	-1.6120
CFU-GEMM	HE+PSCs	5ug/ml	CFU-GM	HE+PSCs	5ug/ml	-1.1103	0.1236	36	-8.98	<.0001	0.05	-1.3610	-0.8596
CFU-GM	AoE+PSCs	0ug/ml	CFU-GM	AoE+PSCs	1ug/ml	0.8443	0.07706	36	10.96	<.0001	0.05	0.6880	1.0006
CFU-GM	AoE+PSCs	0ug/ml	CFU-GM	AoE+PSCs	5ug/ml	1.5608	0.08714	36	17.91	<.0001	0.05	1.3840	1.7375
CFU-GM	AoE+PSCs	0ug/ml	CFU-GM	E10.5AGM	0ug/ml	-0.4449	0.06922	36	-6.43	<.0001	0.05	-0.5853	-0.3045
CFU-GM	AoE+PSCs	0ug/ml	CFU-GM	E10.5AGM	1ug/ml	0.2030	0.07203	36	2.82	0.0078	0.05	0.05689	0.3491
CFU-GM	AoE+PSCs	0ug/ml	CFU-GM	E10.5AGM	5ug/ml	0.8542	0.07716	36	11.07	<.0001	0.05	0.6977	1.0107
CFU-GM	AoE+PSCs	0ug/ml	CFU-GM	HE+PSCs	0ug/ml	0.08753	0.07140	36	1.23	0.2282	0.05	-0.05728	0.2323
CFU-GM	AoE+PSCs	0ug/ml	CFU-GM	HE+PSCs	1ug/ml	0.9543	0.07824	36	12.20	<.0001	0.05	0.7956	1.1130
CFU-GM	AoE+PSCs	0ug/ml	CFU-GM	HE+PSCs	5ug/ml	1.7200	0.09028	36	19.05	<.0001	0.05	1.5369	1.9031
CFU-GM	AoE+PSCs	1ug/ml	CFU-GM	AoE+PSCs	5ug/ml	0.7165	0.09217	36	7.77	<.0001	0.05	0.5296	0.9034
CFU-GM	AoE+PSCs	1ug/ml	CFU-GM	E10.5AGM	0ug/ml	-1.2892	0.07546	36	-17.09	<.0001	0.05	-1.4422	-1.1362
CFU-GM	AoE+PSCs	1ug/ml	CFU-GM	E10.5AGM	1ug/ml	-0.6413	0.07804	36	-8.22	<.0001	0.05	-0.7996	-0.4830
CFU-GM	AoE+PSCs	1ug/ml	CFU-GM	E10.5AGM	5ug/ml	0.009888	0.08280	36	0.12	0.9056	0.05	-0.1580	0.1778
CFU-GM	AoE+PSCs	1ug/ml	CFU-GM	HE+PSCs	0ug/ml	-0.7568	0.07746	36	-9.77	<.0001	0.05	-0.9139	-0.5997
CFU-GM	AoE+PSCs	1ug/ml	CFU-GM	HE+PSCs	1ug/ml	0.1100	0.08380	36	1.31	0.1975	0.05	-0.05994	0.2800
CFU-GM	AoE+PSCs	1ug/ml	CFU-GM	HE+PSCs	5ug/ml	0.8757	0.09514	36	9.20	<.0001	0.05	0.6828	1.0687
CFU-GM	AoE+PSCs	5ug/ml	CFU-GM	E10.5AGM	0ug/ml	-2.0057	0.08573	36	-23.40	<.0001	0.05	-2.1795	-1.8318
CFU-GM	AoE+PSCs	5ug/ml	CFU-GM	E10.5AGM	1ug/ml	-1.3578	0.08801	36	-15.43	<.0001	0.05	-1.5363	-1.1793
CFU-GM	AoE+PSCs	5ug/ml	CFU-GM	E10.5AGM	5ug/ml	-0.7066	0.09225	36	-7.66	<.0001	0.05	-0.8937	-0.5195
CFU-GM	AoE+PSCs	5ug/ml	CFU-GM	HE+PSCs	0ug/ml	-1.4732	0.08750	36	-16.84	<.0001	0.05	-1.6507	-1.2958
CFU-GM	AoE+PSCs	5ug/ml	CFU-GM	HE+PSCs	1ug/ml	-0.6065	0.09316	36	-6.51	<.0001	0.05	-0.7954	-0.4175
CFU-GM	AoE+PSCs	5ug/ml	CFU-GM	HE+PSCs	5ug/ml	0.1592	0.1035	36	1.54	0.1326	0.05	-0.05064	0.3691

The GLIMMIX Procedure

Differences of Colony*Condit*Treatm Least Squares Means													
Colony_Type	Condition	Treatment	_Colony_Type	_Condition	_Treatment	Estimate	Standard Error	DF	t Value	Pr > |t|	Alpha	Lower	Upper
CFU-GM	E10.5AGM	0ug/ml	CFU-GM	E10.5AGM	1ug/ml	0.6479	0.07032	36	9.21	<.0001	0.05	0.5053	0.7905
CFU-GM	E10.5AGM	0ug/ml	CFU-GM	E10.5AGM	5ug/ml	1.2991	0.07556	36	17.19	<.0001	0.05	1.1458	1.4523
CFU-GM	E10.5AGM	0ug/ml	CFU-GM	HE+PSCs	0ug/ml	0.5324	0.06967	36	7.64	<.0001	0.05	0.3911	0.6737
CFU-GM	E10.5AGM	0ug/ml	CFU-GM	HE+PSCs	1ug/ml	1.3992	0.07666	36	18.25	<.0001	0.05	1.2437	1.5547
CFU-GM	E10.5AGM	0ug/ml	CFU-GM	HE+PSCs	5ug/ml	2.1649	0.08892	36	24.35	<.0001	0.05	1.9846	2.3452
CFU-GM	E10.5AGM	1ug/ml	CFU-GM	E10.5AGM	5ug/ml	0.6512	0.07814	36	8.33	<.0001	0.05	0.4927	0.8097
CFU-GM	E10.5AGM	1ug/ml	CFU-GM	HE+PSCs	0ug/ml	-0.1154	0.07247	36	-1.59	0.1199	0.05	-0.2624	0.03152
CFU-GM	E10.5AGM	1ug/ml	CFU-GM	HE+PSCs	1ug/ml	0.7513	0.07921	36	9.49	<.0001	0.05	0.5907	0.9120
CFU-GM	E10.5AGM	1ug/ml	CFU-GM	HE+PSCs	5ug/ml	1.5170	0.09112	36	16.65	<.0001	0.05	1.3322	1.7018
CFU-GM	E10.5AGM	5ug/ml	CFU-GM	HE+PSCs	0ug/ml	-0.7666	0.07756	36	-9.88	<.0001	0.05	-0.9239	-0.6093
CFU-GM	E10.5AGM	5ug/ml	CFU-GM	HE+PSCs	1ug/ml	0.1001	0.08389	36	1.19	0.2405	0.05	-0.07002	0.2703
CFU-GM	E10.5AGM	5ug/ml	CFU-GM	HE+PSCs	5ug/ml	0.8658	0.09522	36	9.09	<.0001	0.05	0.6727	1.0589
CFU-GM	HE+PSCs	0ug/ml	CFU-GM	HE+PSCs	1ug/ml	0.8668	0.07863	36	11.02	<.0001	0.05	0.7073	1.0263
CFU-GM	HE+PSCs	0ug/ml	CFU-GM	HE+PSCs	5ug/ml	1.6325	0.09062	36	18.01	<.0001	0.05	1.4487	1.8163
CFU-GM	HE+PSCs	1ug/ml	CFU-GM	HE+PSCs	5ug/ml	0.7657	0.09609	36	7.97	<.0001	0.05	0.5708	0.9606

The GENMOD Procedure

Model Information	
Data Set	WORK.EXT_FIG10BIII_E10USAG_TRANS
Distribution	Normal
Link Function	Identity
Dependent Variable	Percentage

Number of Observations Read	46
Number of Observations Used	45
Missing Values	1

Class Level Information		
Class	Levels	Values
Condition	3	AoE+PSCs E10.5AGM HE+PSCs
Treatment	3	0ug/ml 1ug/ml 5ug/ml

Parameter Information			
Parameter	Effect	Condition	Treatment
Prm1	Intercept		
Prm2	Condition	AoE+PSCs	
Prm3	Condition	E10.5AGM	
Prm4	Condition	HE+PSCs	
Prm5	Treatment		0ug/ml
Prm6	Treatment		1ug/ml
Prm7	Treatment		5ug/ml
Prm8	Condition*Treatment	AoE+PSCs	0ug/ml
Prm9	Condition*Treatment	AoE+PSCs	1ug/ml
Prm10	Condition*Treatment	AoE+PSCs	5ug/ml

The GENMOD Procedure

Parameter Information			
**Parameter**	**Effect**	**Condition**	**Treatment**
**Prm11**	Condition*Treatment	E10.5AGM	0ug/ml
**Prm12**	Condition*Treatment	E10.5AGM	1ug/ml
**Prm13**	Condition*Treatment	E10.5AGM	5ug/ml
**Prm14**	Condition*Treatment	HE+PSCs	0ug/ml
**Prm15**	Condition*Treatment	HE+PSCs	1ug/ml
**Prm16**	Condition*Treatment	HE+PSCs	5ug/ml

Criteria For Assessing Goodness Of Fit			
**Criterion**	**DF**	**Value**	**Value/DF**
**Deviance**	36	695.6000	19.3222
**Scaled Deviance**	36	45.0000	1.2500
**Pearson Chi-Square**	36	695.6000	19.3222
**Scaled Pearson X2**	36	45.0000	1.2500
**Log Likelihood**		-125.4598	
**Full Log Likelihood**		-125.4598	
**AIC (smaller is better)**		270.9195	
**AICC (smaller is better)**		277.3901	
**BIC (smaller is better)**		288.9861	

Algorithm converged.

The GENMOD Procedure

Analysis Of Maximum Likelihood Parameter Estimates									
Parameter			DF	Estimate	Standard Error	Wald 95% Confidence Limits		Wald Chi-Square	Pr > ChiSq
Intercept			1	10.0000	1.7583	6.5538	13.4462	32.35	<.0001
Condition	AoE+PSCs		1	1.4000	2.4866	-3.4736	6.2736	0.32	0.5734
Condition	E10.5AGM		1	13.8000	2.4866	8.9264	18.6736	30.80	<.0001
Condition	HE+PSCs		0	0.0000	0.0000	0.0000	0.0000	.	.
Treatment	0ug/ml		1	16.2000	2.4866	11.3264	21.0736	42.44	<.0001
Treatment	1ug/ml		1	6.6000	2.4866	1.7264	11.4736	7.04	0.0079
Treatment	5ug/ml		0	0.0000	0.0000	0.0000	0.0000	.	.
Condition*Treatment	AoE+PSCs	0ug/ml	1	4.2000	3.5166	-2.6923	11.0923	1.43	0.2323
Condition*Treatment	AoE+PSCs	1ug/ml	1	2.2000	3.5166	-4.6923	9.0923	0.39	0.5316
Condition*Treatment	AoE+PSCs	5ug/ml	0	0.0000	0.0000	0.0000	0.0000	.	.
Condition*Treatment	E10.5AGM	0ug/ml	1	16.8000	3.5166	9.9077	23.6923	22.82	<.0001
Condition*Treatment	E10.5AGM	1ug/ml	1	10.2000	3.5166	3.3077	17.0923	8.41	0.0037
Condition*Treatment	E10.5AGM	5ug/ml	0	0.0000	0.0000	0.0000	0.0000	.	.
Condition*Treatment	HE+PSCs	0ug/ml	0	0.0000	0.0000	0.0000	0.0000	.	.
Condition*Treatment	HE+PSCs	1ug/ml	0	0.0000	0.0000	0.0000	0.0000	.	.
Condition*Treatment	HE+PSCs	5ug/ml	0	0.0000	0.0000	0.0000	0.0000	.	.
Scale			1	3.9316	0.4144	3.1978	4.8339		

Note: The scale parameter was estimated by maximum likelihood.

The GENMOD Procedure

Condition*Treatment Least Squares Means								
Condition	Treatment	Estimate	Standard Error	z Value	Pr > |z|	Alpha	Lower	Upper
AoE+PSCs	0ug/ml	31.8000	1.7583	18.09	<.0001	0.05	28.3538	35.2462
AoE+PSCs	1ug/ml	20.2000	1.7583	11.49	<.0001	0.05	16.7538	23.6462
AoE+PSCs	5ug/ml	11.4000	1.7583	6.48	<.0001	0.05	7.9538	14.8462
E10.5AGM	0ug/ml	56.8000	1.7583	32.30	<.0001	0.05	53.3538	60.2462
E10.5AGM	1ug/ml	40.6000	1.7583	23.09	<.0001	0.05	37.1538	44.0462
E10.5AGM	5ug/ml	23.8000	1.7583	13.54	<.0001	0.05	20.3538	27.2462
HE+PSCs	0ug/ml	26.2000	1.7583	14.90	<.0001	0.05	22.7538	29.6462
HE+PSCs	1ug/ml	16.6000	1.7583	9.44	<.0001	0.05	13.1538	20.0462
HE+PSCs	5ug/ml	10.0000	1.7583	5.69	<.0001	0.05	6.5538	13.4462

Differences of Condition*Treatment Least Squares Means										
Condition	Treatment	_Condition	_Treatment	Estimate	Standard Error	z Value	Pr > |z|	Alpha	Lower	Upper
AoE+PSCs	0ug/ml	AoE+PSCs	1ug/ml	11.6000	2.4866	4.67	<.0001	0.05	6.7264	16.4736
AoE+PSCs	0ug/ml	AoE+PSCs	5ug/ml	20.4000	2.4866	8.20	<.0001	0.05	15.5264	25.2736
AoE+PSCs	0ug/ml	E10.5AGM	0ug/ml	-25.0000	2.4866	-10.05	<.0001	0.05	-29.8736	-20.1264
AoE+PSCs	0ug/ml	E10.5AGM	1ug/ml	-8.8000	2.4866	-3.54	0.0004	0.05	-13.6736	-3.9264
AoE+PSCs	0ug/ml	E10.5AGM	5ug/ml	8.0000	2.4866	3.22	0.0013	0.05	3.1264	12.8736
AoE+PSCs	0ug/ml	HE+PSCs	0ug/ml	5.6000	2.4866	2.25	0.0243	0.05	0.7264	10.4736
AoE+PSCs	0ug/ml	HE+PSCs	1ug/ml	15.2000	2.4866	6.11	<.0001	0.05	10.3264	20.0736
AoE+PSCs	0ug/ml	HE+PSCs	5ug/ml	21.8000	2.4866	8.77	<.0001	0.05	16.9264	26.6736
AoE+PSCs	1ug/ml	AoE+PSCs	5ug/ml	8.8000	2.4866	3.54	0.0004	0.05	3.9264	13.6736
AoE+PSCs	1ug/ml	E10.5AGM	0ug/ml	-36.6000	2.4866	-14.72	<.0001	0.05	-41.4736	-31.7264
AoE+PSCs	1ug/ml	E10.5AGM	1ug/ml	-20.4000	2.4866	-8.20	<.0001	0.05	-25.2736	-15.5264
AoE+PSCs	1ug/ml	E10.5AGM	5ug/ml	-3.6000	2.4866	-1.45	0.1477	0.05	-8.4736	1.2736

The GENMOD Procedure

Differences of Condition*Treatment Least Squares Means										
Condition	Treatment	_Condition	_Treatment	Estimate	Standard Error	z Value	Pr > |z|	Alpha	Lower	Upper
AoE+PSCs	1ug/ml	HE+PSCs	0ug/ml	-6.0000	2.4866	-2.41	0.0158	0.05	-10.8736	-1.1264
AoE+PSCs	1ug/ml	HE+PSCs	1ug/ml	3.6000	2.4866	1.45	0.1477	0.05	-1.2736	8.4736
AoE+PSCs	1ug/ml	HE+PSCs	5ug/ml	10.2000	2.4866	4.10	<.0001	0.05	5.3264	15.0736
AoE+PSCs	5ug/ml	E10.5AGM	0ug/ml	-45.4000	2.4866	-18.26	<.0001	0.05	-50.2736	-40.5264
AoE+PSCs	5ug/ml	E10.5AGM	1ug/ml	-29.2000	2.4866	-11.74	<.0001	0.05	-34.0736	-24.3264
AoE+PSCs	5ug/ml	E10.5AGM	5ug/ml	-12.4000	2.4866	-4.99	<.0001	0.05	-17.2736	-7.5264
AoE+PSCs	5ug/ml	HE+PSCs	0ug/ml	-14.8000	2.4866	-5.95	<.0001	0.05	-19.6736	-9.9264
AoE+PSCs	5ug/ml	HE+PSCs	1ug/ml	-5.2000	2.4866	-2.09	0.0365	0.05	-10.0736	-0.3264
AoE+PSCs	5ug/ml	HE+PSCs	5ug/ml	1.4000	2.4866	0.56	0.5734	0.05	-3.4736	6.2736
E10.5AGM	0ug/ml	E10.5AGM	1ug/ml	16.2000	2.4866	6.51	<.0001	0.05	11.3264	21.0736
E10.5AGM	0ug/ml	E10.5AGM	5ug/ml	33.0000	2.4866	13.27	<.0001	0.05	28.1264	37.8736
E10.5AGM	0ug/ml	HE+PSCs	0ug/ml	30.6000	2.4866	12.31	<.0001	0.05	25.7264	35.4736
E10.5AGM	0ug/ml	HE+PSCs	1ug/ml	40.2000	2.4866	16.17	<.0001	0.05	35.3264	45.0736
E10.5AGM	0ug/ml	HE+PSCs	5ug/ml	46.8000	2.4866	18.82	<.0001	0.05	41.9264	51.6736
E10.5AGM	1ug/ml	E10.5AGM	5ug/ml	16.8000	2.4866	6.76	<.0001	0.05	11.9264	21.6736
E10.5AGM	1ug/ml	HE+PSCs	0ug/ml	14.4000	2.4866	5.79	<.0001	0.05	9.5264	19.2736
E10.5AGM	1ug/ml	HE+PSCs	1ug/ml	24.0000	2.4866	9.65	<.0001	0.05	19.1264	28.8736
E10.5AGM	1ug/ml	HE+PSCs	5ug/ml	30.6000	2.4866	12.31	<.0001	0.05	25.7264	35.4736
E10.5AGM	5ug/ml	HE+PSCs	0ug/ml	-2.4000	2.4866	-0.97	0.3345	0.05	-7.2736	2.4736
E10.5AGM	5ug/ml	HE+PSCs	1ug/ml	7.2000	2.4866	2.90	0.0038	0.05	2.3264	12.0736
E10.5AGM	5ug/ml	HE+PSCs	5ug/ml	13.8000	2.4866	5.55	<.0001	0.05	8.9264	18.6736
HE+PSCs	0ug/ml	HE+PSCs	1ug/ml	9.6000	2.4866	3.86	0.0001	0.05	4.7264	14.4736
HE+PSCs	0ug/ml	HE+PSCs	5ug/ml	16.2000	2.4866	6.51	<.0001	0.05	11.3264	21.0736
HE+PSCs	1ug/ml	HE+PSCs	5ug/ml	6.6000	2.4866	2.65	0.0079	0.05	1.7264	11.4736

The GENMOD Procedure

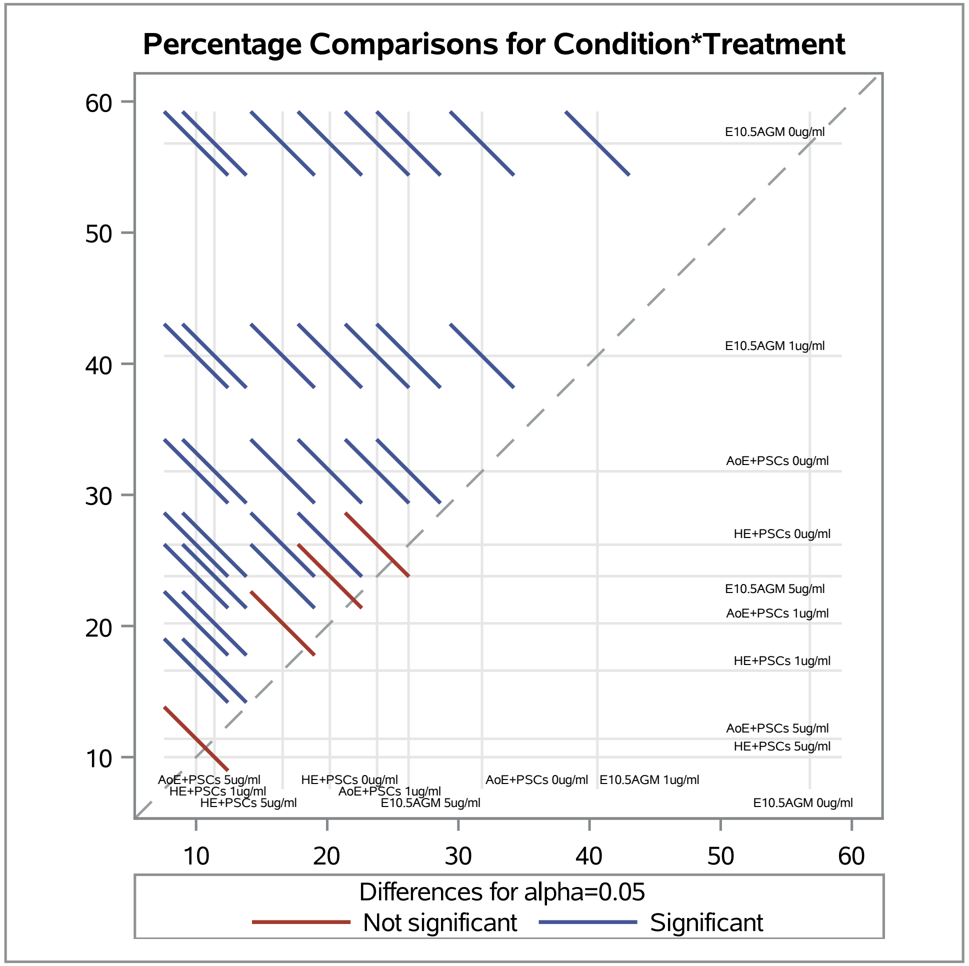

The GLIMMIX Procedure

Model Information	
**Data Set**	WORK.EXT_FIG10BIII_E11USAG
**Response Variable**	Count
**Response Distribution**	Poisson
**Link Function**	Log
**Variance Function**	Default
**Variance Matrix Blocked By**	Dish_ID
**Estimation Technique**	Maximum Likelihood
**Likelihood Approximation**	Laplace
**Degrees of Freedom Method**	Containment

Class Level Information		
**Class**	**Levels**	**Values**
**Colony_Type**	3	BFU-E CFU-GEMM CFU-GM
**Condition**	3	AoE+PSCs E11.5AGM HE+PSCs
**Treatment**	3	0ug/ml 1ug/ml 5ug/ml
**Dish_ID**	27	1 2 3 4 5 6 7 8 9 10 11 12 13 14 15 16 17 18 19 20 21 22 23 24 25 26 27

**Number of Observations Read**	81
**Number of Observations Used**	81

Dimensions	
**G-side Cov. Parameters**	1
**Columns in X**	64
**Columns in Z per Subject**	1
**Subjects (Blocks in V)**	27
**Max Obs per Subject**	3

The GLIMMIX Procedure

Optimization Information	
Optimization Technique	Dual Quasi-Newton
Parameters in Optimization	28
Lower Boundaries	1
Upper Boundaries	0
Fixed Effects	Not Profiled
Starting From	GLM estimates

Iteration History					
Iteration	Restarts	Evaluations	Objective Function	Change	Max Gradient
0	0	4	796.17847729	.	786.1027
1	0	8	795.16673422	1.01174307	65.95679
2	0	4	795.14581227	0.02092195	18.48854
3	0	4	795.11367415	0.03213812	17.86769
4	0	2	795.10837642	0.00529773	2.880824
5	0	2	795.10771196	0.00066446	2.598127
6	0	2	795.10679409	0.00091787	3.291257
7	0	4	795.10253685	0.00425724	4.578882
8	0	3	795.10179363	0.00074322	1.781945
9	0	4	795.09676491	0.00502873	19.12193
10	0	3	795.09510317	0.00166174	1.591201
11	0	2	795.09423598	0.00086719	1.999056
12	0	3	795.09380102	0.00043496	1.514349
13	0	2	795.09313639	0.00066463	1.638302
14	0	2	795.09212909	0.00100731	1.758599
15	0	3	795.09187203	0.00025706	1.64373

The GLIMMIX Procedure

Iteration History					
**Iteration**	**Restarts**	**Evaluations**	**Objective Function**	**Change**	**Max Gradient**
**16**	**0**	4	795.09098419	0.00088784	1.312126
**17**	**0**	3	795.09075695	0.00022724	0.470879
**18**	**0**	3	795.09071797	0.00003898	0.596988
**19**	**0**	6	795.08971687	0.00100110	1.672304
**20**	**0**	3	795.08967657	0.00004030	0.404172
**21**	**0**	6	795.08883753	0.00083904	4.722155
**22**	**0**	3	795.08846522	0.00037231	0.322624
**23**	**0**	2	795.08837444	0.00009077	0.811315
**24**	**0**	3	795.08834111	0.00003333	1.097956
**25**	**0**	4	795.08820003	0.00014108	1.55033
**26**	**0**	3	795.08817193	0.00002810	0.19611
**27**	**0**	4	795.08806925	0.00010269	0.510383

Convergence criterion (GCONV=1E-8) satisfied.

Fit Statistics	
**-2 Log Likelihood**	795.09
**AIC (smaller is better)**	851.09
**AICC (smaller is better)**	882.32
**BIC (smaller is better)**	887.37
**CAIC (smaller is better)**	915.37
**HQIC (smaller is better)**	861.88

The GLIMMIX Procedure

Fit Statistics for Conditional Distribution	
**-2 log L(Count | r. effects)**	728.40
**Pearson Chi-Square**	199.67
**Pearson Chi-Square / DF**	2.47

Covariance Parameter Estimates			
**Cov Parm**	**Subject**	**Estimate**	**Standard Error**
**Intercept**	Dish_ID	0.01015	0.003737

The GLIMMIX Procedure

Solutions for Fixed Effects								
Effect	Colony_Type	Condition	Treatment	Estimate	Standard Error	DF	t Value	Pr > |t|
Intercept				4.5047	0.08403	18	53.61	<.0001
Colony_Type	BFU-E			-1.2397	0.1278	36	-9.70	<.0001
Colony_Type	CFU-GEMM			-1.4662	0.1398	36	-10.49	<.0001
Colony_Type	CFU-GM			0	.	.	.	.
Condition		AoE+PSCs		0.2570	0.1153	36	2.23	0.0322
Condition		E11.5AGM		0.7803	0.1101	36	7.08	<.0001
Condition		HE+PSCs		0	.	.	.	.
Colony_Typ*Condition	BFU-E	AoE+PSCs		-0.6437	0.1941	36	-3.32	0.0021
Colony_Typ*Condition	BFU-E	E11.5AGM		0.4085	0.1479	36	2.76	0.0090
Colony_Typ*Condition	BFU-E	HE+PSCs		0	.	.	.	.
Colony_Typ*Condition	CFU-GEMM	AoE+PSCs		0.4838	0.1729	36	2.80	0.0082
Colony_Typ*Condition	CFU-GEMM	E11.5AGM		0.2877	0.1634	36	1.76	0.0867
Colony_Typ*Condition	CFU-GEMM	HE+PSCs		0	.	.	.	.
Colony_Typ*Condition	CFU-GM	AoE+PSCs		0	.	.	.	.
Colony_Typ*Condition	CFU-GM	E11.5AGM		0	.	.	.	.
Colony_Typ*Condition	CFU-GM	HE+PSCs		0	.	.	.	.
Treatment			0ug/ml	1.5522	0.1060	36	14.65	<.0001
Treatment			1ug/ml	0.8114	0.1099	36	7.38	<.0001
Treatment			5ug/ml	0	.	.	.	.
Colony_Typ*Treatment	BFU-E		0ug/ml	-0.3426	0.1446	36	-2.37	0.0233
Colony_Typ*Treatment	BFU-E		1ug/ml	-0.6056	0.1681	36	-3.60	0.0009
Colony_Typ*Treatment	BFU-E		5ug/ml	0	.	.	.	.
Colony_Typ*Treatment	CFU-GEMM		0ug/ml	-0.03625	0.1543	36	-0.23	0.8157
Colony_Typ*Treatment	CFU-GEMM		1ug/ml	0.07661	0.1665	36	0.46	0.6481

The GLIMMIX Procedure

Solutions for Fixed Effects								
Effect	Colony_Type	Condition	Treatment	Estimate	Standard Error	DF	t Value	Pr > |t|
**Colony_Typ*Treatment**	CFU-GEMM		5ug/ml	0	.	.	.	.
**Colony_Typ*Treatment**	CFU-GM		0ug/ml	0	.	.	.	.
**Colony_Typ*Treatment**	CFU-GM		1ug/ml	0	.	.	.	.
**Colony_Typ*Treatment**	CFU-GM		5ug/ml	0	.	.	.	.
**Condition*Treatment**		AoE+PSCs	0ug/ml	-0.06149	0.1466	36	-0.42	0.6774
**Condition*Treatment**		AoE+PSCs	1ug/ml	-0.1000	0.1520	36	-0.66	0.5146
**Condition*Treatment**		AoE+PSCs	5ug/ml	0	.	.	.	.
**Condition*Treatment**		E11.5AGM	0ug/ml	-0.1686	0.1418	36	-1.19	0.2423
**Condition*Treatment**		E11.5AGM	1ug/ml	-0.05853	0.1461	36	-0.40	0.6910
**Condition*Treatment**		E11.5AGM	5ug/ml	0	.	.	.	.
**Condition*Treatment**		HE+PSCs	0ug/ml	0	.	.	.	.
**Condition*Treatment**		HE+PSCs	1ug/ml	0	.	.	.	.
**Condition*Treatment**		HE+PSCs	5ug/ml	0	.	.	.	.
**Colony*Condit*Treatm**	BFU-E	AoE+PSCs	0ug/ml	0.4623	0.2159	36	2.14	0.0391
**Colony*Condit*Treatm**	BFU-E	AoE+PSCs	1ug/ml	1.3028	0.2357	36	5.53	<.0001
**Colony*Condit*Treatm**	BFU-E	AoE+PSCs	5ug/ml	0	.	.	.	.
**Colony*Condit*Treatm**	BFU-E	E11.5AGM	0ug/ml	-0.1487	0.1687	36	-0.88	0.3838
**Colony*Condit*Treatm**	BFU-E	E11.5AGM	1ug/ml	0.5439	0.1911	36	2.85	0.0073
**Colony*Condit*Treatm**	BFU-E	E11.5AGM	5ug/ml	0	.	.	.	.
**Colony*Condit*Treatm**	BFU-E	HE+PSCs	0ug/ml	0	.	.	.	.
**Colony*Condit*Treatm**	BFU-E	HE+PSCs	1ug/ml	0	.	.	.	.
**Colony*Condit*Treatm**	BFU-E	HE+PSCs	5ug/ml	0	.	.	.	.
**Colony*Condit*Treatm**	CFU-GEMM	AoE+PSCs	0ug/ml	-0.6526	0.1955	36	-3.34	0.0020
**Colony*Condit*Treatm**	CFU-GEMM	AoE+PSCs	1ug/ml	-0.2802	0.2098	36	-1.34	0.1900

The GLIMMIX Procedure

Solutions for Fixed Effects								
Effect	Colony_Type	Condition	Treatment	Estimate	Standard Error	DF	t Value	Pr > |t|
**Colony*Condit*Treatm**	CFU-GEMM	AoE+PSCs	5ug/ml	0	.	.	.	.
**Colony*Condit*Treatm**	CFU-GEMM	E11.5AGM	0ug/ml	-0.2274	0.1822	36	-1.25	0.2199
**Colony*Condit*Treatm**	CFU-GEMM	E11.5AGM	1ug/ml	0.01593	0.1949	36	0.08	0.9353
**Colony*Condit*Treatm**	CFU-GEMM	E11.5AGM	5ug/ml	0	.	.	.	.
**Colony*Condit*Treatm**	CFU-GEMM	HE+PSCs	0ug/ml	0	.	.	.	.
**Colony*Condit*Treatm**	CFU-GEMM	HE+PSCs	1ug/ml	0	.	.	.	.
**Colony*Condit*Treatm**	CFU-GEMM	HE+PSCs	5ug/ml	0	.	.	.	.
**Colony*Condit*Treatm**	CFU-GM	AoE+PSCs	0ug/ml	0	.	.	.	.
**Colony*Condit*Treatm**	CFU-GM	AoE+PSCs	1ug/ml	0	.	.	.	.
**Colony*Condit*Treatm**	CFU-GM	AoE+PSCs	5ug/ml	0	.	.	.	.
**Colony*Condit*Treatm**	CFU-GM	E11.5AGM	0ug/ml	0	.	.	.	.
**Colony*Condit*Treatm**	CFU-GM	E11.5AGM	1ug/ml	0	.	.	.	.
**Colony*Condit*Treatm**	CFU-GM	E11.5AGM	5ug/ml	0	.	.	.	.
**Colony*Condit*Treatm**	CFU-GM	HE+PSCs	0ug/ml	0	.	.	.	.
**Colony*Condit*Treatm**	CFU-GM	HE+PSCs	1ug/ml	0	.	.	.	.
**Colony*Condit*Treatm**	CFU-GM	HE+PSCs	5ug/ml	0	.	.	.	.

Type III Tests of Fixed Effects				
Effect	Num DF	Den DF	F Value	Pr > F
**Colony_Type**	2	36	1790.77	<.0001
**Condition**	2	36	160.64	<.0001
**Colony_Typ*Condition**	4	36	28.96	<.0001
**Treatment**	2	36	243.58	<.0001
**Colony_Typ*Treatment**	4	36	13.52	<.0001

The GLIMMIX Procedure

Type III Tests of Fixed Effects				
Effect	Num DF	Den DF	F Value	Pr > F
Condition*Treatment	4	36	3.23	0.0232
Colony*Condit*Treatm	8	36	8.00	<.0001

Condition*Treatment Least Squares Means													
Condition	Treatment	Estimate	Standard Error	DF	t Value	Pr > |t|	Alpha	Lower	Upper	Mean	Standard Error Mean	Lower Mean	Upper Mean
AoE+PSCs	0ug/ml	5.1073	0.06520	36	78.33	<.0001	0.05	4.9751	5.2396	165.23	10.7731	144.76	188.59
AoE+PSCs	1ug/ml	4.6823	0.06752	36	69.35	<.0001	0.05	4.5454	4.8192	108.02	7.2929	94.1959	123.87
AoE+PSCs	5ug/ml	3.8064	0.08141	36	46.75	<.0001	0.05	3.6413	3.9715	44.9867	3.6624	38.1397	53.0628
E11.5AGM	0ug/ml	5.7470	0.06170	36	93.14	<.0001	0.05	5.6219	5.8722	313.26	19.3285	276.41	355.01
E11.5AGM	1ug/ml	5.3783	0.06287	36	85.55	<.0001	0.05	5.2508	5.5058	216.64	13.6194	190.71	246.10
E11.5AGM	5ug/ml	4.6151	0.06793	36	67.94	<.0001	0.05	4.4773	4.7528	101.00	6.8604	87.9989	115.91
HE+PSCs	0ug/ml	5.0286	0.06545	36	76.83	<.0001	0.05	4.8958	5.1613	152.71	9.9946	133.73	174.39
HE+PSCs	1ug/ml	4.2378	0.07380	36	57.42	<.0001	0.05	4.0881	4.3875	69.2546	5.1110	59.6275	80.4360
HE+PSCs	5ug/ml	3.6027	0.08352	36	43.14	<.0001	0.05	3.4333	3.7721	36.6970	3.0648	30.9792	43.4702

Differences of Condition*Treatment Least Squares Means											
Condition	Treatment	_Condition	_Treatment	Estimate	Standard Error	DF	t Value	Pr > |t|	Alpha	Lower	Upper
AoE+PSCs	0ug/ml	AoE+PSCs	1ug/ml	0.4250	0.09386	36	4.53	<.0001	0.05	0.2347	0.6154
AoE+PSCs	0ug/ml	AoE+PSCs	5ug/ml	1.3009	0.1043	36	12.47	<.0001	0.05	1.0894	1.5125
AoE+PSCs	0ug/ml	E11.5AGM	0ug/ml	-0.6397	0.08977	36	-7.13	<.0001	0.05	-0.8218	-0.4576
AoE+PSCs	0ug/ml	E11.5AGM	1ug/ml	-0.2709	0.09057	36	-2.99	0.0050	0.05	-0.4546	-0.08725
AoE+PSCs	0ug/ml	E11.5AGM	5ug/ml	0.4922	0.09415	36	5.23	<.0001	0.05	0.3013	0.6832
AoE+PSCs	0ug/ml	HE+PSCs	0ug/ml	0.07874	0.09238	36	0.85	0.3996	0.05	-0.1086	0.2661

The SAS System

The GLIMMIX Procedure

Differences of Condition*Treatment Least Squares Means

Condition	Treatment	_Condition	_Treatment	Estimate	Standard Error	DF	t Value	Pr > |t|	Alpha	Lower	Upper
AoE+PSCs	0ug/ml	HE+PSCs	1ug/ml	0.8695	0.09847	36	8.83	<.0001	0.05	0.6698	1.0692
AoE+PSCs	0ug/ml	HE+PSCs	5ug/ml	1.5046	0.1060	36	14.20	<.0001	0.05	1.2897	1.7195
AoE+PSCs	1ug/ml	AoE+PSCs	5ug/ml	0.8759	0.1058	36	8.28	<.0001	0.05	0.6615	1.0904
AoE+PSCs	1ug/ml	E11.5AGM	0ug/ml	-1.0647	0.09146	36	-11.64	<.0001	0.05	-1.2502	-0.8792
AoE+PSCs	1ug/ml	E11.5AGM	1ug/ml	-0.6959	0.09225	36	-7.54	<.0001	0.05	-0.8830	-0.5089
AoE+PSCs	1ug/ml	E11.5AGM	5ug/ml	0.06722	0.09577	36	0.70	0.4873	0.05	-0.1270	0.2614
AoE+PSCs	1ug/ml	HE+PSCs	0ug/ml	-0.3463	0.09403	36	-3.68	0.0008	0.05	-0.5370	-0.1556
AoE+PSCs	1ug/ml	HE+PSCs	1ug/ml	0.4445	0.1000	36	4.44	<.0001	0.05	0.2417	0.6474
AoE+PSCs	1ug/ml	HE+PSCs	5ug/ml	1.0796	0.1074	36	10.05	<.0001	0.05	0.8618	1.2974
AoE+PSCs	5ug/ml	E11.5AGM	0ug/ml	-1.9407	0.1021	36	-19.00	<.0001	0.05	-2.1478	-1.7335
AoE+PSCs	5ug/ml	E11.5AGM	1ug/ml	-1.5719	0.1028	36	-15.28	<.0001	0.05	-1.7805	-1.3633
AoE+PSCs	5ug/ml	E11.5AGM	5ug/ml	-0.8087	0.1060	36	-7.63	<.0001	0.05	-1.0237	-0.5937
AoE+PSCs	5ug/ml	HE+PSCs	0ug/ml	-1.2222	0.1045	36	-11.70	<.0001	0.05	-1.4340	-1.0104
AoE+PSCs	5ug/ml	HE+PSCs	1ug/ml	-0.4314	0.1099	36	-3.93	0.0004	0.05	-0.6542	-0.2086
AoE+PSCs	5ug/ml	HE+PSCs	5ug/ml	0.2037	0.1166	36	1.75	0.0892	0.05	-0.03280	0.4401
E11.5AGM	0ug/ml	E11.5AGM	1ug/ml	0.3688	0.08809	36	4.19	0.0002	0.05	0.1901	0.5474
E11.5AGM	0ug/ml	E11.5AGM	5ug/ml	1.1319	0.09177	36	12.33	<.0001	0.05	0.9458	1.3180
E11.5AGM	0ug/ml	HE+PSCs	0ug/ml	0.7185	0.08995	36	7.99	<.0001	0.05	0.5360	0.9009
E11.5AGM	0ug/ml	HE+PSCs	1ug/ml	1.5092	0.09619	36	15.69	<.0001	0.05	1.3141	1.7043
E11.5AGM	0ug/ml	HE+PSCs	5ug/ml	2.1443	0.1038	36	20.65	<.0001	0.05	1.9337	2.3549
E11.5AGM	1ug/ml	E11.5AGM	5ug/ml	0.7632	0.09255	36	8.25	<.0001	0.05	0.5755	0.9509
E11.5AGM	1ug/ml	HE+PSCs	0ug/ml	0.3497	0.09075	36	3.85	0.0005	0.05	0.1656	0.5337
E11.5AGM	1ug/ml	HE+PSCs	1ug/ml	1.1405	0.09694	36	11.76	<.0001	0.05	0.9439	1.3371
E11.5AGM	1ug/ml	HE+PSCs	5ug/ml	1.7756	0.1045	36	16.99	<.0001	0.05	1.5636	1.9875

The GLIMMIX Procedure

Differences of Condition*Treatment Least Squares Means											
Condition	Treatment	_Condition	_Treatment	Estimate	Standard Error	DF	t Value	Pr > |t|	Alpha	Lower	Upper
E11.5AGM	5ug/ml	HE+PSCs	0ug/ml	-0.4135	0.09432	36	-4.38	<.0001	0.05	-0.6048	-0.2222
E11.5AGM	5ug/ml	HE+PSCs	1ug/ml	0.3773	0.1003	36	3.76	0.0006	0.05	0.1739	0.5807
E11.5AGM	5ug/ml	HE+PSCs	5ug/ml	1.0124	0.1076	36	9.40	<.0001	0.05	0.7941	1.2307
HE+PSCs	0ug/ml	HE+PSCs	1ug/ml	0.7908	0.09864	36	8.02	<.0001	0.05	0.5907	0.9908
HE+PSCs	0ug/ml	HE+PSCs	5ug/ml	1.4259	0.1061	36	13.44	<.0001	0.05	1.2107	1.6411
HE+PSCs	1ug/ml	HE+PSCs	5ug/ml	0.6351	0.1114	36	5.70	<.0001	0.05	0.4091	0.8611

Colony*Condit*Treatm Least Squares Means														
Colony_Type	Condition	Treatment	Estimate	Standard Error	DF	t Value	Pr > |t|	Alpha	Lower	Upper	Mean	Standard Error Mean	Lower Mean	Upper Mean
BFU-E	AoE+PSCs	0ug/ml	4.4886	0.08437	36	53.20	<.0001	0.05	4.3174	4.6597	88.9931	7.5084	74.9970	105.60
BFU-E	AoE+PSCs	1ug/ml	4.2869	0.08919	36	48.06	<.0001	0.05	4.1060	4.4678	72.7381	6.4878	60.7017	87.1611
BFU-E	AoE+PSCs	5ug/ml	2.8782	0.1481	36	19.43	<.0001	0.05	2.5778	3.1786	17.7826	2.6339	13.1685	24.0135
BFU-E	E11.5AGM	0ug/ml	5.3461	0.07052	36	75.81	<.0001	0.05	5.2030	5.4891	209.78	14.7925	181.82	242.03
BFU-E	E11.5AGM	1ug/ml	5.1450	0.07294	36	70.54	<.0001	0.05	4.9970	5.2929	171.56	12.5139	147.97	198.92
BFU-E	E11.5AGM	5ug/ml	4.4538	0.08515	36	52.30	<.0001	0.05	4.2811	4.6265	85.9511	7.3191	72.3184	102.15
BFU-E	HE+PSCs	0ug/ml	4.4745	0.08470	36	52.83	<.0001	0.05	4.3027	4.6463	87.7487	7.4326	73.8985	104.19
BFU-E	HE+PSCs	1ug/ml	3.4708	0.1171	36	29.65	<.0001	0.05	3.2334	3.7082	32.1619	3.7648	25.3652	40.7797
BFU-E	HE+PSCs	5ug/ml	3.2649	0.1267	36	25.76	<.0001	0.05	3.0079	3.5220	26.1785	3.3176	20.2452	33.8506
CFU-GEMM	AoE+PSCs	0ug/ml	4.5811	0.08239	36	55.60	<.0001	0.05	4.4140	4.7482	97.6190	8.0432	82.5967	115.37
CFU-GEMM	AoE+PSCs	1ug/ml	4.2870	0.08919	36	48.07	<.0001	0.05	4.1061	4.4679	72.7494	6.4885	60.7116	87.1739
CFU-GEMM	AoE+PSCs	5ug/ml	3.7792	0.1046	36	36.13	<.0001	0.05	3.5671	3.9914	43.7831	4.5800	35.4135	54.1306
CFU-GEMM	E11.5AGM	0ug/ml	5.2264	0.07193	36	72.66	<.0001	0.05	5.0805	5.3723	186.13	13.3883	160.86	215.36
CFU-GEMM	E11.5AGM	1ug/ml	4.9519	0.07571	36	65.41	<.0001	0.05	4.7984	5.1054	141.45	10.7081	121.31	164.92

The GLIMMIX Procedure

Colony*Condit*Treatm Least Squares Means														
Colony_Type	Condition	Treatment	Estimate	Standard Error	DF	t Value	Pr > |t|	Alpha	Lower	Upper	Mean	Standard Error Mean	Lower Mean	Upper Mean
CFU-GEMM	E11.5AGM	5ug/ml	4.1065	0.09410	36	43.64	<.0001	0.05	3.9156	4.2973	60.7326	5.7146	50.1815	73.5021
CFU-GEMM	HE+PSCs	0ug/ml	4.5544	0.08297	36	54.89	<.0001	0.05	4.3861	4.7227	95.0484	7.8860	80.3281	112.47
CFU-GEMM	HE+PSCs	1ug/ml	3.9265	0.09965	36	39.40	<.0001	0.05	3.7244	4.1286	50.7288	5.0549	41.4464	62.0901
CFU-GEMM	HE+PSCs	5ug/ml	3.0385	0.1389	36	21.88	<.0001	0.05	2.7569	3.3201	20.8732	2.8982	15.7504	27.6622
CFU-GM	AoE+PSCs	0ug/ml	6.2523	0.06346	36	98.52	<.0001	0.05	6.1236	6.3810	519.21	32.9497	456.51	590.53
CFU-GM	AoE+PSCs	1ug/ml	5.4730	0.06916	36	79.13	<.0001	0.05	5.3328	5.6133	238.18	16.4728	207.01	274.04
CFU-GM	AoE+PSCs	5ug/ml	4.7616	0.07898	36	60.29	<.0001	0.05	4.6015	4.9218	116.94	9.2356	99.6291	137.25
CFU-GM	E11.5AGM	0ug/ml	6.6686	0.06171	36	108.07	<.0001	0.05	6.5434	6.7937	787.29	48.5821	694.68	892.25
CFU-GM	E11.5AGM	1ug/ml	6.0379	0.06465	36	93.39	<.0001	0.05	5.9068	6.1690	419.01	27.0901	367.51	477.71
CFU-GM	E11.5AGM	5ug/ml	5.2850	0.07122	36	74.21	<.0001	0.05	5.1406	5.4294	197.35	14.0558	170.81	228.02
CFU-GM	HE+PSCs	0ug/ml	6.0568	0.06454	36	93.84	<.0001	0.05	5.9260	6.1877	427.03	27.5614	374.63	486.75
CFU-GM	HE+PSCs	1ug/ml	5.3161	0.07087	36	75.01	<.0001	0.05	5.1724	5.4598	203.59	14.4280	176.33	235.06
CFU-GM	HE+PSCs	5ug/ml	4.5047	0.08403	36	53.61	<.0001	0.05	4.3343	4.6751	90.4394	7.6000	76.2678	107.24

The GLIMMIX Procedure

Differences of Colony*Condit*Treatm Least Squares Means													
Colony_Type	Condition	Treatment	_Colony_Type	_Condition	_Treatment	Estimate	Standard Error	DF	t Value	Pr > |t|	Alpha	Lower	Upper
BFU-E	AoE+PSCs	0ug/ml	BFU-E	AoE+PSCs	1ug/ml	0.2017	0.1228	36	1.64	0.1091	0.05	-0.04730	0.4507
BFU-E	AoE+PSCs	0ug/ml	BFU-E	AoE+PSCs	5ug/ml	1.6103	0.1705	36	9.45	<.0001	0.05	1.2646	1.9560
BFU-E	AoE+PSCs	0ug/ml	BFU-E	E11.5AGM	0ug/ml	-0.8575	0.1100	36	-7.80	<.0001	0.05	-1.0805	-0.6345
BFU-E	AoE+PSCs	0ug/ml	BFU-E	E11.5AGM	1ug/ml	-0.6564	0.1115	36	-5.89	<.0001	0.05	-0.8826	-0.4302
BFU-E	AoE+PSCs	0ug/ml	BFU-E	E11.5AGM	5ug/ml	0.03478	0.1199	36	0.29	0.7734	0.05	-0.2083	0.2779
BFU-E	AoE+PSCs	0ug/ml	BFU-E	HE+PSCs	0ug/ml	0.01408	0.1196	36	0.12	0.9069	0.05	-0.2284	0.2565
BFU-E	AoE+PSCs	0ug/ml	BFU-E	HE+PSCs	1ug/ml	1.0178	0.1443	36	7.05	<.0001	0.05	0.7251	1.3104
BFU-E	AoE+PSCs	0ug/ml	BFU-E	HE+PSCs	5ug/ml	1.2236	0.1522	36	8.04	<.0001	0.05	0.9149	1.5324
BFU-E	AoE+PSCs	0ug/ml	CFU-GEMM	AoE+PSCs	0ug/ml	-0.09251	0.08445	36	-1.10	0.2806	0.05	-0.2638	0.07875
BFU-E	AoE+PSCs	0ug/ml	CFU-GEMM	AoE+PSCs	1ug/ml	0.2015	0.1228	36	1.64	0.1094	0.05	-0.04745	0.4505
BFU-E	AoE+PSCs	0ug/ml	CFU-GEMM	AoE+PSCs	5ug/ml	0.7093	0.1344	36	5.28	<.0001	0.05	0.4368	0.9819
BFU-E	AoE+PSCs	0ug/ml	CFU-GEMM	E11.5AGM	0ug/ml	-0.7379	0.1109	36	-6.66	<.0001	0.05	-0.9627	-0.5130
BFU-E	AoE+PSCs	0ug/ml	CFU-GEMM	E11.5AGM	1ug/ml	-0.4634	0.1134	36	-4.09	0.0002	0.05	-0.6932	-0.2335
BFU-E	AoE+PSCs	0ug/ml	CFU-GEMM	E11.5AGM	5ug/ml	0.3821	0.1264	36	3.02	0.0046	0.05	0.1258	0.6384
BFU-E	AoE+PSCs	0ug/ml	CFU-GEMM	HE+PSCs	0ug/ml	-0.06583	0.1183	36	-0.56	0.5814	0.05	-0.3058	0.1742
BFU-E	AoE+PSCs	0ug/ml	CFU-GEMM	HE+PSCs	1ug/ml	0.5621	0.1306	36	4.30	0.0001	0.05	0.2973	0.8269
BFU-E	AoE+PSCs	0ug/ml	CFU-GEMM	HE+PSCs	5ug/ml	1.4501	0.1625	36	8.93	<.0001	0.05	1.1206	1.7796
BFU-E	AoE+PSCs	0ug/ml	CFU-GM	AoE+PSCs	0ug/ml	-1.7638	0.06611	36	-26.68	<.0001	0.05	-1.8978	-1.6297
BFU-E	AoE+PSCs	0ug/ml	CFU-GM	AoE+PSCs	1ug/ml	-0.9845	0.1091	36	-9.02	<.0001	0.05	-1.2057	-0.7632
BFU-E	AoE+PSCs	0ug/ml	CFU-GM	AoE+PSCs	5ug/ml	-0.2731	0.1156	36	-2.36	0.0236	0.05	-0.5074	-0.03871
BFU-E	AoE+PSCs	0ug/ml	CFU-GM	E11.5AGM	0ug/ml	-2.1800	0.1045	36	-20.86	<.0001	0.05	-2.3920	-1.9680
BFU-E	AoE+PSCs	0ug/ml	CFU-GM	E11.5AGM	1ug/ml	-1.5493	0.1063	36	-14.58	<.0001	0.05	-1.7649	-1.3338
BFU-E	AoE+PSCs	0ug/ml	CFU-GM	E11.5AGM	5ug/ml	-0.7964	0.1104	36	-7.21	<.0001	0.05	-1.0204	-0.5725
BFU-E	AoE+PSCs	0ug/ml	CFU-GM	HE+PSCs	0ug/ml	-1.5683	0.1062	36	-14.76	<.0001	0.05	-1.7837	-1.3529

The GLIMMIX Procedure

Differences of Colony*Condit*Treatm Least Squares Means													
Colony_Type	Condition	Treatment	_Colony_Type	_Condition	_Treatment	Estimate	Standard Error	DF	t Value	Pr > |t|	Alpha	Lower	Upper
BFU-E	AoE+PSCs	0ug/ml	CFU-GM	HE+PSCs	1ug/ml	-0.8275	0.1102	36	-7.51	<.0001	0.05	-1.0510	-0.6041
BFU-E	AoE+PSCs	0ug/ml	CFU-GM	HE+PSCs	5ug/ml	-0.01612	0.1191	36	-0.14	0.8931	0.05	-0.2576	0.2254
BFU-E	AoE+PSCs	1ug/ml	BFU-E	AoE+PSCs	5ug/ml	1.4086	0.1729	36	8.15	<.0001	0.05	1.0580	1.7593
BFU-E	AoE+PSCs	1ug/ml	BFU-E	E11.5AGM	0ug/ml	-1.0592	0.1137	36	-9.32	<.0001	0.05	-1.2898	-0.8286
BFU-E	AoE+PSCs	1ug/ml	BFU-E	E11.5AGM	1ug/ml	-0.8581	0.1152	36	-7.45	<.0001	0.05	-1.0918	-0.6244
BFU-E	AoE+PSCs	1ug/ml	BFU-E	E11.5AGM	5ug/ml	-0.1669	0.1233	36	-1.35	0.1843	0.05	-0.4170	0.08318
BFU-E	AoE+PSCs	1ug/ml	BFU-E	HE+PSCs	0ug/ml	-0.1876	0.1230	36	-1.53	0.1359	0.05	-0.4371	0.06185
BFU-E	AoE+PSCs	1ug/ml	BFU-E	HE+PSCs	1ug/ml	0.8161	0.1472	36	5.55	<.0001	0.05	0.5176	1.1145
BFU-E	AoE+PSCs	1ug/ml	BFU-E	HE+PSCs	5ug/ml	1.0219	0.1550	36	6.59	<.0001	0.05	0.7076	1.3362
BFU-E	AoE+PSCs	1ug/ml	CFU-GEMM	AoE+PSCs	0ug/ml	-0.2942	0.1214	36	-2.42	0.0206	0.05	-0.5405	-0.04795
BFU-E	AoE+PSCs	1ug/ml	CFU-GEMM	AoE+PSCs	1ug/ml	-0.00016	0.09557	36	-0.00	0.9987	0.05	-0.1940	0.1937
BFU-E	AoE+PSCs	1ug/ml	CFU-GEMM	AoE+PSCs	5ug/ml	0.5076	0.1375	36	3.69	0.0007	0.05	0.2288	0.7864
BFU-E	AoE+PSCs	1ug/ml	CFU-GEMM	E11.5AGM	0ug/ml	-0.9396	0.1146	36	-8.20	<.0001	0.05	-1.1719	-0.7072
BFU-E	AoE+PSCs	1ug/ml	CFU-GEMM	E11.5AGM	1ug/ml	-0.6650	0.1170	36	-5.68	<.0001	0.05	-0.9023	-0.4278
BFU-E	AoE+PSCs	1ug/ml	CFU-GEMM	E11.5AGM	5ug/ml	0.1804	0.1296	36	1.39	0.1727	0.05	-0.08256	0.4433
BFU-E	AoE+PSCs	1ug/ml	CFU-GEMM	HE+PSCs	0ug/ml	-0.2675	0.1218	36	-2.20	0.0346	0.05	-0.5146	-0.02047
BFU-E	AoE+PSCs	1ug/ml	CFU-GEMM	HE+PSCs	1ug/ml	0.3604	0.1337	36	2.69	0.0106	0.05	0.08915	0.6316
BFU-E	AoE+PSCs	1ug/ml	CFU-GEMM	HE+PSCs	5ug/ml	1.2484	0.1650	36	7.56	<.0001	0.05	0.9137	1.5831
BFU-E	AoE+PSCs	1ug/ml	CFU-GM	AoE+PSCs	0ug/ml	-1.9655	0.1095	36	-17.96	<.0001	0.05	-2.1875	-1.7434
BFU-E	AoE+PSCs	1ug/ml	CFU-GM	AoE+PSCs	1ug/ml	-1.1862	0.07721	36	-15.36	<.0001	0.05	-1.3428	-1.0296
BFU-E	AoE+PSCs	1ug/ml	CFU-GM	AoE+PSCs	5ug/ml	-0.4748	0.1191	36	-3.99	0.0003	0.05	-0.7164	-0.2332
BFU-E	AoE+PSCs	1ug/ml	CFU-GM	E11.5AGM	0ug/ml	-2.3817	0.1085	36	-21.96	<.0001	0.05	-2.6017	-2.1618
BFU-E	AoE+PSCs	1ug/ml	CFU-GM	E11.5AGM	1ug/ml	-1.7510	0.1102	36	-15.90	<.0001	0.05	-1.9744	-1.5276
BFU-E	AoE+PSCs	1ug/ml	CFU-GM	E11.5AGM	5ug/ml	-0.9981	0.1141	36	-8.75	<.0001	0.05	-1.2296	-0.7667

The GLIMMIX Procedure

Differences of Colony*Condit*Treatm Least Squares Means													
Colony_Type	Condition	Treatment	_Colony_Type	_Condition	_Treatment	Estimate	Standard Error	DF	t Value	Pr > |t|	Alpha	Lower	Upper
BFU-E	AoE+PSCs	1ug/ml	CFU-GM	HE+PSCs	0ug/ml	-1.7700	0.1101	36	-16.08	<.0001	0.05	-1.9933	-1.5467
BFU-E	AoE+PSCs	1ug/ml	CFU-GM	HE+PSCs	1ug/ml	-1.0292	0.1139	36	-9.03	<.0001	0.05	-1.2603	-0.7982
BFU-E	AoE+PSCs	1ug/ml	CFU-GM	HE+PSCs	5ug/ml	-0.2178	0.1225	36	-1.78	0.0839	0.05	-0.4663	0.03071
BFU-E	AoE+PSCs	5ug/ml	BFU-E	E11.5AGM	0ug/ml	-2.4678	0.1640	36	-15.04	<.0001	0.05	-2.8005	-2.1351
BFU-E	AoE+PSCs	5ug/ml	BFU-E	E11.5AGM	1ug/ml	-2.2667	0.1651	36	-13.73	<.0001	0.05	-2.6016	-1.9319
BFU-E	AoE+PSCs	5ug/ml	BFU-E	E11.5AGM	5ug/ml	-1.5756	0.1708	36	-9.22	<.0001	0.05	-1.9220	-1.2291
BFU-E	AoE+PSCs	5ug/ml	BFU-E	HE+PSCs	0ug/ml	-1.5963	0.1706	36	-9.36	<.0001	0.05	-1.9423	-1.2502
BFU-E	AoE+PSCs	5ug/ml	BFU-E	HE+PSCs	1ug/ml	-0.5926	0.1888	36	-3.14	0.0034	0.05	-0.9754	-0.2097
BFU-E	AoE+PSCs	5ug/ml	BFU-E	HE+PSCs	5ug/ml	-0.3867	0.1949	36	-1.98	0.0549	0.05	-0.7820	0.008583
BFU-E	AoE+PSCs	5ug/ml	CFU-GEMM	AoE+PSCs	0ug/ml	-1.7029	0.1695	36	-10.05	<.0001	0.05	-2.0466	-1.3591
BFU-E	AoE+PSCs	5ug/ml	CFU-GEMM	AoE+PSCs	1ug/ml	-1.4088	0.1729	36	-8.15	<.0001	0.05	-1.7594	-1.0582
BFU-E	AoE+PSCs	5ug/ml	CFU-GEMM	AoE+PSCs	5ug/ml	-0.9010	0.1614	36	-5.58	<.0001	0.05	-1.2283	-0.5738
BFU-E	AoE+PSCs	5ug/ml	CFU-GEMM	E11.5AGM	0ug/ml	-2.3482	0.1647	36	-14.26	<.0001	0.05	-2.6821	-2.0143
BFU-E	AoE+PSCs	5ug/ml	CFU-GEMM	E11.5AGM	1ug/ml	-2.0737	0.1663	36	-12.47	<.0001	0.05	-2.4110	-1.7364
BFU-E	AoE+PSCs	5ug/ml	CFU-GEMM	E11.5AGM	5ug/ml	-1.2283	0.1755	36	-7.00	<.0001	0.05	-1.5841	-0.8724
BFU-E	AoE+PSCs	5ug/ml	CFU-GEMM	HE+PSCs	0ug/ml	-1.6762	0.1698	36	-9.87	<.0001	0.05	-2.0205	-1.3319
BFU-E	AoE+PSCs	5ug/ml	CFU-GEMM	HE+PSCs	1ug/ml	-1.0483	0.1785	36	-5.87	<.0001	0.05	-1.4103	-0.6863
BFU-E	AoE+PSCs	5ug/ml	CFU-GEMM	HE+PSCs	5ug/ml	-0.1602	0.2030	36	-0.79	0.4351	0.05	-0.5720	0.2515
BFU-E	AoE+PSCs	5ug/ml	CFU-GM	AoE+PSCs	0ug/ml	-3.3741	0.1611	36	-20.94	<.0001	0.05	-3.7009	-3.0473
BFU-E	AoE+PSCs	5ug/ml	CFU-GM	AoE+PSCs	1ug/ml	-2.5948	0.1635	36	-15.87	<.0001	0.05	-2.9263	-2.2633
BFU-E	AoE+PSCs	5ug/ml	CFU-GM	AoE+PSCs	5ug/ml	-1.8834	0.1461	36	-12.89	<.0001	0.05	-2.1796	-1.5872
BFU-E	AoE+PSCs	5ug/ml	CFU-GM	E11.5AGM	0ug/ml	-3.7904	0.1605	36	-23.62	<.0001	0.05	-4.1158	-3.4650
BFU-E	AoE+PSCs	5ug/ml	CFU-GM	E11.5AGM	1ug/ml	-3.1597	0.1616	36	-19.55	<.0001	0.05	-3.4874	-2.8319
BFU-E	AoE+PSCs	5ug/ml	CFU-GM	E11.5AGM	5ug/ml	-2.4068	0.1643	36	-14.65	<.0001	0.05	-2.7401	-2.0735

The GLIMMIX Procedure

Differences of Colony*Condit*Treatm Least Squares Means													
Colony_Type	Condition	Treatment	_Colony_Type	_Condition	_Treatment	Estimate	Standard Error	DF	t Value	Pr > |t|	Alpha	Lower	Upper
BFU-E	AoE+PSCs	5ug/ml	CFU-GM	HE+PSCs	0ug/ml	-3.1786	0.1616	36	-19.67	<.0001	0.05	-3.5063	-2.8510
BFU-E	AoE+PSCs	5ug/ml	CFU-GM	HE+PSCs	1ug/ml	-2.4379	0.1642	36	-14.85	<.0001	0.05	-2.7709	-2.1049
BFU-E	AoE+PSCs	5ug/ml	CFU-GM	HE+PSCs	5ug/ml	-1.6265	0.1703	36	-9.55	<.0001	0.05	-1.9718	-1.2811
BFU-E	E11.5AGM	0ug/ml	BFU-E	E11.5AGM	1ug/ml	0.2011	0.1015	36	1.98	0.0551	0.05	-0.00467	0.4068
BFU-E	E11.5AGM	0ug/ml	BFU-E	E11.5AGM	5ug/ml	0.8923	0.1106	36	8.07	<.0001	0.05	0.6680	1.1165
BFU-E	E11.5AGM	0ug/ml	BFU-E	HE+PSCs	0ug/ml	0.8716	0.1102	36	7.91	<.0001	0.05	0.6481	1.0951
BFU-E	E11.5AGM	0ug/ml	BFU-E	HE+PSCs	1ug/ml	1.8753	0.1367	36	13.72	<.0001	0.05	1.5981	2.1524
BFU-E	E11.5AGM	0ug/ml	BFU-E	HE+PSCs	5ug/ml	2.0811	0.1450	36	14.35	<.0001	0.05	1.7870	2.3752
BFU-E	E11.5AGM	0ug/ml	CFU-GEMM	AoE+PSCs	0ug/ml	0.7650	0.1084	36	7.05	<.0001	0.05	0.5450	0.9849
BFU-E	E11.5AGM	0ug/ml	CFU-GEMM	AoE+PSCs	1ug/ml	1.0590	0.1137	36	9.31	<.0001	0.05	0.8284	1.2896
BFU-E	E11.5AGM	0ug/ml	CFU-GEMM	AoE+PSCs	5ug/ml	1.5668	0.1262	36	12.42	<.0001	0.05	1.3110	1.8227
BFU-E	E11.5AGM	0ug/ml	CFU-GEMM	E11.5AGM	0ug/ml	0.1196	0.05811	36	2.06	0.0468	0.05	0.001777	0.2375
BFU-E	E11.5AGM	0ug/ml	CFU-GEMM	E11.5AGM	1ug/ml	0.3941	0.1035	36	3.81	0.0005	0.05	0.1843	0.6040
BFU-E	E11.5AGM	0ug/ml	CFU-GEMM	E11.5AGM	5ug/ml	1.2396	0.1176	36	10.54	<.0001	0.05	1.0011	1.4780
BFU-E	E11.5AGM	0ug/ml	CFU-GEMM	HE+PSCs	0ug/ml	0.7917	0.1089	36	7.27	<.0001	0.05	0.5708	1.0125
BFU-E	E11.5AGM	0ug/ml	CFU-GEMM	HE+PSCs	1ug/ml	1.4196	0.1221	36	11.63	<.0001	0.05	1.1720	1.6671
BFU-E	E11.5AGM	0ug/ml	CFU-GEMM	HE+PSCs	5ug/ml	2.3076	0.1557	36	14.82	<.0001	0.05	1.9918	2.6234
BFU-E	E11.5AGM	0ug/ml	CFU-GM	AoE+PSCs	0ug/ml	-0.9063	0.09487	36	-9.55	<.0001	0.05	-1.0987	-0.7139
BFU-E	E11.5AGM	0ug/ml	CFU-GM	AoE+PSCs	1ug/ml	-0.1270	0.09877	36	-1.29	0.2068	0.05	-0.3273	0.07334
BFU-E	E11.5AGM	0ug/ml	CFU-GM	AoE+PSCs	5ug/ml	0.5844	0.1059	36	5.52	<.0001	0.05	0.3697	0.7991
BFU-E	E11.5AGM	0ug/ml	CFU-GM	E11.5AGM	0ug/ml	-1.3225	0.04484	36	-29.50	<.0001	0.05	-1.4135	-1.2316
BFU-E	E11.5AGM	0ug/ml	CFU-GM	E11.5AGM	1ug/ml	-0.6918	0.09567	36	-7.23	<.0001	0.05	-0.8859	-0.4978
BFU-E	E11.5AGM	0ug/ml	CFU-GM	E11.5AGM	5ug/ml	0.06105	0.1002	36	0.61	0.5462	0.05	-0.1422	0.2643
BFU-E	E11.5AGM	0ug/ml	CFU-GM	HE+PSCs	0ug/ml	-0.7108	0.09559	36	-7.44	<.0001	0.05	-0.9047	-0.5169

The GLIMMIX Procedure

Differences of Colony*Condit*Treatm Least Squares Means													
Colony_Type	Condition	Treatment	_Colony_Type	_Condition	_Treatment	Estimate	Standard Error	DF	t Value	Pr > |t|	Alpha	Lower	Upper
BFU-E	E11.5AGM	0ug/ml	CFU-GM	HE+PSCs	1ug/ml	0.02996	0.09997	36	0.30	0.7662	0.05	-0.1728	0.2327
BFU-E	E11.5AGM	0ug/ml	CFU-GM	HE+PSCs	5ug/ml	0.8414	0.1097	36	7.67	<.0001	0.05	0.6189	1.0639
BFU-E	E11.5AGM	1ug/ml	BFU-E	E11.5AGM	5ug/ml	0.6912	0.1121	36	6.16	<.0001	0.05	0.4638	0.9186
BFU-E	E11.5AGM	1ug/ml	BFU-E	HE+PSCs	0ug/ml	0.6705	0.1118	36	6.00	<.0001	0.05	0.4438	0.8972
BFU-E	E11.5AGM	1ug/ml	BFU-E	HE+PSCs	1ug/ml	1.6742	0.1379	36	12.14	<.0001	0.05	1.3945	1.9539
BFU-E	E11.5AGM	1ug/ml	BFU-E	HE+PSCs	5ug/ml	1.8800	0.1462	36	12.86	<.0001	0.05	1.5835	2.1766
BFU-E	E11.5AGM	1ug/ml	CFU-GEMM	AoE+PSCs	0ug/ml	0.5639	0.1100	36	5.12	<.0001	0.05	0.3407	0.7871
BFU-E	E11.5AGM	1ug/ml	CFU-GEMM	AoE+PSCs	1ug/ml	0.8579	0.1152	36	7.45	<.0001	0.05	0.6243	1.0916
BFU-E	E11.5AGM	1ug/ml	CFU-GEMM	AoE+PSCs	5ug/ml	1.3657	0.1275	36	10.71	<.0001	0.05	1.1071	1.6243
BFU-E	E11.5AGM	1ug/ml	CFU-GEMM	E11.5AGM	0ug/ml	-0.08146	0.1024	36	-0.80	0.4317	0.05	-0.2892	0.1263
BFU-E	E11.5AGM	1ug/ml	CFU-GEMM	E11.5AGM	1ug/ml	0.1931	0.06536	36	2.95	0.0055	0.05	0.06049	0.3256
BFU-E	E11.5AGM	1ug/ml	CFU-GEMM	E11.5AGM	5ug/ml	1.0385	0.1191	36	8.72	<.0001	0.05	0.7970	1.2799
BFU-E	E11.5AGM	1ug/ml	CFU-GEMM	HE+PSCs	0ug/ml	0.5906	0.1105	36	5.35	<.0001	0.05	0.3665	0.8146
BFU-E	E11.5AGM	1ug/ml	CFU-GEMM	HE+PSCs	1ug/ml	1.2185	0.1235	36	9.87	<.0001	0.05	0.9680	1.4689
BFU-E	E11.5AGM	1ug/ml	CFU-GEMM	HE+PSCs	5ug/ml	2.1065	0.1568	36	13.43	<.0001	0.05	1.7884	2.4246
BFU-E	E11.5AGM	1ug/ml	CFU-GM	AoE+PSCs	0ug/ml	-1.1074	0.09668	36	-11.45	<.0001	0.05	-1.3034	-0.9113
BFU-E	E11.5AGM	1ug/ml	CFU-GM	AoE+PSCs	1ug/ml	-0.3281	0.1005	36	-3.26	0.0024	0.05	-0.5319	-0.1242
BFU-E	E11.5AGM	1ug/ml	CFU-GM	AoE+PSCs	5ug/ml	0.3833	0.1075	36	3.57	0.0010	0.05	0.1653	0.6013
BFU-E	E11.5AGM	1ug/ml	CFU-GM	E11.5AGM	0ug/ml	-1.5236	0.09554	36	-15.95	<.0001	0.05	-1.7174	-1.3299
BFU-E	E11.5AGM	1ug/ml	CFU-GM	E11.5AGM	1ug/ml	-0.8929	0.05216	36	-17.12	<.0001	0.05	-0.9987	-0.7871
BFU-E	E11.5AGM	1ug/ml	CFU-GM	E11.5AGM	5ug/ml	-0.1400	0.1019	36	-1.37	0.1780	0.05	-0.3468	0.06671
BFU-E	E11.5AGM	1ug/ml	CFU-GM	HE+PSCs	0ug/ml	-0.9119	0.09739	36	-9.36	<.0001	0.05	-1.1094	-0.7144
BFU-E	E11.5AGM	1ug/ml	CFU-GM	HE+PSCs	1ug/ml	-0.1711	0.1017	36	-1.68	0.1011	0.05	-0.3774	0.03511
BFU-E	E11.5AGM	1ug/ml	CFU-GM	HE+PSCs	5ug/ml	0.6403	0.1113	36	5.75	<.0001	0.05	0.4146	0.8659

The GLIMMIX Procedure

Differences of Colony*Condit*Treatm Least Squares Means													
Colony_Type	Condition	Treatment	_Colony_Type	_Condition	_Treatment	Estimate	Standard Error	DF	t Value	Pr > |t|	Alpha	Lower	Upper
BFU-E	E11.5AGM	5ug/ml	BFU-E	HE+PSCs	0ug/ml	-0.02070	0.1201	36	-0.17	0.8641	0.05	-0.2643	0.2229
BFU-E	E11.5AGM	5ug/ml	BFU-E	HE+PSCs	1ug/ml	0.9830	0.1447	36	6.79	<.0001	0.05	0.6894	1.2766
BFU-E	E11.5AGM	5ug/ml	BFU-E	HE+PSCs	5ug/ml	1.1888	0.1527	36	7.79	<.0001	0.05	0.8792	1.4985
BFU-E	E11.5AGM	5ug/ml	CFU-GEMM	AoE+PSCs	0ug/ml	-0.1273	0.1185	36	-1.07	0.2898	0.05	-0.3676	0.1130
BFU-E	E11.5AGM	5ug/ml	CFU-GEMM	AoE+PSCs	1ug/ml	0.1668	0.1233	36	1.35	0.1847	0.05	-0.08333	0.4168
BFU-E	E11.5AGM	5ug/ml	CFU-GEMM	AoE+PSCs	5ug/ml	0.6745	0.1349	36	5.00	<.0001	0.05	0.4010	0.9481
BFU-E	E11.5AGM	5ug/ml	CFU-GEMM	E11.5AGM	0ug/ml	-0.7726	0.1115	36	-6.93	<.0001	0.05	-0.9987	-0.5466
BFU-E	E11.5AGM	5ug/ml	CFU-GEMM	E11.5AGM	1ug/ml	-0.4981	0.1139	36	-4.37	0.0001	0.05	-0.7292	-0.2671
BFU-E	E11.5AGM	5ug/ml	CFU-GEMM	E11.5AGM	5ug/ml	0.3473	0.09655	36	3.60	0.0010	0.05	0.1515	0.5431
BFU-E	E11.5AGM	5ug/ml	CFU-GEMM	HE+PSCs	0ug/ml	-0.1006	0.1189	36	-0.85	0.4030	0.05	-0.3417	0.1405
BFU-E	E11.5AGM	5ug/ml	CFU-GEMM	HE+PSCs	1ug/ml	0.5273	0.1311	36	4.02	0.0003	0.05	0.2615	0.7931
BFU-E	E11.5AGM	5ug/ml	CFU-GEMM	HE+PSCs	5ug/ml	1.4153	0.1629	36	8.69	<.0001	0.05	1.0850	1.7456
BFU-E	E11.5AGM	5ug/ml	CFU-GM	AoE+PSCs	0ug/ml	-1.7985	0.1062	36	-16.94	<.0001	0.05	-2.0139	-1.5832
BFU-E	E11.5AGM	5ug/ml	CFU-GM	AoE+PSCs	1ug/ml	-1.0192	0.1097	36	-9.29	<.0001	0.05	-1.2417	-0.7968
BFU-E	E11.5AGM	5ug/ml	CFU-GM	AoE+PSCs	5ug/ml	-0.3079	0.1161	36	-2.65	0.0119	0.05	-0.5434	-0.07234
BFU-E	E11.5AGM	5ug/ml	CFU-GM	E11.5AGM	0ug/ml	-2.2148	0.1052	36	-21.06	<.0001	0.05	-2.4281	-2.0015
BFU-E	E11.5AGM	5ug/ml	CFU-GM	E11.5AGM	1ug/ml	-1.5841	0.1069	36	-14.82	<.0001	0.05	-1.8009	-1.3673
BFU-E	E11.5AGM	5ug/ml	CFU-GM	E11.5AGM	5ug/ml	-0.8312	0.07444	36	-11.17	<.0001	0.05	-0.9822	-0.6803
BFU-E	E11.5AGM	5ug/ml	CFU-GM	HE+PSCs	0ug/ml	-1.6031	0.1068	36	-15.00	<.0001	0.05	-1.8198	-1.3864
BFU-E	E11.5AGM	5ug/ml	CFU-GM	HE+PSCs	1ug/ml	-0.8623	0.1108	36	-7.78	<.0001	0.05	-1.0870	-0.6376
BFU-E	E11.5AGM	5ug/ml	CFU-GM	HE+PSCs	5ug/ml	-0.05090	0.1196	36	-0.43	0.6730	0.05	-0.2935	0.1917
BFU-E	HE+PSCs	0ug/ml	BFU-E	HE+PSCs	1ug/ml	1.0037	0.1445	36	6.95	<.0001	0.05	0.7107	1.2967
BFU-E	HE+PSCs	0ug/ml	BFU-E	HE+PSCs	5ug/ml	1.2095	0.1524	36	7.94	<.0001	0.05	0.9004	1.5187
BFU-E	HE+PSCs	0ug/ml	CFU-GEMM	AoE+PSCs	0ug/ml	-0.1066	0.1182	36	-0.90	0.3730	0.05	-0.3462	0.1331

The GLIMMIX Procedure

Differences of Colony*Condit*Treatm Least Squares Means													
Colony_Type	Condition	Treatment	_Colony_Type	_Condition	_Treatment	Estimate	Standard Error	DF	t Value	Pr > |t|	Alpha	Lower	Upper
BFU-E	HE+PSCs	0ug/ml	CFU-GEMM	AoE+PSCs	1ug/ml	0.1875	0.1230	36	1.52	0.1362	0.05	-0.06200	0.4369
BFU-E	HE+PSCs	0ug/ml	CFU-GEMM	AoE+PSCs	5ug/ml	0.6952	0.1346	36	5.17	<.0001	0.05	0.4223	0.9682
BFU-E	HE+PSCs	0ug/ml	CFU-GEMM	E11.5AGM	0ug/ml	-0.7519	0.1111	36	-6.77	<.0001	0.05	-0.9773	-0.5266
BFU-E	HE+PSCs	0ug/ml	CFU-GEMM	E11.5AGM	1ug/ml	-0.4774	0.1136	36	-4.20	0.0002	0.05	-0.7078	-0.2470
BFU-E	HE+PSCs	0ug/ml	CFU-GEMM	E11.5AGM	5ug/ml	0.3680	0.1266	36	2.91	0.0062	0.05	0.1112	0.6248
BFU-E	HE+PSCs	0ug/ml	CFU-GEMM	HE+PSCs	0ug/ml	-0.07991	0.08534	36	-0.94	0.3553	0.05	-0.2530	0.09317
BFU-E	HE+PSCs	0ug/ml	CFU-GEMM	HE+PSCs	1ug/ml	0.5480	0.1308	36	4.19	0.0002	0.05	0.2827	0.8132
BFU-E	HE+PSCs	0ug/ml	CFU-GEMM	HE+PSCs	5ug/ml	1.4360	0.1626	36	8.83	<.0001	0.05	1.1062	1.7659
BFU-E	HE+PSCs	0ug/ml	CFU-GM	AoE+PSCs	0ug/ml	-1.7778	0.1058	36	-16.80	<.0001	0.05	-1.9925	-1.5632
BFU-E	HE+PSCs	0ug/ml	CFU-GM	AoE+PSCs	1ug/ml	-0.9985	0.1094	36	-9.13	<.0001	0.05	-1.2203	-0.7768
BFU-E	HE+PSCs	0ug/ml	CFU-GM	AoE+PSCs	5ug/ml	-0.2872	0.1158	36	-2.48	0.0180	0.05	-0.5220	-0.05229
BFU-E	HE+PSCs	0ug/ml	CFU-GM	E11.5AGM	0ug/ml	-2.1941	0.1048	36	-20.94	<.0001	0.05	-2.4067	-1.9816
BFU-E	HE+PSCs	0ug/ml	CFU-GM	E11.5AGM	1ug/ml	-1.5634	0.1066	36	-14.67	<.0001	0.05	-1.7795	-1.3473
BFU-E	HE+PSCs	0ug/ml	CFU-GM	E11.5AGM	5ug/ml	-0.8105	0.1107	36	-7.32	<.0001	0.05	-1.0350	-0.5861
BFU-E	HE+PSCs	0ug/ml	CFU-GM	HE+PSCs	0ug/ml	-1.5824	0.06757	36	-23.42	<.0001	0.05	-1.7194	-1.4453
BFU-E	HE+PSCs	0ug/ml	CFU-GM	HE+PSCs	1ug/ml	-0.8416	0.1104	36	-7.62	<.0001	0.05	-1.0656	-0.6176
BFU-E	HE+PSCs	0ug/ml	CFU-GM	HE+PSCs	5ug/ml	-0.03020	0.1193	36	-0.25	0.8016	0.05	-0.2722	0.2118
BFU-E	HE+PSCs	1ug/ml	BFU-E	HE+PSCs	5ug/ml	0.2058	0.1725	36	1.19	0.2406	0.05	-0.1440	0.5557
BFU-E	HE+PSCs	1ug/ml	CFU-GEMM	AoE+PSCs	0ug/ml	-1.1103	0.1431	36	-7.76	<.0001	0.05	-1.4006	-0.8200
BFU-E	HE+PSCs	1ug/ml	CFU-GEMM	AoE+PSCs	1ug/ml	-0.8162	0.1472	36	-5.55	<.0001	0.05	-1.1147	-0.5178
BFU-E	HE+PSCs	1ug/ml	CFU-GEMM	AoE+PSCs	5ug/ml	-0.3085	0.1570	36	-1.97	0.0572	0.05	-0.6268	0.009886
BFU-E	HE+PSCs	1ug/ml	CFU-GEMM	E11.5AGM	0ug/ml	-1.7556	0.1374	36	-12.78	<.0001	0.05	-2.0343	-1.4770
BFU-E	HE+PSCs	1ug/ml	CFU-GEMM	E11.5AGM	1ug/ml	-1.4811	0.1394	36	-10.62	<.0001	0.05	-1.7638	-1.1984
BFU-E	HE+PSCs	1ug/ml	CFU-GEMM	E11.5AGM	5ug/ml	-0.6357	0.1502	36	-4.23	0.0002	0.05	-0.9403	-0.3311

The GLIMMIX Procedure

Differences of Colony*Condit*Treatm Least Squares Means													
Colony_Type	Condition	Treatment	_Colony_Type	_Condition	_Treatment	Estimate	Standard Error	DF	t Value	Pr > |t|	Alpha	Lower	Upper
BFU-E	HE+PSCs	1ug/ml	CFU-GEMM	HE+PSCs	0ug/ml	-1.0836	0.1435	36	-7.55	<.0001	0.05	-1.3746	-0.7926
BFU-E	HE+PSCs	1ug/ml	CFU-GEMM	HE+PSCs	1ug/ml	-0.4557	0.1298	36	-3.51	0.0012	0.05	-0.7189	-0.1925
BFU-E	HE+PSCs	1ug/ml	CFU-GEMM	HE+PSCs	5ug/ml	0.4323	0.1816	36	2.38	0.0227	0.05	0.06401	0.8006
BFU-E	HE+PSCs	1ug/ml	CFU-GM	AoE+PSCs	0ug/ml	-2.7815	0.1332	36	-20.89	<.0001	0.05	-3.0516	-2.5115
BFU-E	HE+PSCs	1ug/ml	CFU-GM	AoE+PSCs	1ug/ml	-2.0022	0.1360	36	-14.73	<.0001	0.05	-2.2780	-1.7265
BFU-E	HE+PSCs	1ug/ml	CFU-GM	AoE+PSCs	5ug/ml	-1.2909	0.1412	36	-9.14	<.0001	0.05	-1.5772	-1.0045
BFU-E	HE+PSCs	1ug/ml	CFU-GM	E11.5AGM	0ug/ml	-3.1978	0.1323	36	-24.17	<.0001	0.05	-3.4662	-2.9294
BFU-E	HE+PSCs	1ug/ml	CFU-GM	E11.5AGM	1ug/ml	-2.5671	0.1337	36	-19.20	<.0001	0.05	-2.8383	-2.2959
BFU-E	HE+PSCs	1ug/ml	CFU-GM	E11.5AGM	5ug/ml	-1.8142	0.1370	36	-13.24	<.0001	0.05	-2.0921	-1.5363
BFU-E	HE+PSCs	1ug/ml	CFU-GM	HE+PSCs	0ug/ml	-2.5861	0.1337	36	-19.35	<.0001	0.05	-2.8572	-2.3150
BFU-E	HE+PSCs	1ug/ml	CFU-GM	HE+PSCs	1ug/ml	-1.8453	0.1093	36	-16.89	<.0001	0.05	-2.0669	-1.6237
BFU-E	HE+PSCs	1ug/ml	CFU-GM	HE+PSCs	5ug/ml	-1.0339	0.1441	36	-7.18	<.0001	0.05	-1.3261	-0.7417
BFU-E	HE+PSCs	5ug/ml	CFU-GEMM	AoE+PSCs	0ug/ml	-1.3161	0.1512	36	-8.71	<.0001	0.05	-1.6227	-1.0096
BFU-E	HE+PSCs	5ug/ml	CFU-GEMM	AoE+PSCs	1ug/ml	-1.0221	0.1550	36	-6.60	<.0001	0.05	-1.3364	-0.7078
BFU-E	HE+PSCs	5ug/ml	CFU-GEMM	AoE+PSCs	5ug/ml	-0.5143	0.1643	36	-3.13	0.0035	0.05	-0.8475	-0.1811
BFU-E	HE+PSCs	5ug/ml	CFU-GEMM	E11.5AGM	0ug/ml	-1.9615	0.1457	36	-13.46	<.0001	0.05	-2.2570	-1.6660
BFU-E	HE+PSCs	5ug/ml	CFU-GEMM	E11.5AGM	1ug/ml	-1.6870	0.1476	36	-11.43	<.0001	0.05	-1.9864	-1.3876
BFU-E	HE+PSCs	5ug/ml	CFU-GEMM	E11.5AGM	5ug/ml	-0.8415	0.1578	36	-5.33	<.0001	0.05	-1.1617	-0.5214
BFU-E	HE+PSCs	5ug/ml	CFU-GEMM	HE+PSCs	0ug/ml	-1.2894	0.1515	36	-8.51	<.0001	0.05	-1.5966	-0.9822
BFU-E	HE+PSCs	5ug/ml	CFU-GEMM	HE+PSCs	1ug/ml	-0.6616	0.1612	36	-4.10	0.0002	0.05	-0.9885	-0.3346
BFU-E	HE+PSCs	5ug/ml	CFU-GEMM	HE+PSCs	5ug/ml	0.2265	0.1690	36	1.34	0.1885	0.05	-0.1162	0.5691
BFU-E	HE+PSCs	5ug/ml	CFU-GM	AoE+PSCs	0ug/ml	-2.9874	0.1417	36	-21.08	<.0001	0.05	-3.2748	-2.6999
BFU-E	HE+PSCs	5ug/ml	CFU-GM	AoE+PSCs	1ug/ml	-2.2081	0.1444	36	-15.29	<.0001	0.05	-2.5009	-1.9153
BFU-E	HE+PSCs	5ug/ml	CFU-GM	AoE+PSCs	5ug/ml	-1.4967	0.1493	36	-10.02	<.0001	0.05	-1.7995	-1.1939

The GLIMMIX Procedure

Differences of Colony*Condit*Treatm Least Squares Means													
Colony_Type	Condition	Treatment	_Colony_Type	_Condition	_Treatment	Estimate	Standard Error	DF	t Value	Pr > |t|	Alpha	Lower	Upper
BFU-E	HE+PSCs	5ug/ml	CFU-GM	E11.5AGM	0ug/ml	-3.4037	0.1410	36	-24.15	<.0001	0.05	-3.6895	-3.1178
BFU-E	HE+PSCs	5ug/ml	CFU-GM	E11.5AGM	1ug/ml	-2.7729	0.1423	36	-19.49	<.0001	0.05	-3.0615	-2.4844
BFU-E	HE+PSCs	5ug/ml	CFU-GM	E11.5AGM	5ug/ml	-2.0201	0.1454	36	-13.90	<.0001	0.05	-2.3149	-1.7252
BFU-E	HE+PSCs	5ug/ml	CFU-GM	HE+PSCs	0ug/ml	-2.7919	0.1422	36	-19.63	<.0001	0.05	-3.0803	-2.5035
BFU-E	HE+PSCs	5ug/ml	CFU-GM	HE+PSCs	1ug/ml	-2.0512	0.1452	36	-14.13	<.0001	0.05	-2.3456	-1.7567
BFU-E	HE+PSCs	5ug/ml	CFU-GM	HE+PSCs	5ug/ml	-1.2397	0.1278	36	-9.70	<.0001	0.05	-1.4989	-0.9806
CFU-GEMM	AoE+PSCs	0ug/ml	CFU-GEMM	AoE+PSCs	1ug/ml	0.2941	0.1214	36	2.42	0.0206	0.05	0.04780	0.5403
CFU-GEMM	AoE+PSCs	0ug/ml	CFU-GEMM	AoE+PSCs	5ug/ml	0.8018	0.1332	36	6.02	<.0001	0.05	0.5318	1.0719
CFU-GEMM	AoE+PSCs	0ug/ml	CFU-GEMM	E11.5AGM	0ug/ml	-0.6454	0.1094	36	-5.90	<.0001	0.05	-0.8672	-0.4235
CFU-GEMM	AoE+PSCs	0ug/ml	CFU-GEMM	E11.5AGM	1ug/ml	-0.3708	0.1119	36	-3.31	0.0021	0.05	-0.5978	-0.1439
CFU-GEMM	AoE+PSCs	0ug/ml	CFU-GEMM	E11.5AGM	5ug/ml	0.4746	0.1251	36	3.79	0.0005	0.05	0.2209	0.7282
CFU-GEMM	AoE+PSCs	0ug/ml	CFU-GEMM	HE+PSCs	0ug/ml	0.02669	0.1169	36	0.23	0.8208	0.05	-0.2105	0.2638
CFU-GEMM	AoE+PSCs	0ug/ml	CFU-GEMM	HE+PSCs	1ug/ml	0.6546	0.1293	36	5.06	<.0001	0.05	0.3924	0.9168
CFU-GEMM	AoE+PSCs	0ug/ml	CFU-GEMM	HE+PSCs	5ug/ml	1.5426	0.1615	36	9.55	<.0001	0.05	1.2152	1.8700
CFU-GEMM	AoE+PSCs	0ug/ml	CFU-GM	AoE+PSCs	0ug/ml	-1.6712	0.06356	36	-26.29	<.0001	0.05	-1.8002	-1.5423
CFU-GEMM	AoE+PSCs	0ug/ml	CFU-GM	AoE+PSCs	1ug/ml	-0.8920	0.1076	36	-8.29	<.0001	0.05	-1.1101	-0.6738
CFU-GEMM	AoE+PSCs	0ug/ml	CFU-GM	AoE+PSCs	5ug/ml	-0.1806	0.1141	36	-1.58	0.1224	0.05	-0.4120	0.05090
CFU-GEMM	AoE+PSCs	0ug/ml	CFU-GM	E11.5AGM	0ug/ml	-2.0875	0.1029	36	-20.28	<.0001	0.05	-2.2963	-1.8787
CFU-GEMM	AoE+PSCs	0ug/ml	CFU-GM	E11.5AGM	1ug/ml	-1.4568	0.1047	36	-13.91	<.0001	0.05	-1.6692	-1.2444
CFU-GEMM	AoE+PSCs	0ug/ml	CFU-GM	E11.5AGM	5ug/ml	-0.7039	0.1089	36	-6.46	<.0001	0.05	-0.9248	-0.4831
CFU-GEMM	AoE+PSCs	0ug/ml	CFU-GM	HE+PSCs	0ug/ml	-1.4758	0.1047	36	-14.10	<.0001	0.05	-1.6880	-1.2635
CFU-GEMM	AoE+PSCs	0ug/ml	CFU-GM	HE+PSCs	1ug/ml	-0.7350	0.1087	36	-6.76	<.0001	0.05	-0.9554	-0.5146
CFU-GEMM	AoE+PSCs	0ug/ml	CFU-GM	HE+PSCs	5ug/ml	0.07639	0.1177	36	0.65	0.5204	0.05	-0.1623	0.3151
CFU-GEMM	AoE+PSCs	1ug/ml	CFU-GEMM	AoE+PSCs	5ug/ml	0.5078	0.1375	36	3.69	0.0007	0.05	0.2290	0.7865

The GLIMMIX Procedure

Differences of Colony*Condit*Treatm Least Squares Means													
Colony_Type	Condition	Treatment	_Colony_Type	_Condition	_Treatment	Estimate	Standard Error	DF	t Value	Pr > |t|	Alpha	Lower	Upper
CFU-GEMM	AoE+PSCs	1ug/ml	CFU-GEMM	E11.5AGM	0ug/ml	-0.9394	0.1146	36	-8.20	<.0001	0.05	-1.1718	-0.7070
CFU-GEMM	AoE+PSCs	1ug/ml	CFU-GEMM	E11.5AGM	1ug/ml	-0.6649	0.1170	36	-5.68	<.0001	0.05	-0.9021	-0.4276
CFU-GEMM	AoE+PSCs	1ug/ml	CFU-GEMM	E11.5AGM	5ug/ml	0.1805	0.1296	36	1.39	0.1723	0.05	-0.08239	0.4435
CFU-GEMM	AoE+PSCs	1ug/ml	CFU-GEMM	HE+PSCs	0ug/ml	-0.2674	0.1218	36	-2.19	0.0347	0.05	-0.5144	-0.02032
CFU-GEMM	AoE+PSCs	1ug/ml	CFU-GEMM	HE+PSCs	1ug/ml	0.3605	0.1337	36	2.70	0.0106	0.05	0.08931	0.6317
CFU-GEMM	AoE+PSCs	1ug/ml	CFU-GEMM	HE+PSCs	5ug/ml	1.2486	0.1650	36	7.57	<.0001	0.05	0.9139	1.5832
CFU-GEMM	AoE+PSCs	1ug/ml	CFU-GM	AoE+PSCs	0ug/ml	-1.9653	0.1095	36	-17.95	<.0001	0.05	-2.1873	-1.7433
CFU-GEMM	AoE+PSCs	1ug/ml	CFU-GM	AoE+PSCs	1ug/ml	-1.1860	0.07721	36	-15.36	<.0001	0.05	-1.3426	-1.0294
CFU-GEMM	AoE+PSCs	1ug/ml	CFU-GM	AoE+PSCs	5ug/ml	-0.4746	0.1191	36	-3.98	0.0003	0.05	-0.7162	-0.2330
CFU-GEMM	AoE+PSCs	1ug/ml	CFU-GM	E11.5AGM	0ug/ml	-2.3816	0.1085	36	-21.96	<.0001	0.05	-2.6015	-2.1616
CFU-GEMM	AoE+PSCs	1ug/ml	CFU-GM	E11.5AGM	1ug/ml	-1.7509	0.1102	36	-15.89	<.0001	0.05	-1.9743	-1.5275
CFU-GEMM	AoE+PSCs	1ug/ml	CFU-GM	E11.5AGM	5ug/ml	-0.9980	0.1141	36	-8.74	<.0001	0.05	-1.2295	-0.7665
CFU-GEMM	AoE+PSCs	1ug/ml	CFU-GM	HE+PSCs	0ug/ml	-1.7698	0.1101	36	-16.08	<.0001	0.05	-1.9931	-1.5466
CFU-GEMM	AoE+PSCs	1ug/ml	CFU-GM	HE+PSCs	1ug/ml	-1.0291	0.1139	36	-9.03	<.0001	0.05	-1.2601	-0.7980
CFU-GEMM	AoE+PSCs	1ug/ml	CFU-GM	HE+PSCs	5ug/ml	-0.2177	0.1225	36	-1.78	0.0841	0.05	-0.4662	0.03086
CFU-GEMM	AoE+PSCs	5ug/ml	CFU-GEMM	E11.5AGM	0ug/ml	-1.4472	0.1269	36	-11.40	<.0001	0.05	-1.7046	-1.1897
CFU-GEMM	AoE+PSCs	5ug/ml	CFU-GEMM	E11.5AGM	1ug/ml	-1.1727	0.1291	36	-9.08	<.0001	0.05	-1.4345	-0.9108
CFU-GEMM	AoE+PSCs	5ug/ml	CFU-GEMM	E11.5AGM	5ug/ml	-0.3272	0.1407	36	-2.33	0.0258	0.05	-0.6126	-0.04191
CFU-GEMM	AoE+PSCs	5ug/ml	CFU-GEMM	HE+PSCs	0ug/ml	-0.7751	0.1335	36	-5.81	<.0001	0.05	-1.0459	-0.5044
CFU-GEMM	AoE+PSCs	5ug/ml	CFU-GEMM	HE+PSCs	1ug/ml	-0.1472	0.1445	36	-1.02	0.3148	0.05	-0.4402	0.1457
CFU-GEMM	AoE+PSCs	5ug/ml	CFU-GEMM	HE+PSCs	5ug/ml	0.7408	0.1738	36	4.26	0.0001	0.05	0.3883	1.0933
CFU-GEMM	AoE+PSCs	5ug/ml	CFU-GM	AoE+PSCs	0ug/ml	-2.4731	0.1223	36	-20.21	<.0001	0.05	-2.7212	-2.2249
CFU-GEMM	AoE+PSCs	5ug/ml	CFU-GM	AoE+PSCs	1ug/ml	-1.6938	0.1254	36	-13.51	<.0001	0.05	-1.9481	-1.4395
CFU-GEMM	AoE+PSCs	5ug/ml	CFU-GM	AoE+PSCs	5ug/ml	-0.9824	0.1017	36	-9.66	<.0001	0.05	-1.1886	-0.7762

The GLIMMIX Procedure

Differences of Colony*Condit*Treatm Least Squares Means

Colony_Type	Condition	Treatment	_Colony_Type	_Condition	_Treatment	Estimate	Standard Error	DF	t Value	Pr > |t|	Alpha	Lower	Upper
CFU-GEMM	AoE+PSCs	5ug/ml	CFU-GM	E11.5AGM	0ug/ml	-2.8893	0.1214	36	-23.79	<.0001	0.05	-3.1357	-2.6430
CFU-GEMM	AoE+PSCs	5ug/ml	CFU-GM	E11.5AGM	1ug/ml	-2.2586	0.1230	36	-18.37	<.0001	0.05	-2.5080	-2.0093
CFU-GEMM	AoE+PSCs	5ug/ml	CFU-GM	E11.5AGM	5ug/ml	-1.5058	0.1265	36	-11.90	<.0001	0.05	-1.7624	-1.2491
CFU-GEMM	AoE+PSCs	5ug/ml	CFU-GM	HE+PSCs	0ug/ml	-2.2776	0.1229	36	-18.53	<.0001	0.05	-2.5269	-2.0283
CFU-GEMM	AoE+PSCs	5ug/ml	CFU-GM	HE+PSCs	1ug/ml	-1.5368	0.1263	36	-12.17	<.0001	0.05	-1.7931	-1.2806
CFU-GEMM	AoE+PSCs	5ug/ml	CFU-GM	HE+PSCs	5ug/ml	-0.7254	0.1341	36	-5.41	<.0001	0.05	-0.9975	-0.4534
CFU-GEMM	E11.5AGM	0ug/ml	CFU-GEMM	E11.5AGM	1ug/ml	0.2745	0.1044	36	2.63	0.0125	0.05	0.06272	0.4863
CFU-GEMM	E11.5AGM	0ug/ml	CFU-GEMM	E11.5AGM	5ug/ml	1.1199	0.1184	36	9.46	<.0001	0.05	0.8797	1.3601
CFU-GEMM	E11.5AGM	0ug/ml	CFU-GEMM	HE+PSCs	0ug/ml	0.6720	0.1098	36	6.12	<.0001	0.05	0.4493	0.8947
CFU-GEMM	E11.5AGM	0ug/ml	CFU-GEMM	HE+PSCs	1ug/ml	1.2999	0.1229	36	10.58	<.0001	0.05	1.0507	1.5492
CFU-GEMM	E11.5AGM	0ug/ml	CFU-GEMM	HE+PSCs	5ug/ml	2.1880	0.1564	36	13.99	<.0001	0.05	1.8708	2.5051
CFU-GEMM	E11.5AGM	0ug/ml	CFU-GM	AoE+PSCs	0ug/ml	-1.0259	0.09592	36	-10.69	<.0001	0.05	-1.2204	-0.8314
CFU-GEMM	E11.5AGM	0ug/ml	CFU-GM	AoE+PSCs	1ug/ml	-0.2466	0.09979	36	-2.47	0.0183	0.05	-0.4490	-0.04423
CFU-GEMM	E11.5AGM	0ug/ml	CFU-GM	AoE+PSCs	5ug/ml	0.4648	0.1068	36	4.35	0.0001	0.05	0.2481	0.6814
CFU-GEMM	E11.5AGM	0ug/ml	CFU-GM	E11.5AGM	0ug/ml	-1.4422	0.04703	36	-30.66	<.0001	0.05	-1.5376	-1.3468
CFU-GEMM	E11.5AGM	0ug/ml	CFU-GM	E11.5AGM	1ug/ml	-0.8115	0.09672	36	-8.39	<.0001	0.05	-1.0076	-0.6153
CFU-GEMM	E11.5AGM	0ug/ml	CFU-GM	E11.5AGM	5ug/ml	-0.05857	0.1012	36	-0.58	0.5664	0.05	-0.2639	0.1467
CFU-GEMM	E11.5AGM	0ug/ml	CFU-GM	HE+PSCs	0ug/ml	-0.8304	0.09664	36	-8.59	<.0001	0.05	-1.0264	-0.6344
CFU-GEMM	E11.5AGM	0ug/ml	CFU-GM	HE+PSCs	1ug/ml	-0.08967	0.1010	36	-0.89	0.3804	0.05	-0.2945	0.1151
CFU-GEMM	E11.5AGM	0ug/ml	CFU-GM	HE+PSCs	5ug/ml	0.7217	0.1106	36	6.52	<.0001	0.05	0.4974	0.9461
CFU-GEMM	E11.5AGM	1ug/ml	CFU-GEMM	E11.5AGM	5ug/ml	0.8454	0.1208	36	7.00	<.0001	0.05	0.6005	1.0904
CFU-GEMM	E11.5AGM	1ug/ml	CFU-GEMM	HE+PSCs	0ug/ml	0.3975	0.1123	36	3.54	0.0011	0.05	0.1697	0.6253
CFU-GEMM	E11.5AGM	1ug/ml	CFU-GEMM	HE+PSCs	1ug/ml	1.0254	0.1251	36	8.19	<.0001	0.05	0.7716	1.2792
CFU-GEMM	E11.5AGM	1ug/ml	CFU-GEMM	HE+PSCs	5ug/ml	1.9134	0.1581	36	12.10	<.0001	0.05	1.5927	2.2342

The GLIMMIX Procedure

Differences of Colony*Condit*Treatm Least Squares Means													
Colony_Type	Condition	Treatment	_Colony_Type	_Condition	_Treatment	Estimate	Standard Error	DF	t Value	Pr > |t|	Alpha	Lower	Upper
CFU-GEMM	E11.5AGM	1ug/ml	CFU-GM	AoE+PSCs	0ug/ml	-1.3004	0.09878	36	-13.16	<.0001	0.05	-1.5007	-1.1001
CFU-GEMM	E11.5AGM	1ug/ml	CFU-GM	AoE+PSCs	1ug/ml	-0.5211	0.1025	36	-5.08	<.0001	0.05	-0.7291	-0.3132
CFU-GEMM	E11.5AGM	1ug/ml	CFU-GM	AoE+PSCs	5ug/ml	0.1903	0.1094	36	1.74	0.0905	0.05	-0.03157	0.4121
CFU-GEMM	E11.5AGM	1ug/ml	CFU-GM	E11.5AGM	0ug/ml	-1.7167	0.09767	36	-17.58	<.0001	0.05	-1.9148	-1.5186
CFU-GEMM	E11.5AGM	1ug/ml	CFU-GM	E11.5AGM	1ug/ml	-1.0860	0.05597	36	-19.40	<.0001	0.05	-1.1995	-0.9725
CFU-GEMM	E11.5AGM	1ug/ml	CFU-GM	E11.5AGM	5ug/ml	-0.3331	0.1039	36	-3.20	0.0028	0.05	-0.5439	-0.1223
CFU-GEMM	E11.5AGM	1ug/ml	CFU-GM	HE+PSCs	0ug/ml	-1.1049	0.09948	36	-11.11	<.0001	0.05	-1.3067	-0.9032
CFU-GEMM	E11.5AGM	1ug/ml	CFU-GM	HE+PSCs	1ug/ml	-0.3642	0.1037	36	-3.51	0.0012	0.05	-0.5745	-0.1539
CFU-GEMM	E11.5AGM	1ug/ml	CFU-GM	HE+PSCs	5ug/ml	0.4472	0.1131	36	3.95	0.0003	0.05	0.2179	0.6766
CFU-GEMM	E11.5AGM	5ug/ml	CFU-GEMM	HE+PSCs	0ug/ml	-0.4479	0.1254	36	-3.57	0.0010	0.05	-0.7023	-0.1935
CFU-GEMM	E11.5AGM	5ug/ml	CFU-GEMM	HE+PSCs	1ug/ml	0.1800	0.1370	36	1.31	0.1974	0.05	-0.09796	0.4579
CFU-GEMM	E11.5AGM	5ug/ml	CFU-GEMM	HE+PSCs	5ug/ml	1.0680	0.1677	36	6.37	<.0001	0.05	0.7279	1.4082
CFU-GEMM	E11.5AGM	5ug/ml	CFU-GM	AoE+PSCs	0ug/ml	-2.1458	0.1135	36	-18.91	<.0001	0.05	-2.3760	-1.9157
CFU-GEMM	E11.5AGM	5ug/ml	CFU-GM	AoE+PSCs	1ug/ml	-1.3665	0.1168	36	-11.70	<.0001	0.05	-1.6034	-1.1297
CFU-GEMM	E11.5AGM	5ug/ml	CFU-GM	AoE+PSCs	5ug/ml	-0.6552	0.1228	36	-5.33	<.0001	0.05	-0.9043	-0.4060
CFU-GEMM	E11.5AGM	5ug/ml	CFU-GM	E11.5AGM	0ug/ml	-2.5621	0.1125	36	-22.77	<.0001	0.05	-2.7903	-2.3339
CFU-GEMM	E11.5AGM	5ug/ml	CFU-GM	E11.5AGM	1ug/ml	-1.9314	0.1142	36	-16.92	<.0001	0.05	-2.1629	-1.6999
CFU-GEMM	E11.5AGM	5ug/ml	CFU-GM	E11.5AGM	5ug/ml	-1.1785	0.08452	36	-13.94	<.0001	0.05	-1.3499	-1.0071
CFU-GEMM	E11.5AGM	5ug/ml	CFU-GM	HE+PSCs	0ug/ml	-1.9504	0.1141	36	-17.09	<.0001	0.05	-2.1818	-1.7190
CFU-GEMM	E11.5AGM	5ug/ml	CFU-GM	HE+PSCs	1ug/ml	-1.2096	0.1178	36	-10.27	<.0001	0.05	-1.4485	-0.9707
CFU-GEMM	E11.5AGM	5ug/ml	CFU-GM	HE+PSCs	5ug/ml	-0.3982	0.1261	36	-3.16	0.0032	0.05	-0.6540	-0.1424
CFU-GEMM	HE+PSCs	0ug/ml	CFU-GEMM	HE+PSCs	1ug/ml	0.6279	0.1297	36	4.84	<.0001	0.05	0.3649	0.8909
CFU-GEMM	HE+PSCs	0ug/ml	CFU-GEMM	HE+PSCs	5ug/ml	1.5159	0.1617	36	9.37	<.0001	0.05	1.1879	1.8440
CFU-GEMM	HE+PSCs	0ug/ml	CFU-GM	AoE+PSCs	0ug/ml	-1.6979	0.1045	36	-16.26	<.0001	0.05	-1.9098	-1.4861

The GLIMMIX Procedure

Differences of Colony*Condit*Treatm Least Squares Means													
Colony_Type	Condition	Treatment	_Colony_Type	_Condition	_Treatment	Estimate	Standard Error	DF	t Value	Pr > |t|	Alpha	Lower	Upper
CFU-GEMM	HE+PSCs	0ug/ml	CFU-GM	AoE+PSCs	1ug/ml	-0.9186	0.1080	36	-8.50	<.0001	0.05	-1.1377	-0.6996
CFU-GEMM	HE+PSCs	0ug/ml	CFU-GM	AoE+PSCs	5ug/ml	-0.2072	0.1145	36	-1.81	0.0788	0.05	-0.4396	0.02506
CFU-GEMM	HE+PSCs	0ug/ml	CFU-GM	E11.5AGM	0ug/ml	-2.1142	0.1034	36	-20.45	<.0001	0.05	-2.3239	-1.9045
CFU-GEMM	HE+PSCs	0ug/ml	CFU-GM	E11.5AGM	1ug/ml	-1.4835	0.1052	36	-14.10	<.0001	0.05	-1.6968	-1.2702
CFU-GEMM	HE+PSCs	0ug/ml	CFU-GM	E11.5AGM	5ug/ml	-0.7306	0.1093	36	-6.68	<.0001	0.05	-0.9524	-0.5089
CFU-GEMM	HE+PSCs	0ug/ml	CFU-GM	HE+PSCs	0ug/ml	-1.5025	0.06538	36	-22.98	<.0001	0.05	-1.6351	-1.3699
CFU-GEMM	HE+PSCs	0ug/ml	CFU-GM	HE+PSCs	1ug/ml	-0.7617	0.1091	36	-6.98	<.0001	0.05	-0.9830	-0.5404
CFU-GEMM	HE+PSCs	0ug/ml	CFU-GM	HE+PSCs	5ug/ml	0.04971	0.1181	36	0.42	0.6763	0.05	-0.1898	0.2892
CFU-GEMM	HE+PSCs	1ug/ml	CFU-GEMM	HE+PSCs	5ug/ml	0.8880	0.1709	36	5.20	<.0001	0.05	0.5414	1.2346
CFU-GEMM	HE+PSCs	1ug/ml	CFU-GM	AoE+PSCs	0ug/ml	-2.3258	0.1181	36	-19.69	<.0001	0.05	-2.5654	-2.0862
CFU-GEMM	HE+PSCs	1ug/ml	CFU-GM	AoE+PSCs	1ug/ml	-1.5465	0.1213	36	-12.75	<.0001	0.05	-1.7925	-1.3005
CFU-GEMM	HE+PSCs	1ug/ml	CFU-GM	AoE+PSCs	5ug/ml	-0.8351	0.1271	36	-6.57	<.0001	0.05	-1.0930	-0.5773
CFU-GEMM	HE+PSCs	1ug/ml	CFU-GM	E11.5AGM	0ug/ml	-2.7421	0.1172	36	-23.40	<.0001	0.05	-2.9798	-2.5044
CFU-GEMM	HE+PSCs	1ug/ml	CFU-GM	E11.5AGM	1ug/ml	-2.1114	0.1188	36	-17.78	<.0001	0.05	-2.3523	-1.8705
CFU-GEMM	HE+PSCs	1ug/ml	CFU-GM	E11.5AGM	5ug/ml	-1.3585	0.1225	36	-11.09	<.0001	0.05	-1.6069	-1.1101
CFU-GEMM	HE+PSCs	1ug/ml	CFU-GM	HE+PSCs	0ug/ml	-2.1304	0.1187	36	-17.94	<.0001	0.05	-2.3711	-1.8896
CFU-GEMM	HE+PSCs	1ug/ml	CFU-GM	HE+PSCs	1ug/ml	-1.3896	0.09035	36	-15.38	<.0001	0.05	-1.5728	-1.2064
CFU-GEMM	HE+PSCs	1ug/ml	CFU-GM	HE+PSCs	5ug/ml	-0.5782	0.1303	36	-4.44	<.0001	0.05	-0.8425	-0.3138
CFU-GEMM	HE+PSCs	5ug/ml	CFU-GM	AoE+PSCs	0ug/ml	-3.2138	0.1527	36	-21.05	<.0001	0.05	-3.5235	-2.9042
CFU-GEMM	HE+PSCs	5ug/ml	CFU-GM	AoE+PSCs	1ug/ml	-2.4346	0.1551	36	-15.69	<.0001	0.05	-2.7492	-2.1200
CFU-GEMM	HE+PSCs	5ug/ml	CFU-GM	AoE+PSCs	5ug/ml	-1.7232	0.1597	36	-10.79	<.0001	0.05	-2.0471	-1.3992
CFU-GEMM	HE+PSCs	5ug/ml	CFU-GM	E11.5AGM	0ug/ml	-3.6301	0.1519	36	-23.89	<.0001	0.05	-3.9383	-3.3220
CFU-GEMM	HE+PSCs	5ug/ml	CFU-GM	E11.5AGM	1ug/ml	-2.9994	0.1532	36	-19.58	<.0001	0.05	-3.3100	-2.6888
CFU-GEMM	HE+PSCs	5ug/ml	CFU-GM	E11.5AGM	5ug/ml	-2.2465	0.1560	36	-14.40	<.0001	0.05	-2.5630	-1.9301

The GLIMMIX Procedure

Differences of Colony*Condit*Treatm Least Squares Means													
Colony_Type	Condition	Treatment	_Colony_Type	_Condition	_Treatment	Estimate	Standard Error	DF	t Value	Pr > |t|	Alpha	Lower	Upper
CFU-GEMM	HE+PSCs	5ug/ml	CFU-GM	HE+PSCs	0ug/ml	-3.0184	0.1531	36	-19.71	<.0001	0.05	-3.3289	-2.7078
CFU-GEMM	HE+PSCs	5ug/ml	CFU-GM	HE+PSCs	1ug/ml	-2.2776	0.1559	36	-14.61	<.0001	0.05	-2.5938	-1.9615
CFU-GEMM	HE+PSCs	5ug/ml	CFU-GM	HE+PSCs	5ug/ml	-1.4662	0.1398	36	-10.49	<.0001	0.05	-1.7498	-1.1826
CFU-GM	AoE+PSCs	0ug/ml	CFU-GM	AoE+PSCs	1ug/ml	0.7793	0.09386	36	8.30	<.0001	0.05	0.5889	0.9697
CFU-GM	AoE+PSCs	0ug/ml	CFU-GM	AoE+PSCs	5ug/ml	1.4907	0.1013	36	14.71	<.0001	0.05	1.2852	1.6961
CFU-GM	AoE+PSCs	0ug/ml	CFU-GM	E11.5AGM	0ug/ml	-0.4163	0.08852	36	-4.70	<.0001	0.05	-0.5958	-0.2368
CFU-GM	AoE+PSCs	0ug/ml	CFU-GM	E11.5AGM	1ug/ml	0.2144	0.09059	36	2.37	0.0234	0.05	0.03070	0.3982
CFU-GM	AoE+PSCs	0ug/ml	CFU-GM	E11.5AGM	5ug/ml	0.9673	0.09539	36	10.14	<.0001	0.05	0.7739	1.1608
CFU-GM	AoE+PSCs	0ug/ml	CFU-GM	HE+PSCs	0ug/ml	0.1955	0.09051	36	2.16	0.0375	0.05	0.01190	0.3790
CFU-GM	AoE+PSCs	0ug/ml	CFU-GM	HE+PSCs	1ug/ml	0.9362	0.09513	36	9.84	<.0001	0.05	0.7433	1.1291
CFU-GM	AoE+PSCs	0ug/ml	CFU-GM	HE+PSCs	5ug/ml	1.7476	0.1053	36	16.60	<.0001	0.05	1.5341	1.9612
CFU-GM	AoE+PSCs	1ug/ml	CFU-GM	AoE+PSCs	5ug/ml	0.7114	0.1050	36	6.78	<.0001	0.05	0.4985	0.9243
CFU-GM	AoE+PSCs	1ug/ml	CFU-GM	E11.5AGM	0ug/ml	-1.1956	0.09269	36	-12.90	<.0001	0.05	-1.3835	-1.0076
CFU-GM	AoE+PSCs	1ug/ml	CFU-GM	E11.5AGM	1ug/ml	-0.5649	0.09467	36	-5.97	<.0001	0.05	-0.7569	-0.3729
CFU-GM	AoE+PSCs	1ug/ml	CFU-GM	E11.5AGM	5ug/ml	0.1880	0.09927	36	1.89	0.0663	0.05	-0.01331	0.3894
CFU-GM	AoE+PSCs	1ug/ml	CFU-GM	HE+PSCs	0ug/ml	-0.5838	0.09460	36	-6.17	<.0001	0.05	-0.7757	-0.3920
CFU-GM	AoE+PSCs	1ug/ml	CFU-GM	HE+PSCs	1ug/ml	0.1569	0.09902	36	1.58	0.1217	0.05	-0.04389	0.3578
CFU-GM	AoE+PSCs	1ug/ml	CFU-GM	HE+PSCs	5ug/ml	0.9683	0.1088	36	8.90	<.0001	0.05	0.7476	1.1891
CFU-GM	AoE+PSCs	5ug/ml	CFU-GM	E11.5AGM	0ug/ml	-1.9070	0.1002	36	-19.03	<.0001	0.05	-2.1102	-1.7037
CFU-GM	AoE+PSCs	5ug/ml	CFU-GM	E11.5AGM	1ug/ml	-1.2763	0.1021	36	-12.51	<.0001	0.05	-1.4832	-1.0693
CFU-GM	AoE+PSCs	5ug/ml	CFU-GM	E11.5AGM	5ug/ml	-0.5234	0.1063	36	-4.92	<.0001	0.05	-0.7390	-0.3077
CFU-GM	AoE+PSCs	5ug/ml	CFU-GM	HE+PSCs	0ug/ml	-1.2952	0.1020	36	-12.70	<.0001	0.05	-1.5021	-1.0884
CFU-GM	AoE+PSCs	5ug/ml	CFU-GM	HE+PSCs	1ug/ml	-0.5545	0.1061	36	-5.23	<.0001	0.05	-0.7696	-0.3393
CFU-GM	AoE+PSCs	5ug/ml	CFU-GM	HE+PSCs	5ug/ml	0.2570	0.1153	36	2.23	0.0322	0.05	0.02314	0.4908

The GLIMMIX Procedure

Differences of Colony*Condit*Treatm Least Squares Means													
Colony_Type	Condition	Treatment	_Colony_Type	_Condition	_Treatment	Estimate	Standard Error	DF	t Value	Pr > |t|	Alpha	Lower	Upper
CFU-GM	E11.5AGM	0ug/ml	CFU-GM	E11.5AGM	1ug/ml	0.6307	0.08937	36	7.06	<.0001	0.05	0.4494	0.8120
CFU-GM	E11.5AGM	0ug/ml	CFU-GM	E11.5AGM	5ug/ml	1.3836	0.09424	36	14.68	<.0001	0.05	1.1925	1.5747
CFU-GM	E11.5AGM	0ug/ml	CFU-GM	HE+PSCs	0ug/ml	0.6117	0.08929	36	6.85	<.0001	0.05	0.4306	0.7928
CFU-GM	E11.5AGM	0ug/ml	CFU-GM	HE+PSCs	1ug/ml	1.3525	0.09397	36	14.39	<.0001	0.05	1.1619	1.5431
CFU-GM	E11.5AGM	0ug/ml	CFU-GM	HE+PSCs	5ug/ml	2.1639	0.1043	36	20.76	<.0001	0.05	1.9525	2.3754
CFU-GM	E11.5AGM	1ug/ml	CFU-GM	E11.5AGM	5ug/ml	0.7529	0.09619	36	7.83	<.0001	0.05	0.5578	0.9480
CFU-GM	E11.5AGM	1ug/ml	CFU-GM	HE+PSCs	0ug/ml	-0.01896	0.09135	36	-0.21	0.8367	0.05	-0.2042	0.1663
CFU-GM	E11.5AGM	1ug/ml	CFU-GM	HE+PSCs	1ug/ml	0.7218	0.09592	36	7.52	<.0001	0.05	0.5272	0.9163
CFU-GM	E11.5AGM	1ug/ml	CFU-GM	HE+PSCs	5ug/ml	1.5332	0.1060	36	14.46	<.0001	0.05	1.3182	1.7482
CFU-GM	E11.5AGM	5ug/ml	CFU-GM	HE+PSCs	0ug/ml	-0.7719	0.09611	36	-8.03	<.0001	0.05	-0.9668	-0.5769
CFU-GM	E11.5AGM	5ug/ml	CFU-GM	HE+PSCs	1ug/ml	-0.03110	0.1005	36	-0.31	0.7587	0.05	-0.2349	0.1727
CFU-GM	E11.5AGM	5ug/ml	CFU-GM	HE+PSCs	5ug/ml	0.7803	0.1101	36	7.08	<.0001	0.05	0.5569	1.0037
CFU-GM	HE+PSCs	0ug/ml	CFU-GM	HE+PSCs	1ug/ml	0.7408	0.09585	36	7.73	<.0001	0.05	0.5464	0.9352
CFU-GM	HE+PSCs	0ug/ml	CFU-GM	HE+PSCs	5ug/ml	1.5522	0.1060	36	14.65	<.0001	0.05	1.3373	1.7671
CFU-GM	HE+PSCs	1ug/ml	CFU-GM	HE+PSCs	5ug/ml	0.8114	0.1099	36	7.38	<.0001	0.05	0.5885	1.0343

The GENMOD Procedure

Model Information	
**Data Set**	WORK.EXT_FIG10BIII_E11USAG_TRANS
**Distribution**	Normal
**Link Function**	Identity
**Dependent Variable**	Percentage

**Number of Observations Read**	45
**Number of Observations Used**	45

Class Level Information		
**Class**	**Levels**	**Values**
**Condition**	3	AoE+PSCs E11.5AGM HE+PSCs
**Treatment**	3	0ug/ml 1ug/ml 5ug/ml

Parameter Information			
**Parameter**	**Effect**	**Condition**	**Treatment**
**Prm1**	Intercept		
**Prm2**	Condition	AoE+PSCs	
**Prm3**	Condition	E11.5AGM	
**Prm4**	Condition	HE+PSCs	
**Prm5**	Treatment		0ug/ml
**Prm6**	Treatment		1ug/ml
**Prm7**	Treatment		5ug/ml
**Prm8**	Condition*Treatment	AoE+PSCs	0ug/ml
**Prm9**	Condition*Treatment	AoE+PSCs	1ug/ml
**Prm10**	Condition*Treatment	AoE+PSCs	5ug/ml
**Prm11**	Condition*Treatment	E11.5AGM	0ug/ml

The GENMOD Procedure

Parameter Information			
**Parameter**	**Effect**	**Condition**	**Treatment**
**Prm12**	Condition*Treatment	E11.5AGM	1ug/ml
**Prm13**	Condition*Treatment	E11.5AGM	5ug/ml
**Prm14**	Condition*Treatment	HE+PSCs	0ug/ml
**Prm15**	Condition*Treatment	HE+PSCs	1ug/ml
**Prm16**	Condition*Treatment	HE+PSCs	5ug/ml

Criteria For Assessing Goodness Of Fit			
**Criterion**	**DF**	**Value**	**Value/DF**
**Deviance**	36	726.0000	20.1667
**Scaled Deviance**	36	45.0000	1.2500
**Pearson Chi-Square**	36	726.0000	20.1667
**Scaled Pearson X2**	36	45.0000	1.2500
**Log Likelihood**		-126.4222	
**Full Log Likelihood**		-126.4222	
**AIC (smaller is better)**		272.8444	
**AICC (smaller is better)**		279.3150	
**BIC (smaller is better)**		290.9110	

Algorithm converged.

The GENMOD Procedure

Analysis Of Maximum Likelihood Parameter Estimates									
Parameter			DF	Estimate	Standard Error	Wald 95% Confidence Limits		Wald Chi-Square	Pr > ChiSq
Intercept			1	5.8000	1.7963	2.2793	9.3207	10.43	0.0012
Condition	AoE+PSCs		1	4.2000	2.5403	-0.7790	9.1790	2.73	0.0983
Condition	E11.5AGM		1	17.6000	2.5403	12.6210	22.5790	48.00	<.0001
Condition	HE+PSCs		0	0.0000	0.0000	0.0000	0.0000	.	.
Treatment	0ug/ml		1	14.6000	2.5403	9.6210	19.5790	33.03	<.0001
Treatment	1ug/ml		1	4.6000	2.5403	-0.3790	9.5790	3.28	0.0702
Treatment	5ug/ml		0	0.0000	0.0000	0.0000	0.0000	.	.
Condition*Treatment	AoE+PSCs	0ug/ml	1	4.4000	3.5926	-2.6413	11.4413	1.50	0.2207
Condition*Treatment	AoE+PSCs	1ug/ml	1	1.0000	3.5926	-6.0413	8.0413	0.08	0.7807
Condition*Treatment	AoE+PSCs	5ug/ml	0	0.0000	0.0000	0.0000	0.0000	.	.
Condition*Treatment	E11.5AGM	0ug/ml	1	20.6000	3.5926	13.5587	27.6413	32.88	<.0001
Condition*Treatment	E11.5AGM	1ug/ml	1	12.6000	3.5926	5.5587	19.6413	12.30	0.0005
Condition*Treatment	E11.5AGM	5ug/ml	0	0.0000	0.0000	0.0000	0.0000	.	.
Condition*Treatment	HE+PSCs	0ug/ml	0	0.0000	0.0000	0.0000	0.0000	.	.
Condition*Treatment	HE+PSCs	1ug/ml	0	0.0000	0.0000	0.0000	0.0000	.	.
Condition*Treatment	HE+PSCs	5ug/ml	0	0.0000	0.0000	0.0000	0.0000	.	.
Scale			1	4.0166	0.4234	3.2669	4.9384		

Note: The scale parameter was estimated by maximum likelihood.

The GENMOD Procedure

Condition*Treatment Least Squares Means								
Condition	Treatment	Estimate	Standard Error	z Value	Pr > |z|	Alpha	Lower	Upper
AoE+PSCs	0ug/ml	29.0000	1.7963	16.14	<.0001	0.05	25.4793	32.5207
AoE+PSCs	1ug/ml	15.6000	1.7963	8.68	<.0001	0.05	12.0793	19.1207
AoE+PSCs	5ug/ml	10.0000	1.7963	5.57	<.0001	0.05	6.4793	13.5207
E11.5AGM	0ug/ml	58.6000	1.7963	32.62	<.0001	0.05	55.0793	62.1207
E11.5AGM	1ug/ml	40.6000	1.7963	22.60	<.0001	0.05	37.0793	44.1207
E11.5AGM	5ug/ml	23.4000	1.7963	13.03	<.0001	0.05	19.8793	26.9207
HE+PSCs	0ug/ml	20.4000	1.7963	11.36	<.0001	0.05	16.8793	23.9207
HE+PSCs	1ug/ml	10.4000	1.7963	5.79	<.0001	0.05	6.8793	13.9207
HE+PSCs	5ug/ml	5.8000	1.7963	3.23	0.0012	0.05	2.2793	9.3207

Differences of Condition*Treatment Least Squares Means										
Condition	Treatment	_Condition	_Treatment	Estimate	Standard Error	z Value	Pr > |z|	Alpha	Lower	Upper
AoE+PSCs	0ug/ml	AoE+PSCs	1ug/ml	13.4000	2.5403	5.27	<.0001	0.05	8.4210	18.3790
AoE+PSCs	0ug/ml	AoE+PSCs	5ug/ml	19.0000	2.5403	7.48	<.0001	0.05	14.0210	23.9790
AoE+PSCs	0ug/ml	E11.5AGM	0ug/ml	-29.6000	2.5403	-11.65	<.0001	0.05	-34.5790	-24.6210
AoE+PSCs	0ug/ml	E11.5AGM	1ug/ml	-11.6000	2.5403	-4.57	<.0001	0.05	-16.5790	-6.6210
AoE+PSCs	0ug/ml	E11.5AGM	5ug/ml	5.6000	2.5403	2.20	0.0275	0.05	0.6210	10.5790
AoE+PSCs	0ug/ml	HE+PSCs	0ug/ml	8.6000	2.5403	3.39	0.0007	0.05	3.6210	13.5790
AoE+PSCs	0ug/ml	HE+PSCs	1ug/ml	18.6000	2.5403	7.32	<.0001	0.05	13.6210	23.5790
AoE+PSCs	0ug/ml	HE+PSCs	5ug/ml	23.2000	2.5403	9.13	<.0001	0.05	18.2210	28.1790
AoE+PSCs	1ug/ml	AoE+PSCs	5ug/ml	5.6000	2.5403	2.20	0.0275	0.05	0.6210	10.5790
AoE+PSCs	1ug/ml	E11.5AGM	0ug/ml	-43.0000	2.5403	-16.93	<.0001	0.05	-47.9790	-38.0210
AoE+PSCs	1ug/ml	E11.5AGM	1ug/ml	-25.0000	2.5403	-9.84	<.0001	0.05	-29.9790	-20.0210
AoE+PSCs	1ug/ml	E11.5AGM	5ug/ml	-7.8000	2.5403	-3.07	0.0021	0.05	-12.7790	-2.8210

The GENMOD Procedure

Differences of Condition*Treatment Least Squares Means										
Condition	Treatment	_Condition	_Treatment	Estimate	Standard Error	z Value	Pr > |z|	Alpha	Lower	Upper
AoE+PSCs	1ug/ml	HE+PSCs	0ug/ml	-4.8000	2.5403	-1.89	0.0588	0.05	-9.7790	0.1790
AoE+PSCs	1ug/ml	HE+PSCs	1ug/ml	5.2000	2.5403	2.05	0.0407	0.05	0.2210	10.1790
AoE+PSCs	1ug/ml	HE+PSCs	5ug/ml	9.8000	2.5403	3.86	0.0001	0.05	4.8210	14.7790
AoE+PSCs	5ug/ml	E11.5AGM	0ug/ml	-48.6000	2.5403	-19.13	<.0001	0.05	-53.5790	-43.6210
AoE+PSCs	5ug/ml	E11.5AGM	1ug/ml	-30.6000	2.5403	-12.05	<.0001	0.05	-35.5790	-25.6210
AoE+PSCs	5ug/ml	E11.5AGM	5ug/ml	-13.4000	2.5403	-5.27	<.0001	0.05	-18.3790	-8.4210
AoE+PSCs	5ug/ml	HE+PSCs	0ug/ml	-10.4000	2.5403	-4.09	<.0001	0.05	-15.3790	-5.4210
AoE+PSCs	5ug/ml	HE+PSCs	1ug/ml	-0.4000	2.5403	-0.16	0.8749	0.05	-5.3790	4.5790
AoE+PSCs	5ug/ml	HE+PSCs	5ug/ml	4.2000	2.5403	1.65	0.0983	0.05	-0.7790	9.1790
E11.5AGM	0ug/ml	E11.5AGM	1ug/ml	18.0000	2.5403	7.09	<.0001	0.05	13.0210	22.9790
E11.5AGM	0ug/ml	E11.5AGM	5ug/ml	35.2000	2.5403	13.86	<.0001	0.05	30.2210	40.1790
E11.5AGM	0ug/ml	HE+PSCs	0ug/ml	38.2000	2.5403	15.04	<.0001	0.05	33.2210	43.1790
E11.5AGM	0ug/ml	HE+PSCs	1ug/ml	48.2000	2.5403	18.97	<.0001	0.05	43.2210	53.1790
E11.5AGM	0ug/ml	HE+PSCs	5ug/ml	52.8000	2.5403	20.78	<.0001	0.05	47.8210	57.7790
E11.5AGM	1ug/ml	E11.5AGM	5ug/ml	17.2000	2.5403	6.77	<.0001	0.05	12.2210	22.1790
E11.5AGM	1ug/ml	HE+PSCs	0ug/ml	20.2000	2.5403	7.95	<.0001	0.05	15.2210	25.1790
E11.5AGM	1ug/ml	HE+PSCs	1ug/ml	30.2000	2.5403	11.89	<.0001	0.05	25.2210	35.1790
E11.5AGM	1ug/ml	HE+PSCs	5ug/ml	34.8000	2.5403	13.70	<.0001	0.05	29.8210	39.7790
E11.5AGM	5ug/ml	HE+PSCs	0ug/ml	3.0000	2.5403	1.18	0.2376	0.05	-1.9790	7.9790
E11.5AGM	5ug/ml	HE+PSCs	1ug/ml	13.0000	2.5403	5.12	<.0001	0.05	8.0210	17.9790
E11.5AGM	5ug/ml	HE+PSCs	5ug/ml	17.6000	2.5403	6.93	<.0001	0.05	12.6210	22.5790
HE+PSCs	0ug/ml	HE+PSCs	1ug/ml	10.0000	2.5403	3.94	<.0001	0.05	5.0210	14.9790
HE+PSCs	0ug/ml	HE+PSCs	5ug/ml	14.6000	2.5403	5.75	<.0001	0.05	9.6210	19.5790
HE+PSCs	1ug/ml	HE+PSCs	5ug/ml	4.6000	2.5403	1.81	0.0702	0.05	-0.3790	9.5790

The GENMOD Procedure

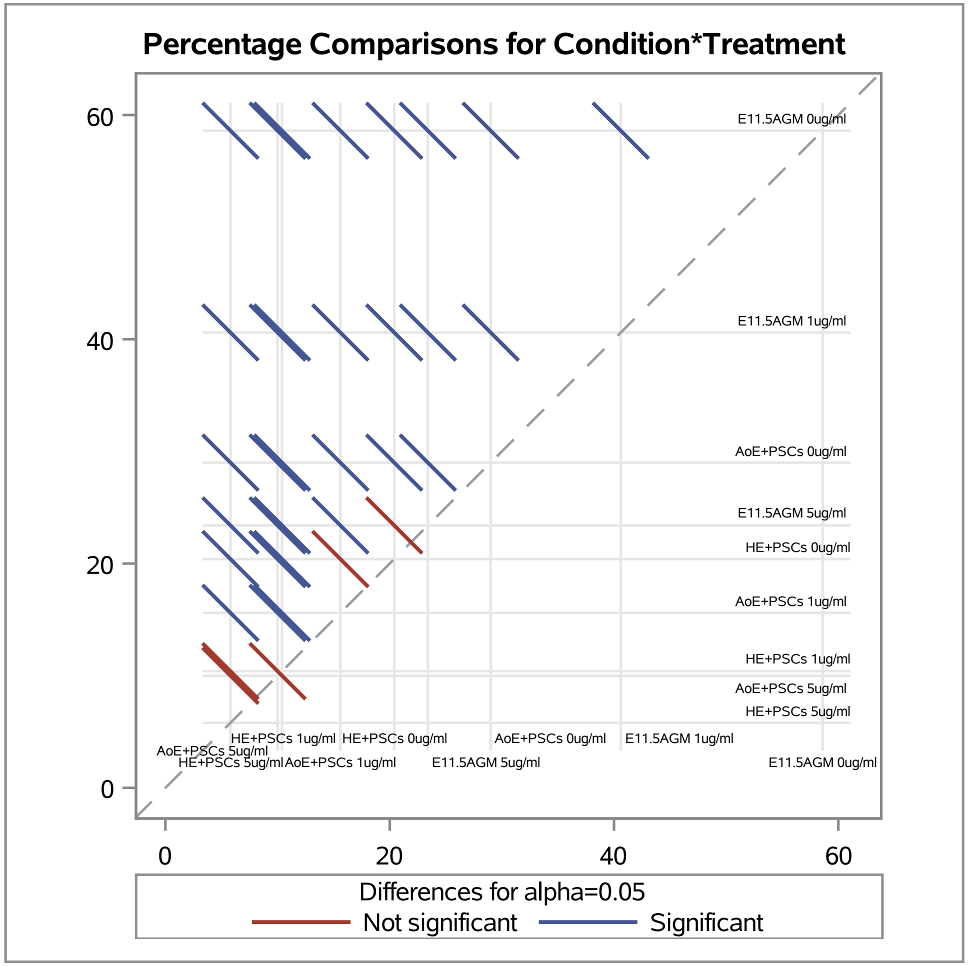

The GLIMMIX Procedure

Model Information	
Data Set	WORK.EXT_FIG10CII
Response Variable	Count
Response Distribution	Poisson
Link Function	Log
Variance Function	Default
Variance Matrix	Diagonal
Estimation Technique	Maximum Likelihood
Degrees of Freedom Method	Residual

Class Level Information		
Class	Levels	Values
Colony_Type	3	BFU-E CFU-GEMM CFU-GM
Condition	6	AoE+E10PSC AoE+E11PSC E10.5AGM E11.5AGM HE+E10PSC HE+E11PSC
Treatment	2	No Inhi With Inhi
Dish_ID	36	1 2 3 4 5 6 7 8 9 10 11 12 13 14 15 16 17 18 19 20 21 22 23 24 25 26 27 28 29 30 31 32 33 34 35 36

Number of Observations Read	108
Number of Observations Used	108

Dimensions	
Columns in X	84
Columns in Z	0
Subjects (Blocks in V)	1
Max Obs per Subject	108

The GLIMMIX Procedure

Optimization Information	
**Optimization Technique**	Newton-Raphson
**Parameters in Optimization**	36
**Lower Boundaries**	0
**Upper Boundaries**	0
**Fixed Effects**	Not Profiled

Iteration History

Iteration	Restarts	Evaluations	Objective Function	Change	Max Gradient
0	0	4	292.76599915	.	99.56982
1	0	3	286.10581166	6.66018749	4.169808
2	0	3	283.92417198	2.18163967	1.269717
3	0	3	283.1216308	0.80254118	0.46706
4	0	3	282.82639178	0.29523903	0.171822
5	0	3	282.71777935	0.10861243	0.06321
6	0	3	282.67782306	0.03995629	0.023254
7	0	3	282.66312396	0.01469910	0.008555
8	0	3	282.65771647	0.00540750	0.003147
9	0	3	282.65572716	0.00198931	0.001158
10	0	3	282.65499533	0.00073183	0.000426
11	0	3	282.65472611	0.00026922	0.000157
12	0	3	282.65462707	0.00009904	0.000058
13	0	3	282.65459063	0.00003644	0.000021
14	0	3	282.65457723	0.00001340	7.801E-6

Convergence criterion (ABSGCONV=0.00001) satisfied.

The GLIMMIX Procedure

Fit Statistics	
-2 Log Likelihood	565.31
AIC (smaller is better)	637.31
AICC (smaller is better)	674.83
BIC (smaller is better)	733.87
CAIC (smaller is better)	769.87
HQIC (smaller is better)	676.46
Pearson Chi-Square	178.50
Pearson Chi-Square / DF	1.65

The GLIMMIX Procedure

Parameter Estimates								
**Effect**	**Colony_Type**	**Condition**	**Treatment**	**Estimate**	**Standard Error**	**DF**	**t Value**	**Pr > |t|**
**Intercept**				-53.7142	2556.86	72	-0.02	0.9833
**Colony_Type**	BFU-E			38.0212	2952.37	72	0.01	0.9898
**Colony_Type**	CFU-GEMM			38.0212	2087.71	72	0.02	0.9855
**Colony_Type**	CFU-GM			0	.	.	.	.
**Condition**		AoE+E10PSC		38.0211	2952.40	72	0.01	0.9898
**Condition**		AoE+E11PSC		38.0211	2952.40	72	0.01	0.9898
**Condition**		E10.5AGM		38.0211	2952.41	72	0.01	0.9898
**Condition**		E11.5AGM		38.0211	2952.41	72	0.01	0.9898
**Condition**		HE+E10PSC		38.0211	2087.68	72	0.02	0.9855
**Condition**		HE+E11PSC		0	.	.	.	.
**Colony_Typ*Condition**	BFU-E	AoE+E10PSC		-38.0213	3615.95	72	-0.01	0.9916
**Colony_Typ*Condition**	BFU-E	AoE+E11PSC		-38.0213	3615.94	72	-0.01	0.9916
**Colony_Typ*Condition**	BFU-E	E10.5AGM		-38.0213	3615.94	72	-0.01	0.9916
**Colony_Typ*Condition**	BFU-E	E11.5AGM		-38.0213	3615.95	72	-0.01	0.9916
**Colony_Typ*Condition**	BFU-E	HE+E10PSC		-38.0213	2952.42	72	-0.01	0.9898
**Colony_Typ*Condition**	BFU-E	HE+E11PSC		0	.	.	.	.
**Colony_Typ*Condition**	CFU-GEMM	AoE+E10PSC		-38.0213	2952.47	72	-0.01	0.9898
**Colony_Typ*Condition**	CFU-GEMM	AoE+E11PSC		-38.0213	2952.47	72	-0.01	0.9898
**Colony_Typ*Condition**	CFU-GEMM	E10.5AGM		-38.0213	2952.47	72	-0.01	0.9898
**Colony_Typ*Condition**	CFU-GEMM	E11.5AGM		-38.0213	2952.47	72	-0.01	0.9898
**Colony_Typ*Condition**	CFU-GEMM	HE+E10PSC		-38.0213	0	72	-Infty	<.0001
**Colony_Typ*Condition**	CFU-GEMM	HE+E11PSC		0	.	.	.	.
**Colony_Typ*Condition**	CFU-GM	AoE+E10PSC		0	.	.	.	.
**Colony_Typ*Condition**	CFU-GM	AoE+E11PSC		0	.	.	.	.

The GLIMMIX Procedure

Parameter Estimates								
**Effect**	**Colony_Type**	**Condition**	**Treatment**	**Estimate**	**Standard Error**	**DF**	**t Value**	**Pr > |t|**
**Colony_Typ*Condition**	CFU-GM	E10.5AGM		0	.	.	.	.
**Colony_Typ*Condition**	CFU-GM	E11.5AGM		0	.	.	.	.
**Colony_Typ*Condition**	CFU-GM	HE+E10PSC		0	.	.	.	.
**Colony_Typ*Condition**	CFU-GM	HE+E11PSC		0	.	.	.	.
**Treatment**			No Inhi	59.7717	2556.86	72	0.02	0.9814
**Treatment**			With Inhi	0	.	.	.	.
**Colony_Typ*Treatment**	BFU-E		No Inhi	-39.6014	2952.37	72	-0.01	0.9893
**Colony_Typ*Treatment**	BFU-E		With Inhi	0	.	.	.	.
**Colony_Typ*Treatment**	CFU-GEMM		No Inhi	-39.5284	2087.71	72	-0.02	0.9849
**Colony_Typ*Treatment**	CFU-GEMM		With Inhi	0	.	.	.	.
**Colony_Typ*Treatment**	CFU-GM		No Inhi	0	.	.	.	.
**Colony_Typ*Treatment**	CFU-GM		With Inhi	0	.	.	.	.
**Condition*Treatment**		AoE+E10PSC	No Inhi	-37.8802	2952.40	72	-0.01	0.9898
**Condition*Treatment**		AoE+E10PSC	With Inhi	0	.	.	.	.
**Condition*Treatment**		AoE+E11PSC	No Inhi	-37.8236	2952.40	72	-0.01	0.9898
**Condition*Treatment**		AoE+E11PSC	With Inhi	0	.	.	.	.
**Condition*Treatment**		E10.5AGM	No Inhi	-37.4371	2952.41	72	-0.01	0.9899
**Condition*Treatment**		E10.5AGM	With Inhi	0	.	.	.	.
**Condition*Treatment**		E11.5AGM	No Inhi	-37.4092	2952.41	72	-0.01	0.9899
**Condition*Treatment**		E11.5AGM	With Inhi	0	.	.	.	.
**Condition*Treatment**		HE+E10PSC	No Inhi	-37.9694	2087.68	72	-0.02	0.9855
**Condition*Treatment**		HE+E10PSC	With Inhi	0	.	.	.	.
**Condition*Treatment**		HE+E11PSC	No Inhi	0	.	.	.	.
**Condition*Treatment**		HE+E11PSC	With Inhi	0	.	.	.	.

The GLIMMIX Procedure

Parameter Estimates								
**Effect**	**Colony_Type**	**Condition**	**Treatment**	**Estimate**	**Standard Error**	**DF**	**t Value**	**Pr > |t|**
**Colony*Condit*Treatm**	BFU-E	AoE+E10PSC	No Inhi	38.1451	3615.95	72	0.01	0.9916
**Colony*Condit*Treatm**	BFU-E	AoE+E10PSC	With Inhi	0	.	.	.	.
**Colony*Condit*Treatm**	BFU-E	AoE+E11PSC	No Inhi	37.8388	3615.94	72	0.01	0.9917
**Colony*Condit*Treatm**	BFU-E	AoE+E11PSC	With Inhi	0	.	.	.	.
**Colony*Condit*Treatm**	BFU-E	E10.5AGM	No Inhi	38.2482	3615.94	72	0.01	0.9916
**Colony*Condit*Treatm**	BFU-E	E10.5AGM	With Inhi	0	.	.	.	.
**Colony*Condit*Treatm**	BFU-E	E11.5AGM	No Inhi	38.2807	3615.95	72	0.01	0.9916
**Colony*Condit*Treatm**	BFU-E	E11.5AGM	With Inhi	0	.	.	.	.
**Colony*Condit*Treatm**	BFU-E	HE+E10PSC	No Inhi	38.0566	2952.42	72	0.01	0.9898
**Colony*Condit*Treatm**	BFU-E	HE+E10PSC	With Inhi	0	.	.	.	.
**Colony*Condit*Treatm**	BFU-E	HE+E11PSC	No Inhi	0	.	.	.	.
**Colony*Condit*Treatm**	BFU-E	HE+E11PSC	With Inhi	0	.	.	.	.
**Colony*Condit*Treatm**	CFU-GEMM	AoE+E10PSC	No Inhi	37.9517	2952.47	72	0.01	0.9898
**Colony*Condit*Treatm**	CFU-GEMM	AoE+E10PSC	With Inhi	0	.	.	.	.
**Colony*Condit*Treatm**	CFU-GEMM	AoE+E11PSC	No Inhi	37.8584	2952.47	72	0.01	0.9898
**Colony*Condit*Treatm**	CFU-GEMM	AoE+E11PSC	With Inhi	0	.	.	.	.
**Colony*Condit*Treatm**	CFU-GEMM	E10.5AGM	No Inhi	38.0872	2952.47	72	0.01	0.9897
**Colony*Condit*Treatm**	CFU-GEMM	E10.5AGM	With Inhi	0	.	.	.	.
**Colony*Condit*Treatm**	CFU-GEMM	E11.5AGM	No Inhi	38.0829	2952.47	72	0.01	0.9897
**Colony*Condit*Treatm**	CFU-GEMM	E11.5AGM	With Inhi	0	.	.	.	.
**Colony*Condit*Treatm**	CFU-GEMM	HE+E10PSC	No Inhi	37.9004	0	72	Infty	<.0001
**Colony*Condit*Treatm**	CFU-GEMM	HE+E10PSC	With Inhi	0	.	.	.	.
**Colony*Condit*Treatm**	CFU-GEMM	HE+E11PSC	No Inhi	0	.	.	.	.
**Colony*Condit*Treatm**	CFU-GEMM	HE+E11PSC	With Inhi	0	.	.	.	.

The GLIMMIX Procedure

Parameter Estimates								
Effect	Colony_Type	Condition	Treatment	Estimate	Standard Error	DF	t Value	Pr > |t|
Colony*Condit*Treatm	CFU-GM	AoE+E10PSC	No Inhi	0	.	.	.	.
Colony*Condit*Treatm	CFU-GM	AoE+E10PSC	With Inhi	0	.	.	.	.
Colony*Condit*Treatm	CFU-GM	AoE+E11PSC	No Inhi	0	.	.	.	.
Colony*Condit*Treatm	CFU-GM	AoE+E11PSC	With Inhi	0	.	.	.	.
Colony*Condit*Treatm	CFU-GM	E10.5AGM	No Inhi	0	.	.	.	.
Colony*Condit*Treatm	CFU-GM	E10.5AGM	With Inhi	0	.	.	.	.
Colony*Condit*Treatm	CFU-GM	E11.5AGM	No Inhi	0	.	.	.	.
Colony*Condit*Treatm	CFU-GM	E11.5AGM	With Inhi	0	.	.	.	.
Colony*Condit*Treatm	CFU-GM	HE+E10PSC	No Inhi	0	.	.	.	.
Colony*Condit*Treatm	CFU-GM	HE+E10PSC	With Inhi	0	.	.	.	.
Colony*Condit*Treatm	CFU-GM	HE+E11PSC	No Inhi	0	.	.	.	.
Colony*Condit*Treatm	CFU-GM	HE+E11PSC	With Inhi	0	.	.	.	.

Type III Tests of Fixed Effects				
Effect	Num DF	Den DF	F Value	Pr > F
Colony_Type	2	72	0.00	1.0000
Condition	5	72	0.00	1.0000
Colony_Typ*Condition	10	72	1.94E34	<.0001
Treatment	1	72	0.00	0.9523
Colony_Typ*Treatment	2	72	0.00	1.0000
Condition*Treatment	5	72	0.00	1.0000
Colony*Condit*Treatm	9	72	0.00	1.0000

The GLIMMIX Procedure

Condition*Treatment Least Squares Means													
Condition	Treatment	Estimate	Standard Error	DF	t Value	Pr > |t|	Alpha	Lower	Upper	Mean	Standard Error Mean	Lower Mean	Upper Mean
AoE+E10PSC	No Inhi	5.1874	0.02761	72	187.85	<.0001	0.05	5.1324	5.2425	179.00	4.9431	169.42	189.13
AoE+E10PSC	With Inhi	-15.6931	852.31	72	-0.02	0.9854	0.05	-1714.75	1683.36	1.53E-7	0.000130	0	.
AoE+E11PSC	No Inhi	5.1108	0.02939	72	173.91	<.0001	0.05	5.0522	5.1694	165.81	4.8727	156.37	175.81
AoE+E11PSC	With Inhi	-15.6931	852.32	72	-0.02	0.9854	0.05	-1714.75	1683.37	1.53E-7	0.000130	0	.
E10.5AGM	No Inhi	5.7101	0.02097	72	272.33	<.0001	0.05	5.6683	5.7519	301.89	6.3299	289.53	314.78
E10.5AGM	With Inhi	-15.6931	852.32	72	-0.02	0.9854	0.05	-1714.75	1683.37	1.53E-7	0.000130	0	.
E11.5AGM	No Inhi	5.7474	0.02056	72	279.59	<.0001	0.05	5.7064	5.7884	313.37	6.4417	300.79	326.48
E11.5AGM	With Inhi	-15.6931	852.32	72	-0.02	0.9854	0.05	-1714.75	1683.37	1.53E-7	0.000130	0	.
HE+E10PSC	No Inhi	5.0516	0.03454	72	146.26	<.0001	0.05	4.9827	5.1204	156.27	5.3973	145.87	167.41
HE+E10PSC	With Inhi	-15.6931	852.32	72	-0.02	0.9854	0.05	-1714.75	1683.37	1.53E-7	0.000130	0	.
HE+E11PSC	No Inhi	5.0284	0.02998	72	167.73	<.0001	0.05	4.9687	5.0882	152.69	4.5776	143.83	162.10
HE+E11PSC	With Inhi	-28.3667	1301.86	72	-0.02	0.9827	0.05	-2623.57	2566.84	4.79E-13	6.24E-10	0	.

The GLIMMIX Procedure

Differences of Condition*Treatment Least Squares Means											
Condition	Treatment	_Condition	_Treatment	Estimate	Standard Error	DF	t Value	Pr > |t|	Alpha	Lower	Upper
AoE+E10PSC	No Inhi	AoE+E10PSC	With Inhi	20.8805	852.31	72	0.02	0.9805	0.05	-1678.18	1719.94
AoE+E10PSC	No Inhi	AoE+E11PSC	No Inhi	0.07659	0.04033	72	1.90	0.0616	0.05	-0.00380	0.1570
AoE+E10PSC	No Inhi	AoE+E11PSC	With Inhi	20.8805	852.32	72	0.02	0.9805	0.05	-1678.18	1719.94
AoE+E10PSC	No Inhi	E10.5AGM	No Inhi	-0.5227	0.03467	72	-15.07	<.0001	0.05	-0.5918	-0.4536
AoE+E10PSC	No Inhi	E10.5AGM	With Inhi	20.8805	852.32	72	0.02	0.9805	0.05	-1678.18	1719.94
AoE+E10PSC	No Inhi	E11.5AGM	No Inhi	-0.5600	0.03443	72	-16.27	<.0001	0.05	-0.6286	-0.4914
AoE+E10PSC	No Inhi	E11.5AGM	With Inhi	20.8805	852.32	72	0.02	0.9805	0.05	-1678.18	1719.94
AoE+E10PSC	No Inhi	HE+E10PSC	No Inhi	0.1358	0.04422	72	3.07	0.0030	0.05	0.04768	0.2240
AoE+E10PSC	No Inhi	HE+E10PSC	With Inhi	20.8805	852.32	72	0.02	0.9805	0.05	-1678.18	1719.94
AoE+E10PSC	No Inhi	HE+E11PSC	No Inhi	0.1590	0.04076	72	3.90	0.0002	0.05	0.07773	0.2402
AoE+E10PSC	No Inhi	HE+E11PSC	With Inhi	33.5541	1301.86	72	0.03	0.9795	0.05	-2561.65	2628.76
AoE+E10PSC	With Inhi	AoE+E11PSC	No Inhi	-20.8040	852.31	72	-0.02	0.9806	0.05	-1719.86	1678.25
AoE+E10PSC	With Inhi	AoE+E11PSC	With Inhi	7.293E-7	1205.36	72	0.00	1.0000	0.05	-2402.83	2402.83
AoE+E10PSC	With Inhi	E10.5AGM	No Inhi	-21.4032	852.31	72	-0.03	0.9800	0.05	-1720.46	1677.65
AoE+E10PSC	With Inhi	E10.5AGM	With Inhi	3.645E-7	1205.36	72	0.00	1.0000	0.05	-2402.83	2402.83
AoE+E10PSC	With Inhi	E11.5AGM	No Inhi	-21.4405	852.31	72	-0.03	0.9800	0.05	-1720.50	1677.62
AoE+E10PSC	With Inhi	E11.5AGM	With Inhi	3.645E-7	1205.36	72	0.00	1.0000	0.05	-2402.83	2402.83
AoE+E10PSC	With Inhi	HE+E10PSC	No Inhi	-20.7447	852.31	72	-0.02	0.9806	0.05	-1719.80	1678.31
AoE+E10PSC	With Inhi	HE+E10PSC	With Inhi	3.646E-7	1205.36	72	0.00	1.0000	0.05	-2402.83	2402.83
AoE+E10PSC	With Inhi	HE+E11PSC	No Inhi	-20.7216	852.31	72	-0.02	0.9807	0.05	-1719.78	1678.34
AoE+E10PSC	With Inhi	HE+E11PSC	With Inhi	12.6736	1556.05	72	0.01	0.9935	0.05	-3089.25	3114.59
AoE+E11PSC	No Inhi	AoE+E11PSC	With Inhi	20.8040	852.32	72	0.02	0.9806	0.05	-1678.26	1719.86
AoE+E11PSC	No Inhi	E10.5AGM	No Inhi	-0.5993	0.03610	72	-16.60	<.0001	0.05	-0.6712	-0.5273
AoE+E11PSC	No Inhi	E10.5AGM	With Inhi	20.8040	852.32	72	0.02	0.9806	0.05	-1678.26	1719.86

The GLIMMIX Procedure

Differences of Condition*Treatment Least Squares Means											
Condition	Treatment	_Condition	_Treatment	Estimate	Standard Error	DF	t Value	Pr > |t|	Alpha	Lower	Upper
AoE+E11PSC	No Inhi	E11.5AGM	No Inhi	-0.6366	0.03586	72	-17.75	<.0001	0.05	-0.7081	-0.5651
AoE+E11PSC	No Inhi	E11.5AGM	With Inhi	20.8040	852.32	72	0.02	0.9806	0.05	-1678.26	1719.86
AoE+E11PSC	No Inhi	HE+E10PSC	No Inhi	0.05925	0.04535	72	1.31	0.1956	0.05	-0.03116	0.1496
AoE+E11PSC	No Inhi	HE+E10PSC	With Inhi	20.8040	852.32	72	0.02	0.9806	0.05	-1678.26	1719.86
AoE+E11PSC	No Inhi	HE+E11PSC	No Inhi	0.08240	0.04198	72	1.96	0.0535	0.05	-0.00129	0.1661
AoE+E11PSC	No Inhi	HE+E11PSC	With Inhi	33.4775	1301.86	72	0.03	0.9796	0.05	-2561.73	2628.69
AoE+E11PSC	With Inhi	E10.5AGM	No Inhi	-21.4032	852.32	72	-0.03	0.9800	0.05	-1720.46	1677.66
AoE+E11PSC	With Inhi	E10.5AGM	With Inhi	-3.65E-7	1205.36	72	-0.00	1.0000	0.05	-2402.83	2402.83
AoE+E11PSC	With Inhi	E11.5AGM	No Inhi	-21.4405	852.32	72	-0.03	0.9800	0.05	-1720.50	1677.62
AoE+E11PSC	With Inhi	E11.5AGM	With Inhi	-3.65E-7	1205.36	72	-0.00	1.0000	0.05	-2402.83	2402.83
AoE+E11PSC	With Inhi	HE+E10PSC	No Inhi	-20.7447	852.32	72	-0.02	0.9806	0.05	-1719.80	1678.31
AoE+E11PSC	With Inhi	HE+E10PSC	With Inhi	-3.65E-7	1205.36	72	-0.00	1.0000	0.05	-2402.83	2402.83
AoE+E11PSC	With Inhi	HE+E11PSC	No Inhi	-20.7216	852.32	72	-0.02	0.9807	0.05	-1719.78	1678.34
AoE+E11PSC	With Inhi	HE+E11PSC	With Inhi	12.6736	1556.05	72	0.01	0.9935	0.05	-3089.25	3114.59
E10.5AGM	No Inhi	E10.5AGM	With Inhi	21.4032	852.32	72	0.03	0.9800	0.05	-1677.66	1720.46
E10.5AGM	No Inhi	E11.5AGM	No Inhi	-0.03731	0.02936	72	-1.27	0.2080	0.05	-0.09584	0.02123
E10.5AGM	No Inhi	E11.5AGM	With Inhi	21.4032	852.32	72	0.03	0.9800	0.05	-1677.66	1720.46
E10.5AGM	No Inhi	HE+E10PSC	No Inhi	0.6585	0.04040	72	16.30	<.0001	0.05	0.5780	0.7390
E10.5AGM	No Inhi	HE+E10PSC	With Inhi	21.4032	852.32	72	0.03	0.9800	0.05	-1677.66	1720.46
E10.5AGM	No Inhi	HE+E11PSC	No Inhi	0.6817	0.03658	72	18.63	<.0001	0.05	0.6087	0.7546
E10.5AGM	No Inhi	HE+E11PSC	With Inhi	34.0768	1301.86	72	0.03	0.9792	0.05	-2561.13	2629.28
E10.5AGM	With Inhi	E11.5AGM	No Inhi	-21.4405	852.32	72	-0.03	0.9800	0.05	-1720.50	1677.62
E10.5AGM	With Inhi	E11.5AGM	With Inhi	5.88E-11	1205.36	72	0.00	1.0000	0.05	-2402.83	2402.83
E10.5AGM	With Inhi	HE+E10PSC	No Inhi	-20.7447	852.32	72	-0.02	0.9806	0.05	-1719.80	1678.31

The GLIMMIX Procedure

Differences of Condition*Treatment Least Squares Means											
Condition	Treatment	_Condition	_Treatment	Estimate	Standard Error	DF	t Value	Pr > |t|	Alpha	Lower	Upper
E10.5AGM	With Inhi	HE+E10PSC	With Inhi	1.35E-10	1205.36	72	0.00	1.0000	0.05	-2402.83	2402.83
E10.5AGM	With Inhi	HE+E11PSC	No Inhi	-20.7216	852.32	72	-0.02	0.9807	0.05	-1719.78	1678.34
E10.5AGM	With Inhi	HE+E11PSC	With Inhi	12.6736	1556.05	72	0.01	0.9935	0.05	-3089.25	3114.59
E11.5AGM	No Inhi	E11.5AGM	With Inhi	21.4405	852.32	72	0.03	0.9800	0.05	-1677.62	1720.50
E11.5AGM	No Inhi	HE+E10PSC	No Inhi	0.6958	0.04019	72	17.31	<.0001	0.05	0.6157	0.7759
E11.5AGM	No Inhi	HE+E10PSC	With Inhi	21.4405	852.32	72	0.03	0.9800	0.05	-1677.62	1720.50
E11.5AGM	No Inhi	HE+E11PSC	No Inhi	0.7190	0.03635	72	19.78	<.0001	0.05	0.6465	0.7914
E11.5AGM	No Inhi	HE+E11PSC	With Inhi	34.1141	1301.86	72	0.03	0.9792	0.05	-2561.09	2629.32
E11.5AGM	With Inhi	HE+E10PSC	No Inhi	-20.7447	852.32	72	-0.02	0.9806	0.05	-1719.80	1678.32
E11.5AGM	With Inhi	HE+E10PSC	With Inhi	7.57E-11	1205.36	72	0.00	1.0000	0.05	-2402.83	2402.83
E11.5AGM	With Inhi	HE+E11PSC	No Inhi	-20.7216	852.32	72	-0.02	0.9807	0.05	-1719.78	1678.34
E11.5AGM	With Inhi	HE+E11PSC	With Inhi	12.6736	1556.05	72	0.01	0.9935	0.05	-3089.25	3114.60
HE+E10PSC	No Inhi	HE+E10PSC	With Inhi	20.7447	852.32	72	0.02	0.9806	0.05	-1678.31	1719.80
HE+E10PSC	No Inhi	HE+E11PSC	No Inhi	0.02315	0.03850	72	0.60	0.5496	0.05	-0.05361	0.09991
HE+E10PSC	No Inhi	HE+E11PSC	With Inhi	33.4183	1301.86	72	0.03	0.9796	0.05	-2561.79	2628.63
HE+E10PSC	With Inhi	HE+E11PSC	No Inhi	-20.7216	852.32	72	-0.02	0.9807	0.05	-1719.78	1678.34
HE+E10PSC	With Inhi	HE+E11PSC	With Inhi	12.6736	1556.06	72	0.01	0.9935	0.05	-3089.28	3114.63
HE+E11PSC	No Inhi	HE+E11PSC	With Inhi	33.3951	1301.86	72	0.03	0.9796	0.05	-2561.81	2628.60

The GLIMMIX Procedure

Colony*Condit*Treatm Least Squares Means														
Colony_Type	Condition	Treatment	Estimate	Standard Error	DF	t Value	Pr > |t|	Alpha	Lower	Upper	Mean	Standard Error Mean	Lower Mean	Upper Mean
BFU-E	AoE+E10PSC	No Inhi	4.7420	0.05392	72	87.95	<.0001	0.05	4.6345	4.8495	114.67	6.1824	102.98	127.68
BFU-E	AoE+E10PSC	With Inhi	-15.6932	1476.28	72	-0.01	0.9915	0.05	-2958.61	2927.22	1.529E-7	0.000226	0	.
BFU-E	AoE+E11PSC	No Inhi	4.4924	0.06108	72	73.54	<.0001	0.05	4.3706	4.6141	89.3333	5.4569	79.0914	100.90
BFU-E	AoE+E11PSC	With Inhi	-15.6932	1476.28	72	-0.01	0.9915	0.05	-2958.61	2927.22	1.529E-7	0.000226	0	.
BFU-E	E10.5AGM	No Inhi	5.2883	0.04103	72	128.89	<.0001	0.05	5.2065	5.3701	198.00	8.1240	182.45	214.88
BFU-E	E10.5AGM	With Inhi	-15.6932	1476.28	72	-0.01	0.9915	0.05	-2958.60	2927.22	1.529E-7	0.000226	0	.
BFU-E	E11.5AGM	No Inhi	5.3487	0.03981	72	134.36	<.0001	0.05	5.2693	5.4281	210.33	8.3732	194.29	227.71
BFU-E	E11.5AGM	With Inhi	-15.6932	1476.28	72	-0.01	0.9915	0.05	-2958.60	2927.22	1.529E-7	0.000226	0	.
BFU-E	HE+E10PSC	No Inhi	4.5643	0.05893	72	77.46	<.0001	0.05	4.4469	4.6818	96.0000	5.6568	85.3604	107.97
BFU-E	HE+E10PSC	With Inhi	-15.6932	1476.28	72	-0.01	0.9915	0.05	-2958.61	2927.22	1.529E-7	0.000226	0	.
BFU-E	HE+E11PSC	No Inhi	4.4773	0.06155	72	72.75	<.0001	0.05	4.3546	4.6000	88.0000	5.4160	77.8394	99.4869
BFU-E	HE+E11PSC	With Inhi	-15.6930	1476.14	72	-0.01	0.9915	0.05	-2958.32	2926.93	1.53E-7	0.000226	0	.
CFU-GEMM	AoE+E10PSC	No Inhi	4.6217	0.05726	72	80.71	<.0001	0.05	4.5076	4.7358	101.67	5.8214	90.6997	113.96
CFU-GEMM	AoE+E10PSC	With Inhi	-15.6932	1476.28	72	-0.01	0.9915	0.05	-2958.60	2927.21	1.529E-7	0.000226	0	.
CFU-GEMM	AoE+E11PSC	No Inhi	4.5850	0.05832	72	78.62	<.0001	0.05	4.4687	4.7012	98.0000	5.7155	87.2438	110.08
CFU-GEMM	AoE+E11PSC	With Inhi	-15.6932	1476.28	72	-0.01	0.9915	0.05	-2958.61	2927.22	1.529E-7	0.000226	0	.
CFU-GEMM	E10.5AGM	No Inhi	5.2003	0.04287	72	121.29	<.0001	0.05	5.1149	5.2858	181.33	7.7746	166.48	197.51
CFU-GEMM	E10.5AGM	With Inhi	-15.6932	1476.28	72	-0.01	0.9915	0.05	-2958.60	2927.21	1.529E-7	0.000226	0	.
CFU-GEMM	E11.5AGM	No Inhi	5.2240	0.04237	72	123.29	<.0001	0.05	5.1395	5.3084	185.67	7.8670	170.63	202.03
CFU-GEMM	E11.5AGM	With Inhi	-15.6932	1476.28	72	-0.01	0.9915	0.05	-2958.60	2927.22	1.529E-7	0.000226	0	.
CFU-GEMM	HE+E10PSC	No Inhi	4.4811	0.07101	72	63.11	<.0001	0.05	4.3396	4.6227	88.3333	6.2722	76.6745	101.76
CFU-GEMM	HE+E10PSC	With Inhi	-15.6932	1476.28	72	-0.01	0.9915	0.05	-2958.60	2927.21	1.529E-7	0.000226	0	.
CFU-GEMM	HE+E11PSC	No Inhi	4.5504	0.05934	72	76.68	<.0001	0.05	4.4321	4.6687	94.6667	5.6174	84.1055	106.55
CFU-GEMM	HE+E11PSC	With Inhi	-15.6930	1476.15	72	-0.01	0.9915	0.05	-2958.34	2926.95	1.53E-7	0.000226	0	.

The GLIMMIX Procedure

Colony*Condit*Treatm Least Squares Means														
Colony_Type	Condition	Treatment	Estimate	Standard Error	DF	t Value	Pr > |t|	Alpha	Lower	Upper	Mean	Standard Error Mean	Lower Mean	Upper Mean
CFU-GM	AoE+E10PSC	No Inhi	6.1985	0.02603	72	238.14	<.0001	0.05	6.1466	6.2504	492.00	12.8063	467.12	518.20
CFU-GM	AoE+E10PSC	With Inhi	-15.6931	1476.20	72	-0.01	0.9915	0.05	-2958.44	2927.05	1.53E-7	0.000226	0	.
CFU-GM	AoE+E11PSC	No Inhi	6.2551	0.02530	72	247.22	<.0001	0.05	6.2047	6.3055	520.67	13.1740	495.06	547.60
CFU-GM	AoE+E11PSC	With Inhi	-15.6931	1476.20	72	-0.01	0.9915	0.05	-2958.44	2927.05	1.53E-7	0.000226	0	.
CFU-GM	E10.5AGM	No Inhi	6.6416	0.02086	72	318.45	<.0001	0.05	6.6000	6.6832	766.33	15.9826	735.13	798.87
CFU-GM	E10.5AGM	With Inhi	-15.6931	1476.20	72	-0.01	0.9915	0.05	-2958.45	2927.06	1.53E-7	0.000226	0	.
CFU-GM	E11.5AGM	No Inhi	6.6695	0.02057	72	324.28	<.0001	0.05	6.6285	6.7105	788.00	16.2070	756.35	820.98
CFU-GM	E11.5AGM	With Inhi	-15.6931	1476.20	72	-0.01	0.9915	0.05	-2958.45	2927.06	1.53E-7	0.000226	0	.
CFU-GM	HE+E10PSC	No Inhi	6.1092	0.02722	72	224.47	<.0001	0.05	6.0550	6.1635	450.00	12.2474	426.24	475.09
CFU-GM	HE+E10PSC	With Inhi	-15.6931	1476.19	72	-0.01	0.9915	0.05	-2958.43	2927.05	1.53E-7	0.000226	0	.
CFU-GM	HE+E11PSC	No Inhi	6.0576	0.02793	72	216.89	<.0001	0.05	6.0019	6.1132	427.33	11.9350	404.19	451.80
CFU-GM	HE+E11PSC	With Inhi	-53.7142	2556.86	72	-0.02	0.9833	0.05	-5150.72	5043.29	4.7E-24	1.2E-20	0	.

The GLIMMIX Procedure

Differences of Colony*Condit*Treatm Least Squares Means													
Colony_Type	Condition	Treatment	_Colony_Type	_Condition	_Treatment	Estimate	Standard Error	DF	t Value	Pr > |t|	Alpha	Lower	Upper
BFU-E	AoE+E10PSC	No Inhi	BFU-E	AoE+E10PSC	With Inhi	20.4352	1476.28	72	0.01	0.9890	0.05	-2922.48	2963.35
BFU-E	AoE+E10PSC	No Inhi	BFU-E	AoE+E11PSC	No Inhi	0.2497	0.08148	72	3.06	0.0031	0.05	0.08724	0.4121
BFU-E	AoE+E10PSC	No Inhi	BFU-E	AoE+E11PSC	With Inhi	20.4352	1476.28	72	0.01	0.9890	0.05	-2922.48	2963.35
BFU-E	AoE+E10PSC	No Inhi	BFU-E	E10.5AGM	No Inhi	-0.5462	0.06775	72	-8.06	<.0001	0.05	-0.6813	-0.4112
BFU-E	AoE+E10PSC	No Inhi	BFU-E	E10.5AGM	With Inhi	20.4352	1476.28	72	0.01	0.9890	0.05	-2922.47	2963.35
BFU-E	AoE+E10PSC	No Inhi	BFU-E	E11.5AGM	No Inhi	-0.6067	0.06702	72	-9.05	<.0001	0.05	-0.7403	-0.4731
BFU-E	AoE+E10PSC	No Inhi	BFU-E	E11.5AGM	With Inhi	20.4352	1476.28	72	0.01	0.9890	0.05	-2922.48	2963.35
BFU-E	AoE+E10PSC	No Inhi	BFU-E	HE+E10PSC	No Inhi	0.1777	0.07987	72	2.22	0.0292	0.05	0.01846	0.3369
BFU-E	AoE+E10PSC	No Inhi	BFU-E	HE+E10PSC	With Inhi	20.4352	1476.28	72	0.01	0.9890	0.05	-2922.48	2963.35
BFU-E	AoE+E10PSC	No Inhi	BFU-E	HE+E11PSC	No Inhi	0.2647	0.08182	72	3.23	0.0018	0.05	0.1016	0.4278
BFU-E	AoE+E10PSC	No Inhi	BFU-E	HE+E11PSC	With Inhi	20.4350	1476.14	72	0.01	0.9890	0.05	-2922.19	2963.06
BFU-E	AoE+E10PSC	No Inhi	CFU-GEMM	AoE+E10PSC	No Inhi	0.1203	0.07865	72	1.53	0.1304	0.05	-0.03645	0.2771
BFU-E	AoE+E10PSC	No Inhi	CFU-GEMM	AoE+E10PSC	With Inhi	20.4352	1476.28	72	0.01	0.9890	0.05	-2922.47	2963.34
BFU-E	AoE+E10PSC	No Inhi	CFU-GEMM	AoE+E11PSC	No Inhi	0.1571	0.07942	72	1.98	0.0518	0.05	-0.00127	0.3154
BFU-E	AoE+E10PSC	No Inhi	CFU-GEMM	AoE+E11PSC	With Inhi	20.4352	1476.28	72	0.01	0.9890	0.05	-2922.48	2963.35
BFU-E	AoE+E10PSC	No Inhi	CFU-GEMM	E10.5AGM	No Inhi	-0.4583	0.06889	72	-6.65	<.0001	0.05	-0.5956	-0.3210
BFU-E	AoE+E10PSC	No Inhi	CFU-GEMM	E10.5AGM	With Inhi	20.4352	1476.28	72	0.01	0.9890	0.05	-2922.47	2963.34
BFU-E	AoE+E10PSC	No Inhi	CFU-GEMM	E11.5AGM	No Inhi	-0.4819	0.06857	72	-7.03	<.0001	0.05	-0.6186	-0.3452
BFU-E	AoE+E10PSC	No Inhi	CFU-GEMM	E11.5AGM	With Inhi	20.4352	1476.28	72	0.01	0.9890	0.05	-2922.47	2963.34
BFU-E	AoE+E10PSC	No Inhi	CFU-GEMM	HE+E10PSC	No Inhi	0.2609	0.08916	72	2.93	0.0046	0.05	0.08318	0.4386
BFU-E	AoE+E10PSC	No Inhi	CFU-GEMM	HE+E10PSC	With Inhi	20.4352	1476.28	72	0.01	0.9890	0.05	-2922.47	2963.34
BFU-E	AoE+E10PSC	No Inhi	CFU-GEMM	HE+E11PSC	No Inhi	0.1917	0.08018	72	2.39	0.0194	0.05	0.03184	0.3515
BFU-E	AoE+E10PSC	No Inhi	CFU-GEMM	HE+E11PSC	With Inhi	20.4350	1476.15	72	0.01	0.9890	0.05	-2922.21	2963.08
BFU-E	AoE+E10PSC	No Inhi	CFU-GM	AoE+E10PSC	No Inhi	-1.4564	0.05987	72	-24.33	<.0001	0.05	-1.5758	-1.3371

The GLIMMIX Procedure

Differences of Colony*Condit*Treatm Least Squares Means													
Colony_Type	Condition	Treatment	_Colony_Type	_Condition	_Treatment	Estimate	Standard Error	DF	t Value	Pr > |t|	Alpha	Lower	Upper
BFU-E	AoE+E10PSC	No Inhi	CFU-GM	AoE+E10PSC	With Inhi	20.4351	1476.20	72	0.01	0.9890	0.05	-2922.31	2963.18
BFU-E	AoE+E10PSC	No Inhi	CFU-GM	AoE+E11PSC	No Inhi	-1.5131	0.05956	72	-25.41	<.0001	0.05	-1.6318	-1.3944
BFU-E	AoE+E10PSC	No Inhi	CFU-GM	AoE+E11PSC	With Inhi	20.4351	1476.20	72	0.01	0.9890	0.05	-2922.31	2963.18
BFU-E	AoE+E10PSC	No Inhi	CFU-GM	E10.5AGM	No Inhi	-1.8996	0.05781	72	-32.86	<.0001	0.05	-2.0148	-1.7843
BFU-E	AoE+E10PSC	No Inhi	CFU-GM	E10.5AGM	With Inhi	20.4351	1476.20	72	0.01	0.9890	0.05	-2922.32	2963.19
BFU-E	AoE+E10PSC	No Inhi	CFU-GM	E11.5AGM	No Inhi	-1.9275	0.05771	72	-33.40	<.0001	0.05	-2.0425	-1.8124
BFU-E	AoE+E10PSC	No Inhi	CFU-GM	E11.5AGM	With Inhi	20.4351	1476.20	72	0.01	0.9890	0.05	-2922.32	2963.19
BFU-E	AoE+E10PSC	No Inhi	CFU-GM	HE+E10PSC	No Inhi	-1.3672	0.06040	72	-22.64	<.0001	0.05	-1.4876	-1.2468
BFU-E	AoE+E10PSC	No Inhi	CFU-GM	HE+E10PSC	With Inhi	20.4351	1476.19	72	0.01	0.9890	0.05	-2922.30	2963.17
BFU-E	AoE+E10PSC	No Inhi	CFU-GM	HE+E11PSC	No Inhi	-1.3155	0.06072	72	-21.67	<.0001	0.05	-1.4366	-1.1945
BFU-E	AoE+E10PSC	No Inhi	CFU-GM	HE+E11PSC	With Inhi	58.4562	2556.86	72	0.02	0.9818	0.05	-5038.55	5155.46
BFU-E	AoE+E10PSC	With Inhi	BFU-E	AoE+E11PSC	No Inhi	-20.1856	1476.28	72	-0.01	0.9891	0.05	-2963.10	2922.73
BFU-E	AoE+E10PSC	With Inhi	BFU-E	AoE+E11PSC	With Inhi	2.415E-6	2087.78	72	0.00	1.0000	0.05	-4161.91	4161.91
BFU-E	AoE+E10PSC	With Inhi	BFU-E	E10.5AGM	No Inhi	-20.9814	1476.28	72	-0.01	0.9887	0.05	-2963.89	2921.93
BFU-E	AoE+E10PSC	With Inhi	BFU-E	E10.5AGM	With Inhi	-1.82E-6	2087.78	72	-0.00	1.0000	0.05	-4161.91	4161.91
BFU-E	AoE+E10PSC	With Inhi	BFU-E	E11.5AGM	No Inhi	-21.0419	1476.28	72	-0.01	0.9887	0.05	-2963.96	2921.87
BFU-E	AoE+E10PSC	With Inhi	BFU-E	E11.5AGM	With Inhi	-1.55E-6	2087.78	72	-0.00	1.0000	0.05	-4161.91	4161.91
BFU-E	AoE+E10PSC	With Inhi	BFU-E	HE+E10PSC	No Inhi	-20.2575	1476.28	72	-0.01	0.9891	0.05	-2963.17	2922.66
BFU-E	AoE+E10PSC	With Inhi	BFU-E	HE+E10PSC	With Inhi	-4.56E-7	2087.78	72	-0.00	1.0000	0.05	-4161.91	4161.91
BFU-E	AoE+E10PSC	With Inhi	BFU-E	HE+E11PSC	No Inhi	-20.1705	1476.28	72	-0.01	0.9891	0.05	-2963.08	2922.74
BFU-E	AoE+E10PSC	With Inhi	BFU-E	HE+E11PSC	With Inhi	-0.00019	2087.68	72	-0.00	1.0000	0.05	-4161.70	4161.70
BFU-E	AoE+E10PSC	With Inhi	CFU-GEMM	AoE+E10PSC	No Inhi	-20.3149	1476.28	72	-0.01	0.9891	0.05	-2963.23	2922.60
BFU-E	AoE+E10PSC	With Inhi	CFU-GEMM	AoE+E10PSC	With Inhi	-3.19E-6	2087.78	72	-0.00	1.0000	0.05	-4161.90	4161.90
BFU-E	AoE+E10PSC	With Inhi	CFU-GEMM	AoE+E11PSC	No Inhi	-20.2781	1476.28	72	-0.01	0.9891	0.05	-2963.19	2922.64

The GLIMMIX Procedure

Differences of Colony*Condit*Treatm Least Squares Means													
Colony_Type	Condition	Treatment	_Colony_Type	_Condition	_Treatment	Estimate	Standard Error	DF	t Value	Pr > |t|	Alpha	Lower	Upper
BFU-E	AoE+E10PSC	With Inhi	CFU-GEMM	AoE+E11PSC	With Inhi	-8.66E-7	2087.78	72	-0.00	1.0000	0.05	-4161.91	4161.91
BFU-E	AoE+E10PSC	With Inhi	CFU-GEMM	E10.5AGM	No Inhi	-20.8935	1476.28	72	-0.01	0.9887	0.05	-2963.81	2922.02
BFU-E	AoE+E10PSC	With Inhi	CFU-GEMM	E10.5AGM	With Inhi	-3.74E-6	2087.78	72	-0.00	1.0000	0.05	-4161.90	4161.90
BFU-E	AoE+E10PSC	With Inhi	CFU-GEMM	E11.5AGM	No Inhi	-20.9171	1476.28	72	-0.01	0.9887	0.05	-2963.83	2922.00
BFU-E	AoE+E10PSC	With Inhi	CFU-GEMM	E11.5AGM	With Inhi	-2.37E-6	2087.78	72	-0.00	1.0000	0.05	-4161.91	4161.91
BFU-E	AoE+E10PSC	With Inhi	CFU-GEMM	HE+E10PSC	No Inhi	-20.1743	1476.28	72	-0.01	0.9891	0.05	-2963.09	2922.74
BFU-E	AoE+E10PSC	With Inhi	CFU-GEMM	HE+E10PSC	With Inhi	-1.41E-6	2087.78	72	-0.00	1.0000	0.05	-4161.90	4161.90
BFU-E	AoE+E10PSC	With Inhi	CFU-GEMM	HE+E11PSC	No Inhi	-20.2435	1476.28	72	-0.01	0.9891	0.05	-2963.16	2922.67
BFU-E	AoE+E10PSC	With Inhi	CFU-GEMM	HE+E11PSC	With Inhi	-0.00018	2087.68	72	-0.00	1.0000	0.05	-4161.72	4161.72
BFU-E	AoE+E10PSC	With Inhi	CFU-GM	AoE+E10PSC	No Inhi	-21.8917	1476.28	72	-0.01	0.9882	0.05	-2964.80	2921.02
BFU-E	AoE+E10PSC	With Inhi	CFU-GM	AoE+E10PSC	With Inhi	-0.00011	2087.72	72	-0.00	1.0000	0.05	-4161.79	4161.79
BFU-E	AoE+E10PSC	With Inhi	CFU-GM	AoE+E11PSC	No Inhi	-21.9483	1476.28	72	-0.01	0.9882	0.05	-2964.86	2920.96
BFU-E	AoE+E10PSC	With Inhi	CFU-GM	AoE+E11PSC	With Inhi	-0.00012	2087.72	72	-0.00	1.0000	0.05	-4161.79	4161.79
BFU-E	AoE+E10PSC	With Inhi	CFU-GM	E10.5AGM	No Inhi	-22.3348	1476.28	72	-0.02	0.9880	0.05	-2965.25	2920.58
BFU-E	AoE+E10PSC	With Inhi	CFU-GM	E10.5AGM	With Inhi	-0.00011	2087.72	72	-0.00	1.0000	0.05	-4161.80	4161.80
BFU-E	AoE+E10PSC	With Inhi	CFU-GM	E11.5AGM	No Inhi	-22.3627	1476.28	72	-0.02	0.9880	0.05	-2965.28	2920.55
BFU-E	AoE+E10PSC	With Inhi	CFU-GM	E11.5AGM	With Inhi	-0.00011	2087.72	72	-0.00	1.0000	0.05	-4161.79	4161.79
BFU-E	AoE+E10PSC	With Inhi	CFU-GM	HE+E10PSC	No Inhi	-21.8024	1476.28	72	-0.01	0.9883	0.05	-2964.72	2921.11
BFU-E	AoE+E10PSC	With Inhi	CFU-GM	HE+E10PSC	With Inhi	-0.00011	2087.72	72	-0.00	1.0000	0.05	-4161.78	4161.78
BFU-E	AoE+E10PSC	With Inhi	CFU-GM	HE+E11PSC	No Inhi	-21.7507	1476.28	72	-0.01	0.9883	0.05	-2964.66	2921.16
BFU-E	AoE+E10PSC	With Inhi	CFU-GM	HE+E11PSC	With Inhi	38.0210	2952.44	72	0.01	0.9898	0.05	-5847.57	5923.61
BFU-E	AoE+E11PSC	No Inhi	BFU-E	AoE+E11PSC	With Inhi	20.1856	1476.28	72	0.01	0.9891	0.05	-2922.73	2963.10
BFU-E	AoE+E11PSC	No Inhi	BFU-E	E10.5AGM	No Inhi	-0.7959	0.07359	72	-10.82	<.0001	0.05	-0.9426	-0.6492
BFU-E	AoE+E11PSC	No Inhi	BFU-E	E10.5AGM	With Inhi	20.1855	1476.28	72	0.01	0.9891	0.05	-2922.72	2963.10

The GLIMMIX Procedure

Differences of Colony*Condit*Treatm Least Squares Means													
Colony_Type	Condition	Treatment	_Colony_Type	_Condition	_Treatment	Estimate	Standard Error	DF	t Value	Pr > |t|	Alpha	Lower	Upper
BFU-E	AoE+E11PSC	No Inhi	BFU-E	E11.5AGM	No Inhi	-0.8563	0.07291	72	-11.74	<.0001	0.05	-1.0017	-0.7110
BFU-E	AoE+E11PSC	No Inhi	BFU-E	E11.5AGM	With Inhi	20.1855	1476.28	72	0.01	0.9891	0.05	-2922.72	2963.10
BFU-E	AoE+E11PSC	No Inhi	BFU-E	HE+E10PSC	No Inhi	-0.07197	0.08487	72	-0.85	0.3992	0.05	-0.2412	0.09722
BFU-E	AoE+E11PSC	No Inhi	BFU-E	HE+E10PSC	With Inhi	20.1855	1476.28	72	0.01	0.9891	0.05	-2922.73	2963.10
BFU-E	AoE+E11PSC	No Inhi	BFU-E	HE+E11PSC	No Inhi	0.01504	0.08671	72	0.17	0.8628	0.05	-0.1578	0.1879
BFU-E	AoE+E11PSC	No Inhi	BFU-E	HE+E11PSC	With Inhi	20.1854	1476.14	72	0.01	0.9891	0.05	-2922.44	2962.81
BFU-E	AoE+E11PSC	No Inhi	CFU-GEMM	AoE+E10PSC	No Inhi	-0.1293	0.08373	72	-1.54	0.1268	0.05	-0.2962	0.03758
BFU-E	AoE+E11PSC	No Inhi	CFU-GEMM	AoE+E10PSC	With Inhi	20.1855	1476.28	72	0.01	0.9891	0.05	-2922.72	2963.09
BFU-E	AoE+E11PSC	No Inhi	CFU-GEMM	AoE+E11PSC	No Inhi	-0.09259	0.08446	72	-1.10	0.2766	0.05	-0.2610	0.07577
BFU-E	AoE+E11PSC	No Inhi	CFU-GEMM	AoE+E11PSC	With Inhi	20.1855	1476.28	72	0.01	0.9891	0.05	-2922.73	2963.10
BFU-E	AoE+E11PSC	No Inhi	CFU-GEMM	E10.5AGM	No Inhi	-0.7080	0.07463	72	-9.49	<.0001	0.05	-0.8567	-0.5592
BFU-E	AoE+E11PSC	No Inhi	CFU-GEMM	E10.5AGM	With Inhi	20.1855	1476.28	72	0.01	0.9891	0.05	-2922.72	2963.09
BFU-E	AoE+E11PSC	No Inhi	CFU-GEMM	E11.5AGM	No Inhi	-0.7316	0.07434	72	-9.84	<.0001	0.05	-0.8798	-0.5834
BFU-E	AoE+E11PSC	No Inhi	CFU-GEMM	E11.5AGM	With Inhi	20.1855	1476.28	72	0.01	0.9891	0.05	-2922.72	2963.09
BFU-E	AoE+E11PSC	No Inhi	CFU-GEMM	HE+E10PSC	No Inhi	0.01126	0.09367	72	0.12	0.9047	0.05	-0.1755	0.1980
BFU-E	AoE+E11PSC	No Inhi	CFU-GEMM	HE+E10PSC	With Inhi	20.1855	1476.28	72	0.01	0.9891	0.05	-2922.72	2963.09
BFU-E	AoE+E11PSC	No Inhi	CFU-GEMM	HE+E11PSC	No Inhi	-0.05799	0.08516	72	-0.68	0.4981	0.05	-0.2278	0.1118
BFU-E	AoE+E11PSC	No Inhi	CFU-GEMM	HE+E11PSC	With Inhi	20.1854	1476.15	72	0.01	0.9891	0.05	-2922.46	2962.83
BFU-E	AoE+E11PSC	No Inhi	CFU-GM	AoE+E10PSC	No Inhi	-1.7061	0.06640	72	-25.69	<.0001	0.05	-1.8385	-1.5737
BFU-E	AoE+E11PSC	No Inhi	CFU-GM	AoE+E10PSC	With Inhi	20.1854	1476.20	72	0.01	0.9891	0.05	-2922.56	2962.93
BFU-E	AoE+E11PSC	No Inhi	CFU-GM	AoE+E11PSC	No Inhi	-1.7627	0.06612	72	-26.66	<.0001	0.05	-1.8945	-1.6309
BFU-E	AoE+E11PSC	No Inhi	CFU-GM	AoE+E11PSC	With Inhi	20.1854	1476.20	72	0.01	0.9891	0.05	-2922.56	2962.93
BFU-E	AoE+E11PSC	No Inhi	CFU-GM	E10.5AGM	No Inhi	-2.1492	0.06455	72	-33.30	<.0001	0.05	-2.2779	-2.0206
BFU-E	AoE+E11PSC	No Inhi	CFU-GM	E10.5AGM	With Inhi	20.1854	1476.20	72	0.01	0.9891	0.05	-2922.57	2962.94

The GLIMMIX Procedure

Differences of Colony*Condit*Treatm Least Squares Means

Colony_Type	Condition	Treatment	_Colony_Type	_Condition	_Treatment	Estimate	Standard Error	DF	t Value	Pr > |t|	Alpha	Lower	Upper
BFU-E	AoE+E11PSC	No Inhi	CFU-GM	E11.5AGM	No Inhi	-2.1771	0.06445	72	-33.78	<.0001	0.05	-2.3056	-2.0486
BFU-E	AoE+E11PSC	No Inhi	CFU-GM	E11.5AGM	With Inhi	20.1854	1476.20	72	0.01	0.9891	0.05	-2922.57	2962.94
BFU-E	AoE+E11PSC	No Inhi	CFU-GM	HE+E10PSC	No Inhi	-1.6169	0.06687	72	-24.18	<.0001	0.05	-1.7502	-1.4836
BFU-E	AoE+E11PSC	No Inhi	CFU-GM	HE+E10PSC	With Inhi	20.1854	1476.19	72	0.01	0.9891	0.05	-2922.55	2962.92
BFU-E	AoE+E11PSC	No Inhi	CFU-GM	HE+E11PSC	No Inhi	-1.5652	0.06717	72	-23.30	<.0001	0.05	-1.6991	-1.4313
BFU-E	AoE+E11PSC	No Inhi	CFU-GM	HE+E11PSC	With Inhi	58.2066	2556.86	72	0.02	0.9819	0.05	-5038.80	5155.21
BFU-E	AoE+E11PSC	With Inhi	BFU-E	E10.5AGM	No Inhi	-20.9814	1476.28	72	-0.01	0.9887	0.05	-2963.90	2921.94
BFU-E	AoE+E11PSC	With Inhi	BFU-E	E10.5AGM	With Inhi	-4.24E-6	2087.78	72	-0.00	1.0000	0.05	-4161.91	4161.91
BFU-E	AoE+E11PSC	With Inhi	BFU-E	E11.5AGM	No Inhi	-21.0419	1476.28	72	-0.01	0.9887	0.05	-2963.96	2921.87
BFU-E	AoE+E11PSC	With Inhi	BFU-E	E11.5AGM	With Inhi	-3.97E-6	2087.78	72	-0.00	1.0000	0.05	-4161.91	4161.91
BFU-E	AoE+E11PSC	With Inhi	BFU-E	HE+E10PSC	No Inhi	-20.2575	1476.28	72	-0.01	0.9891	0.05	-2963.17	2922.66
BFU-E	AoE+E11PSC	With Inhi	BFU-E	HE+E10PSC	With Inhi	-2.87E-6	2087.78	72	-0.00	1.0000	0.05	-4161.91	4161.91
BFU-E	AoE+E11PSC	With Inhi	BFU-E	HE+E11PSC	No Inhi	-20.1705	1476.28	72	-0.01	0.9891	0.05	-2963.09	2922.75
BFU-E	AoE+E11PSC	With Inhi	BFU-E	HE+E11PSC	With Inhi	-0.00020	2087.68	72	-0.00	1.0000	0.05	-4161.71	4161.71
BFU-E	AoE+E11PSC	With Inhi	CFU-GEMM	AoE+E10PSC	No Inhi	-20.3149	1476.28	72	-0.01	0.9891	0.05	-2963.23	2922.60
BFU-E	AoE+E11PSC	With Inhi	CFU-GEMM	AoE+E10PSC	With Inhi	-5.61E-6	2087.78	72	-0.00	1.0000	0.05	-4161.91	4161.91
BFU-E	AoE+E11PSC	With Inhi	CFU-GEMM	AoE+E11PSC	No Inhi	-20.2781	1476.28	72	-0.01	0.9891	0.05	-2963.19	2922.64
BFU-E	AoE+E11PSC	With Inhi	CFU-GEMM	AoE+E11PSC	With Inhi	-3.28E-6	2087.78	72	-0.00	1.0000	0.05	-4161.91	4161.91
BFU-E	AoE+E11PSC	With Inhi	CFU-GEMM	E10.5AGM	No Inhi	-20.8935	1476.28	72	-0.01	0.9887	0.05	-2963.81	2922.02
BFU-E	AoE+E11PSC	With Inhi	CFU-GEMM	E10.5AGM	With Inhi	-6.15E-6	2087.78	72	-0.00	1.0000	0.05	-4161.91	4161.91
BFU-E	AoE+E11PSC	With Inhi	CFU-GEMM	E11.5AGM	No Inhi	-20.9171	1476.28	72	-0.01	0.9887	0.05	-2963.83	2922.00
BFU-E	AoE+E11PSC	With Inhi	CFU-GEMM	E11.5AGM	With Inhi	-4.79E-6	2087.78	72	-0.00	1.0000	0.05	-4161.91	4161.91
BFU-E	AoE+E11PSC	With Inhi	CFU-GEMM	HE+E10PSC	No Inhi	-20.1743	1476.28	72	-0.01	0.9891	0.05	-2963.09	2922.74
BFU-E	AoE+E11PSC	With Inhi	CFU-GEMM	HE+E10PSC	With Inhi	-3.83E-6	2087.78	72	-0.00	1.0000	0.05	-4161.91	4161.91

The GLIMMIX Procedure

Differences of Colony*Condit*Treatm Least Squares Means													
Colony_Type	Condition	Treatment	_Colony_Type	_Condition	_Treatment	Estimate	Standard Error	DF	t Value	Pr > |t|	Alpha	Lower	Upper
BFU-E	AoE+E11PSC	With Inhi	CFU-GEMM	HE+E11PSC	No Inhi	-20.2435	1476.28	72	-0.01	0.9891	0.05	-2963.16	2922.67
BFU-E	AoE+E11PSC	With Inhi	CFU-GEMM	HE+E11PSC	With Inhi	-0.00018	2087.68	72	-0.00	1.0000	0.05	-4161.72	4161.72
BFU-E	AoE+E11PSC	With Inhi	CFU-GM	AoE+E10PSC	No Inhi	-21.8917	1476.28	72	-0.01	0.9882	0.05	-2964.81	2921.02
BFU-E	AoE+E11PSC	With Inhi	CFU-GM	AoE+E10PSC	With Inhi	-0.00012	2087.72	72	-0.00	1.0000	0.05	-4161.79	4161.79
BFU-E	AoE+E11PSC	With Inhi	CFU-GM	AoE+E11PSC	No Inhi	-21.9483	1476.28	72	-0.01	0.9882	0.05	-2964.86	2920.97
BFU-E	AoE+E11PSC	With Inhi	CFU-GM	AoE+E11PSC	With Inhi	-0.00012	2087.72	72	-0.00	1.0000	0.05	-4161.79	4161.79
BFU-E	AoE+E11PSC	With Inhi	CFU-GM	E10.5AGM	No Inhi	-22.3348	1476.28	72	-0.02	0.9880	0.05	-2965.25	2920.58
BFU-E	AoE+E11PSC	With Inhi	CFU-GM	E10.5AGM	With Inhi	-0.00011	2087.72	72	-0.00	1.0000	0.05	-4161.80	4161.80
BFU-E	AoE+E11PSC	With Inhi	CFU-GM	E11.5AGM	No Inhi	-22.3627	1476.28	72	-0.02	0.9880	0.05	-2965.28	2920.55
BFU-E	AoE+E11PSC	With Inhi	CFU-GM	E11.5AGM	With Inhi	-0.00011	2087.72	72	-0.00	1.0000	0.05	-4161.80	4161.80
BFU-E	AoE+E11PSC	With Inhi	CFU-GM	HE+E10PSC	No Inhi	-21.8024	1476.28	72	-0.01	0.9883	0.05	-2964.72	2921.11
BFU-E	AoE+E11PSC	With Inhi	CFU-GM	HE+E10PSC	With Inhi	-0.00012	2087.72	72	-0.00	1.0000	0.05	-4161.79	4161.79
BFU-E	AoE+E11PSC	With Inhi	CFU-GM	HE+E11PSC	No Inhi	-21.7507	1476.28	72	-0.01	0.9883	0.05	-2964.67	2921.17
BFU-E	AoE+E11PSC	With Inhi	CFU-GM	HE+E11PSC	With Inhi	38.0210	2952.45	72	0.01	0.9898	0.05	-5847.57	5923.61
BFU-E	E10.5AGM	No Inhi	BFU-E	E10.5AGM	With Inhi	20.9814	1476.28	72	0.01	0.9887	0.05	-2921.93	2963.89
BFU-E	E10.5AGM	No Inhi	BFU-E	E11.5AGM	No Inhi	-0.06043	0.05717	72	-1.06	0.2941	0.05	-0.1744	0.05354
BFU-E	E10.5AGM	No Inhi	BFU-E	E11.5AGM	With Inhi	20.9814	1476.28	72	0.01	0.9887	0.05	-2921.93	2963.89
BFU-E	E10.5AGM	No Inhi	BFU-E	HE+E10PSC	No Inhi	0.7239	0.07180	72	10.08	<.0001	0.05	0.5808	0.8671
BFU-E	E10.5AGM	No Inhi	BFU-E	HE+E10PSC	With Inhi	20.9814	1476.28	72	0.01	0.9887	0.05	-2921.93	2963.89
BFU-E	E10.5AGM	No Inhi	BFU-E	HE+E11PSC	No Inhi	0.8109	0.07397	72	10.96	<.0001	0.05	0.6635	0.9584
BFU-E	E10.5AGM	No Inhi	BFU-E	HE+E11PSC	With Inhi	20.9812	1476.14	72	0.01	0.9887	0.05	-2921.64	2963.61
BFU-E	E10.5AGM	No Inhi	CFU-GEMM	AoE+E10PSC	No Inhi	0.6666	0.07044	72	9.46	<.0001	0.05	0.5261	0.8070
BFU-E	E10.5AGM	No Inhi	CFU-GEMM	AoE+E10PSC	With Inhi	20.9814	1476.28	72	0.01	0.9887	0.05	-2921.93	2963.89
BFU-E	E10.5AGM	No Inhi	CFU-GEMM	AoE+E11PSC	No Inhi	0.7033	0.07131	72	9.86	<.0001	0.05	0.5611	0.8454

The GLIMMIX Procedure

Differences of Colony*Condit*Treatm Least Squares Means													
Colony_Type	Condition	Treatment	_Colony_Type	_Condition	_Treatment	Estimate	Standard Error	DF	t Value	Pr > |t|	Alpha	Lower	Upper
BFU-E	E10.5AGM	No Inhi	CFU-GEMM	AoE+E11PSC	With Inhi	20.9814	1476.28	72	0.01	0.9887	0.05	-2921.93	2963.89
BFU-E	E10.5AGM	No Inhi	CFU-GEMM	E10.5AGM	No Inhi	0.08793	0.05934	72	1.48	0.1428	0.05	-0.03037	0.2062
BFU-E	E10.5AGM	No Inhi	CFU-GEMM	E10.5AGM	With Inhi	20.9814	1476.28	72	0.01	0.9887	0.05	-2921.93	2963.89
BFU-E	E10.5AGM	No Inhi	CFU-GEMM	E11.5AGM	No Inhi	0.06431	0.05898	72	1.09	0.2792	0.05	-0.05326	0.1819
BFU-E	E10.5AGM	No Inhi	CFU-GEMM	E11.5AGM	With Inhi	20.9814	1476.28	72	0.01	0.9887	0.05	-2921.93	2963.89
BFU-E	E10.5AGM	No Inhi	CFU-GEMM	HE+E10PSC	No Inhi	0.8071	0.08201	72	9.84	<.0001	0.05	0.6437	0.9706
BFU-E	E10.5AGM	No Inhi	CFU-GEMM	HE+E10PSC	With Inhi	20.9814	1476.28	72	0.01	0.9887	0.05	-2921.93	2963.89
BFU-E	E10.5AGM	No Inhi	CFU-GEMM	HE+E11PSC	No Inhi	0.7379	0.07214	72	10.23	<.0001	0.05	0.5941	0.8817
BFU-E	E10.5AGM	No Inhi	CFU-GEMM	HE+E11PSC	With Inhi	20.9813	1476.15	72	0.01	0.9887	0.05	-2921.66	2963.62
BFU-E	E10.5AGM	No Inhi	CFU-GM	AoE+E10PSC	No Inhi	-0.9102	0.04859	72	-18.73	<.0001	0.05	-1.0071	-0.8133
BFU-E	E10.5AGM	No Inhi	CFU-GM	AoE+E10PSC	With Inhi	20.9813	1476.20	72	0.01	0.9887	0.05	-2921.76	2963.72
BFU-E	E10.5AGM	No Inhi	CFU-GM	AoE+E11PSC	No Inhi	-0.9668	0.04820	72	-20.06	<.0001	0.05	-1.0629	-0.8707
BFU-E	E10.5AGM	No Inhi	CFU-GM	AoE+E11PSC	With Inhi	20.9813	1476.20	72	0.01	0.9887	0.05	-2921.76	2963.72
BFU-E	E10.5AGM	No Inhi	CFU-GM	E10.5AGM	No Inhi	-1.3534	0.04603	72	-29.40	<.0001	0.05	-1.4451	-1.2616
BFU-E	E10.5AGM	No Inhi	CFU-GM	E10.5AGM	With Inhi	20.9813	1476.20	72	0.01	0.9887	0.05	-2921.77	2963.74
BFU-E	E10.5AGM	No Inhi	CFU-GM	E11.5AGM	No Inhi	-1.3812	0.04590	72	-30.09	<.0001	0.05	-1.4727	-1.2897
BFU-E	E10.5AGM	No Inhi	CFU-GM	E11.5AGM	With Inhi	20.9813	1476.20	72	0.01	0.9887	0.05	-2921.77	2963.73
BFU-E	E10.5AGM	No Inhi	CFU-GM	HE+E10PSC	No Inhi	-0.8210	0.04924	72	-16.67	<.0001	0.05	-0.9191	-0.7228
BFU-E	E10.5AGM	No Inhi	CFU-GM	HE+E10PSC	With Inhi	20.9813	1476.19	72	0.01	0.9887	0.05	-2921.76	2963.72
BFU-E	E10.5AGM	No Inhi	CFU-GM	HE+E11PSC	No Inhi	-0.7693	0.04963	72	-15.50	<.0001	0.05	-0.8682	-0.6704
BFU-E	E10.5AGM	No Inhi	CFU-GM	HE+E11PSC	With Inhi	59.0024	2556.86	72	0.02	0.9817	0.05	-5038.00	5156.01
BFU-E	E10.5AGM	With Inhi	BFU-E	E11.5AGM	No Inhi	-21.0419	1476.28	72	-0.01	0.9887	0.05	-2963.95	2921.87
BFU-E	E10.5AGM	With Inhi	BFU-E	E11.5AGM	With Inhi	2.734E-7	2087.78	72	0.00	1.0000	0.05	-4161.90	4161.90
BFU-E	E10.5AGM	With Inhi	BFU-E	HE+E10PSC	No Inhi	-20.2575	1476.28	72	-0.01	0.9891	0.05	-2963.17	2922.65

The GLIMMIX Procedure

Differences of Colony*Condit*Treatm Least Squares Means													
Colony_Type	Condition	Treatment	_Colony_Type	_Condition	_Treatment	Estimate	Standard Error	DF	t Value	Pr > |t|	Alpha	Lower	Upper
BFU-E	E10.5AGM	With Inhi	BFU-E	HE+E10PSC	With Inhi	1.367E-6	2087.78	72	0.00	1.0000	0.05	-4161.90	4161.90
BFU-E	E10.5AGM	With Inhi	BFU-E	HE+E11PSC	No Inhi	-20.1705	1476.28	72	-0.01	0.9891	0.05	-2963.08	2922.74
BFU-E	E10.5AGM	With Inhi	BFU-E	HE+E11PSC	With Inhi	-0.00019	2087.67	72	-0.00	1.0000	0.05	-4161.70	4161.70
BFU-E	E10.5AGM	With Inhi	CFU-GEMM	AoE+E10PSC	No Inhi	-20.3149	1476.28	72	-0.01	0.9891	0.05	-2963.22	2922.60
BFU-E	E10.5AGM	With Inhi	CFU-GEMM	AoE+E10PSC	With Inhi	-1.37E-6	2087.77	72	-0.00	1.0000	0.05	-4161.90	4161.90
BFU-E	E10.5AGM	With Inhi	CFU-GEMM	AoE+E11PSC	No Inhi	-20.2781	1476.28	72	-0.01	0.9891	0.05	-2963.19	2922.63
BFU-E	E10.5AGM	With Inhi	CFU-GEMM	AoE+E11PSC	With Inhi	9.571E-7	2087.78	72	0.00	1.0000	0.05	-4161.90	4161.90
BFU-E	E10.5AGM	With Inhi	CFU-GEMM	E10.5AGM	No Inhi	-20.8935	1476.28	72	-0.01	0.9887	0.05	-2963.80	2922.02
BFU-E	E10.5AGM	With Inhi	CFU-GEMM	E10.5AGM	With Inhi	-1.91E-6	2087.77	72	-0.00	1.0000	0.05	-4161.90	4161.90
BFU-E	E10.5AGM	With Inhi	CFU-GEMM	E11.5AGM	No Inhi	-20.9171	1476.28	72	-0.01	0.9887	0.05	-2963.83	2921.99
BFU-E	E10.5AGM	With Inhi	CFU-GEMM	E11.5AGM	With Inhi	-5.47E-7	2087.77	72	-0.00	1.0000	0.05	-4161.90	4161.90
BFU-E	E10.5AGM	With Inhi	CFU-GEMM	HE+E10PSC	No Inhi	-20.1743	1476.28	72	-0.01	0.9891	0.05	-2963.08	2922.74
BFU-E	E10.5AGM	With Inhi	CFU-GEMM	HE+E10PSC	With Inhi	4.102E-7	2087.77	72	0.00	1.0000	0.05	-4161.90	4161.90
BFU-E	E10.5AGM	With Inhi	CFU-GEMM	HE+E11PSC	No Inhi	-20.2435	1476.28	72	-0.01	0.9891	0.05	-2963.15	2922.67
BFU-E	E10.5AGM	With Inhi	CFU-GEMM	HE+E11PSC	With Inhi	-0.00018	2087.68	72	-0.00	1.0000	0.05	-4161.72	4161.72
BFU-E	E10.5AGM	With Inhi	CFU-GM	AoE+E10PSC	No Inhi	-21.8917	1476.28	72	-0.01	0.9882	0.05	-2964.80	2921.02
BFU-E	E10.5AGM	With Inhi	CFU-GM	AoE+E10PSC	With Inhi	-0.00011	2087.72	72	-0.00	1.0000	0.05	-4161.79	4161.79
BFU-E	E10.5AGM	With Inhi	CFU-GM	AoE+E11PSC	No Inhi	-21.9483	1476.28	72	-0.01	0.9882	0.05	-2964.86	2920.96
BFU-E	E10.5AGM	With Inhi	CFU-GM	AoE+E11PSC	With Inhi	-0.00011	2087.72	72	-0.00	1.0000	0.05	-4161.78	4161.78
BFU-E	E10.5AGM	With Inhi	CFU-GM	E10.5AGM	No Inhi	-22.3348	1476.28	72	-0.02	0.9880	0.05	-2965.24	2920.58
BFU-E	E10.5AGM	With Inhi	CFU-GM	E10.5AGM	With Inhi	-0.00011	2087.72	72	-0.00	1.0000	0.05	-4161.79	4161.79
BFU-E	E10.5AGM	With Inhi	CFU-GM	E11.5AGM	No Inhi	-22.3627	1476.28	72	-0.02	0.9880	0.05	-2965.27	2920.55
BFU-E	E10.5AGM	With Inhi	CFU-GM	E11.5AGM	With Inhi	-0.00011	2087.72	72	-0.00	1.0000	0.05	-4161.79	4161.79
BFU-E	E10.5AGM	With Inhi	CFU-GM	HE+E10PSC	No Inhi	-21.8024	1476.28	72	-0.01	0.9883	0.05	-2964.71	2921.11

The GLIMMIX Procedure

Differences of Colony*Condit*Treatm Least Squares Means													
Colony_Type	Condition	Treatment	_Colony_Type	_Condition	_Treatment	Estimate	Standard Error	DF	t Value	Pr > |t|	Alpha	Lower	Upper
BFU-E	E10.5AGM	With Inhi	CFU-GM	HE+E10PSC	With Inhi	-0.00011	2087.71	72	-0.00	1.0000	0.05	-4161.78	4161.78
BFU-E	E10.5AGM	With Inhi	CFU-GM	HE+E11PSC	No Inhi	-21.7507	1476.28	72	-0.01	0.9883	0.05	-2964.66	2921.16
BFU-E	E10.5AGM	With Inhi	CFU-GM	HE+E11PSC	With Inhi	38.0210	2952.45	72	0.01	0.9898	0.05	-5847.57	5923.61
BFU-E	E11.5AGM	No Inhi	BFU-E	E11.5AGM	With Inhi	21.0419	1476.28	72	0.01	0.9887	0.05	-2921.87	2963.95
BFU-E	E11.5AGM	No Inhi	BFU-E	HE+E10PSC	No Inhi	0.7843	0.07111	72	11.03	<.0001	0.05	0.6426	0.9261
BFU-E	E11.5AGM	No Inhi	BFU-E	HE+E10PSC	With Inhi	21.0419	1476.28	72	0.01	0.9887	0.05	-2921.87	2963.95
BFU-E	E11.5AGM	No Inhi	BFU-E	HE+E11PSC	No Inhi	0.8714	0.07330	72	11.89	<.0001	0.05	0.7252	1.0175
BFU-E	E11.5AGM	No Inhi	BFU-E	HE+E11PSC	With Inhi	21.0417	1476.14	72	0.01	0.9887	0.05	-2921.58	2963.67
BFU-E	E11.5AGM	No Inhi	CFU-GEMM	AoE+E10PSC	No Inhi	0.7270	0.06974	72	10.42	<.0001	0.05	0.5880	0.8660
BFU-E	E11.5AGM	No Inhi	CFU-GEMM	AoE+E10PSC	With Inhi	21.0419	1476.28	72	0.01	0.9887	0.05	-2921.87	2963.95
BFU-E	E11.5AGM	No Inhi	CFU-GEMM	AoE+E11PSC	No Inhi	0.7637	0.07061	72	10.82	<.0001	0.05	0.6230	0.9045
BFU-E	E11.5AGM	No Inhi	CFU-GEMM	AoE+E11PSC	With Inhi	21.0419	1476.28	72	0.01	0.9887	0.05	-2921.87	2963.95
BFU-E	E11.5AGM	No Inhi	CFU-GEMM	E10.5AGM	No Inhi	0.1484	0.05851	72	2.54	0.0134	0.05	0.03173	0.2650
BFU-E	E11.5AGM	No Inhi	CFU-GEMM	E10.5AGM	With Inhi	21.0419	1476.28	72	0.01	0.9887	0.05	-2921.87	2963.95
BFU-E	E11.5AGM	No Inhi	CFU-GEMM	E11.5AGM	No Inhi	0.1247	0.05814	72	2.15	0.0353	0.05	0.008843	0.2406
BFU-E	E11.5AGM	No Inhi	CFU-GEMM	E11.5AGM	With Inhi	21.0419	1476.28	72	0.01	0.9887	0.05	-2921.87	2963.95
BFU-E	E11.5AGM	No Inhi	CFU-GEMM	HE+E10PSC	No Inhi	0.8676	0.08140	72	10.66	<.0001	0.05	0.7053	1.0299
BFU-E	E11.5AGM	No Inhi	CFU-GEMM	HE+E10PSC	With Inhi	21.0419	1476.28	72	0.01	0.9887	0.05	-2921.87	2963.95
BFU-E	E11.5AGM	No Inhi	CFU-GEMM	HE+E11PSC	No Inhi	0.7983	0.07146	72	11.17	<.0001	0.05	0.6559	0.9408
BFU-E	E11.5AGM	No Inhi	CFU-GEMM	HE+E11PSC	With Inhi	21.0417	1476.15	72	0.01	0.9887	0.05	-2921.60	2963.68
BFU-E	E11.5AGM	No Inhi	CFU-GM	AoE+E10PSC	No Inhi	-0.8498	0.04756	72	-17.87	<.0001	0.05	-0.9446	-0.7550
BFU-E	E11.5AGM	No Inhi	CFU-GM	AoE+E10PSC	With Inhi	21.0418	1476.20	72	0.01	0.9887	0.05	-2921.70	2963.78
BFU-E	E11.5AGM	No Inhi	CFU-GM	AoE+E11PSC	No Inhi	-0.9064	0.04717	72	-19.22	<.0001	0.05	-1.0004	-0.8124
BFU-E	E11.5AGM	No Inhi	CFU-GM	AoE+E11PSC	With Inhi	21.0418	1476.20	72	0.01	0.9887	0.05	-2921.70	2963.78

The GLIMMIX Procedure

Differences of Colony*Condit*Treatm Least Squares Means													
Colony_Type	Condition	Treatment	_Colony_Type	_Condition	_Treatment	Estimate	Standard Error	DF	t Value	Pr > |t|	Alpha	Lower	Upper
BFU-E	E11.5AGM	No Inhi	CFU-GM	E10.5AGM	No Inhi	-1.2929	0.04494	72	-28.77	<.0001	0.05	-1.3825	-1.2033
BFU-E	E11.5AGM	No Inhi	CFU-GM	E10.5AGM	With Inhi	21.0418	1476.20	72	0.01	0.9887	0.05	-2921.71	2963.80
BFU-E	E11.5AGM	No Inhi	CFU-GM	E11.5AGM	No Inhi	-1.3208	0.04481	72	-29.48	<.0001	0.05	-1.4101	-1.2315
BFU-E	E11.5AGM	No Inhi	CFU-GM	E11.5AGM	With Inhi	21.0418	1476.20	72	0.01	0.9887	0.05	-2921.71	2963.80
BFU-E	E11.5AGM	No Inhi	CFU-GM	HE+E10PSC	No Inhi	-0.7606	0.04822	72	-15.77	<.0001	0.05	-0.8567	-0.6644
BFU-E	E11.5AGM	No Inhi	CFU-GM	HE+E10PSC	With Inhi	21.0418	1476.19	72	0.01	0.9887	0.05	-2921.70	2963.78
BFU-E	E11.5AGM	No Inhi	CFU-GM	HE+E11PSC	No Inhi	-0.7089	0.04863	72	-14.58	<.0001	0.05	-0.8058	-0.6119
BFU-E	E11.5AGM	No Inhi	CFU-GM	HE+E11PSC	With Inhi	59.0629	2556.86	72	0.02	0.9816	0.05	-5037.94	5156.07
BFU-E	E11.5AGM	With Inhi	BFU-E	HE+E10PSC	No Inhi	-20.2575	1476.28	72	-0.01	0.9891	0.05	-2963.17	2922.65
BFU-E	E11.5AGM	With Inhi	BFU-E	HE+E10PSC	With Inhi	1.094E-6	2087.78	72	0.00	1.0000	0.05	-4161.91	4161.91
BFU-E	E11.5AGM	With Inhi	BFU-E	HE+E11PSC	No Inhi	-20.1705	1476.28	72	-0.01	0.9891	0.05	-2963.08	2922.74
BFU-E	E11.5AGM	With Inhi	BFU-E	HE+E11PSC	With Inhi	-0.00019	2087.67	72	-0.00	1.0000	0.05	-4161.70	4161.70
BFU-E	E11.5AGM	With Inhi	CFU-GEMM	AoE+E10PSC	No Inhi	-20.3149	1476.28	72	-0.01	0.9891	0.05	-2963.23	2922.60
BFU-E	E11.5AGM	With Inhi	CFU-GEMM	AoE+E10PSC	With Inhi	-1.64E-6	2087.77	72	-0.00	1.0000	0.05	-4161.90	4161.90
BFU-E	E11.5AGM	With Inhi	CFU-GEMM	AoE+E11PSC	No Inhi	-20.2781	1476.28	72	-0.01	0.9891	0.05	-2963.19	2922.63
BFU-E	E11.5AGM	With Inhi	CFU-GEMM	AoE+E11PSC	With Inhi	6.836E-7	2087.78	72	0.00	1.0000	0.05	-4161.91	4161.91
BFU-E	E11.5AGM	With Inhi	CFU-GEMM	E10.5AGM	No Inhi	-20.8935	1476.28	72	-0.01	0.9887	0.05	-2963.80	2922.02
BFU-E	E11.5AGM	With Inhi	CFU-GEMM	E10.5AGM	With Inhi	-2.19E-6	2087.77	72	-0.00	1.0000	0.05	-4161.90	4161.90
BFU-E	E11.5AGM	With Inhi	CFU-GEMM	E11.5AGM	No Inhi	-20.9171	1476.28	72	-0.01	0.9887	0.05	-2963.83	2921.99
BFU-E	E11.5AGM	With Inhi	CFU-GEMM	E11.5AGM	With Inhi	-8.2E-7	2087.77	72	-0.00	1.0000	0.05	-4161.90	4161.90
BFU-E	E11.5AGM	With Inhi	CFU-GEMM	HE+E10PSC	No Inhi	-20.1743	1476.28	72	-0.01	0.9891	0.05	-2963.08	2922.74
BFU-E	E11.5AGM	With Inhi	CFU-GEMM	HE+E10PSC	With Inhi	1.367E-7	2087.77	72	0.00	1.0000	0.05	-4161.90	4161.90
BFU-E	E11.5AGM	With Inhi	CFU-GEMM	HE+E11PSC	No Inhi	-20.2435	1476.28	72	-0.01	0.9891	0.05	-2963.15	2922.67
BFU-E	E11.5AGM	With Inhi	CFU-GEMM	HE+E11PSC	With Inhi	-0.00018	2087.68	72	-0.00	1.0000	0.05	-4161.72	4161.72

The GLIMMIX Procedure

Differences of Colony*Condit*Treatm Least Squares Means													
Colony_Type	Condition	Treatment	_Colony_Type	_Condition	_Treatment	Estimate	Standard Error	DF	t Value	Pr > |t|	Alpha	Lower	Upper
BFU-E	E11.5AGM	With Inhi	CFU-GM	AoE+E10PSC	No Inhi	-21.8917	1476.28	72	-0.01	0.9882	0.05	-2964.80	2921.02
BFU-E	E11.5AGM	With Inhi	CFU-GM	AoE+E10PSC	With Inhi	-0.00011	2087.72	72	-0.00	1.0000	0.05	-4161.79	4161.79
BFU-E	E11.5AGM	With Inhi	CFU-GM	AoE+E11PSC	No Inhi	-21.9483	1476.28	72	-0.01	0.9882	0.05	-2964.86	2920.96
BFU-E	E11.5AGM	With Inhi	CFU-GM	AoE+E11PSC	With Inhi	-0.00011	2087.72	72	-0.00	1.0000	0.05	-4161.78	4161.78
BFU-E	E11.5AGM	With Inhi	CFU-GM	E10.5AGM	No Inhi	-22.3348	1476.28	72	-0.02	0.9880	0.05	-2965.25	2920.58
BFU-E	E11.5AGM	With Inhi	CFU-GM	E10.5AGM	With Inhi	-0.00011	2087.72	72	-0.00	1.0000	0.05	-4161.79	4161.79
BFU-E	E11.5AGM	With Inhi	CFU-GM	E11.5AGM	No Inhi	-22.3627	1476.28	72	-0.02	0.9880	0.05	-2965.27	2920.55
BFU-E	E11.5AGM	With Inhi	CFU-GM	E11.5AGM	With Inhi	-0.00011	2087.72	72	-0.00	1.0000	0.05	-4161.79	4161.79
BFU-E	E11.5AGM	With Inhi	CFU-GM	HE+E10PSC	No Inhi	-21.8024	1476.28	72	-0.01	0.9883	0.05	-2964.71	2921.11
BFU-E	E11.5AGM	With Inhi	CFU-GM	HE+E10PSC	With Inhi	-0.00011	2087.72	72	-0.00	1.0000	0.05	-4161.78	4161.78
BFU-E	E11.5AGM	With Inhi	CFU-GM	HE+E11PSC	No Inhi	-21.7507	1476.28	72	-0.01	0.9883	0.05	-2964.66	2921.16
BFU-E	E11.5AGM	With Inhi	CFU-GM	HE+E11PSC	With Inhi	38.0210	2952.45	72	0.01	0.9898	0.05	-5847.57	5923.61
BFU-E	HE+E10PSC	No Inhi	BFU-E	HE+E10PSC	With Inhi	20.2575	1476.28	72	0.01	0.9891	0.05	-2922.65	2963.17
BFU-E	HE+E10PSC	No Inhi	BFU-E	HE+E11PSC	No Inhi	0.08701	0.08521	72	1.02	0.3106	0.05	-0.08284	0.2569
BFU-E	HE+E10PSC	No Inhi	BFU-E	HE+E11PSC	With Inhi	20.2573	1476.14	72	0.01	0.9891	0.05	-2922.37	2962.88
BFU-E	HE+E10PSC	No Inhi	CFU-GEMM	AoE+E10PSC	No Inhi	-0.05735	0.08216	72	-0.70	0.4874	0.05	-0.2211	0.1064
BFU-E	HE+E10PSC	No Inhi	CFU-GEMM	AoE+E10PSC	With Inhi	20.2575	1476.28	72	0.01	0.9891	0.05	-2922.65	2963.17
BFU-E	HE+E10PSC	No Inhi	CFU-GEMM	AoE+E11PSC	No Inhi	-0.02062	0.08291	72	-0.25	0.8043	0.05	-0.1859	0.1447
BFU-E	HE+E10PSC	No Inhi	CFU-GEMM	AoE+E11PSC	With Inhi	20.2575	1476.28	72	0.01	0.9891	0.05	-2922.65	2963.17
BFU-E	HE+E10PSC	No Inhi	CFU-GEMM	E10.5AGM	No Inhi	-0.6360	0.07287	72	-8.73	<.0001	0.05	-0.7813	-0.4907
BFU-E	HE+E10PSC	No Inhi	CFU-GEMM	E10.5AGM	With Inhi	20.2575	1476.28	72	0.01	0.9891	0.05	-2922.65	2963.16
BFU-E	HE+E10PSC	No Inhi	CFU-GEMM	E11.5AGM	No Inhi	-0.6596	0.07258	72	-9.09	<.0001	0.05	-0.8043	-0.5149
BFU-E	HE+E10PSC	No Inhi	CFU-GEMM	E11.5AGM	With Inhi	20.2575	1476.28	72	0.01	0.9891	0.05	-2922.65	2963.17
BFU-E	HE+E10PSC	No Inhi	CFU-GEMM	HE+E10PSC	No Inhi	0.08323	0.09227	72	0.90	0.3701	0.05	-0.1007	0.2672

The GLIMMIX Procedure

Differences of Colony*Condit*Treatm Least Squares Means													
Colony_Type	Condition	Treatment	_Colony_Type	_Condition	_Treatment	Estimate	Standard Error	DF	t Value	Pr > |t|	Alpha	Lower	Upper
BFU-E	HE+E10PSC	No Inhi	CFU-GEMM	HE+E10PSC	With Inhi	20.2575	1476.28	72	0.01	0.9891	0.05	-2922.65	2963.17
BFU-E	HE+E10PSC	No Inhi	CFU-GEMM	HE+E11PSC	No Inhi	0.01399	0.08363	72	0.17	0.8676	0.05	-0.1527	0.1807
BFU-E	HE+E10PSC	No Inhi	CFU-GEMM	HE+E11PSC	With Inhi	20.2573	1476.15	72	0.01	0.9891	0.05	-2922.39	2962.90
BFU-E	HE+E10PSC	No Inhi	CFU-GM	AoE+E10PSC	No Inhi	-1.6341	0.06442	72	-25.37	<.0001	0.05	-1.7625	-1.5057
BFU-E	HE+E10PSC	No Inhi	CFU-GM	AoE+E10PSC	With Inhi	20.2574	1476.20	72	0.01	0.9891	0.05	-2922.49	2963.00
BFU-E	HE+E10PSC	No Inhi	CFU-GM	AoE+E11PSC	No Inhi	-1.6908	0.06413	72	-26.37	<.0001	0.05	-1.8186	-1.5629
BFU-E	HE+E10PSC	No Inhi	CFU-GM	AoE+E11PSC	With Inhi	20.2574	1476.20	72	0.01	0.9891	0.05	-2922.48	2963.00
BFU-E	HE+E10PSC	No Inhi	CFU-GM	E10.5AGM	No Inhi	-2.0773	0.06251	72	-33.23	<.0001	0.05	-2.2019	-1.9527
BFU-E	HE+E10PSC	No Inhi	CFU-GM	E10.5AGM	With Inhi	20.2574	1476.20	72	0.01	0.9891	0.05	-2922.50	2963.01
BFU-E	HE+E10PSC	No Inhi	CFU-GM	E11.5AGM	No Inhi	-2.1051	0.06241	72	-33.73	<.0001	0.05	-2.2296	-1.9807
BFU-E	HE+E10PSC	No Inhi	CFU-GM	E11.5AGM	With Inhi	20.2574	1476.20	72	0.01	0.9891	0.05	-2922.50	2963.01
BFU-E	HE+E10PSC	No Inhi	CFU-GM	HE+E10PSC	No Inhi	-1.5449	0.06491	72	-23.80	<.0001	0.05	-1.6743	-1.4155
BFU-E	HE+E10PSC	No Inhi	CFU-GM	HE+E10PSC	With Inhi	20.2574	1476.19	72	0.01	0.9891	0.05	-2922.48	2963.00
BFU-E	HE+E10PSC	No Inhi	CFU-GM	HE+E11PSC	No Inhi	-1.4932	0.06521	72	-22.90	<.0001	0.05	-1.6232	-1.3632
BFU-E	HE+E10PSC	No Inhi	CFU-GM	HE+E11PSC	With Inhi	58.2785	2556.86	72	0.02	0.9819	0.05	-5038.73	5155.28
BFU-E	HE+E10PSC	With Inhi	BFU-E	HE+E11PSC	No Inhi	-20.1705	1476.28	72	-0.01	0.9891	0.05	-2963.08	2922.74
BFU-E	HE+E10PSC	With Inhi	BFU-E	HE+E11PSC	With Inhi	-0.00019	2087.67	72	-0.00	1.0000	0.05	-4161.70	4161.70
BFU-E	HE+E10PSC	With Inhi	CFU-GEMM	AoE+E10PSC	No Inhi	-20.3149	1476.28	72	-0.01	0.9891	0.05	-2963.23	2922.60
BFU-E	HE+E10PSC	With Inhi	CFU-GEMM	AoE+E10PSC	With Inhi	-2.73E-6	2087.78	72	-0.00	1.0000	0.05	-4161.90	4161.90
BFU-E	HE+E10PSC	With Inhi	CFU-GEMM	AoE+E11PSC	No Inhi	-20.2781	1476.28	72	-0.01	0.9891	0.05	-2963.19	2922.63
BFU-E	HE+E10PSC	With Inhi	CFU-GEMM	AoE+E11PSC	With Inhi	-4.1E-7	2087.78	72	-0.00	1.0000	0.05	-4161.91	4161.91
BFU-E	HE+E10PSC	With Inhi	CFU-GEMM	E10.5AGM	No Inhi	-20.8935	1476.28	72	-0.01	0.9887	0.05	-2963.81	2922.02
BFU-E	HE+E10PSC	With Inhi	CFU-GEMM	E10.5AGM	With Inhi	-3.28E-6	2087.77	72	-0.00	1.0000	0.05	-4161.90	4161.90
BFU-E	HE+E10PSC	With Inhi	CFU-GEMM	E11.5AGM	No Inhi	-20.9171	1476.28	72	-0.01	0.9887	0.05	-2963.83	2922.00

The GLIMMIX Procedure

Differences of Colony*Condit*Treatm Least Squares Means													
Colony_Type	Condition	Treatment	_Colony_Type	_Condition	_Treatment	Estimate	Standard Error	DF	t Value	Pr > |t|	Alpha	Lower	Upper
BFU-E	HE+E10PSC	With Inhi	CFU-GEMM	E11.5AGM	With Inhi	-1.91E-6	2087.78	72	-0.00	1.0000	0.05	-4161.90	4161.90
BFU-E	HE+E10PSC	With Inhi	CFU-GEMM	HE+E10PSC	No Inhi	-20.1743	1476.28	72	-0.01	0.9891	0.05	-2963.09	2922.74
BFU-E	HE+E10PSC	With Inhi	CFU-GEMM	HE+E10PSC	With Inhi	-9.57E-7	2087.77	72	-0.00	1.0000	0.05	-4161.90	4161.90
BFU-E	HE+E10PSC	With Inhi	CFU-GEMM	HE+E11PSC	No Inhi	-20.2435	1476.28	72	-0.01	0.9891	0.05	-2963.16	2922.67
BFU-E	HE+E10PSC	With Inhi	CFU-GEMM	HE+E11PSC	With Inhi	-0.00018	2087.68	72	-0.00	1.0000	0.05	-4161.72	4161.72
BFU-E	HE+E10PSC	With Inhi	CFU-GM	AoE+E10PSC	No Inhi	-21.8917	1476.28	72	-0.01	0.9882	0.05	-2964.80	2921.02
BFU-E	HE+E10PSC	With Inhi	CFU-GM	AoE+E10PSC	With Inhi	-0.00011	2087.72	72	-0.00	1.0000	0.05	-4161.79	4161.79
BFU-E	HE+E10PSC	With Inhi	CFU-GM	AoE+E11PSC	No Inhi	-21.9483	1476.28	72	-0.01	0.9882	0.05	-2964.86	2920.96
BFU-E	HE+E10PSC	With Inhi	CFU-GM	AoE+E11PSC	With Inhi	-0.00012	2087.72	72	-0.00	1.0000	0.05	-4161.79	4161.79
BFU-E	HE+E10PSC	With Inhi	CFU-GM	E10.5AGM	No Inhi	-22.3348	1476.28	72	-0.02	0.9880	0.05	-2965.25	2920.58
BFU-E	HE+E10PSC	With Inhi	CFU-GM	E10.5AGM	With Inhi	-0.00011	2087.72	72	-0.00	1.0000	0.05	-4161.79	4161.79
BFU-E	HE+E10PSC	With Inhi	CFU-GM	E11.5AGM	No Inhi	-22.3627	1476.28	72	-0.02	0.9880	0.05	-2965.28	2920.55
BFU-E	HE+E10PSC	With Inhi	CFU-GM	E11.5AGM	With Inhi	-0.00011	2087.72	72	-0.00	1.0000	0.05	-4161.79	4161.79
BFU-E	HE+E10PSC	With Inhi	CFU-GM	HE+E10PSC	No Inhi	-21.8024	1476.28	72	-0.01	0.9883	0.05	-2964.71	2921.11
BFU-E	HE+E10PSC	With Inhi	CFU-GM	HE+E10PSC	With Inhi	-0.00011	2087.72	72	-0.00	1.0000	0.05	-4161.78	4161.78
BFU-E	HE+E10PSC	With Inhi	CFU-GM	HE+E11PSC	No Inhi	-21.7507	1476.28	72	-0.01	0.9883	0.05	-2964.66	2921.16
BFU-E	HE+E10PSC	With Inhi	CFU-GM	HE+E11PSC	With Inhi	38.0210	2952.45	72	0.01	0.9898	0.05	-5847.57	5923.61
BFU-E	HE+E11PSC	No Inhi	BFU-E	HE+E11PSC	With Inhi	20.1703	1476.14	72	0.01	0.9891	0.05	-2922.45	2962.80
BFU-E	HE+E11PSC	No Inhi	CFU-GEMM	AoE+E10PSC	No Inhi	-0.1444	0.08406	72	-1.72	0.0902	0.05	-0.3119	0.02321
BFU-E	HE+E11PSC	No Inhi	CFU-GEMM	AoE+E10PSC	With Inhi	20.1705	1476.28	72	0.01	0.9891	0.05	-2922.74	2963.08
BFU-E	HE+E11PSC	No Inhi	CFU-GEMM	AoE+E11PSC	No Inhi	-0.1076	0.08479	72	-1.27	0.2084	0.05	-0.2767	0.06139
BFU-E	HE+E11PSC	No Inhi	CFU-GEMM	AoE+E11PSC	With Inhi	20.1705	1476.28	72	0.01	0.9891	0.05	-2922.74	2963.08
BFU-E	HE+E11PSC	No Inhi	CFU-GEMM	E10.5AGM	No Inhi	-0.7230	0.07501	72	-9.64	<.0001	0.05	-0.8725	-0.5735
BFU-E	HE+E11PSC	No Inhi	CFU-GEMM	E10.5AGM	With Inhi	20.1705	1476.28	72	0.01	0.9891	0.05	-2922.74	2963.08

The GLIMMIX Procedure

Differences of Colony*Condit*Treatm Least Squares Means													
**Colony_Type**	**Condition**	**Treatment**	**_Colony_Type**	**_Condition**	**_Treatment**	**Estimate**	**Standard Error**	**DF**	**t Value**	**Pr > |t|**	**Alpha**	**Lower**	**Upper**
BFU-E	HE+E11PSC	No Inhi	CFU-GEMM	E11.5AGM	No Inhi	-0.7466	0.07472	72	-9.99	<.0001	0.05	-0.8956	-0.5977
BFU-E	HE+E11PSC	No Inhi	CFU-GEMM	E11.5AGM	With Inhi	20.1705	1476.28	72	0.01	0.9891	0.05	-2922.74	2963.08
BFU-E	HE+E11PSC	No Inhi	CFU-GEMM	HE+E10PSC	No Inhi	-0.00378	0.09397	72	-0.04	0.9680	0.05	-0.1911	0.1835
BFU-E	HE+E11PSC	No Inhi	CFU-GEMM	HE+E10PSC	With Inhi	20.1705	1476.28	72	0.01	0.9891	0.05	-2922.74	2963.08
BFU-E	HE+E11PSC	No Inhi	CFU-GEMM	HE+E11PSC	No Inhi	-0.07303	0.08549	72	-0.85	0.3958	0.05	-0.2435	0.09740
BFU-E	HE+E11PSC	No Inhi	CFU-GEMM	HE+E11PSC	With Inhi	20.1703	1476.15	72	0.01	0.9891	0.05	-2922.47	2962.81
BFU-E	HE+E11PSC	No Inhi	CFU-GM	AoE+E10PSC	No Inhi	-1.7211	0.06682	72	-25.76	<.0001	0.05	-1.8544	-1.5879
BFU-E	HE+E11PSC	No Inhi	CFU-GM	AoE+E10PSC	With Inhi	20.1704	1476.20	72	0.01	0.9891	0.05	-2922.57	2962.91
BFU-E	HE+E11PSC	No Inhi	CFU-GM	AoE+E11PSC	No Inhi	-1.7778	0.06654	72	-26.72	<.0001	0.05	-1.9104	-1.6451
BFU-E	HE+E11PSC	No Inhi	CFU-GM	AoE+E11PSC	With Inhi	20.1704	1476.20	72	0.01	0.9891	0.05	-2922.57	2962.91
BFU-E	HE+E11PSC	No Inhi	CFU-GM	E10.5AGM	No Inhi	-2.1643	0.06498	72	-33.31	<.0001	0.05	-2.2938	-2.0347
BFU-E	HE+E11PSC	No Inhi	CFU-GM	E10.5AGM	With Inhi	20.1704	1476.20	72	0.01	0.9891	0.05	-2922.58	2962.92
BFU-E	HE+E11PSC	No Inhi	CFU-GM	E11.5AGM	No Inhi	-2.1922	0.06489	72	-33.78	<.0001	0.05	-2.3215	-2.0628
BFU-E	HE+E11PSC	No Inhi	CFU-GM	E11.5AGM	With Inhi	20.1704	1476.20	72	0.01	0.9891	0.05	-2922.58	2962.92
BFU-E	HE+E11PSC	No Inhi	CFU-GM	HE+E10PSC	No Inhi	-1.6319	0.06730	72	-24.25	<.0001	0.05	-1.7661	-1.4978
BFU-E	HE+E11PSC	No Inhi	CFU-GM	HE+E10PSC	With Inhi	20.1704	1476.19	72	0.01	0.9891	0.05	-2922.57	2962.91
BFU-E	HE+E11PSC	No Inhi	CFU-GM	HE+E11PSC	No Inhi	-1.5802	0.06759	72	-23.38	<.0001	0.05	-1.7150	-1.4455
BFU-E	HE+E11PSC	No Inhi	CFU-GM	HE+E11PSC	With Inhi	58.1915	2556.86	72	0.02	0.9819	0.05	-5038.81	5155.20
BFU-E	HE+E11PSC	With Inhi	CFU-GEMM	AoE+E10PSC	No Inhi	-20.3147	1476.14	72	-0.01	0.9891	0.05	-2962.94	2922.31
BFU-E	HE+E11PSC	With Inhi	CFU-GEMM	AoE+E10PSC	With Inhi	0.000191	2087.67	72	0.00	1.0000	0.05	-4161.70	4161.70
BFU-E	HE+E11PSC	With Inhi	CFU-GEMM	AoE+E11PSC	No Inhi	-20.2779	1476.14	72	-0.01	0.9891	0.05	-2962.90	2922.35
BFU-E	HE+E11PSC	With Inhi	CFU-GEMM	AoE+E11PSC	With Inhi	0.000194	2087.67	72	0.00	1.0000	0.05	-4161.70	4161.70
BFU-E	HE+E11PSC	With Inhi	CFU-GEMM	E10.5AGM	No Inhi	-20.8933	1476.14	72	-0.01	0.9887	0.05	-2963.52	2921.73
BFU-E	HE+E11PSC	With Inhi	CFU-GEMM	E10.5AGM	With Inhi	0.000191	2087.67	72	0.00	1.0000	0.05	-4161.70	4161.70

The GLIMMIX Procedure

Differences of Colony*Condit*Treatm Least Squares Means													
Colony_Type	Condition	Treatment	_Colony_Type	_Condition	_Treatment	Estimate	Standard Error	DF	t Value	Pr > |t|	Alpha	Lower	Upper
BFU-E	HE+E11PSC	With Inhi	CFU-GEMM	E11.5AGM	No Inhi	-20.9169	1476.14	72	-0.01	0.9887	0.05	-2963.54	2921.71
BFU-E	HE+E11PSC	With Inhi	CFU-GEMM	E11.5AGM	With Inhi	0.000192	2087.67	72	0.00	1.0000	0.05	-4161.70	4161.70
BFU-E	HE+E11PSC	With Inhi	CFU-GEMM	HE+E10PSC	No Inhi	-20.1741	1476.14	72	-0.01	0.9891	0.05	-2962.80	2922.45
BFU-E	HE+E11PSC	With Inhi	CFU-GEMM	HE+E10PSC	With Inhi	0.000193	2087.67	72	0.00	1.0000	0.05	-4161.70	4161.70
BFU-E	HE+E11PSC	With Inhi	CFU-GEMM	HE+E11PSC	No Inhi	-20.2433	1476.14	72	-0.01	0.9891	0.05	-2962.87	2922.38
BFU-E	HE+E11PSC	With Inhi	CFU-GEMM	HE+E11PSC	With Inhi	0.000012	2087.58	72	0.00	1.0000	0.05	-4161.51	4161.51
BFU-E	HE+E11PSC	With Inhi	CFU-GM	AoE+E10PSC	No Inhi	-21.8915	1476.14	72	-0.01	0.9882	0.05	-2964.52	2920.73
BFU-E	HE+E11PSC	With Inhi	CFU-GM	AoE+E10PSC	With Inhi	0.000082	2087.61	72	0.00	1.0000	0.05	-4161.58	4161.58
BFU-E	HE+E11PSC	With Inhi	CFU-GM	AoE+E11PSC	No Inhi	-21.9481	1476.14	72	-0.01	0.9882	0.05	-2964.57	2920.68
BFU-E	HE+E11PSC	With Inhi	CFU-GM	AoE+E11PSC	With Inhi	0.000079	2087.61	72	0.00	1.0000	0.05	-4161.58	4161.58
BFU-E	HE+E11PSC	With Inhi	CFU-GM	E10.5AGM	No Inhi	-22.3346	1476.14	72	-0.02	0.9880	0.05	-2964.96	2920.29
BFU-E	HE+E11PSC	With Inhi	CFU-GM	E10.5AGM	With Inhi	0.000085	2087.62	72	0.00	1.0000	0.05	-4161.59	4161.59
BFU-E	HE+E11PSC	With Inhi	CFU-GM	E11.5AGM	No Inhi	-22.3625	1476.14	72	-0.02	0.9880	0.05	-2964.99	2920.26
BFU-E	HE+E11PSC	With Inhi	CFU-GM	E11.5AGM	With Inhi	0.000083	2087.62	72	0.00	1.0000	0.05	-4161.59	4161.59
BFU-E	HE+E11PSC	With Inhi	CFU-GM	HE+E10PSC	No Inhi	-21.8022	1476.14	72	-0.01	0.9883	0.05	-2964.43	2920.82
BFU-E	HE+E11PSC	With Inhi	CFU-GM	HE+E10PSC	With Inhi	0.000081	2087.61	72	0.00	1.0000	0.05	-4161.58	4161.58
BFU-E	HE+E11PSC	With Inhi	CFU-GM	HE+E11PSC	No Inhi	-21.7505	1476.14	72	-0.01	0.9883	0.05	-2964.38	2920.87
BFU-E	HE+E11PSC	With Inhi	CFU-GM	HE+E11PSC	With Inhi	38.0212	2952.37	72	0.01	0.9898	0.05	-5847.42	5923.47
CFU-GEMM	AoE+E10PSC	No Inhi	CFU-GEMM	AoE+E10PSC	With Inhi	20.3149	1476.28	72	0.01	0.9891	0.05	-2922.59	2963.22
CFU-GEMM	AoE+E10PSC	No Inhi	CFU-GEMM	AoE+E11PSC	No Inhi	0.03673	0.08173	72	0.45	0.6545	0.05	-0.1262	0.1997
CFU-GEMM	AoE+E10PSC	No Inhi	CFU-GEMM	AoE+E11PSC	With Inhi	20.3149	1476.28	72	0.01	0.9891	0.05	-2922.60	2963.23
CFU-GEMM	AoE+E10PSC	No Inhi	CFU-GEMM	E10.5AGM	No Inhi	-0.5786	0.07153	72	-8.09	<.0001	0.05	-0.7212	-0.4360
CFU-GEMM	AoE+E10PSC	No Inhi	CFU-GEMM	E10.5AGM	With Inhi	20.3149	1476.28	72	0.01	0.9891	0.05	-2922.59	2963.22
CFU-GEMM	AoE+E10PSC	No Inhi	CFU-GEMM	E11.5AGM	No Inhi	-0.6023	0.07123	72	-8.45	<.0001	0.05	-0.7443	-0.4603

The GLIMMIX Procedure

Differences of Colony*Condit*Treatm Least Squares Means													
Colony_Type	Condition	Treatment	_Colony_Type	_Condition	_Treatment	Estimate	Standard Error	DF	t Value	Pr > |t|	Alpha	Lower	Upper
CFU-GEMM	AoE+E10PSC	No Inhi	CFU-GEMM	E11.5AGM	With Inhi	20.3149	1476.28	72	0.01	0.9891	0.05	-2922.59	2963.22
CFU-GEMM	AoE+E10PSC	No Inhi	CFU-GEMM	HE+E10PSC	No Inhi	0.1406	0.09122	72	1.54	0.1277	0.05	-0.04126	0.3224
CFU-GEMM	AoE+E10PSC	No Inhi	CFU-GEMM	HE+E10PSC	With Inhi	20.3149	1476.28	72	0.01	0.9891	0.05	-2922.59	2963.22
CFU-GEMM	AoE+E10PSC	No Inhi	CFU-GEMM	HE+E11PSC	No Inhi	0.07134	0.08246	72	0.87	0.3899	0.05	-0.09305	0.2357
CFU-GEMM	AoE+E10PSC	No Inhi	CFU-GEMM	HE+E11PSC	With Inhi	20.3147	1476.15	72	0.01	0.9891	0.05	-2922.33	2962.96
CFU-GEMM	AoE+E10PSC	No Inhi	CFU-GM	AoE+E10PSC	No Inhi	-1.5768	0.06290	72	-25.07	<.0001	0.05	-1.7022	-1.4514
CFU-GEMM	AoE+E10PSC	No Inhi	CFU-GM	AoE+E10PSC	With Inhi	20.3148	1476.20	72	0.01	0.9891	0.05	-2922.43	2963.06
CFU-GEMM	AoE+E10PSC	No Inhi	CFU-GM	AoE+E11PSC	No Inhi	-1.6334	0.06260	72	-26.09	<.0001	0.05	-1.7582	-1.5086
CFU-GEMM	AoE+E10PSC	No Inhi	CFU-GM	AoE+E11PSC	With Inhi	20.3148	1476.20	72	0.01	0.9891	0.05	-2922.43	2963.06
CFU-GEMM	AoE+E10PSC	No Inhi	CFU-GM	E10.5AGM	No Inhi	-2.0199	0.06094	72	-33.15	<.0001	0.05	-2.1414	-1.8984
CFU-GEMM	AoE+E10PSC	No Inhi	CFU-GM	E10.5AGM	With Inhi	20.3148	1476.20	72	0.01	0.9891	0.05	-2922.44	2963.07
CFU-GEMM	AoE+E10PSC	No Inhi	CFU-GM	E11.5AGM	No Inhi	-2.0478	0.06084	72	-33.66	<.0001	0.05	-2.1691	-1.9265
CFU-GEMM	AoE+E10PSC	No Inhi	CFU-GM	E11.5AGM	With Inhi	20.3148	1476.20	72	0.01	0.9891	0.05	-2922.44	2963.07
CFU-GEMM	AoE+E10PSC	No Inhi	CFU-GM	HE+E10PSC	No Inhi	-1.4875	0.06340	72	-23.46	<.0001	0.05	-1.6139	-1.3612
CFU-GEMM	AoE+E10PSC	No Inhi	CFU-GM	HE+E10PSC	With Inhi	20.3148	1476.19	72	0.01	0.9891	0.05	-2922.42	2963.05
CFU-GEMM	AoE+E10PSC	No Inhi	CFU-GM	HE+E11PSC	No Inhi	-1.4359	0.06371	72	-22.54	<.0001	0.05	-1.5629	-1.3089
CFU-GEMM	AoE+E10PSC	No Inhi	CFU-GM	HE+E11PSC	With Inhi	58.3359	2556.86	72	0.02	0.9819	0.05	-5038.67	5155.34
CFU-GEMM	AoE+E10PSC	With Inhi	CFU-GEMM	AoE+E11PSC	No Inhi	-20.2781	1476.28	72	-0.01	0.9891	0.05	-2963.19	2922.63
CFU-GEMM	AoE+E10PSC	With Inhi	CFU-GEMM	AoE+E11PSC	With Inhi	2.324E-6	2087.77	72	0.00	1.0000	0.05	-4161.90	4161.90
CFU-GEMM	AoE+E10PSC	With Inhi	CFU-GEMM	E10.5AGM	No Inhi	-20.8935	1476.28	72	-0.01	0.9887	0.05	-2963.80	2922.01
CFU-GEMM	AoE+E10PSC	With Inhi	CFU-GEMM	E10.5AGM	With Inhi	-5.47E-7	2087.77	72	-0.00	1.0000	0.05	-4161.90	4161.90
CFU-GEMM	AoE+E10PSC	With Inhi	CFU-GEMM	E11.5AGM	No Inhi	-20.9171	1476.28	72	-0.01	0.9887	0.05	-2963.82	2921.99
CFU-GEMM	AoE+E10PSC	With Inhi	CFU-GEMM	E11.5AGM	With Inhi	8.203E-7	2087.77	72	0.00	1.0000	0.05	-4161.90	4161.90
CFU-GEMM	AoE+E10PSC	With Inhi	CFU-GEMM	HE+E10PSC	No Inhi	-20.1743	1476.28	72	-0.01	0.9891	0.05	-2963.08	2922.73

The GLIMMIX Procedure

Differences of Colony*Condit*Treatm Least Squares Means													
Colony_Type	Condition	Treatment	_Colony_Type	_Condition	_Treatment	Estimate	Standard Error	DF	t Value	Pr > |t|	Alpha	Lower	Upper
CFU-GEMM	AoE+E10PSC	With Inhi	CFU-GEMM	HE+E10PSC	With Inhi	1.777E-6	2087.77	72	0.00	1.0000	0.05	-4161.90	4161.90
CFU-GEMM	AoE+E10PSC	With Inhi	CFU-GEMM	HE+E11PSC	No Inhi	-20.2435	1476.28	72	-0.01	0.9891	0.05	-2963.15	2922.66
CFU-GEMM	AoE+E10PSC	With Inhi	CFU-GEMM	HE+E11PSC	With Inhi	-0.00018	2087.68	72	-0.00	1.0000	0.05	-4161.71	4161.71
CFU-GEMM	AoE+E10PSC	With Inhi	CFU-GM	AoE+E10PSC	No Inhi	-21.8917	1476.28	72	-0.01	0.9882	0.05	-2964.80	2921.02
CFU-GEMM	AoE+E10PSC	With Inhi	CFU-GM	AoE+E10PSC	With Inhi	-0.00011	2087.71	72	-0.00	1.0000	0.05	-4161.78	4161.78
CFU-GEMM	AoE+E10PSC	With Inhi	CFU-GM	AoE+E11PSC	No Inhi	-21.9483	1476.28	72	-0.01	0.9882	0.05	-2964.86	2920.96
CFU-GEMM	AoE+E10PSC	With Inhi	CFU-GM	AoE+E11PSC	With Inhi	-0.00011	2087.71	72	-0.00	1.0000	0.05	-4161.78	4161.78
CFU-GEMM	AoE+E10PSC	With Inhi	CFU-GM	E10.5AGM	No Inhi	-22.3348	1476.28	72	-0.02	0.9880	0.05	-2965.24	2920.57
CFU-GEMM	AoE+E10PSC	With Inhi	CFU-GM	E10.5AGM	With Inhi	-0.00011	2087.72	72	-0.00	1.0000	0.05	-4161.79	4161.79
CFU-GEMM	AoE+E10PSC	With Inhi	CFU-GM	E11.5AGM	No Inhi	-22.3627	1476.28	72	-0.02	0.9880	0.05	-2965.27	2920.55
CFU-GEMM	AoE+E10PSC	With Inhi	CFU-GM	E11.5AGM	With Inhi	-0.00011	2087.72	72	-0.00	1.0000	0.05	-4161.79	4161.79
CFU-GEMM	AoE+E10PSC	With Inhi	CFU-GM	HE+E10PSC	No Inhi	-21.8024	1476.28	72	-0.01	0.9883	0.05	-2964.71	2921.11
CFU-GEMM	AoE+E10PSC	With Inhi	CFU-GM	HE+E10PSC	With Inhi	-0.00011	2087.71	72	-0.00	1.0000	0.05	-4161.78	4161.78
CFU-GEMM	AoE+E10PSC	With Inhi	CFU-GM	HE+E11PSC	No Inhi	-21.7507	1476.28	72	-0.01	0.9883	0.05	-2964.66	2921.16
CFU-GEMM	AoE+E10PSC	With Inhi	CFU-GM	HE+E11PSC	With Inhi	38.0210	2952.44	72	0.01	0.9898	0.05	-5847.57	5923.61
CFU-GEMM	AoE+E11PSC	No Inhi	CFU-GEMM	AoE+E11PSC	With Inhi	20.2781	1476.28	72	0.01	0.9891	0.05	-2922.63	2963.19
CFU-GEMM	AoE+E11PSC	No Inhi	CFU-GEMM	E10.5AGM	No Inhi	-0.6154	0.07238	72	-8.50	<.0001	0.05	-0.7597	-0.4711
CFU-GEMM	AoE+E11PSC	No Inhi	CFU-GEMM	E10.5AGM	With Inhi	20.2781	1476.28	72	0.01	0.9891	0.05	-2922.63	2963.19
CFU-GEMM	AoE+E11PSC	No Inhi	CFU-GEMM	E11.5AGM	No Inhi	-0.6390	0.07209	72	-8.86	<.0001	0.05	-0.7827	-0.4953
CFU-GEMM	AoE+E11PSC	No Inhi	CFU-GEMM	E11.5AGM	With Inhi	20.2781	1476.28	72	0.01	0.9891	0.05	-2922.63	2963.19
CFU-GEMM	AoE+E11PSC	No Inhi	CFU-GEMM	HE+E10PSC	No Inhi	0.1038	0.09189	72	1.13	0.2621	0.05	-0.07932	0.2870
CFU-GEMM	AoE+E11PSC	No Inhi	CFU-GEMM	HE+E10PSC	With Inhi	20.2781	1476.28	72	0.01	0.9891	0.05	-2922.63	2963.19
CFU-GEMM	AoE+E11PSC	No Inhi	CFU-GEMM	HE+E11PSC	No Inhi	0.03461	0.08320	72	0.42	0.6787	0.05	-0.1313	0.2005
CFU-GEMM	AoE+E11PSC	No Inhi	CFU-GEMM	HE+E11PSC	With Inhi	20.2780	1476.15	72	0.01	0.9891	0.05	-2922.37	2962.92

The GLIMMIX Procedure

Differences of Colony*Condit*Treatm Least Squares Means													
Colony_Type	Condition	Treatment	_Colony_Type	_Condition	_Treatment	Estimate	Standard Error	DF	t Value	Pr > |t|	Alpha	Lower	Upper
CFU-GEMM	AoE+E11PSC	No Inhi	CFU-GM	AoE+E10PSC	No Inhi	-1.6135	0.06387	72	-25.26	<.0001	0.05	-1.7408	-1.4862
CFU-GEMM	AoE+E11PSC	No Inhi	CFU-GM	AoE+E10PSC	With Inhi	20.2780	1476.20	72	0.01	0.9891	0.05	-2922.46	2963.02
CFU-GEMM	AoE+E11PSC	No Inhi	CFU-GM	AoE+E11PSC	No Inhi	-1.6701	0.06357	72	-26.27	<.0001	0.05	-1.7969	-1.5434
CFU-GEMM	AoE+E11PSC	No Inhi	CFU-GM	AoE+E11PSC	With Inhi	20.2780	1476.20	72	0.01	0.9891	0.05	-2922.46	2963.02
CFU-GEMM	AoE+E11PSC	No Inhi	CFU-GM	E10.5AGM	No Inhi	-2.0566	0.06194	72	-33.20	<.0001	0.05	-2.1801	-1.9332
CFU-GEMM	AoE+E11PSC	No Inhi	CFU-GM	E10.5AGM	With Inhi	20.2780	1476.20	72	0.01	0.9891	0.05	-2922.48	2963.03
CFU-GEMM	AoE+E11PSC	No Inhi	CFU-GM	E11.5AGM	No Inhi	-2.0845	0.06184	72	-33.71	<.0001	0.05	-2.2078	-1.9613
CFU-GEMM	AoE+E11PSC	No Inhi	CFU-GM	E11.5AGM	With Inhi	20.2780	1476.20	72	0.01	0.9891	0.05	-2922.48	2963.03
CFU-GEMM	AoE+E11PSC	No Inhi	CFU-GM	HE+E10PSC	No Inhi	-1.5243	0.06436	72	-23.68	<.0001	0.05	-1.6526	-1.3960
CFU-GEMM	AoE+E11PSC	No Inhi	CFU-GM	HE+E10PSC	With Inhi	20.2780	1476.19	72	0.01	0.9891	0.05	-2922.46	2963.02
CFU-GEMM	AoE+E11PSC	No Inhi	CFU-GM	HE+E11PSC	No Inhi	-1.4726	0.06466	72	-22.77	<.0001	0.05	-1.6015	-1.3437
CFU-GEMM	AoE+E11PSC	No Inhi	CFU-GM	HE+E11PSC	With Inhi	58.2991	2556.86	72	0.02	0.9819	0.05	-5038.71	5155.30
CFU-GEMM	AoE+E11PSC	With Inhi	CFU-GEMM	E10.5AGM	No Inhi	-20.8935	1476.28	72	-0.01	0.9887	0.05	-2963.81	2922.02
CFU-GEMM	AoE+E11PSC	With Inhi	CFU-GEMM	E10.5AGM	With Inhi	-2.87E-6	2087.77	72	-0.00	1.0000	0.05	-4161.90	4161.90
CFU-GEMM	AoE+E11PSC	With Inhi	CFU-GEMM	E11.5AGM	No Inhi	-20.9171	1476.28	72	-0.01	0.9887	0.05	-2963.83	2922.00
CFU-GEMM	AoE+E11PSC	With Inhi	CFU-GEMM	E11.5AGM	With Inhi	-1.5E-6	2087.78	72	-0.00	1.0000	0.05	-4161.90	4161.90
CFU-GEMM	AoE+E11PSC	With Inhi	CFU-GEMM	HE+E10PSC	No Inhi	-20.1743	1476.28	72	-0.01	0.9891	0.05	-2963.09	2922.74
CFU-GEMM	AoE+E11PSC	With Inhi	CFU-GEMM	HE+E10PSC	With Inhi	-5.47E-7	2087.77	72	-0.00	1.0000	0.05	-4161.90	4161.90
CFU-GEMM	AoE+E11PSC	With Inhi	CFU-GEMM	HE+E11PSC	No Inhi	-20.2435	1476.28	72	-0.01	0.9891	0.05	-2963.16	2922.67
CFU-GEMM	AoE+E11PSC	With Inhi	CFU-GEMM	HE+E11PSC	With Inhi	-0.00018	2087.68	72	-0.00	1.0000	0.05	-4161.72	4161.72
CFU-GEMM	AoE+E11PSC	With Inhi	CFU-GM	AoE+E10PSC	No Inhi	-21.8917	1476.28	72	-0.01	0.9882	0.05	-2964.80	2921.02
CFU-GEMM	AoE+E11PSC	With Inhi	CFU-GM	AoE+E10PSC	With Inhi	-0.00011	2087.72	72	-0.00	1.0000	0.05	-4161.79	4161.79
CFU-GEMM	AoE+E11PSC	With Inhi	CFU-GM	AoE+E11PSC	No Inhi	-21.9483	1476.28	72	-0.01	0.9882	0.05	-2964.86	2920.96
CFU-GEMM	AoE+E11PSC	With Inhi	CFU-GM	AoE+E11PSC	With Inhi	-0.00011	2087.72	72	-0.00	1.0000	0.05	-4161.79	4161.79

The GLIMMIX Procedure

Differences of Colony*Condit*Treatm Least Squares Means													
Colony_Type	Condition	Treatment	_Colony_Type	_Condition	_Treatment	Estimate	Standard Error	DF	t Value	Pr > |t|	Alpha	Lower	Upper
CFU-GEMM	AoE+E11PSC	With Inhi	CFU-GM	E10.5AGM	No Inhi	-22.3348	1476.28	72	-0.02	0.9880	0.05	-2965.25	2920.58
CFU-GEMM	AoE+E11PSC	With Inhi	CFU-GM	E10.5AGM	With Inhi	-0.00011	2087.72	72	-0.00	1.0000	0.05	-4161.79	4161.79
CFU-GEMM	AoE+E11PSC	With Inhi	CFU-GM	E11.5AGM	No Inhi	-22.3627	1476.28	72	-0.02	0.9880	0.05	-2965.27	2920.55
CFU-GEMM	AoE+E11PSC	With Inhi	CFU-GM	E11.5AGM	With Inhi	-0.00011	2087.72	72	-0.00	1.0000	0.05	-4161.79	4161.79
CFU-GEMM	AoE+E11PSC	With Inhi	CFU-GM	HE+E10PSC	No Inhi	-21.8024	1476.28	72	-0.01	0.9883	0.05	-2964.71	2921.11
CFU-GEMM	AoE+E11PSC	With Inhi	CFU-GM	HE+E10PSC	With Inhi	-0.00011	2087.72	72	-0.00	1.0000	0.05	-4161.78	4161.78
CFU-GEMM	AoE+E11PSC	With Inhi	CFU-GM	HE+E11PSC	No Inhi	-21.7507	1476.28	72	-0.01	0.9883	0.05	-2964.66	2921.16
CFU-GEMM	AoE+E11PSC	With Inhi	CFU-GM	HE+E11PSC	With Inhi	38.0210	2952.45	72	0.01	0.9898	0.05	-5847.57	5923.61
CFU-GEMM	E10.5AGM	No Inhi	CFU-GEMM	E10.5AGM	With Inhi	20.8935	1476.28	72	0.01	0.9887	0.05	-2922.01	2963.80
CFU-GEMM	E10.5AGM	No Inhi	CFU-GEMM	E11.5AGM	No Inhi	-0.02362	0.06028	72	-0.39	0.6964	0.05	-0.1438	0.09655
CFU-GEMM	E10.5AGM	No Inhi	CFU-GEMM	E11.5AGM	With Inhi	20.8935	1476.28	72	0.01	0.9887	0.05	-2922.02	2963.80
CFU-GEMM	E10.5AGM	No Inhi	CFU-GEMM	HE+E10PSC	No Inhi	0.7192	0.08295	72	8.67	<.0001	0.05	0.5539	0.8846
CFU-GEMM	E10.5AGM	No Inhi	CFU-GEMM	HE+E10PSC	With Inhi	20.8935	1476.28	72	0.01	0.9887	0.05	-2922.01	2963.80
CFU-GEMM	E10.5AGM	No Inhi	CFU-GEMM	HE+E11PSC	No Inhi	0.6500	0.07321	72	8.88	<.0001	0.05	0.5040	0.7959
CFU-GEMM	E10.5AGM	No Inhi	CFU-GEMM	HE+E11PSC	With Inhi	20.8933	1476.15	72	0.01	0.9887	0.05	-2921.75	2963.54
CFU-GEMM	E10.5AGM	No Inhi	CFU-GM	AoE+E10PSC	No Inhi	-0.9981	0.05016	72	-19.90	<.0001	0.05	-1.0981	-0.8982
CFU-GEMM	E10.5AGM	No Inhi	CFU-GM	AoE+E10PSC	With Inhi	20.8934	1476.20	72	0.01	0.9887	0.05	-2921.85	2963.64
CFU-GEMM	E10.5AGM	No Inhi	CFU-GM	AoE+E11PSC	No Inhi	-1.0548	0.04978	72	-21.19	<.0001	0.05	-1.1540	-0.9555
CFU-GEMM	E10.5AGM	No Inhi	CFU-GM	AoE+E11PSC	With Inhi	20.8934	1476.20	72	0.01	0.9887	0.05	-2921.85	2963.64
CFU-GEMM	E10.5AGM	No Inhi	CFU-GM	E10.5AGM	No Inhi	-1.4413	0.04768	72	-30.23	<.0001	0.05	-1.5363	-1.3462
CFU-GEMM	E10.5AGM	No Inhi	CFU-GM	E10.5AGM	With Inhi	20.8934	1476.20	72	0.01	0.9887	0.05	-2921.86	2963.65
CFU-GEMM	E10.5AGM	No Inhi	CFU-GM	E11.5AGM	No Inhi	-1.4692	0.04755	72	-30.90	<.0001	0.05	-1.5640	-1.3744
CFU-GEMM	E10.5AGM	No Inhi	CFU-GM	E11.5AGM	With Inhi	20.8934	1476.20	72	0.01	0.9887	0.05	-2921.86	2963.65
CFU-GEMM	E10.5AGM	No Inhi	CFU-GM	HE+E10PSC	No Inhi	-0.9089	0.05078	72	-17.90	<.0001	0.05	-1.0101	-0.8077

The GLIMMIX Procedure

Differences of Colony*Condit*Treatm Least Squares Means													
Colony_Type	Condition	Treatment	_Colony_Type	_Condition	_Treatment	Estimate	Standard Error	DF	t Value	Pr > |t|	Alpha	Lower	Upper
CFU-GEMM	E10.5AGM	No Inhi	CFU-GM	HE+E10PSC	With Inhi	20.8934	1476.19	72	0.01	0.9887	0.05	-2921.85	2963.63
CFU-GEMM	E10.5AGM	No Inhi	CFU-GM	HE+E11PSC	No Inhi	-0.8572	0.05117	72	-16.75	<.0001	0.05	-0.9592	-0.7552
CFU-GEMM	E10.5AGM	No Inhi	CFU-GM	HE+E11PSC	With Inhi	58.9145	2556.86	72	0.02	0.9817	0.05	-5038.09	5155.92
CFU-GEMM	E10.5AGM	With Inhi	CFU-GEMM	E11.5AGM	No Inhi	-20.9171	1476.28	72	-0.01	0.9887	0.05	-2963.82	2921.99
CFU-GEMM	E10.5AGM	With Inhi	CFU-GEMM	E11.5AGM	With Inhi	1.367E-6	2087.77	72	0.00	1.0000	0.05	-4161.90	4161.90
CFU-GEMM	E10.5AGM	With Inhi	CFU-GEMM	HE+E10PSC	No Inhi	-20.1743	1476.28	72	-0.01	0.9891	0.05	-2963.08	2922.73
CFU-GEMM	E10.5AGM	With Inhi	CFU-GEMM	HE+E10PSC	With Inhi	2.324E-6	2087.77	72	0.00	1.0000	0.05	-4161.90	4161.90
CFU-GEMM	E10.5AGM	With Inhi	CFU-GEMM	HE+E11PSC	No Inhi	-20.2435	1476.28	72	-0.01	0.9891	0.05	-2963.15	2922.66
CFU-GEMM	E10.5AGM	With Inhi	CFU-GEMM	HE+E11PSC	With Inhi	-0.00018	2087.68	72	-0.00	1.0000	0.05	-4161.71	4161.71
CFU-GEMM	E10.5AGM	With Inhi	CFU-GM	AoE+E10PSC	No Inhi	-21.8917	1476.28	72	-0.01	0.9882	0.05	-2964.80	2921.02
CFU-GEMM	E10.5AGM	With Inhi	CFU-GM	AoE+E10PSC	With Inhi	-0.00011	2087.71	72	-0.00	1.0000	0.05	-4161.78	4161.78
CFU-GEMM	E10.5AGM	With Inhi	CFU-GM	AoE+E11PSC	No Inhi	-21.9483	1476.28	72	-0.01	0.9882	0.05	-2964.86	2920.96
CFU-GEMM	E10.5AGM	With Inhi	CFU-GM	AoE+E11PSC	With Inhi	-0.00011	2087.71	72	-0.00	1.0000	0.05	-4161.78	4161.78
CFU-GEMM	E10.5AGM	With Inhi	CFU-GM	E10.5AGM	No Inhi	-22.3348	1476.28	72	-0.02	0.9880	0.05	-2965.24	2920.57
CFU-GEMM	E10.5AGM	With Inhi	CFU-GM	E10.5AGM	With Inhi	-0.00011	2087.72	72	-0.00	1.0000	0.05	-4161.79	4161.79
CFU-GEMM	E10.5AGM	With Inhi	CFU-GM	E11.5AGM	No Inhi	-22.3627	1476.28	72	-0.02	0.9880	0.05	-2965.27	2920.54
CFU-GEMM	E10.5AGM	With Inhi	CFU-GM	E11.5AGM	With Inhi	-0.00011	2087.72	72	-0.00	1.0000	0.05	-4161.79	4161.79
CFU-GEMM	E10.5AGM	With Inhi	CFU-GM	HE+E10PSC	No Inhi	-21.8024	1476.28	72	-0.01	0.9883	0.05	-2964.71	2921.10
CFU-GEMM	E10.5AGM	With Inhi	CFU-GM	HE+E10PSC	With Inhi	-0.00011	2087.71	72	-0.00	1.0000	0.05	-4161.78	4161.78
CFU-GEMM	E10.5AGM	With Inhi	CFU-GM	HE+E11PSC	No Inhi	-21.7507	1476.28	72	-0.01	0.9883	0.05	-2964.66	2921.16
CFU-GEMM	E10.5AGM	With Inhi	CFU-GM	HE+E11PSC	With Inhi	38.0210	2952.44	72	0.01	0.9898	0.05	-5847.57	5923.61
CFU-GEMM	E11.5AGM	No Inhi	CFU-GEMM	E11.5AGM	With Inhi	20.9171	1476.28	72	0.01	0.9887	0.05	-2921.99	2963.83
CFU-GEMM	E11.5AGM	No Inhi	CFU-GEMM	HE+E10PSC	No Inhi	0.7428	0.08269	72	8.98	<.0001	0.05	0.5780	0.9077
CFU-GEMM	E11.5AGM	No Inhi	CFU-GEMM	HE+E10PSC	With Inhi	20.9171	1476.28	72	0.01	0.9887	0.05	-2921.99	2963.82

The GLIMMIX Procedure

Differences of Colony*Condit*Treatm Least Squares Means													
Colony_Type	Condition	Treatment	_Colony_Type	_Condition	_Treatment	Estimate	Standard Error	DF	t Value	Pr > |t|	Alpha	Lower	Upper
CFU-GEMM	E11.5AGM	No Inhi	CFU-GEMM	HE+E11PSC	No Inhi	0.6736	0.07291	72	9.24	<.0001	0.05	0.5282	0.8189
CFU-GEMM	E11.5AGM	No Inhi	CFU-GEMM	HE+E11PSC	With Inhi	20.9169	1476.15	72	0.01	0.9887	0.05	-2921.73	2963.56
CFU-GEMM	E11.5AGM	No Inhi	CFU-GM	AoE+E10PSC	No Inhi	-0.9745	0.04973	72	-19.60	<.0001	0.05	-1.0737	-0.8754
CFU-GEMM	E11.5AGM	No Inhi	CFU-GM	AoE+E10PSC	With Inhi	20.9170	1476.20	72	0.01	0.9887	0.05	-2921.83	2963.66
CFU-GEMM	E11.5AGM	No Inhi	CFU-GM	AoE+E11PSC	No Inhi	-1.0312	0.04935	72	-20.89	<.0001	0.05	-1.1295	-0.9328
CFU-GEMM	E11.5AGM	No Inhi	CFU-GM	AoE+E11PSC	With Inhi	20.9170	1476.20	72	0.01	0.9887	0.05	-2921.83	2963.66
CFU-GEMM	E11.5AGM	No Inhi	CFU-GM	E10.5AGM	No Inhi	-1.4177	0.04723	72	-30.02	<.0001	0.05	-1.5118	-1.3235
CFU-GEMM	E11.5AGM	No Inhi	CFU-GM	E10.5AGM	With Inhi	20.9170	1476.20	72	0.01	0.9887	0.05	-2921.84	2963.67
CFU-GEMM	E11.5AGM	No Inhi	CFU-GM	E11.5AGM	No Inhi	-1.4455	0.04710	72	-30.69	<.0001	0.05	-1.5394	-1.3517
CFU-GEMM	E11.5AGM	No Inhi	CFU-GM	E11.5AGM	With Inhi	20.9170	1476.20	72	0.01	0.9887	0.05	-2921.84	2963.67
CFU-GEMM	E11.5AGM	No Inhi	CFU-GM	HE+E10PSC	No Inhi	-0.8853	0.05036	72	-17.58	<.0001	0.05	-0.9857	-0.7849
CFU-GEMM	E11.5AGM	No Inhi	CFU-GM	HE+E10PSC	With Inhi	20.9170	1476.19	72	0.01	0.9887	0.05	-2921.82	2963.66
CFU-GEMM	E11.5AGM	No Inhi	CFU-GM	HE+E11PSC	No Inhi	-0.8336	0.05075	72	-16.43	<.0001	0.05	-0.9348	-0.7324
CFU-GEMM	E11.5AGM	No Inhi	CFU-GM	HE+E11PSC	With Inhi	58.9381	2556.86	72	0.02	0.9817	0.05	-5038.07	5155.94
CFU-GEMM	E11.5AGM	With Inhi	CFU-GEMM	HE+E10PSC	No Inhi	-20.1743	1476.28	72	-0.01	0.9891	0.05	-2963.08	2922.74
CFU-GEMM	E11.5AGM	With Inhi	CFU-GEMM	HE+E10PSC	With Inhi	9.571E-7	2087.77	72	0.00	1.0000	0.05	-4161.90	4161.90
CFU-GEMM	E11.5AGM	With Inhi	CFU-GEMM	HE+E11PSC	No Inhi	-20.2435	1476.28	72	-0.01	0.9891	0.05	-2963.15	2922.67
CFU-GEMM	E11.5AGM	With Inhi	CFU-GEMM	HE+E11PSC	With Inhi	-0.00018	2087.68	72	-0.00	1.0000	0.05	-4161.71	4161.71
CFU-GEMM	E11.5AGM	With Inhi	CFU-GM	AoE+E10PSC	No Inhi	-21.8917	1476.28	72	-0.01	0.9882	0.05	-2964.80	2921.02
CFU-GEMM	E11.5AGM	With Inhi	CFU-GM	AoE+E10PSC	With Inhi	-0.00011	2087.72	72	-0.00	1.0000	0.05	-4161.78	4161.78
CFU-GEMM	E11.5AGM	With Inhi	CFU-GM	AoE+E11PSC	No Inhi	-21.9483	1476.28	72	-0.01	0.9882	0.05	-2964.86	2920.96
CFU-GEMM	E11.5AGM	With Inhi	CFU-GM	AoE+E11PSC	With Inhi	-0.00011	2087.72	72	-0.00	1.0000	0.05	-4161.78	4161.78
CFU-GEMM	E11.5AGM	With Inhi	CFU-GM	E10.5AGM	No Inhi	-22.3348	1476.28	72	-0.02	0.9880	0.05	-2965.24	2920.57
CFU-GEMM	E11.5AGM	With Inhi	CFU-GM	E10.5AGM	With Inhi	-0.00011	2087.72	72	-0.00	1.0000	0.05	-4161.79	4161.79

The GLIMMIX Procedure

Differences of Colony*Condit*Treatm Least Squares Means													
Colony_Type	Condition	Treatment	_Colony_Type	_Condition	_Treatment	Estimate	Standard Error	DF	t Value	Pr > |t|	Alpha	Lower	Upper
CFU-GEMM	E11.5AGM	With Inhi	CFU-GM	E11.5AGM	No Inhi	-22.3627	1476.28	72	-0.02	0.9880	0.05	-2965.27	2920.55
CFU-GEMM	E11.5AGM	With Inhi	CFU-GM	E11.5AGM	With Inhi	-0.00011	2087.72	72	-0.00	1.0000	0.05	-4161.79	4161.79
CFU-GEMM	E11.5AGM	With Inhi	CFU-GM	HE+E10PSC	No Inhi	-21.8024	1476.28	72	-0.01	0.9883	0.05	-2964.71	2921.11
CFU-GEMM	E11.5AGM	With Inhi	CFU-GM	HE+E10PSC	With Inhi	-0.00011	2087.71	72	-0.00	1.0000	0.05	-4161.78	4161.78
CFU-GEMM	E11.5AGM	With Inhi	CFU-GM	HE+E11PSC	No Inhi	-21.7507	1476.28	72	-0.01	0.9883	0.05	-2964.66	2921.16
CFU-GEMM	E11.5AGM	With Inhi	CFU-GM	HE+E11PSC	With Inhi	38.0210	2952.44	72	0.01	0.9898	0.05	-5847.57	5923.61
CFU-GEMM	HE+E10PSC	No Inhi	CFU-GEMM	HE+E10PSC	With Inhi	20.1743	1476.28	72	0.01	0.9891	0.05	-2922.73	2963.08
CFU-GEMM	HE+E10PSC	No Inhi	CFU-GEMM	HE+E11PSC	No Inhi	-0.06924	0.03900	72	-1.78	0.0800	0.05	-0.1470	0.008495
CFU-GEMM	HE+E10PSC	No Inhi	CFU-GEMM	HE+E11PSC	With Inhi	20.1741	1476.15	72	0.01	0.9891	0.05	-2922.47	2962.82
CFU-GEMM	HE+E10PSC	No Inhi	CFU-GM	AoE+E10PSC	No Inhi	-1.7174	0.07563	72	-22.71	<.0001	0.05	-1.8681	-1.5666
CFU-GEMM	HE+E10PSC	No Inhi	CFU-GM	AoE+E10PSC	With Inhi	20.1742	1476.20	72	0.01	0.9891	0.05	-2922.57	2962.92
CFU-GEMM	HE+E10PSC	No Inhi	CFU-GM	AoE+E11PSC	No Inhi	-1.7740	0.07538	72	-23.53	<.0001	0.05	-1.9243	-1.6237
CFU-GEMM	HE+E10PSC	No Inhi	CFU-GM	AoE+E11PSC	With Inhi	20.1742	1476.20	72	0.01	0.9891	0.05	-2922.57	2962.92
CFU-GEMM	HE+E10PSC	No Inhi	CFU-GM	E10.5AGM	No Inhi	-2.1605	0.07401	72	-29.19	<.0001	0.05	-2.3080	-2.0130
CFU-GEMM	HE+E10PSC	No Inhi	CFU-GM	E10.5AGM	With Inhi	20.1742	1476.20	72	0.01	0.9891	0.05	-2922.58	2962.93
CFU-GEMM	HE+E10PSC	No Inhi	CFU-GM	E11.5AGM	No Inhi	-2.1884	0.07393	72	-29.60	<.0001	0.05	-2.3357	-2.0410
CFU-GEMM	HE+E10PSC	No Inhi	CFU-GM	E11.5AGM	With Inhi	20.1742	1476.20	72	0.01	0.9891	0.05	-2922.58	2962.93
CFU-GEMM	HE+E10PSC	No Inhi	CFU-GM	HE+E10PSC	No Inhi	-1.6281	0.06558	72	-24.83	<.0001	0.05	-1.7589	-1.4974
CFU-GEMM	HE+E10PSC	No Inhi	CFU-GM	HE+E10PSC	With Inhi	20.1742	1476.19	72	0.01	0.9891	0.05	-2922.57	2962.91
CFU-GEMM	HE+E10PSC	No Inhi	CFU-GM	HE+E11PSC	No Inhi	-1.5764	0.08592	72	-18.35	<.0001	0.05	-1.7477	-1.4052
CFU-GEMM	HE+E10PSC	No Inhi	CFU-GM	HE+E11PSC	With Inhi	58.1953	2556.86	72	0.02	0.9819	0.05	-5038.81	5155.20
CFU-GEMM	HE+E10PSC	With Inhi	CFU-GEMM	HE+E11PSC	No Inhi	-20.2435	1476.28	72	-0.01	0.9891	0.05	-2963.15	2922.66
CFU-GEMM	HE+E10PSC	With Inhi	CFU-GEMM	HE+E11PSC	With Inhi	-0.00018	2087.68	72	-0.00	1.0000	0.05	-4161.71	4161.71
CFU-GEMM	HE+E10PSC	With Inhi	CFU-GM	AoE+E10PSC	No Inhi	-21.8917	1476.28	72	-0.01	0.9882	0.05	-2964.80	2921.02

The GLIMMIX Procedure

Differences of Colony*Condit*Treatm Least Squares Means													
Colony_Type	Condition	Treatment	_Colony_Type	_Condition	_Treatment	Estimate	Standard Error	DF	t Value	Pr > |t|	Alpha	Lower	Upper
CFU-GEMM	HE+E10PSC	With Inhi	CFU-GM	AoE+E10PSC	With Inhi	-0.00011	2087.71	72	-0.00	1.0000	0.05	-4161.78	4161.78
CFU-GEMM	HE+E10PSC	With Inhi	CFU-GM	AoE+E11PSC	No Inhi	-21.9483	1476.28	72	-0.01	0.9882	0.05	-2964.86	2920.96
CFU-GEMM	HE+E10PSC	With Inhi	CFU-GM	AoE+E11PSC	With Inhi	-0.00011	2087.72	72	-0.00	1.0000	0.05	-4161.78	4161.78
CFU-GEMM	HE+E10PSC	With Inhi	CFU-GM	E10.5AGM	No Inhi	-22.3348	1476.28	72	-0.02	0.9880	0.05	-2965.24	2920.57
CFU-GEMM	HE+E10PSC	With Inhi	CFU-GM	E10.5AGM	With Inhi	-0.00011	2087.72	72	-0.00	1.0000	0.05	-4161.79	4161.79
CFU-GEMM	HE+E10PSC	With Inhi	CFU-GM	E11.5AGM	No Inhi	-22.3627	1476.28	72	-0.02	0.9880	0.05	-2965.27	2920.54
CFU-GEMM	HE+E10PSC	With Inhi	CFU-GM	E11.5AGM	With Inhi	-0.00011	2087.72	72	-0.00	1.0000	0.05	-4161.79	4161.79
CFU-GEMM	HE+E10PSC	With Inhi	CFU-GM	HE+E10PSC	No Inhi	-21.8024	1476.28	72	-0.01	0.9883	0.05	-2964.71	2921.11
CFU-GEMM	HE+E10PSC	With Inhi	CFU-GM	HE+E10PSC	With Inhi	-0.00011	2087.71	72	-0.00	1.0000	0.05	-4161.77	4161.77
CFU-GEMM	HE+E10PSC	With Inhi	CFU-GM	HE+E11PSC	No Inhi	-21.7507	1476.28	72	-0.01	0.9883	0.05	-2964.66	2921.16
CFU-GEMM	HE+E10PSC	With Inhi	CFU-GM	HE+E11PSC	With Inhi	38.0210	3616.03	72	0.01	0.9916	0.05	-7170.41	7246.45
CFU-GEMM	HE+E11PSC	No Inhi	CFU-GEMM	HE+E11PSC	With Inhi	20.2434	1476.15	72	0.01	0.9891	0.05	-2922.40	2962.89
CFU-GEMM	HE+E11PSC	No Inhi	CFU-GM	AoE+E10PSC	No Inhi	-1.6481	0.06480	72	-25.44	<.0001	0.05	-1.7773	-1.5189
CFU-GEMM	HE+E11PSC	No Inhi	CFU-GM	AoE+E10PSC	With Inhi	20.2434	1476.20	72	0.01	0.9891	0.05	-2922.50	2962.99
CFU-GEMM	HE+E11PSC	No Inhi	CFU-GM	AoE+E11PSC	No Inhi	-1.7047	0.06451	72	-26.43	<.0001	0.05	-1.8333	-1.5762
CFU-GEMM	HE+E11PSC	No Inhi	CFU-GM	AoE+E11PSC	With Inhi	20.2434	1476.20	72	0.01	0.9891	0.05	-2922.50	2962.99
CFU-GEMM	HE+E11PSC	No Inhi	CFU-GM	E10.5AGM	No Inhi	-2.0913	0.06290	72	-33.25	<.0001	0.05	-2.2166	-1.9659
CFU-GEMM	HE+E11PSC	No Inhi	CFU-GM	E10.5AGM	With Inhi	20.2434	1476.20	72	0.01	0.9891	0.05	-2922.51	2963.00
CFU-GEMM	HE+E11PSC	No Inhi	CFU-GM	E11.5AGM	No Inhi	-2.1191	0.06280	72	-33.74	<.0001	0.05	-2.2443	-1.9939
CFU-GEMM	HE+E11PSC	No Inhi	CFU-GM	E11.5AGM	With Inhi	20.2434	1476.20	72	0.01	0.9891	0.05	-2922.51	2963.00
CFU-GEMM	HE+E11PSC	No Inhi	CFU-GM	HE+E10PSC	No Inhi	-1.5589	0.06528	72	-23.88	<.0001	0.05	-1.6890	-1.4287
CFU-GEMM	HE+E11PSC	No Inhi	CFU-GM	HE+E10PSC	With Inhi	20.2434	1476.19	72	0.01	0.9891	0.05	-2922.50	2962.98
CFU-GEMM	HE+E11PSC	No Inhi	CFU-GM	HE+E11PSC	No Inhi	-1.5072	0.06558	72	-22.98	<.0001	0.05	-1.6379	-1.3765
CFU-GEMM	HE+E11PSC	No Inhi	CFU-GM	HE+E11PSC	With Inhi	58.2645	2556.86	72	0.02	0.9819	0.05	-5038.74	5155.27

The GLIMMIX Procedure

Differences of Colony*Condit*Treatm Least Squares Means													
Colony_Type	Condition	Treatment	_Colony_Type	_Condition	_Treatment	Estimate	Standard Error	DF	t Value	Pr > |t|	Alpha	Lower	Upper
CFU-GEMM	HE+E11PSC	With Inhi	CFU-GM	AoE+E10PSC	No Inhi	-21.8915	1476.15	72	-0.01	0.9882	0.05	-2964.53	2920.75
CFU-GEMM	HE+E11PSC	With Inhi	CFU-GM	AoE+E10PSC	With Inhi	0.000070	2087.62	72	0.00	1.0000	0.05	-4161.60	4161.60
CFU-GEMM	HE+E11PSC	With Inhi	CFU-GM	AoE+E11PSC	No Inhi	-21.9481	1476.15	72	-0.01	0.9882	0.05	-2964.59	2920.70
CFU-GEMM	HE+E11PSC	With Inhi	CFU-GM	AoE+E11PSC	With Inhi	0.000067	2087.62	72	0.00	1.0000	0.05	-4161.59	4161.59
CFU-GEMM	HE+E11PSC	With Inhi	CFU-GM	E10.5AGM	No Inhi	-22.3346	1476.15	72	-0.02	0.9880	0.05	-2964.98	2920.31
CFU-GEMM	HE+E11PSC	With Inhi	CFU-GM	E10.5AGM	With Inhi	0.000073	2087.63	72	0.00	1.0000	0.05	-4161.61	4161.61
CFU-GEMM	HE+E11PSC	With Inhi	CFU-GM	E11.5AGM	No Inhi	-22.3625	1476.15	72	-0.02	0.9880	0.05	-2965.01	2920.28
CFU-GEMM	HE+E11PSC	With Inhi	CFU-GM	E11.5AGM	With Inhi	0.000071	2087.63	72	0.00	1.0000	0.05	-4161.60	4161.60
CFU-GEMM	HE+E11PSC	With Inhi	CFU-GM	HE+E10PSC	No Inhi	-21.8022	1476.15	72	-0.01	0.9883	0.05	-2964.45	2920.84
CFU-GEMM	HE+E11PSC	With Inhi	CFU-GM	HE+E10PSC	With Inhi	0.000069	2087.62	72	0.00	1.0000	0.05	-4161.60	4161.60
CFU-GEMM	HE+E11PSC	With Inhi	CFU-GM	HE+E11PSC	No Inhi	-21.7506	1476.15	72	-0.01	0.9883	0.05	-2964.39	2920.89
CFU-GEMM	HE+E11PSC	With Inhi	CFU-GM	HE+E11PSC	With Inhi	38.0212	2087.71	72	0.02	0.9855	0.05	-4123.75	4199.79
CFU-GM	AoE+E10PSC	No Inhi	CFU-GM	AoE+E10PSC	With Inhi	21.8915	1476.20	72	0.01	0.9882	0.05	-2920.85	2964.63
CFU-GM	AoE+E10PSC	No Inhi	CFU-GM	AoE+E11PSC	No Inhi	-0.05663	0.03630	72	-1.56	0.1231	0.05	-0.1290	0.01573
CFU-GM	AoE+E10PSC	No Inhi	CFU-GM	AoE+E11PSC	With Inhi	21.8915	1476.20	72	0.01	0.9882	0.05	-2920.85	2964.63
CFU-GM	AoE+E10PSC	No Inhi	CFU-GM	E10.5AGM	No Inhi	-0.4431	0.03335	72	-13.29	<.0001	0.05	-0.5096	-0.3766
CFU-GM	AoE+E10PSC	No Inhi	CFU-GM	E10.5AGM	With Inhi	21.8915	1476.20	72	0.01	0.9882	0.05	-2920.86	2964.65
CFU-GM	AoE+E10PSC	No Inhi	CFU-GM	E11.5AGM	No Inhi	-0.4710	0.03317	72	-14.20	<.0001	0.05	-0.5372	-0.4049
CFU-GM	AoE+E10PSC	No Inhi	CFU-GM	E11.5AGM	With Inhi	21.8915	1476.20	72	0.01	0.9882	0.05	-2920.86	2964.64
CFU-GM	AoE+E10PSC	No Inhi	CFU-GM	HE+E10PSC	No Inhi	0.08923	0.03766	72	2.37	0.0205	0.05	0.01416	0.1643
CFU-GM	AoE+E10PSC	No Inhi	CFU-GM	HE+E10PSC	With Inhi	21.8915	1476.19	72	0.01	0.9882	0.05	-2920.85	2964.63
CFU-GM	AoE+E10PSC	No Inhi	CFU-GM	HE+E11PSC	No Inhi	0.1409	0.03818	72	3.69	0.0004	0.05	0.06481	0.2170
CFU-GM	AoE+E10PSC	No Inhi	CFU-GM	HE+E11PSC	With Inhi	59.9127	2556.86	72	0.02	0.9814	0.05	-5037.09	5156.92
CFU-GM	AoE+E10PSC	With Inhi	CFU-GM	AoE+E11PSC	No Inhi	-21.9482	1476.20	72	-0.01	0.9882	0.05	-2964.69	2920.79

The GLIMMIX Procedure

Differences of Colony*Condit*Treatm Least Squares Means													
Colony_Type	Condition	Treatment	_Colony_Type	_Condition	_Treatment	Estimate	Standard Error	DF	t Value	Pr > abs(t)	Alpha	Lower	Upper
CFU-GM	AoE+E10PSC	With Inhi	CFU-GM	AoE+E11PSC	With Inhi	-2.55E-6	2087.66	72	-0.00	1.0000	0.05	-4161.67	4161.67
CFU-GM	AoE+E10PSC	With Inhi	CFU-GM	E10.5AGM	No Inhi	-22.3347	1476.20	72	-0.02	0.9880	0.05	-2965.08	2920.41
CFU-GM	AoE+E10PSC	With Inhi	CFU-GM	E10.5AGM	With Inhi	3.463E-6	2087.66	72	0.00	1.0000	0.05	-4161.67	4161.67
CFU-GM	AoE+E10PSC	With Inhi	CFU-GM	E11.5AGM	No Inhi	-22.3626	1476.20	72	-0.02	0.9880	0.05	-2965.11	2920.38
CFU-GM	AoE+E10PSC	With Inhi	CFU-GM	E11.5AGM	With Inhi	1.823E-6	2087.66	72	0.00	1.0000	0.05	-4161.67	4161.67
CFU-GM	AoE+E10PSC	With Inhi	CFU-GM	HE+E10PSC	No Inhi	-21.8023	1476.20	72	-0.01	0.9883	0.05	-2964.55	2920.94
CFU-GM	AoE+E10PSC	With Inhi	CFU-GM	HE+E10PSC	With Inhi	-2.28E-7	2087.66	72	-0.00	1.0000	0.05	-4161.67	4161.67
CFU-GM	AoE+E10PSC	With Inhi	CFU-GM	HE+E11PSC	No Inhi	-21.7506	1476.20	72	-0.01	0.9883	0.05	-2964.49	2920.99
CFU-GM	AoE+E10PSC	With Inhi	CFU-GM	HE+E11PSC	With Inhi	38.0211	2952.40	72	0.01	0.9898	0.05	-5847.49	5923.53
CFU-GM	AoE+E11PSC	No Inhi	CFU-GM	AoE+E11PSC	With Inhi	21.9482	1476.20	72	0.01	0.9882	0.05	-2920.79	2964.69
CFU-GM	AoE+E11PSC	No Inhi	CFU-GM	E10.5AGM	No Inhi	-0.3865	0.03279	72	-11.79	<.0001	0.05	-0.4519	-0.3211
CFU-GM	AoE+E11PSC	No Inhi	CFU-GM	E10.5AGM	With Inhi	21.9482	1476.20	72	0.01	0.9882	0.05	-2920.81	2964.70
CFU-GM	AoE+E11PSC	No Inhi	CFU-GM	E11.5AGM	No Inhi	-0.4144	0.03261	72	-12.71	<.0001	0.05	-0.4794	-0.3494
CFU-GM	AoE+E11PSC	No Inhi	CFU-GM	E11.5AGM	With Inhi	21.9482	1476.20	72	0.01	0.9882	0.05	-2920.81	2964.70
CFU-GM	AoE+E11PSC	No Inhi	CFU-GM	HE+E10PSC	No Inhi	0.1459	0.03716	72	3.93	0.0002	0.05	0.07178	0.2199
CFU-GM	AoE+E11PSC	No Inhi	CFU-GM	HE+E10PSC	With Inhi	21.9482	1476.19	72	0.01	0.9882	0.05	-2920.79	2964.69
CFU-GM	AoE+E11PSC	No Inhi	CFU-GM	HE+E11PSC	No Inhi	0.1975	0.03769	72	5.24	<.0001	0.05	0.1224	0.2727
CFU-GM	AoE+E11PSC	No Inhi	CFU-GM	HE+E11PSC	With Inhi	59.9693	2556.86	72	0.02	0.9814	0.05	-5037.04	5156.97
CFU-GM	AoE+E11PSC	With Inhi	CFU-GM	E10.5AGM	No Inhi	-22.3347	1476.20	72	-0.02	0.9880	0.05	-2965.08	2920.41
CFU-GM	AoE+E11PSC	With Inhi	CFU-GM	E10.5AGM	With Inhi	6.015E-6	2087.66	72	0.00	1.0000	0.05	-4161.67	4161.67
CFU-GM	AoE+E11PSC	With Inhi	CFU-GM	E11.5AGM	No Inhi	-22.3626	1476.20	72	-0.02	0.9880	0.05	-2965.10	2920.38
CFU-GM	AoE+E11PSC	With Inhi	CFU-GM	E11.5AGM	With Inhi	4.375E-6	2087.66	72	0.00	1.0000	0.05	-4161.67	4161.67
CFU-GM	AoE+E11PSC	With Inhi	CFU-GM	HE+E10PSC	No Inhi	-21.8023	1476.20	72	-0.01	0.9883	0.05	-2964.54	2920.94
CFU-GM	AoE+E11PSC	With Inhi	CFU-GM	HE+E10PSC	With Inhi	2.324E-6	2087.65	72	0.00	1.0000	0.05	-4161.66	4161.66

The GLIMMIX Procedure

Differences of Colony*Condit*Treatm Least Squares Means													
Colony_Type	Condition	Treatment	_Colony_Type	_Condition	_Treatment	Estimate	Standard Error	DF	t Value	Pr > |t|	Alpha	Lower	Upper
CFU-GM	AoE+E11PSC	With Inhi	CFU-GM	HE+E11PSC	No Inhi	-21.7506	1476.20	72	-0.01	0.9883	0.05	-2964.49	2920.99
CFU-GM	AoE+E11PSC	With Inhi	CFU-GM	HE+E11PSC	With Inhi	38.0211	2952.40	72	0.01	0.9898	0.05	-5847.48	5923.52
CFU-GM	E10.5AGM	No Inhi	CFU-GM	E10.5AGM	With Inhi	22.3347	1476.20	72	0.02	0.9880	0.05	-2920.42	2965.09
CFU-GM	E10.5AGM	No Inhi	CFU-GM	E11.5AGM	No Inhi	-0.02788	0.02929	72	-0.95	0.3444	0.05	-0.08627	0.03051
CFU-GM	E10.5AGM	No Inhi	CFU-GM	E11.5AGM	With Inhi	22.3347	1476.20	72	0.02	0.9880	0.05	-2920.42	2965.09
CFU-GM	E10.5AGM	No Inhi	CFU-GM	HE+E10PSC	No Inhi	0.5324	0.03429	72	15.53	<.0001	0.05	0.4640	0.6007
CFU-GM	E10.5AGM	No Inhi	CFU-GM	HE+E10PSC	With Inhi	22.3347	1476.19	72	0.02	0.9880	0.05	-2920.40	2965.07
CFU-GM	E10.5AGM	No Inhi	CFU-GM	HE+E11PSC	No Inhi	0.5841	0.03486	72	16.76	<.0001	0.05	0.5146	0.6535
CFU-GM	E10.5AGM	No Inhi	CFU-GM	HE+E11PSC	With Inhi	60.3558	2556.86	72	0.02	0.9812	0.05	-5036.65	5157.36
CFU-GM	E10.5AGM	With Inhi	CFU-GM	E11.5AGM	No Inhi	-22.3626	1476.20	72	-0.02	0.9880	0.05	-2965.12	2920.39
CFU-GM	E10.5AGM	With Inhi	CFU-GM	E11.5AGM	With Inhi	-1.64E-6	2087.67	72	-0.00	1.0000	0.05	-4161.68	4161.68
CFU-GM	E10.5AGM	With Inhi	CFU-GM	HE+E10PSC	No Inhi	-21.8023	1476.20	72	-0.01	0.9883	0.05	-2964.56	2920.95
CFU-GM	E10.5AGM	With Inhi	CFU-GM	HE+E10PSC	With Inhi	-3.69E-6	2087.66	72	-0.00	1.0000	0.05	-4161.67	4161.67
CFU-GM	E10.5AGM	With Inhi	CFU-GM	HE+E11PSC	No Inhi	-21.7506	1476.20	72	-0.01	0.9883	0.05	-2964.50	2921.00
CFU-GM	E10.5AGM	With Inhi	CFU-GM	HE+E11PSC	With Inhi	38.0211	2952.41	72	0.01	0.9898	0.05	-5847.49	5923.53
CFU-GM	E11.5AGM	No Inhi	CFU-GM	E11.5AGM	With Inhi	22.3626	1476.20	72	0.02	0.9880	0.05	-2920.39	2965.12
CFU-GM	E11.5AGM	No Inhi	CFU-GM	HE+E10PSC	No Inhi	0.5603	0.03411	72	16.42	<.0001	0.05	0.4922	0.6283
CFU-GM	E11.5AGM	No Inhi	CFU-GM	HE+E10PSC	With Inhi	22.3626	1476.19	72	0.02	0.9880	0.05	-2920.38	2965.10
CFU-GM	E11.5AGM	No Inhi	CFU-GM	HE+E11PSC	No Inhi	0.6119	0.03468	72	17.64	<.0001	0.05	0.5428	0.6811
CFU-GM	E11.5AGM	No Inhi	CFU-GM	HE+E11PSC	With Inhi	60.3837	2556.86	72	0.02	0.9812	0.05	-5036.62	5157.39
CFU-GM	E11.5AGM	With Inhi	CFU-GM	HE+E10PSC	No Inhi	-21.8023	1476.20	72	-0.01	0.9883	0.05	-2964.56	2920.95
CFU-GM	E11.5AGM	With Inhi	CFU-GM	HE+E10PSC	With Inhi	-2.05E-6	2087.66	72	-0.00	1.0000	0.05	-4161.67	4161.67
CFU-GM	E11.5AGM	With Inhi	CFU-GM	HE+E11PSC	No Inhi	-21.7506	1476.20	72	-0.01	0.9883	0.05	-2964.50	2921.00
CFU-GM	E11.5AGM	With Inhi	CFU-GM	HE+E11PSC	With Inhi	38.0211	2952.41	72	0.01	0.9898	0.05	-5847.49	5923.53

The GLIMMIX Procedure

Differences of Colony*Condit*Treatm Least Squares Means													
Colony_Type	Condition	Treatment	_Colony_Type	_Condition	_Treatment	Estimate	Standard Error	DF	t Value	Pr > |t|	Alpha	Lower	Upper
CFU-GM	HE+E10PSC	No Inhi	CFU-GM	HE+E10PSC	With Inhi	21.8023	1476.19	72	0.01	0.9883	0.05	-2920.94	2964.54
CFU-GM	HE+E10PSC	No Inhi	CFU-GM	HE+E11PSC	No Inhi	0.05168	0.03900	72	1.33	0.1893	0.05	-0.02606	0.1294
CFU-GM	HE+E10PSC	No Inhi	CFU-GM	HE+E11PSC	With Inhi	59.8234	2556.86	72	0.02	0.9814	0.05	-5037.18	5156.83
CFU-GM	HE+E10PSC	With Inhi	CFU-GM	HE+E11PSC	No Inhi	-21.7506	1476.19	72	-0.01	0.9883	0.05	-2964.49	2920.99
CFU-GM	HE+E10PSC	With Inhi	CFU-GM	HE+E11PSC	With Inhi	38.0211	2087.68	72	0.02	0.9855	0.05	-4123.69	4199.73
CFU-GM	HE+E11PSC	No Inhi	CFU-GM	HE+E11PSC	With Inhi	59.7717	2556.86	72	0.02	0.9814	0.05	-5037.23	5156.78
