# Supplementary material for: Mesoderm-derived PDGFRA+ cells regulate the emergence of hematopoietic stem cells in the dorsal aorta
Source: Nat Cell Biol. 2022 Jul 28;24(8):1211–25. doi: 10.1038/s41556-022-00955-3 (PMC9359911; doi:10.1038/s41556-022-00955-3)
Supplement: Supplementary file 2 — Reporting Summary [file 41556_2022_955_MOESM2_ESM.pdf]

nature portfolio

Corresponding author(s): Professor John E Pimanda
Dr Vashe Chandrakanthan

Last updated by author(s): 25/04/22

# Reporting Summary

Nature Portfolio wishes to improve the reproducibility of the work that we publish. This form provides structure for consistency and transparency in reporting. For further information on Nature Portfolio policies, see our Editorial Policies and the Editorial Policy Checklist.

## Statistics

For all statistical analyses, confirm that the following items are present in the figure legend, table legend, main text, or Methods section.

| n/a | Confirmed | |
|---|---|---|
| ☐ | ☒ | The exact sample size (*n*) for each experimental group/condition, given as a discrete number and unit of measurement |
| ☐ | ☒ | A statement on whether measurements were taken from distinct samples or whether the same sample was measured repeatedly |
| ☐ | ☒ | The statistical test(s) used AND whether they are one- or two-sided<br>*Only common tests should be described solely by name; describe more complex techniques in the Methods section.* |
| ☒ | ☐ | A description of all covariates tested |
| ☐ | ☒ | A description of any assumptions or corrections, such as tests of normality and adjustment for multiple comparisons |
| ☐ | ☒ | A full description of the statistical parameters including central tendency (e.g. means) or other basic estimates (e.g. regression coefficient) AND variation (e.g. standard deviation) or associated estimates of uncertainty (e.g. confidence intervals) |
| ☐ | ☒ | For null hypothesis testing, the test statistic (e.g. *F*, *t*, *r*) with confidence intervals, effect sizes, degrees of freedom and *P* value noted<br>*Give P values as exact values whenever suitable.* |
| ☒ | ☐ | For Bayesian analysis, information on the choice of priors and Markov chain Monte Carlo settings |
| ☐ | ☒ | For hierarchical and complex designs, identification of the appropriate level for tests and full reporting of outcomes |
| ☒ | ☐ | Estimates of effect sizes (e.g. Cohen's *d*, Pearson's *r*), indicating how they were calculated |

*Our web collection on statistics for biologists contains articles on many of the points above.*

## Software and code

Policy information about availability of computer code

| | |
|---|---|
| Data collection | L780 LSM Zeiss, Leica SP8 DLS, Imaris software (v9.1), The BD LSRFortessa™ SORP X-20, BD Influx™, Illumina HiSeqX, 10X Genomics Chromium™ Single Cell 3' platform (v3 chemistry) |
| Data analysis | 1. Bulk RNA sequencing - reads were aligned to the mouse genome (mm10) (https://www.ncbi.nlm.nih.gov/assembly/GCF_000001635.20/) using the software STAR (v2.5.0b). Gene expression levels were quantified using HTSeq (v0.9). Expression levels were TMM-normalized using the software package EdgeR (v3.5) in the R statistical analysis software (v3.3.3). Hierarchical clustering with average linkage and Euclidean distance was performed using the Partek Genomics Suite (v 6.6).<br><br>2. Single cell RNA sequencing – To analyze the filtered gene expression counts, we wrote custom Python 3.9 scripts, available at: https://github.com/iosonofabio/scpaper_Vashe. We then used scanpy (v1.8.2) (https://scanpy.readthedocs.io) to log the counts, calculate overdispersed features, perform PCA and UMAP embedding (https://arxiv.org/abs/1802.03426), compute a similarity graph with 10 neighbors, and cluster with the Leiden algorithm then used singlet (https://singlet.readthedocs.io) to make dot plots with a threshold of 0.5 cptt and UMAP projections by cluster and logged gene expression. Pseudotime analysis was performed using scanpy (https://scanpy.readthedocs.io/en/stable/api/scanpy.tl.dpt.html).<br><br>3. Cell Ranger (v3.1.0) was used to process raw datasets including – quality control, the extraction of gene expression matrices, and the aggregation of gene expression matrices from different sequencing runs with batch effect removal.<br><br>4. Statistical data analysis – All statistical analyses were performed using SAS v9.4 (SAS version 9.4, SAS Institute (2016), Cary, NC). SAS statistical analysis and codes are available as Chandrakanthan et al_Statistics_SAS Output.<br><br>5. Flow cytometry- FlowJo™ v10.5+, BD FACSDiva™ v6.0 |

6. GraphPad Prism v8+

For manuscripts utilizing custom algorithms or software that are central to the research but not yet described in published literature, software must be made available to editors and reviewers. We strongly encourage code deposition in a community repository (e.g. GitHub). See the Nature Portfolio guidelines for submitting code & software for further information.

## Data

Policy information about availability of data

All manuscripts must include a data availability statement. This statement should provide the following information, where applicable:

- Accession codes, unique identifiers, or web links for publicly available datasets
- A description of any restrictions on data availability
- For clinical datasets or third party data, please ensure that the statement adheres to our policy

Bulk and single cell RNA-sequencing data were aligned to mm10 (https://www.ncbi.nlm.nih.gov/assembly/GCF_000001635.20/) and have been deposited in Gene Expression Omnibus under GSE163757 and GSE114464 respectively. These data are publicly available. Source data are provided with this paper. All other data supporting the findings of this study are available from the corresponding authors on reasonable request.

# Field-specific reporting

Please select the one below that is the best fit for your research. If you are not sure, read the appropriate sections before making your selection.

☒ Life sciences ☐ Behavioural & social sciences ☐ Ecological, evolutionary & environmental sciences

For a reference copy of the document with all sections, see nature.com/documents/nr-reporting-summary-flat.pdf

# Life sciences study design

All studies must disclose on these points even when the disclosure is negative.

| | |
|---|---|
| Sample size | No sample size calculation was performed. Sample sizes were selected on the basis of previous experiments (Reference #80 (Medvinsky et al, 2008), #84 (Taoudi et al., 2008)). Standard practice in molecular and cell biology involves at least three independent biological replicates for each experiment and the number performed for each are mentioned in the figure legends. Data presented in the figures reflect multiple independent experiments (independent cultures and passages were used for each repeat and were obtained on different days) with orthogonal validation where possible. |
| Data exclusions | No data were excluded from analysis. |
| Replication | Data presented in the figures reflect multiple independent experiments, performed on different days using different animals. All experiments were reproduced at least three times. |
| Randomization | Randomization does not apply to cell based experiments, in which large numbers of cells from a given source were partitioned among experimental conditions. Tissues from multiple embryos and adult (12-16 week, female) mice were pooled and then divided as embryo equivalents or defined cell numbers for downstream experiments. |
| Blinding | Investigators were blinded to group allocation during data collection and analysis. |

# Reporting for specific materials, systems and methods

We require information from authors about some types of materials, experimental systems and methods used in many studies. Here, indicate whether each material, system or method listed is relevant to your study. If you are not sure if a list item applies to your research, read the appropriate section before selecting a response.

## Materials & experimental systems

| n/a | Involved in the study | |
|---|---|---|
| ☐ | ☒ | Antibodies |
| ☒ | ☐ | Eukaryotic cell lines |
| ☒ | ☐ | Palaeontology and archaeology |
| ☐ | ☒ | Animals and other organisms |
| ☒ | ☐ | Human research participants |
| ☒ | ☐ | Clinical data |
| ☒ | ☐ | Dual use research of concern |

## Methods

| n/a | Involved in the study | |
|---|---|---|
| ☒ | ☐ | ChIP-seq |
| ☐ | ☒ | Flow cytometry |
| ☒ | ☐ | MRI-based neuroimaging |

## Antibodies

Antibodies used

A list of antibodies and their source are included in the supplemental information file as a resource table under Reagents or Resource Antibodies (extended data)
REAGENT or RESOURCE SOURCE IDENTIFIER
Antibodies
AcLDL-Alexa Fluor 488 Invitrogen L23380
Anti-GFP Invitrogen A10263
Anti-GFP-Alex-488 Invitrogen A21311
Anti-GFP-Alex-647 Invitrogen A31852
αSarcomaric actinin Sigma 051M4773
B220-BV421 Biolegend 103239
Calponin Abcam ab46794
CD146 Biolegend 134701
CD31 Abcam ab28364
CD31-APC eBioscience 17-0453-82
CD31-PE BD Biosciences 553373
CD31-PE/Cy7 BD Biosciences 561410
CD3-Alexa Fluor 647 Biolegend 100209
CD41- PE/Cy7 Biolegend 133915
CD43-PE/Cy7 Biolegend 143210
CD45 eBioscience 14-0451-82
CD45-eFluor eBioscience 48-0451-82
CD45-FITC Biolegend 103107
CD4-APC/CY7 Biolegend 100414
CD8-BV421 Biolegend 100737
cKIT BD Bioscience 103101
cKIT-APC Biolegend 105811
Gr1-BV421 Biolegend 108433
Hnf4α Santa Cruz Biotechnology sc-6556
Mac1-APCCy7 Biolegend 101226
Mouse Lineage Panel BD Pharmigen 557791
Myh11 Thermo Fisher Scientific MA5-11971
Nestin Millipore MAB353
PDGFRA BD Pharmigen 558774
PDGFRA (APA5) Biolegend 135901
PDGFRA-APC Biolegend 135908
PDGFRA-BV421 Biolegend 562774
PDGFRB Biolegend 136002
PDGFRB (APB5) Biolegend 136002
Purified Anti-YFP Biovision 3991-100
Sca1-PE/Cy7 Biolegend 108114
Serum response factor Abcam ab53147
Tuj1 Santa Cruz Biotechnology sc-80005
VE-Cadherin Santa Cruz Biotechnology sc-9989
VE-Cadherin-PE Biolegend 138105

Validation

REAGENT or RESOURCE SOURCE IDENTIFIER DILUTION REFERENCE (PMID)
AcLDL-Alexa Fluor 488 Invitrogen L23380 5ug/ml PMID:20453163, 21885849
Anti-GFP-Biotin Invitrogen A10263 1;400 PMID: 25347465
Anti-GFP-Alex-488 Invitrogen A21311 1;500 PMID: 33907215
Anti-GFP-Alex-647 Invitrogen A31852 1;500 PMID: 28815216
αSarcomaric actinin Sigma 051M4773 1;500 PMID: 21084676
B220-BV421 Biolegend 103239 1;200 PMID: 24719463
Calponin Abcam ab46794 1;300 PMID: 28077619
CD146 Biolegend 134701 1;400 PMID: 24067916
CD31 Abcam ab28364 1;300 PMID: 29208669
CD31-APC eBioscience 17-0453-82 1;200 PMID: 27022143
CD31-PE BD Biosciences 553373 1;350 PMID: 7956830
CD31-PE/Cy7 BD Biosciences 561410 1;350 PMID: 7956830
CD3-Alexa Fluor 647 Biolegend 100209 1;200 PMID: 29466757
CD41- PE/Cy7 Biolegend 133915 1;300 PMID: 27183606
CD41-PE Biolegend 133906 1;100 PMID: 26193121
CD41-BV421 Biolegend 133911 1;150 PMID: 25840412
CD43-PE/Cy7 Biolegend 143210 1;300 PMID: 15778363
CD45 eBioscience 14-0451-82 1;300 PMID: 26347471
CD45-eFluor eBioscience 48-0451-82 1;150 PMID: 27525437
CD45-FITC Biolegend 103107 1;200 PMID: 16709810
CD45-PE/Cy7 Biolegend 103114 1;300 PMID: 16709810
CD4-APC/CY7 Biolegend 100414 1;200 PMID: 23851361
CD8-BV421 Biolegend 100737 1;150 PMID: 16116223
cKIT BD Bioscience 103101 1;300 PMID: 7508684
cKIT-APC Biolegend 105811 1;300 PMID: 20512127
Gr1-BV421 Biolegend 108433 1;150 PMID: 16142239

Hnf4α Santa Cruz Biotechnology sc-6556 1;400 PMID: 32880442
Mac1-APCCy7 Biolegend 101226 1;400 PMID: 24431111
Mouse Lineage Panel BD Pharmigen 557791 1;400 PMID: 9169840
Myh11 Thermo Fisher Scientific MA5-11971 1;400 PMID: 32102389
Nestin Millipore MAB353 1;300 PMID: 25683249
PDGFRA BD Pharmigen 558774 1;400 PMID: 8875964
PDGFRA (APA5) Biolegend 135901 1;300 PMID: 8875964
PDGFRA-APC Biolegend 135908 1;200 PMID: 26056396
PDGFRA-BV421 Biolegend 562774 1;200 PMID: 26056396
PDGFRB Biolegend 136002 1;350 PMID: 29861387
PDGFRB (APB5) Biolegend 136002 1;200 PMID: 11413086
Purified Anti-YFP Biovision 3991-100 1;250 PMID: 31387989
Sca1-PE/Cy7 Biolegend 108114 1;300 PMID: 19443245
Serum response factor Abcam ab53147 1;250 PMID: 27323859
Tuj1 Santa Cruz Biotechnology sc-80005 1;300 PMID: 31619962
VE-Cadherin Santa Cruz Biotechnology sc-9989 1;300 PMID: 31863691
VE-Cadherin-PE Biolegend 138105 1;300 PMID: 11156369
VE-Cadherin-APC Biolegend 138012 1;200 PMID: 11156369

## Animals and other organisms

Policy information about studies involving animals; ARRIVE guidelines recommended for reporting animal research

Laboratory animals

1. C57BL/6J- 12-16 weeks old Males and females used for time mating to get age appropriate embryos. Females were used for transplant experiments. 3-4 females used to get age appropriate embryos and 3-5 females used for each transplant experiments (please see figures for exact numbers).
2. Pdgfratm11(EGFP)Sor- 12-16 weeks old Males and females used for time mating to get age appropriate embryos. 4-6 females used to get age appropriate embryos per experiment.
3. Gt(ROSA)26Sortm(EYFP)Cos/J-This is a reporter mouse strain. 12-16 weeks old Males or females used for time mating with male or female Cre-lines to get age appropriate embryos. 4-8 females used to get age appropriate embryos per experiment
Tg(CAG-DsRed*MST)1Nagy- This is a reporter mouse strain. Males or females used for time mating with male or female Mesp1 or Wnt1 Cre-lines to get age appropriate embryos. 4-8 females used to get age appropriate embryos per experiment.
C57BL/6Tg(UBC-GFP)30Scha/J- 12-16 weeks old Males and females used for time mating to get age appropriate embryos. 12-16 weeks old Females were used to harvest heart, lung, aorta and IVC endothelial cells. 3 females used to get age appropriate embryos per experiment.
Mesp1tm2(cre)Ysa- 12-16 weeks old Males and females used for time mating with reporter mouse lines to get age appropriate embryos.4-8 females used to get age appropriate embryos per experiment.
Tg(Wnt1-cre)11Rth- 12-16 weeks old Males and females used for time mating with reporter mouse lines to get age appropriate embryos.4-8 females used to get age appropriate embryos per experiment.
Sox1tm1(cre)Take- 12-16 weeks old Males and females used for time mating with reporter mouse lines to get age appropriate embryos.4-8 females used to get age appropriate embryos per experiment.
Tg(Pdgfra-cre/ERT2)1Wdr- 12-16 weeks old Males and females used for time mating with reporter mouse lines to get age appropriate embryos for lineage tracing studies. 6-8 females used to get age appropriate embryo.
Tg(Nes-EGFP)33Enik-12-16 weeks old Males and females used for time mating to get age appropriate embryos. 4-6 females used to get age appropriate embryos per experiment.
Gt(ROSA)26Sortm1(HBEGF)Awai-12-16 weeks old Males and females used for time mating with reporter mouse lines to get age appropriate embryos for lineage tracing studies. 6-8 females used to get age appropriate embryos

Animals were housed and bred at the Biological Resources Centre at UNSW, a specific pathogen free, PC2 facility with semi-natural light cycle of 12:12 hours light: dark, and regulated air quality, ventilation (15 ACH), humidity (55%) and temperature (22C)- see methods.

Wild animals

Study did not involve wild animals.

Field-collected samples

Study did not involve samples collected from the field.

Ethics oversight

All animal experiments were approved by the Animal Ethics Committee of UNSW Sydney, Sydney, NSW, Australia

Note that full information on the approval of the study protocol must also be provided in the manuscript.

## Flow Cytometry

### Plots

Confirm that:

☒ The axis labels state the marker and fluorochrome used (e.g. CD4-FITC).

☒ The axis scales are clearly visible. Include numbers along axes only for bottom left plot of group (a 'group' is an analysis of identical markers).

☒ All plots are contour plots with outliers or pseudocolor plots.

☒ A numerical value for number of cells or percentage (with statistics) is provided.

## Methodology

| | |
|---|---|
| Sample preparation | Sample preparation was in accordance with published protocols (references are included in the methods section). |
| Instrument | Mononuclear staining was analysed on a BD LSRFortessa (BD Biosciences). Cell sorts were performed on a BD Influx (BD Biosciences). |
| Software | BD FACSDiva™ Software v6 (BD Biosciences) and FACS data were analysed using FlowJo v10.5+ software (TreeStar). |
| Cell population abundance | We sorted PSCs and endothelial cells separately. |
| Gating strategy | First gated for FSC-A/FSC-H and eliminated all doublets and then gated for FSC-A/SSC-A to select mononuclear cells. From there we used relevant fluorochromes to gate and select respective cell populations for our experiments. Experimental details are listed in the main and supplemental information files (including controls). |

☒ Tick this box to confirm that a figure exemplifying the gating strategy is provided in the Supplementary Information.
